# A new avenue for obtaining insight into the functional characteristics of long noncoding RNAs associated with estrogen receptor signaling

**DOI:** 10.1038/srep31716

**Published:** 2016-08-19

**Authors:** Liangcai Wu, Qianqian Xu, Haohai Zhang, Ming Li, Chengpei Zhu, Minjie Jiang, Xinting Sang, Yi Zhao, Qiang Sun, Haitao Zhao

**Affiliations:** 1 Department of Liver Surgery, Peking Union Medical College Hospital, Chinese Academy of Medical Sciences and Peking Union Medical College, Beijing, China; 2 Key Laboratory of Intelligent Information Processing, Institute of Computing Technology, Chinese Academy of Sciences, Beijing, China; 3 Department of Breast Surgery, Peking Union Medical College Hospital, Chinese Academy of Medical Sciences and Peking Union Medical College, Beijing, China

## Abstract

Estrogen receptor signalling plays important regulatory roles in multiple mammalian physiological processes. Dysregulation of estrogen receptor (ER) expression and/or its associated signalling pathway is strongly associated with the development, progression, transition, and endocrine-resistance of breast cancer. Non-coding transcripts are essential regulators of almost every level of gene regulation. However, few long non-coding transcripts (lncRNAs) associated with the estrogen receptor signalling pathway have been well-described. We used array-based methods to identify 33 estrogen receptor agitation-related (ERAR) lncRNAs. A coding–non-coding gene co-expression network analysis suggested that 15 ERAR lncRNAs were associated with mitosis, DNA damage, and DNA repair. Kaplan–Meier analysis indicated that five ERAR lncRNAs selected using the Random Forest-Recursive Feature Elimination algorithm were significantly correlated with endocrine resistance-free survival and distant metastasis-free survival as well as disease free survival. Our results suggest that ERAR lncRNAs may serve as novel biomarkers for guiding breast cancer treatment and prognosis. Furthermore, our findings reveal a new avenue by which estrogen receptor signalling can be further explored.

Breast cancer remains a major health issue among middle-aged and older women worldwide. The majority of breast cancer patients are diagnosed with estrogen receptor-positive (ER+) breast cancer. Since Jensen *et al*.^1^ first established a link between estrogen receptors and breast cancer in 1971, clinical and experimental evidence has confirmed that abnormal regulation of ER signalling is closely associated with breast cancer. For example, excessive exposure to estradiol (E2) and 27-hydroxycholesterol (a primary metabolite of cholesterol) can mediate breast cancer cell proliferation^2^,^3^. Conversely, blocking unduly activated ER signalling can significantly improve disease-free survival of ER+ breast cancer patients^4^,^5^,^6^. Therefore, these observations highlight the importance of the ER signalling pathway in most types of breast cancer.

Emerging studies reveal that non-coding RNAs, such as long non-coding RNAs (lncRNAs), can serve as critical modulators of breast cancer development and progression. For example, HOTAIR—an antisense transcript from the HOXC locus—is up-regulated in breast cancer and is significantly associated with cancer metastasis and poor patient prognosis. The HOTAIR promoter region contains estrogen response elements, enabling estrogen receptor co-regulators such as CBP/p300 and MLL1 to bind the promoter of HOTAIR and activate its transcription^7^. H19 is another well-known lncRNA that is significantly over-expressed in both ER+ breast cancers and E2-treated breast cancer cell lines^8^,^9^. Therefore, lncRNAs associated with ER signalling may serve important regulatory roles in most types of breast cancer. However, few lncRNAs associated with ER signalling have been well-characterized^10^,^11^.

A gene co-expression network can be modelled as an undirected graph in which each node represents a gene, while each edge represents the co-expression relationship between a gene pair^12^. A co-expression relationship between a gene pair is established only when the two genes in the pair show similar expression patterns, their expression levels rise and fall together across samples, and the correlation coefficient reaches a statistical cut-off value. Bute *et al*. first employed this strategy for functional genomic clustering in 2000^13^. Since then, this co-expression method has been widely employed to predict the functions of unknown molecules, including both protein-coding and non-coding genes^12^,^14^,^15^,^16^,^17^. This methodology provides an avenue for exploring the functional role of lncRNA genes.

We used an array-based method to explore ER-targeted lncRNAs and their potential functional roles. First, we identified ER-targeted lncRNAs in MCF-7 cells cultured with E2 and the full ER antagonist compound ICI-182,780. Based on the expression profiles of protein-coding genes and lncRNAs, we then constructed an array-based coding–non-coding gene co-expression (CNC) network to explore the potential functions of the estrogen receptor agitation-related (ERAR) lncRNAs identified. Finally, we conducted a survival analysis and found that the expression profiles of some ERAR lncRNAs were significantly correlated with endocrine resistance-free survival and distant metastasis-free survival of ER+ breast cancer patients.

## Results

### Estrogen receptor alpha agitation-related (ERAR) lncRNA genes

**Figure 1:**
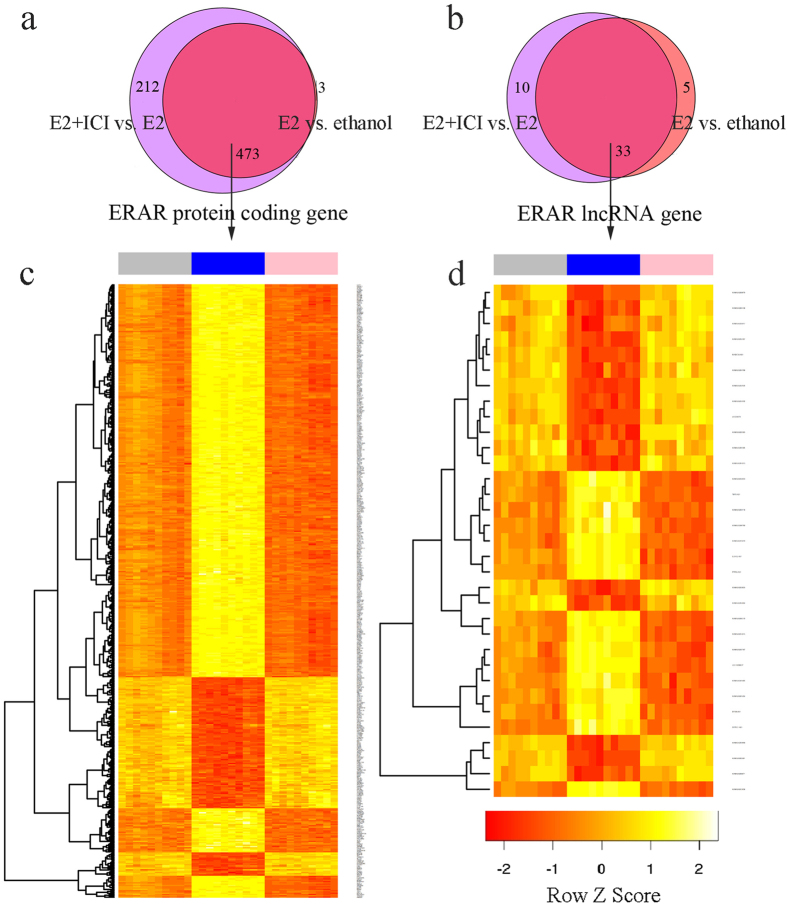
Identification of estrogen receptor alpha agitation related genes. (**a,b**) Protein-coding genes (**a**) and Long noncoding genes (**b**) that are significantly differentially expressed in the E2 vs. ethanol and E2&ICI vs. E2 were regarded as *ERAR* genes. (**c,d**) Expression profile of 473 ERAR protein-coding genes and 33 ERAR lncRNA genes, ethanol-treated (grey), E2 treated (blue) and E2&ICI-treated (pink) groups.

The MCF-7 cell line has been widely used to study estrogen signalling. Presently, raw gene expression data of MCF-7 cells cultured with ethanol, E2, or E2 + ICI-182,780 (ICI) respectively were obtained from the Gene Expression Omnibus database. A new chip-description-file provided by ncFANs version 2^16^ was used to calculate gene expression profiles, through which the expression profiling of 2,812 lncRNAs and 17,282 protein-coding genes was accomplished simultaneously. Next, we used the limma package in R to identify genes with significant differential expression between E2- and ethanol-treated groups (E2 vs. ethanol), and the E2+ ICI- and E2-treated groups (E2+ ICI vs. E2). Only genes showing a > 2-fold change with Benjamini-Hochberg adjusted P-value < 0.01 were classified as significantly differentially expressed. As ICI functions as a full ER antagonist and can completely reverse E2 agitation activity on ER, only genes that were agitated E2 and reversed by ER antagonist ICI were regarded as *ERAR* genes. (Fig. 1). We identified 33 ERAR lncRNAs and 473 ERAR coding genes. Gene ontology biological process (GOBP) enrichment analysis of these ERAR protein-coding genes revealed significant associations with the mitotic cell cycle, DNA replication, and DNA repair.

### Characterization of ERAR lncRNA genes

**Figure 2:**
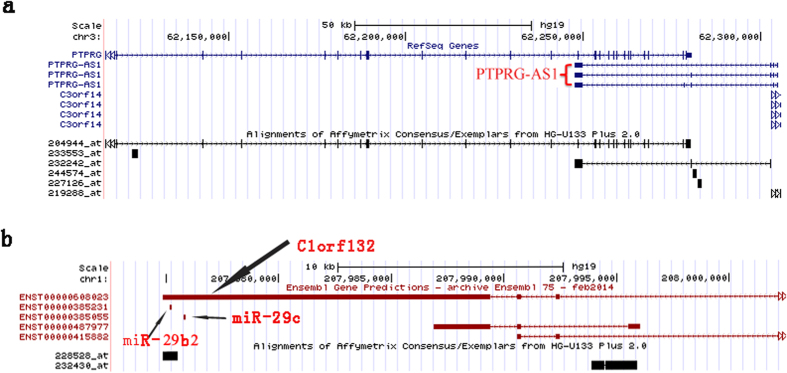
Genomic context of PTPRG-AS1 (**a**) and C1orf132 (**b**). The sequences of miR-29b and miR-29c located within C1orf132 lncRNA.

We next characterized the 33 ERAR lncRNA genes identified. Firstly, we queried each of the ERAR lncRNA genes in the NONCODE v4 and refseq database for detailed information. Among the 33 ERAR lncRNA genes are 24 long intergenic non-coding RNA genes and six antisense lncRNA genes (Table S4). For example, EP300-AS1 and PTPRG-AS1 are two up-regulated ERAR lncRNA genes identified in the E2-treated group. In breast cancer, the overlapping protein encoding genes in the opposite sense of EP300-AS1 and PTPRG-AS1 act as an oncogene and a tumour suppressor, respectively^18^,^19^,^20^. Notably, PTPRG-AS1, the antisense lncRNA of PTPRG, has three isoforms (Fig. 2a), and its expression is closely associated with tumour grade and clinical outcome^15^ for breast cancer. EP300-AS1 is a 1,405 bp gene with three exons whose paired protein-coding gene is EP300. EP300 acts as a critical regulator of cell division and the cell cycle. EP300 is significantly overexpressed in breast cancer tissue and serves as an independent biomarker of poor prognosis for breast cancer patients. Consistently, inhibition of p300 can suppress the growth and invasion of breast cancer^18^,^21^.

Approximately 17.5% of miRNAs are located within lncRNAs, and these miRNAs possess a distinct processing mechanism^22^. We extracted the sequences of identified ERAR lncRNAs from the NONCODE v4 database. These sequences were examined using the UCSC genome browser and BLAST to identify the sequences of miRNAs located within ERAR lncRNA genes. For instance, the sequences of miR-29b2 and miR-29c are located within the lncRNA C1orf132 (Fig. 2b). MiR-29b is the major member of the miR-29 family and acts as a critical tumour suppressor and a core regulator of EMT in breast cancer^23^,^24^. Additionally, the sequences of miR-1251 and miR-135A2 are located within the transcript of RMST. By targeting HOXA10, miR-135a promotes breast cancer cell migration and invasion^25^.

### Functional prediction of lncRNAs based on the identity of their co-expressed protein-coding genes

**Figure 3:**
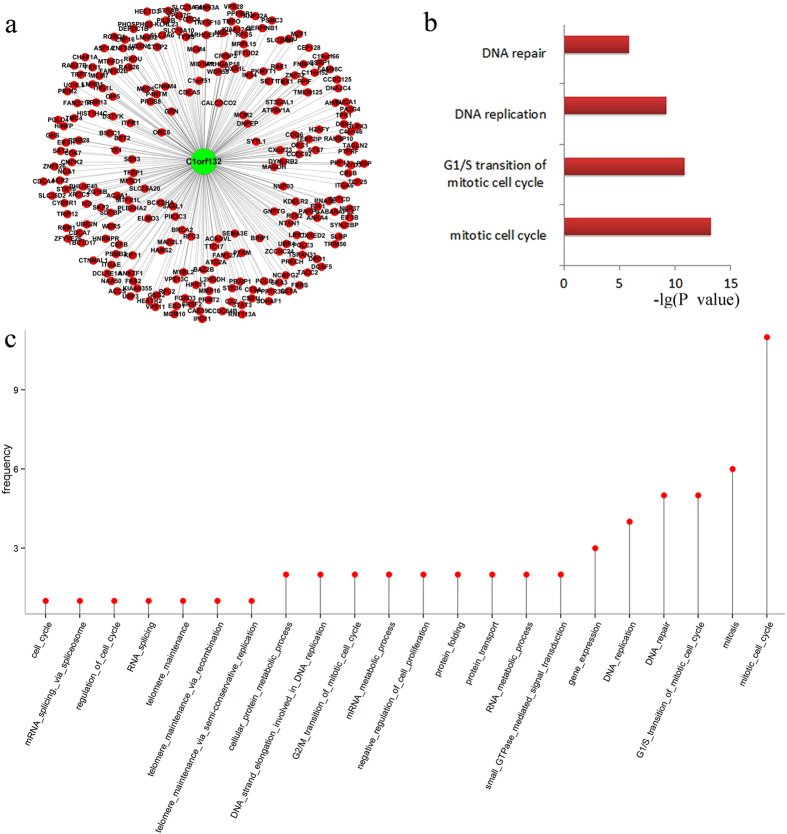
Predicting the function of lncRNAs based on the two color co-expression network. (**a,b**) Predicting of the function of C1orf132 based on the Hub network. (**c**) Frequency of the predicted functions of 15 ERAR lncRNA genes.

We further explored the functional roles of ERAR lncRNA genes by constructing a two-color CNC network based on an expression profile determined using a re-annotated Affymetrix Human Genome U133 Plus2 array data, as previously described^16^,^17^. The final CNC network contained 11,008 protein-coding genes and 1,116 lncRNA genes. Among these nodes, 414,946 coding–coding edges, 25,631 coding–noncoding edges, and 572 noncoding–noncoding edges were formed with a Pearson correlation coefficient >0.93 (Table S5, Figure S2). Next, a hub-based method was used to predict the function of these lncRNA genes. In this method, a single lncRNA gene is the hub of sub-network. lncRNA genes that were significantly co-expressed with ten or more protein-coding genes and showed at least one significantly enriched GOBP term were further examined (Figs 3a,b and S2, Table S6). Then, significantly enriched GOBP terms of neighbouring protein coding genes were assigned to the hub lncRNA gene as its predicted function. We parsed the topology of the whole co-expression network into separated hub-bused subnetworks and identified 15 ERAR lncRNA genes that met the above criteria (corrected P value > 0.93) (Figure S2, Table S6). These genes were significantly (cumulative hypergeometric P-values^16^,^26^ <0.01) associated with mitosis, gene expression, RNA metabolic processes, signal transduction, and protein transport (Fig. 3c, Table S6).

### Prognostic value of ERAR lncRNA genes

**Figure 4:**
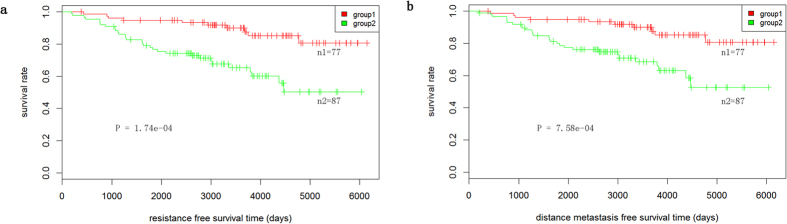
Kaplan-Meier survival curves for resistance-free survival (**a**) and distance metastasis-free survival. (**b**) between two groups that were clustered based on five lncRNA expression patterns.

ER signalling status significantly correlates with prognosis for ER+ breast cancer patients, especially those who are sensitive to anti-estrogen therapy^27^. We next investigated whether ERAR lncRNA genes were of clinical importance by conducting Kaplan–Meier survival analysis on 164 ER+ breast cancer patients. We first employed the Random Forest-Recursive Feature Elimination (RF-RFE) algorithm introduced by Granitto *et al*.^28^ using the caret package in R and filtered the most predictive five features (as they maximize the accuracy to 0.76) for further analysis (Figure S1). The 164 ER+ breast cancer patients were then divided into two groups using the k-means clustering method based on the expression profiles of the five selected ERAR lncRNAs. Finally, Kaplan–Meier curves of the two groups were plotted, and significance was estimated using the log-rank *t*-test (Fig. 4). ER+ breast cancer patients were classified as either high- or low-risk for endocrine-resistant and distant metastasis based on the expression profile of the five ERAR lncRNA genes. In another 140 ER+ breast cancer patients validation cohort, patients can also be classified into good and poor prognosis groups by its expression profile (Figure S3). In particular, patients with high expression of C1orf132 and low expression of CTC-260E6.6, LOC100288637, RP11-48B3.4, and EP300-AS1 enjoyed a favourable prognosis.

## Discussion

The ER signalling pathway plays a critical role in mammalian physiological processes. Clinical and experimental studies have shown that dysregulation of ER expression and/or its associated signalling pathway is strongly associated with breast cancer development, progression, transition, and endocrine resistance^29^,^30^. However, few studies have examined ER-targeted lncRNA genes and their functions^10^. We used an array-based method to identify ERAR lncRNA genes and employed an array-based gene co-expression method to explore the potential functions of these lncRNA genes. Kaplan–Meier survival analysis revealed that the expression pattern of several ERAR lncRNA genes could classify ER+ breast cancer patients into high- or low-risk endocrine-resistant and distant metastasis groups.

Microarrays have been widely used to assess gene expression abundance. Even though expression levels of lncRNA genes are generally lower than those of protein coding genes, multiple studies have employed microarray-based techniques to assess expression of lncRNA genes^31^,^32^,^33^. The capacity for microarrays to detect gene expression signals is affected by various factors, and biases are generated depending on the platform used and the source of data^34^. Presently, we selected ER positive datasets with prognostic information on the same microarray platform from the same laboratory or medical centre in an attempt to reduce bias to an extent.

High-throughput sequencing methods are powerful tools for studying whole-genome transcripts, including the transcripts of both protein-coding and non-coding genes. Nevertheless, the costs associated with these techniques may prevent their application to large-scale sampling. Instead, microarrays are a relatively economical alternative means to assess long non-coding transcript expression. Arguably, detailed analysis of human genome transcripts revealed that microarray probes map perfectly to non-coding transcripts^32^,^35^. This could potentially ease to determine the expression profiles of both protein-coding and non-coding genes simultaneously.

Currently, the NONCODE database lists 167,150 human lncRNAs transcript from 101,700 lncRNA genes^36^. However, only limited numbers of lncRNA genes were involved into analysis in re-annotated microarray. In our re-annotation microarray only nearly 3 percent of total known lncRNAs genes were annotated. This may be a limitation of our study. In the further analysis, RNA-Seq should be performed to identify ERAR lncRNA and its co-expressed genes. Nevertheless, the use of hub co-expression network analysis may overcome this limitation to some degree. This is because the hub-based method defines a single lncRNA as the hub of a co-expression network, and functional enrichment of neighbouring genes is assigned to the hub lncRNA gene.

Antisense lncRNAs are long non-coding transcripts from the antisense strand of protein-coding genes. They can serve as positive or negative modulators of paired protein-coding genes^37^,^38^. We identified six antisense lncRNAs, with PTPRG-AS1 and EP300-AS1 being two of the most interesting. PTPRG, the paired protein-coding gene of PTPRG-AS1, is a tumour suppressor gene frequently down-regulated in human breast cancer. *In vitro* studies using MCF-7 cells have shown that PTPRG is significantly down-regulated by E2, and high expression of PTPRG can repress cell growth^20^. Additionally, a recent study demonstrated that PTPRG-AS1 is significantly differentially expressed between ER+ and ER− breast cancers, and is closely associated with tumour grade and clinical outcomes^15^. EP300-AS1, the antisense transcript of the protein-coding gene EP300, was significantly up-regulated in our poor prognosis group. EP300 encodes p300 protein, which plays a critical role in cell division and the cell cycle^18^,^19^. Inhibition of p300 can suppress the growth and invasion of breast cancer^18^,^21^. Moreover, CBP/p300 can activate HOTAIR transcription by binding to its promoter region^7^. Further studies should explore the interaction between these antisense non-coding transcripts and their paired sense protein-coding transcripts.

Although most miRNAs are co-expressed from the introns of their host gene mRNAs^39^, Dhir *et al*. revealed that multiple miRNAs are derived from lncRNAs (lnc-pri-miRNAs)^22^. For example lncRNA H19 is the precursor of miR-675^40^. MiR-29b is a member of the miR-29 family which is generated from both chromosome 7q32.3 (miR-291) and chromosome 1q32.2 (miR-292). Presently, we identified the sequence of miR-29b2 (a main member of miR-29b^41^) within C1orf132. MiR-29b is a tumour suppressor and a core regulator of EMT. Additionally, repression of miR-29b enhances tumour invasion and metastasis in breast cancer^23^,^24^. We found that C1orf132 was significantly down-regulated in the poor prognosis group. Furthermore, Zhao *et al*. have reported that miR-29b serves as a critical modulator of the NF-κB–miR-29b–p53 pathway and is significantly down-regulated in ER+ breast cancer^41^. Consistently, we found that expression of C1orf132 significantly correlated with levels of UBE2N, TNFSF10, and BST2—which are all enriched in the IκB kinase/NF-κB cascade GOBP term in the hub network analysis. These findings suggest that the C1orf132 lncRNA may serve as a precursor of miR-29b2 and regulate the NF-κB signalling pathway to ultimately play a critical regulatory role in breast cancer. Further experiments will be necessary to identify the regulator role of C1orf132 breast cancer.

Feature selection, also known as variable selection, is the process of selecting a small subset of relevant features for further analysis with the minimum possible generalization error. We employed the popular RF-RFE algorithm^28^ to automatically select the most relevant of the 33 ERAR lncRNA genes identified for further Kaplan–Meier survival analysis. In this analysis, individual genes mean individual predictive power of resistance-free survival and distant metastasis-free survival are only 0.5724 ± 0.10146 and 0.6178 ± 0.08422, respectively. While, C1orf132, CTC-260E6.6, LOC100288637, RP11-48B3.4, and EP300-AS1 were the top five predictive lncRNA genes, with maximum accuracies of 0.763 and 0.764 for predicting resistance-free survival and distant metastasis-free survival, respectively (Figure S1). Kaplan–Meier survival analysis of both cohorts revealed that these five ERAR lncRNA genes do have clinical relevance. However, because of the limited number of total lncRNAs able to be detected by the microarray chip, only 33 total ERAR lncRNA genes were identified. These genes may therefore represent only a small subset of total ERAR lncRNA genes. Nevertheless, our results demonstrate the importance of ERAR lncRNA genes and provide valuable information for further exploration of the functional role of ERAR lncRNA genes in human breast cancer.

### Dataset preparation

The raw datasets (CEL format) used in this study can be downloaded from the Gene Expression Omnibus database (http://www.ncbi.nlm.nih.gov/geo). The datasets from GSE35428 (E2, E2 + ICI, ethanol treated group) were used to identify ERAR lncRNA genes. The GSE46924 were employed to construct gene co-expression network. Both GSE35428 and GSE46924 were contributed by the same laboratory. Datasets GSE9195 and partial of GSE6532 (only GPL570 platform) were used to validate the clinical value of these ERAR lncRNA genes. Both GSE9195 and GSE6532 (GPL570 platform) were contributed by the same Guys Hospital (GUYT), London, United Kingdom. All 164 patients were diagnosed with ER + breast cancer and received tamoxifen therapy (Table S7). Another 140 ER + breast cancer patients with detailed clinical information from GSE31448 were used to further test the prognosis value of five ERAR lncRNA genes.

### Array probe annotation and differential expression analysis

A re-annotated chip-description-file (CDF) of Affymetrix HG-U133 plus 2.0 microarrays was provided by the ncFANs v2 website (http://www.bioinfo.org/ncfans/download.php). Using this file, the expression levels (log2-transformed) of both 17,282 protein-coding genes and 2,812 lncRNA genes could be calculated at the same time. The microarray analysis was performed with the Affy and limma Bioconductor packages^42^,^43^ after probe-level data were normalized via the Robust Multichip Average (RMA) method. Significant differentially expressed genes were detected by one-way ANOVA, FDR was corrected by Benjamini-Hochberg method. Only genes showing a fold change >2.0 and Benjamini-Hochberg adjust P value < 0.01 were regarded as Significant differentially expressed genes.

### Gene Ontology enrichment analysis of protein-coding genes

Differentially expressed protein-coding genes were submitted to the Database for Annotation, Visualization and Integrated Discovery (DAVID), v6.7^44^, to perform Gene Ontology enrichment and visualized with the Enrichment Map plug-in in Cytoscape^45^.

### Co-expression network construction

The expression profile including both coding and non-coding genes was used to construct the coding-non-coding gene co-expression network. Based on the expressional variance of each gene (both coding and non-coding genes), the top 75 percent (a default parameter of ncFNAs)^16^,^17^ were selected for further analysis. The Pearson correlation coefficient (Pcc) was employed to estimate the co-expression relationship of each gene pair. In ncFANs v2, “Correlation cut off value” is a user-defined cut-off. We have tried the cut off value from 0.95 to 0.93 to obtain more predict function of ERAR lncRNA gene. The P-value of each Pcc was estimated using Fisher’s asymptotic test and adjusted with the Bonferroni multiple test correction. Only gene pairs with a p ≤ 0.01 and a Pcc value in the top or bottom 0.05 percentile for each gene were regarded as co-expressed gene pairs^16^,^17^ The hub method provided by ncFANs v2 was used to predict the function of co-expressed lncRNAs. Briefly, single lncRNA is the hub co-expression network. LncRNAs that significantly co-expressed with ten or more protein-coding genes and showed at least one significantly enriched GOBP term were further examined. Significant enriched (cumulative hypergeometric P-value^17^,^26^ <0.01) gene ontology biological process (GOBP) of its neighboring protein coding genes were assigned to the lncRNA genes as its predicted function.

### Analysis of the clinical importance of ERAR lncRNAs

First, the expression profiles of 33 ERAR lncRNA genes in 164 ER + breast cancer patients were extracted. Next, we used the Random Forest-Recursive Feature Elimination (RF-RFE) algorithm introduced by Granitto *et al*.^28^ in R caret package to select the most important features (lncRNAs). Then, based on the expression profiles of the selected lncRNAs, k-means clustering was performed to divide the 164 ER + breast cancer patients into 2 groups. Finally, Kaplan-Meier survival curves were plotted with the R package survival, and the P value between the two curves was estimated with log-rank tests. In another 140 ER + breast cancer patients cohort, the same k-means cluster and Kaplan-Meier survival analysis were performed based on the expression profile of five lncRNA genes.

## Additional Information

**How to cite this article**: Wu, L. *et al*. A new avenue for obtaining insight into the functional characteristics of long noncoding RNAs associated with estrogen receptor signaling. *Sci. Rep.* **6**, 31716; doi: 10.1038/srep31716 (2016).

## Supplementary Material

Supplementary Information
