# Supplementary material for: A new avenue for obtaining insight into the functional characteristics of long noncoding RNAs associated with estrogen receptor signaling
Source: Sci Rep. 2016 Aug 19;6:31716. doi: 10.1038/srep31716 (PMC4991000; doi:10.1038/srep31716)
Supplement: Supplementary Information [file srep31716-s1.pdf]

A new avenue for obtaining insight into the functional characteristics of long noncoding RNAs associated with estrogen receptor signaling

Liangcai Wu[1,3†], Qianqian Xu[2†], Haohai Zhang[1†], Ming Li[3], Chengpei Zhu[1], Minjie Jiang[3], Xinting Sang[1], Yi Zhao[1,3*‡], Qiang Sun[2*‡], Haitao Zhao[1*‡]

[1] Department of Liver Surgery, Peking Union Medical College Hospital, Chinese Academy of Medical Sciences and Peking Union Medical College, Beijing, China.
[2] Department of Breast Surgery, Peking Union Medical College Hospital, Chinese Academy of Medical Sciences and Peking Union Medical College, Beijing, China.
[3] Key Laboratory of Intelligent Information Processing, Institute of Computing Technology, Chinese Academy of Sciences, Beijing, China.

[*] To whom correspondence should be addressed;
[†] These authors contributed equally to this work;
[‡] These authors share senior co-authorship.

Qianqian Xu, Qiang Sun*
Department of Breast Surgery, Peking Union Medical College Hospital, Chinese Academy of Medical Sciences and Peking Union Medical College (CAMS & PUMC), 1 Shuaifuyuan, Wangfujing, Beijing 100730, China. Tel.: +86 10 69152700;
Email: qqx8899@126.com (Qianqian Xu); sunqiangpumc@163.com (Qiang Sun*)

Liangcai Wu, Haohai Zhang, Chengpei Zhu, Xinting Sang, Haitao Zhao*
Department of Liver Surgery, Peking Union Medical College Hospital, Chinese Academy of Medical Sciences and Peking Union Medical College (CAMS & PUMC), 1 Shuaifuyuan, Wangfujing, Beijing 100730, China. Tel.: +86 10 69156042; fax: +86 10 69156043.
Email: wsxpyzr@163.com (Liangcai Wu); zhhlovesyj@163.com (Haohai Zhang); aydzcp0617@126.com (Chengpei Zhu); SangXT@pumch.cn (Xinting Sang) ZhaoHT@pumch.cn (Haitao Zhao*)

Ming Li, Minjie Jiang, Yi Zhao*
Key Laboratory of Intelligent Information Processing, Institute of Computing Technology, Chinese Academy of Sciences, Beijing, Beijing 100190, China. Tel.: +86 10 62601010

Email: liming@bioinfo.ac.cn (Ming Li); jiangminjie0881@126.com (Minjie Jiang); biozy@ict.ac.cn (Yi Zhao*)

Additional File

Figure S1. Select the most predictive features from 33 ERAR lncRNA genes. (a) Most predictive features for distance metastasis-free survival. (b). Most predictive features for resistance-free survival.

Figure S2: Two color coding-noncoding co-expression network. (a) Total co-expression network of both protein coding genes and lncRNA genes. Yellow nodes represent the protein coding genes, red nodes represent the lncRNA genes. The green lines between nodes represent their co-expression relationship. (b). Distribution of gene nodes' edge. (c) Edges of 15 ERAR lncRNA genes.

Figure S3: Kaplan-Meier survival curves for 140 ER+ breast cancer patients.

Table S1. Expression profile of 2812 lncRNA genes.

Table S2. Statistical result of 2812 lncRNA genes.

Table S3. Probe position of re-annotated 2812 lncRNA gene

Table S4. List of 33 ERAR lncRNA genes

Table S5. Statistics of total co-expression network.

Table S6. Functions of ERAR lncRNAs predicted from Hub co-expression network.

Table S7. Detail clinical information of 164 patients*.

Table S8. Detail clinical information of 140 patients.*

Table S9. Hub network of 15ERAR lncRNA genes

Figure S1

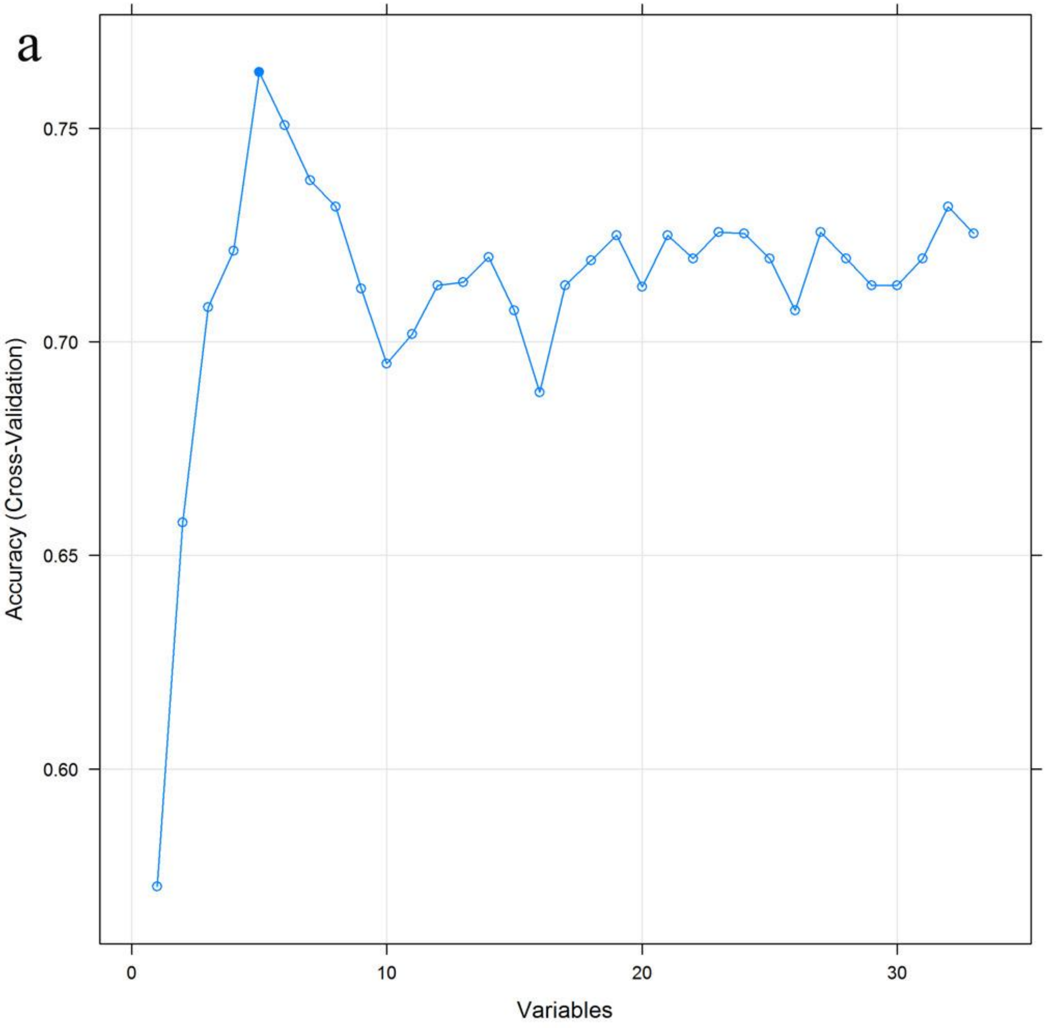

Top 5 variables: CTC-260E6.6, LOC100288637, C1orf132, EP300-AS1, RP11-48B3.4,

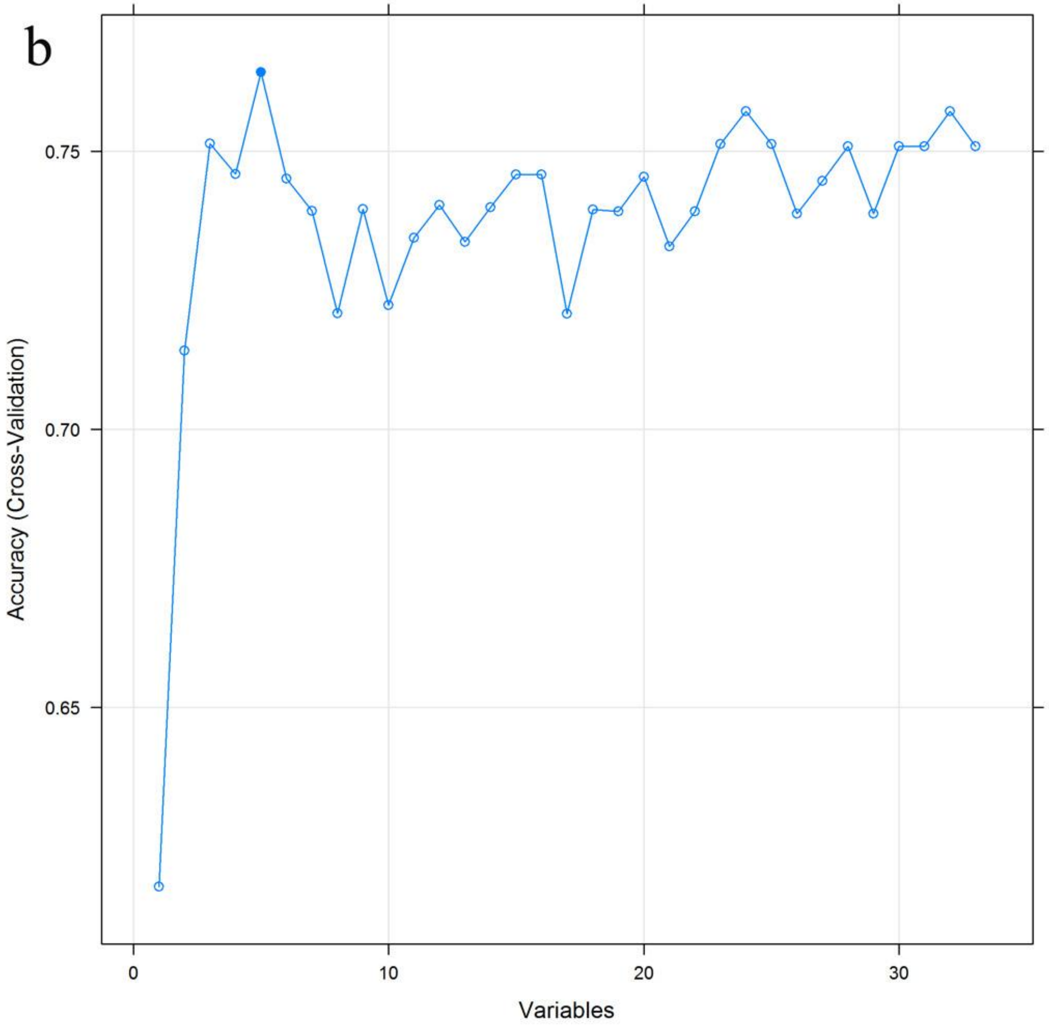

Top 5 variables: CTC-260E6.6, LOC100288637, RP11-48B3.4, C1orf132, EP300-AS1

Figure S2

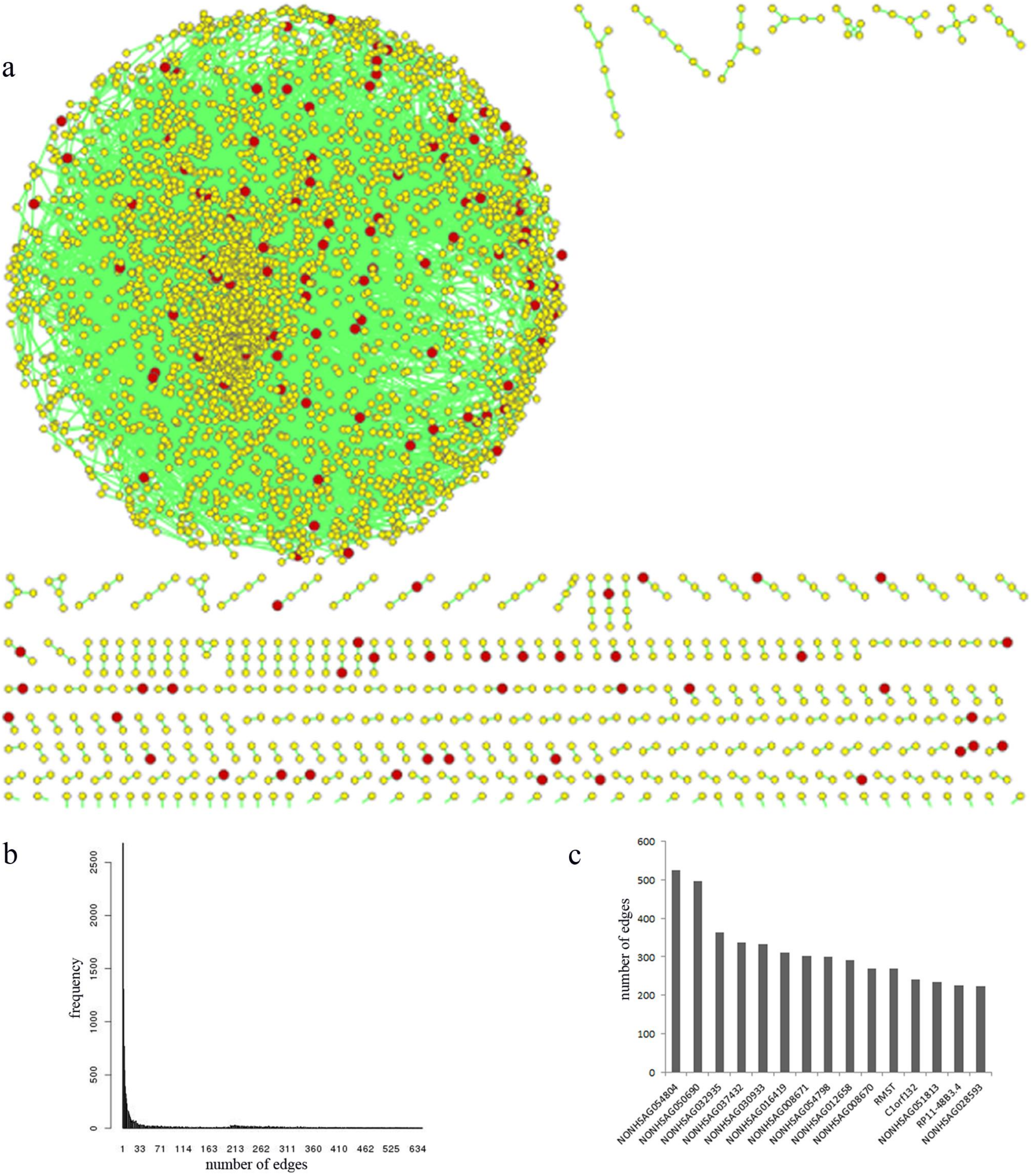
a
b
frequency
0
500
1000
1500
2000
2500
1
33
71
114
163
213
262
311
360
410
462
525
634
number of edges
c
number of edges
0
100
200
300
400
500
600
NONHSAG054804
NONHSAG050690
NONHSAG032935
NONHSAG037432
NONHSAG030933
NONHSAG016419
NONHSAG008671
NONHSAG054798
NONHSAG012658
NONHSAG008670
RMST
C1orf132
NONHSAG051813
RP11-48B3.4
NONHSAG028593

Figure S3

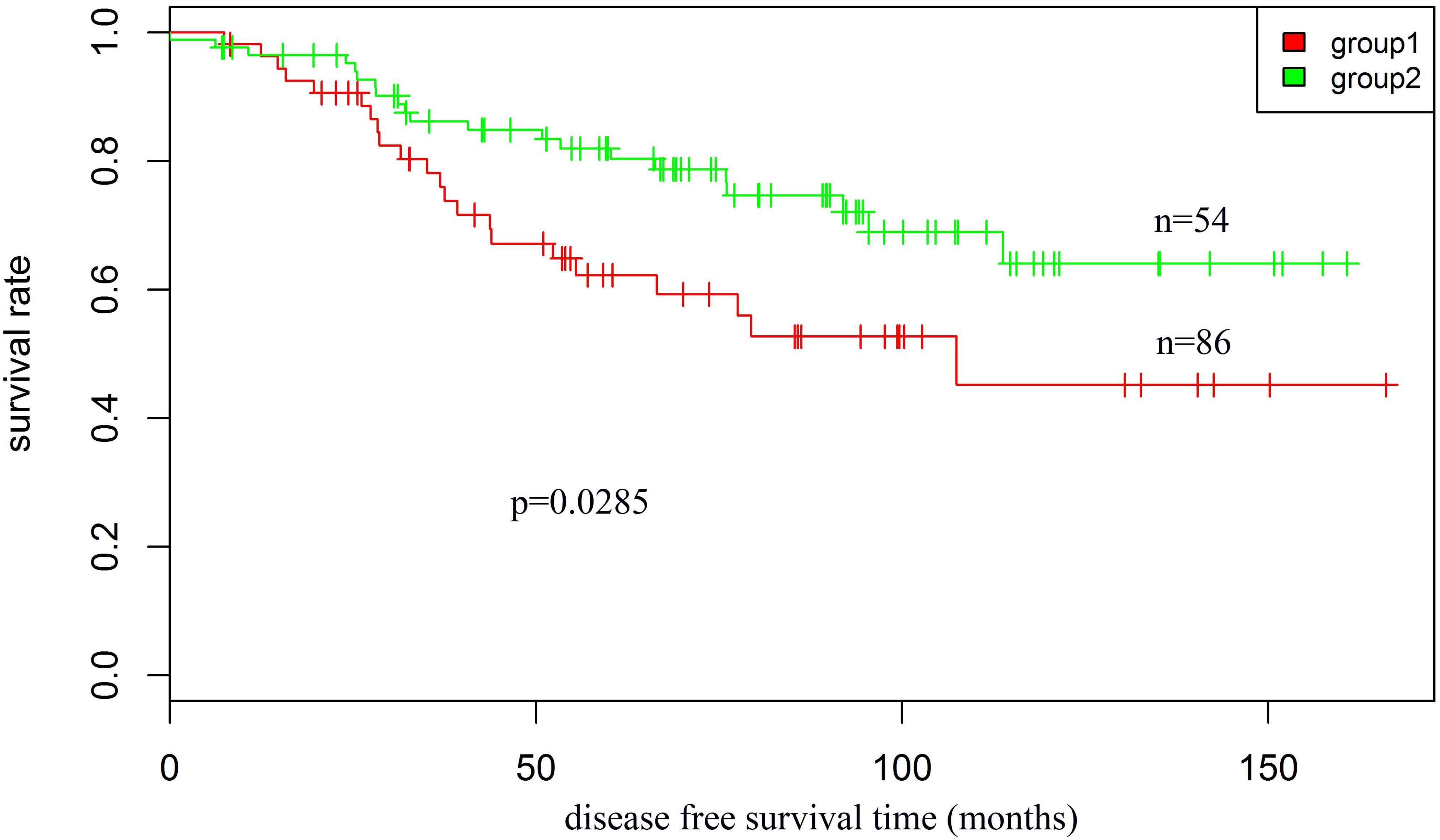

group1
group2
n=54
n=86
p=0.0285
survival rate
1.0
0.8
0.6
0.4
0.2
0.0
0
50
100
150
disease free survival time (months)

Table S1. Expression profile of 2812 lncRNA genes.

	ethanol_ethanol_1	ethanol_ethanol_2	ethanol_ethanol_3	ethanol_ethanol_4	ethanol_ethanol_5	ethanol_ethanol_6	ethanol_ethanol_7	ethanol_ethanol_8	ethanol_ethanol_9
NONHSAG000057	5.40996453	5.30842055	5.32115584	5.58305352	5.45609102	5.37690918	5.37393947	5.18350302	5.50040479
NONHSAG000058	5.92457229	6.30761057	5.92916791	6.19915334	6.29469224	6.10077128	6.14736374	6.04383186	6.04955028
NONHSAG000070	6.76601366	6.93625966	6.84788051	6.93864333	7.11998003	6.95612844	7.06680535	7.09734489	7.0372319
NONHSAG000071	5.38552133	5.43054644	5.4566588	5.50476647	5.30105878	5.54252436	5.39308488	5.50826515	5.56438466
NONHSAG000075	6.1867931	6.27602938	6.25710905	6.33632781	6.20186076	6.16570522	6.44450523	6.21385822	6.2133445
NONHSAG000111	3.77037336	3.81476762	3.83784528	3.84541243	4.06110475	3.83899014	3.79511312	3.84448364	3.80844982
NONHSAG000113	5.88814663	6.07691242	6.11580772	6.07693621	5.98681529	6.12295406	6.07867717	6.04094728	5.75290582
NONHSAG000117	6.592963	6.72225065	6.59837069	6.59002669	6.47562048	6.63496342	6.52147768	6.74445506	6.30015609
NONHSAG000134	3.17970533	3.16072596	3.12881691	3.42637504	3.49930119	3.14719908	3.21941208	3.04083707	3.37295175
NONHSAG000139	5.51596934	5.39236747	5.66001706	5.83027398	5.74954861	5.69372062	5.66832035	5.66866475	5.86576567
NONHSAG000169	3.2536015	3.18423617	2.94205224	3.55767694	3.65573836	3.23875083	3.45448748	3.38462841	3.33479758
NONHSAG000174	4.75962423	4.79758773	4.84432023	4.60652683	4.70665799	4.6029105	4.79283686	4.72539923	5.09843955
NONHSAG000181	2.89416901	3.12651819	2.8417443	2.98637827	2.9632081	3.0538465	2.90553559	2.85749407	2.9695055
NONHSAG000185	3.9868267	3.90842957	4.22308426	4.23357421	4.21003339	3.85709075	4.05509151	4.25786444	4.14535618
NONHSAG000199	6.33865835	6.30611863	6.47217795	6.24321936	6.40227744	6.39437151	6.34267381	6.24321936	6.52267577
NONHSAG000202	3.27722961	3.15405782	3.47515416	3.43082712	3.14786881	3.20395607	2.89485173	3.07875702	2.91913617
NONHSAG000215	3.55684071	3.4146553	3.61609864	3.61053301	3.71751957	3.62282817	3.46264353	3.57770198	3.48806894
NONHSAG000222	3.9954388	3.79803663	3.8530356	3.8237387	3.74495648	3.78261914	3.76182932	3.79943129	4.44932641
NONHSAG000254	6.16315109	6.38637334	6.13993631	5.93064831	5.94292627	6.2154011	6.24593225	6.38823892	6.03172611
NONHSAG000264	3.92815805	3.54585229	3.97933179	3.71024635	3.70130866	4.09190468	4.03905252	3.77603067	4.05589496
NONHSAG000267	5.29021524	5.08439098	5.22072651	5.15702505	5.26038692	4.95198973	5.18631882	5.26073274	5.5264225
NONHSAG000310	6.70601363	6.57191305	6.68318103	6.70870922	6.60345999	6.75805389	6.88798806	6.84877201	6.77389924
NONHSAG000312	4.9866955	4.58441805	4.86283399	4.93608262	5.12601901	5.09290071	5.01892911	4.64461974	5.20450618
NONHSAG000322	2.99013254	3.2711725	3.05343844	3.23041329	3.1206092	3.01202788	3.14508294	2.9573645	3.01860822
NONHSAG000329	7.48332306	7.57544407	7.39981406	7.27706528	7.5577686	7.44038568	7.6448477	7.59935559	7.50225405
NONHSAG000345	9.14881554	9.19135507	9.13617378	9.25018143	9.12289906	9.21215265	9.15169172	9.14323697	9.00022037
NONHSAG000364	5.58571636	5.48476713	5.6859101	5.60215068	5.50114909	5.36637355	5.548242	5.37121347	5.46522348
NONHSAG000381	4.2611789	4.17514809	4.45998471	4.11124959	4.4642961	4.1979344	4.25396484	3.89264569	4.58114379
NONHSAG000395	4.65447365	4.5473528	4.28376063	4.40157134	4.51630958	4.50760046	4.56777906	4.48676857	4.47578162
NONHSAG000423	2.5554503	2.70899027	2.7275368	2.57158327	2.65950129	2.92169987	2.61501003	2.64714009	2.73968694
NONHSAG000442	8.86594871	9.01271702	8.85736146	8.88730757	8.92373958	8.87519824	8.95406369	9.01384718	8.86348391
NONHSAG000476	5.79207574	5.71114572	5.952527	6.25859334	6.08569988	6.18980262	6.0564098	6.09616283	6.08181083
NONHSAG000490	2.78093399	2.72122609	2.86254835	2.76985748	2.80012421	2.78936234	2.56436639	2.61119396	2.80076486
NONHSAG000522	3.12078298	2.88361477	3.24867601	3.40187666	3.11478218	3.31428668	3.25233371	3.11080979	3.48563168
NONHSAG000529	4.45506057	4.04026497	4.31278797	4.22841164	4.53503816	4.02475621	4.49148023	4.16416978	4.2250267
NONHSAG000546	5.12238819	5.37113655	5.26005455	5.4093393	5.20928591	5.51804764	5.53241501	5.26722644	5.46078238
NONHSAG000628	4.32144724	3.99220456	4.34556407	4.09280157	4.28138921	4.55393968	4.77034505	4.22300631	4.89436338
NONHSAG000648	5.61211683	5.67719335	5.61046433	5.92737134	5.62709033	5.69159127	5.6628498	5.64359716	5.37416097
NONHSAG000655	4.64326464	4.67678723	4.75751425	4.50355723	5.05461727	4.6241	4.51917564	4.73393529	4.56486115
NONHSAG000666	5.26483107	5.19399415	5.39414869	5.04420626	5.43547008	5.13168112	5.35617545	4.83238529	5.66682659
NONHSAG000716	4.08358933	3.68104163	3.94959652	3.85968934	4.08839776	3.99913423	4.04974791	4.00109303	4.26111938
NONHSAG000738	3.93302401	3.96401327	3.93639403	3.69380485	4.30995598	4.01183663	3.76298684	4.16304772	4.34466327
NONHSAG000751	2.85643282	2.84314585	3.1010264	2.91560538	2.87277369	2.96929711	2.97401765	2.77928627	3.08743617
NONHSAG000769	4.58610881	4.42926981	4.73103511	4.56316815	4.79824638	4.67405151	4.49088807	4.54988081	4.59887472
NONHSAG000778	2.34125207	2.90752554	2.58478554	2.67313563	2.76795238	2.53571498	2.76155775	2.72315507	2.56123209
NONHSAG000812	3.77763969	4.05019419	4.09506567	4.17057023	3.94779147	4.2738133	4.47122619	4.08219672	4.33725742
NONHSAG000824	2.7524731	2.87887905	2.75849514	2.80255616	2.93062928	2.81487891	2.89981487	2.6958391	3.01664959
NONHSAG000847	3.7928095	3.64931402	4.02913909	3.92257776	3.92163354	3.83605114	3.97820342	3.72626365	4.28156273
NONHSAG000884	3.68260372	3.55963649	3.48873565	3.92361289	3.34759284	3.635617	3.61166007	3.71543205	4.04382817
NONHSAG000939	4.21500575	3.98675734	4.01528522	4.3480007	3.89871934	3.6112595	4.26048337	3.97829731	4.36149261
NONHSAG000946	5.8758859	5.88216947	5.98532946	5.72919144	5.94221658	5.71660294	5.60021795	5.81403638	5.85761063
NONHSAG000965	3.29431073	3.27547484	3.29912543	3.46785016	3.48921591	3.21614941	3.28857353	3.31439253	3.23034366
NONHSAG000972	2.55337421	2.61576244	2.72313794	2.5954172	2.72761565	2.49767043	2.60033512	2.35080188	2.79438849
NONHSAG000976	4.96533236	4.73912144	4.91752632	4.70851221	4.91683248	5.17380256	5.18085526	5.2088944	4.619519
NONHSAG000979	4.19664274	4.05908102	4.15912631	4.1288095	4.19403309	4.38497731	4.20208714	4.052028	4.17835839
NONHSAG001012	9.02317662	9.29143686	8.8541989	8.8527235	9.01591637	8.94050706	8.95816536	9.00000145	9.0486511
NONHSAG001018	2.33670522	2.22063043	2.34068944	2.60903096	2.44765611	2.40149702	2.43904531	2.31901953	2.34113249
NONHSAG001041	5.19843129	5.32887617	4.82413309	5.09141438	5.05204139	5.33836336	5.01355957	5.1632867	5.08865976
NONHSAG001043	5.05893176	5.20712732	5.33445129	5.51107707	5.29466558	5.60322607	5.53310801	5.36031863	5.29976166
NONHSAG001075	3.29160242	3.42722993	3.34042296	3.46593459	3.48875248	3.65411633	3.19412977	3.43632679	3.5113316
NONHSAG001146	7.79050021	7.90733491	7.68302961	7.81849736	7.60837951	7.87749284	7.73413441	7.85850572	7.7396198
NONHSAG001183	2.36045813	2.66425069	2.43458715	2.32882706	2.48987728	2.40402888	2.52562865	2.57942873	2.35755997
NONHSAG001185	2.94848545	2.89343926	3.15099302	3.22428601	3.01284744	2.66745573	2.85695532	3.21564346	2.91138538
NONHSAG001211	3.17948543	3.30799227	3.57921011	3.42917273	3.38763574	3.10810699	3.24755622	3.05526336	3.44567369
NONHSAG001222	4.27330376	4.41163161	4.35202165	4.51883245	4.6310685	4.81062412	4.71539346	4.28849013	4.38490794
NONHSAG001244	3.40449612	2.9715598	3.20040557	3.41966375	3.58562761	3.26405191	3.0631833	3.11305295	3.2344378
NONHSAG001246	5.22427341	5.34558277	5.4066026	4.93736078	5.31903673	5.34569943	5.28112989	5.33774002	5.74495108
NONHSAG001247	5.05123083	5.1941679	4.95916444	4.89141148	4.97341632	4.90223491	4.98773598	5.03763788	4.939435

NONHSAG001264	3.66798875	3.66585096	3.8817941	3.76950641	3.76680395	3.76124383	3.70673258	3.74781267	3.77621792
NONHSAG001304	3.31056672	3.33470334	3.41839024	3.24355437	3.24229753	3.3043169	3.26552035	3.17951087	3.28540357
NONHSAG001323	4.87988844	4.66427402	4.43952529	4.60069395	4.69120449	4.76724384	4.52985146	4.17503303	4.56082391
NONHSAG001341	2.8370958	2.76674274	2.81778458	2.90481797	3.04335726	2.63610717	2.88309141	2.8863159	2.93079825
NONHSAG001391	4.28470921	4.69476658	4.29245127	4.5200869	4.47785632	4.23724012	4.49353476	4.36254566	4.34778536
NONHSAG001489	2.83910557	2.74745803	2.73198166	3.15313183	2.85042461	2.97056479	2.82890939	2.68008716	2.95309451
NONHSAG001510	4.98419844	4.80239285	5.01950485	4.86079909	4.89301877	4.99681213	4.99062259	4.80558548	4.85619093
NONHSAG001530	6.38400401	6.53680664	5.99967429	6.66230818	6.47700384	6.47835885	6.50182439	6.4348008	6.27034543
NONHSAG001569	2.32605166	2.45906439	2.51905005	2.63973065	2.62873001	2.32383002	2.47191941	2.26941916	2.83172785
NONHSAG001574	3.8023418	3.72976586	3.57916748	3.52032512	3.87148514	3.73529106	3.70884458	4.07944082	3.90273814
NONHSAG001576	3.36282079	3.12945171	3.26953259	3.3381234	3.29142561	3.30225056	2.78257529	3.10420405	3.46780612
NONHSAG001587	3.26416476	3.11864313	2.84462291	3.1228446	3.14232676	3.56185137	3.23606431	3.23249408	2.69290294
NONHSAG001597	2.72242439	2.81440445	2.88989219	2.7543562	2.95000522	3.18263029	2.95492939	2.95804959	3.1891167
NONHSAG001601	4.12802561	4.03404207	4.27384768	4.11010624	4.1985571	4.30281401	4.29492361	4.13307566	4.10517081
NONHSAG001622	6.43300647	6.46722185	6.17406808	6.34786323	6.08787824	6.28348301	6.18641996	6.3078053	6.05180793
NONHSAG001627	5.2706484	5.07446248	5.07996203	5.04876911	5.19176942	5.05901268	5.06857644	5.34976456	5.2833899
NONHSAG001640	4.268451	4.05589752	4.27384656	4.41205006	4.17454245	4.15673122	4.25075067	4.06552529	3.98796021
NONHSAG001660	5.00924235	5.09661842	5.33039423	5.22490878	5.00137919	4.93071237	4.90233057	4.90523316	5.31715905
NONHSAG001683	4.20288798	4.06904759	4.18816766	4.17057023	4.0652102	4.24797138	4.40009318	3.97555359	4.23025037
NONHSAG001696	6.12079026	6.45633079	6.1134921	6.338882	6.36993737	6.3691779	6.07050586	6.02692516	6.06466904
NONHSAG001722	4.1609801	4.21139182	4.47000698	4.31261833	4.53546005	4.40956787	4.45599213	4.47624005	4.6976841
NONHSAG001723	3.38099704	3.35995421	3.29296227	3.50839608	3.18457	3.31069477	3.4815003	3.27957194	3.4656055
NONHSAG001768	2.7891082	2.33858839	2.51955331	2.9971658	2.3203963	2.7822199	2.7789496	2.41016217	2.60193475
NONHSAG001788	2.25402432	2.54984962	2.5263152	2.53386054	2.46944796	2.56424435	2.45340601	2.65944142	2.15091778
NONHSAG001803	2.87943922	2.51790641	2.64460777	2.74232374	2.68162625	2.82840727	2.95234112	2.88341511	2.72452136
NONHSAG001824	2.6940849	2.58580243	2.63328193	2.43587083	2.69685542	2.51447182	2.64227162	2.59016006	2.64937656
NONHSAG001832	2.84485648	2.68359294	2.61041843	2.74525846	2.72036297	2.72911446	2.78610003	2.56092266	2.56645411
NONHSAG001845	2.21923185	2.42193528	2.42880458	2.3993033	2.39815812	2.16862215	2.48324127	2.42453865	2.52957208
NONHSAG001849	10.94298972	10.74509991	10.78093656	10.64671279	10.95162391	10.66924205	10.86926766	10.76520363	10.75832063
NONHSAG001855	2.93049058	2.76039605	2.87792207	2.73360744	2.78129708	2.93346984	2.95397747	2.71678649	2.83697636
NONHSAG001898	3.09671245	3.07641854	2.94936565	2.91560538	3.18528359	2.83276796	2.92283417	2.87756919	3.18385427
NONHSAG001910	2.71448979	2.76279221	2.99709732	2.93158875	2.97275458	3.02453756	2.70801744	2.83188564	2.77856762
NONHSAG001920	2.64315959	2.46046169	2.83781338	2.60826052	2.7356423	2.690061	2.60928774	2.75287598	2.62217822
NONHSAG001926	2.86611436	2.68376919	3.08358443	2.57668969	2.78054682	2.86392395	2.67110832	2.84354208	2.9018614
NONHSAG001938	3.11176199	3.00852762	3.0596699	3.39049502	2.98751939	3.05521599	3.05193569	3.14038312	3.15902366
NONHSAG001941	3.44975593	3.4926975	3.4722255	3.50126776	3.46260633	3.56233596	3.55235006	3.51058824	3.5531601
NONHSAG001965	8.48215579	8.46905617	8.35801299	8.18012785	8.47816498	8.12731902	8.43595957	8.36810663	8.58377022
NONHSAG001966	8.69470419	8.99116579	8.5878483	8.13786045	8.40812076	8.62237124	8.630668	8.61919941	8.68889262
NONHSAG001970	3.38262996	3.35481709	3.36138695	3.26651875	3.32611365	3.35060511	3.24604088	3.19056721	3.0929692
NONHSAG001984	4.18592967	4.37828985	4.40819948	4.45906623	4.3632346	4.44094038	4.40201651	4.3775123	4.64474854
NONHSAG001992	4.26333348	4.4719475	4.16115071	4.23452513	4.12306688	4.4117117	4.45955961	4.39613938	4.21352501
NONHSAG002015	3.45079857	3.16625037	3.49366727	3.74579901	3.41408158	3.70265735	3.39042841	3.42144213	3.50024761
NONHSAG002029	2.96558091	2.93317084	3.04824665	3.10186721	2.8763742	2.92485344	2.91173692	2.96451033	3.11755196
NONHSAG002048	2.26543877	2.40694295	2.3051124	2.50784786	2.26554431	2.26143522	2.28085107	2.43904531	2.30368249
NONHSAG002049	4.36135683	4.35952399	4.44032089	4.59293933	4.21898684	4.46146242	4.30991237	4.37027568	4.2728342
NONHSAG002063	3.82878325	4.10081555	4.07521837	4.06884448	4.14998948	3.90790709	4.12756748	3.89306344	4.02049604
NONHSAG002096	3.9065542	4.03833117	3.85328045	3.9753272	4.13870071	4.28038988	4.09330991	4.11158612	3.98512401
NONHSAG002128	3.94934306	3.96763288	3.84815697	4.00799455	3.96923938	3.94870989	3.95398196	3.94423446	4.02316445
NONHSAG002154	2.25402432	2.52655827	2.32395806	2.33368383	2.27492035	2.34757692	2.51141759	2.35548544	2.3997424
NONHSAG002156	4.81264261	4.61429711	4.88526594	4.67482363	4.86334378	4.79970065	4.79182698	4.65528036	4.77571166
NONHSAG002177	2.72969136	2.85585124	2.66478266	2.70823018	2.79069138	2.71593523	2.5821651	2.53802109	2.89106259
NONHSAG002178	2.201867	2.12591989	2.21413798	2.21133279	2.13596969	2.248685	2.11807156	2.110786	2.02288323
NONHSAG002204	3.64678594	3.71686944	3.41142147	3.47194118	3.53772194	3.74015946	3.55498927	3.61836647	3.84820863
NONHSAG002211	2.56212399	2.44297059	2.54433333	2.62232188	2.56001005	2.56532209	2.51527751	2.55315007	2.60921004
NONHSAG002218	2.29053862	2.21607697	2.31293721	2.36888778	2.28110339	2.36904302	2.39043114	2.44434096	2.44312647
NONHSAG002223	4.23851547	3.91840797	4.15358316	4.20814639	4.26872824	3.64675076	4.30196488	4.12091927	4.35823638
NONHSAG002277	4.76947993	4.41847085	4.53313035	4.35878461	4.68997288	4.61849131	4.97119529	4.4613004	4.93164627
NONHSAG002281	4.64028955	4.21229416	4.38369871	4.16554751	3.96745483	4.41959847	4.02587096	3.9776738	4.32930068
NONHSAG002337	4.19499796	4.10368771	4.0332837	3.5859572	3.92344069	4.12471526	3.74702401	4.29193415	3.5995128
NONHSAG002360	5.66748659	5.24765141	5.45365854	5.39669078	5.79738317	5.59330299	5.62098103	5.64723653	5.85350915
NONHSAG002376	2.29568771	2.53755163	2.45771525	2.3018429	2.53504742	2.27533223	2.2760031	2.16723616	2.33905518
NONHSAG002377	5.30480308	5.546077	5.37744903	5.56425175	5.51653783	5.40757782	5.55845663	5.5521334	5.48244882
NONHSAG002389	3.17075331	3.24445131	3.40437838	3.36114015	3.59181909	3.3382559	3.43763256	3.63355855	3.61643482
NONHSAG002392	2.63483747	2.90137358	2.77870232	3.09785616	2.89485173	2.86461713	2.62681177	2.60000583	2.68064247
NONHSAG002406	3.09600385	3.17362139	3.21271549	3.45299449	3.2909284	3.21710645	3.21777834	3.18972731	3.39489882
NONHSAG002410	4.977711	4.82715243	4.82693166	4.80464108	4.68546229	4.82759382	5.12054321	4.80719639	4.5445216
NONHSAG002419	4.3172635	4.50264084	4.46794278	4.82192195	4.96624651	4.54841543	4.4468999	4.62336972	4.82955317
NONHSAG002421	3.16715384	2.97613068	3.05118728	3.23832663	3.31439253	3.03723582	3.25045205	2.98033939	3.20803631
NONHSAG002440	5.61911199	6.03912858	5.93178908	5.65153214	6.29031037	5.94885801	5.83186674	5.83498854	6.05954374
NONHSAG002455	2.9485672	2.92493313	3.11879548	3.00383074	3.15112215	2.96589468	3.08595533	3.08201036	3.12335015
NONHSAG002458	3.09251043	2.92491322	3.12371814	2.97210831	3.06101566	2.98032546	3.14126736	2.87601985	3.03032502

NONHSAG002465	6.56207173	6.74213701	6.48846973	6.10404873	6.45418916	6.5454919	6.71354631	6.5694308	6.39747404
NONHSAG002516	2.82309003	2.77744034	3.07930076	3.28772754	3.16589038	3.26627614	3.17934876	2.9221564	3.14615314
NONHSAG002535	3.86505775	3.78979732	3.53784085	3.82670941	3.71465503	3.68725691	3.67855676	3.78520955	3.57765248
NONHSAG002538	5.21559093	5.20763335	5.20103344	5.12297655	5.1608825	5.14586607	5.14540068	4.97429661	5.52740201
NONHSAG002549	4.25653799	4.3545067	4.49332564	4.40462158	4.56393617	4.40169359	4.37916382	4.31760173	4.5944527
NONHSAG002555	2.82800764	2.67989181	2.85496962	2.81662407	2.8608223	2.66729104	2.83784513	2.72450745	2.78914008
NONHSAG002615	7.53351866	7.25557813	7.51628866	7.62835016	7.31074366	7.49889482	7.06378482	7.29185203	6.9568241
NONHSAG002728	4.56723189	4.34470923	4.02085094	4.12298123	4.32861228	4.41077084	4.57795642	4.3242452	3.87115769
NONHSAG002758	4.64026488	4.86774386	4.9465864	4.55239089	5.08641771	4.86289231	4.97989123	5.17967245	5.02690682
NONHSAG002759	2.93187196	2.96206187	3.05983835	3.01822662	2.79264776	2.9435413	2.93011062	2.89280616	2.89254744
NONHSAG002764	3.17461342	3.32131317	3.09375725	3.21689085	2.91089489	2.93391873	3.08291097	3.59794639	3.40783396
NONHSAG002857	3.53855459	3.39134474	3.55515759	3.52817651	3.61146285	3.80318798	3.56259562	3.74994619	3.56879286
NONHSAG002865	4.06754103	3.58482827	3.73141855	4.01264949	4.1056659	4.05042174	3.8258005	3.6414113	4.29249152
NONHSAG002891	7.11978979	7.29697666	7.0520991	7.04772729	7.24980229	7.32534795	7.30756554	7.35078888	7.27220937
NONHSAG002908	6.3670742	6.53455847	6.43976207	6.55039251	6.24610473	5.95913732	6.41954759	6.6050899	6.40368419
NONHSAG002936	7.76580626	7.89340579	7.79723195	7.78332906	7.7360157	7.91062975	7.91886125	8.02846626	7.97767487
NONHSAG002956	3.99474385	3.83835674	3.91524775	3.97180465	4.05457555	4.10005283	4.12630387	4.11193845	4.12236875
NONHSAG003018	4.6501918	4.53717936	4.66397719	4.68486891	4.81846839	4.73655626	4.72393952	4.75076593	4.75406022
NONHSAG003022	4.51049107	4.33971947	4.64017773	4.77070129	4.59018359	4.77320325	4.76664267	4.60199506	4.72270799
NONHSAG003057	7.17978525	7.14634739	6.92486072	7.33669793	7.03634211	7.10566468	7.19735417	7.27702975	6.81463506
NONHSAG003089	4.06120743	4.06764492	4.19729982	4.00367822	4.69653883	3.88695954	4.40279426	4.23097875	4.9177626
NONHSAG003123	2.52667025	2.54492736	2.60136502	2.51193666	2.8897334	2.7721872	2.26757386	2.25457605	2.45550852
NONHSAG003195	3.63949639	3.76306689	3.77457709	3.6579683	3.85871642	3.93528905	3.86024445	3.81367109	3.96836345
NONHSAG003198	3.30562449	2.94542085	3.19372985	3.12414139	3.51159754	3.32557607	3.24009801	3.15639778	3.17252813
NONHSAG003202	7.34730617	7.30497306	7.20343726	7.28084764	6.9633397	7.19402349	7.15279075	7.09823748	6.9927758
NONHSAG003261	3.70776828	4.31350841	3.88931875	4.2529204	3.99644222	4.34209727	3.9873452	3.9525516	4.14096556
NONHSAG003289	2.72646729	2.76162678	2.73844611	2.84894184	2.82535534	2.82759641	2.81624326	2.960048	3.0789561
NONHSAG003322	6.05021244	6.21586208	6.04219542	6.10364834	6.09981482	6.70171428	6.42553617	6.30986465	6.31387775
NONHSAG003325	6.04075765	6.1914382	5.79738771	6.16926728	5.9451951	6.23067085	6.50483463	6.22006528	6.46661403
NONHSAG003332	3.77593053	3.68719629	3.76600679	3.86221536	4.08966302	3.71947681	3.77789803	3.82913789	3.72174
NONHSAG003339	2.6981018	2.29328658	2.65722437	2.376165	2.60130139	2.82694646	2.57866525	2.63221041	2.33230557
NONHSAG003345	4.20728596	3.86271124	3.40899311	3.69748846	3.98484686	4.09046216	3.89178532	3.62131707	3.76971866
NONHSAG003405	3.1031992	3.07396747	3.02091269	3.05043224	3.18366876	2.9168617	2.88442738	3.24976798	2.79653889
NONHSAG003407	7.419053	7.11193647	7.38838588	6.71138456	7.7288517	6.86981345	7.49780316	7.28109061	7.54403602
NONHSAG003412	2.53568727	2.44163878	2.61501003	2.45092231	2.39386833	2.43455754	2.61349677	2.10383497	2.51618556
NONHSAG003464	3.68903848	3.82579696	3.85232431	3.96717558	3.99914301	3.733663	3.88026691	3.67516063	3.83013786
NONHSAG003486	5.48953104	5.17501498	5.34254465	5.1738367	5.40218794	5.50243963	5.6271566	5.48721208	5.82683355
NONHSAG003512	3.82734478	4.02416579	4.00149911	3.92100498	4.18879973	3.54867357	4.47183223	3.95909459	4.70861635
NONHSAG003533	3.84415612	3.67143893	3.99404491	3.64963418	3.64788887	3.63563664	3.67384304	3.77673164	3.93328531
NONHSAG003536	4.02163784	3.79208478	4.09015322	4.14830305	3.87129146	3.80466562	3.9416577	3.9815608	3.90049147
NONHSAG003539	3.09645745	2.85295086	3.11240535	3.11072144	2.94297566	3.22963708	3.21579183	3.08156124	3.17937674
NONHSAG003554	2.41062337	2.34116057	2.43062965	2.36586175	2.48284625	2.37373463	2.44849748	2.40844711	2.5723663
NONHSAG003555	5.5644046	5.65749749	5.9625714	5.53915306	5.74708679	5.48584399	5.6382755	5.81234908	5.74680603
NONHSAG003562	3.04961294	3.13245173	3.00521348	3.22339574	2.85031962	3.20287097	2.99820762	3.03038466	3.10231277
NONHSAG003567	2.25639243	2.19876791	2.17093092	2.27674215	2.24269553	2.35307995	2.17097885	2.25022452	2.31173228
NONHSAG003604	4.21766362	4.25485196	4.43783277	4.22732327	4.21368764	4.02344198	4.13589638	3.98925293	4.31048092
NONHSAG003613	4.7085864	4.43386167	4.59668008	4.69415481	4.76268389	4.81355585	4.66706747	4.54670993	4.34912013
NONHSAG003615	2.7553375	2.77600569	2.80350352	2.90635417	2.51389731	2.7533156	2.80044819	2.86096201	2.60099888
NONHSAG003627	4.1492054	3.6385225	3.97025251	4.33037382	4.34218736	4.3390215	3.89784041	4.06468948	4.02151102
NONHSAG003635	4.48962423	4.64384482	4.60673005	4.8112537	4.75644787	4.73907475	4.49518131	4.75631773	4.79837402
NONHSAG003645	4.59625922	4.79922851	4.85472566	4.31906075	4.69473507	4.62642335	4.60890588	4.60613977	4.75927968
NONHSAG003668	2.56743753	2.60496919	2.3488071	2.45965585	2.531842	3.03524146	2.58943916	2.46256243	2.55637076
NONHSAG003758	2.15969548	2.09579947	2.15065028	2.10908578	2.17374732	2.36435738	2.22590417	2.25402432	2.2441939
NONHSAG003765	2.32041226	2.29405622	2.2521118	2.273023	2.22805304	2.29689303	2.44536865	2.43660571	2.16226612
NONHSAG003799	3.28479958	2.88811889	2.9987424	3.26288161	3.07739505	3.27962509	3.12808532	3.19694493	3.07738023
NONHSAG003829	3.49666186	3.44952052	3.78939875	3.59336363	3.46837319	3.53775714	3.39322782	3.67201492	3.64122667
NONHSAG003840	2.81704522	2.85300498	3.04945624	2.68807159	2.98061625	3.1872773	2.89485173	3.19160852	3.32846301
NONHSAG003843	2.10161499	2.47809934	2.41183836	2.30593555	2.04071091	2.13271625	2.18572295	2.34272657	2.26067581
NONHSAG003854	2.84236219	3.17461342	3.26623436	2.79456987	3.08730987	3.14717175	3.24176875	3.1301462	3.07121401
NONHSAG003880	5.11039539	5.07479365	5.24296541	5.07429614	5.12980504	5.06507016	5.30542114	5.00536663	5.19542572
NONHSAG003894	3.57432702	3.39119887	3.62319256	3.58772895	3.42790995	3.61568651	3.4865204	3.42101719	3.2918699
NONHSAG003900	2.87243188	2.77723005	2.7917732	3.14107729	2.77710478	2.8920863	2.6869794	2.95423366	3.32961553
NONHSAG003927	6.48087381	6.40329034	6.16038189	6.3077619	5.89610269	6.51842231	6.44346007	6.8785682	6.68697457
NONHSAG004034	4.68446151	4.1811832	4.8434995	4.98749632	5.07098778	4.7935632	5.02777412	4.60472876	4.7174776
NONHSAG004049	2.67682451	2.55581464	2.5081571	2.42004864	2.50877794	2.78865565	2.44682791	2.4332594	2.4686249
NONHSAG004092	4.16315871	4.17046876	4.41935457	4.1859277	4.39457188	4.28333828	4.16594303	4.22437244	4.5497631
C1orf132	6.83347691	6.99995038	6.66732665	6.63469756	6.38150803	6.75283963	6.90809854	6.92747958	7.15856892
NONHSAG004140	3.58317199	3.96165286	3.88246334	3.99527987	3.84992671	3.94051168	3.97068841	3.79590634	3.90542837
NONHSAG004156	3.31298187	3.45582492	3.4939143	3.50742507	3.53839444	3.69994456	3.53499181	3.55837656	3.39279514
NONHSAG004159	5.65074104	5.63126378	5.55884252	5.1938743	5.81807174	5.33795349	5.27174138	5.4558033	5.34739311
NONHSAG004205	2.84903076	2.97771332	2.99933236	3.00363781	3.22827373	2.98751237	2.89122741	3.19426995	3.12966465

NONHSAG004221	3.42333261	3.27221271	3.5406404	3.12959281	3.37257134	3.17461342	3.39052491	3.50234847	3.6580714
NONHSAG004235	4.59335361	4.38124825	4.21219469	4.38948252	4.12590248	4.6219106	4.44422771	4.6166465	4.50155778
NONHSAG004261	2.8879734	2.83588656	2.94797169	2.96567448	2.90877002	2.94763114	2.96745658	2.79384073	3.15652796
NONHSAG004265	2.94388653	2.82976169	2.53315332	2.78140396	2.75356636	2.71128886	2.45556147	2.86521424	2.92960627
NONHSAG004289	7.06383114	7.31137297	6.75052103	6.64611334	6.72442414	7.04102978	7.00624437	7.01883853	6.62052489
NONHSAG004292	5.31299634	5.76048368	5.29323564	5.3145106	5.47752929	6.0059313	5.38714707	5.45645447	5.50823318
NONHSAG004319	3.00124583	3.0498004	2.96407466	2.94314296	3.11443669	3.12794863	3.00660173	2.97333336	2.99479116
NONHSAG004322	4.50498134	4.42795119	4.42058768	4.14066445	4.5109958	4.46583442	4.51662994	4.36346628	4.68032699
NONHSAG004342	5.25874569	5.12079666	5.48324648	5.40913215	5.26361738	5.46180724	5.41305467	5.17118905	5.53543904
NONHSAG004374	4.39674943	4.19919847	4.10616205	4.61448463	4.49775415	4.2194696	4.02559004	4.25881946	4.38316939
NONHSAG004468	4.43062905	4.54380486	4.34759656	4.55038003	4.73720049	4.66921275	4.53097211	4.51407343	4.44383136
NONHSAG004469	3.31173999	3.1613993	3.21397687	3.6136115	3.54213796	3.49010609	3.65409036	3.35335481	3.58734869
NONHSAG004473	4.24060404	4.14445395	4.33357593	4.27638402	4.47597668	4.34744013	4.39705556	4.35620226	4.66384017
NONHSAG004494	2.55203853	2.48561153	2.32865754	2.42294136	2.49512283	2.49553803	2.5687782	2.45263033	2.34906783
NONHSAG004522	2.84030626	2.8400113	2.63179896	2.7538697	2.70549327	2.71887739	2.68587367	2.56869258	2.82184853
NONHSAG004544	5.9199245	5.61535234	5.89167008	6.20409549	6.13118768	5.90171153	5.89461596	5.65788732	5.72826492
NONHSAG004554	2.40189516	2.53425408	2.74369461	2.5005415	2.75159894	2.73736564	2.5510598	2.49472937	2.65195427
NONHSAG004567	5.05879431	5.18351387	5.3201177	5.25142701	5.33159982	5.50837172	4.93978456	5.16156044	5.43950703
NONHSAG004569	4.49429966	4.36438018	4.33660985	4.26781692	4.31266228	4.53044045	4.26739889	4.45207067	4.26578122
NONHSAG004584	4.08657709	3.75922647	4.19561307	4.22468183	4.08361657	4.05222103	4.03458649	4.02406099	4.08900632
NONHSAG004588	3.11410232	2.80755618	3.04182538	2.77475645	3.24960507	2.82902183	3.01131699	2.81162593	3.02921495
NONHSAG004592	2.31707566	2.28588975	2.30108304	2.4453448	2.28828488	2.51375909	2.36709657	2.28285078	2.30347336
NONHSAG004614	6.56332234	6.58149609	6.45320039	6.26986666	6.40307216	6.1564748	6.04144162	6.41461452	6.05618414
NONHSAG004619	4.71661686	4.72402934	4.83143586	4.82828584	4.76091573	4.84197291	4.61308337	4.70245226	4.56692859
NONHSAG004632	2.65735784	2.65359187	2.80750452	2.806113	2.85644966	2.611096	2.80485335	2.70082129	2.7990529
NONHSAG004636	3.86576573	3.83352416	4.03935023	4.10873796	3.90570044	3.92492065	3.92081634	3.57027443	4.08967771
NONHSAG004663	3.46049121	3.01679236	3.24028656	3.1419586	3.40681296	3.46561924	3.23300762	3.11276012	3.32286019
NONHSAG004683	3.88620591	3.42127663	3.71348304	3.90944875	3.95420184	4.08942797	4.04861594	3.93708063	4.14698863
NONHSAG004693	3.19857629	2.8537691	2.98551957	3.23786385	3.19088204	3.23573812	3.24168836	3.03694233	3.30345513
NONHSAG004694	4.95018638	4.34154996	4.8813522	4.96841694	5.02460936	4.74872903	4.7510759	5.16922832	4.60101773
NONHSAG004774	2.63383479	2.85357419	2.80527017	2.86161594	2.7553704	2.64441837	2.46439641	2.84769268	3.14215667
NONHSAG004778	2.77269719	2.717867	2.89648541	3.06696891	2.7456564	2.51918519	2.76857447	2.79964669	2.86485911
NONHSAG004794	3.91448765	3.85150644	3.72808215	4.05871809	3.74011162	3.97363355	4.13615341	3.89224806	3.91856496
NONHSAG004884	4.40831698	4.51435684	4.4559002	4.55038492	4.30319861	4.52600843	4.65947616	4.31632209	4.38174978
NONHSAG004887	5.00447722	4.59911938	4.82405097	4.92924047	4.92617731	4.95540565	4.76340394	4.88231364	5.04515625
NONHSAG004895	4.00138227	4.2238784	3.92503668	4.47946647	4.23888061	3.81649029	4.29342578	4.04124771	4.26564702
NONHSAG004899	3.58101492	3.49204569	3.66293625	4.0974493	3.90016228	3.71947681	3.7824799	3.81986569	4.00058291
NONHSAG004902	5.37097303	5.27554407	5.12037609	5.19852553	5.23999862	5.2594961	5.28733954	5.25855555	5.55290329
NONHSAG004908	4.90268147	4.8853698	5.0343845	5.25588202	5.18388245	5.17820719	4.9162242	4.89816002	5.12066417
NONHSAG004919	7.69508008	7.63289167	7.48594094	7.0592599	7.43168759	7.15402365	7.33836958	7.50276962	7.57005237
NONHSAG004920	3.09993464	3.11523782	3.1556226	3.28604795	3.27232917	3.22302382	3.1558965	3.21267843	3.19263094
NONHSAG004943	2.88072542	2.79184924	3.01884483	3.03427789	3.08950323	2.64444413	2.99407859	3.15730867	3.05077233
NONHSAG004961	5.0896539	5.09233598	5.22274069	5.07860036	5.14757338	4.9225053	4.91163379	5.10390663	5.31209646
NONHSAG004977	6.51479	6.7206333	6.6522785	6.51461396	6.42010927	6.54694475	6.52415663	6.41375044	6.42271876
NONHSAG005016	5.64758517	5.6379045	5.71107703	5.76529322	5.74954861	5.86873584	5.88129065	5.74421149	5.9362575
NONHSAG005033	2.34842656	2.5416332	2.34045298	2.20031069	2.51920025	2.26864784	2.41676875	2.32445954	2.51605725
NONHSAG005060	2.43341207	2.51692391	2.46830726	2.77235262	2.5758998	2.50730555	2.59828301	2.44713379	2.55281008
NONHSAG005063	3.02618771	2.95894543	3.21670839	3.14025536	3.29759586	2.99091737	3.3105122	2.93961723	3.16189643
NONHSAG005080	2.82732619	2.6330245	2.68281115	2.83361028	2.36435738	2.58467159	2.52672246	2.51454275	2.70270208
NONHSAG005082	4.30331249	4.20371293	4.32756866	4.20935128	4.56929366	4.1842443	4.4105071	4.07578621	4.27619027
NONHSAG005119	2.77285884	2.49419772	2.72227205	2.59051083	2.95296405	2.79316671	2.86689088	2.86260381	2.74122128
NONHSAG005138	4.74587828	4.56761329	4.65312496	4.86262215	4.85405515	4.66138921	4.65497831	4.62707695	5.08157093
NONHSAG005145	3.09382825	2.87784522	3.08353202	3.36448895	3.02159832	3.06618428	3.18168984	3.25083478	3.0452792
NONHSAG005150	4.73411176	4.63466108	4.57959441	4.98569196	4.59115228	4.49125816	4.94830184	4.57696585	4.86774802
NONHSAG005165	4.93361897	4.85080806	4.98186493	4.77679627	5.10659743	4.38610133	4.96228786	4.91921424	5.21713673
NONHSAG005205	2.88893368	2.71480808	2.61501003	2.90049153	2.87656145	3.01966474	2.91530272	2.58380792	2.84531785
NONHSAG005207	4.4698869	4.34583835	4.40312897	4.56165473	4.36596838	4.29082911	4.59259224	4.46906597	4.51437854
NONHSAG005225	3.29753399	3.46786716	3.27115283	3.43755913	3.26275036	3.4939158	3.4458347	3.69292901	3.20047323
NONHSAG005226	3.13888554	3.10767023	3.21430614	3.25981582	3.18025774	3.04097341	3.16615274	2.99243196	3.15955764
NONHSAG005232	6.2743627	6.43772095	6.19000238	6.35141374	5.90266403	6.08014333	6.24066059	6.34218347	6.17121016
NONHSAG005234	4.04612339	3.75594557	4.15179825	3.96948757	4.00792531	3.94716308	3.66857298	3.75099115	3.95542549
NONHSAG005239	5.43627208	5.5941162	5.46235505	5.33316406	5.2295926	5.27188884	5.34340135	5.42738601	5.23348962
NONHSAG005282	3.53530091	3.42274581	3.57585846	3.52338978	3.7289973	3.64675076	3.48359397	3.91821293	3.68604581
NONHSAG005284	4.46591551	4.70023185	4.04328662	4.71029568	4.36652652	4.46005646	4.774264	5.09646105	4.55648725
NONHSAG005342	5.01010974	5.19082114	5.29024274	5.14851987	5.04390111	5.14057573	5.31180328	5.28303101	4.9558991
NONHSAG005356	2.69289749	2.54826035	2.7408173	2.86652496	2.93451901	2.968357	2.73482385	2.8443743	2.78660015
NONHSAG005375	4.29944798	3.93988588	3.91808997	4.13914143	4.01293422	4.97680422	4.75509487	4.47807079	4.77205887
NONHSAG005386	3.74538216	3.23138573	3.58761441	3.43765317	3.65533712	3.45793081	3.59018022	3.36182108	3.52879517
NONHSAG005407	6.31055965	6.21791964	6.54100161	6.09549251	6.66898015	6.34441368	6.32445405	6.18225404	6.28156789
NONHSAG005420	2.98066819	2.70079751	2.97060434	2.85219174	3.05178401	3.24029832	3.0348995	2.73759472	2.88308245
NONHSAG005464	5.27731975	5.01024017	5.24974759	5.35498795	5.53598244	5.37869859	5.33401449	5.23802997	5.2879144

NONHSAG005471	2.66590677	2.68995667	2.72244386	3.02990685	2.70850987	2.6148776	2.82184128	2.91625613	2.66323299
NONHSAG005501	4.04547028	3.96138616	3.87550957	4.24698363	3.99808212	4.13691143	4.32623637	4.08715987	4.40994883
NONHSAG005503	4.27878336	4.28238335	4.31236747	4.55228133	4.52156446	4.79553615	4.55626949	4.14056369	5.0062425
NONHSAG005505	2.41916816	2.25689442	2.39409774	2.42976638	2.43471409	2.23943055	2.48008557	2.3919004	2.47823731
NONHSAG005523	6.1670304	6.32553961	5.77454873	5.92522052	6.13266226	6.33752311	6.09919315	6.30999712	5.77172351
NONHSAG005543	2.94008784	3.06801892	3.10156628	3.36439959	3.3943255	3.24199474	3.13573168	3.21093419	3.26539074
NONHSAG005545	2.66890614	2.91429577	2.52504889	2.74477882	2.67619179	2.73821231	2.61525991	2.75572219	2.72711576
NONHSAG005589	4.59906311	4.17946324	4.67452826	4.30916686	4.62058683	4.49435478	4.3589702	4.25519773	4.76039001
NONHSAG005603	3.31838887	3.55570395	3.23914748	3.238326	3.22827373	3.44121412	3.41676735	3.26777917	3.24194233
NONHSAG005687	4.57601746	4.41002232	4.78727693	3.69272711	4.85375018	4.45520726	4.52500872	4.4283366	4.67995063
NONHSAG005692	3.17461342	3.0490987	2.9373414	3.18559203	3.36441549	3.185245	3.3698387	3.14181452	3.22123137
NONHSAG005715	6.40254678	6.46299226	6.37955143	6.25219583	6.29336732	6.34698018	6.39910024	6.3870131	6.02014274
NONHSAG005734	4.30771944	4.01750984	4.17722378	4.39180281	4.29656545	4.28919296	4.21330632	4.14269892	4.26721644
NONHSAG005754	4.19259675	4.12960492	4.24108473	4.47683821	4.07762446	4.18277599	3.74485709	3.81441747	3.70707171
NONHSAG005762	3.47003267	3.32921514	3.77874206	3.36367353	3.68368268	3.76633289	3.36167818	3.42620971	3.84648476
NONHSAG005767	3.17461342	3.11719651	3.33011753	3.36477332	3.37488202	3.15323937	3.35199779	3.38219707	3.258766
NONHSAG005812	2.64739777	2.73952633	2.56197472	2.57528482	2.84601793	2.71227971	2.60924227	2.67992362	2.80959805
NONHSAG005834	5.27465478	5.15970836	5.14315363	4.79854493	5.15237741	4.82256931	5.34676458	5.13688504	5.21743268
NONHSAG005850	3.03045761	3.07414915	3.24556107	3.23545164	3.24336612	3.10403184	3.36890484	3.12481343	3.19363869
NONHSAG005870	4.59894037	4.66324534	4.44688934	4.45958168	4.6310685	4.62982852	4.79078638	4.77955631	4.61905897
NONHSAG005912	2.57294056	2.64008	2.66744713	2.59643162	2.6894684	2.47014696	2.72082781	2.77760755	2.44070459
NONHSAG005923	6.50643242	5.9109988	5.90035363	5.94422937	5.80017088	6.43845795	6.73866141	6.46569496	6.33540677
NONHSAG005953	3.61080142	3.41959652	3.69624806	3.65225238	3.82672514	3.67916718	3.82411278	3.66735265	4.21190316
NONHSAG005957	2.86194432	2.87878705	3.06165908	3.08898486	2.86689575	2.95918197	3.08208827	2.74997108	2.92783592
NONHSAG005965	3.00761109	3.18282067	3.11908144	3.03890974	3.27317219	3.18127157	3.29736575	2.97688432	3.13480438
NONHSAG005970	3.324074	3.19305786	3.16035871	3.2296782	3.16311425	3.25332303	3.360141	3.27260522	3.14102799
NONHSAG006053	2.57118433	2.3052707	2.29689303	2.44436115	2.5758998	2.35432506	2.41866264	2.49503075	2.32677161
NONHSAG006081	5.95152059	6.41876617	5.90707996	6.1459491	5.92764569	6.33161393	6.2324873	6.33088505	6.16490264
NONHSAG006083	2.89084665	2.91560538	3.17525382	3.12299601	3.55946647	3.25569947	2.84746976	2.92084871	2.92529681
NONHSAG006087	4.31042875	4.88638535	4.19909449	3.98331627	4.36378082	4.31541081	4.31042875	4.35946057	4.15451158
NONHSAG006140	3.08112023	2.93533931	3.05691401	3.45403643	3.05178401	2.86796109	3.01820672	3.12355535	3.52217765
NONHSAG006147	4.73212447	4.90737265	4.58953329	4.6623354	4.85700414	4.78815389	4.69225595	4.67026105	4.88692512
NONHSAG006149	4.65359633	4.90666493	4.57197166	4.88617789	4.67817396	4.72580196	4.72486947	4.66158537	4.79043014
NONHSAG006187	2.00774858	2.03058891	2.08903341	2.35013428	2.18066289	2.4081385	2.17694197	2.07946214	2.09065037
NONHSAG006196	5.23360403	5.42623623	5.50581501	5.61567042	5.38158989	5.53878137	5.45709063	5.2775389	5.55591211
NONHSAG006210	5.53481741	5.5171514	5.38368151	5.7244134	5.6241533	5.61833217	5.71977977	5.50040479	5.3292337
NONHSAG006244	3.26134799	3.2756744	3.19280318	3.38078692	3.06547715	3.64589844	3.03796553	3.23167934	3.00653859
NONHSAG006270	2.7366492	2.70579298	2.90569801	3.00950833	2.91112144	2.74526321	2.93618169	2.90468451	2.89587867
NONHSAG006274	3.05723792	3.23533824	2.88170361	2.56424435	2.96580813	2.88451456	3.00951203	2.92252374	2.90200098
NONHSAG006311	3.69453771	3.20054086	3.64585364	3.51845787	3.54798461	3.3684377	3.4847217	4.13369689	3.55424115
NONHSAG006312	2.80874323	2.81498352	2.94740021	2.80295322	3.08146895	2.97097547	3.06659651	2.85978714	3.40525038
NONHSAG006326	3.69963466	3.87275442	3.8572742	3.85210182	3.80801646	3.75521878	3.63181378	3.73660564	3.91430276
NONHSAG006406	3.47266057	3.30224018	3.62527969	3.51340343	3.62059784	3.76773322	3.20979371	3.30495221	3.80029689
NONHSAG006407	4.3333253	3.84361032	4.22184516	4.06481821	4.07580986	4.02682196	4.38892084	3.92634751	3.96103088
NONHSAG006417	4.30630405	4.29173422	4.16915673	4.32561741	4.22627709	4.36409737	4.33572081	4.39119809	4.50609678
NONHSAG006458	5.10137355	4.94977045	5.08287307	5.13172113	5.17372953	5.25013665	5.12968802	5.21232562	5.33858573
NONHSAG006506	2.34480777	2.35691176	2.25325335	2.3590135	2.38767648	2.2932777	2.26960998	2.27935441	2.30376346
NONHSAG006557	4.06399513	3.98775458	4.09046216	3.56522572	4.07242725	4.17582973	4.17607687	4.08728858	3.9612709
NONHSAG006570	7.73997005	7.77216453	7.73221206	7.35388586	7.54913061	7.410488	7.19232183	6.84691262	6.70177449
NONHSAG006585	6.65989173	6.55700275	6.72330059	6.33983479	6.95979604	6.81966895	7.04450139	6.58372885	6.79896774
NONHSAG006591	5.33791638	5.16012463	5.23541518	4.88053101	5.09566495	5.03915467	5.80048209	5.49484029	5.29505993
NONHSAG006615	5.38814933	5.46915093	5.28182061	5.54526869	5.34881082	5.60562504	5.56189923	5.39436077	5.3745739
NONHSAG006619	7.54858773	7.63348354	7.27463116	7.49612555	7.42956413	7.60454524	7.46751749	7.50954338	7.48177693
NONHSAG006623	5.36446071	4.98357079	5.27065853	5.43977552	5.57689838	5.35231982	5.3285796	5.01542563	5.34026471
NONHSAG006631	10.67184741	10.38665447	10.34816837	9.69257395	10.52560815	10.08552904	10.51061001	10.17740761	10.46505507
NONHSAG006633	3.86783552	3.9930872	3.96590073	3.86308134	3.85122578	4.08430913	3.98722636	3.89638201	4.10191184
NONHSAG006679	5.72805358	5.98075115	5.97834397	6.14840233	5.82446801	6.00001416	6.17970298	5.96348607	5.72782092
NONHSAG006690	3.65953395	3.88185358	4.10045645	4.07798439	3.55620786	3.94768268	4.03389128	3.63217618	3.88656933
NONHSAG006700	5.60641773	5.66267213	5.54555525	5.79027029	5.51468397	5.7842499	5.39738389	5.29534136	5.14178389
NONHSAG006702	6.09823809	6.04395153	6.03938601	6.25495328	6.21335731	6.2571602	6.14879233	6.17816065	5.98546207
NONHSAG006706	4.48584267	4.47304888	4.52037903	4.36547324	4.51530902	4.49133907	4.44632736	4.34928469	4.40347973
NONHSAG006711	3.06372238	3.08210633	3.19794903	3.45724816	3.12759099	2.95679765	3.02238099	3.22333839	3.09228136
NONHSAG006726	2.899555	2.88107688	2.86194758	2.79101787	2.82881079	2.88920289	3.02425528	2.88835715	2.87143363
NONHSAG006750	2.36744741	2.23930722	2.24345275	2.42403367	2.30377586	2.35469024	2.17466598	2.36990304	2.50601102
NONHSAG006793	5.66491015	5.88644548	5.72212625	6.02523583	5.68447084	5.92440863	5.92815182	5.84385431	5.31522178
NONHSAG006798	3.42570238	3.07615802	3.17348864	3.49466498	3.44712705	3.16295409	3.46753091	3.27057796	3.49183342
NONHSAG006806	4.53153508	4.45322107	4.47165175	4.3895403	4.68706252	4.51441477	4.42295594	4.39069396	4.79355171
NONHSAG006870	3.00724592	3.20273083	3.07749943	3.26523526	3.29228287	3.49335418	3.5869608	3.27145085	3.05826694
NONHSAG006890	6.09575902	6.06466408	6.03866021	6.02490742	5.90765296	6.31135757	6.00413568	6.05325093	5.98310154
NONHSAG006892	4.97595688	4.81895944	5.05758735	5.23561431	4.85605719	4.82695986	5.26018554	4.86071856	4.81066627
NONHSAG006896	2.66089151	2.50353022	2.58077659	2.78093718	2.77869344	2.56424435	2.57889379	2.61028897	2.66346911

NONHSAG006898	5.5618974	5.61766878	5.53296347	5.61068507	5.5582252	6.1800217	5.92770242	5.71119846	6.24143484
NONHSAG006900	4.76547341	4.39794896	4.96189665	4.72228568	5.00360205	4.72348762	4.57687528	4.68827902	5.0259795
NONHSAG006943	3.03026817	2.89162884	3.19442292	3.13879123	3.33158837	2.9502714	3.31871726	2.97438404	3.11940353
NONHSAG006949	2.41036198	2.24217842	2.26533832	2.42895814	2.35395589	2.55544911	2.56347911	2.4210434	2.42924107
NONHSAG006969	3.76332332	3.67861608	3.91216812	3.75637307	3.75929898	3.77998167	3.84980838	3.87889975	4.06861918
NONHSAG006971	5.5629459	5.92329879	5.55108314	5.59596051	5.41623032	5.59093362	5.61027349	5.71446402	5.37106441
NONHSAG006980	2.30560215	2.28990561	2.2514933	2.23522834	2.22864767	2.35001179	2.22349544	2.30843098	2.17912806
NONHSAG006992	4.23395319	4.03762011	4.24260757	4.20621092	3.84430103	4.4850154	4.23414061	4.17432848	4.29247794
NONHSAG007038	6.17490537	6.22310481	6.01556075	6.35138306	6.31936155	6.68289767	6.48385749	6.51410401	6.62071179
NONHSAG007047	2.64520325	2.54307537	2.99071389	3.2636833	3.40681296	2.67493831	2.98552372	2.70731534	2.88960078
NONHSAG007108	2.69172978	2.5433387	2.58395996	2.90784066	2.58644189	2.8764116	2.67009259	3.07350122	2.73038049
NONHSAG007115	2.16316798	2.18172353	2.32880003	2.11048584	2.19063826	2.36432261	2.25388772	2.30003071	2.28761755
NONHSAG007133	4.0056374	3.61506314	3.57354014	3.6719654	3.61769216	3.71827534	3.72489391	3.88500731	3.93097459
NONHSAG007156	5.21981504	5.54743111	5.26485558	5.40035863	5.54860205	5.45069675	5.43825638	5.26243925	5.72671947
NONHSAG007158	4.963576	4.6662246	4.67003114	4.98713433	5.03072318	5.08525686	4.95553309	4.92597929	5.04866084
NONHSAG007166	3.95660029	4.09228725	4.13069502	4.00252932	4.1210143	3.70292641	4.24538598	4.16296221	4.28883243
NONHSAG007181	5.44756763	5.69913318	5.36580169	5.42665399	5.43821705	5.67322545	5.4627099	5.71754026	5.75491998
NONHSAG007207	5.30572172	5.54743111	5.1489609	5.39839443	5.36041469	5.56705439	5.58299099	5.54228067	5.40355628
NONHSAG007221	3.8418527	3.77163599	3.71792214	3.72121116	3.74195166	4.05645663	3.92226301	3.77263135	3.654372
NONHSAG007222	4.9168769	4.8334692	4.872371	5.06287824	4.98863822	4.99288145	5.04218188	4.8153163	5.13478672
NONHSAG007253	4.89260989	4.62925683	4.88761074	4.74758897	4.98749099	4.88410067	4.96252063	4.72756542	4.60369188
NONHSAG007359	6.08581454	5.95984023	6.3285346	5.71269017	5.65756982	6.17037579	6.16452359	6.09672192	5.77862824
NONHSAG007368	5.30927841	5.39236747	5.35792187	5.20102323	5.34900443	5.43152938	5.28268566	5.29332161	5.28187729
NONHSAG007373	4.95503814	4.85861924	4.91183171	4.86672923	5.06875223	4.88438226	4.97121543	4.83529305	4.9376068
NONHSAG007386	5.02768648	4.45659675	5.20275882	4.6611902	4.84221399	4.8842978	5.2000877	5.09096108	5.05577266
NONHSAG007407	4.78421494	4.88599907	5.01598476	4.83546352	4.9903849	4.97162806	4.61729022	4.6522826	5.05636482
NONHSAG007504	4.7638117	4.59632838	4.64105378	4.78131529	4.66005247	4.5050354	4.48103641	4.70648229	4.80796634
NONHSAG007506	2.36428478	2.45886829	2.40885895	2.52273471	2.25127395	2.32410367	2.30606775	2.36563236	2.34392172
NONHSAG007524	4.24612922	4.00636408	4.21673454	4.04454383	4.25288149	4.22800273	4.06249382	4.28959577	4.20124726
NONHSAG007531	4.10893341	3.863955	4.31561182	4.17441165	4.37159959	4.13240433	4.10719344	4.251902	4.21608039
NONHSAG007550	4.78818714	4.92437277	4.6274033	4.84902813	4.84536003	4.32896277	4.07468005	4.62884511	4.92072165
NONHSAG007560	2.75709689	2.73117888	2.88492814	2.66489533	2.74208307	2.86675524	3.07445722	2.81706543	2.95592411
NONHSAG007562	2.63905194	2.54984924	2.75707241	2.67449156	2.67649479	2.76461028	2.61652397	2.56756287	2.69777249
NONHSAG007577	4.33976035	4.22138053	4.48779973	4.09315825	4.32205551	4.29890992	4.29722134	4.48099765	4.56208727
NONHSAG007580	5.02740322	5.03066415	4.99004742	4.93518698	5.15331792	4.69176873	4.94607819	4.95518039	5.14197942
NONHSAG007590	5.74697972	6.1729191	5.51126827	5.71517601	5.79586207	6.16279472	6.0532436	5.90453682	6.14946768
NONHSAG007653	3.13360222	2.95708049	3.24272269	3.16765223	3.2701573	3.19843877	3.23148437	3.40519463	3.20052326
NONHSAG007683	3.99431525	3.52816139	3.88785483	3.92524292	3.82637078	3.78386434	3.76955458	3.75419278	3.80036053
NONHSAG007686	3.71676772	3.66304855	3.78890842	3.82419571	3.8859934	4.00535247	3.89583729	3.88075652	4.09042448
NONHSAG007695	4.32327319	4.43911224	4.46662634	4.30418304	4.41264978	4.23026669	4.23716993	4.23992328	4.54299875
NONHSAG007714	4.21749959	3.87438017	4.22971453	3.76303633	4.00143788	3.89490702	4.01544396	4.11547378	4.42149309
NONHSAG007751	7.09567369	7.06042341	6.96357947	7.01739849	6.96766165	7.08955987	7.10536959	7.14253367	7.14002719
NONHSAG007766	6.74881862	6.73652166	6.55912048	6.90288375	6.19834477	7.05382016	6.84085412	6.83042102	6.164988
NONHSAG007767	3.65530351	3.98050796	3.72668131	3.92489251	3.32536367	3.87323643	3.38365082	3.49742944	3.65560622
NONHSAG007770	2.81994786	2.95732516	2.89105583	3.12184117	3.04860181	2.66973076	3.07906997	2.89868846	2.83508631
NONHSAG007825	3.92246512	3.47562286	3.4237044	4.14854557	3.82709985	3.57040278	3.73486404	3.34619495	3.61529566
NONHSAG007828	3.06704512	3.16208009	3.16546829	3.18379313	3.16024914	3.33711285	3.09933132	3.20829428	3.21466653
NONHSAG007837	4.42017393	4.38229077	4.80747341	4.40267162	4.43259753	4.5586302	4.27873451	4.44340424	4.75101847
NONHSAG007839	4.82513427	5.00922586	4.97843962	5.10668759	4.86942008	4.96297566	4.80692639	5.02716393	4.93909769
NONHSAG007862	3.33774817	3.25640882	3.48856464	3.42775571	3.54030312	3.72215858	3.34623064	3.47359698	3.76945389
NONHSAG007868	3.3943443	3.22513952	3.34223436	3.58545009	3.62993845	3.15374774	3.55911033	3.20561626	3.26655609
NONHSAG007885	2.65739639	2.62164467	2.82221717	2.8230756	2.6296192	2.5902075	2.52274554	2.57646412	2.5758998
NONHSAG007890	2.89574152	3.06376702	3.34632415	2.97147406	3.27205653	3.08039687	3.03955652	3.10104832	3.39282179
NONHSAG007906	2.33780735	2.33522736	2.50055735	2.28033628	2.59197762	2.17159404	2.45237039	2.4026879	2.46590676
NONHSAG007910	3.57968118	3.64319015	3.66915985	3.68383031	3.69047759	3.75292344	3.59899209	3.62626514	3.66511155
NONHSAG007917	2.5070779	2.46612955	2.44785135	2.42405044	2.55354666	2.51169247	2.49758026	2.41072598	2.4868315
NONHSAG007923	3.24180022	3.30918005	3.369555	3.33892158	3.44132589	3.29879232	3.25547567	3.40377395	3.31616618
NONHSAG007937	3.37533484	3.19194518	3.43403559	3.33022874	3.20073871	3.18800197	3.1866922	3.08941358	3.33625484
NONHSAG007942	3.14102341	3.11865366	3.02598127	3.09065048	3.27013535	3.06471554	3.03602932	3.18797253	3.09430083
NONHSAG007976	4.0180931	3.87661972	3.8454702	4.05869677	4.01245165	4.1109623	4.10884965	3.82238966	3.98014016
NONHSAG007987	2.81671288	2.7849462	3.01587661	2.757698	2.86106692	2.88159491	3.01783669	2.64739115	3.01776565
NONHSAG008006	4.83635062	4.62383291	4.80502785	4.75874185	4.89249023	4.80091861	4.71402555	4.6271787	5.06117202
NONHSAG008015	4.21303531	4.15673122	4.39632945	4.68285546	4.6310685	4.22005443	4.24874056	4.1172248	4.21032466
NONHSAG008021	3.33176485	3.307305	3.41497359	3.32688647	3.39864866	3.34907637	3.28582408	3.31607979	3.35218974
NONHSAG008036	3.54233838	3.52526498	3.72280492	3.67949586	3.43094572	3.67063875	3.55992461	3.4169966	3.61362363
NONHSAG008052	3.4349327	3.2816602	3.82536449	3.88858091	3.63768598	3.8146015	3.58795253	3.50845445	3.55153859
NONHSAG008061	4.23310536	4.48063737	4.50994916	4.19967097	3.95975263	4.60664178	4.45933953	4.57256363	4.39905852
NONHSAG008068	2.320778	2.27676475	2.09893332	2.18990595	2.39287352	2.1599262	2.21580971	2.36111994	2.18745494
NONHSAG008074	3.76259817	3.72665601	3.78253828	3.66428107	3.75633584	3.71453458	3.61365981	3.7674257	3.7179982
NONHSAG008085	2.20752565	2.09816373	2.18333345	2.30143229	2.3122438	2.18842352	2.1525332	2.18198753	2.12576676
NONHSAG008088	3.26072423	2.90234597	2.88979993	2.87981241	3.13742791	2.87000741	3.03543682	3.09289371	3.61565724

NONHSAG008123	4.83407783	4.80929617	5.01891761	4.86358912	4.71892855	4.89233515	4.9793861	4.78278393	4.8662597
NONHSAG008125	2.52185015	2.40695052	2.55557824	2.32864421	2.68263437	2.41308909	2.46197667	2.42338362	2.46337769
NONHSAG008197	3.74116186	3.68650588	3.66653068	3.45861208	3.57712825	3.56220764	3.81497941	3.58631841	3.91469118
NONHSAG008233	3.0248467	2.86917429	2.83515369	3.10413802	2.83495595	2.91648383	2.88470894	2.96381908	2.98404826
NONHSAG008613	3.78018141	3.73163859	3.67060991	3.63605201	3.84146825	3.73512977	3.55129878	3.51411305	3.91275433
NONHSAG008637	5.14467447	5.12641934	5.16843748	5.35420137	5.25488252	5.33592501	5.22971647	5.34290357	5.36877916
NONHSAG008664	4.79405206	4.92351109	5.06885465	5.23359829	5.0111746	5.34934108	5.01942426	4.98622635	4.95212417
NONHSAG008670	6.95098	6.45907719	6.37810859	6.62822887	6.83907293	7.13422328	7.26207155	7.20233064	7.24577596
NONHSAG008671	10.85569694	10.25540362	10.56721784	10.67468418	10.8255352	11.17382285	11.23185153	11.25079152	11.33801624
NONHSAG008684	4.37721723	3.84549016	4.55526554	4.09112765	4.08105817	4.1442402	4.47737928	3.93295094	3.90242833
NONHSAG008765	5.37593705	5.58604858	5.56499008	5.60921757	5.18719961	5.15650739	5.3509646	5.46141237	5.60178113
NONHSAG008818	5.16640633	5.54743111	5.59072882	5.05990296	5.59602958	5.12846444	5.28182061	5.17549273	5.85405731
NONHSAG008819	4.37260629	4.94976668	4.64229703	4.42745559	4.56365494	4.49493778	4.96763522	4.41044107	4.55147307
NONHSAG008903	4.03230008	4.04426937	4.08247043	3.89812963	4.06110475	3.86109058	3.87676095	3.59664616	4.29534964
NONHSAG008932	5.23788291	5.13804456	5.20121393	5.19675252	5.33542197	5.31077186	5.18075913	5.07247465	5.1451552
NONHSAG008943	4.82978951	4.98898743	4.73997654	4.83407783	4.63837954	4.70904876	4.88555567	4.75638401	4.63244479
NONHSAG008986	2.30769192	2.01776623	2.0635145	2.14093751	2.36435738	2.22733542	2.12751747	2.19765773	2.15020602
NONHSAG009036	3.87633783	3.99499433	3.93360377	3.92489251	3.64105108	4.02149839	3.87993315	3.56616235	3.89832395
NONHSAG009072	6.44644442	6.36227312	6.43279718	6.40343064	6.52969178	6.4098351	6.31047069	6.27633442	6.4443827
NONHSAG009076	2.85870676	3.04772562	2.72940969	2.75967969	2.65892159	3.10886314	2.69398439	2.74983477	2.77155942
NONHSAG009130	6.60746888	6.60919697	6.73721085	6.58925316	6.41903141	6.63910156	6.62834788	6.50524451	6.48055609
NONHSAG009132	2.59896262	2.72860123	2.7502858	2.73469234	2.96698104	2.51834359	2.53812107	2.98763856	2.88969608
NONHSAG009142	4.90540734	5.07271841	4.92594296	5.12732089	4.85087666	5.09223029	5.06575207	5.11935116	4.98584772
NONHSAG009168	9.08084068	9.20515664	9.13961512	9.11220207	9.15149693	9.10254088	8.95424725	9.03934518	8.83770257
NONHSAG009184	3.40543666	3.13425233	3.40645763	3.35107346	3.31064266	3.48594365	3.27623156	3.13134547	3.06305608
NONHSAG009186	3.31900654	3.11837118	3.24749212	3.203591	3.65882369	3.42689052	3.36364932	3.35213791	3.23699083
NONHSAG009191	2.94978943	2.98294748	2.92718097	3.28333824	3.05751597	3.20903312	3.18648527	2.95452798	2.97905542
NONHSAG009210	4.80046402	4.40057985	4.81277257	4.7324923	4.74059984	4.88975531	5.06220613	4.53630705	5.12397931
NONHSAG009216	2.76845464	2.98479846	2.69869124	2.79132451	3.06385275	2.63589983	2.61501003	2.61390579	2.54217606
NONHSAG009222	4.89223942	4.68869597	4.9303263	4.7371256	4.72290455	5.33917931	4.86676214	4.9193313	4.82244762
NONHSAG009269	4.40915577	4.5717794	4.47920019	4.68117191	4.63248562	4.33780592	4.55449428	4.67432968	4.77224842
NONHSAG009275	2.45928295	2.3966955	2.40593448	2.67877255	2.33513389	2.50900713	2.4502177	2.66233711	2.70269653
NONHSAG009283	4.96934902	4.97912108	4.87541066	4.86264061	4.89362127	5.03497077	4.92961931	4.83960519	4.7268349
NONHSAG009302	3.31757226	3.33809465	3.32719349	3.46292482	3.47475045	3.41273364	3.32231264	3.41406736	3.28271269
NONHSAG009308	2.47397667	2.36530534	2.67537463	2.61607957	2.86605332	2.53604343	2.48456024	2.80298157	2.75558171
NONHSAG009381	2.64143568	2.69798558	2.61534717	2.78521586	2.92640344	2.53157391	2.45658226	2.67497569	2.60009686
NONHSAG009397	2.85184736	2.85365581	2.98713209	3.16531241	3.16449811	3.25671889	2.93681941	3.00559598	3.19665625
NONHSAG009424	3.52964659	3.55835906	3.55333378	3.76480747	3.73437044	3.86135217	3.5979369	3.57668345	3.50669486
NONHSAG009434	2.55217836	2.54044192	2.74896015	2.58024401	2.65218355	2.65224639	2.54242476	2.58544871	2.76953632
NONHSAG009452	4.46405997	4.37633249	4.63190286	4.25614755	4.56022214	4.29464075	4.23106186	4.53482358	4.08985955
NONHSAG009462	3.53420515	3.63058072	3.95776059	3.57227194	3.86711939	3.62171572	3.29802274	3.44633353	3.73791114
NONHSAG009465	2.89670332	2.60117949	3.01643667	2.81545565	2.96077902	2.96197322	2.89506917	2.87934273	2.79686117
NONHSAG009524	2.86627758	2.78925471	3.06271024	2.77441618	2.8041363	2.63507311	2.96990265	3.110263	2.98714329
NONHSAG009530	3.25921748	3.22977291	3.08877315	3.11459088	2.98360895	3.26746915	2.86471916	2.76744603	2.89229795
NONHSAG009590	2.37895222	2.47923533	2.5914188	2.6090488	2.61813335	2.58820848	2.49449633	2.62722838	2.63135082
NONHSAG009627	5.3911118	5.4144048	5.55639098	5.53993261	5.63912626	5.37417338	5.48092867	5.55092159	5.4930193
NONHSAG009630	2.60722092	2.33189883	2.80919056	2.52419443	2.81135001	2.49259185	2.6625093	2.58974808	2.42527617
NONHSAG009643	4.82614581	4.77514847	4.64650304	4.71329388	4.75099923	4.69741769	5.26758952	5.08990934	5.0566965
NONHSAG009663	2.70943556	2.67430538	2.8554182	2.69204583	2.80932431	2.67496392	2.66607986	2.61229967	2.61650969
NONHSAG009678	4.26693435	3.85570969	4.06403568	4.05722258	4.30601199	4.03266887	4.13870013	4.15220961	4.06332981
NONHSAG009688	3.37204455	3.17765861	3.4463843	3.90136983	3.41854331	3.44585251	3.43521925	3.44961264	3.44585251
NONHSAG009690	4.83225751	4.58535296	4.99762477	4.88209862	4.87531971	5.01583681	4.74196247	4.90706435	4.95355094
NONHSAG009700	3.17541453	3.30561728	3.22028535	3.15604227	2.93395173	3.10700584	3.18100271	3.43565152	2.99393546
NONHSAG009711	3.07281858	3.01892823	2.90685693	2.90739193	3.15584294	2.91011105	2.84484106	2.93239357	2.70188553
NONHSAG009736	3.110026	3.17854034	3.03941628	3.18135975	3.18769284	3.27914068	3.33348672	3.4141146	2.88969608
NONHSAG009783	2.87916553	2.65568848	2.88162621	2.80189397	2.93832972	2.99410116	2.80889646	2.92456484	3.14987294
NONHSAG009834	4.55946943	4.58630206	4.48036273	4.66626699	4.64310566	4.61947541	4.71530651	4.57366285	4.15455098
NONHSAG009878	2.22394536	2.32619055	2.36851151	2.35431524	2.31680856	2.25492939	2.26445421	2.37137063	2.25090018
NONHSAG009880	2.30507151	2.31813702	2.07289269	1.97798227	2.29689303	2.211374	2.35473809	2.20099038	2.32442104
NONHSAG009903	4.15673122	4.30881918	4.29483044	4.15062461	4.46277683	4.34725495	4.36961223	4.37672927	4.46277683
NONHSAG009919	3.64471657	3.6664301	3.64392929	3.81370034	3.76423716	3.71947681	3.72060424	3.87482818	3.78346053
NONHSAG009921	4.17432095	4.26982704	4.1762646	4.29829948	4.23054402	4.21516364	4.13425905	4.17498106	4.41580105
NONHSAG009942	3.52414954	3.68735935	3.65900513	3.28750029	3.54626658	3.19423406	3.48833491	3.55017946	3.48391474
NONHSAG009977	4.57128363	4.45192239	4.38619261	4.49621189	4.71775031	4.16196453	4.53304754	4.30833791	4.619519
NONHSAG009994	5.33779906	5.64238696	5.03012596	5.35268997	5.14257455	5.48601291	5.37184259	5.26254529	5.20544002
NONHSAG010005	2.69284643	2.60573227	2.33823589	2.73571511	2.64937656	2.52091119	2.54808239	2.55990352	2.43933443
NONHSAG010007	2.88889833	2.84385708	2.9056139	3.139751	3.05560383	2.94700828	3.000491	2.94767657	2.82341399
NONHSAG010015	2.47245519	2.45177519	2.44178116	2.22720577	2.64824711	2.6107549	2.46833247	2.5588514	2.46742966
NONHSAG010022	3.03699604	2.8636808	3.06803763	3.17863311	3.49394859	3.11448169	2.90004783	3.12365159	3.5032104
NONHSAG010026	4.24809862	4.13328131	4.21219469	4.07191594	4.43585271	4.1572819	4.30537129	4.23992328	4.50356973
NONHSAG010034	3.05087606	3.25454939	3.34918917	3.16395702	3.16084107	3.13155775	3.33197727	3.37039334	3.172847

NONHSAG010061	3.73301374	3.78423174	3.84993467	3.83449421	3.83851544	3.94017152	3.84515169	3.90053055	3.99509705
NONHSAG010086	2.91722507	2.99050866	2.94115062	3.03683074	2.87229014	2.96296756	3.04835137	2.88937006	3.01284105
NONHSAG010089	3.12800348	3.13696648	3.33135558	3.21184668	3.19450498	3.07424392	3.29013145	3.16023483	3.19100539
NONHSAG010109	5.26164861	5.12516562	5.30898594	5.01986195	5.32214001	4.95095573	5.00677112	4.9991441	5.38197832
NONHSAG010134	6.15673626	6.48265371	5.758979	6.05392666	6.08611395	5.99259917	5.96261719	6.18906404	5.9112771
NONHSAG010144	4.26100047	4.07659777	4.4038547	4.2452373	4.47385558	4.45409545	4.13516751	3.75934345	4.32675085
NONHSAG010152	4.63474598	4.55874134	4.48987743	4.49800643	4.32105338	4.52240958	4.80341802	4.54199816	4.55820459
NONHSAG010157	2.5599862	2.30781094	2.539016	2.5725916	2.906496	2.63299391	2.75670772	2.47308559	2.59493019
NONHSAG010160	3.68761388	4.20300968	3.82151786	4.07399474	4.20325344	4.0574538	4.23302814	4.09195346	4.18420189
NONHSAG010163	2.93905167	2.72839151	3.05307669	2.90835591	2.72949882	2.85740289	2.83496681	2.92228296	2.74721436
NONHSAG010226	4.43190173	4.40574399	4.35862928	4.60359735	4.36135683	4.62069404	4.54163311	4.65145304	4.23025037
NONHSAG010232	2.90926719	2.86422196	2.69173802	2.84356672	2.89902833	2.86392395	2.84203674	3.12214307	2.92118549
NONHSAG010260	4.74470016	4.79224534	4.85715599	4.76092554	4.87658794	4.87473118	4.61793378	4.42259069	4.77590155
NONHSAG010262	5.18950595	5.00781902	5.15298628	5.10504145	5.45521848	5.12753891	5.17934536	4.74205691	5.17707461
NONHSAG010264	5.22287427	5.13410539	5.0372001	5.38802371	5.21973994	5.61420486	5.21697055	5.21058612	5.32883724
NONHSAG010352	5.80570215	5.92353307	5.79728714	5.91156746	5.77705994	5.77699518	5.85932726	5.81136461	5.78841172
NONHSAG010413	2.2506371	2.16341674	2.31989669	2.3915075	2.27401025	2.27131766	2.42386459	2.25118855	2.28384177
NONHSAG010420	6.74844643	7.00682438	6.598682	6.57500104	6.69001249	6.78610623	6.7231889	6.70526361	6.70132174
NONHSAG010447	2.31280339	2.60199514	2.49466205	2.67780588	2.64735337	2.39024674	2.68231192	2.34422827	2.50877593
NONHSAG010455	6.88194012	6.85174712	6.69486619	6.81599455	6.64674743	6.56776357	6.56021333	6.88012833	6.61592261
NONHSAG010492	2.81549854	2.9464301	2.87363758	3.03217978	2.87119941	2.84544152	2.96964867	2.93412072	2.95054914
NONHSAG010499	2.41837321	2.4283125	2.5081571	2.5682092	2.63912477	2.47240367	2.52274554	2.67675079	2.51549678
NONHSAG010524	2.417992	2.47807062	2.42251304	2.3216007	2.72449776	2.57059886	2.35596584	2.37899963	2.4724114
NONHSAG010532	4.64028955	4.67974399	4.73253233	4.71907662	4.36713292	4.79996153	4.60176033	4.64549922	4.725476
NONHSAG010566	2.35564974	2.41116081	2.44623628	2.4994047	2.70947242	2.43853249	2.49840093	2.48818793	2.46855662
NONHSAG010571	4.84274346	4.71947799	4.55819314	4.55009755	5.02028578	4.99821353	4.85906921	5.00097853	5.09913613
NONHSAG010573	5.88330495	6.07343396	6.02314963	6.11127676	5.79083381	6.05633287	6.05696278	6.0993739	5.96118725
NONHSAG010585	2.87063369	2.65176353	2.91830633	2.97385124	2.85778527	3.09178679	2.82765065	2.84593675	2.79017663
NONHSAG010628	4.88511864	4.87977435	5.01045401	5.12171208	5.32508081	5.21721531	5.06459485	5.4293506	5.79405391
NONHSAG010678	3.22438463	3.13692342	3.0286406	3.12927316	3.24099994	3.18501697	3.24013802	3.30351271	3.05178401
NONHSAG010682	2.83990147	2.65293634	2.82708703	2.75618186	2.77820231	2.89294323	2.84922375	2.7348408	2.98817192
NONHSAG010721	2.49256486	2.5111507	2.65193018	2.29310466	2.5369189	2.50723626	2.51719643	2.71055993	2.3669514
NONHSAG010724	2.72725223	2.93687475	2.78443962	3.00046807	2.78336932	3.0550491	3.17079271	2.72879087	2.96352639
NONHSAG010765	3.23840535	3.03488748	3.1390658	2.82594981	3.01227974	3.19681602	3.15365552	2.87793334	3.39473899
NONHSAG010767	2.93436453	3.05444913	2.86970188	2.94521947	3.09699439	3.13646826	3.25785525	3.11633916	3.09867981
NONHSAG010791	2.26835375	2.17489475	2.30220636	2.42823499	2.29512082	2.44963323	2.35462482	2.41341363	2.30127915
NONHSAG010808	4.35328106	4.22588006	4.31561182	4.48450766	4.58511762	4.57040537	4.68370457	4.43863441	4.39679685
NONHSAG010846	3.54830497	4.12357604	3.94016806	3.78153733	4.3283037	4.23120562	4.19859665	4.06447989	3.31309438
NONHSAG010848	3.74258461	4.22451037	3.75128388	4.24350536	3.83843807	4.63110362	4.27296448	4.0375505	4.35545376
NONHSAG010888	2.74557727	2.57091641	2.87000703	2.84756254	2.75890979	2.70701426	2.69239922	2.71259926	2.75953903
NONHSAG010892	3.18530123	3.67209631	3.89327537	3.60146469	3.3381695	3.2988283	3.28474948	3.22233374	3.47964189
NONHSAG010908	2.32069847	2.06024958	2.27254991	2.18362081	2.3476338	2.06643981	2.11878239	2.50571946	2.1135434
NONHSAG010921	3.35450439	3.44139407	3.47003267	3.70139009	3.3289159	3.46064347	3.22281891	3.28635607	3.33424277
NONHSAG010938	4.63390993	4.73581364	4.58634454	4.56018265	4.73719882	4.43464116	4.67141659	4.56182599	4.48822614
NONHSAG010946	5.58378335	6.03659896	5.48742769	5.76718704	5.60855547	5.74422592	5.48189276	5.66272903	5.38158712
NONHSAG010964	3.51141326	3.5374491	3.5406404	3.59812064	3.69455726	3.61864437	3.62705042	3.30509563	3.74364821
NONHSAG010989	3.33090016	3.21466339	3.2356707	3.12734699	3.31847459	3.56942904	3.43747843	3.22697263	3.2692229
NONHSAG010991	3.1148484	3.09996442	3.01656166	3.23990812	3.05178401	3.22041215	3.24957278	3.08305867	3.16647854
NONHSAG010992	5.18749553	5.61766878	5.33755349	5.31056227	5.31127546	6.5336606	5.75084016	6.03662989	5.74534585
NONHSAG011000	4.57470154	4.61423103	4.7889567	4.42409589	4.70277161	4.48749841	4.80054104	4.55276206	4.61093943
NONHSAG011016	4.3472685	4.21022643	4.36817739	4.14185461	4.35453951	4.26699746	4.34837296	4.3908325	4.619519
NONHSAG011035	2.74010779	2.76882767	3.04252135	3.00282228	2.65550519	2.89169483	2.50412843	2.89721018	3.16955651
NONHSAG011091	3.62632441	3.56446896	3.70522792	3.53203858	3.74928028	3.63390417	3.59104248	3.71056997	3.84134947
NONHSAG011182	5.26026537	5.4105704	5.30420282	5.34666224	5.46793171	5.57323382	5.51580811	5.4686291	5.08474063
NONHSAG011270	3.77944136	3.56614874	3.75816807	3.70357163	4.01794815	3.95755061	3.95383913	3.677604	4.12685401
NONHSAG011279	3.73436442	3.77553274	3.52825626	4.0974493	3.98086799	4.09835914	3.750988	3.88622993	3.88929789
NONHSAG011288	8.95111171	9.22804882	8.87148348	8.61137183	8.7768345	8.64457922	8.79139112	8.61800415	8.83022427
NONHSAG011425	4.54137312	4.62635092	4.48605465	5.09597201	4.80057327	4.73997654	4.69477968	5.00588095	4.61721618
NONHSAG011459	5.74543548	5.57072882	5.71057447	5.67795111	5.84446919	5.85658818	5.73485463	5.43422314	5.67643686
NONHSAG011460	5.44547344	5.43362832	5.29389218	5.60924506	5.24790574	5.40507429	5.54485303	5.61404182	5.38549546
NONHSAG011467	2.89550237	2.72052613	2.90569801	3.00376049	2.93890068	2.94374802	3.07328568	2.8443743	2.97332346
NONHSAG011471	3.4401638	3.3581403	3.53892203	3.43351682	3.52633086	3.4621876	3.39052491	3.74111355	3.76238303
NONHSAG011496	3.61327473	3.92489251	3.66293625	4.07676487	3.55286786	3.87388305	3.79743931	3.7361303	4.05341258
NONHSAG011553	3.32626153	3.27319972	3.36646859	3.24998511	3.4173362	3.33251023	3.38614192	3.3630871	3.44773611
NONHSAG011555	3.11272186	2.9943031	3.55505605	3.34647313	3.71669613	3.46731451	3.21061809	3.13969783	3.1068789
NONHSAG011584	4.86007657	5.22905214	5.04464718	5.12145375	5.13794304	5.0827058	5.04592003	4.96687327	4.8993279
NONHSAG011594	3.06636214	3.02990193	2.88294455	3.03816451	3.00255673	3.17133732	3.02747381	2.91960224	2.92409619
NONHSAG011643	2.74601636	2.78292298	2.72244386	2.88082891	2.70351678	2.56569139	2.64561276	2.52594795	2.5758998
NONHSAG011647	2.37658458	2.3741287	2.79964166	2.56424435	3.00738152	2.4689214	2.59821946	2.7348408	2.77287136
NONHSAG011680	3.741622	3.47405196	3.50159269	3.50399231	3.94009186	3.65230628	3.3692509	3.66816081	3.51837315
NONHSAG011703	4.56955547	4.65210835	4.2440321	4.68226279	4.69520749	4.87282633	4.64040528	4.86611583	4.26039515

NONHSAG011730	3.82281847	3.94610968	3.86732765	3.73998203	3.84944407	4.06538897	4.05627158	4.08843034	3.53285252
NONHSAG011744	3.51863489	3.62858249	3.82350519	3.59165072	3.75573231	3.40899311	3.39052491	3.81457503	3.69543198
NONHSAG011747	6.96311817	6.60743722	7.01024646	6.12538934	7.191557	6.29540282	7.01313274	6.5520347	7.04137871
NONHSAG011762	3.32973838	3.33250511	3.28205108	3.26650507	3.55962223	3.38710179	3.44944183	3.2447671	3.55535444
NONHSAG011768	2.13713386	2.19527725	2.25435213	2.4473539	2.44652102	2.23212042	2.27097571	2.29821954	2.10794337
NONHSAG011773	2.17573542	2.28876945	2.21387135	2.31748206	2.27601462	2.56424435	2.30780017	2.23341152	2.23542542
NONHSAG011781	7.02707944	7.28680107	6.76918905	7.03071466	6.83594567	7.0699042	7.10119324	7.05268938	7.19776361
NONHSAG011800	2.52349792	2.51597834	2.59534042	2.77557879	2.74486492	2.91766284	2.48548251	2.64903467	2.41145962
NONHSAG011802	9.99334531	10.00796238	9.72178218	9.54254634	9.73620547	9.86279713	9.94417379	9.94856776	9.74126365
NONHSAG011805	3.3428836	3.03579837	3.56647668	3.32512693	2.98111071	3.08229468	3.08191763	2.90149651	3.21715447
NONHSAG011821	2.63788232	2.6262953	2.92145278	2.78275248	2.71816786	2.66290626	2.49778616	2.77341609	2.68893474
NONHSAG011830	2.23061611	2.42119856	2.25405493	2.5312565	2.26643406	2.43666762	2.52274554	2.19568048	2.24608246
NONHSAG011840	2.08620149	2.29665008	2.49118328	2.24990343	2.5060962	2.26240033	2.41408441	2.23841436	2.37856921
NONHSAG011877	5.16621311	5.28487724	4.83717145	5.28900056	4.75009142	5.34436117	5.36053617	5.52447077	4.9897942
NONHSAG011915	5.77427402	6.1940194	5.61156095	6.04168088	5.67625622	5.96348607	5.67995154	6.0680052	5.33171168
NONHSAG011924	3.3918733	3.49245938	3.34283213	3.16825346	2.98240989	3.34055813	3.24751536	3.30897907	2.58081681
NONHSAG011933	5.58798446	5.78318741	5.69860421	5.51127588	5.58798446	5.87686898	5.74730982	5.59359977	5.79261368
NONHSAG011964	6.31672486	6.6123985	6.30717374	6.47411989	6.19246739	6.32096922	6.20422507	6.20817044	6.0890256
NONHSAG011968	4.30037208	4.49916579	4.40158694	4.3445598	4.41726576	4.26581149	4.14989014	4.2034775	4.19668014
NONHSAG011977	2.64263504	2.82759102	2.99566661	2.84191067	2.75756225	2.88162512	2.63624003	2.82450672	2.97332346
NONHSAG011978	4.91151451	4.83322476	4.80974967	5.01013182	5.04289293	4.98722799	4.84062942	4.77414859	4.6229025
NONHSAG012011	2.65117463	2.67200812	2.76312564	2.71372998	2.56522454	2.80996532	3.19362045	2.57406746	2.66613495
RMST	6.85867111	7.45115709	7.27552981	6.99829514	6.89994483	7.48849481	6.92244188	6.95727238	6.55388376
NONHSAG012031	4.3105211	4.20768916	4.11776191	4.43364303	4.28434085	4.3250987	4.32983403	3.98743238	4.07765352
NONHSAG012043	2.20467989	2.18638	2.19298069	2.08357012	2.40436835	2.19450602	2.19578976	2.42422696	2.30347336
NONHSAG012048	5.01820456	5.17555549	4.91448465	5.17155132	4.97942761	5.53130929	5.18640106	4.97345281	5.08495622
NONHSAG012050	6.76319205	7.1237507	6.58424631	6.80392429	6.57636243	6.80566824	6.59250016	6.79153143	6.73648089
NONHSAG012055	7.15653558	7.19742753	7.09139107	7.21173112	7.00134612	7.14088545	7.19048827	7.29897523	7.12688185
NONHSAG012099	2.34248694	2.45517164	2.47974252	2.48581651	2.47514608	2.48144349	2.3961649	2.42444715	2.59048116
NONHSAG012152	2.54231647	2.26501376	2.33605971	2.38917667	2.38784099	2.48279529	2.48896586	2.39801559	2.64671896
NONHSAG012158	4.42843523	4.40006572	4.45506468	4.67711251	4.12268148	4.22800273	4.54685395	4.66350618	4.70713137
NONHSAG012172	3.86542128	3.69797185	3.72254903	4.07169182	3.9459583	3.76125477	3.90502289	3.96244347	3.76971563
NONHSAG012182	4.26951779	3.95634179	4.110683	4.07393156	4.04375056	4.24687977	4.26844188	4.26654056	4.06610161
NONHSAG012199	4.31619372	4.112655	4.39850649	4.08285438	4.52361357	4.14452133	4.58733203	4.19607031	4.36562393
NONHSAG012226	4.11824233	3.87600034	4.15953936	4.20730159	4.21817094	4.17337156	4.05411047	4.03898941	4.09315853
NONHSAG012236	2.64266104	2.69314792	2.52111522	2.5237698	2.69598815	2.85884166	2.7285833	2.64438301	2.64251404
NONHSAG012367	3.17959474	3.59396473	2.82617317	3.03914247	3.02679	3.527609	2.81145288	3.31439253	2.78918244
NONHSAG012392	2.40878144	2.56834425	2.48568579	2.45999219	2.59263883	2.47742629	2.5312135	2.65052955	2.57302456
NONHSAG012393	4.26653858	4.61200682	4.48400486	4.56304797	4.52760686	4.60767428	4.95048862	4.52767754	4.71900901
NONHSAG012430	2.84320519	2.61355086	2.81834703	2.99640133	2.94865402	2.86634115	2.90682936	3.08923569	2.88577218
NONHSAG012433	4.98926646	5.0430644	4.7980981	5.00810578	4.83817198	4.78857992	5.02192577	5.06320282	5.17032224
NONHSAG012556	6.2624766	6.38319471	6.23007298	6.54719655	6.63913969	6.52249835	6.42783111	6.30231773	6.24420307
NONHSAG012565	4.3451173	4.29758471	4.18872748	4.46514293	4.36059044	4.28332336	4.30480411	4.39728901	4.32081059
NONHSAG012566	4.1653911	3.87985215	4.16217628	4.04166986	4.36762571	4.1277672	4.1379168	4.1399761	4.29379432
NONHSAG012599	5.88032379	6.30670737	6.06609368	6.33280486	5.82836478	6.11410151	5.91731267	6.00081542	5.60093598
NONHSAG012608	4.5231766	4.20649195	4.41060704	4.58027776	4.35981583	4.25361224	4.11517042	4.24847935	4.2085082
NONHSAG012629	2.55559564	2.50237332	2.51251742	2.5358055	2.37704511	2.56268342	2.61505116	2.52063793	2.37155209
NONHSAG012630	5.92204364	5.69284804	5.77598668	5.70781407	5.96967578	5.854146	5.8621336	5.74977941	5.75415211
NONHSAG012635	3.16979784	3.16580856	3.36033514	3.26867497	3.42442198	3.15670008	3.38432731	3.28566096	3.58887889
NONHSAG012657	7.74210031	8.184616	8.08234999	8.03827066	7.9447283	7.94804276	7.79389116	7.60461882	7.53071277
NONHSAG012658	9.5297224	9.79605039	9.86718657	9.75547805	9.77061868	9.72641299	9.46193463	9.47823268	9.36289379
NONHSAG012682	3.10556188	2.90213595	3.45581432	2.88600782	3.14686191	3.36614726	3.23583477	3.19659306	3.4632143
NONHSAG012683	3.33118617	3.24346999	3.31133929	3.54928282	3.3162325	3.17931083	3.17814145	3.23420192	3.58412222
NONHSAG012684	2.52484738	2.48544916	2.5081571	2.56424435	2.39021818	2.56424435	2.27836483	2.62675517	2.86225145
NONHSAG012695	4.58489103	4.33623904	4.47146471	4.42524891	4.59604524	4.43023379	4.50968609	4.30880819	4.50892711
NONHSAG012727	5.14649976	5.32458141	5.2477863	5.66836565	5.26311215	5.41651933	5.12968802	5.44769714	5.28527861
NONHSAG012732	3.49858747	3.3642677	3.42452266	3.28257853	3.53273078	3.33422673	3.29935739	3.00781292	3.66486453
NONHSAG012739	5.36580169	5.26332232	5.4914603	5.56886323	5.82341348	5.57843503	5.54290565	5.55002546	5.63645041
NONHSAG012744	2.89492354	3.02966359	2.82432567	3.08812279	3.04787386	2.93724632	3.13057799	2.97991266	3.22349577
NONHSAG012745	3.3397241	3.6729171	3.58965653	3.83210297	3.41757731	3.80560132	3.60544716	3.43606294	3.72225713
NONHSAG012747	5.03154117	4.98987897	5.28656784	4.56345309	5.43185051	5.21743268	5.0528487	4.98961923	5.29865237
NONHSAG012753	5.81519568	5.73264518	5.71410249	5.87465005	5.89483032	5.70230333	5.72138046	5.9315377	6.28740874
NONHSAG012762	4.3433537	4.44743189	4.67083059	4.64426322	4.6954956	4.40329888	4.49900931	4.28795085	4.6917183
NONHSAG012764	4.73821377	4.35801907	4.51255368	4.71451407	4.87160652	4.44043386	4.71954407	4.5258251	4.93512686
NONHSAG012780	4.22625119	4.21305184	4.13315289	4.19206043	4.32230442	4.13511782	4.36762146	4.32288715	4.2407727
NONHSAG012793	6.32970053	6.48926555	6.32762463	6.42151074	6.1894474	6.35267615	6.28161691	6.21760735	6.3070652
NONHSAG012799	5.53504559	5.57297814	5.56947663	5.60857949	5.50547158	5.5669218	5.37252581	5.33792901	5.59142102
NONHSAG012823	4.2246533	4.21673872	4.10563236	4.02751942	4.15048122	4.24553295	4.04782575	4.11476963	4.24848705
NONHSAG012881	2.64536571	2.80368885	2.80746432	2.86713314	3.03416917	2.56379496	2.88347107	2.96650709	2.82906828
NONHSAG012884	2.52415024	2.32619649	2.435193	2.47408995	2.6032729	2.83748917	2.57152249	2.73347736	2.92600129
NONHSAG012909	5.90670396	5.94487055	5.87448187	6.30005298	5.8453031	5.99242386	5.96057905	5.80894645	5.89914294

NONHSAG012910	6.3270842	6.38586544	6.61037696	6.52887245	6.48893143	6.66629052	6.34195099	6.54620205	6.63304426
NONHSAG012920	2.72055236	2.87478693	2.77987405	2.58299726	2.80279727	2.81680113	2.84346042	2.8443743	2.87478693
NONHSAG012971	2.77974719	2.75735198	2.31767344	2.6191058	2.48207117	2.38759133	2.72569314	2.50607426	2.2598516
NONHSAG012980	2.58896593	2.37211924	2.83685134	2.55963025	2.69865244	2.59230152	2.67881974	2.7348408	2.49747659
NONHSAG012986	8.1737496	8.17978259	8.02515254	8.05937268	8.0362964	8.30658023	8.26202188	8.15826957	8.00545314
NONHSAG013018	3.10395655	2.99558656	3.18484694	3.1398599	3.25898326	3.13033605	2.78371458	3.04808891	2.88809933
NONHSAG013032	2.70053339	2.71856311	2.68793414	2.83789322	2.65023903	2.70211827	2.78753376	2.58388315	2.7249921
NONHSAG013037	3.9117169	3.82976147	3.97601641	3.93061691	4.21817661	4.04703918	3.92257511	3.70259012	3.85168057
NONHSAG013039	4.09583843	3.82406276	4.04959871	3.92489251	4.32325519	3.89544422	3.97347896	4.11712778	4.49332079
NONHSAG013044	4.10932736	4.21474162	4.33705698	4.20345606	4.13824013	4.02149839	4.04703793	4.28973287	4.63048487
NONHSAG013052	4.88756538	4.59174612	4.57480438	4.67688567	4.6911329	4.88372618	4.7326955	4.65531145	4.66035113
NONHSAG013055	2.25402432	2.06795318	2.04791048	2.33897274	2.16434921	2.18729947	2.24559824	2.16699253	2.1096765
NONHSAG013061	2.90168275	2.58325726	2.97456457	2.76502683	3.02850148	2.92185562	2.57515145	2.69377363	2.76324423
NONHSAG013087	4.98501738	4.67046665	4.79070049	4.79392896	4.91974835	5.06385749	4.86693961	4.65126909	4.84655996
NONHSAG013088	4.92095185	4.85410711	4.94856807	4.98796533	4.7733007	4.98060059	4.77652465	4.77617915	4.82075078
NONHSAG013092	3.01699524	3.04723198	3.14747289	3.10052106	3.10090855	3.0255762	2.76089946	3.25934025	2.89838643
NONHSAG013093	4.0796433	3.8596974	3.96332864	4.43967179	4.09230962	3.85738242	3.94541387	3.53212356	3.69729326
NONHSAG013140	4.55635123	4.54015509	4.54424304	4.62782446	4.74742159	4.60979035	4.6704169	4.62174243	4.67157721
NONHSAG013147	2.82301283	2.56950083	2.80769352	2.8967331	2.67707562	2.85683002	2.84660775	2.62722838	2.88969608
NONHSAG013148	3.74163002	3.83671997	3.82737727	3.96123455	3.75958173	3.8980167	3.65726235	3.93575369	4.15124074
NONHSAG013176	3.17952266	3.49263726	3.48638856	3.32081508	3.36122329	3.05084236	3.36122329	3.36804777	3.31966728
NONHSAG013184	5.30403882	5.54492615	5.50051854	5.38535371	5.44339656	5.88295182	5.56119399	5.45755913	5.85923876
NONHSAG013195	5.63076628	5.65035865	5.76050664	5.86121348	5.65163392	5.82244171	5.48253247	5.74959529	5.93532698
NONHSAG013196	3.91320676	3.81278675	3.88843394	3.68315096	4.32972473	3.97744455	4.22307082	3.87217733	4.13062569
NONHSAG013213	2.73149017	2.71901621	2.66296679	2.74889828	3.0037498	2.72842036	2.91766693	2.57733133	2.9255437
NONHSAG013219	3.48089074	3.3325074	3.43745587	3.83966959	3.67649077	3.38678876	3.38737245	3.43565152	3.55137509
NONHSAG013224	3.2423472	3.23354937	3.03388195	3.55752757	3.13379349	3.1907573	3.28335086	3.31439253	3.3724201
NONHSAG013270	2.27723545	2.50544744	2.50232388	2.47958163	2.40550352	2.21734214	2.27092309	2.4214816	2.15837891
NONHSAG013289	3.48776701	3.60981827	3.38439297	3.70262484	3.35812415	3.64617455	3.80347923	3.67201492	3.70016059
NONHSAG013312	4.40395538	4.8689497	4.06305942	4.60069395	4.06894241	4.86379308	4.49311215	4.88939008	4.55490452
NONHSAG013343	8.25282554	8.26744183	8.0734319	8.10930719	8.05703373	8.23767971	8.12063483	8.16324357	8.23255168
NONHSAG013371	3.9750466	4.04638865	3.81879539	4.46498395	3.79415258	4.07472371	4.33315867	4.09041959	3.52633935
NONHSAG013387	4.09710922	4.10186891	4.05146743	3.99533254	4.27149975	4.01964366	4.08590738	4.07971399	4.09149217
NONHSAG013400	6.97569438	7.15809686	6.94087881	7.01467773	6.90092134	7.09231567	7.02090279	7.00828556	6.91846862
NONHSAG013413	3.35610044	3.05259911	3.23584658	3.78277515	3.48350922	3.28924314	3.85220303	3.34179616	3.14788975
NONHSAG013415	4.81192443	4.64020582	4.5349931	4.8454931	4.63911752	4.87250592	4.67537363	4.85237682	4.6917183
NONHSAG013417	2.34243303	2.43598198	2.26481006	2.15721029	2.42554935	2.49911723	2.11878239	2.17141657	2.04686924
NONHSAG013418	4.45868941	4.53257862	4.56427503	4.61416911	4.73072738	4.6913034	4.54102254	4.3420885	4.75928181
NONHSAG013429	8.87130128	8.97720427	8.79812692	8.75614413	8.83694234	8.83471404	8.47378528	8.78544914	8.62753169
NONHSAG013433	2.47929238	2.40171358	2.56197472	2.57095994	2.58408664	2.60094428	2.39265312	2.66031118	2.59378316
NONHSAG013449	2.64121166	2.49635994	2.66930491	3.06054735	2.71351813	2.54137602	2.73894322	2.67740336	2.70792901
NONHSAG013472	3.17534636	3.14501253	3.17301662	3.32523539	3.19413258	3.03165079	3.46106732	3.44099414	3.38980758
NONHSAG013488	3.19962552	3.23754024	3.40290478	3.19629275	3.22738749	3.24311421	3.25119665	3.14256221	3.14258112
NONHSAG013496	2.96315305	2.93174139	2.97060434	2.67523878	2.79884681	2.97709169	2.72841353	3.04720192	2.89210286
NONHSAG013505	5.6287412	5.75981963	5.74058715	5.60632173	5.83164375	5.54662482	5.65903713	5.70414802	5.98350279
NONHSAG013509	4.90874894	5.08898295	4.97787499	4.90870286	5.05666977	5.38723062	5.19294696	5.07734608	5.31263383
NONHSAG013521	3.65523463	3.28408794	3.67688792	3.39511244	3.57727915	3.80055394	4.16311133	3.86393743	3.56647007
NONHSAG013525	4.27244367	4.19404317	4.20195656	4.38110914	3.92051458	4.28076123	4.18836351	4.08013803	4.17189168
NONHSAG013533	3.39314525	3.47344814	3.44306327	3.64698597	3.37881913	3.45601587	3.26732668	3.43895391	3.70292171
NONHSAG013546	3.00511056	2.89703307	3.34514034	3.01906841	3.36350929	3.29146252	3.18898946	3.18803654	3.60254134
NONHSAG013584	5.3912437	5.45254015	5.14281337	5.17092679	5.28948685	5.334666	5.29229593	5.26363739	5.0251076
NONHSAG013596	3.61178042	3.78544417	3.70182532	3.54936381	3.74222161	3.7949489	3.66400859	3.6649731	3.70635119
NONHSAG013598	2.57039643	2.55355649	2.61138389	2.64917725	2.69182498	2.84827754	2.46175548	2.53670834	2.60657131
NONHSAG013622	2.44909967	2.3956457	2.34465552	2.67069935	2.57654138	2.47056125	2.21822795	2.4809999	2.43904531
NONHSAG013653	2.39237029	2.46182699	2.48249961	2.57168372	2.51061361	2.73115866	2.60849695	2.29455238	2.46752254
NONHSAG013655	2.84911875	2.90571585	3.06769484	3.29243197	2.90269852	3.02373384	2.9330634	2.74637628	2.85778021
NONHSAG013658	2.51094991	2.55220311	2.29689303	2.52949208	2.94442819	2.76254066	2.61501003	2.78802388	2.49066485
NONHSAG013688	2.81704522	3.35601776	3.30854744	3.22061106	3.21308054	3.06022166	3.08880426	2.80658245	3.25585024
NONHSAG013701	2.19457576	2.37607558	2.34444738	2.40535166	2.35551853	2.2929215	2.34147659	2.34637714	2.26956345
NONHSAG013735	3.92127655	3.99437298	3.97871122	4.05205815	3.92919499	3.96635404	4.10538548	3.99152462	4.24215912
NONHSAG013737	2.41580188	2.37154572	2.40344499	2.5295488	2.41850315	2.61363898	2.26513698	2.4448854	2.25078041
NONHSAG013751	2.46248381	2.35124703	2.70923271	2.57067048	2.52323616	2.50015468	2.6771528	2.62406997	2.50431669
NONHSAG013766	2.20634873	2.21293184	2.32202555	2.25632184	2.40978744	2.30429138	2.33776388	2.17050848	2.14479065
NONHSAG013770	2.19396063	2.20637923	2.15972265	2.26483961	2.08302032	2.24865812	2.17329755	2.07909445	2.25712562
NONHSAG013789	4.54316555	4.72274613	4.83418594	4.70463523	4.8614249	4.80611902	4.81268314	4.60191272	5.19132287
NONHSAG013796	3.00331386	3.26716349	3.1692262	2.9631569	2.71393206	2.71757138	2.86818781	3.17469606	3.24011715
NONHSAG013836	3.73522875	3.60385855	3.78003995	3.62825062	3.96947206	3.74534555	3.49665889	3.67850776	3.95889148
NONHSAG013843	6.67215708	6.76626371	6.44628736	6.44922922	6.5360302	6.58912452	6.58771279	6.56021333	6.37473588
NONHSAG013856	3.1159256	3.45356162	3.32890533	3.33209753	3.40482613	3.44013553	3.34183353	3.52563029	3.45284705
NONHSAG013867	2.22058315	2.28399345	2.24633542	2.1092876	2.30348525	2.49903927	2.35588206	2.44175807	2.45214456
NONHSAG013871	2.6721346	2.70152708	2.64784552	2.89245924	2.64937656	2.71835109	2.95994964	2.87587069	2.70265103

NONHSAG013889	2.17733763	2.20352646	2.28011431	2.13168234	2.09125154	2.17733763	2.2908527	2.3341942	2.36129397
NONHSAG013908	2.20467989	2.26964014	2.45527834	2.24436127	2.36308033	2.31025641	2.3643999	2.5269204	2.47528907
NONHSAG013910	2.17148109	2.21676992	2.18519501	2.3691478	2.21541597	2.41015019	2.22407855	2.13657026	2.30347336
NONHSAG013921	3.29021508	3.20547328	3.21670839	3.30254548	3.22223975	3.4752051	3.35400247	3.48467593	3.12158166
NONHSAG013938	5.0217666	4.62985353	4.85704981	5.16233282	5.25868766	4.84345706	4.86998205	4.93335925	4.74377745
NONHSAG013940	4.57315875	4.49305026	4.64861099	4.39389691	4.57890618	4.27628054	4.60984169	4.37299784	4.76365697
NONHSAG013950	2.67384914	2.55300135	2.56108797	2.68395775	2.72650889	2.62549253	2.61600517	2.58749394	2.71278067
NONHSAG013963	4.76235357	4.97675713	5.07055807	5.16382113	4.90556088	5.09012807	5.22903198	4.80091519	5.39333389
NONHSAG013971	3.94411729	3.68439808	3.8147973	3.92844025	3.74118111	3.78974809	3.73698772	3.62902785	3.84270362
NONHSAG013976	3.87198058	3.77752335	3.70221775	3.78999826	4.10556637	3.82067181	3.76674888	3.72315225	4.0073135
NONHSAG013982	2.83468377	2.69691424	2.95250453	3.0771042	2.79841159	2.93564939	2.97640171	2.92343398	3.00860386
NONHSAG013992	4.13173265	4.21525022	4.17426262	3.99644247	3.98717351	4.295116	4.349834	4.01962469	4.04414734
NONHSAG014007	5.8737323	5.86005927	6.18422334	5.7514024	6.00223289	5.80275229	5.8014078	5.7065269	5.81165658
NONHSAG014012	4.29365811	4.28698464	4.31704313	4.46410584	4.34906094	4.26284706	4.27089759	4.36211919	4.27983207
NONHSAG014023	2.66127364	2.6963539	2.89967706	2.72462996	2.88259968	2.80482974	3.17432668	3.02504296	2.72502192
NONHSAG014030	5.34141221	5.49998385	5.33838529	5.6166404	5.40406423	5.51387865	5.56795441	5.3612844	5.16702061
NONHSAG014035	6.05890486	5.97479454	5.93601424	5.974925	5.96254262	6.09088799	5.75233368	5.94834837	6.1066717
NONHSAG014073	6.34849326	6.58915091	6.54160531	6.51408335	6.40729248	6.62118161	6.28127573	6.37061221	6.10009715
NONHSAG014079	3.77037148	3.86245889	3.79225591	3.95862755	3.81319075	3.85972026	3.77621396	3.67782276	3.94856661
NONHSAG014090	3.86738345	3.87260548	3.76655163	3.72245369	3.89913683	3.9031304	3.83471697	3.8318053	4.18511502
NONHSAG014099	3.57313868	3.47335168	3.75782534	3.84353753	3.78133143	3.84603397	3.60615213	3.91484458	3.84145511
NONHSAG014114	2.62743359	2.64273677	2.67969752	2.86273107	2.8102554	2.63745474	2.96540816	2.7348408	2.70299302
NONHSAG014130	3.2737618	3.27820708	3.2535703	3.48203589	3.26533308	3.35560267	3.29341635	3.31570006	3.63255494
NONHSAG014136	5.2487315	5.18294255	5.39720348	5.20171366	5.45533086	5.21253162	5.24772072	5.05478332	5.4407126
NONHSAG014165	2.85170751	2.71105044	2.79065942	2.68481082	2.91820187	2.90673276	2.93528022	2.86912917	2.98376734
NONHSAG014169	2.84658724	2.99426063	2.89061315	2.76690143	3.05054825	2.82206093	2.8192279	2.76532184	2.93313994
NONHSAG014185	3.15211769	3.09163857	3.40899311	3.12789659	2.97332346	3.05006329	3.14630001	2.947132	3.27166138
NONHSAG014197	2.52484738	2.27049737	2.51775377	2.86879913	2.68298043	2.69672877	2.87445253	2.52383734	2.57389936
NONHSAG014198	4.23340022	4.24103985	4.27076799	4.41472042	4.3705332	4.09894664	3.88963794	4.33558356	4.66248175
NONHSAG014202	4.3408455	3.92489251	4.14277665	4.14766486	4.32409073	4.21516883	4.4146097	4.07817584	4.14039009
NONHSAG014208	3.5894405	3.50819543	3.59020455	3.99686771	3.91103439	3.64934588	3.73473578	3.64692372	3.62987197
NONHSAG014212	2.97669837	3.01964229	3.10082887	3.28757326	3.09601214	3.41687223	3.20172593	3.07135153	3.16357873
NONHSAG014215	2.31001577	2.22990959	2.27493056	2.4198436	2.25658876	2.24248195	2.09085371	2.41831623	2.1601626
NONHSAG014232	4.31561182	4.18904984	4.43212844	4.23114091	4.23836864	4.50632657	4.41664108	4.02574663	4.1948648
NONHSAG014249	4.42173232	4.31955513	4.43650514	4.6908523	4.5387511	4.40136384	4.36192415	4.32826491	4.29909259
NONHSAG014269	4.76804717	4.74660639	4.70040471	4.69326473	4.76288233	4.7674516	4.68006081	4.66856453	4.76790129
NONHSAG014294	4.35507442	4.51662013	4.63618393	4.47738402	4.57201648	4.52660287	4.61991716	4.53570562	4.29737039
NONHSAG014358	3.67494061	4.03067395	3.49510265	3.76401666	3.45398007	4.00896396	4.00083833	4.04032808	3.58242474
NONHSAG014410	2.96636594	2.66802856	2.73174738	2.99664619	2.72009308	2.96265838	2.87144714	2.86006751	3.11722596
NONHSAG014436	2.84672094	2.82027789	2.92390012	2.96965461	2.91298558	3.02688131	2.83606828	2.88754872	2.90777423
NONHSAG014438	3.62848337	3.34826071	3.2990295	3.28841643	3.57093946	3.66434306	3.49261522	3.46928915	3.63029999
NONHSAG014440	2.48435509	2.65402209	2.66328481	2.44350532	2.52716454	2.39924138	2.58539616	2.49072322	2.66503718
NONHSAG014443	2.12988754	2.25755575	2.21059202	2.30433871	2.10725518	2.33103152	2.3308697	2.0897976	2.05074649
NONHSAG014445	3.46227575	3.38450189	3.37351115	3.55526479	3.76767531	3.41153288	3.45192271	3.6485087	3.41636622
NONHSAG014448	4.14431486	4.04417036	4.51662013	4.10341669	4.29107959	3.57749111	4.266041	3.88292083	4.84482743
NONHSAG014454	2.43471409	2.43297419	2.41985209	2.63346748	2.5758998	2.5829612	2.36299012	2.53456667	2.64937656
NONHSAG014569	3.14547687	3.22011917	3.21210852	3.13468102	3.44554142	3.89610046	3.00793059	3.38140057	3.41045571
NONHSAG014574	2.56252012	2.53172331	2.51347429	2.67812119	2.38425971	2.45687758	2.55106937	2.50311382	2.45122815
NONHSAG014590	2.66416791	2.92695402	2.96331715	2.99305774	2.77450097	2.84485768	2.79603759	2.76837073	3.18121507
NONHSAG014611	3.25936472	3.20885719	3.30695242	3.10186721	3.33452186	3.22205147	3.48594365	2.98157358	3.53081014
NONHSAG014661	2.68758361	2.68401863	2.75990479	2.82151637	2.78654577	2.84318623	2.72006501	2.80042139	3.34206149
NONHSAG014663	6.11097169	6.58232096	6.38601506	6.40020292	6.417349	6.49985411	5.9993265	6.30986465	6.3329475
NONHSAG014667	3.68098485	3.59944393	4.01160676	3.42250547	4.07860242	3.99280531	3.77334459	3.74418852	4.16363546
NONHSAG014746	2.289135	2.16827153	2.10985461	2.23897864	2.27868176	2.43471409	2.44825941	2.2385253	2.36327358
NONHSAG014747	2.29669047	2.13591181	2.14452925	2.48218843	2.20329134	2.22999699	2.04227234	2.10429397	2.21101854
NONHSAG014790	4.95143359	4.65001652	4.98746728	5.03957206	4.97566433	4.9706838	4.95541017	4.98053942	5.0149716
NONHSAG014806	3.9496222	4.21132058	4.22995702	4.18852303	4.34983118	4.42332858	3.98809977	4.47103493	4.48751183
NONHSAG014811	2.26052527	2.47534164	2.58497511	2.38631498	2.34100537	2.3107581	2.36067996	2.19899539	2.17517274
NONHSAG014813	2.73665661	2.76401933	2.79492316	2.81817172	2.89540018	2.64662456	2.64227273	2.45274404	2.42258962
NONHSAG014829	4.32551468	4.12345061	4.01426634	4.21350903	4.27134543	4.10470639	4.14479629	4.057778	4.03325438
NONHSAG014834	4.02848456	4.25124941	4.42825149	4.13835615	4.04576253	4.29543581	4.18961857	3.95647164	4.3466904
NONHSAG014845	3.54478242	3.23780491	3.44820988	3.82697362	3.54251556	3.84921434	3.59805578	3.96703444	3.6609577
NONHSAG014849	2.9650544	2.92662971	2.96565127	3.00263426	2.7036278	2.92170429	3.08704339	2.82432359	2.72663583
NONHSAG014850	2.10989913	1.92665237	2.18480351	2.24451362	2.19719623	2.25739089	2.47225694	2.56633926	2.62587344
NONHSAG014898	3.89123646	3.89158531	3.70229556	4.0507634	3.79469933	3.64396568	3.30682406	3.68325181	3.78426851
NONHSAG014905	4.84202593	4.97467995	4.88880859	4.5342481	5.05492132	4.97962432	4.7230607	4.86318565	5.08163824
NONHSAG014910	5.96982499	6.1545929	6.0039413	6.02249692	5.91297688	6.3389042	6.33911378	6.23651193	6.00629443
NONHSAG014924	5.72169932	5.78837009	6.0957783	5.81716473	5.65536825	5.94394782	5.91305295	5.82746636	5.70537921
NONHSAG014926	4.9675668	4.99077784	4.55740331	4.70270975	4.45096289	4.91673743	4.68974478	5.07140174	4.54059913
NONHSAG014964	2.05083153	2.11404207	2.13830451	2.28051691	2.38418839	2.32067379	2.17598659	2.23943106	2.12965085
NONHSAG015027	7.21616725	7.56989963	7.27708606	6.94017281	7.13737589	6.92798156	6.97574298	7.07500093	7.28867669

NONHSAG015035	2.46380306	2.46327094	2.72244386	2.85463455	2.55200518	2.31091068	2.87695028	2.52768119	2.87179464
NONHSAG015052	3.86596433	3.85891735	3.9248009	3.82656844	3.86374691	3.94051399	3.9647804	3.99038197	4.00589919
NONHSAG015063	2.99014602	2.91560538	3.03717802	3.01073822	3.03405942	3.05521641	3.16452472	2.87962528	3.05077566
NONHSAG015092	2.53288143	2.72090195	2.77295867	2.57575011	2.55653594	2.95197971	2.52274554	2.75111791	2.63372457
NONHSAG015143	5.65640215	5.79831024	5.63829023	6.04333561	5.91967023	5.96144287	6.16004	5.94914669	6.26575505
NONHSAG015144	4.5411645	5.13330515	4.69032332	4.90865805	4.73030405	4.88439913	4.71408527	4.88922285	4.79482349
NONHSAG015185	2.18544917	2.33676757	2.16627199	2.35534508	2.31899594	2.23937613	2.38807471	2.35919476	2.13305316
NONHSAG015188	2.67179018	2.74925124	2.72523147	2.8141943	2.91996569	2.95668117	2.80940345	2.96730094	2.57103838
NONHSAG015211	2.78443899	2.5547319	2.98554256	2.90587853	2.72632874	3.23585728	2.96719802	2.72404466	2.86629243
NONHSAG015231	5.8980596	6.0494968	5.96357214	6.26345329	5.9435076	6.32885349	6.17950144	6.06819552	5.89812477
NONHSAG015256	2.84977374	3.02079534	2.94544518	2.90417453	3.11872042	3.25502652	3.12387872	2.80552848	3.15477619
NONHSAG015273	3.39567736	3.37493497	3.47490193	3.75708027	3.55535485	3.42099446	3.49199262	3.43984956	3.70676771
NONHSAG015286	5.28428999	5.22243061	5.29155601	5.4689042	5.40266846	5.31134375	5.71552718	5.12210895	5.27792437
NONHSAG015320	2.49831811	2.443642	2.43454768	2.27038453	2.51210658	2.60885048	2.43816287	2.38343582	2.66167015
NONHSAG015338	2.81704522	2.96226059	2.65532146	2.57160434	3.02258337	2.87657292	2.85684717	2.92276119	2.73643367
NONHSAG015357	3.75893346	3.76991839	4.0375286	3.78525011	3.70360691	3.7724469	3.78995903	3.70448461	4.05145367
NONHSAG015371	4.31886759	4.19031234	4.54819167	4.40306795	4.50409821	4.42274322	4.68439415	4.37921808	4.35280851
NONHSAG015373	2.93898656	2.7907752	2.77895572	3.02731012	3.0000911	2.85657869	2.8889393	2.79736449	2.98141536
NONHSAG015387	5.91728497	5.60743445	5.70677821	5.91021786	6.17571421	6.07213526	5.81929575	5.57286333	5.84741687
NONHSAG015435	4.08943089	3.99736256	4.07646816	4.20716978	4.19152696	4.06534182	4.03742262	4.04571324	4.38928396
NONHSAG015438	2.80965183	2.73546937	2.61501003	2.40478818	3.05178401	2.78586472	2.85123066	2.49589008	2.69565182
NONHSAG015450	2.39354056	2.40385204	2.59741447	2.68864217	2.37388832	2.80421322	2.8792941	2.35620992	2.40851808
NONHSAG015466	4.42348547	4.38776743	4.68952089	4.76966706	4.97836132	4.91444014	4.58172515	4.74841811	4.87174479
NONHSAG015491	3.448617	3.34242237	3.52040333	3.68233994	3.4101179	3.26536968	3.28734606	3.44460978	3.88295845
NONHSAG015511	7.71362315	7.93172549	7.55396471	7.5308655	7.48945074	7.77831683	7.79246832	7.73922992	7.79963027
NONHSAG015535	2.28828709	2.28660664	2.18830231	2.30984789	2.15310741	2.33000015	2.16107905	2.22320738	2.33537324
NONHSAG015539	3.61808488	4.15133164	3.46183236	3.97964662	3.81465603	4.12341447	3.36689745	3.4819962	3.60548509
NONHSAG015555	5.84299192	5.83173741	5.8313347	5.1030378	6.03636096	5.75222753	5.54245564	5.99356154	6.20490723
NONHSAG015654	2.92967005	2.81987844	3.05941757	3.02704931	3.2621793	3.2086941	3.12382304	3.01308852	3.19358504
NONHSAG015660	3.29194013	3.46370416	3.60275884	3.34514702	3.71678898	3.75020975	3.31342318	3.50152312	3.60039723
NONHSAG015664	3.16615773	3.23749701	3.31115054	3.12352237	3.22717237	3.1648694	3.258404	3.37169126	3.1299349
NONHSAG015694	6.45705318	6.3486445	6.52210507	6.36235548	6.61994185	6.48212079	6.53258121	6.36492863	6.72212796
NONHSAG015695	4.44023868	4.25820409	4.51558564	4.21057428	4.66141771	4.5105732	4.57410364	4.4101317	4.53152173
NONHSAG015708	3.60052508	3.68275876	3.69721587	3.79514307	3.47125459	3.86648337	3.68553381	3.9487346	3.64371359
NONHSAG015712	5.39830049	6.13807601	5.34378386	5.35125546	5.22021187	5.48213596	5.58436777	5.32048594	5.49902764
NONHSAG015718	3.32760519	3.26559475	3.54072498	3.22302834	3.37489694	3.511699	3.16896784	3.11226654	3.33488124
NONHSAG015732	5.98094151	6.12064128	5.93508452	6.22427879	6.16341006	6.21872399	6.1837076	6.20312335	6.30205782
NONHSAG015756	2.95506173	2.98106839	3.15518539	3.05201793	3.12568385	2.99664616	3.02221912	3.05999734	3.4352713
NONHSAG015757	4.91061552	4.7524661	5.01282922	5.10608909	5.00260768	5.10341236	4.93346553	4.88582859	5.04199379
NONHSAG015797	3.77433469	3.73349262	3.69554686	3.80881608	3.78145384	3.89711019	4.03930615	3.85424148	3.92263955
NONHSAG015798	3.88064585	3.88684278	4.44015152	3.90537431	4.22095926	4.13044181	3.72338762	4.38471491	3.87159402
NONHSAG015800	2.27656374	2.30117798	2.39619714	2.28582464	2.26855714	2.27798982	2.39205287	2.32787291	2.39938898
NONHSAG015828	5.42233565	5.34590329	5.37627555	5.32997703	5.31105746	5.52187029	5.46024146	5.44138883	5.45089793
NONHSAG015833	3.1484855	3.11176199	3.09284163	3.10262341	3.34169014	2.92204669	3.11998275	3.00897093	2.90730206
NONHSAG015852	2.7931932	2.82070232	2.86587531	2.66195617	2.48098447	2.716325	3.01144965	3.40713353	3.13566589
NONHSAG015872	5.33127421	5.49999957	5.38426715	5.36839195	5.66067289	5.50387037	5.54790803	5.51407762	5.70611067
NONHSAG015874	3.34649659	3.49065131	3.1195518	3.478394	3.60413679	3.07160642	3.00448711	3.47894496	3.40641585
NONHSAG015923	4.00474622	3.94872694	4.09582933	3.94748601	4.03893434	3.98301421	3.84781374	3.98729765	4.15662884
NONHSAG015927	2.7234625	2.64978171	2.67531209	2.63251169	2.78304396	2.6401767	2.7907752	2.55433403	2.74771369
NONHSAG015948	4.02154843	3.89764985	3.94647359	3.85907025	4.11045375	3.85457786	3.96803798	4.21259946	3.57946708
NONHSAG015952	4.32454237	4.28813077	4.20016335	4.3717043	4.16479585	4.13085935	4.24092823	3.99010661	4.26308577
NONHSAG015966	5.29640953	5.66108166	5.20132335	5.67306563	5.43225361	5.35644567	5.22508447	5.40966571	5.41169876
NONHSAG015980	6.48382409	6.62887948	6.32446913	6.30166568	6.73063661	6.43125096	6.60664824	6.80023403	6.82239468
NONHSAG015981	5.95215398	6.03606694	5.81374269	6.18994088	5.68120289	6.19384039	6.02910671	5.97598933	5.79292556
NONHSAG016009	3.18931136	3.25657826	2.90569801	3.47476518	3.3781158	3.06263186	3.19631313	3.36919526	2.76165135
NONHSAG016021	5.26064148	5.03638223	5.13376651	4.98749632	5.40459587	5.14633866	5.39445761	5.13988521	5.58740172
NONHSAG016024	3.80922606	3.43604118	3.61134821	3.83399415	3.54969631	3.70534408	3.32247456	3.59723113	3.26919823
NONHSAG016025	4.16573526	3.85777909	4.31391564	4.52244901	4.18982473	4.33244886	4.11266015	4.2612423	3.81367486
NONHSAG016027	7.03723125	7.26722261	7.1983144	7.20087053	6.90417348	7.13514072	6.79759417	6.96147121	6.92179314
NONHSAG016062	3.21398268	3.27910525	3.25357026	3.33877012	3.29449382	3.32527667	3.3463189	3.50462032	3.61660919
NONHSAG016074	9.62732613	9.3120851	9.38354059	8.82340054	9.69961591	9.20050815	9.43180529	9.21034104	9.73805288
NONHSAG016107	2.54906688	2.5698479	2.79717308	2.67790079	2.79901965	2.65310107	2.52274554	2.54906688	2.52313242
NONHSAG016109	4.55445647	4.5519577	4.41178463	4.60069395	4.48751653	4.56029403	4.52631915	4.49055908	4.52320865
NONHSAG016111	5.45654925	5.24721898	5.45682803	5.56111766	5.57629568	5.52604976	5.49515917	5.33468794	5.47353768
NONHSAG016116	2.94620439	2.69020817	2.95648441	2.91548203	2.59179815	2.64444413	2.89485173	2.56172295	2.7249921
NONHSAG016136	2.78350457	2.64959635	3.52874859	3.82117555	3.17669625	3.21342237	2.72766861	2.96749148	2.89264461
NONHSAG016155	3.0185638	2.97172334	3.25713814	3.53133456	3.56391926	3.09178679	3.62716871	2.8443743	3.69543198
NONHSAG016158	3.18370933	3.31211434	3.33662215	3.66897109	3.61048388	3.43048524	3.47480527	3.90147993	3.25801305
NONHSAG016166	2.59129912	2.43565522	2.47420249	2.71993738	2.7103445	2.15020642	2.81794447	2.42554644	3.24611871
NONHSAG016202	3.22295859	3.29721051	3.39479518	3.19540935	3.35128649	3.55612945	3.08072427	3.56644987	3.64521988
NONHSAG016213	2.25402432	2.19998494	2.09021977	2.13120199	2.09530165	2.19246941	2.36505811	2.18880933	2.27862664

NONHSAG016262	3.25452063	3.51426565	3.30586201	3.41522605	3.57030233	3.62579268	3.66637631	3.50553025	3.57793363
NONHSAG016297	2.64177103	2.32442973	2.58774142	2.40533592	2.60233648	2.71564409	2.7355253	2.52688873	2.36892301
NONHSAG016313	3.11687143	2.90433314	2.9619265	3.27196453	3.32593373	3.22789769	3.09002417	3.15216463	3.06487611
NONHSAG016316	2.18457372	1.98583795	2.27352746	2.20141003	2.1250544	2.26962507	1.96663911	2.09321776	2.27740551
NONHSAG016330	3.00674433	2.83768589	2.59301077	2.80017225	2.72569345	2.69140121	2.92571199	2.71333225	2.66617068
NONHSAG016336	5.24652713	4.8006283	5.03232007	5.04996137	5.11383053	5.06722651	5.20862417	5.01786644	5.25078268
NONHSAG016353	2.44693708	2.09885324	2.36338045	2.3358189	2.27875768	2.6070259	2.20060806	2.56003782	2.4515896
NONHSAG016383	3.91675614	3.89578346	4.03383871	4.11633743	4.23459764	4.15673122	4.59250298	3.93808849	4.42419069
NONHSAG016385	3.06104228	3.04278594	3.30765924	3.15523733	3.23724854	3.26505252	3.2963562	3.00673112	3.2390177
NONHSAG016388	5.04298031	4.94870196	4.84747495	4.83407783	4.91721593	5.47852868	4.89823518	5.03640431	5.36388076
NONHSAG016419	5.32509606	5.68151455	5.40459718	5.30045503	5.20264352	5.44261043	5.02343642	4.45091316	4.91142362
NONHSAG016427	5.2398599	5.2418712	5.36580169	5.47904464	5.44959674	5.4500706	5.51875608	5.40270567	5.37816153
NONHSAG016434	3.01528666	3.04225703	3.13166365	3.24240451	2.85583383	3.1537958	2.99959418	2.71685093	3.0994699
NONHSAG016460	3.48414347	3.14889428	3.39770585	3.47714683	3.43181541	3.21738623	3.30541768	3.31439253	3.69593626
NONHSAG016472	5.8134225	5.66635616	5.96109553	5.92905576	6.06486307	5.86102266	5.82581754	5.91700409	5.95839566
NONHSAG016517	3.79082331	3.43823361	3.68430595	3.59548981	3.88485879	3.81236791	3.41490413	3.83631774	3.76459729
NONHSAG016520	2.09897221	2.07083638	2.21281426	2.09270339	2.23169618	2.22942218	1.98972738	2.17954589	2.12114135
NONHSAG016540	3.26263431	3.12773786	3.45144365	3.36926611	3.69936632	3.30005635	3.39150966	3.71604313	3.96832656
NONHSAG016542	3.14765925	3.16083131	3.28368626	3.22731876	3.22827373	3.24490723	3.13449373	3.14765925	3.24630003
NONHSAG016544	2.19933193	2.25951813	2.49337026	2.64556466	2.3480384	2.28182577	2.05522865	2.28067454	2.20349017
NONHSAG016555	3.73178083	3.97780333	3.6772029	3.45791297	3.65739409	3.61479399	3.92851629	3.98040216	3.84268773
NONHSAG016582	4.13081715	4.15432393	4.0575637	4.04816444	3.94155142	4.41453534	4.30995303	3.89195886	4.23087955
NONHSAG016589	6.59633005	6.54596407	6.60196916	6.67307277	6.52067873	6.612402	6.73873768	6.71564621	6.68176244
NONHSAG016595	4.17298767	3.92489251	4.36153634	4.4016125	4.07433831	4.4179552	4.26464594	4.14646192	4.25239616
NONHSAG016623	4.2393835	4.1130833	4.24073246	4.18165489	4.41314793	4.07111112	4.14263414	4.23547786	4.53211077
NONHSAG016650	3.35370888	2.88383926	3.24313424	3.18026768	3.49500653	3.08616911	3.42065139	3.07997225	3.28408874
NONHSAG016655	3.23236567	3.30957529	3.1831656	3.29872384	3.4620187	3.27276432	3.42513544	3.34280669	3.35411596
NONHSAG016687	4.1450946	3.81089376	3.66040962	3.81161583	3.93523941	3.78775329	3.58927717	4.09772317	3.86515449
NONHSAG016786	6.25779881	6.17157328	6.36864676	6.30216203	6.07326004	6.3355038	6.42646962	6.68724292	6.31262008
NONHSAG016826	2.58638138	2.61751337	2.65613815	2.5615774	2.73057508	2.80434341	2.78995693	2.8443743	2.65930168
NONHSAG016852	3.32218175	3.67417694	2.79355941	3.16484597	3.34292763	3.55234941	3.3962993	3.7582624	2.6574
NONHSAG016854	2.57463669	2.50353022	2.79274234	2.55452565	2.57846293	2.80451811	2.65315908	2.711694	2.84782767
NONHSAG016887	3.55104307	3.63870061	3.22445231	3.72698247	3.48875248	4.04468132	4.11984499	3.97647245	4.04210819
NONHSAG016890	2.70440177	2.67030081	3.01234428	2.22657987	2.71593764	2.60959753	2.56887778	2.25402432	2.38920195
NONHSAG016921	3.47715975	3.34546153	3.47317699	3.30717066	3.40652797	3.41712467	3.5252462	3.23318835	3.46633005
NONHSAG016956	5.77059197	5.59418658	5.85762802	5.65632196	5.80908487	5.70299701	5.89123313	5.50040479	5.77701974
NONHSAG016984	2.52278672	2.76665779	2.75253888	2.59182612	2.78162486	2.61257663	2.52790771	2.62814732	2.70359812
NONHSAG016991	5.99929195	5.75136821	5.89771826	5.5669861	5.87669924	6.00739343	6.21385974	5.78755086	5.85409439
NONHSAG017020	2.74448367	2.75156507	2.92256131	2.86615621	2.89872106	2.73356713	2.72974449	2.9740101	2.80469735
NONHSAG017021	3.71788309	3.56785304	3.77983528	3.65125129	3.9460191	3.63260398	3.67816109	3.75746243	3.8046749
NONHSAG017030	4.50198603	4.51213961	3.86286936	4.22041661	4.45972725	4.36789828	4.15170458	4.58228376	4.30897267
NONHSAG017048	2.80739553	2.60225069	2.81462378	2.65079892	2.29227334	2.59005319	3.47770682	2.67901097	2.94327136
NONHSAG017077	5.85916417	5.83797344	6.06640721	5.82303363	6.12604444	5.87871289	6.11279878	5.7397623	6.26438081
NONHSAG017083	3.61316564	3.44746092	3.55992676	3.80570203	3.68637025	3.8444268	3.67924193	3.52865198	3.72600168
NONHSAG017088	2.84199775	2.94905051	2.88486646	3.24459426	2.95233081	3.17461342	2.94971868	3.3022468	3.26947163
NONHSAG017091	2.64425865	2.51340518	2.96296551	2.49476728	2.59881827	2.57795247	2.87070102	2.63883452	2.88354659
NONHSAG017106	3.04122113	3.10148712	2.63328974	3.45094447	2.86221239	2.6895036	3.05890475	3.31487761	2.89920463
NONHSAG017112	4.46121553	4.109549	4.63519746	4.22039295	4.56022214	4.40080261	4.2486193	4.36239195	4.92100232
NONHSAG017118	3.56691303	3.58516471	3.58441354	3.57283715	3.54074309	3.56842312	3.57929197	3.59424528	3.48041882
NONHSAG017164	4.80046402	4.73460011	4.49978704	4.48912436	4.88909906	4.95821589	4.71158698	4.8946985	4.50042517
NONHSAG017207	3.94301478	4.1122463	3.89590014	4.72150357	3.82281847	5.11677001	4.50402651	4.51175915	5.00369963
NONHSAG017233	2.99014602	3.13774203	2.90569801	2.87325208	3.22827373	3.18401238	2.96759765	3.16670915	3.22541726
NONHSAG017237	6.39386093	6.48221125	6.38223494	6.52951681	6.7440735	6.74716443	7.21548576	7.14927862	7.54551866
NONHSAG017254	4.5933811	4.55819314	4.90021629	4.72172023	4.9748596	4.73996145	4.90122832	4.90035593	5.07960564
NONHSAG017262	2.94349341	2.9119	2.73256857	3.08619923	2.83154066	2.78717051	3.06006283	2.71131459	2.81997041
NONHSAG017302	3.45275927	3.47902158	3.5238194	3.13782339	3.43173988	3.47910382	3.74710125	3.63215016	3.60254134
NONHSAG017304	3.20082875	3.11161418	3.00964329	3.25854476	3.20710307	3.09124621	3.02440703	2.79270471	3.15253743
NONHSAG017314	3.8100666	3.71412193	4.1779436	3.8537266	4.17692007	3.89545727	3.70466169	3.87758428	4.19117616
NONHSAG017331	7.90022229	7.97483224	7.78174437	7.57323151	7.69443074	7.8521137	7.90947746	7.82420705	7.93013867
NONHSAG017346	4.99099801	4.83036873	5.00758017	4.91935947	5.13073292	4.96123947	4.90114161	5.12178761	5.08834268
NONHSAG017352	3.57372806	3.2492285	2.86294589	2.68403875	2.7789723	3.06862769	3.13290597	3.02420956	2.96431116
NONHSAG017356	4.38973211	4.46781538	4.27485285	4.34614574	4.668333	4.63406009	4.26663098	4.29175815	4.30230173
NONHSAG017360	4.05740182	3.86006945	4.00742109	3.94209874	4.17216685	4.19199686	3.86018215	4.35149422	4.1571242
NONHSAG017376	4.90529566	4.96471331	4.65558647	4.94185843	4.83676786	5.07776354	5.06203814	4.81160879	5.48442537
NONHSAG017384	5.54756014	5.56349711	5.35314908	5.69996798	5.53936293	5.73066869	5.33385602	5.46871913	5.28137253
NONHSAG017387	4.36135683	4.29340324	4.39195918	4.49750525	4.69757801	4.25822016	4.52572271	4.32752461	4.21960339
NONHSAG017486	4.46796516	4.56931914	4.64599115	4.3262084	4.48057171	4.44627146	4.46284665	4.40230228	4.42521216
NONHSAG017508	2.35973698	2.33475808	2.43554777	2.35557584	2.52569893	2.61924286	2.23560232	2.50212292	2.46479677
NONHSAG017514	2.71948591	2.62244045	2.43562622	2.49124453	2.45057374	2.55866006	2.46384193	2.42173729	2.41232192
NONHSAG017529	5.23485862	4.935727	5.15276264	5.31963799	5.46369982	5.1981918	5.39204575	5.12215245	5.403114
NONHSAG017532	3.40179571	3.37682677	3.26979134	3.8208083	3.30740548	3.09461904	3.29162126	3.28473284	3.25157466

NONHSAG017549	3.32066035	3.27669444	3.44391944	3.14203409	3.53848055	3.27861314	3.32871187	3.30296912	3.62967486
NONHSAG017579	2.4033405	2.42257651	2.36375063	2.37582985	2.4646695	2.36435738	2.43986007	2.51375619	2.49886355
NONHSAG017593	4.53104037	4.6013877	4.77284176	4.77764609	4.65021887	4.71807729	4.64548762	4.83993094	4.88310501
NONHSAG017594	4.99551339	4.78247816	5.00864119	4.98433632	4.99937937	5.20472302	4.80954101	4.78229371	4.94489913
NONHSAG017604	3.44873631	3.1864755	3.34712744	3.46010502	3.19130338	3.27898093	3.1099691	3.03025834	3.64858437
NONHSAG017613	4.13775291	4.19995139	4.2585302	4.30414609	4.18885303	4.25376165	4.22400416	4.21396959	4.32886828
NONHSAG017640	2.30602089	2.44232242	2.44765046	2.18617551	2.4978297	2.52774314	2.5002029	2.43880427	2.24295728
NONHSAG017669	2.85218556	2.80237836	2.90674183	2.71934649	2.93000874	2.80103588	2.85466887	2.77924309	3.01658647
NONHSAG017670	3.3010398	3.60057654	3.45995994	3.55696565	3.70623406	3.53311958	3.71046963	3.38414918	3.33878021
NONHSAG017671	2.94849298	2.51443063	2.87339512	2.6781399	2.85725127	2.96440396	2.97328453	2.78714638	2.94542511
NONHSAG017675	6.32854902	6.41977675	6.17484872	5.84538882	6.39228902	6.02022663	6.27877401	6.26067348	6.34576763
NONHSAG017708	5.71029972	5.82313393	5.72378635	6.21719219	6.05606881	6.09749184	6.02482032	6.0047071	5.97052767
NONHSAG017741	4.44904004	4.27413978	4.52531548	4.63068216	4.5482872	4.45734985	4.58460876	4.50968633	4.65878183
NONHSAG017742	4.26806313	4.25420754	4.19622583	4.4410852	4.42213985	4.4540924	4.39848063	4.41382881	4.56522868
NONHSAG017775	2.88942955	2.651172	2.97133712	2.67793238	3.14800646	2.74042941	2.92070043	3.18972731	3.21574915
NONHSAG017777	3.04984726	3.05484184	3.34257121	3.29614615	3.41031391	3.34029721	3.26268246	3.31439253	3.0712445
NONHSAG017785	2.82893431	2.76050308	2.990321	2.93102594	2.71957584	2.81194771	3.06995875	2.79153952	2.72730304
NONHSAG017797	5.76458131	6.06521653	5.64426255	6.07781692	5.95062225	5.75309093	5.89065067	5.96669991	5.88181078
NONHSAG017837	5.36871579	5.41202516	5.42694642	5.46794516	5.31917771	5.21813343	5.12522656	5.25203372	5.30508215
NONHSAG017855	4.78311236	4.85216788	4.89382053	5.17826906	5.00602906	5.00177921	4.8130455	4.71716485	5.48213314
NONHSAG017868	4.8092034	4.8092418	4.77673046	4.25042414	4.63029893	4.96697422	4.90319125	4.94835866	5.08528092
NONHSAG017869	7.95985617	7.88477659	7.86542717	7.54382294	8.05263415	7.7366259	8.01310918	7.83777083	8.00172513
NONHSAG017885	3.64076105	3.69204046	3.652442	3.72277577	3.8471941	3.69573206	3.85152532	3.94704668	3.90699614
NONHSAG017934	3.54002921	3.80263047	3.65524839	3.2509449	3.71259012	3.73012502	3.56951192	3.55837656	3.77491181
NONHSAG017937	4.82171181	4.5739517	5.01523544	5.16728477	5.182321	4.92628663	4.86633373	4.81100304	4.9418053
NONHSAG017938	3.56458725	3.39222203	3.37141469	3.64776538	3.57214143	3.59299381	3.65927681	3.49362008	3.49409787
NONHSAG017946	3.84605142	3.78513925	3.78939875	4.01428291	3.72250875	3.90311192	4.0447091	3.9385637	4.046749
NONHSAG017952	5.80599014	5.57864491	5.85968146	5.47320702	5.66317251	5.53112445	5.90059569	5.75674332	6.00151365
NONHSAG017967	2.66220775	2.66372931	2.87491137	2.76198448	2.93502666	2.69192652	2.89833963	2.52421208	2.80079873
NONHSAG017971	3.91862903	3.68393279	3.97797532	3.9479141	3.87970202	3.82199611	3.83323906	3.80334179	3.93721756
NONHSAG017972	2.90588485	2.95550397	2.78277562	2.83179041	2.74467374	3.01321301	2.82712347	2.54730735	2.76026804
NONHSAG017996	4.18829257	4.14265361	4.0038033	3.97189545	4.07344397	4.03984613	4.08619273	4.13446642	4.26606394
NONHSAG018000	3.07259898	3.20896152	3.69167566	3.28834721	3.25142059	3.27594262	3.26975162	3.1511855	3.24144488
NONHSAG018018	5.12577919	4.90222712	5.02910172	5.08794772	5.2189612	4.98685894	4.95139123	5.04654346	5.1819248
NONHSAG018031	3.06885853	3.21046925	3.17152432	3.15388793	3.05306099	3.42046458	3.37156829	3.2256795	3.28631462
NONHSAG018033	3.0967752	2.83273324	3.10619646	3.0299937	3.1913613	2.96239674	3.00310949	2.97433598	3.48217532
NONHSAG018039	3.74159898	3.46759875	3.682529	4.03196701	3.64914489	3.56121252	3.81777016	3.47225473	3.90584209
NONHSAG018040	3.17461342	3.27460167	3.09482462	3.25416348	3.05178401	3.09178679	3.20991782	3.23319415	3.27460167
NONHSAG018059	3.60292997	3.49739582	3.61592146	3.59454306	3.73500829	3.73271468	3.63165843	3.65896886	3.84644205
NONHSAG018075	4.39820792	4.45303733	4.65337582	4.94215574	4.83485317	4.73728798	4.79540334	4.51249224	4.43179676
NONHSAG018098	3.95303171	3.74989771	4.04593221	3.47664255	3.90217927	3.71947681	4.15673122	3.57604839	4.15521729
NONHSAG018192	4.91036258	5.05981447	4.94983126	4.98749632	5.1679729	5.21197853	5.02977194	4.99344231	4.69362478
NONHSAG018224	5.55371019	5.60453415	5.60765011	5.62648938	5.53836609	5.63990906	5.58596547	5.62462306	5.44635464
NONHSAG018230	5.65211335	5.64770614	5.47497116	5.47785975	5.44106894	5.38410167	5.55159706	5.58679581	5.6975684
NONHSAG018271	3.60973691	3.66401372	3.61844635	3.57214143	3.68529893	3.76227712	3.79134243	3.68837111	3.94673151
NONHSAG018315	11.30729363	11.12063765	11.11239998	11.17005377	11.18445899	11.15078644	11.34685833	11.32921182	11.26152515
NONHSAG018332	4.07350737	4.15036685	4.08904826	3.88827829	3.72534362	4.04971467	3.62540614	4.59124749	4.13304727
NONHSAG018336	4.59188952	4.49884259	4.88058249	4.72748395	4.75235567	4.5504426	4.70882493	4.62007623	4.77980997
NONHSAG018350	3.72351155	3.83052521	4.12956617	3.55111963	3.89213485	3.74724449	4.00133445	3.62968206	3.62558573
NONHSAG018377	5.96450452	5.90690304	6.09392624	5.93953997	5.88681675	6.04586192	5.92491704	5.75169387	5.86259955
NONHSAG018379	6.4493599	6.5236343	6.40553704	6.63465647	6.60916788	6.55287402	6.53916682	6.47813316	6.71596082
NONHSAG018419	4.17859926	4.62304964	4.08593079	4.60943158	4.49573562	4.66383278	4.1611794	4.55252056	4.49095782
NONHSAG018426	3.53530091	3.65256284	3.52890993	3.45901194	3.45181534	3.3239738	3.48594365	3.65596529	3.25612525
NONHSAG018457	4.8059098	4.59593605	4.46219569	4.66261214	4.74301272	4.50813891	4.61270685	4.65149496	4.74241669
NONHSAG018506	3.74914493	3.76444811	4.00716845	3.89118519	3.91019016	3.71947681	3.78355393	3.97390008	3.64033197
NONHSAG018536	5.12076854	5.10372977	4.94203816	5.04934478	4.7496591	5.25713138	4.82707026	4.9410686	4.44365693
NONHSAG018538	8.38322923	8.80539882	8.42223224	8.31962913	8.4194022	8.38784156	8.29882118	8.31874477	8.39210651
NONHSAG018575	3.95759115	3.82490846	3.90795102	3.90921932	4.10091817	4.43896408	4.30256007	3.99344524	4.348721
NONHSAG018592	5.0791692	5.20349726	5.33046062	5.03863126	5.11404566	4.78646216	5.19602454	4.78337106	5.16185322
NONHSAG018593	4.74712106	4.67951633	4.96855942	4.75604199	4.95562904	4.92141452	4.86626241	4.78349789	5.17641912
NONHSAG018602	3.99201316	4.18025316	4.0170759	4.03764813	4.24387319	4.05780059	4.0398833	4.1399761	4.11353083
NONHSAG018627	6.451763	6.2692511	6.55779182	6.12040619	6.63801577	6.52642341	6.49104104	6.29440332	6.36541776
NONHSAG018649	5.07499812	4.66213788	5.05381106	5.14993255	4.83293154	5.00809932	4.99292677	4.96742063	5.16700491
NONHSAG018664	5.49294922	5.50186871	5.72989276	5.8452026	5.86873249	5.98832992	5.82023068	5.72422482	6.09297995
NONHSAG018700	2.90830771	2.66802856	2.90337474	3.07881977	2.73933107	3.10396207	3.08595533	2.87274407	3.10927143
NONHSAG018743	3.90725169	3.62937443	3.91253607	3.60824215	3.55817819	3.71927624	3.97537964	3.53074187	3.50928725
NONHSAG018775	2.24291603	2.43702906	2.29689303	2.46080994	2.2716534	2.22999699	2.5377697	2.56652898	2.45850892
NONHSAG018846	6.06421237	6.25050323	6.2160063	6.49075308	6.3138679	6.48018471	6.30612476	6.23141422	6.36558255
NONHSAG018860	6.01374996	6.14840233	5.96993834	5.99214494	5.80558033	5.82907555	6.00840539	6.00288511	6.23540933
NONHSAG018901	4.13484834	3.82231283	3.78939875	3.80544958	3.79326247	4.03757631	3.80403404	3.96749259	3.40661481
NONHSAG018925	5.07804225	5.08036347	5.02742688	4.97261331	5.28174405	5.04805424	5.12799903	4.94782116	5.54897751

NONHSAG018928	3.34916893	3.12535658	3.27631797	3.11009052	3.32353524	3.04298756	3.54501992	3.13601867	3.10840371
NONHSAG018930	4.64936828	4.27236356	4.7275377	4.20355429	4.94296629	4.5292575	4.71425161	4.56662234	4.59704021
NONHSAG018934	6.15877048	6.41954759	5.93132964	6.22417869	5.95953283	6.23522634	6.28121584	6.20800415	6.08863301
NONHSAG018936	3.05996864	3.05066424	2.90623289	3.06142194	3.31798743	3.23186925	3.19614129	2.84323408	3.26920378
NONHSAG019021	3.20928504	3.37464388	3.39599187	3.37877496	3.57082434	3.40310053	3.53145385	3.2431382	3.35485462
NONHSAG019026	3.80721791	4.02511941	3.78024742	3.92616972	3.7583111	3.81008994	3.56204358	3.91073344	3.93168226
NONHSAG019061	4.45525111	4.36986744	4.3907365	4.48435965	4.52470975	4.60059217	4.41358538	4.32864623	4.50501741
NONHSAG019100	5.4050738	5.64911504	5.37998819	5.56219454	5.36346287	5.69270685	5.44776982	5.32133048	5.49457809
NONHSAG019129	5.73114648	5.88725863	5.68412876	5.85510553	5.92010309	5.81070429	5.86264313	6.05555289	6.01845465
NONHSAG019176	6.38978263	6.30069529	6.25788955	6.37926984	6.19685992	6.19058501	6.25428879	6.2548809	6.10054509
NONHSAG019180	2.78483438	2.58990661	2.52152866	2.58085273	2.53396923	2.43450504	2.70289262	3.17999505	2.37959269
NONHSAG019199	2.15402524	2.41572265	2.29689303	2.33366453	1.94793431	2.05305606	1.81245825	2.34653481	2.23866526
NONHSAG019275	2.99014602	3.12591959	3.02751481	3.21725742	3.46143731	3.25703786	3.27419198	3.16243728	3.36386027
NONHSAG019322	2.46380306	2.52927001	2.30076478	2.48151492	2.48431352	2.4714345	2.43904531	2.47195231	2.42902395
NONHSAG019335	3.40248095	3.17461342	2.93469341	3.61870099	3.27422889	3.48594365	3.13996437	3.11379116	3.41636622
NONHSAG019355	4.6123365	4.33004958	4.66809315	4.57464232	4.8423707	4.72383828	4.72120058	4.58812452	4.66758925
NONHSAG019359	3.22396132	3.17279407	3.43955697	3.5345726	3.29904801	3.60956259	3.67046635	3.43380755	3.24650525
NONHSAG019389	2.80081608	2.81879053	3.01424913	3.20474286	2.80548173	2.83205164	2.66245396	2.7525026	2.6630666
NONHSAG019395	3.3151389	3.16590605	3.32279061	3.19062379	3.12675947	3.26152011	3.33775174	3.25646239	3.29365594
NONHSAG019396	3.91422418	3.30529566	3.47412475	3.57319982	3.2415967	3.30250003	2.91869867	3.11376406	2.80959805
NONHSAG019423	3.44102598	3.63738639	3.77689822	3.88644042	3.51181652	3.70412258	3.62059471	3.78553161	3.91902783
NONHSAG019434	3.32558192	3.53759688	3.46680223	3.61957919	3.32568047	3.35516339	3.29244408	3.46751132	3.37042286
NONHSAG019460	2.92864283	2.90603248	2.93115308	2.91429506	3.1258873	2.36435738	2.70760641	2.58919264	2.80959805
NONHSAG019464	5.49395121	5.22446857	5.48948201	5.60901036	5.75844345	5.72010844	5.48302414	5.50534523	5.77693606
NONHSAG019471	5.34303425	5.17521382	5.39606873	5.46893429	5.36519313	5.2330396	5.46624252	5.20010194	5.54090514
NONHSAG019477	4.58207371	4.73649836	5.09492531	4.80428584	4.46972407	4.84839058	4.54498917	4.57351217	4.21021182
NONHSAG019496	3.43490047	3.54442637	3.52879445	3.48277682	3.62348066	3.80957567	3.48000946	3.62348066	3.95600106
NONHSAG019516	3.03510335	2.89733549	3.04008832	2.9252318	3.04261479	3.15816815	2.91798931	2.60459183	3.12996002
NONHSAG019517	2.44659301	2.44928313	2.44514364	2.6532238	2.48068153	2.55976252	2.39776247	2.62722838	2.39241179
NONHSAG019583	4.22599356	4.48442814	4.36513245	4.2117691	4.19909074	4.31373297	4.0899288	4.3982197	3.98853784
NONHSAG019638	3.10383572	2.97874106	3.131874	3.17268238	3.3789975	3.31988956	3.34931146	3.18433144	3.21518661
NONHSAG019649	2.31113154	2.23169518	2.16520324	2.32264358	2.14155262	2.18671861	2.11119348	2.30153963	2.30125719
NONHSAG019657	2.51817152	2.61409578	2.54724077	2.49444473	2.52840223	2.49386133	2.58460897	2.56369943	2.51948957
NONHSAG019671	3.54469149	3.45160826	3.69141269	3.84565009	3.60785605	3.74180724	3.79281733	3.81976019	3.7665474
NONHSAG019696	2.61774016	2.61011814	2.62746048	2.55532885	2.65309996	2.7178516	2.606637	2.43921664	2.43964629
NONHSAG019733	5.04744505	4.91528922	5.10991408	4.96266049	5.10636385	5.12282972	5.05031774	5.00495944	5.22130798
NONHSAG019747	4.1077371	3.97897442	4.05521675	4.28647186	4.25817054	4.07748515	3.79433468	4.1399761	4.03814426
NONHSAG019755	3.49398083	3.7257987	3.25481237	3.79776492	3.35591241	3.50223246	3.6017138	3.59345603	3.64482687
NONHSAG019799	2.61393765	3.03480923	2.90569801	2.83427778	3.04610551	2.91515381	2.96706705	3.00210012	3.01743245
NONHSAG019803	4.61576425	4.82308633	4.68078925	4.6434364	4.43306529	4.94856038	4.64028955	4.24971049	5.22474777
NONHSAG019855	8.28990554	8.34488586	8.34978394	8.04664033	8.2795112	8.21835615	8.26897122	8.13402509	8.09772514
NONHSAG019888	4.3281231	4.18377293	4.52364069	4.23724071	4.6668595	4.46410584	4.41306976	4.33754632	4.59886368
NONHSAG019921	3.35145842	3.422761	3.50109543	3.52701429	3.42200499	3.40779664	3.43293793	3.44311926	3.48127301
NONHSAG019946	5.36230101	5.37776949	5.58624748	5.50040479	5.4396293	5.47570442	5.38250483	5.22386654	5.23176011
NONHSAG019954	2.93049058	2.76960147	2.8417443	2.68383999	2.66087576	2.77469786	2.67309853	2.69660298	2.77202479
NONHSAG020059	3.50656859	3.45566727	3.29198745	3.79252397	3.42588861	4.35849796	3.56873687	3.93309409	3.66317539
NONHSAG020065	3.29736562	3.23153409	3.04667606	3.11769767	3.36469089	3.26200598	3.30548791	3.17052011	3.57169812
NONHSAG020081	9.84279515	9.79900948	9.25243573	9.5281231	9.52639703	9.61163393	9.968426	9.81615846	9.89630975
NONHSAG020082	3.42992083	3.09790706	3.43951289	3.24593611	3.31679146	2.9795529	3.21856388	2.99821932	3.28065288
NONHSAG020086	5.4408723	5.38753825	5.31609886	5.51224522	5.25860184	5.6307626	5.76759962	5.06699439	5.56618904
NONHSAG020094	2.28950322	2.17962868	2.13303874	2.18577843	2.3164733	2.06271102	2.571898	2.21242231	2.33774686
NONHSAG020096	4.35295089	3.80805396	4.26409415	4.42665889	4.22922342	4.7440424	4.42665889	3.80993972	4.21246561
NONHSAG020099	8.04319037	7.935953	7.92403835	7.89104596	8.1275858	8.14145138	8.13991868	7.98365556	8.08098327
NONHSAG020107	3.39187803	2.9615475	3.13512024	3.32430313	3.48735038	3.53273689	3.23363492	3.13856472	3.33572791
NONHSAG020130	2.75191968	2.57269992	2.56197472	2.60663031	2.5879507	2.57013058	2.54097465	2.46772464	2.53679344
NONHSAG020182	4.70165525	4.51054666	4.85196708	4.60714816	4.87669899	4.8528699	4.79452301	4.58836162	4.90116332
NONHSAG020190	4.12874319	3.84417108	3.95742574	3.87913897	4.21313316	3.79770652	4.01171344	3.88955719	4.1794416
NONHSAG020197	4.21085023	3.99903132	4.04569568	4.26999375	4.38285287	4.13295935	4.18707528	4.10070018	4.42931703
NONHSAG020211	5.51449549	5.46582043	5.45676852	5.38643395	5.50216865	5.67384543	5.53224352	5.46350203	5.38310128
NONHSAG020225	4.30932645	4.19307017	4.32981809	4.05638407	4.6001023	4.15995306	4.48700716	4.31408678	4.34892692
NONHSAG020230	3.02295498	2.72752575	2.77657067	2.78650594	2.72658634	2.87432119	3.04470334	2.85872826	2.81579991
NONHSAG020240	3.08081999	2.92456328	3.08901176	3.09557959	3.42693357	3.07445004	3.12437112	3.21294777	3.182325
NONHSAG020323	5.22644049	5.06402415	5.14980322	5.15207285	5.16258749	4.81782549	5.00610474	4.97318678	5.16888726
NONHSAG020329	6.53763211	6.44044991	6.35422387	6.55728541	6.57038813	6.51061043	6.57743662	6.65443869	6.31291493
NONHSAG020334	3.84483423	4.02161558	3.97382095	4.46509851	4.34530932	4.40092888	4.47893016	4.30701039	4.46219569
NONHSAG020338	3.91025002	4.36185892	4.17748103	4.37737714	4.02742861	4.2687343	3.80560456	4.0608431	4.18117704
NONHSAG020339	6.80330144	6.88596084	6.77727	7.08981624	6.81349782	6.40288632	6.83847401	6.86504523	6.99809851
NONHSAG020347	7.40464133	7.28795984	7.35039901	7.35861307	7.27696231	7.63636012	7.6060439	7.44475101	7.52812118
NONHSAG020419	3.66504774	3.7053987	3.60033167	3.86215082	3.53445683	3.7730018	3.46018654	3.83644266	3.32189597
NONHSAG020424	3.74122052	3.7514494	4.12457414	4.14344913	4.2302581	4.33236267	3.87178576	3.79121119	3.79720875
NONHSAG020428	3.09746479	3.03072015	3.10816117	2.97033432	3.03534703	3.09337478	2.94573755	2.8443743	3.23034366

NONHSAG020432	6.28541176	6.10346228	6.23581903	6.21185827	6.20624347	6.21019044	6.38528188	6.13567627	6.64008018
NONHSAG020434	3.4418616	3.4907804	3.57538852	3.64303796	3.57320838	3.64793951	3.48040514	3.59094403	3.50814004
NONHSAG020483	3.08501448	3.09918596	3.35471089	3.51384392	3.42222563	3.27140849	2.9458049	2.98565719	3.16032328
NONHSAG020539	5.18821983	4.78173512	4.78337106	5.39379512	5.34066736	5.26321815	5.19946167	5.40093611	5.30164032
NONHSAG020550	5.93727527	6.16590475	5.60997342	6.25545657	5.94538143	6.12475807	5.90250894	6.02930916	5.77172351
NONHSAG020567	3.89759976	4.04834159	4.04844791	4.40830357	4.23095422	4.33431455	3.99053521	3.92418175	4.02077686
NONHSAG020580	4.12404999	4.13815572	4.30771345	4.27996611	4.47958895	4.27940584	4.36903125	4.01832972	4.74918285
NONHSAG020618	4.92522668	4.60765093	4.77592837	5.26763613	4.79690345	5.06648092	4.8460682	5.00097853	4.16341044
NONHSAG020638	6.9784419	7.01351714	6.92016753	7.15757978	7.1368993	7.09557829	6.85905083	7.09264767	6.83951203
NONHSAG020650	2.43077471	2.40737098	2.85068099	2.85184082	2.66756288	2.66756288	2.8953206	2.62722838	2.83709076
NONHSAG020678	5.82359833	6.38972084	5.95591533	5.92522052	6.21842613	6.32433987	6.21338774	6.09115033	6.19256452
NONHSAG020737	3.11661334	2.89029079	3.07568116	3.21919971	3.22827373	3.48594365	3.20756269	3.26511899	3.51581552
NONHSAG020798	2.32230169	2.25639634	2.24035532	2.42853437	2.55836629	2.28125121	2.62223917	2.42558017	2.42195105
NONHSAG020806	4.26832826	4.25970648	4.24817864	4.35001171	4.22047934	4.36872597	4.37714081	4.13881822	4.26783249
NONHSAG020809	3.24855077	3.00157392	2.93292887	3.15087452	3.11374269	3.07063707	3.23820698	3.09146506	3.60254134
NONHSAG020830	3.95600752	4.1403467	4.05765564	4.14915369	4.02822397	4.37599312	4.22580038	4.1988153	4.1114905
NONHSAG020837	2.48978139	2.4429131	2.67916931	2.58255946	2.56671684	2.77755794	2.57581081	2.45413998	2.53939126
NONHSAG020855	3.17461342	3.03540437	3.18995238	3.51570325	3.10544963	3.38908663	3.09576479	3.0665718	3.24663534
NONHSAG020867	2.16817762	2.10449677	2.10651863	2.12624938	2.13595827	2.0900715	2.11888333	2.16183309	2.10931245
NONHSAG020883	3.67163109	4.04173894	4.02497271	4.34058737	3.91019016	3.91749842	4.23571123	4.03551161	4.11059215
NONHSAG020899	2.71329837	2.50513475	2.68624756	2.78125094	2.70529177	2.48104604	2.60067483	2.69243007	3.01511986
NONHSAG020905	3.36942198	3.33877983	3.32016828	3.26985992	3.4144259	3.24032478	3.1802818	3.39178285	3.26251722
NONHSAG020941	6.50763668	6.37190068	6.43680854	6.54578609	6.36569707	6.78502182	6.6064242	6.53300132	6.78917219
NONHSAG020993	5.07689603	5.02403336	5.09620771	5.17741361	5.1967295	5.11903043	5.26825642	5.2064992	5.46875828
NONHSAG021003	5.14036602	4.94426321	5.35341794	5.24244005	5.41389741	5.12394902	5.4627412	5.27243206	5.41199133
NONHSAG021015	5.51520055	5.66820673	5.81343151	5.57243752	5.46473454	5.60209572	5.5453731	5.27613805	5.80564991
NONHSAG021045	4.15673122	3.7409522	4.20912144	4.02736867	4.19822937	3.91866567	3.81900737	3.82756233	3.84617224
NONHSAG021143	4.23008789	4.18908716	4.07722629	4.22687942	3.98212884	4.16056855	4.32504481	3.87521319	3.98079452
NONHSAG021154	4.27349253	5.07114383	4.79197614	5.19226775	4.6107392	5.02690429	4.46244454	5.20773864	5.00641623
NONHSAG021213	4.60656497	4.9047375	4.51220864	4.73320695	4.65827908	4.50993953	4.37173131	4.77263353	4.39204566
NONHSAG021241	2.94698221	2.83238167	2.91249806	3.09131578	2.83238167	2.94374802	2.96673985	2.60686313	2.7249921
NONHSAG021246	5.98945353	5.92026672	5.68840975	5.87448187	5.56453742	5.9645553	5.75114388	5.89450825	5.50760594
NONHSAG021274	3.84568222	3.76714786	3.85952317	3.77152967	3.93481106	3.84406401	3.8751917	3.99381388	4.501756
NONHSAG021287	5.57799477	5.45382002	5.60908121	5.58091097	5.87022875	5.66848532	5.79989	5.69016546	5.67732642
NONHSAG021374	2.75771875	2.51989489	2.53890957	2.62137225	2.72752575	2.72257374	2.79552346	2.57927535	2.61093955
NONHSAG021403	5.46955329	5.22990808	5.42619705	5.07953513	5.50840841	5.40299574	5.25518517	5.01338302	5.83955646
NONHSAG021436	4.31998077	4.25149689	4.15179825	3.8594205	4.20413751	4.16329561	3.97737786	4.18947312	4.42795579
NONHSAG021444	5.66250637	5.06033726	5.67996511	5.51889653	5.98667811	5.54724595	6.06567703	5.58583594	5.80564991
NONHSAG021459	2.21711116	2.2499249	2.17980898	2.59127087	2.8153537	2.30303942	2.67442994	2.17116804	2.15053545
NONHSAG021464	3.37433301	3.30921902	3.21887803	3.33267925	3.00761396	3.03245117	3.57570703	3.36485718	3.55538243
NONHSAG021479	4.87549332	4.682104	4.74553468	4.60157721	4.57837891	5.21574721	5.25943323	4.70074276	4.86718535
NONHSAG021502	3.367195	3.17089699	3.57192383	3.19795546	3.22297934	3.35544992	3.01883065	3.14842919	3.44728461
NONHSAG021504	2.93413842	2.86699862	3.18164224	2.95830333	3.40321645	2.82560399	3.06691017	3.18179215	3.16426575
NONHSAG021505	3.74927394	3.5142928	3.61204169	4.01161194	3.50176041	3.72009772	3.79362898	3.71958988	3.91894026
NONHSAG021537	3.57161564	3.41022742	3.50806692	3.38275965	3.76160004	3.46898753	3.58094465	3.70993555	3.58846992
NONHSAG021546	4.03872545	4.04417036	4.09410557	4.0524446	4.06035918	3.75891075	3.99314022	3.95677763	4.02971764
NONHSAG021557	6.36888642	6.63591232	6.30175887	6.02841237	6.05705297	6.28602925	6.19859829	6.41242159	5.69879756
NONHSAG021577	4.39081122	4.83055524	4.58438889	4.17057023	4.60553045	4.7180064	4.58696932	5.0186972	5.0906919
NONHSAG021582	4.76975915	5.05006723	4.96310363	5.16988663	4.89925822	5.091516	4.94535945	4.74291572	5.14366688
NONHSAG021608	2.8273882	2.68695115	2.60824534	3.20016908	2.57298392	2.91660081	2.9931536	2.81780862	2.67979858
NONHSAG021662	5.02322284	4.89421659	5.14673281	5.04156209	4.98245268	4.62019333	4.81720244	4.65778086	5.01817768
NONHSAG021671	2.76526035	3.10387577	2.98614576	3.10196442	3.12732516	2.94692515	2.7401578	2.93970606	2.89248214
NONHSAG021696	3.8159383	3.71138674	3.91253607	3.83942457	3.75775389	3.9921244	3.8012747	3.78594213	4.10858916
NONHSAG021701	4.52958342	4.67998473	4.59916182	4.61922439	4.59234427	4.65914041	4.58574537	4.8869152	4.56592075
NONHSAG021755	6.21664376	6.42265528	6.29153451	6.30802568	6.12048535	6.378247	6.30957143	6.32247432	6.26501821
NONHSAG021758	3.62743012	4.04417036	3.47153843	3.50530465	3.71083165	3.57847817	3.59771208	3.78614521	3.6181697
NONHSAG021801	6.03809248	5.67509588	5.95954199	5.92326929	5.89006858	6.0667439	5.98426946	5.97612437	5.72943444
NONHSAG021825	4.16163243	3.95849623	4.18572968	4.0717596	4.27184061	4.39467003	4.27814456	4.31803441	4.25357898
NONHSAG021851	4.11197697	4.31837018	4.06584419	4.25042414	4.23377869	4.36894604	4.4080267	3.89039595	4.07960254
NONHSAG021881	3.8759627	4.1552642	4.38690248	4.3901486	4.14972123	4.50355263	4.14316194	3.94045217	3.80067191
NONHSAG021904	4.3672163	4.39071522	4.49174895	4.59193586	4.37534004	4.5280379	4.29427155	4.31918504	4.57627394
NONHSAG021905	5.84647158	5.82636433	5.78088945	5.85400874	5.85504228	6.04867159	5.9532792	5.99060154	6.2253241
NONHSAG021933	2.2461145	2.10603466	2.1497832	2.07849375	2.15175159	2.12661224	2.22377175	2.15632628	2.21431327
NONHSAG022026	3.3171102	2.9611321	3.16494898	3.2269686	3.15314122	3.35184016	3.52318495	2.9638047	3.23034366
NONHSAG022055	4.10647856	3.96668824	3.99055801	4.1581494	4.23205042	4.25826523	4.00746393	4.03579947	4.09063959
NONHSAG022065	5.26690639	4.9905068	5.35915293	5.33961893	5.29497836	5.47992644	5.39792458	5.23044564	5.36637372
NONHSAG022068	4.66956977	4.77782706	4.6645728	4.6366602	4.9413272	4.83934555	4.70216083	4.77913418	4.56261662
NONHSAG022073	2.54554922	2.57922492	2.51478514	2.8230756	2.74667008	2.94491019	2.46974264	2.80023426	2.60532012
NONHSAG022088	3.81098622	3.60518374	3.66482758	3.94088216	3.45129272	3.57665891	3.35582962	3.76597666	4.12127707
NONHSAG022089	2.8790431	2.87541029	2.92052517	2.87113618	3.02226984	3.07305572	3.28361998	2.74771006	3.00383317
NONHSAG022092	4.8116467	4.68388742	4.75032123	4.55068369	4.91866025	4.71975177	4.64028955	4.50250285	4.94784191

NONHSAG022177	4.94788212	5.03631163	4.9876243	5.06422106	5.25827452	5.20041787	5.07927749	5.07384924	5.41569477
NONHSAG022214	4.36544183	4.22595707	4.55439625	4.37855078	4.46452278	4.46284528	4.32587052	4.38185557	4.29625593
NONHSAG022224	4.29357845	4.29659945	4.18056004	4.15040739	4.39710174	4.48304617	4.00121612	4.09479033	4.25576887
NONHSAG022252	6.79127629	6.87204309	6.80267147	6.83341296	6.76150282	6.84151915	6.89629606	6.81515454	6.78432319
NONHSAG022256	3.65530351	3.67627955	3.66365439	3.68892355	3.86856894	3.64675076	3.60751813	3.96646913	3.76054395
NONHSAG022261	3.656681	3.06152869	3.23995083	3.20688886	3.14232676	3.55672202	3.42021203	3.3271618	3.51723455
NONHSAG022297	4.35872275	4.33787922	4.4127848	4.34230148	4.55702997	4.39477233	4.40871059	4.26297553	4.31829368
NONHSAG022308	4.87219904	4.94550841	4.8598703	5.10716162	5.24429671	4.93801899	4.95906916	4.59650616	5.0804359
NONHSAG022311	5.4395296	5.66996064	5.47060409	5.56018397	5.45654301	5.41255671	5.43559254	5.40736907	5.31839458
NONHSAG022328	4.70727913	4.80951811	4.91040986	4.84730027	4.84638989	4.53514518	4.79924145	4.65626738	4.93651326
NONHSAG022337	5.08050522	4.95442813	5.18248443	5.36296327	5.21176927	5.14057573	5.43655118	5.09766533	5.17601956
NONHSAG022342	2.56301254	2.50689711	2.34293343	2.5041088	2.5758998	2.54566215	2.56758481	2.40546276	2.49941474
NONHSAG022353	3.40328461	3.37791735	3.41024658	3.70274129	3.57214143	3.52719041	3.13982658	3.41946347	3.42689165
NONHSAG022357	7.67068909	7.90616677	7.61817947	7.65528566	7.4069519	7.72338396	7.66041923	7.76980864	7.29683095
NONHSAG022358	6.85955616	6.85206851	6.92965996	6.53800703	6.83416513	6.98144249	6.83588041	6.70312688	7.0851062
NONHSAG022437	5.55729912	5.20913383	5.6486485	5.65746933	5.53931206	5.73066894	5.63714085	5.73132541	5.7333501
NONHSAG022492	7.86171049	8.00716446	7.64419346	7.86235962	7.63918688	7.90225086	8.06447611	7.96361096	8.15532684
NONHSAG022576	3.47219972	3.52405689	3.38040753	3.68477523	3.39465061	3.63877681	3.63115647	3.28651486	3.48051533
NONHSAG022580	5.04173974	5.21237961	5.26829555	4.99416846	4.63419352	5.03719462	4.74013666	5.06598107	5.00318269
NONHSAG022588	7.6785379	7.7179084	7.6665785	7.88714198	7.74923856	7.61760908	7.67246045	7.62571261	7.59501912
NONHSAG022627	3.66250132	4.04344957	3.97393113	3.90270932	3.86766513	4.08289346	4.29885101	3.72760666	3.88877908
NONHSAG022655	3.25029865	3.22453105	3.18871523	3.60693285	3.35435648	3.63838823	3.38134298	3.88256953	3.59795712
NONHSAG022656	3.80365398	4.39481494	3.94665118	3.65276188	3.89286577	4.00161505	4.00553066	4.05895258	3.9605325
NONHSAG022668	4.29325748	4.33053405	4.43728781	4.45221639	4.34432123	4.61996508	4.34467187	4.27404569	4.22727146
NONHSAG022676	3.77427239	3.88409551	3.95929286	3.92411333	3.91019016	3.91155192	3.86792268	3.90413449	3.79494449
NONHSAG022677	3.70569505	3.6801098	3.92658596	3.98164156	3.74495648	3.66632739	3.61174086	3.87036368	4.06965887
NONHSAG022735	5.85707709	6.28109125	5.71427972	5.82726695	5.76835175	5.79589253	5.83039798	5.83076525	5.62218845
NONHSAG022759	8.59208885	8.65151728	8.34961525	8.55152661	8.5047388	8.40510131	8.5213591	8.51293998	8.44827344
NONHSAG022817	4.31013497	4.41874732	4.46757357	4.11056728	4.68310111	4.4994425	4.22259776	4.45819345	4.43804373
NONHSAG022830	5.38868896	5.26695901	5.30480856	5.28342773	5.18413176	5.38663837	4.89507525	5.05234552	5.11175112
NONHSAG022841	5.5339891	5.53479321	5.59677589	5.42164499	5.51784239	5.44932418	5.48729831	5.50727396	5.87267474
NONHSAG022852	3.51410095	3.2769186	3.30660725	3.3983842	3.58280823	3.45961806	3.32854543	3.25260226	3.69139161
NONHSAG022858	3.03447948	3.03282035	3.21073534	3.28458464	3.21408776	3.17992858	2.92102501	3.20067247	3.03637116
NONHSAG022867	5.31536882	5.2464907	5.10746616	5.35663794	5.35679368	5.52669182	5.30523609	5.18480656	5.36105049
NONHSAG022906	3.64351448	3.5486178	3.5255374	3.9249351	3.59613561	3.52847316	3.49498442	3.49150118	3.52048501
NONHSAG022924	6.12290971	6.09758761	6.11366174	6.0919002	6.37895368	6.02179461	5.9655579	5.9315377	6.07459287
NONHSAG022925	5.28428999	5.48301751	5.21109482	5.0849465	4.89863918	5.35965593	5.56665897	5.18315637	5.0566965
NONHSAG022957	4.60027075	4.17513512	4.39627753	4.3007599	4.24641723	4.46275664	4.1943852	4.42626724	4.32246214
NONHSAG022968	5.58369084	5.52561304	5.76731568	5.86452282	5.65572751	5.38467185	5.58598352	5.62049835	5.57509236
NONHSAG022969	3.28541892	3.13360772	3.62004707	3.45614562	3.18986399	3.71699984	3.56753596	3.2712623	3.22996616
NONHSAG022994	5.78729836	5.86153844	5.69727481	6.02082768	5.91911483	5.94897216	5.83319394	5.60046387	5.65629857
NONHSAG023009	2.6781207	2.73232122	2.77061911	2.65595711	2.68476478	2.78885366	2.91011528	3.09753533	2.77672999
NONHSAG023036	6.08522801	6.55710067	6.21855528	6.24769624	6.35978264	6.1170028	6.0969913	5.92075706	5.82135298
NONHSAG023052	4.68304109	4.6379484	4.66306589	4.68001674	4.65957273	4.78434533	4.71768122	4.76100641	4.62424932
NONHSAG023067	5.73511052	5.69634908	5.46044933	5.74507246	5.46761697	5.41621001	5.44358506	5.6068925	5.58297692
NONHSAG023085	2.64799832	2.85492252	2.9932679	2.87428277	2.82691326	2.7474233	2.70760641	2.9573645	3.04547808
NONHSAG023088	5.83897873	5.60128684	5.66653526	5.99682815	5.97280608	6.06726285	5.88953465	6.14877259	5.83059989
NONHSAG023107	3.52780961	3.50231543	3.59006643	3.43654133	3.86107858	3.37298605	3.53303693	3.80881111	3.73618031
NONHSAG023114	3.9081851	3.76343358	3.70872428	3.79564542	3.74980112	3.86631395	3.64392561	3.49883177	3.490129
NONHSAG023118	3.85630934	3.87131154	3.75995434	3.91853951	3.77250024	3.91312961	3.94672739	3.91068029	4.34093015
NONHSAG023129	6.354769	6.63006541	6.12501619	6.13411458	6.41576391	5.83238761	6.12727358	5.99846166	6.17459255
NONHSAG023173	4.45613212	4.81019344	4.80350479	4.9524345	4.81859313	4.84562813	5.01838481	4.95177354	5.16650712
NONHSAG023180	3.04697559	3.04858266	3.21503167	3.37290526	3.4177479	3.19788941	3.39052491	2.94450669	3.2709205
NONHSAG023185	3.19914875	3.31526518	3.19334772	3.59005008	3.31439253	3.38290844	3.33282823	3.1750979	3.19734849
NONHSAG023194	2.81580867	2.6509622	3.08866773	2.88529759	2.61500386	3.12813029	3.0770918	3.10339299	3.04508612
NONHSAG023237	4.16433079	3.90641219	4.03935023	4.19202249	4.07488558	4.05370485	4.50743946	4.0106264	3.84945237
NONHSAG023238	3.16247075	3.33347184	3.14422233	3.15565693	3.11381449	3.25237421	3.33354586	3.16744797	3.36682801
NONHSAG023245	2.36456823	2.54659962	2.56970507	2.56091458	2.59541766	2.72421442	2.46133891	2.46510476	2.53629176
NONHSAG023247	2.33110494	2.22440783	2.15451263	2.11667009	2.38749879	2.16857836	2.26218252	2.32352958	2.24468699
NONHSAG023281	2.52484738	2.49584513	2.4881262	2.64737333	2.62977123	2.62977123	2.40813376	2.67710648	2.69124891
NONHSAG023286	3.17029505	3.1873681	3.6284468	3.2564574	3.17510347	3.01074289	2.65724219	3.44422802	2.93297105
NONHSAG023298	3.31161554	3.24798191	3.41834896	3.22552966	3.45498567	3.48594365	3.48992868	3.52341713	3.64466795
NONHSAG023327	4.07102951	4.23342739	4.3354236	4.53398727	4.0630533	4.02237161	4.15673122	4.24859077	3.93195055
NONHSAG023350	3.37828774	3.45400513	3.28140339	3.30748608	3.33881381	3.35848648	3.48072382	3.41374601	3.36235875
NONHSAG023373	3.86125615	3.87131154	3.89581778	3.82669866	4.11571189	3.8779741	3.93117561	3.73037801	4.05916832
NONHSAG023390	5.41665041	5.30167381	5.34550817	5.38772876	5.59103423	5.23755569	5.51593652	5.31723463	4.90462361
NONHSAG023427	2.80023245	3.00503902	2.98275005	2.84601253	3.28922183	2.67479887	3.04942001	2.73789135	2.83408264
NONHSAG023436	6.2225327	6.16329279	6.25989031	6.33373908	6.51451111	6.23973718	6.40851865	6.18202844	6.4431059
NONHSAG023437	3.67702534	3.39832199	3.64363562	3.69662321	3.72190006	3.77918991	3.67007777	3.57077805	4.14557141
NONHSAG023456	2.7885241	2.84227465	2.9341664	2.84653499	3.05919672	2.9253172	2.87056739	3.22058197	3.16925338
NONHSAG023530	2.17196242	2.44217815	2.31900415	2.46540516	2.32655293	2.30979906	2.29861482	2.34422827	2.24699573

NONHSAG023534	2.90025576	2.78978835	2.72962126	2.75833156	2.5758998	2.68607639	2.83725751	2.53456667	2.83210187
NONHSAG023539	5.75752418	5.90481121	5.82214387	5.8725978	5.87164408	5.974674	5.88852784	5.87036534	5.97022932
NONHSAG023582	3.41172564	3.18143534	3.3864936	3.28990698	3.51239915	3.17533238	3.33596201	3.16227216	3.39789643
NONHSAG023627	3.23338422	3.13367339	3.2459104	3.39441885	3.22987935	3.37240327	3.12047377	3.61839715	3.32403432
NONHSAG023639	2.38938267	2.30359831	2.52428978	2.40370369	2.71178286	2.43945296	2.48699755	2.44108101	2.67482492
NONHSAG023666	2.76448552	2.46882405	2.70643976	2.83707151	2.77418245	2.79624095	2.80684074	2.45230697	2.80959805
NONHSAG023691	2.7756391	2.76172463	2.82226475	3.0620134	2.93767499	3.39892822	2.88214043	2.86839543	2.97332346
NONHSAG023704	4.01793067	4.55819314	4.1102405	4.28901824	3.99738871	4.46405911	4.12180107	4.3294884	4.58951475
NONHSAG023707	2.55419881	2.45443167	2.55317052	2.66267403	2.60658677	2.66267403	2.64358075	2.57704367	2.5081571
NONHSAG023711	3.07416257	3.03695565	2.7152926	2.96588087	3.1388632	3.13079752	2.8818836	3.16017777	3.0607755
NONHSAG023714	3.9402296	3.8980672	4.02399452	4.09920166	3.89420552	4.09990709	3.86551509	3.80037578	3.78432845
NONHSAG023715	2.3619195	2.49744401	2.43670353	2.48454722	2.55151548	2.4653595	2.5159692	2.4653595	2.30234292
NONHSAG023765	3.02212477	3.15853209	3.13071925	3.07420036	3.20034828	2.78739491	3.22258296	2.87238423	3.36576559
NONHSAG023770	2.94632739	2.80802589	2.74318517	3.08056464	3.18847136	2.93252149	2.70792559	2.88418076	2.9610762
NONHSAG023771	3.12170276	2.98508042	2.61501003	2.88552418	2.84917963	2.8937484	2.58385181	2.85352593	2.8154622
NONHSAG023772	2.34520729	2.31698135	2.43508382	2.38954096	2.49776601	2.46467554	2.30903228	2.41889481	2.18172381
NONHSAG023787	5.03017132	4.83648019	5.00681612	5.0677264	5.14147826	4.98832175	4.9499675	4.98661215	5.15486912
NONHSAG023790	2.48093436	2.73599934	2.76154971	2.76590068	2.70455313	2.56424435	2.80618932	2.68601545	2.84577259
NONHSAG023801	4.81347517	4.75893263	4.7201379	4.82427519	4.86851308	4.93382116	4.79649715	4.78191094	4.82365848
NONHSAG023810	3.56639408	3.37366281	3.35859926	3.20848377	3.55171582	4.13070679	3.39052491	3.55837656	3.18256836
NONHSAG023839	4.6198843	4.55021972	4.91063973	4.88477296	5.07326737	5.00086135	5.25507107	5.00620392	5.08038214
NONHSAG023915	3.11512452	3.03391306	3.08329328	3.23964304	3.22743536	3.32518518	3.01112929	3.03678493	3.1970188
NONHSAG023920	4.47696541	4.6398888	4.64271774	4.51965721	4.74968879	4.77571737	4.58990823	4.49250473	4.92699403
NONHSAG023923	3.88965823	3.51968624	3.37119222	3.92489251	4.02799005	3.46874729	4.29721697	3.55837656	4.13441689
NONHSAG023954	5.4348303	5.09302066	5.45460918	5.57137635	5.66033117	5.21382669	5.64004319	5.27476241	5.47117773
NONHSAG023966	5.32532066	5.26191721	5.33755349	5.30722841	5.37790431	5.43180079	5.19596635	5.45731136	5.29427693
NONHSAG023972	3.9752136	3.87356021	4.32233367	4.61786138	4.15362061	4.11261058	4.10511493	4.18507886	4.1618006
NONHSAG024013	3.40228717	2.92024373	3.13067454	3.31166755	3.50773263	2.88912553	3.37729085	3.12547716	3.58248506
NONHSAG024040	4.99487458	4.83910799	5.1259992	5.17155132	5.05140082	4.99893373	4.64022515	4.62404935	5.04964323
NONHSAG024043	2.91835098	2.87527675	2.77214409	2.82600736	3.00193668	3.14179354	2.96375643	3.18972731	3.10990647
NONHSAG024046	5.03322685	5.4085035	5.00023755	5.36665709	5.1311528	5.5268224	5.14517075	4.88672573	4.8441647
NONHSAG024080	2.80473532	3.12097619	2.66296679	2.67519579	2.80590444	2.66441995	2.39071273	2.74539557	2.4191437
NONHSAG024104	2.25402432	2.35171348	2.36583295	2.63318428	2.66156518	2.19747497	2.30201166	2.59174341	2.34796971
NONHSAG024105	3.34544374	3.47859388	3.33572874	3.2843323	3.38470377	3.33572874	3.39052491	3.18470817	3.37663284
NONHSAG024135	4.39077142	4.3404805	4.44757896	4.53855472	4.4615212	4.48066362	4.3502888	4.56970665	4.99481629
NONHSAG024143	2.57699838	2.77752132	2.77987405	2.82661478	2.46373896	2.63896058	2.62291863	2.62722838	2.77126405
NONHSAG024146	2.81649553	2.76154335	2.9564871	3.0054099	2.87196136	3.04313383	2.75317928	2.70769109	2.93179494
NONHSAG024157	3.57130852	3.48630555	3.49231216	3.73728471	3.65739409	3.57232115	3.69113284	3.4708427	3.64325037
NONHSAG024162	2.79844964	2.70336044	2.61452269	2.78460018	3.01214643	2.86392395	2.85249564	2.63410074	2.6076682
NONHSAG024182	5.09445686	5.13242618	5.22280682	5.19204284	5.11061834	5.40285784	5.1221529	5.01903402	5.17509767
NONHSAG024209	5.5107921	5.80536462	5.83511762	5.73817399	5.46570888	5.93078589	5.7365789	5.60584402	5.50031132
NONHSAG024232	4.25841523	4.56762476	4.37200301	4.42397438	4.54374018	4.3526186	4.32674997	4.45599261	4.4754438
NONHSAG024234	3.47003267	3.76487282	3.52317257	3.58575159	3.25980998	3.45861257	3.36666291	3.77314985	3.44697662
NONHSAG024244	4.21525646	4.21432898	4.26268776	4.40456335	4.5052663	4.55021668	4.32895147	4.42073236	4.35892948
NONHSAG024248	6.23524382	6.27219145	6.12143306	6.40639125	6.19810717	6.23917885	6.22911237	6.21854281	6.1229349
NONHSAG024264	3.97022323	3.84193944	4.0690457	3.8894174	3.94976959	3.9483414	4.2124075	3.86003651	3.96605276
NONHSAG024327	2.99014602	3.18223198	3.67807091	2.97880354	3.28374929	3.1826568	3.18797039	3.4694604	3.69543198
NONHSAG024381	5.49576622	5.47290144	5.56189927	5.67606605	5.35873688	5.65629736	5.42720893	5.45588568	5.65757274
NONHSAG024388	5.41147507	5.3297059	5.43617442	5.48900089	5.32715634	5.25274233	5.2396011	5.34686992	5.5564362
NONHSAG024397	6.23625555	6.37279487	6.19123268	6.24176898	6.30423451	6.30525688	6.23933894	6.28566659	6.00151365
NONHSAG024439	6.03015166	5.94820093	5.8159883	6.04489835	6.13011084	6.0356826	5.95933374	6.11919882	5.94588316
NONHSAG024442	3.70427033	3.50887619	3.50717858	3.80980795	3.49095693	3.70748861	3.92995398	4.00761532	3.70849313
NONHSAG024446	3.66182192	3.47416715	3.69480873	3.35202648	3.59153736	3.58508292	3.46875392	3.54274708	3.60555644
NONHSAG024447	2.99014602	2.84627035	2.95203532	2.79431069	2.72752575	2.73307833	2.5216836	2.87491953	2.89020909
NONHSAG024529	7.88508565	7.62115941	7.75376862	7.28556501	7.9135204	7.63226884	7.7256538	7.42676063	8.04293894
NONHSAG024540	3.96665816	3.97690164	3.69145005	4.08160442	3.56520927	3.5766953	3.85275177	3.57237113	3.73931463
NONHSAG024542	5.08932872	5.10429759	5.27258662	5.16706426	4.97240965	5.15779059	5.14640816	5.14365871	5.03598449
NONHSAG024613	4.15606645	4.46532422	4.27982773	4.10240305	4.64789925	4.29825572	4.63236166	4.25568532	4.33983245
NONHSAG024628	2.74338592	2.59881261	2.72060361	2.56900417	2.67344914	2.55402563	2.39814695	2.56747837	2.57368954
NONHSAG024729	3.47126715	3.58152123	3.45736617	3.64039972	3.32961163	3.42484994	3.80364618	3.39651349	3.63562589
NONHSAG024733	2.25693773	2.29310032	2.15576274	2.36848551	2.23760689	2.29459903	2.35132012	2.14944121	2.28202216
NONHSAG024740	2.8263571	2.7907752	2.86537349	2.91560538	3.02659744	2.80791452	2.66804917	2.81443827	2.86079538
NONHSAG024741	3.40459494	3.32200794	3.44852135	3.47057088	3.45972603	3.38877073	3.40298384	3.42419001	3.43111138
NONHSAG024742	9.10141768	9.23823447	9.16263409	9.04076527	9.2209438	8.94565469	9.04486591	8.95830171	8.96337928
NONHSAG024745	3.51618813	2.88491058	3.12777629	2.8230756	3.24758256	2.8620296	3.21323635	3.07276411	3.16482323
NONHSAG024752	4.42203409	4.58472467	4.3942598	4.44674234	4.45914678	4.13713987	4.41414921	4.41392519	4.36628741
NONHSAG024859	2.58955817	2.57296615	2.54853975	2.72953853	2.80637743	2.70248862	2.49673599	2.72537477	2.66708815
NONHSAG024870	2.79345134	3.13796709	2.77192277	2.92086297	2.59153228	2.93701869	2.6432817	2.51793228	2.79373069
NONHSAG024871	6.28367143	6.16853155	6.24539757	6.06882436	6.46738386	6.25252577	6.46763754	6.33930109	6.49427856
NONHSAG024916	4.53816889	4.36954419	4.62588128	4.60457165	4.75470927	4.46578452	4.60573264	4.34592904	4.45388434
NONHSAG024953	3.82981034	3.69326482	3.85928108	4.04773137	3.98331879	4.00921855	3.65949092	3.52886677	3.68646594

NONHSAG024968	3.87602505	3.7453943	3.74129555	3.941712	3.69787195	3.6936352	3.77011406	3.47712207	3.80365559
NONHSAG024985	7.02138055	7.41833372	6.90501633	6.92579274	6.9514319	7.01928422	6.88222267	6.75150627	6.87276262
NONHSAG024988	5.7762204	5.74907543	5.73905109	5.93767018	6.05541223	5.68090283	5.81660785	5.67807129	5.93335543
NONHSAG024994	5.36386016	5.27227763	4.91788349	5.04349601	5.26684599	5.28621696	5.16248622	5.4001357	4.99825701
NONHSAG025009	5.47361273	5.33343461	5.50937865	5.5192353	5.41133624	5.4893025	5.5108062	5.32142585	5.86792008
NONHSAG025049	5.17846342	5.42172787	5.27676131	5.71481627	5.17268671	5.5102918	5.65524984	5.6766464	5.42900288
NONHSAG025052	7.21075258	7.25620877	6.93074344	7.04915163	7.03863491	7.24476514	7.20550758	7.27885091	7.04518536
NONHSAG025054	6.76925258	6.92970618	6.51072384	6.85124861	6.78529573	7.07607093	6.92088241	7.12481594	7.01995147
NONHSAG025151	5.49213207	5.62046005	5.46087598	5.58515309	5.87465401	5.51491913	5.0992417	5.29387527	5.76758518
NONHSAG025174	4.10156721	3.44354594	4.00320308	4.20200851	3.87029304	4.0293599	4.1461015	4.32516893	4.10990787
NONHSAG025195	6.8670083	6.89948976	6.84649244	6.54446781	6.52455044	6.75270191	6.72017412	6.98173523	6.56390616
NONHSAG025224	4.3475194	4.28074686	4.58066919	4.47709706	4.26670077	4.76569992	4.40431932	3.79215362	3.52842923
NONHSAG025230	2.93077546	2.72179285	3.0073274	2.80057158	2.91982472	3.14127151	2.7930969	2.81190582	2.79531692
NONHSAG025265	2.81180259	2.74839282	3.00641933	2.72624693	2.85208943	2.63044678	2.87584011	2.61676039	2.87873266
NONHSAG025272	4.95882127	4.73337188	4.98951467	4.87841999	5.24843125	4.85204029	4.9967147	5.01937037	5.16554396
NONHSAG025274	2.71227395	2.76603643	2.74340477	2.60771141	2.89485173	2.93683446	2.77092913	2.50587857	2.65935961
NONHSAG025288	2.46380306	2.85897101	2.98502288	2.54730178	3.10493156	3.03491486	3.22069982	2.54206924	2.77026498
NONHSAG025295	4.14113403	4.18040944	4.21371416	4.23149276	4.42435804	4.2779561	4.26968704	4.01654967	4.27238252
NONHSAG025343	3.77171422	3.82192512	3.79798214	3.79370728	3.66826959	3.98235869	3.76467043	3.91009522	3.94649521
NONHSAG025349	4.28961543	4.4011791	4.14841148	4.31960961	4.2370789	4.32326784	4.44716932	4.51811919	4.32485711
NONHSAG025352	3.47284981	3.31051431	3.50705831	3.15618581	3.74403679	3.69852224	3.68967635	3.36746929	3.74478261
NONHSAG025367	4.73536659	4.93082543	4.84796183	4.59095789	4.43013146	4.87990693	4.69512757	4.44963347	4.6822399
NONHSAG025376	5.64586832	5.3454744	5.43222528	5.68705729	5.6072402	5.81854559	6.10176558	5.50537492	5.3100707
NONHSAG025378	5.3456848	5.46376737	5.27491543	5.16299519	5.32024084	5.3093007	5.35129006	5.30955284	5.16725981
NONHSAG025393	4.59306736	4.31655316	4.65696741	4.52275911	4.81684535	4.50138452	4.71843933	4.57993744	4.82102231
NONHSAG025422	3.24422717	3.04099418	3.10573544	2.63294074	3.05178401	3.21911523	3.17934876	3.12699993	3.04730346
NONHSAG025431	4.66584746	4.69049548	4.92471658	5.08336471	5.25256854	4.75283351	4.69889541	4.80153012	4.86219831
NONHSAG025438	3.05318658	2.84729488	2.85568798	2.84252348	2.90476794	2.75309065	2.96213896	3.07294086	2.88969608
NONHSAG025448	4.82598288	4.88689561	5.00466658	4.97490871	5.08244314	4.82602165	5.07282552	4.66861844	5.20167902
NONHSAG025466	4.55047801	4.50980353	4.41401584	4.3604675	4.4131854	4.71756458	4.51900874	4.80853324	4.75679006
NONHSAG025491	4.90357067	4.82597853	4.69567472	4.88273285	4.93170112	5.16271897	5.03889308	5.05049451	5.03865003
NONHSAG025494	3.853284	3.75648234	3.91142549	3.85257426	3.72436117	3.86322384	3.96198618	3.90317971	4.03665207
NONHSAG025500	4.99617806	5.00424433	4.92078564	5.07895932	4.97079869	5.07521055	5.15188303	4.75837963	5.05335453
NONHSAG025531	4.14169847	4.12119299	4.08499448	4.34393484	4.2612051	4.35359429	4.29567016	4.2448393	4.19163616
NONHSAG025536	3.44285804	3.63043569	3.62494753	3.18972731	3.32952341	3.65365606	3.40773758	3.27403444	3.21217843
NONHSAG025542	3.83925261	3.7492438	3.94693841	3.9334516	4.26661298	3.9114083	4.16649304	3.93228273	4.33951798
NONHSAG025649	3.9174991	4.15739681	4.66721902	3.94727507	3.44747606	4.18183282	4.3312854	3.88571142	4.44881885
NONHSAG025666	4.57925867	4.63329125	4.68406642	4.68282903	4.5462462	4.72977482	4.48120961	4.48079451	4.72144463
NONHSAG025675	3.59020038	3.5958589	3.6064276	3.53572554	3.70163066	3.64213111	3.53283025	3.52286933	3.50675835
NONHSAG025695	4.32298419	4.24371664	4.24516244	3.95154954	4.1466568	4.23273415	4.15344337	4.34378952	4.01038706
NONHSAG025699	2.51522107	2.40333931	2.3268637	2.30164133	2.41438137	2.22918934	2.34474408	2.54923659	2.41758255
NONHSAG025705	4.45100829	4.22692002	4.37757643	4.26560311	4.12101366	4.26809688	4.18372171	4.46023675	4.11400188
NONHSAG025710	2.82487402	2.40048443	2.58037532	2.7320522	2.66349334	2.67387149	2.89725412	2.53456667	2.720645
NONHSAG025719	4.19009112	4.1259397	4.44636982	4.41788807	4.20489417	4.43135752	4.21087259	4.34821852	4.40708181
NONHSAG025790	5.35972343	5.00006547	5.2781595	5.07768844	5.17476879	5.02088763	5.26853755	5.32100768	5.14741429
NONHSAG025812	2.47954294	2.46617664	2.62512285	2.60949118	2.61117129	2.63911375	2.47238293	2.72318162	2.46200805
NONHSAG025869	3.88627483	3.93265696	4.10599913	4.11719526	4.32775749	4.38236468	3.92245538	4.13975699	4.21126516
NONHSAG025872	3.36856023	3.60135364	3.53698718	3.47664255	3.36354323	3.55106428	3.17934876	3.15878376	3.38647824
NONHSAG025881	2.36308287	2.25577765	2.28406798	2.29911238	2.34715452	2.65879635	2.39306696	2.44984301	2.24485664
NONHSAG025897	5.4099178	5.19534557	5.69860421	5.53994918	5.61189836	5.31261069	5.57542796	5.37893095	5.60599483
NONHSAG025979	2.21080662	2.19540744	2.25041159	2.54340944	2.37063766	2.52171402	2.37864426	2.40486507	2.55189786
NONHSAG025986	3.33122398	3.380715	2.92418446	3.17433976	3.35837106	3.34251539	3.34725072	3.05432065	3.32076472
NONHSAG026033	2.9556289	2.81212346	2.67212643	2.88164432	3.2129157	2.83602919	2.86567543	3.00999305	2.76682591
NONHSAG026052	3.11659722	2.98391453	3.26913836	3.49898563	3.05514932	2.86392395	3.07192486	3.12322159	3.12284592
NONHSAG026094	4.87375633	5.01574957	5.00760144	4.78402921	4.68233237	4.86531997	4.62053277	4.60703694	4.83005174
NONHSAG026202	3.74091987	3.75560997	3.82264837	3.52704546	3.78314368	3.61845872	3.6502378	3.56265089	4.29542852
NONHSAG026216	3.53602561	3.6746972	4.01103744	3.70731324	3.89799372	3.78350329	3.74551155	3.91793281	4.19608948
NONHSAG026311	5.02571539	4.92514452	4.86698099	4.97572546	5.13433	4.79395496	4.90344371	4.8323742	5.104006
NONHSAG026360	4.31799684	4.41236586	4.22038274	4.48010527	4.09655217	4.4075023	3.94319056	4.48230123	4.15323972
NONHSAG026376	2.47389238	2.37866194	2.64401557	2.65702392	2.44648287	2.4512419	2.50184431	2.1424625	2.54116311
NONHSAG026396	3.65708997	3.40357797	3.47821806	3.20305513	2.93414596	3.32706162	3.20646223	3.60272672	3.41636622
NONHSAG026410	4.04010809	4.39847358	4.38283064	4.47740315	4.39761406	4.17975719	4.26659105	4.26955131	4.40024555
NONHSAG026448	2.22201607	2.13081064	2.29077606	2.26520281	2.07069846	2.30675615	2.42484448	2.28169073	2.32080954
NONHSAG026475	5.06424591	5.02693642	5.00225023	5.12783475	4.87548243	5.04346069	5.0422017	4.83959655	5.25389024
NONHSAG026539	2.33198739	2.21508432	2.37398311	2.62196817	2.46219818	2.43173558	2.43904531	2.36489779	2.25242532
NONHSAG026542	2.67282441	3.10956656	2.88934155	3.18664675	2.62447012	2.66089189	2.84200941	2.97977465	3.12249466
NONHSAG026552	6.86221812	6.58301896	6.62169301	6.66989075	6.68174576	6.8483625	7.21911177	6.9614233	7.04103974
NONHSAG026569	5.61357852	5.46685584	5.64058494	5.75602765	5.69880961	5.66526462	5.34536513	5.48396035	5.27586415
NONHSAG026592	4.1814552	4.30753123	4.41669902	4.4016125	4.61085341	4.44088708	4.54773176	4.52419479	4.72491666
NONHSAG026607	3.97896157	3.88333963	4.18209797	3.99077222	3.95366104	3.80095749	3.79753412	4.09045847	3.97242562
NONHSAG026618	4.83490211	4.60855167	4.69520678	4.61912376	4.92094008	4.72832899	4.53025724	4.48975067	4.82113194

NONHSAG026620	3.32275724	3.29130808	3.40719038	3.34595988	3.48427923	3.53447777	3.51079969	3.43244001	3.59358007
NONHSAG026634	3.81863751	3.45972175	3.67974468	3.65609281	3.95822307	3.76667405	3.89159869	3.75604576	3.74856686
NONHSAG026661	6.72365701	6.41770313	6.23236348	5.89634402	6.26888456	6.27744049	6.24158074	6.27958393	6.2665169
NONHSAG026676	2.37146552	2.48634814	2.49505726	2.39261146	2.361116	2.24886776	2.37463997	2.60987828	2.30347336
NONHSAG026716	7.95207229	7.93914023	7.9109832	7.9495775	7.8701735	7.8918412	8.07271272	8.00928226	7.98175086
NONHSAG026725	6.40939572	6.430966	6.42037221	6.33801719	6.42113912	6.34522322	6.63351527	6.42231377	6.58499956
NONHSAG026729	4.28717485	4.27602894	4.46358545	4.80018525	4.62896744	4.7828419	4.41375367	4.39479718	4.23184401
NONHSAG026751	3.24712253	3.33600111	3.35837864	3.42375507	3.06555444	3.41005396	3.57979253	3.31670474	3.39811567
NONHSAG026771	4.65393584	4.69387159	4.71400621	4.84628394	4.80955788	4.97237703	4.56590449	4.53283369	4.97567447
NONHSAG026775	5.94379915	5.80824592	5.84465707	5.80653915	5.76590382	5.8269119	5.9684026	5.82781704	5.81146334
NONHSAG026798	2.92608377	2.77136191	2.84575727	2.70630505	2.76070892	2.77231426	2.73018098	2.73056173	2.76933764
NONHSAG026834	5.98606794	5.74407474	6.04261828	6.13501436	6.0347766	6.01449928	6.1491098	5.90659229	5.99555871
NONHSAG026851	5.8901251	6.01472842	6.06531486	5.93192407	6.14950062	5.77241316	5.90076605	6.01720775	6.30986465
NONHSAG026854	4.76568484	4.76040868	4.82621147	4.95755385	4.83786039	4.6527428	4.82196009	4.82190143	4.68887067
NONHSAG026890	2.81961831	2.78476758	3.0071106	2.8289608	2.95909735	2.8438939	2.96680293	2.85565251	2.92964736
NONHSAG026912	5.26376567	5.40277212	5.43474036	5.53542556	5.52137481	5.38356227	5.42675137	5.42964244	5.71065444
NONHSAG026940	6.22997134	6.36911525	5.87448187	6.42291442	6.20460683	6.11029664	6.258669	5.98913556	6.15848601
NONHSAG026942	4.50335878	4.31218541	4.3607594	4.40238874	4.53583439	4.72401429	4.66507214	4.57082334	4.58127248
NONHSAG026944	6.55938999	6.65559974	6.46045632	6.82766443	6.4654003	6.47342626	6.47560585	6.34492479	6.122391
NONHSAG026956	5.01573495	5.50223235	5.25656821	4.942771	5.37639579	5.52325942	5.71726461	5.24302968	5.64244773
NONHSAG026967	4.72391038	4.32262952	4.98169705	4.64821781	5.0376248	4.73355176	5.1205662	4.71980488	5.13923491
NONHSAG026969	2.7045468	2.61011814	2.74018015	2.85827863	2.78933447	2.80791987	2.71079951	2.69218559	2.64637482
NONHSAG026973	2.90152735	2.67742444	2.59576197	2.9471267	3.0308289	2.91466458	2.71507079	2.64038546	2.88245866
NONHSAG026985	3.74442926	3.79372997	3.66948191	3.65529032	3.69692688	3.7985901	3.66245598	3.70762201	3.71110558
NONHSAG026995	5.31704878	4.8348239	5.07405708	5.22432083	5.42464058	5.19815839	5.1171274	4.93077729	5.36861026
NONHSAG027017	3.31115267	3.25987083	3.45739027	3.31204119	3.31439253	3.48763772	3.72769827	3.23845439	3.59860973
NONHSAG027018	4.33284498	4.331249	4.43493578	4.58844872	4.69500435	4.55851741	4.29471452	4.39340997	4.8631134
NONHSAG027025	5.00174355	4.89172929	4.84594257	4.74182248	5.03284001	4.68024286	5.15835764	4.88450363	5.08501259
NONHSAG027026	3.5585646	3.29174474	3.76032325	3.38384743	3.79928868	3.83938889	3.59617206	3.97442959	3.95068655
NONHSAG027036	4.26267774	4.10014681	4.26779764	4.3299528	4.30611053	4.19968724	3.78669888	4.14105779	4.28474824
NONHSAG027063	2.68030526	2.6008068	2.58476623	2.70544104	2.66787201	2.71886428	2.62643037	2.69044336	2.70905327
NONHSAG027074	3.92935675	3.74139529	3.9921375	4.38523822	4.04211293	4.02495384	3.85870909	3.90296046	3.87658417
NONHSAG027078	3.59757361	3.5559093	3.64912162	3.49284785	3.82540166	3.67119884	3.65641129	3.45778795	4.09481841
NONHSAG027116	2.50007909	2.55010152	2.42100957	2.60029981	2.48556853	2.52026368	2.43904531	2.71540727	2.57644773
NONHSAG027134	2.71669181	2.7178493	2.59812923	3.00506881	2.7940252	2.73595029	2.74028151	2.8038332	2.7474071
NONHSAG027135	2.28329801	2.22895828	2.70824146	2.09929322	2.22475941	2.29414336	2.21250236	2.19860132	2.42273013
NONHSAG027140	3.21020173	3.16721427	3.59540741	3.26753015	3.44494052	3.44250879	3.17934876	3.28310582	3.25274471
NONHSAG027170	3.83231989	3.7661138	3.8310223	3.92771714	3.44406531	3.82294372	3.877845	3.60527673	3.86538883
NONHSAG027173	2.22565584	2.12895288	2.09490797	2.37330634	2.06627068	2.17425467	2.28085107	2.14629184	2.27267624
NONHSAG027186	4.22806737	4.3576521	4.21855354	4.37866234	4.36135683	4.07898954	4.51609155	4.15748913	4.3651848
NONHSAG027205	4.61737612	4.57742279	4.77329179	4.47402033	4.90590114	4.87715689	4.51974781	4.41752171	4.82622947
NONHSAG027215	3.03199756	3.17897944	3.22629221	3.15999644	3.60285857	3.23156582	3.38305562	3.31439253	3.3516813
NONHSAG027327	2.58275384	2.64083099	2.87058066	2.68598539	2.59950293	2.62307144	2.68285408	2.7050183	2.57093398
NONHSAG027345	2.39416233	2.44793801	2.55197072	2.28522758	2.68357564	2.28837866	2.3843826	2.3618943	2.56759727
NONHSAG027346	2.70170462	2.54528713	2.74174528	2.50584324	2.71095975	2.45598737	2.58979282	2.49457398	2.45639054
NONHSAG027354	3.29607515	3.39814919	3.22725234	3.6175628	3.29607515	3.71109612	3.26617463	3.2866162	3.49270091
NONHSAG027362	4.50380335	4.50463744	4.49860509	4.58579787	4.55784165	4.64329081	4.61156488	4.77251384	4.92923662
NONHSAG027390	4.55032147	4.35652018	4.50076914	4.53153925	4.40793483	4.32015007	4.60156089	4.42986515	4.81262338
NONHSAG027419	3.84064239	3.87131154	3.34096815	3.18972731	3.60534171	3.45065411	3.41075368	3.37634717	3.52847686
NONHSAG027464	4.15128862	4.02222746	4.00799194	4.16490309	3.88467556	4.11383295	4.04494177	4.25536576	4.19618277
NONHSAG027468	4.57244435	4.1996922	4.58115035	4.53474297	4.68838067	4.49785942	4.31165113	4.41291137	4.51731705
NONHSAG027505	3.56507863	3.48113496	3.60816402	3.60602309	3.36230841	3.51563092	3.50928426	3.56863317	3.67431551
NONHSAG027515	3.05947013	3.12139925	3.04585393	3.21351379	2.91543443	3.0150075	3.0789663	2.89912017	3.08801619
NONHSAG027519	6.27608606	6.45570592	5.9888725	6.36012362	6.22132314	6.36530382	6.12084234	6.41631502	6.22074399
NONHSAG027535	3.79979175	3.48964563	4.03935023	4.15363087	3.97406919	4.11063207	3.86088943	3.83397972	3.63549154
NONHSAG027545	2.45119168	2.52943839	2.64925128	2.68718368	2.6083118	2.70482238	2.78845963	2.69209945	2.96996524
NONHSAG027550	2.32367024	2.40487776	2.36830618	2.36748544	2.6240706	2.44168543	2.33444994	2.61189472	2.43904531
NONHSAG027551	2.89256306	2.66306459	2.82951749	2.96285685	2.85208423	2.99339146	2.81237086	2.55955319	2.74162199
NONHSAG027552	3.96821491	3.83039933	4.11633654	3.9932903	4.00601396	3.75427441	3.97770338	3.89840184	3.90195031
NONHSAG027560	3.10289581	2.5858777	2.93068635	2.35394584	3.00529457	2.78662875	3.01891211	2.89625869	3.34966183
NONHSAG027603	3.76763245	3.37152198	3.85994882	3.57214143	4.04056004	3.70286513	3.78669888	3.67201492	4.0951466
NONHSAG027626	6.11415292	6.37199141	6.26110342	6.29954023	6.40799662	6.42396602	6.55441678	6.33214485	6.81769416
NONHSAG027634	4.01819035	3.94517824	4.08060456	4.05190265	4.05140798	4.05379293	4.03185886	3.9548773	4.07258747
NONHSAG027639	5.41076082	5.4796894	5.51485504	5.65048155	5.46047573	5.36642562	5.48043777	5.51661323	5.56415593
NONHSAG027647	4.86845971	4.96325715	4.91105341	5.05214747	4.27038183	5.03512098	4.98469891	4.61814122	5.04227159
NONHSAG027670	2.24458855	2.33295301	2.35961864	2.399765	2.39625839	2.19876685	2.17896015	2.43330179	2.50279249
NONHSAG027674	3.22390355	3.16854792	3.07618057	3.39712174	3.24388307	3.01183439	2.92560749	3.29547378	3.40932339
NONHSAG027684	4.49498849	3.95394702	4.25337898	4.70875874	4.09874837	4.30931069	4.37187379	4.34228093	4.34879273
NONHSAG027686	3.75092962	3.98775458	3.8206993	3.51265318	3.12478608	3.80043246	3.65526611	3.90083101	3.77653668
NONHSAG027687	3.27461718	3.2987187	3.15931671	3.31958364	3.33110823	3.31942838	3.31690828	3.02326141	3.92307357
NONHSAG027711	2.71079027	2.63801457	2.79780457	2.82656588	3.13406321	2.96741621	2.72207725	2.77640268	2.96953457

NONHSAG027714	4.51685977	4.5958126	4.41483448	4.7105544	4.66245776	4.59631001	4.18274021	4.52460508	4.31102987
NONHSAG027715	4.85329368	4.90691571	4.91832959	4.76921508	5.13662505	5.20567391	4.9526307	4.93405425	5.13264621
NONHSAG027736	3.08943583	3.11044529	3.18207487	3.38531726	3.39871987	3.21990504	3.3106697	3.21668318	3.60254134
NONHSAG027755	3.43905553	3.94050426	3.6446027	3.59534797	3.41946433	3.3443568	3.36495831	3.4435504	3.43493602
NONHSAG027769	3.69835515	3.59422947	3.84596902	3.65127975	3.56577779	3.44259145	3.7312292	3.86130699	3.78438346
NONHSAG027811	3.82127787	4.1237538	3.66837671	4.22149973	3.86641633	3.85767513	3.84171926	3.79648988	3.93679771
NONHSAG027819	4.82876714	5.00156014	4.97643399	5.09194463	5.35264965	5.18017546	5.11851801	4.92702777	5.03067307
NONHSAG027821	2.10836554	2.16341674	2.29471984	2.24932034	2.22805304	2.17154276	2.07369521	2.35990073	2.19580336
NONHSAG027833	2.86106227	2.74170056	2.90617432	2.64706891	2.82950948	2.82533599	2.74860531	2.94763051	2.74179152
NONHSAG027841	6.31228276	6.41433612	6.55459026	6.46251425	6.23556506	6.42469434	6.52881111	6.19260159	6.65634627
NONHSAG027868	8.86274599	8.96424756	8.88637697	8.87944224	8.87537806	8.81527164	8.58496444	8.66844026	8.79018724
NONHSAG027869	8.48520028	8.83045193	8.54195496	8.13562644	8.44524935	8.0918289	8.15701116	8.22337781	8.20924835
NONHSAG027897	3.32882582	3.30529566	3.29535918	3.31607048	3.28555468	3.49337744	3.35234581	3.51922844	3.7289359
NONHSAG027917	3.50011581	3.11599992	3.05462686	3.34288024	3.15127993	3.17047125	3.25981762	2.62366785	3.26294429
NONHSAG027919	4.59692519	4.55819314	4.68659657	4.55315969	4.74478503	4.55530843	4.54546827	4.56721116	4.94537927
NONHSAG027954	4.68675417	4.70219618	4.79002052	4.54004828	4.7905694	4.87728801	4.82384387	4.73102946	4.92248109
NONHSAG027959	3.05614145	3.06823286	3.04133902	3.00376049	3.22827373	3.02706269	3.0503287	3.14890655	3.34190848
NONHSAG027967	3.18308758	3.23515444	2.75266241	3.04374833	3.2082276	3.02223455	3.17551186	3.00282564	3.05724114
NONHSAG027970	4.9385306	4.99919214	5.24933441	5.23012124	4.97240965	5.08386504	5.08688034	4.89287102	5.08727702
NONHSAG027998	3.26818387	3.35194643	3.28683785	3.24085161	3.37984466	3.33580188	3.23599572	3.26712083	3.41911583
NONHSAG028005	3.80287878	3.6822943	3.73711488	3.84299822	3.82554899	3.67482599	3.83917362	3.74890448	3.8078533
NONHSAG028023	3.82639558	3.69762037	3.90868745	4.06063309	3.76122406	3.90278392	3.89951852	4.05714663	4.23025037
NONHSAG028024	2.44948166	2.49356529	2.46449797	2.62164147	2.53274104	2.63366228	2.52241104	2.36949507	2.53061728
NONHSAG028072	2.45833364	2.66342315	2.61247076	2.9884648	2.14075447	2.29185137	2.19187368	2.42207029	2.22718328
NONHSAG028118	3.58725553	3.59985971	3.45062427	3.65868984	3.73046597	3.4461509	3.66760599	3.70736621	3.84444002
NONHSAG028155	4.68123234	4.58001972	4.78701743	4.70526857	4.78695406	4.64614069	4.75182967	4.57568967	4.99764497
NONHSAG028160	6.59948332	6.72988522	6.52249215	6.46756312	6.41458834	6.62803993	6.53584531	6.61422283	6.52908621
NONHSAG028184	3.5033958	3.76062432	3.89002547	3.5149396	3.88820422	3.73173265	3.93097427	3.64171351	3.89680295
NONHSAG028242	4.36570589	4.35456925	4.17937965	4.21520525	4.39293209	4.73468708	4.5166728	4.36401338	4.16611138
NONHSAG028243	3.77917348	3.75858823	3.93967765	3.94329753	3.76143101	3.55333766	3.88735379	3.86102336	3.90304785
NONHSAG028249	2.47641894	2.45832158	2.41065813	2.36403975	2.39424088	2.43471409	2.47961391	2.29233528	2.36709254
NONHSAG028283	2.77826457	3.28892093	2.89023336	2.95596885	3.06620566	3.45789757	2.88383376	3.12871843	3.43210741
NONHSAG028291	4.39596394	4.22287889	4.5114258	4.47117125	4.64427185	4.52660287	4.70626084	4.45641362	4.67951438
NONHSAG028305	2.67833525	2.80597907	2.92484661	2.76448315	2.53841692	2.77834731	2.75393117	2.7433792	2.87675642
NONHSAG028340	2.85373002	2.90343557	2.79673751	2.97714902	3.19423468	2.99878766	2.87337705	2.87818723	2.9893075
NONHSAG028349	3.21886245	3.46680806	3.34157849	3.59137984	3.4088371	3.20687431	3.11971254	3.21993898	3.39477509
NONHSAG028408	2.71154179	2.77515514	3.04329595	2.80197109	3.1267067	2.91835584	2.77515514	2.82875423	2.80959805
NONHSAG028420	2.80180878	2.51556416	2.49294796	2.48892348	2.64937656	2.53068767	2.49547963	2.40113434	2.5758998
NONHSAG028439	4.16857229	4.18740976	4.407343	4.13683014	4.27210344	4.26724504	4.30303647	4.13054199	4.26066044
NONHSAG028464	2.72150494	2.65555812	2.57105434	3.00204318	3.16879824	2.80664866	2.73147661	2.59987559	2.63670721
NONHSAG028465	5.108968	5.03799523	5.19270593	5.06531598	5.77332801	5.47747097	5.56874418	5.58711647	5.72858452
NONHSAG028467	6.62205736	6.00068187	6.23245774	6.48149105	6.30495968	6.57330541	6.48005799	6.51314954	6.42909858
NONHSAG028469	3.94489306	4.08541951	3.84777532	3.91475716	3.98457647	3.88934588	3.93215058	3.66037515	3.96520794
NONHSAG028525	5.19672253	5.70611741	4.96144588	5.16977893	5.28621007	5.22838121	5.28712288	5.13003574	5.16712024
NONHSAG028576	4.23548982	4.44052814	3.98617148	4.25042414	4.3187484	4.51179965	4.34982432	4.45984632	4.16043491
NONHSAG028585	6.26358484	6.44923062	6.29906005	6.17884208	6.36460777	6.26769423	6.28219577	6.27713101	6.20996204
NONHSAG028593	6.42291442	6.64351795	5.93193913	6.47960981	5.95656458	6.62242326	6.05964471	6.31473489	5.04694629
NONHSAG028595	5.95236367	6.23516618	6.06546221	5.98898751	5.91494661	6.19992852	6.06999442	5.92079346	5.53445912
NONHSAG028611	2.6698261	2.81062125	2.72659142	2.78037388	2.59713082	2.79287987	2.68663954	2.72201259	2.61241609
NONHSAG028627	7.658797	8.13579502	7.60697232	7.95238646	7.82634093	7.89397733	7.93989965	7.94694969	8.00265282
NONHSAG028726	3.3499304	3.4600941	3.40306353	3.1367356	3.3489018	3.26069107	3.24806469	3.50367902	3.47136998
NONHSAG028734	3.77037148	3.98759493	3.82999525	3.77075221	4.00506067	3.71418143	3.82054627	4.05056466	3.93558155
NONHSAG028743	3.44201767	3.28082569	3.39341331	3.21840707	3.62290463	3.37070606	3.57809605	3.53703352	3.86509059
NONHSAG028794	3.04801183	2.77773051	3.07853189	3.07924046	2.95147606	3.08289003	2.96088371	3.09785564	3.29839181
NONHSAG028929	4.86898397	4.63696261	5.03206001	5.11771685	4.90626943	5.11771685	5.04513064	5.18070088	4.88287116
NONHSAG028931	2.42140593	2.61989266	2.65463152	2.70884947	2.59618422	2.64175164	2.50765576	2.58069146	2.53801248
NONHSAG028939	5.10389089	4.94005253	4.95650176	4.97313079	5.28677689	4.95411485	5.28825973	4.96348676	5.31224711
NONHSAG028975	6.21025935	6.3674601	6.11477574	6.44826711	6.14636704	6.53382305	6.42660468	6.2047759	6.00753991
NONHSAG028980	5.29342338	5.44442504	5.22857853	5.41166152	5.25293877	5.23099514	5.4627412	5.30087895	5.30589522
NONHSAG029002	7.06583668	7.08447238	6.84849117	7.04767488	6.90340358	7.05691916	7.28764541	7.05962888	7.22363657
NONHSAG029008	3.34190058	3.21855033	3.02601951	3.18462356	3.05687483	3.11187352	3.08595533	2.8827046	3.01471098
NONHSAG029029	8.1791148	8.43332644	8.01858455	7.93685814	8.21652476	7.98603456	8.04746878	8.20441064	8.22603338
NONHSAG029047	3.15585929	3.19712634	2.9714762	3.05266634	2.98940596	2.93606254	3.01761798	3.05734366	3.10927932
NONHSAG029080	5.108968	5.03638223	5.00947893	5.07953513	5.29177955	5.30664219	5.22751986	4.95971388	5.55940506
NONHSAG029181	3.25319908	3.20862085	3.23959686	3.30588364	3.45356427	3.25107568	3.60100788	3.49873254	3.35427038
NONHSAG029187	5.2572941	5.41154475	5.02145556	5.42405472	5.23057838	5.26377879	5.21716879	5.23401543	5.09200509
NONHSAG029193	4.21043073	3.95790685	4.19937951	4.03901605	4.81129119	4.15849949	3.92951525	4.47708938	4.30234556
NONHSAG029202	6.97193262	7.08246753	6.97931988	7.02332814	6.80816951	6.84005004	6.79830193	6.79008366	6.68784207
NONHSAG029206	4.17656666	4.27062755	3.94378287	4.17197312	4.12898525	4.63277739	4.42086916	4.6652836	4.16469166
NONHSAG029225	3.32975416	3.4839968	3.66024501	3.41445661	3.62737502	3.37131784	3.75529343	3.35646578	3.79627883
NONHSAG029338	5.66070809	5.19945807	5.32781104	5.52554302	5.48296021	5.71540332	5.52313601	5.48325799	5.79023391

NONHSAG029343	4.21561429	4.25214326	4.07946606	4.0857061	4.33038754	4.32130176	4.36595276	4.30411971	4.01630255
NONHSAG029351	4.23767511	4.32603564	4.30206751	4.52860316	4.59623118	4.60951416	4.60455265	4.45313146	4.60286669
NONHSAG029390	3.1413411	2.6507453	3.13116486	2.7961571	2.67910479	3.05514228	3.06717183	2.87095147	3.01973271
NONHSAG029407	2.96701909	3.05702041	3.14375171	3.08231981	3.22827373	3.22838723	3.16951934	3.09873085	3.3018636
NONHSAG029451	2.67629185	2.76640754	2.72262886	2.90566241	2.74021569	2.64859949	2.68628576	2.62722838	2.62156983
NONHSAG029458	5.15328831	5.7308376	5.31115734	5.2370286	5.24807422	4.72673418	4.46885795	5.30973284	5.59707352
NONHSAG029466	2.35109563	2.59624823	2.63108684	2.92056616	2.88013536	2.59237072	2.74295754	2.59428409	2.5758998
NONHSAG029469	4.18025761	3.9188053	4.32876552	4.60069395	4.3586489	4.28164949	4.27275602	4.51295886	4.30897267
NONHSAG029483	2.65247112	2.6715212	2.74889253	2.75299761	2.77950615	2.69425846	2.74530231	2.62254765	2.79664038
NONHSAG029505	2.61914089	2.62696698	2.57647415	2.4087907	2.43639641	2.74481406	2.60727336	2.72749676	2.71780691
NONHSAG029519	3.25317123	3.30650999	3.21425063	3.37887788	3.43088621	3.29574921	3.35395798	3.18172766	3.45009065
NONHSAG029550	3.11860631	3.03667577	2.99070346	3.12474306	3.00506998	3.09074411	3.11472726	3.04704408	3.16181125
NONHSAG029563	3.94795324	3.73642993	3.77452148	4.25172937	3.92924113	4.1282922	3.76675373	4.21450789	4.25643884
NONHSAG029600	6.01579832	6.18784368	6.2792612	6.1113031	6.2951603	6.33688566	6.21678954	6.1121002	5.88313142
NONHSAG029615	3.25534647	3.03999691	3.15801282	3.09720797	3.14477409	3.17551331	3.11164394	2.84723127	3.2852963
NONHSAG029629	5.83861029	5.9009827	5.90168508	5.959955	5.80060612	5.91525696	5.86338124	5.81714801	5.88656941
NONHSAG029651	3.06535367	3.55581558	3.0421844	4.10873452	3.46490738	3.20725433	3.30307415	3.29796925	3.23034366
NONHSAG029656	3.03072605	2.93617843	3.28321598	3.14887386	3.09264443	3.11965473	3.07865625	2.86097291	3.11843683
NONHSAG029691	4.15673122	3.76457586	4.04559414	3.66305045	3.75101696	3.88543838	4.39063183	4.05377291	4.53511312
NONHSAG029703	3.54720595	3.63718482	3.47346568	3.66131611	3.25826472	4.19496703	3.94522219	3.28511935	3.67173513
NONHSAG029707	3.07279162	3.27172089	2.97237913	3.40272088	3.03116074	3.25107568	3.34263154	3.10917078	2.79175029
NONHSAG029741	2.44545306	2.31894984	2.41245794	2.38974886	2.4066172	2.41381703	2.75182471	2.57039999	2.73291012
NONHSAG029752	2.75031287	2.83275617	2.84994889	2.87270225	2.51971911	2.91047856	2.67334489	2.64749663	2.84009215
NONHSAG029789	3.07141006	2.63709791	2.61467411	2.7365298	2.8715657	2.66208092	2.55418672	2.50101296	2.63786744
NONHSAG029815	2.62737603	2.58001863	2.49457345	2.44866354	2.93415464	2.80559432	2.79272252	2.60875221	2.81485956
NONHSAG029818	2.76147783	2.90543985	3.01052953	2.96294463	3.05217245	2.96035946	2.94727333	3.08320163	2.93260079
NONHSAG029852	5.67012518	5.73249435	5.8162828	5.59596051	5.8371154	5.93976422	5.79904373	5.67067856	6.16365335
NONHSAG029854	6.70384839	6.47188207	6.30460198	6.16077079	6.35141882	6.40892921	6.71977813	6.27047611	6.59972865
NONHSAG029904	2.80631527	2.91713659	2.80378029	3.25992742	2.92130104	2.76906172	3.1113385	2.66690118	3.26782989
NONHSAG029916	2.51595413	2.95540771	2.71316069	3.1539238	2.89994253	2.95768011	2.55112948	2.84042143	2.67298062
NONHSAG029920	3.52998187	3.34360462	3.38593269	3.60627645	3.58221209	3.37500816	3.43217142	3.42697536	3.38902761
NONHSAG029991	2.44410747	2.40616669	2.51711826	2.82396398	2.47757076	2.67832216	2.59390941	2.52684935	2.6150601
NONHSAG029995	3.48071228	3.17284388	3.21080245	3.1053945	3.15807836	3.53028278	3.36410365	2.8443743	3.22349413
NONHSAG030001	2.67093457	2.79291817	2.66403756	2.71313997	2.75708551	2.72318003	2.74932409	2.70179047	2.69333423
NONHSAG030002	2.2584955	2.38580168	2.21566807	2.41279071	2.47319205	2.37792099	2.28193178	2.2169435	2.31769562
NONHSAG030016	3.67396573	3.53912172	3.54994	3.8649012	3.74758884	3.60584012	3.5412446	3.32922934	3.73454053
NONHSAG030028	2.47937566	2.24240631	2.40505641	2.29021524	2.43366885	2.43944153	2.62051178	2.24225579	2.32438145
NONHSAG030049	2.46106108	2.41050634	3.00747161	2.99454866	2.63081385	2.58936873	2.59086755	2.57103432	2.61327396
NONHSAG030056	2.94009781	2.85878502	2.62129249	3.07615233	2.58486825	2.99304678	2.79849756	2.9573645	2.75380116
NONHSAG030133	2.35138783	2.47566099	2.56052762	2.46995011	2.54884006	2.28270967	2.36359257	2.62722838	2.46702138
NONHSAG030140	2.57644296	2.37607558	2.35563548	2.5041295	2.36435738	2.47591358	2.51565541	2.36856846	2.4101225
NONHSAG030166	3.33149467	3.14093563	3.28964718	3.49520541	3.38844655	3.50893083	3.23620589	3.44151269	3.33015932
NONHSAG030168	2.62332806	2.6631599	2.72749232	2.75698211	2.36552661	2.49892535	2.60285952	2.7348408	2.65960736
NONHSAG030187	2.47736808	2.47553987	2.56765448	2.5139862	2.48974091	2.51587645	2.43494486	2.52865783	2.6756508
NONHSAG030263	2.86654309	2.93832342	3.03352853	3.08856646	2.8692014	2.886544	2.79292945	2.887775	2.79434405
NONHSAG030278	6.5099151	6.68357434	6.68528743	6.64051755	6.65392977	6.7677499	6.7319359	6.62429338	6.61893153
NONHSAG030325	4.26653858	4.01998582	4.42089686	4.37048291	4.56803261	4.29838231	4.27623966	4.31229831	4.12258559
NONHSAG030326	3.12178748	3.03238292	3.05178652	3.2143069	2.86407929	3.03961284	3.0974814	3.18972731	3.1313038
NONHSAG030351	2.69889402	2.65543579	2.68662733	2.79784058	2.97935103	2.70243537	2.7907752	3.2578037	2.69620414
NONHSAG030358	3.10991162	2.91505602	2.90807578	3.18419007	3.0679521	2.87400086	2.85832951	3.03900991	3.07052224
NONHSAG030374	2.49587873	2.41362176	2.3488071	2.79865525	2.742568	2.62208485	2.43501896	2.49184671	2.66522005
NONHSAG030400	4.94124719	5.19399415	4.8411022	4.9160064	4.64948457	4.93801065	4.76557706	4.94566501	4.96012628
NONHSAG030406	4.82382248	4.77440057	4.78849916	4.52417721	4.94156715	4.57406511	4.76584149	4.52920263	5.10779533
NONHSAG030459	3.21713944	3.13291828	3.30105626	3.35063266	3.25836077	3.40689654	3.32645278	3.27076059	3.55969149
NONHSAG030469	5.22644049	5.21820576	5.30937244	5.57411983	5.15083915	5.39985462	4.98287961	5.22971051	5.54353257
NONHSAG030483	4.20840425	3.99622797	4.14843912	4.05152202	4.33365907	4.1337942	4.11868322	3.94046889	4.11671017
NONHSAG030489	3.22045071	3.44629401	3.38832007	3.45550599	3.53474157	3.42210259	3.63682197	3.54414453	3.58227929
NONHSAG030493	2.45638093	2.52524649	2.59758151	2.48705412	2.52803558	2.35932804	2.73477507	2.59723602	2.75062281
NONHSAG030495	3.65455671	3.69809068	3.6501385	3.7275746	3.66332062	3.60043976	3.86910297	3.63346885	3.78892857
NONHSAG030509	3.00069122	2.95716258	2.95685012	2.90310677	2.87560396	2.93945397	3.17934876	2.9573645	2.76420392
NONHSAG030613	2.35806304	2.36431174	2.58923851	2.15151102	2.5081571	2.25401204	2.37230384	2.44730001	2.62457324
NONHSAG030629	3.82657316	3.67419831	3.73014636	3.63287044	3.71437944	3.48529223	3.73286613	3.7450899	3.85255753
NONHSAG030633	8.54444144	8.65950667	8.47875468	8.30532739	8.53436802	8.08267534	8.59862783	8.55492669	8.56066831
NONHSAG030634	5.81823595	5.88481218	5.50650325	5.83343191	5.80018249	5.54364799	5.71547864	5.68214283	5.45911245
NONHSAG030637	3.580164	3.52914785	3.3771209	3.56422016	3.40385766	3.76580986	3.49579021	3.50409123	3.41732282
NONHSAG030676	2.46467624	2.4004756	2.76215165	2.16215073	2.47725793	2.46130398	2.49915371	2.58264927	2.71220405
NONHSAG030681	4.65026932	4.5081508	4.7056729	4.59799465	4.7067767	4.5096495	4.56491059	4.4320356	5.02696677
NONHSAG030683	4.32957074	3.7053087	4.13602322	4.06956356	4.35823414	4.42298291	4.37479025	4.18382011	4.22852465
NONHSAG030688	2.39110994	2.32390107	2.38999678	2.64737333	2.36517861	2.64444413	2.52274554	2.34368673	2.57047656
NONHSAG030757	6.59481005	6.16575802	6.61355685	6.54280571	6.78258162	6.62482549	6.43380246	6.66333749	6.67132533
NONHSAG030767	6.46906305	6.47087059	6.53397641	6.47279338	6.7036388	6.61044871	6.67954107	6.35787323	6.77024117

NONHSAG030783	4.33903434	4.45933376	4.87926764	4.17489563	4.32977266	4.58203012	4.62460806	4.50821317	4.25628962
NONHSAG030819	3.55390508	3.22663275	3.30521979	3.62479723	3.24289009	3.47209476	3.3406808	3.39796264	3.20156854
NONHSAG030850	2.38066434	2.34299793	2.33963628	2.30898893	2.21844103	2.36791591	2.16763981	2.32671218	2.15364433
NONHSAG030878	4.05929176	3.9535268	3.85950892	3.92489251	3.96006444	4.35565085	4.14440124	4.03209218	4.49939524
NONHSAG030888	5.3376043	5.13505527	5.32240087	5.24449954	5.49907518	5.37780869	5.24850406	5.28693523	5.68378092
NONHSAG030933	5.07767384	5.20062244	5.24092589	5.4077918	5.03821457	5.19013038	4.86888652	4.61347462	4.66427019
NONHSAG030937	3.92329186	4.12735206	4.01907543	4.09465024	4.12659745	4.08281069	4.24474714	4.15365894	4.2659509
NONHSAG030940	3.3541671	3.15222485	3.25632424	3.01521871	3.34034973	3.46559535	3.45446798	3.37788085	3.64202029
NONHSAG030957	4.04431178	3.7935599	4.1246485	4.04283109	4.00683223	4.15926865	3.98627936	3.91527989	4.19815649
NONHSAG030971	4.4586089	4.36963343	4.53177163	4.67776891	4.56100592	4.43232136	4.43104513	4.48422661	4.4640123
NONHSAG030992	5.52597455	5.42172703	5.78410784	5.53398467	5.70347149	5.5608566	5.46104326	5.58913371	5.72496977
NONHSAG031103	4.26804224	4.29711652	4.04306985	4.46418274	4.15051277	4.45647876	4.25827518	4.36365832	3.9077045
NONHSAG031137	3.27585219	3.40778846	3.19130783	3.38930475	3.39968981	3.06272844	3.28872451	3.45857366	3.82187815
NONHSAG031167	4.42782558	4.77400171	4.54091324	4.51139884	4.09274516	4.46046017	4.67171042	4.35366037	3.88364311
NONHSAG031168	5.60652558	5.55057898	5.60522332	5.71517601	5.62920241	5.54926223	5.58027644	5.70592264	5.56139434
NONHSAG031178	3.0110828	2.7686853	2.94805433	3.02409952	3.31439253	3.41567387	3.01982465	3.06649703	3.21956273
NONHSAG031180	6.62360827	6.51603788	6.58932366	6.24570956	7.0567895	6.45769029	6.86933707	6.47293196	6.73394516
NONHSAG031184	3.11816793	3.03774161	2.89323665	2.80270704	3.24682526	3.12630723	3.24181779	3.2113864	3.66073359
NONHSAG031203	3.9156568	3.82151308	4.036871	3.71758407	3.94144356	3.99536749	4.129223	3.93011148	3.9480239
NONHSAG031219	2.93049058	2.70157186	2.85301243	3.13386087	3.05346083	2.90190832	2.84428433	2.8238016	2.80521907
NONHSAG031232	3.85099232	3.94765765	3.98404918	3.98554885	4.11329387	4.27352022	4.1500205	3.85428868	4.02266848
NONHSAG031237	2.86611436	2.88820394	2.90772376	3.2126073	3.18156675	3.05291737	2.97052911	2.98660055	3.0832698
NONHSAG031249	3.47552703	3.77160423	3.83878806	3.68696233	3.79740251	3.42902229	3.68967635	3.65922546	3.79607995
NONHSAG031255	2.2520632	2.25483833	2.24526539	2.37429365	2.34645605	2.19272353	2.17157687	2.28574271	2.38741325
NONHSAG031276	5.62882688	5.170863	5.37104111	5.26230097	5.74025893	5.46051428	5.5509417	5.23542848	5.51455389
NONHSAG031296	2.62896701	2.45334009	2.85479589	2.65704869	2.78491652	2.68772224	2.86902852	2.88372678	2.6254214
NONHSAG031312	2.76968458	2.80824838	3.11601541	3.05173274	2.69015417	2.86403562	3.13192639	2.88738575	2.92324202
NONHSAG031314	3.07838727	2.90772071	3.08243712	3.24867684	3.08322876	3.23377026	3.09171753	2.97009507	3.2933539
NONHSAG031315	7.44385018	7.1501249	7.36419912	6.81627629	7.4260349	7.03120062	7.45889131	7.26632161	7.73544471
NONHSAG031322	6.33455216	6.06466408	6.36957659	6.34389049	6.42300107	6.31137621	6.37225946	6.2548809	6.35373349
NONHSAG031335	3.27315777	3.61799353	3.43423211	3.48853429	3.3912648	3.38712541	3.37851065	3.44279508	4.16290523
NONHSAG031372	2.60183582	2.69017747	2.71165126	2.79904162	2.75470808	2.73531466	2.69490797	2.50028193	2.59797038
NONHSAG031377	5.80392936	5.39216004	5.56531521	5.57386792	5.74354306	5.45069675	5.30167798	5.28447312	6.08914451
NONHSAG031385	5.3254619	5.11347778	5.43461334	5.41407388	5.30387955	5.33355396	5.15815302	5.12700326	5.54122999
NONHSAG031401	2.35185988	2.33224344	2.45904605	2.45050146	2.4649509	2.54500167	2.42189746	2.31623342	2.43299084
NONHSAG031461	2.99014602	3.03773437	3.06083729	3.06503033	3.08827418	3.14763624	3.08501822	3.04608373	2.90774957
NONHSAG031476	4.84835705	4.92390582	4.74903493	5.12860476	5.16715118	4.88003014	4.92013373	4.81287297	4.92303726
NONHSAG031485	4.81464254	4.637607	4.83937652	4.93539116	4.93956869	4.90977734	4.9207686	4.84526094	4.84986084
NONHSAG031489	3.54819118	3.47391165	3.59234108	3.68570631	3.99461088	3.87022803	3.62786839	3.34987118	3.62314515
NONHSAG031516	6.15703259	5.96127466	6.17255448	6.22357007	6.22389182	6.2715612	6.27083399	6.09648381	6.47056247
NONHSAG031596	5.92465226	5.9291165	6.13118519	6.11708738	5.69548258	6.19972448	5.95343249	5.8669117	5.74295336
NONHSAG031603	3.12579612	2.84045862	3.14162299	3.11605859	3.25527893	3.02373928	2.93544498	3.10733999	3.16103451
NONHSAG031617	5.91951152	6.0594104	6.04691686	5.88909396	6.03497873	5.87818004	5.90948457	5.84840068	5.71188231
NONHSAG031626	2.26493844	2.05863017	2.40568127	2.16151803	2.27575447	1.94130701	2.47652238	2.3108674	2.23971113
NONHSAG031665	2.7636427	2.91263912	2.7730624	2.9476601	2.90666647	2.80317028	3.09492915	2.93932949	3.05046619
NONHSAG031701	3.99484204	3.94053328	4.13256907	4.13512842	4.05541721	3.98217261	3.77581218	3.76699742	4.11154102
NONHSAG031748	4.62489653	4.52329174	4.47081811	4.54207822	4.32284501	4.56484476	4.51191904	4.25458068	4.53896381
NONHSAG031783	7.20468312	7.08108943	7.11678764	7.09745929	7.25926994	7.27108752	7.12034195	7.34824889	7.28173327
NONHSAG031784	4.70596038	4.46612859	4.49260531	4.89912794	4.74879326	4.90253594	4.76118003	4.70194936	4.83050863
NONHSAG031801	3.92909452	3.64150169	4.08192208	4.04050974	4.27753907	3.9483414	4.19208228	3.95680951	3.89057986
NONHSAG031805	2.63272732	2.50693456	2.36943254	2.72612989	2.4384292	2.52867632	2.42238725	2.52868336	2.52401976
NONHSAG031818	5.41247531	4.9579006	5.21706195	5.28640152	5.23355256	4.94410158	5.58995035	5.07327769	5.21743268
NONHSAG031824	7.02981799	6.92705004	6.88746335	6.38979132	6.91482328	6.5216354	6.75837258	6.79622978	6.85825002
NONHSAG031831	4.44692067	4.25715131	4.37323574	4.29342614	4.53872545	4.48468727	4.36376483	4.32153112	4.51739807
NONHSAG031845	5.12984507	5.08974343	4.95810617	5.36565067	5.22392895	5.2909753	5.41160985	5.39281482	5.39595526
NONHSAG031890	3.42422965	3.39538268	3.30683977	3.28933514	3.44381002	3.1691453	3.40262435	3.35332421	3.33304692
NONHSAG031913	5.17022262	5.04816221	5.17422983	5.26017495	5.28895945	5.0702499	5.10485453	4.94069656	5.17451461
NONHSAG031945	5.07891723	5.39821805	4.99446921	4.99610269	4.92835472	5.38145008	5.12143445	5.46237704	4.76831129
NONHSAG031951	4.34789569	4.16098022	4.46027903	4.20568658	4.42470716	4.40841626	4.39765001	4.23962259	4.29111512
NONHSAG031952	4.20246648	4.53415106	4.4946396	4.11245283	4.45421697	4.33774519	3.97842961	4.27514566	4.40683027
NONHSAG031964	4.35558282	4.11775725	4.38718481	4.33673004	4.36879855	4.41985174	4.33267896	4.35322393	4.40935488
NONHSAG032009	3.458222	3.36194785	3.53833601	3.3158453	3.51439288	3.25164992	3.53229781	3.30533277	3.70330145
NONHSAG032015	5.0783852	5.28145653	5.10590099	5.18940124	5.11729987	5.28064216	5.19976634	5.24214847	5.30783489
NONHSAG032104	5.09610612	5.25250343	5.14412842	5.21210488	4.97994166	5.25996663	5.13110237	5.04608518	5.19424084
NONHSAG032106	4.21511258	4.11563486	4.2195654	4.24206276	4.29189706	4.18767204	4.23678141	4.16440581	4.31362707
NONHSAG032135	5.68183956	5.66306653	5.3922623	5.97688116	5.45922327	5.87153367	5.41661191	5.67892476	5.45158053
NONHSAG032143	2.33779625	2.52018238	2.529047	2.60948538	2.32724269	2.55780395	2.46956242	2.42872079	2.42219407
NONHSAG032153	4.38850188	4.06611588	4.40489958	4.43270351	4.361949	4.50324889	4.18913106	4.31382453	4.54624561
NONHSAG032186	3.53001446	3.33095185	3.71295524	3.2275192	3.43397762	3.43117799	3.33656469	3.46093211	3.49145874
NONHSAG032194	2.40493179	2.64688447	2.61501003	2.54014122	2.71746106	2.61129339	2.47803482	2.3641562	2.30347336
NONHSAG032197	4.03427353	3.81592282	3.98804342	4.17843909	4.17850289	3.99001737	3.83693535	3.87971768	3.8745313

NONHSAG032235	4.59841555	4.55819314	4.91873646	4.67222411	4.8835825	5.03336435	4.57777597	4.66488139	4.56060023
NONHSAG032245	3.61419054	3.79470507	3.99355904	3.80998619	4.40159651	3.93845348	3.95156796	3.78720556	3.61542079
NONHSAG032250	3.92861453	3.80805396	4.0809156	3.99819851	4.15455916	3.92299817	4.02555404	4.03757601	3.84939293
NONHSAG032270	4.74511803	4.81214173	4.55819314	4.72224322	4.86531423	4.78451501	4.83528068	4.76170066	4.75406022
NONHSAG032285	3.55966874	3.79265169	4.00149195	3.73178275	3.78647222	3.78347703	4.0291645	4.14552406	3.64102573
NONHSAG032299	4.71201082	4.37333943	4.715594	4.49339632	4.94412406	4.61947541	4.67987991	4.61741948	5.11663523
NONHSAG032337	7.83012468	7.61421987	8.06458814	7.71859349	7.77289367	7.64233471	7.96463265	7.82579111	7.696107
NONHSAG032387	2.28855103	2.35097168	2.42083892	2.54067554	2.64959313	2.43064868	2.58461149	2.30322705	2.50481037
NONHSAG032422	5.08368467	5.40924874	4.46989927	4.87885379	5.16768136	5.20552405	5.16653005	5.00229191	5.12403258
NONHSAG032427	2.58595986	2.79450884	2.88287838	2.90529669	2.62167166	2.66647668	2.6038796	2.62007084	2.70276618
NONHSAG032431	5.14277189	4.82652215	5.08379493	5.20266187	4.87516793	4.88971553	5.12400269	5.11098215	5.08279391
NONHSAG032480	3.82281847	4.00591872	3.88657488	3.98815075	3.89149495	3.82232123	3.94677116	3.93376153	3.90206267
NONHSAG032559	3.00226984	3.04594658	3.09026644	3.29223048	3.19845572	3.30353639	3.24509489	3.02891292	3.02859609
NONHSAG032592	2.44603801	2.44435843	2.38954979	2.52042511	2.33985432	2.26994991	2.23085025	2.63292922	2.50377192
NONHSAG032593	4.09655713	4.24762122	4.21102631	4.03089997	4.51384608	4.29160743	4.38398835	4.32226491	4.55262759
NONHSAG032601	3.12999492	3.37459659	3.50244409	3.28823489	3.32784428	3.48497769	3.33249517	3.44113345	3.41636622
NONHSAG032608	6.30724588	6.37473942	6.60490903	6.61031287	6.59390957	6.44060749	6.60309148	6.46019904	6.40988678
NONHSAG032613	3.95362177	4.19058265	3.79779832	4.33044898	4.44842204	4.05650469	3.9770732	4.16591732	4.13705266
NONHSAG032620	2.75771875	2.78008535	2.51205298	2.64309166	2.68409404	2.85745935	2.63274693	2.38365285	2.47940314
NONHSAG032629	2.37907945	2.44913209	2.46755443	2.43514161	2.1872216	2.47031749	2.48801416	2.40323102	2.40493114
NONHSAG032632	3.6588075	3.71120544	3.72398139	3.88686273	4.02820627	3.70262361	3.70108145	3.74237286	3.79185349
NONHSAG032643	4.71002626	4.61750091	4.69559182	4.89762515	4.60543148	4.70945658	4.57465201	4.4484725	4.29631133
NONHSAG032649	3.22735942	2.7729115	2.90532118	2.84739379	3.40556128	3.24911647	2.87566411	2.84938913	3.09818749
NONHSAG032662	2.70604774	2.93319059	3.21670839	3.10357575	2.97689743	2.85287219	3.07427451	3.07691358	3.13120737
NONHSAG032723	3.35496384	3.30529566	3.12541829	3.07155943	3.19575899	3.23895836	3.25381502	3.07772245	3.01135388
NONHSAG032740	4.36375233	3.97602632	4.38740302	4.36852444	4.51268664	4.36888036	4.62194119	4.2646485	4.58147816
NONHSAG032746	6.13457899	6.0243254	5.99756609	5.92031795	6.10112072	6.0614599	6.21840314	6.16301284	6.39417991
NONHSAG032750	3.36378568	3.1533397	3.30909537	3.0654406	3.36924968	3.24023458	2.89220629	3.16578385	3.30042441
NONHSAG032785	5.21745624	5.24179785	5.31596874	5.34829184	5.25104189	5.45188981	5.41558255	5.29262433	5.47430158
NONHSAG032791	3.41212174	3.51491819	3.95253933	3.58508616	3.65803456	3.85528151	3.74591312	3.68156323	4.00090481
NONHSAG032815	2.24840526	2.31300805	2.04935397	2.22391401	2.22805304	2.21967338	2.16008215	2.31884364	2.07912542
NONHSAG032817	3.45397135	3.08385081	3.38706642	3.74175413	3.63146581	3.18622513	3.39989419	3.43573705	3.2954017
NONHSAG032839	3.23477783	2.73454343	2.88355783	2.77359392	2.87365703	3.04236158	3.02713465	2.66892352	2.98943748
NONHSAG032855	4.67712443	4.52456248	4.87876423	4.71710131	4.83409731	4.77613879	4.73971202	4.71928839	4.97978117
NONHSAG032935	7.17719661	7.21322103	6.67652888	6.94947602	6.96515946	6.94149494	7.08903404	7.39704204	7.10801979
NONHSAG032949	4.55332697	4.70225697	4.3057357	4.5617876	4.72741512	4.9156952	4.7007042	4.36973837	4.94016489
NONHSAG032951	3.17232626	3.11092306	3.35576589	3.51797839	2.98027225	3.19217503	3.1522006	3.07860966	3.21347741
NONHSAG032956	6.55222693	6.4048665	6.45861827	6.24740706	6.042469	6.63533966	6.08407364	6.34434071	5.68806671
NONHSAG032959	3.75140792	3.59145665	3.89942019	3.87479276	3.85324764	3.96184791	3.92734607	3.62071335	3.80857398
NONHSAG032960	3.98344091	3.99339258	3.96549204	4.21406022	4.13091253	4.02506571	3.95421304	3.98536976	4.06126035
NONHSAG032961	4.98740339	4.87004964	5.16986423	4.70661899	4.99372174	4.71271464	4.99414092	4.88137563	5.21743268
NONHSAG032979	5.34878506	5.34015858	5.35380807	5.31448408	5.31920643	5.17644843	5.28557536	5.2239373	5.50936297
NONHSAG032984	3.59401326	3.60730534	3.6842452	3.74701373	3.60098467	3.52808418	3.64088796	3.83935773	3.89001259
NONHSAG032988	4.15650394	3.85841986	4.07244356	3.74851697	4.31278787	4.3877295	4.30683464	4.01125907	4.29412805
NONHSAG033011	2.99014602	3.12110632	2.91231646	2.88760802	2.85617071	3.04743704	3.1562794	3.01560832	3.09735348
NONHSAG033026	4.96085708	4.84475726	4.83412469	4.92634942	5.16218845	4.7722805	4.99340914	4.77432932	4.80052738
NONHSAG033060	3.43917312	3.24150552	3.39066162	3.45529714	4.03275019	3.6852335	3.69405479	3.45286601	3.35671922
NONHSAG033068	4.13319285	4.14359727	4.19879993	4.24870944	4.29937293	4.24956097	4.13909008	4.12018748	4.33264294
NONHSAG033071	6.09349514	6.28201528	6.21350897	6.4174943	6.36380324	6.41719997	6.12463069	6.23923142	6.45505961
NONHSAG033082	3.27622276	3.06861337	3.02751481	3.56243064	2.84991181	2.92901088	3.62456223	3.07997225	3.17020558
NONHSAG033092	3.45501224	3.55245179	3.41776967	3.77254182	3.78225836	3.47614583	3.70597519	3.67418977	3.47952284
NONHSAG033099	7.31498125	7.2510998	7.30283589	7.20523343	7.53465723	7.4922267	7.52814617	7.38768104	7.77028568
NONHSAG033167	4.14209435	4.03898565	4.07849235	4.03140309	4.20283388	4.35217082	4.12856353	3.88863878	3.98590117
NONHSAG033226	4.10327661	4.22205896	4.03935023	4.31763403	3.7282758	4.27258228	3.81820002	3.75783287	3.87251683
NONHSAG033228	4.24230134	3.87597919	4.21120581	4.28924713	4.24356585	4.26590968	4.3826587	3.91102836	4.0650901
NONHSAG033247	4.11763071	3.96913099	4.26454594	4.20073119	4.4930459	4.21392493	4.27120015	4.15833595	4.44496524
NONHSAG033254	4.95859767	4.61921384	4.80676108	4.73915281	5.15005331	4.79454027	5.04524894	4.91869677	5.09773725
NONHSAG033260	3.92002667	3.63038121	3.78428916	3.92900466	3.86028194	3.77446148	3.95487948	3.68026574	3.93920438
NONHSAG033274	2.50927261	2.72929453	2.43683846	3.00376049	2.54956514	2.5058831	2.96344275	2.75889952	3.05178401
NONHSAG033291	5.37659231	5.13862713	5.21743268	5.37715718	5.3675094	5.21411677	5.44860485	5.58698433	5.18995604
NONHSAG033405	4.19128097	4.27600917	4.32253814	4.22280209	4.53125528	4.50747909	4.28124161	4.26982838	4.22497182
NONHSAG033416	4.44251188	4.7811744	5.3060107	4.6997967	4.32141426	4.59204478	4.66620636	4.00716121	4.67199546
NONHSAG033484	5.48055268	5.31628875	5.23159446	5.37417758	5.31297095	5.38489954	4.92761002	5.71479797	5.18209626
NONHSAG033545	2.3603755	2.48074659	2.72244386	2.41519229	2.38611999	2.64444413	2.82549679	2.46939353	2.41142791
NONHSAG033550	4.4256887	3.91853919	4.63933215	4.39451581	4.60542077	4.42745388	4.65922606	4.06337208	4.50886729
NONHSAG033553	4.59692519	4.74307155	4.60809702	4.74567332	4.85055241	4.83407783	4.72315221	5.12709356	4.44461518
NONHSAG033558	2.27685044	2.21660171	2.28890029	2.47271935	2.30912652	2.19760993	2.56887778	2.0439527	2.21212171
NONHSAG033585	3.45550414	3.57328544	3.27057866	3.31067867	3.35864798	3.53973414	3.33779797	3.4904149	3.44200526
NONHSAG033587	5.19346494	4.94150788	5.15774143	5.12689008	5.04124187	4.95217908	5.08154177	5.18671963	5.04779449
NONHSAG033588	5.55918168	5.33323979	5.50319209	5.27488657	5.27766173	5.11306555	5.43388313	5.28702711	5.37484966
NONHSAG033592	3.67814718	3.52127037	3.64135785	3.52722586	3.74494361	3.73583038	3.64206548	3.56326488	3.58187479

NONHSAG033593	3.04955169	3.12440632	3.35362234	3.14656796	3.24832737	3.37510645	3.17934876	3.04574916	2.9933771
NONHSAG033604	3.65777852	3.67189163	3.86806145	3.92550947	3.95396294	3.89809406	3.94598036	3.7237491	4.12323051
NONHSAG033616	5.3464326	5.40612159	5.56309139	5.56637786	5.534072	5.36271938	5.48440495	5.43040159	5.74127807
NONHSAG033639	3.86593979	4.09613119	3.88312102	4.19656172	3.9799591	3.88432241	3.85532737	3.98155478	3.89174221
NONHSAG033752	3.73491271	4.04886812	3.91762256	3.95739499	3.99700125	3.94462008	3.93954112	3.86044592	4.07361942
NONHSAG033788	4.30919811	4.27061238	4.33540724	3.98910765	4.25230795	4.23488801	4.13860304	4.1338591	3.98338063
NONHSAG033805	4.62563019	4.32343189	4.69946954	4.76358235	4.94263727	4.52293355	4.49806764	4.27419847	4.41306011
NONHSAG033897	5.16180334	4.89934132	4.98824799	5.05438836	5.04608585	4.91029083	4.93562773	4.59519399	4.92560007
NONHSAG033898	5.70137949	5.59387537	5.5067642	6.01410947	5.84728962	5.77699518	5.69424607	5.62885306	5.78972799
NONHSAG033967	4.81767426	4.68944264	4.91382204	4.70372353	5.08003694	4.96718453	4.90775875	4.75826062	4.93748056
NONHSAG033980	3.60664979	3.47217672	3.74345555	3.538409	3.37154954	3.55870585	3.38791044	3.55281915	3.75747204
NONHSAG033989	5.47392479	5.57292432	5.57240694	5.41183701	4.96570688	5.33353803	5.49645276	5.54823608	5.73665116
NONHSAG034032	3.77361549	3.76436222	3.99578705	3.83767624	3.77405468	3.89537003	3.8451452	3.73922018	3.92413125
NONHSAG034050	5.08118602	5.04412462	4.98114054	5.59596051	5.14581836	5.41361697	4.91019647	4.71331115	4.89667454
NONHSAG034053	5.15123604	5.43839346	5.28955442	5.42666202	5.32829766	5.3758055	5.03574545	5.40967526	5.06065375
NONHSAG034077	5.38478743	5.57067635	5.68627157	5.5923789	5.7072549	5.38079881	5.76970126	5.44907466	5.65296846
NONHSAG034081	3.45573692	3.45772405	3.18986363	3.31537526	3.63477038	3.40461404	3.31199062	3.16658053	3.27681025
NONHSAG034105	3.16331163	3.27368397	3.16053632	3.29678338	3.23154947	3.17461342	2.98552372	3.07997225	3.45753113
NONHSAG034111	4.78843768	4.6388345	5.02258674	4.81385357	5.00771974	4.88313336	4.83904024	5.01176404	5.13356703
NONHSAG034128	2.91068625	3.29498978	3.48662818	3.28183959	3.388705	3.16712663	3.29700603	3.20218899	3.24452655
NONHSAG034158	3.97547082	4.07402293	4.05620222	4.0849802	4.23730883	4.13120099	4.04120169	4.23343945	4.2887625
NONHSAG034163	3.12885536	2.79068095	2.93194811	3.03576304	3.14232676	2.77176863	3.10568139	3.11193552	2.98001181
NONHSAG034175	5.68477542	5.71607199	5.7790603	6.01341666	5.78554177	5.96543111	5.70035688	5.75104943	5.64020663
NONHSAG034179	6.26093512	6.4832995	6.20700417	6.27178389	6.17775204	6.410781	6.10535281	6.3168211	5.91503637
NONHSAG034184	4.12358485	4.23239564	4.27000849	4.02995177	4.36135683	4.20375277	4.12191159	4.24490867	4.47343636
NONHSAG034187	5.11082076	4.92352759	4.9578859	5.07213902	5.06835914	5.28198178	5.04889751	5.04209168	5.0566965
NONHSAG034193	4.45919229	4.14620889	4.37957798	4.581139	4.54810024	4.31402992	4.13427673	4.21252427	4.56493175
NONHSAG034209	6.35006266	5.9778359	6.38128225	6.27950101	6.44651208	6.37330918	6.13403346	6.17807946	6.0465337
NONHSAG034242	5.66004831	5.28727357	5.65068228	5.20689123	5.45603505	5.38327891	5.41845823	5.24302968	6.03269999
NONHSAG034302	7.82315819	7.6661665	7.67223411	7.36618711	7.4706296	7.70583859	7.70359838	7.84398428	7.63012742
NONHSAG034336	2.7663352	2.67858162	2.86087476	2.83515163	3.01809784	2.82714847	2.82137854	2.94194917	3.04702784
NONHSAG034346	2.52232228	2.47480469	2.56968369	2.47480469	2.44571379	2.48402654	2.62619239	2.49304903	2.68625644
NONHSAG034364	3.06782712	3.20282084	3.0831303	3.05925171	3.07784698	3.07456414	3.09238033	3.07821503	3.35048456
NONHSAG034372	7.19831228	7.24645833	7.45787826	7.22962295	7.12568562	7.41883609	7.11809868	7.39120728	7.19794382
NONHSAG034414	4.84840198	4.49695568	4.6315682	4.64421556	4.3929418	4.01582369	4.6730097	4.80931202	4.52848822
NONHSAG034441	4.00459476	4.17278331	4.08065268	4.29649133	4.06222739	4.15673122	3.95573149	4.04711593	4.06222739
NONHSAG034453	4.09729855	4.16942395	4.11730266	3.99528848	3.96350903	4.43543371	4.19988488	4.2426473	4.38674875
NONHSAG034459	5.13832386	4.94123892	4.90227997	5.08503917	5.03127504	5.27758949	5.00104008	4.80840526	4.98176042
NONHSAG034470	3.65253562	3.5177688	3.85535993	3.64277455	3.52304494	3.5930665	3.48594365	3.59682309	3.59533289
NONHSAG034501	5.41363597	5.21134894	5.28050796	5.23089474	5.58391793	5.20164595	5.19292595	5.26360537	5.38345432
NONHSAG034507	2.43829905	2.21708425	2.39640594	2.40811447	2.44822287	2.24916306	2.6368553	2.32537202	2.26105174
NONHSAG034552	4.33527467	4.46545907	4.36505834	4.37065574	4.7187897	4.28817921	4.28945658	4.43279514	4.29939709
NONHSAG034566	5.23730073	4.97713889	5.26060735	5.37284876	5.49291913	5.45458637	5.24456539	5.0439768	5.16658788
NONHSAG034569	2.96693044	2.70446843	3.02751481	2.77278714	2.91134767	2.91096723	2.88766788	2.6815097	3.21134776
NONHSAG034584	3.02129899	2.98808898	2.79550744	2.72248082	3.03007889	3.05503617	2.80909895	2.76661229	2.97332346
NONHSAG034600	3.26218499	3.49640481	3.52673946	3.69118351	3.39388038	3.60950766	3.04098461	3.30899155	3.40679706
NONHSAG034618	4.08937314	3.70772997	4.09305132	3.99134639	4.03943152	4.0781704	4.1224296	3.88619123	4.21943572
NONHSAG034646	2.38648532	2.21582507	2.38943438	2.66360569	2.39090681	2.39775892	2.7094521	2.31128043	2.30547745
NONHSAG034655	2.04444356	2.3704181	2.35214032	2.23237618	2.28293196	2.21676539	2.36180103	2.32233327	2.52054215
NONHSAG034677	3.07314103	2.87668232	3.06988485	3.23870499	3.1259875	3.06456149	3.03230333	3.16228042	2.92410757
NONHSAG034678	2.65669441	2.52750642	2.42809411	2.70201236	2.30633178	2.72558517	2.56272683	2.39775061	2.49662581
NONHSAG034689	4.60284345	4.49751642	4.58136171	4.56135966	4.25788398	4.54282528	4.46645454	4.35471115	4.37900307
NONHSAG034701	3.81114441	3.80952068	3.78360651	3.66253973	3.74129971	3.797596	3.98722635	3.76241322	3.83992893
NONHSAG034702	4.41724853	4.32927986	4.38362881	4.50756865	4.64408259	4.55086481	4.39954948	4.084652	4.35494207
NONHSAG034714	4.44462545	4.48314858	4.56020069	4.72896289	4.89139499	4.41216642	4.29255883	4.34708038	4.86733229
NONHSAG034778	2.82069264	3.05550068	2.86323698	2.8183537	3.00863856	2.79231596	3.07505922	3.32877482	2.57323211
NONHSAG034831	6.73771851	7.22311922	6.68557367	6.93168876	6.87941746	7.00520504	6.68417142	6.94658325	6.07741222
NONHSAG034858	3.23034366	2.98808898	3.19068778	3.28604795	3.21143007	3.56851432	3.5452553	3.04881864	3.16942936
NONHSAG034900	2.66338003	2.98808898	2.62408542	2.84354312	2.83681729	2.57553296	2.59785355	2.79346308	2.95304854
NONHSAG034902	3.01970568	3.09217695	3.06124336	3.09482193	2.98034207	3.19053606	2.96888113	3.18927529	3.41302216
NONHSAG034909	5.16026631	4.92753747	4.95872906	5.26715514	4.95931112	4.99426786	4.89343727	4.96401008	5.04983943
NONHSAG034919	3.29410857	3.5746239	3.54100912	3.4225387	3.51249686	3.27505557	3.52444485	3.56671318	3.54156264
NONHSAG034946	3.95516372	3.6807366	3.82077016	3.71431477	4.12803478	3.65672134	3.99404043	3.88169635	4.12133962
NONHSAG034982	4.81941976	4.81244262	4.6273326	4.56689145	4.7220407	4.62532694	4.48365528	4.74772989	4.64058756
NONHSAG035114	3.5309994	3.86834796	4.07639539	3.94423508	4.22679823	4.0798047	3.91188517	3.78589443	4.06613688
NONHSAG035116	5.54124834	5.23243311	5.33965354	5.37208856	5.61132522	5.460006	5.3267747	5.2099568	5.77661537
NONHSAG035135	2.89720531	2.81157531	2.86788222	3.0781633	2.98537223	3.03907425	3.07127541	3.09696318	2.83744626
NONHSAG035143	6.7174502	6.70769909	6.7923677	6.81539618	6.74992091	6.98848988	6.61678131	6.57588491	6.62055033
NONHSAG035159	5.41982958	5.45047133	5.53738213	4.87000803	5.91574849	5.47161868	5.29615249	5.40851954	6.12716214
NONHSAG035266	2.24267711	2.13977885	2.20822861	2.26381398	2.28528021	2.34579992	2.36335168	2.28399065	2.05730695
NONHSAG035270	4.08076582	4.30995754	4.13608952	4.25635091	4.28366866	4.27858741	4.28332275	4.2309295	4.31579218

NONHSAG035294	3.59310929	3.76860855	3.82684508	3.7745127	4.06795001	3.78158799	3.76949596	3.47404153	3.86964757
NONHSAG035333	3.69547567	3.92355183	3.9387202	3.92489251	3.99045741	3.91872902	3.84348612	3.87170186	3.94434672
NONHSAG035347	4.64649156	4.63288835	4.60525065	4.67294226	4.54205543	4.4122986	4.74451801	4.4559439	4.37165684
NONHSAG035395	2.90332553	2.27698271	2.62192106	2.59724196	2.54168128	3.32699655	3.08595533	3.09849915	2.8195967
NONHSAG035415	4.80060759	4.30108416	4.68539169	4.81916719	4.94630877	4.38201083	4.60220128	4.98437521	4.68401793
NONHSAG035432	4.83721124	4.66523722	5.00527987	4.91776703	5.02028578	5.02255473	4.8942769	4.86157724	5.02041558
NONHSAG035436	2.56600801	2.61768441	2.29805767	2.75237008	2.7448872	2.72244443	2.54519695	2.7674543	2.54334868
NONHSAG035491	3.465495	3.2490834	3.44143066	3.56139259	3.67156008	3.30826912	3.30900858	3.50132194	3.65991909
NONHSAG035503	2.30239559	2.16493431	2.15478786	2.33729215	2.43971907	2.10995882	2.189567	2.14299693	2.02742133
NONHSAG035505	3.43476496	3.10461662	3.5427178	3.30262973	3.68554584	3.25864015	3.25595666	3.38466336	3.66257986
NONHSAG035508	2.49165523	2.2893682	2.35371323	2.42869003	2.44602928	2.29570182	2.27556165	2.29667592	2.37967631
NONHSAG035519	4.425116	4.38572578	4.15790646	4.43801911	4.27890954	4.66412748	4.63593106	4.54176935	4.67819427
NONHSAG035522	6.03116294	6.2467403	5.78016113	5.56544703	5.62034132	5.97072904	5.63641501	5.92865919	5.53085661
NONHSAG035547	4.04314605	4.64466295	4.26889677	4.22013336	4.25582261	4.23892841	4.13472223	4.35254844	3.9241311
NONHSAG035579	2.44035014	2.38468309	2.39988077	2.48820421	2.45581113	2.51649944	2.35275013	2.39996941	2.41196738
NONHSAG035588	4.73076744	4.81825194	4.93378571	5.01009958	5.09458602	5.47440746	4.86639094	5.29082095	5.04820205
NONHSAG035621	3.73312324	3.68768789	3.80273315	3.77261269	3.65061346	3.41794999	3.59710889	3.64137689	3.8030066
NONHSAG035667	3.41212174	3.25966085	3.26694709	3.41471616	3.29600673	3.43483604	3.00201037	3.13941806	3.22252997
NONHSAG035675	3.8464249	3.28888938	3.23710544	2.99807292	3.45490081	3.56157721	3.4271887	4.20827192	2.73930018
NONHSAG035700	2.52001688	2.58927659	2.75084092	2.66790307	2.49358614	2.72232948	2.59568344	2.73528622	2.55080893
NONHSAG035708	2.17791867	2.57000948	2.33605256	2.54454163	2.61579835	2.50205877	2.42768798	2.36355963	2.35881439
NONHSAG035712	2.50447564	2.37607558	2.62872746	2.78913569	2.64937656	2.56424435	2.74578631	2.81037739	2.64937656
NONHSAG035715	2.19962681	2.37607558	2.18645676	2.38996264	2.22805304	2.10840751	2.35653025	2.36885869	2.19586357
NONHSAG035722	4.32484158	4.24715772	4.21628228	4.16046489	4.50556094	4.41441399	4.14794331	4.21250192	4.45386503
NONHSAG035731	2.23575034	2.18883524	1.9419248	2.64737333	2.22853276	2.17799278	2.25237392	2.04357049	2.05231187
NONHSAG035768	6.13284563	6.33146118	6.20989943	6.1430407	6.38966534	6.24047557	6.02002628	6.02120103	5.89556504
NONHSAG035770	6.42433794	6.65409183	6.50402498	6.27554595	6.4803152	6.56093478	6.82074904	6.64145715	7.1569471
NONHSAG035786	3.03914713	2.89660563	2.95426643	3.12235726	2.9238328	3.23083055	3.22023962	3.17515089	3.12432471
NONHSAG035791	3.05456933	3.05522808	3.18787119	3.1598614	3.04005516	3.2661051	3.27501052	3.60957619	3.44503768
NONHSAG035807	2.85747891	2.4379203	2.64183308	2.40478818	2.83576113	2.23861641	2.70261902	2.48432227	2.61548401
NONHSAG035911	10.08812062	10.04930529	10.11917739	9.82423659	10.03619627	10.00371296	10.02712516	9.9216302	9.88757068
NONHSAG035915	2.61842974	2.28774649	2.37630743	2.52808174	2.39571676	2.32350507	2.2273268	2.37479378	2.467088
NONHSAG035927	2.73596558	2.6247233	2.82463834	2.64566315	2.77542621	2.69201871	2.67598892	2.76374739	2.6952466
NONHSAG035942	2.54077443	2.71139478	2.73691787	2.57935405	2.64937656	2.55414771	2.53174141	2.59181266	2.87797878
NONHSAG035943	5.56398951	5.28428999	5.61685137	5.29228296	5.694292	5.45215028	5.32920948	5.25743271	5.61110145
NONHSAG035971	4.94578782	4.77681118	4.77163383	4.71394911	4.58956931	4.44376966	4.77481254	4.44587608	4.74571386
NONHSAG035976	5.59601398	5.44964878	5.43477662	5.94464318	5.30761508	5.63853762	5.30542946	5.41393094	5.62822776
NONHSAG035982	5.3788965	5.38717997	5.33255443	5.4240872	5.35803377	5.33465732	5.38964969	5.38317283	5.23825049
NONHSAG036030	3.02406422	3.52446511	3.25877489	3.29193104	3.18302228	3.1053004	3.32906245	3.31439253	3.32076472
NONHSAG036052	6.0804731	6.26688673	5.87897897	5.84912137	5.97720279	6.34674634	5.99618829	6.07914661	6.02435624
NONHSAG036065	2.41321398	2.3493797	2.5081571	2.61830494	2.17805242	2.58052089	2.31143225	2.40170258	2.47119327
NONHSAG036067	8.28987723	8.77089547	8.28626393	8.31298007	8.01010323	8.2460189	8.19172861	8.35069563	8.13250276
NONHSAG036098	4.95046797	5.24490037	4.9894863	5.04215505	5.12455036	4.8749863	4.79444098	4.93442796	4.98020129
NONHSAG036130	3.67275794	3.84671482	3.77759486	3.99403468	3.6928538	4.12839943	3.14899678	3.96330037	3.69532947
NONHSAG036141	5.64187787	5.39070496	5.39554111	5.69406595	5.60448278	5.45640818	5.65632196	5.45002315	5.48028448
NONHSAG036146	5.35115164	5.51025765	5.46706379	5.44289014	5.72028952	5.40110205	5.36029878	5.71059195	5.46269837
NONHSAG036147	4.48203644	4.38857289	4.45721556	4.49654608	4.61481599	4.5754648	4.31256163	4.60899376	4.56705662
NONHSAG036184	3.04749053	3.22674425	3.38291457	3.27950266	3.46816382	3.58443332	3.43497279	2.93919411	3.21440214
NONHSAG036217	4.49679507	4.39443159	4.46430087	4.52164688	4.30342725	4.36340814	4.46144074	4.5734033	4.40616172
NONHSAG036227	7.587669	8.07171436	7.8514622	7.76704098	7.72769059	8.23195672	7.70866911	7.89905787	7.41357034
NONHSAG036242	3.34804599	2.93519359	3.21427158	3.34541933	3.36210844	3.46221821	3.17440057	3.07997225	3.49243852
NONHSAG036289	3.12540095	2.85340826	3.03049689	3.21390262	3.09358344	3.24450729	3.32744498	3.26110053	3.41770965
NONHSAG036290	2.56965547	2.52053771	2.26097206	2.55815712	2.23796461	2.46018376	2.55505048	2.37876574	2.26198868
NONHSAG036298	3.88358564	3.78430316	3.742428	4.01890973	3.82601821	4.02289934	3.94622204	3.59713629	3.9197134
NONHSAG036307	2.88069903	2.70639625	2.77111943	2.76407108	2.85203425	2.80540629	2.77230365	2.9044472	2.93524737
NONHSAG036327	2.9159225	3.19350067	3.16298475	3.3660202	3.22921318	3.15930747	3.19832119	2.98984888	3.35832276
NONHSAG036374	3.61957215	3.30966436	3.42014695	3.48287358	3.23880182	3.60180335	3.50038996	3.65858419	3.51961163
NONHSAG036383	6.58368232	6.13371482	6.33992452	6.24200563	6.47225306	6.10076335	6.32854374	6.20152358	6.53205372
NONHSAG036413	2.29078101	2.45780198	2.27872283	2.37356463	2.44172017	2.54174392	2.48346711	2.22693802	2.22364671
NONHSAG036444	2.70510602	2.69914894	2.82802753	2.71957815	2.65239664	2.75269429	2.54791871	2.6122813	2.97332346
NONHSAG036455	2.59563669	2.54945218	2.58595543	2.40478818	2.79721951	2.29689303	2.46692903	2.52112783	2.68415901
NONHSAG036462	3.48944458	3.3571277	3.67764633	3.72749823	3.70856066	3.47902296	3.65490468	3.46182234	3.58912158
NONHSAG036486	2.64911562	2.51988431	2.29689303	2.55713951	2.55898233	2.58461703	2.43904531	2.62722838	2.63475828
NONHSAG036538	3.24790909	3.34719888	3.3331454	3.43880706	3.2108098	3.54363621	3.39716073	3.46127072	3.51283804
NONHSAG036539	6.30090476	6.52448181	6.10430082	6.12994438	6.25504867	6.33528464	6.30280923	6.356838	6.18989294
NONHSAG036566	2.90432113	2.84379365	2.98868461	3.02602861	2.75953463	2.86828355	3.10315152	3.17679286	2.88969608
NONHSAG036587	3.6053686	4.06505635	3.3560818	3.65291842	3.56668933	3.54249975	3.60498382	3.48682538	3.04861354
NONHSAG036631	5.57251262	5.63377055	5.5067642	5.46376887	5.475551	5.57731936	5.64304624	5.42779703	5.65632196
NONHSAG036645	2.25635085	2.41819787	2.34967666	2.33024051	2.40047215	2.13841638	2.26161342	2.15622073	2.23199083
NONHSAG036667	2.77556068	2.69041285	2.81209363	2.52492255	2.76656511	2.36954055	2.58036498	2.66271347	2.72623241
NONHSAG036669	3.49854498	3.50530877	3.60254134	3.38512384	3.45578938	3.61950773	3.59699424	3.70942536	3.51581552

NONHSAG036696	2.54615241	2.6585952	2.9140252	2.58472748	2.72511602	2.56509713	2.70879863	3.03479597	3.06944228
NONHSAG036703	2.42944848	2.38295267	2.20125758	2.17383605	2.21800365	2.3747515	2.24094697	2.24472008	2.20998221
NONHSAG036710	2.62087909	2.85095809	2.98563112	3.04721314	3.00138231	2.86514992	2.64185036	3.10220012	2.77479009
NONHSAG036741	1.87169474	1.83432135	2.04305062	2.03351843	1.8852537	2.07546115	2.18287166	2.03351843	1.98472074
NONHSAG036745	4.09613687	3.94038674	3.91917456	3.91588709	4.30151146	3.73951661	3.99497712	3.96289827	4.15863536
NONHSAG036767	2.62864122	2.67820323	2.69522555	2.98481328	2.87482537	2.62088555	2.80431939	2.74680697	2.57993912
NONHSAG036776	3.4895573	3.45404926	3.64229127	3.57836535	3.70902175	3.39054309	3.41185685	3.4535339	3.45158604
NONHSAG036803	2.98130939	2.9583024	3.12053756	3.15106147	3.43218343	2.96868029	3.23171411	3.15895024	3.54568199
NONHSAG036808	4.28161116	4.41322174	4.27233381	4.14102912	4.20900663	4.15037012	4.1749586	4.20767403	3.99096306
NONHSAG036869	4.31561182	4.40597426	4.58307812	4.33044898	4.22391918	4.29206744	4.79332376	4.31679167	4.36850821
NONHSAG036874	3.1738573	3.25213197	3.20473642	3.12524047	3.16546846	3.42954928	3.28292155	3.32358059	3.14406342
NONHSAG036892	2.73918698	2.59033231	2.71502032	2.79304109	2.75943057	2.65866758	2.71437327	2.70359255	2.86274296
NONHSAG036926	6.49531352	6.23217656	6.57693298	6.10125068	6.55152395	6.46817438	6.52512575	6.30553766	6.56097429
NONHSAG036955	3.25847989	3.30925691	3.24999689	3.43130524	3.48306235	3.08609665	3.30449721	3.22838732	3.35285431
NONHSAG037015	3.35440083	3.37891266	3.57188886	3.42575146	3.6960163	3.35789236	3.39078085	3.39763832	3.81228994
NONHSAG037017	4.6398492	4.80730007	4.610576	4.66221977	4.5034325	4.54786626	4.47048935	4.79654272	4.48752129
NONHSAG037020	3.18838925	3.24458599	3.38404636	3.67145796	3.74410556	3.41842639	3.58951015	3.30353365	3.21786232
NONHSAG037021	2.54217904	2.39694837	2.5933512	2.74415386	2.4035413	2.56005501	2.35652891	2.61750068	2.58553112
NONHSAG037022	7.29370629	6.96721509	7.07072837	7.36981992	7.00000582	7.37091906	7.07789624	7.03912073	7.05454699
NONHSAG037025	2.72488526	2.42561967	2.71820026	2.82651684	2.80653162	2.99011496	2.67504023	2.54574216	2.63283881
NONHSAG037030	6.31940114	6.27776487	6.32468781	6.57607202	6.19367604	6.39544643	6.32604242	6.32961608	6.56373623
NONHSAG037031	5.31453229	5.21846188	5.52392531	5.39604037	5.34070208	5.51266821	5.24076797	5.26109958	5.42033901
NONHSAG037032	3.27686059	3.11200664	3.27286975	3.22054949	3.52772046	3.30929808	3.38232068	3.18972731	3.09279665
NONHSAG037083	3.39145355	3.34188269	3.59246726	3.31159286	3.6089927	3.4743358	3.63042899	3.06069368	3.6978946
NONHSAG037087	7.60090788	7.62692956	7.71251773	7.83896546	7.75579186	7.8399889	7.62304714	7.53722552	7.69837235
NONHSAG037150	4.56355697	4.53004215	4.6978124	5.01003558	4.96360507	4.5833506	4.38422233	4.61887937	4.62087617
NONHSAG037173	7.43650955	7.80773764	7.39970033	7.59517893	7.46755645	7.42565675	7.52502257	7.7313564	7.27053376
NONHSAG037197	4.6625143	4.93235596	5.08418441	5.27478816	4.9228056	5.24241818	4.04642127	3.67482449	4.33911074
NONHSAG037200	5.7657605	5.62961023	5.41945771	5.66579151	5.7786797	5.60755211	5.91706626	5.88491604	5.70680454
NONHSAG037224	6.95641794	6.47553156	6.93652034	6.93027974	6.81635031	6.96309836	7.1027645	6.86959055	6.99503828
NONHSAG037227	5.50554202	5.41154675	5.64905126	5.91573092	5.47736456	5.43209437	5.57873711	5.67532818	5.55257101
NONHSAG037229	5.17932436	5.25566317	5.30804997	5.27339406	5.2757357	5.11490536	5.18589186	5.22150432	5.3062186
NONHSAG037251	2.52014202	2.50966521	2.40300478	2.75579962	2.62223685	2.54277235	2.49026803	2.99497329	2.69301677
NONHSAG037262	6.07919926	5.83164748	6.12744821	6.13168858	6.1846229	6.21724072	6.12746903	6.05557787	6.275549
NONHSAG037272	4.58922872	4.58472141	4.77729441	4.47337668	4.83127664	4.3090876	4.47266255	4.47724043	4.66570239
NONHSAG037287	4.83407783	4.58436738	4.70553781	4.68032985	4.75235567	4.97536083	4.89429618	4.7447408	4.69208126
NONHSAG037322	2.82340723	2.49459952	2.19298641	2.30696758	2.44791886	2.45200797	2.32908254	2.59014464	2.48412724
NONHSAG037336	3.51209104	3.74931366	3.72337332	3.63315599	3.51530681	3.70075082	3.53395984	3.65754725	3.95930543
NONHSAG037338	5.20384381	5.05119359	5.31799802	5.25609223	5.35805248	5.12556353	5.16617644	5.20013858	5.6512602
NONHSAG037352	5.37020985	5.38615697	5.30600974	5.42797095	5.49537352	5.36479139	5.49066047	5.24835413	5.52687186
NONHSAG037375	3.10226081	2.97860051	3.23607446	2.78153316	3.05998447	3.32076472	3.39843537	3.15440677	3.48369779
NONHSAG037376	3.94051264	3.73588443	3.46593758	3.85140593	3.80192403	3.51122592	3.56904646	3.51344359	3.51243898
NONHSAG037395	7.23994351	7.11628025	6.96236013	6.95554812	6.96987848	7.21256817	7.35406562	7.30612388	7.08225598
NONHSAG037396	5.91289197	5.83101032	5.86303082	5.83752695	5.89576317	5.82856075	5.81229687	5.85667801	5.86528514
NONHSAG037407	4.83857175	4.80735226	4.89937288	5.11096481	4.43429307	4.65681424	4.78530353	4.5458524	4.48818422
NONHSAG037426	4.7871109	4.84654782	4.91911453	4.90038952	4.73345882	4.70164842	4.8511912	4.44989119	4.79468701
NONHSAG037431	4.66031513	4.62128782	4.86786965	4.53497699	4.87227668	4.66540199	4.45519535	4.67317819	4.84585373
NONHSAG037432	6.15722147	6.29060679	5.74229024	5.92493573	6.19635082	5.95885467	6.39539697	6.47175923	6.39323378
NONHSAG037433	4.40784722	4.42671313	4.44187034	4.78115601	4.48169823	4.37249315	4.64639126	4.59045685	4.36377315
NONHSAG037464	3.88576936	3.82883483	3.65019668	4.52460922	4.21572998	3.89009517	3.83488056	3.88429956	3.91222188
NONHSAG037470	3.79996836	3.91537255	3.86628699	3.71715545	3.72002794	3.90447567	3.82400474	3.74807666	3.81032506
NONHSAG037504	3.21308583	3.22255651	3.40410175	3.58250755	3.3886621	3.72566723	3.41285386	3.41889597	3.26958592
NONHSAG037513	3.38796412	3.24744509	3.27097314	3.44685919	3.06729806	3.78635707	3.39052491	3.26838934	3.31053609
NONHSAG037525	3.98311523	4.10832785	3.73423763	4.1405628	3.63220981	3.87274026	3.93058015	3.7210216	3.88539109
NONHSAG037563	4.66047369	4.69244315	4.63890549	5.01728495	4.94400744	4.88172152	4.72380011	4.80038256	4.95970169
NONHSAG037570	3.48750101	3.54695638	3.66926349	3.50288523	3.66378372	3.52433124	3.55046506	3.55973272	3.54994894
NONHSAG037575	5.28428999	5.31450333	5.3695023	5.6305485	5.45068196	5.59212951	5.4580727	5.2920109	5.16715147
NONHSAG037577	3.71031001	3.88080456	3.93977368	3.69056636	3.67192217	3.583405	3.65072585	3.2707752	3.87111621
NONHSAG037622	3.29499263	3.23197172	3.3657559	3.59878384	3.2851172	3.42108297	3.49010198	3.2494948	3.08209859
NONHSAG037629	4.60083537	5.2225893	4.17514871	4.32327114	4.52793451	4.62287003	4.12582227	4.65113359	4.42423396
NONHSAG037701	3.25978348	3.44298381	3.3724707	3.2606893	3.51510753	3.49940315	3.33045822	3.50504517	3.50643271
NONHSAG037719	2.7063503	2.49790028	2.71554339	2.96555505	2.91662426	2.67046008	2.84070326	3.0635176	2.87692559
NONHSAG037724	2.31463997	2.36132605	2.22377165	2.49344269	2.2270392	2.23217905	2.19383249	2.45605048	2.27296884
NONHSAG037726	2.28567774	2.32784565	2.25312682	2.32120518	2.26539654	2.25827353	2.24461314	2.11102248	2.25848082
NONHSAG037747	3.94628391	3.70008375	4.04705965	3.93698475	3.93295823	3.96675194	3.88684404	3.90727868	3.81404888
NONHSAG037751	4.1351249	4.11923849	4.09081597	4.4016125	4.11292152	4.2524141	4.04830885	4.22681468	4.36341924
NONHSAG037786	7.92330523	7.9262402	7.69633102	7.8071838	7.85276992	7.77731463	7.97543949	7.9848907	7.8428327
NONHSAG037791	2.53118973	2.38854564	2.46616326	2.39631225	2.33451558	2.40943834	2.52075432	2.37107494	2.65320588
NONHSAG037804	3.85409228	3.98775458	3.68458618	3.77412829	4.13407144	3.94678475	3.95026438	3.55837656	4.23180394
NONHSAG037839	5.57336785	5.68277606	5.2228941	5.46379299	5.62073768	5.50723843	5.49611925	5.58970009	5.33843206
NONHSAG037842	3.07360095	3.22047081	3.34347658	3.09719877	3.4716141	3.3186502	3.11922536	3.37271291	3.32651618

NONHSAG037849	3.40324456	3.16242712	3.27459432	3.21208101	3.18971347	3.18537836	3.381772	2.96819478	3.53400352
NONHSAG037860	3.67588406	3.55382461	3.83458861	3.13401112	3.25697008	3.67016757	3.52062621	3.45726397	3.7405978
NONHSAG037870	2.3619195	2.29529872	2.31578938	2.40261737	2.34062994	2.3465855	2.2764817	2.12342025	2.25884315
NONHSAG037873	2.39969013	2.39895202	2.24473826	2.21075807	2.41925303	2.35390069	2.191947	2.27690076	2.33091837
NONHSAG037878	2.35347284	2.40464556	2.414187	2.46179841	2.37784241	2.43187679	2.38421551	2.36870554	2.48877449
NONHSAG037943	3.01683962	2.85763922	2.96589794	3.01567608	3.01724205	3.05833126	3.00745549	3.04674212	2.98333731
NONHSAG037955	2.09768276	2.31135026	2.19961553	2.17392472	2.21934728	2.12946573	2.08394834	2.29130514	2.22363673
NONHSAG037959	2.93878709	3.42656846	3.2615402	3.4708115	2.76071462	3.55944221	3.12725172	3.33392785	3.09613153
NONHSAG037962	3.83225112	4.25094428	4.04990373	4.33044898	4.1647022	4.21795094	4.154651	4.13981701	4.17717501
NONHSAG037977	6.81485607	6.72360691	6.60190223	7.01735907	6.76863228	7.10526088	7.0116793	7.03469287	7.28809112
NONHSAG037987	2.44677766	2.50786485	2.52589892	2.59527823	2.52995253	2.42703947	2.26290098	2.27275496	2.55619643
NONHSAG037991	4.04899824	3.8016612	4.00253193	4.43730302	4.41629641	3.85817023	3.94437367	3.9413623	3.89005025
NONHSAG038027	3.59765676	3.33850568	3.47213538	3.57799081	3.99251358	3.40877766	3.74864963	3.62610225	3.96970534
NONHSAG038035	2.18287925	2.21134047	2.10004704	2.25467521	2.25417004	2.41655269	2.22213848	2.386875	2.18263664
NONHSAG038048	2.42224036	2.48043831	2.46342109	2.45027684	2.51820201	2.38447709	2.39169304	2.4155478	2.25940364
NONHSAG038071	4.10498295	4.05363789	4.12001564	4.11919607	4.36378445	4.16855708	4.2966783	4.06446863	3.93316847
NONHSAG038091	3.39970625	3.3642677	3.55289299	3.66602538	3.4223955	3.54867152	3.41875258	3.6363477	3.33861141
NONHSAG038132	4.75759498	4.73527255	4.83142766	4.60301304	4.91505161	4.71312442	4.83904024	4.6730855	4.99533041
NONHSAG038179	3.42412855	3.38967484	3.9773471	3.48790627	3.7603507	3.2675589	3.78669888	3.46729781	3.67714386
NONHSAG038210	3.90914406	4.07356899	4.31936131	4.19085548	4.40716875	4.3280447	4.17577113	4.24522734	4.49815963
NONHSAG038213	2.77915054	2.90393232	2.79118455	2.74749208	2.76570135	3.14219517	3.09534746	3.03694445	2.83728639
NONHSAG038259	3.2601551	3.11188929	3.36965828	3.4665324	3.41712121	3.20851221	3.378493	3.3410175	3.33153623
NONHSAG038285	3.47003267	3.58782117	3.42385119	3.40205543	3.4564072	3.61722164	3.61095829	3.66985878	3.55615235
NONHSAG038292	2.39682413	2.27864707	2.36311316	2.34506505	2.43641762	2.49335362	2.35178952	2.50558346	2.38280634
NONHSAG038333	5.83704035	5.51743539	5.50805454	5.63548682	5.71974578	5.32081236	5.62868496	5.50040479	5.54455067
NONHSAG038350	3.2874109	3.12554833	3.32877069	3.03746678	3.42898096	3.46898373	3.39052491	3.13449582	3.22155806
NONHSAG038364	2.98521928	3.24482083	3.34039208	3.23677457	3.21866416	3.4234583	3.80306487	3.26511914	3.29332255
NONHSAG038417	6.69134316	6.23361064	5.96418494	6.54392746	6.63041062	6.89893582	7.05424706	7.21981887	6.83713584
NONHSAG038444	2.63874477	2.56982605	2.96523024	2.94046247	2.76930597	2.83313069	2.75048312	2.89507993	2.80959805
NONHSAG038460	10.07445386	10.03087159	10.03610441	9.83134688	10.03123082	9.96324157	10.11799001	10.07164182	10.03265572
NONHSAG038476	3.55800647	3.83975798	3.7030315	3.85204852	3.94190842	4.09046216	3.6328282	3.32662332	3.72651868
NONHSAG038511	2.35677302	2.38202716	2.28005627	2.47929897	2.64263891	2.42899339	2.44525145	2.48792558	2.37136139
NONHSAG038512	4.33873235	4.22785845	3.9663267	4.49051719	4.41811797	4.66054441	4.48271909	4.35285333	4.48969965
NONHSAG038522	5.64792492	5.59770083	5.6083449	5.85617033	5.69206444	5.62831909	5.39296388	5.65630818	5.89589238
NONHSAG038586	5.80922087	6.54056139	6.06749322	6.01396244	5.74861371	5.77699518	5.60715182	5.71582167	5.58386233
NONHSAG038591	2.57157641	2.50323881	2.55239373	2.54807891	2.58245636	2.51209964	2.69989256	2.73026398	2.50760933
NONHSAG038599	3.07911253	2.94020727	3.06442595	3.38423354	3.53511608	3.26651385	3.23057164	3.25004038	3.27356781
NONHSAG038627	6.40039078	6.69581545	6.25377447	6.71104307	6.46724284	6.64050439	6.33092613	6.33880598	5.82677482
NONHSAG038649	3.96655784	3.94935523	3.95807045	3.92840139	3.74550021	3.97286182	4.01048714	3.7473732	3.54378563
NONHSAG038724	2.72495091	2.55542469	2.61501003	2.45805257	2.72710609	2.62268313	2.70760641	2.17356951	2.67563544
NONHSAG038765	4.16734019	4.22255633	4.26178695	3.98313085	4.07464991	3.78523462	4.01844826	4.1399761	4.44740156
NONHSAG038779	3.35074869	3.31378804	3.5763547	3.31631467	3.61794923	3.30501613	3.04324879	3.74550342	3.28484465
NONHSAG038780	2.19984166	2.17951091	2.27722948	2.29133894	2.17326221	2.14996885	2.20790607	2.160901	2.06762503
NONHSAG038818	2.52900182	2.19386644	2.33372134	2.25589064	2.22325618	2.42661188	2.23126278	2.15006023	2.22941451
NONHSAG038845	5.52698007	5.89082342	5.75365066	5.42614922	5.71994152	5.74058465	5.82862182	5.74492159	5.13005414
NONHSAG038869	1.99810781	2.06368428	2.38469876	2.20605249	2.43019559	2.36435738	2.10297581	2.38000659	2.11070301
NONHSAG038874	2.58827202	2.54676542	2.53458383	2.32268168	2.48528011	2.43471409	2.61303283	2.62672441	2.62091536
NONHSAG038882	2.29464858	2.47017718	2.33282171	2.56917767	2.43471409	2.38735488	2.29803957	2.47966957	2.40448941
NONHSAG038890	6.19914705	6.24574237	6.01428441	5.89678789	6.07326004	6.22063521	6.26548598	6.14281031	6.00223895
NONHSAG038899	2.63820087	2.6994588	2.72360028	2.62465628	2.78178297	2.75460753	2.81689313	2.48299776	2.5731153
NONHSAG038901	4.06343144	4.01624534	4.0363669	3.54294659	3.68127841	3.84815698	3.75895216	3.67201492	3.92394345
NONHSAG038916	2.42919114	2.44591502	2.2817272	2.58330095	2.36651606	2.23103851	2.28085107	2.37665532	2.36892301
NONHSAG038917	2.35402521	2.29641889	2.42192047	2.55784544	2.42941459	2.43135854	2.40034572	2.7348408	2.71250778
NONHSAG038920	3.74573515	3.89786994	3.78800527	3.80175	3.6585356	3.87865113	3.74409075	3.6068239	3.69631471
NONHSAG038949	4.92649082	4.94158686	4.97038885	5.02025358	4.98051911	5.22458062	5.13290215	5.00572973	5.10656561
NONHSAG038955	2.70609412	2.85357419	2.92577796	2.98740106	2.87293359	2.81795825	2.8788131	2.9573645	2.78112069
NONHSAG038958	2.73099357	2.46747189	2.93826082	2.80374191	2.77333645	2.67373308	2.6218584	2.77533075	2.96138579
NONHSAG038962	3.52727807	3.43439929	3.40112619	3.64056277	3.4625708	3.52493059	3.57508165	3.61013118	3.5163694
NONHSAG038974	4.17929527	3.87574382	4.15525395	4.33044898	4.15109435	4.06290284	4.30561119	4.25753128	4.29814763
NONHSAG038975	3.51985585	3.29486263	3.60519888	3.56208784	3.68054515	3.4243637	3.54146312	3.59027469	3.56570818
NONHSAG038999	2.31090334	2.27228398	2.51257015	2.36051637	2.43318537	2.56424435	2.43712876	2.23145821	2.28915571
NONHSAG039007	2.1405686	2.22079467	2.44813063	2.82948853	2.19373247	2.34257168	2.23116955	2.16733969	2.23683038
NONHSAG039009	2.09165168	2.24224154	2.27966755	2.12699922	2.41502181	2.12915322	2.23267013	1.99810781	2.37604979
NONHSAG039017	3.02254632	3.05740382	3.29997798	3.01771732	3.29875872	3.11918557	3.00811027	2.92819048	3.11828171
NONHSAG039019	2.93632899	2.95711002	2.9092023	3.0565205	3.33168124	3.04666205	3.50236863	3.16703781	3.29678385
NONHSAG039034	3.42644042	3.08131583	3.76344944	3.43058439	3.23940608	3.64501997	3.37596902	3.70160764	3.55783579
NONHSAG039040	2.85442002	2.79102781	2.64222945	2.99678879	2.97907592	2.6073441	2.63272696	2.86639489	3.09915379
NONHSAG039046	3.35370888	3.45381781	3.21452165	3.48903254	3.47797596	3.20905118	3.36630719	3.12764369	3.18605203
NONHSAG039047	2.71175191	3.12698874	2.76616278	2.92456928	2.95739286	2.68283447	2.65197728	2.79510346	2.70963922
NONHSAG039060	3.03230757	2.92877408	3.2609136	3.05813987	2.99485206	3.17826132	3.04463212	2.9553438	2.92464901
NONHSAG039061	2.85455395	2.87702621	2.99815545	3.1386409	2.81933902	2.67652543	2.80972467	2.95679985	2.90967465

NONHSAG039063	3.02264367	2.980425	3.13777498	2.93500308	3.04110364	2.95340355	2.9852717	3.14650163	3.04283773
NONHSAG039082	4.21522018	3.88738882	4.03935023	4.1623147	4.52795527	4.58052341	4.25762649	4.48403771	4.26166778
NONHSAG039102	3.51452452	3.34481028	3.3360405	3.35334273	3.52117305	3.60084353	3.5027356	3.32992629	3.44458165
NONHSAG039105	4.90177801	5.33720698	5.03126416	4.99628597	4.88470851	5.09593291	4.81651174	5.0436889	4.68033096
NONHSAG039107	5.69439395	5.72704025	5.88138097	5.65938189	5.89775755	5.710232	5.75312326	5.8090939	5.79485658
NONHSAG039111	3.25885686	2.81106585	2.90950263	3.28728824	3.07039404	3.02134853	3.051786	3.23840765	2.91017255
NONHSAG039112	4.5936883	4.46911401	4.80481566	4.88820184	5.01684226	4.63386851	4.71378649	4.53593337	4.82755314
NONHSAG039130	3.25150721	3.3764061	3.7568422	3.37756173	3.27278	3.3040868	3.49665846	3.13633893	3.70245393
NONHSAG039135	4.59692519	4.54397637	4.76490884	4.67674969	4.68241548	4.7037831	4.7980212	4.58607966	4.78484192
NONHSAG039189	2.04871751	2.14175055	2.20060806	2.20154639	2.16391653	2.36267658	2.25209021	2.13543377	2.17778624
NONHSAG039190	3.28410277	3.38212413	3.91655207	3.4912759	3.48760763	3.33628029	3.43599332	3.67443057	3.71612138
NONHSAG039191	2.58827202	2.68750814	2.92220257	2.55403711	2.62064989	2.74741233	2.71255424	2.45899226	2.50395391
NONHSAG039225	3.57099819	3.46001797	3.70374255	3.46577225	3.54897395	3.62835151	3.66501712	3.57710114	3.52552005
NONHSAG039294	2.6393161	2.62735817	2.52608036	2.8141644	2.65391226	2.70022846	2.71176516	2.61104356	2.91401172
NONHSAG039315	4.37480961	4.39817228	4.32998033	4.34399751	4.37011549	4.50274577	4.36938654	4.62336972	4.71125454
NONHSAG039321	2.57474232	2.54438722	3.02751481	2.7320522	2.80261065	2.71315349	2.98405717	2.74664325	2.7249921
NONHSAG039327	3.5745547	3.60693985	3.70258214	3.68024527	3.68552116	3.43215142	3.63541611	3.65126869	3.86955185
NONHSAG039332	2.80247714	2.83425192	2.91851396	2.82220674	3.02491908	2.87707941	2.78890884	2.95054915	2.7524059
NONHSAG039337	2.82184123	2.90256581	3.24694883	2.58841043	2.74198524	3.08911726	2.63993195	2.97220854	2.57724485
NONHSAG039356	2.86283998	2.75755952	2.69196712	2.75505187	2.66234492	2.73787649	2.85335528	2.76087691	2.72466279
NONHSAG039358	4.25233616	4.19130161	4.3799529	4.47875681	4.31831837	4.35853775	4.32623637	4.18893281	4.42083926
NONHSAG039367	3.07422364	3.17461342	3.1015262	3.2487359	3.41356524	3.55239455	3.57128617	3.43565152	3.20323399
NONHSAG039377	4.12060081	3.7869093	3.79703373	3.74863648	4.24783419	4.12493275	4.18356037	3.95637409	4.38967259
NONHSAG039399	5.60689631	5.59801639	5.1720538	5.48550083	5.52610344	5.68236943	5.59117113	5.61854742	5.48978273
NONHSAG039414	2.75457851	2.98918984	2.9759699	2.90363771	2.80707001	3.0891144	2.97012242	3.12389661	2.74301734
NONHSAG039470	4.82502244	4.7618508	4.64598311	4.47691562	4.76583135	4.67706346	4.75311935	4.76363749	4.91268606
NONHSAG039489	2.79275679	2.72752575	2.8417443	2.74667542	2.79713959	2.78767781	2.6917109	2.92983009	2.92407481
NONHSAG039490	4.66449863	4.80670405	4.52202484	4.51837722	4.19756976	4.40268914	3.94698518	4.07085531	4.41279302
NONHSAG039502	4.33309535	3.89779447	4.04785932	4.40661684	4.35113224	4.44354575	4.68125792	4.21503561	4.29483996
NONHSAG039503	5.37292012	5.18584221	5.37339991	5.23294721	5.60256222	5.48088601	5.39878095	5.65530562	5.39281633
NONHSAG039511	3.85340922	3.63162367	3.84013967	3.70698404	3.68079631	3.53661811	3.80091049	3.62239323	3.63377233
NONHSAG039515	3.03830155	3.58880397	3.10750467	3.573939	3.31433475	3.27415624	3.29454669	3.14119446	3.14788975
NONHSAG039551	4.94698771	4.86782129	4.91573511	5.31149987	4.7647035	4.96375604	4.89330776	4.8824839	4.79758884
NONHSAG039586	2.15967096	2.32175452	2.1977094	2.20250087	2.33245928	2.47456832	2.02457075	2.36024288	2.32172661
NONHSAG039670	4.8332985	4.69345343	4.55994251	5.07644132	5.11603894	5.02418351	4.62528848	4.92637134	4.8915951
NONHSAG039676	9.0131664	9.10403844	8.96535584	9.01222975	8.8102627	8.8918864	8.94126999	8.90914049	8.90759253
NONHSAG039681	3.11176199	3.04585675	2.92610606	2.97917177	3.00928421	3.09102418	2.91945543	2.88237021	2.86604341
NONHSAG039701	5.03423064	4.6181445	4.92031369	4.79900165	4.78028935	5.05098075	4.90349412	4.79110258	5.01344979
NONHSAG039715	2.82535338	2.67166698	3.09346	2.93499347	3.01494305	3.04608242	2.66028867	3.10922805	2.78275286
NONHSAG039740	2.54652366	2.19106209	2.56197472	2.5334134	2.75714288	2.53767984	2.42646524	2.38255564	2.56070386
NONHSAG039770	2.72564179	2.51467444	2.8834347	2.7269566	2.80959805	2.7165721	2.59368562	2.75290782	2.90063714
NONHSAG039813	2.6428133	2.80571603	2.5125279	3.15536981	2.86435239	3.32076472	2.55635273	2.80971538	2.92058999
NONHSAG039828	3.0778571	2.80351667	3.04575538	3.70002533	2.79570335	3.77868869	3.10644552	3.01688111	2.5875569
NONHSAG039846	2.79560465	2.55288786	2.7190864	3.12031519	2.74325584	2.64129772	2.67483806	2.61758902	2.76478851
NONHSAG039857	3.87444937	3.85506318	4.02838848	3.93012152	3.82281847	3.83508171	3.82433703	3.76613394	3.87965351
NONHSAG039863	6.88375232	6.65925959	6.82345794	6.4924802	6.61269197	6.64050856	6.77273353	6.63051235	6.77981425
NONHSAG039875	6.65874309	6.68000203	6.597229	6.7914987	6.71130736	6.57340254	6.52380363	6.68297598	6.87078205
NONHSAG039889	3.73744233	3.54462345	3.67889632	3.62820989	3.6988185	3.96565286	3.893914	3.39210431	4.107169
NONHSAG039949	2.98564934	2.90931273	2.93172537	3.10053157	3.0146458	2.96065625	3.08595533	3.11110998	3.09391604
NONHSAG039968	2.24486422	2.16341674	2.09984056	2.00663531	2.04812047	2.36435738	2.20060806	2.34622487	2.19688448
NONHSAG039971	2.9181012	3.10096567	3.28412618	3.21567809	3.2703624	3.25098072	3.16904021	3.21309343	3.34304895
NONHSAG039976	2.4615223	2.6388696	2.51842536	2.57520549	2.53927112	2.52376637	2.49163008	2.64874692	2.66697586
NONHSAG039991	4.26325362	4.36338392	4.22444019	4.19923734	4.46893189	4.39224356	4.17710271	4.43744084	4.68547177
NONHSAG040004	2.63537953	2.38997933	2.44681291	2.41370041	2.58220758	2.48021727	2.30669524	2.34417946	2.7656768
NONHSAG040070	3.06706643	2.91569165	2.97219334	2.91864789	3.05178401	3.05462304	2.94523974	2.94432334	2.95040756
NONHSAG040081	2.27527947	2.40723152	2.20060806	2.50181233	2.36435738	2.52006728	2.87058823	3.18972731	2.69420489
NONHSAG040093	2.34522321	2.41537963	2.32828278	2.33687382	2.28316429	2.27597651	2.21011887	2.43904531	2.4111386
NONHSAG040100	2.56434123	2.38724192	2.67810296	2.37302913	2.54777113	2.85354002	2.77019222	2.82007498	2.76307727
NONHSAG040122	5.58594658	5.55847399	5.69396791	5.76039739	5.70494562	5.7337079	5.82871005	5.569702	5.49538048
NONHSAG040157	4.77966085	4.61014222	4.64598311	4.51769849	4.97240965	4.59107187	4.8136052	4.78033683	4.84611013
NONHSAG040159	4.79475319	4.62886391	4.75354274	4.99595862	5.0532569	5.03753192	4.75584724	4.67027576	4.90069366
NONHSAG040185	7.8216287	7.70380982	7.59343049	7.68596997	7.60545406	7.46398924	7.58379779	7.6320052	7.6124273
NONHSAG040189	3.08202537	3.09353745	2.93803468	3.46134666	3.29020601	2.97326306	2.99510289	2.6501728	3.10290043
NONHSAG040206	5.34481237	5.52874853	5.17522156	5.28466269	5.21865039	5.22889439	4.74923838	5.12932234	4.85078933
NONHSAG040218	2.31482672	2.49932216	2.37534823	2.71075403	2.41304583	2.5058831	2.63322259	2.6439529	2.68604663
NONHSAG040274	2.09738918	2.39270839	2.11879162	2.13330291	2.35152196	2.31352584	2.27500894	2.43904531	2.30347336
NONHSAG040276	2.42625213	2.56981885	2.55318391	2.84638033	2.44128395	2.37500411	2.44796949	2.68235441	2.46381153
NONHSAG040302	5.46191782	5.47035249	5.46884	5.46091012	5.29764701	5.66122049	5.2983644	5.45069675	5.31294286
NONHSAG040333	7.60477096	7.71311221	7.40035769	7.68001357	7.7868348	7.40770248	7.45452322	7.51479452	7.5667184
NONHSAG040375	3.14407625	3.55570395	3.52251188	3.43028441	2.90280289	3.56958145	3.16014587	3.19424577	3.09167266
NONHSAG040378	4.10327661	3.9776852	3.94530151	3.88847485	4.25695284	3.99910197	4.13871887	3.83628406	3.95418189

NONHSAG040385	3.88431807	3.76309917	3.90315284	3.82533101	3.68317497	3.54759522	3.795719	3.91793281	3.94301181
NONHSAG040395	3.09585879	2.93731557	3.0273345	3.02939082	3.05076367	3.21305245	3.12703694	3.0394088	3.04257074
NONHSAG040459	2.47493427	2.7279997	2.68415808	2.7700025	2.67816074	2.41638656	2.74501324	2.7348408	2.58214337
NONHSAG040465	2.96466736	3.06789729	3.14907301	3.01602321	3.17978636	2.94672015	3.13265205	3.13964084	3.25554812
NONHSAG040492	3.12072208	2.66915528	3.04373919	3.23883785	3.31712192	2.96920914	2.91437982	3.00077403	3.28425623
NONHSAG040535	5.0884247	4.99001619	4.78196507	5.08125775	5.01676353	4.84331306	5.16321117	5.09119463	5.12468951
NONHSAG040538	3.16447177	3.18450309	3.30589489	3.24338158	3.189399	3.19376158	3.21501261	3.23848568	3.27862076
NONHSAG040545	3.78815374	3.5955203	3.74342496	3.84926286	3.58447379	3.695829	3.75300539	3.64573982	3.75724227
NONHSAG040546	4.12841586	4.15141458	4.03613616	4.05013731	4.16014046	4.016647	4.29017838	3.83322031	4.25124981
NONHSAG040559	4.52074695	4.54152797	4.5827233	4.58444322	4.66795129	4.67015422	4.45981543	4.65035086	4.63348547
NONHSAG040605	5.9449411	5.90312435	6.06487136	5.82216474	5.86943336	6.07927091	5.88046314	6.04414889	5.94449659
NONHSAG040664	8.69630217	8.82814047	8.84791228	8.86007856	8.61687098	8.60225218	8.65831278	8.81897205	8.71327851
NONHSAG040668	4.50856847	4.55819314	4.53803775	4.42211224	4.41638444	4.360718	4.51910616	4.83491356	4.24293742
NONHSAG040671	2.67938856	2.3696725	2.61501003	2.77541561	2.79069572	2.51613052	2.64081789	2.66479359	2.74352643
NONHSAG040680	3.28663625	3.39556238	3.38814074	3.7840535	3.39759503	3.89112008	3.29682197	3.63836451	3.48102078
NONHSAG040684	3.39339716	3.32282626	3.47827375	3.10766845	3.40058623	3.59904585	3.40288177	3.62843001	3.53209736
NONHSAG040703	4.94996174	4.89725571	4.81149894	5.05148576	4.6496601	5.07342236	5.06639397	5.11423413	4.60069029
NONHSAG040714	3.72734731	3.78812118	3.94756138	3.52543036	4.02053763	3.95864724	3.8638699	3.54890376	4.10679345
NONHSAG040766	3.64879681	3.43478531	3.82512931	3.69197994	3.70024052	3.71185575	3.74150907	3.70578402	3.65672089
NONHSAG040771	2.78823575	2.89879154	3.22977019	2.83139816	2.96142214	2.96488868	2.85635859	2.68524795	2.94443053
NONHSAG040781	6.48811441	6.78796613	6.52009854	6.68697626	6.32679385	6.54452047	6.38820802	6.75464174	6.29391295
NONHSAG040790	4.18257723	3.96280201	4.23674789	3.9869787	4.65026142	4.0508088	3.98702187	4.27424419	4.46098433
NONHSAG040792	3.75917626	3.61478619	3.89775019	3.73849471	4.10285928	3.88639125	3.93862652	3.83341926	3.89389341
NONHSAG040794	4.90076794	4.75765932	4.56386857	4.74905424	4.57963207	4.9085682	4.74289617	4.78688142	4.59547929
NONHSAG040797	3.79343759	3.31747125	3.58795454	3.4330312	4.02123156	3.54143144	3.53655295	3.3936599	3.53470468
NONHSAG040826	6.38624735	6.21206052	6.18449636	6.25692806	6.24066986	6.54243299	6.57307986	6.27340065	6.92449346
NONHSAG040838	3.48278897	2.9658788	3.35356947	3.34956438	3.64839208	3.36971935	3.23700854	3.27642232	3.18258688
NONHSAG040876	4.77305427	5.07142322	4.70944587	4.93722317	4.78832709	4.84484533	4.57734195	4.75915122	4.59779658
NONHSAG040878	4.55445647	4.63072096	5.13341003	4.58217102	4.58537637	5.20889106	4.99311407	4.83936076	4.99113995
NONHSAG040919	2.10215881	2.08040679	2.02686076	1.95227404	2.22805304	2.01527414	2.05534727	1.99810781	2.19828277
NONHSAG040944	2.6107609	2.46670108	2.93278708	2.96712054	3.19003222	3.08317025	2.89717939	2.49723146	2.91964945
NONHSAG040959	3.10728283	2.91595295	3.16458585	3.03020543	3.1281271	3.2148812	3.46856524	3.00700692	2.91899008
NONHSAG040973	4.26653858	4.19905209	4.30987399	4.36746645	4.24430513	4.40461487	4.07131237	4.23992328	4.1198246
NONHSAG040975	3.03206375	3.07515028	3.08584351	3.62258756	3.07900814	3.04855062	3.14657564	2.99115902	3.18771736
NONHSAG040987	3.94936853	3.94527363	3.99683533	4.04587891	3.90731775	4.0675834	3.91789776	3.81390114	3.87423431
NONHSAG040990	2.38362074	2.34342292	2.31601482	2.48681641	2.34273547	2.36435738	2.243638	2.45438435	2.16024009
NONHSAG040992	2.58585826	2.70300583	2.97020518	2.88806824	2.7808816	2.55037186	2.39960526	3.34428093	3.02320094
NONHSAG041024	2.28102046	2.44150986	2.30535128	2.57021665	2.30355567	2.60124168	2.35994819	2.38935784	2.40274291
NONHSAG041029	2.30941835	2.33504435	2.75481795	2.56682748	2.1869872	2.51962789	2.31562761	2.53363452	2.3829012
NONHSAG041052	5.51299665	5.35520666	5.09462931	5.26748531	5.24807422	5.57734921	5.39385984	5.61337656	5.5235884
NONHSAG041087	3.37795328	3.51605941	3.24656467	3.74205424	3.21771188	3.45247876	3.49275829	3.19467383	3.77612613
NONHSAG041094	9.19137308	9.27142705	9.18691349	8.92387866	9.20086252	9.21427365	9.27356371	9.30906405	9.44517501
NONHSAG041153	2.65452325	2.60577772	2.70348811	2.76082476	2.6574658	2.71355305	2.76439187	2.67258995	2.5758998
NONHSAG041157	3.48471359	3.62484837	3.06389148	2.96846604	3.41800246	3.7345943	3.40940353	3.46173172	3.91981284
NONHSAG041202	2.7876105	2.60142535	2.80798871	2.91752741	2.87711273	2.79091291	2.82813904	2.77282293	2.78281371
NONHSAG041205	4.350636	3.79922677	4.22330033	4.16139107	4.13260683	4.29601649	4.22474499	4.23062075	4.09076785
NONHSAG041207	4.44875251	4.22499004	4.14668916	4.26635884	4.07955059	4.40036471	4.39776885	4.22853283	4.5727547
NONHSAG041271	2.95069523	2.96939848	3.02994709	3.29517495	3.17368317	3.09552186	2.95531583	3.07997225	3.03486195
NONHSAG041280	6.59147023	6.8259286	6.45725253	6.65855693	6.66208542	6.6362624	6.5479371	6.63473836	6.5854711
NONHSAG041308	2.52484738	2.39461846	2.36979828	2.54525167	2.24889006	2.21921442	2.4234487	2.32692957	2.5758998
NONHSAG041310	4.66679704	4.62692528	4.47341839	4.50967903	4.70423269	4.6246316	4.46604835	4.60187207	4.60338252
NONHSAG041311	2.46380306	2.71109682	2.45486542	2.42516775	2.4335224	2.621537	2.70760641	2.60137266	2.2932184
NONHSAG041331	8.22909924	8.66928772	8.4177023	8.60463659	8.32407367	8.57724706	8.40117357	8.53248254	8.07812651
NONHSAG041341	2.79139117	2.91386677	2.87180724	2.87896777	2.96383397	2.74269416	2.8236908	2.95490454	2.86110957
NONHSAG041345	2.54557286	2.41799218	2.55548805	2.5976875	2.68077991	2.66294875	2.59979089	2.4793827	2.40247206
NONHSAG041359	2.52484738	2.51500971	2.96225642	2.76068743	2.95074083	2.53839612	2.81222168	2.56744335	2.69310994
NONHSAG041393	2.28679227	2.21964874	2.21256499	2.18844364	2.21353723	2.06216309	2.29160871	2.25402432	2.23577944
NONHSAG041442	2.91895467	2.93772467	2.7649953	2.75016886	2.81170799	3.13229845	2.60410654	3.1601693	3.22935161
NONHSAG041473	6.25445976	6.6562465	6.17277404	5.65596085	5.44924648	6.41967535	6.21335731	6.64747441	6.06397663
NONHSAG041474	5.41456298	5.47698191	5.35129946	5.6757945	5.29651831	5.47868636	5.42607844	5.35239581	5.2194929
NONHSAG041475	3.67424617	3.7081499	3.6960393	3.87901363	3.77825554	3.69165184	3.56683882	3.73887584	3.63080212
NONHSAG041496	2.69933802	2.62078283	2.93194596	2.74034316	3.10582552	2.75017286	2.72251865	2.71710385	2.88969383
NONHSAG041512	4.56773313	4.49281952	4.21769616	4.55413911	4.75229321	4.6625231	4.70620852	4.70099397	4.69132106
NONHSAG041520	3.08885204	3.25143602	2.87710517	3.19361082	3.48875248	3.1751157	2.86258333	3.26855625	3.03618385
NONHSAG041523	4.99612138	5.11964481	5.2546002	5.08428215	4.72866719	5.142484	4.99150868	5.17830407	5.96819639
NONHSAG041551	5.23884116	5.08644801	5.332575	5.50040479	5.39354248	5.72185186	5.52195117	5.62040901	5.53733011
NONHSAG041552	3.99898263	4.08216695	4.2870436	4.15271446	4.12487686	4.08974344	3.97770338	4.03442233	4.23600564
NONHSAG041579	5.59926232	5.42165379	5.64990551	5.24867615	5.84018577	5.4184839	5.68847178	5.80219486	5.96810266
NONHSAG041596	11.21524773	10.90286097	10.96584158	10.38482889	11.32533389	10.75278266	11.01909617	10.88770912	11.09841125
NONHSAG041601	4.07660621	3.71977783	4.03646735	4.25400444	3.87201885	4.05536332	4.00429955	4.23601746	4.41445323
NONHSAG041618	4.22566067	4.66841092	4.44560017	4.51249034	4.42629032	4.55329892	4.54495148	4.16452841	4.58000266

NONHSAG041677	4.77103214	4.62275285	4.63106863	4.64623506	4.51127538	4.47420644	4.60405962	4.74459977	4.49744299
NONHSAG041723	4.26869134	4.45684577	3.70070751	4.43945795	3.87868078	4.329052	4.34770634	4.46230349	3.75444803
NONHSAG041789	4.01058891	3.87131154	4.11240168	4.16432074	4.08986365	3.75913244	3.9816203	3.93734812	4.1614468
NONHSAG041804	2.22193018	2.23973058	2.29408271	2.47670883	2.29997368	2.31547264	2.33964048	2.30383628	2.32816608
NONHSAG041810	3.12845291	3.04918367	3.11021276	3.08334594	3.13019747	3.03230993	3.176843	3.14944694	3.51581552
NONHSAG041826	2.06254948	2.11765509	2.26367951	2.18844364	2.12299967	2.07973553	2.13464169	2.23729509	2.05272811
NONHSAG041886	2.99040966	2.67323661	2.67131663	2.63565532	3.26851748	2.79536538	3.11691389	2.35015829	2.99084168
NONHSAG041903	3.92870108	3.84794587	4.05278437	4.16682371	4.01911205	3.85840899	3.8962611	3.9916968	3.88756955
NONHSAG041984	4.95068021	5.04829049	5.1626999	5.12614844	5.13795537	5.23973997	5.43515749	5.21395703	5.77396755
NONHSAG041987	3.37263284	3.21394334	3.38567075	3.2516687	3.27664772	3.18872092	3.45849105	3.29506615	3.27678412
NONHSAG041994	2.22444551	2.43409198	2.47332033	2.36902279	2.45696282	2.61993128	2.63418579	2.46969192	2.34962577
NONHSAG041998	2.51282838	2.50187142	2.74538469	2.39509256	2.71480602	2.85109305	2.59336259	2.65237677	2.74433736
NONHSAG041999	3.0600264	3.13136608	3.18166732	3.34368352	3.11055662	3.11222081	3.20131239	3.10110648	3.06491513
NONHSAG042046	2.6027792	2.47625574	2.57899246	2.55410038	2.63487639	2.59256685	2.64389789	2.62763175	2.59954566
NONHSAG042051	4.83934652	5.02110503	5.061703	5.32287356	5.16914595	5.09234961	5.00768271	4.90453332	5.37703714
NONHSAG042059	3.11969447	3.21794844	3.50886676	3.2941214	3.28913781	3.43975152	3.25262258	3.03500503	3.43082699
NONHSAG042103	3.35901939	3.23699522	3.34372524	3.62809531	3.58905358	3.45123252	3.1165766	3.40065909	3.34525882
NONHSAG042145	3.21374744	3.17463854	3.1603904	3.15269002	3.21951449	3.41145044	3.34833452	3.24217083	3.24394463
NONHSAG042163	3.72029188	3.82711056	3.5406404	3.81721578	3.76713258	3.91432825	3.99896856	3.4607981	3.87276929
NONHSAG042168	2.3239093	2.48746987	2.51418908	2.38368566	2.5081571	2.60764718	2.35096043	2.19877341	2.64937656
NONHSAG042176	10.75098784	10.73881516	10.68117419	10.62809287	10.75041862	10.6553681	10.5694131	10.64097953	10.70024577
NONHSAG042182	4.32533905	3.8412396	4.17602935	4.23131193	4.22389462	4.31410497	4.11152146	3.81086603	4.12992907
NONHSAG042192	3.23034366	3.23141546	3.27505435	3.11864843	3.17800755	3.15252818	3.21363886	3.13585218	3.19636653
NONHSAG042210	2.74704101	2.87233968	2.97060434	3.2080716	3.04930247	3.27549382	2.97187463	3.14340457	3.05178401
NONHSAG042250	3.50108996	3.55742495	3.45219604	3.72111303	3.55107022	3.55789178	3.53011377	3.53364519	3.48799259
NONHSAG042261	3.71495792	3.75447179	3.79886707	3.99422917	3.65992196	4.08965322	3.66387246	3.87076971	3.59443161
NONHSAG042272	4.10503763	4.18637976	4.2035161	4.17057023	4.26838651	4.09291402	4.05840505	3.95987194	4.24165598
NONHSAG042283	3.94229408	3.77866059	4.05799317	4.18919393	4.02154106	3.85436478	3.90082291	4.06359623	4.08406899
NONHSAG042315	4.78642819	4.79653912	4.87569637	5.00895626	4.90541673	5.00207894	5.00177686	4.78047081	5.09328408
NONHSAG042323	4.27156558	4.31287767	4.41192643	4.45378812	4.00946209	4.02022942	4.50861115	4.10623479	4.66053235
NONHSAG042369	4.38784762	4.23692006	4.4327548	4.25597354	4.60086137	4.62979449	4.36423507	4.59872581	4.93334158
NONHSAG042381	2.67985678	2.86900216	2.71741599	2.71968562	2.81728381	2.71968562	2.86048724	3.05277808	2.74785537
NONHSAG042418	4.79488088	4.99570984	4.9559258	4.63114006	5.30543298	5.10246586	5.00962042	4.41079063	5.37606593
NONHSAG042419	5.30312973	5.2471835	5.39991135	5.35336309	5.24543373	5.40946523	5.42214296	5.56986443	5.71579667
NONHSAG042424	4.80724459	4.8090256	5.12142212	5.20122543	4.832859	4.67948999	4.9944999	4.76299965	4.88618059
NONHSAG042444	4.14543274	4.3847312	4.55151572	4.86276125	4.20730448	4.15046664	4.56480467	4.4824884	4.85165718
NONHSAG042451	3.83413026	3.97338399	3.83466735	4.40951173	4.00079972	4.09046216	4.12428889	4.01363717	4.01645916
NONHSAG042471	2.99014602	3.07794084	2.86852726	3.57214143	2.81707985	3.1095166	3.34201742	3.47706119	2.97332346
NONHSAG042535	2.50218192	2.39209348	2.26372548	2.55681045	2.69839252	2.41298357	2.34367813	2.47971325	2.40207311
NONHSAG042546	2.81986881	3.39664424	3.03311755	3.23338593	3.30655263	3.0791256	3.26957134	3.08078914	3.43982914
NONHSAG042558	3.75490985	3.71941366	3.94455885	4.1374835	4.24223321	3.83501967	3.80422126	4.16260382	4.31184481
NONHSAG042615	2.68568507	2.52933928	2.56197472	2.70747152	2.42910281	2.54097368	2.63191092	2.47421662	2.56377439
NONHSAG042636	4.45793334	4.47142071	4.51618989	4.4781174	4.63900121	4.24901126	4.3201143	4.5318884	4.68819449
NONHSAG042638	4.62034508	4.63586151	4.5360203	4.47202758	4.62002445	4.58898734	4.89282569	4.69949437	4.55691585
NONHSAG042726	4.31768509	4.57155384	4.40918271	4.37736338	4.48308117	4.46410584	4.24773217	4.62827261	3.98798035
NONHSAG042795	3.31549018	3.15602391	3.29880888	3.33806385	3.58710902	3.33310909	3.39401285	3.26783552	3.37379336
NONHSAG042834	3.64810405	3.49404868	3.71473134	3.82307483	3.66805893	3.4846934	3.59395999	3.72192858	3.41405223
NONHSAG042877	3.53530091	3.12673977	3.1487686	3.46247169	3.1612187	3.44557288	3.50028271	3.43565152	3.46237768
NONHSAG042907	6.71596108	6.74487223	6.59608848	6.78030401	6.59114492	6.80365318	6.66740464	6.69025698	6.55180566
NONHSAG042913	2.41228161	2.24739708	2.57322742	2.45604591	2.40477167	2.52153976	2.38818831	2.15056925	2.29322328
NONHSAG042922	3.04549718	2.90664079	3.02751481	2.93946832	2.80742762	3.03787903	3.02634021	3.05605269	3.08897463
NONHSAG042934	3.37830178	3.1121902	3.4379291	3.00933991	3.42995588	3.38913366	3.15798994	3.41075649	3.40943588
NONHSAG042947	5.74878216	5.1676233	5.6429714	5.71503393	5.94912404	5.90819322	5.94103565	5.72534852	5.88732273
NONHSAG042959	3.40542149	3.30197717	3.53399533	3.42017725	3.40734558	3.40640466	3.35220817	3.39164955	3.47033686
NONHSAG042979	3.17461342	3.08378232	3.20943781	3.15472425	3.24238894	3.36664028	3.24144124	3.21259461	3.50978151
NONHSAG042982	2.637963	2.48086848	2.78699615	2.50478613	2.78594887	2.52602179	2.64848441	2.73649555	2.91404607
LOC284379	6.17243055	6.26921399	6.0867849	5.97945525	6.05003241	5.34580802	5.7139302	5.67247478	5.59052046
EP300-AS1	4.09930814	3.98024309	3.243335	3.9484758	4.41874732	4.43209471	4.15622402	4.07734361	4.00259969
DEPDC1-AS1	2.75192676	2.61469047	2.5090536	2.64639227	2.69391891	2.77512465	2.54704817	2.5758998	2.87893422
RUNDC3A-AS1	6.46785943	6.71102639	6.41691511	6.7112381	6.64722109	6.2497928	6.61265806	6.44336353	6.76573312
PTPRG-AS1	6.003507	5.71143596	5.60120997	5.57600166	6.06317253	6.1434596	6.12503625	5.92953115	6.01571073
ELOVL2-AS1	6.16815131	6.48979289	5.92025913	6.08464222	6.75361414	6.40427733	6.53512099	6.09199749	6.63194671
NONHSAG016418	4.44699906	3.94546239	4.02534058	4.56070289	5.26797545	4.96790324	4.72788775	4.46182408	4.62843123
TMPO-AS1	4.86621321	4.7325124	4.41944745	4.81003359	5.31088901	5.47632336	5.20210701	4.91032086	4.83931277
NONHSAG042983	2.38972479	2.21781812	2.42116422	2.33287694	2.356428	2.2760638	2.40012046	2.21404453	2.43021751
NONHSAG042995	3.66449108	3.27532274	4.0725302	3.53156942	3.97087602	3.35177378	3.9332123	3.42227928	3.89694158
NONHSAG043003	3.14793607	2.67860531	2.91195707	2.93162841	2.82066067	2.76950867	2.92784052	2.58016459	3.20252658
NONHSAG043014	2.92233304	3.0822532	3.05330293	3.32963908	3.29006315	3.09178679	3.23594719	3.0663154	3.01229141
NONHSAG043016	2.54211292	2.68132019	2.51726441	2.71074648	2.53580185	2.45850125	2.54145137	2.65269842	2.49899989
NONHSAG043027	4.59171351	4.39914069	4.55862574	4.70808694	4.70733745	4.79264111	4.25272123	4.01431975	4.56129648
NONHSAG043033	3.3058171	3.45347823	3.34055272	3.57214143	3.60275129	3.28775464	3.2512735	3.13135132	3.28526316

NONHSAG043043	6.7123985	6.79268014	6.75890729	6.64840587	6.80983784	6.95564291	6.80701258	6.7718742	6.78321832
NONHSAG043047	5.32548321	5.54773156	5.27564231	5.38023169	5.19632721	5.26497154	5.58598352	5.29282964	5.53837033
NONHSAG043071	5.4150834	4.94384862	5.07757141	5.30900623	5.12788914	5.25976162	5.26634787	4.98278862	5.07863608
NONHSAG043074	4.55102206	4.00380766	4.20998793	4.58583294	4.13167704	4.37911687	4.46194095	4.14823659	4.27290836
NONHSAG043077	4.20503353	4.41874732	4.29615676	4.40508299	4.19758636	4.26545648	4.18892874	4.42185141	4.15849994
NONHSAG043093	3.32897317	3.41156072	3.22646017	3.20828859	3.37400526	3.35839684	3.48387075	3.39084239	3.36884962
NONHSAG043129	3.60464327	3.60578931	3.24230724	3.57214143	3.48875248	3.58654842	3.63840867	3.78531432	3.66593212
NONHSAG043135	6.25551048	6.08993877	6.1277565	5.55535619	6.27054379	5.9259568	6.14471186	6.01625307	6.25505453
NONHSAG043144	3.32685554	3.49034079	3.74168103	3.68054349	3.57911538	3.66463543	3.36004419	3.69212057	3.50852578
NONHSAG043195	3.14869935	3.10230356	3.02326569	3.27522749	3.30847112	2.99851935	2.86699099	3.43012569	3.24714846
NONHSAG043203	5.74167194	5.86801809	6.00607278	5.78979834	5.91056335	5.58152687	5.40757844	5.46111488	5.87366194
NONHSAG043218	2.11398103	2.26252783	2.22330983	2.28753125	2.33759507	2.41027868	2.22883169	2.23433368	2.12428099
NONHSAG043252	2.58827202	2.48068909	2.24769987	2.57129238	2.37804613	2.69565351	2.39905246	2.42109373	2.6954522
NONHSAG043277	4.35090812	4.37272565	4.22825847	4.22655056	4.24242642	4.16961595	4.41367122	4.15715342	4.26432018
NONHSAG043281	2.67438427	2.503307	2.65813922	2.49035286	2.53033066	2.55921838	2.55044704	2.58968604	2.72959762
NONHSAG043292	2.92063336	2.61986754	2.96059292	3.14454936	3.03981534	2.87223284	2.74099147	3.01484877	2.8847583
NONHSAG043304	2.97271538	2.72316822	2.8417443	2.83059115	2.82692057	2.7452559	2.82692057	3.12155442	2.8422466
NONHSAG043313	3.62235642	3.52608403	3.73476731	3.6203205	3.67488656	3.90031331	3.9016467	3.54737084	3.6827517
NONHSAG043379	6.34710893	6.29256883	6.26903421	6.25553553	6.4303921	6.22926402	6.03658805	6.19771321	6.52287715
NONHSAG043614	3.4282852	3.42497764	3.50298338	3.50837479	3.48875248	3.47815091	4.03427365	3.55837656	3.67582
NONHSAG043616	4.78382951	4.4353955	4.86792978	5.00636974	4.7713594	4.84222533	4.81356032	4.8277501	5.07818295
NONHSAG043628	8.92255347	9.18414805	8.86191562	8.98303274	8.87947287	9.0338792	8.95175108	8.97493224	8.87407612
NONHSAG043629	7.83395235	8.06100546	7.70891762	7.95465542	7.88266857	8.00631509	7.93578709	8.02137791	7.86676918
NONHSAG043634	2.55750791	2.49906396	2.3776379	2.72478501	2.48786599	2.50259086	2.41885733	2.29740634	2.48213784
NONHSAG043637	6.84008804	6.81424839	6.66350577	6.76458982	6.5527968	6.60945896	6.90623681	6.85130993	6.8707273
NONHSAG043646	5.61826017	5.85166777	5.72080419	5.82359281	5.82117902	5.64223176	5.28269903	5.66994553	5.80829084
NONHSAG043677	2.76090416	2.94364246	2.61115106	2.81807155	2.61361677	2.80588528	2.62471211	2.86109521	2.74788323
NONHSAG043699	2.90989579	2.7086277	2.79549024	2.67417498	3.06868642	2.60240145	2.74457624	3.10172455	3.05942266
NONHSAG043706	4.41453534	4.89246546	5.08570994	5.05327528	4.95361436	5.75232484	5.19733893	5.26216046	5.57596091
NONHSAG043726	2.55464363	2.77885032	2.72244386	2.80420936	2.74180988	2.68497204	2.4623731	2.53683712	2.74810729
NONHSAG043748	5.12063796	4.95557036	5.02124226	5.00635874	5.22142368	5.11919548	5.05700047	4.80380276	4.86718883
NONHSAG043765	7.10912604	6.84698398	6.92219454	6.75025821	7.09138064	6.83255611	6.92972626	6.86635745	7.02885174
NONHSAG043790	5.95964318	6.1450436	6.08517351	5.92672839	5.97347007	5.97120702	6.00577691	6.07695848	5.87764752
NONHSAG043821	5.80392936	5.51862776	5.9449886	5.89663679	5.91350865	5.56977407	5.91743148	5.87388871	6.01708247
NONHSAG043825	2.92114628	2.97830566	2.98856562	2.91136817	3.2341358	2.8853606	3.1228485	2.94175142	3.1292868
NONHSAG043835	3.19041479	3.36475932	3.16053632	3.22336553	3.36029131	3.1600517	3.0633436	2.95719894	3.33024503
NONHSAG043845	4.61543413	4.66768769	4.97536083	4.79228655	4.89464284	4.66414021	4.67113145	4.78337106	4.90599441
NONHSAG043849	4.2729298	4.15306967	4.15927504	4.04988357	4.32810749	4.17845955	4.15698144	4.1362024	4.20327925
NONHSAG043854	3.99436196	3.93059952	4.02138106	4.06837109	4.01243383	4.06976904	3.85501675	4.03443266	3.98264743
NONHSAG043856	5.01088112	4.88127739	5.24633832	4.76963513	5.12737309	5.14539786	5.11314768	4.86739924	4.79685317
NONHSAG043865	3.34646823	3.61958547	3.5406404	3.73729403	3.6086668	3.6365151	3.47258058	3.43565152	3.68335473
NONHSAG043905	8.87690776	9.42957246	9.39758286	9.17229937	9.05112027	9.23212844	8.72459147	8.74459767	8.64845238
NONHSAG043910	3.71945769	3.77914769	3.81749916	3.57348743	3.65739409	3.64453156	3.81302822	3.79399268	3.982649
NONHSAG043913	2.80304998	2.99420777	2.87998997	2.99999678	2.8751608	3.01968532	3.0058702	2.95081127	2.97396208
NONHSAG043916	3.73456744	3.43867389	3.44779455	3.60666753	3.50053441	3.29885396	3.56648129	3.57668893	3.73965834
NONHSAG043927	3.75769789	3.59924647	3.70638001	3.70220897	3.85603887	3.45661084	3.37311168	3.44751482	3.95155391
NONHSAG043945	2.96735162	2.91881679	3.13245973	3.00614626	2.90305517	3.21852591	3.13333011	3.22021281	3.18061953
NONHSAG043980	4.28935398	4.21583438	4.33401512	4.1479332	4.14957011	4.22800273	4.08052928	3.82233141	4.19295228
NONHSAG043986	3.13469715	3.30419465	3.21670839	3.12698579	3.26896248	3.28198865	3.22485979	3.08279001	3.28882204
NONHSAG044024	2.12741124	1.98955751	2.36224787	2.158696	2.26546991	2.46430087	2.19981673	2.04812049	2.4931269
NONHSAG044064	3.23587684	2.81181314	2.81244466	3.50879935	2.73251899	3.09713908	3.18802432	2.9008694	3.3227415
NONHSAG044066	2.93049058	2.7014646	2.85272264	2.81303487	2.76844783	2.88365562	2.73424979	2.84031727	2.98777013
NONHSAG044070	3.25293555	3.14329903	3.25173897	3.25566775	3.48234846	3.63231141	3.43530911	3.14990124	3.36934217
NONHSAG044072	2.57555886	2.40014974	2.37793066	2.45167069	2.613163	2.44567452	2.43640772	2.70221002	2.56682842
NONHSAG044100	8.15218733	8.55454968	7.87336374	7.97844716	7.84336445	7.42066639	7.21994633	7.44469835	7.48007368
NONHSAG044111	2.6383497	2.63112274	2.71606079	2.86806322	2.75334207	2.82567702	2.81319895	2.73783364	2.60507692
NONHSAG044194	3.76803459	3.4167291	3.77058245	3.67192195	4.09879862	3.99997441	3.72589071	3.62642003	3.66335889
NONHSAG044196	4.52032725	4.72945533	4.76016966	4.5156366	4.95481265	4.6184277	4.68688322	4.73437456	4.73677602
NONHSAG044199	3.3127822	3.45604828	3.25357773	3.36555864	3.18776339	3.17062411	3.04593997	3.40044566	3.19166968
NONHSAG044203	4.49645531	4.38042342	4.52818073	4.23024052	4.66464101	4.72439189	4.65537418	4.42010303	4.51625031
NONHSAG044217	4.80112169	5.26392463	4.95654688	5.09184511	4.74740371	5.34675182	5.01146303	4.85960174	4.89705831
NONHSAG044226	3.17514945	2.80822171	3.21266154	2.78562881	3.07929461	2.75776789	3.20583572	3.03386336	3.38745858
NONHSAG044243	3.70776828	3.78845069	3.62105765	4.00970079	3.67290526	3.56535373	3.87662502	3.67412477	3.6753498
NONHSAG044245	3.53031622	3.64200209	3.50108056	3.53924211	3.38713215	3.23709228	3.27068379	3.73622992	3.55504926
NONHSAG044258	3.46151756	3.76083517	3.35164137	3.90457395	3.38147669	3.67786074	3.42245305	3.3089476	3.14777843
NONHSAG044264	4.38548782	4.20800042	4.21337623	4.03420843	4.39410238	3.87755452	4.32459554	4.06247303	4.12230607
NONHSAG044265	2.24249829	2.33206717	2.44270089	2.32313527	2.44241916	2.41291681	2.2460234	2.36119446	2.07979827
NONHSAG044266	2.54284049	2.52145066	2.72503882	2.52342819	2.79101601	2.48796177	2.50039874	2.58392058	2.76202867
NONHSAG044270	2.94944571	3.19509333	3.09170004	3.04827357	3.03752579	3.06845631	3.28363356	3.16540233	3.25979693
NONHSAG044283	7.58235052	7.46137485	7.75343344	7.57125389	7.49778326	7.59542584	7.77609316	7.63042435	7.57126003
NONHSAG044290	2.46329582	2.39724989	2.5081571	2.34856551	2.09992095	2.43832325	2.46273939	2.41819414	2.30643142

NONHSAG044304	3.59327958	4.15102602	3.79013529	4.02599981	3.79322072	4.01897944	4.12929283	3.7931398	3.95262656
NONHSAG044336	4.07177556	3.77447545	4.08987093	3.90700047	4.02446012	4.11860699	4.02873946	3.99040835	4.14493766
NONHSAG044342	3.44844192	3.49771597	3.62037698	3.58720124	3.47913969	3.42237474	3.43965379	3.46062688	3.31992542
NONHSAG044349	5.45488466	5.47974627	5.59492433	5.6169089	5.78081213	5.64276894	5.58304259	5.65148904	5.84415918
NONHSAG044369	2.25862461	2.21145417	2.22764699	2.39003808	2.4389298	2.33487881	2.32432639	2.34849529	2.33205359
NONHSAG044447	2.1923419	2.34229727	2.16070661	2.54939368	2.16131036	2.43789477	2.324634	2.24856306	2.10004378
NONHSAG044503	2.20903058	2.24676454	2.39981772	2.25997741	2.40733514	2.52467044	2.41711449	2.9573645	2.38612043
NONHSAG044507	1.86984295	2.12986028	2.10942223	2.12725247	1.92915979	2.28976903	1.90377245	1.97303749	2.03727338
NONHSAG044512	3.03425298	2.74864649	2.85683966	2.83119496	2.86308295	2.77784952	2.86856323	2.81112703	2.81620241
NONHSAG044540	2.89660486	3.11431206	2.79970002	2.89047155	2.66943927	3.0060036	2.67843795	2.80444041	2.73459372
NONHSAG044561	2.76538569	2.98691893	2.94604449	2.76772351	2.9454477	2.68932168	2.66365423	2.85702184	2.84734893
NONHSAG044585	2.51494069	2.48971524	2.57094116	2.52410243	2.65844019	2.36287692	2.65963739	2.38557156	2.49752789
NONHSAG044626	2.76974958	2.38661208	2.66296679	2.91560538	2.770804	2.67128773	2.6684787	2.78912167	2.62948152
NONHSAG044627	2.75460625	2.73182399	2.66296679	2.62757059	2.71266925	2.86392395	2.64859998	2.7298485	2.68545239
NONHSAG044671	3.32660501	3.07226139	2.92003017	2.92993754	3.12266183	2.84845891	3.15174572	2.88914667	3.06050046
NONHSAG044686	2.66769427	2.48157438	2.6551274	2.63812055	2.75424925	2.74335949	2.575813	2.6695286	2.77289213
NONHSAG044742	6.58881453	6.7116142	6.55815131	6.61950742	6.58130951	6.61551368	6.4503728	6.6364511	6.59222977
NONHSAG044767	3.53941777	3.47676372	3.59687909	3.51809852	3.53587066	3.48462222	3.13414774	3.16455703	3.66175982
NONHSAG044790	3.2357584	3.32973406	3.19019522	3.80601601	3.14013192	3.59543013	3.14429212	3.62288155	3.23714002
NONHSAG044806	5.1953159	5.15895383	5.21892611	5.05609284	4.98627418	5.77699518	5.45805182	5.20608495	5.80564991
NONHSAG044810	6.71765328	6.6039002	6.63438182	6.44723234	6.63958788	6.61718368	6.56728545	6.48902516	6.65521905
NONHSAG044811	2.7900346	2.53464699	2.56197472	2.67608569	2.98212895	2.86789762	2.89696292	2.86913324	2.80959805
NONHSAG044815	2.04238725	2.05840356	1.99997093	2.15736244	2.024529	2.06079759	2.06602493	2.11729887	1.90737293
NONHSAG044846	4.47253136	4.49047812	4.55483012	4.60735882	4.7792307	4.46410584	4.51899466	4.46093744	4.52581478
NONHSAG044851	2.75771875	2.82435943	2.90836697	2.94616033	2.95704212	2.94374802	3.16832689	3.04214444	2.95988569
NONHSAG044892	3.87732834	4.0974493	4.03949951	4.18604265	4.43024731	4.16567358	4.15673122	4.10808875	4.11839392
NONHSAG044894	4.37946125	4.30201149	4.50604815	4.41731915	4.79056207	4.73997654	4.53871549	4.48304631	5.05049966
NONHSAG044918	2.72979721	2.61629612	2.68309798	2.66279009	2.53947704	3.07427359	2.63880202	2.68417302	2.46635846
NONHSAG044920	2.99412085	2.76724001	2.94983176	3.65900078	2.77617502	3.0094737	3.26004868	2.9239105	2.90758899
NONHSAG044929	5.82152576	6.05493218	5.78680168	6.15507594	5.86580432	5.96437751	5.85716214	6.01759983	5.97282844
NONHSAG044999	3.10196128	3.02319554	2.90591245	3.13772055	3.10836342	3.12581284	3.06667875	3.02488357	3.0399372
NONHSAG045020	2.4998632	2.38038835	2.44813063	2.56424435	2.78209917	2.51874229	2.5747684	2.32507591	2.51874229
NONHSAG045068	2.35165504	2.43621966	2.0845535	2.51109193	2.5081571	2.32434419	2.27889655	2.43175458	2.30347336
NONHSAG045080	3.23034366	3.30668571	3.35884294	3.10929947	3.43722958	3.25991369	3.48594365	3.19137768	3.25038622
NONHSAG045081	4.18026848	3.85922924	4.37536503	4.0482872	4.26522974	4.54361274	4.02671754	4.02073446	4.23025037
NONHSAG045085	4.90161085	4.76759173	5.00005257	5.29905021	5.09559114	5.03714693	5.05361746	4.93669196	5.00973915
NONHSAG045101	4.07676094	4.10396251	4.01141084	3.93988345	4.20398557	4.06660809	3.97770338	4.10804527	4.39711709
NONHSAG045116	2.54948961	2.67054281	2.96400808	2.71075175	2.57216392	2.98073804	2.66715378	2.6545672	2.48978235
NONHSAG045138	3.33467482	3.42152171	3.50833278	3.18207478	3.28099396	3.22040976	3.4219016	3.01782186	3.23281178
NONHSAG045167	3.6588241	3.65409399	3.69126497	3.30052234	3.92661946	3.52964078	3.69482453	3.45362589	4.03890713
NONHSAG045192	3.57870751	3.19706434	3.25416406	3.14307653	3.50097249	3.23915575	3.50257009	3.54431528	3.639065
NONHSAG045208	3.73090481	4.02242681	3.77031528	4.30109617	3.57214143	3.99679293	3.55837707	4.0794451	3.43371198
NONHSAG045235	6.18542276	6.31061475	6.37214853	6.22256637	6.33985919	6.69328301	6.45253846	6.53021302	6.92244188
NONHSAG045258	7.91290536	7.65038322	8.07815689	8.03120247	7.80937795	8.09483107	8.25286678	8.19819481	8.06952073
NONHSAG045265	4.51732123	4.44989984	4.89838544	4.59571064	4.76235935	4.78473142	4.55561196	4.60890366	4.89196337
NONHSAG045281	2.81147788	2.92913336	2.90416267	3.04939588	2.92189022	2.88963002	2.80011242	3.19147901	3.31780215
NONHSAG045316	2.20415057	2.35835306	2.26410905	2.20116547	2.37412745	2.2032107	2.3205913	2.25787611	2.0499668
NONHSAG045329	2.45340027	2.36517919	2.36133177	2.24421449	2.51428339	2.24144469	2.41657462	2.33129216	2.46020231
NONHSAG045333	4.72801647	5.07371522	4.68348559	4.77218005	4.16537728	4.63460478	4.55919476	4.81492844	3.82864576
NONHSAG045338	2.5894831	2.60700515	2.50118627	2.99308921	2.7194377	2.57295496	2.48764567	2.6019288	2.42771577
NONHSAG045350	2.64578774	2.95885163	2.63022786	2.70666222	2.61519741	2.86818279	2.69262476	2.86226814	2.77017762
NONHSAG045363	5.25564239	5.17050769	5.4134371	5.62645666	5.31803207	5.3049012	5.3855132	5.48202563	5.36046891
NONHSAG045364	4.18982813	4.16910311	4.07679386	4.36031827	4.22354707	4.1509514	4.25137015	4.04981293	4.60058656
NONHSAG045389	2.81662521	2.87282827	2.59749096	2.85742513	2.59491091	2.61259053	2.66440256	2.33105873	2.58844642
NONHSAG045403	5.55684315	5.7596772	5.58568799	5.7996447	5.72177982	5.58802516	5.19430344	5.52034046	5.6958236
NONHSAG045405	4.44671714	4.74265871	5.06151452	4.65327884	4.44832219	4.65414708	4.5811222	4.07033026	4.42161631
NONHSAG045408	4.18662212	4.01577313	3.9725563	4.27004584	4.50491839	4.64941459	4.29743826	4.40953227	4.30897267
NONHSAG045419	3.99460324	3.71035981	4.09046216	3.99487963	3.94683622	4.26165794	4.32623637	4.06470045	4.11503992
NONHSAG045425	3.45649214	3.40174641	3.41885299	3.4764282	3.69364822	3.69790454	3.52801521	3.49049971	3.98712958
NONHSAG045429	3.51480491	3.53984858	3.59524081	3.62146126	3.60435787	3.60544942	3.91183218	3.70479923	3.59897162
NONHSAG045445	3.74254335	3.34279085	3.52830464	3.55465429	4.02599885	3.79642807	3.53096773	3.40541966	4.00779165
NONHSAG045449	2.25434194	2.40232937	2.37257282	2.38401809	2.39287218	2.35180261	2.4225306	2.39211067	2.16348724
NONHSAG045463	3.42696001	3.14617872	3.55370311	3.65114978	3.42013298	3.38710786	3.49891029	3.14822158	3.65399606
NONHSAG045467	4.76416288	4.68558807	4.61272671	5.1298569	4.74142446	4.87102566	4.80330148	4.63843617	4.88618244
NONHSAG045472	3.53530091	3.52209625	3.59612081	3.54032873	3.82788479	3.64582031	3.58054947	3.59245523	3.59793516
NONHSAG045505	3.75868118	3.77468669	3.98027098	4.17979447	4.09533554	4.22217294	3.938991	3.79518776	4.04686554
NONHSAG045512	4.00550429	3.45151603	4.18146457	3.96936462	4.01531679	3.75815822	3.8210184	3.83775066	3.96734181
NONHSAG045517	2.97492878	2.86690424	2.99128679	3.13893562	3.06714191	3.20085794	2.91818082	3.31439253	3.05375831
NONHSAG046716	5.47992589	5.53658619	5.52707572	5.74507246	5.72610263	5.54021528	5.66126205	5.53214329	5.62575523
NONHSAG046750	5.47154559	5.45246878	5.74714775	5.62956997	5.53096538	5.45638643	5.45923406	5.27553121	5.54561568
NONHSAG046781	5.20898649	5.25243417	5.41628781	5.17325586	5.45801239	5.29262251	5.32640988	5.29613864	5.60221801

NONHSAG046784	5.03063516	4.91180024	5.1291013	4.80094419	5.24376928	5.12877274	5.19806114	5.00584504	5.39150999
NONHSAG046797	4.52649889	4.40417678	4.55059994	4.48650954	4.50597013	4.71789077	4.47435891	4.48838149	4.64540764
NONHSAG046806	6.53512354	6.6153131	6.32880665	6.55925795	6.40115407	6.53008803	6.51116245	6.41624337	6.20374822
NONHSAG046817	4.00487814	4.07333975	3.85452382	4.07660274	4.15675516	4.03623385	4.23745594	4.45396273	4.17545282
NONHSAG046838	3.91592615	3.73503743	4.08057364	4.18581399	3.84850077	4.08533144	4.28548546	4.02756227	4.07481365
NONHSAG046851	4.15673122	4.11517099	4.51255038	4.32428187	4.45986525	4.35238559	4.17219962	4.33500294	4.56963557
NONHSAG046903	4.45825805	4.5994912	4.51033714	4.59070499	4.45943843	4.73871506	4.38639301	4.79488534	4.64557766
NONHSAG046927	2.86172923	2.91509464	2.6450651	2.56470462	3.16771042	2.7312347	2.81889147	2.94959239	2.69438157
NONHSAG046961	2.95506303	2.96115242	2.97944946	3.03052452	3.10757379	3.1149064	2.92653532	2.93877019	3.14290164
NONHSAG047021	2.37769343	2.35768046	2.54625476	2.33132784	2.49089949	2.67503732	2.35554067	2.40002033	2.39598121
NONHSAG047026	4.64028955	4.73438691	4.51662013	4.70496137	4.7461669	4.5901262	4.71844847	4.81727599	4.8648177
NONHSAG047060	2.54713271	2.6137877	2.71436885	2.88855331	2.63632652	2.4925268	2.59774241	2.65501981	2.78585529
NONHSAG047082	3.64984437	3.37240089	3.54245818	3.66373397	3.69747995	3.6029532	3.43405657	3.53388493	3.57210465
NONHSAG047088	2.46380306	2.3231852	2.3488071	2.61051034	2.52323752	2.33732803	2.3540321	2.51010666	2.59949961
NONHSAG047097	4.32777824	4.78525797	4.05333583	4.61143558	4.18212963	4.74526571	4.2033964	4.7299979	3.92622727
NONHSAG047101	2.79614698	2.96879352	3.02540775	2.45836433	2.81912892	2.84932777	3.0456015	2.56407752	2.74085742
NONHSAG047126	2.4360168	2.3204464	2.34253408	2.69379962	2.60315434	2.55152416	2.7523595	2.53873908	2.6476339
NONHSAG047149	3.94046858	3.80445549	3.85843349	4.34424461	4.06586685	3.93665841	3.96081879	4.03311811	3.99274267
NONHSAG047152	2.75771875	2.82671401	2.86615405	2.90257064	2.91364051	2.74782349	2.66100744	2.85706873	3.02264307
NONHSAG047169	2.68770079	2.45649078	2.6150727	2.47680171	2.54611161	2.59459972	2.69656047	2.72054294	2.47301024
NONHSAG047175	3.41609269	3.21288433	3.52665171	3.28277725	3.53936241	3.49093225	3.30476634	3.39207693	3.44662677
NONHSAG047181	3.4319354	3.12568362	3.28703803	3.20794184	3.30428947	3.33905188	3.34633963	3.29093962	3.22562243
NONHSAG047192	4.46806567	4.52907209	4.56397694	4.92023323	4.48883839	4.89877038	4.78341939	4.78055829	4.64523743
NONHSAG047193	3.23507436	3.15158009	3.08799542	3.14901257	3.18138783	3.25764571	3.01322668	3.39823717	3.20966046
NONHSAG047194	3.28301585	3.24646067	3.35164137	3.3698319	3.34155759	3.3130311	3.08534884	3.29220894	3.67418724
NONHSAG047208	2.59529721	2.36921049	2.63965125	2.47422508	2.63965125	2.56620824	2.61755783	2.52320838	2.41581049
NONHSAG047221	3.4021733	3.54221571	3.58008668	3.47411041	3.49159591	3.22875022	3.59206409	3.18837608	3.40282233
NONHSAG047229	4.61896303	4.5353322	4.91905262	4.91244029	4.69345343	4.5353322	5.07952515	4.88797241	5.0566965
NONHSAG047232	4.20434955	4.02632627	3.92929317	3.88042315	3.97372107	3.94912449	3.8944184	3.95528118	3.69268031
NONHSAG047253	3.27212991	3.18750312	3.2226648	3.23671234	3.23122105	3.39809771	3.17092269	3.21826228	3.34604054
NONHSAG047257	3.47898514	3.54361338	3.81650609	3.57942643	3.43135673	3.8228067	3.62918679	3.81430731	3.44698157
NONHSAG047259	3.54497556	3.72358599	3.28757205	3.87542658	3.56887029	3.48375774	3.36770065	3.34315588	3.53918418
NONHSAG047273	5.78233606	5.83171531	6.12168294	6.111928	5.93946212	6.17282763	6.06140486	5.67501745	6.17494832
NONHSAG047282	2.6430233	2.79080559	2.70383229	2.77657987	2.82709645	2.77246221	2.71807934	2.77345352	2.78680514
NONHSAG047295	2.95989323	2.81412638	2.90939766	2.91150118	2.94297739	2.91104621	2.97136181	2.9154274	3.07198997
NONHSAG047314	4.36643677	4.5378238	4.43466393	4.53087107	4.38809075	4.12892845	4.26056907	3.95171214	4.28079125
NONHSAG047336	5.22644049	5.65189872	5.13638009	5.32700061	4.99851174	5.49085494	5.07268502	5.29133472	5.15250471
NONHSAG047348	2.74906415	2.74006198	2.78581112	2.65322669	2.77837464	2.74649064	2.7887035	2.88845608	2.8248597
NONHSAG047356	2.62748829	2.82460404	3.00377653	2.7320522	2.75690744	3.00289018	2.50248295	2.74200568	2.72636752
NONHSAG047375	3.37328824	3.19146211	3.23074637	3.61630897	3.72431459	3.22735055	3.10478548	3.34931646	3.42484455
NONHSAG047399	2.70711679	2.78201599	2.6524787	2.56143043	2.5660594	2.81937267	2.52143052	2.79026449	2.54102249
NONHSAG047416	2.75225812	2.6704387	2.7317916	2.85215537	2.80863551	2.71903267	2.7729525	2.55506951	2.6699682
NONHSAG047425	2.40677765	2.26404112	2.40596298	2.61392027	2.47863587	2.4761616	2.61501003	2.40467945	2.38772277
NONHSAG047431	2.90844129	2.85030173	2.64995491	2.57159481	2.87851731	2.91067494	2.91500616	3.31673953	2.71572406
NONHSAG047443	3.14820338	2.94422553	3.09896541	2.80464611	3.11652279	2.94639316	2.88300499	3.0574955	2.9367545
NONHSAG047447	2.32604622	2.31162657	2.32175047	2.34151375	2.54497833	2.40109718	2.41433244	2.43859559	2.30347336
NONHSAG047469	4.25964622	4.04343492	3.96196456	4.21049719	4.19582435	4.12230145	4.14848107	4.17238163	4.2962462
NONHSAG047503	2.59892392	2.52643646	2.32770103	2.37889769	2.41172491	2.43377035	2.44600629	2.42956729	2.37402978
NONHSAG047517	6.35962097	6.53059484	6.44269625	6.47793081	6.36465012	6.4643418	6.3650234	6.26477099	6.22871289
NONHSAG047524	2.90106144	3.11397522	2.9974487	3.18209277	3.14054913	3.13244382	3.05835246	3.32671559	2.87693531
NONHSAG047536	2.32292519	2.04603172	2.19406267	2.1174464	2.20932452	2.215052	2.23446328	2.03729735	2.1415165
NONHSAG047547	6.88986105	6.95043175	6.56941838	6.13850756	6.90623196	6.61821159	6.64962287	6.55727507	6.51425398
NONHSAG047553	4.47045439	4.36982038	4.54083846	4.39873057	4.79601902	4.51878862	4.70620852	4.27980589	4.59945179
NONHSAG047570	4.85107814	4.80111404	4.79762558	4.63387852	4.94829838	4.67088823	4.82392496	4.83145761	4.7516952
NONHSAG047611	3.93082731	4.08303426	4.15026901	4.27036609	4.29122253	4.17434943	4.14880102	4.17471242	3.94537266
NONHSAG047665	4.22427837	4.36655066	4.02006233	4.41235865	3.93559878	3.95028605	4.13015265	4.09598535	4.13002414
NONHSAG047674	2.70302871	2.69648787	2.65599749	2.66304292	2.83149391	2.92890185	2.70519649	2.64348437	2.69189762
NONHSAG047730	2.76740747	2.8830631	2.99104109	2.57645214	2.80959805	2.78053071	2.72030089	2.83126332	2.95873814
NONHSAG047747	3.63783084	3.70412016	3.57927861	3.75157388	3.72944966	4.30915861	3.58364411	3.65737857	3.6096204
NONHSAG047777	4.60318865	4.77538011	4.82480165	4.4120107	4.37088727	4.44890452	4.03148857	4.37319424	4.03646664
NONHSAG047807	2.88754961	2.86330838	3.10932808	3.08571821	3.05178401	2.72966657	2.94183538	2.94539926	3.00410604
NONHSAG047837	2.17384601	2.39114591	2.6801673	2.74415386	2.37576768	2.82801557	2.49975706	2.55186356	2.23574065
NONHSAG047842	7.10498907	7.20025488	6.89946005	6.7066703	6.87231397	6.96224	6.85737849	7.06337044	6.41820058
NONHSAG047845	4.17815644	4.51243021	4.23605291	4.43281793	4.17037719	3.85623813	4.0335227	4.34430871	4.3194695
NONHSAG047847	5.14558082	5.20462381	5.41835316	5.1531691	5.53065492	5.45898104	5.61429257	5.40958271	5.80694525
NONHSAG047907	9.31395389	9.0834639	9.06965806	8.84747313	9.32387341	8.89753218	9.16069028	8.99959959	8.91235962
NONHSAG048011	4.45569807	4.47844285	4.59211665	4.68121969	4.17144306	4.63652629	4.52675869	4.64436087	4.15617467
NONHSAG048092	2.25568222	2.3259299	2.46359742	2.59125824	2.55446743	2.43925675	2.5272882	2.53456667	2.69661972
NONHSAG048098	2.94139091	2.20887712	2.3488071	2.61649193	2.3759494	2.55940728	2.63147598	2.4346446	2.30245425
NONHSAG048120	3.35648474	3.50867055	3.36392103	3.53730224	3.76724747	3.4285312	3.28361998	3.48171471	3.60254134
NONHSAG048123	2.9674253	2.6085274	2.7299192	2.82613954	2.73092333	2.9121255	2.83554889	2.85114957	3.05871417

NONHSAG048148	2.40540353	2.22570001	2.29319643	2.49706462	2.12837275	2.51337676	2.28220871	2.30988584	2.10840751
NONHSAG048155	4.00057006	4.05521425	4.10961239	4.11793812	4.30540265	4.0879843	4.2464537	4.16241834	4.39569711
NONHSAG048166	2.71299291	2.84710382	2.77317335	2.72360109	2.78570671	2.65996253	3.03864074	2.68402366	3.08504757
NONHSAG048202	3.14753211	3.01184317	3.04970895	2.99591926	3.0883146	2.88066733	3.07220662	2.73097501	3.46993818
NONHSAG048386	6.05784138	5.87540681	6.18204418	6.32334292	6.25802606	6.0860587	6.01640637	6.07308014	6.13180425
NONHSAG048397	8.38663783	8.3651796	8.28762948	8.05898304	8.07462759	8.24834466	8.36774428	8.47889797	8.75314368
NONHSAG048404	5.04031309	5.14057304	5.2788474	5.07005791	4.92139863	5.18133447	4.96927215	4.88235642	5.04883388
NONHSAG048405	2.23964001	2.33996715	2.14349046	2.42465562	2.34266688	2.20620959	2.24516342	2.12299065	2.07569253
NONHSAG048439	2.90945379	2.97102756	3.05506189	2.92399415	2.97332346	3.01593991	2.98639108	3.18427926	2.88969608
NONHSAG048458	2.59048077	2.70218702	2.4650028	2.40641364	2.35609476	2.39815106	2.48626882	2.63830036	2.46367969
NONHSAG048471	6.36818629	6.33654495	6.0602807	6.00777733	6.21459816	6.49363967	6.56021333	6.3943437	6.23139111
NONHSAG048506	2.95768454	2.83801482	2.73051225	2.83764747	3.07921296	2.90808094	2.98552372	3.04961157	3.22913448
NONHSAG048520	4.26653858	4.39092752	4.10889283	4.63109242	4.21898684	4.12284471	4.38818294	4.3211345	4.21015475
NONHSAG048562	2.33443533	2.2927899	2.43128105	2.72641782	2.67604155	2.45525657	2.52422871	2.53974213	2.49551694
NONHSAG048563	6.1184438	5.34873195	5.84322345	5.66326363	6.00754977	5.85130814	6.02297415	5.91257511	6.06944186
NONHSAG048569	4.76190272	4.34210675	4.03191188	3.77072369	4.47818601	4.0547895	4.30003085	4.29468182	4.23089732
NONHSAG048593	2.43858344	2.46143017	2.54676489	2.68039769	2.51925465	2.60036155	2.72649746	2.45170754	2.68896342
NONHSAG048597	2.2222894	2.30012119	2.38444316	2.40118686	2.74811028	2.28884513	2.53559759	2.27534828	2.30347336
NONHSAG048604	3.63904856	3.57763251	3.5622757	3.63768192	3.89296181	4.03674092	3.73059557	3.71170884	3.76206115
NONHSAG048605	4.72521061	4.88638535	4.86490871	4.93539116	5.20965714	4.82021252	4.91825838	4.62997346	5.16096874
NONHSAG048651	3.38713997	3.42423471	3.31154392	3.14287796	3.30685003	3.25001218	3.1952701	3.23653561	3.11001643
NONHSAG048682	5.41160736	5.19918321	5.21319623	5.51872858	5.52044579	5.33038711	5.22900271	5.28646131	5.27439142
NONHSAG048687	2.89945387	2.86213374	2.85470974	3.01433264	2.85695346	3.01464635	2.76351068	2.99414676	2.86595649
NONHSAG048746	5.76520278	6.12218239	5.80248592	5.96928284	5.79001278	6.33033848	5.91126109	5.67210325	5.73587981
NONHSAG048747	7.99336332	8.03024503	7.67285241	8.13232101	8.01221786	8.15714236	8.30866545	8.16359093	7.86623803
NONHSAG048781	5.28172959	5.28894986	5.19904517	5.16110932	4.92650074	5.25562695	5.24931699	5.12746774	5.14828096
NONHSAG048790	2.31916921	2.35799103	2.48207782	2.0867147	2.30431082	2.40882509	2.38453792	2.70644081	2.31623914
NONHSAG048794	5.32696589	5.24100311	5.34199852	5.09692138	5.46985631	5.32123608	5.28221183	5.24302968	5.77599018
NONHSAG048813	3.51902643	3.49509726	3.70809434	3.66276697	3.72549725	3.58240717	3.71853367	3.64901238	3.65638546
NONHSAG048817	4.09430104	4.13623351	4.30423318	4.31521535	3.87103271	4.24207693	4.12931815	3.9813883	4.09954944
NONHSAG048836	4.69678131	4.47115588	4.6864211	4.80145456	4.82517706	4.87962584	4.62514843	4.66040461	5.03727147
NONHSAG048840	4.84722512	5.03272964	4.93639735	4.84651824	4.96188895	5.26316035	4.55734227	4.70250624	5.04735235
NONHSAG048846	3.45285371	3.69681786	3.49372671	3.53152006	3.73490414	3.85009083	3.9351217	3.59710074	3.60366341
NONHSAG048902	2.73037487	2.65659008	2.83040912	2.71079547	2.74730813	2.7558432	2.78326012	2.73064055	3.10448377
NONHSAG048962	6.26201488	6.45307285	6.37849159	6.259806	6.28014876	6.35856639	6.21060931	6.26560497	6.24661474
NONHSAG048976	5.23392367	5.15037525	5.24681772	5.37740032	5.17170241	5.23034592	5.28182061	5.28263503	5.01468646
NONHSAG048984	2.50777396	2.69976796	2.70822361	2.92053635	2.56614293	2.8579105	2.52185212	2.66089281	2.66509588
NONHSAG048993	5.76112201	5.84493704	5.81079928	5.75583455	6.03829081	5.89644586	5.83947071	5.87827988	6.1282265
NONHSAG049003	4.33363565	4.0966374	4.35557543	4.24402168	4.25935455	4.29052877	4.50067076	4.7021067	4.25332527
NONHSAG049010	3.93353372	3.91281582	4.11322903	4.07592526	3.99542386	3.98633794	3.98056595	3.90146486	4.05287245
NONHSAG049037	5.90990241	5.78507816	5.80994553	5.88577623	5.87527681	5.80550061	5.92599467	5.99418598	5.82165156
NONHSAG049079	7.23383967	7.61972534	7.36441109	7.50745593	7.24126086	7.23554328	7.39710115	7.29172414	7.08592355
NONHSAG049095	6.85999892	6.75349561	6.75477581	6.23078363	6.89827164	6.32118533	6.65437334	6.43967003	6.73564005
NONHSAG049099	2.62552895	2.76354234	2.66204038	2.83574995	2.81814368	2.80838424	2.75166654	2.71157187	2.72299076
NONHSAG049119	4.12508416	3.3972107	4.17752234	4.12153425	4.24084186	3.65459455	3.90038051	3.73956832	4.05466747
NONHSAG049134	3.79778702	3.74581313	3.90310427	3.96614093	4.12949412	4.0118468	3.65300419	4.04653121	3.94807866
NONHSAG049182	4.18320508	4.25130304	4.40589042	4.36924641	4.38613174	4.46596019	4.33267679	4.14282722	4.4336761
NONHSAG049240	4.18281225	4.43084692	4.34012542	4.5391469	4.3217587	4.49588516	4.76764073	4.76077422	4.619519
NONHSAG049298	3.12970729	3.3144757	3.19348696	3.24111962	3.11576089	3.26853064	3.28946781	3.15675408	3.04039375
NONHSAG049309	6.10780535	6.29940681	6.09246811	6.058725	6.13153767	6.13599188	6.14024387	6.12151666	6.1893734
NONHSAG049336	5.48144297	5.31115734	5.24681772	5.37298597	5.47510207	5.32132059	5.13687752	5.36178142	5.63091642
NONHSAG049339	2.68933439	2.63395159	2.52591427	2.68856586	2.61574996	3.0152711	2.66864499	2.80653914	2.8153661
NONHSAG049341	5.89286313	6.0494968	5.87768101	5.76453563	6.01953056	5.71254729	6.19049337	5.77480086	5.8958884
NONHSAG049344	3.97958312	4.18965385	4.09416455	4.27873583	4.18358193	4.07472824	4.40575228	4.32808256	4.14944385
NONHSAG049350	4.54637035	4.35366478	4.71523579	4.70398025	4.45358588	4.50222679	4.42461671	4.74443138	4.9035232
NONHSAG049357	2.98914013	3.08915823	3.23447207	3.39476458	3.2192166	3.19418464	3.10793799	3.16792638	3.18896258
NONHSAG049388	2.68403318	2.71376009	2.90429695	2.90841444	3.08309043	2.96771694	2.80734451	2.63662064	2.88339358
NONHSAG049409	3.00367042	2.82101347	2.82513845	3.08108631	2.93335359	2.88678205	2.92260199	2.81312752	2.63632508
NONHSAG049422	3.53530091	3.51140965	3.57272246	3.55556725	3.71530159	3.78036678	3.76489503	3.85045632	3.75118656
NONHSAG049424	5.46505942	5.68766641	5.736065	5.92638595	5.71019374	5.62811479	5.72611925	5.42293475	6.04741539
NONHSAG049464	4.28256064	4.69367381	4.08669579	4.08148967	4.44479439	4.03484823	4.18314954	4.15773275	4.22913152
NONHSAG049487	4.46088595	4.35920071	4.62198829	4.32456892	4.21818456	4.43221474	4.43698632	4.29991798	4.38406992
NONHSAG049492	2.9541498	2.87424427	2.89296353	3.10186721	2.95702752	2.88237029	2.86231706	3.11145007	3.0143009
NONHSAG049507	3.60598297	4.24893984	4.18735388	3.92673205	3.95767456	3.94673313	3.91861773	3.94487103	3.99379928
NONHSAG049508	3.70378356	3.5414095	3.6605219	3.96195317	3.65623182	3.77912338	4.07961289	3.86011812	3.84129313
NONHSAG049511	3.48569345	3.37354122	3.46111568	3.40571263	3.20891492	3.52897321	3.53470557	3.41758925	3.39880936
NONHSAG049515	2.34005431	2.28842348	2.30577216	2.53858763	2.3306252	2.55313707	2.82854483	2.44997034	2.47224905
NONHSAG049523	2.10942818	2.30867436	2.48610733	2.21523982	2.30988352	2.26723963	2.39650168	2.22076344	2.1311687
NONHSAG049524	3.49083317	3.49867102	3.77045446	3.73595858	4.03734545	3.56012958	3.89651451	3.53128893	3.8039364
NONHSAG049526	3.97989141	4.02597299	3.93331775	4.11894208	4.43106043	4.08445715	3.98570379	4.11663151	4.06102433
NONHSAG049528	4.45051176	4.73460011	4.30100265	4.53246543	4.40673728	4.66856092	4.4910142	4.46373328	4.35996268

NONHSAG049529	3.91687407	3.93458131	3.6797938	3.9162022	3.6777125	3.95848268	3.97770338	3.94532688	4.14491061
NONHSAG049530	2.75709754	2.92887169	3.00511277	3.00381633	2.64553598	2.98984294	2.91013299	2.72874357	2.71501802
NONHSAG049531	4.77911136	4.95802136	5.30167688	5.04257065	5.15905491	4.98773146	4.79239233	4.88510135	5.02877106
NONHSAG049532	3.08128725	3.11431803	3.45370152	3.24430087	3.2814111	3.47729635	3.507146	3.68388699	3.23034366
NONHSAG049534	2.67458462	2.66202239	2.53758367	2.91564761	2.65234742	2.54935594	2.54143662	2.33322087	2.82351444
NONHSAG049539	4.88654533	4.76673523	4.86155216	4.71909769	4.69212	4.75178386	4.81628349	4.85134702	4.71807168
NONHSAG049594	3.05468821	3.27791154	3.16053632	3.06149385	3.15722691	3.09783483	3.08560681	3.04519109	3.21685891
NONHSAG049595	3.32226263	3.21378283	3.47049735	3.57522208	3.38322873	3.61122711	3.4483809	3.36592201	3.57972239
NONHSAG049661	2.41837321	2.32197031	2.45846378	2.04363075	2.22805304	2.43946555	2.32492938	2.27431968	2.5081571
NONHSAG049665	3.16475936	2.61698273	3.1987805	2.41991622	2.47479391	2.79553346	3.02515376	3.09859574	2.7606074
NONHSAG049689	3.10976322	2.87627032	3.1945778	2.93266988	2.97332346	2.86293277	2.89850887	2.93388641	3.18220047
NONHSAG049734	3.68496771	3.55176648	3.98306095	3.81202298	3.81813657	3.55455844	4.01889375	3.78350065	4.26520067
NONHSAG049792	4.04441911	3.78423211	4.04252593	3.99998283	4.22950131	3.93477107	3.94510538	3.79894723	4.10645289
NONHSAG049868	5.68139749	5.40431767	5.53198896	5.70906798	5.66920649	5.5201956	5.61349054	5.42589943	5.48242245
NONHSAG049879	3.0172819	3.02136306	2.97203093	3.10257877	3.03744249	2.95704498	2.9162584	3.10084038	2.8704694
NONHSAG049885	3.72375799	3.60848505	3.74220784	3.67374381	3.73146874	3.35907044	3.5960579	3.72375799	3.65010289
NONHSAG049895	4.25226468	4.09528205	4.07097167	4.0846181	4.11189584	3.95368724	4.07263473	4.26182376	4.35567892
NONHSAG049902	2.45777694	2.64580691	2.28472336	2.51891608	2.52857134	2.69493448	2.49717003	2.73947734	3.05867396
NONHSAG049904	6.58629294	6.38184473	6.54986906	7.09955217	6.69467582	6.66629052	6.89931775	6.62763979	6.66215232
NONHSAG049930	3.31152363	3.29575568	3.20242989	3.2421035	3.16970724	3.17961962	3.08595533	3.01026742	3.14875857
NONHSAG049931	2.93049058	3.04323621	3.08676484	3.17939669	3.04018045	3.0371247	2.89589779	3.02641172	3.09301354
NONHSAG049936	2.58592968	2.59470891	2.48772676	2.89537735	2.6709249	2.94634509	2.49167419	2.46695095	2.47718396
NONHSAG049939	4.70618395	5.10061394	4.69520678	4.80034579	4.71198597	4.91078111	4.75611229	4.92141956	4.49950044
NONHSAG049977	4.99730544	4.90834224	5.03193491	5.14317658	5.12252675	4.77955058	4.76564054	5.00395749	5.15483202
NONHSAG049980	4.46713731	4.44838147	4.57727858	4.6384519	4.57996198	4.53641177	4.23745594	4.39448388	4.37971874
NONHSAG049984	2.55764215	2.43310502	2.71899689	2.37856891	2.49033997	3.04709153	2.88432028	2.69771776	2.97332346
NONHSAG049989	4.21474162	4.18796155	4.29356075	4.22539684	4.29903549	4.49892247	4.21552918	4.3410303	4.35819791
NONHSAG050013	5.8800966	5.84786858	5.62436218	5.57228734	5.94005432	5.5787184	5.91539317	5.61952162	5.90378602
NONHSAG050025	5.05621404	5.14280038	5.64684505	5.60500775	5.23333837	5.15920348	5.01885625	5.15021466	5.42047628
NONHSAG050057	3.88893704	3.51316012	3.77088046	3.83028365	3.83355302	3.99994916	4.15409236	3.7768456	3.95772074
NONHSAG050083	4.86054593	5.17460736	4.98932638	5.25811795	5.20477369	5.06385749	4.88995915	5.20501364	4.77635028
NONHSAG050088	4.31992699	4.19560651	4.28712029	4.45384796	4.50491839	4.2619737	4.31981576	4.18640662	4.29275033
NONHSAG050093	5.50894254	5.19847097	5.48922767	5.12047022	5.59005977	5.42156114	5.46085781	5.2050115	5.57560249
NONHSAG050175	4.53949993	4.26653858	4.31841036	4.34329365	4.38822581	4.55977693	4.48210704	4.38770151	4.42689256
NONHSAG050188	2.88920083	3.13712732	3.15083488	3.30053103	3.41740928	2.69227308	3.01709841	3.00913407	3.15449899
NONHSAG050210	4.98047603	4.8106176	4.61012538	4.85581427	4.97657928	4.98691009	4.95744532	5.24392466	4.94095549
NONHSAG050227	3.09745755	2.97664594	3.43391244	3.53545223	3.10074276	3.13767956	3.18699951	3.31439253	3.43889561
NONHSAG050230	2.81704522	3.18168222	2.97405082	3.05532747	3.10466029	3.22827181	3.19498639	3.16981498	3.21248806
NONHSAG050243	3.58362309	3.44140385	3.52442099	3.72687346	3.61575873	3.57392949	3.77688038	3.43638454	3.74861176
NONHSAG050259	2.31416983	2.28409169	2.28109781	2.54326744	2.3147172	2.26810426	2.48089542	2.26465897	2.2852434
NONHSAG050280	4.91429072	4.69249657	4.54843618	4.93719548	5.07098778	4.83925495	4.6423199	4.79789137	4.4767178
NONHSAG050284	4.25178442	3.88168666	4.14353714	4.1379176	4.03335295	3.97049553	4.11528443	4.45788224	4.25892833
NONHSAG050285	4.19156208	4.09908444	4.31402733	4.1544932	4.28584337	4.16593794	4.35947043	4.20321687	4.42573071
NONHSAG050296	5.14716301	5.09179477	5.08625145	5.26968896	5.27169219	5.21471672	5.07746519	5.0197105	4.6917183
NONHSAG050300	3.5730385	3.5816287	3.73460381	3.5070524	4.05899827	3.3981437	3.75783268	3.50764476	3.64323103
NONHSAG050305	5.33832873	5.73278714	5.41594588	5.76232149	5.42168469	6.59076687	5.74376841	5.7678945	6.17735341
NONHSAG050335	4.14418716	4.3518349	4.24156064	4.72160928	4.39293193	4.32680191	4.31968374	4.28209439	4.40295754
NONHSAG050348	2.42730748	2.38836269	2.5081571	2.25755681	2.36223906	2.58002691	2.61787705	2.60430853	2.50752334
NONHSAG050370	3.09991819	2.96199934	3.08651772	3.25928467	3.2050008	3.16335005	3.05050109	3.0884409	3.44297973
NONHSAG050372	4.24117408	4.10634039	4.35727318	4.32383598	4.2331907	4.36483837	4.23745594	4.23528504	3.87474462
NONHSAG050397	4.37147006	4.21622778	4.26275089	3.95473512	4.28135173	4.23053818	4.4910142	4.62336972	4.0844779
NONHSAG050419	5.90151379	6.15033845	5.95011091	6.2216656	5.79050591	6.11430755	5.98722833	6.16986148	5.8862983
NONHSAG050438	2.09404947	2.14858749	2.12457102	2.19038818	2.04072436	2.22412854	2.26433888	2.35015175	2.2084722
NONHSAG050440	2.4198718	2.32467891	2.43060927	2.56424435	2.50954055	2.53126423	2.54865079	2.70509404	2.46473743
NONHSAG050454	3.46097718	3.31375674	3.5966467	3.39426408	3.48875248	3.5334082	3.38413285	3.37421961	3.73786616
NONHSAG050461	2.59779081	2.40646709	2.69846519	2.57673419	2.60157298	2.47203889	2.61501003	2.37131503	2.54652372
NONHSAG050473	6.74231249	6.56960595	6.6594861	6.58847253	6.89135276	6.67001461	6.98236593	6.41605335	6.73050648
NONHSAG050503	4.41224728	4.04792474	4.42880435	4.58800118	4.39623098	4.3989099	4.41181903	4.34821852	4.38466528
NONHSAG050531	2.93714284	2.82184029	2.93374334	2.95688067	2.91405767	3.14898801	3.12886144	2.96108651	3.11061694
NONHSAG050576	3.37379786	3.31899149	3.43286484	3.39718484	3.27321071	3.12121764	3.24391248	3.37001972	3.26966202
RP11-48B3.4	6.72025663	6.84714548	6.04506488	6.43565027	6.32998742	6.81746177	6.89652689	6.96990385	6.73714522
NONHSAG050607	3.00910977	3.3642677	3.25099741	3.20559791	3.24486919	3.20158845	3.16954672	3.54168466	3.32076472
NONHSAG050643	2.05075286	2.12149935	2.20060806	2.33571918	2.16524535	2.2409691	2.203556	2.16733969	2.65141191
NONHSAG050690	9.74190851	9.89052887	9.72556386	9.6579501	9.8189025	10.21472449	10.01076191	10.01467125	9.92222188
NONHSAG050699	5.76363741	5.83902401	5.89027701	6.03808999	5.66128256	5.81985864	5.95067659	6.21049718	6.23246302
NONHSAG050714	2.63018897	2.91540359	2.75033486	2.79316542	2.69335043	2.82461003	2.87422051	2.77183818	2.90924823
NONHSAG050718	4.81022754	5.41443713	4.91992971	4.93275349	4.86127995	5.43047383	4.83904024	5.00097853	4.77042594
NONHSAG050752	2.49690508	2.52051266	2.66141352	2.37628602	2.38495232	2.5742384	2.25553317	2.41471087	2.32896215
NONHSAG050804	6.54846272	6.52896112	6.41594195	6.46066336	6.23142766	6.68235875	6.58420013	6.67626461	6.26506937
NONHSAG050806	5.8489566	5.63417653	5.69217983	5.73937413	5.84208567	5.88030534	5.80285055	5.74641105	5.82374735
NONHSAG050815	2.66614839	2.82117965	2.69553278	2.65796879	2.70799584	2.43512024	2.62206754	2.89073571	2.78316798

NONHSAG050829	2.51046679	2.28867714	2.45976003	2.42304389	2.17968954	2.43595482	2.4512015	2.92769605	2.65077094
NONHSAG050841	3.9291123	4.01237159	3.72372536	3.78621227	3.96358086	3.94645937	3.86683938	3.89344751	3.8098662
NONHSAG050865	7.56223836	7.73697253	7.76617845	7.70578907	7.6088231	7.74160033	7.76440446	7.74279443	7.74450308
NONHSAG050866	5.8011013	6.01964403	5.85301058	6.23235088	5.59852936	6.06681788	5.8147553	5.87271281	5.58309411
NONHSAG050902	3.78199928	3.70693629	3.95834736	3.65973855	4.11157806	3.8018351	3.95036382	3.68744126	3.88020545
NONHSAG050903	7.53502931	7.79852915	7.29697416	7.3399274	7.38097301	7.34913144	7.38293656	7.49029501	7.33599038
NONHSAG050913	5.46709596	5.43603329	5.38002504	5.55790703	5.48089406	5.6289008	5.68926001	5.44230558	5.5879142
NONHSAG050914	3.19168464	3.1887839	3.03118296	3.12724296	3.16436571	3.31861065	3.13457185	3.20823287	3.40958107
NONHSAG050915	4.46012623	4.82452637	4.58240378	4.58367754	4.48210725	4.70730661	4.60744224	4.4571856	4.74497948
NONHSAG050921	3.09805493	3.00341964	3.10202983	3.1922346	3.31427015	3.01966474	3.13206449	3.03756427	3.20103002
NONHSAG050966	4.94797321	5.38609045	4.76972983	5.25444126	5.01559066	5.34258163	5.0286462	5.45922115	4.60496938
NONHSAG050969	2.91017356	2.77453622	2.81934155	3.04621706	2.91575871	2.87555482	3.06961943	3.26860571	2.92405079
NONHSAG050970	3.22235076	2.97318064	2.97778593	3.21417922	3.21684398	3.3206808	3.20738399	3.19773309	3.28486673
NONHSAG050978	3.7470159	3.89989002	3.86518595	3.80499456	3.76461245	3.72753195	3.64665105	3.88784105	4.06130302
NONHSAG050982	6.17249395	6.37909845	6.05109995	6.08574211	6.03491741	6.34725284	6.44018563	6.39299965	6.46775691
NONHSAG051053	3.9824164	3.95955028	3.99604309	4.11418995	4.31424066	4.30987455	4.23073435	4.03968088	4.25722604
NONHSAG051068	2.07223132	1.99169887	2.14317182	2.33368383	2.19522593	2.1536669	2.07391005	2.31839226	2.1148234
NONHSAG051082	5.85789213	5.89259725	5.92467358	5.97955431	6.36555619	6.5130383	6.32573506	6.22427353	6.46265065
NONHSAG051104	4.481121	4.50177412	4.56683275	4.59074606	4.50786411	4.37543137	4.37249046	4.33052524	4.40527299
NONHSAG051105	3.59548957	4.23573302	3.48163395	3.81140105	3.85686823	4.15386169	3.59217212	4.00639845	4.04494261
NONHSAG051129	3.35687784	3.04510708	3.28101343	3.1563115	3.29973324	3.22336781	3.13173048	3.12943901	3.32076472
NONHSAG051137	3.91476485	4.00131573	3.90605573	4.13861695	4.06110475	4.04948642	3.85252896	3.79748025	4.17806758
NONHSAG051145	3.88533811	3.95103364	4.00002049	3.73405713	4.16207448	3.71947681	3.90505698	4.07779687	4.30897267
NONHSAG051156	2.31267476	2.39283173	2.48762131	2.48974334	2.58849731	2.63130616	2.28181335	2.49560856	2.54950682
NONHSAG051181	3.43115778	3.18121015	3.43070965	3.3605179	3.30566764	3.51218457	3.78868236	3.48789273	3.39953202
NONHSAG051194	3.09155255	3.1725505	3.05504565	3.26187886	3.07931485	3.25225286	3.18427621	3.17457848	3.11736304
NONHSAG051196	5.20225707	5.51512132	5.46443073	5.5834741	5.16871225	5.48750444	5.4376496	5.28738689	5.10815544
NONHSAG051205	4.15689312	3.99119668	4.04852823	4.12030072	4.17017594	4.21769606	4.49870867	4.30579696	4.91399869
NONHSAG051207	2.35889243	2.86079209	2.66846073	2.3725069	2.6830558	2.83382677	2.79866152	2.70862772	2.95685576
NONHSAG051233	2.32651135	2.43702906	2.39893946	2.76021357	2.55348835	2.43230761	2.32074165	2.41671531	2.75061511
NONHSAG051237	2.54460488	2.42602322	2.56149261	2.54438992	3.01276379	2.8571329	2.29978495	2.90588002	2.63502595
NONHSAG051255	3.25964816	3.41237256	3.21670839	3.26373726	3.36710221	3.54107499	3.457863	3.60811519	3.48252669
NONHSAG051258	4.7874483	5.15390756	4.8717988	4.97737029	5.05215087	4.99038087	5.12659728	4.94244366	4.80904478
NONHSAG051259	3.46953267	3.35618755	3.31329181	3.60744402	3.46781468	3.67199962	3.6931222	3.63058623	3.68228572
NONHSAG051263	3.36885183	3.01085913	3.09199213	3.33298871	3.32326401	3.34353905	3.24339089	3.20788478	3.02386339
NONHSAG051266	4.25755754	4.29346233	4.25999545	4.2302007	4.33440754	4.18163119	4.26127021	4.20833241	4.34028642
NONHSAG051273	2.73497324	2.53345067	2.7553394	2.65556077	2.53497944	2.60445349	2.43267192	3.05918276	2.53634066
NONHSAG051275	2.65563627	2.1763374	2.4297217	2.4258846	2.47390812	2.32925487	2.41691165	2.43802592	2.32594066
NONHSAG051297	2.50588027	2.56867948	2.6977054	2.68056301	2.78029557	2.4767913	2.71482014	2.67029735	2.84803827
NONHSAG051305	3.0102001	3.0952954	3.22877169	3.07000522	3.11456214	2.77840487	2.98352008	3.10525201	3.14057123
NONHSAG051327	5.22732118	5.01998408	5.17577091	5.05404794	5.03333324	5.24686559	5.11298018	5.21263179	5.26926111
NONHSAG051334	2.95316114	2.91600955	2.71553713	2.86741267	3.05655349	3.14578815	2.89485173	3.05217205	3.09173622
NONHSAG051339	5.34221111	5.09809953	5.27471876	5.36437439	5.23146834	5.20445702	5.10857547	5.04111131	5.31067981
NONHSAG051345	3.23278383	3.1547713	3.26410977	3.09817287	3.2465072	3.26488541	3.09302772	3.20171158	3.34463724
NONHSAG051356	4.55797744	4.42948342	4.44485297	4.39960864	4.56442951	4.67801843	4.51049913	4.44084824	4.40722263
NONHSAG051375	3.06772141	2.99383813	3.11624691	3.14955237	3.00231064	3.06202306	3.37261156	3.14885361	3.17169593
NONHSAG051392	3.37100087	3.17974555	3.13498572	3.52850311	3.44582747	3.65126578	3.46694924	3.38961709	3.24858713
NONHSAG051400	6.74208811	6.73725013	6.78415664	6.73487156	6.80169484	6.75963053	6.89436612	6.75673593	6.89026887
NONHSAG051424	5.89656294	5.88916164	5.91246652	5.96456855	6.13196122	6.03738673	6.22676072	6.00186748	6.10252683
NONHSAG051435	4.31971769	4.15181837	4.41449496	4.13662182	4.4363643	4.38474506	4.2739768	4.42620265	4.4952012
NONHSAG051445	5.62971188	5.53947818	5.64837429	5.56769429	5.78382792	5.58689121	5.76480517	5.51987185	5.86649774
NONHSAG051446	4.41095398	4.6042117	4.59927873	4.92757367	4.82839779	4.34006949	4.51466272	4.57524196	4.6266254
NONHSAG051464	4.87462098	4.82990602	4.61489729	4.85298887	5.22630498	4.96926459	4.90722942	4.64408828	5.12689581
NONHSAG051484	5.65638508	5.58958257	5.670245	5.66489844	5.48224525	5.64639054	5.51830028	5.50633317	5.43491203
NONHSAG051497	5.15149771	5.25312272	5.27991892	5.31889956	5.48760038	5.13353417	5.07440323	5.04640987	5.46043642
NONHSAG051574	4.98771645	4.79174399	5.11993288	4.69454233	5.05965186	4.80291378	5.12968802	4.88242487	4.98617509
NONHSAG051638	7.23845531	7.32489741	7.14738808	7.25266263	7.21219466	7.39699402	7.1602907	7.29115731	7.08033668
NONHSAG051650	2.96371777	3.24763772	3.16993126	3.48916597	3.02620071	3.76433054	3.50455047	3.35239371	3.81942519
NONHSAG051658	5.3625506	5.14116668	5.39304249	5.35815001	5.57514415	5.55281424	5.3591756	5.32456198	5.55453356
NONHSAG051661	2.71667357	2.4793929	3.11617035	2.96136075	3.03179285	2.81138724	2.84150424	2.70267608	3.22139233
NONHSAG051677	4.59500931	4.91207907	5.07489025	4.88484079	4.97565988	4.77627431	4.73358547	4.83172228	4.88963563
NONHSAG051728	3.39696064	3.55121851	3.69520597	4.0556232	3.65739409	4.02498166	3.78669888	3.89676042	3.66874185
NONHSAG051730	3.09150202	3.37371374	3.29630728	3.30699182	3.23034685	3.0273715	3.26536043	3.19743627	3.59020067
NONHSAG051770	2.6231458	2.56761435	2.62474825	2.38661843	2.33456429	2.37835585	2.44618396	2.33483328	2.69078516
NONHSAG051806	2.75859534	2.86940223	2.70945661	3.03113558	2.86304089	3.31845115	2.86164249	2.85709235	2.67683939
NONHSAG051813	5.65119114	5.66793835	5.40313444	5.11135727	5.25310878	5.48424248	5.63690802	5.36657723	5.50483562
NONHSAG051826	2.56476359	2.73619806	2.63902115	2.813025	2.70686627	2.723553	2.70511592	2.80011169	2.69444485
NONHSAG051866	2.52461121	2.49093516	2.55944341	2.46051261	2.46099415	2.38807313	2.28980837	2.43342859	2.66510011
NONHSAG051885	2.3619195	2.66802856	2.47365259	2.49178527	2.54574429	2.41539632	2.46600602	2.60723969	2.48189521
NONHSAG051943	3.07057711	3.11304567	2.95728334	3.02967356	3.18650253	3.23694949	3.17934876	3.34518463	3.23685166
NONHSAG051945	2.47241464	2.5644211	2.64333034	2.90952541	2.71079011	2.31564503	2.69583081	2.70099103	2.6947427

NONHSAG051962	3.99860354	3.93861075	4.2402768	4.09210777	4.28722223	4.19337268	4.38905055	3.97597514	4.16457026
NONHSAG051968	2.2386161	2.26568162	2.284892	2.40478818	2.49067177	2.46589831	2.28484946	2.28193811	2.2310491
NONHSAG051985	2.68310593	2.66148085	2.90206517	2.84729043	2.64977583	2.65740673	2.97655053	2.61895221	2.75203047
NONHSAG052010	3.31960573	2.94193347	3.16053632	2.95100947	3.40681296	3.28314546	3.10408599	2.94120787	3.02926594
NONHSAG052012	6.53769635	6.4677653	6.67322893	6.63892078	6.62680331	6.64784083	6.57798036	6.36593391	6.43614455
NONHSAG052042	3.27591072	3.13601041	3.32126381	2.85738434	3.17003044	2.96963533	3.01891145	2.94031346	3.02890683
NONHSAG052090	2.77998741	2.6992253	2.6571729	2.94275256	2.66463834	2.6416895	2.7584245	2.7032451	2.79208517
NONHSAG052112	4.0329088	3.87176734	3.82427238	3.92055061	3.73259449	3.90753948	3.87676095	3.94334366	3.4074779
NONHSAG052118	4.40500021	4.6698032	4.82373608	4.46522811	4.60004657	4.23633129	4.54758533	4.29957199	4.69264273
NONHSAG052125	8.70899902	9.02526963	8.85821931	8.95503973	8.90126248	8.8417805	8.81646736	8.90443218	8.89743467
NONHSAG052126	3.82281847	3.93518578	3.23229341	3.56213412	3.44591116	3.67954978	3.47525451	3.92196119	3.5166322
NONHSAG052157	3.68721417	3.63599511	3.90981921	3.7480626	3.82332684	3.98140583	4.07730843	3.71361808	3.96553163
NONHSAG052162	5.18491138	5.51740757	5.36402389	5.18726042	5.38646006	5.61653815	5.77681256	5.3977428	5.76030491
NONHSAG052178	6.41320823	6.62142021	6.30855041	6.29212288	6.2323995	6.51086382	6.34141934	6.44193735	6.41728405
NONHSAG052186	6.53704339	6.68950367	6.50819569	6.52690057	6.59222114	6.63852857	6.64962287	6.52864167	6.47904718
NONHSAG052188	4.25841208	4.15280426	4.12990253	4.18644465	4.19762521	4.29549694	4.23955485	4.29144607	4.36561402
NONHSAG052409	5.86016941	5.71445856	5.76356696	5.73869773	5.65347954	5.71107324	5.84380997	5.87813998	6.02426592
NONHSAG052414	4.6741579	4.74688517	4.77932849	4.66673275	4.65749316	5.33277293	4.55550151	4.78337106	5.07114343
NONHSAG052449	7.43125206	7.59890539	7.43382737	7.35933619	7.25780288	7.38226431	7.48056521	7.53135797	7.38957146
NONHSAG052499	3.78498814	3.82972794	3.88022576	3.84433165	3.7993193	3.7138086	3.73542289	3.68494546	3.66941711
NONHSAG052508	5.01034501	5.04054634	5.13834637	5.14496534	5.01889009	5.08747815	5.3278138	5.10572292	5.24167299
NONHSAG052530	3.33550801	3.20447109	3.4127079	3.75911506	3.49910674	3.36386806	3.43109013	3.43654487	3.5707971
NONHSAG052547	5.85299008	5.7419436	5.57709822	6.01936155	5.92019882	5.92382786	5.87069126	5.960324	5.60975663
NONHSAG052583	2.50538696	2.77973238	2.40159002	2.45826708	2.64023239	2.31645639	2.56803529	2.31634238	2.35880576
NONHSAG052586	2.20346522	2.22781293	2.00905451	2.18106628	2.19064289	2.20766717	2.06122374	1.99810781	2.14453192
NONHSAG052609	2.76671945	3.34548317	3.13616236	2.76006397	3.2275083	3.1860262	3.03466087	3.46495337	2.85494097
NONHSAG052620	5.44561555	5.74358048	5.5381383	5.72563972	5.76102543	5.72916291	5.59247912	5.46789082	5.14858586
NONHSAG052621	3.52575894	3.5701876	3.43317909	3.69633707	3.56905559	3.66920763	3.50904842	3.55910317	3.56563774
NONHSAG052627	5.88470861	6.21297404	6.00676266	6.02073441	5.8726189	5.9565457	6.1463758	6.27874929	6.0139728
NONHSAG052636	4.49380076	4.18740082	4.32157575	4.22286355	4.55835899	4.49623532	4.3957937	4.15466666	4.32323903
NONHSAG052644	3.42259076	3.46702297	3.55890225	3.46198773	3.38437897	3.64675076	3.62959484	3.33678869	3.569559
NONHSAG052726	5.1332624	4.92450652	5.0758177	4.86973079	5.04359992	4.98297991	5.14569568	4.95379281	4.53276282
NONHSAG052734	3.72843773	3.55056304	3.66600863	3.76042013	3.93988781	3.84311819	3.64128168	3.75751835	3.7029857
NONHSAG052752	5.91728497	5.90205028	5.69214023	5.86098081	5.47112966	6.00136156	6.06631017	6.19441949	5.79212459
NONHSAG052756	2.66922889	2.81398848	2.78592318	2.99352025	2.72752575	3.1295547	2.71111346	2.6726992	2.82817104
NONHSAG052760	4.87371281	5.30796611	4.20535979	4.78002149	4.77956952	4.93797675	4.83433282	4.91243656	4.53491258
NONHSAG052772	6.38767162	6.18592221	6.40244042	6.3829829	6.46627807	6.50925717	6.48282893	6.23941618	6.44327289
NONHSAG052776	5.45685795	5.18125657	5.620282	5.4183363	5.51289874	5.44857151	5.48685913	5.40221886	5.68460877
NONHSAG052817	4.32403251	4.276459	4.24273329	4.22717579	4.29122253	4.41453534	4.96479578	4.73085504	4.41391182
NONHSAG052818	3.17105282	3.31359857	3.41434046	2.83682022	2.89793564	3.65373594	2.64221817	2.85360032	3.14106383
NONHSAG052837	6.13172024	6.0494968	5.87723658	5.89276066	5.99086084	5.90524385	6.12523267	5.92224021	6.47327464
NONHSAG052879	4.57636705	4.27684409	4.33363021	4.52727626	4.49528299	4.58256987	4.39262246	4.33451254	4.24794902
NONHSAG052880	5.60902965	5.79028462	5.46036794	5.61326684	5.47335601	5.67462208	5.53120903	5.51118716	5.4221214
NONHSAG052885	4.61128694	4.5610185	4.55039496	4.40682883	4.87390301	4.41406535	4.63287518	4.83850735	4.80322293
NONHSAG052886	4.34351803	4.32293818	4.54999066	4.55153615	4.40919705	4.55783147	4.52610193	4.2637737	4.50781935
NONHSAG052890	6.13287784	6.18145231	6.06652364	6.28684987	6.19585119	6.10495093	6.09302604	5.98732248	6.08310388
NONHSAG052915	6.4588935	6.39374785	6.53129684	6.47148225	6.1652751	6.40463349	6.71818565	6.35916493	6.37998523
NONHSAG052978	3.40639199	3.39040876	3.5889736	3.29390485	3.36997442	3.5223536	3.65508152	3.4408426	3.37245603
NONHSAG052998	2.71472465	2.61011814	2.63206951	2.94019902	2.63394462	2.81030375	2.79668849	2.57405395	2.73798278
NONHSAG053000	2.63923175	2.62149938	2.77987405	2.98700324	2.72752575	2.64444413	2.91599463	2.80095597	2.52381856
NONHSAG053032	4.2753043	4.39644402	3.81707343	4.05524015	3.52361345	3.88501572	4.06611475	4.03858507	4.24243248
NONHSAG053081	2.28262148	2.50353022	2.4113903	2.40478818	2.34176303	2.44117489	2.44270148	2.56017213	2.36500142
NONHSAG053118	2.12154542	2.31561907	2.40850059	2.45173094	2.30271561	2.42363873	2.12167629	2.22571153	2.31297933
NONHSAG053119	2.5665071	2.66715197	2.69464582	2.64737333	2.53363954	2.53161553	2.616273	2.62722838	2.5935068
NONHSAG053167	3.95944269	4.20931117	4.0385517	3.90972338	3.95520241	3.99011732	4.03130496	3.99930201	4.06275241
NONHSAG053168	2.25402432	2.3013382	2.62894202	2.49918635	2.48822177	2.27019012	2.35349098	2.52456942	2.62654311
NONHSAG053205	4.93569676	5.42016656	4.85541465	5.07953513	4.80719737	5.04981654	4.88496582	5.11592904	4.63649999
NONHSAG053209	2.21654997	2.18169106	2.45893837	2.25118488	2.51101864	2.30506229	2.5864483	2.50741238	2.51101864
NONHSAG053219	6.65743652	6.55193834	6.75803384	6.74375278	6.757668	6.82233038	6.78839705	6.65914179	7.03105562
NONHSAG053240	3.48490542	3.3178105	3.40654473	3.43847186	3.57344868	3.43877767	3.31232234	3.48023097	3.29606745
NONHSAG053246	4.75759498	4.43544234	4.7947023	4.4425805	4.70137849	4.58805303	4.63382126	4.70859572	5.07986232
NONHSAG053259	3.78885524	3.50275841	3.78600666	3.56577553	3.98354271	3.75317456	3.72533067	3.54037808	3.84829258
NONHSAG053273	3.90016896	3.59632614	3.82040205	4.00920797	3.89111161	4.04427229	3.35023034	3.59473298	3.9323322
NONHSAG053274	2.86623424	2.89213286	2.7356388	2.75135217	2.90175189	3.01078776	2.76465367	2.78037633	2.94004895
NONHSAG053293	4.22963183	3.98626989	4.22939843	4.39946888	4.05062498	4.12873581	3.97757956	4.05253241	4.10646985
NONHSAG053296	4.03281127	4.02677144	3.9869852	3.95595219	4.09997053	3.98774587	3.90357177	3.96435269	4.28078009
NONHSAG053299	3.37124066	3.48404235	3.37640376	3.27021987	3.51021968	3.59460311	3.23277796	3.28795016	3.87246688
NONHSAG053306	5.18892776	5.07224914	5.17512037	5.43526479	5.38782498	5.39978166	5.3491458	5.03793451	5.30153113
NONHSAG053322	5.30725638	5.21167194	5.48412355	5.31070925	5.4747411	5.21031351	5.56620954	5.10950427	5.51927368
NONHSAG053329	2.75771875	2.7773807	2.98628413	2.5637207	2.83601921	2.57284788	2.73869245	2.7455334	2.5758998
NONHSAG053359	5.50238384	5.6095518	5.28141473	5.58346496	5.6095518	5.77952844	5.69114347	5.6761518	5.70198759

NONHSAG053361	4.07530778	4.27823328	4.42399859	4.26522831	4.58105312	4.46473605	4.29329212	4.23399015	4.59483104
NONHSAG053376	6.17336181	6.47087059	6.16344596	6.43532477	6.25799588	6.19452633	6.29028476	6.31201063	6.1226323
NONHSAG053377	6.66930401	6.3720878	6.50096379	6.39070747	6.65906898	6.35459718	6.4456168	6.41603385	6.50648943
NONHSAG053380	6.12922345	6.31679261	6.01546868	6.09355666	5.9769343	5.98322714	5.99295983	5.99164739	5.97575631
NONHSAG053392	2.76936505	2.64353752	2.82826257	2.76028008	3.02597533	2.71728697	2.81210396	2.82309966	2.7986882
NONHSAG053406	4.57508911	5.02182885	4.17718444	4.75100015	5.16269648	4.7199629	5.00063512	4.82826878	4.89123507
NONHSAG053407	4.51619469	4.5929585	4.487379	4.33866051	4.53151114	4.41687914	4.53679501	4.54359693	4.81195844
NONHSAG053417	4.12616861	4.15673122	4.0354015	4.50390467	4.28154578	4.25289307	4.31374792	4.44340424	4.35952362
NONHSAG053440	3.973938	4.14658453	3.9628655	3.77836856	4.08547844	4.25565065	4.12978565	4.09969219	4.04491839
NONHSAG053450	3.27198365	3.0230702	3.22653823	3.14252941	2.96378654	3.4728521	3.30400276	3.22963449	3.16961103
NONHSAG053454	2.97163478	2.95596501	3.24056014	2.99237894	2.89482225	2.98951468	3.09474412	2.76185604	3.3390305
NONHSAG053462	3.36339704	3.6012565	3.53453522	3.62495414	3.65973955	3.72617886	3.3812605	3.75858689	3.64870592
NONHSAG053469	3.55440774	3.63758126	3.55026625	3.79430614	3.62987099	3.71947681	3.62419052	3.67246022	3.81004156
NONHSAG053552	6.28080112	6.58673737	6.36399639	6.45114158	6.44637463	6.25545919	6.2336299	5.87492894	6.57802177
NONHSAG053577	7.2028854	7.29193105	6.84528115	7.2531605	7.26184158	7.22754069	7.46428252	7.54719253	7.1991645
NONHSAG053585	4.83549054	4.77759929	4.91270756	4.8115771	4.84215433	4.75192827	4.74767941	4.80299461	4.76855612
NONHSAG053586	7.34075316	7.25545556	6.90892194	7.222564	7.1015572	7.19237581	7.386036	7.4956336	7.41141393
NONHSAG053589	6.10498122	6.14155041	6.37274378	6.23749161	6.38822536	6.42643752	6.41832462	6.32615344	6.89682212
NONHSAG053591	5.27658743	5.35687367	5.55090568	5.40360872	5.58798446	5.29466639	5.37731891	5.42419604	5.50785245
NONHSAG053607	4.15271147	4.08860076	4.42712338	4.26456809	4.16772135	4.32194255	4.14839475	4.0172061	4.1844782
NONHSAG053614	5.56354826	5.49736467	5.59207169	5.66435527	5.56509801	5.53955777	5.34926614	5.35940212	5.37597439
NONHSAG053675	5.12415338	5.24322014	5.08924721	5.06527308	5.00148466	5.33698054	5.14008506	5.04153393	5.32217403
NONHSAG053719	2.68320536	2.53252036	2.58632078	2.65277444	2.87732556	2.62078465	2.58068766	2.64692702	2.52719503
NONHSAG053722	3.48773763	3.49962126	3.62983685	3.33633986	3.3041399	3.46513542	3.19703162	3.30666218	3.74541897
NONHSAG053746	4.10869907	3.99385254	4.0683684	4.01264949	4.12732883	4.44296119	4.05840505	4.07014314	4.45417722
NONHSAG053751	3.79212339	3.94332033	3.93706555	4.11309065	4.12456274	3.84992562	4.15308079	3.91177014	4.09593456
NONHSAG053784	5.65767754	5.6684997	5.60266526	5.70539715	5.81232478	5.5841805	5.6502715	5.69086506	5.72577789
NONHSAG053801	4.06564643	3.87958386	4.25095186	4.32532823	4.31565375	4.10428442	4.08154772	4.04827935	4.14957011
NONHSAG053821	2.77311791	2.83665014	2.85351354	3.00599103	3.12115859	2.85219312	2.84141495	3.0743794	2.99098656
NONHSAG053863	3.9102785	3.810898	3.96529169	4.08618163	4.03338	3.91447688	4.06179185	3.88974943	4.01738253
NONHSAG053877	4.83407783	4.90625305	5.20390268	4.85771282	5.24172865	4.84243468	4.85754112	5.03546355	5.0566965
NONHSAG053892	5.32149184	5.38081587	5.14154463	5.05721564	5.43688053	4.99464932	5.32974596	4.82224217	5.2667754
NONHSAG053893	13.98330959	13.93348717	14.02815097	13.93348717	14.0274128	13.96341396	13.87871083	13.89446186	13.95601326
NONHSAG053895	14.19062022	14.15754566	14.29914849	14.20239667	14.28563825	14.16878143	14.02797873	14.13561425	14.20890438
NONHSAG053900	2.56771839	2.70494705	2.34445403	2.49906389	2.30467519	2.52620917	2.4034445	2.67606933	2.60929493
NONHSAG053985	2.84519403	2.94097052	2.79303642	2.60767756	2.79231459	2.88289899	2.97998459	2.81643777	3.01339958
NONHSAG054015	2.15447376	2.20228237	2.45940695	2.43315242	2.46476576	2.54382142	2.58825113	2.29215121	2.46564337
NONHSAG054016	2.74494046	2.54983915	2.68048326	2.65076824	2.65998991	2.73823572	2.70153392	2.7348408	2.97875958
NONHSAG054030	2.53987584	2.86272186	2.59901064	2.60903553	3.08015404	2.89670079	2.76959235	3.10192825	3.06745865
NONHSAG054071	2.57322184	2.73798353	2.65591899	2.9304063	2.69801179	2.81153775	2.98472128	3.2352385	2.82499129
NONHSAG054103	3.00518807	2.79920026	2.93490116	3.12033075	3.14638061	3.34105517	3.05313247	3.07996659	3.15599777
NONHSAG054109	2.44839321	2.62902128	2.5362314	2.79254167	2.63064984	2.7764432	2.58552619	3.18972731	2.85263574
NONHSAG054140	2.84454376	2.73379122	2.74082689	2.93859613	2.71955398	2.88715611	2.86166037	2.97116321	2.82758378
NONHSAG054179	4.2389023	4.11872109	4.37391758	4.46589109	4.30057431	4.3404942	4.28070425	4.48247937	4.22035881
NONHSAG054188	3.42930827	3.19058278	3.32326546	3.31255735	3.52031805	3.38357305	3.57890345	3.18972731	3.30795234
NONHSAG054199	5.80592649	5.8369389	6.13397869	6.04769362	6.11025364	5.98359811	6.09788568	6.01787812	6.04214546
NONHSAG054326	2.76771125	2.77762391	2.8081015	3.05771884	2.94741301	2.87745723	2.61792473	2.79185945	2.81432198
NONHSAG054344	2.33707676	2.21983472	2.16894163	2.41814766	2.34447599	2.23850764	2.23949926	2.0369078	2.09906191
NONHSAG054345	3.56849163	3.24000419	3.35494396	3.31876343	3.38931049	3.41431869	3.40303629	3.45280682	3.42820792
NONHSAG054445	3.59607882	3.49204569	3.61976889	3.81936932	3.72222467	3.87388305	3.5869608	3.62999713	3.88244617
NONHSAG054462	4.33841186	4.39246717	4.35333632	4.53023587	4.51496561	4.45624807	4.46830815	4.34476434	4.55041348
NONHSAG054468	4.72828213	4.56447934	4.77300689	4.91240003	4.61297735	4.7935632	4.57304128	4.71168318	4.38788475
NONHSAG054478	2.56947789	2.84613939	2.44813063	2.78140418	2.55027199	2.7764553	2.70609919	2.62722838	2.63418604
NONHSAG054549	6.02665843	6.17479254	6.15204806	6.18057637	6.23450694	6.23861862	5.97506967	5.81371529	6.07079053
NONHSAG054576	5.62112283	5.53573049	5.61746487	5.53711206	5.7029591	5.57449333	5.61586948	5.53624119	5.56217531
NONHSAG054653	5.74749831	5.88734134	6.23948617	5.81093063	5.24252306	5.77595477	5.74854593	5.66866475	5.76146403
NONHSAG054655	4.26448717	4.43511147	4.16772595	4.58923776	4.50218454	4.12963604	4.19428917	4.33247935	4.48369813
NONHSAG054664	3.32323244	2.72898638	3.32365994	2.99448907	3.02393735	3.41863614	2.93950135	3.18983907	2.92992229
NONHSAG054671	3.04686076	3.59295589	3.1176682	3.11073583	3.00868377	3.04240212	3.06159951	3.16087983	3.07431631
NONHSAG054679	2.59077908	2.50118022	2.67821724	2.64400403	2.6013629	2.63222185	2.80154368	2.329716	2.80287348
NONHSAG054729	6.25712396	6.20845449	6.20994925	5.9304047	6.30621358	5.79375374	6.16257053	6.22857862	6.03835034
NONHSAG054754	4.0375283	4.13221989	3.7609839	4.00023674	3.77351712	4.37227846	4.13552027	4.33462919	4.0454747
NONHSAG054784	6.51907489	6.48372627	6.30319126	6.03668323	6.19708798	6.48151452	6.39055228	6.49766536	6.40741954
NONHSAG054798	6.23107307	6.43181755	5.99583187	6.0595551	6.06722621	6.49270866	6.44643386	6.39107203	6.69219005
NONHSAG054804	7.79371258	7.92731833	7.73900574	8.03888322	7.98265596	8.42910573	8.05441583	8.1145708	8.34055401
NONHSAG054869	3.98986319	3.71879987	3.89987201	3.86910251	3.84549732	3.71585309	3.77368101	3.89218224	3.78669888
NONHSAG054870	6.73064442	6.52787983	6.76530401	6.54029352	6.78250948	6.57096706	6.63082066	6.5825683	6.73570266
NONHSAG054897	2.40778073	2.38716169	2.61501003	2.3975615	2.53719988	2.30726105	2.39861284	2.32328707	2.55534178
NONHSAG054929	3.307115	3.05108191	3.1624926	3.35510754	3.67267112	3.27205466	3.11617322	3.17162499	2.9979247
NONHSAG054954	3.19122189	2.96034143	3.1468672	3.44974987	3.03728014	3.21054596	3.26148136	3.17712876	3.13199506
NONHSAG054958	3.68710539	3.53456886	3.88313234	3.63936608	3.74536709	3.88764267	3.65479886	3.55338519	3.80219123

NONHSAG054963	4.76018869	4.73586818	4.91128174	4.53200184	4.87162745	4.66691035	4.74185716	4.72970319	5.24651142
NONHSAG055008	2.28650771	2.27997031	2.44188076	2.47831494	2.57053647	2.44924217	2.4750264	2.76297455	2.79165766
NONHSAG055019	2.72062878	2.98808898	2.97060434	2.81918894	2.79368938	3.25247177	3.12746806	3.02101031	2.88676126
NONHSAG055021	3.66371841	3.72365726	3.61141028	3.79097041	3.51438014	3.57403495	3.73936152	3.59948562	3.59965994
NONHSAG055050	2.97165074	2.9467137	2.95707477	2.947633	2.93429478	3.19678	2.99967086	3.45167353	2.84444729
NONHSAG055086	2.63636245	2.72658456	2.60842855	2.88757809	2.7478408	2.80355924	2.79973516	2.9573645	2.647274
NONHSAG055105	2.22825723	2.41270543	2.3488071	2.34650828	2.41615396	2.39036119	2.38633692	2.46494792	2.29845109
NONHSAG055108	4.86404653	4.57064467	4.75632568	4.53855472	4.79488545	4.37626081	4.42216672	4.23111325	4.77971179
NONHSAG055153	3.084633	3.05784543	3.01839061	3.34376067	3.15861886	3.07144384	3.14387179	2.82312105	3.03448033
NONHSAG055204	3.46795958	4.05432724	3.21670839	3.53660899	3.3783709	3.85304905	3.49341666	3.71002905	3.38656373
NONHSAG055266	5.18572467	4.98690572	5.12836361	5.12375268	4.80853519	5.1524416	5.25476886	5.21179887	5.30123274
NONHSAG055280	2.35036196	2.22053106	2.55489543	2.56424435	2.47978111	2.48052407	2.54644303	2.69309452	2.47603047
NONHSAG055289	5.61770048	5.68755765	5.68300073	5.84674617	5.64840932	5.78044116	5.64856636	5.85220934	5.5492843
NONHSAG055301	3.29431073	3.03667181	3.63549839	3.30670818	3.91019016	3.62579043	3.41206965	3.33368835	3.91527048
NONHSAG055339	5.108968	5.09021628	5.14841787	5.14968965	5.20061086	5.15730071	5.04952049	4.98677068	5.0566965
NONHSAG055350	5.96987324	6.00517559	6.02182531	5.97090036	5.97145313	6.06519716	5.85556184	5.71184983	5.71651318
NONHSAG055367	3.41687911	3.35058264	3.39241367	3.54056712	3.32586313	3.67238509	3.46597302	3.37299982	3.42094836
NONHSAG055371	6.00517664	6.32351961	5.42410644	5.95913455	5.47839088	5.70939628	5.92522052	6.01714621	5.57260952
NONHSAG055384	4.72175483	4.54250249	4.83863869	5.18516577	4.76606695	5.21911105	4.70620852	4.81259972	4.87024727
NONHSAG055388	3.56416413	3.74931366	3.43814765	3.54478118	3.58636885	3.59274103	3.51855442	3.71443705	3.25744174
NONHSAG055401	2.60338289	2.66263452	2.8417443	2.68728475	2.97332346	2.58359791	2.9953312	2.69782919	2.69918057
NONHSAG055405	3.78218306	3.90644182	3.70211474	3.68560156	3.74441014	3.9457001	4.01473159	3.81180065	3.69432785
NONHSAG055409	4.23181985	3.89734391	4.0232192	4.19495617	4.07248561	3.90618395	4.27692269	3.83696641	3.86142783
NONHSAG055436	5.28036006	5.38933165	5.27951657	5.43901094	5.4497609	5.34068609	5.32741144	5.18713763	5.44095259
NONHSAG055443	2.90223565	3.20917292	3.27171175	3.39093141	2.96873716	2.45033425	2.66281455	3.03151976	2.76891124
NONHSAG055445	11.53531872	11.44057596	11.39559861	11.16009721	11.53436856	11.25082103	11.25158129	11.29447468	11.40671016
NONHSAG055446	2.50868882	2.58068993	2.72244386	2.53481615	2.69809755	2.62970621	2.51916799	2.61004947	2.51670378
NONHSAG055449	5.94952038	6.11289839	6.02761222	5.92558792	6.11452514	6.1251658	6.17937052	6.22515273	6.3377393
NONHSAG055454	2.40480591	2.51814474	2.56038752	2.41121746	2.3683816	2.56424435	2.44941578	2.47099535	2.4233159
NONHSAG055455	3.03804499	2.84219164	3.06583374	3.07486102	2.82587298	2.94374802	2.70760641	3.26522652	2.7249921
NONHSAG055463	3.10939419	2.86909052	2.95624568	3.00536498	2.8977196	2.99392927	2.86802229	3.19965291	3.13141786
NONHSAG055544	5.31034582	5.03485086	5.33755349	5.12827884	5.15122088	5.11635643	5.11192307	4.99017228	5.24872575
NONHSAG055550	2.5939649	2.75169022	2.47282758	2.54842913	2.72125778	2.63553584	2.39218805	2.47911319	2.7249921
NONHSAG055620	5.55690939	5.70538287	5.54641271	5.5881445	5.48346746	5.5864999	5.45103625	5.65114189	5.18654533
NONHSAG055690	3.61768804	3.50599386	3.65510108	3.66201422	3.78396248	3.76399068	3.44222416	3.60521272	4.09506761
NONHSAG055699	3.78285066	3.4701161	3.5705946	3.78688172	3.62670469	3.70883874	3.81881015	3.59267619	3.54302173
NONHSAG055722	2.9377588	2.8695623	2.8830219	3.21562765	3.14439784	2.98839082	2.93684072	3.04941959	2.99245242
NONHSAG055783	4.69806568	4.5852211	4.75046786	4.50581659	4.61430263	4.47600669	4.62138471	4.70074276	4.95721438
NONHSAG055861	2.42732818	2.41334429	2.5531846	2.51921685	2.72752575	2.61456025	2.7383613	2.67350608	2.36798585
NONHSAG055878	3.85189868	3.79419829	3.86739063	3.65894191	3.85231253	3.80430304	3.77781349	3.84851686	4.04830679
NONHSAG055883	5.09719618	4.86731604	5.44331071	5.53578885	5.10023642	5.49424648	5.47414028	4.95212057	5.3121643
NONHSAG056024	3.63419225	3.52895642	3.70719188	3.68287194	3.84776935	3.71729782	3.73857131	3.63134228	4.00249836
NONHSAG056026	2.32051907	2.2899265	2.15099512	2.27746233	2.30517591	2.31307488	2.38043898	2.71664043	2.23296381
NONHSAG056029	4.32543544	4.40001275	4.37472243	4.48578407	4.36743925	4.29612732	4.25938364	4.31612302	4.55539885
NONHSAG056034	5.28402512	5.08880753	5.42241241	5.47216793	5.32818189	5.32342635	5.43089908	5.34158569	5.23953062
NONHSAG056037	7.28307504	7.3007151	7.20093573	7.36758377	7.37063379	7.6602132	7.46393186	7.45943678	7.69687714
	ethanol_ethanol_10	E2_ethanol_1	E2_ethanol_2	E2_ethanol_3	E2_ethanol_4	E2_ethanol_5	E2_ethanol_6	E2_ethanol_7	E2_ethanol_8
NONHSAG000057	5.54743111	5.32356229	5.19403571	5.17837662	5.23474418	5.35513162	5.13483733	5.40593328	5.29681404
NONHSAG000058	6.25311387	5.571517	5.56284967	5.34912088	5.33026236	5.76543816	5.17365725	5.45521291	5.52458781
NONHSAG000070	7.05044421	6.6840363	6.58237343	6.58639329	6.62515646	6.68271259	6.74675806	6.77116348	6.81917692
NONHSAG000071	5.22386999	5.37445282	5.2058215	5.03507803	5.39286665	5.41645944	5.18847634	5.3378812	5.34296602
NONHSAG000075	6.4241658	6.36418569	6.1001769	6.27162879	6.05977874	6.04034677	6.0669765	6.23809743	6.09415851
NONHSAG000111	3.71770129	3.81637122	3.82832877	3.83691033	3.75958332	3.91030923	3.7979442	3.94100135	3.73518255
NONHSAG000113	6.03994902	6.38385629	6.41004505	6.39098643	6.33087439	6.32231382	6.49729261	6.47598102	6.09089979
NONHSAG000117	6.53892531	6.33465843	6.35814742	6.1331504	6.32869762	5.98338546	6.24578112	6.24193504	6.52771014
NONHSAG000134	3.21199556	3.23477629	3.14821773	3.05647754	3.03440824	3.25377043	3.16049924	3.22235642	3.06246929
NONHSAG000139	5.97061395	5.95808131	5.53122779	5.49427785	5.6754998	5.99961195	5.57042767	5.83119396	5.66128193
NONHSAG000169	3.80779121	3.2693832	3.10990966	3.2722378	3.16207117	3.56229476	3.06698977	3.71489989	3.2279433
NONHSAG000174	4.64283464	4.93839394	4.68210617	4.67983581	4.82654205	4.96070106	4.7173104	4.73368938	4.56203994
NONHSAG000181	2.93354551	3.02317295	3.15950436	3.09924969	3.0091388	2.89044992	3.1649468	2.97484305	2.97759186
NONHSAG000185	4.49141532	4.0680244	3.95682301	4.09418369	4.17039697	3.84755192	4.4048671	4.37659783	3.98030174
NONHSAG000199	6.42749316	6.4296246	6.27517206	6.18934684	6.17749551	6.30957844	6.33988494	6.45996702	6.1173671
NONHSAG000202	3.14841469	3.27081407	2.86170452	2.92987343	2.91710026	3.11176199	3.11176199	3.39914783	2.68841281
NONHSAG000215	3.65059937	3.56765549	3.51074623	3.47885456	3.46632288	3.67102693	3.48594365	3.56732341	3.1657172
NONHSAG000222	3.49744128	3.64103585	3.85381453	3.51360784	3.85174634	3.82641237	3.82488084	3.80841555	3.71276679
NONHSAG000254	6.01799563	6.11898854	6.25402131	6.08599332	6.15339537	6.13862991	5.88593604	6.07849128	5.9862518
NONHSAG000264	3.73410884	3.89909412	3.52258911	3.89110602	3.70596417	4.22037057	3.65912493	4.127107	3.70697323
NONHSAG000267	5.25732358	5.30897439	5.13297788	5.235629	5.02265521	5.29205004	5.25067641	5.1839496	4.93829879
NONHSAG000310	6.83977279	6.33100132	6.226978	6.21522176	6.23766724	6.13717492	6.68481485	6.35785349	6.56739815
NONHSAG000312	5.06478369	5.108968	4.81596448	4.70480759	4.65389059	4.9305756	4.97536083	4.84219432	4.76134701
NONHSAG000322	3.12511641	3.36528647	2.99713877	3.15573345	3.1445445	3.15418016	3.39429498	3.22365125	2.99741263

NONHSAG000329	7.36636247	7.66993545	7.84915208	7.85308667	7.57866015	7.64742993	7.69218738	7.60443613	7.76757222
NONHSAG000345	9.09240291	9.19984807	9.2650458	9.14952185	9.22038102	9.09832799	9.28040162	9.16223479	9.22468905
NONHSAG000364	5.59074612	5.56706133	5.35001397	5.46818271	5.52549525	5.38627285	5.38112173	5.60432461	5.60603617
NONHSAG000381	4.41569172	4.47829794	4.16082224	4.27204885	4.12901605	4.38959486	4.2278942	4.45090912	4.02771872
NONHSAG000395	4.34748176	4.31257538	4.43601988	4.30083163	4.40284416	4.40037099	4.31423528	4.51218842	4.26046881
NONHSAG000423	2.61501003	2.81072215	2.43442377	2.80268421	2.71705354	2.80248815	2.68626915	2.77770583	2.47976997
NONHSAG000442	8.8467832	8.5495502	8.680131	8.71352435	8.77060765	8.51541173	8.84974178	8.6599023	8.72718002
NONHSAG000476	6.02541028	6.13310023	6.03615666	5.69255952	5.99296955	6.02404703	5.8134256	6.03887376	5.85774742
NONHSAG000490	2.82630989	2.92910805	2.76563798	2.72182406	2.61794139	2.98604264	2.77506159	2.78132631	2.75972726
NONHSAG000522	3.21094562	3.33061999	3.19348741	3.19697852	3.37503858	3.3343427	3.06198023	3.21015436	3.12968603
NONHSAG000529	4.08616714	4.36724226	4.43609629	4.41645906	4.14299228	4.57000045	4.14323473	4.31480406	3.96513321
NONHSAG000546	5.43523284	5.31016681	5.0365172	5.37587654	5.16812193	5.2653697	5.37344793	5.43399032	5.01543941
NONHSAG000628	4.57817442	4.30717362	3.91917784	4.54700658	3.4906703	4.08597307	4.34381197	4.09660048	3.95769028
NONHSAG000648	5.88370472	5.73421741	5.65091114	5.80614727	5.63411206	5.61791566	5.82144434	5.65536825	5.5442162
NONHSAG000655	5.12779467	4.71853401	4.74580557	4.49666508	4.32260897	4.54711179	4.80019071	4.52952086	4.30965666
NONHSAG000666	5.21019304	4.92396921	5.13340816	5.08372823	5.24133975	5.51862326	5.33813738	5.47814625	5.23220097
NONHSAG000716	3.49267936	4.12421015	3.83759562	3.94318888	4.04417036	4.12421015	4.183473	4.17853107	3.69210232
NONHSAG000738	3.83693982	3.97072937	3.80067446	3.98760503	3.97934364	4.03935023	4.24886258	4.05840505	4.25878148
NONHSAG000751	2.94158982	3.3427367	2.86002098	3.14418149	3.23663449	2.87710491	2.9462167	3.08896723	2.80959805
NONHSAG000769	4.49272505	4.82197795	4.28129059	4.59240545	4.4812413	4.89526706	4.58751882	4.81619242	4.39013946
NONHSAG000778	3.25578094	2.62561747	2.88677371	2.86618935	2.78407304	2.8635494	2.64408507	3.0421017	2.6438383
NONHSAG000812	4.48451785	4.44798062	4.06129576	3.63434151	3.5763151	4.12753097	3.93013539	4.25409924	4.14553538
NONHSAG000824	2.91415508	2.8091576	2.78780408	2.99009157	2.73618054	2.75290706	2.87208319	2.87332523	2.78683347
NONHSAG000847	3.67955159	3.89209257	3.73052803	3.73263584	3.78875743	3.96926483	3.88264695	4.0072921	3.89670278
NONHSAG000884	3.76653772	3.67242721	3.41247845	3.38678811	3.58829335	3.74241313	3.75875334	3.75875334	3.30373558
NONHSAG000939	4.3174521	4.20294662	4.03990842	3.94458062	3.77223797	3.74574653	3.91549748	4.42321987	4.08892906
NONHSAG000946	5.66850753	5.57645813	5.54021528	5.54686405	5.75317624	5.33140046	5.49041531	5.6883823	5.68368308
NONHSAG000965	3.39354592	3.30292301	2.89905153	3.12800931	3.10958221	3.28205163	3.16618045	3.33078337	3.42491565
NONHSAG000972	2.68194423	2.42617039	2.49903124	2.82997669	2.63288894	2.73067118	2.54593903	2.72136021	2.45997464
NONHSAG000976	5.08023435	4.31172331	4.51062346	4.15911864	4.48579955	4.49528654	4.82560491	4.60460665	4.64992756
NONHSAG000979	4.29947503	4.40969725	4.37963037	4.1262338	4.00814182	4.2230899	4.10245928	4.12719729	4.08765644
NONHSAG001012	8.88745103	8.62663833	8.97383963	8.77631076	8.69662164	8.52342182	9.02375615	8.79632541	8.69055326
NONHSAG001018	2.56074391	2.45044081	2.42920437	2.1510852	2.37157384	2.496259	2.31823565	2.4003329	2.33062596
NONHSAG001041	5.48416902	4.84010935	5.05964433	4.95801929	5.01634773	5.11677993	5.10654505	4.87640898	5.07442715
NONHSAG001043	5.58428551	5.21134697	5.10098557	5.27004627	5.25903957	5.47016773	5.27184749	5.23848258	5.18835182
NONHSAG001075	3.84911324	3.49174412	3.36683131	3.42016439	3.32765648	3.63002795	3.2165155	3.46593459	3.25663524
NONHSAG001146	7.71733729	7.35577522	7.58702118	7.35148016	7.49314329	7.3900815	7.5382022	7.44029633	7.47435686
NONHSAG001183	2.63154799	2.31385308	2.59781228	2.55238083	2.40857602	2.53126656	2.35779679	2.43760594	2.47113881
NONHSAG001185	2.95446052	3.28153891	3.11007241	3.08585896	2.99641432	2.88089074	3.16992342	3.11212678	2.9829106
NONHSAG001211	3.66093366	3.43396431	3.52859525	3.315663	3.37682565	3.38764459	3.20572221	3.37184187	3.24162452
NONHSAG001222	4.97965908	4.37649905	4.49495533	4.47045542	4.08557754	4.3009915	4.5245663	4.37872686	4.02281091
NONHSAG001244	3.43640906	3.43381914	3.34276326	3.41919741	3.10825731	3.50259747	3.33170592	3.59404731	3.02233598
NONHSAG001246	5.03714798	5.53399797	5.40736149	5.45156847	5.35144572	5.42846312	5.61935043	5.64944503	5.25964455
NONHSAG001247	5.109748	4.96066601	5.1608465	4.87036254	4.90232696	4.99941298	4.85608914	4.74122518	5.02891735
NONHSAG001264	4.03829271	3.95846208	3.70155384	3.74362873	3.56196948	3.75383733	3.73603832	3.85293411	3.81366854
NONHSAG001304	3.43393256	3.16490289	3.02570583	2.94476551	3.26841386	3.14959994	3.09313783	3.23357258	2.907344
NONHSAG001323	4.54449652	4.58992226	4.45438326	4.82413309	4.66645995	4.84163888	4.53244943	4.60569361	4.49152562
NONHSAG001341	2.92521255	3.19573557	2.62059127	2.73399661	3.14712684	3.20155189	3.02794895	3.01949588	2.58768559
NONHSAG001391	4.44402303	4.32769186	4.11199419	4.3833187	4.18761898	4.48153803	4.2787102	4.50850327	4.3590121
NONHSAG001489	2.86708593	2.69912163	2.63321896	2.88706261	2.5130166	2.70538266	2.92863166	2.80697332	2.73592122
NONHSAG001510	4.7910178	4.93781813	4.73099448	4.82359468	4.66940625	5.02796441	4.89125224	4.95722165	4.78014675
NONHSAG001530	6.70117361	5.59112589	6.10346219	5.77632574	5.78003211	5.60126815	5.81280302	5.64341247	5.89858524
NONHSAG001569	2.11977236	2.4597555	2.54268153	2.64541366	2.26941916	2.42484323	2.35779679	2.78272181	2.27203582
NONHSAG001574	3.46345842	3.66064951	3.5695091	3.62116228	3.39969215	4.00121246	3.55400185	3.8064806	3.70265949
NONHSAG001576	3.44816273	3.59272713	3.38389222	3.39404638	3.31931215	3.53241607	3.26429808	3.4295915	3.22602182
NONHSAG001587	3.26517257	2.89040016	2.82705291	3.07400299	2.61097819	3.0432299	2.75183306	2.80864912	2.98206338
NONHSAG001597	2.88815493	3.01129439	2.71456368	2.88747334	3.10135781	3.01209661	2.82956382	2.83251782	2.90554133
NONHSAG001601	4.00351742	4.48511846	4.07537869	4.15767179	4.18850744	4.41161979	4.20970924	4.42737989	3.94007318
NONHSAG001622	6.29589743	5.96817053	6.09199913	5.78093568	5.87939174	5.77280008	6.01331982	5.87631859	6.20355346
NONHSAG001627	5.34363581	5.12067691	4.86396071	4.93423389	5.3677664	5.13978141	4.84450061	5.12943946	4.83835639
NONHSAG001640	3.87934133	4.25792706	3.96824253	4.40210533	3.97958473	4.60384502	4.26574061	4.32427859	4.08413566
NONHSAG001660	4.30925926	5.08996628	4.93491021	4.96819812	4.8960077	5.16504006	4.93872873	5.04941871	4.98855397
NONHSAG001683	4.40124858	4.13890207	4.15178832	4.14917007	3.98800809	4.37876773	4.21001008	4.46568331	3.99885611
NONHSAG001696	6.2397902	5.74382629	5.92230539	6.44179122	6.25181955	5.68600125	6.05293873	5.94702652	6.17990643
NONHSAG001722	4.36231305	4.68482324	4.2338007	4.11247724	4.00536108	4.68519404	4.3065281	4.50097708	4.1740482
NONHSAG001723	3.9261141	3.37195679	3.43134078	3.26385435	3.32240769	3.09451012	3.29645169	3.18254411	3.34814892
NONHSAG001768	3.03801957	2.90800611	2.81882558	2.55432008	2.33358628	2.82545051	2.96526471	2.69837685	2.46805711
NONHSAG001788	2.72763225	2.47673195	2.46886153	2.43982587	2.50235941	2.46193914	2.61112052	2.42225128	2.68816773
NONHSAG001803	3.27187454	2.8181846	2.68122699	2.72997651	2.79644634	3.03254697	2.73488776	2.69066417	2.50067716
NONHSAG001824	2.5203132	2.54517047	2.6538203	2.713384	2.46533306	2.85201778	2.98793259	2.61487271	2.48418552
NONHSAG001832	2.46570676	2.67798287	2.55808808	2.69423857	2.6679168	2.76739939	2.59356811	2.63173146	2.66613952

NONHSAG001845	2.42285648	2.41484372	2.41024788	2.27233254	2.3317994	2.44688502	2.37999085	2.48066842	2.53039132
NONHSAG001849	10.50437992	10.49378581	10.55866794	10.55450857	10.34909105	10.59742453	10.33954409	10.54307225	10.57894822
NONHSAG001855	2.95636489	2.90569801	3.04717035	2.94293995	2.69564793	3.66432468	2.75453833	3.02021462	2.95486202
NONHSAG001898	3.00533719	3.0043076	3.11537713	2.99178533	2.91342195	3.04661522	2.91691671	3.07195856	2.96774593
NONHSAG001910	2.90826796	2.83564544	2.82226725	2.84831203	2.85102415	2.86405669	3.03672293	2.6180773	2.99150594
NONHSAG001920	2.54629045	2.55718956	2.69914029	2.84368152	2.76660537	2.52273106	2.60717113	2.553661	2.49381398
NONHSAG001926	3.35543246	2.75782811	2.71227761	2.58702419	2.33368383	2.89985794	2.73536087	2.54674703	2.57688031
NONHSAG001938	3.23297621	3.33009122	2.84278482	2.94786883	3.03695373	3.20034498	2.93763126	2.94896573	2.87141395
NONHSAG001941	3.38267178	3.50142244	3.34357291	3.41039365	3.49244046	3.5583369	3.31693955	3.54900991	3.24015241
NONHSAG001965	8.39490464	7.46456803	7.53180989	7.56494723	7.31275087	7.33711332	7.30558668	7.37320378	7.5603578
NONHSAG001966	8.19101311	8.19020893	8.67802113	8.35043054	8.33095662	8.010687	8.36736331	8.32319466	8.26757866
NONHSAG001970	3.38123434	3.56839799	3.69112902	3.59840193	3.61963934	3.35611518	3.90118204	3.271868	3.58692575
NONHSAG001984	4.10285958	4.40592251	4.35830983	4.53306149	4.3379239	4.34713898	4.31781509	4.34514617	4.59847919
NONHSAG001992	4.26221299	4.31677349	4.36618639	4.15372936	4.32070392	4.28999741	4.26653858	4.40447748	3.97843338
NONHSAG002015	3.26987854	3.6318414	3.37249291	3.52754498	3.53045808	3.45991772	3.76816071	3.18165335	3.48875248
NONHSAG002029	2.80905636	3.06459323	2.90130713	2.64121644	2.68510614	3.15265231	2.90543086	2.79423502	2.82676829
NONHSAG002048	2.26501931	2.22635803	2.28052094	2.29558021	2.37765379	2.38127877	2.21050974	2.28784357	2.5779954
NONHSAG002049	4.7815824	4.93068645	4.20880354	4.31858033	3.82425467	4.43480281	4.27524992	4.34480005	4.18174883
NONHSAG002063	4.48962034	4.09011682	4.02325622	3.94691041	4.05112994	3.89809628	4.31752529	4.0742263	3.67884915
NONHSAG002096	4.11038951	3.18818143	3.76621872	3.82277251	3.65607202	3.80704148	3.78032694	3.70336752	3.97308871
NONHSAG002128	3.98069591	4.17073608	3.99995276	4.0553939	3.90463522	3.91549848	4.03782818	3.97129515	3.7010559
NONHSAG002154	2.39446231	2.34898165	2.193841	2.36531837	2.28463507	2.2795821	2.33201613	2.38459087	2.40849881
NONHSAG002156	4.72967394	4.80498627	4.53087151	4.56193161	4.54379492	4.59089795	4.79939013	5.01389902	4.74818212
NONHSAG002177	2.86088419	2.68768052	2.63125675	2.4845762	2.49984837	2.83115811	2.48286239	2.83177042	2.85626711
NONHSAG002178	2.32868044	2.081915	2.24678494	2.26759285	2.13139838	2.2191803	2.14581744	2.1223322	2.20414764
NONHSAG002204	3.6422762	3.24131047	3.61462438	3.69622468	3.4093025	3.32285571	3.28376414	3.43901295	3.48062443
NONHSAG002211	2.53131406	2.54210587	2.61683806	2.53234093	2.55075027	2.69268989	2.57923079	2.51975551	2.58996313
NONHSAG002218	2.17793563	2.36449586	2.32782474	2.40395558	2.35332516	2.34524597	2.29631794	2.36078107	2.25742963
NONHSAG002223	3.86858686	4.23712973	4.0576354	3.82971513	4.0440963	4.48166598	3.90260219	4.33437092	3.99676896
NONHSAG002277	4.58557395	5.02072943	4.13090488	4.43231103	4.31490181	4.77031444	4.59193072	4.53634328	4.52008192
NONHSAG002281	4.26300945	4.0548694	4.20012224	3.85334941	4.03442171	4.08651958	4.39208855	4.03924859	4.19894218
NONHSAG002337	4.1217627	3.80086551	3.70341899	3.88873199	3.86935921	3.92030669	3.90678388	4.05949525	4.01713427
NONHSAG002360	5.74402577	5.7820357	5.68713854	5.691352	5.35986828	5.57664613	5.57813225	5.92525241	5.4547751
NONHSAG002376	2.09470533	2.3733735	2.32026241	2.49948222	2.4906946	2.32859204	2.44072357	2.49069267	2.1796506
NONHSAG002377	5.44917163	5.17823087	5.27836372	5.31752655	5.37133974	5.26515417	5.32027835	5.23880076	5.16054821
NONHSAG002389	3.05977939	3.43086359	3.04519863	3.22060687	3.13214889	3.70495614	3.2368955	3.59110649	3.08524255
NONHSAG002392	2.91944428	2.75394493	3.23320454	2.72997651	2.60122366	3.01713957	2.85513032	2.64376242	3.06876961
NONHSAG002406	3.64378974	3.46707327	3.12914073	3.33581904	3.26433042	3.38541115	3.26953998	3.34031476	3.16544445
NONHSAG002410	4.64280905	4.59116185	4.84471025	4.87097917	5.1011648	4.60379756	4.75971005	4.82075078	5.15124071
NONHSAG002419	4.57436836	4.68967118	4.1824149	4.53153688	4.69799617	4.60069395	4.49441514	4.6421907	4.26479389
NONHSAG002421	3.09649857	3.0764911	3.13149364	3.12336719	3.03723582	3.30249636	3.11176199	3.28604795	3.12848279
NONHSAG002440	6.17052966	5.51876199	5.50878083	5.79039348	5.62443582	5.60493493	5.87004569	5.4524822	5.39959315
NONHSAG002455	3.16420432	3.14641611	2.94923456	3.06496947	3.08439509	3.04327035	2.95079687	3.12618506	3.09109569
NONHSAG002458	3.07986896	3.23619937	3.15508352	3.07774869	2.98164107	3.14463612	3.11170205	3.06756988	2.84813812
NONHSAG002465	6.85025092	5.7362451	5.97888913	5.86461936	6.10688655	6.26194482	5.89356043	5.84166437	5.76521588
NONHSAG002516	3.44837891	3.07954485	2.9430739	3.05154301	3.04016173	3.24138175	2.841188	2.97261453	2.8239787
NONHSAG002535	3.59366215	3.36682387	3.48005701	3.37082771	3.60553711	3.73734286	3.73745199	3.42266978	3.18910987
NONHSAG002538	5.13062146	5.32592415	5.04617653	4.99007477	5.13141476	5.21864861	5.12377293	5.3242929	5.05837186
NONHSAG002549	4.60584457	4.59178663	4.17568266	4.30383681	4.3074731	4.40981816	4.68581733	4.44082513	4.08814999
NONHSAG002555	2.70510448	2.6910501	2.94229979	2.65228865	2.78728481	2.77074272	3.04693935	2.66021557	2.81559094
NONHSAG002615	7.46397155	7.45342339	7.32510344	7.60805664	7.7197859	7.54226362	7.44499696	7.21376996	7.35547517
NONHSAG002728	4.51887049	4.28077949	4.66531735	4.40196441	4.21258942	3.94882905	4.27149527	4.19788041	4.10488433
NONHSAG002758	4.26453595	5.03615646	4.90076181	4.86661213	4.85604711	4.75575751	5.19801986	5.03464891	4.72427282
NONHSAG002759	3.12725519	2.98501549	2.93594349	2.81732983	2.71878046	3.11217341	2.78905449	3.04834075	2.83257402
NONHSAG002764	2.92352913	2.90875375	2.79640738	2.68094967	3.08756971	2.79882521	3.05760281	3.23797196	3.09109058
NONHSAG002857	3.65360013	3.79915702	3.08382804	3.34268321	3.27948928	3.82502871	3.54168789	3.57751573	3.48875248
NONHSAG002865	4.15911864	3.87724112	3.53530091	3.60011625	3.59107928	3.8841357	4.18893024	4.10643364	3.77331025
NONHSAG002891	7.49958547	7.25874394	7.27145374	7.21216587	7.23022731	7.13934942	7.54941231	7.00484634	7.36337622
NONHSAG002908	6.14846906	6.6999844	6.89361117	6.56113974	6.78616045	6.73136534	6.57493376	6.72815704	6.94463939
NONHSAG002936	7.78615818	7.49483157	7.45361923	7.46407341	7.60913544	7.38031204	7.75743519	7.70816002	7.6059964
NONHSAG002956	4.10978389	3.93895367	3.92786678	4.04633311	3.86186513	4.14396736	4.21423008	4.09577873	3.78317584
NONHSAG003018	4.73790196	4.73225347	4.54811056	4.76386711	4.70255543	5.07563461	4.88692904	4.77670342	4.56551295
NONHSAG003022	4.50130937	4.59692519	4.52924918	4.60069395	4.36791106	4.60069395	4.77672549	4.59018359	4.4135412
NONHSAG003057	7.15653529	7.22403361	7.06680216	6.89699527	7.00546395	7.28690439	6.88916807	7.22600134	7.04535234
NONHSAG003089	4.25358993	4.65021671	4.1829822	4.2305989	4.15773315	4.30009628	4.17255803	4.35710152	3.84585725
NONHSAG003123	2.82557997	2.55532717	2.60961164	2.52811074	2.32534566	2.74533697	2.33406927	2.47133969	2.53237025
NONHSAG003195	3.79426016	3.95132397	3.67565076	3.76284508	3.64435384	3.72797524	3.76153104	4.13912511	3.77331025
NONHSAG003198	2.88848259	3.24702072	3.25148232	2.90476123	3.04807575	3.16764175	3.08811901	2.93550819	2.85812522
NONHSAG003202	7.08450877	7.13555813	7.54435938	7.29699366	7.24677635	7.15771629	7.18669656	7.04216127	7.3243546
NONHSAG003261	4.36786529	4.24429505	3.58785741	4.04652874	3.5918763	3.91096661	3.57383114	3.28538574	3.84563867
NONHSAG003289	3.12429318	2.84838233	2.71641958	2.6094193	2.76999149	2.80252266	3.05387397	2.79433702	2.92721436

NONHSAG003322	6.36525286	6.15272381	6.33593587	6.41987613	6.57743662	6.51129752	7.10656973	6.51961587	6.39845468
NONHSAG003325	6.24740951	5.38260961	5.88177691	5.62859807	5.75807494	5.38653968	6.56447763	6.00586297	5.23384835
NONHSAG003332	3.63585149	4.05404936	3.818346	3.93773922	3.68258791	4.01192963	3.56293286	4.04144976	3.849433
NONHSAG003339	2.56043881	2.7487103	2.41607252	2.60522652	2.2778364	2.49472728	2.32673363	2.5807282	2.29465665
NONHSAG003345	4.2754179	4.1906504	4.02503791	4.07041531	3.90402322	4.03935023	3.69468424	3.98775458	3.76224111
NONHSAG003405	2.97881859	2.91818999	3.18470549	3.38924827	3.24627944	3.22298041	3.22747492	3.0559351	3.24656544
NONHSAG003407	6.48269756	7.26302189	7.35309044	7.31798187	7.08848953	7.05725557	7.06961741	7.32338137	6.99030251
NONHSAG003412	2.5545154	2.5014215	2.32728942	2.43028098	2.36316699	2.60269235	2.43497633	2.60546833	2.69887619
NONHSAG003464	3.98973802	3.66664757	3.87975831	3.69317826	3.64132956	3.81953041	3.66490292	3.65554988	3.37141602
NONHSAG003486	5.64470643	5.79491848	5.37673544	5.66988911	5.25332233	5.62055738	5.39065547	5.61397438	5.42216882
NONHSAG003512	3.41992998	3.30601644	3.88933896	4.30523695	3.64981907	3.59821977	3.40020739	4.00869156	3.57276355
NONHSAG003533	3.80511501	3.67204851	3.65361928	3.61445145	3.53200331	3.81939837	3.60983645	3.71088688	3.66550187
NONHSAG003536	4.10380583	3.88221654	3.73128659	3.84813237	3.73248623	4.03036172	4.07657985	3.93603582	3.88696662
NONHSAG003539	3.26517257	3.21749052	3.11867751	3.01552078	2.68473932	3.22543294	3.08758809	3.15816069	2.99741263
NONHSAG003554	2.5415294	2.47511056	2.3361242	2.33595455	2.3493367	2.52684597	2.43955372	2.28941005	2.38805775
NONHSAG003555	5.27132878	5.49091136	5.93910671	6.05206456	5.80391328	5.671301	5.6429368	5.71880384	5.62153656
NONHSAG003562	3.26047433	2.95739133	3.4531646	3.16504638	3.20940383	3.13081172	2.77039698	2.96756774	3.39228471
NONHSAG003567	2.25511184	2.24419426	2.40319636	2.34596567	2.14589689	2.26221913	2.34966621	2.27623252	2.41267355
NONHSAG003604	3.60217696	4.20565882	4.02277309	4.09025997	4.17546476	4.4350257	4.20058792	4.18962204	3.89288502
NONHSAG003613	4.42580944	4.74872595	4.43760395	4.52276206	4.57855578	4.88396371	4.59512148	4.39949081	4.52409474
NONHSAG003615	2.62735632	2.89602871	2.87361542	2.91789171	2.61361452	3.08457266	2.7675447	2.94374802	2.90243984
NONHSAG003627	3.84607048	4.22026765	4.02589259	3.93131392	3.82217126	4.04156088	3.87426208	4.08651148	3.91421116
NONHSAG003635	4.74045031	4.60169993	4.67795845	4.37961163	4.44839168	4.96817317	4.60704333	4.72455885	4.27155241
NONHSAG003645	4.75290393	4.71123153	4.49702478	4.67649277	4.72832348	4.73054549	4.81066119	4.71060431	4.50508621
NONHSAG003668	3.04971234	2.56811512	3.06262106	2.35409628	2.57971022	2.57807587	2.67323847	2.56720789	2.53569207
NONHSAG003758	2.3241257	2.37695846	2.11457731	2.30724212	2.12399107	2.38587408	2.18304626	2.22897717	2.31150924
NONHSAG003765	2.46957313	2.43407289	2.19074045	2.12003027	2.29705066	2.31262621	2.3005934	2.29213609	2.71335822
NONHSAG003799	3.25949641	3.13282589	3.355692	3.05811003	3.13530107	3.32371385	3.13565887	3.13106835	3.35810937
NONHSAG003829	3.56100033	3.62908043	3.76795975	3.55436473	3.87585572	3.59066331	3.58999931	3.54104873	3.56772624
NONHSAG003840	3.14019732	3.09093601	2.9112659	2.80624972	2.61987386	2.95317128	2.75453833	3.00780362	2.80493615
NONHSAG003843	2.33935345	2.22591189	2.3738881	2.44968379	2.18843104	2.3171845	2.484759	2.24192894	2.13049332
NONHSAG003854	2.93606856	3.34203958	3.06983833	2.96091432	2.96091432	3.14439012	3.04642346	3.22874257	2.89629239
NONHSAG003880	5.06865425	5.32716043	5.09107486	4.97564941	4.76530342	5.24681772	5.18409635	5.11567986	5.00247837
NONHSAG003894	3.60187439	3.77724622	3.38577207	3.47453823	3.31063243	3.49723566	3.29802053	3.55436417	3.42913646
NONHSAG003900	2.90694679	2.78373237	2.88904199	2.8244635	2.84011956	2.85733875	2.88746625	3.08329741	2.63406061
NONHSAG003927	6.90313649	6.16585186	6.17745802	5.99428399	6.2656963	5.879006	6.12807263	6.34373395	6.37673222
NONHSAG004034	4.950977	4.83288424	4.86149903	4.29673636	4.60916944	5.1660142	5.03749244	5.14077425	4.30513154
NONHSAG004049	2.50454664	2.73756237	2.42933182	2.56875883	2.33368383	2.7100533	2.44682791	2.13774864	2.35949732
NONHSAG004092	3.99627245	4.73853181	4.29143747	4.13130727	4.02206805	4.23095045	4.34243942	4.57293602	4.10252087
C1orf132	6.81062292	5.47384924	6.04952515	5.83679987	5.69905561	5.54160191	5.92338521	5.81331803	6.17082461
NONHSAG004140	4.0410173	3.9401576	3.53251245	4.08863642	3.83834069	3.54303647	3.87200968	3.89679151	3.24773001
NONHSAG004156	3.78856388	3.21865751	3.4751949	3.4181608	3.51379684	3.82187741	3.31591011	3.4181608	3.28594638
NONHSAG004159	4.8032238	5.01717972	5.70069048	5.72296013	5.35221671	5.27940076	5.18837691	5.16258993	5.28293616
NONHSAG004205	2.8125832	3.45698445	3.01115449	3.02166622	2.9074487	3.25826468	3.16093797	3.5519035	2.93767055
NONHSAG004221	3.37746652	3.56779258	3.48762967	3.53530091	3.049042	3.45464364	3.49032524	3.4398004	3.6201719
NONHSAG004235	4.61475887	4.41730246	4.44953905	4.36348749	4.23003682	4.17210535	4.16892882	4.00359432	4.11810077
NONHSAG004261	3.22243653	2.91376736	2.91261337	2.88855215	2.92372491	2.99691659	3.15709073	3.05343866	2.92254362
NONHSAG004265	2.62344083	2.72041177	2.56813885	2.74284444	2.51762933	2.88331011	2.75453833	2.70563126	2.42225421
NONHSAG004289	6.64970007	6.02486601	6.44371899	6.26991993	6.22525637	5.8512154	6.22350558	5.88489335	6.32179676
NONHSAG004292	5.7595905	4.6635061	4.62602888	4.53232145	4.55494636	4.68606772	5.15184052	4.54414855	4.71750294
NONHSAG004319	2.81202756	3.03813967	3.01128334	3.01551765	3.23986919	3.0736088	3.03502957	3.03999439	2.94375206
NONHSAG004322	4.20527204	4.85359598	4.48956998	4.54494378	4.40628721	4.5279586	4.471626	4.72506987	4.24233464
NONHSAG004342	5.10975436	5.45832853	5.32474581	5.28798717	5.40007085	5.39223643	5.01127355	5.69331464	5.16384011
NONHSAG004374	4.51216612	4.50995727	4.09240617	3.94205399	4.3124838	4.34148027	4.27119532	4.30971716	3.98302935
NONHSAG004468	4.60684244	4.54800692	4.44630976	4.52889006	4.48504509	4.50954879	4.5157956	4.65094737	4.51331067
NONHSAG004469	3.36245436	3.47503351	3.18608325	3.32788656	3.17543389	3.64394312	3.17925971	3.47133582	3.02486834
NONHSAG004473	4.36879145	4.46821802	4.37359358	4.34381197	4.26084285	4.30009628	4.33419729	4.6421907	4.17071187
NONHSAG004494	2.52918198	2.40233483	2.50940042	2.55221923	2.35475763	2.51149153	2.43129142	2.29415316	2.61348674
NONHSAG004522	2.94872746	2.92222377	2.44118164	2.46409687	2.48298193	2.82667099	2.75453833	2.51112114	2.54137111
NONHSAG004544	6.07887221	6.17325625	5.63217019	5.73599334	5.81711309	5.97561894	5.91451895	5.98040848	5.83773251
NONHSAG004554	2.5834752	2.44958344	2.55929414	2.29465015	2.39764583	2.5592649	2.75808392	2.70375601	2.54983421
NONHSAG004567	5.06837276	5.0307655	5.27643704	5.28153621	5.17687842	4.27338794	5.40533152	5.03784471	5.1523204
NONHSAG004569	4.4764914	4.30523753	4.05704483	4.32340088	4.5106245	3.97112845	4.29182863	3.99768224	4.05410865
NONHSAG004584	3.91473859	3.98539943	3.83702978	3.95987968	3.83265862	3.945194	3.93814969	3.96629507	3.75470169
NONHSAG004588	2.9399737	2.84953753	3.01011045	2.93548636	2.72146245	3.22351491	2.94825609	2.87720857	2.83156682
NONHSAG004592	2.41061241	2.28319857	2.30152558	2.26979063	2.39958987	2.24164865	2.53371318	2.34538845	2.17099185
NONHSAG004614	6.24851383	6.37063781	6.39035635	6.51002679	6.4854516	6.32993628	6.27366972	5.88749159	6.2103259
NONHSAG004619	4.70885785	4.72340616	4.86583706	4.81039715	4.74520192	4.81671658	4.80857414	4.67681956	4.8129415
NONHSAG004632	2.74249217	2.90569801	2.86315175	2.75910076	2.79241188	2.69579673	2.78371129	2.83204734	2.6627464
NONHSAG004636	3.90335821	3.97659259	3.87879283	3.80769843	4.16659788	4.05345538	3.84457615	4.08926451	3.92746282
NONHSAG004663	3.18149075	3.32259661	2.92519934	3.2592679	3.38183221	3.2944272	3.14252473	3.04782881	3.35354536

NONHSAG004683	3.95601956	4.1399761	4.00008486	3.61654494	3.44573324	4.11675212	3.7446371	3.90450683	3.72112764
NONHSAG004693	3.13861995	3.30211809	2.97399255	2.96059007	3.26286343	3.14080338	3.01673028	3.11221541	3.15511927
NONHSAG004694	5.02977759	4.88385377	4.63274884	4.48768152	4.71219236	4.8958519	5.15377741	4.83466671	4.65129939
NONHSAG004774	2.89218414	3.11206069	3.00401874	2.67485931	2.61627978	3.05922793	3.05709715	2.81151887	2.92900084
NONHSAG004778	2.9143079	3.06933175	2.75714711	2.59340836	2.68741242	2.63241684	2.525204	2.67133649	2.79076752
NONHSAG004794	3.78691443	3.90530701	4.03564088	3.99480198	3.60839214	3.94262372	4.00901892	3.99375347	3.6472001
NONHSAG004884	4.94609367	4.50482279	4.48243234	4.61067556	4.47145136	4.61810568	4.12888827	4.51640715	4.29818168
NONHSAG004887	5.07546563	5.01391102	4.3445053	4.96530831	4.75751877	5.32802634	4.58555287	5.33155657	4.63001334
NONHSAG004895	4.35032476	4.36119633	3.75496426	3.74455155	3.79633075	4.49076301	3.79928077	4.02673557	3.73621033
NONHSAG004899	3.27209861	3.71815147	3.41423366	3.92489251	3.37989411	3.76041396	3.70148355	3.67437946	3.28664251
NONHSAG004902	5.41688544	4.97798304	4.81628264	4.70173495	4.58164044	4.85273467	4.77839427	4.77540234	4.50455729
NONHSAG004908	5.14884491	5.2193518	5.27497804	5.09252171	4.97991519	5.13061759	5.07669166	4.99457413	5.1670482
NONHSAG004919	6.91441786	7.38605087	7.67929294	7.71859349	7.32882913	7.27363534	7.46060067	7.37970716	7.30256749
NONHSAG004920	2.94339368	3.08127007	3.18079758	3.11235558	3.2498953	3.24775938	2.91759773	3.39545804	3.11445802
NONHSAG004943	2.85940826	3.19222614	2.96280961	3.38181586	2.86889464	2.69907235	2.83187475	2.94372263	3.0079946
NONHSAG004961	5.21874472	5.0939939	4.99446171	5.24685999	5.13232467	5.17747649	5.21013206	5.21835927	5.125137
NONHSAG004977	6.46421517	6.13250122	6.46710778	6.40768069	6.46221763	6.24475657	6.36966331	6.30348474	6.10387709
NONHSAG005016	6.04295491	5.38179406	5.72512642	5.77808674	5.54027392	5.54107962	5.3863323	5.5826769	5.70606645
NONHSAG005033	2.43121435	2.4771232	2.2503099	2.44795339	2.13427834	2.64320338	2.42530204	2.46855275	2.66488157
NONHSAG005060	2.98661684	2.42912331	2.7264073	2.34335779	2.43165422	2.56107266	2.88091536	2.61032612	2.49325707
NONHSAG005063	3.12511641	3.23489696	2.93308223	3.14729186	2.96335361	3.08291961	3.12014793	3.26923466	2.93551536
NONHSAG005080	2.55755922	2.59126031	2.91180431	2.50222955	2.47738398	2.806474	2.27075254	2.93914091	2.72302048
NONHSAG005082	4.05482195	4.40364697	4.18330989	4.1314006	4.28389193	4.50447447	4.12834918	4.36165922	4.18524779
NONHSAG005119	2.90629013	2.78353716	2.67874647	2.57493594	2.57996588	2.60452023	2.82433348	2.5964601	2.64440964
NONHSAG005138	4.66660389	4.65810626	4.60592846	4.73997654	4.4828565	4.71304544	4.65166871	4.68189072	4.50312295
NONHSAG005145	3.19848367	3.35638743	3.05724545	3.14565069	3.15985366	3.18482078	3.12550233	3.41065172	2.96292533
NONHSAG005150	4.81080013	4.95052752	4.57116452	4.65889812	4.50710569	4.89386763	4.79561084	4.85080652	4.70581506
NONHSAG005165	4.80046402	4.956997	4.6917183	4.97274704	5.00037186	4.39599535	4.8012172	5.03427178	4.99501164
NONHSAG005205	3.02296053	2.90569801	2.71893345	2.85076318	2.91789171	3.01901275	3.00047602	2.94374802	2.80457912
NONHSAG005207	4.46410584	4.63655605	4.57926015	4.73252313	4.50856401	4.62146692	4.39544315	4.85085519	4.41330322
NONHSAG005225	3.53753041	3.23585693	3.23320454	3.16321471	3.41636622	3.36595284	3.30589868	3.26566028	3.31761675
NONHSAG005226	3.26667709	3.26667709	3.22288022	3.12666152	3.22008328	3.44699385	3.32111354	3.19978787	3.18972731
NONHSAG005232	6.47411989	6.33851094	6.40504357	6.54270737	6.4263923	6.37047807	6.27871541	6.35253751	6.57381957
NONHSAG005234	4.141418	4.11643178	3.81482939	3.72607664	3.667347	4.19067025	4.00816312	4.03075769	3.86718041
NONHSAG005239	5.27456363	5.39456459	5.65754072	5.74320094	5.53623526	5.33603884	5.7671749	5.44161416	5.49639262
NONHSAG005282	3.2416995	3.66839778	3.54739312	3.44229675	3.41636622	3.83881237	3.39186851	3.41774201	3.65253062
NONHSAG005284	4.61146116	3.37749423	3.45529251	3.26056043	3.37588862	3.47836995	3.66525625	3.53855789	3.61423907
NONHSAG005342	5.20590428	4.36469076	4.63593557	4.43167295	4.68831076	4.74288935	4.87757334	4.5957778	4.71650533
NONHSAG005356	3.0680117	2.80482647	2.82081016	2.63112417	2.76406563	2.75199371	2.90585673	2.50062622	2.67762887
NONHSAG005375	4.36766215	3.95889093	4.30961746	4.13266639	4.1373901	3.95023308	4.68064543	4.5105795	4.82943864
NONHSAG005386	3.39829772	3.62306041	3.32489806	3.19732057	3.3322543	3.59866722	3.48919006	3.62990336	3.18554652
NONHSAG005407	5.81181026	6.36897765	6.16952326	6.29034285	6.03831371	6.12298807	6.14298492	6.44777751	6.16001325
NONHSAG005420	2.7964573	2.87505648	2.88952763	2.79591934	2.65957184	2.91027069	3.12869654	2.77597213	3.0062853
NONHSAG005464	5.25467412	5.41914919	5.24774322	5.07597019	5.29526455	5.51740335	5.22087053	5.27810811	5.04722875
NONHSAG005471	2.8103655	2.70536511	2.62179174	2.77409755	2.62818776	2.90410607	2.67992423	2.82317814	2.71505575
NONHSAG005501	4.45065603	4.11154708	4.24395637	4.09739158	3.80137879	3.82747999	4.2042169	4.10348351	3.49332462
NONHSAG005503	4.6978796	4.26638724	3.9989	4.44367622	4.30329931	4.26275618	4.32294975	3.95808069	3.91032714
NONHSAG005505	2.50969395	2.5014215	2.23244221	2.66355696	2.53543435	2.5062837	2.43363572	2.42144069	2.3650665
NONHSAG005523	5.66392999	6.03527727	6.00457328	5.96948473	5.82930264	5.87037751	5.91613533	5.56992448	6.19509524
NONHSAG005543	3.03064699	3.17086463	2.93764701	2.88018446	2.78853231	2.75422407	2.9667314	3.0214138	2.86356617
NONHSAG005545	2.8305545	2.87046494	2.76925951	2.74411301	2.83114104	2.70374116	2.86065976	2.68945523	2.61957378
NONHSAG005589	4.48633316	4.28653376	4.41119399	4.27605845	4.45012082	4.56648039	4.61491809	4.43149293	4.4630803
NONHSAG005603	3.39373528	3.29405473	3.21236688	2.98808898	2.87853787	3.2466649	3.38038086	2.96343145	3.04854987
NONHSAG005687	3.8154891	4.36635491	4.47972675	4.80947804	4.25639029	4.31814531	4.31814381	4.47111557	4.64378184
NONHSAG005692	3.78937215	3.45521319	3.37783203	2.92780921	3.38470594	3.39771298	3.43780177	3.19203471	3.64305961
NONHSAG005715	6.32739303	6.08464222	6.47843922	6.32913285	6.41833242	6.04827017	6.20341663	6.38436167	6.3616009
NONHSAG005734	4.22452621	4.4630673	4.06267055	4.12843143	4.03714976	4.32402935	4.18656848	4.3884877	3.96596181
NONHSAG005754	4.24108473	4.01933036	4.01653214	4.03925435	4.0583037	4.01630605	3.93762125	3.96283899	3.92027353
NONHSAG005762	3.61930459	3.88818497	3.2741843	3.43994912	3.61694621	3.72951883	3.61593999	3.58521944	3.87229555
NONHSAG005767	3.49972206	3.33575947	3.20650873	3.28902805	3.28153891	3.3714331	3.15072155	3.52656659	3.06070581
NONHSAG005812	2.78552338	2.48937051	2.47411486	2.43611113	2.54361941	2.74138924	2.5825616	2.50512245	2.97837364
NONHSAG005834	4.96137023	4.83037631	4.92988919	5.12831378	4.68563648	4.76824341	4.97536083	4.73635119	4.97960569
NONHSAG005850	3.23064766	3.21430622	2.99766644	3.05455078	3.05393019	3.4730887	3.3237103	3.22500297	2.98314384
NONHSAG005870	4.76717329	4.95254484	4.97536083	4.73270111	4.87988283	4.71872968	4.73195244	4.75429215	4.93910799
NONHSAG005912	2.38027764	2.50494049	2.43582097	2.47157325	2.58529403	2.5391703	2.48438678	2.44696974	2.71335822
NONHSAG005923	6.30456767	6.26597292	6.08203207	6.09496622	6.12707469	6.07706635	6.70795374	6.81136805	6.90499968
NONHSAG005953	3.67241784	3.89375125	3.87676095	3.49737277	3.55465259	3.88605624	3.67866804	3.53232585	3.40251901
NONHSAG005957	3.13692619	3.00086965	2.93402264	2.95015915	2.86879936	3.076688	2.8531166	3.05903446	2.78136502
NONHSAG005965	3.25497209	2.94365596	3.10488915	3.27561773	3.00783663	3.10703387	2.85331745	3.08925813	2.90595101
NONHSAG005970	3.36518924	3.29030144	3.44522656	3.32787516	3.46319298	3.39202657	3.23820616	3.3023872	3.19778396
NONHSAG006053	2.44045718	2.35122517	2.56868985	2.4676058	2.54581235	2.57373174	2.5081571	2.51034351	2.61283987

NONHSAG006081	6.58848176	5.3689457	5.74890656	5.64977516	5.57835235	5.84815196	6.04900549	5.23907519	6.06459454
NONHSAG006083	3.02887073	3.10090228	3.34047659	3.19085124	3.28153891	2.98211234	3.37674938	3.17861051	2.88607247
NONHSAG006087	4.37430743	3.9880715	4.48569812	4.21047042	3.98286443	4.03935023	4.10243936	3.87161936	3.9132678
NONHSAG006140	2.88997405	3.48189419	3.03730369	3.09195246	3.18457352	2.94186805	3.28762481	3.07789671	2.76916129
NONHSAG006147	4.8231671	4.7417503	4.54720234	4.58786281	4.43809215	4.99298976	4.73231133	4.94328322	4.67157952
NONHSAG006149	4.98254738	4.54414346	4.73451475	4.74841342	4.80907625	4.79210994	4.71712296	4.69408392	4.54891531
NONHSAG006187	2.2382708	2.15116525	2.01896009	2.04937506	2.02938989	2.352281	2.283298	2.00001195	2.09909796
NONHSAG006196	5.65970873	5.40818211	5.49294922	5.50329896	5.77400838	5.61472778	5.45508825	5.22644049	5.34820694
NONHSAG006210	5.72216719	5.53470732	5.51520055	5.43917991	5.71157915	5.41775861	5.61833217	5.80581947	5.5364508
NONHSAG006244	3.42860501	2.92591472	3.15302074	3.19060094	2.99832187	3.23492677	3.42886551	3.18005116	2.56094202
NONHSAG006270	3.01229692	2.75530505	2.83121904	2.75351355	2.79462002	3.0093456	2.83576946	2.85357419	2.89919207
NONHSAG006274	2.90962883	3.07559605	2.97322882	3.14492205	3.02694844	3.31705435	2.79417277	3.08544735	2.85330589
NONHSAG006311	3.56487863	3.52283706	3.24036465	3.69110327	3.70668551	3.72613794	3.2951311	3.52664165	3.35427911
NONHSAG006312	2.63771625	3.06861767	2.95990934	2.93216817	2.7824952	2.77971799	2.99959003	3.17889074	2.93911063
NONHSAG006326	3.57677943	3.83163888	3.68347981	3.72476301	3.78431041	3.77543622	3.7213567	3.69836279	3.66960533
NONHSAG006406	3.18702588	3.7857181	3.41340423	3.30575277	3.57739814	3.91923549	3.47009406	3.73427924	3.71345255
NONHSAG006407	4.05034164	4.28667986	4.01498355	4.09352937	3.80983533	4.40170592	3.97610164	4.04083877	3.61241766
NONHSAG006417	4.35976978	4.21340506	4.36958889	4.54572693	4.43101511	4.25384386	4.59120423	4.45180825	4.19097542
NONHSAG006458	5.30997757	5.03040917	4.90485094	4.9859497	5.02215914	4.99290297	5.18350276	5.09265409	4.99527335
NONHSAG006506	2.18871097	2.48288071	2.29334352	2.15742597	2.14144558	2.43904531	2.37205407	2.34375548	2.143206
NONHSAG006557	4.0714475	4.07477447	3.96745221	3.90860461	3.98765897	3.89104382	4.17255803	4.19244049	3.74631253
NONHSAG006570	6.66407314	9.10081096	9.1855688	8.95576264	8.66388936	8.81057853	8.9033224	9.08369577	8.65838833
NONHSAG006585	6.1582675	6.81505106	6.30771877	6.80298843	6.47143307	6.94720743	6.49191304	6.6495656	6.62326528
NONHSAG006591	5.24788955	5.04527866	5.27810811	5.24368749	5.27810811	5.37856984	5.71928945	5.2601256	5.10061394
NONHSAG006615	5.86868872	5.10146526	5.20530065	5.0581295	5.29089535	5.00586896	5.18900128	5.0291185	5.15359398
NONHSAG006619	7.66703433	7.22088552	7.36949791	7.16617808	7.41627768	7.41739682	7.45341208	7.11237697	7.28864752
NONHSAG006623	5.1786329	5.44738263	5.09544125	4.91351764	5.01078092	5.74393932	4.80972985	5.31902879	5.00579326
NONHSAG006631	9.16733314	10.3263164	10.47613322	10.28305847	9.72675684	10.26038467	9.7283294	10.25508638	9.7392867
NONHSAG006633	3.84229858	4.13801535	3.89447697	3.83110766	3.83107414	4.12358813	3.76394722	3.94463655	3.79261799
NONHSAG006679	5.93549764	5.87539898	5.98630533	6.0408785	5.99977351	5.94004494	6.08845993	6.14703333	6.05891184
NONHSAG006690	3.91709426	3.50872446	3.93641562	4.0615542	3.91019016	3.64394312	3.91812652	3.80763291	3.44638957
NONHSAG006700	5.8968555	6.24917863	6.80655085	6.07819048	6.6976698	6.5720315	6.91490956	6.44901956	6.57968144
NONHSAG006702	6.3233622	5.79903503	5.73506071	5.70363609	5.9467007	5.72656203	5.77905962	5.5996211	5.67281258
NONHSAG006706	3.89354833	4.15209712	4.58998536	3.96056514	4.15829958	4.47166525	4.00690155	4.33643769	4.16415169
NONHSAG006711	3.2520336	3.28595397	2.9991431	2.97831033	3.09392445	3.2068185	3.17673222	3.02549786	2.83554818
NONHSAG006726	2.96880051	2.8417443	2.85967358	2.78859323	2.62760197	2.9618879	2.92124992	2.69481259	2.83194898
NONHSAG006750	2.2453354	2.26074478	2.36892399	2.45202756	2.5211055	2.49906738	2.58900038	2.34961267	2.27427109
NONHSAG006793	6.22168663	5.91686712	5.96516272	5.72738383	5.88443479	5.84213188	5.95239569	5.95964313	5.87565999
NONHSAG006798	3.26488849	3.10807444	3.00953986	2.97919276	3.06947345	3.10504943	3.13954996	3.08824158	3.06448781
NONHSAG006806	4.65989996	4.47098508	4.39439431	4.16840469	4.46834165	4.64696174	4.42136035	4.57662135	4.13740928
NONHSAG006870	3.40681296	3.19141888	3.24411807	3.19189298	3.25870597	3.32330377	3.26981687	3.14562493	3.12092005
NONHSAG006890	6.09545353	5.83623395	5.90044322	5.66400883	5.80953319	5.66660709	5.97311329	5.79910223	5.94179748
NONHSAG006892	5.16443335	4.80867478	5.2362969	4.99906008	5.01592185	5.01511605	5.13555937	5.13649224	5.13275717
NONHSAG006896	2.88181594	2.83417433	2.50526969	2.33054484	2.53957079	2.67290363	2.66857826	2.8305913	2.57590082
NONHSAG006898	6.57527836	5.55512374	5.29421825	5.22900271	5.64078495	5.61369386	6.26528929	5.80076267	5.19783556
NONHSAG006900	4.49508205	4.87860794	4.88530564	4.60585312	4.57042993	4.92512608	5.12458668	4.79705541	4.73983945
NONHSAG006943	2.96151001	3.092249	3.39447759	3.08447595	2.85249211	3.45303159	3.04061507	3.44577992	2.83672027
NONHSAG006949	2.31191597	2.24445146	2.44843877	2.48165356	2.41898339	2.42244383	2.38402938	2.58788152	2.26968003
NONHSAG006969	3.80655261	3.80228829	3.59873477	3.7684298	3.62362218	3.87122864	3.95989797	3.72099066	3.57090142
NONHSAG006971	5.78512237	5.2014107	5.44054911	5.36316108	5.25089609	5.30100047	5.55869659	5.15125562	5.42068513
NONHSAG006980	2.30887049	2.34415373	2.09895387	2.46465732	2.12459668	2.22138314	2.36826668	2.41934753	2.46907043
NONHSAG006992	4.11289823	3.89655766	3.76395114	3.93091625	3.70205211	3.76678416	4.03056925	3.89823685	3.70661575
NONHSAG007038	6.66933124	5.90586503	5.95492032	5.74984998	5.80385801	5.79106543	6.39119916	5.93166767	5.84445695
NONHSAG007047	2.50353022	2.5014215	2.77086141	2.97020072	2.76725874	3.42186006	2.92124992	2.7227344	2.47093481
NONHSAG007108	3.04037773	3.10321184	2.67373815	2.47667875	2.48427513	3.05891773	2.62242585	2.57116365	2.88518349
NONHSAG007115	2.58374788	2.23743128	2.38983856	2.2657762	2.29982274	2.43904531	2.35779679	2.35161432	2.36055308
NONHSAG007133	3.25482168	3.89522952	3.69143388	3.75206013	3.6784412	3.94870638	3.85725177	3.95887587	3.87581016
NONHSAG007156	5.38232197	5.67291704	5.25759931	5.64183931	5.25216142	5.42317776	5.35230422	5.44664711	5.17985143
NONHSAG007158	5.07821963	5.07163234	4.76034335	4.78316183	5.00281997	5.18092865	5.02385344	4.8078074	4.61131746
NONHSAG007166	4.17254993	4.45171629	4.16648575	4.69330939	4.10856493	3.96422028	4.38661243	4.615887	3.96245255
NONHSAG007181	5.61223316	5.46916226	4.82147507	5.11411213	5.16441957	5.25847451	5.49236234	5.2016411	5.08596362
NONHSAG007207	5.54743111	5.20108973	5.20433322	5.19530552	5.24768062	5.46068549	5.22153103	5.28634852	5.25300227
NONHSAG007221	4.02844908	3.69624787	3.62175131	3.50923858	3.39993034	4.2429661	3.49840172	3.80511711	3.80999171
NONHSAG007222	5.13920743	5.13796367	4.79759902	4.78852301	4.65584275	5.11440468	4.75789923	5.09614942	4.67498746
NONHSAG007253	4.82748006	4.90665429	4.82217051	4.98128366	4.7400126	4.97691071	4.65603587	4.71168713	4.54235065
NONHSAG007359	6.26938955	6.45048149	6.16407469	7.11444227	6.73671404	6.31337585	6.61921293	6.52863005	6.77171636
NONHSAG007368	5.36665995	5.35405597	5.59725377	5.26191977	5.29083922	5.32067303	5.4506049	5.38298029	5.05560174
NONHSAG007373	4.97236129	4.86954398	4.78534734	4.93152323	4.69983585	5.01458678	4.80362142	4.89945787	4.71710948
NONHSAG007386	5.14149496	4.60064528	5.07746862	4.81283121	4.66551694	5.13751185	5.21388307	5.08805859	4.68192993
NONHSAG007407	4.85779705	4.95853558	4.58682138	4.70693591	4.40997065	5.00694267	4.51588163	4.89203427	4.69288549
NONHSAG007504	4.58367394	4.67819078	4.45803531	4.67999107	4.81232915	4.58842031	4.7045529	4.7643988	4.49510313

NONHSAG007506	2.64381408	2.31066801	2.42040342	2.42436772	2.46253528	2.45533363	2.47707448	2.50158945	2.46768319
NONHSAG007524	3.6692266	4.0267869	4.09541643	3.98304391	4.01432246	3.96602356	3.89228276	4.0331481	3.91401581
NONHSAG007531	4.25907771	4.33932257	3.83845754	3.92129018	3.90424819	4.28791775	4.04682895	4.25699492	3.90536011
NONHSAG007550	4.37450958	4.86918111	4.44423049	5.05507823	5.08908028	4.61508892	4.1703192	4.44473329	4.58054884
NONHSAG007560	2.95707585	2.66153315	2.82714752	2.68609121	2.7780451	2.72212721	2.78177036	2.73692268	2.9326724
NONHSAG007562	2.64878824	2.67524825	2.65069567	2.85363055	2.59241101	2.37361473	2.65122875	2.75550384	2.56275808
NONHSAG007577	4.24727827	4.58110475	4.38743021	4.12957009	4.10545074	4.54127488	4.30451988	4.47315391	3.92022286
NONHSAG007580	4.83041283	4.90594702	4.95285429	4.95728331	4.70968356	5.13678767	5.04264762	5.02473536	4.76895947
NONHSAG007590	6.29154294	5.49188098	5.49430251	5.38871791	5.4607983	4.87013204	5.97496516	5.33622905	5.2306034
NONHSAG007653	3.05390726	3.25357253	3.27134131	3.14618088	3.02458274	3.10458703	2.97045733	3.2153558	3.20231486
NONHSAG007683	3.53105814	4.21109252	3.73660402	3.65059029	3.7150255	3.73734286	3.70901349	3.88207426	3.45124973
NONHSAG007686	3.87168069	4.00868551	3.78576189	3.6485273	3.67496898	4.03029267	3.84415259	3.91228683	3.56411135
NONHSAG007695	4.3022245	4.19554734	4.26760515	4.41357755	4.14854453	4.45978745	4.33423274	4.23988493	4.16109365
NONHSAG007714	3.50199981	4.12111508	3.95086159	4.12678382	3.8692594	3.91261441	4.15367147	4.22317168	3.59162293
NONHSAG007751	7.14704054	6.24659482	6.37044769	6.20135202	6.28167392	5.95730754	6.30640777	6.11225344	6.43505158
NONHSAG007766	6.8152139	6.35742499	6.70994506	6.59073567	6.64517379	6.45630989	7.03540744	6.94486449	7.19430795
NONHSAG007767	4.06264247	3.70633969	3.43443949	3.61531545	3.60003094	3.64672984	3.4332827	3.33438814	3.87843652
NONHSAG007770	3.13047061	2.71387151	2.89562207	2.86946862	2.94372171	2.74236977	2.85260387	2.85220734	3.00842974
NONHSAG007825	3.15702504	3.52024867	3.40935647	3.54181167	3.6988805	4.08318438	3.58953049	3.66097782	3.39703399
NONHSAG007828	3.29244649	3.13771294	3.03230425	3.06552569	3.17610923	3.18928427	3.14533855	3.01788122	3.1363076
NONHSAG007837	4.18492873	4.74042164	4.3993977	4.39629413	4.08792674	4.37146337	4.35568023	4.4221199	4.22486465
NONHSAG007839	4.85151118	5.12020072	4.59403807	4.60024582	5.07070919	4.75639812	4.82687763	5.36363852	4.75765204
NONHSAG007862	3.83458269	3.08113095	3.26829018	3.24516756	3.27023479	3.37297322	3.18079193	3.41673688	3.31109229
NONHSAG007868	3.52073954	3.05047869	3.17272158	3.40245398	3.21278716	3.18266054	3.14101051	3.27725657	3.49750965
NONHSAG007885	2.59060019	2.76265107	2.5849462	2.53431867	2.5859336	2.76692122	2.57643165	2.51753194	2.61561066
NONHSAG007890	3.12511641	3.02543586	3.11176199	3.07223231	3.23397144	3.04763207	2.90917828	3.26126268	3.06150103
NONHSAG007906	2.42329481	2.33195843	2.29492541	2.30752247	2.39324486	2.4496308	2.38187453	2.46389877	2.34182133
NONHSAG007910	3.65499477	3.62433951	3.63977502	3.58805446	3.60057181	3.77446307	3.64304685	3.55195943	3.48023498
NONHSAG007917	2.61177337	2.53988093	2.50057867	2.52822443	2.67185047	2.73390812	2.55601743	2.41304288	2.48372245
NONHSAG007923	3.45610718	3.26914072	3.14040042	3.26639552	3.2397955	3.4342644	3.23136592	3.38571578	3.04435366
NONHSAG007937	3.0465151	3.24745067	3.15014612	3.29390286	3.39588343	3.2832778	3.07242716	3.18478451	2.98661487
NONHSAG007942	3.15683401	3.06133315	3.006602	3.11437682	3.06070511	3.27446657	2.97292023	3.11176199	3.1303087
NONHSAG007976	4.15547344	4.18751904	3.878861	3.89980269	3.93705789	3.93938043	3.99536805	3.98449714	3.77276391
NONHSAG007987	2.61283489	3.04817205	2.90305525	2.5905442	2.49447953	2.65986049	2.99230764	3.06592869	2.44813063
NONHSAG008006	4.61188303	4.61064832	4.76255709	4.70475393	4.69893041	4.64069918	4.73088236	4.82197462	4.77590595
NONHSAG008015	4.1330663	4.89264884	4.21318555	4.57587661	4.3085902	4.62392413	4.62478691	4.67380204	4.25865322
NONHSAG008021	3.20490532	3.33435799	3.26865522	3.24410074	3.25316647	3.43460527	3.47477825	3.42950904	3.2140242
NONHSAG008036	3.58174036	3.69981517	3.44422693	3.51228816	3.44796744	3.5635095	3.72260969	3.57880082	3.29985546
NONHSAG008052	3.44155651	4.1080854	3.71811052	3.98608957	3.89882301	4.09922865	4.59877326	3.83457671	3.98741153
NONHSAG008061	4.882707	4.42703949	4.00950747	4.24983925	4.47888665	4.3142778	4.42646855	4.15954445	3.96374902
NONHSAG008068	2.41227807	2.39536427	2.04871751	2.37607558	2.27663474	2.75098945	2.52883363	2.44606147	2.29242751
NONHSAG008074	3.71030205	3.71598684	3.71478434	3.60507529	3.7159415	3.79546563	3.50006581	3.74221007	3.73774951
NONHSAG008085	2.24586745	2.07467866	2.10451855	2.25770371	2.30323111	2.20328411	2.16379762	2.1573877	2.08946768
NONHSAG008088	2.83969931	3.1747457	2.97860067	3.05870305	2.73153812	2.80157872	3.10292122	3.11290228	2.69035803
NONHSAG008123	5.14844318	4.75359712	4.74660234	4.54826897	4.57817971	5.079357	4.61669709	5.17858965	4.66601668
NONHSAG008125	2.86101844	2.5442739	2.58534817	2.58701911	2.54136194	2.58110306	2.47875739	2.44585379	2.79334539
NONHSAG008197	3.796532	3.84891196	3.59020038	3.76100137	3.66263321	4.0191398	3.65973855	3.72220819	3.27128234
NONHSAG008233	3.26517257	3.0389302	2.84583463	2.95751181	2.9075989	2.92387792	3.14847733	2.94172618	2.92033841
NONHSAG008613	3.97066756	3.98805965	3.50251262	3.53892813	3.73714107	3.43237468	3.44456422	3.72010232	3.44331452
NONHSAG008637	5.11537535	5.44315721	5.00837475	5.00803163	5.1203989	5.36266797	5.1235939	5.18398685	5.00690791
NONHSAG008664	5.11465456	5.33237733	5.04745633	5.30441584	5.29371895	5.26875851	5.36448132	5.2712439	5.17155301
NONHSAG008670	7.11966354	5.77599153	5.60316546	5.51274929	5.5072009	5.56013112	6.08599583	5.97748916	5.94656042
NONHSAG008671	11.03438518	9.56395712	8.81209367	9.17996678	9.02362886	9.03055485	9.88221064	9.70870199	9.39086854
NONHSAG008684	3.97097589	4.08881154	4.14603729	3.860134	3.95486886	3.91297389	4.21426967	4.02987702	3.54690373
NONHSAG008765	5.17350174	5.14147271	5.66581336	5.60748883	5.61926946	5.35525972	5.43059306	5.36101436	5.37584451
NONHSAG008818	5.00097853	5.38441495	5.46889496	5.48688813	5.52221493	5.13196292	5.40227626	5.53035946	5.42031762
NONHSAG008819	4.36841324	4.55108293	4.64319845	4.56262852	4.61882195	5.35592021	4.82598819	4.27360761	4.38441176
NONHSAG008903	3.85401712	4.34478092	3.89361326	3.78111902	3.72519063	3.77992664	4.17225725	4.32913228	3.69076739
NONHSAG008932	5.38589169	5.24598565	5.00330686	5.05867623	4.97124198	5.22615378	4.94356106	5.13237087	5.11196687
NONHSAG008943	4.68725813	4.81922016	4.81456683	5.00250292	4.81743541	4.90201922	5.21369808	5.0651471	5.0053885
NONHSAG008986	2.54177141	2.2732092	2.21500787	2.07678609	2.5130166	2.12736944	2.21302154	2.33890189	2.08289759
NONHSAG009036	3.436735	3.66308795	4.12919396	3.70515834	3.53825803	3.92826428	3.87881043	3.89891755	3.7838078
NONHSAG009072	6.4421856	6.62598565	6.28058199	6.27842023	6.39539974	6.592963	6.32930092	6.53090557	6.2934045
NONHSAG009076	2.85309292	2.74024615	2.89165775	2.74945756	2.58483035	2.79423307	2.9268868	2.62746409	3.24546765
NONHSAG009130	6.55387444	6.88021157	7.04848348	7.00927081	7.01293139	6.75297422	7.12096398	7.06932043	6.94851084
NONHSAG009132	2.38695516	2.89348147	2.71835109	2.5841199	2.75302251	2.67245033	2.63020175	2.70636687	2.47796886
NONHSAG009142	5.29114884	4.97059478	5.02129112	4.92046746	5.34445031	5.11757061	4.89998319	4.9789286	5.01409082
NONHSAG009168	8.89386718	9.28841761	9.36022795	9.33830787	9.43292354	9.26632108	9.32008665	9.26800292	9.19180988
NONHSAG009184	3.49571803	2.99460307	3.20199331	3.18625748	3.29296942	3.20066705	3.39281897	3.28869293	3.23143409
NONHSAG009186	3.37692161	3.83427004	3.27461959	3.15644557	3.29193819	3.67321926	3.5536443	3.29143272	3.29573691
NONHSAG009191	3.22564634	3.0727733	3.30334283	3.46560804	3.15366611	3.24992954	3.2335051	3.18383106	3.08393982

NONHSAG009210	4.55143325	5.24492132	4.47593398	4.53740233	4.70989865	4.86344234	4.62529875	4.73646262	4.34861454
NONHSAG009216	2.86101844	3.05368665	2.46318162	2.80631324	2.91631376	2.98381391	2.90095272	2.8508295	2.55393699
NONHSAG009222	4.97967979	4.57466008	4.54425225	4.69755994	4.49052846	4.46489012	5.0933302	5.07156892	4.72795956
NONHSAG009269	4.66890184	4.73238303	4.43669863	4.24277865	4.33899342	4.64447652	4.68265733	4.93882985	4.10852443
NONHSAG009275	2.50269137	2.52182163	2.31381786	2.39007212	2.38990739	2.63460791	2.77623225	2.53437385	2.43677693
NONHSAG009283	5.00334709	4.43076025	4.42655088	4.51638871	4.48827048	4.65064967	4.50613727	4.35952972	4.59548571
NONHSAG009302	3.3404199	3.69370768	3.40826591	3.33070361	3.26501019	3.59116345	3.29665692	3.42324156	3.26396872
NONHSAG009308	2.61501003	2.35819136	2.74417866	2.30007527	2.62288493	2.64662892	2.8435018	2.80195621	2.69282469
NONHSAG009381	2.47906311	2.61869426	2.48705732	2.569547	2.7461588	2.4480676	2.59595877	2.77770583	2.41752103
NONHSAG009397	3.01892363	3.05554293	2.75028303	2.95420656	3.12664792	3.15932288	3.06959562	3.00487354	2.93777554
NONHSAG009424	3.95016214	3.50044863	3.5549995	3.50044863	3.55023642	3.79501845	3.70091386	3.38151862	3.62030092
NONHSAG009434	2.95837769	2.87574405	2.63834208	2.58771688	2.36789531	2.71957782	2.63448525	2.34359455	2.5677495
NONHSAG009452	4.39631571	4.43771965	4.14890283	4.28791491	4.06013554	4.23590853	4.28540572	4.39202348	4.19097542
NONHSAG009462	3.76029683	3.69217936	3.42398826	3.75073314	3.48896869	3.85304111	3.65073989	3.99533938	3.78506447
NONHSAG009465	2.84719454	2.73347299	2.86215177	2.76612242	2.61390699	2.96217272	3.07206742	2.98718859	3.0210239
NONHSAG009524	2.90931085	3.14200122	2.83249277	2.87559318	2.74736132	3.02516517	2.90743683	2.91688728	2.99741263
NONHSAG009530	3.06463657	3.06801708	3.38985816	3.04673533	3.21930333	3.04000062	2.86505782	2.99702134	2.99942027
NONHSAG009590	2.34109	2.4938236	2.7094215	2.5014215	2.38827463	2.42997281	2.56360813	2.62326459	2.55569118
NONHSAG009627	5.48349859	5.70616423	5.46749646	5.72429459	5.81708264	5.84179304	5.55562962	5.7620767	5.56138411
NONHSAG009630	2.45792488	2.76883001	2.51727039	2.89277535	2.56916284	2.65951551	2.52627519	2.59596486	2.34334311
NONHSAG009643	4.94874587	4.33501577	4.24234043	4.39370047	4.31888614	4.35629712	4.42893128	4.2922172	4.33253091
NONHSAG009663	2.97581729	2.881996	2.65621224	2.64229023	2.47114658	2.56163702	3.01966474	2.69466328	2.69433083
NONHSAG009678	4.65421235	4.29541346	4.08587307	4.10322108	3.90995441	4.30787939	4.00234371	4.03869451	3.7534045
NONHSAG009688	3.39878256	3.50022164	3.23993358	3.43760667	3.16894626	3.33533474	3.36476399	3.72189227	3.2803926
NONHSAG009690	4.65932707	4.91397451	4.62212186	4.74091294	4.63256166	5.01352892	4.631971	5.10638	4.72828601
NONHSAG009700	3.40368186	2.89015114	3.15068623	3.08834582	3.27626102	2.96547361	3.12046288	3.28732062	3.17566304
NONHSAG009711	3.14146601	3.03838951	2.80215168	2.94452899	2.78469284	3.16702781	2.83000663	2.96043122	2.97743127
NONHSAG009736	3.21767748	3.1654059	3.36717412	3.11437682	3.08134457	3.47475547	3.11023515	3.21731673	3.15199321
NONHSAG009783	2.55248642	3.06723323	2.61013797	2.69174135	2.99853347	2.78072472	3.11176199	2.97942336	2.59506635
NONHSAG009834	4.5478851	4.96348772	5.17737417	4.93207434	4.99365449	5.01305455	4.78694828	4.91316192	4.57974309
NONHSAG009878	2.38985102	2.35031336	2.1973916	2.31156416	2.38694743	2.40807746	2.36292502	2.43068488	2.35800927
NONHSAG009880	2.34722834	2.23254715	2.2064819	2.10629859	2.22006839	2.12940138	1.98204365	2.2031364	2.28514881
NONHSAG009903	4.14164384	4.49963104	4.40917023	4.15062461	4.11536577	4.3761379	4.45937355	4.41185013	4.14594158
NONHSAG009919	3.82570398	3.76140141	3.59738052	3.60471646	3.48684398	3.83861878	3.52743677	3.91659348	3.67201492
NONHSAG009921	4.1744028	4.49000428	4.04718561	4.21442167	4.01819776	4.34408205	4.2187998	4.32560075	3.85514731
NONHSAG009942	3.60288931	3.29699925	3.45459145	3.45475409	3.48818461	3.47805255	3.37299946	3.22915889	3.36341779
NONHSAG009977	4.2089905	4.477138	4.47205269	4.32995465	4.23344312	4.77471825	4.2489171	4.5510154	4.26907571
NONHSAG009994	5.63907544	5.34264553	5.61039545	5.59752191	5.73637867	5.33581478	5.74789345	5.44660082	5.51933935
NONHSAG010005	3.04421449	2.49189793	2.69644743	2.55010152	2.47096336	2.30792047	2.75453833	2.49551492	2.5577071
NONHSAG010007	3.10216904	3.07500764	2.91793795	2.84949196	2.7644828	3.14004596	2.90294234	2.89384827	2.72572239
NONHSAG010015	2.58489628	2.65570216	2.4790989	2.42517016	2.40350458	2.54958099	2.50137999	2.5064409	2.49484468
NONHSAG010022	3.11650182	3.27045201	2.90322566	2.93450177	2.85254812	3.2141566	3.51106779	3.29124406	2.93737851
NONHSAG010026	3.9115689	4.46262017	4.24415581	4.17964035	4.13677948	4.58982766	4.36007594	4.38846831	4.26653858
NONHSAG010034	3.27660321	3.28153891	3.2437307	3.14271186	3.05204621	3.24983972	3.29954365	3.42677018	3.10884956
NONHSAG010061	4.05315626	3.88991625	3.66358125	3.77299864	3.6667565	3.91840459	3.74848121	3.62841297	3.90493743
NONHSAG010086	3.51787017	2.95705629	2.83301856	2.85337591	2.69278928	2.92688295	2.84336035	2.96908933	2.86936817
NONHSAG010089	3.52299286	3.11052143	2.9827834	3.28034532	3.12359624	3.31140859	3.02601894	3.21480342	3.10736075
NONHSAG010109	4.67302897	5.02629678	5.09287625	5.04531043	5.03296658	5.25370741	5.01116653	5.27010762	4.92037103
NONHSAG010134	6.08783079	5.08352199	5.68087047	5.35957697	5.46754323	5.25366084	5.49968308	5.05719474	5.60679117
NONHSAG010144	4.32027322	4.66436208	4.0500336	4.38964062	4.24782495	4.38407955	4.55869262	4.27354639	4.20156491
NONHSAG010152	4.42788953	4.42012604	4.58309244	4.28760197	4.42416534	4.31960587	4.53466577	4.46701232	4.43772997
NONHSAG010157	3.00920534	2.54275645	2.58654697	2.45261325	2.72497951	2.55789627	2.72820286	2.76578418	2.4673898
NONHSAG010160	4.36500668	3.91755354	3.75506696	3.67046909	3.70357027	3.80294821	4.2492387	3.79105039	3.78394514
NONHSAG010163	2.53387666	2.96043045	2.65855406	2.73004383	2.78617566	2.77238893	2.51378431	2.89127477	2.52242898
NONHSAG010226	4.33259094	3.94802952	4.13085307	4.13769362	4.0235672	4.26631546	4.03497148	4.22561184	4.23507212
NONHSAG010232	3.10566553	3.06005409	2.70429411	2.81515685	2.76342037	2.80959805	2.82495176	2.79267553	2.88728411
NONHSAG010260	4.65013871	4.86164407	4.76762419	4.69944891	4.73570532	4.70306675	4.64898295	4.69335535	4.77809039
NONHSAG010262	5.36227915	5.37132601	4.85004221	4.80059934	5.06211762	5.33624875	5.00470183	4.98904031	4.73165705
NONHSAG010264	5.43447682	5.07660996	4.89677494	4.99827273	4.97422756	5.24000175	5.1075196	4.87201233	4.99294338
NONHSAG010352	5.7626753	5.81996501	5.61563998	5.48545361	5.69423601	5.8980596	5.59985208	5.67325424	5.75123339
NONHSAG010413	2.09792751	2.27142431	2.26728647	2.08404902	2.20724228	2.28569004	2.5206553	2.22607516	2.37109002
NONHSAG010420	6.72048962	6.25880881	6.668767	6.2997258	6.15423261	6.26387845	6.34992621	6.31653178	6.33962394
NONHSAG010447	2.57498379	2.64363539	2.4021887	2.44162135	2.24821798	2.59837395	2.55972711	2.51832694	2.41178971
NONHSAG010455	6.79257159	6.26903208	6.39407711	6.13047333	6.20944341	6.19665183	6.07141697	6.17288287	6.2326324
NONHSAG010492	2.93631235	3.10301022	2.91878069	2.71519673	3.00440203	2.99101179	2.92124992	2.88643801	2.85356201
NONHSAG010499	2.41796722	2.36570465	2.48582022	2.45226381	2.38694743	2.64975585	2.49825815	2.28088524	2.51647855
NONHSAG010524	2.55823842	2.32708239	2.36124659	2.58057629	2.51502199	2.14653201	2.43144359	2.39024789	2.39355085
NONHSAG010532	4.6440645	4.93881146	5.30270892	4.74748474	5.06596531	5.00359223	5.20307006	5.18381413	4.82222502
NONHSAG010566	3.10287956	2.44310804	2.29221888	2.41627242	2.69051105	2.64464749	2.39706084	2.40573791	2.58677801
NONHSAG010571	5.00206904	4.0860593	3.97659482	3.95268216	3.87370236	4.18456135	4.22385765	4.22561184	3.94402717
NONHSAG010573	6.37488431	6.02947508	6.29749931	6.08341799	6.21402611	5.83694979	6.61958161	6.09236473	6.22557909

NONHSAG010585	2.93962748	2.97021867	2.78881359	2.83185706	2.94649792	2.95674722	3.43072646	2.87155407	2.89157769
NONHSAG010628	5.33815009	5.45075576	4.73481885	5.11827441	5.02202267	4.91492535	5.11246148	5.52161607	4.52566027
NONHSAG010678	2.90293066	3.19155198	2.94357797	2.84274146	2.88223799	3.21793387	3.10068654	3.25640793	2.99903614
NONHSAG010682	2.99484468	2.83879691	2.8430931	2.78425182	2.75648625	2.77961633	2.83576946	2.72437944	2.6053736
NONHSAG010721	2.52611281	2.47215182	2.7911195	2.70200027	2.47703448	2.80959805	2.50620855	2.57835103	2.53237025
NONHSAG010724	2.5746035	3.12957293	2.939689	2.60775284	2.89130449	3.21311846	2.91611256	2.8964451	2.90287814
NONHSAG010765	3.41872932	3.33178781	3.13885433	3.00612676	2.96890078	2.8056407	3.03644362	3.13577704	3.07731472
NONHSAG010767	3.04308767	2.89911579	3.01492995	2.88277328	2.81701057	2.97271895	2.91391119	2.75674215	2.83074106
NONHSAG010791	2.36138496	2.22591189	2.1553549	2.32593483	2.30415086	2.35011078	2.19205435	2.41596038	2.35011078
NONHSAG010808	4.44712438	4.64063423	4.33933935	4.13583998	4.15380148	4.41306355	4.18852456	4.59704636	4.45427743
NONHSAG010846	4.44808284	3.79517631	4.40764176	4.12848365	3.96657926	3.46213046	3.97636524	3.80082674	4.09560438
NONHSAG010848	4.58894696	3.83211611	3.86408973	3.85727264	3.69611066	4.12753097	3.97303471	3.7081538	3.89314018
NONHSAG010888	2.64813019	2.77275627	2.4851965	2.66875861	2.64404005	2.94241153	2.66121929	2.64175479	2.6692075
NONHSAG010892	3.87919373	3.24004185	3.06492254	3.12121435	3.41260967	3.36526708	3.06880154	3.34086357	3.35139574
NONHSAG010908	2.3598993	2.25265807	2.07027349	2.05251867	2.21508432	2.20060806	2.13795116	2.12552491	2.20176018
NONHSAG010921	3.43295641	3.31708076	3.32906668	3.33958278	3.23078872	3.09171253	3.50118706	3.07317068	3.10533258
NONHSAG010938	4.91615662	4.51374393	4.5128771	4.2825134	4.67805898	4.51738726	4.27614322	4.44993612	4.51889577
NONHSAG010946	5.57137573	5.57539161	6.20204315	5.63444667	5.85462899	5.68226758	5.80966086	5.5421414	5.70854527
NONHSAG010964	3.40681296	3.69959433	3.21474245	3.18393676	3.60040697	3.46871928	3.50610396	3.37471783	3.49923447
NONHSAG010989	3.13831543	3.35632856	3.30691158	3.24676473	3.02922128	3.48673257	3.55140487	3.20225308	3.52429621
NONHSAG010991	3.12820282	3.16435686	3.00669405	3.04697559	3.03103791	3.21670839	3.02275115	2.97756983	3.02243983
NONHSAG010992	5.96541144	4.56375935	4.92442981	4.49651878	4.74914427	4.77524071	6.26679621	4.78097699	5.05447737
NONHSAG011000	4.79585706	4.96679839	4.46789569	4.53816263	4.6020745	4.62707786	4.58903478	4.66127087	4.42844031
NONHSAG011016	4.11515637	4.48361909	4.00992813	4.36568427	4.32078332	4.38679128	4.29363078	4.23648395	4.04878265
NONHSAG011035	2.64501867	2.48676109	2.9365162	2.65882225	3.16433153	2.99789275	2.77302293	2.46523713	2.78972977
NONHSAG011091	3.61112015	3.58468879	3.55740956	3.51705861	3.58525852	3.84184649	3.63092547	3.63287152	3.26728296
NONHSAG011182	5.15510994	5.16639901	5.1534638	5.32954528	5.28156068	4.85713397	5.22414623	5.56972919	5.16458324
NONHSAG011270	3.92053697	3.95363752	3.55455355	3.77767593	3.45490863	4.11237797	3.57644138	3.88367971	3.50066309
NONHSAG011279	4.10448566	3.75499053	3.41423366	3.34796032	3.9311341	3.7488757	3.6035033	3.81346209	3.71625947
NONHSAG011288	8.60245602	8.47154497	9.14291225	8.60927092	8.29707147	8.13998159	8.48789958	8.58132375	8.2905834
NONHSAG011425	4.66547517	4.58834042	4.60555946	4.36406959	4.56670412	4.99826419	4.66875731	4.22054074	4.17107412
NONHSAG011459	5.91125	5.97823538	5.47019427	5.57843456	5.42818523	6.1136163	5.6009884	5.79276578	5.442156
NONHSAG011460	5.75168585	5.41264347	5.36973524	5.24611343	5.1946504	5.37671785	5.34319561	5.51204287	5.361613
NONHSAG011467	2.98463703	3.35327523	2.90238326	2.69795427	2.81180717	3.04624802	2.95451964	3.17604472	2.70010823
NONHSAG011471	3.43287619	3.65865989	3.35090878	3.30022661	3.38949319	3.53591267	3.48463091	3.47398472	3.31729374
NONHSAG011496	3.90641479	3.96620365	3.59941629	3.59811069	4.09658231	4.03935023	3.66610844	3.96862221	3.64829385
NONHSAG011553	3.51442137	3.53016598	3.31833871	3.30389063	3.29509246	3.3740726	3.34924819	3.29358625	3.47211348
NONHSAG011555	3.60233252	3.60656029	3.26402699	3.16737441	3.36659519	3.41603132	3.41876898	3.35885479	3.15377645
NONHSAG011584	4.7921517	5.10332914	5.06612662	5.03654913	5.21281165	5.0452274	5.08209223	5.0025985	5.16980506
NONHSAG011594	3.16858579	2.95345521	2.77021387	2.87101438	2.82544883	2.9362654	2.96160728	2.97021193	2.9801246
NONHSAG011643	2.82688385	2.56265961	2.65855406	2.91789171	2.73352876	2.62478773	2.63007377	2.37547763	2.64349032
NONHSAG011647	2.58632997	2.62986024	2.48127951	2.90295625	2.50769814	2.75007182	2.6682496	2.62448416	2.53903437
NONHSAG011680	2.9779294	3.44500352	3.54480447	3.56167572	3.32672389	3.28846764	3.65861183	3.28604795	3.50999165
NONHSAG011703	5.32242051	4.22679102	4.12746492	4.33372845	4.43101525	4.88293798	4.43000622	4.33106912	4.4369355
NONHSAG011730	4.09888445	3.52611692	4.08981592	3.67867813	3.8201562	3.87750591	3.81454271	3.5423547	3.97755073
NONHSAG011744	3.71950904	3.85233356	3.65681885	3.36074382	3.41636622	3.70301933	3.5998263	4.2101096	3.51703849
NONHSAG011747	5.85336049	6.64806405	6.75273895	6.8954686	6.48215516	6.37786591	6.46709316	7.12765999	6.34235769
NONHSAG011762	3.55656262	3.39999624	3.29303241	3.36455077	3.16338142	3.51538471	3.54020247	3.48310132	3.33921534
NONHSAG011768	2.30564577	2.05608206	2.32377279	2.15498536	2.22549806	2.35779679	2.28662949	2.41767621	2.43734213
NONHSAG011773	2.50729555	2.34972294	2.14864591	2.17666137	2.08138902	2.41343679	2.14221167	2.22567488	2.27705125
NONHSAG011781	7.16231018	6.28918132	6.59085106	6.37238192	6.39071093	6.10463392	6.50311121	6.26252951	6.40461353
NONHSAG011800	2.75010673	2.67530257	2.56671506	2.15966007	2.45046953	2.81541367	2.44991812	2.84989595	2.44948988
NONHSAG011802	9.67460023	9.03564584	9.21342322	9.06560794	9.03009715	8.88783971	9.3418535	9.10330811	9.20542989
NONHSAG011805	2.84652671	3.10226511	3.21064378	3.15526778	3.17732467	3.20920327	2.98301758	3.15412032	3.02713505
NONHSAG011821	2.46394074	2.76279809	2.52027241	2.5425483	2.44671877	3.01840676	2.80734575	2.78458543	2.56116558
NONHSAG011830	2.29550018	2.19710444	2.24332068	2.49907303	2.39396122	2.29103898	2.43471409	2.25173289	2.20518556
NONHSAG011840	2.59802882	2.38235792	2.36664703	2.40851721	2.24587243	2.6757263	2.35779679	2.38571317	2.34822506
NONHSAG011877	5.72147407	4.25881712	4.38879334	4.41042063	3.94718142	4.1289909	4.20192655	4.28771314	4.68569263
NONHSAG011915	5.90316804	5.25832416	5.84466653	5.62343297	5.91967023	5.55530922	5.62548286	5.62158113	5.69212923
NONHSAG011924	3.32505233	3.07847007	3.48848429	3.1940104	3.26949751	3.06448219	3.29980335	3.2915686	3.46782467
NONHSAG011933	5.87335531	5.6530679	5.27012375	5.35604122	5.30680606	5.24681772	5.63409514	5.70477919	5.25801922
NONHSAG011964	5.92068164	5.99956257	6.47764767	6.18788899	6.00129093	5.97244183	6.18119357	6.01038492	5.91816045
NONHSAG011968	4.33365361	4.0409623	4.25541785	4.13177452	4.13709407	4.66465605	4.17941653	4.3421834	4.07747653
NONHSAG011977	2.66586618	3.02097044	2.75701032	2.83767004	2.94405327	2.74386067	2.81282019	2.82349787	2.89138851
NONHSAG011978	4.93961261	4.83953582	4.89708204	4.84207524	4.79603082	4.95642832	4.8106176	5.14000817	4.721199
NONHSAG012011	2.70242469	2.64771561	2.64090767	2.60443642	2.70455704	2.6465497	2.6603859	2.85135469	2.7581655
RMST	6.39186679	8.31455367	8.75987846	8.65430404	8.48701043	8.32643181	8.91532109	8.7301244	8.5520363
NONHSAG012031	4.10730655	4.31560436	4.22503898	4.0990044	4.20951605	4.33327193	4.0717352	3.9424172	3.93216413
NONHSAG012043	2.63797329	2.17585067	2.0265626	2.37144282	2.299598	2.2340582	2.32984056	2.30684578	2.19085492
NONHSAG012048	5.15972355	5.1102576	5.73920517	5.29771311	5.56851798	5.28921751	5.13250112	5.22644049	5.48481031
NONHSAG012050	6.75958866	6.67013881	6.94241332	6.72242121	7.08625923	6.72473565	6.84629562	6.87283511	6.86241301

NONHSAG012055	7.0761085	6.79778884	6.7733835	6.56260916	6.85279601	6.65037209	6.93737251	6.62886484	6.76996857
NONHSAG012099	2.70347265	2.4857514	2.22817327	2.46632597	2.37553419	2.56398214	2.40532164	2.3956628	2.53659326
NONHSAG012152	2.50353022	2.55149636	2.29197435	2.21592034	2.13669759	2.80959805	2.38508304	2.48359725	2.16985555
NONHSAG012158	4.15357339	4.50491839	4.39561316	4.41290436	4.30243731	4.55636753	4.40599785	4.6842482	4.66605818
NONHSAG012172	4.08711152	3.62406242	3.66009183	3.61965475	3.63744949	3.81090549	3.50779294	3.70966873	3.78439671
NONHSAG012182	4.16191216	4.12657572	4.12705583	4.22158789	3.93722431	4.24673575	4.02496437	4.09533327	4.07414732
NONHSAG012199	3.6698085	4.24262653	3.93853918	4.25474127	4.23083227	4.21532352	4.42597673	4.59795402	3.69357679
NONHSAG012226	4.317142	4.21418414	4.09277833	4.08291587	4.12761569	4.12222384	4.05794096	4.0943122	3.78610933
NONHSAG012236	3.05385332	2.63059392	2.59871425	2.4372407	2.5757789	2.86893063	2.38077459	2.63744289	2.730963
NONHSAG012367	3.10655449	2.83116784	2.90022517	3.03490184	3.28153891	2.91649055	2.98178093	3.04522672	2.90303134
NONHSAG012392	2.5312135	2.64113061	2.61011814	2.35666103	2.35692346	2.64833314	2.31303298	2.34645535	2.49449412
NONHSAG012393	4.87693686	4.9352344	4.84966801	4.40745125	4.60711667	4.96970397	4.55438763	4.26986128	4.55260011
NONHSAG012430	2.86757066	2.96728187	2.84138047	2.70470333	2.8116532	2.81589464	3.01544646	2.72385357	2.67881494
NONHSAG012433	5.14382151	4.89249738	4.95390495	4.72868792	4.72280912	5.03147632	4.82249965	5.13634791	4.8464132
NONHSAG012556	6.53305278	6.44098404	6.28326257	5.9279265	6.03673808	6.37743323	6.16463863	6.19853608	6.17752067
NONHSAG012565	4.45994265	4.42899836	4.42702543	4.19097056	4.22951112	4.44184721	4.37379838	4.37466741	4.26498167
NONHSAG012566	4.45552616	4.12129205	4.34417515	4.32058569	4.04166986	4.45991789	4.15153055	4.21360468	3.66703173
NONHSAG012599	6.10892277	6.67777751	7.18269964	6.8769507	7.08573652	6.85345515	6.89693192	6.49618553	6.8095991
NONHSAG012608	4.37423749	4.22079813	4.28748476	4.46846552	4.34867877	4.2085082	4.45410388	4.22635646	4.37876728
NONHSAG012629	2.60677673	2.43575294	2.46403016	2.35442141	2.43600528	2.43362318	2.4964307	2.32502785	2.6436053
NONHSAG012630	5.99775568	5.58998262	5.37972854	5.69481194	5.58698433	5.61472778	5.59197802	5.54126963	5.64500668
NONHSAG012635	3.21546396	3.57642206	3.31201742	3.05693604	3.36135611	3.55973674	3.29860088	3.36938406	3.05198594
NONHSAG012657	7.77593842	8.7349731	8.91436515	8.72351306	8.8894793	8.78024153	8.79410525	8.76859253	8.99534909
NONHSAG012658	9.38916635	10.54910473	10.669217	10.61953093	10.74186063	10.61431358	10.72973567	10.78134133	10.64261781
NONHSAG012682	3.33353429	3.3303444	3.04720027	3.16350539	2.74796634	3.32074815	2.95684303	3.45391196	3.10020358
NONHSAG012683	3.37092463	3.6684664	3.40692329	3.28858595	3.26238117	3.39265855	3.20744719	3.51401481	3.51316756
NONHSAG012684	2.26009265	2.72041177	2.35695805	2.47572481	2.43879989	2.52344344	2.83576946	2.69481259	2.41562278
NONHSAG012695	4.33110594	4.56242135	4.39128072	4.45893007	4.29058265	4.58969161	4.54436663	4.55656501	4.40639825
NONHSAG012727	5.55325353	5.0403519	5.32817467	5.28798717	5.23966768	5.74042437	5.10093556	5.51637191	5.27872247
NONHSAG012732	3.50474547	3.75391536	3.37446514	3.43884077	3.49204569	4.0530752	3.18726199	3.6625387	3.18432325
NONHSAG012739	5.46711527	5.83528426	5.28669251	5.43913714	5.30999732	5.79296742	5.33910281	5.45966058	4.90428772
NONHSAG012744	3.10148332	3.12890054	3.00896492	3.01062045	2.86409625	3.07919756	3.03695707	2.93583012	2.75899019
NONHSAG012745	4.15449417	3.74677766	3.86920433	3.96239519	4.01145367	4.17258411	3.8242349	3.99352991	3.41900356
NONHSAG012747	5.44407095	5.09195954	5.11681192	4.62441551	4.97221451	5.23553933	4.8089606	5.35632226	5.16504841
NONHSAG012753	5.86481836	5.91617415	5.67194128	5.89406254	6.05245298	5.93984223	5.74686456	5.95474334	5.68598914
NONHSAG012762	4.29875174	4.59410142	4.45815135	4.57890506	4.60681304	4.75724743	4.55401328	4.91729362	4.44860091
NONHSAG012764	4.4247466	4.53462575	4.39626862	4.5109766	4.75175802	4.55992121	4.34560355	4.57508883	4.8398737
NONHSAG012780	4.20587033	4.44338127	4.15532407	4.15815683	4.16756333	4.38998487	4.16274559	3.99501951	3.93147865
NONHSAG012793	6.56542323	6.59740921	6.76223554	6.52781042	6.75003977	6.66143742	6.42854093	6.39247649	6.44368498
NONHSAG012799	5.58182657	5.1380824	5.50639974	5.2858126	5.39073661	4.98384891	5.32959725	5.18808382	5.10336054
NONHSAG012823	3.69158465	4.05541472	4.17147631	4.15701475	4.21936861	3.86699982	3.98692905	4.19419852	4.08605028
NONHSAG012881	2.9264367	2.86571654	2.74231648	2.8045088	2.84369018	2.81315874	2.78452331	2.94246934	2.78526936
NONHSAG012884	2.77301182	2.89996669	2.69599011	2.48520519	2.69749145	3.2501603	2.45082408	2.42574866	2.7842444
NONHSAG012909	6.2313205	5.91099607	5.89725296	5.88515239	5.92546468	5.99555505	5.99082546	5.81042828	5.99316522
NONHSAG012910	6.2934045	6.24610185	6.14551666	6.19411986	6.10774662	6.2019603	6.47681654	6.69787486	5.95562643
NONHSAG012920	2.62112331	3.05479992	2.8368028	2.70170928	2.66444846	3.05283165	2.86813921	3.02548329	2.75879606
NONHSAG012971	3.13991727	2.17995014	2.2054694	2.40568545	2.59726322	2.41385024	2.14790146	2.33208911	2.03422282
NONHSAG012980	2.65942086	2.68732432	2.79315006	2.60667002	2.46691712	2.6888047	2.61400532	2.48353873	2.70244074
NONHSAG012986	8.20370288	7.84579394	8.07811641	8.15584409	7.99144661	7.80079959	8.11634134	8.22290822	7.96601417
NONHSAG013018	2.99855922	3.05453054	3.12804652	3.11328207	2.93905232	3.11997915	3.05220145	2.9064935	2.92656369
NONHSAG013032	2.64105505	2.76162601	2.6111954	2.58757252	2.86241008	2.78236805	2.70379228	2.82173792	2.71335822
NONHSAG013037	3.91845131	3.96661709	3.84024487	3.76341271	4.04703918	3.8027293	4.06753254	3.95975051	3.86461476
NONHSAG013039	3.81233009	4.05750726	4.10370315	4.05571297	4.09738437	4.12753097	3.96914774	4.48057883	3.91845017
NONHSAG013044	4.35109878	4.24540421	4.13824013	4.12025852	4.23025037	4.19844397	4.23528449	4.45348506	4.02591613
NONHSAG013052	4.47425813	4.6325553	4.50211371	4.60948935	4.37379006	4.86617441	4.20383218	4.64336259	4.48233639
NONHSAG013055	2.34464515	2.03877117	2.19074045	2.19391447	2.18256797	2.43904531	2.16110535	2.14379124	1.89299962
NONHSAG013061	3.32157528	2.91996061	2.73971539	2.79316542	2.8673823	3.00607347	2.73945548	2.75269761	2.96371325
NONHSAG013087	4.69396805	4.55445647	4.64115922	4.66893902	4.59427864	4.19063807	5.06074626	4.65489649	4.84726452
NONHSAG013088	4.99586732	5.050886	4.80381906	4.62573431	4.7176334	5.08277212	4.91058714	4.77185117	4.68135142
NONHSAG013092	3.21142892	3.24011396	2.80426603	2.7911782	3.07430347	2.9777972	3.03801656	3.04980706	2.93953816
NONHSAG013093	4.09249326	3.9678388	3.64218766	4.15794928	3.814859	4.3992903	3.67717993	4.15535534	3.9865198
NONHSAG013140	4.80285197	4.65637628	4.66168538	4.7165242	4.46784868	4.99818688	4.81001573	4.65519031	4.4080267
NONHSAG013147	2.79067334	2.91849455	2.7475106	2.81955746	2.5130166	2.99346358	2.81651819	2.77770583	2.66148973
NONHSAG013148	3.58763733	4.01667172	3.73667388	3.78178291	3.59419165	4.08201411	3.6874951	3.89896229	3.78070051
NONHSAG013176	3.34567764	3.184942	3.3047146	3.49052835	3.06837601	3.59523814	3.46555	3.26523428	3.29431073
NONHSAG013184	6.11178249	4.35469489	4.88801118	4.42557339	4.70255543	4.82583984	4.87015085	4.85891097	4.59834106
NONHSAG013195	6.02977116	5.94067416	5.50526817	5.57219934	5.57550568	5.56448605	5.66812444	5.88629719	5.66060277
NONHSAG013196	4.17002453	3.6467525	3.91925187	3.91130601	3.87068273	3.89723979	4.02095101	3.97544913	3.75885142
NONHSAG013213	2.73433633	2.51391871	2.35886664	2.56259873	2.61558057	2.65223699	2.43330616	2.66869317	2.3682625
NONHSAG013219	3.5798689	3.64204227	3.35652023	3.3127676	3.27907645	3.69023385	3.63736209	3.60408882	3.21831203
NONHSAG013224	3.26249598	3.29794181	3.26684473	3.07460729	3.06852288	3.03910376	3.32426075	3.06394849	3.24857266

NONHSAG013270	2.47402799	2.28085107	2.4226609	2.5424896	2.30223423	2.51991395	2.57377263	2.44547274	2.33148843
NONHSAG013289	3.68426387	3.47898853	3.4067739	3.53271209	3.52177159	3.58279609	3.45178377	3.59583376	3.58243499
NONHSAG013312	4.1648812	3.89065545	4.91911161	4.39290538	4.70204976	4.69138675	4.5748551	4.30247655	4.308053
NONHSAG013343	8.43490922	8.62903799	8.55179881	8.38510575	8.45358682	8.36033544	8.75688897	8.36183333	8.47998215
NONHSAG013371	4.39606068	3.61008665	4.13515236	4.06444869	3.97172884	4.08119056	3.97655697	4.1618144	3.8722878
NONHSAG013387	4.13171044	4.10380408	3.81370584	4.06529566	3.77741079	4.1256318	3.91580143	4.15142483	3.86228435
NONHSAG013400	6.89766759	6.84283147	7.10650224	6.79065598	6.99657113	6.68763399	7.06380172	6.7704442	7.00701715
NONHSAG013413	4.19013474	3.0887301	3.03952974	2.58785915	2.98359332	2.88355984	2.85778268	2.94374802	2.80959805
NONHSAG013415	5.19773911	4.2677286	4.43890282	4.26111824	4.34821852	4.42865046	4.26275601	4.61655605	4.4765914
NONHSAG013417	2.32926638	2.21862948	2.53506567	2.27983359	2.43040885	2.11800031	2.42234847	2.18011795	2.18953388
NONHSAG013418	4.55873351	4.68453256	4.41668589	4.49650074	4.42864691	4.43890621	4.57044691	4.63300463	4.23681212
NONHSAG013429	8.82334253	8.67611129	8.9129139	8.74099749	8.8375254	8.78583555	8.7914902	8.60652742	8.75359616
NONHSAG013433	2.36015434	2.41792908	2.74500551	2.62729404	2.29617866	2.82993251	2.64853683	2.49588683	2.49367985
NONHSAG013449	2.6953994	2.68288738	2.5193767	2.53073196	2.58359763	2.72017601	2.63134772	2.45957877	2.75777763
NONHSAG013472	3.46683781	3.30055623	2.99474378	3.41297482	3.05600905	3.10145491	3.33191577	3.27921846	3.13387025
NONHSAG013488	3.10634938	3.04188388	3.32667129	3.4193355	3.33861353	3.32594708	3.32848412	3.385161	3.33979392
NONHSAG013496	2.45824733	2.80617196	2.73165311	2.58531787	2.87485726	2.80959805	2.93197907	2.77729297	2.60708368
NONHSAG013505	5.49227013	5.86019483	5.63742969	5.68302943	5.47472567	5.62078747	5.66705174	5.83697915	5.41205399
NONHSAG013509	5.58319618	4.89207754	4.93541373	5.34968591	4.96797056	5.18647929	5.56514988	4.85928686	4.74284412
NONHSAG013521	3.63341707	4.23502501	3.40867438	3.31349727	3.22418907	3.98891588	3.76081295	3.27324422	3.35715132
NONHSAG013525	4.22648641	4.28921081	4.12932903	4.1025268	4.1482734	4.33155297	3.93281226	3.87794165	3.97554557
NONHSAG013533	3.53378918	3.50002017	3.11176138	3.17029003	3.00299625	3.40798429	3.29132469	3.29424072	3.24270956
NONHSAG013546	2.78578257	3.45247445	2.98775321	3.17097334	3.12924344	3.2002212	3.2029717	3.40109446	3.19522832
NONHSAG013584	5.09490858	5.12419796	5.5089345	5.09611063	5.17556764	5.36320057	5.34645792	4.98897902	5.31774127
NONHSAG013596	3.69455783	3.94776658	3.39477717	3.72471784	3.38506109	3.39977461	3.46154945	3.7477903	3.52819499
NONHSAG013598	2.51672791	2.50897156	2.44715409	2.94446443	2.72129372	2.65512078	2.6182697	2.71771176	2.49591876
NONHSAG013622	2.34058369	2.55171516	2.5803362	2.37122361	2.20886419	2.9550148	2.44335088	2.54696594	2.62173594
NONHSAG013653	2.6675794	2.42344948	2.40323248	2.24609992	2.25769502	2.4435482	2.68171019	2.6009153	2.09611666
NONHSAG013655	2.9947558	2.91721089	2.86077428	3.03385732	2.83767185	3.15476126	3.21676368	3.29824037	2.90625248
NONHSAG013658	2.51137123	2.70196163	2.49701047	2.35878869	2.39247205	2.58712109	2.67323847	2.53703141	2.50209597
NONHSAG013688	2.86101844	3.4320977	3.09341813	3.26010477	3.04297548	3.47889363	3.48288601	3.5392618	3.01409617
NONHSAG013701	2.42433466	2.10971499	2.23229417	2.23543892	2.33585288	2.54254038	2.28514881	2.28912034	2.17756799
NONHSAG013735	4.19791001	4.01204014	3.92318627	3.94084446	4.02260646	3.96597458	4.14599454	3.96976052	3.75223456
NONHSAG013737	2.71994969	2.11079396	2.33015849	2.48521056	2.33368383	2.2355156	2.47286976	2.29842353	2.46962445
NONHSAG013751	2.84979674	2.5853819	2.29478549	2.53333859	2.52947066	2.79774662	2.47892157	2.53706772	2.44130116
NONHSAG013766	2.27505803	2.25483597	2.13973857	2.23257758	2.10179054	2.25701812	2.07488783	2.20472786	2.28248222
NONHSAG013770	2.20727411	2.22315285	2.28859919	2.1633705	2.19236026	2.23691171	2.26149851	2.14037632	2.18344654
NONHSAG013789	4.62497078	4.92577078	4.73584166	4.67876332	4.54704397	4.68417391	4.84620093	4.78299136	4.53287801
NONHSAG013796	3.30888799	2.88696577	2.99743113	3.05752461	2.83085285	2.7635891	2.95908278	3.17233185	3.22142873
NONHSAG013836	3.54401749	4.0189914	4.02945004	3.48629827	3.55304978	3.52031352	3.75345751	3.73192437	3.30657032
NONHSAG013843	6.42527796	6.35725305	6.73787397	6.42468924	6.39779824	6.29976878	6.52882286	6.28047863	6.47292226
NONHSAG013856	3.34348903	3.366652	3.31716917	3.14033637	3.31207466	3.40330997	3.32242042	3.62371471	3.02103328
NONHSAG013867	2.56993827	2.31429777	2.26069282	2.28950569	2.22008204	2.70787704	2.32569058	2.37725798	2.32475309
NONHSAG013871	2.96886265	2.76824181	2.86150038	2.86564017	2.59635889	2.80477259	2.97236311	2.71791394	2.82318853
NONHSAG013889	2.28085107	2.23268178	2.27942534	2.29691528	2.20981048	2.2908527	2.25402255	2.17623746	2.28514881
NONHSAG013908	2.3654871	2.38027764	2.34363676	2.38612031	2.36024657	2.54090227	2.43471409	2.41197662	2.46149402
NONHSAG013910	2.137793	2.21040598	2.21541597	2.16956727	2.25564908	2.43236655	2.43801392	2.11219494	2.20648623
NONHSAG013921	3.27186567	3.47951301	3.25183344	3.29216164	3.28153891	3.66145913	3.11176199	3.36379819	3.3898178
NONHSAG013938	4.7715126	4.99172151	4.9448843	4.93521139	5.06050293	4.91244186	5.07889823	4.9612254	5.18021145
NONHSAG013940	4.34769678	4.74824156	4.25544694	4.43610529	4.20642576	4.47248606	4.47487215	4.51452865	4.23165704
NONHSAG013950	2.54198623	2.54976026	2.52491416	2.55010152	2.56323643	2.54198623	2.64532446	2.68101841	2.53237025
NONHSAG013963	4.85448956	5.14898353	4.98953516	5.02574687	4.8956569	5.04399941	4.98953516	5.05678067	4.75315638
NONHSAG013971	4.15424028	4.02653829	3.95595494	3.88217283	4.0891586	3.9512462	3.95554394	3.98775458	3.94909172
NONHSAG013976	3.83659207	3.8710821	3.91062136	3.9206119	3.72016786	4.10123753	3.81411122	4.26360466	3.95547248
NONHSAG013982	2.92230322	2.99380295	2.81848803	2.70409233	2.76480833	3.24602668	2.81438763	2.76800319	2.99680485
NONHSAG013992	4.26520946	4.16450996	4.12851092	4.04200557	4.12839587	4.03114297	4.11607899	4.15346626	4.26333292
NONHSAG014007	5.97524841	6.10875952	5.55934686	5.63093272	5.7421934	5.83530235	5.87660215	6.10578245	5.5772698
NONHSAG014012	4.57899751	4.3197143	4.05037937	4.20982835	4.23152841	4.32161597	4.34367534	4.21586779	4.34172196
NONHSAG014023	2.8018262	2.86483645	2.69150349	2.82353732	2.81322342	2.78212154	2.66156429	2.8697776	2.88259968
NONHSAG014030	5.34470006	5.70854928	5.93686963	5.76357244	6.2597352	5.82260593	5.6982597	5.70681809	5.59959015
NONHSAG014035	6.32986422	5.98701599	5.66350955	5.9169638	5.95087888	6.01530788	6.08691667	5.98945353	5.60624345
NONHSAG014073	6.28537865	6.21034929	6.61313489	6.39414887	6.33665202	5.90939583	6.50399593	6.58964087	6.45191061
NONHSAG014079	3.84185101	3.89203906	3.67366123	3.65198715	3.79934827	3.91291729	3.85614007	3.95867246	3.71898575
NONHSAG014090	3.89305013	3.64308824	3.65210871	3.86850037	3.56792268	4.21394639	3.90377225	4.01075947	3.77994528
NONHSAG014099	3.43498234	3.78585004	3.52872015	3.91259479	3.58648273	3.63692468	3.49219756	4.01712225	3.69755417
NONHSAG014114	2.53073914	2.59148559	2.67580493	2.67323847	2.71835109	2.85673184	2.74206878	3.22385982	2.53237025
NONHSAG014130	3.29282901	3.65398878	3.19957857	3.1301839	3.21698792	3.4982018	3.62488084	3.45801645	3.31392575
NONHSAG014136	5.32051528	5.44418992	5.41664407	5.28277757	5.31372042	5.44174566	5.32550897	5.21761551	5.17122435
NONHSAG014165	3.54438213	2.97092425	2.8963361	2.56770854	2.92872083	3.18237795	2.60390928	2.78956428	3.18577766
NONHSAG014169	2.78314166	2.88979664	2.7518779	2.825932	2.74937753	2.79020327	2.7556433	2.89829802	2.8238097
NONHSAG014185	3.12511641	2.99831833	2.93470224	3.02579199	3.1978167	3.29111849	2.98077368	3.17987193	3.05262401

NONHSAG014197	2.72209066	2.81286256	2.71781627	2.44187057	2.38371537	2.75534666	2.67323847	2.89110433	2.6964421
NONHSAG014198	3.87894424	4.43983747	3.97418015	4.21449756	4.00563993	4.21982911	4.32829423	4.39167959	3.97418015
NONHSAG014202	4.08305396	3.8519448	4.3311413	4.40495212	4.29263794	4.39360044	3.99309178	4.11663728	3.99111601
NONHSAG014208	3.15195225	3.93308304	4.08576975	3.90798178	3.47623716	3.68249453	3.74701113	3.68682723	3.4940794
NONHSAG014212	3.11964396	3.10661747	3.20381713	3.1076899	2.89646848	3.38983495	3.13126786	3.09843183	2.94144836
NONHSAG014215	2.23078137	2.16278367	2.21869957	2.24527438	2.3022323	2.24799103	2.19580272	2.13451816	2.39140617
NONHSAG014232	4.55819314	4.19554734	3.75343636	4.14551257	3.7786547	4.55259636	3.72494991	3.98485556	4.26653858
NONHSAG014249	4.61111393	4.50491839	4.31906276	4.25133826	3.8461241	4.49668987	4.06509628	4.626039	4.23955683
NONHSAG014269	4.77143367	4.83944897	4.71557838	4.70768295	4.83731903	4.83666151	4.85736913	4.80187664	4.68194147
NONHSAG014294	4.76547103	4.73322376	4.33933935	4.51281511	4.55819314	4.59350072	4.22582182	4.55946979	4.3812265
NONHSAG014358	4.03018004	3.17699196	3.31670578	3.20968115	3.3759988	3.26931728	3.29972857	3.06793665	3.18564032
NONHSAG014410	2.61662787	2.8694309	2.72434374	2.78645176	2.84855935	2.85808617	3.11176199	3.06606723	2.96477209
NONHSAG014436	2.8225638	2.95146836	2.80847234	2.88607944	2.80282663	2.98716461	2.97250065	2.80033389	2.79016777
NONHSAG014438	3.44618143	3.5036472	3.38027871	3.60199074	3.65042923	3.3737678	3.65973855	3.86796201	3.42218679
NONHSAG014440	2.46230318	2.57984536	2.58244614	2.54405412	2.52161706	2.33874994	2.50478782	2.67218214	2.31577827
NONHSAG014443	2.53549588	2.33114292	2.12280777	2.307089	2.52978624	2.17709346	2.21265901	2.14426273	2.37712929
NONHSAG014445	3.63889997	3.68438552	3.47003267	3.57399147	3.61216196	3.64394312	3.4412499	3.37143251	3.57638845
NONHSAG014448	2.85285897	4.67401485	4.33069049	3.86717328	4.14047368	4.21829818	4.43457917	5.02145365	4.04486918
NONHSAG014454	2.50353022	2.48172233	2.19074045	2.43471409	2.07797714	2.19735993	2.43471409	2.40879724	2.49255441
NONHSAG014569	3.38912135	3.18840959	3.20490794	3.3801103	3.36848488	3.65620727	3.21488255	3.52961969	2.99525739
NONHSAG014574	2.43035534	2.62082283	2.55300482	2.60393926	2.45007513	2.38135245	2.43471409	2.53456667	2.69179033
NONHSAG014590	3.12511641	2.65870894	2.93871928	2.67434853	2.71873004	2.79337579	2.75641903	2.76488286	2.72167275
NONHSAG014611	3.16077487	3.13935301	3.11176199	3.21837572	3.13649629	3.16426421	3.13848705	3.39610246	3.19854072
NONHSAG014661	2.77134213	2.60267447	2.49686523	2.65139339	2.3688486	2.57187196	2.71521495	2.6913481	2.47225149
NONHSAG014663	6.68848024	5.24629732	5.33994147	5.48231385	5.27406079	5.4978507	6.04299222	4.76182927	4.78885667
NONHSAG014667	3.86842868	3.81028257	3.53832445	3.59140038	3.71833354	3.66710984	3.51440705	3.99308532	3.66639708
NONHSAG014746	2.37131369	2.28863211	2.33175757	2.12878207	1.99441703	2.27868176	2.5081571	2.13083599	2.23515313
NONHSAG014747	2.30486885	2.1270049	2.24065649	2.23207647	2.27663474	2.43260013	2.37891268	2.15626231	2.47484672
NONHSAG014790	4.85621383	5.33633366	4.9260031	4.82189762	4.91941204	5.102404	5.03372202	4.97451625	4.63292135
NONHSAG014806	4.16399331	4.65090319	4.32055677	4.01393797	4.32424725	4.58676563	4.27372181	4.31247976	4.21477638
NONHSAG014811	2.12946363	2.30433111	2.35608323	2.21079368	2.27806251	2.44473018	2.55960516	2.37921308	2.23567503
NONHSAG014813	2.90958103	2.71496927	2.50540112	2.45859507	2.40391622	2.90950828	2.48000995	2.76724712	2.77886885
NONHSAG014829	4.30448756	4.37970715	4.04417036	4.22507107	4.2352961	4.39408188	4.08191629	4.17468013	3.89966522
NONHSAG014834	3.91903029	3.87178022	4.14029429	4.06634358	4.28205413	3.85018314	4.0672482	3.95801554	4.08070686
NONHSAG014845	3.71245463	3.74679376	3.49569684	3.72174548	3.68534037	3.65999973	3.9002565	3.84411158	3.74192032
NONHSAG014849	3.30987062	2.82094475	2.87979923	3.2729748	2.94325809	2.8707025	2.83664585	2.93045073	2.89977281
NONHSAG014850	2.54826134	2.28347537	2.0844066	2.31955843	2.34418	2.04969419	2.07802325	2.51534064	2.1526455
NONHSAG014898	4.19646287	3.37899905	3.83164718	3.77831016	3.93103628	3.69085373	3.63091693	3.63869259	3.59665467
NONHSAG014905	4.91356617	5.11486903	4.93393197	4.98815744	5.03177424	4.53075218	4.90468685	4.95705141	4.66323276
NONHSAG014910	5.97014115	5.92293707	6.50816898	5.88597423	6.06151651	5.76792978	6.06314806	6.0575766	6.19872186
NONHSAG014924	5.84131535	6.75187722	6.56174133	6.92720682	6.85893935	6.4982591	6.8887445	6.79187898	6.77359041
NONHSAG014926	4.96110402	4.2183424	5.0459438	4.45220115	4.78730384	4.32990647	4.95236625	4.60323323	4.77156698
NONHSAG014964	2.27095715	2.24265788	2.12385935	2.21007863	2.28580182	2.06882144	2.18607599	2.25061313	2.19085828
NONHSAG015027	6.90568817	7.03468857	7.66133551	7.27053376	7.1602607	6.83127832	7.41895077	7.0133702	7.009636
NONHSAG015035	2.71752202	2.47064877	2.70044964	2.66129283	2.48381684	2.97418571	2.72740046	2.49856388	2.29203115
NONHSAG015052	3.93104934	4.00358009	3.58726272	4.00156797	3.94567142	4.0427415	3.93422457	4.18088725	3.78672237
NONHSAG015063	3.00562416	3.09948447	2.88727299	2.93619232	2.92637485	2.69845762	2.83235009	2.98629712	2.89086976
NONHSAG015092	2.67449215	2.72497442	2.53497369	2.4376434	2.77978044	2.5627667	2.82799354	2.77588146	2.71335822
NONHSAG015143	6.1775879	5.59324776	5.51113825	5.50336223	5.42140858	5.34156674	5.75299465	5.43437335	5.18754223
NONHSAG015144	4.7711932	4.7990587	4.9501836	4.73073989	5.09326461	4.90585219	4.91052557	4.79023179	4.9402748
NONHSAG015185	2.48754936	2.18884371	2.26876985	2.36094356	2.34618684	2.41429454	2.30942349	2.25012874	2.27283389
NONHSAG015188	3.22560275	2.81202114	2.54146294	2.85247011	2.44627427	3.02903365	2.68573352	2.62246191	2.75815869
NONHSAG015211	3.13777728	3.13566856	2.83500463	2.93610793	3.29930372	2.91781328	2.99930866	3.05267231	3.09420255
NONHSAG015231	5.91493389	5.98955047	5.64382947	6.0646022	6.0474711	5.42651095	6.11929257	5.64161091	5.88934383
NONHSAG015256	3.40352311	3.42099938	2.95318576	3.11437682	2.6196853	3.14029083	2.9376721	2.92483272	3.04759412
NONHSAG015273	3.43766879	3.70686644	3.58663266	3.50404734	3.44335675	3.71262487	3.40789391	3.59195781	3.44247812
NONHSAG015286	5.50080863	5.59765074	4.91110349	5.46702741	5.22762337	5.52698097	5.12420691	4.94876514	4.74277995
NONHSAG015320	2.52304025	2.46948889	2.36320671	2.66645096	2.6045185	2.73999686	2.46358989	2.38592892	2.44784535
NONHSAG015338	3.08190179	2.83835501	2.74799186	2.81503029	2.86693116	2.92326324	2.66461931	2.81930566	2.45141449
NONHSAG015357	3.94116119	3.98036443	3.56102055	3.61095817	3.63600594	3.78868477	3.70987205	3.86134393	3.65435004
NONHSAG015371	4.17109119	4.70799969	4.46086268	4.3208652	4.44735824	4.74532723	4.59381835	4.3840186	4.15317037
NONHSAG015373	2.96572457	2.94861785	2.83526406	3.023953	2.85075847	3.09828788	2.56373608	3.12165025	2.58642302
NONHSAG015387	5.84131535	5.98794337	5.44905765	5.67432599	5.68633142	6.03381979	5.66388548	6.12518975	5.4918627
NONHSAG015435	4.04929733	4.0379086	4.18776433	4.11092785	4.05423827	3.95889718	4.13299079	4.07094642	4.0123727
NONHSAG015438	2.54565984	2.41256786	2.65855406	2.42327727	2.58827202	2.61279341	2.3421385	2.57874973	2.56361185
NONHSAG015450	2.92204408	2.77987405	2.92794471	3.06283241	2.99733635	3.01699148	2.85936504	2.66360755	2.99741263
NONHSAG015466	4.71508012	5.09330216	4.64494291	4.77903893	4.54913655	5.08699337	4.8473976	4.70763587	4.63548484
NONHSAG015491	2.96111238	3.46099961	3.76826957	3.53255504	3.41840991	3.46436674	3.63499552	3.8202708	3.35805314
NONHSAG015511	7.34074487	7.6202373	7.79799564	7.67714262	7.6738198	7.35281689	7.72850561	7.73514079	7.62476394
NONHSAG015535	2.29376921	2.1530256	2.5103894	2.21316474	2.30967353	2.39870707	2.32360506	2.26593066	2.27591784
NONHSAG015539	3.26538816	3.48929874	3.1774078	3.82409047	3.58173288	3.30012937	4.16686069	3.82295245	3.47767644

NONHSAG015555	5.6681911	5.47960015	5.92640584	5.6958236	5.69853642	5.6928377	5.81513365	5.73190247	5.55869659
NONHSAG015654	2.91916328	2.93741783	2.94827922	3.0828183	2.96865205	3.12738485	2.90608227	3.11547972	2.7343911
NONHSAG015660	3.75880363	3.67381658	4.03058071	3.81646365	3.71799548	3.47790451	3.60929974	3.49188581	3.75401064
NONHSAG015664	2.76866939	3.56968055	3.06137094	3.05961379	3.18292893	3.20346055	3.3399033	3.15979483	3.38749804
NONHSAG015694	6.33795613	6.46607883	6.34315552	6.43827174	6.28064959	6.28622536	6.49597819	6.63772644	6.17650017
NONHSAG015695	4.27712945	4.80300945	4.39456783	4.55429337	4.28067912	4.60268084	4.45388313	4.70952265	4.20092653
NONHSAG015708	3.54096283	3.95230002	3.59593465	3.5806398	3.79339872	3.70923696	3.57841358	3.70782698	3.55976944
NONHSAG015712	5.39672338	4.48182578	5.13342298	4.67031463	4.88530738	4.44662788	4.90953005	4.10349169	4.68315789
NONHSAG015718	3.20783924	3.43274714	3.27278857	3.31904428	3.28845636	3.39396983	3.35503264	3.43652711	3.63600644
NONHSAG015732	6.42727034	5.31775575	5.28727693	5.36540069	5.26507996	5.19763463	5.47060681	5.03258118	5.19685567
NONHSAG015756	2.84864273	3.18240515	2.96604715	3.10089726	2.84952736	3.18188927	3.08497885	3.04814884	2.94931402
NONHSAG015757	4.92553955	5.26441911	4.70200808	4.9300907	4.76060465	5.38538481	5.00688828	5.19744868	4.51701576
NONHSAG015797	4.07557269	3.71697962	3.26417233	3.78145384	3.66146781	3.70414962	3.89025728	3.72398139	3.5565052
NONHSAG015798	4.07721764	4.29658242	4.11094356	4.29733423	4.50079888	4.23627028	4.15163294	4.10051667	3.9031905
NONHSAG015800	2.57553774	2.18658637	2.07285533	2.18132444	2.31390402	2.22162568	2.18447031	2.26561254	2.67342694
NONHSAG015828	5.60640645	5.53147097	5.37526469	5.37983778	5.24057567	5.32163139	5.43735208	5.26179689	5.26514649
NONHSAG015833	3.21996338	3.1212193	2.8809554	2.96806219	2.74861174	2.94803834	3.23187033	3.14159346	2.74486529
NONHSAG015852	2.59714262	2.87015405	2.66466592	2.58235575	2.31946707	3.10931215	2.74146348	2.59509873	2.22027364
NONHSAG015872	5.71127268	5.11222393	5.5901184	5.48205618	5.4074811	5.40537317	5.40147575	5.38145234	5.09914303
NONHSAG015874	3.17495139	2.93959046	3.11096357	2.9658689	3.24090916	3.11732379	3.20662675	3.01802056	3.18972731
NONHSAG015923	4.12893753	4.00697659	4.02311768	4.0325458	4.11235847	3.93052145	3.99692933	3.9424769	3.911348
NONHSAG015927	2.70051801	2.65239282	2.69331034	2.67409543	2.69693391	2.89943417	2.66392585	2.99339589	2.66558055
NONHSAG015948	4.03294708	4.22227703	3.79678848	3.67274917	3.79704519	4.0456741	3.71483258	4.19311938	3.82539721
NONHSAG015952	3.80511501	4.48327523	4.21274431	4.39966798	4.20204469	4.3761379	4.41669902	4.2313728	4.04919738
NONHSAG015966	5.61542923	5.12456188	5.30074174	5.63775516	5.29886331	5.35544087	5.33921687	5.27810811	5.15972458
NONHSAG015980	6.83131302	6.83449946	6.42591573	6.53494291	6.36106755	6.49808133	6.54484365	6.53129885	6.51723197
NONHSAG015981	5.97746733	6.11181386	6.24632955	6.13883905	6.26028818	6.1053332	6.11959776	6.06168862	6.3485663
NONHSAG016009	3.53251332	3.02624247	3.39326022	3.49888725	3.40042276	2.9806746	3.20419701	2.78165406	3.25089465
NONHSAG016021	4.74131848	4.94325767	4.94591922	5.1234159	4.90668687	5.1945904	5.18575634	5.23420875	4.87672286
NONHSAG016024	3.68886916	3.46209367	3.46530474	3.98775458	3.49204569	3.59389424	3.86090251	3.60913793	3.57761067
NONHSAG016025	4.36927364	4.45814828	4.2816007	4.3258272	4.4937307	4.4711522	4.38167711	4.09847135	4.30366444
NONHSAG016027	7.13255454	7.57725436	7.6032773	7.47237524	7.70002331	7.71980303	7.64718755	7.67507367	7.55094144
NONHSAG016062	3.1700776	3.22516586	3.04367725	3.24880019	3.32227689	3.53368819	3.33346078	3.36412546	3.10969093
NONHSAG016074	8.87152133	9.23895845	9.5144382	9.1274591	8.97493641	8.92044872	9.14494946	9.44128602	8.51346037
NONHSAG016107	2.91493411	2.9386075	2.56502488	2.60458176	2.87607628	2.92986617	2.94939902	2.74519832	2.69354248
NONHSAG016109	4.70218351	4.50457341	4.58787815	4.47023713	4.58042072	4.56896925	4.3497681	4.46145103	4.48094974
NONHSAG016111	5.15479525	5.44187347	5.44286791	5.58253177	5.47958606	5.71231319	5.40623463	5.29036834	5.10749966
NONHSAG016116	2.66128329	2.61011814	2.70942729	2.87261293	2.71025351	2.80959805	2.83874475	2.60052571	2.90303134
NONHSAG016136	2.93964248	2.77583507	3.02708629	2.96370989	2.77036319	2.83691666	2.58597065	3.1424882	3.18982255
NONHSAG016155	2.7348408	3.52330278	3.02713396	3.2711076	3.31456833	3.42186006	3.13065305	3.59419664	3.06318536
NONHSAG016158	3.63214352	3.76367644	3.86192665	3.2829991	3.75372508	3.75828118	3.87501723	3.70421007	4.07654664
NONHSAG016166	2.36826635	2.65203941	2.46668892	2.19002707	2.54701251	2.40440083	2.63174308	2.70584776	2.55292202
NONHSAG016202	2.75735548	3.2440434	3.09982735	3.16904806	3.29381138	3.1909551	3.50968345	3.48711944	2.97145058
NONHSAG016213	2.28592742	2.22591189	2.0791316	2.13796718	2.06106462	2.27289168	2.12231424	2.19052045	2.18776422
NONHSAG016262	3.40554685	3.59219638	3.49369336	3.47965086	3.48770462	3.71796394	3.57800982	3.35003221	3.58150864
NONHSAG016297	2.5695169	2.68183703	2.34636943	2.53642354	2.38694743	2.5695169	2.5324583	2.32964751	2.49572131
NONHSAG016313	3.22131947	3.05710723	2.98368294	3.02717989	3.00782443	3.26514005	3.05975499	3.10223262	3.10146666
NONHSAG016316	2.00228694	2.27797255	2.09073819	2.28655507	2.31970901	2.4426034	2.33728221	2.19876122	2.20340876
NONHSAG016330	3.00595499	2.95489749	2.76659703	2.61935475	3.01294326	3.25786234	2.75453833	2.99538219	3.07166528
NONHSAG016336	5.07227087	5.17666117	4.80738919	4.91360112	4.93996343	4.97488793	4.89309791	5.02301712	4.94941513
NONHSAG016353	2.55927012	2.8129613	2.29484505	2.68332842	2.39187948	2.27482265	2.27256412	2.16205959	2.22654843
NONHSAG016383	4.55819314	4.26426728	4.04059728	4.05329949	3.70557067	4.20089425	4.01376016	3.95007192	3.95807073
NONHSAG016385	3.49984395	3.26699134	2.99732674	2.79761458	3.02684589	3.27477343	3.10319588	3.22005336	2.84625022
NONHSAG016388	5.17786482	5.10917327	4.70867187	4.71245663	4.85947549	4.62403686	5.14062242	4.84245289	4.55825425
NONHSAG016419	5.39091554	6.44127648	6.89424136	6.63478007	6.56034052	6.20202682	6.90128338	6.62275456	6.56569266
NONHSAG016427	5.32681512	5.51520055	5.24681772	5.25456918	5.51646761	5.98244827	5.37718602	5.35993767	5.12383054
NONHSAG016434	3.11936772	3.0058452	3.10294528	2.91789171	2.93382743	3.41121055	3.11176199	2.88920614	2.98819945
NONHSAG016460	3.41938406	3.69620715	3.39255699	3.41805429	3.29846407	3.44765896	3.51773012	3.53631953	3.1694203
NONHSAG016472	5.72672293	6.16070133	5.55426724	5.74875552	5.588063	5.97903732	5.80446385	5.81844754	5.68971653
NONHSAG016517	3.83710271	3.69202498	3.39646097	3.53971903	3.41336418	3.91257081	3.86837041	3.95780259	3.59777233
NONHSAG016520	2.18238162	2.22591189	2.16079436	2.36897243	2.11376483	2.20060806	2.04653179	2.12061371	2.20222706
NONHSAG016540	3.47653737	3.85599448	3.30027496	3.12879609	3.28252366	3.73814798	3.35192088	3.48381696	3.27780424
NONHSAG016542	3.26089314	3.35152412	3.0607931	3.18481011	3.12061095	3.32908088	3.13449373	3.18691903	3.23012475
NONHSAG016544	2.62136983	2.1270049	2.36330604	2.0355106	2.2701301	2.444225	2.31019467	2.13712354	2.13018912
NONHSAG016555	3.9076429	3.62565064	3.35458793	3.49833513	3.3434072	3.26895868	3.28845706	3.62512648	3.31516593
NONHSAG016582	4.34816237	4.27639511	3.97719203	3.90199671	4.19394207	4.48890236	4.20738146	3.85645523	4.07417366
NONHSAG016589	6.6508879	6.97442543	6.92102638	7.05540436	7.03213489	6.80150225	6.92337902	6.88865527	7.21789545
NONHSAG016595	4.40677198	4.77051693	3.66401177	4.1134302	4.05894674	4.48143302	4.17298767	4.60236831	3.96248609
NONHSAG016623	3.9202758	4.65344191	4.09999716	4.22295566	4.13814916	4.26603186	4.3909704	4.31278307	4.09064786
NONHSAG016650	2.70430637	3.22576151	3.24027584	3.13705968	2.84211253	3.33152281	3.0481109	3.10501339	2.94970289
NONHSAG016655	3.35634537	3.45059132	3.070735	3.16141349	3.5740438	3.48031342	3.32731006	3.28544215	3.26410959

NONHSAG016687	3.75134586	4.0267869	3.75974119	3.78816476	3.64528635	3.72400638	3.64003121	3.76849609	3.46313284
NONHSAG016786	6.41790272	5.89979754	6.14146046	6.15003836	5.80790049	6.00881529	5.9735982	5.94574777	6.47949905
NONHSAG016826	3.10539841	2.54640829	2.57977443	2.63452511	2.84855935	2.89992432	2.7471298	2.53078491	2.73591444
NONHSAG016852	3.31416363	2.80172593	2.57795412	2.62137345	2.67635992	2.91027069	2.92124992	2.54631752	3.15506551
NONHSAG016854	2.68039347	2.58764043	2.69080957	2.64414873	2.69275418	2.99988885	2.76485583	2.67166937	2.7221175
NONHSAG016887	3.71986301	3.62271289	3.30027496	3.67714933	3.58916434	3.38604188	3.96786086	3.3802227	3.54842667
NONHSAG016890	2.45861121	2.63773342	2.42554597	2.5717714	2.70057306	2.75542215	2.86792063	2.73971235	2.85562416
NONHSAG016921	3.65988373	3.29817091	3.03909993	2.79331355	3.28900859	3.21433847	3.28196052	2.94374802	3.3853715
NONHSAG016956	5.71747037	5.83659979	5.45630413	5.73184236	5.3283155	5.69919585	5.66010428	5.69114729	5.4570502
NONHSAG016984	2.51632197	2.60395846	2.4609296	2.56347899	2.62990601	2.67234181	2.55510038	2.61310573	2.74840313
NONHSAG016991	6.03489915	6.02405469	6.10818305	6.12867831	6.07890892	6.10818305	6.06412331	6.09622326	5.87530135
NONHSAG017020	3.04052124	2.90569801	2.76449465	2.79178249	2.93487463	2.79550426	3.06845506	2.93933202	2.93811158
NONHSAG017021	3.60596399	3.99572524	3.7856312	3.52469212	3.52046274	3.71648831	3.87195924	4.01662412	3.67429146
NONHSAG017030	4.53168821	4.50871938	4.29142481	4.48358916	4.02543501	4.60409401	4.0954995	4.26035991	3.96806059
NONHSAG017048	2.79792962	2.74571872	2.83104955	2.78244275	2.7012306	3.5247871	2.9440899	3.32322383	2.78613854
NONHSAG017077	5.68031421	6.19572482	5.852861	5.73893907	5.66491242	5.96666852	6.07049488	6.15847402	5.46412017
NONHSAG017083	3.9742693	3.80786754	3.44112331	3.30159399	3.39697142	3.75303773	3.16236091	3.71416961	3.41480646
NONHSAG017088	3.10736417	3.02500249	2.8798857	2.75820549	3.22846038	3.1483827	3.07078258	3.32002339	3.00109112
NONHSAG017091	2.76523373	2.68263926	2.62256638	2.5014215	2.76454512	2.67108295	2.92124992	2.7357523	2.548051
NONHSAG017106	3.60210794	2.96077844	3.15057577	2.98862257	3.04248311	3.13673976	2.76744489	3.04138245	3.12674651
NONHSAG017112	4.57085216	4.64264115	4.30270892	4.58184893	4.37575936	4.52860241	4.44752327	4.7223428	4.07429088
NONHSAG017118	3.75430165	3.5108275	3.5053085	3.65020672	3.57358701	3.60903005	3.42599665	3.63828792	3.51869154
NONHSAG017164	4.68296893	4.62973039	4.73156846	4.20224874	4.55685425	4.19062332	4.39352215	4.48205782	4.33933935
NONHSAG017207	4.6440645	4.44052854	3.76776112	4.39663768	4.22447731	3.779932	4.8106176	4.11699873	3.65482154
NONHSAG017233	3.00610081	3.1378781	2.77770583	2.93245299	3.08659018	3.1966465	2.89736504	2.85420272	2.64178815
NONHSAG017237	7.36856273	5.91816919	5.4128731	5.58720578	5.79467827	5.8915944	6.23404135	6.14802536	5.54664955
NONHSAG017254	4.80414548	4.99258887	4.61286493	4.69973955	4.6236752	4.76409809	4.9434936	4.78302246	4.47223021
NONHSAG017262	2.98864754	3.07629186	3.05357766	2.74908482	2.76910892	3.10288848	2.69351859	2.85357419	3.00936099
NONHSAG017302	3.4594395	3.53824848	3.25946033	3.46984348	3.44488076	3.54748594	3.48594365	3.46865994	3.59196138
NONHSAG017304	2.92580481	3.14747669	3.14072261	3.04697559	3.06947345	2.99463943	3.26210915	3.1563571	2.96467531
NONHSAG017314	3.80867616	3.90208827	3.61091042	3.53667394	3.68993284	3.83286582	3.84432689	4.05808909	3.876138
NONHSAG017331	7.79695198	7.47409474	7.73365019	7.58959336	7.71239864	7.53343538	7.6452287	7.40420568	7.55026941
NONHSAG017346	5.20208668	4.48331439	4.46397609	4.49922215	4.23096176	4.33585356	4.81032008	4.63420086	4.5148188
NONHSAG017352	3.12511641	2.59812445	2.93767924	2.93251977	2.75107084	3.15977555	3.03366351	3.01851971	3.008465
NONHSAG017356	4.64248855	4.12696879	4.05953799	4.28705172	4.1683537	4.29503109	4.33836911	4.05068012	4.09247093
NONHSAG017360	3.92090197	3.83821557	3.9963453	3.99530867	3.80970915	3.92765413	4.01592132	4.25245591	3.92041478
NONHSAG017376	4.6440645	4.90994386	4.82081286	4.57589469	5.07122276	4.68915595	4.50555395	5.09213161	4.90540668
NONHSAG017384	5.73607844	5.43969143	5.47260283	5.37610557	5.67583154	5.28173152	5.80645408	5.4980739	5.34298802
NONHSAG017387	4.8215182	4.6418504	4.28470692	4.31269754	4.20240672	4.57057088	4.21549897	4.42740212	4.38231541
NONHSAG017486	4.48381405	4.43371077	4.19810259	4.85046144	4.42994584	4.65652713	4.45191007	4.51043247	4.70261455
NONHSAG017508	2.46347599	2.54038904	2.57313839	2.4967197	2.47040239	2.37176594	2.39479288	2.45005524	2.45624719
NONHSAG017514	2.62143094	2.41379637	2.50283195	2.37443078	2.71104988	2.41438334	2.55606111	2.45303424	2.51642729
NONHSAG017529	5.36305222	5.50358303	5.03909871	5.17599226	4.88189379	5.20262512	5.09648677	5.29783292	5.201916
NONHSAG017532	3.55335288	3.5269333	3.44352723	3.39318638	3.14964471	3.54507967	3.31699685	3.24291791	3.30790941
NONHSAG017549	3.29510285	3.352731	3.25065312	3.27470292	3.42625243	3.29079326	3.46822554	3.34929801	3.38060941
NONHSAG017579	2.3316978	2.47375771	2.30787728	2.45466834	2.30903573	2.30787728	2.43358453	2.29683231	2.41876552
NONHSAG017593	4.73126249	4.64400376	4.62243657	4.37109512	4.79987726	4.49529525	4.70297475	4.61504282	4.38335207
NONHSAG017594	4.94580798	5.21564921	4.94908663	5.00265802	5.00893262	5.01071038	5.00923944	5.05356601	4.84726452
NONHSAG017604	3.52757888	3.22331151	3.3952974	3.28994335	3.21262707	3.09638948	3.37695963	3.20354911	3.10618068
NONHSAG017613	4.23892128	4.32591716	4.10859118	4.01802047	4.1874391	4.19003206	4.14740868	4.23852144	4.03098189
NONHSAG017640	2.50505814	2.54057403	2.28622722	2.19658461	2.39149037	2.52522426	2.17985654	2.46012737	2.63695014
NONHSAG017669	2.94203254	2.77775421	2.79831528	2.8986405	2.839438	3.07488191	2.97107436	2.82801224	2.7398524
NONHSAG017670	3.66546091	3.55488337	3.46175402	3.41415676	3.28223207	3.93652191	3.57920561	3.67639889	3.25502097
NONHSAG017671	2.87060397	2.81144764	2.71679208	2.62830677	2.75622102	2.88899573	2.75453833	2.88108397	2.56450802
NONHSAG017675	6.13235697	5.98647483	6.33206981	6.09944209	5.95662532	6.01914152	6.05365304	6.07214114	6.16646498
NONHSAG017708	6.14263423	6.64347044	5.68874	5.83728628	5.63073754	6.38785247	5.91056345	6.22181176	5.83853112
NONHSAG017741	4.75964839	4.60216769	4.49506214	4.4212859	4.31393341	4.69084205	4.27683149	4.72773325	4.26856576
NONHSAG017742	4.31968516	4.66822721	4.32416689	4.42823609	4.32331838	4.25864854	4.45534571	4.43186378	4.08113702
NONHSAG017775	2.73938383	2.8879619	2.83285837	3.11461828	2.90424785	2.84617185	3.0585347	2.97758582	2.80946087
NONHSAG017777	3.360144	3.20803296	3.18178494	3.25475481	3.41157178	3.31439253	3.32349169	3.24432609	3.34570788
NONHSAG017785	2.81404933	2.82922693	2.50968889	2.61872505	2.71759455	2.87391715	2.61566072	2.71671115	2.28514881
NONHSAG017797	5.90449656	5.52302295	5.48762661	5.43559916	5.53058962	5.41340245	6.0675279	5.48755148	5.37318915
NONHSAG017837	5.38634793	5.36680532	5.14774966	5.22676317	5.18064266	5.43430584	5.3559224	5.42020333	4.99893653
NONHSAG017855	5.41175394	5.05895138	4.92778352	4.63755285	4.93648906	4.96382127	5.48726606	4.94617348	4.58145091
NONHSAG017868	4.72269847	4.7637732	4.80747326	4.69700634	4.3192999	4.58056674	4.7235747	4.70417873	4.59207104
NONHSAG017869	7.48793652	7.65832022	7.99021979	7.88846105	7.80156601	7.72068935	7.74469066	8.03532288	7.66847517
NONHSAG017885	4.15122851	4.0573931	3.5522053	3.5697219	3.78156335	4.15731118	3.70009093	3.84663615	3.76319641
NONHSAG017934	3.91031161	3.77033148	3.53530091	3.55264671	3.70881054	3.87443218	3.61086932	3.54884554	3.27281056
NONHSAG017937	4.60270976	5.09881396	5.05387795	4.66854417	4.51799623	5.06354682	5.09758679	5.13176134	4.67646761
NONHSAG017938	3.5406404	3.55367896	3.08989445	3.02532136	2.94826788	3.77431194	3.33501977	3.46208738	3.02778881
NONHSAG017946	3.89283817	3.78598654	3.78822824	3.63043738	3.90617113	3.88054819	4.21495411	3.96200491	3.99578114

NONHSAG017952	5.75461079	4.95772918	5.25526735	5.42397842	5.05547269	4.68178414	5.37066191	5.20757176	5.11165892
NONHSAG017967	2.78116364	2.8339964	2.5650203	2.71985114	2.7963462	2.76277341	2.7735285	2.93073746	2.73863723
NONHSAG017971	3.97274455	3.89160016	3.69853022	3.96508783	3.69999813	3.88407167	3.86626444	3.87486121	4.1625873
NONHSAG017972	2.90908418	2.80534028	2.75401934	2.63097079	2.90931197	3.02771275	2.82025083	2.90467925	2.695642
NONHSAG017996	4.02450368	4.04935891	3.84558567	3.76093462	3.95446609	3.98190921	4.03843777	4.19180313	4.06775417
NONHSAG018000	3.02802518	3.39374769	3.02716079	3.44300638	2.99054125	3.51444758	3.19481086	3.14077307	3.10082826
NONHSAG018018	5.1733338	5.30066421	4.95647384	4.93097011	4.62981604	5.06828727	4.67105541	5.05856938	4.68418708
NONHSAG018031	3.09172659	3.23419049	3.24227019	3.05644763	2.97271152	3.51337549	2.99208783	3.55212582	2.95370331
NONHSAG018033	3.14219155	3.45370829	3.12827476	3.15615704	2.93172319	3.40771991	3.27279594	3.37574519	2.82578804
NONHSAG018039	3.36679462	3.46099961	3.4826375	3.5350269	3.64928467	3.60298458	3.67690377	3.46875994	3.33966882
NONHSAG018040	3.14617919	3.14706965	3.25340163	3.02721121	3.00204792	3.13767279	3.08036858	3.34631211	2.91555202
NONHSAG018059	3.62805567	3.88713494	3.71862228	3.62077701	3.7221185	3.62585126	4.1042574	3.96971591	3.78947601
NONHSAG018075	4.69431546	4.33438427	4.43816901	4.62318685	4.40094431	4.43525713	4.59997211	4.41139145	4.4479505
NONHSAG018098	3.12511641	3.80162467	3.73512688	4.30370145	3.39607916	3.73734286	3.75542429	3.9490211	3.57889761
NONHSAG018192	4.84477006	4.99705227	4.92779046	4.71890403	4.82412227	4.74532723	4.92591884	4.76431203	4.73500236
NONHSAG018224	5.9547309	5.81577832	5.48741476	5.52976076	5.592011	5.58451011	5.38022468	5.60261813	5.69867229
NONHSAG018230	5.54743111	5.5875775	5.56899066	5.51014634	5.52939142	5.38586309	5.63330061	5.52609687	5.51217699
NONHSAG018271	4.09426459	3.84518062	3.66363068	3.75864435	3.59090668	3.90731696	3.75797465	3.81893766	3.39040645
NONHSAG018315	11.18060203	10.7508567	10.801639	10.70421604	10.73992132	10.61242305	10.75384432	10.88185244	10.9984213
NONHSAG018332	4.04808185	3.68153955	4.04571888	3.70156891	3.83815674	3.95540465	4.17981664	3.85586799	3.98855417
NONHSAG018336	4.81815237	4.70205035	4.34918997	4.79438394	4.57170874	4.80890691	4.5971773	4.70576747	4.41264719
NONHSAG018350	4.1416154	4.16150472	3.82585981	3.98955517	3.8818035	3.8612335	3.67203088	3.6718056	3.62888453
NONHSAG018377	6.17377107	5.8980596	5.82613797	5.7545835	5.9057574	5.706422	5.83248129	5.93482573	5.74596878
NONHSAG018379	6.40417519	6.34211666	6.02977086	6.13250689	5.98230837	6.19772682	6.03871391	6.0962139	6.15449105
NONHSAG018419	5.04067275	4.62413355	4.77366552	4.66642292	4.88907817	4.68100382	4.64040621	4.71490994	4.96941641
NONHSAG018426	3.13226748	3.22615291	4.25780561	3.71973633	3.89478119	3.38209539	3.73829972	3.82638005	3.26706125
NONHSAG018457	4.71706051	4.67135174	4.53325085	4.64202815	4.56352277	4.89864486	4.64256455	4.61959144	4.59736089
NONHSAG018506	3.82440558	3.74206876	3.76059582	3.70923451	3.8247351	4.08741146	3.79141877	3.94446215	3.89148003
NONHSAG018536	5.1117013	5.33232401	5.83101138	5.63076328	5.47959086	5.46933184	5.11620116	5.4119647	5.43883746
NONHSAG018538	8.34408349	8.46559174	8.84429645	8.64314754	8.71813972	8.56908763	8.48736199	8.47187232	8.5309811
NONHSAG018575	4.13628341	4.13628341	3.70776828	3.85056783	4.06653991	4.33636282	4.26653858	4.0251309	3.98754604
NONHSAG018592	4.93471047	5.07628798	4.55819984	5.02796091	5.02796091	4.88886189	4.91889103	5.0604247	4.73180514
NONHSAG018593	4.75628879	4.99599323	4.80488403	4.9373454	4.8728598	4.96837101	5.02969474	4.74207138	4.56909034
NONHSAG018602	3.70417389	4.4872707	4.02817792	3.92285013	3.63987787	4.36781911	4.02420619	4.05783165	3.7418032
NONHSAG018627	6.14019818	6.72197753	6.83221811	6.86291385	6.38743079	6.55283162	6.60347288	6.76345678	6.66687831
NONHSAG018649	4.90195212	5.12707275	4.74236562	4.58175865	4.26990965	4.89377641	4.92203492	4.62277968	4.68669754
NONHSAG018664	6.16169708	5.46038572	5.51711658	5.73288546	5.51418801	5.39190164	6.36868027	5.55692571	5.26794865
NONHSAG018700	2.90372557	2.71249585	2.68633145	3.06976556	2.57231219	2.9045338	2.88401434	3.05549441	2.69580315
NONHSAG018743	3.6692266	3.82523642	3.7514732	3.6235844	3.77799178	3.73395591	3.83194467	3.69098779	3.75367934
NONHSAG018775	2.39409011	2.29510564	2.37032445	2.26079365	2.51124692	2.45383172	2.22677131	2.33589149	2.31710516
NONHSAG018846	6.57991391	6.18373945	6.01608563	5.98809082	6.10766142	6.0571403	6.31525302	6.02243102	5.94765096
NONHSAG018860	6.0834074	5.9666928	5.98464476	5.8008319	5.69236089	5.71964594	5.92709006	6.011789	6.00498509
NONHSAG018901	3.81478368	3.57674472	3.57012181	3.4940711	3.79142076	3.63673675	4.03757631	3.70849571	3.84772258
NONHSAG018925	4.97555319	5.16316965	4.82165722	4.99633858	5.04739404	5.32423625	5.09862671	5.08792355	5.02528534
NONHSAG018928	3.48700322	3.25793341	3.15607453	3.24354214	3.06282609	3.74042463	2.90441909	3.46188463	3.26035137
NONHSAG018930	4.6117665	4.8710328	4.59754817	4.6468892	4.46003445	4.68100382	4.29085821	5.02640392	4.36899811
NONHSAG018934	6.05600229	5.67706867	5.70651388	5.44128299	5.52694519	5.39372711	5.52646241	5.62817488	5.77421692
NONHSAG018936	3.40275791	2.91102244	3.06002136	2.93991529	3.27940825	3.21307726	3.18036196	3.05611584	3.06926535
NONHSAG019021	3.35762812	3.2999542	3.37684278	3.39961024	3.19125501	3.38870794	3.2191057	3.05339296	3.61769304
NONHSAG019026	3.95908798	3.88597357	3.80712562	3.8181158	3.39274696	4.21377506	3.93413647	3.65465078	3.56486157
NONHSAG019061	4.90071385	4.47421516	4.49963278	4.48125786	4.26435443	4.50979277	4.49867751	4.64924847	4.13712206
NONHSAG019100	5.81272507	5.0734117	5.70391256	5.38078379	5.58876939	5.22663229	5.35465683	5.35862324	5.67000774
NONHSAG019129	5.98734557	5.31106739	5.44349084	5.63370994	5.23803505	5.16531479	5.61907321	5.11549896	5.48729564
NONHSAG019176	6.33834768	6.11895178	6.17460315	6.05997574	5.99066624	5.8980596	5.89803292	6.01981529	6.01981529
NONHSAG019180	2.7840447	2.38851186	2.42359564	2.43377052	2.25545588	2.40066836	2.71586412	2.44559478	2.53421864
NONHSAG019199	2.2171321	2.1035721	2.29535929	2.19619437	2.14656193	2.27136137	2.17638873	2.12199013	2.44735672
NONHSAG019275	3.17454723	3.22513964	3.02265817	3.02287737	2.86520086	2.93807286	2.72762987	3.22650506	2.80488588
NONHSAG019322	2.51611331	2.68389028	2.34636943	2.46474897	2.63719809	2.37730558	2.37018929	2.37484282	2.37730558
NONHSAG019335	3.35112209	2.82907883	2.8329322	3.28859148	3.17180025	3.70327116	3.36628543	3.18516444	3.07827403
NONHSAG019355	4.72224322	4.96541118	4.61955483	4.55632536	4.37012721	4.70485069	4.51903006	4.79224748	4.35767581
NONHSAG019359	3.86793882	3.47986949	3.1901763	3.41426971	3.28563591	3.31439253	3.26134601	3.10997804	3.30602914
NONHSAG019389	2.88621876	2.78427978	2.88899652	2.98808898	2.68924083	3.11747748	2.82070383	2.69481259	2.54987165
NONHSAG019395	2.99484468	3.30762776	3.05178401	3.00500503	3.36199639	3.28093674	3.3151389	3.14541804	3.29324497
NONHSAG019396	3.40681296	3.46940784	3.22243262	3.15111732	3.29681497	3.21670839	3.2811774	3.52321303	3.19594416
NONHSAG019423	3.7484006	3.7400749	3.87028764	3.89484665	3.80319552	4.12090387	3.7230806	3.79587103	3.76608541
NONHSAG019434	3.26976461	3.41282774	3.20587818	3.188684	3.24055791	3.48093483	3.20193807	3.46418672	3.17128076
NONHSAG019460	2.79931688	2.76450264	2.91539795	2.88151295	3.07425971	3.02974731	2.99975422	2.71842992	2.42645366
NONHSAG019464	5.55918582	5.49930811	5.63617206	5.50911814	5.16746262	5.83102935	5.31229425	5.64122584	5.57541885
NONHSAG019471	5.31513999	5.1805143	5.27869348	5.15075746	5.12815765	5.23065916	5.17799341	5.51204287	4.98299573
NONHSAG019477	4.70504406	4.79966503	5.09591237	4.81284214	4.76944081	4.69212225	4.84109255	4.68627424	4.73028606
NONHSAG019496	3.4934395	4.02328581	3.25730621	3.41592918	3.37855681	3.82486492	3.83459568	3.64628008	3.38379528

NONHSAG019516	2.79316661	3.27981468	2.79618724	2.769847	3.01834782	2.82024835	3.10352792	2.98315741	2.79391292
NONHSAG019517	2.38375765	2.42780732	2.5058831	2.34982685	2.37401055	2.50489468	2.58087856	2.54268391	2.44813063
NONHSAG019583	4.6292331	4.39043006	4.06012693	3.92505232	3.92510061	4.20725997	3.85948034	4.37157965	3.89882538
NONHSAG019638	3.2294756	3.00857121	3.30027496	3.16733466	3.22806854	3.16390294	3.21612118	3.1629043	3.34742978
NONHSAG019649	2.38281662	2.2043741	2.20074861	2.21722977	2.00523165	2.26825036	2.34722148	2.09824824	2.49137535
NONHSAG019657	2.64229568	2.4711092	2.49316959	2.43331954	2.69407099	2.57631316	2.47716312	2.54537715	2.43410767
NONHSAG019671	4.01940778	4.05769893	3.59902356	3.68650017	3.52026336	3.95101157	3.67755591	3.8089691	3.57059644
NONHSAG019696	2.80220209	2.44472613	2.76846639	2.67323847	2.56849692	2.57814455	2.72625763	2.75338091	2.57619485
NONHSAG019733	4.91539949	5.060033	4.91341553	4.99294339	4.93526842	4.99368725	5.07166991	5.07379014	4.92330868
NONHSAG019747	4.53272824	4.40364697	3.99088046	3.75171902	3.75377606	3.83997744	4.16383742	4.01435837	4.19421019
NONHSAG019755	3.77547677	3.84636674	3.63402518	3.5318537	3.46883792	3.82112048	3.82486571	3.64675076	3.59664214
NONHSAG019799	2.76723269	2.7192875	2.62349568	2.77574367	2.91207097	2.80870352	2.75695744	2.79677817	2.72736862
NONHSAG019803	5.07063965	4.55257255	4.63668612	4.82413309	4.62128839	4.61670075	4.65321414	4.62230699	4.62051512
NONHSAG019855	8.13264066	8.27289162	8.58154459	8.23420114	8.36089978	8.25931454	8.34833558	8.43443073	8.32917608
NONHSAG019888	3.95570534	4.44857474	4.21482377	4.31797135	4.15911864	4.44913289	4.09513627	4.33571392	4.23102869
NONHSAG019921	3.5131251	3.65530351	3.55482077	3.50638103	3.50895581	3.48414374	3.61195816	3.43538262	3.60656867
NONHSAG019946	5.51827609	6.19452247	6.27860569	6.2623327	6.44803982	6.32194804	6.64213719	6.24301265	6.32697778
NONHSAG019954	2.51801722	2.89240079	2.65534625	2.56876395	2.87627828	2.88157871	2.6821256	2.69481259	2.4818215
NONHSAG020059	4.0047589	3.40512949	3.0159948	2.85330774	3.37463771	2.88679268	3.49959852	2.82305854	3.09164669
NONHSAG020065	3.46640871	3.11832112	3.11207715	2.99553107	3.25183168	3.43252999	3.12279413	3.28114472	3.38275954
NONHSAG020081	9.6148479	8.13442095	8.10604568	8.02977163	7.97029855	7.62222791	8.14313346	8.02195624	8.21293581
NONHSAG020082	3.26919685	3.17608427	3.2051332	3.2893725	3.23845106	3.26028717	3.31841855	3.64830977	3.51927226
NONHSAG020086	5.37393947	5.50610691	5.18181729	5.41818586	5.61357035	5.55153281	5.59005185	5.57886993	5.47272104
NONHSAG020094	2.4915408	2.28085107	2.08454726	2.21302154	2.16431192	2.62277394	2.41710254	2.56815673	2.13508602
NONHSAG020096	4.36968264	3.996503	3.88875953	4.32327285	3.65080045	4.45703803	3.64764093	4.03618054	4.06253701
NONHSAG020099	8.12057458	8.06757188	8.04948837	7.97251461	8.03697231	8.04461016	8.16356842	8.0387137	8.02532701
NONHSAG020107	3.50189092	3.10841272	3.14565431	3.47370344	3.03634493	3.25644303	2.92848166	3.27088835	3.52094663
NONHSAG020130	2.75435416	2.55115226	2.45038319	2.5453476	2.47472705	2.79685497	2.61769417	2.48370909	2.62173594
NONHSAG020182	4.73767843	4.8271331	4.55424548	4.75536625	4.43021565	4.94148384	4.74944817	4.88055619	4.60317162
NONHSAG020190	4.18408472	4.35500292	3.64314552	3.72717165	4.03945891	4.10881436	3.98627091	3.99337418	3.78854058
NONHSAG020197	4.27005084	4.25663691	3.97483404	4.07748828	4.29157815	4.79715552	4.19310738	4.37920179	3.99909104
NONHSAG020211	5.71322452	5.78703012	5.10188086	5.28441404	5.4163408	5.74218316	5.37117657	5.67166886	5.24216509
NONHSAG020225	4.18089576	4.49899139	3.99184866	4.07510171	4.31591177	4.33348957	4.53298323	4.49548784	3.88621643
NONHSAG020230	3.03328295	2.86672238	2.81848176	2.91622303	2.57921646	3.04978174	2.77855661	2.96425998	2.72846603
NONHSAG020240	3.38357999	3.27586558	2.85536735	2.91789171	3.05485627	3.44424914	3.22145836	2.99281059	2.58697803
NONHSAG020323	5.04805399	5.14352575	5.10505086	4.86613132	4.75235567	4.92390817	4.92757546	4.83752484	4.96130285
NONHSAG020329	6.4787736	6.05802568	6.08676236	5.93232541	6.034902	6.26897613	6.10881988	5.96986975	6.32798513
NONHSAG020334	4.24461762	4.22412927	4.05915196	4.21720199	4.28129011	4.29697094	4.33354687	4.10103283	4.07531153
NONHSAG020338	4.35666506	4.58369156	4.38209765	4.27835365	4.47156252	4.53905956	4.10938463	4.12953222	4.46628905
NONHSAG020339	6.89260294	6.91705284	6.80166285	6.8556512	7.02777626	6.96774972	6.70356069	7.08750196	6.99339762
NONHSAG020347	7.56567701	7.10849588	6.98242256	7.10628077	7.08474089	6.79268857	7.35721631	7.03970026	6.9483852
NONHSAG020419	3.87011529	4.14791015	3.85617975	3.87357283	3.86173773	3.76717409	3.96585558	3.86428935	3.9053409
NONHSAG020424	4.03013626	4.320425	3.86957572	4.12954714	4.46099815	3.93678642	3.75760215	4.10517729	4.07102873
NONHSAG020428	2.88046882	3.2476017	2.88677371	3.02861143	3.12444105	3.0278259	3.4245606	3.02479115	3.12067596
NONHSAG020432	6.26202135	6.64038992	5.98533581	6.12835166	6.14312148	6.49434788	6.16228506	6.4285084	5.89610774
NONHSAG020434	3.80749141	3.34297996	3.27670116	3.37513707	3.63832853	3.49682613	3.4289966	3.59177984	3.48001285
NONHSAG020483	2.9330826	3.09580345	3.14528396	3.59466214	2.99914065	3.57240879	3.43516284	3.18448991	2.94194647
NONHSAG020539	5.6355783	5.11680895	4.77384669	4.7326673	5.15202136	5.20933055	5.12420691	4.88607108	4.73460011
NONHSAG020550	6.29286891	5.49218405	5.75402687	5.41303288	5.76992352	5.39837906	5.6899758	5.07805539	5.78034588
NONHSAG020567	4.39728956	4.23141034	3.66488987	3.99396462	4.25059697	4.05831344	4.05544676	4.21644441	3.96388022
NONHSAG020580	4.06817698	4.62482703	4.05273997	4.10860398	4.18580168	3.95455257	4.20934724	4.27944456	4.20558007
NONHSAG020618	5.26833426	4.94303295	4.8827751	4.70405504	5.07550917	5.19317987	4.65749361	4.86373019	5.00847579
NONHSAG020638	7.17076427	7.05528803	7.00810531	6.92083078	7.05678397	7.04500993	7.18621804	7.10507876	7.06570912
NONHSAG020650	2.80471278	2.69211308	2.51828459	2.68848051	2.5789819	2.66096743	2.92659735	2.70721408	2.58835431
NONHSAG020678	6.25658144	6.05072357	5.8886572	5.67432599	5.76417139	5.98011189	5.83345304	6.05954776	5.86463513
NONHSAG020737	3.47233892	3.28153891	3.11176199	2.87317276	2.99014602	3.30583119	3.05177717	3.19978787	3.44023227
NONHSAG020798	2.37368214	2.22152354	2.23820938	2.40550201	2.33257972	2.38802873	2.31252547	2.27720716	2.52117902
NONHSAG020806	4.29464613	4.49384781	3.97834973	4.20258833	4.2456123	4.37714081	4.10591568	4.35215287	4.01871721
NONHSAG020809	3.58778702	3.16126127	3.17015847	3.20041381	3.09549451	3.35338033	2.84246527	3.18093676	3.17743344
NONHSAG020830	4.34596952	4.43120731	4.08894766	4.10413431	3.89684217	4.15133443	4.35257951	4.18759097	4.24369465
NONHSAG020837	2.50453216	2.54739626	2.44586426	2.59486001	2.68144844	2.72384818	2.47055994	2.48588037	2.520064
NONHSAG020855	3.33173654	3.302977	3.18679303	3.00928727	3.00095334	3.12221979	3.07894571	3.42065154	3.28412551
NONHSAG020867	2.21885674	2.05001973	2.15599503	2.15045675	2.09764138	2.32902991	2.16859067	2.10840751	2.14970237
NONHSAG020883	4.25440896	3.94953522	3.90255511	3.95361771	3.63832853	3.77815882	3.96103026	3.55622681	3.84461798
NONHSAG020899	2.7205056	2.84043153	2.84305704	2.71564131	2.9084734	2.89593549	2.78183642	2.746944	2.59011168
NONHSAG020905	3.57365265	2.87805827	2.71271414	2.80040713	2.6200303	3.06136357	2.71533219	2.88004375	2.90581848
NONHSAG020941	7.12137194	5.90091561	5.81007634	6.17950764	6.10621044	5.99029633	6.47597909	6.01140169	5.79874497
NONHSAG020993	5.36348177	5.20530535	4.83719897	4.95924644	4.98973024	4.913895	5.45262495	5.03502217	4.99501499
NONHSAG021003	5.07770495	5.24815808	5.05073323	5.16353082	4.80971848	5.24681772	5.06837604	4.97982937	4.84726452
NONHSAG021015	5.87823837	5.84601859	5.48864677	5.50985278	5.71724057	5.58787344	5.56982434	5.74499336	5.03021383
NONHSAG021045	4.06033294	3.91660221	3.68332923	3.99401298	3.8990498	4.07397658	3.75585034	3.93956793	3.86461476

NONHSAG021143	4.5415427	3.79139999	3.78232843	3.49347779	4.01145999	3.597078	3.64485837	3.70889038	4.07172702
NONHSAG021154	5.04208167	4.81941418	4.41125928	4.92818088	4.31415775	5.07114383	4.92818088	4.88684547	4.34269224
NONHSAG021213	4.65566485	4.68950567	4.83536098	4.20607352	4.9053623	4.28479992	4.55511808	4.57820363	4.81145964
NONHSAG021241	3.0997006	3.14079152	2.62403022	3.11986285	2.83078467	3.32156431	2.63343028	2.69534402	2.90303134
NONHSAG021246	6.10295109	5.16026338	5.39576076	4.93093559	5.24638255	5.08854297	5.07305784	5.19704607	5.59152517
NONHSAG021274	4.10732526	3.92769708	3.63725077	3.824582	3.62757785	3.68031903	3.96182365	4.28092815	3.68843359
NONHSAG021287	5.49627764	5.76864925	5.59131666	5.60682691	5.48140476	5.75632598	5.72020506	5.61655425	5.34333693
NONHSAG021374	2.62172053	2.54448601	2.69014718	2.68458261	2.58572475	2.78718067	2.79119224	2.71006656	2.94661066
NONHSAG021403	4.74783918	5.54063533	5.21548024	5.45545592	5.108968	5.54374097	5.46293193	5.37420031	5.30131111
NONHSAG021436	3.94943862	4.05520508	4.05556289	3.85781853	4.04417036	3.74833109	4.15439398	4.09995564	3.88916088
NONHSAG021444	5.51573189	5.75074388	5.16132741	4.92967819	5.45332745	5.7954341	5.32764167	5.90013249	5.11944901
NONHSAG021459	2.06413328	2.2353862	2.05713487	2.43738666	2.43753781	2.36176932	2.14784429	2.27489962	2.2003487
NONHSAG021464	3.79174065	3.52879803	3.11923997	3.27097684	2.79444012	3.24309118	3.00345145	3.26269531	3.29431073
NONHSAG021479	5.29095252	4.14356855	4.15142548	4.48210527	4.1308303	3.96734124	4.43801265	4.11680279	3.81933714
NONHSAG021502	3.26567157	3.36374273	3.28167044	3.09257028	2.93496228	3.32204531	3.39310931	3.48836521	3.09868229
NONHSAG021504	2.81419713	3.37844159	2.93388968	2.78413883	2.81588075	3.13943664	3.29897689	3.16520383	2.91340298
NONHSAG021505	3.36847888	3.68078259	3.32583984	3.60797015	3.81098156	3.85224027	3.33346279	3.77731533	3.61766684
NONHSAG021537	3.64805713	3.36275062	3.19652598	3.38057729	3.19401568	3.56561008	3.27192897	3.30066254	3.42962257
NONHSAG021546	4.13331145	4.51639461	4.01948185	4.12192779	3.97340784	3.83286582	3.91292507	4.20841134	3.96172939
NONHSAG021557	5.97668692	6.29534699	6.95361378	6.59654408	6.67886782	6.36355833	6.63909187	6.70559494	6.86522598
NONHSAG021577	4.20177152	4.86382098	4.46117375	4.60069395	4.94035497	4.87146989	4.62529875	4.83216251	4.24168567
NONHSAG021582	5.06122028	5.01999534	4.92759383	5.22583648	4.97821306	4.76001311	5.2256985	4.86116764	4.61415278
NONHSAG021608	3.09493291	3.08596066	2.5464325	2.7733267	2.81665296	3.05879241	3.11774483	3.05192375	2.8731195
NONHSAG021662	4.69780174	4.86708688	5.16006604	5.0581295	5.19932183	4.99052338	5.04437704	4.97972195	5.21146233
NONHSAG021671	3.16948655	2.92078864	2.99939822	2.90597236	2.72265264	3.06401458	2.86353801	2.91429141	3.03829225
NONHSAG021696	3.79337062	3.78051999	3.55697169	3.7192929	3.75756422	3.52495812	3.8328545	3.76273374	3.61132663
NONHSAG021701	4.61795051	4.3122341	4.27714543	4.25149704	4.25938254	3.96851099	4.20742103	4.22605626	4.20999789
NONHSAG021755	6.32031852	6.03814143	6.59701045	6.28994504	6.49625957	5.75586426	6.4294704	6.23569187	6.38359995
NONHSAG021758	3.31462388	3.72658809	3.72580523	3.7207527	4.16238926	3.38847787	3.66729199	3.72480582	3.66160172
NONHSAG021801	6.03355278	5.51019852	5.74291945	5.66821298	5.84358171	5.31718837	6.26564922	6.00171817	5.56124368
NONHSAG021825	4.24523846	4.08618171	4.23992328	3.92855338	3.97581874	4.09431267	4.19066413	3.99585094	3.93375987
NONHSAG021851	4.55819314	3.58627946	4.02214928	4.44581454	4.01658578	4.09863156	4.09528669	3.98989788	4.25763072
NONHSAG021881	4.79637336	4.58627474	4.47189038	4.46469144	4.3053739	4.35836308	4.74845967	4.60713195	4.33061134
NONHSAG021904	4.43843805	4.50044739	4.10744056	4.42253363	4.5159866	4.39705351	4.52912948	4.42358066	4.14773201
NONHSAG021905	6.31573415	5.2083574	5.49493033	5.42985133	5.47278875	5.3920825	5.78868687	5.41215812	5.36525722
NONHSAG021933	2.36707284	2.1673365	2.37801871	2.10732941	2.34295648	2.29950729	2.28932803	2.36234597	2.18970718
NONHSAG022026	3.3105366	3.82208682	3.26107622	3.12485568	3.17758824	3.34018311	3.53371329	3.42706478	3.1787384
NONHSAG022055	4.19464623	4.23031407	3.917741	4.09690621	3.86680848	4.34940041	4.19292286	4.22783464	4.07241818
NONHSAG022065	5.4150834	5.00097853	4.87796456	5.08205402	4.96483469	4.89796539	5.59695782	5.40289284	5.24107014
NONHSAG022068	4.4605498	4.70365392	4.07069224	4.41653897	4.56539387	4.58057276	4.38353779	4.61306144	4.16602346
NONHSAG022073	2.7348408	2.50767073	2.39856786	2.66752384	2.66258448	2.71835109	2.6990996	2.63036218	2.65479118
NONHSAG022088	3.66697303	3.76817333	3.48108484	3.64775764	3.65648415	4.02514277	4.03998072	4.00918511	3.34196277
NONHSAG022089	3.22017403	2.85382061	3.04699893	2.96525605	2.87113638	2.65418883	3.09262656	3.01669913	2.95294343
NONHSAG022092	4.66722327	4.79242133	4.43666678	4.68772945	4.7349163	5.19305899	4.62335382	4.78934238	4.70473221
NONHSAG022177	5.01585348	5.069123	5.09919618	5.11891598	5.15903717	5.03033862	5.222714	5.19294915	5.07912525
NONHSAG022214	4.32055212	4.58548669	4.13694661	4.27393438	4.46750103	4.47016821	4.32881124	4.31870024	4.37753056
NONHSAG022224	4.58138348	3.94627694	4.22519132	4.31814506	4.47185836	4.15137735	4.13470669	4.02978558	3.87535373
NONHSAG022252	7.00401615	6.32660779	6.4925611	6.46358095	6.38362633	6.36728253	6.56999933	6.44884188	6.46944654
NONHSAG022256	3.68209908	3.72726828	3.76826957	3.59838572	3.49814502	3.69580796	3.83946165	3.64675076	3.92771077
NONHSAG022261	3.40236092	3.62192558	3.26540396	3.23510482	3.18729532	3.33724127	3.30723209	3.31325628	3.28243794
NONHSAG022297	4.33675794	4.32069378	4.26651986	4.2424104	4.49626914	4.38323032	4.33084755	4.53181961	4.34622331
NONHSAG022308	5.04710365	5.08340039	4.83921978	4.89503104	4.69196298	4.99521794	5.0180007	5.06071304	4.91082519
NONHSAG022311	5.60680121	5.11072605	5.42294891	5.26191977	5.41451082	5.55958044	5.13718264	5.36406368	5.43985708
NONHSAG022328	4.78620978	4.86689164	4.75914263	4.63700255	4.87408706	4.7087223	4.70228296	4.78316778	4.59566976
NONHSAG022337	5.31004831	5.42640895	4.86322112	4.93555915	4.92748649	5.45926256	4.97536083	5.15774892	5.00210262
NONHSAG022342	2.84122589	2.66897726	2.83612557	2.53318197	2.5408468	2.94960792	2.88462194	2.46120246	2.63174373
NONHSAG022353	3.85289612	3.531432	3.26499184	3.52220222	3.26696664	3.35619104	3.37585458	3.55054461	3.44798185
NONHSAG022357	7.79845037	7.36542215	7.48364452	7.39319917	7.43660844	7.01983859	7.50792036	7.44208243	7.42865079
NONHSAG022358	6.80243038	6.532825	6.86957416	6.67172619	6.65255335	6.4738471	7.28085096	6.89935285	6.38364866
NONHSAG022437	5.52604504	5.71604673	5.23382788	5.53433486	5.28303059	5.56695894	5.62167516	5.57421494	5.25928674
NONHSAG022492	7.73770727	7.87875533	8.10991821	8.07264551	8.07749597	7.79027041	8.38305594	8.1421985	8.13722243
NONHSAG022576	4.3957928	3.45099774	3.10332438	3.46560804	3.3596347	3.43285493	3.93613115	2.9483642	3.45174638
NONHSAG022580	4.80035713	5.17980891	5.17856605	5.23523735	5.32900186	5.12255744	5.53252057	5.10478383	5.42294891
NONHSAG022588	7.89239416	7.00871666	7.3056105	7.15580557	7.39689457	7.25014537	7.14577978	7.16189288	7.38415191
NONHSAG022627	4.20369663	3.71697962	3.81028351	3.54530896	3.58488658	3.79873793	3.84432689	3.59777874	3.94323766
NONHSAG022655	3.56929324	3.44752564	3.44757542	3.35051334	3.43924971	3.62383927	3.65713926	3.71509724	3.36449341
NONHSAG022656	3.90606651	3.77127992	3.82095034	4.08582921	3.77676488	3.78833662	3.99260911	3.8029631	3.75391168
NONHSAG022668	4.66243823	4.60089917	4.29074889	4.14004333	4.34821852	4.7541942	4.20437462	4.6421907	4.30419881
NONHSAG022676	3.77152611	4.21003254	3.78384595	3.84223128	3.95655589	3.94200251	3.87269877	4.06767912	3.64109737
NONHSAG022677	4.40164633	3.97636183	4.03128845	3.67530387	3.96574405	3.55449042	4.15886806	3.91269944	3.88210662
NONHSAG022735	5.59743433	5.86096824	6.40535813	5.6811201	5.83724084	5.85843595	5.46651733	5.72136632	5.88936512

NONHSAG022759	8.74785749	7.99784181	8.18451332	7.99283341	8.00610294	7.84033985	8.19292749	8.16848318	8.2090388
NONHSAG022817	4.44495124	4.400003	4.29482059	4.40314914	4.37587283	4.46727928	4.62529875	4.3045626	4.2421921
NONHSAG022830	5.39678509	5.31026457	5.32647967	5.20444612	5.43201647	5.33517418	5.33124364	5.22644049	5.06081234
NONHSAG022841	5.69932104	5.48741888	5.52638818	5.64361503	5.6921117	5.57518853	5.60382655	5.39943235	5.35461162
NONHSAG022852	3.51432077	3.58008417	3.42917091	3.3315509	3.13978181	3.52287721	3.24636722	3.5269508	3.0318285
NONHSAG022858	3.0862083	3.30593537	3.06311181	3.06311181	3.01732694	3.32086112	2.98886739	3.14434582	3.08739143
NONHSAG022867	5.37210924	5.01661109	5.2087508	4.95757108	5.36862249	5.40317946	5.5934085	5.30498441	4.75101402
NONHSAG022906	3.62289553	3.96310499	3.55105611	3.2733276	3.31828028	3.72544545	3.7982413	3.39145496	3.60251919
NONHSAG022924	6.04722689	6.12170431	6.04486851	5.86193061	6.05577464	6.21848374	5.7100741	6.10153051	5.76505049
NONHSAG022925	5.1855639	4.53504449	5.34366007	4.80150903	4.83818583	4.47239687	5.2309468	5.02076573	4.77886909
NONHSAG022957	4.65342415	4.45969475	4.31931845	4.31845508	4.48266995	4.88723036	3.91279536	4.39944834	4.12563687
NONHSAG022968	5.58865326	5.83254228	5.46972403	5.50594852	5.31380557	5.61207755	5.32517367	5.52309562	5.71525108
NONHSAG022969	3.37543263	3.50532664	3.27325823	3.48735515	3.10471691	3.37105568	3.45087453	3.4297958	3.45994153
NONHSAG022994	5.81033817	5.61765347	5.62108082	5.57137817	5.75237361	5.77116291	5.68882072	5.79219623	5.49496453
NONHSAG023009	3.04359343	2.43967411	2.41682991	2.57819524	2.33924867	2.91079475	2.94851108	2.82160981	2.59153846
NONHSAG023036	6.05673475	6.63240881	6.90240463	6.76734541	6.97294744	6.72258096	6.61964192	6.41321121	6.48307777
NONHSAG023052	4.84987829	4.81247628	4.69382483	4.36444452	4.17316119	5.01440403	4.59389482	4.57756723	4.45238847
NONHSAG023067	5.54570242	5.48341868	5.51520055	5.64826	5.59044969	5.54338675	5.62548286	5.90690304	5.63824655
NONHSAG023085	2.7981711	3.02543586	2.99741263	2.97634437	3.0403959	2.87422908	2.7207929	3.01718119	2.59308364
NONHSAG023088	6.01829504	5.79699651	5.49647183	5.34464342	5.62525931	5.91019883	5.54117439	5.63365041	5.30633878
NONHSAG023107	3.62968711	3.95738929	3.47065531	3.30140913	3.58466951	3.8939373	3.80668769	3.87615355	3.23721269
NONHSAG023114	4.05087176	3.75133907	3.4657513	3.17877808	3.35060776	3.30259074	3.01708711	3.19116129	3.18822152
NONHSAG023118	3.93127471	4.14060576	4.0327145	3.52277713	4.04202168	4.40456107	3.76017857	3.99041699	3.58418618
NONHSAG023129	6.23677035	5.63617785	6.15248955	5.64790408	5.52204111	5.78622222	5.5420433	5.64935751	5.68334288
NONHSAG023173	5.46276177	4.55335975	4.68512796	4.89037441	4.72249986	4.54756995	4.93627273	4.63326719	4.81685239
NONHSAG023180	3.21718329	3.23361804	2.9104953	3.32076472	3.33365908	3.66766471	3.4092196	3.26085986	3.12809321
NONHSAG023185	3.27645551	3.55403451	3.05178401	3.46051982	3.13159681	3.64754167	3.43628781	3.5603724	3.32800662
NONHSAG023194	3.20765676	3.12437722	2.55304245	3.26775139	2.97714692	3.29792697	2.92287112	2.47387537	2.75079778
NONHSAG023237	4.15432473	3.8303686	3.41423366	3.60068681	3.67658166	3.88532881	3.57214143	3.84105697	3.5997522
NONHSAG023238	3.20093439	3.00423284	2.85636901	2.92770809	3.00275641	2.97169168	2.95189918	3.05147744	3.10998445
NONHSAG023245	2.85416261	2.43454153	2.53575817	2.50692993	2.54837809	2.38719124	2.56078945	2.62436244	2.36344413
NONHSAG023247	2.25308009	2.12624548	2.192191	2.06246181	2.25481835	2.15929833	2.28397067	2.17476016	2.14574464
NONHSAG023281	2.50812671	2.67074019	2.38159572	2.32718366	2.77987405	2.46441027	2.56793839	2.59434796	2.27800857
NONHSAG023286	2.99484468	3.04392938	3.00900775	3.18625748	3.17875668	3.53530091	2.92039627	3.2316451	3.18972731
NONHSAG023298	3.40358636	3.55160058	3.29773907	3.46755503	3.12584744	3.59817439	3.84432689	3.46099961	3.36590181
NONHSAG023327	4.41522925	4.0267869	3.88803911	4.2369078	4.04830038	4.20808131	4.31376982	4.22561184	3.97158383
NONHSAG023350	3.47496909	3.29538522	3.38155645	3.29594183	3.41636622	3.25624568	3.45942787	3.29512384	3.37620698
NONHSAG023373	3.50586825	4.12419837	3.74354702	3.73104799	3.69428363	3.92604256	3.76017857	4.12306538	3.29340722
NONHSAG023390	5.34882681	5.2437438	5.30413787	4.8987235	5.30696238	5.45449228	5.20123222	5.30024083	5.41416859
NONHSAG023427	2.83380516	2.99316591	2.98350915	2.94642431	2.96564267	2.77960068	2.83724896	2.96522402	2.93206741
NONHSAG023436	6.52197478	6.42717158	6.09311438	6.25114014	6.03415857	6.39152897	6.30565207	6.43416573	5.98081022
NONHSAG023437	3.80086803	3.72322001	3.26123406	3.59086524	3.26243658	3.77197451	3.57871598	3.5966575	3.57426013
NONHSAG023456	2.77573521	3.06444577	2.87639787	3.08582007	2.92703742	2.96938693	2.79512043	2.85357419	2.81623025
NONHSAG023530	2.2321761	2.2255048	2.06970191	2.36246739	2.47297632	2.28085107	2.25785568	2.38073705	2.11136334
NONHSAG023534	2.80509029	2.94493826	2.94768233	2.80420534	3.11197891	3.20016068	3.02582988	3.13029724	3.06594963
NONHSAG023539	5.9215468	5.41265664	5.43012561	5.276248	5.41131061	5.61472778	5.69253022	5.37253166	5.21638677
NONHSAG023582	3.64308864	3.50581977	3.21108789	3.45834937	3.34661335	3.27642772	3.40020739	3.4080458	3.62988715
NONHSAG023627	3.3247879	3.28153891	3.03358761	3.18151046	3.25868752	3.2776625	3.22042953	3.45443238	3.42755509
NONHSAG023639	2.37021298	2.56205549	2.30319433	2.4639578	2.33379422	2.63638003	2.37394696	2.52033711	2.26807216
NONHSAG023666	3.23273646	2.6551546	2.62493806	2.71236947	2.60348153	2.93839324	2.82697156	2.62593888	2.55700403
NONHSAG023691	3.29994865	3.02543586	2.90263462	3.2215096	2.86446038	2.81576703	2.78998432	3.00946876	2.99741263
NONHSAG023704	4.17732474	4.21761709	4.47725804	4.2340048	4.62001509	4.42167353	4.28688761	4.43854711	4.45289476
NONHSAG023707	2.7328401	2.7328401	2.71835109	2.565584	2.57826967	2.70763589	2.60658677	2.6199644	2.52575847
NONHSAG023711	3.07774667	2.92542763	3.11641897	2.99844117	3.01187132	2.91027069	3.14822607	2.81147257	3.0246851
NONHSAG023714	3.87565018	4.19803059	4.05460479	3.96007501	3.80673519	3.90912792	4.09317	3.73741363	3.67201492
NONHSAG023715	2.49539081	2.66263733	2.58920113	2.33487836	2.38694743	2.64015648	2.51091701	2.63912245	2.36438623
NONHSAG023765	2.91163549	3.13792114	2.95609747	3.32391619	2.90810746	2.99091928	3.40057242	3.08549058	3.22433158
NONHSAG023770	2.69609412	2.94930177	2.74459883	2.89652958	2.65156568	2.95893802	2.88946707	3.12387074	2.99741263
NONHSAG023771	2.99484468	2.59212814	2.80152619	2.82587412	2.88101596	2.95465216	2.96063291	2.76168518	3.09178679
NONHSAG023772	2.55791856	2.35035593	2.49106098	2.49473477	2.23795391	2.27396431	2.27557755	2.2209332	2.24236192
NONHSAG023787	5.07703891	5.17752994	5.02207968	4.96239703	5.08891514	5.01927476	5.04282614	5.13090026	5.02480375
NONHSAG023790	2.85604379	2.71888475	2.53437566	2.60550775	2.38694743	2.87103644	2.52386916	2.63314958	2.63847736
NONHSAG023801	4.77198585	5.10125292	4.7242886	4.70415633	4.50553172	4.8339067	4.51176411	4.93068645	4.77760583
NONHSAG023810	3.71167928	3.06393539	3.49734445	3.4880208	3.42480741	3.197984	3.58116581	3.36850347	3.1121882
NONHSAG023839	4.83700852	5.00100803	4.65918763	4.88479213	4.82230618	5.16921755	4.92648564	5.11542758	4.7935632
NONHSAG023915	3.07893362	3.06453021	2.93839603	3.11732157	3.04476377	3.05984164	2.9531225	3.20834919	2.99628876
NONHSAG023920	4.58021712	4.64632737	4.53813758	4.38484508	4.46293196	4.64266851	4.50606882	4.65033982	4.57709543
NONHSAG023923	3.39177961	3.54868035	3.53530091	3.72607664	3.09485102	3.93475496	3.74166045	3.39823142	3.38290028
NONHSAG023954	5.33367721	5.32889672	5.26513233	5.37928161	5.09051054	5.51088728	5.37968898	5.44108926	5.07579127
NONHSAG023966	5.48135456	5.32386594	5.30897138	5.43779217	5.2531402	5.44791296	5.35193295	5.39651887	5.32849591
NONHSAG023972	4.04949558	4.47097763	4.07524456	3.91644376	3.83695865	4.14145232	3.8446454	4.36982038	3.93069497

NONHSAG024013	3.4734825	3.59017334	2.97325563	3.21220591	3.05785248	3.58814717	3.46687693	3.35271749	3.27168853
NONHSAG024040	5.14655747	4.85808773	4.70284459	4.80183741	4.92276477	4.78072313	5.05899118	4.96013356	5.00247837
NONHSAG024043	3.36029054	3.02543586	2.75508657	2.97179945	2.97609232	3.20897842	2.86748599	2.80632385	2.99983487
NONHSAG024046	5.1381351	5.29534136	5.72611925	5.69693219	5.6332548	5.51237437	5.73418721	5.50744528	5.25634082
NONHSAG024080	2.77501549	2.49111043	2.87122001	2.99172245	2.92286645	2.57704897	2.44702399	2.70846293	2.6609903
NONHSAG024104	2.57247014	2.53414522	2.41029701	2.43968411	2.35742776	2.36996424	2.38616146	2.36170166	2.498335
NONHSAG024105	3.69298824	3.34158971	3.3221624	3.28176727	3.03299179	3.34266172	3.18892314	3.3030055	3.17236844
NONHSAG024135	4.87294908	4.52886097	4.26380371	4.34826396	4.36896513	4.4128603	4.32043011	4.35388987	4.31409141
NONHSAG024143	2.88897016	2.54517047	2.70567669	2.5553953	2.37301116	3.30179392	2.46373896	2.88122829	2.48296489
NONHSAG024146	2.75512234	2.99400939	2.87996641	2.88048429	2.85818139	2.80282816	2.75860435	3.08493494	2.75548132
NONHSAG024157	3.6692266	3.63387362	3.44074314	3.32076472	3.40748677	3.75424419	3.63785361	3.57647938	3.29431073
NONHSAG024162	2.61501003	3.03867726	2.57081687	2.59309796	2.57739351	3.33016075	2.68306504	2.84530414	2.64180217
NONHSAG024182	5.01315785	5.3166149	5.2353576	5.25159435	5.14433214	5.30983848	5.28616952	5.39250311	5.22233639
NONHSAG024209	5.74885605	5.49526478	5.85107264	5.69408106	5.58463351	5.98440237	5.8648647	5.41590865	5.69761859
NONHSAG024232	4.51686643	4.35063016	4.47673001	4.5075867	4.45850197	4.4420029	4.33180785	4.46995715	4.30020331
NONHSAG024234	3.24805327	3.70899839	3.58578941	3.55325669	3.82112633	3.76923158	3.71014795	3.60608106	3.56963828
NONHSAG024244	4.45824342	4.43608097	4.40217792	4.42981232	4.06675832	4.20322369	4.36860198	4.24649682	4.04312817
NONHSAG024248	6.25850649	5.66169487	5.84985999	5.91838426	5.77809754	6.0619552	5.95087844	5.81005901	6.00978398
NONHSAG024264	4.18626372	3.94762845	3.91933496	3.96605276	3.74294072	4.2553957	4.00480285	4.08990993	3.76721175
NONHSAG024327	3.00946095	3.99299282	3.3327464	3.08996079	3.13127378	2.80351896	2.82916501	3.64675076	3.36568881
NONHSAG024381	5.46585748	5.79553015	5.66783207	5.6947519	5.75171106	6.17129545	5.77341973	5.68316583	5.4855481
NONHSAG024388	5.22658571	5.72619602	5.27720613	5.0578389	5.36084963	5.44296098	5.53203745	5.59797312	5.19141623
NONHSAG024397	6.23068571	6.03830161	6.24855322	6.26679621	6.32123122	6.06807009	6.09029981	5.9612889	6.16387519
NONHSAG024439	6.2560849	6.24110443	5.85501421	6.00035813	5.8292254	6.25332854	5.57331949	6.04615299	5.55108667
NONHSAG024442	3.61880973	3.70980358	3.59020038	3.92489251	3.78173908	3.97917595	3.52729374	3.7470424	3.83341091
NONHSAG024446	3.28415137	3.55076413	3.40979843	3.51089475	3.49737162	3.56539486	3.52044299	3.82234013	3.31509174
NONHSAG024447	2.51771185	2.71406365	2.83852288	2.88444273	3.02070658	3.05098512	2.72870324	2.84580583	2.86929997
NONHSAG024529	7.40804207	7.43547826	7.58878841	7.46352494	7.30496816	7.25999848	7.76169768	7.84303769	7.30256749
NONHSAG024540	4.24154282	3.91693501	3.5081871	3.93506703	3.58534902	3.88174618	3.63808515	3.60109772	3.87941204
NONHSAG024542	5.39065961	5.1604243	5.10061394	5.1530801	5.26835727	5.15930048	4.94272642	5.29358672	5.12934751
NONHSAG024613	3.79076551	4.29959883	4.53172569	4.30550774	4.34490855	4.37579302	4.59702773	4.40669806	4.31707625
NONHSAG024628	2.67083893	2.44118586	2.67123268	2.62561703	2.91960273	2.4627343	2.77780507	2.43323337	2.70086065
NONHSAG024729	3.31207034	3.15413139	3.47003267	3.23430894	3.41714548	3.66423635	3.74142998	3.62135037	3.34040131
NONHSAG024733	2.37540713	2.27288669	2.11419128	2.12698788	2.28899261	2.42092865	2.19827265	2.33611998	2.17306206
NONHSAG024740	2.99484468	3.06082618	2.67268251	2.49868881	2.56011462	2.93137382	2.83724358	2.96635249	2.62173594
NONHSAG024741	3.35761588	3.38242095	3.39922942	3.34456616	3.33853065	3.47125054	3.48373695	3.45777262	3.20396935
NONHSAG024742	9.06967512	9.02778502	9.21125413	9.23665329	9.21206712	9.0355002	8.9902353	9.1336506	9.05781266
NONHSAG024745	2.59715965	3.11575066	2.88701499	3.34331416	2.91789171	2.86040441	3.02846364	3.24439682	2.93981473
NONHSAG024752	4.65436134	4.54367473	4.16216698	4.49054473	4.59774184	4.36516225	4.46222953	4.57495934	4.26771074
NONHSAG024859	2.71022907	2.38231468	2.57608765	2.48809924	2.44031963	2.62396293	2.64044338	2.56808083	2.34357406
NONHSAG024870	3.11369985	3.05289525	2.75963219	2.74216008	2.68574264	2.76769595	2.72905909	2.71941947	2.61674556
NONHSAG024871	6.04320966	6.15391995	6.20360281	6.39122321	6.01867533	5.96772647	6.35773075	6.1183996	6.05486842
NONHSAG024916	4.94538757	4.80622005	4.26699785	4.45430438	4.38024629	4.60161621	4.55513288	4.61763463	4.47223021
NONHSAG024953	3.75654792	3.89911712	3.82689589	3.73776414	3.63230964	4.14620837	3.9799054	4.03151568	3.79193376
NONHSAG024968	3.81519699	3.88689634	3.44604874	3.59133856	3.75684741	4.02480051	3.78546907	3.97386488	3.72973375
NONHSAG024985	6.69998908	6.87307301	7.32806232	6.57707274	6.74269378	6.86977074	6.87589578	6.70442333	6.78665348
NONHSAG024988	5.55408527	5.86216392	5.75337551	5.5195802	5.46175459	5.75751686	5.66078992	5.98574928	5.55274651
NONHSAG024994	5.43564394	4.82346458	4.76100084	5.02625637	4.95762761	4.49649088	5.38425936	4.92707152	4.832625
NONHSAG025009	5.42825425	5.47474996	5.35639778	5.45687529	5.42170792	5.5919707	5.30550995	5.72878216	5.06731317
NONHSAG025049	5.96975474	5.41725729	5.29779965	5.03368975	5.32002245	5.31957587	5.4168909	5.46709773	5.09377867
NONHSAG025052	7.11637247	6.64748722	6.76642532	6.5865007	6.7674165	6.64208463	7.03382902	6.53848514	6.89239729
NONHSAG025054	7.08653882	6.54870326	6.73153324	6.38930771	6.46036463	6.39106715	6.86404475	6.64168581	6.62246326
NONHSAG025151	5.13573249	5.1122305	5.49223397	5.39970872	5.63213317	5.0985285	5.54931316	5.25113017	5.3078777
NONHSAG025174	4.01804179	4.07479322	3.79470423	4.10487918	4.2476774	3.73021244	4.19546185	4.15052062	3.61425363
NONHSAG025195	6.59871874	6.84004275	7.32141016	7.18451364	7.43046358	7.04353961	7.48247215	7.25086703	7.3417957
NONHSAG025224	4.49754376	5.40861787	5.7260872	5.52017583	5.36929518	5.15141292	6.03479119	5.97455893	6.04278031
NONHSAG025230	3.38395005	3.00907661	2.90243435	2.93458922	2.84069418	3.22838703	3.14565773	2.8121422	3.09630654
NONHSAG025265	2.7348408	3.0887301	3.15370295	3.05953884	2.63758028	2.85935135	2.81772897	2.81937626	3.05770747
NONHSAG025272	4.7788404	5.42939746	4.9550506	4.77816177	5.04391795	5.52703727	4.88429887	4.85152811	4.76757402
NONHSAG025274	2.61501003	2.88463594	2.65303102	2.42635088	2.71835109	2.88351807	2.59595877	2.57192757	2.65773891
NONHSAG025288	2.71141338	3.24944353	2.73386316	2.52713957	2.71835109	2.72963983	2.77590174	3.17875855	2.36435738
NONHSAG025295	4.10164199	4.50783396	4.2450674	4.20551579	4.06809347	4.17036962	4.120722	4.24349003	4.00080019
NONHSAG025343	3.60532107	3.88377498	3.58937698	3.75064841	3.58426562	3.91042964	3.89566981	3.7403174	3.87643873
NONHSAG025349	4.51587004	4.25097488	4.28037198	4.18612556	4.19052741	4.53167903	4.19811987	4.33356296	4.18927065
NONHSAG025352	3.64102904	3.47970414	3.3836651	3.33855248	3.31451082	3.30003456	3.50108347	3.69282882	3.18972731
NONHSAG025367	4.43774975	5.46449257	5.32320959	5.49473956	5.2002621	5.40052244	5.54573831	5.44919515	5.31410701
NONHSAG025376	5.50537492	5.49321534	5.44531217	5.52661406	5.15640218	4.95996373	5.74686456	5.58819133	5.45065503
NONHSAG025378	5.45490315	5.29028702	5.46035057	5.31765271	5.26662355	5.00504225	5.35079986	5.24938639	5.32924117
NONHSAG025393	4.50640317	4.84969655	4.56679959	4.62588598	4.42684797	5.12533889	4.82151741	4.6659149	4.27343334
NONHSAG025422	2.78062262	3.12307285	3.11176199	3.27425461	3.04730346	3.11444597	3.01635329	2.95202681	3.03114464
NONHSAG025431	4.80046402	5.19280635	4.47920979	4.75384036	4.43729358	4.99845342	4.93046898	4.95148969	4.69886758

NONHSAG025438	2.88706448	2.79244271	2.88677371	2.81576248	2.73864046	3.37191155	3.02431729	2.89082004	3.03941428
NONHSAG025448	5.36251601	5.11469711	4.78790161	4.64538974	4.98204461	4.74430153	5.0526304	4.94356384	4.76990718
NONHSAG025466	4.47474855	4.34019217	4.27289312	4.1759419	4.19273664	4.47438033	4.69345343	4.51499548	4.4080267
NONHSAG025491	5.17155132	4.92627242	4.94102466	4.82413309	4.59638341	5.10693339	4.63797046	5.14277295	4.85144489
NONHSAG025494	3.84705263	4.04121846	3.79492984	3.99256589	3.79645444	4.22592533	3.99570262	3.91385675	4.21922596
NONHSAG025500	5.07089027	4.79933354	4.94450828	4.68756312	4.82858925	4.85983233	5.07031694	5.16179055	4.78241122
NONHSAG025531	3.9723563	4.21616733	3.96637527	3.91054771	4.0318415	4.12414636	4.09406263	4.12959721	4.10969788
NONHSAG025536	3.1603443	3.02543586	3.11330135	2.98808898	2.91971678	2.88075491	3.04395094	2.67051802	3.19960198
NONHSAG025542	3.83584514	4.11890804	3.85731674	4.02382187	3.77052351	4.12753097	4.07263516	4.06700549	3.90068205
NONHSAG025649	3.48892883	3.94318841	4.35634082	4.22087691	4.24208383	3.71937427	4.5600988	4.157697	3.92512235
NONHSAG025666	4.65771917	4.66121176	4.44214842	4.49216793	4.44689704	4.54549113	4.51992687	4.71811914	4.46707936
NONHSAG025675	3.35536534	3.48512908	3.27744112	3.37773591	3.61652221	3.35763872	3.30035763	3.41607845	3.263552
NONHSAG025695	4.11151212	3.99830535	4.18571706	4.42278221	4.14538232	4.21419267	3.94813062	3.89602723	3.98475855
NONHSAG025699	2.58845712	2.3003807	2.39712499	2.33892286	2.36351033	2.43395749	2.54999294	2.31932857	2.37894692
NONHSAG025705	4.25396495	4.01231669	4.3675247	4.34381197	4.10206634	4.00315288	4.06585298	4.17585726	4.22113361
NONHSAG025710	2.7432791	2.72041177	2.87590288	2.83390635	2.48174033	2.67069631	2.71035409	3.05538974	2.73613126
NONHSAG025719	4.10415218	4.20578116	4.20616849	4.10084318	4.30432667	4.22629155	4.11236131	4.21114287	4.39555942
NONHSAG025790	5.26196224	4.74790977	5.22822654	5.34259085	5.15871792	5.31503665	5.36472943	5.29931551	5.16711118
NONHSAG025812	2.81637363	2.6873661	2.36850323	2.44938214	2.45007513	2.71835109	2.59595877	2.40558455	2.4793115
NONHSAG025869	4.27359992	4.27294943	3.78166958	3.93628061	4.25799099	4.14775526	4.16870481	4.19269373	3.86589857
NONHSAG025872	3.49795368	3.14351077	3.15415669	3.15834786	3.38613328	3.52892953	3.31873554	3.17930379	3.32866382
NONHSAG025881	2.68893922	2.42173058	2.1997673	2.17240065	2.29538059	2.53016909	2.35847179	2.31463448	2.27279053
NONHSAG025897	5.13002187	5.6881877	5.32667284	5.4381946	5.38139813	5.59384988	5.3796747	5.48565964	5.3491992
NONHSAG025979	2.50524033	2.29978822	2.32936172	2.35695791	2.41920108	2.26206124	2.7234444	2.25908805	2.44192686
NONHSAG025986	3.67591839	3.37946484	2.82645603	3.21550627	3.35164137	3.27182661	3.26098575	3.52632346	3.17846784
NONHSAG026033	2.74716162	2.91202564	2.8683347	2.76551664	2.87386226	2.96110397	2.94085067	2.84692387	2.7181489
NONHSAG026052	3.01613032	3.04644619	3.05178401	3.01254622	3.10673188	2.94931882	3.08354057	2.88731213	3.29958722
NONHSAG026094	4.46410584	4.53667952	4.69052974	4.71382715	4.81343577	4.73712561	4.40515062	4.67034	4.69605544
NONHSAG026202	3.5066163	3.78042456	3.83726467	3.79957713	3.80795827	3.64394312	3.97259489	4.00960808	3.74144244
NONHSAG026216	4.16318665	3.52330278	3.77846011	3.77286754	3.768557	3.93475496	3.50791482	3.75733268	3.61647261
NONHSAG026311	5.08642894	4.54841706	4.67914951	4.6094006	4.62694429	4.43648033	4.68709352	4.5081124	4.5476186
NONHSAG026360	4.3335968	4.2589512	4.14756428	4.06293383	4.50277355	4.10819693	4.46210915	4.01805221	4.04382224
NONHSAG026376	3.12280563	2.59219589	2.51745293	2.3833558	2.50650003	2.48301801	2.54609265	2.42968594	2.48917468
NONHSAG026396	3.34450858	3.58163073	3.68504066	3.72607664	3.53850316	3.29579076	3.60857732	3.49447554	3.71537157
NONHSAG026410	4.18973961	4.40355049	4.22559077	4.08921261	4.03963697	4.16025614	4.34255202	4.26798224	3.94737523
NONHSAG026448	2.35303154	2.27209929	2.29077606	2.07028828	2.28726478	2.19342925	2.35080252	2.18233116	2.15267897
NONHSAG026475	5.0138657	5.15362505	4.94253209	5.17264567	5.14315171	5.10697366	5.35281952	5.12453034	5.027436
NONHSAG026539	2.42919206	2.62161428	2.38547962	2.5681853	2.53765813	2.6018534	2.57340338	2.38232194	2.39250975
NONHSAG026542	2.96690386	3.16554575	2.8025624	2.33104352	2.6507014	2.9016769	2.62071396	2.98794546	2.62173594
NONHSAG026552	6.68756369	6.67380226	6.56391305	6.48768068	6.44696636	6.32421057	6.48332271	6.47401485	6.63854302
NONHSAG026569	5.51226377	5.51297739	5.37736813	5.63101002	5.58821021	5.6413264	5.44353611	5.80462037	5.43634874
NONHSAG026592	4.14947757	4.60754544	4.18038	4.39966798	4.23229461	4.55949028	4.12715957	4.44340424	4.37545791
NONHSAG026607	3.74302974	3.95802414	3.72242795	4.04834995	3.70670317	3.97457829	4.03732075	3.88587668	3.71480553
NONHSAG026618	4.41105819	4.65430266	4.48768529	4.34960017	4.74271954	4.51932626	4.60662571	4.83353766	4.32944344
NONHSAG026620	3.43969745	3.43095215	3.22640467	3.33666069	3.5258337	3.53951434	3.19346625	3.44715359	3.44073034
NONHSAG026634	4.11507842	3.76664601	3.41079955	3.94764005	3.51684862	4.10674195	3.56661155	3.7558441	3.60129161
NONHSAG026661	6.24865517	6.18159559	6.43358953	6.20206935	6.29639683	6.12000304	6.29709892	6.32673129	6.28643717
NONHSAG026676	2.44423049	2.22306379	2.19074045	2.35010857	2.35319912	2.53849767	2.54100639	2.46123914	2.62173594
NONHSAG026716	7.95426376	7.8184746	7.84919986	7.97922255	7.88755782	7.88328349	7.93059682	7.92911509	8.07323805
NONHSAG026725	6.36066449	6.58584476	6.41258531	6.32410329	6.46957735	6.74187588	6.47740978	6.44728686	6.17350123
NONHSAG026729	4.43847867	4.78839082	4.50426454	4.21671241	4.31836994	4.86481541	4.23789373	4.42510314	4.46838215
NONHSAG026751	3.2841757	3.27049297	3.06026613	3.11751071	3.38519857	3.73734286	3.33988357	3.43403737	3.29431073
NONHSAG026771	5.03462842	4.81392422	4.64151481	5.06693676	4.69623019	4.82182585	4.74788567	5.2235385	4.33163041
NONHSAG026775	5.55322076	6.04577552	5.48916263	5.63480663	5.33958999	6.08919023	5.93583169	5.90540581	5.67643481
NONHSAG026798	2.61501003	2.82421959	2.66919913	2.675143	2.71860526	2.7547126	2.64549776	2.88103918	2.75556764
NONHSAG026834	6.27060402	6.26612903	5.75015222	5.87923404	5.49975302	6.47695529	5.74788641	6.02036642	5.65035937
NONHSAG026851	5.58570633	5.98778027	5.95032494	6.11432285	5.95962759	6.00177804	6.1604047	6.02252676	6.04446268
NONHSAG026854	5.06097921	4.76652221	4.94656852	4.76060705	5.09990863	4.87372216	4.81536086	4.4888333	4.98194535
NONHSAG026890	3.0670744	2.95448118	2.92175983	2.98490768	2.92070006	3.05149728	2.93800522	2.95680525	2.78853414
NONHSAG026912	5.6813016	5.72824905	5.17521646	5.39676332	5.30177541	5.59101472	5.18854315	5.49544975	5.23759485
NONHSAG026940	6.45484677	5.87530135	5.61826791	5.66295941	5.93679839	5.54124834	5.97879366	5.80841228	5.82945394
NONHSAG026942	4.90973987	4.67858485	4.25412407	4.4269916	4.39448883	4.94901993	4.33683404	4.65122521	4.01977817
NONHSAG026944	6.27008751	6.49406437	6.59686565	6.24644178	6.58526897	6.76412297	6.50440008	6.42575574	6.42613609
NONHSAG026956	5.5038455	5.50157494	5.21858402	5.41717469	5.25777897	5.66496508	5.88672536	5.31874158	4.96859896
NONHSAG026967	4.62793168	4.98147189	4.41443579	4.54291569	4.50826085	4.7479121	4.65943271	4.80386497	4.58942065
NONHSAG026969	2.53761332	2.98485628	2.59460025	2.60528145	2.76367306	2.71905808	3.03564458	2.86097999	2.6177531
NONHSAG026973	2.85379714	2.8417443	2.61465201	2.64658495	2.5130166	3.09619592	2.64242281	2.75910325	2.55536926
NONHSAG026985	3.79230762	3.70265563	3.78746706	3.69997988	3.75677247	3.77267101	3.61751976	3.76811149	3.69760465
NONHSAG026995	4.80247517	5.38099476	5.28858659	5.36889513	5.06539504	5.44910492	5.39627167	5.20503142	5.17656036
NONHSAG027017	3.28054016	3.85963088	3.35652023	3.2055078	3.40393821	3.57096911	3.37788865	3.57704463	3.30524067
NONHSAG027018	4.04652874	4.78746146	4.14353739	4.44520745	4.62376943	4.68223967	4.34545512	4.44340424	4.26669805

NONHSAG027025	4.87793804	5.10179872	4.80234868	4.71402347	4.91754847	4.98951801	5.26777172	5.11084944	4.72669505
NONHSAG027026	3.7139678	3.7167796	3.3273344	3.55999629	3.49255869	3.64068381	3.68286863	3.78179525	3.45392492
NONHSAG027036	4.5557239	4.70090531	4.05126505	4.15605776	4.27480668	4.6310558	4.22643627	4.49647103	4.19097542
NONHSAG027063	2.60233767	2.53276797	2.61953512	2.7810387	2.50295752	2.71731585	2.58664596	2.45689357	2.44196704
NONHSAG027074	4.1483335	4.04182595	4.14356617	3.94406814	3.90304249	4.655517	3.80392628	4.10162409	3.90041136
NONHSAG027078	3.73762909	3.76080323	3.77186197	3.28955739	3.38561731	3.93265832	3.60950107	3.73863807	3.35797649
NONHSAG027116	2.99484468	2.5014215	2.58064697	2.27212734	2.4951567	2.6259745	2.35099761	2.33745221	2.32182795
NONHSAG027134	3.12123949	2.69269945	2.59314847	2.40201788	2.73683676	2.89538408	2.56596905	2.627141	2.58638501
NONHSAG027135	2.40955133	2.29078975	2.26594366	2.34390817	2.45007513	2.31103059	2.05116877	2.45771098	2.22421561
NONHSAG027140	3.26517257	3.29885452	3.37948406	3.54674383	2.96798399	3.48480576	3.53258446	3.6721162	3.26162324
NONHSAG027170	3.95635452	3.79281549	3.70776828	3.82751852	3.68525668	4.02561033	3.56975118	3.85851441	3.78824357
NONHSAG027173	2.40082301	2.38352805	2.18757836	2.17970739	2.1297614	2.13709875	2.33043725	2.12895288	2.55221832
NONHSAG027186	3.85229486	4.53432811	4.24983424	4.28245938	4.17657804	4.1920766	4.26653858	4.63202783	4.03855084
NONHSAG027205	4.6977406	4.50671534	4.54692558	4.52210283	4.60848982	4.63216749	4.52527496	4.75047026	4.42904506
NONHSAG027215	3.26517257	3.07977134	3.12329235	3.24249589	3.22089099	3.29718515	3.25069466	3.44632493	3.03318113
NONHSAG027327	2.90184249	2.96055105	2.82105775	2.67099457	2.91404678	2.96936777	2.9094886	2.97231892	2.73571223
NONHSAG027345	2.31653936	2.36286489	2.41203525	2.45367588	2.52402687	2.37551389	2.40568046	2.52214022	2.42220579
NONHSAG027346	2.38615096	2.54995584	2.43915241	2.49457398	2.38500962	2.80025604	2.61846244	2.54518368	2.46223317
NONHSAG027354	3.31878405	3.71023336	3.1761167	3.54521495	3.62635008	3.45657315	3.65554334	3.45657315	3.46540775
NONHSAG027362	4.3335601	4.69673839	4.66238517	4.58129637	4.45996676	4.74010947	4.74695332	4.63439248	4.49322421
NONHSAG027390	4.44102312	4.52466271	4.33118961	4.39966798	4.24695565	4.43086685	4.43611436	4.44637532	4.33293599
NONHSAG027419	3.44197001	3.07177592	4.25600525	3.43176732	3.0723784	3.079969	3.48594365	3.21871635	3.42126137
NONHSAG027464	4.18085801	4.05178089	4.03612776	3.98437771	3.87551249	4.06305255	4.18080734	4.03309768	4.0209799
NONHSAG027468	4.60884661	4.52465459	4.48647305	4.44698929	4.73936829	4.58839477	4.3806543	4.2540317	4.42434034
NONHSAG027505	3.44132574	3.52189941	3.51636959	3.41502314	3.53838179	3.50530303	3.40693908	3.54050292	3.618127
NONHSAG027515	2.80433385	3.19309235	3.01195241	3.01113677	2.8493671	3.23716054	3.15591202	3.26085152	2.97252439
NONHSAG027519	6.45514614	6.02975874	6.21488317	6.04659761	6.32200034	6.16718007	6.12725507	5.89508667	6.23438508
NONHSAG027535	4.01096803	3.77650854	4.23604772	4.03744134	4.16216167	4.50810955	3.89919223	3.94558332	4.16399622
NONHSAG027545	2.57275289	2.57887934	2.76016578	2.60503863	2.86394062	2.61541012	2.85653682	2.57147318	2.80959805
NONHSAG027550	2.26893409	2.51435712	2.09569626	2.43471409	2.24001864	2.28085107	2.45887938	2.31704265	2.20648623
NONHSAG027551	2.79151598	2.88071148	3.00855014	2.59132746	2.74320195	2.78351811	2.53097799	2.81918629	2.92639288
NONHSAG027552	3.87850982	3.84810916	3.99542714	4.05088302	3.87413719	3.99542714	4.082692	4.03038533	3.69104943
NONHSAG027560	2.81305834	2.91988932	2.63695076	2.70947729	2.96286985	3.07360655	2.66946444	2.75221943	2.79708024
NONHSAG027603	3.62634353	4.0674186	3.70492827	3.61556717	3.84435536	3.96112832	3.92441525	4.05840505	3.5403806
NONHSAG027626	6.14391921	6.09732541	6.19507553	5.96841478	6.16178756	5.71969239	6.44774476	6.00785554	5.8117692
NONHSAG027634	4.03987647	4.04347862	3.87728257	3.86989462	3.92501568	4.12935102	3.88187586	4.09957502	3.81765558
NONHSAG027639	5.25076584	5.5359224	5.59544531	5.64727151	5.5245429	5.53695881	5.49097795	5.61754869	5.43742821
NONHSAG027647	4.75540028	5.08864093	5.34643098	5.28363053	5.56547404	4.98958229	4.83862728	5.35761876	5.17687267
NONHSAG027670	2.44834372	2.3912957	2.48154346	2.56750267	2.399765	2.4451188	2.35122998	2.57209431	2.56632399
NONHSAG027674	3.27043399	3.28876055	3.01877307	3.12559606	3.20154529	3.32593735	3.15078583	3.55720876	3.07033203
NONHSAG027684	4.32426788	4.24429505	4.33187618	4.52186862	4.29739678	4.28774802	4.21571683	4.28982349	4.55466439
NONHSAG027686	3.6692266	3.60346137	3.68789536	3.56430604	3.49161116	3.52728942	3.60900464	3.90450683	3.5179986
NONHSAG027687	3.30600467	3.42230224	3.5310367	3.02080701	3.37631349	3.31439253	3.31030241	3.46099961	3.13067293
NONHSAG027711	2.99942306	3.16975896	2.82574416	2.85823006	2.94237167	2.8372287	2.67589941	2.67294406	2.63585562
NONHSAG027714	4.83803017	4.74519834	4.56014613	4.40365287	4.44245427	4.39947878	4.59359853	4.4185336	4.22474331
NONHSAG027715	4.92688523	5.21757109	4.74310506	4.73881422	4.57879024	5.42789403	4.72355468	4.82012212	4.64201749
NONHSAG027736	3.26084496	3.54782176	3.32610007	3.29075969	2.98225415	3.1992017	3.40447864	3.28333481	3.19340592
NONHSAG027755	3.91859806	3.40350034	3.44191407	3.95028336	3.35164137	3.8909704	3.35878926	3.36475422	3.31948127
NONHSAG027769	3.37814106	3.26158757	3.61519484	3.61119536	3.51034604	3.64752897	3.80255985	3.44928552	3.39765441
NONHSAG027811	4.08551113	3.70290274	3.63429963	3.61249881	3.29617671	3.82246282	4.07880038	3.33278004	3.33667636
NONHSAG027819	5.11634138	5.19528986	4.92920613	4.72510297	4.54472796	5.32168958	4.9447645	5.27182325	4.6202202
NONHSAG027821	2.44325452	2.20117462	2.12802728	2.34514836	2.09651545	2.36472653	2.2404267	2.40674269	2.08483093
NONHSAG027833	2.81504394	2.73769063	2.74368421	2.68028955	2.72620636	2.86265369	2.83249505	2.80775077	2.76255976
NONHSAG027841	7.00447768	6.0506016	6.3025025	6.48258067	6.50588876	6.20484923	6.57567569	6.50384664	6.22152449
NONHSAG027868	8.85860432	8.59071372	8.60071842	8.58875498	8.52291466	8.5666074	8.76324479	8.49090185	8.5464111
NONHSAG027869	8.39632156	8.19002705	8.56264548	8.18457902	8.19493912	7.99018852	7.99399738	8.05394004	8.181121
NONHSAG027897	3.46248993	3.39363311	3.39153248	3.27050867	3.25856452	3.38654375	3.28973425	3.36643639	3.01064191
NONHSAG027917	3.30833372	3.33221865	3.13828728	3.09548094	2.78219572	2.90704534	2.96957001	3.24037989	3.15262744
NONHSAG027919	4.68191149	4.70651964	4.51780752	4.57632959	4.3072261	4.74643442	4.42937204	4.78536438	3.99674574
NONHSAG027954	4.86075617	5.02779845	4.68948133	4.76729971	4.68975949	4.8747418	4.80641292	4.87735309	4.50428931
NONHSAG027959	3.20577675	3.14155548	3.14066255	3.06860749	3.02621574	3.20483366	3.08527723	3.05236155	3.08325899
NONHSAG027967	3.39773897	3.11509708	3.02525463	2.85406699	2.94374815	3.0697212	3.15724432	3.1321966	2.91454536
NONHSAG027970	5.03361483	5.15056	5.10983257	5.14001449	5.2256019	5.08158329	4.87965806	4.96805036	4.9555909
NONHSAG027998	3.3110498	3.3266242	3.25312229	3.25885272	3.3110011	3.71192235	3.33814429	3.36304708	3.22461296
NONHSAG028005	3.63685255	3.86223257	3.81461109	3.85778042	3.80914154	3.87555974	3.70675481	3.77567043	3.84284889
NONHSAG028023	4.2688109	4.02261409	3.72070578	3.3165919	3.62436926	3.81338166	4.05066157	4.12148773	3.63366371
NONHSAG028024	2.12053619	2.69691083	2.64593968	2.46594607	2.69820574	2.86221228	2.26114513	2.51052385	2.44003673
NONHSAG028072	2.73418891	2.70761944	2.38993948	2.29614595	2.64737333	2.65945501	2.39222201	2.37120092	2.51767642
NONHSAG028118	3.48734399	3.92002448	3.45513745	3.4513076	3.51694125	3.79361245	3.80720371	3.68398468	3.40470422
NONHSAG028155	4.70268484	4.73900785	4.49528221	4.4975481	4.66420859	4.5829873	4.69032241	4.79236272	4.50398611
NONHSAG028160	6.52118911	6.15892544	6.43898604	6.16669029	6.35242849	6.07850625	6.13806918	6.24945822	6.35726802

NONHSAG028184	4.29154351	4.031265	3.76870548	3.72026955	3.64445023	3.97402893	3.58811922	3.8202708	3.55906619
NONHSAG028242	4.33367492	4.42357077	4.57992576	4.27037363	4.55312574	4.4618939	4.74540414	4.79664327	4.73883033
NONHSAG028243	3.66648626	3.8334583	3.80631576	3.85678493	4.05504214	4.04499733	3.82742515	4.012429	3.62799359
NONHSAG028249	2.38701753	2.43720538	2.50124251	2.56517291	2.3941277	2.40146504	2.47874441	2.47209097	2.51376668
NONHSAG028283	3.51729231	3.19416766	2.91176731	3.12378108	2.98623081	2.97592064	3.57884695	2.95634539	2.90303134
NONHSAG028291	4.51776505	4.68437024	4.29122253	4.3506192	4.3058999	4.74532723	4.57082884	4.49320813	4.04945666
NONHSAG028305	2.57773141	2.76068005	2.6708375	2.90702473	2.91983754	3.01508482	2.46537627	3.08980453	2.78252574
NONHSAG028340	3.04955333	3.24883142	2.84217764	2.94054607	3.06405745	3.74609231	2.8911147	3.05359117	3.07752428
NONHSAG028349	3.13171058	3.46207122	3.42838119	3.27550263	3.24063826	3.24018572	3.40181119	3.37329158	3.50853379
NONHSAG028408	2.93646961	2.85104379	2.72204234	2.90910368	2.83602464	3.04309779	3.02845004	2.72408594	2.83874697
NONHSAG028420	2.54844045	2.38027764	2.39304651	2.53644699	2.49780746	2.53237025	2.44912575	2.64683366	2.33354573
NONHSAG028439	4.35772697	4.69928529	4.03845394	4.12473853	4.25639029	4.7119816	4.23641036	4.18234508	3.94372159
NONHSAG028464	2.44431143	2.58771406	2.77994631	2.74380827	2.90020928	2.52355846	2.75860435	2.60457908	2.893281
NONHSAG028465	5.31537666	5.41881101	5.81158414	5.73729211	5.36617133	5.44296837	5.63234582	5.73162278	5.59443173
NONHSAG028467	6.81089062	6.58200503	6.13053987	6.02112066	6.10783813	6.67901812	6.27807446	6.38980273	5.9871945
NONHSAG028469	3.99323367	4.05562652	3.98380086	3.88582182	4.03428403	3.9957897	3.90990653	4.03418687	4.10996862
NONHSAG028525	4.94328565	5.12837306	5.54234502	4.81512785	4.99004161	4.79577059	4.69449246	4.73665135	5.01558882
NONHSAG028576	4.72496496	4.4613192	4.06985405	4.44291556	4.45023579	4.40783472	4.41453521	4.18830376	4.20416392
NONHSAG028585	6.24482581	5.98397023	6.05427649	6.08881384	6.1126767	6.00433535	6.21874723	6.02317726	5.99035224
NONHSAG028593	6.45506166	4.42413346	4.26408794	3.84224999	4.48574783	4.04032804	4.65698525	3.88572317	4.45513004
NONHSAG028595	5.78081041	6.32253171	6.44427163	6.31303005	6.30622389	6.1639694	6.74446443	6.30837492	6.33580599
NONHSAG028611	2.86101844	2.75586334	2.66966931	2.67323847	2.86564597	2.57420778	2.59080586	2.72546009	2.63511255
NONHSAG028627	8.10650722	6.8373452	7.08183314	6.81131857	6.98013847	6.79078424	7.13559576	7.06270326	6.96026973
NONHSAG028726	3.35845033	3.36584688	3.45242179	3.32076472	3.63832853	3.13408665	3.57508081	3.49208539	3.40020739
NONHSAG028734	3.80511501	4.06607904	3.76016526	3.80997159	3.93030501	4.06131501	4.26653858	3.80187928	3.93588916
NONHSAG028743	3.43952219	3.45113996	3.46945723	3.40731127	3.44900857	3.42090732	3.37007441	3.62490994	3.25437743
NONHSAG028794	3.19301727	3.63303659	3.23220582	3.13676033	3.12725052	3.02320003	3.48263962	2.99808853	3.10956855
NONHSAG028929	5.16848252	5.05066267	4.72907717	4.79778695	4.83570175	5.16654887	4.91126928	4.98144757	4.69499723
NONHSAG028931	2.64893222	2.61854522	2.59319588	2.59078762	2.49137341	2.60296403	2.47036554	2.49637379	2.64126648
NONHSAG028939	5.58640333	4.3604711	4.33965372	3.72607664	3.99489351	3.92519162	3.88266008	5.1510319	4.79003483
NONHSAG028975	6.21167081	5.99714469	5.75140714	6.00871418	5.90066676	5.97792049	6.1766594	6.05948451	5.85051243
NONHSAG028980	5.41319931	5.76304071	5.85793492	5.67432599	5.87230509	5.71586482	5.48792359	5.58098991	6.0696447
NONHSAG029002	7.27448252	6.11895178	6.12549297	6.32540283	6.11270014	6.09528496	6.16529066	6.27424006	6.28057933
NONHSAG029008	3.25721842	2.99810075	3.00706535	3.05598469	2.89879548	3.06903441	3.20572221	2.67618244	3.09221246
NONHSAG029029	7.93791221	7.50723378	8.00944123	7.79969551	7.95730471	7.67679341	7.81773314	7.59681494	7.73408614
NONHSAG029047	2.90374729	3.06326395	2.94279571	3.03077849	3.04157264	3.41717959	2.89730448	3.346747	2.89864294
NONHSAG029080	5.17656101	5.45134814	5.09074678	5.00512967	4.77837603	5.44138142	5.15705841	5.18826655	4.87878166
NONHSAG029181	3.2860824	3.37977551	3.17420381	3.11554781	3.33505261	3.31936254	3.332047	3.58814162	3.08281766
NONHSAG029187	5.13605148	4.98916602	5.09041068	5.13041033	4.9518409	4.75127954	5.140749	4.94938625	5.27439142
NONHSAG029193	3.79428003	3.40962703	3.98943672	4.01510564	3.69987025	3.35721769	4.05518882	3.8946545	3.89836368
NONHSAG029202	6.84026739	7.04092291	7.38179879	7.10618689	7.22245669	7.10823995	7.08928707	7.01001637	7.14430354
NONHSAG029206	4.50915099	4.14770045	4.02828701	4.36603762	4.30519437	4.08188822	4.09117903	3.98425081	4.18363757
NONHSAG029225	3.57678607	3.49610032	3.44620792	2.98808898	3.27884767	3.41768445	3.41768445	3.89753196	3.48119074
NONHSAG029338	5.86795282	5.27828132	5.30847838	5.54396699	5.40138141	5.27742092	5.73166492	5.50995442	5.85563634
NONHSAG029343	4.22853998	4.47290789	4.08121917	4.20107754	4.24076832	4.30883976	4.1443395	4.58078617	3.9631912
NONHSAG029351	4.8091723	4.52444525	4.2717202	4.33687291	4.30020137	4.56440869	4.85177385	4.49929354	4.05470117
NONHSAG029390	3.11653894	2.88093276	2.88219893	2.76218512	2.54347403	2.90783576	2.91409342	3.01971078	2.90300982
NONHSAG029407	3.21243585	3.12539514	3.11587196	2.76832625	2.91789171	3.06823253	3.21141178	3.17340125	3.32837801
NONHSAG029451	2.60098412	2.77987405	2.84323549	2.71864991	2.84665411	2.99036853	2.63162404	2.61904786	2.64021538
NONHSAG029458	5.44962774	4.98214891	5.3280128	5.43802821	5.64893511	5.18231644	5.40579388	4.82960893	5.44155019
NONHSAG029466	2.67868837	2.84310659	2.46488365	2.62024515	2.46445442	2.63244807	2.80164357	2.54063792	2.46486327
NONHSAG029469	4.21280256	4.27054986	4.38722409	4.2916956	4.20695419	4.04826561	4.07233595	4.20358981	3.91575857
NONHSAG029483	2.79525758	2.63915548	2.48242121	2.45581354	2.64653665	3.07056019	2.47717193	2.73287581	2.55744884
NONHSAG029505	2.40729209	2.52874885	2.32827234	2.59805678	2.42092226	2.79043175	2.62220512	2.61015978	2.56718642
NONHSAG029519	3.34337505	3.40505753	3.27529019	3.0838306	3.11759573	3.31537033	3.35469954	3.36650868	3.09178679
NONHSAG029550	3.13519645	2.98325067	3.08726034	3.16495008	2.77709127	2.95415278	2.96415786	2.9383414	2.91666413
NONHSAG029563	3.97506636	4.31816532	3.56131779	4.00413304	4.05639367	4.25126134	4.111503	3.80305572	3.81236004
NONHSAG029600	5.8749636	5.81083621	6.04944142	6.16723511	6.09029981	5.80761176	6.0566207	5.86901771	5.9637973
NONHSAG029615	3.16452364	3.07462105	2.89087629	3.07428447	2.91744307	3.16394208	2.76775301	3.20978914	3.07944723
NONHSAG029629	5.71075693	6.01311384	6.03485266	5.81194455	5.59911455	6.0530241	6.082637	6.10747556	6.1234185
NONHSAG029651	2.67492541	3.48574922	3.03645008	3.45282314	3.13075808	3.9028617	2.68637111	3.42684503	3.01787104
NONHSAG029656	3.12511641	2.99317205	2.56757681	2.78071213	2.88952915	3.02505415	3.0476085	2.9228192	2.72323087
NONHSAG029691	4.34467126	4.0267869	3.42754351	4.01465186	3.75757285	3.37410222	3.76009879	3.8202708	4.03630986
NONHSAG029703	3.3240334	3.17363345	3.57713271	3.65632843	3.44006223	3.31834958	3.82078536	3.40598062	3.4704892
NONHSAG029707	3.32722122	2.99777589	2.77546334	2.90868875	2.98339409	3.31439253	3.13354806	3.02156035	3.01644244
NONHSAG029741	2.68722863	2.56222477	2.38133305	2.45752367	2.47173352	2.50877018	2.26870312	2.51480739	2.51368975
NONHSAG029752	2.98025889	3.09176429	3.47173792	3.40354932	3.47433657	3.26853338	3.90289899	3.19520456	3.06607092
NONHSAG029789	2.56549787	2.25805639	2.77921875	2.31463448	2.66028309	2.71835109	2.5081571	2.46537057	2.65592076
NONHSAG029815	2.59956913	2.6776016	2.39538757	2.46991027	2.59874338	2.18664231	2.42513685	2.60020163	2.28158047
NONHSAG029818	2.85306569	2.96868871	2.79175578	2.69667499	2.73326103	2.86708279	2.92182738	2.90317439	2.8016453
NONHSAG029852	5.93439288	5.53519934	5.57508657	5.77430702	5.57878894	5.25035259	6.1386098	5.78505579	5.3915253

NONHSAG029854	6.13249352	5.84639763	5.68951332	5.73819515	5.4363039	5.10201444	5.82838779	5.64612328	5.56768915
NONHSAG029904	3.4383599	3.04481805	2.99286076	2.99664282	2.94424054	3.14157972	3.01988422	2.80032805	2.9784047
NONHSAG029916	2.49997112	2.84883838	2.38455434	2.74960999	2.67210297	2.9639761	2.51646682	2.59678261	2.52814351
NONHSAG029920	3.55672391	3.35132489	3.40754101	3.63501269	3.26410794	3.68047315	3.36896971	3.36342866	3.30529975
NONHSAG029991	2.68779583	2.67783013	2.43217521	2.46321243	2.58357807	2.43910246	2.70961046	2.59933541	2.54741915
NONHSAG029995	3.50348699	3.15745816	3.10380913	3.31758576	3.09805198	3.23156195	3.11143896	2.91039194	2.99741263
NONHSAG030001	2.87633466	2.74925552	2.59364247	2.59361774	2.69077771	2.78686175	2.69292645	2.70141316	2.66858115
NONHSAG030002	2.66667961	2.2090456	2.13425516	2.32231141	2.33368383	2.27981948	2.21940393	2.35039238	2.36963563
NONHSAG030016	3.40681296	3.4805904	3.55570296	3.78953326	3.45451605	3.62948143	3.53538457	3.5507598	3.70867952
NONHSAG030028	2.36044871	2.49803151	2.27769925	2.44720075	2.57526958	2.21471528	2.39407024	2.38332911	2.52804396
NONHSAG030049	2.39030864	2.5759595	2.74210715	2.66468209	2.56326216	2.59086755	2.67666835	2.8040689	2.65542709
NONHSAG030056	3.02759912	2.70951626	2.78712786	2.75330151	2.86965688	2.81745293	2.84958428	3.02636901	2.80959805
NONHSAG030133	2.29558765	2.50091413	2.3382129	2.19984497	2.27291931	2.43079906	2.48840558	2.49646758	2.33944771
NONHSAG030140	2.43992906	2.56149769	2.43230403	2.22581674	2.58827202	2.62173594	2.47591358	2.55251856	2.49498185
NONHSAG030166	3.57635546	3.30054874	3.1346113	3.13994604	3.31769633	3.59943883	3.441019	3.4641865	3.47263719
NONHSAG030168	2.70993115	2.75227546	2.59467883	2.57347349	2.4611838	2.72815759	2.55089737	2.66889529	2.7406872
NONHSAG030187	2.59483669	2.65695666	2.5028226	2.38490351	2.51268025	2.61369409	2.57236313	2.44825186	2.51548146
NONHSAG030263	3.08440349	2.91040979	2.96005946	2.9658376	2.95732729	3.03970328	2.90043379	2.93059752	2.91591542
NONHSAG030278	6.78656004	6.43400442	6.26991553	6.37596665	6.27364983	6.46590672	6.34312033	6.42070134	6.26639947
NONHSAG030325	4.29855031	4.49228282	4.30265883	3.97587965	4.25061565	4.46558154	4.11825437	4.61316859	3.92956371
NONHSAG030326	3.02745945	2.92245905	3.12476205	2.84228357	3.11488171	3.0533822	2.7593914	2.97658177	3.00326029
NONHSAG030351	2.64531849	3.11662334	2.74964715	2.74830981	2.518603	2.85447286	2.75192115	2.89170756	2.70064139
NONHSAG030358	3.07333612	3.39788793	2.96647754	3.1249755	2.83492303	3.07403042	3.04875068	3.13919079	2.93792056
NONHSAG030374	2.7348408	2.69140371	2.41192401	2.37607558	2.34218932	2.590531	2.66912499	2.57006341	2.56289482
NONHSAG030400	4.79310714	4.34191378	4.90353985	4.90355227	4.93698091	4.21285769	4.92473358	4.62747695	4.89203695
NONHSAG030406	4.1450268	5.11680661	4.76661794	4.72506868	4.71678159	4.88159935	4.92059248	4.98987775	4.50358582
NONHSAG030459	3.59780197	3.3439171	3.14516236	3.04396653	3.09841016	3.49296182	3.17318283	3.21151518	3.25974383
NONHSAG030469	5.15536214	5.29429889	5.06900701	5.15045768	5.19908999	5.30812014	5.28555175	5.51204287	5.06806219
NONHSAG030483	3.75231474	4.29017387	3.8674248	4.06375384	4.14229654	4.31301671	4.05974808	4.35563498	3.84012441
NONHSAG030489	3.58143643	3.40988218	3.4003291	3.52628009	3.38701088	3.50332902	3.54695267	3.84487667	3.48462653
NONHSAG030493	2.43499283	2.35417172	2.66876322	2.59739884	2.529536	2.92146635	2.84321945	2.4536076	2.58528089
NONHSAG030495	3.53937928	3.77115317	3.58150392	3.6277857	3.55465196	3.80066889	3.53111305	3.59787451	3.38648749
NONHSAG030509	3.26098584	3.06387519	2.94724992	2.80843848	2.88826806	2.93291322	2.85992481	2.94756238	2.75903851
NONHSAG030613	2.99074503	2.46429286	2.44730753	2.48451348	2.41392923	2.30994578	2.64706677	2.45271571	2.49923864
NONHSAG030629	3.89265183	3.72789244	3.78053162	3.71915966	3.68795599	3.75572461	3.85945506	3.77564556	3.23800394
NONHSAG030633	8.23362422	7.51923872	7.43905644	7.62022485	7.43238502	7.11837047	7.00810208	7.36087297	7.61503547
NONHSAG030634	5.91740972	4.88783424	5.11890948	5.06294144	5.06959696	5.04366034	5.27881112	5.05992714	5.12591461
NONHSAG030637	3.36770983	3.42811218	3.7386054	3.50369091	3.92921595	3.48911832	3.92883318	3.57834578	3.79443188
NONHSAG030676	2.47546345	2.53120926	2.40166544	2.47117459	2.5130166	2.4783765	2.39667697	2.68633338	2.57715682
NONHSAG030681	4.45356386	4.81232826	4.34934237	4.47622396	4.32323045	4.590394	4.58643309	4.53432155	4.26653858
NONHSAG030683	4.36270692	4.01499234	3.76389982	3.94740378	3.70233396	3.71826141	3.76797121	3.78598285	3.7957098
NONHSAG030688	2.47021959	2.48713386	2.38977274	2.43471409	2.21508432	2.43197274	2.20459582	2.4140749	2.26859956
NONHSAG030757	6.15896371	6.8137584	6.33881568	6.32279468	6.45356778	6.66210921	6.4507062	6.58862754	6.16395284
NONHSAG030767	6.60512176	6.63384595	6.31483777	6.5762953	6.40790691	6.80893208	6.4058398	6.57850311	6.45067308
NONHSAG030783	4.13534172	4.38750617	4.51551517	4.5209102	4.7228769	4.38006915	4.62272229	4.54178323	4.82892866
NONHSAG030819	3.37922868	3.34809997	3.14187854	3.25526444	3.20990947	3.58841974	3.24180695	3.39042967	3.4170981
NONHSAG030850	2.12329332	2.10707236	2.03622701	2.29887536	2.10865442	2.08319375	2.11089009	2.30252006	2.32558032
NONHSAG030878	4.0619668	3.92691625	3.78362761	3.89816855	4.05614295	4.15319868	4.72811315	3.83596191	3.79113323
NONHSAG030888	5.47615455	5.38566319	5.02569696	5.18538325	4.89141762	5.42646658	5.19005319	5.12913097	4.97646116
NONHSAG030933	5.20347077	5.97652823	6.20765559	6.21535429	6.55536001	6.17238475	6.11774256	6.10081068	6.41382104
NONHSAG030937	4.05853215	4.36447153	4.02191806	3.75556401	4.04120569	3.9847934	3.90308533	3.78209013	3.9849536
NONHSAG030940	3.48103992	3.31900868	3.40885881	3.76401869	3.06499229	3.10573431	3.10803605	3.22686265	3.22814608
NONHSAG030957	4.21518608	4.22029922	3.7819853	3.83820089	3.90862692	4.16235956	3.826456	4.08733066	3.91735525
NONHSAG030971	4.4991015	4.74214903	4.42117752	4.56454617	4.3839585	4.33761338	4.51530971	4.48013342	4.30398429
NONHSAG030992	5.48155396	5.61173938	5.5104084	5.80314004	5.52466123	5.52144508	5.53750599	5.54415662	5.27697099
NONHSAG031103	4.88388685	3.99927521	4.08912504	3.94205841	4.13357839	4.24143819	4.30089065	3.76955499	4.11718475
NONHSAG031137	3.19141363	3.50223146	3.59515904	3.3320402	3.82026498	3.28328814	3.38199815	3.62999775	3.27720228
NONHSAG031167	4.45842687	4.83412092	5.51013597	4.90148817	5.10181162	4.79804571	4.80821105	4.78092005	5.24255254
NONHSAG031168	5.84199085	5.50188652	5.52037729	5.61478341	5.75343507	5.7962424	5.51638148	5.46331368	5.52037729
NONHSAG031178	3.30083342	3.11153028	3.21638612	3.0391157	2.80709558	3.14125429	3.13676549	3.15948711	3.03193718
NONHSAG031180	6.36376689	6.61911139	6.6654673	6.39962325	6.34896337	6.47872443	6.18124903	6.71040338	6.0659415
NONHSAG031184	3.42128399	3.25462316	3.17461342	3.29672263	2.85647964	3.26723388	3.11525828	3.46641983	2.97092644
NONHSAG031203	3.61384901	4.00803405	3.79014133	3.93648367	3.83191289	4.03059931	3.95498529	3.96233787	3.45356604
NONHSAG031219	2.98230767	2.85985096	2.92770396	2.5014215	2.92713607	2.91027069	2.83459105	2.86416419	2.81519798
NONHSAG031232	4.34944704	4.33337677	3.7971504	3.84130747	3.73709015	4.24825185	3.84456705	4.09756539	4.05070917
NONHSAG031237	3.10282702	3.10111324	2.95803065	2.88786559	2.77790425	3.18743937	2.98536494	2.97532561	2.85138715
NONHSAG031249	3.50103122	3.83072637	3.68954502	3.6562168	3.57338244	3.73734286	3.43452425	3.80813867	3.77331025
NONHSAG031255	2.432281	2.26887629	2.30698609	2.26396749	2.16454693	2.25262576	2.54250156	2.22658455	2.08272577
NONHSAG031276	5.35564712	5.54690574	5.21929893	5.41353854	5.39864556	5.24864696	5.12305449	5.4521808	4.9804703
NONHSAG031296	2.71999256	2.76039393	2.6547091	2.43820489	2.62496911	2.70818394	2.60851367	2.49978756	2.63348318
NONHSAG031312	2.6432576	2.72041177	2.82809103	2.50221626	3.10223084	2.92400967	2.68517887	2.83227365	2.84932174

NONHSAG031314	3.25967449	2.82887943	3.07288327	3.00712787	3.09443748	3.06367166	2.92350225	2.80951607	3.0469172
NONHSAG031315	6.87364243	7.11875649	7.34106701	7.15113515	6.94629235	6.8404109	7.01130663	7.15022135	6.7769926
NONHSAG031322	5.90449656	6.62463656	5.96536105	6.06431061	6.20881342	6.68233689	6.12931751	6.58190374	5.96241964
NONHSAG031335	4.15587463	3.81436867	3.57545658	3.39760443	3.4622589	3.18299709	3.28720116	3.72695902	3.65845738
NONHSAG031372	2.70926496	2.65560688	2.67056225	2.62057943	2.57743972	2.57789856	2.59308357	2.61451609	2.69973181
NONHSAG031377	5.18013063	5.25324407	5.79548203	5.71366557	5.77504155	5.86043948	5.38373491	5.7605371	5.3430307
NONHSAG031385	5.32681512	5.05671118	5.1333126	5.29939384	4.79455772	5.45113567	5.24606468	5.32367674	4.94773553
NONHSAG031401	2.25476998	2.62115548	2.3631346	2.37993482	2.38170645	2.37707904	2.44845014	2.5948538	2.33741935
NONHSAG031461	2.99484468	3.15697877	3.10440071	3.08219085	3.1011406	3.08571822	3.02591184	3.12929104	2.93900845
NONHSAG031476	4.93372213	5.03805019	4.71314148	4.79511097	4.85622577	4.86570343	4.96293602	5.35116446	4.87360927
NONHSAG031485	4.67530451	5.07827439	4.70413641	4.68515663	4.56669225	5.01068215	4.79699809	4.90635797	4.57930452
NONHSAG031489	3.9571918	4.0247365	3.33200192	3.35961732	3.15807268	3.75588021	3.54467528	3.47210623	3.25727445
NONHSAG031516	6.0047082	6.5041925	5.87021397	6.03409665	5.84938235	6.49237114	6.26679621	6.26378278	5.81609499
NONHSAG031596	6.1786848	5.99086094	6.03232054	6.02198744	6.05632943	5.7914265	5.8169337	5.95525563	5.826561
NONHSAG031603	3.24217274	3.08731676	2.8309376	2.98460463	3.01656052	3.20601569	3.14124217	3.14113968	3.1133149
NONHSAG031617	5.9166751	5.86807674	5.92548452	5.93014316	5.86892219	6.03435423	5.98266864	5.85270056	5.9708791
NONHSAG031626	2.28085107	2.39368972	2.01779073	2.13874382	1.93713356	2.41702855	1.95218575	2.15429342	2.13508602
NONHSAG031665	3.04640214	2.54748508	2.55405168	2.74988432	2.80837179	2.65728436	2.72105452	2.72392494	2.42146212
NONHSAG031701	4.29717992	4.23874993	4.17673339	4.07127438	4.25727463	4.07603022	4.04079752	4.11973887	3.88365484
NONHSAG031748	4.99768911	5.19942361	4.34263467	4.49237011	4.45086762	4.72827115	4.66007437	4.51022236	4.4080267
NONHSAG031783	7.12542055	7.06713041	6.77657906	6.7981266	6.79010047	6.81264971	6.92932788	6.97954981	6.88101828
NONHSAG031784	4.56713601	4.9884705	4.49843227	4.5168831	4.75108981	4.71551787	4.39507992	4.61416162	4.58506786
NONHSAG031801	3.80511501	3.99506912	3.95054673	3.92608184	4.04215071	3.76648927	4.08255947	3.98859924	3.97564346
NONHSAG031805	2.62849602	2.72041177	2.45105336	2.69535831	2.64647513	2.71835109	2.36020432	2.52612705	2.93142292
NONHSAG031818	4.96159336	5.38663837	5.24681772	5.32111828	4.65337925	4.76317528	4.681119	5.22644049	5.04801035
NONHSAG031824	6.34770635	6.74533189	7.00812518	6.73315103	6.64834749	6.8386082	6.43808893	6.75063075	6.5385666
NONHSAG031831	4.12632653	4.37988191	4.20418031	4.1831335	4.21447515	4.31174922	4.17038227	4.37402302	4.02213535
NONHSAG031845	5.52317484	5.58998262	5.33456386	5.67923921	5.60065847	5.54015584	5.72744807	5.87052264	5.74596878
NONHSAG031890	2.99484468	3.37390539	3.19983638	3.41490606	3.33546347	3.33854076	3.26435763	3.4659895	3.13749904
NONHSAG031913	5.15363451	5.20734219	5.1197263	4.98314596	4.93339266	5.13115366	5.07513703	5.23763567	4.99835468
NONHSAG031945	5.42368977	4.57453013	4.90407414	4.86851308	4.51968842	5.15669571	4.5234853	4.62451032	4.85418369
NONHSAG031951	4.31046374	4.87014046	4.6879082	4.4862803	4.55083312	4.80077964	4.65889821	4.73746678	4.44386545
NONHSAG031952	4.16805752	4.28057614	4.42234912	4.31877496	4.53469607	4.35852399	4.08181896	4.33723693	4.33943761
NONHSAG031964	4.31084878	4.52755222	4.32773943	4.26139982	4.10206634	4.37814545	4.30817114	4.30817114	4.1512051
NONHSAG032009	3.37924721	3.68389415	3.51888021	3.46656365	3.45574854	3.8525074	3.53869091	3.71194966	3.38862813
NONHSAG032015	5.25415931	5.32676397	4.9707682	5.16982503	5.33652466	5.38345786	5.45460825	5.19792651	4.99976502
NONHSAG032104	5.1658099	5.13646365	5.02205541	5.10215663	5.28882754	4.95472453	4.96379593	5.0009385	5.2345468
NONHSAG032106	4.14282097	4.5363614	4.00815349	4.20305313	3.92385015	4.37546337	4.46291448	4.41475201	4.11810077
NONHSAG032135	5.65370272	5.23203962	5.44060975	5.56706241	5.3649787	5.16404481	5.59197802	5.21832168	5.46589483
NONHSAG032143	2.55947972	2.1270049	2.44910288	2.50994205	2.45966002	2.43854575	2.35331309	2.45436352	2.27839435
NONHSAG032153	4.64706764	4.61266208	4.09068015	4.30765227	4.12145854	4.50474147	4.07975068	4.40509706	4.3013872
NONHSAG032186	3.52015255	3.98239785	3.66160728	3.66415855	3.67429725	3.61671246	3.9850988	3.68155172	3.68243199
NONHSAG032194	2.51272562	2.55112148	2.45576814	2.45596563	2.47688664	2.53628947	2.45335079	2.31463448	2.38337802
NONHSAG032197	4.21158699	3.88050898	3.95255768	4.14458132	3.94978152	4.18910613	4.3812578	3.93971685	3.96426174
NONHSAG032235	4.53069496	4.94785816	4.59546232	4.31487806	4.57515759	4.89153215	4.68820828	4.65692853	4.38700533
NONHSAG032245	3.45935769	3.86541475	3.69795352	3.9314948	4.04417036	3.96976795	3.84432689	3.8202708	3.62827132
NONHSAG032250	4.03411542	4.26212286	4.00486539	4.03099556	3.99761722	4.55030145	3.90371427	4.21818109	4.16171188
NONHSAG032270	4.80741155	4.6885406	4.83474945	4.71107645	4.66064944	4.88397511	4.90898194	4.73044993	4.65577517
NONHSAG032285	3.32993766	3.77043026	3.65928255	3.86407666	3.71292513	3.91682823	3.76855644	3.93886736	3.68728774
NONHSAG032299	4.69800252	4.78277572	4.67102762	4.57568151	4.63667852	4.64495135	4.50653505	4.7618571	4.28215603
NONHSAG032337	7.54614505	7.80858881	7.87682704	8.36807213	7.59201723	7.99829409	7.42766636	7.638215	7.32852997
NONHSAG032387	2.922718	2.61012928	2.48558993	2.51196294	2.77552845	2.63154465	2.45944301	2.40525241	2.53237025
NONHSAG032422	5.27489121	4.6801675	4.67344602	4.7914691	4.89586403	4.33375982	4.94779849	4.49091447	4.20714709
NONHSAG032427	3.20779412	2.53106145	2.66119538	2.72526786	2.52801898	3.36563486	2.84939784	2.5999097	2.54194619
NONHSAG032431	5.37393947	4.83204801	4.6319183	4.86906127	4.84436913	5.12053553	4.81280856	5.15722879	4.74147444
NONHSAG032480	3.77766472	3.96650893	3.72845472	3.88435274	3.72204789	4.25458863	3.94445985	3.97576935	3.86622268
NONHSAG032559	3.57609416	3.08267098	2.93351188	2.95732505	2.96669416	3.39084753	2.92988865	3.02153775	3.11361973
NONHSAG032592	2.64084	2.53101116	2.31170538	2.23955282	2.43821728	2.6586954	2.19120379	2.53726473	2.47353352
NONHSAG032593	4.3925824	4.50735979	4.03004443	4.30342079	4.01374906	4.635418	4.13283718	4.60108684	4.14975543
NONHSAG032601	3.38064232	3.34909035	3.4025234	3.23211448	3.0447776	3.2485572	3.32274721	3.17588035	3.27655237
NONHSAG032608	6.50725729	6.71568575	6.3410839	6.34969324	6.25440828	6.68916886	6.44508556	6.49407603	6.26510293
NONHSAG032613	3.89077615	3.84731759	4.02105353	3.72892521	3.75687735	3.79834599	4.19803368	4.13536178	3.60332222
NONHSAG032620	2.6776906	2.51760086	2.73462993	2.7632766	3.50132923	3.00607347	2.67013162	2.62659394	3.08305332
NONHSAG032629	2.55928104	2.45703873	2.357828	2.36870792	2.43514161	2.45925457	2.62849454	2.4240231	2.27996354
NONHSAG032632	3.8043204	3.82842593	3.82023311	3.70814596	3.73562349	3.45903122	3.77452424	3.78312132	3.5818773
NONHSAG032643	4.41059791	5.38989405	4.98039209	5.19073775	5.34649124	5.09971728	5.20210077	5.33596068	5.24372725
NONHSAG032649	3.30752529	3.07914997	3.28074495	3.19369628	3.28089865	3.02407826	3.271598	2.99050512	3.08831066
NONHSAG032662	3.18452599	2.90474628	3.14310711	2.92235274	2.94780054	2.83991576	3.10357575	2.91371817	3.05110935
NONHSAG032723	2.93554896	3.19986836	3.0156979	2.99416755	2.902405	3.08618539	3.13154003	2.89699181	2.95758375
NONHSAG032740	4.58642439	4.66722528	4.33933935	3.93842749	4.25945254	4.6103428	4.22661846	4.42021854	4.13442888
NONHSAG032746	6.75011732	5.7051778	6.05788518	5.84868176	6.10971728	5.69843663	6.89938694	6.2976764	6.07993921

NONHSAG032750	3.28470653	3.13724249	3.27713642	3.08897209	3.20910721	3.04361057	3.36228267	3.12708414	3.12912961
NONHSAG032785	5.55157654	5.53931212	5.17198401	5.25021821	5.2069251	5.39637644	5.1212245	5.46511702	4.99890734
NONHSAG032791	4.11833288	4.042055	3.67520968	3.62540509	3.75814216	3.44224102	4.02420619	3.73837615	3.68265364
NONHSAG032815	2.50914676	2.34300538	2.13281102	2.06406125	2.22610439	2.28382479	2.28514881	2.17758663	2.05723597
NONHSAG032817	3.46721081	3.43573705	3.4185038	3.32076472	3.20894497	3.39269412	3.43953275	3.63364197	3.6569156
NONHSAG032839	2.87857773	3.21309911	3.11320671	2.89695947	3.06862576	3.39572438	2.76550674	2.91082458	2.86342583
NONHSAG032855	4.39591678	4.91702119	4.56337819	4.72512032	4.63239139	4.87718933	4.73018559	4.9485729	4.40660474
NONHSAG032935	7.08607097	4.91633988	4.78985201	4.81656506	4.30532231	4.62605811	4.01819545	4.23657492	4.52638692
NONHSAG032949	4.76703651	4.82801147	4.62784923	4.48644781	4.57512332	5.04626891	4.42540343	4.51437818	4.26637878
NONHSAG032951	3.7305486	3.48570924	3.04409359	3.09046888	2.91966305	3.20710522	3.11176199	3.17876065	3.29292008
NONHSAG032956	6.26866861	6.38255969	7.08011151	6.39055764	6.79058184	6.44184612	7.31988509	6.91079583	7.01069104
NONHSAG032959	3.86379182	3.9861633	3.74082779	3.58476845	3.76722845	3.83138332	3.63081606	4.04148993	3.80467302
NONHSAG032960	4.09501177	3.90240851	3.92081796	3.8256298	3.94916474	4.08817393	4.09965841	4.19315483	3.63045366
NONHSAG032961	5.07108255	5.01108187	4.94549451	4.99608313	4.88747929	4.80057885	4.95744923	5.00208159	4.82347247
NONHSAG032979	5.39372337	5.17288502	5.10061394	5.33694788	5.30210704	5.30914938	5.30746221	5.35596418	5.08588203
NONHSAG032984	3.5571048	3.53809684	3.59188886	3.71171618	3.86293155	3.78228462	3.59064978	3.81435824	3.81375239
NONHSAG032988	4.23771846	4.17985391	3.88977012	3.94964235	4.09268802	4.41618001	4.47860575	4.12206287	3.75516131
NONHSAG033011	3.26517257	2.84153038	2.76960073	2.98607691	3.08900567	2.76960073	3.19537701	3.08613995	2.72659518
NONHSAG033026	5.00000224	5.04503549	4.70447145	4.93176811	4.66662547	5.37710378	4.93839991	5.09641018	4.62951882
NONHSAG033060	3.53931726	3.42313568	3.5653497	3.35457798	3.58435051	3.7263853	3.5769484	3.76764955	3.3634173
NONHSAG033068	4.13286601	4.42567552	3.94253287	4.2139558	4.15205635	4.3761379	4.27174099	4.43591592	3.97084665
NONHSAG033071	6.09174857	6.42223965	6.02437414	6.14961317	6.04904533	6.25362973	5.96279152	6.1091532	5.91332416
NONHSAG033082	3.57715541	3.22508753	3.05178401	3.0625355	2.98912804	2.62210646	2.6995515	2.90210705	2.71335822
NONHSAG033092	3.59360225	3.51654499	3.53624439	3.61654362	3.58039466	3.81586697	3.59141142	3.6199288	3.45482408
NONHSAG033099	7.58869606	7.29247977	7.10364292	7.04700589	6.90772027	7.4216369	7.05096593	7.0513052	6.88303403
NONHSAG033167	3.96881968	3.94766711	4.05163602	3.7802228	4.04691084	4.13179972	3.94365747	3.95924796	4.1685918
NONHSAG033226	3.83780685	4.0267869	4.13692243	4.19309198	4.07654713	3.96713173	4.3710278	3.83880179	4.44980204
NONHSAG033228	4.38675425	4.33030927	3.86256131	3.97835832	3.63374098	4.02710364	4.27720935	4.00365312	4.16525295
NONHSAG033247	4.54296283	4.4861447	4.20342398	3.90787504	4.1406185	4.1971043	4.11067374	4.17945174	4.03253666
NONHSAG033254	4.74825806	4.84015764	4.74180803	4.55574544	5.0168973	5.29879326	5.22238731	4.68487845	4.82927937
NONHSAG033260	3.56523321	3.72620274	3.58357383	3.56926611	3.59966018	3.59037045	3.76185772	3.98241473	3.37945162
NONHSAG033274	2.62232496	2.35132368	2.61237282	2.53754449	2.64348161	3.00607347	2.60242069	2.52307875	2.90303134
NONHSAG033291	5.25977956	5.55466063	4.88277283	5.07215966	5.108968	5.52916951	5.2659382	5.29476665	5.05871187
NONHSAG033405	4.37515698	4.09851769	4.2632679	4.16550134	4.26795902	4.14246273	4.28155297	4.16194761	4.35646094
NONHSAG033416	4.49063835	4.300467	4.72392964	4.69583	4.614892	4.56012891	4.56518921	4.6197554	4.91066685
NONHSAG033484	5.30666474	5.21919765	5.00693941	5.06646341	5.05509335	5.16624911	5.15129863	4.81053871	5.37369414
NONHSAG033545	2.41062586	2.33368383	2.37475028	2.31463448	2.40723426	2.20060806	2.6851834	2.37009478	2.1371313
NONHSAG033550	4.07841401	4.55513751	4.4308538	4.44613391	4.34821852	4.41824153	4.1811812	4.35292596	4.22247118
NONHSAG033553	4.77690527	4.3309293	4.67232061	4.27951329	4.54237957	4.73538577	4.31631911	4.34958599	4.47678883
NONHSAG033558	2.26744663	2.25448454	2.02790625	2.10584034	1.92732728	2.24072957	1.99706394	2.16903961	2.32777904
NONHSAG033585	3.77661779	3.43016738	3.46629076	3.19708667	3.41326284	3.60151686	3.37117158	3.39683524	3.34454295
NONHSAG033587	5.24558877	4.97711414	4.88954177	5.04736268	4.92499713	5.22481726	4.7443758	4.98936013	4.72204887
NONHSAG033588	5.19284542	5.36455882	5.39484793	5.37629131	5.29590536	5.30044351	5.31424433	5.42501348	5.09600769
NONHSAG033592	3.32088279	3.64931796	3.62986235	3.65297853	3.49120343	3.72080806	3.78918598	3.71527058	3.65459571
NONHSAG033593	3.12767717	3.33412942	2.87449916	3.32076472	3.06651557	3.56610358	3.10714126	3.19978787	3.29431073
NONHSAG033604	4.32695941	3.93492609	3.63004087	3.55812041	3.76342993	3.72781831	3.74840747	3.66793877	3.3191991
NONHSAG033616	5.04665158	5.58733718	5.16645017	5.45089771	5.32602832	5.33563307	5.55984652	5.50558613	5.22007226
NONHSAG033639	3.55090937	4.14400499	4.21810202	3.91115957	4.42721923	4.1332323	4.18909595	4.13272156	3.86929833
NONHSAG033752	4.10041712	3.93939215	3.75734219	4.10531421	3.86464468	4.03035521	3.7202317	3.92326298	3.77662334
NONHSAG033788	4.25700458	4.43941648	4.32947354	4.57133455	4.36113012	4.50516373	4.41225172	4.57875552	4.50079681
NONHSAG033805	4.64616922	4.59169338	4.29696452	4.54247355	4.55622519	4.46244311	4.48085995	4.54322139	4.36137366
NONHSAG033897	5.05067814	4.71861244	5.03896879	4.98677195	4.93014545	5.16048353	4.75533179	5.11357758	5.01326213
NONHSAG033898	6.08464222	5.87599476	5.37114831	5.65423707	5.42801059	5.64253057	5.56425934	5.61865383	5.46132318
NONHSAG033967	4.90166115	5.05294559	4.85986995	4.92183238	4.7178772	5.10068189	4.72605806	5.02294773	4.46702449
NONHSAG033980	3.23204847	3.82679218	3.48229414	3.39844311	3.3525413	3.68601478	3.5889002	3.60455773	3.38476462
NONHSAG033989	5.32360221	5.36863546	5.587474	5.519408	5.5514907	5.10841341	5.65758549	5.62757594	5.40840253
NONHSAG034032	4.06512371	4.3003349	4.29122253	3.88288164	3.85977243	4.11492421	3.93589416	3.99780842	3.73130205
NONHSAG034050	5.36168119	5.28512244	5.1608465	5.26993168	5.1919993	5.41958223	5.23856323	5.30775162	5.08091698
NONHSAG034053	5.21333473	5.2225172	5.27818151	5.07936876	5.1918524	5.41321304	4.96629803	5.13516252	5.02924606
NONHSAG034077	5.20003115	5.75133522	5.4413804	5.48511749	5.45951904	5.64963197	5.53129818	5.51441358	5.5522517
NONHSAG034081	3.08116195	3.18603449	3.16581151	3.49559073	3.31086622	3.14404421	3.52527509	3.32008671	3.4295347
NONHSAG034105	2.95470065	3.36556421	3.21810411	3.49271433	3.13744374	3.17083451	3.41182874	3.20666104	3.02663244
NONHSAG034111	4.80610893	4.89091818	4.86873061	4.68421155	4.47470778	4.91447784	4.51356118	4.88823721	4.73781133
NONHSAG034128	3.29460877	3.28153891	3.23320454	3.14591462	3.14835037	3.07907896	3.2631588	3.15078452	3.18972731
NONHSAG034158	3.92489251	4.29622714	3.96371938	4.23554702	3.80748462	4.41010383	4.16753199	3.7956588	3.89305993
NONHSAG034163	3.07244743	3.0887301	3.47092216	3.35707889	3.51798525	3.42870008	3.03691286	2.65472433	3.12653614
NONHSAG034175	5.979515	5.75430911	5.94768986	5.73768733	5.80156716	5.9991209	5.86305947	5.72088568	5.89855065
NONHSAG034179	6.22744345	6.02275891	6.29580337	6.04586192	6.25651668	5.86787522	6.08805201	5.92332188	6.38191114
NONHSAG034184	4.11422748	4.23051661	4.3044507	4.35472664	4.10127254	4.49942383	4.08856158	4.14957011	4.12827174
NONHSAG034187	5.43935896	5.19768655	4.60324733	4.95087835	4.93336618	5.16500143	5.08151037	4.97430375	4.6915793
NONHSAG034193	4.63925916	4.14708209	4.2918844	4.08818951	4.38796454	4.45174512	4.3040975	4.5632624	4.17451732

NONHSAG034209	5.94887016	6.14581231	6.10611952	6.13841743	6.02091384	6.24589862	5.93939547	6.21755405	5.98589265
NONHSAG034242	5.69765651	5.40223074	5.22321365	5.19462526	5.29274154	5.25386844	5.12447428	5.36185468	5.18392288
NONHSAG034302	7.28170795	7.36224061	7.81858547	7.86707539	7.79351912	7.45203952	8.0225111	7.81527538	7.75054297
NONHSAG034336	3.16631598	2.77915282	2.97630331	3.00460926	2.80772644	3.03201663	2.93506184	3.04680087	2.82901012
NONHSAG034346	2.56754464	2.52980365	2.33578322	2.71814163	2.50754396	2.62619239	2.68985997	2.488013	2.34985933
NONHSAG034364	2.86101844	3.1881488	2.95744418	3.11176199	3.49295341	3.10986141	3.07737055	3.22206168	2.85361768
NONHSAG034372	7.14138427	7.07670918	7.11716253	7.07302876	7.04576452	6.96245056	7.55131947	6.95566958	6.98479444
NONHSAG034414	4.57196549	4.48826369	4.68145954	4.77771652	4.40824588	4.40586465	4.33993749	4.42282249	4.32709066
NONHSAG034441	4.00528805	4.08284076	3.93475496	3.94876922	3.90338634	4.32483591	4.03956276	4.10284916	3.95808984
NONHSAG034453	4.08712716	4.30545379	4.21287585	4.22115788	4.36350399	3.99183149	4.36313343	4.26095018	4.15194437
NONHSAG034459	5.13565167	5.32680256	5.0014153	5.02900277	4.74884383	5.3513627	4.89643151	5.00599935	5.0578208
NONHSAG034470	3.26517257	3.74800089	3.58419571	3.26871442	3.76802873	3.44722881	3.48815277	3.72508595	3.47215786
NONHSAG034501	5.08012552	5.71858906	5.14988825	5.09460882	5.24959302	5.28921751	5.29063427	5.37280276	5.04801035
NONHSAG034507	2.5226924	2.54271803	2.26244582	2.38059581	2.48191729	2.75838528	2.22295142	2.76868407	2.3272906
NONHSAG034552	4.1995158	4.54539297	4.49518417	4.60432352	4.43912944	4.26865485	4.88122349	4.57549558	4.44521607
NONHSAG034566	5.4831692	5.31547016	4.85152059	5.30623959	4.86927994	5.43143789	5.17333557	5.22092307	4.8164243
NONHSAG034569	2.78188408	3.06938136	2.92321357	3.23316804	2.66077866	3.21670839	3.18761798	3.04468106	2.80959805
NONHSAG034584	3.16925174	2.88400494	3.09361154	2.92015523	3.18192178	3.26801498	2.82101202	2.99268512	3.19111941
NONHSAG034600	3.26950557	3.55232124	3.45417103	3.42740891	3.14473697	3.50576843	3.27334323	3.38767427	3.48207552
NONHSAG034618	3.92758627	4.40750072	4.01635648	4.08013646	4.0926967	4.56860137	4.04794187	4.10440733	3.62075584
NONHSAG034646	2.62185731	2.4146885	2.35053016	2.23630361	2.35165122	2.46128849	2.51299937	2.29208008	2.54395826
NONHSAG034655	2.05750246	2.33368383	2.215789	2.24533041	2.33576276	2.24248214	2.34832619	2.31151837	2.27558423
NONHSAG034677	3.16433413	3.18937878	3.02960573	3.06486498	2.99798754	3.11176199	2.68519448	3.14769576	2.98612095
NONHSAG034678	2.63742177	2.66452184	2.61739508	2.30095003	2.24585953	2.73824247	2.54763518	2.51230409	2.37227087
NONHSAG034689	4.47411325	4.51148984	4.74188875	4.50447799	4.49380781	4.50885378	4.40161991	4.56572117	4.49757518
NONHSAG034701	3.95051002	3.63325729	3.80999718	3.75658915	3.66197465	3.75213382	3.78013446	3.80801903	3.85348276
NONHSAG034702	4.29609867	4.24704149	4.50755191	4.28977882	4.35526681	4.83252452	4.31884516	4.41920499	4.31630551
NONHSAG034714	4.50454371	4.7043928	4.59170151	4.61556226	4.66959796	4.27031728	5.23637452	4.6875838	4.14086459
NONHSAG034778	2.84576617	2.89341997	2.75983198	2.97568225	2.75517267	2.73427187	2.87565046	2.64039922	2.85283833
NONHSAG034831	6.53459811	6.53069235	6.9947364	6.57390742	6.92459064	6.60385474	6.86919697	6.70101245	6.82103644
NONHSAG034858	3.61940616	3.084868	3.17424187	3.11437682	3.27122838	3.17190601	3.12621214	3.19045499	3.13848851
NONHSAG034900	2.73398862	2.73008587	2.67764723	2.55943614	2.57258874	2.80005226	2.62869683	2.97308811	2.73311697
NONHSAG034902	2.7348408	2.98935532	2.97994233	2.96099245	2.94418474	3.24336523	3.02712691	3.16699756	2.97317887
NONHSAG034909	5.37393947	5.14900382	4.95648014	5.03828692	4.64857026	4.90063288	4.9084504	4.93441628	4.77500206
NONHSAG034919	3.47866357	3.71334183	3.5685798	3.60136216	3.6167232	3.64147044	3.5685798	3.28604795	3.54860459
NONHSAG034946	3.47938494	4.14831311	3.55167154	4.0603216	3.73034011	3.76428876	3.77574983	3.99142913	3.86461476
NONHSAG034982	4.42998253	4.61249268	4.38001413	4.81240986	4.59931415	4.30356697	4.57168039	4.44237774	4.40873396
NONHSAG035114	4.19181445	3.8303013	3.70586329	3.68046944	3.96625333	4.01555074	3.9710875	4.01870812	3.76405136
NONHSAG035116	5.26139694	5.44187347	5.18974887	5.32100903	5.35971069	5.44755639	5.39977284	5.33801115	5.34225615
NONHSAG035135	3.0771364	2.90958959	2.79392024	2.78926847	2.69647488	3.06671948	2.65410797	2.87077351	3.04137073
NONHSAG035143	6.63710831	6.74185927	6.61543151	6.65936402	6.60059127	6.74194276	6.62934357	6.50073796	6.43900141
NONHSAG035159	5.24845525	5.6151264	5.35858592	5.43320629	5.33246848	5.42371544	5.34459802	5.77733083	4.92879667
NONHSAG035266	2.16505414	2.27105108	2.25577218	2.31335327	2.19865734	2.26750094	2.52351985	2.61904786	2.32228917
NONHSAG035270	3.86568032	4.03009774	4.11217703	4.28999391	4.28462714	4.59054723	4.21038409	4.53060219	4.54654568
NONHSAG035294	3.57905849	3.63398143	3.75686244	3.60097433	3.76580043	3.79735454	3.9898194	3.71287855	3.55366674
NONHSAG035333	3.98497326	3.83656754	3.80920248	3.89129025	3.54505085	3.98497326	3.78156488	4.01157771	3.76216966
NONHSAG035347	4.80436621	4.85225308	4.32822843	4.50481469	4.38889959	4.54860379	4.85612093	4.44340424	4.45563074
NONHSAG035395	3.17436327	2.39056304	2.57587367	2.42272015	2.52193289	2.56740149	2.89977892	2.61904786	2.39864416
NONHSAG035415	4.70709496	4.72966734	4.58482683	4.68282328	4.26676913	4.81771708	4.71191843	4.64615316	4.36007276
NONHSAG035432	5.00575895	5.07824976	4.67431456	4.56415646	4.52578222	5.08106809	4.87362005	4.75045137	4.65543182
NONHSAG035436	3.1214243	2.59408969	2.64500812	2.61011814	2.48707436	2.6970875	2.52831829	2.76890215	2.51289784
NONHSAG035491	3.35667089	3.756721	3.17693298	3.32469995	3.32601154	3.55693433	3.53761337	3.90376671	3.51576567
NONHSAG035503	2.22882063	2.19210166	2.36519074	2.34249876	2.43570637	2.18576548	2.12327641	2.43102988	2.1993073
NONHSAG035505	3.26527762	3.6200358	3.26784557	3.16838203	3.05330567	3.38219493	3.65973855	3.4062393	3.42554636
NONHSAG035508	2.46734916	2.29807422	2.30911837	2.420609	2.32105884	2.37712743	2.21878452	2.28247458	2.30046977
NONHSAG035519	4.59114408	4.30168287	4.34895237	4.35971521	4.50175919	4.46276036	4.95885758	4.30169627	4.49349406
NONHSAG035522	5.57117052	5.57850717	6.34913425	5.76320454	5.96887824	5.72704018	6.03143293	5.80404773	5.8941721
NONHSAG035547	4.27201001	4.33448413	4.46771729	4.28647145	4.51038403	4.59019351	4.36396411	4.27412444	4.28327712
NONHSAG035579	2.44190415	2.27842085	2.50509757	2.42065572	2.55327565	2.38669933	2.52232058	2.54521608	2.43729087
NONHSAG035588	5.46689139	5.19073464	5.04676224	5.10304807	5.36929518	5.46080448	6.1375141	5.29816211	4.93842217
NONHSAG035621	3.65315257	3.9859873	3.47730473	3.60496178	3.59330589	3.80556032	3.59154951	3.73656261	3.46877265
NONHSAG035667	3.62140825	3.15289596	3.22450932	3.18354378	3.05706035	3.17900042	3.08134426	3.03122104	3.18241829
NONHSAG035675	3.36732121	3.13015302	3.26825528	3.25016092	2.92266484	3.28391096	3.1127463	3.25533971	2.86143948
NONHSAG035700	2.60870589	2.96561992	2.43687161	2.45098304	2.6141603	2.85012927	2.85099907	2.5927612	2.78628553
NONHSAG035708	2.67360012	2.478627	2.32697343	2.55010152	2.45800324	2.72484361	2.33273692	2.3910213	2.45828441
NONHSAG035712	2.63494829	2.63283957	2.74887405	2.44809388	2.77712919	2.69725385	2.62651029	2.67935612	2.58986629
NONHSAG035715	2.4451162	2.31239847	2.10215881	2.24661808	2.05081685	2.2129719	2.21726963	2.24495651	2.19393565
NONHSAG035722	4.23282314	4.55029824	4.23805236	4.53942685	3.81307114	4.33863058	3.97471938	4.21884012	3.63982537
NONHSAG035731	2.2113224	2.11831227	2.31637326	2.43471409	2.13169935	2.29208076	2.28223745	2.29660824	2.28925208
NONHSAG035768	6.21121667	6.1527989	6.43715614	6.3817868	6.59748376	6.34564955	6.35267627	6.08893048	6.32613873
NONHSAG035770	6.60289207	6.56719723	6.47304474	6.59942466	6.53481237	6.05886133	7.09276259	6.9074584	6.34873729

NONHSAG035786	3.13809662	2.88280223	2.77770583	2.70817338	2.89279474	2.74360986	2.70617746	2.65115972	2.65320519
NONHSAG035791	3.90867801	3.22038509	3.01147622	2.58559688	2.87514308	2.99628228	2.78059361	2.8487181	2.71738102
NONHSAG035807	2.65930451	2.65719579	2.34636943	2.37353332	2.6769234	2.76817037	2.80770975	2.67466066	2.34095752
NONHSAG035911	9.91123423	10.09419109	10.18181659	10.23438883	10.07547569	10.12513338	10.14148297	10.11729666	10.12574432
NONHSAG035915	2.54433489	2.39636294	2.2731429	2.3575189	2.32884741	2.4438647	2.52457908	2.34570451	2.4612007
NONHSAG035927	2.53069645	2.93810666	2.57527944	2.4660362	2.42618381	2.83855972	2.77250462	2.66034067	2.51394267
NONHSAG035942	2.7348408	2.37622727	2.71143709	2.70274524	2.60676928	2.62322293	2.74590755	2.62831934	2.44674094
NONHSAG035943	5.29824234	5.88164121	5.82613797	5.61990626	5.44048177	5.68658546	5.69541013	5.78835137	5.5819322
NONHSAG035971	4.55819314	4.73753093	4.66581755	5.16353082	4.61453147	4.10344913	4.5514635	5.03784471	4.47223021
NONHSAG035976	5.66482391	5.4711727	5.25649807	5.40742189	5.33510422	5.6491817	5.66130002	5.69138253	5.32863188
NONHSAG035982	5.3700838	5.34916525	5.44244951	5.5014681	5.38204723	5.49553516	5.19378453	5.24939031	5.57724439
NONHSAG036030	3.52544922	3.00457475	3.06236948	3.15170599	3.59658095	3.24380877	3.49389276	3.07843907	3.29262686
NONHSAG036052	5.92025787	5.62802635	5.47685264	5.58531361	5.58116462	5.43297724	5.8531772	5.45699988	5.57104911
NONHSAG036065	2.61600019	2.41664692	2.46799191	2.53946162	2.59951048	2.35512396	2.36877277	2.47086747	2.5195117
NONHSAG036067	8.23657091	8.39207819	8.93819146	8.55276287	8.72253684	8.32056289	8.80648093	8.58110219	8.68838799
NONHSAG036098	5.06202854	4.85754059	5.21696357	4.84276792	5.041968	5.01727237	4.76492147	4.84563598	4.91533193
NONHSAG036130	3.59125793	3.94247469	4.20982747	4.30703116	4.41699838	4.17314547	4.97342125	4.06701911	4.01135721
NONHSAG036141	5.596845	5.09019409	5.3154613	5.54948611	5.48115529	5.23348469	5.32818819	5.18085526	5.09154428
NONHSAG036146	5.18722423	5.51786694	5.43079117	5.66719875	5.2072645	5.50218651	5.3774744	5.57646274	5.50944632
NONHSAG036147	4.33415302	4.56372354	4.32405007	4.18913381	4.43825963	4.69002308	4.49899986	4.50785993	4.28350936
NONHSAG036184	3.47611027	3.43713331	3.17069125	3.03857657	3.13375445	3.15690337	3.17037097	3.1358986	3.24851441
NONHSAG036217	4.470359	4.502573	4.37359608	4.43196344	4.35965702	4.57913847	4.25956591	4.39137586	4.50893008
NONHSAG036227	7.29076315	7.11013888	7.54520182	7.4361701	7.3652373	7.07572469	7.62799118	7.33913308	7.37915547
NONHSAG036242	3.35581582	3.37457628	3.05956272	3.18334359	3.19064515	3.04479291	2.72697373	3.10120402	2.78934227
NONHSAG036289	3.21080042	3.20869171	2.89259503	3.06108482	3.01618145	3.14631551	3.14735172	3.15621682	3.14945863
NONHSAG036290	2.36471291	2.36537867	2.38425727	2.41120181	2.38694743	2.31392378	2.28514881	2.34502512	2.53136132
NONHSAG036298	3.82684842	4.35469489	4.64858461	4.03067638	4.42290832	4.25290128	4.79543699	3.92928257	4.42040109
NONHSAG036307	2.75292018	2.69126354	2.714003	3.11369438	2.81015397	3.21601224	2.69484751	2.88936702	2.68562801
NONHSAG036327	3.04581602	3.43636976	3.10269909	3.04317938	3.15058546	3.23708853	3.12905432	3.17092641	3.10058137
NONHSAG036374	3.32010609	3.71396441	3.37724034	3.05864377	3.03221374	3.42186006	3.06045743	3.43702341	3.35726514
NONHSAG036383	6.38037437	6.28436537	6.00061581	6.160086	6.11744714	6.18201848	5.99171942	6.02469248	6.2394364
NONHSAG036413	2.66169606	2.31621549	2.4140699	2.27032305	2.17174544	2.69408197	2.15550794	2.42100677	2.30270151
NONHSAG036444	2.71846044	2.53599456	2.68534446	2.73897808	2.82086831	2.70510602	2.92177576	2.73897808	2.77171298
NONHSAG036455	2.40983344	2.66640535	2.51806605	2.6435339	2.68430508	2.6328778	2.73604156	2.47157081	2.55726287
NONHSAG036462	3.49801955	4.01966976	3.26646806	3.77745393	3.51150281	3.73126229	3.82032804	3.82888642	3.40020739
NONHSAG036486	2.39669437	2.46093026	2.69659599	2.72997651	2.47431733	3.03453893	3.02152816	2.44433284	2.5348397
NONHSAG036538	3.29624745	3.27353914	3.11618079	3.17235201	3.28998033	3.27975774	3.2457599	3.36734902	3.2754505
NONHSAG036539	6.28087015	5.87950595	6.10210485	5.89690011	5.99332735	5.79356679	5.90330312	5.62719749	6.14854292
NONHSAG036566	3.04885055	3.06349686	2.78253571	2.6983055	2.89236003	2.816717	2.89034063	3.01770291	2.85495861
NONHSAG036587	3.80511501	3.48479973	3.85995112	3.53480931	3.67257997	3.50168045	3.50778875	3.46099961	3.70227393
NONHSAG036631	5.40295402	5.29073697	5.47837207	5.24611743	5.55369696	5.49394922	5.68802542	5.55755309	5.18674319
NONHSAG036645	2.4484179	2.24591708	2.33344343	2.21531611	2.32988005	2.47963911	2.20877633	2.2603153	2.27034387
NONHSAG036667	2.7462788	2.82121819	2.6727558	2.50672707	2.52123455	2.69336292	2.80320482	2.87117726	2.65742068
NONHSAG036669	3.49431369	3.92208089	3.79408872	3.75018739	3.68297976	3.66160345	3.48801972	3.64675076	3.86461476
NONHSAG036696	2.72707229	2.45888403	2.84612001	2.68977013	2.23694144	2.40040164	2.48357459	2.69886583	2.26115243
NONHSAG036703	2.19672996	2.27275165	2.25517493	2.31316179	2.49715166	2.23114126	2.34076964	2.24085004	2.25435653
NONHSAG036710	2.59179628	2.83818817	2.79775338	2.95562756	2.88272783	2.78725259	3.25535864	3.21472318	3.17987777
NONHSAG036741	2.09792751	2.21429345	1.82976242	1.99442531	2.21787082	2.39320741	2.21302154	1.89080719	2.09404079
NONHSAG036745	4.31879078	4.25786808	3.7441979	3.63004795	3.6991725	4.05689706	4.03983645	3.70811098	3.74264307
NONHSAG036767	2.71499811	2.75926958	2.68825348	2.8816543	2.84548754	2.90901729	2.82571223	2.68204398	2.95304349
NONHSAG036776	3.72340135	3.84121649	3.35441591	3.42252662	3.48233722	3.48664048	3.35417722	3.31994348	3.64514621
NONHSAG036803	3.09100618	3.41921356	3.1728267	3.0132352	3.0717717	3.21524169	3.11922621	3.20174069	3.18590216
NONHSAG036808	4.03768478	4.18290958	4.55771	4.31792716	4.31403469	4.20118994	4.17916446	4.12079693	4.10884672
NONHSAG036869	4.36475024	4.36007047	4.5795361	4.52727176	4.20719766	4.44651494	4.31357657	4.2738976	4.4080267
NONHSAG036874	4.1455092	2.88409199	2.73104102	3.04536512	3.13258255	2.9703244	3.62813608	3.05156818	2.96743878
NONHSAG036892	2.63107637	2.808658	2.5242035	2.73123917	2.74446585	2.8719379	2.79088177	2.73091783	2.80833891
NONHSAG036926	6.0462196	6.72510164	6.16528832	6.60450286	6.44488539	6.63483017	5.87275567	6.3722155	6.24114833
NONHSAG036955	3.40112283	2.98905844	3.32367319	3.29662523	3.14685066	2.90458055	3.20569846	3.02946966	3.09178679
NONHSAG037015	3.30198114	3.541758	3.21131083	3.21356478	3.19373252	3.59854911	3.28540232	3.26898413	3.17855798
NONHSAG037017	4.53996925	4.55484154	4.56039773	4.4984578	4.43873957	4.47502374	4.5609032	4.57549185	4.47223021
NONHSAG037020	3.38806468	3.72527607	3.49869216	3.23170185	3.22443806	3.49373049	3.62653289	3.46099961	3.30767347
NONHSAG037021	2.38971312	2.49646699	2.70685029	2.44313	3.13246279	2.95794533	2.55616756	2.44492214	2.81145012
NONHSAG037022	7.26835188	7.1115603	6.79928192	6.85341997	7.12460637	7.22272699	7.11020221	6.96276233	7.0379137
NONHSAG037025	2.86078307	2.9106361	2.77770583	2.80359052	2.75658717	2.67503533	2.66485057	2.81246598	3.04889728
NONHSAG037030	6.32900974	6.14172086	6.35869025	6.41848011	6.50083739	6.45746275	6.91952517	6.41393619	6.09259604
NONHSAG037031	5.44750481	5.66470177	5.36070142	5.47160046	5.39576406	5.3399499	5.3111315	5.39874699	5.27791423
NONHSAG037032	3.26889883	3.38416831	3.29420964	3.26494474	3.44026563	3.26299092	3.17533415	3.27636855	3.47395183
NONHSAG037083	3.45700263	4.19101354	3.27615546	3.4598574	3.50774314	3.28647581	3.56800483	3.51760624	3.38085967
NONHSAG037087	7.54481624	7.62863156	7.3932016	7.44701874	7.36109707	7.47303341	7.69640653	7.55678567	7.22484097
NONHSAG037150	4.87791015	4.63258638	4.51490392	4.71173834	4.67652391	4.63259733	4.4910142	4.79012204	4.60800747
NONHSAG037173	7.55139528	6.81975992	7.17159899	7.03935618	7.05782358	6.89933868	7.1050073	6.63451853	7.15549812

NONHSAG037197	4.96822847	6.40464062	6.59774613	6.42678138	6.61470295	6.2715543	6.73902821	6.23182823	6.17474341
NONHSAG037200	5.78188591	5.64623173	5.37020121	5.59693169	5.28595325	5.28444231	5.23532113	5.50300496	5.29340311
NONHSAG037224	7.2218148	6.75578467	6.4048665	6.71217405	6.46316527	6.7434035	6.50826058	6.37782619	6.15428458
NONHSAG037227	5.63683016	5.82499135	5.38916418	5.55660167	5.52956188	5.73540397	5.26999326	5.60988631	5.35310091
NONHSAG037229	5.17269344	5.29572605	5.16527897	5.1641326	5.22547174	5.36506301	5.21743268	5.30043989	5.04489468
NONHSAG037251	2.82304514	2.7342101	2.50622055	2.56797005	2.59575653	2.62898364	2.69713083	2.82022274	2.62173514
NONHSAG037262	6.21434286	6.28180373	5.98337697	6.0932642	6.09688839	6.33867131	6.05238667	6.13898363	5.88136362
NONHSAG037272	4.12913734	4.74402371	4.85221309	4.61423162	4.58523011	4.4843145	4.70909385	4.80109626	4.64731512
NONHSAG037287	4.74358187	4.58225578	4.5357801	4.50074796	4.34821852	4.2380769	4.31198068	4.35367541	4.74658167
NONHSAG037322	2.49085722	2.33804123	2.714246	2.42043221	2.3671449	2.39937501	2.43222699	2.57541751	2.46334196
NONHSAG037336	3.25433625	3.79212319	3.83707377	3.927496	4.05808238	3.27806868	3.93837812	4.1453301	3.44701403
NONHSAG037338	5.06160635	5.04961337	5.24304715	5.02176112	5.11703123	5.14876995	4.95429	4.9846731	4.90634161
NONHSAG037352	5.34216376	5.19797532	5.08356326	5.15432796	4.93033754	5.49393585	4.93459066	5.18761075	5.03556258
NONHSAG037375	3.03109596	3.22045441	2.89058811	3.11437682	3.30071797	2.75363651	3.18225684	3.23931746	3.06857415
NONHSAG037376	3.42080948	3.38577244	3.45927525	3.40773503	3.7908893	3.73111514	3.55414854	3.45898915	3.44065023
NONHSAG037395	7.04668395	6.67522879	6.93665794	6.71080802	6.77183993	6.77710264	6.84106931	6.82402186	7.23309225
NONHSAG037396	5.88948922	5.51969164	5.34827304	5.27633351	5.46327159	5.40306421	5.5962019	5.56823522	5.47590512
NONHSAG037407	5.22163818	4.39532183	4.53694036	4.54760958	4.48637163	4.44891195	4.41761557	4.42010417	4.63758139
NONHSAG037426	4.8479273	4.579464	4.70118746	4.58355682	4.34795917	5.21282241	4.43471246	4.65347371	4.68134105
NONHSAG037431	4.34233975	4.79153259	4.53938567	4.76019438	4.70205606	4.30331278	4.45926086	4.89823427	4.19293079
NONHSAG037432	6.21226906	4.77028605	4.68554123	4.48267373	4.5285104	4.81585284	4.7124944	3.99295333	4.90276984
NONHSAG037433	4.81823203	3.83821557	4.15661223	3.89775753	4.13603446	4.30009628	4.04266739	4.19847686	4.31220437
NONHSAG037464	4.05755971	4.01641369	3.87146101	3.87453086	4.37805964	3.99850471	4.05447695	3.97057692	4.00216013
NONHSAG037470	4.22413411	3.7860625	3.4392414	3.52295415	3.31875141	3.71085328	3.70712638	3.68731478	3.4420742
NONHSAG037504	3.31755632	3.36913502	3.07944985	3.36171319	3.25957398	3.33048059	3.18287707	3.38391328	3.14474568
NONHSAG037513	3.95829397	3.14834501	3.14759433	3.36231579	3.33220869	3.58358148	3.17808699	3.10427901	3.12891935
NONHSAG037525	3.62092038	4.13239126	3.88846224	3.62981027	3.87688254	3.97059548	3.83988291	3.93985833	3.7263694
NONHSAG037563	5.07078185	4.19554734	4.38682705	4.50778673	4.5784418	4.7168008	4.37313264	4.48318909	4.65912256
NONHSAG037570	3.5406404	3.54634565	3.33250387	3.66698207	3.43104938	3.83818792	3.45096168	3.64821691	3.55404155
NONHSAG037575	5.4580727	5.40313352	5.25043583	5.41671253	5.17658992	5.47779164	5.28816374	5.50829798	5.21515302
NONHSAG037577	3.81101199	3.62322775	3.49106654	3.78537854	3.81222317	3.91276488	3.66117483	3.76914959	3.93861763
NONHSAG037622	3.36396585	3.51611976	3.33413671	3.15945999	3.07775645	3.60754153	3.12509641	3.80130344	3.19884807
NONHSAG037629	5.26330917	3.83585155	4.80242228	4.43119268	4.603998	4.01950857	4.37632667	4.05840505	4.28930222
NONHSAG037701	3.4230612	3.34802821	3.44254395	3.36074582	3.66851596	3.95613302	3.14789393	3.3777201	3.35887408
NONHSAG037719	3.02037284	2.71861262	2.74382703	2.56499456	2.91789171	2.75811685	3.07480929	2.72736448	2.98760954
NONHSAG037724	2.53917852	2.13957428	2.22622555	2.28573186	2.22070082	2.21787808	2.21874852	2.10826876	2.30417989
NONHSAG037726	2.3905297	2.09329525	2.25566232	2.29514374	2.21654057	2.28286998	2.19124211	2.23730325	2.45468822
NONHSAG037747	3.72542558	3.93303966	3.71339683	3.50071849	3.88384783	4.07337352	3.87056118	4.03965483	3.77036284
NONHSAG037751	4.1106706	4.25805777	4.12099084	4.04267949	4.16804172	4.49815462	4.17255803	4.22561184	3.95820101
NONHSAG037786	7.69765714	7.22428641	7.41821466	7.25187525	7.38698529	7.3674681	7.30983661	7.46527954	7.51720247
NONHSAG037791	2.61501003	2.5014215	2.51599293	2.22522333	2.74519776	2.4501213	2.43471409	2.48374024	2.43431386
NONHSAG037804	3.72210315	3.65530351	3.68737308	3.47686224	3.62074314	3.55942092	3.90697316	3.64675076	3.22651972
NONHSAG037839	5.7662915	4.86252819	5.15980775	5.22104829	5.08590783	4.866821	5.0256424	5.03784471	5.28450293
NONHSAG037842	3.23809594	3.38668721	2.878721	3.52186089	3.26468455	3.11534361	3.29718675	3.19144204	2.95364975
NONHSAG037849	3.24693006	3.48851532	3.21715731	3.20605842	3.25313616	3.3593338	3.71416664	3.27424585	3.17348495
NONHSAG037860	3.50034864	3.58465545	3.57518709	3.43209614	3.59483001	3.47104221	3.53331081	3.35485865	3.16885804
NONHSAG037870	2.19892121	2.34389158	2.14864591	2.25174728	2.22764984	2.55905349	2.3488595	2.43861432	2.13608181
NONHSAG037873	2.58343043	2.13285915	2.36897272	2.45564538	2.18773067	2.36224575	2.45188477	2.35398316	2.34438415
NONHSAG037878	2.50961313	2.36970796	2.33208354	2.28581901	2.24181717	2.48664813	2.40170027	2.33457831	2.45820623
NONHSAG037943	2.90989036	2.96331802	2.95767232	2.94204744	3.0131169	2.97706211	2.96318681	3.00546744	2.87598441
NONHSAG037955	2.30549648	2.34310037	2.24491185	2.16390099	2.00022035	2.28085107	2.19980333	2.03933642	2.14265242
NONHSAG037959	3.64247636	3.16643767	3.27753838	3.18125024	3.2161751	3.04918737	3.05368678	3.2020594	3.04289639
NONHSAG037962	4.15723681	4.39533042	4.15723152	3.98000023	3.8553439	4.01885936	4.02983859	4.47413826	4.15428125
NONHSAG037977	7.28948234	6.30275785	6.26340004	6.16590159	6.27556214	6.39470831	6.98546835	6.53801632	6.25193073
NONHSAG037987	2.23475141	2.27183068	2.42498729	2.2397921	2.29370218	2.46523629	2.46810617	2.34146701	2.43954574
NONHSAG037991	3.96758242	4.07501102	3.78751577	4.04652874	3.73226726	3.90947052	3.621272	3.97116775	3.86293948
NONHSAG038027	3.72391008	3.67131685	3.30027496	3.53530091	3.39429456	4.23404118	3.62424176	3.72398139	3.34736376
NONHSAG038035	2.56027428	2.20472537	2.04273787	2.38777265	2.27572728	2.09178485	2.19171325	2.02142354	2.20648623
NONHSAG038048	2.41288376	2.33748223	2.52117914	2.66830356	2.42895935	2.298528	2.43761928	2.50580021	2.29476624
NONHSAG038071	4.32941791	4.32430624	4.14700094	4.1119761	4.07885113	4.09008983	3.94690116	4.13922802	3.91068823
NONHSAG038091	3.70874386	3.39118669	3.24602544	3.20773303	3.73403167	3.52596333	3.44139569	3.3075856	3.318515
NONHSAG038132	5.32681512	4.73842153	4.88475326	5.02384006	4.63407345	4.84836674	5.13918788	4.81647451	4.84458552
NONHSAG038179	2.50353022	3.84946764	3.28369836	3.36346864	3.34146585	3.62918824	3.54436469	3.81592231	3.31692021
NONHSAG038210	3.92489251	4.41945057	4.07474176	4.19969126	4.03167001	4.35543461	4.00304333	4.24565899	3.76188994
NONHSAG038213	2.76578699	2.92299378	2.82857693	2.79756126	2.74925656	2.88130707	2.85492809	2.80000761	2.8258188
NONHSAG038259	3.39389861	3.41731634	3.27150148	3.23582306	3.35164137	3.39446013	3.37101784	3.29009907	3.15041294
NONHSAG038285	3.44492933	3.62148773	3.21658446	3.39997973	3.30329728	3.55169135	3.57214143	3.72831326	3.10662542
NONHSAG038292	2.43170958	2.45248206	2.390435	2.43203091	2.34317289	2.51394955	2.50125224	2.45487205	2.32180984
NONHSAG038333	5.4627412	5.43894523	5.70773585	5.55592388	5.71698916	5.45300031	5.43010234	5.54901463	5.51963325
NONHSAG038350	2.90521252	3.4083416	3.09119803	3.13352322	3.157071	3.37268851	3.01966474	3.31823407	2.80959805
NONHSAG038364	3.62737998	3.62787976	3.28339383	3.41951249	3.36010852	3.3267666	3.46468863	3.35918632	3.45304117

NONHSAG038417	6.88145035	5.38786389	5.61579905	4.91640991	4.97189317	5.0136797	5.94231795	6.07425529	6.07468533
NONHSAG038444	2.79873387	2.90569801	2.87531228	3.07165507	2.8458569	2.80959805	2.63324827	2.63252802	3.09178679
NONHSAG038460	9.64170374	10.03285381	10.23733505	10.05224117	9.95427167	10.02877751	9.9103139	10.11219497	10.05347211
NONHSAG038476	3.66474305	3.91867022	3.75250791	3.70276377	3.37726388	3.68698955	3.61377694	3.74732555	3.76300987
NONHSAG038511	2.54841629	2.19846226	2.22857772	2.38916284	2.38159908	2.3693034	2.3618802	2.38199689	2.36435738
NONHSAG038512	4.64864882	4.15464422	3.9834188	4.50446517	4.04297651	4.37303845	4.38294503	3.83101066	3.88588235
NONHSAG038522	5.50832195	5.73447194	5.45210027	5.41409572	5.52260182	5.35253167	5.6249084	5.72798878	5.59595401
NONHSAG038586	5.33204074	5.77953454	6.84628724	6.17742176	6.35242421	6.03007615	6.01941688	5.65536825	6.18585262
NONHSAG038591	2.68259452	2.56718682	2.48012634	2.56829634	2.43565327	2.51841354	2.43498166	2.62076797	2.61509416
NONHSAG038599	3.04588809	3.36285408	2.98690361	3.09154929	3.16398025	3.27084507	3.11681643	3.2134499	2.70789036
NONHSAG038627	6.64825458	6.65489853	7.09359763	6.7235133	7.13798862	6.66344685	7.05640336	6.80344319	7.11462401
NONHSAG038649	3.74760741	3.94046205	4.3206254	3.77969859	3.7030673	3.73049941	3.817558	3.78530785	3.86868285
NONHSAG038724	2.75491953	2.43702906	2.4247168	2.37136416	2.53788394	2.52229358	2.74141385	2.70627638	2.41068634
NONHSAG038765	3.80511501	4.13986439	4.21175216	4.10648917	4.05091743	4.21474162	4.31412956	4.34373267	4.06995375
NONHSAG038779	3.19622178	3.27554933	3.57062048	3.40122555	3.69284461	3.23006274	3.70610351	3.7521947	3.41136607
NONHSAG038780	2.37521043	2.23320989	2.08401352	2.17277667	2.17229112	2.46680241	2.19640408	2.1662107	2.19437017
NONHSAG038818	2.17858754	2.26321036	2.18962211	2.00331308	2.18117887	2.43904531	1.99102096	2.26056969	2.21479667
NONHSAG038845	5.61179412	5.96443034	6.37674147	6.15654843	6.48543514	6.08162056	6.40130326	6.04151572	6.4190221
NONHSAG038869	2.27633257	2.12510348	2.2930941	2.4324533	2.38694743	2.21104201	2.31207172	2.1292865	2.2610683
NONHSAG038874	2.18238162	2.35772073	2.41676307	2.32204239	2.51555389	2.62732051	2.65029107	2.39513073	2.62103325
NONHSAG038882	2.58183925	2.53264689	2.27362368	2.35525874	2.58463459	2.50122509	2.34184435	2.45689357	2.44308587
NONHSAG038890	6.11400539	5.08596501	5.70275296	5.49377028	5.1929107	5.21498264	5.70877957	5.08153417	5.51939288
NONHSAG038899	3.26517257	2.72041177	2.66771269	2.7869822	2.64017566	2.78775169	2.63353586	2.71616001	2.71335822
NONHSAG038901	3.94876833	3.93143072	4.0883004	4.20583446	4.4016125	4.26190571	3.85319131	4.25835488	3.55365669
NONHSAG038916	2.62616122	2.41435521	2.21028663	2.3865985	2.34390638	2.36912059	2.60025638	2.17567915	2.37499876
NONHSAG038917	2.58163919	2.86429864	2.5058831	2.22326553	2.50644208	2.48221262	2.56969181	2.43412912	2.43600542
NONHSAG038920	3.71939143	3.69732081	3.80572714	3.75885518	3.70342209	3.59334664	3.87642846	3.97989237	3.89272448
NONHSAG038949	5.29918541	4.88807515	4.66378443	4.73529012	4.85397953	4.7352578	5.14307501	4.88591367	4.80965575
NONHSAG038955	2.86101844	2.72824942	2.686331	2.74650072	2.70579606	2.65127622	2.65636469	2.58972407	3.0693752
NONHSAG038958	2.66579726	2.58490623	2.89671249	2.84227851	2.60591371	2.80959805	2.69033132	2.60625637	2.73682037
NONHSAG038962	3.54947386	3.74746836	3.39549287	3.3195804	3.43377683	3.72707936	3.65564014	3.51140726	3.48571949
NONHSAG038974	4.46518981	4.25476746	4.03389109	3.88961659	3.88784777	4.21474162	4.21797474	4.2244045	3.82144066
NONHSAG038975	3.74660207	3.40399485	3.40290972	3.49158854	3.40420081	3.52239527	3.51895643	3.68251133	3.46191333
NONHSAG038999	2.37624603	2.27180728	2.49316335	2.44658435	2.26041129	2.35779679	2.14864591	2.47091216	2.37855896
NONHSAG039007	2.3439372	2.01253925	2.04871751	2.54710125	2.32747148	2.36905883	2.21302154	2.16110468	2.44813063
NONHSAG039009	2.52690854	2.36444781	2.28355346	2.19397448	2.40291253	2.1289223	2.02754241	2.36001917	2.30785398
NONHSAG039017	3.09565942	3.0706538	2.94342908	2.88724947	3.14657798	3.28418742	3.03770869	3.10017997	2.94989437
NONHSAG039019	3.6221994	3.25884942	2.83271399	2.935195	2.87627232	3.21467493	3.19521026	3.0638811	2.96745348
NONHSAG039034	4.19965806	3.48248467	3.03616577	3.25885272	3.17979253	3.72858871	3.71409502	3.23602089	2.88358087
NONHSAG039040	2.80640163	2.86883149	2.89984057	2.98682443	2.83658219	3.07660304	2.58891994	2.5847921	2.91101353
NONHSAG039046	3.14834529	3.83821557	3.32830395	3.31451394	3.14695215	3.35627907	3.13915936	3.37184187	3.35967368
NONHSAG039047	2.86605331	2.84964351	3.05178401	3.10723825	2.88237509	2.84758283	2.88573331	2.94374802	3.0576753
NONHSAG039060	3.16926968	3.13440934	2.90577292	3.09408739	3.04922747	3.1804752	3.01006824	3.07161631	3.33460596
NONHSAG039061	2.95618298	3.06097944	2.93813137	2.79372078	2.88862316	2.87593997	2.88586581	2.98747874	2.89477879
NONHSAG039063	2.97728857	3.12859078	3.07895732	3.10070505	3.03872788	3.16749898	3.11176199	3.28604795	3.14988583
NONHSAG039082	4.45513733	4.45578014	4.20783218	4.25685936	3.84726429	4.21595725	4.26653858	4.43512278	3.92848515
NONHSAG039102	3.31279541	3.69733609	3.3234542	3.47879712	3.46408485	3.65035172	3.51728078	3.51256647	3.37735658
NONHSAG039105	4.87732308	4.84923503	5.2679545	4.9098891	4.83738973	4.89033245	4.8257239	4.60327261	4.89827518
NONHSAG039107	5.6622854	5.56941022	5.75456877	5.47864304	5.53837191	5.57223422	5.66356147	5.61776815	5.20258455
NONHSAG039111	3.06422575	3.06683085	3.20562181	2.87091413	2.97914708	3.27904662	3.0031072	3.01847546	2.84874379
NONHSAG039112	4.86243992	4.90554867	4.53752882	4.66507728	4.48855022	4.67789048	4.40741534	4.62539352	4.38463107
NONHSAG039130	3.57720737	3.00128685	3.45375363	3.15120011	3.02018496	3.01539255	3.30526315	3.09900132	3.11559145
NONHSAG039135	4.5451368	5.05835914	4.95558539	4.8230478	4.77337154	5.03157308	4.87015085	4.92841579	4.97842909
NONHSAG039189	2.32233344	2.26164633	2.40283283	2.1586773	2.10868833	2.20881357	2.1586773	2.14821202	2.3174068
NONHSAG039190	3.73891548	3.46129715	3.57017313	3.40020739	3.60191939	3.53530091	3.49067126	3.95869711	2.98860553
NONHSAG039191	2.67087508	2.57008634	2.63793934	2.59960592	2.59262707	2.73649993	2.78576748	2.64670228	2.56799977
NONHSAG039225	3.87231026	3.31553455	3.39844013	3.30295695	3.34259095	3.33318182	3.45009052	3.43568876	3.32437991
NONHSAG039294	2.37960231	2.85384268	2.44813063	2.62373371	2.63985749	2.71481603	2.63985749	2.79098278	2.80514987
NONHSAG039315	4.25759976	4.71257596	4.28839267	4.23286335	4.14254991	4.5371051	4.39712807	4.33804419	4.15643644
NONHSAG039321	2.97863775	2.49043836	2.75439145	2.5014215	2.5289541	2.66006371	2.46110504	2.84082749	2.73964065
NONHSAG039327	3.57698166	3.97215576	3.64026886	3.83690729	3.60205733	3.55763049	3.85461589	3.78611034	3.57003235
NONHSAG039332	2.8031679	2.79367651	2.64632926	2.79565561	3.04677388	3.00537648	2.76167015	2.97044195	2.71567749
NONHSAG039337	3.20463008	2.45744551	2.46433453	2.45017581	2.5073283	2.61300013	2.50142294	2.47072209	2.75242397
NONHSAG039356	2.89991398	2.97784433	2.77447774	2.70325834	2.81880693	3.04012633	2.8275294	2.89822944	2.55791778
NONHSAG039358	4.00123183	4.50779722	4.197942	4.10956767	4.28865406	4.55756188	4.25892244	4.51224846	4.23371024
NONHSAG039367	3.43782769	3.15745816	3.13698302	3.61667463	3.27720528	2.96479674	3.27760261	3.20812298	3.57773841
NONHSAG039377	3.95572022	4.12674308	3.9687353	3.77414647	4.05646103	3.915294	4.10206634	3.79321617	3.80314523
NONHSAG039399	5.34339815	5.57120476	5.61976498	5.31326638	5.34507148	5.45875813	5.57835492	5.55429882	5.76161814
NONHSAG039414	2.84002728	2.90095005	2.91671761	2.84316032	2.79019956	2.90075581	2.87150467	2.97329112	2.86484737
NONHSAG039470	4.28898389	4.65120431	4.53475916	4.85155636	4.31397162	4.42061863	4.42646821	4.80143314	4.55192829
NONHSAG039489	3.42381157	2.80011666	2.88560495	2.67390901	2.78771485	3.06493546	3.11176199	2.87703796	2.69727102

NONHSAG039490	4.50124423	4.39649715	4.52488294	4.62989398	4.592541	4.37403037	4.49421412	4.40528409	4.57456292
NONHSAG039502	4.18165407	4.65053308	3.83592453	4.14748814	4.23420882	4.35588794	4.35345994	4.15073527	4.00246321
NONHSAG039503	5.26868278	5.58166422	5.44335949	5.3579892	5.3254744	5.73618027	5.33796637	5.32103146	5.22457502
NONHSAG039511	3.22950545	3.80804262	3.55234129	3.53530091	3.51974713	3.74061239	3.66681739	3.70108169	3.51550684
NONHSAG039515	3.92031294	3.32950415	3.05894595	3.12271757	3.19684503	3.23045558	3.03149692	2.97962807	3.33233256
NONHSAG039551	5.3063386	4.87366063	4.85787257	5.0372909	4.97754919	5.34507842	4.72786709	4.93669107	4.92473637
NONHSAG039586	2.23360157	2.33147048	2.28292994	2.33535827	2.24784476	2.31142272	2.31043551	2.10294132	2.32290196
NONHSAG039670	5.18471182	4.85894712	4.55834389	4.60878479	4.59501364	4.90102546	4.65394454	5.01246287	4.72650761
NONHSAG039676	8.78133674	8.90413091	9.02181374	8.94346235	8.98349338	8.66818648	8.92754363	8.93198371	8.88130723
NONHSAG039681	2.96505203	2.99412943	3.09100637	2.97977804	2.68252352	3.53948372	2.99291787	3.07767932	3.31525516
NONHSAG039701	5.0668431	5.22383091	4.8953437	4.72310464	4.64955475	4.83187312	4.74378403	5.00947369	4.63460227
NONHSAG039715	2.58824499	2.90907916	2.77085388	2.76909525	2.71667342	3.06604809	2.82103824	2.94536134	2.83777428
NONHSAG039740	2.50353022	2.70115107	2.41842479	2.5364412	2.5130166	2.61571185	2.37881905	2.35775546	2.30315662
NONHSAG039770	2.5314997	2.79254397	2.49042306	2.6512667	2.68866164	2.69188198	2.75453833	2.69037438	2.63882996
NONHSAG039813	2.98046898	2.64579406	2.43710878	2.61011814	2.85746001	2.85644018	2.75453833	2.7507832	2.5547436
NONHSAG039828	3.27709277	3.29227663	2.90285611	3.29912261	3.15154057	3.56604356	2.87427073	2.82209612	3.36991835
NONHSAG039846	2.92928784	2.63976799	2.63729147	2.75664616	2.69229814	2.94102532	2.84695668	2.83456175	2.87868724
NONHSAG039857	4.05303958	4.32586488	3.69018129	3.63277651	3.63091996	4.02700131	3.78583147	4.1993057	3.68572024
NONHSAG039863	6.54006068	6.73931916	7.0200395	7.15914184	6.85314899	6.59005605	7.28502322	7.19616159	6.86960316
NONHSAG039875	7.06250149	6.55189554	6.71288622	6.68554838	6.61808397	6.356029	6.67746259	6.67007338	6.33375519
NONHSAG039889	3.66579964	3.74689753	3.7267387	3.51437676	3.45310072	3.92693394	3.72612334	3.6825207	3.41038546
NONHSAG039949	2.79057934	2.79259403	2.78697363	2.66897815	2.82382394	2.91027069	2.93510891	3.03331261	2.70320038
NONHSAG039968	2.16905444	2.04584049	2.0126854	2.34755709	2.31458956	2.44999734	2.08075288	2.24086061	2.28514881
NONHSAG039971	3.09485335	3.02897147	3.23299414	3.09821159	3.10241025	3.19352192	3.20679937	3.62732592	2.79815408
NONHSAG039976	2.81929475	2.56332481	2.45634086	2.43466543	2.51680298	2.48523649	2.5612124	2.51904902	2.58076218
NONHSAG039991	4.27911336	4.48560517	4.27972173	4.4581638	4.30542693	4.11984643	4.26625051	4.20891383	4.25719533
NONHSAG040004	2.55726493	2.27169579	2.25890684	2.40210804	2.1867359	2.66320832	2.44469779	2.36171732	2.27886756
NONHSAG040070	2.94790744	3.24434217	2.83565837	2.91833807	2.87703488	3.28311693	2.95584147	3.22547146	2.85480318
NONHSAG040081	2.94216216	2.2171546	2.40630251	2.37744321	2.01743012	2.47968773	2.19149896	2.4762583	2.59950995
NONHSAG040093	2.50353022	2.15799753	2.26314554	2.69738727	2.18811301	2.44183781	2.35779679	2.21625511	2.21961691
NONHSAG040100	2.45580781	2.82357541	2.68478529	2.45558396	2.4971483	2.78285844	2.83116903	2.64349683	2.60934078
NONHSAG040122	5.4733608	5.56383453	5.42007327	5.34842841	5.62109892	5.51295337	5.63580748	5.60087796	5.53646092
NONHSAG040157	5.00369618	5.07914568	5.39625014	5.1302796	5.37283922	5.05216604	4.80613533	5.08379493	5.08368314
NONHSAG040159	4.99601196	4.9575136	5.01801544	4.76117291	4.83215719	4.49445061	5.01360152	4.59168569	4.75516374
NONHSAG040185	7.5591533	7.52252019	7.49164237	7.47621709	7.35411346	7.35511841	7.48339397	7.27795766	7.48675088
NONHSAG040189	3.00098226	3.11323503	3.03823558	2.91206154	2.97803058	3.00346511	2.79608777	3.10976559	2.80945835
NONHSAG040206	5.21532196	5.70360978	6.3361776	6.1846455	6.18142571	5.77236229	6.04926335	5.55837522	6.162561
NONHSAG040218	2.40080296	2.38152882	2.44813063	2.37411433	2.57619396	2.48195195	2.32614295	2.49018972	2.52300324
NONHSAG040274	2.2236425	2.24254471	2.3545167	2.33126729	2.25228004	2.20116165	2.14864591	2.09306121	2.38099019
NONHSAG040276	2.45900675	2.35400635	2.33648536	2.51056704	2.57210518	2.44634911	2.59595877	2.47775555	2.46493461
NONHSAG040302	5.72716488	5.30794469	5.41571444	5.30457557	5.29632689	5.2962521	5.63643019	5.34137778	5.46473082
NONHSAG040333	7.41985669	7.08813286	7.29924566	6.99082728	7.19778601	7.05055742	7.18441589	7.02362703	7.074377
NONHSAG040375	3.26969104	3.28490977	2.89664191	3.10160752	3.06489099	3.07972125	3.47315732	3.00926367	2.99741263
NONHSAG040378	3.89539762	4.10478547	3.82281847	3.92055416	3.90980147	4.21014695	3.99356679	4.09200061	3.82605864
NONHSAG040385	3.61641135	3.73888462	3.6842096	3.49844594	3.69586642	4.29128765	4.04057673	3.63911143	3.56136166
NONHSAG040395	3.27264575	2.94220433	2.75348983	2.95838304	2.78318011	3.12321423	2.9169337	2.92951112	2.75134998
NONHSAG040459	2.60013032	2.85826053	2.53348546	2.65157679	2.50388654	2.670327	2.63523271	2.34807299	2.64800141
NONHSAG040465	3.16014374	3.27547594	2.98196638	3.00113159	3.08877614	3.00053284	3.40694694	3.05554477	2.96946434
NONHSAG040492	3.10259632	3.1517197	3.12339821	3.02577913	3.32866588	2.91945758	3.13895261	3.2364405	3.18856845
NONHSAG040535	5.13282729	4.87501233	4.58827743	4.80659046	4.76935773	4.52479521	4.85266739	5.05186598	4.85950176
NONHSAG040538	3.50772673	3.38245267	3.11517778	3.33460491	3.08075326	3.26749454	3.15308998	3.3004788	3.11641502
NONHSAG040545	3.6700426	3.61838899	3.5578148	3.58184561	3.4814652	3.7505046	3.63024014	3.67038246	3.73727743
NONHSAG040546	4.2026838	4.30347786	3.55948141	3.87547875	3.77790833	3.93844848	4.55704466	3.95030315	3.79777618
NONHSAG040559	4.65050884	4.96250575	4.8507867	4.66272562	4.78878259	4.59735771	4.43463377	4.80200646	4.65749948
NONHSAG040605	5.93547193	5.7758695	5.98192611	6.06343673	5.87939174	5.97026921	5.92436622	5.72339121	5.52498269
NONHSAG040664	8.52775391	8.99025333	9.19422784	9.10486887	9.12678917	8.79489086	9.09415316	8.95243707	9.05997997
NONHSAG040668	4.44536144	4.43739819	4.44570174	4.3701966	4.31226498	3.88773113	4.37827863	4.44340424	4.47223021
NONHSAG040671	2.6279304	2.78564739	2.53650647	2.39393898	2.72663398	2.40825588	2.78325005	2.79708062	2.58755626
NONHSAG040680	4.17384566	3.52895553	3.39675038	3.72824567	3.50636368	3.26096395	3.86900711	3.46064538	3.4538332
NONHSAG040684	3.44268084	3.25191818	3.2291612	3.50996799	3.22665721	3.37019915	3.69123453	3.27860976	3.12012573
NONHSAG040703	5.11478781	4.62190723	4.81183612	4.50160147	4.71202415	4.46003445	4.49668875	4.62377173	4.7450938
NONHSAG040714	4.13280497	3.94056491	3.7409121	3.91071619	3.72400992	4.06569091	3.73413884	3.96019366	3.57974748
NONHSAG040766	3.81205871	3.83641941	3.61723498	3.55248285	3.60061561	3.81476525	3.86545455	3.87164178	3.78575487
NONHSAG040771	2.62905505	3.02710945	2.88633506	2.85623352	3.09844694	2.84872323	3.12255989	3.0313359	2.77696961
NONHSAG040781	6.7863776	6.48010285	6.53043816	6.34347241	6.51875339	6.4489113	6.36622286	6.0671951	6.53566886
NONHSAG040790	4.18117658	3.89794902	4.29542118	4.0190246	4.24059274	3.99987388	4.14455845	4.33796364	4.00674307
NONHSAG040792	3.85626154	4.02786674	3.55518275	3.66352015	3.74110041	3.89340918	4.35606502	3.86078925	3.48458376
NONHSAG040794	5.2806429	4.66483172	4.47167942	4.72511307	5.21900071	4.67634334	4.722785	4.82440371	4.80515525
NONHSAG040797	3.64668702	3.92567439	3.61410799	3.92254952	3.54375506	3.56287485	3.48885596	3.50851147	3.65341293
NONHSAG040826	6.63710634	5.54843765	5.59176197	5.68472425	5.23914616	5.24681772	6.0722897	5.80398924	5.27492694
NONHSAG040838	3.13015491	3.38640729	3.42476553	3.25729678	3.02407468	3.57490078	3.36895175	3.58093657	3.53715482

NONHSAG040876	4.82998219	4.25994821	4.49437419	4.17227629	4.23066766	4.6101752	4.28267109	4.19565279	4.3141557
NONHSAG040878	5.23627028	3.56261874	3.99694896	4.26085991	4.0007138	3.96929702	4.44755143	4.35661485	4.24200453
NONHSAG040919	2.22791977	2.09109666	2.13390084	2.19598818	2.13551603	1.99055891	2.32909236	2.17870977	2.11761677
NONHSAG040944	2.67135266	2.74290254	2.80813556	2.65532021	2.70628619	2.99267666	2.97045733	3.00817017	3.04924029
NONHSAG040959	3.31982289	2.56711893	2.76371579	2.52423952	2.81835158	2.83791497	2.73717637	2.96878197	2.72701969
NONHSAG040973	4.15911864	4.43981734	4.10243613	4.0755246	4.10206634	4.25539654	4.16736462	4.3509179	4.23241142
NONHSAG040975	3.09832379	3.34054785	3.09419304	3.19829117	3.19951177	3.66569296	3.08429215	3.02747198	3.12049674
NONHSAG040987	4.27918915	4.16671638	4.11852563	3.89165302	3.92749536	4.12694144	3.7034912	3.98538964	3.7930617
NONHSAG040990	2.52142773	2.17359903	2.36794832	2.09344153	2.4372457	2.32546367	2.20496649	2.23326312	2.32378829
NONHSAG040992	2.65807567	2.70464032	3.00907538	3.11598127	2.79633482	2.67054677	2.95266816	3.01483936	2.80629919
NONHSAG041024	2.40541726	2.23407899	2.58244614	2.57696789	2.39151797	2.44866561	2.38382791	2.29638261	2.40638446
NONHSAG041029	2.35866766	2.34945457	2.43003228	2.47109886	2.39762335	2.27761738	2.6378235	2.45725682	2.42749096
NONHSAG041052	5.88208882	4.98684349	5.26263966	5.31294018	5.09045639	4.73134147	5.74686456	4.87926638	5.11100589
NONHSAG041087	3.4398332	3.42855275	3.39121072	3.61252397	3.11634281	3.53274504	3.30027496	3.63470346	3.2282095
NONHSAG041094	9.38846308	8.87282784	8.98446079	8.92481335	8.82387553	8.74354116	8.93686076	8.88623842	8.96145119
NONHSAG041153	2.43015977	2.72777148	2.69260004	2.97358266	2.68322983	2.5505771	2.91475218	2.73401607	2.95890675
NONHSAG041157	3.47388845	3.30472518	3.30027496	3.34976211	3.39540085	3.10073861	3.566317	3.23582432	2.95876901
NONHSAG041202	2.92484971	2.95910233	2.96016804	2.95081445	2.96085714	2.97387562	2.97013919	3.00650122	2.86790058
NONHSAG041205	4.1142534	4.12089906	3.94477472	4.1573407	4.12380666	4.02514141	3.88791395	3.71825284	3.92041917
NONHSAG041207	4.53010287	4.42158106	4.01306244	4.15066199	4.26705362	4.4451761	4.21261239	4.40132808	4.14081753
NONHSAG041271	3.1783092	2.97388224	3.0371482	2.97909435	2.84377196	3.03809841	3.01966474	2.96443706	2.94410958
NONHSAG041280	6.55677462	6.21645791	6.7286193	6.30299715	6.43981004	6.11491275	6.42456668	6.2496758	6.4438365
NONHSAG041308	2.58885592	2.53082734	2.41927468	2.35348503	2.38694743	2.44248817	2.29528834	2.32058143	2.44813063
NONHSAG041310	4.42840828	4.44498555	4.57843113	4.51340492	4.49259008	4.64441377	4.54047824	4.55506455	4.48469461
NONHSAG041311	2.61215478	2.3804937	2.52758746	2.37322033	2.28424934	2.50821033	2.59310352	2.53171142	2.4335224
NONHSAG041331	8.32989533	8.50046678	8.8194282	8.62805136	8.77597877	8.60106828	8.83198236	8.72602359	8.81910803
NONHSAG041341	3.02334365	2.72990714	2.95014118	2.8054727	2.8007644	2.90407932	2.60515534	3.07881589	3.00100225
NONHSAG041345	2.67338917	2.50987102	2.6633841	2.75505215	2.49238289	2.58429828	2.64649913	2.71344895	2.42632544
NONHSAG041359	2.86101844	2.77987405	2.55306605	2.77635728	2.61536602	2.71835109	2.76222173	2.60518992	2.97118555
NONHSAG041393	2.49927515	1.93550837	2.34531848	2.18664728	2.32230363	2.20347966	2.19914844	2.04871751	2.05655608
NONHSAG041442	3.22584967	3.04219917	2.52741729	2.67503356	2.53197781	2.70319752	2.86219612	2.87644329	2.82024043
NONHSAG041473	5.97546698	5.3124264	6.3265207	5.86000923	6.23403469	5.88005261	6.47909924	6.20752864	6.45981314
NONHSAG041474	5.43821003	5.44879211	5.39490145	5.27903619	5.4449204	5.31233016	5.33563499	5.40971221	5.45339974
NONHSAG041475	3.86347521	3.56499083	3.66939973	3.66172092	3.67944516	3.67604826	3.69165184	3.66496947	3.62743741
NONHSAG041496	2.69305362	3.02602498	2.67019326	2.74372158	2.92725015	3.06114989	2.62686663	2.88899386	2.88195404
NONHSAG041512	4.4203504	4.77352725	4.82593397	4.65280162	4.89378303	4.77017196	4.97687152	4.53574936	5.00761636
NONHSAG041520	3.60788901	3.14479358	3.09289073	2.85623352	3.0759292	2.843562	3.1653309	3.07147149	3.12789074
NONHSAG041523	5.4781077	4.46202485	4.52555489	4.83340652	4.74961696	4.74122291	5.13131287	4.94238741	4.62016338
NONHSAG041551	5.53689666	5.50579401	4.94134277	5.00138882	4.97514214	5.3643596	5.42002333	5.67746998	5.10075663
NONHSAG041552	4.22451469	4.24736028	4.00007345	3.75709499	4.0283677	3.96090713	4.16849836	3.97912526	3.93148062
NONHSAG041579	5.17155132	5.90755977	5.396443	5.55333655	5.45078925	5.53119244	5.52465421	5.73965267	5.16296633
NONHSAG041596	10.11823548	10.73607488	10.96203218	11.19378937	10.68129276	10.82598983	10.75407803	10.72207706	10.67618472
NONHSAG041601	3.6313607	4.47341329	3.78822638	4.08175478	4.07246375	4.20619172	4.14253798	4.32051079	3.87415538
NONHSAG041618	4.79843689	4.82832676	4.60248351	4.48147008	4.62382685	5.05668776	4.52596497	4.57459745	4.62993953
NONHSAG041677	4.5264651	4.43418582	4.46003023	4.36757696	4.4168547	4.60051424	4.6448111	4.43817011	4.32341326
NONHSAG041723	4.49471162	3.99203503	3.85228815	3.8355701	3.64119115	4.06021912	4.04593344	3.80254967	3.51096615
NONHSAG041789	4.02828231	3.95667625	3.76626388	4.03367281	4.11038141	4.04665706	3.79595161	4.03952653	3.86461476
NONHSAG041804	2.44727456	2.39319439	2.22335791	2.33240217	2.1752798	2.31933855	2.36840449	2.30284486	2.42094726
NONHSAG041810	3.81958218	3.32519873	3.17765834	3.06471509	2.953755	3.27950255	3.23993276	3.20586974	3.1089489
NONHSAG041826	2.30850639	2.06803497	2.27168177	2.4109203	1.91238891	2.35218995	2.11938509	2.2183146	2.4818045
NONHSAG041886	2.73976	3.24689142	3.05001665	3.14062285	2.63815749	2.96252133	3.21222049	2.95414746	3.01285797
NONHSAG041903	4.15890446	4.06844954	3.94870468	3.89465202	3.94653874	3.95880371	3.79208004	4.0138547	3.82222825
NONHSAG041984	5.7021042	4.93666867	5.07566714	4.88250545	5.16727401	4.5410851	5.33882479	5.13774472	5.00470561
NONHSAG041987	3.26517257	3.15745816	3.12461207	3.09247241	2.87506272	3.39775053	3.13168213	3.28960834	3.04737637
NONHSAG041994	2.18768888	2.389136	2.40509013	2.35009123	2.45919511	2.40557925	2.51136232	2.53200528	2.24667393
NONHSAG041998	2.99166134	2.81676706	2.76526752	2.59786016	2.54033275	3.13724918	2.59595877	2.84271516	2.65477955
NONHSAG041999	3.08150082	3.45862186	2.93057474	3.11437682	3.09799739	3.64394312	3.68454735	3.51225354	3.15128023
NONHSAG042046	2.62558762	2.5162785	2.54613393	2.58893926	2.4925497	2.68572535	2.65560801	2.65919606	2.46370805
NONHSAG042051	5.09356915	5.11200654	5.00442042	4.97222967	5.17186547	4.96428936	5.10781666	5.05384073	5.06843944
NONHSAG042059	3.08787227	3.43669484	3.11176199	2.95808287	3.24289365	3.55026854	3.15821889	3.40636749	2.94744543
NONHSAG042103	3.3356332	3.28153891	3.16003748	3.26938457	3.3589076	3.35494756	3.29830056	3.22304742	3.28942552
NONHSAG042145	3.19523647	3.19694651	3.1388346	3.0470044	3.17640378	3.256817	3.23795314	3.25937859	3.13024317
NONHSAG042163	3.68863419	3.6411348	3.47710214	3.57158209	3.72099749	4.03560442	3.78064975	3.72013128	3.71276121
NONHSAG042168	2.64606321	2.31109709	2.44813063	2.65746863	2.50493938	2.44067513	2.41540008	2.64657991	2.62634642
NONHSAG042176	10.52619318	10.08511448	10.1826657	10.11506286	10.05683262	10.12494048	10.02592464	9.8288525	10.05489533
NONHSAG042182	4.24763518	4.18482854	4.22196674	4.28274582	3.87616708	4.68100382	4.48357839	4.059637	3.92251375
NONHSAG042192	3.14466823	3.28328546	3.12153303	3.03298179	3.08023807	3.18570346	3.16538442	3.12898968	3.06612397
NONHSAG042210	3.39384776	3.22294172	2.98201694	3.08180224	2.77987405	3.19304359	3.57214143	3.28604795	3.08599177
NONHSAG042250	3.62965734	3.51099277	3.42468017	3.44193847	3.47473771	3.45639875	3.59928307	3.70814167	3.47894004
NONHSAG042261	3.80233628	3.88868582	3.78638436	3.7836341	4.11198279	4.27101275	4.50139244	3.83021197	4.19097542
NONHSAG042272	4.15674518	4.03311699	4.1532391	4.23957659	4.12610256	4.11885238	4.11430996	4.22021132	4.19190817

NONHSAG042283	4.03867733	4.35954905	4.09537649	4.02259345	3.86023403	4.24861722	4.00494163	3.82663126	4.09472072
NONHSAG042315	5.23207291	4.94899919	4.68593419	4.75584275	4.55341382	4.94780375	4.93944469	4.94931219	4.55946133
NONHSAG042323	4.09808631	4.46508644	4.18509141	4.46558493	4.45714916	4.27090556	4.51265258	4.60605712	4.29166732
NONHSAG042369	4.1760103	4.50522545	3.72919439	4.02888735	3.98423526	4.52396588	4.63861652	4.32180594	4.52219845
NONHSAG042381	2.49461849	2.56855448	2.60337033	2.7232287	2.84499574	2.77377765	2.88187365	2.61904786	2.71335822
NONHSAG042418	4.91973132	4.95202392	5.12330973	4.80620653	4.51695954	4.80691693	4.86487223	5.27494379	5.01772395
NONHSAG042419	5.38969272	5.68175395	5.15398138	5.09730798	5.25886415	5.20247144	5.52474136	5.39317531	5.20677207
NONHSAG042424	4.86611243	5.00184776	4.57976042	4.51211323	4.82449114	4.93900733	4.89869936	5.0161481	4.56760036
NONHSAG042444	4.42233677	4.73598512	4.47519501	4.32412999	4.50856847	4.38631135	4.72937761	4.59725455	4.50321943
NONHSAG042451	4.1590141	4.35307139	4.08898403	4.07504219	3.98137932	4.15612893	4.07329914	3.89126146	3.7605011
NONHSAG042471	3.4447173	3.08207151	2.86755769	2.97253783	2.86972591	2.80033869	2.59005091	3.25634455	2.86371812
NONHSAG042535	2.50654	2.47295039	2.33240348	2.01920573	2.23133022	2.40035606	2.49670751	2.35013213	2.34762051
NONHSAG042546	3.18802582	3.24168928	3.41778794	3.01271568	3.33288376	3.31920272	3.074279	3.36919911	3.07932372
NONHSAG042558	4.23898892	4.25890514	3.84643002	3.95601487	4.02989131	3.96176974	3.98253587	3.91142436	3.99310564
NONHSAG042615	2.5191857	2.51403448	2.55477414	2.34013615	2.39329982	2.54011302	2.79036296	2.48280129	2.5385278
NONHSAG042636	4.83010583	4.92401183	4.57123624	4.41189211	4.32737901	4.3761379	4.37126677	4.80397523	4.43022696
NONHSAG042638	4.77171181	4.65911236	4.53069463	4.58569409	4.47912639	4.37157882	4.63893478	4.60806177	4.58659423
NONHSAG042726	4.58316087	4.2848935	4.44716066	4.08769869	4.44716066	4.56769647	4.40320788	4.41850635	4.20327837
NONHSAG042795	3.34862635	3.37413521	3.09079004	3.2094584	3.34841309	3.39401285	3.18636337	3.45097603	3.14087753
NONHSAG042834	3.56873511	3.40239192	3.51918192	3.57217417	3.49300196	3.7922018	3.69352772	3.63881465	3.51945452
NONHSAG042877	3.53480229	3.46450326	3.19144005	3.002665	2.96124656	3.00056956	3.29448066	3.10618006	2.99108146
NONHSAG042907	6.7351606	6.78076625	6.75813278	6.75615558	6.78721362	6.81246973	6.72309131	6.720889	6.80480611
NONHSAG042913	2.55805506	2.17737284	2.52912439	2.36230224	2.20562458	2.35413895	2.35225744	2.44372615	2.336771
NONHSAG042922	2.96551765	3.0940586	2.70532375	2.91779942	2.84540784	2.97196832	3.03787903	2.9561351	2.62223604
NONHSAG042934	3.28380195	3.5025681	3.54794212	3.91356201	3.65754965	3.90779749	3.45921712	3.52469646	3.47668668
NONHSAG042947	6.06513989	5.40091722	5.22516515	5.73267561	5.4240694	5.48800794	5.78418506	5.7888113	5.43406475
NONHSAG042959	2.92390977	3.60585631	3.27411289	3.46574406	3.21936265	3.5348988	3.25815755	3.4908353	3.39359219
NONHSAG042979	3.00342204	3.35414744	3.12987331	3.23711002	3.06947345	3.29766239	3.02637439	3.19429912	3.10618923
NONHSAG042982	2.73994535	2.77987405	2.66415588	2.58197148	2.70473389	2.81297728	2.67323847	2.73054341	2.40602763
LOC284379	6.02569302	4.61813378	4.57566367	4.60743063	4.44886349	4.47629109	4.34232054	4.77147511	4.0121287
EP300-AS1	3.78389887	5.48395522	5.18185364	5.29255654	5.53678471	5.27425999	5.26220646	5.20235918	4.75331553
DEPDC1-AS1	2.66500552	3.96129159	3.76227392	4.08882976	4.54389061	4.12944377	4.4904785	3.64708011	3.75166945
RUNDC3A-AS1	6.89970964	5.45441009	5.28612536	5.13959658	5.52142439	4.95985352	5.26686079	5.43661333	5.23610065
PTPRG-AS1	5.58238712	7.25299184	6.99511343	6.83876114	7.11275826	7.1912799	7.10728355	6.87445268	6.77611078
ELOVL2-AS1	6.35403804	7.63272569	7.35531003	7.59437339	7.84014343	7.54244569	7.49666269	7.30624566	7.1825285
NONHSAG016418	4.06092644	6.07274148	6.12456181	6.17884397	6.50779281	6.27409285	6.44887944	6.01997142	6.09430538
TMPO-AS1	4.67540752	6.39009333	6.16881563	6.32567109	6.5000807	6.56754639	6.54610309	6.19824254	6.06242328
NONHSAG042983	2.39855124	2.28870297	2.1941518	2.27752824	2.18066475	2.39280945	2.24565819	2.2308631	2.24413438
NONHSAG042995	3.26517257	4.20810903	3.35652023	3.8824961	3.8605419	3.73734286	4.1650051	3.78779527	3.33289424
NONHSAG043003	3.14313274	2.85800096	2.48172379	2.85765903	2.76290563	2.68832003	2.73446289	3.25865038	2.80008821
NONHSAG043014	3.08135732	3.0887301	2.83994412	2.94417916	2.99014602	3.06995591	3.01966474	3.03590805	3.03304021
NONHSAG043016	3.15809205	2.53625172	2.80011609	2.5392042	2.44564985	2.62670444	2.59215743	2.53326205	2.50508034
NONHSAG043027	4.70941161	4.16088038	4.33614929	4.4381698	4.58383647	4.47840655	4.3756956	4.10157501	4.47777442
NONHSAG043033	3.24008582	3.46027793	3.27755268	3.1618992	3.28153891	3.355064	3.673108	3.02751481	3.40149185
NONHSAG043043	6.90778573	6.98183499	6.9840848	6.92056593	6.95558457	6.83046512	6.73937311	6.87454512	7.04464137
NONHSAG043047	5.51808356	5.19416336	4.98450643	5.03729693	5.19090462	5.01771583	5.10315022	5.31438551	5.17989172
NONHSAG043071	5.06897362	5.33694327	4.77562895	5.0073713	4.85509198	5.37726787	5.0703702	5.11975117	4.7735036
NONHSAG043074	4.29772606	4.3244456	4.15635406	4.22285148	4.17335001	4.12171974	4.54620246	4.57445039	3.79092717
NONHSAG043077	4.22870982	4.32069158	4.01875387	4.2005662	4.18564585	4.13587765	3.91659019	4.24156249	4.05941036
NONHSAG043093	3.20359995	3.20444067	3.36704995	3.22302567	3.17345296	3.54448481	3.43586934	3.48250608	3.1797147
NONHSAG043129	3.55595005	3.66188645	3.41423366	3.43038526	3.22236246	3.31301805	3.63283631	3.38026204	3.44393653
NONHSAG043135	5.80370644	6.07840572	6.08313133	6.03728812	5.71420959	5.93341785	5.79796653	6.09258868	5.69584983
NONHSAG043144	3.35018669	3.3125885	3.41976377	3.69421019	3.56328802	3.85043077	3.425488	3.5881519	3.6110632
NONHSAG043195	3.1095056	3.26591216	2.94374802	3.03194416	3.17286316	3.15262608	3.23307457	3.28135277	3.2967269
NONHSAG043203	5.68075134	6.01070563	5.69300075	6.11961593	6.15181825	5.98338546	5.99247803	6.01075836	6.10263511
NONHSAG043218	2.24427288	2.36010458	2.29607366	2.08507406	2.37155371	2.37260005	2.31027264	2.1206855	2.26759255
NONHSAG043252	2.50668148	2.49823989	2.36953369	2.40201767	2.84641615	2.38763855	2.49332706	2.32166731	2.26586926
NONHSAG043277	4.46560453	4.38157326	4.18405325	4.50123983	4.10871943	4.21511293	4.5826855	4.74713992	4.22124214
NONHSAG043281	2.40566788	2.81918054	2.48499416	2.49248042	2.71751136	2.56344299	2.47614115	2.5235903	2.40128327
NONHSAG043292	2.97845981	2.84982957	2.71834322	2.78797916	2.75081708	2.89268378	2.93040707	2.91114218	2.84298064
NONHSAG043304	2.88135683	3.0113318	2.82526816	2.85095525	2.83791899	2.89164695	2.95651998	2.61904786	2.76404754
NONHSAG043313	3.6203963	3.6203205	3.45745723	3.7504117	3.57896159	3.61828459	3.46889072	3.80538895	3.29793703
NONHSAG043379	6.27087452	6.25334894	6.23492299	6.39462655	6.01087751	6.1751554	6.25499378	6.23938658	6.02419003
NONHSAG043614	3.8852377	3.33765716	3.59020038	3.51762575	3.13127378	3.42996475	3.40020739	3.26546237	3.20920515
NONHSAG043616	4.88305041	5.03380684	4.5907713	4.87683948	4.65441978	5.03621888	4.7447892	4.93068645	4.53298182
NONHSAG043628	9.08070723	8.66815731	8.95044541	8.64346212	8.83908543	8.90082492	8.71111863	8.8441857	8.91438667
NONHSAG043629	8.0144188	7.64753981	7.86455251	7.65071151	7.83107255	7.70100584	7.7850258	7.79225205	7.89640845
NONHSAG043634	2.27871762	2.44752264	2.41000544	2.32639033	2.25757451	2.50525978	2.37315526	2.59491806	2.53049801
NONHSAG043637	6.72683318	6.56206454	6.72109674	6.63882141	6.57607353	6.58834129	6.56856997	6.71215063	6.87960164
NONHSAG043646	5.89425628	5.5863157	5.73403316	5.55886211	5.73570755	5.7477598	5.5487646	5.42864815	5.58337925
NONHSAG043677	2.7348408	2.90888952	2.93569781	3.000901	2.97368761	2.47065543	2.73894917	2.66690271	2.99741263

NONHSAG043699	2.87585224	2.87385044	2.79503409	2.88591424	3.01228134	3.07245464	2.68775215	2.86899723	2.90303134
NONHSAG043706	5.59922302	4.62751037	4.35229262	4.70331229	4.43473442	4.5020413	5.36020568	3.87625643	4.19359007
NONHSAG043726	2.56546824	2.64391564	2.81846449	2.6674305	2.71678753	2.82543504	2.6591746	2.76364196	2.54949867
NONHSAG043748	5.61012854	5.2374458	5.18084972	5.02075471	4.96098732	5.58756042	4.66056396	4.91938602	5.11574054
NONHSAG043765	6.29616358	7.06124027	6.70988644	6.82635425	6.7042907	6.96000181	6.84080833	7.06979851	6.47652925
NONHSAG043790	6.08829217	5.47326627	5.71276635	5.71086131	5.65229012	5.61080644	5.80527338	5.64009665	5.63012299
NONHSAG043821	6.12592772	5.51089733	5.52539966	5.77583161	5.42627096	5.67979343	5.68942603	5.21578535	5.69489649
NONHSAG043825	3.06826296	3.09055941	2.9130983	3.00770602	3.0355664	2.92970952	2.88887939	2.97599708	3.05684565
NONHSAG043835	3.36720113	3.09041536	3.18850886	3.18625748	2.93262889	3.16831428	3.18287823	3.27909568	3.12276919
NONHSAG043845	4.57963821	4.89750297	4.91789212	4.50957395	4.70255543	4.97582009	4.98485504	4.65854846	4.41704334
NONHSAG043849	4.37535834	4.49827364	4.28220846	4.26856536	4.03523957	4.46449053	4.29431498	4.25522795	4.01509228
NONHSAG043854	4.22078496	3.95023688	3.91504769	3.99927622	3.93682909	4.14061723	4.16503044	4.00230996	3.82291882
NONHSAG043856	5.11712176	5.23831492	4.94551969	4.9026049	4.66129442	5.38170552	4.86925786	5.24639064	4.88019655
NONHSAG043865	3.32495129	3.55062598	3.42010153	3.57875283	3.42723503	3.27201522	3.45261161	3.44878509	3.31777278
NONHSAG043905	8.81428185	9.62608124	9.94489338	9.8892852	10.03615376	9.71364959	10.21489803	9.91146677	9.6917496
NONHSAG043910	3.47896191	3.89671845	3.57335978	3.89325328	3.58723299	3.77036851	4.00447454	3.77914769	3.49989214
NONHSAG043913	3.24864011	2.98622559	2.84135503	2.84062176	2.93351505	2.98699279	2.79535144	2.92470111	2.72807208
NONHSAG043916	3.63538962	3.61837881	3.30036875	3.39729551	3.37529487	3.32753568	3.51833579	3.45036786	3.6264687
NONHSAG043927	3.12289299	3.7327337	3.21470279	3.37939453	3.61308283	3.57182235	4.02705941	3.63817435	3.56689415
NONHSAG043945	3.01448436	3.09817449	2.72344536	3.09325165	2.74861058	3.32532396	2.98980415	3.05565547	2.97084321
NONHSAG043980	3.93149333	4.35889859	4.42185115	4.11692957	4.04417036	4.23163862	4.02012801	4.25580896	4.16064474
NONHSAG043986	3.07566707	3.45891206	3.36511701	3.36662613	3.19299786	3.06141238	3.02417495	3.36300473	3.28007465
NONHSAG044024	2.58401076	2.23020847	2.35644064	2.06029576	2.13994487	2.0826938	2.32625571	2.26787609	2.24882245
NONHSAG044064	2.79792962	3.47291439	2.72481556	2.97704888	2.85424856	2.82206612	2.98128068	3.15678138	2.89265337
NONHSAG044066	2.79202375	2.8417443	2.79972524	2.90401758	2.64737333	2.87424121	2.90861805	2.46553263	2.77662744
NONHSAG044070	3.69795993	3.44245241	3.09298427	3.35510476	3.34079354	3.26841383	3.15328256	3.31827369	3.32969729
NONHSAG044072	2.68788397	2.57800804	2.44842656	2.66563516	2.37673386	2.49462015	2.54214353	2.66704328	2.53438962
NONHSAG044100	7.71058042	6.93418134	7.42483751	6.96916101	7.20820259	7.10674784	6.59249765	6.26522079	6.74880019
NONHSAG044111	2.95815197	2.40959774	2.6853445	2.91884171	2.68353872	3.0052466	2.78980513	2.77934808	2.66690028
NONHSAG044194	3.74016316	3.67688611	3.39037329	3.71389324	3.57432216	3.68035909	3.91455224	3.89609409	3.61959009
NONHSAG044196	4.50677618	4.68300609	4.51841083	4.91034927	4.70073172	4.66334704	4.7605423	4.85844301	4.69134705
NONHSAG044199	3.64040638	3.35791095	3.17035117	3.50419595	3.40129699	3.29375791	3.2959745	3.46541317	3.39990856
NONHSAG044203	4.5477188	4.61376692	4.50512397	4.37986191	4.83170564	4.87640682	4.42279367	4.55026653	4.27086383
NONHSAG044217	5.2966338	4.11116712	4.42183888	4.16404821	4.22514243	4.00526475	4.25387954	4.32819763	4.65188334
NONHSAG044226	3.09690288	3.16870016	2.73393999	2.89510858	3.02352301	2.97948906	3.11083427	3.19903576	2.8870975
NONHSAG044243	3.84386951	3.53233868	3.54881853	3.75412014	3.84897097	3.95114185	3.58813157	3.95834888	3.66981792
NONHSAG044245	3.66074694	3.37093118	3.39892965	3.63892297	3.59906809	3.32663623	3.57050142	3.73095406	3.3468811
NONHSAG044258	3.19006894	2.9506339	3.66307474	3.30185514	3.58764143	3.12671934	3.42107634	3.19978787	3.66307474
NONHSAG044264	4.03219452	3.83989241	4.20968571	4.54648302	3.91059378	4.12297136	4.13804538	3.9803424	3.9985362
NONHSAG044265	2.62757634	2.35698073	2.34032954	2.29164345	2.22877478	2.40320829	2.37211392	2.37642972	2.46153306
NONHSAG044266	2.42305134	2.69389422	2.50633491	2.5014215	2.57280623	2.55068291	2.43471409	2.7779521	2.4765912
NONHSAG044270	2.90403432	3.20395772	3.08074398	3.23328365	3.04518336	3.09395881	2.9191008	2.89268871	2.99427201
NONHSAG044283	7.20273454	7.83613273	7.55280113	7.97959008	7.50625756	7.28386483	8.0982565	7.72724329	7.59033763
NONHSAG044290	2.38027764	2.51295148	2.44047919	2.33594896	2.42433051	2.13258871	2.29779064	2.33727735	2.37731069
NONHSAG044304	4.1801333	3.40076965	3.39325683	3.70891885	3.56121252	3.70656025	3.78775255	3.76483511	3.54250916
NONHSAG044336	3.70593214	4.31039031	3.98775458	3.82176412	4.0808759	4.45577813	4.24959809	4.11240853	3.94117748
NONHSAG044342	3.46478467	3.46190441	3.48933433	3.52002542	3.38868533	3.47000387	3.34380385	3.44314989	3.39038037
NONHSAG044349	5.37299106	5.3167324	5.27810811	5.33846385	5.136531	5.75045295	5.69545832	5.76136811	4.92668534
NONHSAG044369	2.42846293	2.33874241	2.31178299	2.27440137	2.20331952	2.62173594	2.59595877	2.6003874	2.31452443
NONHSAG044447	2.23655548	2.19342985	2.16969318	2.01710725	2.05887631	2.07988728	2.08824072	2.2173882	2.18748799
NONHSAG044503	2.48260801	2.45354161	2.55477414	2.33124262	2.28118456	2.40357643	2.37901805	2.61045955	2.33637482
NONHSAG044507	2.09792751	1.89996814	1.96237128	2.27454987	2.09011524	1.97699354	2.22360392	2.06033188	2.12859088
NONHSAG044512	2.88432033	2.55321607	2.79024886	2.77287136	2.7309267	2.67455715	2.77377998	2.71006593	2.64774241
NONHSAG044540	3.26307514	2.65455531	2.73946413	2.98808898	2.63892086	2.86288186	2.92124992	2.69987696	2.78587241
NONHSAG044561	2.7348408	2.75199829	3.19855385	2.8938545	2.79119495	2.8569617	3.18012375	2.6459549	2.87515557
NONHSAG044585	2.52253616	2.49940043	2.3270144	2.35459757	2.17043052	2.54432567	2.48520927	2.43854651	2.26133834
NONHSAG044626	3.24542635	2.80978172	2.5691301	2.82339344	2.58296098	2.99871983	2.82765248	2.78741408	2.8170116
NONHSAG044627	2.96225115	2.98718982	2.66901612	2.73921525	2.80944696	2.86005666	2.32520326	2.74769676	2.94642517
NONHSAG044671	3.11155872	3.29163348	2.94374802	3.00242115	3.29026596	2.85339938	3.02559214	3.03739561	2.87559061
NONHSAG044686	2.72513034	2.66755936	2.65226305	2.54775752	2.5142386	2.46012713	2.63622405	2.64709299	2.66177692
NONHSAG044742	6.63503919	6.38354022	6.57917724	6.14849473	6.19923836	6.24000319	6.26265952	6.20012121	6.14077469
NONHSAG044767	3.7237116	3.57296995	3.58257594	3.99594617	3.5363733	3.70167192	3.6236026	3.89250717	3.18303684
NONHSAG044790	3.72309167	3.28177341	3.22492589	3.27384523	3.21943436	3.13516633	3.30027496	3.32366015	3.17184049
NONHSAG044806	5.95718177	4.52308647	4.54335723	4.64369746	4.46420722	4.35038346	5.37856984	4.89455361	4.59003493
NONHSAG044810	6.58727856	6.29732931	6.34742896	6.73515492	6.46716587	6.71908852	6.29732987	6.62656031	6.51147685
NONHSAG044811	2.71262795	2.63230642	2.81596901	2.64861397	2.78070814	2.96806099	2.62446357	2.67978427	2.53237025
NONHSAG044815	2.21117122	2.05385813	2.03120984	1.9232611	2.0804708	2.10410589	1.98631437	2.01799124	2.04325386
NONHSAG044846	4.49792237	4.58678887	4.501602	4.39966798	4.45526663	4.40403506	4.31031546	4.45942232	4.30941967
NONHSAG044851	2.99484468	2.8417443	2.91052206	2.90740199	2.78649177	3.11898682	3.0280135	2.90360083	2.77731079
NONHSAG044892	4.2754179	4.09451001	4.09975782	4.11209988	4.15585527	4.52996926	4.02420619	4.06346988	3.9378507
NONHSAG044894	4.33831047	4.79938183	4.16066487	4.72767187	4.45276708	4.69348339	4.63250436	4.68423766	4.4017988

NONHSAG044918	2.40935151	2.48495585	2.77167963	3.12873025	2.71835109	2.69110089	2.63539582	2.69481259	2.66175049
NONHSAG044920	2.84955111	3.01444394	3.07939161	2.90398756	3.07849821	3.18762456	3.22381181	2.92616705	3.11651035
NONHSAG044929	6.11322247	5.80208255	5.68799268	5.57593516	5.86652812	5.48384688	5.84977028	5.57140229	5.9197182
NONHSAG044999	3.02219351	3.00565032	3.05707888	2.99342434	3.05277051	3.16481447	2.9755508	3.13872848	2.92073898
NONHSAG045020	2.65100745	2.57311142	2.40010714	2.5005584	2.27668862	2.5478076	2.72225095	2.51299857	2.49402309
NONHSAG045068	2.71282795	2.54321594	2.36978126	2.5334805	2.259103	2.39795851	2.59400425	2.55443643	2.29674596
NONHSAG045080	3.30039844	3.45542228	3.17581989	3.51889582	2.84753034	3.23699686	3.30502532	3.40401125	3.24499885
NONHSAG045081	4.17590505	4.11283764	3.53530091	3.79497036	3.58711578	3.65911329	3.92690497	3.71846803	3.7018127
NONHSAG045085	4.84824356	5.09928521	5.0208984	4.92733715	4.88470761	5.20695235	4.81206036	5.15709847	4.78211183
NONHSAG045101	4.2733607	4.00130265	3.96057093	4.14590605	3.96095016	4.01433692	4.32326918	4.43652394	4.04438459
NONHSAG045116	2.89916163	3.07714272	2.75628587	2.71449701	2.86825617	2.79007447	2.86479626	2.77185106	2.66618917
NONHSAG045138	3.34809207	3.21979343	3.38324129	3.27124305	3.45283413	3.38226107	3.42686783	3.44172031	3.27512558
NONHSAG045167	3.35119428	3.75547016	3.4168887	3.70448076	3.63793445	3.57218816	3.55874258	3.90450683	3.69763336
NONHSAG045192	3.5442335	3.46794677	3.39718225	3.06826491	3.52774379	3.64394312	3.09454151	3.51580135	3.17375008
NONHSAG045208	4.02393115	4.0369379	4.72577083	4.09050027	4.44003752	4.3525351	4.18073082	3.65577789	4.41956116
NONHSAG045235	6.45506166	6.45206528	6.126428	6.29951583	6.38143012	6.48005799	6.98084736	6.50336211	6.01620054
NONHSAG045258	8.09733259	7.74647409	7.43610594	8.05282278	7.63542997	7.57296474	7.95430516	7.76177626	7.60348972
NONHSAG045265	4.59928681	5.00227754	4.23609115	4.4664596	4.49640374	4.7579835	4.54897572	4.77739478	4.24958732
NONHSAG045281	2.81679651	3.20479117	3.10755327	2.97716611	3.09623641	3.19258297	2.91500959	3.06761962	2.82199556
NONHSAG045316	2.03020328	2.38150363	2.31063915	2.31589087	2.23491614	2.4792986	2.31133219	2.22982991	2.19982234
NONHSAG045329	2.18507215	2.49898114	2.37174907	2.26252032	2.25741181	2.45680481	2.44435707	2.31850576	2.34607632
NONHSAG045333	4.74400019	4.91858642	5.47618783	5.17723962	5.12006092	4.77449303	5.03711499	4.9709564	5.05740286
NONHSAG045338	2.79768679	2.68400852	2.5475756	2.37467428	2.54518337	2.46926785	2.82030612	2.43554089	2.47526077
NONHSAG045350	3.07435159	2.64253458	2.58193247	2.56732668	2.65331155	2.59185452	2.81445603	2.68502688	2.61750758
NONHSAG045363	5.32681512	5.45078184	5.21356514	5.20756923	5.15089453	5.42958944	5.37740898	5.61494866	5.29145642
NONHSAG045364	4.68609325	4.03017707	4.15428628	3.83324793	4.08695568	4.17487406	4.22100044	4.29219816	4.18749092
NONHSAG045389	2.68240179	2.68414003	2.65855406	2.56704005	2.84855935	2.70114437	2.55338697	2.62503091	2.86962309
NONHSAG045403	6.05017173	5.64194795	5.60740004	5.55878821	5.83963684	5.66409573	5.50583337	5.65514244	5.64119313
NONHSAG045405	4.70540461	6.23624372	6.18889658	6.40372886	6.28370952	5.89802463	6.6707297	5.83026228	5.75975237
NONHSAG045408	4.58669973	4.5214155	4.26532409	4.30530498	4.39471214	4.666066	4.40239889	4.57102476	4.19922995
NONHSAG045419	4.52129567	4.26832178	4.16984138	3.93072821	4.08389587	4.31043206	4.66311986	4.24534858	3.87062618
NONHSAG045425	3.49262687	3.78037376	3.37018935	3.55129927	3.37953395	3.79786018	3.44787194	3.55286093	3.34568982
NONHSAG045429	3.37945797	3.7991703	3.36953917	3.44077615	3.54916212	3.41634351	3.41134421	3.73730778	3.40575468
NONHSAG045445	3.58334286	3.71697962	3.41646346	3.55832372	3.49204569	3.62995813	3.83499696	3.70303478	3.38069342
NONHSAG045449	2.27264611	2.34892744	2.32527513	2.30442038	2.37571304	2.30612356	2.30083771	2.40679037	2.31719134
NONHSAG045463	3.65164785	3.39269754	3.44712371	2.97665435	3.25723135	3.5514115	2.99252102	3.22517749	3.25073616
NONHSAG045467	4.9988024	4.93002402	4.75440848	4.77047	4.76861345	4.91522826	4.72757727	4.92582917	4.64742006
NONHSAG045472	3.60418917	3.69935873	3.41444685	3.53530091	3.35164137	3.74477268	3.45768665	3.7469691	3.40531331
NONHSAG045505	3.93186801	3.69149813	3.8564742	3.64852517	3.58978513	4.16594502	4.15515509	3.8568097	3.56519702
NONHSAG045512	4.37521968	3.82825705	3.956055	3.62143016	3.66871005	3.73734286	3.71268455	3.84585606	3.59530004
NONHSAG045517	3.30887202	3.17113493	2.83121904	2.91155039	3.04793222	3.08610286	3.74171175	3.42287738	3.0101533
NONHSAG046716	5.51832433	5.63777888	5.2612688	5.23008113	5.19281488	5.7697792	5.59698007	5.44053827	5.35280413
NONHSAG046750	5.50580935	5.31206906	5.70680914	5.56036097	5.60869948	5.54241566	5.5592287	5.49104092	5.40955267
NONHSAG046781	5.12486613	5.31559424	5.15396781	5.20480365	5.16111116	5.42102554	5.4540029	5.38723539	5.09769411
NONHSAG046784	4.9554726	5.15242974	4.95133027	4.89969028	5.11858527	4.88092579	5.2153578	5.28369253	4.90160503
NONHSAG046797	4.73591628	4.49037543	4.44850046	4.3305277	4.38708592	4.57589849	4.52146297	4.36207188	4.46225517
NONHSAG046806	6.47148981	6.36374321	6.42173679	6.40455251	6.53143588	6.373389	6.40824865	6.37743722	6.29098743
NONHSAG046817	3.71008048	3.90208827	4.03212235	3.88704118	4.04417036	4.48988873	4.07261272	4.04827727	3.97136886
NONHSAG046838	3.68554041	4.30247655	4.10679319	4.0280918	4.12163653	4.0533519	4.28772015	4.25433073	4.03935023
NONHSAG046851	4.26356774	4.3100041	4.06452277	4.31167949	4.04220206	4.13922263	4.05292295	4.37908538	4.03935023
NONHSAG046903	4.45700605	4.62100033	4.34619883	4.36572003	4.75594534	4.68580779	4.62268081	4.50594832	4.29826335
NONHSAG046927	2.63744224	3.02543586	2.76238278	2.91037434	2.75309569	3.01220142	3.01966474	3.02201588	2.85830492
NONHSAG046961	3.45078575	3.2847779	2.8092924	2.92477548	3.13710474	3.18751773	2.84080634	2.86258373	2.72682718
NONHSAG047021	2.55081279	2.6860449	2.40278742	2.51720843	2.45249589	2.48670338	2.51533643	2.3892288	2.33025287
NONHSAG047026	4.73597891	4.59692519	4.72099739	4.90552502	4.68808493	4.72300143	4.56590779	4.82868994	4.47618257
NONHSAG047060	2.35742286	2.67333989	2.71835109	2.64908431	2.71108205	2.84581883	2.90826916	3.09953442	2.61999469
NONHSAG047082	3.7850139	3.57015315	3.52711862	3.56697851	3.56721019	3.83709263	3.66987352	3.73432128	3.38258682
NONHSAG047088	2.57671125	2.40242021	2.41708495	2.33714853	2.39120105	2.53437796	2.58414129	2.26464155	2.56371353
NONHSAG047097	4.27158653	4.42982339	5.40213005	4.49980717	4.98677327	4.32264227	4.65356199	4.74188408	4.92680951
NONHSAG047101	3.02638618	2.62949655	2.78552837	2.78814321	2.77901122	2.80370703	2.75453833	2.72968236	2.86349369
NONHSAG047126	2.63749343	2.53780842	2.39565096	2.31880689	2.38528405	2.9144431	2.5865549	2.4918066	2.49377169
NONHSAG047149	3.56668277	4.00955249	3.98872628	3.9608324	3.9487386	3.73734286	3.92818889	3.90234059	4.00083841
NONHSAG047152	2.99460441	2.78836646	2.63177567	2.76568027	3.0175152	3.04212846	2.82773893	3.10807753	2.73449925
NONHSAG047169	2.86101844	2.66562614	2.5394108	2.49451917	2.48527283	2.72054294	2.5870732	2.84802152	2.76485382
NONHSAG047175	3.57626228	3.48713229	3.67288604	3.7165397	3.6089161	3.50935199	3.68468823	3.38802998	3.46627512
NONHSAG047181	3.52797845	2.97488002	2.94324359	2.99643773	2.97218129	3.16263053	3.02812856	2.82857324	3.18902984
NONHSAG047192	5.20573263	4.44478377	4.30166559	4.41668832	4.76302245	4.39790694	4.52519699	4.31552934	4.29419735
NONHSAG047193	3.06897717	3.01307402	3.21171059	3.39786275	3.15419875	3.40902886	3.20107031	3.09087004	3.22582763
NONHSAG047194	3.49378219	3.04816014	3.2155141	3.27929407	3.30109313	3.21670839	3.1416601	3.46099961	3.00276398
NONHSAG047208	2.67416939	2.72675346	2.62812288	2.44612863	2.58245351	2.74228564	2.82129073	2.60400357	2.62711074
NONHSAG047221	3.32920708	3.16137696	3.52319185	3.48043191	3.61583741	3.42732043	3.30930282	3.65434878	3.50491022

NONHSAG047229	4.86851308	4.95052316	4.61896303	4.69795807	4.66900036	4.76553975	5.02603302	4.95984129	4.6935055
NONHSAG047232	4.08586811	3.7554632	3.8896848	3.97670335	3.97762504	3.70520418	3.84802732	3.89285082	4.06474482
NONHSAG047253	3.57750689	3.03763651	3.00769684	3.05577298	3.29692088	3.2301436	3.36475026	3.01900163	3.35121194
NONHSAG047257	3.52383208	3.3444532	3.79288791	3.66619085	3.32260708	3.32136154	4.22153188	3.47207565	2.94618385
NONHSAG047259	3.86521372	3.43143352	3.56121836	3.49628384	3.63171016	3.33123446	3.35777098	3.40578124	3.45297108
NONHSAG047273	6.43240998	6.40756647	5.93445115	5.79161468	5.86630155	6.24486846	5.94990802	6.00945105	5.67859015
NONHSAG047282	2.72930727	2.77372993	2.90114469	2.97180051	2.81676589	2.99208623	2.96545604	2.93185911	2.60577181
NONHSAG047295	3.16318125	3.27596423	2.84121626	3.32076472	3.04595336	2.76347922	3.00424727	3.03014575	3.04869138
NONHSAG047314	4.57832029	4.16862125	4.11512069	4.03067759	3.80849131	4.37617509	4.40959918	4.30806578	4.19109417
NONHSAG047336	5.23749996	4.93760913	5.40887093	5.00871592	5.24380041	4.98651674	5.36509185	5.07195509	5.21951092
NONHSAG047348	2.40243248	2.80337983	2.71540666	2.73476508	2.83750041	2.66504093	2.50719565	2.74115448	2.84129918
NONHSAG047356	2.82244904	2.88018594	2.72751498	2.58351459	3.08206955	2.706938	2.58169927	2.72978898	2.75400213
NONHSAG047375	3.40905862	3.60501018	2.93921717	3.35866834	3.17829457	3.4038916	3.44600634	3.18587469	3.00129799
NONHSAG047399	2.65111044	2.51161416	2.64260694	2.68357464	2.58019497	2.46813442	2.49812382	2.59711529	2.65627799
NONHSAG047416	2.8281596	2.91116333	2.72794493	2.45608811	2.54899151	2.81296673	2.52539044	2.68563606	2.88098211
NONHSAG047425	2.7087128	2.47591675	2.25421589	2.26396749	2.25329529	2.42128893	2.68005138	2.35426812	2.23122752
NONHSAG047431	2.75681193	2.88364872	2.82233028	2.78560951	2.81741277	2.82599647	2.8942798	2.59699857	2.83720516
NONHSAG047443	3.07324612	2.93782416	2.92781284	2.78199101	2.82556737	3.1722251	2.84037438	3.22831996	2.8162252
NONHSAG047447	2.78611022	2.45075555	2.13898004	2.19598325	2.1981063	2.54973275	2.30286691	2.35145571	2.2172603
NONHSAG047469	3.76490699	3.79519382	4.08480866	4.22707429	3.84988823	4.04838722	4.34405506	3.9101164	4.14726275
NONHSAG047503	2.44460922	2.3892325	2.18271244	2.50333262	2.34745666	2.39824293	2.30058677	2.31463448	2.27177633
NONHSAG047517	6.48643355	6.34045623	6.49740831	6.27975042	6.41521646	6.3644394	6.27227751	6.33920763	6.36342692
NONHSAG047524	3.56157411	3.17173118	2.97706264	2.85742102	3.009694	3.1553302	2.91357722	2.99763119	3.12675878
NONHSAG047536	2.37543339	2.10342976	2.193139	2.28930099	2.34199427	2.23446328	2.31803012	2.12617953	2.27474792
NONHSAG047547	6.29799631	6.04164773	6.59275115	6.3373704	6.38512336	6.2735127	6.18619399	6.09140864	6.17393724
NONHSAG047553	4.53405629	4.72987313	4.43535204	4.41771423	4.15936619	4.55828976	4.30350148	4.5439605	4.40561142
NONHSAG047570	5.1044918	4.59884952	4.67618949	4.50511351	4.68204058	4.57523767	4.31562916	4.43770057	4.82787984
NONHSAG047611	4.24822758	3.90672112	3.98291991	4.23280065	3.88060878	4.25630021	4.08097149	4.05894042	4.03358944
NONHSAG047665	4.30636295	4.25595635	4.22785655	4.07309309	4.0029764	3.77657259	4.26896482	4.09432061	4.07399725
NONHSAG047674	2.83493461	2.92586903	2.67935961	2.87128892	2.31599355	2.64843143	2.95829218	2.85727061	2.4823264
NONHSAG047730	2.73291534	2.83138479	2.89095994	2.62360678	2.94516819	2.7326648	2.73697281	2.86625097	2.62173594
NONHSAG047747	3.64051029	3.67168102	3.23268762	3.24521764	3.66096369	3.55291079	3.57184138	3.49075602	3.6957567
NONHSAG047777	4.2604467	5.03724258	5.36924587	5.26816171	5.01245747	4.81967525	5.03133553	4.89047712	4.89696398
NONHSAG047807	2.91294951	2.82155831	3.11176199	2.50257564	2.72121587	2.85813515	2.44072547	2.93090885	2.46757446
NONHSAG047837	2.62676734	2.44648405	2.48700898	2.46781607	2.20644124	3.26872695	2.57547198	2.39114591	2.53014202
NONHSAG047842	6.80141363	6.5621867	7.02389925	6.84958519	6.96691925	6.47503741	6.80525552	6.57945474	6.83799267
NONHSAG047845	4.54014994	4.31488917	4.37218322	4.34381197	4.70605733	4.48204723	3.838512	4.25447056	4.7935632
NONHSAG047847	5.01150558	5.68422881	5.31164248	5.37423572	5.03512206	5.58645445	5.28372343	5.41181234	5.1933566
NONHSAG047907	8.87638058	8.96316817	9.15289645	8.84120095	8.72643845	9.04920089	8.54091833	8.93597538	8.76581944
NONHSAG048011	4.58382137	4.59357677	4.44517961	4.36272194	4.59515541	4.64080403	4.59941244	4.71950978	4.47310462
NONHSAG048092	2.61955269	2.71058323	2.40622032	2.21302154	2.34020421	2.44358797	2.53825888	2.44741767	2.3259299
NONHSAG048098	2.31942542	2.25041081	2.46637943	2.31749168	2.45007513	2.47607324	2.43471409	2.61904786	2.44813063
NONHSAG048120	3.32779543	3.3746619	3.4268384	3.69662844	3.13360071	3.90959416	3.25509347	3.46099961	3.31922348
NONHSAG048123	2.83615174	2.89734735	2.55477414	2.82893723	2.66779712	3.12394309	2.76604049	2.98151667	2.61444168
NONHSAG048148	2.39966855	2.22517449	2.14160444	2.21302154	2.23028232	2.28220871	2.18869222	2.23193013	2.13508602
NONHSAG048155	4.43703472	4.62637036	4.24706406	3.99718938	3.79171041	4.46373122	4.10987204	4.17358094	4.07743833
NONHSAG048166	2.50047716	2.97588289	2.58626922	2.89007043	2.78010537	3.07732857	3.06670199	2.77862802	2.69647186
NONHSAG048202	2.84679196	3.0604098	3.00315575	3.04328959	2.81692196	3.05388147	2.9468144	2.99861325	2.9553853
NONHSAG048386	6.44190549	6.32186483	5.87972977	5.95264825	6.15975059	6.06821461	6.1375141	6.13449155	5.87085377
NONHSAG048397	8.52735436	7.7668118	7.81058192	7.78525802	7.71187749	7.57973343	7.92340894	7.64192374	8.10152466
NONHSAG048404	4.85544003	4.77680369	4.77441898	4.65660889	4.75586723	4.91673463	5.21830491	4.77025422	4.56435868
NONHSAG048405	2.30726446	2.36522555	2.28171224	2.3027445	2.33996715	2.3254909	2.20880231	2.12785238	2.53910242
NONHSAG048439	3.03224041	3.23219032	2.97158208	2.79316542	2.9207631	3.19904067	3.11239028	2.93960744	2.79530161
NONHSAG048458	2.7348408	2.45425851	2.63696788	2.43099174	2.50482787	2.31441083	2.43699841	2.3486513	2.49457413
NONHSAG048471	6.49993788	4.86982605	5.83524456	5.61794945	5.64679617	5.24348085	6.13524976	5.28698578	5.24348085
NONHSAG048506	3.0418815	3.04438747	2.71835109	2.9235197	3.09287857	3.21670839	3.14107724	2.97067373	2.84095011
NONHSAG048520	3.96145744	4.18202787	4.10656485	4.44742603	4.56539418	4.28428422	3.52644203	3.93030271	4.26737963
NONHSAG048562	2.45954702	2.45195937	2.43436373	2.31736831	2.6220341	2.34318269	2.46321795	2.16402119	2.49220405
NONHSAG048563	5.87998287	5.70354272	5.1361743	5.48823174	5.41489569	5.51463797	5.30077596	5.31158614	5.50155443
NONHSAG048569	4.40273502	4.20002608	4.58069578	4.32996279	3.67577069	4.47180234	3.74561036	4.48750229	3.97337429
NONHSAG048593	2.62458877	2.58377826	2.59068056	2.48513931	2.49006186	2.84820585	2.38129081	2.74693657	2.75196601
NONHSAG048597	2.69150758	2.61139574	2.44813063	2.30933775	2.53188509	2.42790003	2.38925319	2.45689357	2.20337529
NONHSAG048604	3.74189031	3.80393112	3.96580316	3.70416797	3.74326082	4.05480434	3.72856688	3.89452568	4.1079535
NONHSAG048605	4.57504482	4.89516646	4.78395204	5.14197871	4.48757735	4.55855511	4.87837018	5.12625198	4.55741517
NONHSAG048651	3.10578367	3.57863374	3.49903314	3.46560804	3.52855211	3.30649185	3.21962792	3.25118915	3.17771547
NONHSAG048682	4.97175882	5.19707449	5.43047801	5.16322108	5.22056228	5.24704049	5.20219274	5.0564903	5.24704049
NONHSAG048687	3.21092584	2.93752377	2.70547695	2.79877094	2.9131213	3.16001832	2.91493335	3.03974749	2.9386502
NONHSAG048746	6.11021552	5.67103268	5.15467982	5.87476289	5.68594286	6.04717279	4.66503421	5.3425878	5.47888749
NONHSAG048747	8.33708433	7.61954677	7.84913779	7.73633409	7.80505646	7.42716339	7.89164408	7.80817965	7.48211809
NONHSAG048781	5.1800245	5.08465333	5.13932888	5.06915921	5.23286111	5.02354465	5.28648318	5.04335112	5.0373204
NONHSAG048790	3.03212277	2.31268689	2.37897473	2.39991738	2.29055969	2.50949194	2.36734656	2.45984464	2.5208598

NONHSAG048794	5.24339053	5.28909214	5.37625611	5.54418861	5.36724437	5.28537387	4.97246819	5.29758099	5.28642548
NONHSAG048813	3.61370525	3.85050398	3.39228181	3.49579154	3.4939749	3.86447071	3.42680596	3.79276399	3.42225327
NONHSAG048817	4.2754179	4.05342932	3.7842449	4.10594124	3.89319588	3.7842449	4.01975907	4.15300286	3.80646525
NONHSAG048836	5.03118342	4.93189761	4.61353441	4.59670512	4.84186183	5.10414268	4.76212162	5.04944842	4.38501245
NONHSAG048840	4.51149444	4.93347131	4.79595974	4.42511084	5.10648481	4.99883644	5.48526884	5.31170947	4.8418196
NONHSAG048846	3.84660664	3.45885665	3.26356127	3.40806788	3.32989194	3.46272699	3.89049167	3.29056122	3.43625083
NONHSAG048902	2.81712817	3.06403125	2.65667549	2.58166911	2.57265992	2.99346405	2.64260398	2.58025527	2.46898313
NONHSAG048962	6.32691758	6.38406595	6.37352033	6.16867565	6.37140382	6.35833393	6.27695124	6.32898943	6.39006445
NONHSAG048976	5.40358672	4.96612165	4.98757392	4.61056691	5.09544485	4.6892242	5.15884111	4.92336185	4.81853953
NONHSAG048984	2.61501003	2.84142619	2.54566192	2.61689769	2.58983231	2.74546664	2.64219903	2.60196063	2.53250167
NONHSAG048993	5.66392999	5.7952957	5.65881364	5.77558676	5.65645568	6.04696485	5.84527321	5.75324176	5.85341679
NONHSAG049003	4.6440645	4.38029084	4.17191807	4.03260019	3.81279455	4.46110754	4.24293952	4.49189086	4.07737893
NONHSAG049010	4.10647737	3.80378224	3.93475496	4.08616695	3.75123734	4.03683862	4.06508499	4.05984072	4.15880965
NONHSAG049037	6.20010598	5.72820969	5.65687247	5.77694923	5.77707672	5.80119956	5.61631354	5.77541354	5.77965549
NONHSAG049079	7.09099999	7.69243366	8.00548271	7.89085652	7.96019804	7.58211061	7.80851441	7.6357864	7.8366422
NONHSAG049095	6.27319092	6.74379351	7.24061492	7.18586158	7.1308506	6.54267712	6.98546632	7.15676851	6.70105546
NONHSAG049099	2.68753391	2.6829112	2.57925439	2.69070827	2.62314707	2.78240065	2.74651477	2.70665918	2.82398462
NONHSAG049119	3.60805571	4.2459909	3.97424018	3.39639621	3.42861764	4.35481952	3.70816451	3.83715921	3.52045021
NONHSAG049134	3.74416029	3.93377012	3.66449098	3.77987208	3.77948996	3.72636543	3.87669211	3.77177313	3.76794777
NONHSAG049182	4.26011422	4.36209708	4.30352638	4.16841878	4.21366673	4.31031167	4.28876503	4.2984555	4.04483778
NONHSAG049240	4.43315519	4.69933241	4.48324944	4.36568941	4.10813313	4.68871551	4.55819314	4.4869647	4.01930572
NONHSAG049298	3.41808451	3.44701585	4.21764224	3.64223472	3.8424662	3.37433641	3.57104191	3.40822492	3.49197313
NONHSAG049309	6.2435012	6.01002533	6.21651851	6.14736608	6.38718354	6.13645956	6.09768017	6.24449782	6.11515766
NONHSAG049336	5.53378908	5.3165299	5.33688869	5.31290658	5.35565756	5.45024288	5.38427408	5.20697694	5.00092314
NONHSAG049339	2.79413549	2.66050517	2.53638385	2.67832747	2.64737333	2.67486475	2.68977712	2.62052331	2.43699845
NONHSAG049341	5.87510042	5.53798416	5.68503694	5.51335536	5.63930977	5.4438163	5.45748053	5.73467318	5.68464723
NONHSAG049344	4.18546177	4.02511845	3.91141685	4.03293778	4.07279368	3.90371709	3.98069075	3.94154462	3.78108648
NONHSAG049350	4.77197144	4.90277099	4.39412693	4.20325691	4.64586349	4.87907213	4.34258458	4.62349288	4.47915082
NONHSAG049357	2.88252447	3.30045226	3.11521441	2.94273467	3.00750136	3.31124362	3.19113175	3.15491394	3.00122684
NONHSAG049388	3.03516929	2.88267932	2.93896276	2.87565274	2.57854397	3.14300417	2.69613326	2.98419188	2.7737835
NONHSAG049409	2.83121772	2.6536558	3.02026626	2.84197085	2.89075048	2.99697069	3.10566421	2.93623377	2.95127285
NONHSAG049422	3.80593987	4.09905049	3.53530091	3.80317944	3.62528267	4.06884981	3.59317429	3.79605075	3.58690675
NONHSAG049424	5.86334493	5.71361346	5.7896284	5.63690683	5.56709598	5.91329929	6.01941688	5.62481795	5.47272281
NONHSAG049464	3.74366305	4.64339984	4.82728507	4.64723289	4.48885084	4.09590082	4.73692216	4.68217502	4.92034387
NONHSAG049487	4.63376423	4.43996371	3.99182471	4.46401671	4.34441889	4.03995112	4.64863153	4.47311204	4.01145096
NONHSAG049492	2.93667908	2.9240947	2.91527981	2.98808898	2.80013203	3.1661746	2.83399997	2.81923504	2.7213964
NONHSAG049507	3.31770789	4.04679317	4.32400112	3.61737405	4.02910942	4.0037843	4.0609005	3.7292083	3.64011669
NONHSAG049508	3.56660707	3.86704589	3.66920682	3.46560804	3.69134638	3.89727943	3.56335755	3.71014232	3.26255826
NONHSAG049511	3.54360171	3.61106403	3.38720726	3.32076472	3.09193374	3.5123882	3.1250591	3.36262623	3.25258439
NONHSAG049515	2.62605037	2.59098427	2.34089342	2.32249745	2.16128507	2.57133159	2.59990293	2.24172283	2.34862083
NONHSAG049523	2.31152595	2.4686935	2.58364517	2.25488019	2.47374125	2.22425782	2.57413849	2.3567329	2.31846572
NONHSAG049524	4.07340385	3.77207366	3.35835935	3.72735125	3.40305669	3.55318833	3.5354709	3.7205345	3.53007857
NONHSAG049526	4.20610294	3.97889003	3.96648866	4.16643796	4.02266189	4.20601831	3.94835011	4.2006937	3.8230364
NONHSAG049528	4.44116974	4.90584481	4.32153782	4.59863985	4.55819314	4.5325702	4.62695463	4.7643988	4.66206043
NONHSAG049529	4.1176987	3.64443004	3.92332736	3.79077473	3.56528796	3.95567504	3.92187279	3.93341696	3.72657729
NONHSAG049530	3.2484536	2.68472535	2.84686372	2.6836941	2.64021648	2.73551282	3.20276831	2.76134779	2.74123719
NONHSAG049531	5.33958468	5.06511066	4.78892126	4.98399915	4.95854016	5.101896	5.1292647	4.86168357	4.74407996
NONHSAG049532	3.56210373	3.49761104	3.22888576	3.22325333	3.15566163	3.18415058	3.43547147	3.26963823	3.13398835
NONHSAG049534	2.43196033	2.59315189	2.39725003	2.66513178	2.5216941	2.94026567	2.59595877	2.46399563	2.36834184
NONHSAG049539	4.65175242	5.00439196	5.06449352	4.81000909	4.81696689	4.82109365	5.12614096	4.75340803	4.99165075
NONHSAG049594	3.22769777	3.8945757	3.33792816	3.48003024	3.30804475	3.62152537	3.56357948	3.49845197	3.3673848
NONHSAG049595	3.33599277	3.61875606	3.41935461	3.63165266	3.49847858	3.20707893	3.19646142	3.53435363	3.42847298
NONHSAG049661	2.37413938	2.21172024	2.04363075	2.26144795	2.4382055	2.30182158	2.28583781	2.25371131	2.26779799
NONHSAG049665	2.94037666	2.98626502	2.69907672	2.85778226	2.86576799	2.58369742	2.91558289	2.88525964	2.93786607
NONHSAG049689	3.12511641	2.91954506	2.81481742	2.83546424	2.69106304	3.02305237	2.85732935	2.85357419	2.89850887
NONHSAG049734	3.86036631	4.17976886	3.64324209	3.61871632	3.71562117	3.67662325	3.81172089	3.79386737	3.48207208
NONHSAG049792	4.0756383	4.19439587	3.8201348	4.05875472	3.65428074	3.92050013	3.94343314	3.9425602	3.51861413
NONHSAG049868	5.60574128	5.33475449	5.33659475	5.39297235	5.59483908	5.53540506	5.4068583	5.30500268	5.3028958
NONHSAG049879	2.97531073	2.90007219	2.89324772	2.73762117	3.1033755	2.94989384	3.08867718	3.01463619	3.00540588
NONHSAG049885	3.81214343	3.72579623	3.76273499	3.86717328	4.04077406	3.85764741	3.82289538	3.87500551	3.6048197
NONHSAG049895	4.43532814	4.05574922	4.05316606	4.05440821	3.97492085	4.28393236	4.36384297	3.75579314	3.92460109
NONHSAG049902	2.80049418	2.34650445	2.29195451	2.33541284	2.36847741	2.43439386	2.49895086	2.20270548	2.22697377
NONHSAG049904	7.12256268	6.85073586	6.58280294	6.79156973	7.04868631	6.92640096	7.00034921	6.81487777	6.81358024
NONHSAG049930	3.09663182	3.26119518	3.39169587	3.10406261	3.03086507	3.348151	3.25049484	3.27413739	3.34590721
NONHSAG049931	3.26686415	3.21430622	2.88935187	2.96535212	2.8232631	3.44229404	2.62105309	3.16688397	2.89638073
NONHSAG049936	2.6577884	2.53679848	2.74880514	2.40288715	2.42807048	2.40828713	2.57363375	2.52351053	2.80959805
NONHSAG049939	4.6536962	4.79320415	4.86850684	4.70877217	5.12720946	4.72982492	4.99416838	4.71995861	4.84339511
NONHSAG049977	4.85822793	5.21803064	4.79878863	5.16447117	4.85306973	5.24106349	5.0218824	4.90792986	4.73841426
NONHSAG049980	4.507445	4.46608636	5.00875801	4.54842911	4.77131329	4.46608636	4.70512619	4.56982616	4.25233245
NONHSAG049984	2.70139672	2.33815577	2.51979852	2.40075521	2.39334001	2.51476097	2.43310502	2.48014275	2.90303134
NONHSAG049989	4.28706618	4.2949186	4.14728603	4.07107863	4.02331198	4.74812653	3.94430405	4.40943546	3.99811487

NONHSAG050013	5.34125225	5.77458893	5.86897872	5.62609	5.63449095	5.67207307	5.5900372	5.65806755	5.50961389
NONHSAG050025	5.22386999	4.96834398	5.23285787	5.53722195	5.37977494	5.08340314	5.17874574	5.28614526	4.98554516
NONHSAG050057	3.82930686	4.11160282	3.52472454	3.70341914	3.54421551	3.92957559	3.77410761	3.53494679	3.72085232
NONHSAG050083	5.23781561	5.03784471	4.9389686	5.15640123	5.19648175	5.13535778	5.3130174	5.16006088	5.09264559
NONHSAG050088	4.30772666	4.40868534	3.92962918	4.11823282	4.28920261	4.36209986	4.31824523	4.36999524	4.05539054
NONHSAG050093	5.43910491	5.72864575	5.05101491	5.23022239	4.92542636	5.82718993	5.8287428	5.28734738	5.10895946
NONHSAG050175	4.17712122	4.62815271	4.26951256	4.16913314	4.32332002	4.70344048	4.23377629	4.30197084	4.31424765
NONHSAG050188	2.97707723	3.07789755	2.93723305	2.99460628	2.80761137	3.09084303	2.96482254	3.21367609	2.92123349
NONHSAG050210	5.37794511	4.78719781	4.25056415	4.50491839	4.7766543	4.92420516	4.80138565	4.82695507	5.09368065
NONHSAG050227	3.1645641	3.55362449	3.03517608	3.08814414	3.06849014	3.17824144	3.2537052	3.38060949	3.0770042
NONHSAG050230	2.78448883	2.89297734	2.61011814	2.50696311	2.80823177	2.73419631	2.92208867	3.02325905	2.69587023
NONHSAG050243	3.62355068	3.35440107	3.41813294	3.57221347	3.52537379	3.55393159	3.7853216	3.50275993	3.34639644
NONHSAG050259	2.44793055	2.27348259	2.49538469	2.45827653	2.3364327	2.24906836	2.40473059	2.41282834	2.41900362
NONHSAG050280	5.06920948	4.80283338	4.79748583	4.77700627	4.78480023	5.0599348	4.57394075	4.78352902	4.92941037
NONHSAG050284	3.79591581	4.33712981	3.89538531	4.1308264	4.07486817	4.27954231	4.09220962	4.09706595	4.04120351
NONHSAG050285	4.01704801	4.52286091	4.37135934	4.32644081	4.15469047	4.39360403	4.22242987	4.39338498	4.13805811
NONHSAG050296	5.00259327	5.23243377	4.88454013	4.95226822	5.07270467	5.31665803	5.21782614	5.2641706	5.07044627
NONHSAG050300	3.55564936	3.44733316	3.57579171	3.63952419	3.55449142	4.08038763	3.32726431	3.74533031	3.68323381
NONHSAG050305	6.41330729	4.26346217	4.71838767	3.8203354	4.21301285	4.73991536	4.81557479	4.78119393	4.14478931
NONHSAG050335	4.41078457	4.43848323	4.08304025	4.10887461	4.28036557	4.64377562	4.23783207	4.38126702	4.14397463
NONHSAG050348	2.54093133	2.54747519	2.29054414	2.27606045	2.41413075	2.79245051	2.70089866	2.70663931	2.62173594
NONHSAG050370	3.11220598	3.25432266	2.83973733	3.09053599	3.10529251	3.21833265	2.9831318	3.13994301	2.85048068
NONHSAG050372	4.37522028	4.08148505	4.20001758	4.11954065	4.16827415	4.05618122	4.14596859	4.2014026	4.06205939
NONHSAG050397	4.2204983	3.90765535	4.28759631	4.20109778	4.12276081	3.73571964	3.7874212	3.67755661	4.26653858
NONHSAG050419	6.40710535	6.07284532	6.33202521	5.95790646	6.27162344	5.96678203	6.46044229	5.90074219	6.21110443
NONHSAG050438	2.39291884	2.14874985	2.05066205	2.21496608	2.00666673	2.18310025	2.19495427	2.16078528	2.21913567
NONHSAG050440	2.56687549	2.32895395	2.45678919	2.22810902	2.35396731	2.4666568	2.5081571	2.46820742	2.81619638
NONHSAG050454	3.12366033	3.74704366	3.50708311	3.25667673	3.35714171	3.49801324	3.50817492	3.25878564	3.18972731
NONHSAG050461	2.96518848	2.50742932	2.9419354	2.43870737	2.26896658	2.28085107	2.55254604	2.45911993	2.64005177
NONHSAG050473	6.43296802	6.59367709	6.71123702	6.56586517	6.5311881	6.58153861	6.52116927	6.67516301	6.46660226
NONHSAG050503	4.07202126	4.60476367	4.18016961	4.34381197	4.19525425	4.3225794	4.44948503	4.51568378	4.3313296
NONHSAG050531	3.02814628	2.92267653	3.30802874	3.00063911	2.7031417	3.20225542	2.95248168	2.88370155	2.89361421
NONHSAG050576	3.36468341	3.42149963	3.27183166	3.40639673	3.13413076	3.4116504	3.4003266	3.38433162	3.26939897
RP11-48B3.4	6.49454102	5.38111596	5.62889353	5.19720504	5.39745066	5.12047877	5.42567173	4.94606786	5.16913041
NONHSAG050607	2.96792715	3.13355934	2.89509054	3.19564783	3.12292845	3.36071946	3.07606305	3.06881359	2.88588364
NONHSAG050643	2.24173779	2.13955923	2.06615535	2.20735716	2.01166144	2.19981721	2.29806708	2.18356681	2.07757689
NONHSAG050690	10.01656272	8.3819082	8.67219141	8.52594038	8.42584747	8.22529027	9.08765723	8.50344697	8.80526955
NONHSAG050699	6.24531921	5.8368221	5.85373134	5.57918447	5.83826581	5.77351495	6.22762901	5.88702728	5.74626986
NONHSAG050714	2.94096919	2.94518166	2.91128317	2.80861845	2.83052014	2.90049404	2.73727141	2.68798334	2.62281125
NONHSAG050718	5.27881804	5.90877976	6.34460097	6.06684566	5.85171183	5.78117338	6.7903106	5.98079649	6.21335731
NONHSAG050752	2.48087793	2.44039116	2.53498186	2.24403181	2.39490353	2.80959805	2.55726295	2.57586792	2.33665805
NONHSAG050804	6.41046274	6.76559515	6.85466849	6.78386553	6.67097645	6.56918096	7.35519599	6.74837173	6.57851165
NONHSAG050806	5.94816663	5.6860186	5.21804839	5.35047929	5.2885016	5.38398161	5.60431442	5.48228077	5.21476825
NONHSAG050815	2.66063565	2.59998463	2.76431761	2.68335897	2.62689174	2.67713451	2.52742079	2.82411264	2.53439911
NONHSAG050829	2.64250885	2.48939074	2.24332068	2.52656161	2.22422636	2.43021015	2.52656161	2.60707542	2.62173594
NONHSAG050841	4.46587213	3.90336014	3.96411138	3.90048737	3.85324203	4.05495709	3.85463358	3.77895228	3.69129617
NONHSAG050865	7.5496287	7.2704331	7.26682287	7.48502925	7.30223754	7.06386857	7.63670147	7.28892737	7.11427048
NONHSAG050866	6.00869513	5.86147356	5.90801588	5.91245223	5.64853507	5.50255395	5.69514071	5.59850726	5.57328212
NONHSAG050902	3.79463485	4.08707148	3.65034885	3.79770652	3.79578613	3.61585426	4.03381984	3.98775458	3.83634968
NONHSAG050903	7.24433043	7.34441599	7.63284295	7.34150169	7.41691203	7.23293359	7.23729972	7.21760553	7.29370163
NONHSAG050913	5.80822689	5.54690791	5.29051322	5.2537557	5.28690928	5.48939273	5.32162201	5.27201517	5.10999722
NONHSAG050914	3.64466888	2.91506139	3.13295803	3.12356785	3.27880439	2.95787621	3.19783629	3.04778229	3.34200323
NONHSAG050915	4.21038312	4.6996059	4.25820029	4.12312168	4.54363782	4.53873163	4.6283271	4.25708384	4.24134738
NONHSAG050921	2.98793461	3.18499258	2.86753774	3.18155712	2.97167515	3.18739475	3.20572221	2.92983197	3.03731084
NONHSAG050966	5.23558843	4.80218095	4.85175323	4.59296832	4.53468049	4.72786246	4.66315375	4.58685665	5.12780833
NONHSAG050969	3.24351821	2.68452391	2.7829298	2.76365672	2.9172691	2.98114449	2.61365336	2.95740494	3.00990622
NONHSAG050970	3.34587046	3.29189608	3.04496947	3.0850233	3.18619748	3.46391602	3.36486158	3.19504725	3.02002962
NONHSAG050978	3.57731659	4.00224536	3.74999211	3.64175915	3.41465204	3.82598936	3.81247108	3.92736056	3.55321879
NONHSAG050982	6.39299965	6.10354703	6.29269005	6.07522184	5.93939547	6.20072423	6.48237585	6.33442461	6.46775691
NONHSAG051053	4.28403634	4.21431498	3.9824164	4.03812994	3.90358388	4.39182914	4.09852919	3.96062407	3.8098937
NONHSAG051068	2.08310314	2.0366566	2.0217045	2.12664218	1.96626014	2.12019742	2.31126421	2.0915194	2.02457769
NONHSAG051082	6.0787146	5.65029438	5.47440102	5.50269223	5.61054111	5.14380481	6.18668723	5.41308835	5.12939303
NONHSAG051104	4.70020217	4.60227387	4.40526226	4.09738817	4.38402333	4.77788581	4.50011972	4.50903117	4.38186528
NONHSAG051105	4.09363981	3.46774954	3.30686358	3.13230476	3.28351814	3.51401913	3.82502765	3.80353631	3.58232758
NONHSAG051129	3.12511641	3.5107758	3.46125204	3.55741306	3.37219329	3.85033099	4.11173783	3.44455154	3.47862956
NONHSAG051137	3.92489251	4.14285975	3.85853206	3.95470445	3.80315066	3.89001377	3.96695949	3.87727876	3.94916099
NONHSAG051145	3.73764123	3.96186121	3.97927	3.65335349	3.71706865	3.68791801	3.96651861	4.17191482	3.57208603
NONHSAG051156	2.70659513	2.52008067	2.28676122	2.30496557	2.33446421	2.4977882	2.44704143	2.45797504	2.50970102
NONHSAG051181	3.34749135	3.26829426	3.25941979	3.14799545	3.27568048	3.69084578	3.31619825	3.50031983	3.27909342
NONHSAG051194	3.32338318	3.11626095	3.09878604	2.93774287	2.87964571	3.08888233	3.06245005	3.24420902	3.09178679
NONHSAG051196	5.06357346	5.40165265	5.44093624	5.43805271	5.34329912	5.5209462	5.60207459	5.54509629	5.35505422

NONHSAG051205	3.50813752	4.35140338	3.76553203	4.26686791	4.23615932	4.04176496	4.45980112	4.14447073	3.68455765
NONHSAG051207	2.70019207	2.77987405	2.76113113	2.42538043	2.54735803	2.37481988	2.80295926	3.01549716	2.80295926
NONHSAG051233	2.45276468	2.48232407	2.68987512	2.25291212	2.17662064	2.57063254	2.35779679	2.66949998	2.63441669
NONHSAG051237	2.3091059	2.50472306	2.56020926	2.62332959	2.45458337	2.96614687	2.52883601	2.69481259	2.60459001
NONHSAG051255	3.30280906	3.47842951	3.30124446	3.43399936	3.34830724	3.67914127	3.54943963	3.55153217	3.434486
NONHSAG051258	4.9106558	5.08714599	4.96044345	4.85817577	5.01941162	5.05875945	4.75217094	5.03834438	4.9330458
NONHSAG051259	3.68598842	3.59676569	3.23410662	3.21848068	3.46062123	3.43464652	3.85161117	3.63740667	3.6424625
NONHSAG051263	3.2457117	3.43456934	3.14115812	3.11978079	3.10260605	3.2370152	3.12283203	3.15567347	3.02811015
NONHSAG051266	4.22679126	4.25420685	4.06421038	4.15429819	4.21584737	4.2714379	3.94474208	4.11456903	3.96230932
NONHSAG051273	2.60728775	2.64284861	2.78141576	2.75122791	2.67244069	2.78079555	2.57971171	2.7239946	2.69673602
NONHSAG051275	2.30235876	2.52005741	2.3219781	2.26114749	2.33368383	2.8276923	2.25756482	2.58824363	2.27103176
NONHSAG051297	2.5566259	2.54349871	2.64161378	2.49673441	2.62818134	2.80814508	2.63357162	2.52183482	2.55279464
NONHSAG051305	2.86101844	3.31225562	2.89746377	3.14481958	3.03085291	3.21451245	2.91151221	3.11176199	3.12224667
NONHSAG051327	5.23288958	5.49902636	5.09922548	5.13837966	4.99940288	5.32852215	5.18636387	5.43822001	5.00341632
NONHSAG051334	2.98367812	2.83494868	2.83121904	3.02257825	3.00513653	2.98879484	2.76028525	2.78944735	2.83869442
NONHSAG051339	5.0624034	5.0990496	4.94273581	5.00417758	5.15397102	5.23146834	5.01967741	5.19836509	4.98615128
NONHSAG051345	3.09663634	3.29620358	3.17204822	3.14548153	3.16867119	3.53414283	3.19812196	3.30665739	3.11538589
NONHSAG051356	4.55095464	4.48538924	4.37770157	4.43135311	4.20559557	4.46355635	4.50470376	4.42140418	4.32568363
NONHSAG051375	3.49150222	3.04184723	3.09500109	3.15878106	3.11508028	3.02469042	3.0324669	3.27524875	3.1973754
NONHSAG051392	3.79129558	3.39223287	3.40964165	3.10701536	3.18068464	3.44717205	3.06838828	3.28604795	3.13058988
NONHSAG051400	6.87931254	6.99311727	6.87395506	6.97682728	7.03723014	7.01146194	7.00353724	6.88751836	6.94370724
NONHSAG051424	6.04737536	6.40120009	5.86597481	6.00120706	5.91627059	5.96569202	6.11548795	6.17310799	5.80717811
NONHSAG051435	4.2146002	4.33579297	4.12447343	4.33376518	4.34737212	4.26445943	4.56557415	4.73383034	4.19185771
NONHSAG051445	5.50252767	5.81894477	5.53145681	5.62649485	5.61551614	5.7477598	5.72332405	5.68362886	5.516862
NONHSAG051446	4.58397198	4.68094524	4.57994666	4.82287993	4.5887413	4.49402797	4.92600627	4.77519136	3.99749643
NONHSAG051464	4.35164524	5.35342936	4.94083143	4.84587637	4.89391263	5.0983041	4.85497667	4.7625622	4.54498011
NONHSAG051484	5.51617321	5.41618642	5.42825531	5.50837967	5.59275084	5.52003468	5.45920571	5.51148984	5.46492764
NONHSAG051497	5.05821558	5.29712987	5.05762015	5.16688396	5.39266172	5.1086014	5.25689952	5.28038604	4.96184182
NONHSAG051574	4.5754921	4.93589187	5.01771164	4.90848782	4.66420421	4.97297647	4.95141151	5.11894236	4.63674405
NONHSAG051638	7.30010647	7.23857713	7.36093376	7.29298517	7.50807535	7.42751631	7.42721783	7.25063417	7.34492094
NONHSAG051650	3.36623329	3.62976181	3.36400385	3.60011625	3.07472119	3.22819319	3.63687645	3.28604795	3.10646949
NONHSAG051658	5.1354621	5.69275813	5.35417667	5.22900271	5.22745452	5.5486146	5.40564149	5.55019439	5.1559486
NONHSAG051661	2.61588124	2.79270365	2.41683272	2.60010463	2.72615501	3.01349969	2.70762504	2.77409178	2.63260705
NONHSAG051677	4.74394667	4.81764191	4.54346464	4.7269491	4.60468614	4.64912661	4.65194963	4.66020233	4.74722865
NONHSAG051728	3.90564083	3.77845511	3.41136602	3.80233901	3.39483559	3.76030474	3.56215809	3.54290332	3.29431073
NONHSAG051730	3.20368618	3.04928352	3.02270768	3.29220882	3.24037183	3.42810868	3.35282472	3.31553999	3.03660847
NONHSAG051770	2.72716862	2.45446301	2.62434713	2.33484971	2.29947689	2.40483837	2.62774513	2.28042754	2.61569864
NONHSAG051806	2.99083691	2.7447637	2.52500672	2.58504346	2.5304014	2.64885231	2.60851161	2.75607512	2.68663952
NONHSAG051813	5.44186763	4.68209787	4.55286154	4.48754615	4.35709793	4.50417424	4.44479693	4.12653526	4.40776701
NONHSAG051826	2.78100338	2.88859114	2.70320188	2.74263897	2.62742509	2.97398832	2.66562575	2.81594595	2.74868732
NONHSAG051866	2.6246936	2.43897465	2.59508341	2.45014112	2.35694315	2.62121855	2.49248844	2.7840129	2.35527684
NONHSAG051885	2.94195982	2.47379714	2.41861427	2.77173702	2.48568809	2.74397582	2.72237976	2.55789977	2.36601296
NONHSAG051943	3.3803646	3.06395253	2.97223416	2.72080285	3.37006837	3.08049183	3.27319663	3.03732951	2.87780201
NONHSAG051945	2.72665811	2.54517047	2.55699187	2.42056495	2.46322034	2.59429889	2.73509566	2.55274743	2.74013757
NONHSAG051962	4.06523225	4.13630206	4.20400418	4.29218685	4.1016609	4.39763816	3.98023288	4.40031528	4.13531754
NONHSAG051968	2.39544949	2.42107893	2.44438018	2.39156906	2.20677048	2.32865135	2.43471409	2.23559943	2.327463
NONHSAG051985	2.78980424	2.90383006	2.82178436	2.64185179	2.85545304	2.96021489	2.43712038	2.89863752	2.75553075
NONHSAG052010	3.17756824	3.1821152	3.08477427	2.80545976	2.72689335	3.28747668	3.11176199	3.11176199	2.99664763
NONHSAG052012	6.56257758	6.62972231	6.52036363	6.67632795	6.57161814	6.77461307	6.50186559	6.48723435	6.42380731
NONHSAG052042	2.77079921	3.34716228	3.28753142	3.22559908	3.11853213	3.01197841	3.07142999	3.20436805	2.88188823
NONHSAG052090	3.0495496	2.69860068	2.67908277	2.67741933	2.61253754	2.83919083	2.70127621	2.66690838	2.59792712
NONHSAG052112	4.17713372	3.54202717	3.86483522	3.98092876	3.96159581	3.90060737	3.97107688	3.77300885	4.11685772
NONHSAG052118	4.7126009	4.55533379	4.55470718	4.38717631	4.56558627	4.77820146	4.67631125	4.60219245	4.35412505
NONHSAG052125	8.93328905	8.5259773	8.59825427	8.45539378	8.73401015	8.56168016	8.60466158	8.40522027	8.32619364
NONHSAG052126	3.80959572	3.542335	3.78217641	3.54071283	3.6565637	3.57847282	3.65261257	3.53805349	3.56922363
NONHSAG052157	3.99919511	3.94142215	3.54718187	3.8778892	3.58248284	4.10629631	3.84199151	4.05198358	4.18170548
NONHSAG052162	5.9414117	5.25163132	5.22384669	4.93021701	5.36657968	4.72498138	5.63516361	5.04831573	5.05058199
NONHSAG052178	6.26588749	6.2728928	6.58626206	6.35132408	6.58965791	6.3452151	6.47250936	6.38551247	6.6721191
NONHSAG052186	6.63310397	6.86042868	7.05594018	6.91487156	6.88101828	6.86996	6.9079827	6.66243139	6.84996684
NONHSAG052188	4.38222549	4.3558072	4.26426734	4.20036754	4.07770763	4.22399569	4.2833355	4.28881125	4.2127654
NONHSAG052409	5.56786092	6.07052285	6.14743212	5.77759286	5.86355852	5.79122572	5.77598044	5.78835137	5.65536825
NONHSAG052414	4.72877094	4.67791348	4.75064075	4.64106052	4.95440763	4.85396858	4.60896143	4.60180991	4.7935632
NONHSAG052449	7.42232351	7.15554079	7.10652852	7.26410644	7.26348933	6.98818154	7.409067	7.279821	7.16011077
NONHSAG052499	3.4225804	3.87260436	3.56805893	3.73782672	3.56121252	3.75967315	3.59510949	3.899847	3.7877961
NONHSAG052508	5.17155132	4.80827597	4.28652421	4.56589028	4.71351498	4.55475355	4.69345343	4.53959118	4.37891126
NONHSAG052530	3.61587091	3.4177229	3.37131718	3.49604512	3.33650943	3.49593013	3.47592731	3.46466205	3.47010586
NONHSAG052547	5.960324	5.41325814	5.56433399	5.42956784	5.76802617	5.53439894	5.38316225	5.54703915	5.68342345
NONHSAG052583	2.21671221	2.55092861	2.4523794	2.38535787	2.65754972	2.61273352	2.53676946	2.35826262	2.58915432
NONHSAG052586	2.13873428	2.22341284	1.91123383	2.22153018	2.28605066	2.04138973	2.03926875	2.08701365	2.09475459
NONHSAG052609	3.23596336	3.38570401	2.97615963	2.8906272	2.92654777	3.15183797	3.33992687	3.38066442	2.68148331
NONHSAG052620	5.75534935	5.80880179	5.96573108	5.37441466	5.49326018	5.64504287	5.75534052	5.57758037	5.32840593

NONHSAG052621	3.53548629	3.53413545	3.6014413	3.51384312	3.29585415	3.80296559	3.49639899	3.56596256	3.4061671
NONHSAG052627	6.24259735	5.76772451	5.99471091	5.77641921	5.78918148	5.79129366	5.79648179	5.81706281	5.86301798
NONHSAG052636	4.36911746	4.55442367	4.49895585	4.40752042	4.15413352	4.46099449	4.03935582	4.42719511	4.45787261
NONHSAG052644	3.24273858	3.66473917	3.14995422	3.46560804	3.40589337	3.71490886	3.32695677	3.44204274	3.56175218
NONHSAG052726	4.92220248	5.3320145	4.92607002	4.93490592	4.68276969	4.88462443	4.85920063	5.10488647	5.01695283
NONHSAG052734	3.80511437	3.81041414	3.56770599	3.81964572	3.47921113	3.63612797	3.74755155	3.66078513	3.46838635
NONHSAG052752	5.97345553	5.57153721	5.9118493	5.46818271	5.61207085	5.71127625	5.63224657	5.39590741	5.93408027
NONHSAG052756	2.90158752	2.9108253	2.86238938	2.65829556	2.71604058	2.96563556	2.75763417	2.8713191	2.71201368
NONHSAG052760	5.1186451	4.40182185	4.93137087	4.81738846	4.73393126	4.68080011	4.77884003	4.76787299	4.91011762
NONHSAG052772	6.53052212	6.61945461	6.30713419	6.3428795	5.99028117	6.16247325	5.97978259	6.42011519	6.19653079
NONHSAG052776	5.20212126	5.73986004	5.39128512	5.34619708	5.22661972	5.12403204	5.74210496	5.63918548	5.54097564
NONHSAG052817	4.18660579	4.97980995	4.29723328	3.82483026	4.59481546	4.81602541	4.39293546	4.2863017	4.39652017
NONHSAG052818	2.75002388	2.77432792	3.36829818	3.33282219	3.15495789	3.52325848	3.1587421	3.16325703	2.87478706
NONHSAG052837	5.9372948	5.96364197	5.83020305	5.72111389	5.76761076	5.90241651	5.69676236	5.98380386	6.13958574
NONHSAG052879	4.35116814	4.29966083	4.2387038	4.42080469	4.05177041	4.03935023	4.204532	4.37627676	4.36751383
NONHSAG052880	5.8342424	5.07578586	5.32711462	5.46476464	5.42480874	5.33696981	5.39089851	5.22644049	5.45133852
NONHSAG052885	4.48785085	4.75796321	4.70031878	4.44213874	4.53853485	4.95890418	4.55455262	4.94057341	4.55497405
NONHSAG052886	4.7314035	4.51163882	4.23098723	4.15225287	4.39169275	4.67953672	4.23716437	4.53445028	4.28448204
NONHSAG052890	6.29308695	6.10065074	6.3026755	6.04897138	5.93878406	6.31465029	6.24164295	6.13733911	6.07977002
NONHSAG052915	6.68070777	7.00669945	7.17966139	7.03385298	6.96804067	6.94876366	6.94892611	6.85676369	6.86367635
NONHSAG052978	3.68450188	3.53756415	3.2636238	3.22089598	3.33118288	3.63975882	3.39333474	3.51554534	3.26948615
NONHSAG052998	2.79362476	2.61011814	2.90461346	2.7799272	2.61677311	2.57720251	2.83576946	2.70439081	2.86779534
NONHSAG053000	2.99484468	2.4458087	2.6212973	2.62997469	2.6922475	2.84966494	2.46136062	2.69044572	2.67614872
NONHSAG053032	4.15808989	3.8506161	3.84581173	3.90032939	3.84314719	4.0221916	4.20565144	3.94141296	3.95414306
NONHSAG053081	2.50630071	2.42210132	2.4990424	2.44162718	2.37067547	2.75386631	2.25001318	2.49825012	2.49228453
NONHSAG053118	2.23499756	2.39756069	2.31958485	2.20128827	2.33719105	2.39842105	2.21006924	2.1542304	2.61852721
NONHSAG053119	2.38366866	2.69464582	2.72841997	2.58604634	2.52421857	2.72122836	2.74370622	2.47547047	2.4649048
NONHSAG053167	3.89611721	4.08124825	4.3902857	4.104899	4.1399132	4.09800985	4.23205101	4.14330968	4.14568042
NONHSAG053168	2.39991792	2.52501258	2.47749405	2.40435029	2.3496738	2.58291457	2.39348061	2.78001804	2.46013699
NONHSAG053205	5.28284014	4.86470552	4.76289587	4.59836641	4.56779747	4.80890691	4.5326944	4.09936735	5.14397796
NONHSAG053209	2.31985341	2.54517047	2.36680461	2.43139709	2.41977167	2.31706623	2.66475127	2.24285918	2.60445193
NONHSAG053219	6.93123384	6.94157185	6.63300181	6.95467398	6.871396	6.82359472	6.81974997	6.53475931	6.82619324
NONHSAG053240	3.51771115	3.46099961	3.5962982	3.10466029	3.54756276	3.43649914	3.72048346	3.71051126	3.65689495
NONHSAG053246	4.79848171	4.85803545	4.77395699	4.6440645	4.63224515	4.58733978	5.06994363	4.88247927	4.51139219
NONHSAG053259	3.64099618	3.9140445	3.66538911	3.75753519	3.46272154	3.82033102	3.93921783	3.97088489	3.50740404
NONHSAG053273	3.5406404	3.72721318	3.68530162	3.7367701	3.64897041	3.4588159	3.84432689	3.76838146	3.69015821
NONHSAG053274	3.05489546	2.9163873	2.93762836	2.72499093	2.66370589	3.11301571	2.89233184	2.87880857	2.9413547
NONHSAG053293	4.05253241	4.40364697	4.03140888	4.35948202	4.08072401	4.11450256	4.14380829	4.09997374	4.15152492
NONHSAG053296	4.08064603	4.01781701	3.95779974	3.98441268	3.88250759	4.11193411	4.19451952	4.14118938	3.77616834
NONHSAG053299	3.314219	3.43181133	3.25726408	3.38058976	3.69311183	3.38670303	3.5805652	3.35283841	3.22203969
NONHSAG053306	5.48491015	5.16259338	4.71110803	4.66180614	4.75235567	5.12260141	4.7078239	5.01547141	4.83586227
NONHSAG053322	5.38143992	5.36067746	5.03305802	5.1571577	4.78087953	5.37790756	5.21734641	5.48327166	5.07874739
NONHSAG053329	2.6821562	2.82064744	2.85604258	2.61011814	2.79824786	2.82310691	2.77375945	2.84915489	2.57588546
NONHSAG053359	5.73788151	5.15632081	5.10608718	5.06676766	5.22428769	5.23539709	5.10252835	5.3300813	5.20223481
NONHSAG053361	4.82056087	4.65232996	4.26927498	4.38414501	4.35698154	4.78036554	4.49769677	4.53873227	4.19893041
NONHSAG053376	6.60715033	6.26402579	6.51693695	6.20490723	6.15094292	6.36928389	6.30415915	6.15055635	6.38445626
NONHSAG053377	6.36238444	6.70085667	6.65422664	6.88462364	6.73756647	6.64577716	6.55050081	6.45758898	6.60531464
NONHSAG053380	6.08599992	5.90215542	5.90761962	5.84650171	5.97077579	5.96840555	5.99621493	5.80621065	6.03342529
NONHSAG053392	2.64237229	2.74147234	2.51081838	2.68846353	2.82476038	3.01637181	2.75425029	2.70940103	2.67853758
NONHSAG053406	4.66383137	4.56636918	4.99027056	5.03350165	4.8827181	4.94160352	5.3147129	4.6781523	5.10755809
NONHSAG053407	4.3010477	4.83077847	4.29943405	4.61765326	4.721358	4.64954164	4.37826147	4.7415184	4.32493371
NONHSAG053417	4.55819314	4.28048365	4.14957011	4.40848045	4.23120604	4.2751339	4.53998144	3.95495814	4.02645426
NONHSAG053440	3.98218233	4.0576999	4.06671673	4.07368047	3.869887	3.87974883	4.08551393	4.19112153	4.01373205
NONHSAG053450	3.27284446	3.19267189	3.15259122	3.31945957	3.22708502	3.28689161	3.35245412	3.28995895	3.35669121
NONHSAG053454	3.24267252	3.06998673	2.85420115	2.94018459	2.99014602	3.21670839	2.83017495	3.04239839	3.12956766
NONHSAG053462	3.55335226	3.85672543	3.50310962	3.59441701	3.72853924	3.74393183	3.74644938	3.6905654	3.76198604
NONHSAG053469	3.51802928	3.76766667	3.80152336	3.66619085	3.763651	3.65552568	4.28213903	4.03746832	4.01252463
NONHSAG053552	6.33944615	6.10472452	6.46187946	6.39068833	6.30759518	6.09657314	6.42138285	6.33973376	6.23928894
NONHSAG053577	7.37850771	6.68343347	6.53478467	6.34072908	6.41726647	6.48005799	6.39811015	6.47789506	6.78964347
NONHSAG053585	5.04946942	5.02556486	4.51259521	4.76644721	4.66565369	4.59799217	4.73702836	4.90984496	4.71877879
NONHSAG053586	7.31027857	6.43926462	6.75025531	6.40396017	6.59869176	6.47594671	6.49933854	6.8589953	6.63155487
NONHSAG053589	6.02925165	6.61163478	6.38674806	6.2472457	6.27087919	6.46467716	6.40965279	6.49353455	6.11304635
NONHSAG053591	5.364648	5.81973452	5.09258532	5.2756326	5.39482274	5.5209928	5.45061248	5.39792832	5.13105865
NONHSAG053607	4.33359451	4.5466706	4.36489761	4.46241559	4.51248654	4.50461175	4.45170064	4.51275739	4.51364449
NONHSAG053614	5.40196514	5.65115474	5.41625977	5.41887606	5.43463876	5.47582351	5.50997148	5.50333932	5.37771081
NONHSAG053675	4.90581802	5.54013579	5.27686282	5.33673948	5.24525312	5.27908043	5.20275988	5.09248024	5.12434048
NONHSAG053719	2.62020136	2.6828694	2.49662581	2.5192842	2.58266578	2.74794077	2.70794336	2.83971173	2.51221129
NONHSAG053722	3.20609619	3.26227478	3.37679075	3.55033496	3.55196956	3.31021529	3.6412057	3.37184187	3.12560549
NONHSAG053746	4.08616841	4.38591218	3.89134046	4.10745963	3.83821557	4.2021606	4.14993903	4.14507028	3.82285245
NONHSAG053751	3.53124517	4.23917888	3.41530965	3.53530091	3.67508828	3.6595006	4.02420619	3.64676282	3.75794158
NONHSAG053784	5.52530009	5.54977615	5.51835435	5.57624567	5.57840505	5.63765757	5.47926278	5.54265039	5.6336268

NONHSAG053801	4.14079993	4.04255763	4.10428442	3.98196489	4.04417036	4.06156997	4.1057602	4.22308295	4.08621455
NONHSAG053821	3.1871153	2.79552661	3.04836692	3.02835632	2.82390946	2.48754491	3.04089926	3.01162794	2.90816758
NONHSAG053863	3.74476429	4.15893314	3.69074899	3.77327121	3.9900964	4.0542256	4.00206597	4.02045402	3.84237085
NONHSAG053877	5.01595276	4.82343179	4.6133329	4.92617884	5.00418197	4.88245726	5.02590837	4.87669747	4.7935632
NONHSAG053892	4.88011373	5.79604251	5.82899323	5.86583231	5.61800506	6.19896337	5.85875688	5.61450569	5.39562411
NONHSAG053893	13.9110281	13.92359198	13.9214935	14.01119239	13.90924312	13.95826852	13.86978271	13.89547505	13.96351368
NONHSAG053895	14.1389565	14.17450375	14.09223549	14.23457966	14.14592104	14.13847237	14.17449826	14.18860236	14.08502415
NONHSAG053900	2.74368808	2.29517215	2.51037397	2.36191663	2.56076152	2.40467894	2.40392122	2.28235441	2.39107524
NONHSAG053985	2.84370401	2.88734602	2.88677371	2.90203043	2.90756047	2.93733161	2.93951862	2.74267242	2.96489134
NONHSAG054015	2.50704073	2.59173173	2.18485296	2.37086142	2.21813998	2.92577598	2.36419896	2.29839274	2.46908675
NONHSAG054016	2.86101844	2.79051962	2.57362604	2.50865407	2.43708454	2.85146963	2.6160416	2.5441082	2.73782634
NONHSAG054030	2.69726804	2.98491425	2.58684929	2.75686591	2.57271794	2.75348995	2.81340135	2.85054162	2.75842423
NONHSAG054071	2.54193347	2.88212574	3.03575245	2.92739344	2.79412625	2.85199247	2.67989604	2.77770583	2.73249799
NONHSAG054103	2.97257062	3.42382863	2.92124903	3.07049761	3.35475365	3.21032119	3.01966474	3.32261459	2.84190423
NONHSAG054109	2.92843052	2.54574385	2.61425046	2.67575766	2.64737333	3.08734048	2.84150952	2.52184706	2.53237025
NONHSAG054140	2.78024813	2.6774464	2.63463575	2.61273637	2.60741026	2.61019986	2.80748608	2.68605803	2.60665368
NONHSAG054179	4.20374407	4.40364697	3.6671127	4.00137286	3.82755049	4.15179699	3.93013539	4.36949269	3.83805648
NONHSAG054188	3.31385347	3.49675	3.41184711	3.27459614	3.41636622	3.57682193	3.4802234	3.56984194	3.39476505
NONHSAG054199	6.05351228	6.04706464	5.73488	5.91487713	5.97678259	6.0860587	5.89208102	6.29843802	5.83374919
NONHSAG054326	2.67461104	2.70371448	2.64019118	2.70405051	2.67081411	2.83637642	2.8574919	2.75463198	2.70397207
NONHSAG054344	2.50162337	2.2637188	2.07471583	2.34973544	2.07002217	2.24810203	2.06791383	2.1117169	2.38003652
NONHSAG054345	3.36952624	3.176963	3.398488	3.53451615	3.39199311	3.63014519	3.45054513	3.3681663	3.34156619
NONHSAG054445	3.78352266	3.79325988	3.50073157	3.44193233	3.72858816	3.90607112	3.57892956	3.66221132	3.3116609
NONHSAG054462	4.21851331	4.77684066	4.44784413	4.39293756	4.16557169	4.48163538	4.52711445	4.58374953	4.30784413
NONHSAG054468	4.6440645	4.95603899	4.59423859	4.53101537	4.7099086	4.68139613	4.73509674	4.7278541	4.36140637
NONHSAG054478	2.50560163	2.8417443	2.65855406	2.81734815	2.80240111	2.45994549	2.66781207	2.65547844	2.70800288
NONHSAG054549	5.67050632	5.76892028	6.02555442	6.04392045	6.06017783	5.93528288	6.21651796	5.98364767	5.74977372
NONHSAG054576	5.09145432	5.64138489	5.630514	5.57286312	5.35465749	5.6194528	5.46398251	5.55293064	5.35554123
NONHSAG054653	6.21788694	5.82335156	6.09782755	5.93232541	5.87013072	5.90969746	5.79254714	5.8329072	5.8394431
NONHSAG054655	4.42309087	4.44093023	4.32881643	4.33904879	4.0225281	4.47064754	4.15027403	4.47328113	4.17440282
NONHSAG054664	3.22276141	3.18147176	3.03071774	3.12951681	2.8736689	2.92768681	3.04270763	3.04068697	3.17528464
NONHSAG054671	3.40698582	2.8328307	2.95398085	2.7628362	3.00266056	2.92050352	3.08122548	2.85357419	2.70514157
NONHSAG054679	2.41548205	2.75148624	2.6529397	2.70367733	2.51085299	2.97981224	2.59595877	2.81967435	2.5910099
NONHSAG054729	5.97941128	6.43663164	6.55190115	6.69018426	6.53509187	6.50375213	6.41210469	6.3596339	6.41803134
NONHSAG054754	4.37674665	4.0194718	3.50625813	4.07495182	3.59291058	3.85330005	3.86213056	3.37651929	3.74772747
NONHSAG054784	6.30376157	5.85592645	6.1036433	6.15428607	6.1744564	5.69925328	6.36553603	6.20097887	6.1240437
NONHSAG054798	6.26315257	5.41317811	5.25649009	4.99454676	5.00211147	4.66236986	5.88322964	5.05928018	4.94866993
NONHSAG054804	8.40303021	7.00296966	6.73461469	6.710506	6.98041638	7.00840389	7.46695378	6.71916804	6.6624527
NONHSAG054869	3.64643571	3.9249697	4.17757011	4.2543532	4.078689	4.12753097	4.1535755	4.34108076	4.31559271
NONHSAG054870	6.65900173	6.63864954	6.6834569	6.57335761	6.80950683	6.62103112	6.44073934	6.60185155	6.44231033
NONHSAG054897	2.22143203	2.39777124	2.44813063	2.43093095	2.44590942	2.47409326	2.49857751	2.59067476	2.13508602
NONHSAG054929	3.31322197	3.28025489	2.96960903	3.08949834	2.97727257	3.45421654	3.11738659	3.08864941	3.07871827
NONHSAG054954	3.07271616	3.18014707	3.00167497	3.07513493	3.06361311	3.2112485	3.20023696	3.1998883	2.95924547
NONHSAG054958	3.70430987	3.86518403	3.52073421	3.86400414	3.59364654	3.59117673	3.53523934	3.82122718	3.28739981
NONHSAG054963	4.94044358	4.6867759	4.73586818	4.75294699	4.66682662	4.63752143	4.7177515	4.94355756	4.57388068
NONHSAG055008	2.59769724	2.67060366	2.44695481	2.33879811	2.64986594	2.65933418	2.5560484	2.54155617	2.64834972
NONHSAG055019	3.12511641	2.97060434	3.04968791	3.01796039	3.14194432	2.85025622	3.19478023	2.97557751	2.80959805
NONHSAG055021	3.74231892	3.48818788	3.41450913	3.58078604	3.3551026	3.61637252	3.49728702	3.47946174	3.49012977
NONHSAG055050	3.15706802	3.06813885	2.99741263	3.15189752	2.97837065	3.06675098	3.18835504	3.35220173	3.33215703
NONHSAG055086	3.0180409	2.7496455	2.52820019	2.85370106	2.58919203	2.72958792	2.99898964	2.75816476	2.38393999
NONHSAG055105	2.49177467	2.16300162	2.32204579	2.33765111	2.25807461	2.37062124	2.38886636	2.29031084	2.34361846
NONHSAG055108	4.42216672	4.62742262	4.80213479	5.12940464	4.83563267	4.64791411	4.73226919	4.86640851	4.58095864
NONHSAG055153	3.32192672	3.000552	3.20344897	2.90972276	2.89549608	3.39104318	3.20359679	3.15755888	2.90106053
NONHSAG055204	3.84412766	3.52549015	3.76518034	3.47835522	3.41165461	3.42032718	3.43167694	3.2386745	3.20210044
NONHSAG055266	5.12677186	5.29085692	5.14480333	5.06740283	4.88135491	4.91365381	5.1054749	5.5034783	4.65559761
NONHSAG055280	2.74335163	2.62060773	2.49239024	2.25636051	2.45007513	2.76775675	2.7117188	2.78219153	2.48339749
NONHSAG055289	5.92901747	5.88841956	5.87744483	5.80429845	6.02754684	5.85922337	5.92629717	5.72085964	5.8529882
NONHSAG055301	3.64281878	3.6851354	3.21288132	3.52312587	3.35997178	3.57146453	3.4540249	3.61544066	3.12956763
NONHSAG055339	4.64116619	5.16307183	5.10278883	5.00214265	5.127782	5.13250656	5.12420691	4.93068645	5.04382124
NONHSAG055350	6.04099453	6.51255333	6.17846836	6.32972899	6.65505188	6.63583167	6.58253799	6.60009404	6.55386926
NONHSAG055367	3.64349462	3.6032906	3.20838068	3.31824721	3.56938386	3.45620238	3.30588344	3.62101701	3.33352528
NONHSAG055371	5.93547193	4.92991511	5.50191217	5.09572634	5.64968965	4.53785244	5.17155132	4.94131228	5.54858802
NONHSAG055384	5.17155132	4.76498979	4.61442335	4.6177904	4.7557035	4.72747847	5.06126317	4.78636122	4.56491513
NONHSAG055388	4.12438902	3.07762011	3.39615016	2.95225018	2.93973453	3.12168227	3.19322624	3.07918592	3.12050221
NONHSAG055401	2.77471399	2.68798112	2.48800382	2.74694288	2.69987207	2.68845527	2.80484544	2.7463818	2.80736921
NONHSAG055405	4.11930655	3.45224016	3.61469424	3.49646559	3.89329831	4.04882597	3.84432689	3.66674288	3.69722491
NONHSAG055409	4.05138394	4.50469236	4.90872869	4.18384938	4.04417036	4.36966957	4.20375909	4.22291196	4.07248561
NONHSAG055436	5.43689434	5.14721184	4.95784416	5.10745168	4.8514212	5.04892898	5.29255217	5.09226355	4.91988474
NONHSAG055443	2.86101844	2.33332395	2.37371181	2.55642486	2.30369564	2.25561142	2.59595877	2.48440766	2.44813063
NONHSAG055445	11.11980363	11.08976339	11.4594347	11.33007518	11.28597222	11.13742343	11.26402073	11.3891034	11.41063065
NONHSAG055446	2.83159023	2.48570786	2.38893462	2.12973381	2.64737333	2.46149125	2.43953626	2.42307321	2.69624694

NONHSAG055449	5.99775568	5.81469572	6.1974627	6.25617156	6.19115168	5.83887654	6.12052974	6.46263703	6.11658013
NONHSAG055454	2.49954155	2.68246511	2.43224084	2.39972186	2.39972186	2.43904531	2.77244223	2.73838304	2.4888691
NONHSAG055455	2.84452341	2.68777687	2.75748872	2.87677945	2.89822039	2.80959805	2.7960981	2.77804411	2.87612284
NONHSAG055463	2.87752283	2.8922389	3.06783623	2.76009381	2.86068494	3.42186006	3.02662892	3.04429772	2.94914717
NONHSAG055544	5.4453162	5.5406658	5.08604698	4.84921349	4.959754	5.29219544	5.41733552	5.22469495	4.96643969
NONHSAG055550	2.60536652	2.59373559	2.68794269	2.53262614	2.53978134	2.60881913	2.55861854	2.45476415	2.71335822
NONHSAG055620	5.251193	5.49367436	5.86910217	5.74160075	5.79669226	5.69077754	5.82395179	5.51237181	5.84868403
NONHSAG055690	3.73871808	3.46430111	3.46683696	3.75660126	3.43308927	3.81926893	3.51916186	3.76849618	3.5097796
NONHSAG055699	3.6135127	3.67155279	3.42429163	3.59226368	3.47440477	3.80824614	3.5686371	3.79496075	3.43451139
NONHSAG055722	2.80336783	3.00900547	3.00046731	2.8535482	2.93449988	3.06656222	2.76154953	3.00436036	2.75859419
NONHSAG055783	4.58538913	4.84855663	4.61475536	4.64291164	4.48206062	4.74710118	4.4910142	4.83473386	4.41260344
NONHSAG055861	2.7383613	2.44084025	2.69761574	2.60041742	2.39399428	2.81530701	2.67323847	2.54187873	2.49844653
NONHSAG055878	3.85423393	3.99529163	3.68285275	3.61024691	3.62296326	3.67812974	3.6540601	3.87088917	3.55759298
NONHSAG055883	5.1121875	5.60624027	4.63207905	5.12608261	4.90390859	5.80368799	5.23578142	5.51244604	4.52109632
NONHSAG056024	3.71016268	4.03221508	3.54855434	3.64953254	3.53330953	3.88664417	3.61966919	3.79294319	3.5194079
NONHSAG056026	2.33067793	2.11702737	2.12469803	2.14783397	2.18018158	2.20779171	2.36018131	2.28697147	2.04426071
NONHSAG056029	4.48387196	4.26047234	4.29484123	4.34126248	4.27830338	4.38385916	4.33900662	4.29349212	4.30854983
NONHSAG056034	5.34181244	5.43578897	5.27255906	5.26772506	5.3349611	5.28921751	5.12819212	5.08379493	5.21835159
NONHSAG056037	7.65378306	6.38364648	6.26656233	6.24626127	6.37924014	6.3536588	7.00091434	6.42382418	6.08108585

	E2_ethanol_9	E2_ethanol_10	E2_ICI_1	E2_ICI_2	E2_ICI_3	E2_ICI_4	E2_ICI_5	E2_ICI_6	E2_ICI_7
NONHSAG000057	5.20384826	4.9966805	5.04923429	5.3156515	5.57139326	5.27115358	5.44006048	5.72813141	5.31495987
NONHSAG000058	5.55806989	5.15365283	6.26909072	6.51616075	6.19192545	5.95520395	6.32880476	5.94834496	6.02641238
NONHSAG000070	6.82708081	6.74066141	7.03337437	7.04829664	6.88403945	7.00368511	6.89627786	6.95549075	7.06455862
NONHSAG000071	5.26114727	5.44525616	5.5018394	5.44968033	5.5452787	5.43552362	5.48639405	5.31763367	5.45420734
NONHSAG000075	5.97816416	6.0092251	6.29233179	6.26394525	6.39651457	6.32933243	6.24867421	6.29217773	6.39165235
NONHSAG000111	3.81218921	3.90418143	3.97165671	3.87292323	3.98415042	3.90879892	4.01712031	3.82671668	3.81275759
NONHSAG000113	6.25252508	6.34377551	6.14461117	6.06673419	6.01310534	6.04316632	6.06322643	6.00151365	5.96917362
NONHSAG000117	6.24679698	6.11132138	6.71279857	6.7392428	6.55431843	6.79425148	6.60833503	6.71793514	6.66345114
NONHSAG000134	3.18980264	3.32906545	3.39638916	3.1007595	3.21637762	3.08109368	3.25327304	3.17363393	3.28983962
NONHSAG000139	5.59686445	5.87641025	5.89653796	5.42916409	5.67193222	5.53359998	6.04313298	5.57312909	5.71123936
NONHSAG000169	3.35779483	3.401671	3.60055663	3.20034822	3.48808469	3.15598348	3.86453173	3.24849481	3.46368912
NONHSAG000174	4.84580538	4.78688504	5.03097433	4.40451985	4.99961666	4.7688636	5.04108838	4.90650659	5.01211605
NONHSAG000181	2.81153414	3.0655647	3.02317295	3.09381359	3.26622718	2.70553273	3.27397946	2.92222081	2.92878664
NONHSAG000185	4.16220066	4.15072448	4.1464007	4.20591894	4.40696845	4.05436932	4.51237187	4.33919111	4.03604024
NONHSAG000199	6.38880449	6.51301159	6.57825162	6.23075091	6.48925063	6.18983484	6.6600315	6.28374563	6.37942597
NONHSAG000202	2.76052433	3.28107951	3.58081712	3.02722861	3.1960413	2.92361448	3.58511529	3.15765038	3.10826145
NONHSAG000215	3.54514504	3.44425882	3.69936919	3.49797506	3.76201759	3.40289587	3.57947314	3.54514504	3.35463399
NONHSAG000222	3.92583536	3.84131425	3.78452942	3.76436503	4.03222302	3.82036344	4.02182573	3.75361204	3.97116136
NONHSAG000254	5.74098159	5.72084458	6.26384702	6.49645529	5.84926852	6.16383718	5.8848918	6.21033441	6.39975148
NONHSAG000264	4.19359442	3.66186158	3.97645411	3.61469964	4.22610858	3.7755835	4.25024968	3.64856033	4.13310208
NONHSAG000267	5.19296153	5.10041303	5.2774585	5.140424	5.2513369	5.2356952	5.41308197	5.31005151	5.17416251
NONHSAG000310	6.47298953	6.37958832	6.55254448	6.6773452	6.73144497	6.77005949	6.49769724	6.90897179	6.68528899
NONHSAG000312	5.26017843	4.82331154	5.05674265	4.72755821	5.02181447	4.7773828	4.99258781	5.04309929	4.67317654
NONHSAG000322	3.2271022	3.13512226	3.23303181	3.17258447	3.33358406	3.32426637	3.51400126	3.07866768	3.08900396
NONHSAG000329	7.69282753	7.69545073	7.26997997	7.49248616	7.2432195	7.44138959	7.29026106	7.59275191	7.50790128
NONHSAG000345	9.0459	9.06845983	9.09425355	9.09507591	9.0395798	9.25054084	9.01915106	9.15537579	9.17229572
NONHSAG000364	5.54999825	5.34558111	5.56895827	5.55956523	5.85076989	5.74507246	5.80478989	5.62776565	5.51640837
NONHSAG000381	4.49319616	4.7374855	4.45806737	4.259565	4.46504377	4.14064372	4.77629151	4.36040536	4.39823349
NONHSAG000395	4.43489343	4.25837118	4.56848992	4.66405399	4.45911412	4.32295591	4.46344351	4.57809853	4.46578563
NONHSAG000423	2.47255347	2.62015541	2.89386335	3.13807134	3.05646254	2.50601893	2.50601893	2.58938507	2.94229999
NONHSAG000442	8.64820051	8.46419706	8.93259788	9.02809459	8.91563176	8.96197386	8.84202605	9.01590889	8.95293223
NONHSAG000476	5.91054352	5.86669218	6.00843523	5.84773516	6.30595867	6.00846039	6.40314534	5.95356472	6.11907283
NONHSAG000490	2.68667668	2.67610577	2.73497252	2.56128757	2.75700815	2.94323044	2.80949224	2.95477427	2.81359254
NONHSAG000522	3.06253257	3.03267449	3.4722479	3.07037948	3.3209277	2.94913994	3.41212174	3.12644489	2.86525433
NONHSAG000529	4.12095364	4.57188401	4.07047033	4.08863252	4.31448817	4.32325111	4.37978999	4.17214225	4.24022664
NONHSAG000546	5.19923511	5.59885109	5.3992091	5.32953271	5.32449242	5.26171014	5.27432864	5.30889819	5.29114709
NONHSAG000628	4.23239067	3.81051411	4.09976453	4.13528465	3.85856274	3.96403814	4.09804243	4.39623226	4.18308879
NONHSAG000648	5.86798701	5.50442893	5.65622981	5.77538511	5.54845893	5.42131269	5.50534351	5.68008097	5.6913286
NONHSAG000655	4.29169967	4.43211073	4.88165935	4.37148767	4.76191631	4.75947144	4.70334701	4.91077641	4.72296243
NONHSAG000666	5.20308804	4.98348132	4.92749128	5.24015553	5.21743268	5.09316056	5.01996877	5.47900067	5.36106537
NONHSAG000716	3.92668621	4.09976977	4.41707587	4.06365502	4.17707433	3.87528073	4.15058673	4.0273015	4.10408048
NONHSAG000738	3.99995054	4.09973088	4.12246306	3.80046228	4.23813254	3.90498889	4.03324268	4.10551575	4.31458391
NONHSAG000751	3.20875033	3.03370765	3.13169741	3.06553476	3.03326966	3.2611352	3.28964268	3.06780375	2.95107621
NONHSAG000769	4.47684276	4.62247158	4.79060266	4.50819525	4.5558061	4.44399275	5.02108621	4.50826428	4.58604797
NONHSAG000778	2.76077219	2.66465494	2.69515614	2.99325587	2.67075628	2.88879755	2.40160994	2.55302604	2.63478288
NONHSAG000812	4.12509762	4.27158658	4.45176637	4.21219469	4.30009628	3.95456302	4.17220164	4.01891672	4.19436909
NONHSAG000824	2.87461157	2.57329498	2.8854137	2.88855587	2.80807307	2.72267122	2.86474064	2.67571552	2.85164361
NONHSAG000847	3.94848154	4.00539844	3.99520184	3.69498125	4.02043051	3.66417042	4.08961947	3.83891807	3.87342845
NONHSAG000884	3.43057288	3.89806703	3.51048584	3.59842864	3.63155322	3.65075184	4.24664547	3.6296504	3.77246313
NONHSAG000939	4.09738856	4.03595207	4.30985383	3.49619451	4.05257538	4.24797125	4.09858785	3.9388201	3.97274433
NONHSAG000946	5.82659099	5.96528503	5.70431387	5.93676401	5.92433845	5.92789954	5.72339121	5.76236267	5.88549103

NONHSAG000965	3.34796071	3.44795141	3.33807428	2.9645294	3.3633418	3.17071785	3.59492467	3.28387623	3.28534007
NONHSAG000972	2.60520503	2.80647229	2.56544779	2.57866928	2.59458215	2.8004814	2.5089691	2.49069312	2.44666747
NONHSAG000976	4.55104408	4.65395115	5.40476569	4.87988283	5.12794357	4.94075976	5.22835694	5.25609721	4.92229986
NONHSAG000979	4.55689333	4.16255366	4.20292255	4.16193903	4.08175215	4.31501314	4.03555789	4.01837763	4.18132504
NONHSAG001012	8.65291242	8.87136554	8.89791828	9.24087205	9.05457317	8.94614966	8.66484212	9.23589966	8.99578308
NONHSAG001018	2.33889758	2.22985569	2.37656111	2.42307053	2.35649106	2.3416497	2.35310292	2.44871171	2.44619447
NONHSAG001041	4.76672241	4.82627265	5.21148436	5.35578868	5.27253286	5.25406376	4.82921049	5.33326596	4.90652951
NONHSAG001043	5.25798467	5.39348231	5.35487424	5.17156655	5.43496334	5.35038377	5.32928939	5.64559779	5.26208796
NONHSAG001075	3.52304699	3.40263976	3.57402562	3.43234943	3.7740545	3.46313559	3.51445665	3.5654891	3.19431388
NONHSAG001146	7.47136373	7.20790813	7.8305697	8.02047824	7.66316647	7.93803324	7.70476965	7.92311232	7.75730605
NONHSAG001183	2.33395009	2.41749107	2.50653213	2.48987728	2.54355018	2.35673635	2.58708051	2.5658127	2.62331631
NONHSAG001185	2.8460735	3.09731691	3.04838815	3.10249473	2.87575825	3.10954273	3.07021791	2.99277558	3.02955298
NONHSAG001211	3.43608726	3.34396728	3.34713866	3.29346165	3.51426508	3.04539905	3.57524188	3.2510155	3.26517257
NONHSAG001222	4.21115205	4.27575132	4.28012847	4.5585343	4.63186609	4.39477406	4.6354323	4.3832189	4.68313918
NONHSAG001244	3.16889755	3.31654443	3.40214626	3.15884119	3.59639622	3.12870934	3.73603023	3.32473438	3.26181399
NONHSAG001246	5.32876913	5.41377054	5.4017081	5.33909575	5.51329736	5.5359844	5.41810536	5.33513743	5.34818777
NONHSAG001247	4.90701126	4.92394025	4.97476116	4.57564695	4.77031489	5.02545918	4.94415199	5.13141794	5.06829261
NONHSAG001264	3.77678535	3.8790375	3.87309939	3.61625071	3.8661719	3.68793864	4.04903346	3.71440941	3.85271629
NONHSAG001304	3.25305657	3.52370134	3.30780187	3.21551951	3.03697864	3.00864105	3.06940389	3.27543508	3.00706224
NONHSAG001323	4.45719708	4.93512869	4.86854788	4.6119001	4.58139852	4.68239629	4.95206428	4.61844692	4.88647979
NONHSAG001341	3.05598866	3.09313458	2.82071596	2.97951626	3.06687588	2.71197231	2.980253	2.6515754	2.88064084
NONHSAG001391	4.31186997	4.31284528	4.61546029	4.21219469	4.44469688	4.21898684	4.6249135	4.36457696	4.34461666
NONHSAG001489	2.53839467	2.71802084	3.03255789	2.75830497	3.01678859	3.0490291	2.602327	2.76348287	2.67905926
NONHSAG001510	4.82853442	4.80765213	5.13362172	4.79652981	4.97150935	4.97213767	5.11880056	4.7368177	4.85633089
NONHSAG001530	5.42911931	5.55070812	6.62322579	6.73614227	6.29959343	6.87402555	6.65446572	6.54752343	6.63227065
NONHSAG001569	2.54362608	2.64082599	2.48639566	2.44111915	2.61501003	2.53436389	2.50538694	2.62974414	2.49855957
NONHSAG001574	3.7934999	3.64672416	3.7862578	3.66565627	3.94876758	3.83386336	3.88960711	3.74291366	3.99780145
NONHSAG001576	3.55650731	3.25749704	3.43565152	3.24435009	3.39377448	3.07738407	3.43137115	2.99069333	3.48747076
NONHSAG001587	2.85901655	2.69705587	3.06956566	3.21701306	3.28680024	3.51715254	3.16935285	3.3344125	3.25339803
NONHSAG001597	3.04045284	2.88478349	3.1290207	3.06316826	3.07682715	2.7620564	2.94499378	2.93948569	3.09551155
NONHSAG001601	4.13249463	4.09759195	4.45992815	4.04239552	4.49743257	4.10213574	4.49727923	4.3790285	4.23179056
NONHSAG001622	5.72339071	5.62674059	6.55470301	6.6497365	6.21206052	6.51598275	6.25718833	6.59608613	6.34036536
NONHSAG001627	5.02810885	5.06886763	5.21305145	4.97857257	5.37187591	5.13054927	5.0876769	5.15919534	5.11322634
NONHSAG001640	4.37190139	4.44811266	4.39828845	3.86354732	4.35183316	4.26765723	4.50135522	4.27721249	4.02315159
NONHSAG001660	5.02178166	5.12156427	5.29327578	4.83721194	5.04717801	5.08364492	5.19819021	5.1643877	5.02759444
NONHSAG001683	4.35184157	4.26000779	4.29760568	4.20323617	4.43288718	4.23088204	4.63255624	4.48633431	4.32013109
NONHSAG001696	5.85738177	5.88219912	6.25238685	6.27622087	6.3279463	6.42320076	6.26325504	6.27685839	6.13528892
NONHSAG001722	4.59720171	4.55509849	4.59623049	4.14720608	4.62773808	4.10927201	5.06849472	4.40154043	4.54626476
NONHSAG001723	2.89773504	3.1587727	3.78521902	3.28830045	3.39158532	3.47095089	3.44913044	3.41239633	3.23197386
NONHSAG001768	2.72437422	2.6832151	2.5720585	2.72056161	2.87071429	2.62948165	2.7711949	2.53935483	2.47953686
NONHSAG001788	2.25036231	2.36416252	2.39669622	2.51004616	2.54789806	2.4640597	2.46554681	2.43656857	2.5130166
NONHSAG001803	2.76492178	2.85280359	2.89137078	2.89945502	2.96500108	2.82753943	2.80954544	3.00108973	2.95650345
NONHSAG001824	2.81574717	2.73454996	2.80697656	2.52717487	2.83829367	2.4244876	3.23943091	2.51331428	2.6534061
NONHSAG001832	2.61484354	2.73842245	2.610935	2.51217086	2.63039169	2.68827576	2.85816588	2.50314384	2.78123143
NONHSAG001845	2.68130656	2.34188462	2.3306962	2.67647861	2.51026222	2.32873456	2.29044792	2.18277673	2.51079418
NONHSAG001849	10.56381812	10.45419699	10.8570676	10.79785669	10.77200557	10.81828084	10.97591173	10.88518704	10.73025958
NONHSAG001855	2.81336649	3.00376049	3.28076482	2.59595877	3.05381136	2.76519536	3.10715886	2.68012528	3.0711428
NONHSAG001898	3.15745816	3.13227391	2.98335511	2.97368109	3.26850031	3.05076084	3.15492413	3.10394566	3.0887301
NONHSAG001910	2.92438133	2.89896782	3.54258719	2.8063049	3.14198939	2.3990492	3.18506258	2.85561002	2.60110407
NONHSAG001920	2.69450257	2.59507447	2.78786312	2.82697358	2.69371305	2.59492433	2.67780323	2.62544656	2.62805154
NONHSAG001926	2.88677371	2.52104124	2.65698059	2.53137904	2.81525199	2.35962878	2.99402199	2.68376919	2.83576946
NONHSAG001938	2.88231686	3.25241063	3.39885303	2.99103193	3.22944395	2.71848761	3.15983002	3.01222944	3.06800802
NONHSAG001941	3.42001955	3.28909482	3.63516807	3.5533659	3.46437758	3.4075427	3.49426277	3.56930773	3.38701595
NONHSAG001965	7.54551129	7.35861201	8.4247188	8.6265627	8.57267187	8.48650194	8.40174321	8.51177453	8.53917507
NONHSAG001966	8.14113971	8.38394901	8.43796223	9.09190724	8.61740814	8.79434605	8.43817049	8.98851777	8.67913
NONHSAG001970	3.09004684	3.59729687	3.02133534	3.4340805	3.48711994	3.2456735	3.01221622	3.3456493	2.92337105
NONHSAG001984	4.54202511	4.34113351	4.27566651	4.34614322	4.31563263	4.36674643	4.72484314	4.15765416	4.28349853
NONHSAG001992	4.27541944	4.37349586	4.26115349	3.86989115	4.32117995	4.03268383	4.41195585	4.12946251	4.13476863
NONHSAG002015	3.32272901	3.11846077	3.82400263	3.55123551	3.71253871	3.07299272	3.51460245	3.32131688	3.651384
NONHSAG002029	3.05313462	2.81953636	3.09124245	2.82993883	2.95362313	3.05745156	3.07541571	3.13655814	3.08036042
NONHSAG002048	2.39793209	2.22764552	2.41027905	2.54626164	2.19205359	2.48299999	2.34666585	2.28303244	2.28692403
NONHSAG002049	4.16675887	4.1278645	4.55091261	4.31244807	4.34002802	4.40373083	4.56966492	4.24270653	3.99675245
NONHSAG002063	4.21666159	4.14384568	3.92640269	3.90217336	3.97777342	3.89857654	4.04717888	4.04417036	4.00086978
NONHSAG002096	3.83806947	3.91763848	3.827333	4.38524783	3.90113248	3.96687584	4.13586791	4.0901283	3.807092
NONHSAG002128	3.90333035	4.08228992	4.17815654	3.92294697	3.82490378	3.82758364	3.84121831	3.92358241	3.91685908
NONHSAG002154	2.37828893	2.27131952	2.33843432	2.41158592	2.39984386	2.44586402	2.16142683	2.26790923	2.09999579
NONHSAG002156	4.69335309	4.76851945	5.07518855	4.86237579	4.88947133	4.43876734	5.03282378	4.85385999	4.86078162
NONHSAG002177	2.73595536	2.8368031	2.76282232	2.72311921	2.69276528	2.59800415	2.9792602	2.56713974	2.69841917
NONHSAG002178	2.21419851	2.12008985	2.1169286	2.2492361	2.00299771	2.15008064	2.07742737	2.13538391	2.29062363
NONHSAG002204	3.36441011	3.40016794	3.19162686	3.60909065	3.53500282	3.61948704	3.27478509	3.65293661	3.62642196
NONHSAG002211	2.58119903	2.51786779	2.44525443	2.53920816	2.78154933	2.56637591	2.48844532	2.64111167	2.63766244

NONHSAG002218	2.40891147	2.25018362	2.31758954	2.40744934	2.56583127	2.32503379	2.68478866	2.29242612	2.3866413
NONHSAG002223	3.95315605	4.07972972	4.23649173	3.90760587	4.27363043	3.9137642	4.23281735	3.83251879	4.36578599
NONHSAG002277	4.85752971	4.55832905	4.58153899	4.64517477	4.7512579	4.01721271	4.77058431	4.50245749	4.65330175
NONHSAG002281	4.57048101	4.49895255	4.09560491	3.96936227	4.17057023	4.1592226	4.4645195	4.20154109	4.31823056
NONHSAG002337	3.91920136	3.95529398	4.22535584	3.73739174	4.11982216	3.7702327	4.09221689	4.3120539	4.24264957
NONHSAG002360	5.50146649	5.7952957	5.54317976	5.31938163	5.60315342	5.38645967	5.80478989	5.69167867	5.69616705
NONHSAG002376	2.44880037	2.50932197	2.30980512	2.19100395	2.45673939	2.21279717	2.33781603	2.31069711	2.36536164
NONHSAG002377	5.27608613	5.23363783	5.60895962	5.77162788	5.31641637	5.74211419	5.70574955	5.80922736	5.65047402
NONHSAG002389	3.21547552	3.14909411	3.56071464	3.33937883	3.614453	3.1439817	3.82221379	3.23423485	3.48442431
NONHSAG002392	2.74726982	2.64275709	2.68165186	3.18972731	2.61880517	2.76519536	2.82988184	2.7953196	2.80842861
NONHSAG002406	3.3586927	3.37510689	3.2409512	3.50994592	3.18974045	3.35093727	3.20090773	3.25269565	3.54049772
NONHSAG002410	4.98547967	4.63846844	4.89401887	4.87940328	4.78368253	4.94818758	4.74474351	4.91597515	4.80667889
NONHSAG002419	4.61572387	4.57715668	4.55633982	4.58334809	4.96633471	4.34903989	4.75523459	4.31986066	4.42989905
NONHSAG002421	2.80343196	3.25107568	3.16717653	3.02019735	3.27988243	2.96354061	3.08989557	3.42283039	3.2266241
NONHSAG002440	5.70224463	5.6648457	5.7106801	5.86185074	6.11505661	5.83186674	5.76466451	5.92285892	5.77665802
NONHSAG002455	3.08018107	2.99523803	3.20971644	3.35138956	3.32384734	3.16038497	3.25155209	3.00572528	3.16420432
NONHSAG002458	3.27832793	3.06485824	2.95462315	3.07438461	3.13476455	2.84142219	3.19728581	2.890247	2.95926155
NONHSAG002465	5.86529516	5.93310482	6.60402333	6.92171136	6.47983708	6.43232108	6.24180007	6.37657246	6.50114059
NONHSAG002516	3.1839878	3.14800141	3.23407445	3.18449402	3.11534716	2.94374802	3.10220565	2.87954229	3.19746829
NONHSAG002535	3.18264333	3.43755856	3.82222814	3.49855952	3.71031315	3.59542196	3.93885497	3.60344483	3.6965666
NONHSAG002538	5.1923567	5.45733215	5.2090349	5.25854976	5.38270361	5.20823179	5.49023318	5.21027755	5.34617826
NONHSAG002549	4.17203644	4.38665894	4.63650214	4.07527681	4.3317752	4.40969992	4.79246396	4.20040252	4.16974616
NONHSAG002555	2.81511687	2.73264637	3.05712104	2.83047077	3.1609969	2.77731401	2.79578863	2.74406065	2.78585373
NONHSAG002615	7.51891732	7.30527102	7.39483662	7.42584071	7.2643268	7.50613915	7.54010387	7.53744103	7.18551226
NONHSAG002728	4.3214219	4.34840384	4.33785159	4.24605395	4.28831569	4.55925773	4.09149436	4.551366	4.42611276
NONHSAG002758	5.11625403	4.97367096	5.09460148	4.997542	4.81663066	4.87885668	4.91220378	5.19315746	4.91041399
NONHSAG002759	2.94328282	2.80671473	3.14526059	2.72963744	2.94102823	2.7750334	2.94106882	2.93252387	2.99337154
NONHSAG002764	2.67260646	3.1715414	3.06743273	3.36694175	2.78246741	3.32076472	3.23503483	2.97052665	3.40300205
NONHSAG002857	3.65621364	3.49427978	3.8724092	3.41509723	3.62449716	3.47191352	3.90398241	3.6255769	3.69245193
NONHSAG002865	3.58223928	3.79217674	3.78020129	3.62730026	3.93047997	3.81195992	4.3828852	3.84451716	3.89155332
NONHSAG002891	7.17912318	7.10865578	7.12675257	7.36962857	7.18700148	7.07176485	6.92080387	7.1082428	7.29930546
NONHSAG002908	6.67902615	6.7182806	6.46323434	6.49552563	6.29379348	6.48536471	6.41690217	6.30437075	6.66875395
NONHSAG002936	7.66719642	7.52957454	7.93746039	7.90066959	7.91818063	7.83198587	7.87604011	8.04952598	8.04930684
NONHSAG002956	4.06200923	4.00597404	4.03791439	4.15509357	4.16659914	4.03352268	3.94390039	4.07513848	4.12504517
NONHSAG003018	4.9322617	4.80672299	4.90834566	4.62398363	4.97116924	4.76515768	4.66309446	4.66397719	4.82713776
NONHSAG003022	4.60005697	4.72832604	4.69586936	4.64728766	4.78007713	4.46610387	4.84062226	4.853799	4.55955496
NONHSAG003057	7.01098634	7.37810585	7.10905564	7.17322697	7.15290995	7.03365793	7.37777534	7.0871423	7.26004807
NONHSAG003089	4.4059359	4.5743609	4.54089147	4.10771724	4.18813879	3.90267684	4.66933354	4.21857314	4.35153068
NONHSAG003123	2.57828596	2.46715638	2.66019226	2.55293905	2.55340825	2.73546936	2.69417188	2.74075008	2.62181386
NONHSAG003195	3.68413034	3.64187325	3.84783211	3.64675076	3.87388305	3.6579683	3.8597309	3.68389995	3.74293792
NONHSAG003198	3.26559874	3.05120137	3.16342542	2.97194321	3.28604795	3.03543659	3.23036406	3.06865849	3.01651262
NONHSAG003202	7.25835977	7.12345658	7.21805711	7.47489656	7.1539199	7.3268978	6.93348815	7.18855963	7.1333148
NONHSAG003261	3.83135621	4.17576742	4.05449408	4.25293805	4.05840505	3.57383114	4.14764782	4.17771163	4.06517707
NONHSAG003289	2.97670872	2.76019135	2.73312679	3.01924559	3.0940757	2.68979886	2.75260584	2.81558538	3.01634956
NONHSAG003322	6.47330601	6.29965597	6.04320603	6.0693309	6.0434067	6.07009784	5.91432503	6.76726383	6.36525286
NONHSAG003325	5.92815384	5.62241734	5.89725296	6.3933948	6.01867533	6.12761704	5.55947873	6.92066942	6.61192452
NONHSAG003332	3.74505159	3.83276083	3.86507005	3.4847686	3.80748152	3.78180264	4.13458175	3.62750615	3.78383435
NONHSAG003339	2.56197472	2.74252357	2.59610115	2.14470741	2.51429976	2.3079806	2.49275013	2.48001808	2.5283111
NONHSAG003345	3.52944488	3.50622379	3.91825042	3.63493802	3.81312805	4.72386691	3.63984722	3.90717708	4.05341258
NONHSAG003405	3.0395665	3.11992793	2.97889628	3.18923234	2.9639712	3.10522582	3.13232148	3.06636895	3.18568346
NONHSAG003407	7.23885191	7.42079977	7.3563087	7.29777397	7.55071193	7.34916677	7.52086453	7.64454841	7.55612379
NONHSAG003412	2.44265389	2.45299657	2.43706457	2.38549829	2.36085876	2.37402683	2.51351119	2.64254233	2.41647421
NONHSAG003464	3.64550985	3.68167257	3.72409066	3.48706621	3.92863584	3.88350036	3.82983733	3.68293122	3.82312181
NONHSAG003486	5.46908961	5.25035465	5.48507828	5.55082927	5.3973428	5.11243902	5.81597885	5.19190913	5.76862617
NONHSAG003512	4.29400691	4.31488451	3.34557394	3.8832507	4.27403398	4.37182086	3.26412434	4.62070441	3.91987033
NONHSAG003533	3.77751435	3.63704121	3.89867051	3.67724609	3.93711789	3.50805978	3.96472075	3.75892114	3.55911804
NONHSAG003536	4.0552001	3.90723448	4.06969248	4.15397724	4.00429143	3.55114834	4.22838154	3.91057753	3.99195794
NONHSAG003539	3.05299312	3.04356588	3.40597483	2.94797229	3.20142854	2.84229511	3.21857203	3.07673965	3.17894171
NONHSAG003554	2.5279982	2.42774973	2.59172827	2.46468124	2.4991649	2.48159724	2.4985161	2.29092097	2.5284882
NONHSAG003555	5.83985298	6.02442843	5.50875999	5.73464337	5.34200822	5.71698081	5.75531049	5.92653713	5.73583433
NONHSAG003562	3.03159263	3.08944047	3.09832709	3.02900037	3.19113495	3.36277549	3.13836794	3.55411916	3.10935466
NONHSAG003567	2.33371537	2.25428426	2.22958818	2.19993992	2.19915336	2.18073179	2.25402562	2.24793802	2.38064075
NONHSAG003604	4.42426359	4.34095847	4.33597986	3.89710836	3.86665871	4.21576907	4.0998973	4.1636803	4.2825963
NONHSAG003613	4.61005051	4.44238792	4.59271927	4.60183147	4.54306614	4.45822118	4.73770948	4.29991228	4.57892093
NONHSAG003615	2.79585372	2.77337369	2.77506636	2.76519536	2.68636709	2.58689714	2.99887097	2.65558311	2.78691094
NONHSAG003627	4.15673122	4.03186115	4.20222038	3.84607048	4.15558262	3.69537131	4.49322179	4.00107009	4.05739609
NONHSAG003635	4.79917375	4.52842189	4.70511186	4.43737286	4.66807413	4.87246277	4.49188862	4.50860632	4.53096728
NONHSAG003645	4.79788686	4.62899958	4.65542971	4.60488528	4.70008277	4.69503064	4.65231187	4.76099267	4.75574822
NONHSAG003668	2.78950034	2.85230144	2.62161905	2.76519536	2.68170365	2.64785055	2.9971842	2.58847837	2.4098997
NONHSAG003758	2.2339448	2.163931	2.24574855	2.40831269	2.26122704	2.46418231	2.21302154	2.14999818	2.14093846
NONHSAG003765	2.27831231	2.4176203	2.41019614	2.42998025	2.35247593	2.48121543	2.31637566	2.09392791	2.23218735

NONHSAG003799	3.04007534	3.0893339	3.10136385	3.0823834	3.40186992	3.17488725	3.17394891	3.0152615	3.23884284
NONHSAG003829	3.487027	3.53034354	3.65070443	3.71118566	3.61487992	3.80705163	3.72008246	3.45962994	3.59426742
NONHSAG003840	3.00718214	2.92841389	3.16903589	2.81910095	3.03860258	2.75594296	3.33683256	2.80281979	3.12643233
NONHSAG003843	2.16382324	2.27975683	2.29516704	2.26067581	2.23477215	2.22050905	2.2888674	2.18398097	2.16341674
NONHSAG003854	3.17554916	3.07121401	3.06478235	3.12876169	3.21585829	3.10002551	3.03387943	3.17431587	3.01155141
NONHSAG003880	5.28171906	5.11591375	5.4150834	5.17873963	5.20479435	5.06507016	5.1475675	5.19041064	5.14028486
NONHSAG003894	3.36040343	3.59011733	3.73642733	3.76039417	3.79125183	3.40667121	3.49093096	3.48024881	3.60943673
NONHSAG003900	2.81773741	2.92761575	2.82749787	2.8084841	3.01450647	2.85337737	2.7929871	2.94689356	2.9540913
NONHSAG003927	6.18012732	6.3810736	6.31856952	6.30986465	6.29011433	6.33793376	6.27607359	6.3650934	6.54888877
NONHSAG004034	4.92762249	5.22272023	5.08218676	4.54654568	4.96633471	4.58150853	4.91569553	4.84809979	5.03137838
NONHSAG004049	2.56644878	2.60048687	2.69525594	2.3265701	2.44733005	2.49125839	2.7100925	2.43333361	2.6849899
NONHSAG004092	4.37001917	4.46686391	4.2469154	4.07803523	4.38974482	4.35177722	4.75407112	3.97612004	4.2224933
C1orf132	5.81507643	5.69355482	6.93973403	7.04949118	6.76642532	6.88576347	6.58222154	6.93664402	7.15954021
NONHSAG004140	3.83120857	3.90340549	4.23192226	3.52468182	3.90568529	3.88204768	4.0666089	3.76261637	4.03536916
NONHSAG004156	3.41410972	3.40912847	3.43721015	3.80805396	3.3336898	3.42323217	3.55771932	3.47505386	3.49689538
NONHSAG004159	5.44364608	5.49932171	5.06222453	5.44597603	5.64366026	5.59647017	5.60776535	5.48904075	5.3293441
NONHSAG004205	3.24581734	3.046407	3.10842694	3.05151733	3.2439072	2.77276367	3.00398334	3.08391403	3.20804739
NONHSAG004221	3.94944774	3.46676776	3.43750643	3.17222784	3.61564993	3.62808269	3.70648639	3.27478973	3.10828904
NONHSAG004235	4.3172872	3.99221852	4.5704957	4.1968388	3.90579266	4.57044324	3.98419338	4.34842014	4.37348149
NONHSAG004261	2.91635316	2.95199194	3.12808358	2.88928821	3.02568048	3.00968755	2.94828854	2.96179227	2.92027953
NONHSAG004265	2.93483674	2.73851351	3.05101199	2.65688712	2.92267616	2.5515578	2.71929819	2.75554502	2.7461242
NONHSAG004289	6.07871451	6.23208694	6.90382707	7.67577132	7.22406307	7.34816811	6.91687887	7.6056981	7.15282658
NONHSAG004292	4.60829481	4.33139545	5.19251909	6.13280236	5.69616707	5.63169971	5.24558314	6.39529221	5.86958074
NONHSAG004319	2.99000047	3.04277682	3.28595116	3.10288669	2.80526286	2.84262577	3.21415503	2.93363251	3.17986278
NONHSAG004322	4.53444495	4.62152473	4.43904015	4.53065691	4.55434135	4.3054737	4.72052475	4.27978983	4.47633911
NONHSAG004342	5.19923511	5.50943519	5.33706039	5.27177593	5.41954937	5.30303222	5.39558761	5.25043785	5.44898068
NONHSAG004374	4.45327911	4.33729545	4.38333102	4.29212525	4.53628557	4.27762786	4.85487701	4.14348383	4.17587289
NONHSAG004468	4.59768721	4.72631213	4.36141361	4.34560558	4.5527884	4.41453534	4.54259985	4.52023794	4.4919747
NONHSAG004469	3.6196904	3.29675253	3.8924705	3.36771378	3.35012118	3.20636159	3.87794653	3.32788656	3.43158479
NONHSAG004473	4.24459194	4.31207079	4.48480168	3.94989978	4.67424736	4.26782518	4.88804757	4.35805216	4.40093061
NONHSAG004494	2.53547823	2.50986034	2.3985577	2.62466168	2.47921564	2.49360602	2.55220707	2.48086845	2.47583288
NONHSAG004522	2.69089441	2.75816935	2.56675138	2.66294737	2.6199912	2.75817066	2.79230183	2.71335822	2.4931044
NONHSAG004544	5.82756413	5.89319246	5.87625661	5.62394338	5.91350361	5.60066335	6.26155485	5.71993195	5.82267506
NONHSAG004554	3.04817614	2.6090914	2.68273309	2.57383521	2.51914372	2.35320434	2.63910011	2.35380795	2.59360085
NONHSAG004567	5.0013468	5.40490574	5.1329622	5.53088473	5.14278997	4.9513335	4.80881151	5.1825021	5.21598744
NONHSAG004569	4.43839754	4.3905521	4.22570027	4.14421293	4.23947348	3.81558492	3.90488337	4.67770854	4.14610679
NONHSAG004584	3.90536235	4.14822261	4.3428025	3.87478846	3.99318242	3.9984395	4.21461475	3.75333343	4.06108496
NONHSAG004588	2.92850712	2.82634701	3.16060482	2.92245185	2.94869708	2.98157012	3.17968827	2.7752451	3.02058594
NONHSAG004592	2.20965671	2.18725994	2.28593823	2.39627531	2.30263944	2.23992242	2.17930183	2.33974683	2.2141882
NONHSAG004614	6.11310495	6.06315323	6.62005508	6.43162796	6.56047966	6.46375495	6.4899952	6.18683427	6.37492294
NONHSAG004619	4.70027458	4.67375367	4.83080295	4.79834525	4.87149892	4.79766191	4.63136289	4.66760486	4.65115866
NONHSAG004632	2.61962412	2.64207894	2.8714037	2.98707186	2.86164114	2.9517334	2.82088623	2.82687422	2.83576946
NONHSAG004636	3.92854274	4.04082595	4.11692558	4.01786961	4.06725138	3.82113168	4.00714583	3.92492065	4.17493429
NONHSAG004663	2.92519934	3.1022057	3.43917301	3.01627081	3.190232	3.16713205	3.56023772	3.01159054	3.10922091
NONHSAG004683	4.15485611	3.9215069	4.45176637	3.70375936	4.14133858	3.87826235	4.03105846	4.04417036	4.01048429
NONHSAG004693	3.06536459	3.59020038	3.14941863	2.92105388	3.19183739	2.98697216	3.41212174	3.04708413	3.3980024
NONHSAG004694	4.67934409	5.03139588	4.92600627	4.62103953	5.01528491	4.85150015	4.93375905	4.97994745	5.1665325
NONHSAG004774	2.82659965	2.72727192	3.00774259	2.6801049	3.12142931	2.65173628	3.36402878	2.68080123	2.74232354
NONHSAG004778	2.96976823	2.87944755	2.73155664	2.57419442	2.7249921	2.74243743	2.80697482	3.12099747	3.08153396
NONHSAG004794	3.93504012	3.78601902	3.91691951	3.82161671	4.10259254	3.80844725	3.98033889	4.16048446	3.87092537
NONHSAG004884	4.27589876	4.51267798	4.60608969	4.69016672	4.35461914	4.43182264	4.55331927	4.45009171	4.67392552
NONHSAG004887	5.0420609	4.86421836	5.16389654	4.9881017	5.0664203	4.5958639	5.46351377	4.80456117	4.88700002
NONHSAG004895	4.11611512	4.0647067	4.40684476	3.80109925	4.31438738	4.03704938	4.18318557	4.09455348	4.03838537
NONHSAG004899	3.60226366	3.89397043	3.92166494	3.55883559	3.90018385	3.77788746	3.63770466	3.83107466	3.71721232
NONHSAG004902	4.8851359	4.95794916	5.3448846	5.31877801	5.16477589	4.89706981	5.17587408	5.21796438	5.38548886
NONHSAG004908	4.97770915	5.06090166	5.27534366	5.06498421	5.03712466	4.73629587	5.17736564	4.64507276	4.89816002
NONHSAG004919	7.43966218	7.5114932	7.3084801	7.66817953	7.44047058	7.48172955	7.21075753	7.73499778	7.32610006
NONHSAG004920	3.20439187	3.20771805	3.0383612	3.02289605	3.23167411	3.01368918	3.12955366	3.24928992	3.0887301
NONHSAG004943	2.83240648	3.14760334	2.99942413	3.17587357	3.0822183	2.88445046	3.13617229	2.6673826	3.16985205
NONHSAG004961	5.14539879	5.1115513	5.153757	5.0956447	5.26374986	5.15014861	5.13973095	5.04881462	5.08058922
NONHSAG004977	6.18128286	6.35295056	6.282973	6.61338099	6.49562616	6.67417832	6.26568633	6.51915578	6.39580128
NONHSAG005016	5.68553256	5.671995	5.89725296	5.59141561	5.9048444	5.90021853	5.7072665	5.65168682	5.78224766
NONHSAG005033	2.26318323	2.38347532	2.68463106	2.18355009	2.40743799	2.12117351	2.383714	2.47752899	2.37918798
NONHSAG005060	2.31913916	2.4055141	2.57657254	2.46008016	2.6562389	2.40005265	2.54827715	2.72743502	2.58101413
NONHSAG005063	3.13401156	2.93308223	3.04778146	3.23992277	3.41707857	2.99681741	3.24480335	2.90731556	3.24182913
NONHSAG005080	2.6127174	2.42007754	2.26146685	2.9905655	2.7118724	2.85286423	2.42070254	2.36847569	2.62249989
NONHSAG005082	4.39018729	4.25356973	4.19384587	4.26770923	4.35392395	4.31697074	4.2618645	4.06887986	4.03557952
NONHSAG005119	2.72031756	2.33977222	2.64189328	2.87403741	2.71675924	2.69700048	2.8173929	2.79214444	2.79294225
NONHSAG005138	4.78931994	4.83147517	4.95208002	4.53435318	4.8031719	4.98430885	4.751048	4.46474312	5.09457554
NONHSAG005145	3.17689175	3.15167646	3.42922775	2.99902944	3.29868142	2.82171308	3.09854194	2.87396237	2.92198933
NONHSAG005150	4.91822166	4.72451014	4.73305454	4.76957835	4.93235374	4.57420221	4.65328418	4.64910523	4.70667506

NONHSAG005165	5.01060014	4.92106778	5.12827733	4.64417086	5.10790493	5.2913796	4.81695004	5.14657531	5.07959477
NONHSAG005205	2.79655642	2.9456001	2.74879229	2.74178081	2.89427957	2.75755523	2.98175178	2.64223861	3.03244692
NONHSAG005207	4.62145842	4.86598458	4.71194508	4.43813186	4.4124186	4.45897418	4.7813546	4.42620968	4.48496447
NONHSAG005225	3.42575042	3.53142076	3.31224505	3.31742303	3.35370888	3.16338261	3.37027431	3.23377148	3.27548475
NONHSAG005226	2.98065431	3.34485391	3.34774071	3.23011751	3.25610196	3.14226747	3.24710977	3.02269521	3.23352893
NONHSAG005232	6.15492485	6.07591249	6.29830119	6.48276447	6.11896931	6.2852353	5.74992151	6.10957069	6.39746956
NONHSAG005234	4.03260115	4.007743	3.97704648	3.80805396	3.93769047	3.83486599	4.17884657	3.80179067	3.89155332
NONHSAG005239	5.4367781	5.4146279	5.15141292	5.47352914	5.25712833	5.11343546	5.25361358	5.2111426	5.40468915
NONHSAG005282	3.69099523	3.51090092	3.46900553	3.35652629	3.74194496	3.46685292	3.78094954	3.66755095	3.50791052
NONHSAG005284	3.73345167	3.21944737	4.0792023	4.45463075	4.15718819	4.42166581	4.01738938	4.77658136	4.76792948
NONHSAG005342	4.33733072	4.57188401	5.15582555	5.19787927	5.24933884	5.48716423	4.82769283	5.17321431	4.93560755
NONHSAG005356	2.76387621	2.66406827	2.91540615	2.61582194	2.93509549	2.58115693	2.79169096	2.7791954	2.81764861
NONHSAG005375	4.5953012	3.9379754	4.32398248	4.44679709	4.00335846	4.22454819	3.81954921	4.75892214	4.40319367
NONHSAG005386	3.72292489	3.39817268	3.68357998	3.68876125	3.53541363	3.15775256	3.62542609	3.43958554	3.55420571
NONHSAG005407	6.30744724	6.1786081	6.42062389	6.34899329	6.32487364	6.18116805	6.3584683	6.35211635	6.42072672
NONHSAG005420	2.96644176	2.83852338	2.78607885	2.75927093	2.90793711	2.59268305	2.79730012	2.85427019	3.13475895
NONHSAG005464	5.13164952	5.50338708	5.77033348	5.09369016	5.57694488	5.02116018	5.57095448	5.25714061	5.51831907
NONHSAG005471	2.80991388	2.76641893	2.80614918	2.93047979	3.01119802	2.67507554	2.77037605	2.9234291	2.85344178
NONHSAG005501	4.04972317	3.94244589	4.10183323	4.13230708	3.93769047	4.17551843	4.08225002	4.35432505	4.27885451
NONHSAG005503	4.3155818	4.06625902	4.24266819	4.77731933	4.48858568	4.66410688	4.17321008	4.9884066	4.51737279
NONHSAG005505	2.59179325	2.33725038	2.39278699	2.20583642	2.3784839	2.17751932	2.56607768	2.41425829	2.31248931
NONHSAG005523	5.79584369	5.66654766	5.71334428	6.34211358	5.73871157	6.06683293	6.04808324	5.95010359	5.93235211
NONHSAG005543	2.95021958	3.08924135	2.8977894	3.00032232	3.18071497	3.15035186	3.24723587	3.37960904	3.18766445
NONHSAG005545	2.72776873	2.92695035	2.80843477	2.78362604	2.73569095	2.64536757	2.87217389	2.61379671	2.81441137
NONHSAG005589	4.28149559	4.577833	4.59391008	4.7351849	4.64191509	4.4634317	4.64946059	4.3247024	4.46317073
NONHSAG005603	3.40154616	3.44552203	3.09178679	3.0727616	3.43462935	2.94374802	3.28565884	3.42186006	3.16544305
NONHSAG005687	4.56319459	4.38506461	5.10129452	4.36724846	4.57160449	4.32590245	5.1313856	4.38842627	4.71652806
NONHSAG005692	3.18984338	3.30577697	3.47490315	3.60689582	3.33999764	3.02276744	3.2782948	3.4141069	3.44612099
NONHSAG005715	6.27677832	6.1744512	6.22015266	6.35351248	6.20845712	6.61704857	6.25229558	6.38428065	6.52800299
NONHSAG005734	4.25345371	4.17859674	4.48313615	4.19123014	4.31170322	4.13888738	4.36695409	4.23233113	4.27953531
NONHSAG005754	4.04482493	4.02200571	4.03127224	4.01586394	4.00297014	4.091102	3.67597256	4.13244694	3.98023909
NONHSAG005762	4.03688168	4.14385555	3.48566928	3.6504903	3.6590998	3.82281847	3.8181695	3.39304664	3.70374166
NONHSAG005767	3.23159928	3.25107568	3.46648032	3.29896866	3.28996071	3.32076472	3.35774288	3.33838176	3.28682159
NONHSAG005812	2.71921847	2.83366758	2.60275645	2.67295964	2.63664083	2.42906865	2.8990803	2.66215666	2.48151996
NONHSAG005834	5.3539431	4.83148821	5.2164705	5.1202919	5.29897939	5.15623945	5.23912882	5.43303442	5.5923402
NONHSAG005850	3.19277255	3.20673903	3.21292729	3.04992076	3.15361168	3.05537607	3.50614564	3.15626939	3.33359211
NONHSAG005870	4.667053	4.74752383	4.85862091	4.90330193	4.15155825	4.36705998	4.72990922	4.91551877	4.75883588
NONHSAG005912	2.33751005	2.34344516	2.39285932	2.58450459	2.67075628	2.83820591	2.56493396	2.68986825	2.66443481
NONHSAG005923	6.81984231	6.45082351	6.16069975	5.58691652	5.37625611	5.3481045	5.53683194	6.39198791	6.44957496
NONHSAG005953	3.73253148	3.75892179	3.88765536	3.46459818	3.67941765	3.69859987	4.12052523	3.81994594	3.50298577
NONHSAG005957	2.69575197	2.91871303	3.00623029	3.01302242	3.0053774	3.09414309	3.11775902	2.8260444	2.83326589
NONHSAG005965	3.11950127	3.07693834	3.17314556	3.07009017	3.45437583	3.18247437	3.3319974	3.11748902	3.20111396
NONHSAG005970	3.07331788	3.29717834	3.2276156	3.28900956	3.20659721	3.13113569	3.27981741	3.30937533	3.21362437
NONHSAG006053	2.54248827	2.36806489	2.41902311	2.42812302	2.26319944	2.70369716	2.46864878	2.48522782	2.3642288
NONHSAG006081	5.27252831	5.45326585	5.91887558	6.62791335	5.96154168	6.25328396	5.56358887	6.59533535	6.33858409
NONHSAG006083	3.74132915	3.159611	3.18170473	3.00400834	3.28604795	2.77034405	3.18545253	3.42364771	3.03479614
NONHSAG006087	3.80057577	4.08793325	3.95843994	4.64201907	4.42274884	4.24584085	4.04647426	4.34002103	4.14929603
NONHSAG006140	3.19684042	2.81328775	3.054736	2.97344446	3.2304645	2.94374802	3.27049254	3.00598437	3.26517257
NONHSAG006147	4.72455294	4.67024691	5.09785654	4.56064693	4.94627648	4.47768594	4.82516178	4.6884566	4.69722531
NONHSAG006149	4.58169634	4.58621239	4.76657146	4.97148019	4.92434988	4.7904732	4.78531083	5.1503664	4.64247273
NONHSAG006187	1.93550837	2.05835828	2.07661189	2.24487385	2.11005626	2.06987415	2.12420029	2.10145492	2.09792751
NONHSAG006196	5.43829676	5.49233723	5.34664696	5.28915302	5.56179805	5.63263543	5.29487657	5.54929429	5.40445102
NONHSAG006210	5.75222753	5.51204287	5.63483495	5.63447268	5.37110251	5.53278142	5.62369659	5.38894868	5.41714488
NONHSAG006244	3.09124491	3.15527132	2.86719917	2.86819982	2.8851899	2.90907091	2.91743843	3.37895838	3.45034828
NONHSAG006270	2.71878477	2.82993209	2.85268492	2.82293711	2.93328602	2.93329317	2.71522692	2.7498961	2.83576946
NONHSAG006274	3.19072541	3.12192744	3.1374514	2.89057758	3.21322555	3.06384099	3.29425141	3.04978356	2.95367772
NONHSAG006311	3.72906669	3.19751793	3.42656599	3.38315585	3.77585244	3.64377613	3.59311394	3.57744649	3.46183072
NONHSAG006312	2.88108721	3.29670073	3.0285603	2.82289363	3.07958993	2.958229	3.23669985	2.7410371	2.82050778
NONHSAG006326	3.67143091	3.74837255	3.83447282	3.67845338	3.86907092	3.79425173	3.75649297	3.75054298	3.8123542
NONHSAG006406	3.62934688	3.37256617	3.79671765	3.24605124	3.82395524	3.3899182	3.6398583	3.7267668	3.6102225
NONHSAG006407	3.98328959	4.12660736	4.24829171	3.91892179	4.21034916	4.0489308	4.39371669	4.08548726	4.10390048
NONHSAG006417	4.54469036	4.3345208	4.78373514	4.12616028	4.35238559	4.15911864	4.48054908	4.51865323	4.22802965
NONHSAG006458	5.09807499	5.06432971	5.02812503	5.08503845	5.29512551	5.00431723	5.11005553	5.26933406	5.33357237
NONHSAG006506	2.28479958	2.17865056	2.24900966	2.16969405	2.32245791	2.20275816	2.39580612	2.25526674	2.31206334
NONHSAG006557	4.12158469	4.17906223	4.35270656	3.95160908	4.11941154	3.89620837	4.55918458	4.38682705	4.34310967
NONHSAG006570	8.94537682	8.96605885	6.30484759	6.83287104	6.66720996	6.64580599	6.09049123	6.57836146	6.350681
NONHSAG006585	6.85540845	6.49932886	6.8968881	6.48275014	6.97823012	6.5178659	6.75877765	6.72871931	6.63851785
NONHSAG006591	6.0164484	5.31768704	4.82730894	4.84460545	5.20985831	4.81533185	4.75700675	5.42994719	5.31861255
NONHSAG006615	5.13023402	4.91452187	5.27829361	5.68195656	5.47736697	5.77962381	5.43623665	5.69741727	5.58467848
NONHSAG006619	7.2679307	7.17242021	7.48014979	7.72031179	7.52784841	7.60227147	7.27996998	7.63035666	7.52163651
NONHSAG006623	5.15478636	5.11255511	5.43373853	5.22239308	5.45372609	5.58616474	5.43074675	5.25841004	5.35029512

NONHSAG006631	10.26977783	10.24978244	10.44398617	10.27520045	10.51941743	10.25209265	10.78899238	10.53106993	10.46000851
NONHSAG006633	4.20141864	3.93600064	3.9273073	3.90170008	4.08768541	3.87464956	4.18178302	4.06030203	4.03762558
NONHSAG006679	5.86365541	5.85992801	5.96790691	5.82786308	5.92729982	6.08385971	5.85842974	6.08107364	6.1141038
NONHSAG006690	3.74139671	4.16875325	3.92013318	3.40704572	4.04780351	3.88217616	3.48592137	4.24842621	3.91338374
NONHSAG006700	6.56923977	6.72247307	5.44518866	5.39655775	5.06533254	5.35188008	5.07661173	5.23997023	5.15724079
NONHSAG006702	5.74578709	5.65100778	6.13101524	6.21035756	6.30477035	6.36873415	6.48559294	6.23915737	6.11922279
NONHSAG006706	4.48628861	4.50875411	4.32911659	4.43213078	4.63297807	4.73318484	4.72220666	4.30854076	4.66074538
NONHSAG006711	2.98525187	3.00974989	3.24249877	3.27852406	3.20487282	3.19175994	3.20983544	3.149551	3.13956056
NONHSAG006726	2.89706488	2.82916383	3.00267996	2.74150333	3.02193208	2.9986017	2.85393853	2.9139435	2.67769534
NONHSAG006750	2.35019668	2.38906321	2.24198433	2.46884972	2.49148777	2.14786987	2.54970679	2.34525021	2.30252051
NONHSAG006793	5.90466824	5.95848962	5.83341898	5.81525607	5.70235615	5.75017052	5.82214055	5.8125081	5.8133671
NONHSAG006798	3.19990756	3.1715414	3.61029674	2.93887209	2.99008177	2.91231051	3.48310385	3.08275431	3.16863795
NONHSAG006806	4.41267962	4.50282198	4.86506318	4.13425168	4.41588918	4.70664567	4.56380148	4.46209018	4.49867207
NONHSAG006870	2.69667839	3.00376049	3.13747457	3.54223469	3.14562493	3.29569133	3.1559077	3.09847451	3.12892372
NONHSAG006890	5.84874202	5.91014741	6.03487732	6.32166682	6.06062005	5.89542553	5.81684392	6.11029191	6.06829469
NONHSAG006892	4.9670361	5.06631498	5.13763712	4.93382116	5.1231034	5.16256963	5.14607869	4.98719029	5.3196087
NONHSAG006896	2.87263112	2.7543613	2.52237994	2.64712646	2.84764733	2.48194291	2.82446586	2.62506705	2.67210527
NONHSAG006898	5.57757157	5.17389244	5.8332551	5.83604883	5.66538141	5.54804523	5.48284351	6.23624413	6.00210902
NONHSAG006900	4.86781941	4.97886055	4.69058678	4.65119531	4.96484537	4.53793	4.78181863	4.89580075	4.58423129
NONHSAG006943	3.14862505	2.79087748	3.25425012	3.04432148	3.01093613	2.93893521	3.50457601	2.96824469	3.26184131
NONHSAG006949	2.40173474	2.29045815	2.47527659	2.3050095	2.42189804	2.38299565	2.66784238	2.23764431	2.26494659
NONHSAG006969	3.86164221	3.76027757	3.99308512	3.74332826	3.79961909	3.49172105	3.83498624	3.76285384	3.7158702
NONHSAG006971	5.13957913	5.14870142	5.64278243	5.66235581	5.43222499	5.69967506	5.42286404	5.55524296	5.54859329
NONHSAG006980	2.19074045	2.1670854	2.50188357	2.47363606	2.24046689	2.62567805	2.28866447	2.15266367	2.20264056
NONHSAG006992	3.66252041	3.81269736	3.81626101	3.88693538	4.11640473	4.02826396	4.12815375	4.18351932	4.23900904
NONHSAG007038	5.94677539	5.84499706	6.19566281	6.4021021	6.17795653	6.01091487	5.87143042	6.56226899	6.25934707
NONHSAG007047	3.15745816	2.86720302	2.10629859	3.00541957	2.76725874	2.77086141	3.01293326	2.61957604	2.37386911
NONHSAG007108	2.8025624	2.77526139	2.75779646	2.59243263	2.90433508	2.64117696	2.80045723	2.65199298	2.79365729
NONHSAG007115	2.32322248	2.31341318	2.21242843	2.36897074	2.24778015	2.30100462	2.30347336	2.42268386	2.16483919
NONHSAG007133	4.15219493	3.56439439	3.78162171	4.03533941	3.6144352	3.52958799	3.88562128	3.93245124	3.69427759
NONHSAG007156	5.62707379	5.10382546	5.41770266	5.23826679	5.30959892	5.21076524	5.23040579	5.47682992	5.39740059
NONHSAG007158	5.05562128	4.87526786	4.86776553	4.62413974	5.12573258	4.84545738	5.16125786	4.84993258	5.23145919
NONHSAG007166	4.11201788	4.30938295	4.14323122	4.20378797	4.20134709	4.54587546	4.07089925	4.51791503	4.3929277
NONHSAG007181	4.8608994	5.03635412	5.51807156	5.76280699	5.56919637	5.51656205	5.43681999	5.80952899	5.48969344
NONHSAG007207	5.07354618	5.29602978	5.52368504	5.36639929	5.23241214	5.130464	5.44405021	5.20108973	5.40263394
NONHSAG007221	3.56724248	3.78194961	4.15764472	3.73876178	3.69583227	3.6607314	4.2892809	3.56566597	4.06217002
NONHSAG007222	4.97934711	4.98484128	5.10943753	4.98543939	5.15126233	5.02863902	5.20734665	4.92787301	5.02763315
NONHSAG007253	4.6432851	4.73786488	4.93828584	4.52808283	4.87629277	4.87998989	4.72592622	4.96844273	4.77434444
NONHSAG007359	6.52866205	6.60895022	6.04634488	5.87396984	5.99460183	6.00890897	5.22241732	6.11311611	6.0611757
NONHSAG007368	5.3643007	5.45165519	5.57101259	5.19044315	5.54845893	5.61253197	5.36608326	5.39093383	5.30484995
NONHSAG007373	4.71519561	4.91761094	4.93861222	4.84107135	4.92586098	4.79113993	5.05811536	4.89092793	4.77274092
NONHSAG007386	4.61785147	4.96202345	4.89613643	4.35725493	4.92983454	4.65225085	4.54173584	5.12683904	4.72267096
NONHSAG007407	4.8312457	4.75771202	4.92177792	4.84010695	4.90025753	4.57331516	5.24114282	4.90535301	4.69665596
NONHSAG007504	4.65391162	5.0893719	4.64038584	4.7645986	4.93912355	4.74787619	4.63286017	4.66717085	4.52308154
NONHSAG007506	2.46827942	2.38876859	2.40656954	2.50773976	2.46150922	2.5161517	2.22556642	2.35251735	2.34162113
NONHSAG007524	4.34941247	4.04055314	4.14684316	3.69547393	4.22434129	3.85575543	4.05754049	4.45691582	4.12571785
NONHSAG007531	3.94628795	3.89297651	4.40820502	3.98023909	4.35973362	3.99695038	4.30392586	3.99052242	4.17657725
NONHSAG007550	4.20977501	4.77353237	4.64015868	4.48960625	5.02397643	4.61386791	4.86994363	4.78010837	4.97278956
NONHSAG007560	2.77227886	2.88440963	2.80160345	2.94908292	2.85647306	2.67087029	3.21679902	2.7572028	2.85889144
NONHSAG007562	2.73498138	2.77831494	2.56545251	2.70699512	2.78801669	2.687924	2.63597991	2.56424435	2.6372056
NONHSAG007577	4.39259931	4.21833924	4.31862274	4.19586831	4.36847143	4.34591386	4.59283857	4.34392955	4.45082737
NONHSAG007580	5.20515782	4.85596693	5.03165211	5.13619628	5.22762344	5.09791963	4.667857	5.04789957	4.62229135
NONHSAG007590	5.0788474	5.18153171	5.50074302	6.16152511	5.76131076	5.73428755	5.26653887	6.36402946	5.87856554
NONHSAG007653	3.50480956	2.99965289	3.50598549	3.23329623	3.25231884	2.90106539	3.75874573	3.33780048	3.4667221
NONHSAG007683	3.82257423	3.80445423	4.3121288	3.73827123	4.15753207	3.70287985	4.24186496	3.63309677	4.06858236
NONHSAG007686	3.93577115	3.86534121	4.05894678	3.84073953	3.91862355	3.64966627	4.11412473	3.824018	3.92329019
NONHSAG007695	4.38745364	4.3127045	4.39866723	4.37785431	4.37390724	4.40417593	4.42849472	4.32103145	4.36821277
NONHSAG007714	3.94980983	4.05890956	4.42412499	4.08644179	4.29163976	3.97601505	4.2511672	4.15959753	4.10318665
NONHSAG007751	6.46135357	6.14840233	7.04876493	7.03975591	7.03747249	7.20920874	7.05745586	7.09461924	7.02496066
NONHSAG007766	6.79425555	6.64694445	6.78529573	6.72126017	6.64774208	7.01048726	6.70045118	7.2246992	6.82364531
NONHSAG007767	3.6874112	3.5396838	4.0217907	3.90443559	3.64616146	3.74060053	3.79816785	3.81647032	3.71833782
NONHSAG007770	2.75734843	2.93024756	2.7364254	3.11970953	3.05183704	2.8853325	2.86244106	2.92710481	2.85265679
NONHSAG007825	3.41287979	3.86341903	3.79704204	3.6204151	3.73670386	3.38626796	4.12205141	3.79048377	3.70483744
NONHSAG007828	3.20776324	3.25517225	3.26645147	3.296785	3.27163761	3.16089437	3.16640924	3.14475823	3.33978777
NONHSAG007837	4.49255855	4.71665248	4.51977941	4.16130018	4.67909792	4.16590628	4.65176885	4.39051787	4.26310116
NONHSAG007839	4.79117989	4.90957463	4.99380486	4.90269331	5.00830215	4.99930104	5.13181469	4.81487456	5.25167438
NONHSAG007862	3.48588633	3.2955392	3.58081712	3.32903904	3.67987533	3.32079678	3.61085171	3.50260511	3.51949169
NONHSAG007868	3.01547787	3.66762381	3.55657752	3.15751559	3.42801185	3.20348948	3.51676891	3.08040501	3.20731789
NONHSAG007885	2.59930686	2.57752465	2.58908599	2.59487196	2.56197472	2.45689357	2.53758472	2.42542928	2.57972751
NONHSAG007890	2.9426424	2.97147406	3.25350254	3.23871547	3.0746072	3.10548016	3.25338387	3.08623919	3.07002585
NONHSAG007906	2.46587964	2.35124712	2.36168272	2.49692705	2.41782684	2.40617297	2.60695841	2.49670316	2.45287183

NONHSAG007910	3.66385596	3.63984085	3.70556385	3.5704822	3.67781908	3.48901098	3.67218739	3.60547269	3.52767106
NONHSAG007917	2.41233306	2.4787568	2.70954193	2.41842676	2.54480301	2.37175256	2.46118428	2.47668378	2.48187697
NONHSAG007923	3.3620031	3.36690875	3.54823039	3.3211365	3.53293373	3.28932189	3.39742437	3.29582676	3.40131139
NONHSAG007937	3.39904636	3.15020847	3.30418575	3.22862226	3.23190413	3.14268159	3.44498484	3.05580929	3.2885691
NONHSAG007942	3.17104728	3.09642961	3.50126284	2.96898173	3.01553921	2.99977525	3.3200196	2.90843808	3.18595885
NONHSAG007976	4.00736836	3.7857593	3.85387561	3.93848657	4.01499101	3.87442553	3.9362472	4.09251072	4.11508277
NONHSAG007987	2.7849462	2.86319571	2.86106692	2.81580383	3.13537723	2.85448658	2.85644004	2.57676681	2.98874416
NONHSAG008006	4.73512508	5.0850273	4.67631724	4.60779478	4.93256074	4.67053064	5.16431665	5.04791566	4.81353462
NONHSAG008015	4.48494812	4.62646726	4.4798519	4.36004692	4.53848271	4.25305329	4.8895482	4.53858327	4.28728816
NONHSAG008021	3.43066261	3.27551384	3.47886431	3.18385047	3.25377283	3.30184733	3.43428853	3.20549327	3.62111588
NONHSAG008036	3.48334658	3.39452642	3.64111864	3.27445517	3.76036732	3.19964228	3.81970207	3.25783684	3.27947409
NONHSAG008052	3.70666051	3.58108482	2.92400749	3.09046823	3.32351276	3.23586991	3.29178279	3.32284494	3.38455035
NONHSAG008061	4.61967634	4.15278089	4.46400248	4.43593118	4.23194012	4.06133502	3.99433952	4.26019615	4.1761926
NONHSAG008068	2.21672701	2.23753111	2.25981555	2.35857346	2.15914828	2.21672701	2.37573438	2.35130601	2.31399431
NONHSAG008074	3.6660652	3.73315434	3.97705612	3.66640787	3.77236703	3.59946588	3.74442073	3.72505083	3.6971354
NONHSAG008085	2.31130281	2.04549772	2.16032932	2.3114451	2.14896032	2.00240498	2.21336973	2.17627174	2.18236771
NONHSAG008088	3.18338523	3.07655152	3.20930731	2.77275486	3.2519478	2.95662996	3.17841552	2.88649663	3.1532787
NONHSAG008123	4.89814032	4.98953175	4.97197387	4.54156077	4.92909473	4.87541865	5.18824319	4.60555417	4.83036032
NONHSAG008125	2.48275094	2.40368769	2.47870466	2.39568109	2.5081571	2.56302492	2.76926607	2.52584962	2.65413033
NONHSAG008197	3.892543	3.93119675	3.74556673	3.55053768	3.83599581	3.54873071	3.80808003	3.56454656	3.69185997
NONHSAG008233	3.15002611	2.97287286	3.35401677	2.84764384	2.9296067	3.06598954	3.30187783	3.16671968	3.09127778
NONHSAG008613	3.69137942	3.67486243	3.62043788	3.51372048	3.69967463	3.60589614	3.83247346	3.64224636	3.81318838
NONHSAG008637	5.18404444	5.31684029	5.42670753	5.22750482	5.38878576	5.06482838	5.54885585	5.04990859	5.31385874
NONHSAG008664	5.21774529	5.29036725	4.7387846	4.70218261	4.87126772	4.57346599	5.08930265	4.87422676	4.72412152
NONHSAG008670	6.05289349	5.88356985	6.87416307	6.7303726	6.92760724	6.70783611	6.71303293	7.31953678	7.48876382
NONHSAG008671	9.8732551	9.41495476	10.74241345	10.75146644	10.82071057	10.53433537	10.6996034	11.50178502	11.30711765
NONHSAG008684	4.19772948	4.13030833	4.05372485	3.93244078	4.14855015	4.1662631	4.28381065	4.29461455	4.25886452
NONHSAG008765	5.30061267	5.10544524	5.25893213	5.42965772	5.49183537	5.26639889	5.4223209	5.70486967	5.35552954
NONHSAG008818	5.53527449	5.3280128	5.53088473	5.37661789	5.96493202	5.70699517	5.4120343	5.76466451	5.4650375
NONHSAG008819	4.94101386	4.67647866	4.24944401	4.44002051	4.67336014	4.31464927	4.82054759	4.66636991	4.43954626
NONHSAG008903	4.30864894	4.12073163	4.23779869	4.09096641	4.09175159	3.7536718	4.01181233	4.21340799	4.13482852
NONHSAG008932	5.10252304	5.23831275	5.33642318	5.01621162	5.44516544	5.28926983	5.38568838	5.19905609	5.28780489
NONHSAG008943	4.82540346	4.81458584	4.81929196	4.63104611	4.73656297	4.71745842	4.65644528	4.87666824	4.94449982
NONHSAG008986	2.1213476	2.1858366	2.10683358	2.18768416	2.15497503	2.16490215	2.59946798	2.37557343	2.17384639
NONHSAG009036	3.89137885	3.98050468	3.6581187	3.86796087	3.94250353	3.71891307	3.58106043	4.19810944	3.66786936
NONHSAG009072	6.54882676	6.54203741	6.44366985	6.36429387	6.64223599	6.2160198	6.62372512	6.32417531	6.48370011
NONHSAG009076	2.68522748	2.70424217	2.96107171	2.52762111	2.88299946	2.76323377	2.77771181	3.14670729	2.76440075
NONHSAG009130	6.91203403	6.71682412	6.49888884	6.45372859	6.37671428	6.44836191	6.22676751	6.5506995	6.47133224
NONHSAG009132	2.7165663	2.52774619	2.96698104	2.45826093	2.70232903	2.70471413	3.04697559	2.65156336	2.92510738
NONHSAG009142	4.68825205	4.9961826	4.87621105	4.96037582	4.83570175	4.94607727	5.02491676	4.79818419	4.85067118
NONHSAG009168	9.31368042	9.28025229	8.99353939	9.26146178	9.07460321	9.21262303	9.11209328	9.16860677	9.04892628
NONHSAG009184	3.01339348	3.25188743	3.18349939	3.36694175	2.96321592	3.54311851	3.07980562	3.41264035	3.19776202
NONHSAG009186	3.26243363	3.39498444	3.32805081	3.45302387	3.42633953	3.26798348	3.83560214	3.3886943	3.43390202
NONHSAG009191	3.27201715	3.17727336	3.45003084	2.6964887	3.01547241	3.10168234	3.0409859	3.27987869	3.3943006
NONHSAG009210	4.89509848	4.98669962	5.16883456	4.36835938	4.82044437	4.54996929	5.05327157	4.94752578	4.68017146
NONHSAG009216	2.80090505	2.98760213	2.84124701	2.62965537	2.61501003	2.54217606	2.59037601	2.85482268	2.80410677
NONHSAG009222	4.71204199	4.31598951	4.57146893	4.52601719	4.68356238	4.91036397	4.36394134	5.05802293	4.68520103
NONHSAG009269	4.6468995	4.51372346	4.55164691	4.49748324	4.54850336	4.38178812	4.83721553	4.28724789	4.45970414
NONHSAG009275	2.44849122	2.47644297	2.59600592	2.40120799	2.41906652	2.40608147	2.95292242	2.37395385	2.58612085
NONHSAG009283	4.60851778	4.43942789	4.94910767	5.19629107	5.04515045	5.17893474	5.04011459	5.1229708	5.07451895
NONHSAG009302	3.35372743	3.37017945	3.34565394	3.23575316	3.35100831	3.3172988	3.41780377	3.26517257	3.39688347
NONHSAG009308	2.63956394	2.8037115	2.72956637	2.43923617	2.71278474	2.21323051	3.1715414	2.36810139	2.59383089
NONHSAG009381	2.72919125	2.64401356	2.59798795	2.6133737	2.7160842	2.76519536	2.82535367	2.51852255	2.68844268
NONHSAG009397	2.89533424	3.0028466	3.09178679	2.842237	3.00053678	2.88401585	2.99431619	2.88478523	3.02957913
NONHSAG009424	3.45596472	3.52263191	3.51755888	3.98023909	3.34431106	3.26907273	3.54817365	3.52880685	3.44366287
NONHSAG009434	2.54059985	2.68674045	2.70007877	2.58591742	2.64310046	2.47855571	2.52083072	2.68169333	2.41117603
NONHSAG009452	4.16703521	3.92828613	4.44838755	4.45426633	4.28419684	4.58414448	4.11481643	4.4256862	4.51327352
NONHSAG009462	3.81855421	3.84597808	3.72199917	3.63091971	3.82352674	3.52601508	3.91999845	3.48851914	3.58770278
NONHSAG009465	2.88677371	2.80324938	2.71882361	2.8005632	2.97060434	2.81605886	3.23619954	2.6688351	3.13110697
NONHSAG009524	3.05887922	2.82626743	2.76351088	2.67767621	2.90000219	2.86664783	3.12325195	2.85419063	2.85406608
NONHSAG009530	3.15773654	2.9243678	3.2335271	3.22744931	3.04973717	2.98451077	3.25202699	3.17977769	3.13760083
NONHSAG009590	2.54134176	2.52901266	2.63568947	2.5669278	2.55193573	2.48208376	2.55898125	2.49907469	2.59789973
NONHSAG009627	5.46441266	5.73322214	5.52734281	5.47470227	5.65730614	5.52982805	5.42906425	5.65230358	5.45478435
NONHSAG009630	2.66195061	2.75936235	2.47742996	2.36206774	2.56400466	2.32980238	2.62948166	2.80179664	2.56431316
NONHSAG009643	4.29532922	4.12328059	4.99148392	5.28182061	4.94145572	4.98728465	4.74525108	4.78751895	4.79873103
NONHSAG009663	2.72561377	2.54889836	2.73687586	2.74616797	2.70292837	2.86854449	2.58666622	2.61071675	2.77164506
NONHSAG009678	4.05534916	3.96672986	4.23619464	3.89558602	4.10567655	3.98822595	4.35985932	4.09033187	4.26797209
NONHSAG009688	3.78028124	3.72438488	3.66836349	3.2334214	3.42644162	3.01844305	3.70049566	3.48002695	3.52609607
NONHSAG009690	4.89008958	4.7825476	4.82940653	4.57910426	4.90487044	4.62459795	5.11801321	4.79932405	4.95656324
NONHSAG009700	2.97332346	2.96031755	2.93576956	3.38397866	3.06250041	3.25278729	2.95730231	2.92168709	2.95068461
NONHSAG009711	2.99688935	3.02717689	2.98999085	2.80641994	2.92833525	2.73019474	3.20154304	3.09218051	2.95635637

NONHSAG009736	3.1274773	3.08024313	3.32328173	3.1584723	3.10939127	3.2222226	3.66600684	3.1662099	3.20580657
NONHSAG009783	3.05981797	2.91560538	3.0881009	3.01357824	2.97576181	2.69520654	3.22352506	3.08258337	2.76354122
NONHSAG009834	5.08054976	4.85221371	4.55738123	4.52925978	4.55304791	4.67521054	4.60692099	4.62497303	4.3190633
NONHSAG009878	2.33562602	2.25583884	2.34898781	2.36321258	2.33828695	2.25433864	2.30347336	2.33753748	2.27679873
NONHSAG009880	2.06361425	2.0854413	2.27663474	2.17821654	2.18958558	2.15051566	2.35927745	2.21722534	2.1685064
NONHSAG009903	4.30051923	4.47306443	4.40500065	4.42981349	4.53154905	4.28051427	4.44378027	4.34654057	4.63609687
NONHSAG009919	3.72805228	3.70786573	3.79781324	3.72585605	3.51424149	3.75034401	3.93786987	4.00899031	3.76565381
NONHSAG009921	4.20000975	4.15591083	4.37430193	3.89629552	4.46417492	4.28125616	4.06174949	4.17468485	4.17656081
NONHSAG009942	3.30125399	3.36164949	3.48624011	3.43003814	3.41426708	3.65093162	3.51577988	3.64116137	3.47960153
NONHSAG009977	4.34129096	4.40050698	4.55919592	4.21219469	4.58380911	4.36606771	4.5607089	4.57188401	4.45176637
NONHSAG009994	5.63456504	5.67557087	5.02974855	5.47422257	5.34846327	5.43552102	5.40430084	5.38048892	5.23921419
NONHSAG010005	2.36096287	2.62426984	2.60485877	2.55897996	2.63678075	2.70471413	2.64027586	2.67108701	2.47242583
NONHSAG010007	2.94662578	2.92677452	3.19710854	2.9190274	2.89924803	3.1235378	3.11458019	2.89915204	2.93764233
NONHSAG010015	2.67692407	2.51329453	2.67728707	2.49565369	2.75966809	2.23632447	2.81704522	2.48482599	2.57280422
NONHSAG010022	3.25270347	2.92939321	2.95247606	2.91883936	3.09698289	2.94691726	3.59384365	3.08491386	3.0087399
NONHSAG010026	4.48156383	4.25207444	4.46165645	4.26458476	4.47146382	4.13733575	4.66329227	4.30614704	4.26817997
NONHSAG010034	3.13595257	3.21250974	3.47099726	3.41017439	3.04408881	3.06919927	3.4286579	3.23050007	3.28491533
NONHSAG010061	3.86196164	3.76036449	3.95999135	3.89683207	3.80601365	3.89295593	3.8506174	3.82677659	3.9018619
NONHSAG010086	2.96937332	2.88345381	3.0345888	3.15737794	2.85387384	2.79444766	2.90935436	2.94036846	3.09363704
NONHSAG010089	3.21479102	3.05617937	3.22134353	3.24037706	3.09721531	3.17246178	3.10963485	3.23410021	3.44462293
NONHSAG010109	5.00920101	4.97844665	5.2236557	4.90174492	5.35556868	4.9671233	5.14593574	5.091681	5.11214137
NONHSAG010134	5.41722569	5.43860925	6.11066464	6.68046145	6.15320512	6.47220572	5.87149318	6.33740403	6.27808207
NONHSAG010144	4.3911291	4.34572558	3.91841841	3.98150838	4.30875703	4.21819636	4.69209651	4.01653054	4.2477356
NONHSAG010152	4.46612181	4.22122148	4.47641751	4.62958793	4.43188486	4.46058033	4.52914804	4.56576105	4.43057159
NONHSAG010157	2.446175	2.67095626	2.8896785	2.46718582	2.72762055	2.80025115	2.53548032	2.41297469	2.56530109
NONHSAG010160	4.06043978	3.73771097	3.64675076	4.03539161	4.0140228	3.83025078	3.53976129	4.10883325	3.92898747
NONHSAG010163	2.77331113	3.00185136	2.83547359	2.92753392	2.90959635	2.52609059	2.98957499	2.67548574	2.90496648
NONHSAG010226	4.16309858	4.31415349	4.26193225	4.34999665	4.17556251	4.56831464	4.28526296	4.26041046	4.39460033
NONHSAG010232	2.87452222	2.75453833	2.82776952	2.62619624	2.78246741	2.69650557	2.64284622	3.23187945	2.8758423
NONHSAG010260	4.69881767	4.78540443	4.99004785	4.34952302	4.88791384	4.61888325	4.66973155	5.01561416	4.4285076
NONHSAG010262	5.1068792	5.00654909	5.11718769	5.19710385	5.43917339	5.22739533	5.56811129	5.07070808	5.15003333
NONHSAG010264	5.03055881	4.89996727	5.17049459	5.41079013	5.24619886	4.82909658	5.06245004	5.31667585	5.06173135
NONHSAG010352	5.64842142	5.70139449	5.97451197	5.86637972	5.94596479	5.7983778	6.01226435	5.73427256	5.87704378
NONHSAG010413	2.10458739	2.15940001	2.20937395	2.18372444	2.2505147	2.05560619	2.44999407	2.28303244	2.50291905
NONHSAG010420	6.14630936	6.17793954	6.63710568	7.00245966	6.65780545	6.63632704	6.29180376	6.58695265	6.69530523
NONHSAG010447	2.54112929	2.59776974	2.69393879	2.34340489	2.56873144	2.40932342	2.95136399	2.35448602	2.45498607
NONHSAG010455	6.1347597	6.10224903	6.93480642	7.00463668	6.69677809	6.80800629	6.95415379	6.51305687	6.61365904
NONHSAG010492	2.87778062	2.94263171	3.04341577	2.82959156	2.90026299	2.62357041	2.95223185	2.83906716	2.97197431
NONHSAG010499	2.50608226	2.33084811	2.63974368	2.54782285	2.42814416	2.58363463	2.34116295	2.59352473	2.48388406
NONHSAG010524	2.3025599	2.40052525	2.24588345	2.46165409	2.45935319	2.54319197	2.61766525	2.45164703	2.38775493
NONHSAG010532	4.99766396	4.90164829	4.54236287	4.60585504	5.02667362	4.47667746	4.44436248	4.42037286	4.39213243
NONHSAG010566	2.44829268	2.56746052	2.49236648	2.41686049	2.36099562	2.50164979	2.40182736	2.37103807	2.27505509
NONHSAG010571	3.84339288	3.83924131	4.52796221	4.55607775	4.64242032	4.52146568	4.43641532	5.05885059	4.71822516
NONHSAG010573	5.98239159	6.31530989	5.70980757	6.22265971	6.03907392	6.03075776	5.7445687	5.98680441	5.68921915
NONHSAG010585	2.91810627	2.86489917	2.79811314	2.91955589	2.95604021	2.66370913	2.71395702	2.86207443	2.71144623
NONHSAG010628	5.09416594	5.21936975	5.08768905	5.46814153	5.25891204	4.79689048	5.41147735	5.1844697	5.05033735
NONHSAG010678	3.18501697	3.01818242	3.03163679	3.0437449	3.49993554	2.86928226	3.58130999	3.19583951	2.94806965
NONHSAG010682	2.91249463	2.68983783	2.76166178	3.19120979	2.89810577	2.78815768	2.8259785	2.93538509	2.75810479
NONHSAG010721	2.59073652	2.41680167	2.67013659	2.4392153	2.47689243	2.47505455	2.59893299	2.36427051	2.67428878
NONHSAG010724	3.23769855	3.01577185	3.05420626	2.94455064	3.25131901	2.71104503	3.27935636	2.97053721	2.88413631
NONHSAG010765	2.82821776	3.29766292	3.19955544	2.91293691	3.08815434	3.19498762	3.22424892	2.92683495	3.05106019
NONHSAG010767	2.92585016	2.94521947	3.33886227	3.13889565	2.95368116	2.78718381	3.10230317	3.02269521	2.93045909
NONHSAG010791	2.48130411	2.43026058	2.30692858	2.35656871	2.26685186	2.31979133	2.38400725	2.13598596	2.44619447
NONHSAG010808	4.48628395	4.72119658	4.58511762	4.37135715	4.55668377	4.37385355	4.44958064	4.36236845	4.43770668
NONHSAG010846	3.70745407	3.73288305	3.80856338	4.17937858	3.95102756	4.02957995	3.87350729	4.16176158	4.30247655
NONHSAG010848	3.63547329	3.41123872	3.95919182	4.17358954	3.87321432	3.49189257	3.9593306	4.45070008	4.06801097
NONHSAG010888	2.70173056	2.84620991	2.85323456	2.76391869	2.76729898	2.55876955	2.8403594	2.71174079	2.70468361
NONHSAG010892	3.63241868	3.57588155	3.3818917	3.3858814	3.69793621	3.80340188	3.0230219	3.80781855	3.57897585
NONHSAG010908	2.07400574	2.23244466	2.32931081	2.29174195	2.24570972	2.11140585	2.11751831	2.03229799	2.06222699
NONHSAG010921	3.18520046	3.20966802	3.73033769	3.28257958	3.61317596	3.40020739	3.64501641	3.17934876	3.21107023
NONHSAG010938	4.46660781	4.49300442	4.55571457	4.33221013	4.73288569	4.42402158	4.89108429	4.50987924	4.50440152
NONHSAG010946	5.53599042	5.58742973	5.53924045	6.06184934	5.49810585	5.95594735	5.58169545	5.63329476	5.57670425
NONHSAG010964	3.52927819	3.45758145	3.71283855	3.39138629	3.68279667	3.44771062	3.90301496	3.50841077	3.55721827
NONHSAG010989	3.41044909	3.43329399	3.35272814	3.51643903	3.50099726	3.11392761	3.40438751	3.1500325	3.14624988
NONHSAG010991	3.0704503	3.2163759	3.38270498	2.97696315	3.09781872	3.12843361	3.04697559	3.22546716	2.911164
NONHSAG010992	5.0992227	4.48731758	4.67095449	5.32340375	5.26107211	5.10518865	4.6853047	6.62342561	6.00315179
NONHSAG011000	4.73570584	4.4553315	4.66923287	4.41805763	4.58064703	4.46919923	4.85686139	4.91342705	4.36801783
NONHSAG011016	4.16925761	4.38384378	4.43353793	4.20807975	4.45023914	4.17398352	4.50453845	4.44576649	4.43491134
NONHSAG011035	2.83435028	2.62139867	2.77775779	3.59713293	2.70234759	2.54404996	2.59488339	3.02054744	2.80169489
NONHSAG011091	3.61944234	3.69922647	3.69268374	3.6884743	3.65038105	3.43981686	3.51042706	3.64789689	3.69544743
NONHSAG011182	5.58040565	5.51572251	5.22146265	5.48114586	5.32753037	5.21252022	5.50773827	5.36579075	5.48015115

NONHSAG011270	3.87825812	3.85655637	4.1227412	3.36963505	4.12522599	3.4585242	4.13385659	4.04818548	3.6625419
NONHSAG011279	3.75301823	4.09349986	4.14208224	3.38502684	3.55381158	3.37013981	3.84522715	3.29057697	3.65742961
NONHSAG011288	8.39219162	8.49544086	8.65463069	9.27353812	8.93879914	8.62923422	8.47000171	8.73498482	8.87968391
NONHSAG011425	4.58771136	4.14871712	3.80772834	4.97155044	4.13373856	4.48955363	4.49609025	4.80744279	4.15518703
NONHSAG011459	5.79493105	5.78414258	5.92502482	5.59613349	5.87524971	5.58961332	6.13278135	5.65828642	5.91168079
NONHSAG011460	4.94481827	5.55312781	5.66734762	5.00575629	5.53654523	5.31424062	5.44990492	5.51306021	5.42539875
NONHSAG011467	2.56197472	3.00376049	3.22395123	2.83416289	2.95583165	3.05550736	2.90019622	3.0149531	3.08604876
NONHSAG011471	3.49639148	3.5443611	3.76924488	3.42566977	3.38754869	3.45032003	3.44294827	3.35985874	3.3821797
NONHSAG011496	3.29348177	3.79024361	3.71058211	4.11411001	3.92889405	3.53489707	3.88923106	3.67357643	3.79940082
NONHSAG011553	3.35082269	3.39251217	3.46288547	3.39251217	3.4298482	3.30043917	3.62029986	3.19632334	3.32127102
NONHSAG011555	3.42275384	3.30298297	3.61427372	3.24596074	3.45759155	3.24453225	3.46340629	3.03593933	3.61548854
NONHSAG011584	5.02551772	4.96649407	5.03101079	5.16403687	4.88979058	5.29829623	5.06698304	4.93596034	4.99088399
NONHSAG011594	2.86363121	3.00376049	3.13409799	3.01627081	2.76904349	2.96836367	3.21076052	3.24025141	2.7962338
NONHSAG011643	2.76105142	2.52084539	2.89270851	2.76800096	2.77017176	2.43045445	2.5971184	2.6744826	2.57966068
NONHSAG011647	2.71835109	2.606389	2.61903267	2.73282926	2.73461744	2.35262479	2.73951985	2.53829821	2.43476509
NONHSAG011680	3.46086829	3.78179918	3.61187489	3.56249034	3.68985726	3.4786626	3.59764788	3.85178762	3.31016864
NONHSAG011703	4.43936545	4.31326439	4.79687795	4.55805728	4.62805835	4.39759514	4.97661483	4.67373689	4.29374138
NONHSAG011730	3.46685909	3.6111497	3.79779982	3.69111733	3.88934021	3.97137697	4.14265933	4.02227908	3.99639025
NONHSAG011744	3.73235133	3.60950025	3.7554224	3.72195073	3.77993122	3.49533007	3.8427187	3.73407647	3.92651477
NONHSAG011747	6.98418841	7.00188686	6.5372269	6.88435144	7.16185401	6.82805701	6.99926614	7.0021871	7.0527405
NONHSAG011762	3.7228723	3.45845007	3.5860694	3.25723845	3.37471785	3.34279215	3.63531931	3.62192809	3.50086442
NONHSAG011768	2.51810534	2.20091083	2.25738713	2.29033822	2.28290093	2.29243296	2.26657607	2.2641699	2.31561006
NONHSAG011773	2.31721241	2.28876945	2.32286707	2.28161688	2.1843102	2.37398861	2.24157743	2.34424325	2.09792751
NONHSAG011781	6.45010741	6.33118917	7.01964136	7.39353373	6.8070546	7.1595588	6.73401624	7.24685066	7.29519916
NONHSAG011800	2.61267256	2.64416306	2.3952468	2.53911141	2.51744127	2.1244005	2.77997772	2.25924531	2.41003179
NONHSAG011802	9.20827627	9.21527555	9.86573567	10.11982435	9.88278209	9.85683646	9.81304777	10.12065732	10.08241256
NONHSAG011805	3.17427618	2.94313763	3.05262562	3.17510145	3.20919963	3.18530715	3.23971459	3.26130409	3.18540684
NONHSAG011821	2.58270021	2.62491932	2.62722838	2.46817204	2.58863005	2.62580728	2.50353022	2.74391793	2.7482288
NONHSAG011830	2.33654089	2.35774927	2.36098941	2.2840574	2.28348509	2.14864591	2.50353022	2.28303244	2.11667299
NONHSAG011840	2.33672875	2.37889473	2.31603733	2.39139162	2.44863712	2.19074045	2.51524016	2.39827016	2.28355335
NONHSAG011877	3.92357868	3.91462193	5.25454813	5.22206539	4.90593792	5.32933202	4.56628408	5.41218995	5.40967524
NONHSAG011915	5.75222753	5.64164539	5.64723281	6.13543406	5.74924904	6.18060834	5.81927623	6.1069035	5.83400282
NONHSAG011924	3.13205423	3.22825075	3.2192659	3.25112323	3.46093153	3.34804974	2.93112552	3.83579076	2.99809177
NONHSAG011933	5.51866955	5.38647331	5.68651599	5.65727517	5.68831745	5.60153618	5.51241646	5.72041687	5.61640956
NONHSAG011964	5.80992499	5.76895349	6.05046509	6.4427013	6.16331983	6.06221736	6.43357076	6.18400089	6.17774777
NONHSAG011968	4.11236612	4.39479848	4.34387744	4.15854908	4.66078931	4.38232245	4.49204925	4.27868598	4.49375632
NONHSAG011977	2.90596398	2.84377606	2.70576425	3.04635622	2.84429222	2.84192779	2.70488519	2.81683794	2.74232354
NONHSAG011978	4.79780919	4.89453763	5.02080408	5.05424941	5.07811585	4.99863388	4.61600568	4.90560542	4.86555852
NONHSAG012011	2.95437426	2.65019827	2.63970243	2.59054407	2.55072637	2.45510915	2.70419934	2.93509238	2.67590107
RMST	8.436442	8.19900366	5.89725296	6.44709696	5.70107957	5.99614214	5.73694999	6.48647347	6.32176791
NONHSAG012031	4.40223467	4.23540546	4.29380984	4.31044939	4.18693717	4.14069081	4.56594728	4.24466498	4.05341258
NONHSAG012043	2.23075249	2.36119136	2.40755413	2.22805304	2.20020778	2.24637919	2.2260516	2.12287445	2.26899558
NONHSAG012048	4.96874595	5.14894495	4.54282133	4.97457635	5.03653278	4.82799386	4.89274945	5.17208463	5.18708637
NONHSAG012050	6.70620903	6.63826465	6.72646023	7.13358418	6.75312579	6.99453315	6.64959032	6.71816169	6.70231909
NONHSAG012055	6.81492017	6.55523991	7.13927163	7.12815098	7.34911817	7.06118556	7.10429335	7.24609623	7.22957585
NONHSAG012099	2.51615911	2.35111747	2.5081571	2.59859589	2.37735334	2.44093531	2.71777934	2.51389191	2.47780636
NONHSAG012152	2.46092626	2.47575117	2.32411112	2.17075533	2.39739424	2.34230038	2.24155684	2.39257752	2.46195394
NONHSAG012158	4.75058857	4.24947615	4.34596882	4.23616186	4.39584427	4.00947684	4.44958064	4.56274063	4.46905415
NONHSAG012172	3.80627288	3.76845593	3.78452942	4.10121417	3.76387347	3.73438277	3.87132867	3.73239544	3.87617436
NONHSAG012182	4.00343716	3.93595207	4.30108315	4.19364963	4.07097016	4.24798672	4.13789397	4.24565122	4.34294034
NONHSAG012199	4.19655253	4.18525871	4.54413583	4.22380104	4.35376947	4.14677965	4.22205237	4.44847482	4.29734531
NONHSAG012226	4.16893479	4.09124445	3.86871366	3.92873927	4.11522416	4.20123439	4.18645213	3.98184467	4.15918076
NONHSAG012236	2.86404345	2.45770793	2.62429398	2.48320054	2.59799354	2.54576789	2.80547076	2.58701701	2.68386926
NONHSAG012367	3.29213812	3.20480874	2.95805596	3.14579991	2.99765354	3.06520647	3.00451003	3.10738759	3.20183452
NONHSAG012392	2.24847025	2.40106443	2.3626936	2.7604957	2.38934064	2.78525848	2.31326223	2.42856381	2.81927794
NONHSAG012393	4.54654568	4.6660355	4.8898732	5.01504216	4.42958041	4.43256319	4.8733025	4.42316487	4.5908859
NONHSAG012430	2.92658279	2.96382777	2.91462832	2.66248591	3.12107402	2.72193231	3.00777759	2.84151458	2.97730991
NONHSAG012433	5.00173771	4.93797207	5.15907897	4.71798595	4.91317938	4.75279281	4.84592658	4.96901131	5.07144282
NONHSAG012556	6.28559191	6.51078903	6.39211911	6.36002748	6.55275231	6.03460709	6.46561307	6.45027645	6.40664825
NONHSAG012565	4.30984286	4.32331344	4.57248047	4.27008697	4.29721094	4.19281696	4.58688821	4.15896992	4.40319933
NONHSAG012566	3.9499851	4.07170697	4.10000236	4.41669902	4.3110709	4.00162549	4.21134174	4.17130007	4.30742783
NONHSAG012599	6.61858527	6.60377522	5.5713364	6.1962363	5.4965963	6.20870447	5.54245874	5.72975878	5.84783693
NONHSAG012608	4.14548993	4.10123202	4.71229814	4.19027511	4.49445499	4.39821531	4.05512082	4.45735761	4.21565736
NONHSAG012629	2.38554485	2.46584758	2.38428901	2.56073937	2.30535548	2.27569114	2.38905767	2.55301071	2.40167605
NONHSAG012630	5.61766878	5.47659929	5.878261	5.66675418	5.97748171	5.57461433	5.74934369	5.82732059	5.8827066
NONHSAG012635	3.42496856	3.3487167	3.4233282	3.2769704	3.54602572	3.01465587	3.65973855	3.17607284	3.43759341
NONHSAG012657	8.79054816	8.91074907	7.41128483	7.88375259	7.68241145	7.86465487	7.49669491	7.58112856	7.37109086
NONHSAG012658	10.58120396	10.60196398	9.28451241	9.48855324	9.51753918	9.69352774	9.37670535	9.48139109	9.27475346
NONHSAG012682	3.10744196	3.34943728	3.38865321	3.00405032	3.36302662	2.95432815	3.65828283	3.12698195	3.226506
NONHSAG012683	3.35194164	3.5893015	3.51391754	3.16436509	3.14731953	3.22125434	3.52562108	3.22547548	3.57561841
NONHSAG012684	2.19074045	2.3514359	2.52950473	2.51013327	2.34422827	2.51741992	2.37518669	2.7264095	2.4802418

NONHSAG012695	4.39324239	4.44913405	4.57504345	4.29109536	4.59875565	4.27347937	4.67410906	4.44953084	4.47224639
NONHSAG012727	5.31743126	5.16719352	5.42993588	5.35761876	5.53154665	5.26705768	5.49401665	5.33627845	5.5320165
NONHSAG012732	3.72211837	3.59020038	3.63027978	3.48578059	3.1613276	3.26231791	3.36307477	3.04718466	3.22678443
NONHSAG012739	5.66809369	5.49251406	5.83242938	5.33970482	5.74949446	5.03784471	5.77599501	5.38211464	5.5912636
NONHSAG012744	3.19615593	2.87266922	2.97659922	2.96431054	2.99620069	2.9230376	3.20468243	2.94280988	2.81698902
NONHSAG012745	4.22686007	3.28265254	3.52143302	3.75901827	3.92377552	3.88152907	4.05037701	3.60487733	3.3885366
NONHSAG012747	4.84550857	4.95151579	5.58725705	4.89461048	5.07530387	4.74756025	5.58898388	4.81195113	5.24901733
NONHSAG012753	5.75029988	5.91738721	5.974422	5.77050213	5.92405411	5.82048439	5.94127862	5.84488063	6.03343437
NONHSAG012762	4.75421729	4.4363896	4.53512044	4.27059494	4.80046402	4.25364917	4.55726985	4.55676122	4.76612999
NONHSAG012764	4.78918826	4.64391194	4.81936443	4.60342221	4.72040177	4.45914609	4.69894409	4.96244814	4.55763687
NONHSAG012780	4.2598943	4.43873022	4.45464925	4.07552225	4.46561029	4.25853486	4.37933274	4.03705783	4.27302274
NONHSAG012793	6.49254525	6.46385887	6.3513263	6.33114717	6.33423177	6.27805699	6.04086866	6.21850937	6.28895122
NONHSAG012799	5.03862386	5.22520267	5.26974923	5.58048938	5.56342396	5.61804265	5.31889461	5.39718402	5.42374905
NONHSAG012823	4.09116781	4.1445785	4.15619878	3.98023909	4.08730417	3.92643329	4.50137101	3.93977716	4.21870059
NONHSAG012881	2.89404174	2.9780436	2.92647307	2.75163837	2.94571604	3.21466605	2.84056493	2.87147329	2.74377582
NONHSAG012884	2.78452264	2.86373111	2.58962567	2.88108684	2.69253499	2.81952375	2.69560558	2.66692126	2.57011996
NONHSAG012909	5.96099355	6.01337366	6.2071436	5.9914933	6.26928051	6.00588399	6.36306625	6.01366228	5.95183979
NONHSAG012910	6.38546699	6.19115168	6.36649108	6.21143852	6.65712896	6.11190343	6.46093167	6.84011951	6.60681046
NONHSAG012920	2.80452679	2.91683621	2.77075416	2.69440881	2.84378333	2.81964839	2.74134938	2.93671864	2.74232354
NONHSAG012971	2.40623459	2.37246834	2.43044687	2.65778988	2.63583142	2.2588141	2.40232266	2.22419006	2.55748296
NONHSAG012980	2.59127278	2.50569904	2.70776078	2.55015536	2.73024876	2.71509866	2.9083305	2.4316711	2.72489373
NONHSAG012986	8.05141938	8.23386039	8.04868831	8.0144958	7.91115594	8.13033004	7.88245843	8.24629225	8.21361772
NONHSAG013018	3.09612743	2.93367323	3.12445674	3.01696632	2.92222458	2.8189744	3.10352293	3.04985586	3.15153554
NONHSAG013032	2.68897202	2.74807345	2.68032913	2.66688213	2.62648625	2.736988	2.99944565	2.83447712	2.78319941
NONHSAG013037	4.12732162	4.15291224	3.82611671	4.00530928	4.12669491	4.16677624	3.75680339	4.22535057	3.84767801
NONHSAG013039	4.29593578	4.30697176	4.01113012	4.26243553	4.30529368	3.93031394	4.03796388	4.21838069	4.06980344
NONHSAG013044	4.70239538	4.25334344	4.2651254	4.16771114	4.16771114	4.11773614	4.33268264	4.18832424	4.04608422
NONHSAG013052	4.94844155	4.89421321	4.73907021	4.45055735	4.69635252	4.34756863	5.03865368	4.63734545	4.87915696
NONHSAG013055	2.00648197	2.17971025	2.17776576	2.0554279	2.08377285	2.33783757	2.29610365	2.04681389	2.15481549
NONHSAG013061	2.74869551	2.75453833	2.88072797	2.81596774	2.93282648	2.71717439	2.81894197	2.78492855	2.95723659
NONHSAG013087	4.55108249	4.15673122	4.74711483	4.72173731	4.85514792	4.5210784	4.22891033	4.98703274	4.73410789
NONHSAG013088	4.87518309	4.89983115	5.07757362	4.76957835	5.01444396	4.86407369	5.26317696	4.92336908	4.76689279
NONHSAG013092	3.1187277	3.18400152	3.06919203	3.12208479	3.30738216	3.18433499	3.13271864	2.8040853	2.97722514
NONHSAG013093	3.84933109	4.22882601	3.95213017	3.55805917	3.93769047	4.07612	4.02160133	3.67601152	3.87536903
NONHSAG013140	4.64852334	4.99342923	4.7209162	4.53206372	4.77806206	4.4634491	5.12733783	4.53012266	4.54212177
NONHSAG013147	2.83617532	2.84338591	3.16580745	2.73636444	2.8053216	2.56124841	3.06641865	2.48126182	2.80222652
NONHSAG013148	3.8166011	3.60716663	3.82392112	3.65478857	3.78452104	3.68625655	3.98246908	3.76530679	3.83623113
NONHSAG013176	3.24580098	3.33655972	3.49386577	3.26280206	3.32892546	3.43120449	3.45322138	3.353396	3.00984384
NONHSAG013184	4.65124962	4.55678964	5.29428336	5.53294198	5.58331774	5.4632096	4.98053114	6.35572849	6.10676633
NONHSAG013195	5.38296947	5.61096936	6.01372927	5.58532005	5.7899022	5.45449782	6.24220593	5.80798028	5.87595902
NONHSAG013196	3.85546657	3.87164105	3.81395498	3.88204749	4.01323198	3.68768002	4.30865688	3.89223353	4.07641964
NONHSAG013213	2.54602336	2.57290917	2.77800471	2.66095601	2.75156416	2.76460633	2.61242829	2.715369	2.72203367
NONHSAG013219	3.63171479	3.54767993	3.48666268	3.39301706	3.5443702	3.49630015	3.62765758	3.47520477	3.26517257
NONHSAG013224	3.10450076	3.348247	3.21129006	3.18887355	3.34468642	3.31791125	3.11880212	3.04483614	3.065341
NONHSAG013270	2.30618175	2.59540702	2.36717981	2.3361254	2.16102946	2.30182085	2.34983525	2.37999479	2.37228535
NONHSAG013289	3.51635967	3.47898853	3.51822217	3.77669242	3.84875272	3.4594805	3.60661122	3.54304352	3.74933233
NONHSAG013312	4.31261213	4.27981642	4.23838565	4.66430746	4.48206215	4.67066687	4.03716983	4.64290334	4.19178822
NONHSAG013343	8.507438	8.35271061	7.92303337	8.37577528	8.05475304	8.21172505	7.69538868	8.18048149	8.21091733
NONHSAG013371	4.41282673	4.69084413	4.30646229	3.92217826	4.05630335	4.40600325	3.94590297	4.02359821	3.97552292
NONHSAG013387	4.19301478	4.11680421	4.1697669	3.84331058	4.19007482	3.93158868	4.35553859	3.81576059	4.02662229
NONHSAG013400	6.86355705	6.76812674	6.89256205	7.2149952	6.77469504	6.99100132	6.76877669	7.12607647	6.99097087
NONHSAG013413	2.74465636	2.78798777	3.15961331	3.14150263	3.28604795	2.80163066	3.39852305	3.24868888	3.43985566
NONHSAG013415	4.38288513	4.1279801	4.86704748	4.7576208	4.59406096	4.71134713	4.54259985	4.49590604	4.59684624
NONHSAG013417	2.27983359	2.32878535	2.27983359	2.51335102	2.17498998	2.37444372	2.21302154	2.28303244	2.11780455
NONHSAG013418	4.46397534	4.61801208	4.72844063	4.4530999	4.59746184	4.42288677	4.64296174	4.58040936	4.58021199
NONHSAG013429	8.6877054	8.74379361	8.80225311	9.06084251	8.89181686	8.88277356	8.86344821	8.85694581	8.66555129
NONHSAG013433	2.41552824	2.67479731	2.62348669	2.53791927	2.41790122	2.71915949	2.56988736	2.78706648	2.4912052
NONHSAG013449	2.60246683	2.62222761	2.83496673	2.70142543	2.67019156	2.35528549	2.82676547	2.65093479	2.60973504
NONHSAG013472	3.4729173	3.16166812	3.32517006	3.11645711	3.32330719	2.97160707	3.62918394	3.37469652	3.11283775
NONHSAG013488	3.2973612	3.29081005	3.18262994	3.20800907	3.20816939	3.24254111	3.19702277	3.09848333	3.32654261
NONHSAG013496	3.16269314	2.6252309	2.76451633	2.55246413	2.94950668	2.48579003	2.65493774	2.86046423	2.7717572
NONHSAG013505	5.72259392	5.59972505	5.85061728	5.75528226	5.84207565	5.46431578	5.8733627	5.9332626	5.68129419
NONHSAG013509	4.93636705	4.91938466	4.6966923	5.37873813	4.95594224	4.54340667	4.33236535	5.5424846	5.19182459
NONHSAG013521	4.10350048	3.63351629	3.43643655	3.40587112	3.38291048	3.47950837	2.89756375	4.11650134	3.55939261
NONHSAG013525	4.24699736	4.03189415	4.3145646	4.19038776	4.17856248	4.29889766	4.32144887	4.32219615	4.22637684
NONHSAG013533	3.3114869	3.15283533	3.71883018	3.45583859	3.53948652	3.0560019	3.30705707	3.70819946	3.76175812
NONHSAG013546	3.34316035	3.25451134	3.24673711	3.49621366	3.21733935	3.1122582	3.54220796	3.34080132	3.12832508
NONHSAG013584	5.21177729	5.04655406	5.06595796	5.72962253	5.05503983	5.25039897	5.02104555	5.23317511	5.22481855
NONHSAG013596	3.69544686	3.69061758	3.9792445	3.79768261	3.76078123	4.08945981	3.9714983	4.07880298	3.75953778
NONHSAG013598	2.35906619	2.66494101	2.5670322	2.44111915	2.4950359	2.56803573	2.86134658	2.66089721	2.56117392
NONHSAG013622	2.49382808	2.35034626	2.61365026	2.55487738	2.34422827	2.26424768	2.44024499	2.35130601	2.58473074

NONHSAG013653	2.28442758	2.60204672	2.43427429	2.38306218	2.48253293	2.15637716	2.56172364	2.43094592	2.29916015
NONHSAG013655	2.96701356	3.05265576	3.05636901	2.96033955	3.04360108	3.07582715	3.08226538	3.04426874	3.02086336
NONHSAG013658	2.566412	2.61964628	2.88564942	2.42834759	2.67873275	2.528779	2.51929156	2.53263016	2.60760683
NONHSAG013688	3.05887922	3.47147124	3.36330709	2.98908604	3.44890196	3.18382026	2.83560368	3.16995968	3.0887301
NONHSAG013701	2.33777488	2.46173106	2.39585661	2.36892301	2.28514881	2.21769747	2.43571575	2.1907337	2.44834334
NONHSAG013735	4.09712176	3.99075262	3.90052614	3.95926196	4.04124989	4.03897747	3.93359045	4.1513639	4.04209817
NONHSAG013737	2.22638698	2.34026677	2.32233166	2.44147027	2.25412814	2.2855258	2.21181466	2.6777553	2.59595877
NONHSAG013751	2.3347327	2.51207706	2.4834988	2.54031193	2.49789611	2.52480609	2.60138971	2.49274266	2.67548842
NONHSAG013766	2.30616549	2.16486508	2.16303002	2.19550815	2.28748451	2.26666176	2.41631015	2.11996633	2.13677815
NONHSAG013770	2.16483555	2.17842571	2.2259012	2.31215285	2.17421271	2.11540994	2.45364086	2.16608005	2.136207
NONHSAG013789	4.72428245	4.63769836	4.9488238	4.76577414	4.92828598	4.57385205	4.88576949	4.74485927	4.7368604
NONHSAG013796	2.83456973	3.18260251	2.91689704	3.03860724	2.88846461	3.1356647	3.33061421	2.99255716	2.98107832
NONHSAG013836	3.55302682	4.08793325	3.59183404	3.86262213	3.59183404	3.68432348	3.33980049	3.63200118	3.84889576
NONHSAG013843	6.29527404	6.44213465	6.44697913	6.8087409	6.43282488	6.41268467	6.21968603	6.68371369	6.43644648
NONHSAG013856	3.54215626	3.29876379	3.43565152	3.33309043	3.34184855	3.64066246	3.75487658	3.40814799	3.63535824
NONHSAG013867	2.22118712	2.18035191	2.44068753	2.27074492	2.41574437	2.21320891	2.28501328	2.38974594	2.21019828
NONHSAG013871	2.8655005	2.83576946	2.85151924	2.76306663	3.00728763	2.934719	3.14555937	2.82165677	2.8840006
NONHSAG013889	2.35412254	2.23865806	2.05793599	2.23702743	2.14638779	2.09322677	2.03698795	2.23455089	1.96568774
NONHSAG013908	2.42097663	2.67174738	2.5928576	2.36892301	2.23567239	2.2763371	2.39612702	2.26578785	2.45754547
NONHSAG013910	2.10065406	2.13973563	2.5098427	2.15396807	2.11188934	2.21541597	2.21338698	2.051659	2.22805304
NONHSAG013921	3.42447495	3.30087605	3.56216168	3.21626065	3.67987533	3.40665719	3.53331437	3.26928617	3.30364203
NONHSAG013938	4.88527138	4.869103	5.00785756	4.58630464	4.62984189	4.66299296	5.01314727	4.87836252	4.58286064
NONHSAG013940	4.54695389	4.50890671	4.54545376	4.47012273	4.56415093	4.19653417	4.77861159	4.61633311	4.73282234
NONHSAG013950	2.56108797	2.80355656	2.76594106	2.64574272	2.64841808	2.58331131	2.97152176	2.63737959	2.70397844
NONHSAG013963	4.94786933	5.11591375	5.21743268	4.98479613	5.29413939	4.93539116	5.10377882	5.05450551	4.78602264
NONHSAG013971	3.90307217	3.75588346	4.12733023	3.82012403	4.05840505	4.07971855	3.70963533	4.21781801	3.78162207
NONHSAG013976	3.94119907	4.20133878	3.72016786	3.72195073	4.12317299	3.72315225	4.18506029	3.86643605	3.89535372
NONHSAG013982	2.91250646	2.99952817	2.91165579	2.86912925	2.75980799	2.7578879	3.10695852	2.92266587	3.11669217
NONHSAG013992	4.24618538	4.0065698	4.25888064	4.16751424	4.24685646	4.24950859	4.14557089	4.12121589	4.10552266
NONHSAG014007	6.04847104	6.12340325	6.12062061	5.77804384	6.11468881	5.6516628	6.0800565	5.75451871	6.05796687
NONHSAG014012	4.24182052	4.4978975	4.3945189	4.40736356	4.46523618	4.27135135	4.49585447	4.29165413	4.31372706
NONHSAG014023	2.76912646	2.87178221	2.84375578	2.87999139	2.70669736	2.70268716	2.89004684	2.6594603	2.84960943
NONHSAG014030	5.59615997	5.47843042	5.41196464	5.69172098	5.31945828	5.51433053	5.43107207	5.37393947	5.50066769
NONHSAG014035	5.96352434	6.03597201	5.86818144	5.89765844	6.13326452	5.95133171	5.87968479	5.89178307	6.30801354
NONHSAG014073	6.16915791	6.41559423	6.343598	6.435503	6.65191175	6.68796167	6.29425868	6.56723249	6.2685788
NONHSAG014079	3.83964415	3.69609209	3.91126464	3.80805396	3.93769047	3.80912786	4.15922047	3.77839533	3.76324523
NONHSAG014090	3.85240072	3.84131425	4.07479654	4.01827579	4.12204992	3.93273351	3.97255422	3.68509184	3.9926265
NONHSAG014099	3.78378936	3.77165309	3.83615005	3.51225945	3.99706735	3.52321714	3.68609851	3.49086756	3.89155332
NONHSAG014114	2.87863403	2.76453128	2.83667739	2.70578054	2.80836979	2.65022384	2.86214332	2.68056651	2.87257346
NONHSAG014130	3.57596568	3.52574713	3.67293947	3.12698292	3.35316676	3.34534189	3.37809711	3.28029979	3.33105887
NONHSAG014136	5.30815707	5.26697329	5.45164767	5.10191526	5.40604034	5.16744862	5.70873062	5.23229626	5.52500606
NONHSAG014165	2.8759418	2.83857533	2.83160251	2.45560175	3.001937	2.78309082	2.84762689	3.07580847	2.75944087
NONHSAG014169	2.95904074	2.97750824	3.03823193	2.7907185	2.96210067	3.00618067	2.80241842	2.97882252	2.90747431
NONHSAG014185	3.04349515	3.06345813	3.11783475	3.18310366	2.88945345	3.15603923	3.14278179	3.10771429	2.93356718
NONHSAG014197	2.66024532	2.64103863	2.75113033	2.34634613	2.49271969	2.38206557	2.87069273	2.69749034	2.47570817
NONHSAG014198	4.25629923	4.35802654	4.53522997	3.87587637	4.11298779	3.99326312	4.27392436	4.22366561	4.34104933
NONHSAG014202	4.07095811	4.04817308	4.05366426	3.97259371	3.9285419	4.11810077	4.07848914	4.18794753	4.05341258
NONHSAG014208	3.85410113	4.05020607	3.75335811	3.51512467	4.08488938	3.63322407	3.95990673	3.62450769	3.61952575
NONHSAG014212	3.06023727	3.14217155	3.28672451	3.0329256	3.06883952	3.09118469	3.37439945	3.27071245	3.153313
NONHSAG014215	2.26184319	2.21520459	2.22490964	2.18164307	2.1044775	2.21173513	2.27127818	2.20740412	2.1915493
NONHSAG014232	3.931293	4.04688675	4.44610295	4.41991093	4.13329879	3.98923221	4.48950829	4.05928533	4.29729355
NONHSAG014249	4.21155628	4.37841743	4.50631248	4.37104696	4.39627403	4.38740365	4.71840857	4.36225359	4.40104829
NONHSAG014269	4.72226529	4.86212289	4.58271371	4.68545156	4.83922748	4.57229125	5.12470789	4.85722716	4.81047952
NONHSAG014294	4.49732783	4.6990665	4.88737189	4.2902121	4.54752016	4.54125763	4.62727477	4.65058708	4.64888399
NONHSAG014358	2.99124892	3.04965408	3.61979577	4.29937148	3.55972426	3.62362349	3.56067491	3.82811691	4.07000052
NONHSAG014410	2.78633325	2.93522885	2.83121904	2.80132484	2.66593171	2.81750717	2.90756368	2.7752878	2.87687455
NONHSAG014436	2.95948975	2.84736651	2.98721498	2.80329078	2.914709	2.89705399	3.10701395	2.92332297	2.95059549
NONHSAG014438	3.50520943	3.30418093	3.78452942	3.45770322	3.48594365	3.41754982	3.78823012	3.58418618	3.18991173
NONHSAG014440	2.35534803	2.70114608	2.33918486	2.54717565	2.60054044	2.49670652	2.6093324	2.55597556	2.43213479
NONHSAG014443	2.29619565	2.15190207	2.3946479	1.96172926	2.21606563	2.27037251	2.66401777	2.06849654	2.26805755
NONHSAG014445	3.44019117	3.50933207	3.6122871	3.49727521	3.60599292	3.13415068	3.53194275	3.449942	3.61561877
NONHSAG014448	4.47947272	4.55418057	4.00870001	3.89629552	4.54048969	3.80511502	4.96655976	3.83317547	4.70620852
NONHSAG014454	2.48882128	2.38240459	2.34469216	2.26939021	2.35688863	2.43286582	2.40062769	2.37810017	2.75986569
NONHSAG014569	3.19949959	3.40076006	3.35484792	3.2128374	3.3583347	3.32145382	3.21776004	3.07261095	3.13908703
NONHSAG014574	2.62722838	2.57182303	2.78376534	2.86121319	2.34387382	2.69936116	2.63239053	2.53330368	2.41130408
NONHSAG014590	3.20253649	2.97344503	2.84887035	2.93120378	2.98599764	2.99130052	3.33223174	2.77680999	3.02820312
NONHSAG014611	3.32883317	3.23109133	3.18833491	3.22010752	3.26752038	2.96678369	3.38825162	3.21187738	3.09003045
NONHSAG014661	2.72437422	2.54068733	2.86548005	2.61310388	2.62931419	2.39568731	3.02064452	2.49121297	2.89126217
NONHSAG014663	5.45499309	5.24417325	5.95708658	6.38524729	6.59246475	6.06815526	5.8562051	6.85271949	6.03053467
NONHSAG014667	4.26673073	3.83890688	4.22176406	3.98914151	3.94431909	3.48513496	3.87026786	3.9698353	3.86283624
NONHSAG014746	2.12960265	2.13918013	2.25387936	2.33212306	2.27067516	2.14798747	2.30348417	2.20604132	2.31851566

NONHSAG014747	2.34711041	2.21491417	2.38097546	2.1965182	2.10139951	2.17497424	2.21302154	2.25372914	2.22805304
NONHSAG014790	4.97036357	4.97472429	4.99208631	4.71151547	5.14870946	4.68052212	5.43053376	4.96649736	5.10157684
NONHSAG014806	4.55068978	4.23861037	4.19921619	4.49069405	4.43183749	3.97527627	4.60514951	4.3541354	4.52862225
NONHSAG014811	2.15905917	2.3077097	2.3270058	2.48966921	2.29347973	2.28995364	2.21302154	2.32675492	2.22805304
NONHSAG014813	2.81342094	2.71753512	2.75287297	2.54233461	2.71280513	2.4331085	2.85251642	2.71394872	2.69897194
NONHSAG014829	4.30590617	4.32258069	4.39770157	3.9589052	4.28931666	4.09638553	4.1650687	3.99156499	4.37423749
NONHSAG014834	4.20223575	4.04299325	4.00176247	4.18628397	4.369549	4.39780797	4.064832	4.05097937	3.91100323
NONHSAG014845	3.56611604	3.51868938	3.63462106	3.86459813	3.67197053	3.43847239	3.95082585	3.66764403	3.69770678
NONHSAG014849	3.07436314	3.03474714	3.04953367	3.02438532	3.23712989	2.82154476	2.9554847	3.14534501	2.9742502
NONHSAG014850	2.33179862	2.12088509	2.49684897	2.25699062	2.38378829	2.33911377	2.5316739	2.31118573	2.12945622
NONHSAG014898	3.42549102	3.61648571	3.84322835	3.8748711	3.83526632	3.89585414	3.52876397	3.54561558	3.73927116
NONHSAG014905	5.10341174	5.02028578	4.95252796	4.81122962	5.17519833	4.56868336	5.20409994	5.03261983	4.84723559
NONHSAG014910	6.02368555	5.96322539	6.11078879	6.46168134	5.8778424	6.41400897	6.00848391	6.30720721	6.35550636
NONHSAG014924	6.84130875	6.55393059	5.55563384	5.6809143	5.92683416	5.64747528	5.90669738	5.67988098	5.86242434
NONHSAG014926	4.68169205	4.33663507	4.75285188	5.25402945	4.10053566	5.17121849	4.69988363	4.7906744	5.01668197
NONHSAG014964	2.17550413	2.24944085	2.17939735	2.25158133	2.20584945	2.22684178	2.36434259	2.28227862	2.23469567
NONHSAG015027	7.0438335	7.23433759	6.89473495	7.45153969	7.26941248	6.94389825	6.66892981	7.19156815	7.15935371
NONHSAG015035	2.59195542	2.45756788	2.58468798	2.49055729	2.7064735	2.31525669	2.64969992	2.586829	2.61326311
NONHSAG015052	4.00264481	4.00077242	4.11065563	3.74859058	4.0111364	3.75304244	4.06551407	3.86521609	4.04807619
NONHSAG015063	2.85726463	2.97445325	3.31337296	2.90980108	3.20512678	2.79124363	3.07830057	2.89580741	2.99295343
NONHSAG015092	2.65550915	2.52274554	2.42015316	2.59595877	2.87134986	2.70471413	2.60431213	2.68921103	2.71099604
NONHSAG015143	5.44782234	5.47788454	5.43236502	5.59869839	5.58343735	5.48722969	5.48806932	6.08190886	5.65476118
NONHSAG015144	4.76366217	4.90173269	4.81336562	5.32607476	4.87844438	5.1362877	4.34178823	4.84382589	4.5833506
NONHSAG015185	2.30149798	2.22059244	2.24925065	2.28282765	2.15679384	2.39607379	2.26227426	2.17241594	2.31670873
NONHSAG015188	2.8470286	2.91560538	3.0662647	2.6786262	2.91088367	2.61841252	2.48020244	2.90610511	2.63657818
NONHSAG015211	3.13260169	3.08595533	3.11310501	2.93146458	3.10848605	2.82706516	2.96997822	3.02802751	3.01434016
NONHSAG015231	6.01294229	5.50356253	5.8678963	5.98755214	5.82663942	6.25520005	5.93480745	6.1100859	6.04441564
NONHSAG015256	2.99508005	2.74582758	2.9308152	3.01818621	2.9924423	2.60468005	3.04697559	2.78246741	2.71723121
NONHSAG015273	3.58766572	3.50172609	3.69096902	3.23627126	3.53692724	3.26328595	3.86869618	3.50222448	3.53530091
NONHSAG015286	5.21083543	5.13292058	5.40569834	5.10231109	5.16663034	5.1166123	5.34777903	5.17102881	5.46859124
NONHSAG015320	2.69962937	2.46948889	2.42663491	2.45397529	2.28732486	2.44784535	2.71038661	2.55604873	2.45705496
NONHSAG015338	2.6455646	2.99804929	3.07142256	2.87288708	2.9233906	2.66180572	2.89458201	2.86952453	2.77575537
NONHSAG015357	3.89450648	3.76442592	3.98286443	3.64221278	3.86490372	3.72694064	3.87220367	3.72356293	3.92474393
NONHSAG015371	4.43934746	4.51817417	4.61234799	4.12546154	4.59089503	4.0860543	4.69133028	4.38897333	4.37902804
NONHSAG015373	2.91268926	2.75453833	2.8977894	2.85037795	3.07701686	2.86368131	3.08200614	2.90024262	3.03983771
NONHSAG015387	5.91440152	6.12585459	6.13680103	5.84569875	6.08532613	5.67343278	6.31683598	5.82406046	5.97184313
NONHSAG015435	4.10043853	4.08765279	4.14577747	4.07098994	4.27994147	4.07650344	4.27720618	4.12714904	4.3551515
NONHSAG015438	2.5063625	2.623583	2.88765782	2.70907837	2.82241808	2.54520915	2.46091303	2.79903834	2.71966068
NONHSAG015450	2.82836679	2.82326698	2.39550887	2.6805319	2.7249921	2.59741447	2.36680757	2.5099927	2.34769556
NONHSAG015466	4.91294089	4.87690528	4.6396995	4.62179462	4.95778995	4.61402555	4.95722286	4.72562063	4.52752592
NONHSAG015491	3.5364143	3.61252839	3.51581552	3.37311564	3.48924859	3.64675076	3.60018365	3.65973855	3.1834391
NONHSAG015511	7.58592541	7.67405796	7.6463408	7.90903331	7.45806902	7.7928651	7.26832824	7.71486092	7.72996407
NONHSAG015535	2.37247472	2.32283178	2.38611519	2.24448726	2.370918	2.32914503	2.41845258	2.36946499	2.33145314
NONHSAG015539	3.47819154	3.45039312	3.56863236	3.95053189	3.90195018	4.50521744	3.72525576	3.9388201	3.39544608
NONHSAG015555	6.22843746	5.8769389	5.72868911	5.58272854	5.8675281	5.77831331	5.73924854	5.58368523	5.65536825
NONHSAG015654	2.96667818	2.91660479	3.19557916	3.03789486	3.12065808	2.86795861	2.94293921	3.00269936	3.06219897
NONHSAG015660	3.55920651	3.90400451	3.73892671	3.54687535	3.58276893	3.30148417	3.47234327	3.66653181	3.63129991
NONHSAG015664	3.26394036	3.01458273	3.28526874	3.22264369	3.60767668	3.23094869	3.27818451	3.13749136	3.30327748
NONHSAG015694	6.52085378	6.70804872	6.54691904	6.18392696	6.55445905	6.48489832	6.62401093	6.5738902	6.45880189
NONHSAG015695	4.78201519	4.63273483	4.55534788	4.41361737	4.40671342	4.48413771	4.84179648	4.18023494	4.90973067
NONHSAG015708	3.59173645	3.52856519	3.58493816	3.47066447	3.84313145	3.59407118	3.64077447	3.7102227	3.5838519
NONHSAG015712	4.62851923	4.58700963	5.30081771	6.37411746	5.55511757	5.39269994	5.30316316	6.03933695	5.28298007
NONHSAG015718	3.35990524	3.58220641	3.24429659	3.29507277	3.42812972	3.27034647	3.2837444	3.3467499	3.25267162
NONHSAG015732	5.13983478	4.98720514	6.08459492	6.32500157	6.2619543	6.28334573	6.06366643	6.38141405	6.30404904
NONHSAG015756	3.00223247	3.1658467	2.98931543	2.9354164	3.13190789	2.92731606	3.39638011	3.04237865	3.13487312
NONHSAG015757	4.97327057	4.59332839	5.28913889	4.7512436	5.47307091	4.79873835	5.13825531	5.23625987	5.03525674
NONHSAG015797	3.77315453	3.77640304	3.94601275	3.33611594	3.88536597	3.75606317	3.77624601	3.69626871	3.75476172
NONHSAG015798	4.24072366	4.12924781	3.77377216	3.95017575	3.89325508	4.01828758	3.96564687	4.06881802	3.97309196
NONHSAG015800	2.30608409	2.37111077	2.31687607	2.22805304	2.31687607	2.28494456	2.41684074	2.35130601	2.25866297
NONHSAG015828	5.59952066	5.33822124	5.18540707	5.30312864	5.40938553	5.51344017	5.44878621	5.18008955	5.40875409
NONHSAG015833	2.9313281	3.09849257	3.16563261	2.86464017	2.95493797	2.83963639	3.08038737	2.90141557	3.17461342
NONHSAG015852	2.76585384	2.81080826	2.60940583	3.02741513	2.91675573	2.54943879	3.00506808	2.93407704	2.72993174
NONHSAG015872	5.24870876	5.21377446	5.28301415	5.40015953	5.52139518	5.21743268	5.50780418	5.44615499	5.37725925
NONHSAG015874	3.21712666	3.2839156	2.94805591	3.35791136	3.06972898	3.27473692	3.6164009	3.00202491	3.00365858
NONHSAG015923	4.04482083	3.99810651	4.07454408	4.01800507	4.21999872	4.08275907	4.14400417	3.92921165	4.14120326
NONHSAG015927	2.70255166	2.82112608	2.76959048	2.66139038	2.79365558	2.71447664	2.74754142	2.65464105	2.70673253
NONHSAG015948	3.71114757	3.82274642	4.31962064	3.82539306	3.80809544	3.8700594	4.24990228	3.73301521	3.97824558
NONHSAG015952	4.18952914	4.21094777	4.44224509	4.27365964	4.23745594	3.94373901	4.17500394	4.36752312	4.27826378
NONHSAG015966	5.23496749	5.37955527	5.29633366	5.6281912	5.45404762	5.34041478	5.25867872	5.55985865	5.37904315
NONHSAG015980	6.47853452	6.59133002	6.66473496	6.5203111	6.52988125	6.42347479	6.78364468	6.40770142	6.56597358
NONHSAG015981	5.96886272	5.68300646	5.91811332	6.13208877	5.68080202	6.33813078	5.98777898	6.00727006	5.82348689

NONHSAG016009	2.83723493	3.29988834	3.12752789	3.29093236	3.02993116	3.28262559	2.86810277	3.10060453	3.08116296
NONHSAG016021	5.22775692	5.3622754	5.29731098	5.09756379	5.28630858	4.83677535	5.19693573	5.35479605	5.26481676
NONHSAG016024	3.86370834	3.60378208	3.69041383	3.36507429	3.61238691	3.63265494	3.5406404	3.7695918	3.77393987
NONHSAG016025	4.29489113	3.97682095	3.70525192	3.98959282	4.09779848	3.83219827	4.23095263	4.10377606	3.87179056
NONHSAG016027	7.59718736	7.53742697	6.96146825	7.07735508	7.04504455	7.13316113	7.17930736	6.80294994	6.81430906
NONHSAG016062	3.3998994	3.37999635	3.20342245	3.42873152	3.39484595	3.20037389	3.48994269	3.65096482	3.3919006
NONHSAG016074	9.3734285	9.27314622	9.18221925	9.391615	9.63958026	9.08928437	9.3825019	9.76807147	9.55327533
NONHSAG016107	2.68243603	2.64918818	2.60679644	2.83928547	2.88812264	3.03364373	2.93858958	2.44770622	2.57227276
NONHSAG016109	4.41369478	4.65057838	4.71857333	4.58107072	4.74610648	4.4827498	4.46514502	4.49082539	4.52236162
NONHSAG016111	5.6118263	5.4576132	5.72412207	5.28182061	5.84506835	5.08615577	5.54618569	5.37393947	5.56217774
NONHSAG016116	2.70030623	2.7083089	2.85084419	2.6760488	2.75084631	2.6576992	3.38910732	2.74967798	2.6760488
NONHSAG016136	3.14038524	2.62453601	3.12513806	2.72349463	2.83647436	2.75998259	3.10508891	3.13854572	2.84839834
NONHSAG016155	3.05887922	3.42186006	3.28452414	2.99981025	3.35370888	3.03132858	3.24508089	3.50684827	3.43235228
NONHSAG016158	3.70541465	3.70317393	3.00974462	3.66926507	3.56692205	3.86628245	3.50686219	3.41799771	3.61754206
NONHSAG016166	2.66055192	2.64973531	2.66966833	2.56908083	2.55713764	2.59045444	2.83114398	2.4207741	2.30480561
NONHSAG016202	3.30337914	3.27412185	3.65464296	3.26434438	3.30411749	3.20328796	3.37000499	3.15967274	3.36183904
NONHSAG016213	2.22477196	2.10840751	2.10519687	2.20116771	2.13612227	2.09237635	2.22806728	2.30097452	2.13888137
NONHSAG016262	3.52802961	3.69104198	3.71398653	3.43204269	3.54884842	3.26173748	3.55230026	3.65635524	3.61528634
NONHSAG016297	2.43519895	2.49217063	2.44937376	2.44434674	2.63289411	2.53724623	2.38576758	2.42391397	2.24655383
NONHSAG016313	2.98878858	3.12004057	3.10190383	3.04764715	3.24355017	2.97054102	3.18701601	2.99650631	3.01652536
NONHSAG016316	2.10501722	2.33069096	2.09434431	2.10371844	2.3353586	2.15484687	2.17425433	2.02845001	2.02324076
NONHSAG016330	2.86649845	2.66464361	2.79820451	2.58706332	2.6606123	2.77201223	2.91460229	2.76328649	2.76768827
NONHSAG016336	5.09888312	5.08362484	5.05981956	4.71033013	5.06595473	4.94280622	5.11200727	4.99804396	5.07034388
NONHSAG016353	2.37379374	2.50275132	2.11774935	2.57249411	2.40404895	2.17142234	2.55991203	2.39105357	2.49445137
NONHSAG016383	4.30236715	4.23253339	4.17575765	4.21219469	4.26973677	3.81062596	4.35182111	3.99962265	3.97642933
NONHSAG016385	3.22753348	3.20751324	3.48720112	2.92799725	3.21628684	3.06681102	3.51679387	3.17648988	3.29629324
NONHSAG016388	5.24379569	4.71674217	4.93849273	4.86018894	4.95689764	4.55445647	4.90771477	5.41688438	5.08618642
NONHSAG016419	6.35039189	6.33454034	4.83804762	5.12146562	5.08095953	4.74564061	5.02751822	5.06109973	4.64367412
NONHSAG016427	5.74082942	5.39548628	5.61192882	5.36619828	5.58780823	5.30971301	5.41535383	5.33648181	5.59324814
NONHSAG016434	2.93747047	2.83576946	3.00513356	3.34258968	2.87645312	2.99458598	3.42435663	2.88861199	3.18949079
NONHSAG016460	3.65096169	3.50660944	3.43335603	3.32715333	3.53786691	3.51581552	3.38160513	3.36930727	3.62265079
NONHSAG016472	5.81270179	5.85717388	5.86108995	5.70356898	5.84884459	5.7365395	6.14186418	5.87209635	5.80375182
NONHSAG016517	3.61741863	3.71113268	3.99610902	3.4307848	3.97973981	3.38175497	4.06658979	3.67263171	3.83414533
NONHSAG016520	2.20294665	2.20591253	2.30464079	1.98919974	2.08211768	2.1328625	2.04136277	2.22261378	2.03081674
NONHSAG016540	3.60828748	3.42186006	3.37307797	3.1398623	3.4869284	3.44285271	3.53358253	3.42813863	3.26615732
NONHSAG016542	3.31830296	3.2534526	3.19925921	3.17824556	3.22314988	3.02444606	3.42503566	3.20645629	3.21816031
NONHSAG016544	2.21525534	2.19176364	2.17939481	2.1644993	2.27333608	2.02755534	2.3330069	2.08560919	2.26058987
NONHSAG016555	3.43278022	3.42989531	3.93995111	3.65764702	3.77108817	3.549767	3.86757526	3.916015	3.96120644
NONHSAG016582	3.7981792	4.12275668	4.44108017	4.18693546	4.27227936	4.04348799	4.35286848	4.17237776	4.43951003
NONHSAG016589	6.95944442	6.98572669	6.73740362	6.64967229	6.44302443	6.77250957	6.54836266	6.63255732	6.64893811
NONHSAG016595	4.21094025	4.30012587	4.35244377	3.95410874	4.44885264	4.09078511	4.37499285	4.23470813	4.1824254
NONHSAG016623	4.44934438	4.34828667	4.31195725	4.08829106	4.23841106	3.98259871	4.39197856	4.25927307	4.39671137
NONHSAG016650	3.26916191	3.25504833	3.18664843	2.89646265	3.13693478	2.97881833	3.43080913	2.73915009	3.05740181
NONHSAG016655	3.44598877	3.41763997	3.50185502	3.393086	3.42985393	3.23932801	3.35114115	3.20413623	3.40813246
NONHSAG016687	3.89629552	3.81318179	3.94889707	3.55138807	4.03559405	3.77378836	3.92411424	3.81055708	3.54147083
NONHSAG016786	6.04102686	6.04393598	6.44793635	6.37903275	6.17260451	6.22778507	6.07097838	5.97866522	6.38872706
NONHSAG016826	2.61042439	2.6805319	2.6725226	2.7371589	2.7131193	2.54641172	2.62366538	3.10394566	2.59733905
NONHSAG016852	2.64090811	2.73663686	3.28582901	3.37496518	3.39759518	3.34141427	3.23871188	3.17934876	3.2077504
NONHSAG016854	2.4806032	2.6805319	2.68632406	2.66579273	2.68280297	2.5267096	2.74613159	2.58166745	2.67140991
NONHSAG016887	3.73182865	3.4814721	3.2864131	3.46284259	3.30657995	3.71170243	3.59134436	4.07665055	3.89211535
NONHSAG016890	2.40951285	2.79820653	2.60937461	2.39691546	2.36678446	2.55918105	2.62154151	2.48576107	2.52292195
NONHSAG016921	3.32769695	3.09198116	3.57657807	3.62425751	3.47729176	3.37867356	3.08368348	3.20467944	3.64501205
NONHSAG016956	5.65502417	5.66599887	5.83446248	5.57351731	5.76817337	5.52027762	5.99387463	5.74598519	5.74840584
NONHSAG016984	2.6945748	2.46443114	2.71875733	2.66730144	2.70511838	2.55928478	2.55422088	2.64606553	2.86015271
NONHSAG016991	5.96826513	6.01191918	5.87849534	5.77832013	5.88833782	5.76623629	5.99641531	5.58577039	5.95730754
NONHSAG017020	2.71835109	2.62247171	2.77896204	2.80180724	2.85890301	2.69189298	2.70823872	2.86392395	2.78435603
NONHSAG017021	3.93890329	3.54744346	3.76070886	3.80868796	3.7742924	3.87410499	4.17303948	3.82305229	3.62869211
NONHSAG017030	3.99561812	4.0757595	4.15100887	4.25146984	4.05404412	4.6263408	4.46925062	4.12968849	4.23296207
NONHSAG017048	2.71571254	2.72104479	2.69899127	3.0170508	2.47814387	3.03768741	2.67227901	2.55304289	2.5135443
NONHSAG017077	6.13130754	6.19837928	6.15606946	5.6037207	6.13729798	5.71784549	6.39555149	6.0021053	6.09169746
NONHSAG017083	3.59393993	3.62315169	3.49334046	3.64993511	3.70396	3.5129098	3.86547773	3.53118567	3.73501708
NONHSAG017088	2.89985394	3.00376049	2.9725643	2.88779258	3.03962502	2.78939298	2.93049058	3.10394566	3.00077009
NONHSAG017091	2.68739992	2.70148481	2.6567675	2.46952177	2.57795247	2.40422945	2.71637001	2.4622833	2.67075628
NONHSAG017106	3.1528304	3.01865288	3.06614558	2.86965808	3.12435555	3.06822221	3.23167152	3.15088255	2.79327868
NONHSAG017112	4.88285169	4.40570129	4.33263594	4.37378456	4.62684651	4.36968338	4.72320792	4.3817202	4.41563409
NONHSAG017118	3.47251858	3.5367683	3.63009643	3.39497295	3.44175157	3.39820251	3.60300507	3.46313301	3.57721976
NONHSAG017164	4.81098397	4.44553491	4.70074276	4.43111431	4.6320741	4.70544721	4.42457157	4.87617719	4.82879246
NONHSAG017207	4.25499255	3.42186006	4.23910962	4.28155342	3.81430669	4.05620652	4.48731567	4.70016949	4.54755245
NONHSAG017233	2.99841464	2.92725661	3.31032241	3.10394566	3.04772535	2.69765877	3.27766042	3.04363043	3.04559974
NONHSAG017237	6.25474271	5.59138346	6.4189553	6.63773288	6.57670564	6.52834121	6.39593237	7.11305294	7.2708505
NONHSAG017254	4.88576955	4.9201158	5.16936483	4.41669902	4.91538213	4.36090609	5.08803011	4.66633812	4.83047733

NONHSAG017262	2.68618903	2.80012566	2.98147106	3.03298833	2.78000317	3.11308391	2.88098046	2.79317503	2.85898643
NONHSAG017302	3.28176613	3.59020038	3.50186991	3.57987576	3.5574944	3.42265059	3.54200571	3.58903937	3.5704846
NONHSAG017304	3.19653637	3.03224785	3.37197517	3.10608887	3.06793148	2.76745553	2.84153894	2.94701685	3.32734946
NONHSAG017314	3.95121092	3.92054909	3.94080837	3.92797514	4.17863988	3.51581552	4.00152034	3.75516292	3.94294128
NONHSAG017331	7.54969098	7.35421474	7.84193541	8.08093614	7.97752856	7.78509941	7.76148665	7.99992775	7.87837524
NONHSAG017346	4.62548828	4.4114688	4.73426222	4.87008123	4.91260508	4.6500488	4.80901365	5.22914098	5.06860296
NONHSAG017352	3.20954924	2.91038454	3.0978287	2.9653955	3.11452991	3.0188244	3.12077419	3.02269521	2.93590451
NONHSAG017356	4.28724851	4.10799534	4.3880714	4.43019486	4.56475849	4.19006004	4.29129647	4.60927429	4.34706727
NONHSAG017360	3.9068665	4.2802591	4.42837767	3.87949706	4.15441487	3.90575994	3.95555873	4.03240929	3.97606999
NONHSAG017376	4.55030696	4.57188401	4.96258598	5.07071046	4.87933113	4.90835695	4.84892198	5.01519896	4.74154192
NONHSAG017384	5.20840003	5.72163602	5.51708661	5.53080674	5.5041452	5.5205887	5.36653463	5.43839195	5.52840607
NONHSAG017387	4.62145842	4.19320184	4.42522754	4.21219469	4.51985517	4.18982227	4.8464611	4.5857521	4.45579812
NONHSAG017486	3.92940528	4.67356941	4.84962677	3.91864117	4.70528616	4.91024336	4.81929607	4.58739678	4.78802497
NONHSAG017508	2.45307593	2.55956112	2.48670242	2.19323703	2.39685791	2.35412835	2.33548592	2.39890365	2.39058621
NONHSAG017514	2.55546447	2.49275464	2.54170516	2.47257542	2.61090888	2.73196201	2.34554121	2.66606594	2.34736746
NONHSAG017529	5.36091741	5.03766092	5.40027778	5.15950227	5.49421334	5.16710944	5.42530427	5.13899939	5.10060239
NONHSAG017532	3.24020202	3.5035849	3.52674299	3.17844507	3.49641806	3.39479041	3.14057081	3.45371491	3.40444091
NONHSAG017549	3.44703576	3.21569663	3.19808027	3.30038157	3.5875248	3.25563387	3.28868269	3.45715697	3.34225431
NONHSAG017579	2.13389635	2.19803369	2.56542559	2.36375063	2.48915676	2.35756976	2.37615134	2.37126026	2.44552718
NONHSAG017593	4.69457575	4.47573794	4.58951331	4.52932012	4.64741004	4.44037698	4.54590847	4.88247908	4.48087089
NONHSAG017594	4.89923217	5.0393288	5.18851912	4.92137027	4.96633471	4.73117446	5.02916182	4.77814954	4.9603047
NONHSAG017604	3.23248692	3.29670073	3.26930644	3.13294313	3.6109809	3.16021357	3.47638107	3.36703234	2.98585703
NONHSAG017613	4.24320696	4.20544211	4.36839469	4.19397607	4.31130284	4.17536586	4.21489218	4.19454984	4.1777487
NONHSAG017640	2.30548384	2.30755621	2.29222901	2.21637738	2.27665304	2.21597124	2.55497039	2.22106589	2.37622909
NONHSAG017669	2.85214717	2.90206671	2.94205285	2.78865058	2.8389699	2.86744137	2.93266797	2.83043621	2.99472688
NONHSAG017670	3.5863451	3.48189163	3.85959405	3.55163348	3.6310029	3.42327187	3.35644044	3.50865511	3.5403071
NONHSAG017671	2.91609555	2.75235277	2.91018593	2.63299421	2.8443743	2.60689392	2.97269923	2.84796647	2.66587345
NONHSAG017675	5.88950157	5.9347353	6.36422133	6.55817347	6.05751437	6.4840964	6.31545074	6.47762824	6.41638592
NONHSAG017708	6.19972676	6.09659373	6.45082488	5.57689823	5.97013029	5.72165479	6.64885813	5.79733989	5.86555898
NONHSAG017741	4.37703211	4.4873318	4.70451354	4.31201425	4.73969009	4.34805885	4.65533046	4.39972558	4.52495279
NONHSAG017742	4.3971181	4.30255472	4.42781952	4.19087629	4.65526901	4.26092817	4.68753399	4.37211746	4.49081407
NONHSAG017775	2.83054938	3.02349439	3.28494112	3.02808286	2.8493659	2.76061359	3.36308964	2.78246741	2.96710582
NONHSAG017777	3.1652501	3.07992211	3.30027496	3.23710261	3.19689936	3.26231791	3.40239784	3.10785119	3.27310241
NONHSAG017785	2.81009259	2.7797184	2.96842003	2.69506784	2.6463135	2.70471413	3.16286874	2.70820613	2.86354634
NONHSAG017797	5.61766878	5.41846366	5.7902073	6.01260678	5.9589481	5.64854121	5.82589254	5.85661393	5.97392553
NONHSAG017837	5.36020465	5.50190875	5.51500208	5.1789639	5.36449602	5.35070531	5.28731961	5.58345141	5.43014782
NONHSAG017855	5.08167013	4.94601305	4.7290274	5.13499157	5.15130156	5.17679316	4.88384855	5.33358878	5.01196025
NONHSAG017868	4.60490826	4.44935897	5.05661091	4.79154361	5.00240652	4.78730937	5.17883703	4.69522423	4.79154361
NONHSAG017869	7.95934676	7.95699102	7.66600058	7.88286058	7.91184971	7.87262633	7.85075667	8.26772547	7.98483487
NONHSAG017885	3.64259422	3.69137075	4.05417323	3.86992468	3.92884185	3.72778374	4.24158623	3.70313395	3.80939444
NONHSAG017934	3.70120702	3.55442201	3.58081712	3.54083894	3.36201039	3.64399824	4.22628738	3.54917132	3.80039179
NONHSAG017937	4.99833684	5.05677209	5.04417183	4.60982862	5.04916746	4.80323758	5.22225334	4.49564823	4.83872994
NONHSAG017938	3.27647417	3.17941444	3.60898468	3.45695314	3.61985013	3.68182155	3.62745703	3.42076924	3.54228022
NONHSAG017946	3.6377508	3.92180043	3.83014014	3.94867195	3.87388305	3.93304265	3.69619651	4.03522081	4.16266517
NONHSAG017952	5.34422454	5.13552033	5.28552346	6.02924202	6.00284382	5.85301058	5.32731392	6.60368458	5.94089206
NONHSAG017967	2.93790395	3.07881723	2.97926049	2.66372931	3.1153226	2.69265302	2.99371621	2.71624334	2.85422005
NONHSAG017971	3.8300772	3.75481815	3.92954098	3.81661547	3.88425654	3.69288304	3.97256484	3.83942107	3.82048301
NONHSAG017972	2.64874355	2.75828117	2.87962336	2.5239525	2.75236153	2.74125258	2.78235904	2.6606367	3.04895251
NONHSAG017996	3.98224399	4.51725484	4.02295332	3.85997011	4.26012707	3.99149995	4.29198574	4.0972967	4.20881155
NONHSAG018000	3.45734918	3.42177422	3.60797772	3.26700077	3.38475305	3.25772403	3.42657257	3.44754431	3.19729688
NONHSAG018018	5.12151729	4.97390222	5.25810876	4.93736569	5.15898956	5.03485216	5.65340184	4.97554981	5.10622303
NONHSAG018031	3.19148625	3.16569286	3.1774978	3.11045985	3.59708305	3.15849491	3.44609146	3.02396365	3.18335222
NONHSAG018033	3.35468551	3.13452346	3.47812143	2.93066817	3.24864669	2.92755803	3.42833763	3.02346865	3.1192906
NONHSAG018039	3.46272023	3.54779183	3.71058211	3.54064906	3.64987588	3.48134841	3.54566304	3.63534277	3.55706624
NONHSAG018040	3.30248423	3.00493841	3.08953381	3.27899688	3.16959624	3.00785912	3.05445307	3.1466577	3.21209603
NONHSAG018059	3.74732407	3.89446623	3.69831273	3.71775362	3.69315591	3.60696008	3.83902355	3.49764504	3.80942688
NONHSAG018075	4.51049189	4.62527838	4.40606156	4.85091375	4.75055039	4.69208854	4.73748097	4.46107404	4.42706727
NONHSAG018098	3.980651	3.66678089	3.71302122	4.0173764	4.02423532	3.52228968	3.8085403	3.8202708	3.79558679
NONHSAG018192	4.93102193	5.06317352	4.91601925	4.91508903	5.136035	4.75910244	5.26157329	4.93506094	4.83313319
NONHSAG018224	5.60795189	5.64039565	5.92674916	5.58208488	5.65217377	5.41175337	5.85397181	5.58096907	5.76417564
NONHSAG018230	5.66512444	5.65680506	5.57927349	5.42531263	5.7967181	5.5466148	5.84641062	5.66916587	5.61660202
NONHSAG018271	3.78833082	3.86373117	3.64325304	3.57556952	3.87588318	3.64548916	3.51204721	3.54025529	3.85845132
NONHSAG018315	10.9333709	10.75737792	11.24281042	11.15686347	11.24161479	11.36241598	11.17387286	11.2749665	11.37460515
NONHSAG018332	3.77510643	3.9361825	3.45643145	4.25553724	4.02140258	3.83847028	3.64538948	3.86462508	3.65453122
NONHSAG018336	5.04664355	4.70474029	4.77928228	4.54864883	4.70474029	4.43742686	4.7860016	4.48892089	4.80692384
NONHSAG018350	3.87535226	3.60093196	4.05300435	3.75595938	3.71971214	3.28836482	4.17346083	3.80663115	3.98137056
NONHSAG018377	5.69601622	5.6967336	5.97563094	5.84454387	6.14502716	6.03956633	6.01026109	5.80475068	5.77793578
NONHSAG018379	5.96039712	6.20469683	6.72840477	6.77981794	6.55712089	6.58055334	6.69585976	6.26037807	6.52788258
NONHSAG018419	4.71239238	4.50517948	4.39301803	4.31225691	4.50664193	4.29795147	3.98222327	4.44007998	4.30869397
NONHSAG018426	3.56639488	3.4551128	3.30619004	3.42526658	3.61450042	3.68952104	3.22980795	3.56813654	3.38928962
NONHSAG018457	4.69092969	4.63532489	4.75460274	4.99360455	4.74624547	4.61175854	4.65276048	4.69345343	4.6440645

NONHSAG018506	3.94929137	3.97082266	4.02164471	3.93463602	4.02100435	4.13556971	3.80219145	3.92454888	3.72156991
NONHSAG018536	5.28069495	5.38796135	4.95828594	5.09963374	4.72248731	4.92468734	4.55889855	4.74433012	4.67927547
NONHSAG018538	8.46780064	8.50696805	8.36345022	8.79700623	8.44623119	8.41358481	8.37485559	8.34626164	8.30809973
NONHSAG018575	3.98142673	3.97889781	4.16910454	3.94357745	4.13760882	4.00110593	4.55779586	3.87363058	3.79770652
NONHSAG018592	4.93344068	4.73811007	4.90668845	4.70604626	5.13360405	5.2793806	4.64849419	4.93669077	5.28468263
NONHSAG018593	4.9727933	4.71451289	4.91105105	4.56591432	4.96741699	4.3831893	4.65381555	4.56377574	4.79419221
NONHSAG018602	4.39410961	3.90268311	4.43513163	4.02367311	4.14416006	3.70557067	4.3790051	4.0688599	4.21859499
NONHSAG018627	6.69091013	6.85652693	6.18695278	6.23124143	6.44655146	6.19396768	6.65946582	6.32821209	6.3813028
NONHSAG018649	4.84698147	4.84310226	5.1020635	5.07573921	5.06029289	4.88874344	5.00509716	5.16034637	5.02825985
NONHSAG018664	5.49177564	5.38977899	5.09688565	6.13760174	5.42539655	5.2479578	5.08093232	5.97805487	5.89141481
NONHSAG018700	2.80390588	2.87872715	3.06905226	2.96342147	3.05167277	3.14469185	3.2019952	2.86392395	2.89878517
NONHSAG018743	3.90436369	3.83194467	3.63917466	3.60112554	3.60995988	3.55756539	3.88080489	3.77333762	3.90883164
NONHSAG018775	2.12081261	2.53519945	2.28942075	2.29689303	2.38207502	2.69205012	2.49413616	2.43890388	2.38199112
NONHSAG018846	6.0183671	5.8723576	6.07634	6.37050184	6.27789149	5.85634817	5.85283391	6.27231975	6.42769424
NONHSAG018860	5.96544564	5.97725364	5.98042159	6.07203295	6.04644668	5.98789031	5.8317315	5.99488159	5.9624704
NONHSAG018901	3.56523166	3.81617754	3.95647882	3.45279184	3.98546462	4.26504619	3.79833591	4.31132033	3.73554439
NONHSAG018925	5.14684619	5.2694246	5.23163768	5.0127108	5.2222165	4.94324885	5.21144057	4.98965974	5.21695611
NONHSAG018928	3.20210205	3.08965539	3.20192762	3.18724291	3.08079701	2.9130882	3.25966098	3.07887732	2.98867235
NONHSAG018930	4.89453255	4.7505333	5.02601443	4.28260075	4.76128699	4.50856847	5.12644257	4.45578014	4.69418737
NONHSAG018934	5.4368876	5.42481004	6.15872272	6.50049778	5.99315597	6.32456976	5.97525355	6.27195104	6.24839522
NONHSAG018936	3.00437446	3.1673251	3.27632577	3.04105063	3.05990499	3.15320342	3.19438518	2.98742205	3.3229475
NONHSAG019021	3.42313048	3.25396956	3.48745401	3.20896005	3.36482123	3.53722571	3.49000738	3.42930085	3.22909134
NONHSAG019026	3.74978776	3.82831761	4.34323255	3.71787863	3.8631446	3.49109712	4.19144262	3.54989143	3.86871051
NONHSAG019061	4.69892947	4.43233446	4.54295749	4.36659143	4.55292832	4.36315238	4.4806961	4.60047461	4.47722069
NONHSAG019100	5.22661163	5.37832714	5.75357464	5.56996616	5.38089594	5.84020555	5.0232403	5.54080191	5.48399958
NONHSAG019129	5.51736359	5.23834006	5.95850492	6.05341028	5.82974363	5.70221883	5.89347783	6.18825877	6.00167327
NONHSAG019176	5.86591354	6.18938699	6.30105418	6.21085115	6.49058496	6.35918929	6.05421317	6.29282961	6.18813773
NONHSAG019180	2.60279217	2.45978438	2.64154053	2.71493491	2.63634651	2.48875981	2.85339247	2.5541315	2.23728893
NONHSAG019199	2.32562681	2.43534672	2.23200874	2.46396599	1.94660322	2.29971521	1.99016133	2.19052922	2.46103591
NONHSAG019275	3.28679395	3.05150609	3.27315699	2.87666564	3.15669751	2.91838181	3.2514827	3.05545305	3.2007157
NONHSAG019322	2.5513038	2.43087222	2.42674924	2.20180054	2.41291737	2.45689357	2.43242708	2.50287011	2.37386911
NONHSAG019335	3.23319758	3.08549577	3.13707209	2.88014366	3.45936812	3.09451165	3.65809245	3.2390055	3.14351824
NONHSAG019355	4.88522807	4.75372286	4.94047373	4.42109185	4.78110028	4.30963902	4.99510242	4.58552318	4.84500877
NONHSAG019359	3.15745816	3.42319314	3.65271323	3.3187014	3.28604795	3.40020739	3.52569111	3.30301153	3.21281309
NONHSAG019389	2.71835109	3.05581527	3.20022921	2.90424021	2.84150485	2.69199884	3.05557194	3.00501582	3.04655472
NONHSAG019395	3.25925048	3.25107568	3.52590965	3.3197028	3.34225644	3.22889793	3.66573533	3.29127948	3.40043996
NONHSAG019396	3.05887922	3.29452864	4.13364671	3.30645719	3.36496809	3.45536367	3.3142276	3.61313719	3.17366021
NONHSAG019423	4.05250239	3.58187084	3.48607624	3.72505677	3.65695385	3.51181652	3.71007711	3.74495648	3.76108808
NONHSAG019434	3.47381956	3.22097592	3.41974308	3.01456487	3.33976577	3.32955765	3.68823714	3.25501798	3.34291583
NONHSAG019460	2.76450264	2.85387058	2.96842003	2.68940751	2.82825601	2.56824011	2.79999258	2.77571141	2.74232354
NONHSAG019464	5.54464037	5.62421977	5.58253487	5.52022476	5.52397685	5.40785959	5.93128748	5.23403249	5.42259799
NONHSAG019471	5.3719032	5.35367794	5.40099327	5.21930857	5.48359533	5.30169607	5.38003337	5.32312444	5.32428072
NONHSAG019477	4.73154865	4.61934588	4.74778839	4.81327455	4.89813902	5.01220296	4.53237765	4.81222846	4.39041459
NONHSAG019496	3.5992113	3.5734846	3.75203385	3.54635956	3.90448847	3.57140604	3.5406404	3.43348808	3.70671494
NONHSAG019516	2.88269893	3.08629399	2.98242503	2.75136547	2.95781734	2.95143755	2.87685544	2.89439853	2.90173405
NONHSAG019517	2.40579886	2.45819238	2.36901618	2.51066551	2.32454452	2.38330914	2.44377295	2.3633898	2.38741204
NONHSAG019583	4.35777734	3.97441313	4.34623157	4.25983758	4.52358422	4.13100174	4.42983239	4.10181951	4.24873858
NONHSAG019638	3.47008863	3.25107568	3.27395653	3.08623745	3.17363962	3.17309275	3.5406404	3.1205925	3.20966648
NONHSAG019649	2.28299692	2.05662742	2.33368383	2.14591429	2.16496555	2.16838447	2.26255227	2.17969967	2.27146616
NONHSAG019657	2.59282111	2.56313853	2.61964309	2.48633607	2.64452771	2.49072127	2.59481409	2.52505479	2.54099209
NONHSAG019671	3.61742958	3.72350716	3.88239894	3.56403476	3.80560334	3.55012576	3.95401522	3.52952994	3.8769795
NONHSAG019696	2.54225333	2.53657212	2.48596243	2.60977668	2.59131097	2.61489405	2.59893299	2.61630235	2.45633161
NONHSAG019733	5.07868787	5.14886569	5.01341808	4.99863585	5.09225535	5.01836964	4.94104824	5.10916895	4.94849536
NONHSAG019747	3.99596652	3.93517143	4.2219662	3.93613656	4.15196693	3.78225638	4.39594669	4.15633672	4.12015026
NONHSAG019755	3.58371719	3.75889127	3.63834447	3.58030386	3.61555281	3.68176199	3.75728383	4.16743954	3.6764222
NONHSAG019799	3.08362654	2.75423781	2.86891734	2.88492997	2.88786884	3.00376049	2.96939636	2.96558721	2.96615171
NONHSAG019803	4.86085484	4.53410908	5.05365375	4.91199835	4.77607142	4.89312399	4.44958064	5.20508606	4.86810055
NONHSAG019855	8.36484362	8.34394508	8.07369261	8.2337083	8.16774386	8.30890387	8.21867645	8.2565152	8.18562124
NONHSAG019888	4.70301736	4.32007286	4.5589236	4.31016915	4.67788082	4.29600862	4.65606088	4.07389166	4.68297517
NONHSAG019921	3.37311982	3.47929572	3.3223691	3.28757721	3.46290845	3.44952611	3.38399109	3.54436499	3.39299626
NONHSAG019946	6.27254647	6.09399054	5.20825705	4.96835142	5.18217913	4.93130031	5.13364665	4.78551324	4.87272916
NONHSAG019954	2.64658354	2.68478185	2.82461071	2.70190488	2.77704634	2.46628694	3.08091291	2.85195262	2.67505292
NONHSAG020059	3.14721468	3.19472206	3.37662191	3.78963529	3.33693226	3.65936965	3.31602423	4.12802982	3.69924308
NONHSAG020065	3.5214875	3.18309314	3.22393242	3.15241895	3.07052381	3.08914326	3.51576862	3.19398063	3.22357529
NONHSAG020081	8.18659126	7.99062024	9.95025287	10.05841724	9.69755039	9.82047905	9.66504432	9.88364805	10.03295354
NONHSAG020082	3.66768384	3.66339909	3.44435541	3.11165896	3.73645188	3.41448124	3.44264152	3.37616908	3.13621375
NONHSAG020086	5.17205209	5.53185231	5.47143623	5.16560093	5.31417912	5.12929013	5.45681977	5.38842322	5.37400848
NONHSAG020094	2.16431192	2.28711383	2.42616314	2.13479934	2.55554475	2.35320434	2.22582267	2.13505212	2.2746799
NONHSAG020096	4.23222163	4.11949633	4.2460962	4.46513595	4.14082131	3.96326945	4.44825573	4.50835705	4.45259471
NONHSAG020099	8.0866226	8.08090662	7.9214839	8.060134	8.10213266	7.85570228	7.82512055	8.06902572	8.2423581
NONHSAG020107	3.30810332	3.35227928	3.2192569	3.19910542	3.28545796	3.22708789	3.262803	3.2539578	3.1717116

NONHSAG020130	2.62722838	2.41176912	2.56675138	2.54712007	2.6604504	2.45689357	2.73876088	2.47135313	2.44460127
NONHSAG020182	4.62435188	4.75826909	4.87021711	4.43575046	4.83857917	4.53067607	5.01964885	4.78201676	4.7713049
NONHSAG020190	4.07630245	3.97417178	3.81194647	4.26319132	3.89169452	3.94301478	3.98139864	3.72164816	4.1151686
NONHSAG020197	4.24056086	4.367729	4.57036271	4.1048696	4.0167301	4.12110626	4.47341618	4.03616027	4.13908155
NONHSAG020211	5.62327226	5.71729447	5.6332548	5.19701131	5.67514686	5.33242636	5.51567228	5.5282611	5.5469459
NONHSAG020225	4.31900776	4.24208238	4.42995851	3.89107939	4.1293842	4.02464206	4.65180278	3.88461487	4.22514205
NONHSAG020230	2.83262934	2.81915806	2.87673914	2.67439662	2.95984122	2.61851229	3.20148662	2.77698754	2.80398239
NONHSAG020240	3.14523058	3.01659386	3.05698794	3.00939978	3.02427881	3.07445004	3.19704456	3.16154261	3.08084556
NONHSAG020323	4.79980246	4.647823	5.15457988	4.88472152	4.93205677	5.04472207	5.30654279	4.95092671	4.93566284
NONHSAG020329	6.34556456	6.09552313	6.62167698	6.58366576	6.58307462	6.51930691	6.49230642	6.64136892	6.65531033
NONHSAG020334	4.47386976	4.22210345	4.37977437	3.75061996	4.35238559	4.04697835	4.38680281	4.17294215	4.38267868
NONHSAG020338	4.00036015	4.3006881	3.92150821	4.09176297	4.15293871	4.21667645	3.86566809	4.1201407	4.12371893
NONHSAG020339	7.05873791	6.99545649	6.72226344	6.84369046	6.64674613	7.05285934	7.00545853	6.69319738	6.81067487
NONHSAG020347	7.36281237	7.07013965	7.24376585	7.32855471	7.47131908	7.19829324	7.23916757	7.82219683	7.56757979
NONHSAG020419	3.74321543	3.84151888	3.25587234	3.50690206	3.42972801	3.63587646	3.54908083	3.67418335	3.51033451
NONHSAG020424	4.18594334	3.93005515	4.20688472	4.13159101	3.92164089	3.78838363	4.25177612	3.87525698	3.91390066
NONHSAG020428	3.24580098	3.05424969	3.19076764	3.06177916	3.14227599	3.16924277	3.04697559	3.39111388	3.02999462
NONHSAG020432	6.38702397	6.36811332	6.50907728	6.23342222	6.27993947	5.58566944	6.55543123	6.35259641	6.20490723
NONHSAG020434	3.49031781	3.52157721	3.6913115	3.27034107	3.367196	3.46373734	3.85520647	3.33568536	3.52291564
NONHSAG020483	3.02056923	3.10198826	3.21652586	3.27188737	3.2268689	3.12762332	3.76361169	3.04289563	3.1793209
NONHSAG020539	5.04076415	4.97390222	5.32997906	4.88810062	5.11914907	4.87654694	5.28114321	5.11149749	5.04258029
NONHSAG020550	5.25923866	5.47792204	5.82110889	6.5486045	5.82013817	6.03712523	5.39236747	5.86191072	5.97467423
NONHSAG020567	4.00452953	4.34092382	4.14913023	3.89821567	4.22185112	3.91860981	4.47383815	3.98930728	4.10099078
NONHSAG020580	4.11091356	4.19344299	4.38573799	3.99797991	4.23175311	4.01275809	4.6142478	4.20955289	3.98023909
NONHSAG020618	4.58505634	4.64028955	5.15141292	4.98363213	4.86188119	5.21106561	4.6282398	4.68326688	4.33533555
NONHSAG020638	7.04602119	6.94882112	7.06152468	7.23958529	7.03599607	6.92394367	7.1199622	6.80384872	6.88058073
NONHSAG020650	2.73075258	2.65165231	2.65473088	2.89410213	2.5204664	2.76519536	3.35925559	2.5908736	2.6392323
NONHSAG020678	5.96847581	5.6411012	6.08531723	6.25923419	6.13775003	6.23096374	5.91583318	6.09804295	6.05370828
NONHSAG020737	2.58617271	3.14498029	3.23034366	2.99962414	3.54773751	3.24429397	3.81092991	3.37889543	3.49796192
NONHSAG020798	2.26731468	2.44851881	2.5810168	2.48459835	2.37341784	2.61160489	2.28182591	2.59687084	2.43147434
NONHSAG020806	4.40375537	4.27224282	4.46145199	4.06736699	4.34778741	4.29863293	4.36089017	4.07016561	4.57660518
NONHSAG020809	3.10885254	3.43295987	3.23284489	3.02366161	3.03326966	3.04279166	3.80082827	3.10137759	3.30058229
NONHSAG020830	3.98286443	4.01475027	4.12554215	3.91294799	4.28580741	4.22788983	3.71822703	4.03307493	4.27734126
NONHSAG020837	2.44571127	2.45930662	2.63014661	2.58927345	2.7873276	2.36999326	2.58008972	2.54893637	2.56218616
NONHSAG020855	3.18242134	3.14075905	3.20336028	3.18312384	2.99216981	3.19286178	3.18979574	3.23349815	3.17951193
NONHSAG020867	2.13328066	2.08881418	2.10730391	2.14220634	2.0396226	2.06960568	2.30357431	2.1655895	2.12191438
NONHSAG020883	3.84827263	3.96789494	3.95904433	3.84785728	4.00279923	4.04125677	3.63282277	4.0518464	3.72473138
NONHSAG020899	2.71030152	2.6661967	2.73818106	2.59595877	2.56197472	2.70471413	2.70970916	2.62513844	2.63766654
NONHSAG020905	2.87791251	2.82757584	3.34247091	3.57975087	3.50348543	3.33918542	3.42222552	3.26878247	3.38352517
NONHSAG020941	5.80083158	5.53068119	5.94758624	6.61693512	6.61026074	6.18511007	5.99382768	7.13933661	6.57566664
NONHSAG020993	5.04698228	5.01606178	5.1585724	5.14327284	5.06955534	4.82083426	5.24272912	5.25168892	5.13568385
NONHSAG021003	5.43967465	5.08029497	5.07630303	5.15950227	5.20144777	5.022074	5.52049106	5.06847587	5.37096013
NONHSAG021015	5.53738882	6.0026735	5.59819885	5.49294808	5.65752562	5.73131748	5.54228067	5.61374606	5.59709951
NONHSAG021045	3.97629342	3.98843398	4.27282182	3.96581777	4.02291354	3.91583788	4.47075999	3.95499007	3.85282023
NONHSAG021143	3.33676751	3.86236529	4.16263747	4.16316212	3.78711252	4.559379	3.86070565	4.09104611	3.9778978
NONHSAG021154	4.64803596	4.74922525	4.87988283	4.93491971	5.41282684	4.68515699	4.73163173	4.74922525	4.6369106
NONHSAG021213	4.9865758	4.41883337	4.53668437	4.66703631	4.88874948	4.96691593	4.66473057	4.39679172	4.70504072
NONHSAG021241	3.02340265	2.91560538	2.93607496	2.94776023	2.75039684	2.67760257	3.04697559	2.66910028	2.7416283
NONHSAG021246	4.95270607	5.1652857	5.85697123	6.34810229	5.80425806	5.91010545	5.58077445	5.8719811	5.74551141
NONHSAG021274	3.9256364	3.74890167	3.7787333	3.61474102	4.111623	3.67704408	4.23270558	3.95185039	4.09772169
NONHSAG021287	5.52847883	5.70741544	5.74862747	5.43802468	5.67209544	5.33369324	5.78704047	5.5925713	5.65354
NONHSAG021374	2.70680407	2.69209168	2.69016198	2.76519536	2.62100824	2.56706224	2.83856919	2.74109124	2.68821102
NONHSAG021403	5.44805075	5.51204287	5.30871502	5.25400554	5.77193676	5.24245329	5.64921307	5.35408936	5.40424807
NONHSAG021436	4.08186071	4.18564979	3.85558266	4.27192517	4.4651506	4.13236138	4.04886824	4.11810077	4.26267639
NONHSAG021444	5.55306039	5.51405791	5.76112201	5.56175419	5.57694488	5.64596693	5.71100965	5.47900067	5.58698433
NONHSAG021459	2.18543719	2.21165112	2.28953906	2.19321048	2.21859599	2.1231029	2.51483585	2.17778617	2.24285725
NONHSAG021464	3.53936558	3.53032857	3.38534957	3.08698192	2.91564978	3.0736554	2.94818206	3.13323975	3.24326214
NONHSAG021479	3.52763011	4.29502452	4.77873205	5.00909234	4.82255358	4.75569627	4.3950224	4.91220826	4.73044564
NONHSAG021502	3.24384794	3.37729415	3.65987373	3.31035078	3.52945196	3.00406973	3.70844169	3.27533008	3.42919012
NONHSAG021504	3.44469956	3.07748112	3.11758731	2.82244239	3.2146668	3.15190729	3.30877169	3.11634572	3.18740462
NONHSAG021505	3.65042817	3.6401029	3.93253951	3.57527072	3.79456866	3.115176	4.00132915	3.40857418	3.68939899
NONHSAG021537	3.48812894	3.5350937	3.54115307	3.52109487	3.53404307	3.52387319	3.52734437	3.42515087	3.52682504
NONHSAG021546	4.32228031	4.09149723	4.21095494	4.22138339	3.79874756	4.1149479	4.02971764	4.11810077	3.81808421
NONHSAG021557	6.54150044	6.60106815	6.03211439	6.4683852	6.0789381	6.48750611	5.84872889	6.32083949	6.39649534
NONHSAG021577	5.088099	4.60863311	5.02602854	4.28012847	4.6380122	4.27074391	4.63855498	4.49953074	4.46109073
NONHSAG021582	5.01739273	4.79001123	4.93842959	4.83344624	5.00737385	4.94605207	4.94175726	5.1408979	4.91456963
NONHSAG021608	2.66027659	2.75453833	3.01147427	2.82100884	2.86601433	2.84219931	2.80953945	2.63727615	2.6122216
NONHSAG021662	4.94558705	5.02916207	4.66931495	4.66847535	5.0434148	5.27081312	4.80381906	4.75058857	4.83755339
NONHSAG021671	2.79239574	2.86721813	2.81959995	2.81138797	2.92276805	2.78592951	3.00423866	2.81455758	2.7551983
NONHSAG021696	3.80237307	3.69187246	3.71852224	3.68367208	3.87641063	3.88780725	3.83448802	3.90510039	3.96842522
NONHSAG021701	4.42570686	4.17284865	4.4127339	4.6934839	4.76796445	4.71310957	4.59824081	4.80187294	4.655248

NONHSAG021755	6.06116992	6.36568433	6.23075091	6.73050836	6.24887573	6.55469623	6.322456	6.47348699	6.35039436
NONHSAG021758	3.80511501	3.62353657	3.70810461	3.81975844	3.60957352	3.26231791	3.82039123	3.52192986	3.72061008
NONHSAG021801	5.66137554	5.33329231	5.90250644	6.05800509	5.65391278	5.94106798	5.62469782	6.24410466	6.05126282
NONHSAG021825	4.25475054	4.15349326	4.19975604	4.329866	4.09968248	4.151364	4.07097166	4.36072591	4.067237
NONHSAG021851	3.71058211	3.51834603	4.06461891	4.24785151	3.96127737	4.45493837	3.74713287	4.71656917	4.40237897
NONHSAG021881	4.52799621	4.24585189	3.79839484	4.09379355	4.13878283	4.19819681	4.25155584	3.89347249	4.20458564
NONHSAG021904	4.27637374	4.3282874	4.35664064	4.22538752	4.64030063	4.37327597	4.47053468	4.33468834	4.36486396
NONHSAG021905	5.65192015	5.42819188	5.52509151	5.83022529	5.62232514	5.71307093	5.67151346	6.16659192	5.94479477
NONHSAG021933	2.17606839	2.22336725	2.21864266	2.23749423	2.16798826	2.16307889	2.17030959	2.20857914	2.17337505
NONHSAG022026	3.3590926	3.45327865	3.46898061	3.34048334	3.39701747	3.09944905	3.3696884	3.10075969	3.27130201
NONHSAG022055	4.07051389	4.17667186	4.37847825	4.03815034	4.15423719	3.94857501	4.46065799	4.05353946	4.07398608
NONHSAG022065	4.96441244	5.07809034	5.32177231	5.17933649	5.26445617	4.97548543	5.39451739	5.16750975	5.37669935
NONHSAG022068	4.60287794	4.52099996	4.62607406	4.77843822	4.66975794	4.11732178	4.90919726	4.6234112	4.37762023
NONHSAG022073	2.65709087	2.56177959	2.62257251	2.95576401	2.77122164	2.59396256	2.59893299	2.56916078	2.86026028
NONHSAG022088	3.88443452	3.72736634	3.92281872	3.48291692	4.12478035	3.36430269	3.57949797	3.58228589	3.46601882
NONHSAG022089	2.98971912	2.82647225	2.92395083	2.69376775	3.15877868	2.81387202	2.88602138	2.88147964	2.93041952
NONHSAG022092	4.67944162	4.80890691	4.75134265	4.40865388	4.66722327	4.68100382	4.55574346	4.71328356	4.8703974
NONHSAG022177	5.26336282	5.21803718	5.13891217	5.08551976	5.33263428	5.06422106	4.93565656	5.10962468	5.23269458
NONHSAG022214	4.47921961	4.40854618	4.45010204	4.28968142	4.35424625	4.10751799	4.35428439	4.24343036	4.37375778
NONHSAG022224	4.01854387	4.32794394	4.34759343	4.63929469	4.16809425	4.30152198	4.10253582	4.21061549	4.16876163
NONHSAG022252	6.41924675	6.15089151	6.7324437	6.81252394	6.76841346	6.65807399	6.65200098	6.87322056	6.78976767
NONHSAG022256	3.69387068	3.50465536	3.90590006	3.71678878	3.66365439	3.69735591	3.61054969	3.98781044	3.72163248
NONHSAG022261	3.7134001	3.37466977	3.4434365	3.81716511	3.48227258	3.33156198	3.7355662	3.38784813	3.3225415
NONHSAG022297	4.36169899	4.57159183	4.69004746	4.25726035	4.63447529	4.36983389	4.5505674	4.34029958	4.54966166
NONHSAG022308	4.85444038	4.74808877	4.89565682	4.85915483	5.05940289	5.10254228	5.06880918	4.63523057	4.81677949
NONHSAG022311	5.09501327	5.34749238	5.64015341	5.53698017	5.73074788	5.60141664	5.59298189	5.38628735	5.05223634
NONHSAG022328	4.88812782	4.87138684	4.81775422	4.71390113	4.87582118	4.7148599	4.72242077	4.90928878	4.82216315
NONHSAG022337	4.96240662	5.12607363	5.29767981	4.95785004	5.03397675	4.73115281	5.20838439	5.00984222	5.08589658
NONHSAG022342	2.38015539	2.62049769	2.54540227	2.58458629	2.36373104	2.53681743	2.39332346	2.60428584	2.5437182
NONHSAG022353	3.37110876	3.53088776	3.3642677	3.36234647	3.66931502	3.23308398	3.43984163	3.46903726	3.58487664
NONHSAG022357	7.07393525	7.17220575	7.74832088	7.91238486	7.48906202	7.72393512	7.40272698	7.72481731	7.71777309
NONHSAG022358	6.69956609	6.53685944	6.46269674	6.77445634	7.07034793	6.60053115	6.43196775	7.33823839	6.98816041
NONHSAG022437	5.58704215	5.48189861	5.71216895	5.47466499	5.71008557	5.38883407	5.76751087	5.75631614	5.72496579
NONHSAG022492	7.93025167	8.05148415	7.97408909	8.01370678	7.72139422	7.77643742	7.62665246	7.91270313	7.83913027
NONHSAG022576	3.4794002	3.33926976	3.38168189	3.60279745	3.43968019	3.28651486	3.28115709	3.93871672	3.53702561
NONHSAG022580	5.33288085	4.91560164	4.83545936	4.85321725	4.81054471	4.591848	5.12906705	5.03500904	5.00294844
NONHSAG022588	7.10870408	7.24000116	7.85425861	7.61365886	7.7726335	8.05636956	7.74022383	7.67704387	7.81510486
NONHSAG022627	3.68404585	3.7473042	4.09225432	4.00903668	4.12491182	4.05846241	3.89969188	3.86746225	4.05341258
NONHSAG022655	3.40755422	3.46364419	3.83708068	3.28617528	3.55694646	3.6307375	3.605752	3.40394803	3.41383766
NONHSAG022656	4.07038305	3.85544181	4.03725795	3.75548507	3.94396753	3.80531637	4.25182595	4.00743955	4.13586899
NONHSAG022668	4.35038785	4.29476556	4.45343365	4.25903488	4.59431478	4.3064332	4.49131906	4.27992455	4.49913137
NONHSAG022676	3.76128722	3.90082558	3.99590003	3.73379025	4.06215845	3.55743585	3.84871172	3.92795207	4.1232849
NONHSAG022677	3.79924478	3.92489251	4.01326209	3.93882702	4.19885167	3.68332576	3.98003878	3.89629552	4.20897176
NONHSAG022735	5.50487532	5.55539911	5.74885611	6.23075091	5.61862691	6.03239976	5.61744798	5.87106823	5.86011976
NONHSAG022759	8.02126049	8.12389168	8.41747709	8.63815953	8.6777851	8.64437762	8.4706323	8.54291134	8.49447841
NONHSAG022817	4.74293498	4.54036609	4.55351141	4.5523333	4.50454536	4.27419182	4.40477373	4.34491247	4.43349107
NONHSAG022830	5.19923511	5.28086543	5.27541037	5.20044315	5.09071788	5.12104654	5.30635599	5.09096418	4.99855981
NONHSAG022841	5.54826635	5.49962695	5.61449989	5.30365451	5.69958012	5.46088303	5.46556476	5.50801954	5.60628594
NONHSAG022852	3.55781075	3.54279199	3.29293849	3.15746226	3.33508592	3.39695613	3.43716917	3.14342812	3.51359856
NONHSAG022858	3.11933444	3.0417313	3.07642038	2.93402606	3.05015893	2.76519536	3.12425699	3.16657596	3.0585871
NONHSAG022867	5.00465927	5.27211726	5.25335505	5.30751051	5.38104673	5.04871843	5.12075364	5.31722263	5.05184804
NONHSAG022906	3.54755752	3.46890179	3.73937317	3.44217628	3.63490006	3.6291238	3.59580126	3.48662205	3.47058948
NONHSAG022924	5.96623514	6.02978542	6.05463101	6.14287634	6.31964814	6.15046293	6.1336565	5.9844436	6.04683133
NONHSAG022925	4.65396641	4.83189487	5.2605083	5.44304765	4.99774925	5.01911264	4.86133073	5.62512983	5.3497348
NONHSAG022957	4.45499525	4.43963423	4.60564505	4.12372235	4.37712392	4.42275618	4.75237902	4.13992948	4.26413798
NONHSAG022968	5.58112223	5.74356788	6.03744761	5.33163284	5.56122027	5.6582277	5.92614587	5.45522436	5.84171467
NONHSAG022969	3.36719952	3.45640596	3.40405927	3.19172016	3.50077012	3.35681259	3.70767165	3.43540537	3.48647043
NONHSAG022994	5.48850123	5.72690971	5.91794158	5.92472307	5.98650824	5.99400951	6.03077888	5.88502533	5.90215668
NONHSAG023009	2.8198636	2.55123574	2.62124633	2.67671856	2.6721414	2.74408562	3.09231784	2.60394835	2.67243879
NONHSAG023036	6.36619688	6.67752313	5.66160178	6.26387288	5.80323019	6.20252777	6.004454	5.9205686	6.00876223
NONHSAG023052	4.80890691	4.58864533	4.7219921	4.27320577	4.88603626	4.63507546	4.93124951	4.82350673	4.71687896
NONHSAG023067	5.52914376	5.61766878	5.42242862	5.83121486	5.60829356	5.60373207	6.00796295	5.64598969	5.63538451
NONHSAG023085	2.99729392	2.9517106	3.2491125	2.69129161	3.03290348	2.92892828	3.24274666	2.88661295	2.86408793
NONHSAG023088	5.84123049	5.71774723	6.08545432	5.69765482	5.72305254	5.75879024	6.11468196	5.8081475	6.00679386
NONHSAG023107	3.82471135	3.73375957	3.55438848	3.44026577	3.61133325	3.47929275	3.75551356	3.55987016	3.93006992
NONHSAG023114	3.15664185	3.43246591	3.66268149	3.57579605	3.66252865	3.60097742	3.74807077	3.42682996	3.60754422
NONHSAG023118	3.97633151	3.87850756	3.89794509	3.57135366	3.95720594	3.82405358	4.27994616	3.66560821	4.0361478
NONHSAG023129	5.49453372	5.7272571	6.10107922	6.75593318	6.38445829	6.59122727	6.42774231	6.0764209	6.39669529
NONHSAG023173	4.83449564	4.57615329	4.57255229	5.19215156	4.69065985	4.8300823	4.38879404	5.29323634	5.20231841
NONHSAG023180	3.18117975	3.21123051	3.61369856	3.2646718	3.44319793	3.03060532	3.6611692	3.02269521	3.12813683
NONHSAG023185	3.29628219	3.13421324	3.4883171	3.24576587	3.60957352	3.1700739	3.16404352	3.37697153	3.44612099

NONHSAG023194	2.79037325	2.74123244	2.88410356	2.31454664	2.54347047	2.6785722	2.94320739	2.38976251	2.44709486
NONHSAG023237	3.96532595	4.00214367	4.06129035	4.00873353	4.27596732	3.85228392	4.16345806	4.08679056	4.4844806
NONHSAG023238	3.12312434	2.80409636	3.42626634	3.44343414	3.32097545	3.05349497	3.49223998	3.02735634	3.37630311
NONHSAG023245	2.4423523	2.42964654	2.75063747	2.75660883	2.55131529	2.47816312	2.4717124	2.52888225	2.53701957
NONHSAG023247	2.17030843	2.25979002	2.29177483	2.06200034	2.3153478	2.16823749	2.27613772	2.35407597	2.19117496
NONHSAG023281	2.50125249	2.39753122	2.49629494	2.27847739	2.41879062	2.49533698	2.44743772	2.37496151	2.68789546
NONHSAG023286	3.19072443	3.15224323	3.15136728	3.14309046	3.32250986	3.07738407	2.85988931	3.19374204	2.83576946
NONHSAG023298	3.57457544	3.39580118	3.37742252	3.39001159	3.78704498	3.28594607	3.50045514	3.54931614	3.58949101
NONHSAG023327	4.09484887	4.12997199	4.02221125	4.40449689	4.48425769	4.39731821	3.87153803	4.37024012	4.34954999
NONHSAG023350	3.43714678	3.35183067	3.48518819	3.559739	3.29668803	3.46074293	3.42777668	3.40355409	3.34135067
NONHSAG023373	4.06713762	4.02248162	3.92903529	3.89629552	4.07846839	3.89786514	3.86717112	3.89629552	3.9240192
NONHSAG023390	5.37695068	5.54519511	5.49460625	5.18596855	5.02210807	5.28112949	5.44709246	5.48218028	5.50969578
NONHSAG023427	2.95038843	3.05996942	2.88210762	2.93476522	2.96581783	2.98508461	3.02123572	2.85124151	3.13415085
NONHSAG023436	6.39885488	6.37562418	6.46583523	6.09965937	6.49395387	5.9865382	6.60032332	6.29269097	6.350664
NONHSAG023437	3.77084071	3.43152386	4.0774515	3.26543115	3.7441439	3.44386005	3.86119925	3.52211236	3.73411041
NONHSAG023456	2.94903913	2.82186392	3.16758169	2.80175697	2.88598636	2.89024661	2.95674916	2.86392395	2.97011614
NONHSAG023530	2.40932342	2.17451008	2.22122288	2.28868479	2.36924414	2.40313679	2.21305941	2.21183477	2.03081674
NONHSAG023534	2.8291795	2.9515244	2.7922926	2.67519748	2.87417752	2.49098023	2.73584394	2.78246741	2.53836456
NONHSAG023539	5.55132546	5.20211091	5.62129699	5.670083	5.75909349	5.65229925	5.72339121	5.82574156	5.73365133
NONHSAG023582	3.69213347	3.33765716	3.20110323	3.53362666	3.46414118	3.58081712	4.03228293	3.19128029	3.0887301
NONHSAG023627	3.18435906	3.22546786	3.13495003	3.28878872	3.50391105	3.14051409	3.1715414	3.22866121	3.00077009
NONHSAG023639	2.37227063	2.50174151	2.46394932	2.42680425	2.49804189	2.32028445	2.73744157	2.45674662	2.48696618
NONHSAG023666	2.69008838	2.48701486	2.77593466	3.00570653	2.71406561	2.79578594	2.48634909	2.57646285	2.45889868
NONHSAG023691	2.83871579	2.8329372	2.71702094	2.8329372	3.05636754	2.51829232	3.0598085	2.6398894	2.85560872
NONHSAG023704	4.31609928	4.18471519	4.02337784	4.4218455	4.24949958	4.13460839	3.83463722	3.96282695	4.35298936
NONHSAG023707	2.75040585	2.51892797	2.60658677	2.60658677	2.62579849	2.32645267	2.85455856	2.49185304	2.53116692
NONHSAG023711	3.01156313	3.0388495	2.93632593	3.10389308	3.0070022	2.96892631	2.98575853	2.91984476	3.10264303
NONHSAG023714	3.99569169	3.92489251	4.29090447	3.98223864	3.93139644	3.66996358	4.35451887	3.77897166	3.74372272
NONHSAG023715	2.40932342	2.30836206	2.65077114	2.6614713	2.56197472	2.32008328	2.3746258	2.43188393	2.35375042
NONHSAG023765	3.43029245	3.1094948	3.27559729	2.93707221	2.97425171	2.83438615	2.90054674	2.82381353	2.84536885
NONHSAG023770	2.92323299	3.06355872	2.9266254	2.51342642	2.96938953	2.87098323	3.3276442	2.80814236	3.0887301
NONHSAG023771	2.72208656	3.07679614	2.91850942	3.03807053	2.84647993	2.95005972	2.92086559	3.01491476	2.70542063
NONHSAG023772	2.11993171	2.42598349	2.39318851	2.25750423	2.26568409	2.06132089	2.28792387	2.30443811	2.44436188
NONHSAG023787	4.92879704	5.03443262	5.14561128	5.15553878	5.1200341	5.02021514	5.05405169	5.1661103	5.02830986
NONHSAG023790	2.49437982	2.65273183	2.58713928	2.55511641	2.78246741	2.62273158	2.69992625	2.58488052	2.55511641
NONHSAG023801	4.97568201	4.81947196	4.74912705	4.79058209	5.00868496	4.53511269	4.99821073	4.74110481	4.71918782
NONHSAG023810	3.34316035	3.18046372	3.47390561	3.2643675	3.35871547	3.56743409	3.04697559	3.36385577	3.17461342
NONHSAG023839	4.90147881	4.76148438	5.12397832	4.7537032	4.98937103	4.93539116	5.23847961	4.95193305	5.0592143
NONHSAG023915	3.08061815	2.99708113	3.33925767	3.04940831	3.09021935	3.0576657	3.12852771	3.19851979	3.10318552
NONHSAG023920	4.74995506	4.76338106	4.94897074	4.563932	4.73859455	4.54181089	4.81278937	4.5479594	4.61163419
NONHSAG023923	3.49052861	3.04125421	3.45116215	3.76271883	3.72503287	3.56952362	3.72503287	3.61037197	3.83517812
NONHSAG023954	5.47346075	5.48458258	5.55512404	5.35504108	5.75619244	5.30701723	5.63228559	5.28619381	5.42774019
NONHSAG023966	5.38414942	5.42100965	5.46163516	5.13804808	5.37518519	5.28990343	5.43294172	5.40740022	5.252514
NONHSAG023972	4.2926105	4.00322496	4.45176637	3.84558124	4.21929097	3.7949392	4.67863335	4.11261058	4.05341258
NONHSAG024013	3.46510034	3.33224342	3.62992775	3.42469992	3.42037841	3.49406952	3.85336842	3.27300225	3.42318977
NONHSAG024040	4.89556277	5.0355143	5.11591375	4.90639846	5.23338294	5.04366288	4.95746676	4.89458963	5.18278488
NONHSAG024043	3.00532731	3.29370431	2.94938819	2.95287948	3.02300833	2.88401585	3.04697559	2.80270725	3.16333185
NONHSAG024046	5.14870946	5.15735042	5.11591375	5.31513679	4.86460187	5.5783529	4.98708137	4.83675995	4.85378117
NONHSAG024080	2.67519579	2.69258827	2.4909276	3.10394566	2.94258006	2.95600953	2.6379729	2.81728076	2.87554512
NONHSAG024104	2.48406158	2.40105702	2.21327007	2.17109873	2.38400176	2.30624602	2.46631569	2.21722534	2.31727497
NONHSAG024105	3.26700175	3.39122128	3.31547046	3.3099491	3.43556187	3.2797264	3.28604795	3.3456493	3.33215175
NONHSAG024135	4.37090731	4.54822576	4.95375836	4.38795239	4.54573737	4.06923573	4.67087789	3.97726194	4.47453193
NONHSAG024143	2.56197472	2.45007513	3.17524657	2.37097948	2.7249921	2.67420268	2.31288538	2.62974414	3.13258813
NONHSAG024146	2.91983373	2.90942275	2.69004134	2.90321436	2.89461157	2.78341198	2.91305744	2.77206901	2.7379688
NONHSAG024157	3.41328026	3.59260528	3.87423782	3.43993228	3.56143613	3.48405637	3.68790123	3.41893332	3.6505919
NONHSAG024162	2.4907294	2.88831743	2.83760244	2.61240774	2.83608825	2.55736346	3.09046303	2.61679233	3.01673623
NONHSAG024182	5.46414652	5.26614197	5.2922498	5.1033908	5.39711602	4.78726093	5.42773988	5.13538558	5.1642385
NONHSAG024209	5.55064176	5.71791072	5.57123273	5.8564927	5.61687723	5.63901641	5.80274825	5.66427993	5.74551483
NONHSAG024232	4.44378338	4.46978899	4.39545828	4.39705591	4.49154258	4.35824719	4.43144727	4.26751643	4.34495977
NONHSAG024234	3.57739954	3.84131425	3.45636701	3.58500351	3.36666291	3.66661534	3.3678562	3.31589723	3.5568288
NONHSAG024244	4.34520361	4.34114593	4.52972672	4.36635867	4.36870059	4.358815	4.30197074	4.30197074	4.42452766
NONHSAG024248	5.98637426	5.92866401	6.29639651	6.53226571	6.1012593	6.26414269	6.43142627	6.14536504	6.29588792
NONHSAG024264	4.1876229	3.83012406	4.15508479	3.96309551	3.74775908	3.89303127	4.13279071	3.87438022	4.07577629
NONHSAG024327	3.72564363	3.42186006	3.04570356	3.34799009	3.22314988	3.18455678	3.28604795	2.98088198	2.87770641
NONHSAG024381	5.71541754	5.57778526	5.55413612	5.53478041	5.47351277	5.44292842	5.39657684	5.39609663	5.4299252
NONHSAG024388	5.31702055	5.49434043	5.5101761	5.04123543	5.78298428	5.43573494	5.34560561	5.45215837	5.56294538
NONHSAG024397	5.83021167	5.95203874	6.34996622	6.46558866	6.21797001	6.39792667	6.24569758	6.20946343	6.33981137
NONHSAG024439	6.22615072	5.96150394	6.26216444	6.00780688	6.25522804	5.89094466	6.28292881	6.04962848	6.0849054
NONHSAG024442	3.89629552	3.62609669	3.72090515	3.67968515	3.78271428	3.68229167	3.77630474	3.64490224	3.54817352
NONHSAG024446	3.83687952	3.57323013	3.54472334	3.56711211	3.74119651	3.64832265	3.57820375	3.45166959	3.61398146
NONHSAG024447	2.95088698	2.99537086	2.84194382	2.94294614	2.89785927	3.3182166	2.81704522	2.86029557	2.75384913

NONHSAG024529	7.71448948	7.72843938	7.45568903	7.52509003	7.8508637	7.47195031	7.72549414	8.07367445	7.68790986
NONHSAG024540	3.44334265	3.39841303	3.59418019	3.40305845	3.81501261	3.91810375	3.56571727	3.62487854	3.75335784
NONHSAG024542	4.89640343	5.22445865	5.25275404	5.20520789	5.30355025	4.97873	5.16195359	5.16763835	5.3678492
NONHSAG024613	4.28587876	4.52958979	4.27781045	4.21112504	4.39813314	4.62312248	4.44650658	4.42030864	4.3033667
NONHSAG024628	2.24132752	2.68284026	2.80131919	2.75522827	2.67337547	2.64965939	2.40505096	2.61350614	2.51829853
NONHSAG024729	3.29989498	3.65160016	3.3642677	3.60284858	3.45559473	3.49745634	3.64186184	3.40276744	3.73790044
NONHSAG024733	2.16576989	2.21752384	2.32325993	2.19565034	2.41352495	2.30035123	2.27721183	2.41733607	2.36119638
NONHSAG024740	2.90023265	2.85837393	3.09766661	2.76433413	3.22081446	2.71006813	3.22289799	2.78181746	2.92312515
NONHSAG024741	3.49715732	3.36293142	3.3605473	3.40743462	3.49837103	3.37129882	3.43775779	3.38406918	3.33974199
NONHSAG024742	9.09939874	9.2442446	8.94934364	9.25647687	9.1015016	9.05355344	8.9789466	8.93444011	9.02699357
NONHSAG024745	3.04610779	2.8557246	2.89443034	2.89741392	3.21323635	2.97088399	3.2638576	3.07842278	2.91560538
NONHSAG024752	4.35648982	4.55679233	4.613713	4.13166771	4.4762233	4.4939069	4.57668878	4.53288481	4.38632658
NONHSAG024859	2.63890747	2.39480806	2.32234281	2.48587117	2.59197093	2.72178169	2.68210709	2.6526298	2.70102574
NONHSAG024870	2.88603161	2.51200644	2.84041996	2.71563244	3.1324924	2.56705312	2.93887267	2.76713743	2.82552362
NONHSAG024871	6.23732653	6.24945374	6.52053243	6.25997945	6.50479738	6.21947217	6.61590841	6.41053861	6.31293312
NONHSAG024916	4.53717491	4.53676967	4.98223918	4.5409845	4.66291325	4.41294741	4.73686229	4.39456236	4.61447834
NONHSAG024953	4.10954025	3.80055236	3.9164998	3.29181413	3.70512638	3.6546874	4.1823139	3.62226816	3.75260406
NONHSAG024968	4.07929933	3.84302583	4.09034638	3.49821039	3.98348917	3.39720063	3.70065111	3.61577191	3.59721641
NONHSAG024985	6.89471116	6.98024885	7.00441314	7.54772878	6.81035829	6.70468472	6.88277277	6.86903801	7.03184368
NONHSAG024988	5.75464624	5.63106157	5.87156409	5.61158941	5.76699336	5.57593708	6.08897684	5.71011002	5.85706414
NONHSAG024994	4.82918561	4.85307834	4.62963429	5.13024064	4.85749312	4.90068053	4.71808609	5.42962868	4.71052366
NONHSAG025009	5.69510849	5.69708364	5.27075835	5.3999188	5.68282241	5.4941495	5.35484504	5.53988743	5.38076089
NONHSAG025049	5.35956593	4.84590748	5.38941685	5.32404871	5.0897817	5.00706625	5.38849781	5.41486834	5.3623026
NONHSAG025052	6.64049333	6.57987417	7.13541831	7.34873272	6.91209712	7.34499101	6.9054203	7.10287732	7.1364277
NONHSAG025054	6.38880449	6.3015271	6.78504916	7.41052639	6.73197085	6.95963461	6.91781251	6.86785429	7.21099436
NONHSAG025151	5.16293595	5.50447995	5.41000675	5.67317299	5.77111841	5.59337137	5.38369513	5.69323825	5.30497361
NONHSAG025174	4.02289207	3.79470423	4.05060933	3.70892495	3.86776115	3.90233569	3.9457335	4.07253441	4.21417206
NONHSAG025195	7.11586205	7.19633152	6.80212743	6.71138548	6.71003979	6.76152346	6.37041323	6.96135719	6.62790569
NONHSAG025224	6.00829647	5.86347642	3.87542309	3.72195073	3.6789554	3.69871978	3.04697559	4.04020865	3.17656565
NONHSAG025230	2.8987911	3.1394712	2.92385324	3.03880923	2.93023935	2.87004188	3.32469337	2.99082469	2.9233332
NONHSAG025265	2.86971882	2.84039341	3.11773394	2.75193788	2.67075628	2.6419836	3.13025819	2.70562838	2.70750435
NONHSAG025272	5.02197752	4.96068632	5.30038185	4.82566324	5.30135083	4.49782751	5.47226942	4.91445445	5.02070977
NONHSAG025274	2.63273209	2.85655426	2.96084982	3.05312201	2.95602548	2.74185505	2.71038661	2.65957381	2.57663228
NONHSAG025288	2.56197472	3.06823956	2.63533036	2.92032041	2.87988312	2.77043465	2.81704522	2.92646148	2.85067299
NONHSAG025295	4.28703501	4.3454252	4.38339463	4.22008461	4.14205551	4.29187828	4.42739218	4.31153794	4.3534892
NONHSAG025343	3.79427674	3.6287362	3.73668489	3.71499432	3.83563963	3.62995233	3.76302787	3.85749931	3.94780557
NONHSAG025349	4.13646754	4.23006214	4.34921215	4.26135339	4.3785892	4.33468014	4.30740082	4.25520354	4.41501977
NONHSAG025352	3.89629552	3.38887994	3.58775429	3.34584592	3.36895945	3.14419918	3.83632588	3.31665184	3.53039751
NONHSAG025367	5.28106308	5.24394892	4.6568353	4.719383	4.65204141	4.16233557	4.29953088	4.59469594	4.20333022
NONHSAG025376	5.59996126	5.32081535	5.65275536	5.66671093	5.42882172	5.5067642	5.28113461	5.61374606	5.50426092
NONHSAG025378	5.2698658	5.13358812	5.2728201	5.34443027	5.32596035	5.45834442	5.22844724	5.4786329	5.44032078
NONHSAG025393	4.7198835	4.70425264	5.01817219	4.31322776	4.79354068	4.53350974	4.81749617	4.835325	4.88371108
NONHSAG025422	3.04405949	2.91560538	2.84721004	2.84170494	3.16844196	3.01719147	3.00330177	3.16644377	2.92892755
NONHSAG025431	4.98132748	4.9147302	4.87851018	4.76392294	5.15902302	4.5818834	5.07530659	4.69765108	4.78677798
NONHSAG025438	2.99423569	2.7478765	2.97122429	2.58459573	2.72225377	2.67532936	2.82097081	2.85375995	2.95503412
NONHSAG025448	5.09776738	5.20670508	5.20029094	4.83236139	5.13217588	4.8261069	5.1664237	5.02794742	4.99670092
NONHSAG025466	4.36054704	4.40191569	4.86535192	4.4542606	4.60619292	4.67532464	4.80146619	4.66608614	4.61252445
NONHSAG025491	5.10207384	5.19053731	4.95559743	4.99533072	4.99663728	4.76488619	5.10571927	4.95092671	4.93539116
NONHSAG025494	3.85455142	4.03325915	3.96420944	3.76760613	3.93976935	3.60740626	4.20104005	3.83788302	4.16471844
NONHSAG025500	5.09257473	4.73554631	5.13931874	4.86335557	4.98143394	4.91825422	5.18051782	5.02383284	5.0151552
NONHSAG025531	4.09501528	4.24804551	4.33936317	4.21683375	4.17375446	4.09315394	4.34520218	4.18487921	4.38437636
NONHSAG025536	2.73820639	2.68324012	3.42084056	3.60768077	3.1108829	3.56416269	3.49642888	3.44402417	3.59575223
NONHSAG025542	4.21896061	4.23517149	4.22800273	3.78177458	4.11741627	4.09471691	4.57866714	3.92589616	4.0478447
NONHSAG025649	4.21589797	4.19205437	3.81332764	4.15559568	4.32247906	4.33124591	4.2001386	4.22942299	4.1455768
NONHSAG025666	4.68785717	4.78649564	4.68671829	4.52238323	4.73980003	4.57624107	4.77459539	4.56059801	4.6563868
NONHSAG025675	3.32379489	3.52169837	3.39332639	3.53968869	3.34601372	3.40773194	3.48271862	3.5014652	3.50038658
NONHSAG025695	3.98286443	3.99892608	4.16073167	4.15344337	4.65526901	4.53100453	4.22684121	4.42110729	4.08522998
NONHSAG025699	2.36510648	2.61306371	2.31310465	2.61165353	2.33753545	2.50552859	2.51857153	2.43973482	2.33920122
NONHSAG025705	3.89629552	4.15621059	4.43431801	4.36728006	4.13216387	4.84753959	4.0267869	4.33556105	4.48592807
NONHSAG025710	2.96576752	2.86893365	2.90066816	2.54918178	2.81764099	2.63181524	2.78009009	2.74655802	2.68535188
NONHSAG025719	4.12506515	4.27858604	4.26715836	4.24355478	4.34141226	4.29322027	4.20355916	4.30432667	3.93924651
NONHSAG025790	5.03980589	5.25803794	5.21797125	5.1034702	5.18018085	5.31230914	4.87988283	5.23326838	5.18358839
NONHSAG025812	2.41223072	2.59263739	2.63239627	2.40356043	2.61501003	2.53697068	2.59893299	2.42972743	2.47531165
NONHSAG025869	4.43499234	4.14849884	4.26447426	3.94202559	4.25420268	4.10099247	4.23745594	4.14636368	4.22579021
NONHSAG025872	3.34316035	3.08433501	3.26891085	3.42675967	3.32276533	3.14456041	3.40951143	3.48930949	3.17461342
NONHSAG025881	2.19074045	2.17585067	2.56872457	2.290746	2.38632614	2.23026477	2.64782281	2.21876953	2.37324261
NONHSAG025897	5.54881305	5.59779272	5.79999996	5.17152246	5.55258747	5.23833846	5.72869374	5.43165656	5.49543124
NONHSAG025979	2.25724661	2.37385035	2.30208425	2.25041159	2.36861825	2.32936172	2.27447472	2.48688246	2.25041667
NONHSAG025986	3.35188334	3.46194254	3.27574588	3.1867163	3.35370888	3.16982375	3.71126424	3.29800395	3.28557064
NONHSAG026033	2.9205951	2.78866041	2.84366964	3.08899168	3.03119351	2.9534678	2.98372627	2.94853354	2.76064426
NONHSAG026052	3.22109457	3.0689127	2.95513217	3.45555756	3.09214541	3.21403648	2.96311485	2.88745549	3.16337649

NONHSAG026094	4.55432601	4.76805012	4.91797347	4.82580101	4.87527366	5.03160791	4.80595032	4.53520033	4.70620852
NONHSAG026202	3.92660651	3.75545741	3.73279835	3.75156899	3.73729824	3.76485581	3.87505522	3.96734181	3.71972042
NONHSAG026216	3.8475299	3.76727497	3.93909613	3.31532559	4.04021692	3.67965959	4.07849048	3.38556731	3.62265079
NONHSAG026311	4.32569836	4.51361341	4.98144007	5.03568774	4.99920238	4.87800522	5.09249908	4.95092671	4.96603586
NONHSAG026360	4.13883771	4.26590926	4.14856319	4.06434649	4.23246211	4.33769834	4.46591125	4.16752189	4.42462407
NONHSAG026376	2.29460112	2.45330405	2.44249131	2.54700628	2.48417101	2.43609532	2.45602529	2.19295307	2.33572101
NONHSAG026396	3.99312483	3.51103548	3.49496745	3.5711454	3.57145975	3.53657335	2.83066522	3.68611645	3.54476688
NONHSAG026410	4.46052242	4.27183992	4.11764031	3.80406782	4.01794804	3.95675246	4.70154569	4.318318	4.35020071
NONHSAG026448	2.26292092	2.31579601	2.35080252	2.44724924	2.2801512	2.36006534	2.21302154	2.34172012	2.27509022
NONHSAG026475	5.00829342	5.13497244	5.13168237	5.09954763	4.83364721	5.21373493	5.29910734	5.09242905	4.97307627
NONHSAG026539	2.38285018	2.50973025	2.4860432	2.25436442	2.38934064	2.29689303	2.48243531	2.44774667	2.39914836
NONHSAG026542	2.69226641	2.35470543	2.7407338	2.59533259	2.98748776	2.87269878	2.71270065	2.96490627	2.7950054
NONHSAG026552	6.62558139	6.54828956	6.94919653	6.79115314	6.54273636	6.90370624	6.73963945	6.89205551	7.1077797
NONHSAG026569	5.64882503	5.58498203	5.65994722	5.60205591	5.54369221	5.45860305	5.44722701	5.35607662	5.51888666
NONHSAG026592	4.44868052	4.39096244	4.55972158	4.06397794	4.54088299	4.36754405	4.76277618	4.14115187	4.15721656
NONHSAG026607	4.0261844	3.9254886	4.06836517	3.78619908	4.15940737	3.91027237	4.13321484	4.0648187	3.88599271
NONHSAG026618	4.78088723	4.63181762	4.82008347	4.654985	4.77275169	4.63024607	4.69361223	4.83158502	4.58697828
NONHSAG026620	3.44209517	3.34911652	3.40287845	3.22113977	3.46677253	3.601809	3.43024789	3.437294	3.34845113
NONHSAG026634	4.03074423	4.25596013	4.00490035	3.6960028	3.85944044	3.56293686	3.93154388	3.70301668	3.84288294
NONHSAG026661	6.39825853	6.42143438	6.46273129	6.34172185	6.58907896	6.39458476	6.30231332	6.6349652	6.53844638
NONHSAG026676	2.45649106	2.30457509	2.41927432	2.56527209	2.3778367	2.27506984	2.42829508	2.34268211	2.47655513
NONHSAG026716	7.97724292	7.8711295	7.96851225	7.79028762	7.87072978	7.94221413	7.90802448	8.02515998	8.03058934
NONHSAG026725	6.46009882	6.4422925	6.56975108	6.18253272	6.5225913	6.26031168	6.85031867	6.60220713	6.62501297
NONHSAG026729	4.78672412	4.73164639	4.77586491	4.68407499	4.52205813	4.43675301	4.9997205	4.23333165	4.43112455
NONHSAG026751	3.29208638	3.2278104	3.67805419	3.58396753	3.4045517	3.28538275	3.48501776	3.36058021	3.33740138
NONHSAG026771	4.79016906	4.91051304	4.88162542	4.73232219	4.88103363	4.64146397	4.92682825	4.77007609	5.08416312
NONHSAG026775	5.79487028	5.70390988	5.99806752	5.53986827	5.78570348	5.56157959	6.03049462	5.79553734	5.91385149
NONHSAG026798	2.82168444	2.78767531	2.44813063	2.57317829	2.95281831	2.76824209	2.59893299	2.75770375	2.93355895
NONHSAG026834	5.85530565	6.10628035	6.20002002	5.87761456	5.98363724	5.97931624	6.3493739	5.973274	6.11658574
NONHSAG026851	6.06825196	6.18104367	5.85301058	5.81797236	6.08389769	5.89483032	6.2177381	6.03431599	6.00900035
NONHSAG026854	4.95572235	4.69772854	4.76232633	4.73093613	4.62656446	4.75584041	4.25420429	4.88746619	4.62861344
NONHSAG026890	2.90136601	3.01933402	3.12405052	2.93019425	2.94720466	2.89049072	2.93197805	2.96449866	2.95499004
NONHSAG026912	5.55050949	5.4325895	5.65937544	4.94995438	5.77342961	5.13952447	5.70539928	5.42657147	5.55311004
NONHSAG026940	5.24220488	5.84552214	6.17669215	6.39744245	6.06115852	6.09345053	6.18061531	6.43768622	6.17963852
NONHSAG026942	4.33740898	4.27378532	4.67340836	4.36955637	4.41957553	4.26794522	4.8581932	4.50063053	4.52987388
NONHSAG026944	6.48457333	6.42950203	6.40647702	6.83170523	6.50568744	6.51149728	6.59573309	6.48059661	6.41079184
NONHSAG026956	5.33978447	5.0845898	5.32126193	5.16493659	5.30282613	5.17244611	4.80013969	5.41334289	5.52103232
NONHSAG026967	4.80960822	4.8476137	5.08961002	4.44823846	4.75406022	4.57898869	5.05775552	4.7636551	4.90126484
NONHSAG026969	2.63042626	2.90799015	2.87703231	2.90511587	2.6696828	2.68318189	2.91731649	2.56801183	2.75113179
NONHSAG026973	2.64888135	2.66277291	2.94589427	2.57288549	2.70259597	2.59867689	3.02364194	2.94299948	2.86617922
NONHSAG026985	3.73073861	3.76307528	3.70375904	3.69692688	3.68520192	3.6508919	3.73389109	3.64346935	3.72593537
NONHSAG026995	5.39860298	5.24896406	5.34403223	4.9916486	5.14086492	5.1505559	5.31667032	5.03019328	5.04577577
NONHSAG027017	3.56789096	3.41191468	3.50358407	3.31698488	3.66884206	3.53751178	3.41628899	3.4514222	3.60145455
NONHSAG027018	4.6587427	4.78654089	4.62151942	4.33964163	4.71973915	3.87811886	4.69771993	4.44413352	4.55288742
NONHSAG027025	4.87793804	4.96837936	5.04482745	4.96037787	5.00247837	4.83843218	4.86341284	4.90560542	4.84651177
NONHSAG027026	3.76391276	3.487705	3.93418953	3.86818209	3.92794957	3.37778866	4.0047638	3.60811423	3.71001895
NONHSAG027036	4.00526994	4.30697176	4.15863397	3.91793766	4.31194903	3.9568548	4.40958866	4.04776903	4.34308521
NONHSAG027063	2.7333792	2.77649644	2.58933457	2.5257422	2.52903023	2.41976544	2.53232253	2.61601897	2.40753405
NONHSAG027074	3.83903151	3.97112275	4.05492032	3.93512203	3.59737996	3.8796054	4.20398154	4.05367824	3.8030818
NONHSAG027078	3.88560416	3.74845524	3.72376596	3.65053398	3.68200115	3.42219824	3.71515669	3.6483742	3.71196233
NONHSAG027116	2.34736488	2.44882504	2.62722838	2.84829493	2.61720264	2.76519536	2.31474721	2.62908151	2.5529726
NONHSAG027134	2.6745216	2.92013516	2.61264082	2.71669181	2.70447346	2.71818702	2.93049058	2.65370779	2.66800476
NONHSAG027135	2.28459746	2.33937804	2.16341674	2.14077839	2.16906379	2.1338746	2.21538767	2.28389994	2.19269043
NONHSAG027140	3.10741084	3.17566254	3.56666042	3.05459432	3.44190237	3.12736614	3.25865163	3.33576718	3.22892597
NONHSAG027170	3.6573108	3.81494527	3.57871576	4.0051725	4.14200462	3.67290585	4.58608783	3.7637487	3.71387527
NONHSAG027173	2.10602625	2.14611605	1.99232112	2.14384525	2.15919694	2.27267624	2.09297192	2.12595051	2.27467584
NONHSAG027186	4.91260304	4.3504122	4.43446068	3.99149088	4.36978535	4.09046009	4.63831237	4.23097859	4.19613171
NONHSAG027205	4.6340402	4.82419537	4.79302431	4.57854355	4.64933117	4.4696758	4.73578082	4.69681073	4.53422501
NONHSAG027215	3.19542289	3.23282525	3.39941355	3.12614814	3.34622574	3.19390899	3.27858111	3.31776854	3.20085576
NONHSAG027327	2.89928706	2.83639276	2.81734523	2.84075368	2.71174859	2.8046544	2.80876265	2.92440679	2.90087425
NONHSAG027345	2.62733926	2.47489683	2.49175839	2.67170193	2.42751008	2.31933703	2.4832046	2.49210606	2.37386911
NONHSAG027346	2.67980521	2.65055297	2.54458188	2.58201058	2.42916227	2.56997162	2.81867557	2.56301345	2.44189722
NONHSAG027354	3.50277058	3.83800486	3.37848995	3.28781519	3.11047726	3.3196792	3.16815222	3.31629388	3.49037682
NONHSAG027362	4.66296844	4.47592418	4.79973179	4.42366446	4.58028718	4.58625789	4.51587872	4.74809925	4.48354499
NONHSAG027390	4.56290771	4.45716146	4.77093009	4.23660408	4.61386037	4.38996485	4.7443793	4.45912665	4.5100562
NONHSAG027419	3.40698796	3.27479172	3.33464377	3.49819328	3.67647581	3.58306837	3.05200318	4.20994359	3.54072978
NONHSAG027464	3.96459541	3.98224872	4.1146404	4.17741341	4.21859724	4.03063624	3.87441545	4.05547926	4.16191268
NONHSAG027468	4.31340231	4.84736617	4.29629112	4.47065031	4.61968103	4.31447596	4.53516217	4.59747249	4.2904703
NONHSAG027505	3.35477765	3.55591307	3.49100873	3.45993593	3.6239349	3.45148404	3.53558027	3.42127048	3.57261863
NONHSAG027515	3.13374124	3.35980857	3.23077503	3.14195452	2.85363445	2.92107424	2.96418202	2.95198264	3.03226644
NONHSAG027519	5.8148874	6.025002	6.14024387	6.58054617	6.13235314	6.34787341	6.35827784	6.32197223	6.15060905

NONHSAG027535	4.01204305	4.23253339	4.09010255	3.91733276	3.97131428	4.27135135	3.67628604	3.84974111	3.64323447
NONHSAG027545	3.13679503	2.60690434	2.78231595	2.91594454	2.70432897	2.4437946	2.64911975	2.62638592	2.59178401
NONHSAG027550	2.22963675	2.47346782	2.33244629	2.4008745	2.30157997	2.377569	2.4286891	2.42542928	2.17877532
NONHSAG027551	2.78097178	2.94375621	2.82495676	2.70886384	2.81754648	2.74045771	2.76532208	2.60513774	2.5869824
NONHSAG027552	4.12992139	4.00162101	4.15368543	3.64297044	4.2040169	3.90562349	4.53096521	3.9664264	3.86956367
NONHSAG027560	2.97332346	2.84370554	2.93395995	2.74285387	2.91027069	2.71429421	2.92890431	2.83056738	2.54480698
NONHSAG027603	3.68013602	3.8239908	3.73130861	3.72017196	3.92888184	3.5165753	3.75613349	3.61646128	3.71221489
NONHSAG027626	6.23586456	6.11929713	6.19471767	6.26950144	6.23940634	6.23773087	5.80435249	6.72488388	6.38607687
NONHSAG027634	4.08507406	3.99284747	4.04666974	3.91745147	4.18989841	3.91750822	4.1964628	3.95205446	3.86383225
NONHSAG027639	5.62423946	5.43015669	5.55922092	5.62420053	5.64877345	5.26091704	5.51361327	5.58735238	5.65536825
NONHSAG027647	4.87795524	4.88292722	5.09364798	5.19570257	4.56383592	4.89351078	4.74265179	4.762824	4.89515193
NONHSAG027670	2.45670255	2.32926314	2.35604501	2.50967831	2.56804821	2.40932342	2.35803391	2.49907469	2.29916015
NONHSAG027674	3.39337333	2.99052637	3.5122756	3.19423564	3.45029656	3.23793767	3.54265892	3.23390183	3.06523812
NONHSAG027684	4.69219243	4.59996584	4.51626211	4.16137295	4.39202786	4.39074229	4.0485397	4.60899029	4.32922935
NONHSAG027686	3.72047257	3.72047257	3.97867536	3.53685678	3.84411373	3.8740832	3.69212736	4.35917245	3.72349971
NONHSAG027687	2.97530694	3.05561553	3.64675076	3.28361998	2.99916095	2.86440236	3.90450683	3.02269521	3.35522359
NONHSAG027711	2.85824094	3.01289569	3.06588888	2.85838754	3.01568709	2.6548577	3.05466806	2.75521374	2.94563205
NONHSAG027714	4.36176259	4.5973798	4.61983064	4.55366903	4.65128412	4.27460056	4.77620947	4.51342844	4.47617511
NONHSAG027715	4.78243351	5.17085545	5.24204084	4.72778906	5.04076415	4.96449483	5.25184728	4.86468179	5.0871238
NONHSAG027736	3.21172654	3.25186202	3.46330388	3.02886784	3.48727103	3.43923062	3.62321816	3.21667022	3.28094425
NONHSAG027755	3.74107791	3.59278965	3.26328443	4.02632286	3.28926118	3.43675458	3.31587636	3.3456493	3.30171795
NONHSAG027769	3.46862565	3.27051268	3.83370419	3.80665133	3.6771528	4.01660252	3.77927732	3.63858523	3.58718507
NONHSAG027811	3.14777158	3.46055994	3.87124991	4.14237011	3.57694751	4.05328764	3.51698373	3.90914011	3.72251233
NONHSAG027819	5.11377944	5.07876197	5.23438564	4.7784799	5.13128833	4.885426	5.43888027	4.96220129	5.07500564
NONHSAG027821	2.19694719	2.14274871	2.08915814	2.16962348	2.21629792	2.04686924	2.20979953	2.06254697	2.24094235
NONHSAG027833	2.63057178	2.88982022	2.61544068	2.78948199	2.68969118	2.58451925	2.74703959	2.8251555	2.81905679
NONHSAG027841	6.4782537	6.44101582	6.25738942	6.72320956	6.637226	6.7242462	6.09743252	6.60658207	6.40794202
NONHSAG027868	8.69604199	8.64396086	8.78839123	8.92005659	8.80446824	8.68513235	8.67693188	8.73897479	8.59525556
NONHSAG027869	8.16565691	8.25488359	8.3121088	8.90569394	8.45220993	8.51836044	8.10713257	8.34865099	8.33108296
NONHSAG027897	3.320213	3.10620464	3.4144847	3.28554017	3.518508	3.02436116	3.06868373	3.35206405	3.15541464
NONHSAG027917	3.15084677	3.32610922	3.73934751	3.03822103	3.22823101	3.00796959	3.02392839	3.27000722	3.56038217
NONHSAG027919	4.64474736	4.46751418	4.81312905	4.36751543	4.65138838	4.3695225	4.46860188	4.62596369	4.71991699
NONHSAG027954	4.96125085	5.01402371	4.9321002	4.79318197	5.00196463	4.7769597	4.81843014	4.89488983	4.88801309
NONHSAG027959	3.29547122	3.06130867	3.2224884	2.90658163	3.06312984	2.81852302	3.11590534	3.13304901	2.95098924
NONHSAG027967	2.91757363	3.13741307	3.53489136	3.19269665	3.12571046	3.04270492	3.00123956	3.17628797	3.16115235
NONHSAG027970	4.87702906	5.19840902	5.13878832	5.16673254	5.25713282	5.20418418	5.19748528	5.25478101	5.0164071
NONHSAG027998	3.33256608	3.36318926	3.3983835	3.22179841	3.36636566	3.15150894	3.46224269	3.33686494	3.46024935
NONHSAG028005	3.7988799	3.81668247	3.91483232	3.83388926	3.8544526	3.64307996	4.04915356	3.68982402	3.71200068
NONHSAG028023	3.48085798	3.99797085	3.97869161	3.59189304	3.48177084	3.88777351	3.95610727	3.60691852	3.84167943
NONHSAG028024	2.81926506	2.41794873	2.64852745	2.4464259	2.73269382	2.64580709	2.42678691	2.59764692	2.45590852
NONHSAG028072	2.65832361	2.48370779	2.70430752	2.36487881	2.47658174	2.54645772	2.61489008	2.56984229	2.86911455
NONHSAG028118	3.51373598	3.65909221	3.80403427	3.64675076	3.71670826	3.64675076	3.97300303	3.57000638	3.64060252
NONHSAG028155	4.8346477	4.64237723	4.83628449	4.58483955	4.76163269	4.58516924	4.86134026	4.69930493	4.67883797
NONHSAG028160	6.32846918	6.09390769	6.69952178	6.87568607	6.43269909	6.68862267	6.58362298	6.71269284	6.49273967
NONHSAG028184	3.81661431	3.75526183	3.7460998	4.12642223	3.7809077	3.62809703	3.74814788	3.60655182	3.60980653
NONHSAG028242	4.71418243	4.67796788	4.26053924	4.04387518	3.98255035	4.21359827	3.87449571	4.10183896	4.24440846
NONHSAG028243	3.71050231	4.00188031	3.93439055	3.36603695	3.8504093	3.73063945	3.69823141	3.77097898	3.74332273
NONHSAG028249	2.49035859	2.41895777	2.21167811	2.33722051	2.52809883	2.32268984	2.33061746	2.35569766	2.44619447
NONHSAG028283	2.92381713	3.20103224	3.3642677	3.09888786	3.15883581	2.9113244	3.19653499	3.3336431	3.12329391
NONHSAG028291	4.68437024	4.30122823	4.70887461	4.24112793	4.52072308	4.28298766	4.73642041	4.52521546	4.53810793
NONHSAG028305	2.55956263	2.5044439	2.73102707	2.95299611	2.66195193	2.81744239	2.78906105	2.36460736	2.87519966
NONHSAG028340	3.00909728	3.17504214	3.07370267	3.15436346	3.07314587	2.96606913	2.85173483	2.90806209	2.93957933
NONHSAG028349	3.64138622	3.65506086	3.4200597	3.04595422	3.44772753	3.27084365	3.79374308	3.44724759	3.27657389
NONHSAG028408	2.75845893	2.99090315	3.4263727	2.87235601	2.99890381	2.8230756	3.15914876	2.75742204	2.81660627
NONHSAG028420	2.49122029	2.74865883	2.55095805	2.36892301	2.43160337	2.41051337	2.62489879	2.43275828	2.33129658
NONHSAG028439	4.5618289	4.23923586	4.5020032	4.28010504	4.24482848	3.9395337	4.78095891	4.0448833	4.33919651
NONHSAG028464	2.5564602	2.55729734	2.92960788	2.86120772	2.76403842	2.56631292	2.77327285	2.77445827	2.78403958
NONHSAG028465	5.75559959	5.6814583	5.49525192	5.13849562	5.40333267	4.96771796	5.25681409	5.46155437	5.49091944
NONHSAG028467	6.13239597	6.17866266	6.43864711	6.03458929	6.40889217	6.16313408	6.79879893	6.22312367	6.17053177
NONHSAG028469	3.91401828	3.95741116	3.98422596	3.91326354	4.04826424	4.04727126	3.83309514	4.01090584	3.95499018
NONHSAG028525	5.22140322	4.54024418	4.97929043	6.06043329	5.33471363	5.12806934	5.01817879	5.08589329	5.06421629
NONHSAG028576	4.1571841	4.51267798	4.62025466	4.37982346	4.17495495	4.27135135	4.34821852	4.57992015	4.51662013
NONHSAG028585	6.0720631	6.00543302	6.28501599	6.30875753	6.35360394	6.29254699	6.23597794	6.3686992	6.31376459
NONHSAG028593	3.2908757	3.37508281	6.45280688	6.59332284	6.4605436	6.58092315	6.60026813	6.97003691	5.94800854
NONHSAG028595	6.2911023	6.15943044	5.7299576	6.21143123	5.71710102	5.768284	5.32544845	6.31392918	6.16558643
NONHSAG028611	2.7674892	2.6805319	2.7789733	2.77593893	2.87091111	2.78710774	2.64164786	2.78246741	2.65732593
NONHSAG028627	6.71138986	7.18566733	7.59942957	8.09661616	7.86891276	7.88551036	7.69008058	7.98406194	8.01232624
NONHSAG028726	3.25278342	3.61167561	3.52115778	3.23166915	3.34678437	3.47863468	3.3175249	3.42186006	3.26331885
NONHSAG028734	3.67069388	3.86479417	4.1289043	3.85840756	4.18823295	3.74309856	4.0162214	3.89455179	4.13937377
NONHSAG028743	3.44201767	3.45933611	3.496934	3.383716	3.29269889	3.3307265	3.77769975	3.44408238	3.4919654
NONHSAG028794	3.26353349	3.02320003	3.30221671	2.92930927	3.07853189	2.74616165	3.39322898	3.24598928	2.94010636

NONHSAG028929	4.94947451	4.91974835	4.782474	4.85036552	4.84658109	4.68595612	4.94854228	4.89240149	5.15724135
NONHSAG028931	2.67919942	2.57470672	2.69867691	2.55949265	2.70350079	2.73984621	2.58221806	2.52481442	2.77989625
NONHSAG028939	3.98286443	3.60595196	4.95818485	5.62548286	4.94108035	5.09492475	5.26760422	5.53763	5.47792204
NONHSAG028975	5.98248438	5.84214738	6.22497184	6.35453121	5.99126222	6.21619274	5.84466653	6.48793545	6.16284575
NONHSAG028980	5.48243787	5.79881948	5.28119585	5.37712483	5.26052209	5.33401571	5.37029909	5.29534136	5.1707461
NONHSAG029002	6.45822482	6.12232933	7.1667183	7.17172354	6.98827803	7.28059067	6.94674616	7.28764541	7.26134326
NONHSAG029008	3.12056104	3.39866234	3.06626209	3.16688276	3.02586842	2.98752631	2.87042823	3.14552013	2.87606932
NONHSAG029029	7.6826321	7.84250301	8.06617693	8.52088395	8.24650555	8.4311697	8.1354069	8.35058036	8.30412528
NONHSAG029047	2.92114268	3.22214288	3.05783297	2.97069648	3.11717249	2.76909576	3.10775962	3.22461721	2.8915247
NONHSAG029080	5.11097656	5.07170612	5.61427668	5.18279591	5.17933541	4.94140826	5.56275613	5.14690208	5.25872751
NONHSAG029181	3.13689251	3.41569839	3.34770019	3.1289504	3.58667659	3.04285893	3.4344421	3.33217535	3.32810702
NONHSAG029187	5.03666877	4.94017876	5.13605148	5.27535241	5.32931843	5.35514974	5.49401665	5.48378535	5.38004125
NONHSAG029193	3.84683996	3.77665327	3.39876059	4.42645674	4.28080446	3.88905104	3.64316375	4.60153589	4.14709828
NONHSAG029202	7.10759623	7.14602229	6.89735277	6.72150392	6.88047658	7.11388594	6.80150162	6.82524167	6.73279901
NONHSAG029206	4.12753608	4.19664847	4.33606395	4.24185337	4.17245172	4.20829611	4.28644548	4.36155644	4.25618253
NONHSAG029225	3.27554629	3.40371858	3.41970675	3.10394566	3.57489872	3.22021224	3.81693837	3.53908785	3.30417679
NONHSAG029338	5.39304284	5.43105983	5.26724255	5.37164226	5.82492822	5.56822514	5.43488611	5.59553461	5.40502837
NONHSAG029343	4.25187772	4.12484867	4.3828515	4.07895411	4.24294337	4.09372117	4.21497794	3.97230033	4.37423749
NONHSAG029351	4.38052772	4.10525616	4.17591898	4.42512027	4.00201032	4.2551389	4.20536134	4.48224447	4.44542881
NONHSAG029390	2.9321687	2.92532213	2.98097861	2.66736206	2.8865342	2.93062166	3.21253229	2.79082466	3.00792355
NONHSAG029407	3.36018671	3.03328377	3.27901114	3.09191026	3.3379848	2.93407828	3.61525721	3.37833061	3.22644327
NONHSAG029451	2.67874448	2.68213372	2.95656945	2.59581268	2.59203413	2.64138201	2.7611079	2.59492942	2.59951868
NONHSAG029458	5.45852293	5.40116664	4.83407783	5.75908031	5.24101088	5.27810811	5.19695618	5.25564239	5.19260634
NONHSAG029466	2.48577512	2.49803913	2.58093147	2.67919472	2.39786958	2.73225106	2.77361792	2.58785946	2.60735027
NONHSAG029469	4.04489836	4.16819948	4.55659843	4.4744677	4.20473881	4.17788248	4.5369667	4.04417036	4.21281224
NONHSAG029483	2.78124228	2.56971827	2.79618869	2.6146839	2.69018581	2.59424253	2.77209673	2.73916283	2.64769363
NONHSAG029505	2.52699827	2.49086097	2.49377072	2.59363251	2.64225193	2.86464403	2.58743779	3.03808344	2.78522197
NONHSAG029519	3.32086278	3.37371734	3.32107304	3.2128058	3.46434846	3.17908954	3.45767308	3.31831053	3.42818565
NONHSAG029550	3.02106652	2.95832736	2.98677862	3.1541594	2.89493322	3.1826801	2.94901228	3.04372013	3.16753419
NONHSAG029563	3.87382323	3.79929107	3.98382173	3.99231373	3.74258053	3.54441956	4.22001397	3.74983342	3.82911288
NONHSAG029600	6.030647	5.81362218	6.0432098	6.4939971	6.27044393	6.47804741	6.32557775	6.61356625	6.27362298
NONHSAG029615	3.07248044	3.4260555	2.93425114	2.83784077	3.15449316	3.10614596	3.08215108	3.00419322	3.15861199
NONHSAG029629	6.02539164	5.83784912	5.94940302	5.76535756	5.90170719	5.73321411	5.46917569	5.854091	5.78197084
NONHSAG029651	3.62022939	3.48075945	3.43455397	3.72841378	3.68626217	3.65369387	3.84828828	3.63124035	3.57836295
NONHSAG029656	3.03677469	3.2231938	3.72531351	2.81460728	3.12105151	3.13031433	3.02433283	3.08098899	3.0887301
NONHSAG029691	4.4036844	3.88710622	4.07106764	4.00480837	3.55501638	4.17956228	3.57794764	3.96237945	4.06944467
NONHSAG029703	3.63943916	3.33890139	3.36215347	3.80805396	3.37061254	3.6131164	3.50343055	4.38682705	3.76689633
NONHSAG029707	2.9131289	3.25107568	3.30116078	3.14033293	3.0417187	2.98105019	3.10204703	3.10394566	3.0887301
NONHSAG029741	2.46947581	2.52411672	2.67667157	2.67949233	2.61501003	2.36438566	2.62873076	2.58260825	2.71486936
NONHSAG029752	3.34254023	3.38977752	2.41221201	2.72286363	2.63018001	2.55990975	2.71038661	2.32947748	2.57239433
NONHSAG029789	2.43050847	2.71065306	2.78149275	2.44111915	2.50333264	2.90963231	2.87901287	2.55968092	2.59595877
NONHSAG029815	2.57483525	2.67035573	2.56679417	2.3935852	2.7642745	2.42197816	2.46994733	2.33053482	2.50208451
NONHSAG029818	2.72876065	2.90916733	3.00381431	3.02587504	3.01846806	2.90570718	2.87470426	3.15150111	2.8368672
NONHSAG029852	5.74209086	5.59605224	5.12481816	5.60507255	5.77557951	5.57395115	5.26124655	5.97509373	5.75443279
NONHSAG029854	5.24550674	5.18525872	5.99212239	6.61237589	6.29015826	6.49153296	6.09332761	6.59556653	6.74657841
NONHSAG029904	2.97114303	2.8329036	3.17601002	2.73270462	3.13808853	3.2643205	2.96634844	2.77071674	2.96724579
NONHSAG029916	2.63422833	2.59125002	2.68717264	2.64524584	2.99430929	2.51886425	2.87392839	2.71408415	2.60887993
NONHSAG029920	3.50784336	3.44568219	3.40074453	3.37457083	3.51514696	3.42172933	3.51883137	3.61974502	3.52209883
NONHSAG029991	2.69979186	2.58014568	2.64253325	2.51328768	2.45260074	2.46912915	2.60144915	2.45167475	2.61002604
NONHSAG029995	3.19973792	2.49035704	3.21501921	3.13252419	3.20101408	2.97232655	2.97878618	3.42488888	3.2360246
NONHSAG030001	2.71137494	2.65750936	2.67777094	2.69652873	2.74983041	2.66712172	2.83735294	2.67185832	2.61803321
NONHSAG030002	2.28396201	2.38062245	2.33368383	2.31292585	2.4482982	2.30993308	2.42279953	2.10143628	2.21232371
NONHSAG030016	3.52769211	3.61550731	3.59759353	3.48802863	3.84673795	3.64875516	3.41212174	3.54876401	3.46700756
NONHSAG030028	2.39829088	2.37966403	2.33528033	2.43366885	2.45897873	2.58115693	2.36447581	2.36261155	2.2307876
NONHSAG030049	2.56197472	2.80119662	2.73935198	2.81252092	2.54020461	2.70471413	2.88637975	2.86422699	2.79624208
NONHSAG030056	3.05887922	2.80919035	2.86650732	2.95931378	2.5913905	2.80093793	2.66776174	2.71335822	2.83462366
NONHSAG030133	2.66306426	2.5274564	2.43841898	2.29689303	2.71135698	2.47250133	2.50350329	2.49222758	2.43726683
NONHSAG030140	2.97964405	2.45007513	2.5493566	2.48560047	2.6647848	2.19074045	2.49201024	2.3373869	2.49179002
NONHSAG030166	3.37822883	3.42856602	3.39119479	3.45755708	3.31819599	3.20604291	3.55659065	3.03095193	3.21549159
NONHSAG030168	2.62722838	2.68425935	2.80196272	2.44803437	2.7406872	2.69377194	2.7706842	2.57754527	2.69096627
NONHSAG030187	2.52278138	2.49054178	2.40336461	2.50400513	2.32386703	2.44328021	2.49433864	2.51596581	2.51375605
NONHSAG030263	2.83494037	2.94238143	2.86612237	2.91998197	2.780541	2.89817014	2.91510663	2.84801713	2.81544044
NONHSAG030278	6.33105086	6.30114299	6.52077455	6.74927385	6.56305209	6.73940953	6.43544985	6.65656556	6.70880295
NONHSAG030325	4.34940474	4.23974379	4.32743136	4.19612308	4.41379636	4.15881049	4.18358962	4.11810077	4.4164938
NONHSAG030326	2.80999727	3.01012191	3.01236187	3.25298549	3.26493879	3.12843361	3.1715414	3.06297912	2.8337981
NONHSAG030351	2.87099244	2.96231028	2.8408359	2.6848832	2.85737962	2.64061692	2.75657073	2.81499319	2.67282795
NONHSAG030358	3.04994267	2.82750471	3.01079354	2.81637454	3.12098363	2.78069987	3.39973268	2.96294819	3.38299695
NONHSAG030374	2.5501018	2.50099502	2.73809954	2.44111915	2.53306347	2.34718336	2.90152193	2.62396673	2.71155405
NONHSAG030400	4.55958492	4.41293364	4.72057247	5.39568472	4.73088656	4.99883079	4.71316788	4.90140096	4.8835118
NONHSAG030406	4.83447639	4.91965731	4.94727549	4.5191467	5.07840276	4.7320034	4.75188698	4.77084127	4.96134937
NONHSAG030459	3.29428252	3.32068307	3.47331989	3.30913776	3.43673757	3.2203237	3.23193721	3.4178331	3.4574752

NONHSAG030469	5.35295827	5.14599927	5.34954916	5.07549138	5.57066595	5.34142958	5.10597059	5.24452072	5.3315173
NONHSAG030483	4.18058262	4.14725009	4.07384721	3.8383064	4.25306029	4.01452031	4.29782008	4.04712324	4.14592916
NONHSAG030489	3.5331263	3.41209593	3.4868454	3.47698716	3.64338537	3.32132581	3.61343966	3.39185568	3.38080478
NONHSAG030493	2.55516704	2.56984342	2.64529718	2.37354255	2.62973035	2.56213133	2.84425412	2.6468643	2.55220812
NONHSAG030495	3.70299679	3.65706438	3.81923988	3.52109215	3.73397109	3.60780919	3.9172587	3.74765136	3.58223563
NONHSAG030509	2.94560944	2.97529246	2.99505545	2.81901725	3.04117367	2.95894502	3.09252853	2.91203234	2.85255912
NONHSAG030613	2.38673402	2.650041	2.73197792	2.35806304	2.54105304	2.44664468	2.75383946	2.41050103	2.59595877
NONHSAG030629	4.02444483	3.62572223	3.61162801	3.67542617	3.57711401	3.39464291	3.60755026	3.6721935	3.81765878
NONHSAG030633	7.45746598	7.42157018	8.76738641	8.87583642	8.76602811	8.89122661	8.56075728	8.40471571	8.63462817
NONHSAG030634	5.18970173	4.90806859	5.76449995	5.93311268	5.84464623	5.92845394	5.72738908	5.83937614	5.76857347
NONHSAG030637	3.82865629	3.49482783	3.43549011	3.37690276	3.52217651	3.46937097	3.40488613	3.74812657	3.26427556
NONHSAG030676	2.48454722	2.46132623	2.36094217	2.46555415	2.73852723	2.38515884	2.42657209	2.47100793	2.55046776
NONHSAG030681	4.8045711	4.56062643	4.70361394	4.57702839	4.57586259	4.18620912	4.9788632	5.00097853	4.68079047
NONHSAG030683	3.80952135	3.92693972	3.87646661	4.07912968	3.95039443	3.53032668	4.23595341	4.17530156	4.18295501
NONHSAG030688	2.3571006	2.45672382	2.47445357	2.44111915	2.54046231	2.23552224	2.64186828	2.38089291	2.37917471
NONHSAG030757	6.55612583	6.7677499	6.74819977	6.48252504	6.8261925	6.46934178	7.11157056	6.43459159	6.64919338
NONHSAG030767	6.63025087	6.58292522	6.77530747	6.41791994	6.76823653	6.43145847	6.87898168	6.60289207	6.59483994
NONHSAG030783	4.19342723	4.72148512	4.64426631	4.56911204	4.45655427	4.56617912	4.46368794	4.58057188	4.68740959
NONHSAG030819	2.97332346	3.37533478	3.39403969	3.38399257	3.54129167	3.5787178	3.40439817	3.35023062	3.2001984
NONHSAG030850	2.01450921	2.37939971	2.20008754	2.4164978	2.13960656	2.17284296	2.39266822	2.3161388	2.20587921
NONHSAG030878	4.18280314	3.92489251	4.1243791	4.23377044	3.89289385	4.03806823	4.21976142	4.19921165	4.07146732
NONHSAG030888	5.39154663	5.15857465	5.41400454	5.23669956	5.22976982	4.97304745	5.6415027	5.37598375	5.23976509
NONHSAG030933	5.70088388	6.05465492	4.81853805	4.71288221	4.54201914	4.86851308	4.67761136	4.57377566	4.7190951
NONHSAG030937	3.9037663	4.15794131	4.14494476	4.02249291	3.97958052	3.90039486	4.19322148	4.02802609	4.17919118
NONHSAG030940	3.36158714	3.63557524	3.50581496	3.61578514	3.39146229	3.5124751	3.53085161	3.66434078	3.74714976
NONHSAG030957	3.90621569	3.93807151	4.02920457	3.90621732	4.10487578	3.76828443	4.27407695	3.99401378	4.0800522
NONHSAG030971	4.40766792	4.6263062	4.58330896	4.36313091	4.38870947	4.22854488	4.55123241	4.49348119	4.4661623
NONHSAG030992	5.61485299	5.70015795	5.65733387	5.54461154	5.6923875	5.51458342	5.65198528	5.56222512	5.67804096
NONHSAG031103	3.92514799	3.99049043	4.15686056	4.53897617	4.21068052	4.2075088	4.06530927	4.22366144	4.21244072
NONHSAG031137	3.74290948	3.28663813	3.733934	3.5667024	3.62302851	3.66582203	3.18465408	3.40524737	3.43287525
NONHSAG031167	4.59018147	4.67820648	4.14738562	4.6084582	4.28339213	4.30575731	4.1351647	4.34403236	4.24422494
NONHSAG031168	5.51054671	5.51463509	5.7054323	5.70592264	5.53945824	5.64462895	5.61093139	5.48564093	5.69080875
NONHSAG031178	3.17597996	3.15591804	3.51581552	3.15531216	3.1503704	3.00994586	3.42027349	3.23883735	3.1916714
NONHSAG031180	6.49917878	6.62684846	6.50149496	6.78617905	7.04805671	6.53019982	6.7136564	6.91530766	7.01872747
NONHSAG031184	3.24114689	3.02905456	3.14971284	3.10641193	3.13226482	3.02080214	3.28604795	3.15253995	3.04722799
NONHSAG031203	4.03228373	3.94830419	3.8942844	3.8643815	3.81346568	3.85433638	3.94686787	3.98836416	4.01124566
NONHSAG031219	2.96475722	2.92786643	2.99464464	3.19768695	2.83071581	2.8733169	2.99001776	2.83779356	2.89398167
NONHSAG031232	4.3150981	4.36137813	3.92623945	3.91533654	3.94572193	3.80605658	3.96919985	4.00007608	3.88025979
NONHSAG031237	3.03917084	2.90174893	2.94446276	2.67931078	3.18177363	2.82666568	3.48779913	2.93960133	3.01859017
NONHSAG031249	3.80422632	3.67201492	3.49681489	3.72195073	3.60957352	3.62716326	3.69549702	3.69846863	3.58106919
NONHSAG031255	2.12940508	2.14720753	2.30465263	2.46353812	2.32186048	2.11651083	2.2520632	2.07474191	2.16842325
NONHSAG031276	5.4812569	5.45123304	5.45561235	5.26533778	5.57351119	5.42069546	5.58582543	5.58344008	5.6575539
NONHSAG031296	2.7753301	2.63898116	2.70464013	2.34424148	2.78246741	2.47201078	2.83909071	2.66417932	2.47380407
NONHSAG031312	2.89428779	2.89841557	3.18803	2.97459355	2.86592257	2.85273634	2.8811615	2.86252409	2.67172029
NONHSAG031314	2.81780618	3.12110568	3.28178699	3.01106687	3.13594143	3.2089183	3.51982866	3.0988229	3.38649168
NONHSAG031315	7.24747221	7.5114932	7.21516044	7.37329716	7.43443541	7.25240974	7.37305011	7.74721076	7.41016158
NONHSAG031322	6.30500711	6.35304795	6.59724736	6.09621043	6.51044816	6.05147897	6.74334832	6.19355916	6.31349709
NONHSAG031335	3.61731163	3.50008888	3.47362457	3.54887903	3.73992944	3.42804246	3.66366964	3.50235951	3.80481645
NONHSAG031372	2.58285135	2.68862719	2.90536587	2.64562967	2.71596873	2.82579572	2.6188435	2.65758595	2.65037748
NONHSAG031377	5.40946208	5.491852	5.52371085	5.64525045	5.77699518	5.48652322	4.97380263	5.57594768	5.73801978
NONHSAG031385	4.98597978	5.38020772	4.95895469	4.95516827	5.46696231	5.20585309	5.33398251	5.3730922	5.32152356
NONHSAG031401	2.28994267	2.37674934	2.37690098	2.46690987	2.36694876	2.30407417	2.67568778	2.57411073	2.58557059
NONHSAG031461	3.1394524	3.22477215	3.16122362	3.16085321	3.10226279	2.86156806	3.10911929	2.95689231	3.06752089
NONHSAG031476	4.92586203	4.90119746	4.97290202	4.99306664	5.24933884	4.96999856	4.86936803	4.98398622	5.02362284
NONHSAG031485	4.90813376	4.86353967	5.03323307	4.8238839	4.97875132	4.41307662	5.21743268	4.74586815	4.92259074
NONHSAG031489	3.21744661	3.58427168	3.54270105	3.35499707	3.76456512	3.66720706	3.77692239	3.69020325	3.75585696
NONHSAG031516	6.31362662	6.08779424	6.3787856	5.86314329	6.27872615	5.75781703	6.57148458	6.21001902	6.23537916
NONHSAG031596	6.00150633	5.98212208	5.81902792	6.07389946	5.85506086	5.99340428	5.9400508	5.94025252	5.91920034
NONHSAG031603	3.11092819	3.02106458	3.26476577	3.24629691	3.230084	3.11500142	3.1539663	3.29995679	3.37120978
NONHSAG031617	5.90561291	5.76628083	6.13666145	6.1338805	5.90170436	5.80753453	5.9933039	5.8669602	5.80291565
NONHSAG031626	2.09442413	1.98870107	2.22174182	2.10461401	2.22384415	2.10673775	2.07763521	1.93288368	2.41158941
NONHSAG031665	2.80001163	2.73384535	2.99193763	2.83976495	2.92406235	2.69245291	2.95634896	2.98058568	2.94559776
NONHSAG031701	4.17989476	4.1309334	4.03443976	3.95258493	4.2486679	4.07727074	3.68355066	4.09874379	3.94963798
NONHSAG031748	4.37395351	4.64494979	4.56980019	4.12607116	4.53284341	4.1894101	4.80381906	4.48368394	4.48794989
NONHSAG031783	6.99511343	6.8857938	7.41689964	6.9046208	7.18614708	7.08413809	7.27489351	7.11673942	7.13129775
NONHSAG031784	4.61169055	4.97091157	4.89912794	4.65710497	4.62128664	4.47481493	4.764625	4.49918308	4.58577412
NONHSAG031801	4.32883539	4.1452328	3.8895292	3.77045451	4.1886209	3.81602647	4.19064926	3.8866011	3.79770652
NONHSAG031805	2.57993309	2.64424664	2.6122111	2.802033	2.52363577	2.7181822	2.6450664	2.58405448	2.75862155
NONHSAG031818	5.15781172	5.28552346	5.40557053	5.415473	5.17759526	4.94215274	5.49401665	5.26371414	5.25749569
NONHSAG031824	6.66295011	6.63535574	6.78983818	6.70187879	6.96363037	6.92278623	6.92397002	6.83222617	6.90809395
NONHSAG031831	4.15899665	4.21552513	4.38431403	4.22055578	4.44336119	4.30461544	4.4208748	4.35647213	4.44158571

NONHSAG031845	5.88557759	5.487268	5.46352912	5.04415369	5.33951175	4.71165722	5.28790327	5.10324677	5.40783422
NONHSAG031890	3.45425352	3.58203615	3.34277976	3.25402503	3.85437117	3.69620816	3.43454302	3.2860103	3.35652023
NONHSAG031913	5.2579041	5.24249421	5.37122778	5.14227988	5.2215617	5.14833688	5.34583588	5.05638932	5.33612539
NONHSAG031945	4.30060517	4.76930311	5.1710216	5.45614193	5.34427764	5.11507684	5.31901434	5.22603905	5.27810811
NONHSAG031951	4.49801073	4.26071629	4.66069149	4.20964388	4.38216486	3.86338664	4.43480883	4.11043384	4.36575962
NONHSAG031952	4.24859466	4.28023701	4.16932201	4.42106112	4.56211421	4.5539509	4.19862492	4.32357489	4.17101242
NONHSAG031964	4.13198847	4.3737011	4.39052205	4.26793082	4.48374091	4.32773943	4.34821852	4.59326815	4.34368661
NONHSAG032009	3.5683094	3.80195036	3.46108549	3.3078737	3.53680417	3.30041135	3.556616	3.49745555	3.53530091
NONHSAG032015	5.22783082	5.24885175	5.45052038	5.22858723	5.27917722	4.90572167	5.19403256	5.19079185	5.07910197
NONHSAG032104	5.15554202	5.32343061	5.26075705	5.04160799	5.20558174	5.28001175	5.21379176	5.23532917	5.16394426
NONHSAG032106	4.36701453	4.75516588	4.59377815	4.20046436	4.23678141	4.00415343	4.26091758	4.41852908	4.01479569
NONHSAG032135	5.41248715	5.11928582	5.53232047	5.52279292	5.5838375	5.79962138	5.26360628	5.63590332	5.27028663
NONHSAG032143	2.32110838	2.44910288	2.66328276	2.2962144	2.38039935	2.45689357	2.6431618	2.39160329	2.36023145
NONHSAG032153	4.28512957	4.40211635	4.62869951	4.14962564	4.40588617	4.20657559	4.66890481	4.18926548	4.48173148
NONHSAG032186	3.85940408	3.8251553	3.47122159	3.46451807	3.44716302	3.14526526	3.67575816	3.4858058	3.43572059
NONHSAG032194	2.56197472	2.8001723	2.51579461	2.51979361	2.5655366	2.44165479	2.71802412	2.40097227	2.35676616
NONHSAG032197	4.00242248	3.85496361	3.84252967	3.87754356	4.18727004	3.86985339	4.04433089	3.90416438	3.77704045
NONHSAG032235	4.42999538	4.78238842	4.61001362	4.67810119	4.56159005	4.55201754	4.91890174	4.64265169	5.03566836
NONHSAG032245	3.8032065	3.80181369	3.88077724	3.76911299	3.87434319	3.74612237	4.04673706	3.74851165	3.98023909
NONHSAG032250	3.7308017	4.03500939	4.21502993	3.78676958	4.26270789	4.23342322	3.84339682	4.01294467	4.07696166
NONHSAG032270	4.9351227	4.76662658	4.7449779	4.7171148	4.70438931	4.53150958	4.97853894	4.71934535	4.81622942
NONHSAG032285	3.68985093	3.81833167	4.0669526	3.8585022	3.93203559	3.68031964	3.69380409	4.01951113	3.91106807
NONHSAG032299	4.80869036	4.71609897	4.80016732	4.41559342	4.78152728	4.48888905	4.8077445	4.65316079	4.72600888
NONHSAG032337	8.00824474	7.58859149	7.65359395	7.97977483	8.39995815	7.94315271	8.34991249	8.1068846	7.94249452
NONHSAG032387	2.61757813	2.4828567	2.55769109	2.51827699	2.52760378	2.60300109	2.38500164	2.35761289	2.77538824
NONHSAG032422	4.6524653	4.92850051	5.01555613	5.02572366	4.91779949	5.02198597	4.85580503	5.68987689	5.16123382
NONHSAG032427	2.95430402	2.71844611	2.82518603	2.27538872	2.74801612	2.61160489	2.85680363	2.60830542	2.65894892
NONHSAG032431	5.06759575	5.14383482	4.93319896	5.19183772	5.1982785	5.3544622	5.3088407	5.09220408	5.01836292
NONHSAG032480	4.08906064	4.08793325	3.85622243	3.89629552	4.28572292	3.73877726	4.08861712	3.8883115	4.03225721
NONHSAG032559	3.01715995	2.96469994	3.06084756	2.92469915	2.98776037	3.18972731	3.07770311	2.92569555	3.17461342
NONHSAG032592	2.58234062	2.35886583	2.62722838	2.2947481	2.47025475	2.34121262	2.70643215	2.48761755	2.5128887
NONHSAG032593	4.44536527	4.5341366	4.46477234	4.23275244	4.56061178	4.32696678	4.51290979	4.35394114	4.32266793
NONHSAG032601	3.09542473	3.65463265	3.37443744	3.26532621	3.24015233	3.24843716	3.31990694	3.26389512	3.27472282
NONHSAG032608	6.29774201	6.57154442	6.83602373	6.24641203	6.69121831	6.19813494	6.77547192	6.39860294	6.48716084
NONHSAG032613	3.74768658	3.59020038	3.98286443	4.04565954	3.72569801	3.95796912	3.95456934	4.35669149	4.1543841
NONHSAG032620	3.2060024	2.68485565	2.70471413	2.78679158	2.76216609	2.81127954	2.50353022	2.54676988	2.76107013
NONHSAG032629	2.41425758	2.39953958	2.66820916	2.74582243	2.6634325	2.41414549	2.57052389	2.29070264	2.41146309
NONHSAG032632	3.80511501	3.76098277	3.75887385	3.54690986	3.86340193	3.71294738	3.75600455	3.50536588	3.62265079
NONHSAG032643	5.4054289	5.3908213	4.72122622	4.50718727	4.50438017	4.50856847	4.81966522	4.30229197	4.32974762
NONHSAG032649	3.02732956	3.20897283	3.25604076	3.31990332	2.894529	3.01585142	3.35598616	3.08311461	3.16058323
NONHSAG032662	2.56197472	2.89976033	2.89719082	2.90943307	3.03994884	2.81293468	2.96185861	2.95912313	3.03225436
NONHSAG032723	2.92835622	3.12541829	3.03928876	3.15236255	3.08197216	3.17074651	3.2995331	3.29576287	3.26797586
NONHSAG032740	4.46410584	4.32433894	4.44473276	4.21741736	4.35982244	3.97033898	4.54148145	4.23538647	4.39616223
NONHSAG032746	6.07573005	5.89670756	5.83487526	6.03076598	6.0106349	5.75320057	5.41706366	6.51545788	6.24903861
NONHSAG032750	3.39685495	3.2071626	3.23963481	3.21687775	3.48594365	3.18904047	3.08995874	2.97586142	3.3126834
NONHSAG032785	5.20995794	5.25546273	5.33964197	5.02809039	5.55502755	5.04666999	5.6903204	5.16663859	5.16973779
NONHSAG032791	3.65803456	3.67201492	3.71058211	3.59684002	3.56269762	3.75890483	3.88747591	4.05117344	3.85929126
NONHSAG032815	2.52068806	2.0534357	2.22823606	2.20786562	2.28569304	2.32630123	2.21302154	2.22492067	2.22679663
NONHSAG032817	3.52606751	3.38202393	3.48671025	3.6145005	3.1921804	3.26028156	3.29269694	3.45322919	3.39038939
NONHSAG032839	3.03911523	3.00481583	2.94390841	2.79650561	2.95524493	2.82213624	3.31143011	2.8079155	3.44591057
NONHSAG032855	4.83963067	4.70760241	4.94113377	4.50421734	4.86340312	4.42158261	4.98280668	4.577492	4.85832407
NONHSAG032935	4.61800059	4.23625445	7.33416506	7.57586696	7.198674	7.19540029	7.18721314	7.34817115	7.35662746
NONHSAG032949	4.75501483	4.53643866	4.80035005	4.46461798	4.72186829	4.53311736	4.93306457	4.33162624	4.75998085
NONHSAG032951	3.3972744	3.15634263	3.09734583	2.98827511	3.18470626	2.98387991	3.25754257	3.12127283	3.16320093
NONHSAG032956	6.96500421	6.8225583	6.07183479	5.94944668	6.5480429	6.0457059	5.85366785	6.69989373	5.75221848
NONHSAG032959	3.72952371	3.8171565	3.88412073	3.70118491	4.04567476	3.86719162	3.81854761	3.70157851	3.75537145
NONHSAG032960	3.7588005	3.9001414	4.11329907	4.00544409	4.14413451	3.97449849	3.90843031	4.05807406	4.27490862
NONHSAG032961	4.82617336	5.00199286	5.15141292	5.05555591	5.14340521	5.12258662	5.11974228	5.14425221	5.11640658
NONHSAG032979	5.08311736	5.39838898	5.21231204	5.35761876	5.33331795	5.42218765	5.41687856	5.21743268	5.25036644
NONHSAG032984	3.5435093	3.61242133	3.8763104	3.80165492	3.79826924	3.65980725	3.57099055	3.76606559	3.61585313
NONHSAG032988	4.39657851	4.20633043	4.10825747	3.91164589	4.39125402	3.91085242	4.60474565	4.20626966	4.07885509
NONHSAG033011	2.87717995	2.71986163	2.9261448	2.8443743	2.99193442	3.05171921	3.09647122	3.11020482	2.99347728
NONHSAG033026	4.92565684	4.84050151	4.948201	4.49457525	4.58296644	4.59720911	4.98536868	5.25367866	5.12954415
NONHSAG033060	3.70655718	3.63640048	3.84591108	3.6203943	3.8248742	3.34842613	3.95844981	3.58418618	3.6203943
NONHSAG033068	4.34936646	4.25934454	4.29303172	4.12687234	4.22128207	3.8767726	4.23745594	4.25907994	4.35111809
NONHSAG033071	6.45899013	6.18128346	6.31797361	6.2657536	6.3518125	6.11217039	6.50799102	6.20329718	6.31643955
NONHSAG033082	3.06014808	2.87972424	3.04646884	3.68031508	2.83455412	3.3390934	3.08132781	2.94374802	3.3418577
NONHSAG033092	3.74131324	3.67828758	3.72910083	3.45023369	3.48390711	3.26231791	3.82835397	3.50561218	3.59265772
NONHSAG033099	7.14227787	7.01512849	7.41513485	7.40182516	7.4184839	7.2400151	7.45750054	7.38465674	7.46684233
NONHSAG033167	4.02638256	4.02349339	4.0547773	4.20169938	4.28408003	4.19665306	3.79805111	4.34735722	4.21761215
NONHSAG033226	4.10189162	4.08793325	3.80127736	4.23577736	3.93769047	4.08578936	4.12469157	3.96514121	4.21564168

NONHSAG033228	4.0168537	4.07872971	3.95930516	3.69038053	4.03240642	4.29142437	4.3523913	4.17932661	4.22944226
NONHSAG033247	4.22354629	4.23253339	4.2865722	4.0452623	4.34010366	4.10076885	4.54259985	4.12531798	4.22456591
NONHSAG033254	4.68842981	4.69257615	4.88563518	4.93632034	4.88751432	4.72623734	5.20975555	4.57213566	5.12840534
NONHSAG033260	3.90447595	3.75548428	3.97680753	3.49862657	3.85232841	3.5760717	3.96221701	3.69667468	3.77677751
NONHSAG033274	2.46011979	2.57450556	3.15162583	2.61237282	2.75889952	2.93372087	2.56510836	2.54170506	2.57808798
NONHSAG033291	5.24681772	5.20856072	5.60888737	4.97997234	5.31478437	5.31972359	5.42493816	5.26707343	5.3552226
NONHSAG033405	4.25475416	4.45606237	4.23798096	4.36132353	4.55016985	4.47533896	4.31156198	4.30905887	4.1731828
NONHSAG033416	4.70189634	4.73858422	4.70049565	4.66830599	5.04076415	4.55445647	4.87988283	4.87691146	4.95680256
NONHSAG033484	5.25815319	4.68660612	5.55257127	5.58751463	5.20728643	5.43048273	4.89450433	5.30140735	5.31118727
NONHSAG033545	2.5998669	2.7147286	2.31865807	2.29689303	2.65563717	2.32724124	2.42654535	2.56179011	2.32950837
NONHSAG033550	4.75937065	4.37272926	4.41987264	4.26699158	4.52881462	4.08537348	4.54259985	4.39510709	4.37258123
NONHSAG033553	4.19392776	4.38653321	4.64117221	4.61150457	4.40488644	4.56896881	4.75541195	4.57130363	4.72427637
NONHSAG033558	2.35197987	1.97298617	2.24855028	2.09605539	2.26890366	2.29730361	2.56025929	1.95099845	2.2954068
NONHSAG033585	3.27846583	3.38331316	3.4679236	3.48893229	3.44087752	3.4691423	3.43113879	3.3002491	3.53014412
NONHSAG033587	4.84574166	4.92179978	5.00980954	5.06769035	5.14863981	4.91208604	5.30207426	4.93403655	5.1509737
NONHSAG033588	5.337021	5.33101522	5.39659286	5.30677982	5.44639585	5.44158096	5.40261071	5.4950291	5.46881037
NONHSAG033592	3.60227901	3.74555914	3.76817513	3.61103501	3.64135785	3.58081712	3.90361382	3.8247733	3.66521276
NONHSAG033593	3.16012258	2.98175942	3.34240153	3.18290523	3.1976036	2.97438941	2.90737963	2.93425086	3.34633164
NONHSAG033604	3.66981459	3.6679832	3.67643193	3.67590579	3.76216112	3.96765059	3.55235428	3.73185742	3.62046289
NONHSAG033616	5.35072504	5.5848531	5.71835987	5.49317007	5.61503297	5.29567752	5.67110233	5.44918956	5.48582779
NONHSAG033639	4.0140129	4.36553278	3.93178957	4.02749781	3.77915923	4.13004084	4.03922657	3.95937311	4.04835144
NONHSAG033752	3.86846505	3.89647784	3.91787509	3.79772808	4.14264104	3.98581868	4.08608273	3.95937847	3.96648857
NONHSAG033788	4.50708326	4.46074052	4.03372228	4.32245731	4.48283299	4.23642348	4.18066738	4.289301	4.11796228
NONHSAG033805	4.47313557	4.46573481	4.67944839	4.4370289	4.62370901	4.54415167	4.38995507	4.53037787	4.24724941
NONHSAG033897	4.92666177	4.9195507	4.9516107	4.91657647	5.10308511	5.18454278	5.01417916	4.88486206	5.02515522
NONHSAG033898	5.68371195	5.90529917	5.85301058	5.58495233	5.90235796	5.46626228	5.9300934	5.70418644	5.67724923
NONHSAG033967	4.86585056	4.89748042	4.99736269	4.84760344	5.11814793	4.64306811	5.19985688	5.0530437	5.00565007
NONHSAG033980	3.31737938	3.34041993	3.52029454	3.17740287	3.57656799	3.4031746	3.64724449	3.46706214	3.5887074
NONHSAG033989	5.54124834	5.68777733	5.43437263	5.38616357	5.66431713	5.78010888	5.64799127	5.45268537	5.6991675
NONHSAG034032	3.73995026	4.1202589	3.94230613	3.92708445	3.8110141	3.54844811	3.60085065	3.75313016	3.82470036
NONHSAG034050	5.33060735	5.60331582	5.29721695	4.91228439	4.92442532	5.20529808	5.31891883	5.17632107	5.22144972
NONHSAG034053	5.08677725	4.95367716	5.27828272	5.23011668	5.3035988	5.24371782	5.06836112	5.28598343	5.34599483
NONHSAG034077	5.68578544	5.3870441	5.73298265	5.59784893	5.76636277	5.63982921	5.69207964	5.72900106	5.59525037
NONHSAG034081	3.28981985	3.20712122	3.58898315	3.55015261	3.28004465	3.23630165	3.44144905	3.4274375	3.52466755
NONHSAG034105	3.16735683	3.27613255	3.49271433	3.17365818	3.24069612	3.13437089	3.07745173	3.20270861	3.34885719
NONHSAG034111	4.97130771	4.740068	4.90506948	4.70071182	4.85772567	4.57408637	5.02400883	4.71050471	4.89512419
NONHSAG034128	3.17843818	3.14396608	3.07848182	3.20402422	3.14970951	3.1443427	3.36149094	3.05298998	3.26517257
NONHSAG034158	4.0156445	4.08054985	4.29250458	3.89106008	4.39367297	3.99889785	4.00273236	4.12102318	3.98961406
NONHSAG034163	3.14409507	3.27692905	3.01394659	3.21956673	3.01394659	3.04987194	2.83678432	3.10367231	2.83576946
NONHSAG034175	5.76081637	5.71979466	5.62016526	5.95914988	5.89239701	5.72835939	5.8636906	5.69874827	5.86433997
NONHSAG034179	5.71020727	5.82938226	6.18501909	6.36261407	6.13073058	6.49822435	6.14721019	6.07804489	6.27484024
NONHSAG034184	4.56239384	4.3558437	4.13526107	3.82872834	4.28422502	3.93118222	4.44939998	4.34307076	4.13948422
NONHSAG034187	4.97318221	4.90715702	5.19103652	4.88138221	5.10235786	4.81869889	5.34183317	5.09304478	5.11350765
NONHSAG034193	4.14876212	4.26243978	4.3317459	4.21458964	4.44701228	4.37041834	4.6207786	4.44936554	4.61913435
NONHSAG034209	6.19115168	6.29169564	6.17500082	6.1993489	6.5240938	6.39500553	6.29068763	6.12588368	6.18836554
NONHSAG034242	5.42294891	5.55595241	5.21743268	5.30725211	5.62351531	5.2415599	5.62862393	5.53403872	5.32806756
NONHSAG034302	7.87850766	7.5435649	7.65167664	7.60798409	7.61678362	7.73733241	7.32517411	7.96242711	7.68062341
NONHSAG034336	2.86200007	2.94735998	2.79159779	2.93579564	2.92402498	2.86673492	3.06442817	2.80932803	2.83057727
NONHSAG034346	2.62604355	2.49802099	2.52754171	2.66186338	2.39683807	2.29689303	2.64648106	2.54934237	2.48570999
NONHSAG034364	3.11176199	3.19462693	3.16876723	3.01627081	3.24436179	3.00982034	3.57325754	3.08409474	3.24630917
NONHSAG034372	6.94616229	6.60490227	7.17339742	7.25315721	7.0271934	6.99352735	7.02064308	7.37269258	7.12964076
NONHSAG034414	4.23345065	4.51222187	4.95258313	4.16539494	4.62422527	4.79151205	4.3970549	4.28441551	4.86562884
NONHSAG034441	3.8841761	3.91377527	4.17921061	3.89622395	4.17443241	3.75531971	4.01433292	4.03313859	4.03786849
NONHSAG034453	3.96334896	4.04996506	4.34457536	4.01229623	4.27026435	4.05561017	4.34395085	4.29906754	4.14555944
NONHSAG034459	5.13524279	5.15120468	5.4716431	5.02966929	5.1948591	4.91958581	5.148625	4.73133449	5.00588716
NONHSAG034470	3.76802873	3.72735178	3.58270552	3.61968567	3.95371435	3.56644598	3.77465534	3.50160378	3.42047786
NONHSAG034501	5.52100055	5.39918594	5.61356268	5.02163564	5.28999556	5.14914291	5.39890462	5.25130993	5.30766583
NONHSAG034507	2.33952939	2.61826957	2.53295964	2.37217835	2.37832956	2.17416338	2.53913891	2.27019116	2.60554869
NONHSAG034552	4.55004467	4.20817193	4.4451341	4.36094798	4.23375897	4.08575668	4.25781908	4.41414396	4.32909941
NONHSAG034566	5.06156316	5.1345291	5.54801072	5.09994639	5.45196118	4.90245416	5.55311239	5.09172787	5.26287447
NONHSAG034569	3.17421868	2.94118655	2.87826232	2.49433681	3.01847269	2.92598924	3.05590439	3.15098894	2.75791567
NONHSAG034584	2.71835109	2.92573947	2.76474821	3.13045535	2.93032466	2.6983098	2.78369968	3.26302211	2.89377455
NONHSAG034600	3.34086722	3.2298787	3.45152603	3.46514938	3.49264855	3.46372418	3.74190763	3.14798887	3.54316152
NONHSAG034618	4.34439141	4.43934931	4.12529714	3.88177153	4.20869227	3.77888128	4.36265675	4.42016541	4.10350671
NONHSAG034646	2.60325598	2.37347732	2.23946941	2.01133276	2.30570533	2.20626679	2.26046584	2.30120205	2.23350683
NONHSAG034655	2.41536004	2.20616645	2.42378173	2.23216366	2.2206065	2.21038512	2.39619714	2.31714593	2.22584897
NONHSAG034677	3.0087926	3.04976329	3.1225425	2.96325504	3.06302836	2.98726711	3.13343467	2.7973191	3.17220645
NONHSAG034678	2.86195686	2.43070969	2.5841298	2.64885493	2.57612255	2.37178245	2.63115052	2.58283246	2.33976969
NONHSAG034689	4.34111121	4.30692855	4.40703821	4.53120223	4.4656959	4.61909001	4.49128263	4.34243149	4.50407244
NONHSAG034701	3.67745394	3.74080981	3.70922331	3.82728358	3.8535514	3.9903884	3.87688838	3.68307785	3.87537292
NONHSAG034702	4.36395778	4.53880232	4.01156345	4.28012847	4.23147221	4.35756237	4.51381194	4.37063141	4.38803282

NONHSAG034714	4.40959334	5.09961426	4.40677527	4.40396285	4.68631862	4.19317995	4.8316189	4.48316248	4.54801033
NONHSAG034778	2.66894473	2.71663563	3.11075955	3.02226119	2.84274686	2.9849486	2.81907007	2.84754502	2.87651035
NONHSAG034831	6.31478729	6.5259846	6.74846453	7.25387674	6.63901155	7.07089858	6.86460526	6.90101961	6.92938722
NONHSAG034858	3.08913877	3.21379712	3.52438848	3.1208166	3.37534665	3.11563204	3.00806373	3.28433053	3.61357187
NONHSAG034900	2.86161404	2.86900247	3.09881283	2.72355783	2.80707542	2.79822109	3.01730126	2.82193684	2.67106678
NONHSAG034902	2.99314286	2.97547485	3.38025603	2.86103886	3.32242421	3.13371599	3.17309665	2.93024283	3.01789845
NONHSAG034909	5.1291281	5.00567876	5.14910823	4.82045332	4.81158905	4.81052226	5.01724219	4.85044733	4.94240712
NONHSAG034919	3.68024127	3.63730872	3.63382342	4.0549829	3.3054856	3.50166098	3.46030704	3.27063938	3.4575879
NONHSAG034946	3.99508998	3.77620464	4.04843438	3.60430165	3.9098471	3.8251674	4.441049	3.74649655	3.91815753
NONHSAG034982	4.56212702	4.58175522	4.71288237	4.48929027	4.64540777	4.64319647	4.65817932	4.61277192	4.53142056
NONHSAG035114	3.90273251	4.13297364	4.06075739	3.64916381	3.95006337	3.89156495	4.07802081	4.08685346	3.97771869
NONHSAG035116	5.37507032	5.40684212	5.39072739	5.35971069	5.35698037	5.18743708	5.29305994	5.54845893	5.45727082
NONHSAG035135	2.86245045	2.89716154	2.91995674	2.92506894	3.07509425	2.91184705	3.05046035	3.00658103	2.95605381
NONHSAG035143	6.53689211	6.75845298	6.74513527	6.72870525	6.87210899	6.76313417	6.91706239	6.48450195	6.613821
NONHSAG035159	5.81728937	5.34422376	5.16340345	5.44440892	5.68183956	5.31060504	5.54460797	5.26712686	5.57175058
NONHSAG035266	2.28653568	2.29165361	2.27202166	2.35245473	2.27531836	2.53288693	2.03716064	2.33161726	2.29147339
NONHSAG035270	4.65184333	4.42042429	4.30493684	3.98525945	4.32577905	4.32104932	4.39002195	4.33027029	4.22800273
NONHSAG035294	4.02351763	3.87680105	4.13960643	3.52194218	3.98995773	3.75609224	4.02680433	3.99269565	3.99797288
NONHSAG035333	3.8840857	3.91033131	4.08929361	3.93159467	3.989271	3.88917391	4.23745594	3.9911386	3.84573295
NONHSAG035347	4.39084177	4.51267798	4.68921426	4.50025102	4.72688994	4.36197939	4.90636625	4.6329413	4.5634349
NONHSAG035395	2.38437075	2.59853535	2.60293921	2.69932381	2.54870694	2.61676248	2.55220342	3.12748209	3.09441393
NONHSAG035415	4.83039518	4.68963058	4.56970391	4.33783539	5.00509212	4.47387908	5.04207292	4.56830192	4.90693094
NONHSAG035432	4.83523141	4.78527576	5.00998546	4.83030763	4.95359228	4.61067724	5.23427635	4.89255608	4.87187465
NONHSAG035436	2.22110165	2.59666654	2.62962399	2.62932887	2.80747727	2.6265111	2.88311522	2.48744915	2.82852378
NONHSAG035491	3.84308911	3.50810976	3.9433446	3.3814176	3.6037896	3.18058891	3.8693436	3.28747349	3.74771147
NONHSAG035503	2.2099821	2.14012611	2.08089322	2.19044284	2.20732507	2.19508492	2.19044284	2.17461725	2.11535576
NONHSAG035505	3.51334229	3.2948274	3.57609697	3.10070345	3.63846479	3.36248954	3.41212174	3.5323296	3.73784557
NONHSAG035508	2.22678714	2.26842501	2.12897388	2.30700513	2.28527112	2.27031116	2.19284985	2.16872453	2.31522101
NONHSAG035519	4.49119927	4.5708711	4.28152648	4.73244919	4.31665903	4.17168077	4.43351076	4.53638558	4.3573141
NONHSAG035522	6.03062856	5.97447368	5.761056	6.03959277	5.95127913	6.09826964	5.56747321	6.07107077	5.96922615
NONHSAG035547	4.31948539	4.35224068	4.50624536	4.83022452	4.46219569	4.31529339	4.26919085	4.23478588	3.84333955
NONHSAG035579	2.39682151	2.3268077	2.39071001	2.45778729	2.35506804	2.35406045	2.54367002	2.44381172	2.3358849
NONHSAG035588	5.22155954	5.15496226	5.38433763	4.69669239	4.42455905	4.63836207	4.61798057	5.23742384	5.19060221
NONHSAG035621	3.94539988	3.80583635	3.47689836	3.42847965	3.73286856	3.70769371	3.90450683	3.71003343	3.86827142
NONHSAG035667	2.97332346	3.33388475	3.16981496	3.16981496	3.2616402	2.93130806	3.60533122	3.14234807	3.23445126
NONHSAG035675	3.27380863	3.05410589	3.31294863	3.27734396	3.31007406	3.18851715	3.25593987	3.62117378	3.30981984
NONHSAG035700	2.4904403	2.83576946	2.62722838	2.4294093	2.65371517	2.51202524	2.71038661	2.53120388	2.82699514
NONHSAG035708	2.28468161	2.43932365	2.51228733	2.4536639	2.45331266	2.66634351	2.73345702	2.56854505	2.3089352
NONHSAG035712	2.71391189	2.79074365	2.81274557	2.46541308	2.80217435	2.40474356	2.80811869	2.57675506	2.52813838
NONHSAG035715	2.32498055	2.4645304	2.18389147	2.24100961	2.15761674	2.28532517	2.14514237	2.17364442	2.30987811
NONHSAG035722	4.33862247	4.35868285	4.1457082	4.42660162	4.55848143	3.86602955	4.5046568	4.24349599	4.283574
NONHSAG035731	2.29433397	2.45007513	2.17523526	2.27931044	2.28622351	2.28223745	2.11479594	2.26413723	2.13230493
NONHSAG035768	6.21021796	6.08039828	5.95182461	6.30337528	5.79433906	6.30897003	5.87657067	5.97102185	6.03791777
NONHSAG035770	6.6860821	6.47399482	5.87448187	6.6358636	6.34467428	6.22082927	5.93228713	6.75711905	6.76244427
NONHSAG035786	2.68678438	2.73656172	3.12102253	3.17648636	2.95105205	3.07011197	2.95857559	2.97211667	3.3938455
NONHSAG035791	3.06993157	3.05366184	3.29106303	3.26532401	3.3313951	2.97229718	2.86937409	3.32288476	3.26868865
NONHSAG035807	2.61901851	2.70500019	2.59869879	2.29788802	2.53943317	2.31805908	3.0055479	2.66280816	2.87679482
NONHSAG035911	10.16101032	10.27156131	9.96254355	10.02260871	10.10125099	9.92897101	10.04627028	10.09913038	10.00795769
NONHSAG035915	2.5320738	2.43571924	2.66172513	2.36221818	2.50641955	2.25120735	2.50063514	2.3946306	2.39038077
NONHSAG035927	2.98300379	2.80096156	2.84876363	2.72219876	2.81240411	2.58107596	2.9818939	2.86503189	2.75573972
NONHSAG035942	2.96206833	2.44193561	2.72098106	2.87520093	2.66041783	2.718272	2.66854103	3.0375007	2.53295467
NONHSAG035943	5.78486675	5.68963112	5.66108166	5.48826764	5.66777921	5.44670715	5.37349429	5.44739455	5.0415537
NONHSAG035971	4.99795244	4.84336489	4.36987372	4.73751674	5.24933884	5.36512606	5.18943974	4.96368319	4.76929568
NONHSAG035976	5.39982127	5.4687921	5.62803358	5.24696883	5.61250293	5.25439733	5.63106808	5.50749064	5.68348469
NONHSAG035982	5.2254391	5.3955637	5.16793572	5.54554965	5.39498648	5.18927076	5.37258922	5.16373764	5.393132
NONHSAG036030	2.89165237	3.07181254	3.30027496	3.33308959	3.34448141	3.31294377	3.15302945	3.23191857	3.32842689
NONHSAG036052	5.63525274	5.36569834	5.90671735	6.16576662	5.9537377	5.82609003	5.8585743	6.07294412	5.9646188
NONHSAG036065	2.30265566	2.21398011	2.39777963	2.32737189	2.41924029	2.39857931	2.40530904	2.6636268	2.44149733
NONHSAG036067	8.41644988	8.36649786	8.21512862	8.60958777	8.12543218	8.44122194	8.06014813	8.22146226	8.26443114
NONHSAG036098	4.89333937	4.85155844	5.14939904	5.27122405	4.82361671	5.46322719	4.43674938	4.96163483	4.88474109
NONHSAG036130	4.3237356	4.35016555	3.00878711	3.59466095	3.39493213	3.51083294	3.66893503	4.00330171	3.8765446
NONHSAG036141	4.97469639	5.16255633	5.4194161	5.65727517	5.62903458	5.61910688	5.17899155	5.637635	5.51198229
NONHSAG036146	5.5561085	5.62965855	5.57427736	5.56094813	5.47589154	5.41055122	5.57953988	5.24044736	5.3623026
NONHSAG036147	4.49897967	4.50474938	4.69569152	4.30796643	4.62455909	4.28545213	4.55462834	4.53980257	4.42778386
NONHSAG036184	3.12072497	3.3792076	3.48535185	3.02432022	3.23829569	3.20194636	3.20304766	3.10750987	3.3733545
NONHSAG036217	4.36672595	4.42854478	4.21260941	4.58569185	4.46555963	4.43261582	4.42544898	4.54167596	4.44373286
NONHSAG036227	7.10086133	6.8694513	7.57366299	8.38281287	7.94049012	7.96822323	7.88967845	8.32834485	7.92078824
NONHSAG036242	3.18842759	3.33765716	3.22311148	3.07000608	3.22342803	3.22522748	3.23091277	3.16257613	3.26517257
NONHSAG036289	3.02561754	3.23001574	3.25674291	3.10338461	3.32228023	2.93139441	3.21080042	3.13269949	3.03049458
NONHSAG036290	2.3286274	2.24808813	2.31556093	2.39360351	2.56689501	2.44921768	2.42083633	2.44741211	2.67546011
NONHSAG036298	4.38644498	3.87742407	3.43698818	3.4516602	3.44257993	3.64675076	3.16802079	3.58418618	3.53560377

NONHSAG036307	2.87802092	2.91593407	2.95421667	2.87862466	2.93940487	3.02266971	2.70412073	2.69046294	2.95703201
NONHSAG036327	3.35503447	3.16936097	3.30987344	2.61518824	3.25312711	2.78745638	3.25990972	3.06325992	3.25681909
NONHSAG036374	3.38215024	3.42505997	3.66660468	3.3997958	3.67987533	3.08855395	3.61573602	3.33723122	3.10124781
NONHSAG036383	6.21829728	5.98677885	6.49305975	6.13181819	6.3286946	6.321478	6.34439914	6.19461778	6.35727438
NONHSAG036413	2.3354888	2.07826642	2.23941173	2.27081218	2.39153403	2.19261669	2.47379828	2.26078627	2.22528622
NONHSAG036444	2.82082242	2.56663017	3.02497405	2.90005751	2.72181479	2.68241038	2.94010032	2.68379817	2.64530014
NONHSAG036455	2.73636062	2.69518883	2.72952618	2.64942697	2.63329068	2.59831804	2.69836331	2.42316354	2.62214884
NONHSAG036462	3.93648971	3.96098738	3.98759792	3.51128805	3.60207611	3.32619253	3.78501238	3.55953135	3.64783822
NONHSAG036486	2.88677371	2.8522851	2.27663474	2.28768498	2.67075628	2.82941386	2.59076904	2.49916036	2.57413703
NONHSAG036538	3.27193307	3.24190409	3.32434887	3.49289142	3.33891912	3.38432468	3.44252988	3.35666468	3.17373854
NONHSAG036539	5.89662281	5.77378114	6.31845788	6.70525258	6.31581436	6.66832523	6.34021001	6.57101897	6.40646065
NONHSAG036566	2.83614214	2.94644184	3.15259035	2.86077588	2.99799648	2.93774006	3.2337821	2.68137497	2.92343358
NONHSAG036587	3.36094575	3.34636056	3.50979483	4.1101205	3.4610432	3.95196997	3.53968951	3.49386411	3.84322226
NONHSAG036631	5.51146441	5.2158183	5.24611743	5.53659641	5.74113907	5.43410958	5.47691469	5.94767536	5.63377055
NONHSAG036645	2.26272935	2.17574983	2.25847465	2.39527264	2.21551101	2.23616122	2.40897909	2.17905697	2.24120058
NONHSAG036667	2.89662563	2.66905168	2.47934921	2.51129154	2.83374136	2.56562717	2.60393069	2.63496035	2.67291991
NONHSAG036669	3.8809334	3.66238841	3.80962116	3.63737288	3.58397126	3.58712663	3.70491975	3.38459358	3.63226282
NONHSAG036696	2.74779245	2.41376334	2.41345922	2.5758425	2.44230321	2.43292739	2.67937248	2.6854197	2.87974319
NONHSAG036703	2.21972471	2.21427742	2.2297354	2.09228325	2.33350543	2.21204891	2.21245283	2.34183305	2.05235165
NONHSAG036710	2.679542	2.9335946	2.85709805	2.78257082	2.79997194	2.90042085	2.77664085	2.81333233	2.96547091
NONHSAG036741	2.59738534	2.10840751	2.08792929	1.98549438	1.98156964	1.99442531	2.42805658	2.42542928	2.09977798
NONHSAG036745	4.14953829	3.82462657	4.25388929	3.88260969	4.08150564	3.64075152	4.04731771	3.98458789	3.83168065
NONHSAG036767	2.61871355	2.87864448	2.85734102	2.74430066	2.72541285	2.64626955	2.60827848	2.74348881	2.72727656
NONHSAG036776	3.55939087	3.27294329	3.61332577	3.38564271	3.47257534	3.23976726	3.9415634	3.46357228	3.73600194
NONHSAG036803	3.39698917	3.0767083	3.07781185	2.78284471	3.13550914	2.87769996	3.21301778	3.07362875	3.20201018
NONHSAG036808	4.19575702	4.24637145	4.09381794	4.53946985	4.07343176	4.52406093	4.05985538	4.26111538	4.19304383
NONHSAG036869	4.46410584	4.47571519	4.87988283	4.65719141	4.48386904	4.77493562	4.76208761	4.47397357	4.53737025
NONHSAG036874	3.28593753	3.1629754	3.30184582	3.18225204	3.13459159	3.19458595	3.20151953	3.03126439	3.16075798
NONHSAG036892	2.90604931	2.74320552	2.84993813	2.70663007	2.92734251	2.59820017	2.64129917	2.80399693	2.81134783
NONHSAG036926	6.67268553	6.47280346	6.74934676	6.41021131	6.66956518	6.33978298	6.80133781	6.4232303	6.606555
NONHSAG036955	3.1599111	3.04034851	3.26949033	3.14125729	3.27167465	3.46017489	3.28604795	3.08677237	3.35652023
NONHSAG037015	3.592583	3.42829911	3.42412583	3.17662226	3.41180481	3.15853507	3.60015868	3.45989463	3.3005181
NONHSAG037017	4.46410584	4.61504815	4.3026253	4.65422862	4.51267798	4.50036122	4.38206594	4.69877781	4.47947802
NONHSAG037020	3.21525508	3.48328918	3.67243924	3.28102718	3.48594365	3.18368636	3.64237542	3.40410708	3.4384708
NONHSAG037021	2.64152806	2.36379036	2.33398604	2.42307028	2.42332886	2.32852661	2.40195022	2.45174557	2.59727778
NONHSAG037022	6.9494857	6.91452497	7.39492218	7.10573935	7.13853308	6.62839231	7.21075753	7.0577612	7.05506999
NONHSAG037025	2.70227525	2.69966332	2.79257598	2.98704395	2.59166085	2.36578482	2.66782479	2.71335822	2.73964808
NONHSAG037030	6.27776487	6.22007965	5.73820413	6.09477813	6.23075091	5.91750162	5.63996143	6.55239472	6.04914784
NONHSAG037031	5.41167168	5.53400003	5.66768648	5.5051817	5.57992225	5.37518842	5.57156319	5.4249464	5.58062541
NONHSAG037032	3.4301284	3.33471489	3.36451235	3.28386463	3.36058841	3.39947384	3.33256219	3.29674936	3.18093474
NONHSAG037083	3.67891129	3.37292264	3.56651163	3.37180123	3.33271046	3.46823738	3.83271249	3.71692456	3.43886977
NONHSAG037087	7.36614241	7.34616247	7.64050957	7.60444376	7.71601527	7.42890872	7.55944477	7.45929887	7.51488399
NONHSAG037150	4.43098772	4.86190692	4.68499029	4.71162751	4.51670682	4.63213944	4.77022113	4.51962986	4.55347738
NONHSAG037173	6.98185854	6.87264141	7.51029587	7.87686891	7.41444473	7.64342231	7.22010627	7.7013678	7.67332796
NONHSAG037197	5.89885006	6.29013769	4.2173154	4.57067087	3.89377855	4.48830716	4.19097217	3.80265792	3.5495853
NONHSAG037200	5.58535142	5.33781454	6.00191284	5.62884615	5.78223875	5.9091017	5.94519589	5.73338881	5.69652589
NONHSAG037224	6.44459474	6.33919207	6.80980839	6.90705619	7.01426182	6.74438187	6.70424836	6.95225474	7.02582533
NONHSAG037227	5.43727364	5.53151843	5.50497266	5.35286285	5.69428532	5.53003489	5.71577282	5.59540423	5.50856336
NONHSAG037229	5.16671102	5.25273256	5.49438313	5.2332309	5.41796196	5.26461004	5.46792831	5.39508188	5.30010867
NONHSAG037251	2.61496502	2.59701222	2.75982242	2.52096729	2.56158356	2.59570462	2.74178741	2.56931494	2.61462577
NONHSAG037262	6.06134748	6.15086466	6.15853596	6.03494635	6.27519783	6.11410009	6.40070689	6.20973736	6.14634547
NONHSAG037272	4.50858532	4.72954719	4.47646372	4.50737381	4.43361845	4.60727325	4.60280716	4.84735497	4.75723792
NONHSAG037287	4.42193441	4.15541808	4.64294959	4.91538963	4.81248956	4.97267079	4.33733531	4.97444158	4.68330391
NONHSAG037322	2.7310673	2.45007513	2.42318443	2.44861404	2.38934064	2.45585574	2.35918042	2.41524755	2.58147806
NONHSAG037336	3.62979941	3.77517647	3.56479684	3.72913877	3.3625936	3.50754752	3.51083161	4.01889651	3.524939
NONHSAG037338	5.07724571	5.21523401	5.55990151	5.23738031	5.475103	4.85669026	5.38628771	5.00196293	4.9696637
NONHSAG037352	5.25318565	5.16308204	5.50347688	5.59120965	5.61002909	5.39972234	5.60089668	5.5457277	5.5700379
NONHSAG037375	3.46803011	3.09980797	3.26337856	3.0757673	3.30111987	2.82274565	2.88712128	3.02269521	3.12029421
NONHSAG037376	3.64100452	3.59775963	3.59164661	3.64768321	3.60460385	3.41243875	3.30061866	3.67418799	3.63993595
NONHSAG037395	6.92613556	6.78917747	7.20927146	7.30732297	7.1279072	7.32715049	7.00723752	7.26400041	7.29948924
NONHSAG037396	5.63366384	5.46877912	6.03969203	5.73847513	5.85217639	5.76558502	5.86170675	5.85353693	5.92094648
NONHSAG037407	4.52902091	4.68728714	4.70318605	4.90544756	4.89405456	4.80032866	4.73977267	4.80578523	4.90894546
NONHSAG037426	4.40836761	4.38770202	4.62025432	4.89008252	4.73630224	4.8169965	5.0988279	4.56956235	4.73000315
NONHSAG037431	4.73108783	4.51803388	5.14059801	4.89396041	5.14965768	4.55438566	4.69801462	4.73304699	4.60074829
NONHSAG037432	4.60434047	4.29605775	6.47361884	6.67174014	5.95000147	6.30970068	6.29706549	5.97071825	6.34819196
NONHSAG037433	3.4267644	3.6970079	4.88778462	4.58814143	4.63808347	4.69103024	4.45454905	4.46706677	4.74399007
NONHSAG037464	3.84620642	4.12522439	3.98372633	3.97767489	3.94652061	3.90205164	4.14584907	3.98912255	3.70219982
NONHSAG037470	3.48803521	3.56392237	3.75459818	3.90610856	3.58157315	3.64675076	3.58742703	3.64668344	4.04134471
NONHSAG037504	3.2216037	3.41017353	3.33989355	3.24468318	3.40676934	3.34986137	3.42346114	3.25957013	3.21872829
NONHSAG037513	3.147063	3.14701129	3.1481168	3.29135612	3.26666139	3.11490152	3.32348618	3.27546708	3.35792424
NONHSAG037525	3.80623359	3.97179464	3.78334156	3.95053317	4.39683979	4.00269156	3.01534248	3.73286358	4.07947661

NONHSAG037563	4.35339536	4.19469767	4.83407783	4.6524936	4.79956411	5.0298223	4.66374423	4.72577701	4.68877919
NONHSAG037570	3.55596785	3.52272	3.62628057	3.42537992	3.92911184	3.56301892	3.62409649	3.69883932	3.57955854
NONHSAG037575	5.04076415	5.41625848	5.39230594	5.23781958	5.55151747	5.16409331	5.85217192	5.40131738	5.48762806
NONHSAG037577	3.90877932	3.66072567	3.85780653	3.56701741	3.73286117	3.79439195	3.7269072	3.88240655	3.60689893
NONHSAG037622	3.4203571	3.38152862	3.5432388	3.63295866	3.42072082	3.40323947	3.19150235	3.07003565	3.80085772
NONHSAG037629	4.40600302	4.54643029	4.5514496	5.02603117	3.99706735	5.04721205	4.24575644	4.11810077	4.52777918
NONHSAG037701	3.45693519	3.18348333	3.37573788	3.20450598	3.49801584	3.34799339	3.66272115	3.21125919	3.45662358
NONHSAG037719	2.75561227	2.59893299	2.90135714	2.8443743	2.94117739	2.77175649	3.03423634	2.63884855	3.04840372
NONHSAG037724	2.11967723	2.09142269	2.2880228	2.26773081	2.29323421	2.12158165	2.50572786	2.23327609	2.37468573
NONHSAG037726	2.40477793	2.32997245	2.31531961	2.32501357	2.28995959	2.27454209	2.20032609	2.31234503	2.20243697
NONHSAG037747	3.88791201	3.89307924	4.12003039	3.66610871	4.07468478	3.5673248	4.06770733	3.77657576	4.01012688
NONHSAG037751	4.48647054	4.20861417	4.33347456	3.99181429	4.1476396	4.18388633	4.39512718	4.06189727	4.2349446
NONHSAG037786	7.45970014	7.2405312	7.94609001	8.13170603	7.88684357	8.17230651	8.08440755	8.05917657	8.13480969
NONHSAG037791	2.64985485	2.40867574	2.58692869	2.60407609	2.65350056	2.35320434	2.52725037	2.4292586	2.44619447
NONHSAG037804	3.45437056	3.50485485	3.45269954	4.43893242	4.04770167	3.79955322	3.88590074	4.14667173	3.87706765
NONHSAG037839	5.18380538	5.1577442	5.54235856	5.65356152	5.57834423	5.74559625	5.44802529	5.31117404	5.50409141
NONHSAG037842	3.42423353	3.36721212	3.67837941	2.97903642	3.49350341	3.34523802	3.56680868	3.3169924	3.34695852
NONHSAG037849	3.08975902	3.25389113	3.28531983	3.17529021	3.32595427	3.29764847	3.18971347	3.21944235	3.32595427
NONHSAG037860	3.62924106	3.39136666	3.80684425	3.3173684	3.42653197	3.71637421	3.48662502	3.29758064	3.51660755
NONHSAG037870	2.27034973	2.36476864	2.29759598	2.26971887	2.34019908	2.2609509	2.30347749	2.28699093	2.32409716
NONHSAG037873	2.42452739	2.45335906	2.63206079	2.16461461	2.32022109	2.42079672	2.30710952	2.41017062	2.37271487
NONHSAG037878	2.37539394	2.36307412	2.41986658	2.37467956	2.41944529	2.30351545	2.55781027	2.33693296	2.44710476
NONHSAG037943	2.95448784	3.07271858	2.90893184	3.07863514	3.00998247	2.93698948	2.93940547	2.95844978	2.91415213
NONHSAG037955	2.30910722	2.23903685	2.16341674	2.01250271	2.21202989	2.1171036	2.19980333	2.20099332	2.22805304
NONHSAG037959	3.24361794	3.05569223	3.09550536	3.07439476	2.97765269	3.26821719	3.3407563	3.20598323	3.43414222
NONHSAG037962	4.13384362	4.24977722	4.15981205	3.88256791	4.27459915	3.93166426	4.30973833	4.13820105	4.0275757
NONHSAG037977	6.5801775	6.44460936	6.39758832	6.82088026	6.61984963	6.63559537	5.99560297	7.25112273	7.09392158
NONHSAG037987	2.52289944	2.65172055	2.58467885	2.55188037	2.29942789	2.39486178	2.71038661	2.48020334	2.48851685
NONHSAG037991	3.77731734	4.00214367	3.99856458	3.74287489	4.00339059	3.88346991	4.23745594	3.66878082	3.8287004
NONHSAG038027	3.69637961	3.88677871	3.78452942	3.47449208	3.60951941	3.58081712	4.12375787	3.7064235	3.68070091
NONHSAG038035	2.12081261	2.23859391	2.21508432	2.31047099	2.14488926	2.2079877	2.23862682	2.35057957	2.14099485
NONHSAG038048	2.58697023	2.5635101	2.54717923	2.56500386	2.28829386	2.40562601	2.63438383	2.39178564	2.40714637
NONHSAG038071	3.90514834	4.11621448	4.25345779	4.08333089	4.10820788	3.87535018	4.24562773	4.0904478	3.90400811
NONHSAG038091	3.2398534	3.42697293	3.21450336	3.36694175	3.41636622	3.53821429	3.71709852	3.46847174	3.44612099
NONHSAG038132	5.0069702	4.87482486	4.87988283	4.80396404	4.98859034	5.04252917	4.50814382	4.76336996	4.64375426
NONHSAG038179	3.48900409	3.69990133	3.73857844	3.38831012	3.84626464	3.20303613	3.68221154	3.36933352	3.59145772
NONHSAG038210	4.4206112	4.57249528	4.31871338	3.90722414	4.25589854	3.7049312	4.33540278	3.92506172	4.05261038
NONHSAG038213	2.85693671	2.83391851	2.89574393	2.87992336	2.830453	2.41957567	2.90392937	2.81768134	2.82897264
NONHSAG038259	3.22966732	3.20856219	3.88539944	3.37857478	3.54733694	3.15503548	3.52050592	3.57840269	3.42248973
NONHSAG038285	3.54710919	3.51103548	3.83877046	3.4891144	3.85642133	3.57816899	3.68452582	3.65330702	3.54568502
NONHSAG038292	2.30475425	2.40709836	2.49520177	2.3159167	2.41450945	2.43854146	2.61681485	2.32086175	2.5130166
NONHSAG038333	5.49908983	5.5360481	5.69742725	5.65727517	5.64320468	5.73826677	5.8296919	5.67330399	5.63145973
NONHSAG038350	3.45648148	3.29032983	3.6572441	2.99785042	3.35794191	2.90390564	3.48844687	3.28699664	3.22836353
NONHSAG038364	3.42988375	3.43640493	3.52513753	3.38236775	3.43667027	3.11062817	3.51546624	3.55542321	3.31023092
NONHSAG038417	5.98801171	5.74289415	6.73644771	6.38606691	6.18433299	6.72917216	6.58431256	6.99302076	7.32481419
NONHSAG038444	2.97332346	2.92116214	2.75845771	2.62672579	2.75456397	2.76519536	2.73758852	2.76903923	2.67075628
NONHSAG038460	10.09178865	10.02839029	9.94178658	10.00382521	10.1154863	10.03418065	9.98456823	10.02500602	10.03249458
NONHSAG038476	3.64725689	3.71174063	3.66474305	3.87785846	3.7154726	4.00391793	4.00869095	3.4158489	3.70776828
NONHSAG038511	2.41088443	2.32699904	2.27663474	2.36692038	2.57551952	2.51132634	2.5652758	2.24390536	2.25239778
NONHSAG038512	4.03462064	4.15284488	4.22244216	4.13532128	4.25762439	4.21679273	4.22676279	4.96254613	4.57497169
NONHSAG038522	5.53503076	5.40293156	5.65278211	5.45501505	5.60414732	5.68502754	6.03803092	5.5666174	5.38629676
NONHSAG038586	5.67163642	5.68185672	5.63565513	5.67314759	5.46256461	5.71654175	5.49081408	5.50703723	5.53967131
NONHSAG038591	2.62214928	2.51243972	2.72255962	2.59482556	2.57452191	2.53524884	2.61476499	2.55671353	2.44975248
NONHSAG038599	3.60224743	3.22027605	3.4149128	2.9952165	3.27744844	2.99479143	3.11167085	2.89742805	3.26517257
NONHSAG038627	6.78752136	6.90239631	6.02763525	6.7513278	6.42072924	6.81785524	6.17671206	6.53144822	6.30689356
NONHSAG038649	3.59340599	3.60514259	3.659662	3.90297162	3.69838341	3.93034672	3.73593592	3.75677516	3.75601322
NONHSAG038724	2.75671637	2.93226216	2.82984261	2.43225314	2.63695201	2.45109959	2.80043324	2.70310922	2.65096336
NONHSAG038765	4.15046177	4.22924265	4.34100754	3.88165381	4.31651238	4.26705716	4.35943935	4.16561755	3.89377235
NONHSAG038779	3.38414787	3.68678011	3.2141733	3.73199394	3.50910221	3.37986556	3.18342321	3.70513472	3.27531558
NONHSAG038780	2.17423331	2.07764414	2.30825212	2.17326221	2.28056991	2.17214833	2.31077137	2.06656077	2.13776744
NONHSAG038818	2.36963758	2.19386644	2.25361802	2.24921301	2.363189	2.09064104	2.29975375	2.20897362	2.11922477
NONHSAG038845	6.21625767	6.14163676	5.19284201	5.94952742	5.35704771	5.73656745	5.34658719	5.6477148	5.35580242
NONHSAG038869	2.40459017	2.24995975	2.23228087	2.29689303	2.24195315	2.05710718	2.42945564	2.13419199	2.42514243
NONHSAG038874	2.68724678	2.23703174	2.56646092	2.64064568	2.54460126	2.61305699	2.59893299	2.51150856	2.44619447
NONHSAG038882	2.75897535	2.76929797	2.3620392	2.2663472	2.54004451	2.35320434	2.78248932	2.31675012	2.16613233
NONHSAG038890	5.46045548	5.12160956	6.19826721	6.30986465	6.11414787	6.10847705	5.95637883	6.58024396	6.32669294
NONHSAG038899	2.53414987	2.83576946	2.86948082	2.63820087	2.68879187	2.62194583	2.69971564	2.57122513	2.74232354
NONHSAG038901	4.03077872	4.16291531	3.82478252	3.67062549	4.35641211	3.80444371	4.39385401	3.7525291	4.01403888
NONHSAG038916	2.19074045	2.5894466	2.40095547	2.3942882	2.44813063	2.46540555	2.33284031	2.65317867	2.30455344
NONHSAG038917	2.45512066	2.27289024	2.34490104	2.46444322	2.25020225	2.40932342	2.41438309	2.49907469	2.22805304
NONHSAG038920	3.94720443	3.84039247	3.69181798	3.80766475	3.9854332	3.81586527	3.96003603	3.66947597	4.08393171

NONHSAG038949	4.81136788	4.77977753	4.81772418	5.05269023	4.97835389	4.87416933	4.85831525	5.23158696	5.17725393
NONHSAG038955	2.55650979	2.59893299	2.79149227	2.64106406	2.93436683	2.67063452	3.08195427	2.92491358	2.77680748
NONHSAG038958	2.78860238	2.70982085	2.68864602	2.61148836	2.81175636	2.88401585	2.72855759	2.86829951	2.67075628
NONHSAG038962	3.53106236	3.56181387	3.70337608	3.45150075	3.41135344	3.38128455	3.60833895	3.47109252	3.6152848
NONHSAG038974	4.06197521	3.85738455	4.14722411	3.87554848	4.39154016	4.14518805	4.31748274	4.15910076	4.10914892
NONHSAG038975	3.75091873	3.56358521	3.62513884	3.30358222	3.53263968	3.64274384	3.86046821	3.61725223	3.66242937
NONHSAG038999	2.42730104	2.35728724	2.34982139	2.42243119	2.22577565	2.46882596	2.38509427	2.19502357	2.49309895
NONHSAG039007	2.03629216	2.17585067	1.92806971	2.2022665	2.45661544	2.10015509	2.37457713	2.44458803	2.14320391
NONHSAG039009	2.49899483	2.37614478	2.48379021	2.29689303	2.62398201	2.28292796	2.34073252	2.27537721	2.26825489
NONHSAG039017	2.93562096	3.05689213	3.13242395	3.18080107	3.00080625	3.18972731	3.31895268	3.07324716	3.26100282
NONHSAG039019	3.1668519	3.04392754	3.34417905	3.02975469	3.02653295	2.92570369	2.87627232	2.75885925	3.05077534
NONHSAG039034	4.03864607	3.43058439	3.68064756	3.10394566	3.60366711	3.43489794	3.93748866	3.14415111	3.3937877
NONHSAG039040	2.69637182	2.71587448	2.78900806	3.07958216	2.96579128	2.88909221	2.88880164	2.67406769	2.74403942
NONHSAG039046	3.3238999	3.40981754	3.40130996	3.26915324	3.29115841	3.29332317	3.48600499	3.30935806	3.39044798
NONHSAG039047	2.90438455	2.95268095	2.69598312	2.87706084	2.94040678	3.08709677	2.81704522	3.06495012	2.91560538
NONHSAG039060	3.11748127	3.00418301	3.2420979	2.91524529	3.000325	3.0350597	3.07354794	3.10669001	3.09559245
NONHSAG039061	3.07229106	2.74178288	2.96842003	2.69304884	2.97207754	3.11993034	2.97631763	2.77505145	2.88214671
NONHSAG039063	3.15181373	2.94960239	3.22121001	3.11510342	3.06419281	3.08382456	3.1715414	2.89095849	3.02865721
NONHSAG039082	4.25805306	4.28849009	4.23464919	4.43406671	4.51164568	3.98254928	4.56484612	4.17462659	4.22800273
NONHSAG039102	3.51781355	3.48809789	3.5664599	3.39873064	3.59392182	3.39237785	3.60613937	3.4714498	3.58174826
NONHSAG039105	4.49660724	4.72009714	4.88794854	5.10380018	4.89643586	4.95362956	4.85477932	4.89348361	4.76868325
NONHSAG039107	5.6356353	5.71726784	5.87887881	5.59309131	5.9346436	5.83489645	5.89415947	5.76523629	5.80260095
NONHSAG039111	3.06308071	2.83930815	3.08843185	2.95698377	3.26581571	2.70934144	3.04697559	3.12471994	2.85985568
NONHSAG039112	4.77676589	4.75515383	4.9292099	4.26509776	4.81960637	4.32283012	4.83359409	4.64227143	4.82923932
NONHSAG039130	3.11399176	3.08380365	3.29221557	3.16769815	3.36097511	3.35022101	3.43034585	3.3146818	3.19438486
NONHSAG039135	5.07914485	4.58143288	4.81685765	4.56600727	4.68336839	4.56403937	5.04116047	4.60171681	4.61509764
NONHSAG039189	2.17748704	2.17016523	2.21721916	2.10215881	2.13655985	2.22485552	2.21302154	2.18531942	2.15968523
NONHSAG039190	3.27401497	3.6321581	3.45046958	3.30006923	3.6835159	3.45951726	3.71612138	3.74495648	3.53530091
NONHSAG039191	2.60930608	2.50100436	2.68251356	2.40897831	2.5528505	2.63713938	2.42434116	2.75061194	2.54090001
NONHSAG039225	3.5115123	3.28844413	3.64818957	3.74838081	3.67892118	3.55234652	3.73363059	3.74260505	4.02935321
NONHSAG039294	2.8274891	2.66262139	2.86631381	2.68029347	2.68867954	2.70089134	2.66171151	2.82975873	2.86147315
NONHSAG039315	4.35579554	4.17595779	4.38860545	4.25152361	4.43988471	4.35204218	4.49672739	4.35594795	4.42212032
NONHSAG039321	2.74195449	2.95579275	2.89249103	2.59595877	2.7249921	2.61659381	2.72742929	2.73362879	2.66913436
NONHSAG039327	3.64277588	4.02910241	3.85351031	3.67398342	3.86852108	4.23556238	3.61896601	3.69673777	3.77154779
NONHSAG039332	2.83154659	2.76135368	2.82810856	2.80769546	2.75231382	2.7514725	2.91649761	2.77995271	2.72968883
NONHSAG039337	2.60842837	2.46054269	2.70003056	2.46121141	2.66940153	3.04533053	3.01983203	2.59373616	3.34427723
NONHSAG039356	2.79686861	2.79068593	2.83393163	2.61793192	2.78120711	2.52590247	2.7770172	2.63916297	2.81001142
NONHSAG039358	4.06658681	4.37048905	4.37054827	4.32071857	4.35755845	4.49069939	4.45723188	4.2430397	4.30182718
NONHSAG039367	3.31719087	3.00322553	3.04082342	3.42776306	2.99410872	3.39992082	3.30299684	3.64963212	3.49323543
NONHSAG039377	4.05731694	4.14155391	4.0426243	4.08559007	3.92438071	3.74262287	4.17857562	3.98008818	3.93047124
NONHSAG039399	5.4829947	5.36131074	5.71758604	5.59285705	5.24498058	5.52597773	5.63256424	5.59549765	5.62158113
NONHSAG039414	2.97288457	2.90842026	3.01885598	2.68765325	2.90088609	2.57080998	2.96364216	2.91780949	3.01660584
NONHSAG039470	4.86135211	4.61651525	4.65827728	4.42672624	4.47268549	4.50199018	4.81768727	4.41944155	4.90852188
NONHSAG039489	2.81203943	2.85665965	2.74682579	2.87135612	2.8443743	2.70715685	3.18561548	2.79690812	2.96945144
NONHSAG039490	3.90644744	4.40494749	4.37718761	4.62307658	4.5086823	4.65241328	4.21892006	4.64959075	4.17197012
NONHSAG039502	4.43633594	4.39400359	4.34903043	4.18040596	4.75673728	4.2470371	4.5608449	4.5967564	4.12212106
NONHSAG039503	5.3808699	5.60128066	5.28903687	5.29633785	5.6271866	5.27014083	5.65298993	5.31645462	5.24837772
NONHSAG039511	3.81664856	3.41502862	4.05642997	3.47243734	3.76082734	3.51258878	3.57829849	3.84240931	3.65203406
NONHSAG039515	2.99627195	3.27727283	3.09178679	3.2914996	3.21721101	3.17415102	3.1715414	3.3456493	3.41751882
NONHSAG039551	5.01488177	4.83147624	5.21708157	4.99020818	5.035788	4.9244995	4.94951163	5.07372943	5.08939504
NONHSAG039586	2.18395021	2.10135867	2.51205643	2.33949894	2.5744581	2.28551289	2.09555942	2.35327802	2.21262461
NONHSAG039670	5.02643904	4.84365394	5.14490577	4.68850482	5.06627663	4.7411679	4.75894396	5.06057629	4.86936817
NONHSAG039676	8.9358184	8.72459026	8.98372261	9.04623371	8.92663295	9.01203005	8.75520536	8.9329592	8.90865374
NONHSAG039681	3.05786498	2.91560538	2.8977894	3.04087452	2.78246741	2.83530205	2.73640787	2.9122025	2.94834596
NONHSAG039701	5.08746312	4.97195766	5.07887676	4.4602902	4.86369157	4.6050307	5.09275789	4.69747509	4.86397044
NONHSAG039715	2.82343265	2.84124737	3.02520195	2.77659844	3.09547111	2.65460626	3.22010909	3.04833777	3.13881223
NONHSAG039740	2.58696094	2.47713164	2.84846564	2.40984166	2.70515993	2.58115693	2.82075462	2.48978413	2.58068182
NONHSAG039770	2.80287342	2.76546766	2.91439866	2.6805319	2.52205005	2.63253861	2.58567902	2.76102674	2.73220896
NONHSAG039813	2.80001163	2.91560538	2.97605952	2.61936923	3.10698311	2.88377858	2.86349824	3.00137085	3.08390826
NONHSAG039828	2.92515847	2.81588029	2.81998721	2.84017987	3.1753158	3.11273599	3.16489179	2.98806843	3.08636593
NONHSAG039846	2.6817902	2.51155949	2.69566556	2.81351762	2.69390069	2.6544493	2.69568564	2.7550685	2.56557252
NONHSAG039857	3.83508171	3.71566362	3.94794966	3.81246105	4.08579624	3.57931115	4.17725853	3.89919803	4.24517034
NONHSAG039863	6.93549645	6.44086352	6.33110113	6.6223974	6.70987158	6.42188673	6.16257326	6.79338005	6.58297771
NONHSAG039875	6.32698258	6.39473023	6.41716252	6.75938703	6.75355795	6.61127759	6.236205	6.79334571	6.59291962
NONHSAG039889	3.68179505	3.75717541	3.69473338	3.58141536	3.70710462	3.58458813	3.9864214	3.5706643	3.71894501
NONHSAG039949	2.85380489	2.71491858	2.8977894	3.02686751	3.19675854	2.58900728	3.11017444	2.96858714	2.8518596
NONHSAG039968	2.28347156	2.14182476	2.10056086	2.11183785	2.48848464	2.38909358	2.20433165	2.15266367	2.57131127
NONHSAG039971	3.2019985	3.14618699	3.18582469	3.28511126	3.33400543	3.22111317	3.23687166	3.33027526	3.29355302
NONHSAG039976	2.47745679	2.56889881	2.80634281	2.35631589	2.5442386	2.42839263	2.6950832	2.42519035	2.49381125
NONHSAG039991	4.33593735	4.01022679	4.76187084	3.99967927	4.33502224	4.26695873	4.58449356	4.2877679	4.3476128
NONHSAG040004	2.29013423	2.76134461	2.6034719	2.29366269	2.69387286	2.4490985	2.59719425	2.55156194	2.44747349

NONHSAG040070	3.15745816	3.04138523	3.13512876	2.82798581	3.10965259	2.87885016	3.27555664	3.08151026	3.03969978
NONHSAG040081	2.35886895	2.28623702	2.44715954	2.33154524	2.20883941	2.35372228	2.34112147	2.89118773	2.6811451
NONHSAG040093	2.48454722	2.28701436	2.37607105	2.52800362	2.32142628	2.26114183	2.59893299	2.24405328	2.35721198
NONHSAG040100	2.68017413	2.88205302	2.89672298	2.56096965	3.04399254	2.59582566	2.93056848	2.56004118	2.79517245
NONHSAG040122	5.7316329	5.66079278	5.70720573	5.53789565	5.76932064	5.84683089	5.57861722	5.89655858	5.73883475
NONHSAG040157	5.0343881	5.36753547	4.6659874	4.65370805	4.61386037	4.61277453	4.85546358	4.57862097	4.70620852
NONHSAG040159	4.71243754	4.60841179	4.84351545	4.74545865	4.59005783	4.83216533	4.69150125	4.77398663	5.03725418
NONHSAG040185	7.44140759	7.45952711	7.51012715	7.66882981	7.66482115	7.66935563	7.56532506	7.59454654	7.60158862
NONHSAG040189	3.10481585	3.00806159	3.09196805	2.58330593	2.82500715	3.02513726	2.89062567	2.86294543	2.7519613
NONHSAG040206	5.86185813	5.9477544	4.81286587	5.51493149	5.04829352	5.22421524	4.90944179	5.26031524	4.8899215
NONHSAG040218	2.4529712	2.26870301	2.61828013	2.47993375	2.34422827	2.55501848	2.35722792	2.43090848	2.4553899
NONHSAG040274	2.07526299	2.33003183	2.47059325	2.06798852	2.18759314	2.23964089	2.21302154	2.09605154	2.31579297
NONHSAG040276	2.55979274	2.45530989	2.48708705	2.43393888	2.61826634	2.70471413	2.57260788	2.60050627	2.29916015
NONHSAG040302	5.23821219	5.1998184	5.36554098	5.31769461	5.51076604	5.36224383	5.24925609	5.70912377	5.37782733
NONHSAG040333	7.03609141	7.02530318	7.50664527	8.09390636	7.61386807	7.83802566	7.55668734	7.54190411	7.71777111
NONHSAG040375	3.03025521	3.35877085	3.19612765	2.95329874	2.98874995	3.05141962	3.04697559	3.18426256	2.93508955
NONHSAG040378	3.86449743	3.9070538	3.76919993	3.75153863	3.95121958	3.99706735	4.13279071	3.86715318	4.08810212
NONHSAG040385	3.77843409	3.6947978	3.80873438	3.73420612	3.6416898	3.86153118	3.65480214	3.68315786	3.58714094
NONHSAG040395	2.95470825	3.02485344	3.02927861	2.88181553	3.03659712	2.98167606	3.11320915	3.04355674	3.00805015
NONHSAG040459	2.64018904	2.64935495	2.64413581	2.52499419	2.49248174	2.56987403	2.50353022	2.73014072	2.69147797
NONHSAG040465	3.27553059	2.9972641	3.02152823	3.14882265	3.08131366	2.93695736	3.2036746	3.14850081	3.19108725
NONHSAG040492	2.91949175	2.92096602	2.79946614	2.92975337	2.89258528	2.84946734	3.4676639	2.95767009	3.01433542
NONHSAG040535	4.78011698	4.62740091	4.9450194	4.66220126	4.69640199	5.03362245	4.95708557	5.0921701	5.26462635
NONHSAG040538	3.25394643	3.19370633	3.25498668	3.17644921	3.21287207	3.05625806	3.31884636	3.19381296	3.22111025
NONHSAG040545	3.60594284	3.86128323	3.77064475	3.4493764	3.74499029	3.79320624	3.94417712	3.74240045	3.59954222
NONHSAG040546	4.24988719	4.15849458	4.26118468	3.85706639	4.29003935	3.75480025	4.41973598	4.05589297	3.83664282
NONHSAG040559	4.6805184	4.72005677	4.50383935	4.53302866	4.44008649	4.46269077	4.71437059	4.47021082	4.75076829
NONHSAG040605	5.93001396	5.68378402	6.10172225	5.91274702	6.09798053	5.85276117	5.93579218	5.87419419	6.03663207
NONHSAG040664	8.95870194	8.85128382	8.67053806	8.73909208	8.66047076	8.83552885	8.53909193	8.61659012	8.54242523
NONHSAG040668	4.37475832	4.44643575	4.55272179	4.63386628	4.59711732	4.58308002	4.49989084	4.60410591	4.39285888
NONHSAG040671	2.73637371	2.70728568	2.68087963	2.58172674	2.70264358	2.38562252	3.00402098	2.65155582	2.61984429
NONHSAG040680	3.51778488	3.36752562	3.4115212	3.68922361	3.16523731	3.50443214	3.09901228	3.67353375	3.76112106
NONHSAG040684	3.24451396	3.35533591	3.18939584	3.53518808	3.34839593	3.21839337	3.52030662	3.58454577	3.34525669
NONHSAG040703	4.27864159	4.30641387	5.13253524	5.120485	4.57727384	4.92641514	4.93115836	4.73072983	4.86035506
NONHSAG040714	3.66171026	3.79898726	3.97025142	3.91523167	4.13088251	3.83582455	4.06819041	3.89629552	3.75173515
NONHSAG040766	3.78643472	3.87888306	3.73351714	3.71688199	3.85796397	3.6617211	3.62172939	3.62583445	3.70299045
NONHSAG040771	3.01244058	2.75337948	3.03026432	2.98035163	3.00843968	2.98483095	2.99466109	3.00631481	3.08852413
NONHSAG040781	6.36866097	6.36095746	6.49036461	6.81737856	6.37401608	6.65010177	6.19246739	6.53044056	6.38578018
NONHSAG040790	4.30644404	4.17075555	4.24271325	4.3923245	4.43895361	3.99371303	3.91949894	4.18075844	4.17841064
NONHSAG040792	3.803793	3.79799541	3.79572444	3.65798784	3.81935445	3.45737852	3.67076924	4.02420639	3.83171846
NONHSAG040794	4.76579793	4.57405685	5.06049283	4.74979598	5.00400948	5.08809297	4.89262723	4.99465679	5.55069462
NONHSAG040797	3.49293056	3.86605332	3.65890538	3.56401335	3.50324702	3.45786077	3.71718459	3.58388698	3.70174909
NONHSAG040826	5.4190522	5.45526596	5.72329839	6.58693802	6.23359127	5.92332825	5.57075877	6.75691288	6.53497316
NONHSAG040838	3.39850369	3.32327997	3.53422608	3.25212539	3.15040191	3.16839123	3.37332068	3.10098578	3.41340131
NONHSAG040876	4.30140044	4.42797768	4.73892828	5.18507721	4.7764179	5.06070816	4.88063415	4.83834532	4.71487475
NONHSAG040878	4.19825808	3.63447373	4.20433964	4.7174855	5.07159153	4.9243272	4.46259317	5.34141169	4.9524264
NONHSAG040919	2.07897788	2.09499229	2.08997807	2.0465483	1.94663667	1.99810781	2.08242932	2.25260867	2.10909334
NONHSAG040944	3.20198424	2.7695388	2.98554314	2.27163898	2.89329817	2.54028899	3.00511625	2.7094637	2.65112531
NONHSAG040959	2.89974941	2.88176978	2.95076863	2.81970701	2.93182503	2.88647684	3.20316248	3.13793813	3.26742555
NONHSAG040973	4.1805366	4.30697176	4.21985276	4.21219469	4.26945932	4.26220008	4.39281474	4.28394646	4.26318571
NONHSAG040975	3.3424988	3.06428765	3.27649022	3.06369153	3.1381991	3.20297759	3.1970182	3.12051684	3.23096869
NONHSAG040987	4.35851963	3.89791432	3.86192267	3.85938785	4.0102306	3.68136612	3.84982634	4.20729512	4.00904925
NONHSAG040990	2.15124039	2.25770653	2.44705467	2.22701442	2.28514881	2.422567	2.59893299	2.14072018	2.28827235
NONHSAG040992	2.80001163	2.79237559	2.9652324	3.14481789	3.04285192	2.59256406	2.72158636	2.68061206	2.59608349
NONHSAG041024	2.39482017	2.54080778	2.46709333	2.53683833	2.30858346	2.27827034	2.20045379	2.20240983	2.32724212
NONHSAG041029	2.26256249	2.20082604	2.31046526	2.54131964	2.34286396	2.27310223	2.42717328	2.59699425	2.25607266
NONHSAG041052	5.3517498	5.38780797	5.09511512	5.48529768	5.48542198	5.17164746	4.8473629	5.77026145	5.34400551
NONHSAG041087	3.36855034	3.42781678	3.53924949	3.37499663	3.40057823	3.31424516	3.77674288	3.60357752	3.39159669
NONHSAG041094	9.01916659	8.95768664	9.17978556	9.41876228	9.28814106	9.1403619	8.96887339	9.2343466	9.26235692
NONHSAG041153	2.52058558	2.6805319	2.6216794	2.58798848	2.76431873	2.86348866	2.75479438	2.73774087	2.80101974
NONHSAG041157	3.21839441	3.03842636	2.92678679	3.35904452	3.18940312	3.20546312	3.09277413	3.68126415	3.47111763
NONHSAG041202	2.79569819	2.95600485	2.76200509	2.81798487	2.77999146	2.87412691	2.94741579	2.95248838	2.91094632
NONHSAG041205	3.96201467	4.13832661	4.07170814	4.44632543	4.0679556	4.06119241	4.18791747	4.11218413	4.14372264
NONHSAG041207	4.42931502	4.38342485	4.64432958	4.2035299	4.57075124	4.07134092	4.39989842	4.47582105	4.33873867
NONHSAG041271	2.8944939	3.03676663	3.28660868	3.11636387	3.15743679	2.94544821	3.35364076	3.1862976	3.0371482
NONHSAG041280	6.29360531	6.19046885	6.58679184	6.98342989	6.49905798	6.78147707	6.41870693	6.81585145	6.73917353
NONHSAG041308	2.74905355	2.3184483	2.40065862	2.30601437	2.38934064	2.40794494	2.38343332	2.28420013	2.43962364
NONHSAG041310	4.58909838	4.59328492	4.76799535	4.5110766	4.80543536	4.57843113	4.6376648	4.69796836	4.62174479
NONHSAG041311	2.55911947	2.21378923	2.82707218	2.36606776	2.29520068	2.37274629	2.71038661	2.56188444	2.60718977
NONHSAG041331	8.53356805	8.43570932	8.08624485	8.39163684	8.17669931	8.33354188	8.16642601	8.37169444	8.250669
NONHSAG041341	2.86533666	2.95968294	2.94127299	2.92758718	2.84487492	2.90717808	3.02449491	2.87818853	3.01425323

NONHSAG041345	2.743076	2.55385505	2.59885461	2.47116522	2.61630634	2.96262509	2.61449394	2.44889721	2.34491185
NONHSAG041359	2.64613472	2.67263308	3.05937689	2.5305105	2.84101068	2.74274443	2.7267453	2.64864436	2.94297688
NONHSAG041393	2.12081261	2.11494772	2.11327768	2.45791159	2.13735144	2.22132792	2.21302154	2.21722534	2.22805304
NONHSAG041442	3.22026917	2.76050892	3.25602735	3.0251742	3.08701107	2.90284746	2.93869404	2.79218533	2.97218627
NONHSAG041473	6.31755263	6.2059458	6.29531108	6.26595566	6.18941995	6.67521818	5.99405739	6.64586521	6.2065541
NONHSAG041474	5.45765976	5.40520487	5.45049449	5.47059049	5.31478437	5.395709	5.28050295	5.48162662	5.6492337
NONHSAG041475	3.47545042	3.78824128	3.63127255	3.6870988	3.77000033	3.59604069	3.54331989	3.84856985	3.65827299
NONHSAG041496	2.69346457	2.59357704	3.00623782	2.73918926	2.74773606	2.59820277	3.28412949	2.72564873	2.78681894
NONHSAG041512	4.90652134	4.28599296	4.60619905	4.59265858	4.42868022	4.50200899	4.78829036	4.5967988	4.68907342
NONHSAG041520	3.05269551	3.08170559	3.14241079	3.27501065	3.39304329	3.42232469	3.23893907	3.30895503	3.00077009
NONHSAG041523	4.92019915	5.01714585	5.46086209	5.11166334	5.09621257	5.04279878	4.88814786	5.10222055	5.11838557
NONHSAG041551	5.25806425	5.17183253	5.73676693	5.43337076	5.13175413	5.12537718	5.45892123	5.3280565	5.46199326
NONHSAG041552	4.07252769	4.1186367	4.20076647	4.2800092	4.07423404	3.99697892	4.08010418	4.0197463	4.14448558
NONHSAG041579	5.63874728	5.5758139	5.59743033	5.48378384	5.7959701	5.44357481	5.6965987	5.69097592	5.6561367
NONHSAG041596	10.9414802	10.91217165	10.87606177	10.92858672	11.07708123	10.83766225	11.14886	11.03941446	10.84553877
NONHSAG041601	4.39167595	4.1664392	4.25870255	3.95906639	4.11585477	4.14336422	4.33395437	4.19478154	4.23903077
NONHSAG041618	4.53672805	4.94747041	4.63019427	4.4688254	4.48183387	4.54077887	4.6439268	4.51935102	4.63772789
NONHSAG041677	4.63906646	4.26546774	4.63393149	4.37915616	4.75406022	4.19673896	4.86633734	4.52818279	4.50307465
NONHSAG041723	3.89263192	3.69334016	3.92811315	4.44569149	4.26303453	3.77392225	4.24927224	4.27805857	4.29642995
NONHSAG041789	3.85933255	3.99191284	4.14663596	4.0791491	4.20927463	3.88625523	4.12145164	4.23750451	4.0771463
NONHSAG041804	2.35258054	2.23242419	2.19155109	2.3558136	2.21783204	2.24071148	2.30347336	2.22898625	2.34178479
NONHSAG041810	3.12367905	3.25644764	3.26430104	3.10540633	3.18697111	3.12205089	3.2622718	3.09673236	3.41790649
NONHSAG041826	2.11301255	2.16628691	2.0176806	2.08569078	2.07531108	2.10411625	2.15349277	2.15455318	2.10706659
NONHSAG041886	3.00976201	3.22885137	3.17533769	2.83351375	2.84030811	2.40529711	3.10151715	2.94330958	3.219893
NONHSAG041903	3.91698174	3.9481673	4.12094859	3.98062856	3.98479339	3.99685317	3.9572715	3.87666825	3.87716598
NONHSAG041984	5.07757293	5.0430191	5.08556492	5.24539109	5.22454676	4.74632339	4.7472032	5.22018472	5.34638992
NONHSAG041987	3.38343834	3.2667349	3.31658848	3.28121335	3.22007168	3.40807924	3.63888673	3.31789193	3.62214025
NONHSAG041994	2.33154425	2.6396913	2.73878568	2.4131157	2.5056062	2.39325589	2.41903231	2.67164328	2.49467932
NONHSAG041998	2.84070318	2.65793494	2.78952814	2.47548043	2.62671804	2.66354249	2.75178452	2.52859639	2.80260769
NONHSAG041999	3.22468063	3.16484006	3.27620871	2.98385367	3.42725402	3.35263041	3.33655626	3.43673817	3.08332643
NONHSAG042046	2.62878102	2.56121962	2.56564995	2.50336383	2.615939	2.55134205	2.62481756	2.58717195	2.54084993
NONHSAG042051	5.01860149	5.09408273	5.03478961	5.10915205	5.0578453	4.96932156	5.09991739	4.83679175	4.87788655
NONHSAG042059	3.3529807	3.15705629	3.1945765	3.1899454	3.42118628	2.99001758	3.28451093	3.26517257	3.29399731
NONHSAG042103	3.48194702	3.46705724	3.57662746	3.26820011	3.36499271	3.24032729	3.3017755	3.18664757	3.38526068
NONHSAG042145	3.25866661	3.21091221	3.1217137	3.31654364	3.25316069	3.1760391	3.40200451	3.12278381	3.18968579
NONHSAG042163	3.56068767	3.78561554	3.81143899	3.87797979	3.89133255	3.73257548	3.57715438	3.55276575	3.80407868
NONHSAG042168	2.4209212	2.4055758	2.51620026	2.65306606	2.38141603	2.3963448	2.59893299	2.47921438	2.59595877
NONHSAG042176	10.09465023	9.96802808	10.78323953	10.86880478	10.89134589	10.6492001	10.74829528	10.82976613	10.56414998
NONHSAG042182	4.21727895	4.41447401	4.17135189	3.93575087	4.05624421	3.92409773	4.13526856	4.02306828	4.29262948
NONHSAG042192	3.15252818	3.18576715	3.29260021	3.10200707	3.24544638	2.99738323	3.2732603	3.19381328	3.18118632
NONHSAG042210	3.09480404	3.11991649	2.8047651	2.78108094	3.19511263	2.73440177	2.88472765	3.3456493	2.94842894
NONHSAG042250	3.56677947	3.58260919	3.55094615	3.37324608	3.60304386	3.37161856	3.61410974	3.47609736	3.45722069
NONHSAG042261	3.88851092	3.91993034	3.35568914	3.44684006	3.28604795	3.2873617	3.4939989	3.50064011	3.3296045
NONHSAG042272	4.07736536	4.21033239	4.28433143	4.0423722	4.22877734	4.37347717	4.2287378	4.2188692	4.08457623
NONHSAG042283	3.85803254	4.04444188	3.81974675	4.01809874	4.01664211	4.06408611	4.03470106	4.16087792	3.89155332
NONHSAG042315	4.71449192	4.94293409	5.17984345	4.76524	5.2420781	4.87878167	5.40881776	4.87287467	4.99043782
NONHSAG042323	4.16378386	4.36854355	4.35986316	3.90766821	4.61639613	3.97802183	4.40867864	4.10483383	4.34917175
NONHSAG042369	4.21598958	4.22554056	4.89451923	4.1177096	4.75686395	4.23397396	4.86151428	4.55677844	4.37492767
NONHSAG042381	2.71741599	2.60133409	2.554309	3.009523	2.75233894	2.90225	2.48787048	2.96821203	2.6752126
NONHSAG042418	5.31203921	5.12170564	5.29979476	4.93527989	5.37731454	5.10388784	5.38048187	5.24803719	4.92988109
NONHSAG042419	5.32156607	5.46354602	5.56711608	5.10586427	5.5100125	5.35053511	5.76208058	5.18349221	5.45986887
NONHSAG042424	4.88100146	4.71458976	5.15272134	5.09591695	5.00896479	5.03871535	4.79988306	4.87001229	4.73692364
NONHSAG042444	4.4824884	4.53793039	4.56191522	4.47704103	4.55021623	4.42412979	4.77433175	4.67180327	4.27011217
NONHSAG042451	3.89991742	4.02233957	3.71058211	3.77426409	4.22078683	3.95057237	4.01041836	4.22806318	3.62398578
NONHSAG042471	2.96268029	3.06056928	3.25861909	3.1681945	3.00012255	3.10641668	3.35486896	3.34061329	3.31749595
NONHSAG042535	2.21919434	2.36864168	2.38645089	2.43533718	2.2827	2.37433484	2.42378013	2.37835259	2.43876057
NONHSAG042546	3.02787709	3.25942316	2.92916536	3.23811037	3.20446084	3.38906672	3.40248175	3.22787569	3.2096276
NONHSAG042558	4.04544245	3.88270952	4.06839878	3.98093062	4.3371833	4.03467078	4.33094684	4.03133036	4.07005374
NONHSAG042615	2.71200676	2.23903685	2.52763173	2.48830004	2.54011302	2.5624156	2.93609639	2.60997644	2.53073638
NONHSAG042636	4.54340294	4.37423749	4.79207458	4.47499614	4.56717777	4.46549903	4.32876588	4.42272735	4.43717714
NONHSAG042638	4.51501638	4.49462167	4.68686405	4.62051193	4.6962221	4.69732126	4.61678782	4.84813823	4.82505564
NONHSAG042726	4.31790904	4.50667758	4.47598863	4.33263377	4.40922551	4.31296096	3.93742796	4.19718256	4.13459134
NONHSAG042795	3.24343787	3.41229164	3.56131035	3.40513907	3.33373191	3.43154536	3.32417216	3.31924851	3.38280899
NONHSAG042834	3.43662768	3.71725484	3.71853473	3.50672776	3.6666263	3.41058437	3.82730267	3.59291581	3.71697314
NONHSAG042877	3.33896765	3.10735527	3.53503893	3.36306762	3.2017213	3.41422231	3.39849635	3.26847255	3.76972944
NONHSAG042907	6.77067807	6.64882314	6.64035698	6.72586185	6.6754485	6.72628406	6.62083939	6.77579953	6.68127075
NONHSAG042913	2.43863375	2.36287289	2.40072949	2.51587279	2.58807581	2.36769561	2.50688787	2.27653681	2.24503289
NONHSAG042922	3.13445449	3.09162557	3.14892653	2.93304601	3.15660325	2.82007923	3.03853106	2.96683138	2.9464664
NONHSAG042934	3.35232873	3.40902533	3.29084357	3.53810688	3.44564861	3.45462467	3.49761747	3.18742234	3.35652023
NONHSAG042947	5.70734893	5.5066904	5.59280328	5.43167451	5.70599303	5.54826192	5.75284163	5.86208503	6.04614489
NONHSAG042959	3.24878888	3.4831716	3.49134205	3.45770322	3.55707713	3.33143588	3.61602099	3.77708682	3.45288618

NONHSAG042979	3.24948415	3.01342954	3.22086925	2.89715491	3.22314988	2.88401585	3.65973855	3.21330015	3.31541253
NONHSAG042982	2.84678149	2.73055461	2.85309413	2.6002479	2.8423662	2.46199812	2.81165976	2.7067409	2.67182741
LOC284379	4.60356838	4.62434457	6.64883275	6.306769	6.13899547	5.93399143	5.28455309	6.0303343	6.01108702
EP300-AS1	5.25830361	5.52088745	3.63660648	3.40518958	3.13421869	4.34922315	3.64392365	3.73227192	3.58499727
DEPDC1-AS1	4.06833596	4.20066504	2.59233512	2.26467766	2.50137507	2.45982448	2.3853262	2.82243477	2.48070278
RUNDC3A-AS1	5.30933524	5.2247486	6.66620945	6.58068124	6.28280232	7.09025069	6.51493947	6.8854069	6.69907202
PTPRG-AS1	7.4608437	7.04772057	4.88893777	5.17392291	4.6725904	5.46033844	5.21965521	5.34497547	4.73394006
ELOVL2-AS1	7.74153775	7.53476558	5.65136416	5.21500427	4.74849471	6.16659192	5.24223516	5.68694243	5.3097782
NONHSAG016418	6.03872102	6.21568075	3.84851728	3.69542023	4.11329932	4.27662206	4.18121296	4.0845538	3.80634358
TMPO-AS1	6.19467173	6.60091171	4.10709952	4.10751515	3.97374855	4.4471274	4.61491551	4.66662325	4.34707788
NONHSAG042983	2.36163767	2.29491134	2.28083441	2.299777	2.33749435	2.28029879	2.52957886	2.34434324	2.33041146
NONHSAG042995	3.97067622	4.00939976	3.8585974	3.58544828	4.15730542	3.47335317	4.49485526	3.80611132	3.84881279
NONHSAG043003	2.85489968	3.08433187	3.01754462	2.59789247	2.93162841	2.99288926	2.85262406	2.85682786	3.06473893
NONHSAG043014	2.78832732	3.14358019	3.42232126	3.01784016	3.1289727	3.01881882	2.89326152	3.12060218	3.06239244
NONHSAG043016	2.5875515	2.53626393	2.60252521	2.65252539	2.81042084	2.44486344	2.68244155	2.54640347	2.57096345
NONHSAG043027	4.55510774	4.41880673	4.5618836	4.55159266	4.73248252	4.77396019	4.52722938	4.59532539	4.41839814
NONHSAG043033	3.42988375	3.26684793	3.43712422	3.36054415	3.52183176	3.19644647	3.34551266	3.21255233	3.43165127
NONHSAG043043	6.88798669	7.08318939	6.82794748	6.79767325	6.67224916	6.67484875	6.47450537	6.80653753	6.83208013
NONHSAG043047	5.15239347	4.96548932	5.32393405	5.64542958	5.26008592	5.0374802	5.2602194	5.37393947	5.54175264
NONHSAG043071	4.905134	5.17883944	5.2699572	5.03059391	5.1000158	5.12398161	5.47623511	5.14261774	4.93381158
NONHSAG043074	4.31115339	4.37880232	4.35703303	4.00278046	4.59286682	4.22061715	4.52788681	4.51476116	4.48290062
NONHSAG043077	4.27476202	4.34723616	4.26249704	4.33462389	4.34155931	4.41268812	4.64153585	4.15368552	4.58483174
NONHSAG043093	3.56925609	3.63785563	3.58081712	3.28361998	3.31208952	3.18030297	3.38933104	3.3856839	3.20092189
NONHSAG043129	3.74115839	3.0903094	3.54017503	3.85871798	3.5356564	3.60363781	3.41734273	3.63203296	3.59162994
NONHSAG043135	5.91812103	6.03562147	6.27025441	6.00179024	6.29272815	6.22066576	6.34553892	6.46182837	6.41786122
NONHSAG043144	3.31192757	3.79600434	3.75583417	3.69113164	3.92770126	4.03231548	3.67935223	3.65874582	3.64977901
NONHSAG043195	3.23498051	3.08595533	3.2066223	2.92753392	3.34454568	2.97784642	3.14513698	2.85310349	3.0660431
NONHSAG043203	5.74955136	5.95140788	5.73409404	5.99832513	6.24963007	6.07518787	5.67981617	5.82574459	5.74924602
NONHSAG043218	2.04377244	2.13321324	2.27651327	2.13593324	2.26521947	2.25392993	2.18899148	2.27586198	2.19921385
NONHSAG043252	2.52472247	2.46752041	2.43289889	2.27330834	2.59017633	2.52748339	2.44462077	2.43626795	2.47473256
NONHSAG043277	4.5600052	4.58583301	4.66255525	4.60657096	4.39138017	4.32921809	4.72504254	4.23168964	4.30444089
NONHSAG043281	2.51851802	2.47023684	2.50094875	2.63870939	2.57465602	2.53463334	2.59461565	2.6723025	2.28423411
NONHSAG043292	2.64372927	2.96056695	3.28087103	2.75945119	3.1499894	2.97903316	3.48146241	2.51845386	3.24032832
NONHSAG043304	2.65041993	2.92897386	3.03710096	2.77579176	2.65905989	2.9357923	2.84456001	2.71453625	2.64021591
NONHSAG043313	3.51518333	3.63393315	3.64675076	3.80857431	3.81362227	3.58355086	3.60198016	3.68129846	3.70683461
NONHSAG043379	6.25381733	6.27847892	6.282615	6.24750528	6.42203713	6.1973389	6.43742474	6.40810866	6.26539894
NONHSAG043614	3.40204646	3.62845384	3.6327308	3.75322078	3.61533121	3.67968423	3.65572766	3.54587295	3.57068822
NONHSAG043616	4.70194789	4.74772754	4.60554213	4.75156724	4.73366327	4.5992186	4.95839786	4.83990837	4.73428148
NONHSAG043628	8.86329144	8.87231735	8.77928331	9.06581246	8.89676901	9.12154914	8.89077557	9.00082225	8.91218035
NONHSAG043629	7.74138635	7.69748144	7.77335227	8.03552686	7.78331469	8.04409276	7.89315449	7.98586087	7.98746857
NONHSAG043634	2.31817504	2.51444803	2.33274828	2.3286019	2.3898345	2.40657141	2.47918481	2.64861341	2.46626093
NONHSAG043637	6.63915522	6.35462581	6.77527079	6.87055474	6.71928198	6.9520343	6.69581545	6.84885645	7.01342934
NONHSAG043646	5.44057036	5.74740797	5.6546432	5.74472653	5.84648014	5.98154674	5.55981229	5.58855773	5.64499455
NONHSAG043677	2.78512078	2.77203988	2.77075416	2.7115718	2.52163888	2.83117755	2.80076907	3.00806694	2.77621595
NONHSAG043699	3.01361706	3.00153633	2.76771038	2.69949636	2.70001628	2.65116289	3.10782386	2.75695731	3.00077009
NONHSAG043706	4.59519857	4.45176637	4.62213704	4.56129761	4.84986788	4.41520291	4.17906194	5.62501095	5.29859296
NONHSAG043726	2.62832765	2.65642196	2.79663468	2.75113149	2.68659327	2.97004545	2.76702458	2.63979195	2.79182937
NONHSAG043748	4.95726302	5.19867844	5.17683806	4.96851675	5.40782657	5.0535132	5.09306764	4.93140314	5.13136236
NONHSAG043765	6.95881481	6.74411162	7.27789837	6.70357784	7.08202774	6.65456516	7.30811695	6.73764955	7.05253751
NONHSAG043790	5.55943907	5.65036722	5.78399618	6.06357001	5.94886161	5.78889175	5.79598259	6.21830438	6.08812904
NONHSAG043821	5.94839652	5.38814053	5.54798623	5.6883823	5.70471963	5.36539873	5.46739152	5.81412396	5.69947751
NONHSAG043825	2.95273064	2.87738777	2.75204249	3.25770251	2.91270164	3.00362911	3.16802757	3.0572276	3.07286439
NONHSAG043835	3.27305458	3.18207653	3.15251288	3.13849821	3.13747298	3.02495374	3.09294552	3.11152773	3.07992872
NONHSAG043845	4.86197729	4.87240624	5.0549459	4.65710497	4.91695908	4.59692519	4.79118932	4.76130527	4.76612999
NONHSAG043849	4.27857675	4.40440429	4.41336157	4.01578217	4.32292735	4.16475885	4.26638931	4.25847512	4.05378518
NONHSAG043854	4.06227329	3.97090943	3.99779755	3.96142593	4.00604683	3.80303097	4.14276052	3.99308224	3.92673982
NONHSAG043856	4.81379963	5.01839233	5.45703947	4.92679773	5.32029522	4.83801522	5.14531235	4.91792528	5.07774517
NONHSAG043865	3.41477894	3.42016009	3.47177035	3.51904277	3.51587849	3.51581552	3.38193056	3.53476311	3.54346957
NONHSAG043905	9.56628511	9.65480891	8.22844195	8.89400648	8.61041455	8.72411723	8.32879177	8.91947199	8.28931003
NONHSAG043910	3.78660279	3.85976809	3.74698341	3.79781349	3.80144779	3.7724771	4.35430212	3.90619323	4.00639344
NONHSAG043913	2.67849399	2.86716185	2.91910297	2.90885056	3.054876	2.87927854	2.84254482	2.93222798	2.98679018
NONHSAG043916	3.3156546	3.71159179	3.59195049	3.58932547	3.45410611	3.65012597	3.55564564	3.80211474	3.50851202
NONHSAG043927	3.62757271	3.78484103	3.40807259	3.54667465	4.0720634	3.54657572	3.78892488	3.65032315	3.66623517
NONHSAG043945	3.00808845	2.73271014	3.13756999	2.8443743	2.96853091	2.98063127	3.01932684	2.94380077	3.10091783
NONHSAG043980	3.93555937	4.07703612	4.05747679	4.2864436	4.40588617	4.4153775	4.13279071	4.19146135	4.31602386
NONHSAG043986	3.47402084	3.24851914	3.16990778	3.1869319	3.54773751	3.12726968	3.45660896	3.15216136	3.01761549
NONHSAG044024	2.22505301	2.14205768	2.61005095	2.14326465	2.09788738	2.00919758	2.39229753	2.32132731	2.42533879
NONHSAG044064	3.36632034	3.56120243	3.48009046	2.92753392	2.80423398	2.37322934	3.35934896	2.78246741	2.60215916
NONHSAG044066	3.11939708	2.59559413	2.79149456	2.71795053	2.94321739	2.65528479	2.80444382	2.70619993	2.79202375
NONHSAG044070	3.10024342	3.45578699	3.50441273	3.37871527	3.34981749	3.26595696	3.47347196	3.10216374	3.36349702
NONHSAG044072	2.63535407	2.68024611	2.5827953	2.52725462	2.84244165	2.47528898	2.57440611	2.58708867	2.55820985

NONHSAG044100	6.73792761	6.85543517	7.97272496	8.49025128	7.92236108	8.20998304	8.03098892	7.63497634	7.44309736
NONHSAG044111	2.75705817	2.85234915	2.86563356	2.9690151	2.66965823	2.76275275	2.80719425	2.75064829	2.88070633
NONHSAG044194	3.67192195	3.84024677	3.82660429	3.80350991	3.58554205	3.54966181	3.80027637	3.57985853	3.37525393
NONHSAG044196	4.97932743	4.84177343	4.63234621	4.61795808	4.61526575	4.75016309	4.71876171	4.71767912	4.67676025
NONHSAG044199	3.34829929	3.24185534	3.46315418	3.2077691	3.32508648	3.37430433	3.31296059	3.5405321	3.38061022
NONHSAG044203	4.71107203	4.49173329	4.58966906	4.20338384	4.5821174	4.18100814	4.89485951	4.71872301	4.51692642
NONHSAG044217	4.3762892	4.07794264	5.11647194	5.3704037	4.9200027	5.1077144	4.91863139	5.60479774	5.06658329
NONHSAG044226	3.09349473	3.28379889	2.74407115	2.94859304	3.05183628	3.00223846	3.25108146	2.98949628	3.01517359
NONHSAG044243	3.56656877	3.67852251	3.72853563	3.82503058	3.9230002	3.49716725	3.99275147	3.7402905	3.61166997
NONHSAG044245	3.24170243	3.63973932	3.49633088	3.55882516	3.43876671	3.29191346	3.29775124	3.49262135	3.29559504
NONHSAG044258	3.37387922	3.32641697	3.71156206	4.11591095	3.35187695	3.39495423	3.1715414	3.62259393	3.62117601
NONHSAG044264	4.37657681	4.28180276	3.85082796	4.18016731	4.15289017	4.32432767	4.16634305	4.24424925	4.10621001
NONHSAG044265	2.30880884	2.25205297	2.31133442	2.41219712	2.22313097	2.5022996	2.46032157	2.29517587	2.22516335
NONHSAG044266	2.74866917	2.60376032	2.80963512	2.25759263	2.22999699	2.56168906	2.4350781	2.50378923	2.48796177
NONHSAG044270	3.35037318	3.25107568	3.10994291	3.17862827	3.26550993	2.89073261	3.13030693	2.90876075	2.97186649
NONHSAG044283	7.73886918	7.46404598	7.24958604	7.49374467	7.25770014	7.35492703	6.96259438	7.57670055	7.57103527
NONHSAG044290	2.38405439	2.15126731	2.27663474	2.35098045	2.471859	2.6551228	2.50505157	2.50930048	2.401088
NONHSAG044304	3.47237142	3.56153412	3.90944605	4.31396466	3.72501906	3.94631661	3.78669888	4.2808432	4.70600063
NONHSAG044336	4.27927445	4.27843237	4.28504222	3.93673979	4.41750299	3.80448482	4.449106	4.14525477	4.18216368
NONHSAG044342	3.2068855	3.43447026	3.65876201	3.32368609	3.4087022	3.48570196	3.45362922	3.59533365	3.50499759
NONHSAG044349	5.308218	5.52969184	5.93669831	5.41788149	5.89208284	5.45789417	5.62745878	5.70507149	5.59654402
NONHSAG044369	2.53807331	2.21476305	2.40654034	2.23550509	2.31114525	2.21429284	2.40652003	2.43282254	2.29916015
NONHSAG044447	2.14163712	2.15960773	2.28348331	2.40688714	2.2339614	2.19299386	2.32920137	2.11045348	2.25012137
NONHSAG044503	2.39003838	2.37607558	2.40357643	2.53250041	2.32492279	2.3992234	2.47087923	2.27413161	2.50577258
NONHSAG044507	2.13139499	1.87801257	2.16341674	1.93576549	2.00546908	2.00564119	1.96961446	1.97620621	2.10850989
NONHSAG044512	2.76289044	2.68881885	3.04877539	2.8653733	2.78107478	2.87132959	2.73653259	2.83858615	2.96420777
NONHSAG044540	2.73797187	2.80979607	2.82025354	2.87153223	2.65794784	3.17606616	2.69085143	2.69505523	2.77699004
NONHSAG044561	2.77186603	2.82451458	2.93092122	2.8443743	2.79236401	3.10794699	3.14351519	2.80371709	3.11025399
NONHSAG044585	2.69793183	2.45402225	2.44237861	2.38468364	2.7227421	2.42779623	2.47420037	2.31531517	2.38371191
NONHSAG044626	2.9348559	2.91422256	2.65571827	3.06010621	2.55106419	2.92061814	3.02218133	2.69428278	2.88385195
NONHSAG044627	2.74684752	2.79184047	2.89325473	2.85205006	2.99702954	2.64905399	2.89789705	2.74987389	2.85751581
NONHSAG044671	3.00058532	3.19051847	3.24860043	2.99382339	2.97268592	3.03862832	3.24397356	3.02347578	3.21650195
NONHSAG044686	2.46595134	2.49466862	2.48594831	2.57342552	2.73041575	2.44187512	2.68125919	2.47869349	2.43153338
NONHSAG044742	6.17072608	6.07935019	6.68627315	6.91600979	6.43980812	6.72117927	6.58090533	6.47205078	6.48609935
NONHSAG044767	3.64498547	3.66153597	3.88542837	3.23788748	3.98784931	3.37379368	3.93702374	3.58209167	3.4933264
NONHSAG044790	3.21654768	3.28852379	3.58081712	3.10411728	2.9873422	3.12889653	3.18852332	3.32918457	3.31925406
NONHSAG044806	4.67618359	4.57188401	4.91074011	5.09745118	5.14754092	4.71172022	4.72052475	5.98029253	5.55153825
NONHSAG044810	6.46162431	6.64253574	6.47953579	6.76465372	6.84768295	6.59581732	6.7066447	6.72253886	6.66622766
NONHSAG044811	2.8227487	2.80476237	2.55662603	2.6877591	3.00127043	2.84193091	2.86265131	2.78246741	2.96517554
NONHSAG044815	2.03495079	2.0302074	1.97671784	2.10106258	2.14935723	1.95488737	2.21455241	2.00098778	2.05674348
NONHSAG044846	4.43691556	4.53957869	4.68025077	4.57554388	4.56483371	4.46946955	4.53481132	4.64067551	4.53746631
NONHSAG044851	2.8785293	2.87240816	2.83080794	3.03208183	2.77108062	2.91700309	3.06119005	2.87783415	2.9577591
NONHSAG044892	4.21484654	4.2038764	4.10206634	3.98975111	4.12830662	3.97540406	4.03830992	3.93951311	3.93868883
NONHSAG044894	4.72645397	4.38705412	4.75185565	4.73678654	4.36809224	4.64242511	4.81294635	4.45591369	4.46801391
NONHSAG044918	2.79966424	2.7799486	2.42708798	2.73114225	2.86368709	2.66217449	2.52961562	2.46587722	2.57227396
NONHSAG044920	2.84645204	3.14980538	2.96842003	2.83662005	2.96320588	2.73652699	3.18056615	2.82213232	2.92463013
NONHSAG044929	5.52861402	5.42524219	5.82948933	6.1521703	5.79793862	6.07201473	6.06386698	6.13072078	6.06331027
NONHSAG044999	3.00463338	3.24469044	3.07926681	2.85803055	3.26372014	3.05569164	3.22655141	3.03681171	3.1575527
NONHSAG045020	2.4035867	2.26568912	2.43838174	2.51386464	2.61476254	2.40932342	2.51915058	2.41973315	2.42175986
NONHSAG045068	2.48530838	2.36293485	2.20222815	2.51013327	2.4602309	2.63885001	2.295316	2.52698215	2.40513522
NONHSAG045080	3.42720949	3.33765716	3.24499885	3.36898464	3.42200852	3.27512816	3.59991391	3.103682	3.49371852
NONHSAG045081	4.11394887	3.83330364	3.97655471	4.17295629	3.99706735	3.88451022	4.00881788	4.33984414	4.31441038
NONHSAG045085	5.12518271	4.98393455	4.83961821	4.79009368	5.09772194	4.90912294	5.24805768	4.75121816	4.85671318
NONHSAG045101	4.21481469	4.08239421	4.02981068	4.02076054	4.15953396	3.91847639	4.15095855	4.17217995	4.02384024
NONHSAG045116	2.90128105	2.59122039	2.86148085	2.82348309	2.72310723	2.56779997	2.75206481	2.83246416	2.7696968
NONHSAG045138	3.18783549	3.34953533	3.28966246	3.33453665	3.71555425	3.42437454	3.49037244	3.24273087	3.27891597
NONHSAG045167	3.64625853	3.65803682	3.66510689	3.78153201	3.71797993	3.7716667	3.90739428	3.67274341	3.67774464
NONHSAG045192	3.26949937	3.41992176	3.03026432	3.53656363	3.58208194	3.39690347	3.9809341	3.47465675	3.20319749
NONHSAG045208	4.05019301	4.00714133	3.83105886	3.97752579	3.69726779	3.75943018	3.91797553	4.00545892	3.90411203
NONHSAG045235	6.26741792	6.49688949	6.36224513	6.50780282	6.4050224	6.31584215	6.50146063	6.78703863	6.55697383
NONHSAG045258	7.72066976	7.2474399	7.47911075	7.78489552	7.72784259	7.8905706	7.81614682	8.19557981	7.97151835
NONHSAG045265	4.78712991	4.5254347	5.10537	4.60529103	4.69352367	4.37598595	4.80465775	4.66830083	4.61631755
NONHSAG045281	2.98814115	2.75961266	3.21411119	2.96331781	3.32860058	2.99613714	3.11743853	2.88606198	3.07639627
NONHSAG045316	2.22435087	2.23381502	2.36372974	2.35594425	2.22815547	2.39493732	2.20859152	2.34050601	2.21404075
NONHSAG045329	2.33917441	2.34635176	2.35776751	2.37517522	2.60003113	2.12231667	2.35563712	2.24471606	2.44212025
NONHSAG045333	4.86517347	4.68402494	4.47757892	4.71987236	4.49341166	5.11893514	4.27194978	4.99913333	4.32467154
NONHSAG045338	2.85605717	2.42899024	2.64107518	2.66467279	2.83342893	2.58475576	2.94675714	2.61747729	2.73512548
NONHSAG045350	2.66225875	2.60480837	2.63419343	2.95059719	2.81069106	2.55971319	2.76400229	2.71189411	2.67790504
NONHSAG045363	5.11530138	5.32455177	5.53443905	5.47934026	5.33953123	5.23688942	5.88428496	5.41169876	5.27560713
NONHSAG045364	4.13759882	4.06795131	4.17340728	4.04998349	4.30985705	4.45720795	4.30168182	4.10954074	4.34030934
NONHSAG045389	2.96881999	2.816933	2.96638524	2.68628118	2.7249921	2.69449743	2.98067142	2.64989031	2.68904873

NONHSAG045403	5.59710633	5.50207325	5.79523285	5.64061682	5.75282308	5.59782621	5.73125625	5.67653828	5.59416019
NONHSAG045405	5.94515994	5.39993745	3.67563054	3.89849538	3.73198535	4.39237216	3.02822444	4.25836938	4.2300051
NONHSAG045408	4.27250745	4.48697203	4.39314348	4.3505508	4.05129046	3.85073769	4.12259921	4.27204152	4.22459926
NONHSAG045419	4.06791553	4.02185012	4.09329386	4.31932869	4.42257439	4.03134156	4.42943089	4.27955331	4.19665049
NONHSAG045425	3.57838025	3.73809459	3.57876833	3.28575061	3.42271372	3.27847672	3.64586768	3.46883058	3.53546086
NONHSAG045429	3.69776726	3.53529261	3.66639172	3.51484707	3.52005458	3.66175714	3.70005714	3.61982222	3.65454942
NONHSAG045445	3.80823382	3.81671963	3.87216938	3.30728646	3.60957352	3.32153717	3.47345302	3.47763107	3.53530091
NONHSAG045449	2.34965628	2.53685259	2.35833566	2.46572982	2.63731008	2.51881556	2.35347215	2.3606404	2.33312982
NONHSAG045463	3.422258	3.44320962	3.64961468	3.29767126	3.36836324	3.0952001	3.26828003	3.24141708	3.43343192
NONHSAG045467	4.67341286	4.76666884	4.99458607	4.76957835	4.89621601	4.94109073	5.00327368	4.9194806	4.79404134
NONHSAG045472	3.42947037	3.544583	3.88787876	3.54925165	3.62466961	3.48045343	3.81133542	3.50841077	3.87371315
NONHSAG045505	4.02509407	4.18784784	4.13016681	3.80492265	3.89596194	3.94916018	3.88369212	3.78130368	3.97339286
NONHSAG045512	3.78089642	3.5350489	4.04935795	3.36694175	3.8674377	3.75531689	3.94482018	3.84265329	3.92152011
NONHSAG045517	2.94239744	2.92931654	3.09062037	2.99099739	3.32745171	3.19762952	2.95556551	3.10294061	2.64176293
NONHSAG046716	5.50493114	5.68203757	5.5365269	5.20784154	5.49733309	5.66032645	5.97822311	5.52844522	5.62356474
NONHSAG046750	5.47633891	5.54906865	5.46944772	5.42619149	5.71891219	5.79893062	5.51976122	5.40881905	5.4523827
NONHSAG046781	5.47718633	5.43388528	5.52513801	5.29115311	5.44751338	5.0627628	5.69269711	5.63853109	5.40308878
NONHSAG046784	5.24171444	5.0009603	5.04076415	4.93852745	5.02058115	5.02126886	5.03428708	5.05557563	5.13754702
NONHSAG046797	4.44961874	4.56796472	4.45350144	4.56427165	4.4659924	4.45060142	4.45690798	4.64508665	4.44573339
NONHSAG046806	6.48521969	6.28829887	6.43356255	6.58083967	6.24817434	6.4629008	6.56723972	6.31807163	6.35761634
NONHSAG046817	4.0564578	4.20949791	4.39764944	3.37912715	4.04313149	3.79716338	4.476205	3.88697953	3.98096647
NONHSAG046838	4.08808463	4.17846845	4.06407209	3.96018693	4.18475233	3.68885762	4.2709417	4.02413396	4.06101541
NONHSAG046851	4.24458474	4.51540622	4.45826827	4.13532128	4.41243414	4.07646346	4.67010314	4.16121139	4.42780219
NONHSAG046903	4.53150728	4.39139612	4.55445647	4.63745164	4.43544222	4.53896884	4.6625519	4.62490094	4.47082114
NONHSAG046927	2.71262324	2.89061544	2.93360154	2.75522736	2.86574744	2.85543253	2.79057437	2.77563053	2.83576946
NONHSAG046961	2.91108946	3.08636426	3.11633003	3.29645671	2.92220394	3.06958542	3.047645	2.98438045	2.97599166
NONHSAG047021	2.45994396	2.62687426	2.54892999	2.48857033	2.39316163	2.29819561	2.1902946	2.6223716	2.50097048
NONHSAG047026	4.20147517	4.39745985	4.7461669	4.7171148	4.56055218	4.65537671	4.68058965	4.58469968	4.86550471
NONHSAG047060	2.66983751	2.75453833	3.04678579	2.97254372	2.71436885	2.50952233	3.13526737	2.77565515	2.87093918
NONHSAG047082	3.53961164	3.63265241	3.7859391	3.477844	3.71183392	3.47178788	3.98135861	3.5588543	3.46744699
NONHSAG047088	2.57588973	2.46741808	2.70040942	2.38823553	2.17725492	2.32649959	3.04697559	2.42448705	2.25475924
NONHSAG047097	4.32718422	4.84063881	3.92123751	4.97875208	3.4876526	4.59119928	4.07964314	4.4739682	4.05979184
NONHSAG047101	2.83665617	2.79068926	2.74829751	2.45532459	2.87958402	2.53460321	2.79239984	2.85311514	2.6862727
NONHSAG047126	2.49499754	2.2172566	2.46332219	2.51430568	2.56614713	2.46049709	2.5524685	2.62974414	2.5130166
NONHSAG047149	3.82086185	4.05821487	3.98885689	4.06253823	3.87725339	4.06860871	3.9320102	3.8600847	3.73681215
NONHSAG047152	2.87052829	2.83273214	2.80223435	2.85935119	2.97539337	3.06944208	2.72149678	3.02269521	2.78273065
NONHSAG047169	2.57039865	2.64173809	2.68633192	2.57839066	2.57895098	2.57839066	2.57762427	2.72290651	2.85490801
NONHSAG047175	3.52943677	3.28756326	3.21048021	3.40976054	3.41090245	3.35636114	3.51619883	3.43774421	3.37434932
NONHSAG047181	2.99020932	2.95283737	3.38222521	3.05518057	3.14602868	3.19723797	3.37323149	3.37323168	3.28374374
NONHSAG047192	4.23444641	4.35223016	4.76387664	4.3727016	4.48758481	4.566185	4.91546187	4.48384141	4.65789946
NONHSAG047193	3.10956222	3.22442073	3.04630689	3.12692712	2.99372693	3.21928261	2.99842888	3.08312619	3.35555598
NONHSAG047194	3.13179748	3.13302594	3.30027496	3.21107356	3.26630528	3.21365964	3.59210086	3.42967883	3.14432129
NONHSAG047208	2.48547394	2.55505568	2.70571239	2.6805319	2.84034199	2.3222346	2.72886218	2.39536719	2.52449211
NONHSAG047221	3.47625178	3.49100254	3.42402716	3.38324237	3.54773751	3.46334458	3.3262723	3.09895548	3.41209702
NONHSAG047229	4.96127536	4.7556773	4.61531421	4.47753554	4.81626533	4.72381772	4.4847361	4.72499201	4.71411066
NONHSAG047232	3.78279179	3.98133552	3.82917375	3.79486847	3.95308539	4.12512649	4.25866681	3.74408098	3.88547722
NONHSAG047253	3.31640364	3.20372984	3.10330952	3.38164653	3.16965338	3.22516171	3.0967195	3.40565266	3.09896638
NONHSAG047257	3.4997293	3.44941568	3.29543375	3.65871193	3.51680247	3.35756161	3.38792428	3.59825901	3.23518576
NONHSAG047259	3.61575882	3.48155047	3.40850268	3.81401862	3.72099751	3.32212457	3.61137331	3.6256966	3.16475597
NONHSAG047273	5.69942061	5.74098006	6.01006313	5.87683492	5.76011074	5.48938472	6.29178398	5.97910298	5.91426375
NONHSAG047282	2.73154562	2.76136775	2.70564027	2.78175046	2.74412438	2.73391142	3.04036958	2.68431913	2.74164561
NONHSAG047295	3.11063664	3.28560644	2.5664123	2.8807124	2.88411074	2.94007984	2.88366906	3.05712413	3.15393002
NONHSAG047314	4.7220035	4.04561311	4.80064858	3.98630755	4.61386037	4.27507975	4.30965259	4.17387485	4.10995964
NONHSAG047336	4.96362993	5.02028578	5.39870085	5.56009571	5.18162768	5.35764103	5.04612028	5.41169876	5.36188269
NONHSAG047348	2.87775404	3.04363826	2.60579967	2.58544449	2.79786375	2.93623549	3.21653611	2.73711167	2.74360947
NONHSAG047356	2.75504746	2.52274554	2.63407926	2.59595877	2.72978898	2.8902838	2.93201848	2.7065137	2.73464848
NONHSAG047375	3.49576324	3.34386203	3.42761163	3.56972073	2.97063463	3.07925511	3.31790181	3.14889216	3.35634822
NONHSAG047399	2.61486113	2.76052363	2.64765485	2.59500579	2.62690325	2.77379776	2.52843013	2.66034979	2.8514616
NONHSAG047416	2.70954744	2.82344277	2.93030671	2.6665585	2.74917583	2.78847844	3.25166432	2.58407764	2.76356793
NONHSAG047425	2.39592303	2.65525771	2.5272513	2.41859892	2.49183874	2.34549239	2.75414677	2.21096113	2.41403329
NONHSAG047431	2.70764445	2.9817112	2.6425592	3.07417198	2.76398197	2.8291652	2.81704522	2.89779452	3.3081347
NONHSAG047443	2.72058455	2.9278532	3.05176499	2.87863869	2.73577986	2.87649666	2.86471383	2.93951657	2.92231412
NONHSAG047447	2.41558411	2.33478053	2.55569106	2.40574424	2.34068443	2.35209033	2.63575422	2.25404657	2.29916015
NONHSAG047469	4.41537418	4.17036442	4.04411724	4.20816817	4.19307228	4.18024992	4.56423281	4.33937705	4.59766244
NONHSAG047503	2.28485722	2.37607558	2.53886234	2.32634239	2.71814618	2.68814509	2.71038661	2.28517844	2.29916015
NONHSAG047517	6.26669153	6.31149389	6.51277554	6.41272979	6.47377597	6.3569712	6.43639978	6.46807541	6.33191365
NONHSAG047524	3.18853544	3.22448451	3.17551474	3.10682265	3.15455585	3.00376049	3.29948867	3.02269521	3.07646197
NONHSAG047536	2.14901469	2.42459463	2.22915429	2.18508926	2.24033991	2.5250428	2.08860702	2.08493681	2.23406669
NONHSAG047547	6.30927293	6.36716489	6.31470588	6.76878039	6.89369489	6.99649538	6.73577693	6.88346421	6.80953443
NONHSAG047553	4.62039528	4.7147769	4.70452505	4.08847888	4.68190074	4.15911864	4.9557527	4.4510506	4.49119151
NONHSAG047570	4.5539002	4.59932675	4.80253798	5.04757772	5.0097653	4.84727061	4.8882417	4.81587648	4.68212805

NONHSAG047611	4.0586904	4.12544091	4.17399654	4.12315543	4.09172361	3.96810504	3.96744791	3.98255486	4.00030122
NONHSAG047665	4.11475443	4.22175118	4.18523382	4.10004559	4.20858764	3.9743756	4.30417936	3.9525281	4.28458769
NONHSAG047674	2.92383033	2.68582649	2.80431003	2.50060412	2.84435992	2.67287466	2.62889513	2.50483756	2.61979327
NONHSAG047730	2.69390393	2.60891757	2.73179756	2.8069334	2.91064778	2.90250669	2.76355526	2.88452509	2.59595877
NONHSAG047747	3.40589025	3.63110765	3.45687328	3.8081661	3.42190311	3.30609714	4.01444324	3.47591151	3.71759219
NONHSAG047777	5.11366221	5.19375322	4.30845181	4.57911223	4.34542537	4.39681643	4.10126972	4.39336315	4.2285526
NONHSAG047807	2.92467695	2.84020783	2.86722048	2.89650063	3.16601049	2.75953843	3.37006211	2.75213368	3.10502577
NONHSAG047837	2.78404225	2.51764692	2.31142149	2.65550179	2.52281356	2.33186986	2.85779755	2.30568925	2.3843419
NONHSAG047842	6.4013703	6.51737592	6.83146023	7.09838362	6.56596981	7.08016857	6.67888983	6.70587751	6.94528519
NONHSAG047845	4.21712862	4.16653527	4.03506903	4.3503807	4.15645212	4.99468479	4.5018963	4.11282231	4.0212561
NONHSAG047847	5.45931315	5.33531737	5.57794456	5.22836209	5.32914642	5.1805559	5.82398709	5.33927967	5.46309803
NONHSAG047907	8.86530273	9.07816194	9.14926797	9.23251103	9.17321057	9.1295952	9.36111776	9.12226587	9.2051141
NONHSAG048011	4.80484601	4.60368891	4.50477924	4.62922198	4.32341791	4.28111323	4.49439376	4.66877117	4.3380517
NONHSAG048092	2.34020421	2.47924674	2.32656725	2.36594825	2.51269976	2.52724422	2.6334007	2.34818817	2.37386911
NONHSAG048098	2.50847396	2.43512107	2.46396629	2.36892301	2.44844112	2.38165667	2.44972132	2.42383779	2.42685328
NONHSAG048120	3.64662243	3.76274742	3.44309825	3.30531959	3.3310224	3.19711059	3.57990534	3.40352531	3.38375042
NONHSAG048123	2.95765106	2.83576946	3.13920164	2.72708026	2.85293458	2.64876321	3.04410867	2.73105508	3.04354786
NONHSAG048148	1.96866951	2.16635312	2.14065396	2.32866143	2.22845857	2.08629974	2.25710566	2.3371375	2.20326315
NONHSAG048155	4.36229809	4.12437581	4.49300893	4.0636485	4.26782797	3.67847137	4.57709972	4.24855407	4.2434995
NONHSAG048166	2.90054172	2.80109388	2.9104287	2.53642777	2.80011925	2.72050368	3.02757851	2.83707745	2.85831939
NONHSAG048202	3.18164664	3.02772635	2.77861851	2.94372117	3.12110048	3.01545755	3.2412213	3.06752702	3.29204095
NONHSAG048386	6.08704325	6.17306155	6.25907134	5.96529092	6.38042227	6.04991488	6.31932853	5.93375873	6.07857996
NONHSAG048397	8.1584514	7.82405838	8.41836101	8.34973462	8.39088655	8.24949295	8.16692722	8.35622156	8.31519134
NONHSAG048404	4.69654429	4.66775803	5.13963324	5.08132599	4.99212311	4.71002777	4.75536035	4.78750508	5.00633994
NONHSAG048405	2.42355068	2.14076185	1.95132083	2.31902482	2.29407486	2.22503793	2.14610124	2.16206974	2.25438853
NONHSAG048439	2.88677371	2.97890512	3.04737498	2.69699544	3.05010845	2.73482614	3.01987072	3.1620612	2.96718353
NONHSAG048458	2.48048401	2.23903685	2.27948273	2.48626882	2.32879881	2.23593012	2.59156126	2.4539458	2.49115625
NONHSAG048471	5.26638112	5.50565815	5.85168515	6.42304526	6.30167226	6.22153932	6.01935297	6.82994065	6.45816218
NONHSAG048506	2.97332346	2.94443687	3.19320197	2.70135568	3.09474066	2.88401585	3.37365532	2.76872848	3.13927657
NONHSAG048520	3.89629552	4.15903196	4.23571267	4.16220236	4.18696104	4.22756922	3.96183864	4.39180225	3.96470344
NONHSAG048562	2.29950856	2.37607558	2.33823649	2.40677121	2.50312525	2.24140289	2.61417358	2.48680502	2.51713659
NONHSAG048563	5.67383413	5.25238663	6.01932181	5.62713063	6.09622335	5.75794312	6.07097239	5.89686327	5.85025961
NONHSAG048569	4.26065743	4.6774441	4.24326983	4.32650955	4.43789479	4.65054939	4.6803847	4.56787411	4.67990295
NONHSAG048593	2.37423381	2.79822439	2.42902319	2.68543028	2.38934064	2.56446269	2.5477845	2.32164024	2.58988156
NONHSAG048597	2.56197472	2.3176086	2.18540126	2.36475749	2.38557455	2.2731371	2.47120966	2.50078625	2.21636742
NONHSAG048604	3.60566526	3.76625426	3.79719707	3.97411523	3.61444726	3.96808663	3.32852473	3.83200417	3.7058897
NONHSAG048605	5.06454247	4.93320551	5.12210427	4.94414968	5.00621451	4.75584041	4.78927541	4.89326104	5.04869832
NONHSAG048651	3.3897936	3.5080319	3.40910581	3.1316129	3.27102272	3.39132016	3.36878908	3.35686952	3.3841321
NONHSAG048682	4.92284561	5.33888974	5.65483776	5.27593344	5.52404672	5.37328542	5.45442991	5.32513564	5.10713845
NONHSAG048687	2.97818668	2.93270732	2.90593344	2.87765201	2.92158164	2.85925719	2.86652602	2.87276904	2.81915761
NONHSAG048746	5.52779784	5.50887039	6.07515769	6.15846172	5.52000759	5.85711679	6.01567025	5.90899177	5.82052924
NONHSAG048747	7.57624513	7.67973203	7.81236185	8.25309071	7.94084201	8.23222796	7.80148398	8.43908423	8.1554827
NONHSAG048781	5.08174706	5.00288723	5.16373031	5.1124232	5.341035	5.16587888	5.21390712	5.29687892	5.24007515
NONHSAG048790	2.53628198	2.37074629	2.27169388	2.92753392	2.39960214	2.32181063	2.27457997	2.123419	2.29877813
NONHSAG048794	5.01062252	5.13594626	5.18432376	5.21415584	5.3007427	5.40841305	5.17096535	5.26949435	5.10661196
NONHSAG048813	3.67025838	3.49708998	3.55102398	3.35331313	3.75981477	3.49270832	3.78487961	3.52681776	3.8100803
NONHSAG048817	3.84383913	3.85444652	4.14013947	4.00259667	3.85048015	3.96269452	4.3817347	4.24900293	4.39203485
NONHSAG048836	4.71497987	4.77976448	5.07859219	4.82303448	5.01001526	4.79572365	5.00904206	5.15777062	4.91670795
NONHSAG048840	5.36005721	5.05691473	4.9375001	4.81130081	5.08392987	4.59975514	4.52827826	5.22654622	4.88351538
NONHSAG048846	3.45245914	3.33762244	3.25439867	3.33904421	3.64441832	3.49679211	3.2804759	3.8599778	3.58581206
NONHSAG048902	2.85995745	2.61732955	2.91352495	2.811918	2.94361853	2.66395997	3.11696967	2.82548858	2.75293548
NONHSAG048962	6.25504973	6.25519614	6.47229179	6.31147366	6.40536904	6.56889213	6.47348667	6.3419304	6.32767821
NONHSAG048976	4.8231784	4.85016083	5.09439194	5.41765912	5.23117391	5.16827412	4.83687223	5.01662366	5.40263394
NONHSAG048984	2.66307307	2.70180507	2.66430615	3.0392706	2.74271334	2.79044615	2.87991252	2.4350662	2.64278609
NONHSAG048993	6.04374619	5.76731568	5.66377055	5.68598719	5.93574471	5.70214117	5.86574073	5.88734292	5.73421542
NONHSAG049003	4.31680364	4.28664633	4.65430903	4.15260355	4.4600149	4.53526359	4.5683366	4.24434991	4.28031242
NONHSAG049010	3.74874418	3.89801944	4.00840785	3.94756841	3.80804951	4.11486261	4.01648651	3.97923758	3.86826539
NONHSAG049037	5.6709441	5.7083215	5.98449916	6.0255208	6.045671	5.9989829	5.77443349	5.84569741	5.97793058
NONHSAG049079	7.51886481	7.74183128	7.19443693	7.36033918	7.1663924	7.45916259	6.98476307	7.10081999	7.33596541
NONHSAG049095	7.04836471	7.18467966	6.17854728	6.39766119	6.68756804	6.98912325	6.41812514	6.67594271	6.74045289
NONHSAG049099	2.69476056	2.73573702	2.66901	2.66092348	2.84323255	2.6460728	2.84808209	2.72699123	2.66358101
NONHSAG049119	4.10101173	3.68460171	4.02006678	3.56748415	4.02397383	3.62173733	4.1906915	4.01826258	4.03484355
NONHSAG049134	3.84983813	3.84607366	3.91215572	3.67168716	3.93302577	3.93692156	3.69240365	3.75992523	4.00620916
NONHSAG049182	4.28922735	4.23290786	4.48823195	4.27411625	4.37572558	4.27838631	4.24551265	4.12698434	4.44409074
NONHSAG049240	4.62238297	4.65043081	4.66027877	4.40941593	4.6833879	4.12806908	4.34821852	4.11305685	4.4826521
NONHSAG049298	3.30581818	3.45260609	3.00630027	3.1277133	3.04657376	3.07830333	3.16381851	3.15374455	3.17835878
NONHSAG049309	6.04246777	6.07193149	6.30464922	6.26890454	6.3747757	6.33522206	5.8465281	6.59700709	6.39746956
NONHSAG049336	5.32267586	5.33159349	5.3761278	5.30268138	5.39957056	5.3064506	5.59488833	5.49447927	5.5656197
NONHSAG049339	2.67038405	2.63704556	2.5081571	2.55203259	2.73898107	2.72368496	2.8153661	2.6645564	2.64807124
NONHSAG049341	5.84721338	5.54876751	5.7086826	5.98333091	6.0910666	5.9732812	5.73552053	6.02681148	6.07635195
NONHSAG049344	4.08991865	3.88817772	4.33531221	4.29176442	4.14878508	4.3289828	4.34369859	4.08806957	4.3081466

NONHSAG049350	4.64750169	4.64445127	4.88501602	4.31866113	4.58335547	4.35791582	4.83939855	4.45145979	4.52779395
NONHSAG049357	3.00680727	3.26020033	3.22934472	2.97535822	3.2478197	3.11303902	3.07833799	3.17863482	3.36664355
NONHSAG049388	2.96870791	2.87643968	3.21015152	2.96465717	3.16696059	2.7398807	2.98614052	2.81487604	2.9169631
NONHSAG049409	2.98985513	2.87860582	3.08979965	2.8890503	2.78815884	2.87237933	2.83053242	2.71335822	3.12069571
NONHSAG049422	3.96759038	3.76274742	3.77561897	3.5468432	3.79204394	3.49865097	3.95890663	3.48187225	3.49159175
NONHSAG049424	5.49995639	5.51204287	5.1915595	5.57044473	5.52978862	5.34213011	5.60558637	5.67996511	5.37366549
NONHSAG049464	4.26787613	4.25921175	3.86640735	4.29621979	4.37149536	4.51263033	3.74482224	4.10941225	4.0903106
NONHSAG049487	4.59091002	4.51522605	4.38658918	4.57361981	4.2858737	4.20073988	4.264364	4.38211221	4.46457734
NONHSAG049492	3.0075631	2.83732814	2.72999448	2.70096181	2.83199345	2.93219277	2.96249145	2.78564671	3.02792677
NONHSAG049507	4.43254829	4.00648457	3.81406765	3.71404247	4.00497395	3.65339665	3.99969619	3.9537768	3.93692283
NONHSAG049508	3.59397621	3.71590837	3.66348143	3.63883359	3.68544322	3.55534576	4.02963425	3.63193698	3.65493345
NONHSAG049511	3.31094046	3.32659751	3.68768935	3.32685079	3.3022351	2.90867762	3.60177482	3.42186006	3.37100805
NONHSAG049515	2.74002301	2.3032562	2.50338896	2.33967784	2.61403883	2.4331085	2.37202695	2.8189479	2.35061239
NONHSAG049523	2.1837199	2.26757666	2.11137139	2.33126144	2.20539069	2.35401518	2.3876077	2.14837908	2.06600326
NONHSAG049524	3.62446098	3.60844164	3.71758033	3.45972389	3.91923554	3.34094608	3.97371684	3.59092255	3.81341471
NONHSAG049526	4.06064476	4.20537547	3.95699341	3.98937286	4.28228022	3.86894759	4.51851323	3.97751073	4.08191971
NONHSAG049528	4.72170707	4.20077968	4.72068666	4.56765848	4.81463761	4.38357297	4.7429512	4.06898805	4.74561337
NONHSAG049529	3.82889847	3.65917188	3.88694814	3.76110935	3.69414415	3.99274409	3.75525967	4.0101593	4.08471697
NONHSAG049530	2.80027592	2.60175407	2.84704085	2.77700132	2.68559873	3.09234885	2.57274093	2.98998941	2.93994919
NONHSAG049531	4.89537602	4.83752334	4.9754105	4.8456932	5.01588399	5.03691879	4.98718204	4.85305439	4.89945057
NONHSAG049532	3.5473988	3.32851615	3.35554204	2.92943412	3.1246615	3.13640572	3.28274854	3.30297433	3.34352168
NONHSAG049534	2.30362969	2.75562048	2.7705106	2.3285254	2.5081571	2.62491469	2.55847061	2.46305104	2.60345347
NONHSAG049539	4.91528763	4.76340738	4.68292129	4.74915253	4.84994353	4.63325723	4.75023213	4.89902467	4.82936602
NONHSAG049594	3.24561451	2.84064426	3.25936908	3.08602706	3.10567855	2.96921291	3.25833769	3.2002913	3.24082662
NONHSAG049595	3.39316414	3.54094301	3.78244246	3.43149681	3.48424081	3.62619221	3.46395275	3.25257759	3.43960014
NONHSAG049661	2.22020093	2.23903685	2.07717975	2.22240124	2.44813063	2.22682462	2.37789965	2.2576223	2.37413938
NONHSAG049665	3.00741738	2.6805319	3.07259386	3.02842165	3.09957866	2.66066215	2.8115633	3.28750472	2.82967642
NONHSAG049689	2.7120242	3.0183964	3.01962738	2.76519536	2.62689838	2.52017318	3.24408586	3.04269602	2.62211789
NONHSAG049734	3.75906792	3.74576915	3.93576331	3.65149494	3.8563445	3.82281847	4.0267869	3.92486956	3.73393295
NONHSAG049792	3.98893391	4.22574026	4.22800273	3.64675076	4.23745594	3.84972695	4.41475701	3.89026544	3.85325676
NONHSAG049868	5.25557613	5.44082535	5.60043376	5.68138503	5.59603231	5.50917546	5.65360145	5.574076	5.56553386
NONHSAG049879	2.92084356	3.11894776	3.08266524	2.85092251	2.9389378	2.90988786	3.07150163	3.08992057	2.93658421
NONHSAG049885	3.68123221	3.64589197	3.44658841	3.73537048	3.87496119	3.74624323	3.43411165	3.73566594	3.63544678
NONHSAG049895	4.052518	4.0834009	4.00183056	4.00308325	4.06220103	4.50780045	4.28708349	4.3438035	4.17989062
NONHSAG049902	2.28431375	1.90818289	2.43708408	3.00066456	2.33711194	2.63980665	2.38580807	2.83970839	2.7594551
NONHSAG049904	6.82892395	6.69302944	6.61169433	6.62071099	6.5287717	6.72495407	6.61523682	6.66676392	6.74863191
NONHSAG049930	3.01332136	3.39254452	3.06098977	3.39581033	3.26898464	3.39693768	3.28604795	3.31485544	3.33387168
NONHSAG049931	2.9201144	3.03671243	3.04018045	3.02845166	3.19359493	2.68929603	2.93049058	3.01784303	3.1618915
NONHSAG049936	2.53675949	2.44942588	2.63139447	2.57980615	2.44813063	2.55141303	2.64221722	2.55642449	2.80354467
NONHSAG049939	4.85017864	4.50603552	4.52511246	5.15950227	4.40588617	4.51747614	4.54925062	4.59243041	4.67164279
NONHSAG049977	5.06347945	5.11175457	5.02717027	4.97992472	5.09794975	4.91960586	5.48804131	4.9433227	5.1392423
NONHSAG049980	4.43470303	4.38922974	4.52218995	4.48738777	4.42025558	4.72948677	4.45896879	4.47863407	4.52639408
NONHSAG049984	2.48454722	2.4708994	2.65598788	2.90036223	2.7249921	2.29370351	2.47275255	2.42559824	2.83152224
NONHSAG049989	3.98286443	4.15911008	4.50009971	4.22393518	4.54091249	4.25013694	4.78024665	4.26038594	4.38455819
NONHSAG050013	5.71856282	5.40264545	5.57470105	5.64075632	5.97478555	5.82522943	6.01300105	6.29703615	6.07186746
NONHSAG050025	5.08496873	5.16194063	5.25312272	5.21697211	5.60290262	5.34850219	4.9228036	5.23668362	5.26055532
NONHSAG050057	3.83117506	3.57789535	3.58958413	3.53991942	3.54458443	3.61358171	3.9029616	3.66182826	3.93299613
NONHSAG050083	5.16414969	5.24315123	4.96120834	5.11098564	5.03646547	5.12245967	5.17896008	4.98450678	5.03016851
NONHSAG050088	4.30400048	4.4916805	4.56639187	4.37291658	4.68368917	3.85912366	4.45961166	4.44051952	4.05341258
NONHSAG050093	5.3429225	5.44297938	5.54106242	5.1033644	5.44312701	5.00245647	5.828769	5.38363809	5.48479415
NONHSAG050175	4.28442943	4.59341719	4.49385797	4.01033094	4.58285133	4.16834862	4.46686879	4.22333532	4.38623433
NONHSAG050188	3.22554878	3.13485202	3.2994738	2.84762587	3.0909312	2.95695723	3.09037846	2.83478362	2.94805815
NONHSAG050210	4.81033295	4.79346124	5.00513214	5.12031092	4.73146716	4.9380073	4.90526206	5.21743268	5.04323003
NONHSAG050227	3.32353635	3.33765716	3.32831301	3.23297455	3.41636622	3.32648658	3.24535322	2.94374802	3.0112583
NONHSAG050230	2.95459878	2.93265745	3.10673022	3.00016996	2.78246741	2.90130969	2.92336215	3.00327438	3.00077009
NONHSAG050243	3.64658285	3.48583772	3.41286478	3.83132544	3.69251718	3.39880864	3.42996794	3.97337742	3.48312953
NONHSAG050259	2.38189534	2.41947433	2.23182814	2.57471394	2.40599571	2.3655701	2.43343604	2.29129617	2.26131534
NONHSAG050280	4.70646371	4.9033255	4.84159422	4.82072993	5.10539234	4.82447688	4.66413794	4.57188401	4.89860576
NONHSAG050284	4.18125082	4.43668193	4.07164785	3.71802463	4.27823691	3.8669022	4.35598087	4.12676536	4.19714115
NONHSAG050285	4.32300395	4.28926994	4.53247771	4.05447861	4.50553652	3.94469932	4.25350917	4.1158279	4.28364384
NONHSAG050296	5.01138667	5.11006151	5.25148356	5.03775997	5.09827374	5.13973555	5.3317983	5.07668048	4.99588942
NONHSAG050300	3.66535617	3.52604444	3.75640657	3.47271219	3.87388305	3.15500878	3.71852678	3.54502307	3.79123549
NONHSAG050305	4.69107823	4.17028626	5.29558319	6.107677	5.41346538	5.2915175	5.04865775	6.47496592	5.65262578
NONHSAG050335	4.39084626	4.4279916	4.75757397	4.18556665	4.42333544	4.15902524	4.6664862	4.28930223	4.32247148
NONHSAG050348	2.38715856	2.56568241	2.57190089	2.36275935	2.69363296	2.50148314	2.79019348	2.30332696	2.43930089
NONHSAG050370	3.39401743	3.28061957	2.98970199	3.05836832	3.30557356	2.960511	3.26203453	3.10389537	3.0858491
NONHSAG050372	4.11951034	4.07013608	4.22858487	4.29669231	4.43232489	4.2586858	3.86907571	4.1386056	4.30935007
NONHSAG050397	3.92095286	4.04889899	4.2707084	4.44532653	4.3699638	4.33060663	4.53853274	4.31485794	4.24962314
NONHSAG050419	6.22006157	6.07916298	6.20323713	6.1269912	5.96149411	5.93388527	5.65489336	5.97639065	6.12061916
NONHSAG050438	2.00005235	2.05234719	2.04038778	2.2709728	2.12001528	2.08323633	2.10444173	2.1893753	2.17653373
NONHSAG050440	2.54016862	2.49308013	2.53377126	2.45805424	2.38934064	2.37864712	2.53432689	2.49189882	2.39341794

NONHSAG050454	3.6969766	3.51103548	3.50790482	3.12737167	3.73336647	3.41279047	3.69360231	3.43341058	3.56772126
NONHSAG050461	2.72759758	2.9260274	2.21398011	2.47660452	2.47304735	2.29696564	2.14839243	2.31712468	2.37386911
NONHSAG050473	6.62774389	6.55637373	6.56486332	6.81684409	6.69755132	6.69488738	6.84081522	6.83184852	6.78820575
NONHSAG050503	4.31594846	4.25115096	4.57978951	4.26356959	4.38323248	3.91420755	4.43243604	4.29439716	4.49584045
NONHSAG050531	3.22126666	3.0323176	2.71826697	3.04424614	2.91876384	2.88876824	2.98365661	2.93103558	3.20365578
NONHSAG050576	3.46439358	3.37096151	3.37255143	3.26235208	3.32428855	3.16686011	3.39929935	3.45304889	3.26037324
RP11-48B3.4	5.15976533	5.2083574	6.66525901	7.22326527	6.66608564	6.78276915	6.54614184	7.11331324	7.06642281
NONHSAG050607	3.26965121	3.31130318	3.23568307	3.17442514	3.27669695	3.18558867	3.45941359	3.36606394	3.47319735
NONHSAG050643	2.12650466	2.02226868	2.33368383	2.16629252	2.1821533	2.01655092	2.31568686	2.15469902	2.3011955
NONHSAG050690	8.76748281	8.92133126	9.74489688	10.01881227	10.00196822	9.74511635	9.6618578	10.38117566	9.95054113
NONHSAG050699	5.8811276	5.8271069	5.78461757	5.86553817	5.76780285	5.61942772	5.5135207	5.93458683	5.851645
NONHSAG050714	2.83639443	2.84497351	2.78490135	2.84215159	2.80740059	2.59808593	2.81903765	2.87125532	2.68776055
NONHSAG050718	6.37623762	5.3550638	4.99009429	4.73453934	4.70183021	4.56444428	4.93906142	4.89707198	4.70620852
NONHSAG050752	2.19674182	2.11695003	2.49395804	2.85437468	2.44135139	2.59438112	2.53790265	2.33801603	2.59595877
NONHSAG050804	6.82453146	6.70713088	6.04363778	6.2721481	6.05858838	6.1893967	5.95920472	6.5352665	6.53125818
NONHSAG050806	5.74534274	5.53454619	5.98562215	5.76403932	5.57881932	5.70214496	5.73947676	5.74091529	5.85253268
NONHSAG050815	2.52787835	2.54926699	2.57835735	2.69501835	2.44317902	2.79921152	2.62354655	2.60368376	2.61535608
NONHSAG050829	2.47460465	2.58979123	2.47266246	2.51609937	2.63019121	2.50548555	2.73791163	2.59258254	2.41146757
NONHSAG050841	3.72400456	3.82508272	3.83882993	3.78841184	4.02788267	4.05086392	4.0743051	3.88994531	3.80317313
NONHSAG050865	7.12375237	7.16230465	7.8603727	7.87889349	7.69473598	7.88361501	7.65596967	7.81938659	7.71911791
NONHSAG050866	5.19923511	5.59544998	6.26896408	5.99859463	5.80056336	6.22916712	5.80723322	5.7670741	5.95269946
NONHSAG050902	3.50918777	3.89322683	3.83054821	3.79110057	3.86595414	3.76087299	4.01397459	3.71279565	3.53361709
NONHSAG050903	7.0507564	7.14602229	7.47828483	7.6825301	7.38197994	7.68953574	7.38513097	7.47302969	7.46835384
NONHSAG050913	5.08922326	5.36463858	5.57660562	5.43384827	5.4627412	5.34957095	5.80478989	5.28861749	5.65536825
NONHSAG050914	3.04318132	3.23778174	3.47113365	3.41704576	3.03512361	3.15380295	3.38313925	3.20170422	3.19592689
NONHSAG050915	4.26426472	4.47558658	4.61654142	4.3742584	4.43747977	4.59346234	4.66583558	4.56914127	4.8657636
NONHSAG050921	3.0299765	2.96255944	3.06658302	2.96127503	2.83624087	2.69351618	3.13851169	2.96316807	3.05743182
NONHSAG050966	4.61151372	4.18628042	5.20273613	5.09550653	4.97070507	5.30196262	5.24866294	5.40141169	5.33629285
NONHSAG050969	2.77154319	2.95776461	2.89205695	2.79833748	2.9910204	3.00760221	2.87075378	3.16074054	3.0131405
NONHSAG050970	3.24204866	3.07277459	3.29582004	3.06458887	3.26711265	3.12008792	3.32090723	3.37946546	3.40714805
NONHSAG050978	3.93898428	3.87429073	3.91095051	3.6137335	3.76625319	3.53067994	3.63057401	3.44584005	3.8628051
NONHSAG050982	6.14270607	6.18090439	6.15643218	6.16829212	5.89072773	6.42335421	6.10345551	6.3049537	6.60542111
NONHSAG051053	4.20085962	4.18892013	4.11418995	3.932033	3.86696061	3.89597307	4.09327914	3.97630914	4.26298278
NONHSAG051068	1.99169887	2.08976459	2.17425275	2.26456496	2.29795115	2.11129474	1.97043157	2.090905	2.12764956
NONHSAG051082	5.58465657	5.54315979	5.39268073	6.1504372	5.8636346	5.84639191	5.43552806	6.78795465	6.40870548
NONHSAG051104	4.44864678	4.44567858	4.81345096	4.19001286	4.55559174	4.28337977	4.79599166	4.48878155	4.41130848
NONHSAG051105	3.41109008	3.41615558	3.84633356	3.97479393	3.93769047	3.24395174	3.31413505	3.88373111	3.9525999
NONHSAG051129	3.91900384	3.26716463	3.22146642	3.08035232	3.19513368	3.11931053	3.12300641	3.25584079	3.26517257
NONHSAG051137	3.94478872	4.18968671	4.1173198	3.96845959	3.99219782	3.75371929	4.01806439	4.15634887	3.98023909
NONHSAG051145	3.89357923	3.92489251	3.95529509	3.85199523	4.47001535	3.6668285	4.25076593	4.19404517	4.03714838
NONHSAG051156	2.6401629	2.3743666	2.57933449	2.28739751	2.35132506	2.3029093	2.34656149	2.43497665	2.42694825
NONHSAG051181	3.29345646	3.49029203	3.51352566	3.10571647	3.43479425	3.33177517	3.53154123	3.4289325	3.44084946
NONHSAG051194	3.02109066	3.07703849	3.04543452	2.75083505	3.07121394	2.94727109	3.22832539	2.92640175	3.2134536
NONHSAG051196	5.07108278	4.81510087	4.98533211	5.34583066	5.03868251	5.12677985	4.99103699	5.23751432	5.35993993
NONHSAG051205	4.19919387	3.88545739	4.32318584	3.81301039	4.04946523	4.13605275	5.07305286	4.11178836	4.08007479
NONHSAG051207	2.45331882	2.91560538	2.70471413	2.61996925	2.83155714	3.03523037	3.04697559	2.42542928	2.7311904
NONHSAG051233	2.26322749	2.21980536	2.66879782	2.10215881	2.48488759	2.18853649	2.50353022	2.43787411	2.63584935
NONHSAG051237	2.63090175	2.54893704	2.31186172	2.41591271	2.69592642	2.36062315	2.67456945	2.73918319	2.69241466
NONHSAG051255	3.41057069	3.44178596	3.41057069	3.49907396	3.39975117	3.25217639	3.36802034	3.41057069	3.36619064
NONHSAG051258	5.13029402	4.85098736	4.9828232	5.03722946	5.01138364	4.79134223	4.88208339	4.92634617	4.88499699
NONHSAG051259	3.81677308	3.43069229	3.4345686	3.26434357	3.19737381	3.19253864	3.40558586	3.69486963	3.56913681
NONHSAG051263	3.50721274	3.23153123	3.35144729	3.0539219	3.07509512	3.0934815	3.42929834	3.13369446	3.19703468
NONHSAG051266	4.16138085	4.20530904	4.2374992	4.23518561	4.32346101	4.04050406	4.15179997	4.23684054	4.27040072
NONHSAG051273	2.58274726	2.84807576	2.68239944	2.59146584	2.6309585	2.69905427	2.889582	2.63239306	2.63073958
NONHSAG051275	2.43802592	2.45446579	2.91047841	2.58824363	2.41119269	2.32594066	2.68534728	2.42055829	2.53694141
NONHSAG051297	2.54112295	2.6805319	2.46941451	2.57684983	2.49020784	2.58597126	2.53624028	2.77484952	2.74232354
NONHSAG051305	2.95301844	3.23778612	3.30027496	3.16659189	3.22694905	2.98490577	3.19997603	2.9548295	3.0952954
NONHSAG051327	5.19923511	5.20034271	5.21217533	5.1902259	5.24486143	5.46242358	5.51758709	5.29471586	5.35059833
NONHSAG051334	2.92777036	2.89820483	3.09982953	3.02617894	3.03326966	2.89868422	2.90837091	2.79122492	2.53162185
NONHSAG051339	5.05842769	5.19594782	5.21390943	5.08044806	5.20673453	4.95376264	4.98050303	5.26272511	5.15594651
NONHSAG051345	3.20211338	3.26494608	3.21370085	3.22661969	3.25458832	3.2228776	3.50444188	3.23801587	3.19816261
NONHSAG051356	4.4755073	4.13961539	4.51592733	4.3904422	4.33915701	4.17982939	4.48106867	4.5200168	4.50418836
NONHSAG051375	3.38335624	3.11303251	3.05261927	3.19062533	3.19061126	3.01095848	2.90929071	3.17934876	3.00077009
NONHSAG051392	3.26155054	3.29861668	3.54419361	3.35631349	3.42216755	3.40092613	3.41212174	3.1777739	3.4562346
NONHSAG051400	6.94301809	6.88071565	6.65205087	6.79605351	6.68338176	6.62186213	6.6388646	6.69905901	6.78111574
NONHSAG051424	6.10388997	6.02910304	6.37532618	5.86794427	6.08987703	5.86837814	6.19926377	6.0509482	6.12327139
NONHSAG051435	4.55150748	4.24037174	4.28747813	4.24623828	4.56003671	4.09001704	4.50098268	4.37600368	4.31740254
NONHSAG051445	5.63565371	5.8299224	5.81828963	5.43160086	5.9061724	5.55056194	5.77178799	5.72950432	5.54365902
NONHSAG051446	4.1111718	4.68001387	4.21450849	4.46546879	4.58691092	4.55445647	4.73694458	4.78342565	4.59850507
NONHSAG051464	5.05213643	4.97276363	5.12115512	4.61129852	4.86529207	4.64456416	5.15815661	4.74601313	4.76612999
NONHSAG051484	5.34136637	5.36478991	5.57595286	5.75460451	5.56875135	5.60403393	5.555238	5.60691846	5.65047852

NONHSAG051497	5.28267861	5.23825749	5.22908766	5.19046289	5.19518438	4.78172455	5.15365482	5.10566273	5.05527709
NONHSAG051574	4.81959872	4.62027968	4.85840593	5.11076598	5.02341299	4.71116184	4.80381906	4.69345343	4.84333874
NONHSAG051638	7.20751992	7.38121398	7.08489082	7.42091215	7.31164538	7.15522748	7.15862319	7.24253402	7.23795638
NONHSAG051650	3.54627837	3.07286277	3.25652578	3.32976315	3.40268584	3.10646949	3.69434218	3.38246677	3.38215464
NONHSAG051658	5.58960859	5.63568569	5.42652594	5.15958345	5.3657285	4.97277309	5.65698729	5.38693995	5.4712198
NONHSAG051661	2.74237343	2.84686142	2.9171234	2.82568507	2.89237817	2.71995158	2.86018336	2.89799945	2.79836443
NONHSAG051677	4.77893099	4.76897771	4.93163728	4.79444522	4.74796649	4.89500853	5.02106435	4.95696279	4.71349774
NONHSAG051728	3.62644805	3.30159626	3.50829175	3.65112127	3.68323976	3.39845115	3.83594885	3.82472171	3.86157364
NONHSAG051730	3.42372161	3.22978239	3.39479088	2.98160388	3.17167371	2.99626363	3.55951972	3.16072258	3.35630171
NONHSAG051770	2.59302144	2.52274554	2.48715131	2.31371901	2.62800653	2.56684137	2.66614548	2.62974414	2.47685686
NONHSAG051806	2.98659152	2.78549011	2.93310471	3.06724668	2.91021787	2.84383761	2.98509944	3.05462308	2.68473126
NONHSAG051813	4.5019674	4.28879819	5.68368468	6.07451291	5.80142079	5.26974891	5.20937038	5.47094762	5.4742359
NONHSAG051826	2.6837399	2.83145728	2.73167305	2.70510897	2.82059917	2.59556715	3.01413996	2.68289336	2.8218733
NONHSAG051866	2.15853261	2.53468602	2.52225208	2.44111915	2.44813063	2.29689303	2.38455344	2.42542928	2.31725335
NONHSAG051885	2.5999484	2.36517297	2.44813063	2.45799942	2.61905422	2.33015363	2.89617465	2.28303244	2.60700222
NONHSAG051943	3.08614037	3.19992297	3.44849806	2.99040355	3.16380258	3.20394368	3.20256514	3.05917981	3.14771108
NONHSAG051945	2.59228152	2.45607882	2.5205691	2.8481956	2.53081387	2.46854131	2.81704522	2.3325623	2.56412537
NONHSAG051962	4.26526616	3.92489251	4.62359237	3.88777393	4.61094438	4.05427057	4.45173867	4.2082591	4.41833804
NONHSAG051968	2.33219311	2.42544373	2.22089701	2.2222218	2.50461607	2.18649907	2.25172499	2.08022225	2.37386911
NONHSAG051985	2.65240146	2.77508066	2.77155321	2.7941798	2.66890513	2.5585851	2.78413662	2.69866414	2.81713649
NONHSAG052010	3.29539122	2.91560538	2.76310311	2.9466602	3.11571782	3.0833195	3.28604795	3.17934876	3.20881472
NONHSAG052012	6.54287897	6.60013606	6.62945717	6.48405055	6.64674613	6.50281812	6.55375796	6.52482338	6.64169007
NONHSAG052042	3.28565216	3.13762472	3.10675107	3.35549991	3.04609291	2.89229109	3.76797754	3.28121202	3.3002141
NONHSAG052090	2.69056519	2.69995299	3.0340802	2.68998327	2.77683658	2.62310765	2.76032477	2.73951907	2.64213488
NONHSAG052112	3.89629552	3.66392453	3.9040927	3.99664718	3.80415979	3.87438483	3.96717011	3.96583206	3.66739727
NONHSAG052118	4.34176127	4.55567699	4.51787388	4.12219874	4.55374593	4.77126961	4.17717663	4.84929154	4.5168462
NONHSAG052125	8.50625778	8.41008212	8.83051308	8.9788089	8.95058996	9.09950213	8.932758	8.75447221	8.76810411
NONHSAG052126	3.24645403	3.38304909	3.23229341	3.80242188	3.27211461	3.57742553	3.32751419	3.74913329	3.49474025
NONHSAG052157	3.71981109	4.07719699	3.61425229	3.89431835	3.93769047	3.76237951	4.05234961	3.74515314	3.84927824
NONHSAG052162	5.17173271	4.81217675	5.14338542	5.63028265	5.5450478	5.10001388	5.04633973	5.90336421	5.74099705
NONHSAG052178	6.27143742	6.21833424	6.40192486	6.71342409	6.1877045	6.50141957	6.29877149	6.40516761	6.39390485
NONHSAG052186	6.76652259	6.77354987	6.4982591	6.78346236	6.56855907	6.55901522	6.46917448	6.61417611	6.50252617
NONHSAG052188	4.43980597	4.00315589	4.46200949	4.03575946	4.45984973	4.25467139	4.68897822	4.29592084	4.41094618
NONHSAG052409	5.67174875	5.96542587	5.76112201	5.88807629	5.8481077	6.185311	5.96863409	5.84884459	5.83755851
NONHSAG052414	4.14017203	4.52842701	4.61864927	4.82580101	5.2425093	4.95440763	4.61829241	4.87059032	4.63510175
NONHSAG052449	7.40303121	7.16238506	7.08272369	7.42072556	7.39255935	7.45216976	7.06932467	7.91651551	7.63251964
NONHSAG052499	3.71276955	3.87769685	3.98710828	3.67790301	3.78683094	4.09607232	3.75648642	3.66684444	3.99377453
NONHSAG052508	4.80751123	4.57400945	4.93411957	5.39097179	5.06189773	5.13037694	5.02495306	5.08162631	5.38498946
NONHSAG052530	3.40236605	3.32178009	3.44898352	3.37650562	3.41245746	3.32544195	3.62781093	3.4162378	3.43294735
NONHSAG052547	5.70577569	5.49895031	6.13370075	5.95837904	5.85345611	5.88343594	5.67835189	5.73037618	5.87535826
NONHSAG052583	2.67278973	2.43549078	2.70795489	2.19205597	2.39932854	2.54407265	2.69809146	2.49094156	2.52718156
NONHSAG052586	2.15693703	2.15920906	2.26243244	2.18670851	2.04451317	2.22686487	2.43232155	2.16044773	2.14822066
NONHSAG052609	2.49342055	3.65241576	3.02364899	3.63845324	3.13764832	2.70126308	3.17834987	3.00253723	3.02534729
NONHSAG052620	5.4627412	5.42438427	5.57053011	5.90251557	5.5207605	5.51353283	5.56211464	5.66430622	5.38770669
NONHSAG052621	3.57934969	3.84151235	3.57698794	3.46317602	3.78928695	3.47830968	3.43875939	3.63553891	3.73228315
NONHSAG052627	5.73674816	5.78919691	5.80776655	6.31765786	5.83694325	6.038144	5.85214771	6.05324915	5.99808202
NONHSAG052636	4.08275682	4.6342592	4.58473201	4.35210793	4.71622465	4.6490369	4.5466023	4.19908651	4.4084973
NONHSAG052644	3.70976859	3.59612959	3.4015661	3.28361998	3.48844542	3.72081898	3.68048685	3.4512828	3.56849966
NONHSAG052726	4.95026966	5.05609084	5.16116944	4.79654349	5.1380143	4.80490725	5.07017311	4.88120658	5.02558618
NONHSAG052734	3.66385106	3.66790103	3.86818438	3.43586085	3.79000936	3.37679131	3.87552849	3.59970258	3.77502313
NONHSAG052752	5.40599448	5.65420566	5.77246881	6.04167533	5.9179472	6.04231919	5.36041016	5.88971985	6.16842
NONHSAG052756	2.84899987	2.80697789	2.97488716	2.78595127	2.80792305	2.71836162	2.95868667	2.68493525	2.90688523
NONHSAG052760	4.10754464	4.58121579	4.70110006	5.15950227	4.56161885	5.06643541	4.59181823	4.80543196	4.82374297
NONHSAG052772	6.2814993	6.43930004	6.55169212	6.16764039	6.3974308	6.00385647	6.60847156	6.07802684	6.31396765
NONHSAG052776	5.45504813	5.44861234	5.40369531	5.37090991	5.45771091	5.30341634	5.59805332	5.35200902	5.45945297
NONHSAG052817	4.49241295	4.66029305	4.69188256	4.68722496	4.52021804	4.20264775	4.76964825	4.41175508	4.18449163
NONHSAG052818	3.31658394	2.67367068	2.9807882	3.08254562	3.10770425	2.76389486	3.07814867	3.3750193	3.04847532
NONHSAG052837	5.90449656	5.76001555	5.85162092	6.15078286	6.09143053	5.86615548	6.01033485	6.23533038	6.08464222
NONHSAG052879	4.02096774	4.49767488	4.39557208	4.54508723	4.58018496	4.46219569	4.43199237	4.54912734	4.45724694
NONHSAG052880	5.28571706	5.29728345	5.66108166	5.7431118	5.58199212	5.81416255	5.33398251	5.61374606	5.44925933
NONHSAG052885	4.92799094	4.72404206	4.73888558	4.53926798	4.72854484	4.58261759	4.90306393	4.50608068	4.54366498
NONHSAG052886	4.47906619	4.5744813	4.55967764	4.36166266	4.36404471	4.38772078	4.86477698	4.42169291	4.53303369
NONHSAG052890	6.1769436	5.89327407	6.01597028	6.29826345	6.1282324	5.99224187	6.16860648	6.28959509	6.12503171
NONHSAG052915	6.87810525	6.91379429	6.28364652	6.30415125	6.07603345	5.94487055	6.04576306	6.10746895	6.26072355
NONHSAG052978	3.29473679	3.24766123	3.54144051	3.48849561	3.40138065	3.45175365	3.41154846	3.36144393	3.38790809
NONHSAG052998	2.56545169	3.17282105	2.59342957	2.52440447	2.67795631	2.61202654	2.44284722	2.95941761	2.88716619
NONHSAG053000	2.56544204	2.65279999	2.81345716	2.66735663	2.67777123	2.58881778	2.51832161	2.48385872	2.74232354
NONHSAG053032	3.57925134	3.82522121	3.84638054	3.93265715	4.0878559	3.81563586	3.75120822	3.94423905	3.88038059
NONHSAG053081	2.37460446	2.20893656	2.27795209	2.55197213	2.41737857	2.42407419	2.62287705	2.40574469	2.55197213
NONHSAG053118	2.33033625	2.27685537	2.32797247	2.27094192	2.35878263	2.47688708	2.43618079	2.30998063	2.27160313
NONHSAG053119	2.90900475	2.66715197	2.64933599	2.3967605	2.66181728	2.43565176	2.91027952	2.4992265	2.49387013

NONHSAG053167	4.13834527	4.08295277	3.97058603	4.00509533	3.74752117	3.96256782	3.95451605	3.99373181	4.18158326
NONHSAG053168	2.37528304	2.4829339	2.59739082	2.3305479	2.6123035	2.53095242	2.85886015	2.28245003	2.60890639
NONHSAG053205	4.68939076	4.47434438	5.21743268	5.41097394	5.11508065	5.03551717	5.26316578	4.63261551	5.04076415
NONHSAG053209	2.26339531	2.41507792	2.33368383	2.34766539	2.20957769	2.24764267	2.16924786	2.27257831	2.31849892
NONHSAG053219	6.96480156	6.60765262	6.83598307	6.73434109	6.75752184	6.59444931	6.62230103	6.61536647	6.79488637
NONHSAG053240	3.45019181	3.2268134	3.63375217	3.63918137	3.48450387	3.45459308	3.78669888	3.38679043	3.35652023
NONHSAG053246	5.11088115	5.05683809	4.74520696	4.3103384	4.59267416	4.39940722	4.54383522	4.57200745	4.50720045
NONHSAG053259	4.09023616	3.65990915	3.91478695	3.41384572	3.56812975	3.5779058	4.15885008	3.7459727	3.91074734
NONHSAG053273	3.62710635	3.81244148	3.57381048	3.36694175	3.96467923	3.96973876	3.77518232	3.78767192	4.01212742
NONHSAG053274	2.88736265	2.79360455	2.96074152	2.92098425	2.73880213	2.89655107	2.82871404	2.71873042	2.8753735
NONHSAG053293	4.03003057	4.08793325	3.7456671	3.957035	4.14354364	4.12627158	4.51802194	3.78650551	4.12873581
NONHSAG053296	3.99172927	4.15915973	3.99152349	4.23187653	4.31723945	4.1028407	4.1577952	4.19860816	4.18667306
NONHSAG053299	3.64527961	3.4245002	3.50969466	3.56683594	3.35126799	3.38486679	3.46687498	3.18404342	3.51129589
NONHSAG053306	5.12762209	5.19022456	5.44712996	5.22796487	5.42479951	5.02082098	5.1593239	5.23754361	5.32458401
NONHSAG053322	5.45744025	5.44176771	5.48568278	5.01875383	5.49004823	5.14375792	5.40940944	5.19991372	5.56727605
NONHSAG053329	3.17352368	2.83124816	2.79824786	2.76519536	2.92438232	2.78682277	2.67758557	2.64204267	2.75433274
NONHSAG053359	5.17827966	4.98024675	5.59070154	5.58626326	5.52044579	5.52739427	5.7959812	5.58096907	5.74259113
NONHSAG053361	4.48774852	4.56411263	4.35518146	4.49357952	4.49040285	4.29926315	4.75376873	4.36514161	4.6441538
NONHSAG053376	6.27544274	6.27227595	6.19442903	6.21593022	6.28601745	6.22546093	6.3141698	6.2328265	6.20499329
NONHSAG053377	6.64624959	6.60550779	6.4360315	6.27265577	6.47225667	6.41338672	6.51831916	6.40720017	6.40757316
NONHSAG053380	5.75248207	5.97339788	5.6127185	6.13753677	6.12378874	5.85229191	5.77832737	6.02756962	6.00519093
NONHSAG053392	2.90742996	2.74324241	2.86560912	2.77241994	2.77680583	2.67344184	2.70549957	2.64397732	2.68571316
NONHSAG053406	4.65115607	4.66156382	5.05835646	4.89453722	4.83182551	5.06066936	5.04904357	5.01690181	5.04665905
NONHSAG053407	4.60990845	4.37905996	4.64700698	4.5023039	4.94332973	4.44241306	4.52653162	4.70166496	4.42998551
NONHSAG053417	4.17805867	4.39924471	4.19171905	4.24542595	4.0354015	4.30427939	4.6844508	4.69054988	4.420836
NONHSAG053440	4.09846567	3.94129785	4.22800273	3.91881948	4.07256522	3.69158264	4.13279071	4.08053052	4.17155215
NONHSAG053450	3.27428087	3.1037899	3.52354959	3.2570573	3.33899096	3.1551094	3.48669197	3.28732531	3.56845821
NONHSAG053454	3.09804204	3.00162569	3.00043465	3.2354608	3.08138163	2.9903721	3.08881879	2.86392395	3.34732158
NONHSAG053462	3.31342884	3.50762638	3.64692281	3.5234246	3.69112404	3.7655223	3.50811919	3.43781031	3.5740183
NONHSAG053469	3.97397086	3.65174379	3.56240907	3.51483685	3.72258134	3.37366174	3.33434613	3.64376574	3.50737861
NONHSAG053552	6.11797692	6.33443739	6.24945787	6.14245751	6.3350126	6.34990487	5.98622032	6.43218473	6.25469396
NONHSAG053577	6.53827662	5.95808131	7.44293294	7.71370407	7.15774721	7.41024371	7.42772184	7.27263114	7.73286794
NONHSAG053585	4.72697032	4.98404117	4.80051218	4.78402418	4.81207993	4.79562331	4.99391936	4.95222422	4.69488138
NONHSAG053586	6.56399856	6.42523955	7.20960985	7.45556741	6.98562547	7.37370707	7.14049882	7.43832356	7.48908986
NONHSAG053589	6.35783513	6.34025979	6.62841986	5.96818424	6.6649086	6.22570194	6.63967218	6.2825957	6.36216162
NONHSAG053591	5.32140199	5.36956666	5.56450679	5.35761876	5.57425397	5.29165019	5.58631989	5.2649346	5.39807257
NONHSAG053607	4.59991232	4.44024588	4.2432567	4.12515023	4.04489428	4.10427116	4.2782316	3.92353212	4.15048169
NONHSAG053614	5.42352352	5.62242208	5.63727128	5.59881105	5.77383573	5.43433806	5.44980935	5.57843979	5.43711623
NONHSAG053675	5.46754766	5.35570246	5.38277651	5.00282564	5.16523283	5.22101882	5.49737526	5.28198499	5.17014796
NONHSAG053719	2.68264122	2.75726919	2.67419056	2.80470934	2.85398624	2.36043895	2.89432189	2.68375945	2.64892184
NONHSAG053722	3.51496855	3.42186006	3.56672634	3.6263801	3.73509409	3.42353836	3.47722993	3.50841077	3.32958148
NONHSAG053746	4.11933493	3.92489251	4.05416877	4.21219469	3.96400135	4.09033923	4.11516411	4.23578228	4.25202795
NONHSAG053751	3.78770763	3.85960055	4.13183978	3.72969945	3.76754475	3.84623335	4.19925635	3.81919074	3.69458737
NONHSAG053784	5.55627497	5.61105828	5.63052734	5.6883823	5.656331	5.64291074	5.71336382	5.65532845	5.4849251
NONHSAG053801	4.00599412	3.89581524	4.42452359	3.87311241	4.08078405	4.04379919	4.2188848	4.21885367	4.03853809
NONHSAG053821	2.74523478	2.99828921	3.02769027	3.11610206	2.89604243	2.79775191	2.98063781	2.72682493	2.93238603
NONHSAG053863	3.86659651	4.04885101	3.99505639	3.81210425	4.06183667	3.73317625	4.05016496	3.82184121	3.84844846
NONHSAG053877	4.6917183	4.72460628	5.05186612	4.93433513	4.81975169	4.85294725	4.927947	4.94740608	5.00979193
NONHSAG053892	6.01952934	5.5172982	4.94874887	5.48043608	5.14118989	4.72132574	5.44163954	5.09388287	4.69311578
NONHSAG053893	13.88169326	13.90615955	13.91570503	13.88859316	13.98099359	13.93388629	14.0171104	13.92953283	13.93425867
NONHSAG053895	14.21724952	14.13432366	14.17862981	14.10441808	14.26787275	14.26724413	14.27411517	14.23876124	14.2059758
NONHSAG053900	2.49767597	2.38217293	2.52396732	2.85349971	2.51473482	2.83744747	2.62487586	2.51321107	2.53768246
NONHSAG053985	2.85449442	2.91158543	2.8977894	2.82475073	3.08301018	2.89442242	3.04697559	2.79927669	2.865574
NONHSAG054015	2.34620546	2.30793454	2.53531345	2.42993087	2.44746197	2.12419569	2.40947757	2.38301083	2.29944608
NONHSAG054016	2.859581	2.54054684	2.54292961	2.59665716	2.6807197	2.66911812	2.84887467	2.75556216	2.6047483
NONHSAG054030	3.14311084	2.79850286	2.66971994	3.01525011	3.13948535	2.73705477	2.96910444	2.80598473	2.79197131
NONHSAG054071	2.7740704	2.80921795	2.63057456	2.71638803	2.72661185	2.95795493	2.88855434	2.92409548	2.8885763
NONHSAG054103	3.10962957	3.08666779	3.02480212	2.95106336	3.19775825	2.936944	3.15599777	3.20383042	3.17333347
NONHSAG054109	2.62255377	2.63773462	2.5810097	2.62902128	2.78246741	2.61425046	2.67862609	2.70093807	2.66665717
NONHSAG054140	2.72097419	2.64019643	2.81644613	2.95847781	2.90704227	3.17154128	2.72991537	2.86802672	2.80489488
NONHSAG054179	4.23253339	4.23108351	4.35944576	3.83205164	4.31720259	4.33324333	4.52705472	4.17462659	4.24763083
NONHSAG054188	3.34191927	3.30429477	3.359304	3.13743407	3.39771719	3.0704	3.57238575	3.32357437	3.51616138
NONHSAG054199	5.93827366	6.09674201	6.0276147	5.87784068	6.16516453	5.90436103	6.43835702	5.95275493	6.05682209
NONHSAG054326	2.80782257	2.66560183	2.77222501	2.83548309	2.951889	2.64314285	3.11732153	2.74407128	2.6848925
NONHSAG054344	2.05193132	2.07094089	2.11480924	2.19183673	1.98174424	2.11945354	2.16456254	2.26006038	2.08204678
NONHSAG054345	3.36052939	3.41418069	3.27199528	3.37260988	3.47338171	3.33361273	3.37259431	3.39506118	3.33589271
NONHSAG054445	3.65654321	3.91394064	3.68015309	3.54091899	3.68785814	3.65765002	3.70984054	3.83769315	3.81694428
NONHSAG054462	4.60728803	4.59249792	4.50724181	4.17863119	4.80832559	4.55445647	4.5094477	4.60229516	4.59619953
NONHSAG054468	4.63663837	4.4694443	4.70629656	4.77707859	4.84580371	4.75105779	4.68964409	4.96116693	4.70307834
NONHSAG054478	2.69872853	2.45994549	2.88034675	2.39497108	2.55276732	2.41703551	2.38555188	2.86392395	2.66073622
NONHSAG054549	5.77175578	5.56329232	5.91077576	6.28008122	6.10372813	6.0773972	5.74658175	6.23113692	5.99871698

NONHSAG054576	5.57393624	5.39452953	5.65453497	5.65751739	5.68331594	5.59690397	5.87630642	5.65522914	5.86660124
NONHSAG054653	5.92210147	5.75814364	5.69983306	5.87724041	6.07661566	5.55153825	5.84031932	5.82383102	5.64418696
NONHSAG054655	4.3439131	4.46031134	4.48369928	4.61174397	4.36971781	4.35367494	4.33762658	4.21640767	4.47416805
NONHSAG054664	3.05767836	3.0043212	3.15275563	2.97064525	2.8929976	2.91616844	2.92730898	3.17761063	3.34231599
NONHSAG054671	3.00601132	2.94283957	3.10596645	3.23113492	2.99326454	2.97710571	2.86294627	3.51043535	2.95841651
NONHSAG054679	2.68115202	2.71160637	2.67986639	2.83948575	2.49145814	2.61466549	2.64607893	2.57452332	2.76194703
NONHSAG054729	6.40504853	6.61034439	5.88449464	6.2419116	6.076975	6.25630172	6.27381353	6.21066765	5.90102685
NONHSAG054754	3.30200834	4.15124845	4.11922322	4.03960184	3.94680121	4.0145255	4.07483176	4.08125739	3.9851408
NONHSAG054784	6.13266848	6.29376672	6.07826295	6.50081697	6.26696358	6.38471283	6.02126764	6.83733749	6.54549095
NONHSAG054798	5.31707481	5.16403554	6.02190724	6.45988329	5.8997899	6.22127055	5.97676812	6.99153025	6.62169061
NONHSAG054804	6.86232552	6.7680828	7.43183033	8.0189134	7.79709565	7.81239043	7.45384961	8.49561136	8.11099214
NONHSAG054869	4.11581702	4.20254904	3.82326966	3.98828637	3.81438759	3.90702534	3.62071275	3.8202708	3.76191226
NONHSAG054870	6.68951129	6.57634433	6.75412122	6.83684535	6.82544259	6.86279022	6.87579027	6.82792612	6.77242249
NONHSAG054897	2.72471375	2.5139528	2.5081571	2.27971321	2.4599484	2.45689357	2.92214146	2.43249412	2.42129526
NONHSAG054929	3.13056832	3.25883677	3.33305823	3.29495153	3.44412738	2.98188415	3.16899189	3.22088799	3.37736439
NONHSAG054954	2.91136709	2.90073981	3.12196339	3.19367199	3.3390018	3.10317196	3.63638929	3.16009865	3.20781507
NONHSAG054958	3.68143125	3.76562468	3.72546152	3.60302828	3.74848279	3.58327889	3.90450683	3.54498044	3.93338566
NONHSAG054963	4.66927561	4.79904684	4.75786277	4.90776246	4.98243171	4.68026534	4.6738435	5.14206603	4.94883411
NONHSAG055008	2.4097478	2.61728699	2.60451896	2.67766702	2.5715268	2.40990745	2.60712272	2.46443799	2.49174464
NONHSAG055019	2.92098549	2.91560538	2.83121904	2.95496954	3.00495346	2.94374802	2.61135587	2.94374802	3.00077009
NONHSAG055021	3.52751387	3.49984622	3.72366911	3.5917495	3.60644208	3.62642517	3.78307167	3.70715671	3.57202028
NONHSAG055050	3.08850764	2.92464669	2.94118301	3.01683074	2.97376363	3.02345724	2.96588375	2.98575849	3.18787423
NONHSAG055086	2.86803065	2.81076498	2.70015836	3.01627081	2.66369152	2.70677049	2.71038661	2.75328632	2.65985
NONHSAG055105	2.51383016	2.30678135	2.13050793	2.39297459	2.40729436	2.40115151	2.35477996	2.34808436	2.43567168
NONHSAG055108	4.93308509	4.82454754	4.58955672	4.41518332	4.71964704	4.26893467	5.13964858	4.54883762	4.5465208
NONHSAG055153	2.95674963	2.88814366	3.03669222	2.92306402	3.15362083	3.06827774	3.24738902	3.00182364	3.05294136
NONHSAG055204	3.47737311	3.18400878	3.31805989	3.9183462	3.40651169	3.38007364	3.48036029	3.67276979	3.48220277
NONHSAG055266	4.84534322	5.11263906	5.05072192	5.14150572	5.0485058	5.00735685	5.30837611	4.79260542	5.06600802
NONHSAG055280	2.62722838	2.66876635	2.62034295	2.64413227	2.49647839	2.24140289	2.71111884	2.59173387	2.44518096
NONHSAG055289	5.67666593	5.72909452	5.77911397	5.62167636	5.64181332	5.68598831	5.59770323	5.68261557	5.86920772
NONHSAG055301	3.65536098	3.55053286	3.51103014	3.51799219	3.35370888	3.25196972	3.62831087	3.48054849	3.8317902
NONHSAG055339	5.19468176	5.10977834	4.90611085	5.2084805	5.20844141	4.92887931	5.06812655	5.00155993	5.15556706
NONHSAG055350	6.35242914	6.2307486	6.0864896	6.08322327	5.91269811	5.67802356	6.02289824	5.75378231	6.03001846
NONHSAG055367	3.50664865	3.32239031	3.74660661	3.38780404	3.48769617	3.12734319	3.8164322	3.34419427	3.50113084
NONHSAG055371	4.76416505	4.90849846	5.73999417	6.37042546	5.52240458	6.02579083	5.30607292	5.72737361	5.94043859
NONHSAG055384	4.58983305	4.60112702	4.77446931	4.81555088	4.85919469	4.7714136	5.07247678	5.4413721	4.90324619
NONHSAG055388	2.86172556	2.99469438	3.85279343	3.36694175	3.21537553	3.77316686	3.29450799	3.6317509	3.53518156
NONHSAG055401	2.68930866	2.8882468	2.70172863	2.72040509	2.71062947	2.63059185	2.83772055	2.67216079	2.5239024
NONHSAG055405	3.51520965	3.59020038	3.76644521	3.9215557	3.74931366	4.0386574	3.74678786	4.01696765	3.79770652
NONHSAG055409	4.28367285	4.34648055	3.91793281	3.56013381	4.32973976	3.60424074	3.86978108	4.07030578	4.00273468
NONHSAG055436	5.18439551	4.87158608	5.37505367	5.62132172	5.6569457	5.20964297	5.30822198	5.72005276	5.30657836
NONHSAG055443	2.5302832	2.55289669	2.65632279	3.45555307	2.87107873	3.02268342	2.59893299	2.55047924	2.61540069
NONHSAG055445	11.31918959	11.32336424	11.24511311	11.42413376	11.51272947	11.51289946	11.41935185	11.43874659	11.41612653
NONHSAG055446	2.48454722	2.53881586	2.6326599	2.53676276	2.48570786	2.65035458	2.5690467	2.50868882	2.58101623
NONHSAG055449	6.03146103	6.11085903	6.18152839	6.0766048	6.54357371	6.20985924	6.1732275	6.14500858	5.97502612
NONHSAG055454	2.51118444	2.51115209	2.64848566	2.70988998	2.49564966	2.44848329	2.61463559	2.49766559	2.66899378
NONHSAG055455	2.96731728	2.61652607	2.73572359	2.85916766	2.73051132	3.02284094	2.69127056	2.82230027	2.59067533
NONHSAG055463	3.04017089	3.25078774	2.81185392	3.25362355	3.25975583	3.38314124	3.13443457	2.91788426	2.85559448
NONHSAG055544	5.11005143	5.25669627	5.34752306	5.10581915	5.51068711	5.13700027	5.35725036	4.98736869	5.24765327
NONHSAG055550	2.46831108	2.6634016	2.60882896	2.70897882	2.57912853	2.61510521	2.69636837	2.57639489	2.38850562
NONHSAG055620	5.74916336	5.68183956	5.50811714	5.58867402	5.54447715	5.62158113	5.23736158	5.5217758	5.5603069
NONHSAG055690	3.76996969	3.67866614	3.57000816	3.7621922	3.43151082	3.43739238	3.81745672	3.5215555	3.86950354
NONHSAG055699	3.58449333	3.71877041	4.10893652	3.5673081	3.71889692	3.37962119	3.87965642	3.66957748	3.80150137
NONHSAG055722	2.8957879	3.02567046	3.18745098	3.00425168	3.02468428	2.97388896	3.27359055	2.88149156	3.11331173
NONHSAG055783	5.04726473	4.74153493	4.7337545	4.54039462	4.76627135	4.71300735	4.63286017	4.77272283	4.80544099
NONHSAG055861	2.66866805	2.32494042	2.43638484	2.47215608	2.47828967	2.28991423	2.54924264	2.30130023	2.54924264
NONHSAG055878	3.84394091	3.75300398	3.90580026	3.76971931	4.00378831	3.81338722	4.01377544	3.85337633	3.86031338
NONHSAG055883	5.17883888	5.15952665	5.52017729	5.09064626	5.3364014	4.96983241	5.74809533	4.97821881	5.39876537
NONHSAG056024	3.79882988	3.69442311	3.88786745	3.54356208	3.89547175	3.55985738	4.24368567	3.71454101	3.80261136
NONHSAG056026	2.1357814	2.22687032	2.29077263	2.1379729	2.4183719	2.47830325	2.29663328	2.44022647	2.3148442
NONHSAG056029	4.27466788	4.39979442	4.17849161	4.47746624	4.48251163	4.37551928	4.31288906	4.31991106	4.33510317
NONHSAG056034	5.15269468	5.17356308	5.52261466	5.09947876	5.32662876	5.48729216	5.36798296	5.39786776	5.51731543
NONHSAG056037	6.4995195	6.2991114	6.96640713	7.31568919	7.29806662	7.25879559	6.97521953	8.03858648	7.58162216

	E2_ICI_8	E2_ICI_9	E2_ICI_10
NONHSAG000057	5.33102782	5.26657737	5.35661345
NONHSAG000058	6.02873027	6.0304283	5.97507804
NONHSAG000070	6.73631376	6.78010441	7.11668344
NONHSAG000071	5.30832693	5.36053691	5.36306759
NONHSAG000075	6.14481208	6.3196501	6.51617704
NONHSAG000111	3.81851236	3.84054463	3.93790324
NONHSAG000113	5.80791308	5.96812949	6.00119517

NONHSAG000117	6.74510987	6.64962287	6.58247835
NONHSAG000134	3.05167243	3.11332574	3.25221448
NONHSAG000139	5.56947446	5.39705042	5.57754006
NONHSAG000169	3.11641109	3.30456653	3.5533695
NONHSAG000174	4.72951951	4.63747794	4.73992846
NONHSAG000181	3.16764324	3.09467683	2.84885601
NONHSAG000185	3.84755192	3.99277487	4.07495336
NONHSAG000199	6.11496683	6.18740831	6.46194004
NONHSAG000202	3.17208197	3.28107951	3.31174181
NONHSAG000215	3.4792916	3.56787297	3.67363487
NONHSAG000222	3.7472158	3.63749832	3.97873175
NONHSAG000254	6.21145005	6.00452604	5.90317932
NONHSAG000264	3.94777179	3.88530011	3.97852414
NONHSAG000267	5.10509898	5.08781713	5.28555927
NONHSAG000310	6.84583779	6.83988374	6.56762815
NONHSAG000312	4.64271417	4.61128775	4.77104162
NONHSAG000322	2.90614849	3.30567789	2.99013254
NONHSAG000329	7.5669546	7.45462799	7.56506146
NONHSAG000345	9.2982597	9.19902829	9.00497911
NONHSAG000364	5.41845823	5.50236302	5.68887028
NONHSAG000381	4.1642775	4.19352226	4.49026604
NONHSAG000395	4.53684989	4.54061921	4.38125297
NONHSAG000423	2.70935824	2.77560498	2.73701227
NONHSAG000442	9.00146673	9.02833426	8.78480403
NONHSAG000476	5.68499367	5.72137565	5.99776825
NONHSAG000490	2.57950818	2.76925326	2.98125814
NONHSAG000522	2.92797045	2.77067804	2.72603499
NONHSAG000529	4.09362993	4.07324449	4.31692776
NONHSAG000546	5.17155132	5.26325936	5.40188219
NONHSAG000628	3.72649975	4.51441187	4.4557368
NONHSAG000648	5.53758733	5.83558945	5.33298858
NONHSAG000655	4.68104186	4.7200965	4.76067196
NONHSAG000666	4.88267277	5.15235964	5.0578967
NONHSAG000716	3.78942301	3.7925788	4.07353455
NONHSAG000738	3.80046228	3.91019016	4.09573982
NONHSAG000751	3.10728471	3.20887567	3.34833029
NONHSAG000769	4.46174938	4.31234097	4.66234295
NONHSAG000778	2.35200514	2.63647363	2.62173594
NONHSAG000812	4.43797565	4.2738133	4.10038756
NONHSAG000824	2.79400669	2.83943198	2.77041828
NONHSAG000847	3.69381551	3.86645637	3.97613806
NONHSAG000884	3.26603921	3.33571606	3.84458202
NONHSAG000939	3.98382965	4.19011715	3.7943807
NONHSAG000946	5.66087701	5.83422734	5.73805574
NONHSAG000965	3.35934847	3.1585084	3.31314444
NONHSAG000972	2.53539822	2.35976631	2.36544654
NONHSAG000976	5.39699617	5.23013312	4.98058334
NONHSAG000979	4.05004525	3.9800561	4.22724039
NONHSAG001012	8.93290223	8.90730383	8.9294393
NONHSAG001018	2.43258895	2.17867671	2.42145182
NONHSAG001041	5.1523013	5.19488576	5.1923481
NONHSAG001043	5.21478649	5.27859023	5.45333554
NONHSAG001075	3.09253861	3.41309118	3.36902776
NONHSAG001146	7.80219117	7.84297541	7.55292214
NONHSAG001183	2.30543156	2.62049486	2.55238083
NONHSAG001185	2.90153582	3.02509642	3.11589833
NONHSAG001211	3.34369984	3.16420168	3.63051352
NONHSAG001222	4.43154805	4.4280521	4.3992481
NONHSAG001244	3.15175398	3.02252711	3.04198309
NONHSAG001246	5.31244576	5.12635452	5.16852245
NONHSAG001247	4.90286183	5.03926918	4.5966136
NONHSAG001264	3.89961462	3.49396805	3.92302969
NONHSAG001304	3.2690712	3.0865404	3.39031428
NONHSAG001323	4.65916524	4.42996147	4.76015677
NONHSAG001341	2.69439521	2.88008586	3.0253868
NONHSAG001391	4.17065986	4.26642966	4.32513008
NONHSAG001489	2.90846763	2.77406573	2.78503484
NONHSAG001510	4.73744914	4.47198727	4.72845281
NONHSAG001530	6.54440851	6.4230794	6.48595377
NONHSAG001569	2.40868991	2.35580908	2.76519536
NONHSAG001574	3.58968153	3.77405501	3.80149254

NONHSAG001576	3.19752376	3.15468262	3.49573274
NONHSAG001587	3.19054544	3.27519797	3.09892985
NONHSAG001597	2.99412619	2.84388981	2.92575776
NONHSAG001601	3.75725972	4.12452736	4.55890841
NONHSAG001622	6.59849402	6.54429919	6.41389392
NONHSAG001627	4.89549969	5.11375973	5.18028798
NONHSAG001640	4.13561197	4.20531809	4.22987845
NONHSAG001660	4.73333981	5.02526275	4.98049971
NONHSAG001683	4.13802346	3.95342257	4.23687778
NONHSAG001696	6.70500451	6.64015712	6.17084823
NONHSAG001722	4.36585397	3.96311538	4.59224571
NONHSAG001723	3.1320822	3.69864201	3.41380733
NONHSAG001768	2.70999257	2.56980669	2.87279019
NONHSAG001788	2.45587603	2.29785446	2.45925786
NONHSAG001803	2.74758422	2.83576946	2.91027069
NONHSAG001824	2.52254079	2.43312074	2.872121
NONHSAG001832	2.58021368	2.71237615	2.60826653
NONHSAG001845	2.3827883	2.38056072	2.36052557
NONHSAG001849	10.84339478	10.78523237	10.78482104
NONHSAG001855	3.04919215	2.71113026	2.77705187
NONHSAG001898	3.10281225	2.85698358	3.25087382
NONHSAG001910	3.02246307	2.8616319	2.80823421
NONHSAG001920	2.5952677	2.73335488	2.63244228
NONHSAG001926	2.48421579	2.53635721	2.73914151
NONHSAG001938	2.8730316	3.03090055	3.11974499
NONHSAG001941	3.19839643	3.28744416	3.4185401
NONHSAG001965	8.51089904	8.55271256	8.50922339
NONHSAG001966	8.75927713	8.64287065	8.58403167
NONHSAG001970	3.22551277	3.23243334	3.38386943
NONHSAG001984	4.15108851	4.4303058	4.38584817
NONHSAG001992	4.18297502	4.08195298	4.13367562
NONHSAG002015	3.16239808	3.37184187	3.38335225
NONHSAG002029	2.52863882	3.02246253	2.96254878
NONHSAG002048	2.28869762	2.4256581	2.5456885
NONHSAG002049	4.41630932	4.48327753	4.24921207
NONHSAG002063	3.86916253	4.14742285	3.99706735
NONHSAG002096	4.09377518	4.15090423	3.97275069
NONHSAG002128	4.07572868	3.80811036	3.96446991
NONHSAG002154	2.21410063	2.26083952	2.40366256
NONHSAG002156	4.53550187	4.65203828	4.85058814
NONHSAG002177	2.4739559	2.66176458	2.9113127
NONHSAG002178	2.21037783	2.15577327	2.13595868
NONHSAG002204	3.39524628	3.34296522	3.33142402
NONHSAG002211	2.55998008	2.4590561	2.725349
NONHSAG002218	2.39839443	2.46668403	2.39324676
NONHSAG002223	4.17434316	3.99676896	4.53308741
NONHSAG002277	4.30673853	4.66912179	4.51119046
NONHSAG002281	4.30314857	4.15563137	4.09503416
NONHSAG002337	3.94242676	3.89411821	3.82975091
NONHSAG002360	5.45774905	5.47320993	5.59110307
NONHSAG002376	2.35014264	2.33631464	2.52983337
NONHSAG002377	5.46830939	5.58942704	5.46070018
NONHSAG002389	3.36283546	3.31019163	3.32672746
NONHSAG002392	2.83567884	2.92500329	2.66801659
NONHSAG002406	3.02781601	3.33966235	3.36215084
NONHSAG002410	4.88826756	4.86831513	4.76996605
NONHSAG002419	4.87513315	4.5770053	4.69705131
NONHSAG002421	3.2855382	3.20742134	3.2418478
NONHSAG002440	5.69210775	5.81139134	5.86873249
NONHSAG002455	3.22969355	3.08172549	3.27484583
NONHSAG002458	2.83450984	3.03419124	3.12588965
NONHSAG002465	6.08415528	6.66256721	6.577623
NONHSAG002516	2.90521988	3.15923072	3.24192938
NONHSAG002535	3.69873698	3.57674869	3.53258369
NONHSAG002538	5.29071289	5.02774114	5.31426778
NONHSAG002549	3.93656551	4.14473793	4.5604276
NONHSAG002555	2.98207873	2.87888183	2.61767891
NONHSAG002615	7.25968457	7.42312989	7.43587517
NONHSAG002728	4.62432452	4.4291786	4.54229955
NONHSAG002758	4.83997845	4.96300666	4.75817128
NONHSAG002759	2.79024643	2.88342357	2.84684945

NONHSAG002764	3.01738409	3.0868945	3.290956
NONHSAG002857	3.24717042	3.5029683	3.60542664
NONHSAG002865	3.84646611	3.74161738	4.02234148
NONHSAG002891	7.11182878	7.25680904	7.06785234
NONHSAG002908	6.58113494	6.39517185	6.63324494
NONHSAG002936	8.11351012	8.00983538	7.93248417
NONHSAG002956	4.17078925	3.98730805	4.05395359
NONHSAG003018	4.68717153	4.36969377	4.84166442
NONHSAG003022	4.49646161	4.32881352	4.51514447
NONHSAG003057	7.17003513	6.98381004	7.16421949
NONHSAG003089	4.18847382	3.90545172	4.23480853
NONHSAG003123	2.45007513	2.41600266	2.71017659
NONHSAG003195	3.67637218	3.85399414	3.65605008
NONHSAG003198	2.84155047	3.08368397	3.09690523
NONHSAG003202	7.25452571	7.18305826	7.16186267
NONHSAG003261	3.71834559	4.10999707	4.27188478
NONHSAG003289	2.83230453	2.84129917	2.68453129
NONHSAG003322	5.90163504	6.07772779	6.31210856
NONHSAG003325	5.92522052	6.12346121	5.80249363
NONHSAG003332	3.5850647	3.48166836	4.07684157
NONHSAG003339	2.52280718	2.34288297	2.54352879
NONHSAG003345	3.42186006	3.78452942	3.80997057
NONHSAG003405	3.08217062	3.29053216	2.9168617
NONHSAG003407	7.31815382	7.36087306	7.51696438
NONHSAG003412	2.10509974	2.41976879	2.21289628
NONHSAG003464	3.81000825	3.73552558	3.91188234
NONHSAG003486	5.34363897	5.08859714	5.49471537
NONHSAG003512	4.12714199	4.28157483	4.02760396
NONHSAG003533	3.62998872	3.64547887	3.79931207
NONHSAG003536	3.92805063	3.83581945	3.97413142
NONHSAG003539	3.18373365	3.03226846	3.09547774
NONHSAG003554	2.30325477	2.35570335	2.37667603
NONHSAG003555	5.63328669	5.72660694	5.75637643
NONHSAG003562	3.25149096	2.83501827	3.2042707
NONHSAG003567	2.11554654	2.28811202	2.18812625
NONHSAG003604	3.91580754	3.85558912	4.31583389
NONHSAG003613	4.83638783	4.39861545	4.563993
NONHSAG003615	2.57972524	2.64221384	2.80642549
NONHSAG003627	3.70201187	3.98510465	3.65956257
NONHSAG003635	4.66262065	4.47683388	4.55223093
NONHSAG003645	4.54099583	4.45663328	4.6926039
NONHSAG003668	2.49498219	2.51972953	2.42085087
NONHSAG003758	2.02084662	2.21984384	2.24574855
NONHSAG003765	2.22845917	2.25200003	2.20592451
NONHSAG003799	3.07025036	2.97697177	3.07800163
NONHSAG003829	3.52963112	3.46414189	3.32186867
NONHSAG003840	2.98061625	2.95814656	2.94570215
NONHSAG003843	2.15594706	2.27569933	2.23904074
NONHSAG003854	3.08568084	3.17183211	3.01686882
NONHSAG003880	5.07522145	5.1475675	5.18403175
NONHSAG003894	3.61053904	3.59181864	3.61625733
NONHSAG003900	2.86366302	2.87183448	3.03765473
NONHSAG003927	6.72547656	6.83251132	6.68076806
NONHSAG004034	4.63269633	4.65857826	4.8278108
NONHSAG004049	2.19003513	2.44633286	2.50988199
NONHSAG004092	4.07667723	4.14052239	4.221088
C1orf132	6.96109203	6.97078291	6.83650978
NONHSAG004140	3.53410767	3.78545314	3.81606732
NONHSAG004156	3.26694306	3.40937525	3.47247308
NONHSAG004159	5.64739157	5.45256681	5.58389214
NONHSAG004205	3.23613027	3.02788107	3.17784178
NONHSAG004221	3.78939875	3.17025167	3.66731564
NONHSAG004235	4.47556286	4.68784511	3.99706735
NONHSAG004261	3.01316295	3.06158627	3.06380261
NONHSAG004265	2.66362068	2.78468833	2.82879325
NONHSAG004289	7.2780778	6.90912077	7.19278421
NONHSAG004292	5.51270327	5.44002094	5.63860316
NONHSAG004319	2.95096445	3.07050729	2.90330478
NONHSAG004322	4.36376597	4.50957875	4.37820695
NONHSAG004342	5.12292469	5.1971995	5.47792603
NONHSAG004374	4.25764734	4.2006282	4.19393331

NONHSAG004468	4.53679347	4.53855472	4.65741684
NONHSAG004469	2.94385896	3.26329902	3.25897744
NONHSAG004473	4.17557009	4.15361362	4.62102338
NONHSAG004494	2.47238424	2.5319469	2.46397083
NONHSAG004522	2.58056694	2.71390826	2.51132632
NONHSAG004544	5.73100553	5.69703554	5.84209419
NONHSAG004554	2.65077389	2.41611425	2.54882254
NONHSAG004567	5.34896631	5.28695588	5.09517411
NONHSAG004569	3.71852796	4.24882004	4.36572057
NONHSAG004584	3.84875466	3.83540356	3.95321778
NONHSAG004588	2.89960321	2.94047861	3.0885487
NONHSAG004592	2.23150767	2.21912365	2.29192432
NONHSAG004614	6.3549045	6.33486918	6.12759967
NONHSAG004619	4.6167961	4.6658267	4.7118027
NONHSAG004632	2.70138942	2.56022606	2.79339689
NONHSAG004636	3.88571142	3.72353432	3.94166557
NONHSAG004663	3.06894855	3.03827696	3.27962935
NONHSAG004683	3.60335106	3.91995059	3.69543198
NONHSAG004693	3.18755003	3.27932451	3.14439857
NONHSAG004694	4.45266976	4.54506774	4.66082595
NONHSAG004774	2.82611025	2.69598472	3.05441738
NONHSAG004778	2.72551224	2.6779137	2.93500534
NONHSAG004794	3.67584223	3.74102853	3.99316001
NONHSAG004884	4.59713157	4.40964496	4.60182559
NONHSAG004887	4.60737254	4.83516792	4.78178315
NONHSAG004895	3.8260924	3.83964485	4.13970674
NONHSAG004899	3.39030695	3.80315428	3.91066307
NONHSAG004902	5.19856114	5.23072344	5.36035918
NONHSAG004908	4.89948069	5.07629172	4.81393051
NONHSAG004919	7.28279117	7.4046689	7.27087722
NONHSAG004920	2.94831987	2.95753218	3.06284239
NONHSAG004943	3.04919215	2.60124868	2.9876071
NONHSAG004961	5.1271605	5.1200279	5.0956447
NONHSAG004977	6.39050561	6.34199818	6.41682149
NONHSAG005016	5.68573047	5.52199438	5.84161523
NONHSAG005033	2.33937244	2.28906911	2.37167663
NONHSAG005060	2.53237025	2.35675418	2.60450109
NONHSAG005063	2.98885478	3.18437953	3.048105
NONHSAG005080	2.71385285	2.5213415	2.71821731
NONHSAG005082	4.12873579	3.96913273	4.27488328
NONHSAG005119	2.93991031	2.63789901	2.81624512
NONHSAG005138	4.6280196	4.46169442	4.70743994
NONHSAG005145	3.03326849	2.88940848	3.27908512
NONHSAG005150	4.48535322	4.72974132	4.58843198
NONHSAG005165	5.04603179	4.84924176	5.12213194
NONHSAG005205	3.14146704	2.78362045	2.83576946
NONHSAG005207	4.50606263	4.49083687	4.15286536
NONHSAG005225	3.56654458	3.34810325	3.6924341
NONHSAG005226	3.15622417	3.13467685	3.29795653
NONHSAG005232	6.27699497	6.34646826	5.99409443
NONHSAG005234	3.85953531	3.69066477	3.95041082
NONHSAG005239	5.34005286	5.42577763	4.86212156
NONHSAG005282	3.42473497	3.44464195	3.77138505
NONHSAG005284	4.47287365	4.81138014	4.3965905
NONHSAG005342	5.0892521	5.11202501	4.97610489
NONHSAG005356	2.58676759	2.53836069	2.63981239
NONHSAG005375	4.59878321	4.850999	4.21264023
NONHSAG005386	3.19434343	3.36087943	3.58000458
NONHSAG005407	6.22488849	6.17640623	6.25683875
NONHSAG005420	2.70478939	2.97060434	3.02091575
NONHSAG005464	5.02435686	5.3635012	5.21001155
NONHSAG005471	2.7392015	2.74854779	2.72867691
NONHSAG005501	3.95671682	3.89628409	4.27292214
NONHSAG005503	4.35878873	4.9558687	4.7349984
NONHSAG005505	2.45689591	2.13026233	2.41072556
NONHSAG005523	6.01941401	5.95922008	5.71699181
NONHSAG005543	3.12240828	2.94328878	3.01409947
NONHSAG005545	2.6557281	2.69495336	2.97642388
NONHSAG005589	4.5523154	4.30582229	4.46410584
NONHSAG005603	3.22866285	3.5869608	3.84633356
NONHSAG005687	4.24099255	4.16548784	4.78078728

NONHSAG005692	3.37977653	3.1143598	3.51513136
NONHSAG005715	6.34651763	6.37625114	6.17478684
NONHSAG005734	3.98007414	4.16354372	4.35694529
NONHSAG005754	4.06868585	4.10225677	3.96559637
NONHSAG005762	3.22075793	3.46209098	3.4590889
NONHSAG005767	3.13113979	3.2082217	3.22557305
NONHSAG005812	2.61904786	2.31910082	2.66874126
NONHSAG005834	5.08502531	5.14383354	5.16353082
NONHSAG005850	2.98986262	2.99240328	3.31788589
NONHSAG005870	4.7658668	4.77730044	4.88986574
NONHSAG005912	2.76346397	2.36073419	2.5938081
NONHSAG005923	6.44118724	6.37173905	6.18135534
NONHSAG005953	3.54378073	3.73662551	3.57546938
NONHSAG005957	2.8961804	2.92546526	2.82081749
NONHSAG005965	2.98751004	2.97060434	3.06947345
NONHSAG005970	3.1952061	3.3023872	3.09571147
NONHSAG006053	2.45305738	2.32870202	2.55093106
NONHSAG006081	6.27384228	6.1635053	6.19778631
NONHSAG006083	3.09022933	3.28988365	3.18233506
NONHSAG006087	4.1560914	4.25040968	4.22071122
NONHSAG006140	2.84256482	2.95986038	3.06947345
NONHSAG006147	4.6093717	4.65712013	5.12896716
NONHSAG006149	4.2144048	4.67120425	4.82044787
NONHSAG006187	2.05752566	2.03556919	2.01428112
NONHSAG006196	5.31229519	5.3773586	5.38572968
NONHSAG006210	5.53481741	5.21301401	5.3889296
NONHSAG006244	2.98373777	3.13210851	3.18679285
NONHSAG006270	2.69147556	2.72983982	2.8879704
NONHSAG006274	2.90971816	2.79324592	2.85140283
NONHSAG006311	3.50332946	3.54392927	3.91007465
NONHSAG006312	2.9979566	2.953965	3.11525055
NONHSAG006326	3.71207952	3.44790687	3.77524204
NONHSAG006406	3.88016027	3.16211295	3.70389903
NONHSAG006407	3.82016091	4.22675223	4.20490209
NONHSAG006417	4.31924778	4.07268214	4.59496651
NONHSAG006458	4.92679416	5.14104689	5.25838322
NONHSAG006506	2.12692323	2.18991318	2.72886586
NONHSAG006557	4.07521329	3.97969449	4.15321044
NONHSAG006570	6.03474994	6.03986898	6.43331272
NONHSAG006585	6.88105466	6.56331074	7.09756169
NONHSAG006591	4.97066499	5.37576901	5.32030372
NONHSAG006615	5.7483377	5.56051231	5.4415391
NONHSAG006619	7.50978421	7.64173453	7.51526995
NONHSAG006623	5.02469551	5.05910664	5.24014483
NONHSAG006631	10.20841489	10.24605143	10.3027923
NONHSAG006633	3.82878528	3.93798344	3.86539746
NONHSAG006679	5.91258981	5.87018934	5.93437973
NONHSAG006690	4.13532128	4.27580194	4.07086697
NONHSAG006700	5.0688984	4.93382116	5.19427283
NONHSAG006702	6.11676587	6.08725031	6.27706515
NONHSAG006706	4.28147403	4.33904443	4.42276819
NONHSAG006711	2.96299866	3.14750772	3.12979165
NONHSAG006726	2.99758305	2.8269662	2.8535149
NONHSAG006750	2.12080108	2.43562726	2.31822741
NONHSAG006793	5.66264124	5.88801483	5.83377466
NONHSAG006798	3.24580098	3.10326842	3.2453009
NONHSAG006806	4.41569552	4.28271769	4.58700463
NONHSAG006870	3.02808058	3.13804746	3.08552535
NONHSAG006890	5.97514017	5.99646955	5.95646324
NONHSAG006892	5.2466445	4.88885145	4.90100404
NONHSAG006896	2.54887904	2.73092102	2.93998972
NONHSAG006898	5.59197802	5.52128942	5.67880723
NONHSAG006900	4.60548219	4.88645549	4.74428544
NONHSAG006943	2.89877789	2.90918299	3.04589456
NONHSAG006949	2.39236519	2.40729255	2.51762158
NONHSAG006969	3.71434358	3.97310745	3.75810193
NONHSAG006971	5.74095495	5.62742979	5.40535433
NONHSAG006980	2.30963005	2.5061377	2.2368146
NONHSAG006992	3.97354793	4.13324087	3.84267523
NONHSAG007038	6.02449098	6.37535258	6.29093537
NONHSAG007047	2.16304464	2.97865664	2.46613611

NONHSAG007108	2.56468932	2.76214722	2.79057179
NONHSAG007115	2.29401788	2.17148387	2.36153408
NONHSAG007133	3.99756899	3.42561828	3.64385556
NONHSAG007156	5.25151695	5.55153973	5.6655359
NONHSAG007158	4.73188982	4.68449645	5.11231496
NONHSAG007166	4.39167901	4.37911442	4.07302874
NONHSAG007181	5.25961138	5.47272392	5.54378973
NONHSAG007207	5.43907708	5.64844037	5.42184264
NONHSAG007221	3.46384296	3.75983525	3.64265221
NONHSAG007222	4.72267538	4.91647885	4.97606072
NONHSAG007253	4.39179942	4.76155887	4.7829101
NONHSAG007359	5.91996431	5.83870697	6.10034283
NONHSAG007368	5.13183916	5.42182753	5.39017573
NONHSAG007373	4.75446597	4.76170666	4.90027317
NONHSAG007386	4.53312393	4.94902345	4.87487136
NONHSAG007407	4.59999053	4.69022951	4.85328522
NONHSAG007504	4.64186712	4.66040117	4.71245099
NONHSAG007506	2.45952909	2.39265337	2.59281818
NONHSAG007524	3.85051029	3.9390544	4.04947127
NONHSAG007531	3.88217616	4.05090187	4.41568916
NONHSAG007550	4.58473283	4.94203732	4.87355587
NONHSAG007560	2.74101491	2.88625137	2.86457908
NONHSAG007562	2.74218095	2.74502385	2.74765346
NONHSAG007577	4.16566746	4.05766467	4.48051777
NONHSAG007580	5.17788276	5.1544082	5.31490113
NONHSAG007590	5.5473506	5.87201074	5.53737875
NONHSAG007653	3.00838971	2.96727406	3.12718607
NONHSAG007683	3.58207567	3.78307673	3.9787094
NONHSAG007686	3.73050219	3.67685254	3.91397517
NONHSAG007695	4.28450081	4.23881412	4.3505508
NONHSAG007714	3.97638672	3.76521479	4.30194562
NONHSAG007751	7.09433911	7.38140321	7.03833237
NONHSAG007766	6.94469152	6.6345702	6.85507153
NONHSAG007767	3.66571136	3.56137518	3.92489251
NONHSAG007770	2.69621791	2.63444354	3.00078125
NONHSAG007825	3.41265974	3.50791752	3.79590551
NONHSAG007828	2.93076674	3.12635775	3.20003718
NONHSAG007837	4.06277667	4.09277379	4.38809049
NONHSAG007839	5.11798092	4.98125838	4.86278313
NONHSAG007862	3.4476257	3.29426849	3.59165582
NONHSAG007868	3.11129775	3.40584894	3.16426214
NONHSAG007885	2.50927952	2.60163283	2.58701609
NONHSAG007890	2.94252567	3.07055791	3.11162056
NONHSAG007906	2.65667217	2.32118286	2.38125705
NONHSAG007910	3.59367374	3.56043979	3.81507129
NONHSAG007917	2.41896166	2.4762262	2.50150998
NONHSAG007923	3.32940562	3.34895403	3.3040195
NONHSAG007937	3.37062301	3.29724858	3.01437056
NONHSAG007942	3.0222665	3.34357836	3.32264369
NONHSAG007976	3.89910235	3.91516727	3.94806491
NONHSAG007987	2.84086348	2.95445135	3.09232082
NONHSAG008006	4.80769121	4.6650766	4.61514502
NONHSAG008015	4.23979485	4.29795056	4.51813455
NONHSAG008021	3.38100623	3.27284877	3.43775187
NONHSAG008036	3.40624595	3.48696403	3.75685856
NONHSAG008052	3.05016961	3.30703068	3.56876763
NONHSAG008061	4.52272883	4.46157001	4.35538915
NONHSAG008068	2.35948003	2.14119481	2.38802202
NONHSAG008074	3.72084318	3.81862393	3.84495044
NONHSAG008085	2.04818801	2.17603044	2.11863497
NONHSAG008088	2.94575746	3.00235492	3.24434223
NONHSAG008123	4.98743119	4.75761802	4.8503342
NONHSAG008125	2.48064916	2.66704766	2.55088465
NONHSAG008197	3.444747	3.61746624	3.61662063
NONHSAG008233	2.96805333	2.97932365	2.8290339
NONHSAG008613	3.59484346	3.45682679	3.80595433
NONHSAG008637	5.14288111	5.06646339	5.33691775
NONHSAG008664	4.55783314	4.72401377	4.71086041
NONHSAG008670	7.10029776	7.2459474	7.13225128
NONHSAG008671	11.10303339	11.27444208	11.35551982
NONHSAG008684	3.97800614	4.04705132	4.40010394

NONHSAG008765	5.44691226	5.49103274	5.35832282
NONHSAG008818	5.55270447	5.7459476	5.35092262
NONHSAG008819	4.47269531	4.59431558	4.81888732
NONHSAG008903	3.85149886	3.94173018	4.01481478
NONHSAG008932	4.95066564	5.08637094	5.3647612
NONHSAG008943	4.83834663	4.80958648	4.65358443
NONHSAG008986	2.15481442	2.22985076	2.22591189
NONHSAG009036	4.11187154	3.98923585	3.91151961
NONHSAG009072	6.11754819	6.32079073	6.45033014
NONHSAG009076	2.71068039	2.8160147	2.73709675
NONHSAG009130	6.38174775	6.31731647	6.23515321
NONHSAG009132	2.8783787	2.64763149	2.98898182
NONHSAG009142	4.8519519	4.78937673	4.98558057
NONHSAG009168	9.04487795	8.98235129	9.01598593
NONHSAG009184	3.24719078	3.06825628	3.29288654
NONHSAG009186	3.23879266	3.38749536	3.55912661
NONHSAG009191	3.14094685	2.98932319	2.91210429
NONHSAG009210	4.33173504	4.67478052	4.96712822
NONHSAG009216	2.62965537	2.75927431	2.83873858
NONHSAG009222	4.66630623	5.00334837	4.60438545
NONHSAG009269	4.38869843	4.38842604	4.33803882
NONHSAG009275	2.39157039	2.53581468	2.51986209
NONHSAG009283	4.85734147	4.95129027	5.05450888
NONHSAG009302	3.38164347	3.37719086	3.31408121
NONHSAG009308	2.42460405	2.56156453	2.69027083
NONHSAG009381	2.45731319	2.77560498	2.42085087
NONHSAG009397	2.80535786	2.85204424	2.82412704
NONHSAG009424	3.50937835	3.54189652	3.72609995
NONHSAG009434	2.79701923	2.59336176	2.55884279
NONHSAG009452	4.20736492	4.27135135	4.28540572
NONHSAG009462	3.88312258	3.49929777	3.78873083
NONHSAG009465	2.61668923	2.72939888	2.86269955
NONHSAG009524	2.79628724	2.7348928	2.71586755
NONHSAG009530	2.86358605	2.93666053	2.93357338
NONHSAG009590	2.42453377	2.6755827	2.83576946
NONHSAG009627	5.40917189	5.36367088	5.60487739
NONHSAG009630	2.48506979	2.37501351	2.42242033
NONHSAG009643	4.89739591	4.91677784	4.43630451
NONHSAG009663	2.7306591	2.62154492	2.56551966
NONHSAG009678	3.94435365	4.24016574	4.39492504
NONHSAG009688	3.30239859	3.49997691	3.47404336
NONHSAG009690	4.54928438	4.49433831	5.04128318
NONHSAG009700	2.89908086	3.13397429	2.96977135
NONHSAG009711	2.88791699	2.72953124	2.99302794
NONHSAG009736	3.0021181	2.79868762	3.55085073
NONHSAG009783	2.85474869	2.88403693	2.95264743
NONHSAG009834	4.07379749	4.36629608	4.43278931
NONHSAG009878	2.31892291	2.34367025	2.16633251
NONHSAG009880	2.10873529	2.0583033	2.25870644
NONHSAG009903	4.08884581	4.23379973	4.28012847
NONHSAG009919	3.59785647	3.6579774	3.6027926
NONHSAG009921	4.01588433	4.11228874	4.39843067
NONHSAG009942	3.47173362	3.74849609	3.47762484
NONHSAG009977	4.41978735	4.30833791	4.16958652
NONHSAG009994	5.3716069	5.44951727	5.09313304
NONHSAG010005	2.78246741	2.38462416	2.69861676
NONHSAG010007	2.72811169	2.87536562	2.97955936
NONHSAG010015	2.43101739	2.79479276	2.42085087
NONHSAG010022	2.74234326	3.10477622	3.17219242
NONHSAG010026	4.21774681	4.21256638	4.36717615
NONHSAG010034	2.8349476	3.12531495	3.19602786
NONHSAG010061	3.94838075	3.79714702	3.87516899
NONHSAG010086	2.76111558	2.86572154	2.99572888
NONHSAG010089	3.11961773	2.94212585	3.42694479
NONHSAG010109	4.96287808	5.02833014	5.22763507
NONHSAG010134	6.39171121	6.28971574	6.0584894
NONHSAG010144	4.24407347	3.96374296	3.96456989
NONHSAG010152	4.33201634	4.47250362	4.49010703
NONHSAG010157	2.56182922	2.71685215	2.50384321
NONHSAG010160	3.57987505	3.79916757	4.09005743
NONHSAG010163	2.77264688	2.96809417	2.86798999

NONHSAG010226	4.20385028	4.25139376	4.4923455
NONHSAG010232	2.87806988	2.92203413	3.07168874
NONHSAG010260	4.67365924	4.56234914	4.74027398
NONHSAG010262	5.0223247	5.38715105	5.15483199
NONHSAG010264	5.36279844	5.29326444	5.18686748
NONHSAG010352	5.81411461	5.88195126	5.79317017
NONHSAG010413	2.10015509	2.23462895	2.43724723
NONHSAG010420	6.52698672	6.64103509	6.42471948
NONHSAG010447	2.47454049	2.53217544	2.48747005
NONHSAG010455	6.75986141	6.88751007	6.74595731
NONHSAG010492	3.0971509	3.16222977	3.01860494
NONHSAG010499	2.35505417	2.54781509	2.39094926
NONHSAG010524	2.42819975	2.35135172	2.4504287
NONHSAG010532	4.74024776	4.41046105	4.28258953
NONHSAG010566	2.47925733	2.48048119	2.58874663
NONHSAG010571	4.65517186	4.92384825	4.73253998
NONHSAG010573	5.86352419	5.69370054	5.67620816
NONHSAG010585	2.70585805	2.74214884	2.75148637
NONHSAG010628	4.938689	5.08768905	5.2840757
NONHSAG010678	3.08727436	2.96910196	2.8601788
NONHSAG010682	2.64582106	2.79847407	3.00597161
NONHSAG010721	2.59007381	2.5992074	2.561478
NONHSAG010724	2.81023693	2.89626921	3.08575137
NONHSAG010765	3.10453002	3.17386617	3.12791277
NONHSAG010767	3.00021843	3.01107337	3.04961324
NONHSAG010791	2.46389547	2.32766867	2.30312742
NONHSAG010808	4.21474162	4.29611347	4.43836738
NONHSAG010846	3.97387231	4.30155003	3.55134034
NONHSAG010848	3.61145233	4.0014693	4.00372932
NONHSAG010888	2.57737432	2.68029097	2.61840074
NONHSAG010892	3.14265698	3.60401935	3.58352948
NONHSAG010908	2.28424908	2.17113841	2.28008211
NONHSAG010921	3.17322354	3.44438827	3.22913828
NONHSAG010938	4.46531729	4.36489594	4.7176783
NONHSAG010946	5.59764653	5.41410442	5.45084327
NONHSAG010964	3.48822078	3.58096075	3.78333941
NONHSAG010989	2.93163962	3.07968143	3.28504398
NONHSAG010991	3.19530561	3.45242722	3.07392196
NONHSAG010992	5.14001138	5.3838112	5.31016377
NONHSAG011000	4.61112918	4.62702512	4.67423646
NONHSAG011016	4.15652109	4.1446682	4.32485597
NONHSAG011035	3.33016944	2.90074021	2.89717123
NONHSAG011091	3.57671894	3.65093906	3.46010854
NONHSAG011182	5.3534859	5.40941322	5.33658578
NONHSAG011270	3.69045582	3.59665716	3.85637137
NONHSAG011279	3.66506971	3.64337582	4.10919623
NONHSAG011288	8.66134023	8.50551654	8.53203319
NONHSAG011425	3.71973766	4.40550489	4.86782025
NONHSAG011459	5.65779848	5.49075876	5.73037212
NONHSAG011460	5.37312568	5.21279551	5.58984728
NONHSAG011467	2.78246741	2.97913429	2.98965365
NONHSAG011471	3.41569144	3.47402023	3.42054105
NONHSAG011496	3.85351413	4.23034846	4.17653264
NONHSAG011553	3.38751946	3.30021571	3.34954098
NONHSAG011555	3.24290359	3.08540089	3.52386922
NONHSAG011584	4.90183525	4.90524411	4.57590453
NONHSAG011594	2.9232546	2.80055493	3.02180465
NONHSAG011643	2.35877642	2.58524499	2.65214429
NONHSAG011647	2.56736509	2.54378249	2.49097161
NONHSAG011680	3.96870991	3.40605491	3.47347463
NONHSAG011703	4.36754405	4.35104964	4.3505508
NONHSAG011730	3.60124107	3.97746993	3.90921412
NONHSAG011744	3.45388606	3.5533235	3.72881506
NONHSAG011747	6.84484568	6.78234679	7.01904947
NONHSAG011762	3.31166336	3.23234128	3.45428358
NONHSAG011768	2.14071088	2.25012083	2.46779543
NONHSAG011773	2.29916015	2.46025439	2.27768845
NONHSAG011781	7.03063146	7.08880796	6.96459744
NONHSAG011800	2.48027025	2.76630208	2.54628772
NONHSAG011802	9.9474181	9.97622404	9.94497881
NONHSAG011805	3.32390592	3.02598931	3.14252709

NONHSAG011821	2.66517338	2.46092681	2.87862681
NONHSAG011830	2.2147627	2.3312825	2.15814289
NONHSAG011840	2.36706957	2.13498411	2.30032643
NONHSAG011877	5.33783214	5.30490148	5.0833692
NONHSAG011915	5.94828178	5.51876798	5.85639936
NONHSAG011924	3.30081649	3.3046426	2.83576946
NONHSAG011933	5.62590998	5.52776094	5.70322664
NONHSAG011964	6.06536856	6.02247715	5.94053307
NONHSAG011968	4.15175024	4.3199707	4.40384052
NONHSAG011977	3.0942187	2.8884169	2.79976623
NONHSAG011978	5.18628896	4.9560404	4.90329931
NONHSAG012011	2.47802585	2.35129673	2.44854722
RMST	5.92122178	5.86532449	5.41377216
NONHSAG012031	4.19967743	4.1924481	4.3505508
NONHSAG012043	2.26729259	2.15368531	2.36468452
NONHSAG012048	4.84319771	5.11363388	5.31474547
NONHSAG012050	6.87494482	6.74962463	6.57188685
NONHSAG012055	6.97171062	7.17884354	6.91421191
NONHSAG012099	2.437687	2.64052065	2.30742852
NONHSAG012152	2.31601162	2.32913231	2.11106824
NONHSAG012158	4.1035082	4.37249019	4.2186967
NONHSAG012172	3.768012	3.80100748	3.81905621
NONHSAG012182	4.13591706	4.10868332	4.20763957
NONHSAG012199	3.98115154	4.13511869	4.48602353
NONHSAG012226	3.97121569	3.99938075	4.15509071
NONHSAG012236	2.86715032	2.84257062	2.76302014
NONHSAG012367	2.92700374	3.11403285	2.95718176
NONHSAG012392	2.37607558	2.75989975	2.54222358
NONHSAG012393	4.85478643	4.49724776	4.90438072
NONHSAG012430	2.68629589	2.6321584	2.84569097
NONHSAG012433	4.85233891	5.16881023	5.15861869
NONHSAG012556	6.17476972	6.21195978	6.6519214
NONHSAG012565	4.47550689	4.36261276	4.3709003
NONHSAG012566	3.83044152	4.0130085	4.23796933
NONHSAG012599	5.74421868	5.31451929	5.66779672
NONHSAG012608	4.31816529	4.3967209	4.28012847
NONHSAG012629	2.48392408	2.47963181	2.45299667
NONHSAG012630	5.60593499	5.94138159	5.70241685
NONHSAG012635	3.01303111	3.10219983	3.15798859
NONHSAG012657	7.50813809	7.38635284	7.46323307
NONHSAG012658	9.29108437	9.18817798	9.28316674
NONHSAG012682	3.12667362	3.04666632	3.20933847
NONHSAG012683	3.16228804	3.13136191	3.17021544
NONHSAG012684	2.61904786	2.63647363	2.64911803
NONHSAG012695	4.33111912	4.32641887	4.38936254
NONHSAG012727	5.09145029	5.16018349	5.54555825
NONHSAG012732	3.67589435	3.47100977	3.40552832
NONHSAG012739	5.28481843	5.24107197	5.67613883
NONHSAG012744	3.07981988	2.98303674	2.90725585
NONHSAG012745	3.82819271	3.52235073	3.92503244
NONHSAG012747	5.12691308	5.1219605	4.89014983
NONHSAG012753	5.8531917	5.90362787	5.87850331
NONHSAG012762	4.36944446	4.25972033	4.65582258
NONHSAG012764	4.48345423	4.52836578	4.76027372
NONHSAG012780	3.95232733	4.03301724	4.34529578
NONHSAG012793	6.06576949	6.23331	6.14249788
NONHSAG012799	5.48712403	5.55639868	5.6737323
NONHSAG012823	4.28601436	4.04167308	4.18332663
NONHSAG012881	2.92454246	2.85641087	3.08048994
NONHSAG012884	2.57793485	2.78754453	2.91725238
NONHSAG012909	6.13928662	6.0444054	6.17388886
NONHSAG012910	6.2864771	6.41416144	6.1910734
NONHSAG012920	2.80744722	2.74925298	2.87004129
NONHSAG012971	2.51851848	2.20039317	2.52606235
NONHSAG012980	2.47852821	2.64035975	2.9395626
NONHSAG012986	8.17048024	7.95902726	8.19101196
NONHSAG013018	2.90886901	2.96848491	2.92972526
NONHSAG013032	2.75843076	2.56892036	2.7035809
NONHSAG013037	4.03152342	4.05098531	4.12023153
NONHSAG013039	4.0959661	3.92931307	4.37020312
NONHSAG013044	3.96759203	4.15673122	4.39971632

NONHSAG013052	4.32998694	4.55045417	4.67581986
NONHSAG013055	2.36029747	2.17512244	2.10629859
NONHSAG013061	2.75269761	2.75794076	2.81173796
NONHSAG013087	4.43633669	4.58926024	4.57834863
NONHSAG013088	4.84286617	4.69681979	4.97489518
NONHSAG013092	3.07590147	3.1367905	2.96718234
NONHSAG013093	3.84274016	3.5869608	3.82872603
NONHSAG013140	4.40725092	4.46154324	4.60888514
NONHSAG013147	2.6551922	2.68370071	2.81390479
NONHSAG013148	3.99906386	3.76720388	3.8826511
NONHSAG013176	3.29310716	3.17320121	3.35240355
NONHSAG013184	5.33262583	5.59163646	5.4721663
NONHSAG013195	5.48990822	5.57513412	5.89943837
NONHSAG013196	3.78939875	3.68864368	3.89300663
NONHSAG013213	2.47664719	2.60849669	2.64043172
NONHSAG013219	3.27645812	3.36548024	3.46068555
NONHSAG013224	3.01010046	3.12885204	3.27728617
NONHSAG013270	2.21301626	2.20260041	2.37299697
NONHSAG013289	3.64617455	3.68893897	3.46588246
NONHSAG013312	4.34341171	4.45793319	3.92489251
NONHSAG013343	8.05275197	8.1325364	8.02663375
NONHSAG013371	4.08676458	3.91141454	4.03664892
NONHSAG013387	3.88372347	3.74890457	4.13673117
NONHSAG013400	7.08842684	7.0071194	6.78953051
NONHSAG013413	3.05173518	3.95329907	3.33471569
NONHSAG013415	4.21474162	4.77211604	4.78138454
NONHSAG013417	2.29346783	2.37038767	2.42388069
NONHSAG013418	4.57203766	4.4314827	4.71124658
NONHSAG013429	8.93648635	8.85138933	8.75664515
NONHSAG013433	2.68887388	2.34069096	2.46156859
NONHSAG013449	2.4442542	2.60265125	2.82573801
NONHSAG013472	3.37952651	3.07437568	3.34941208
NONHSAG013488	3.09438437	3.08362487	3.13934673
NONHSAG013496	2.8783787	2.67885072	2.51048049
NONHSAG013505	5.49599277	5.57706123	5.70419138
NONHSAG013509	4.80214333	4.94100911	4.75171743
NONHSAG013521	3.57756569	3.64658888	3.7343778
NONHSAG013525	4.34676401	4.03497822	4.16390383
NONHSAG013533	3.50100226	3.41425835	3.42906117
NONHSAG013546	2.97060434	2.93618848	3.06947345
NONHSAG013584	5.27656574	5.31868208	5.253649
NONHSAG013596	3.65465664	3.74658877	3.48847431
NONHSAG013598	2.49653763	2.55173187	2.41870919
NONHSAG013622	2.38954181	2.28541754	2.72078409
NONHSAG013653	2.34508497	2.32766867	2.43148301
NONHSAG013655	2.82690219	2.86426034	3.05903903
NONHSAG013658	2.62321843	2.46051908	2.62173594
NONHSAG013688	3.26300281	3.33505787	3.03721114
NONHSAG013701	2.45007513	2.09792751	2.39209703
NONHSAG013735	4.07784441	3.98483084	4.17552619
NONHSAG013737	2.45180549	2.25350939	2.24270541
NONHSAG013751	2.24667641	2.64648121	2.43443829
NONHSAG013766	2.27932308	2.29709234	2.06730371
NONHSAG013770	2.45250234	2.182885	2.2108424
NONHSAG013789	4.58463842	4.75847522	4.85657755
NONHSAG013796	3.07433264	2.9358897	2.83089643
NONHSAG013836	3.70015546	3.44448714	3.65658578
NONHSAG013843	6.47051251	6.4170208	6.23846371
NONHSAG013856	3.3808459	3.23909647	3.60131814
NONHSAG013867	2.13360682	2.34213804	2.42512481
NONHSAG013871	2.85151924	2.90798749	2.88428866
NONHSAG013889	2.09197737	2.11473819	2.17733763
NONHSAG013908	2.14872931	2.31259348	2.34888153
NONHSAG013910	2.2554137	2.20726895	2.32109803
NONHSAG013921	3.20951506	3.4507361	3.14079262
NONHSAG013938	4.88829113	4.68705849	4.90379032
NONHSAG013940	4.38644114	4.36878524	4.44666207
NONHSAG013950	2.42249417	2.58545263	2.61593355
NONHSAG013963	4.90676205	4.88800889	4.92032641
NONHSAG013971	3.99428091	4.05270613	4.11776743
NONHSAG013976	3.92054155	3.87617736	3.98978928

NONHSAG013982	3.06232883	2.84115787	2.8546303
NONHSAG013992	4.34870511	4.13046717	4.01001379
NONHSAG014007	5.73774252	5.74315487	6.00818853
NONHSAG014012	4.28797681	4.28894868	4.35458438
NONHSAG014023	2.29713318	2.70947525	2.76912646
NONHSAG014030	5.41845823	5.2418786	5.17917058
NONHSAG014035	5.69210775	5.87455673	5.8571791
NONHSAG014073	6.65540409	6.15673395	6.49527281
NONHSAG014079	3.68813076	3.71928348	3.87328647
NONHSAG014090	3.92994147	3.91702041	3.84848017
NONHSAG014099	3.36750298	3.51356891	3.76548032
NONHSAG014114	2.69736364	2.53457273	2.76519536
NONHSAG014130	3.25942145	3.17594841	3.32629286
NONHSAG014136	5.45131849	5.30873409	5.4582069
NONHSAG014165	2.81907587	2.90215609	2.76468213
NONHSAG014169	2.89678896	2.89194286	3.15466868
NONHSAG014185	2.95287933	2.83207623	3.18096252
NONHSAG014197	2.77469459	2.68721345	2.82580161
NONHSAG014198	4.06238501	3.90554595	4.52757284
NONHSAG014202	3.70582721	4.09033141	4.13563533
NONHSAG014208	3.48484187	3.79254791	3.86290439
NONHSAG014212	3.01193451	3.06368948	3.22394286
NONHSAG014215	2.29076129	2.29302477	2.2111728
NONHSAG014232	4.25170618	4.15673122	4.09075467
NONHSAG014249	4.35398393	4.10161131	4.41548506
NONHSAG014269	4.50851348	4.63467748	4.70542477
NONHSAG014294	4.38593789	4.49180037	4.52226376
NONHSAG014358	3.76025742	3.78433667	3.49133063
NONHSAG014410	2.65228868	2.77715621	2.70992461
NONHSAG014436	2.86510011	2.90119127	3.02358242
NONHSAG014438	3.90532683	3.4897598	3.53815355
NONHSAG014440	2.43068047	2.4942214	2.51367757
NONHSAG014443	2.24630671	2.15339035	2.2592604
NONHSAG014445	3.3916901	3.55217529	3.33408532
NONHSAG014448	3.7716953	4.06847059	4.16773423
NONHSAG014454	2.45007513	2.43426264	2.455471
NONHSAG014569	3.3248669	3.28660743	3.41532915
NONHSAG014574	2.504514	2.4471721	2.5347277
NONHSAG014590	2.79337579	2.6704415	2.96859427
NONHSAG014611	3.29121463	3.17417561	3.27908282
NONHSAG014661	2.71997267	2.71319819	2.96226703
NONHSAG014663	5.91762812	6.18731985	6.33465079
NONHSAG014667	4.15471168	3.6702233	3.83696005
NONHSAG014746	2.3399397	2.35076435	2.33626285
NONHSAG014747	1.98932724	2.21666897	2.20089685
NONHSAG014790	4.64087864	4.62748596	5.07760742
NONHSAG014806	3.97261646	4.12761704	4.49708978
NONHSAG014811	2.28533507	2.1449664	2.1321115
NONHSAG014813	2.62756764	2.58926859	2.64587177
NONHSAG014829	4.03854114	4.19934803	4.14088848
NONHSAG014834	4.11104064	4.1866143	4.18956203
NONHSAG014845	3.42186006	3.76836585	3.38036009
NONHSAG014849	2.73028082	2.79418399	2.81248221
NONHSAG014850	2.1442921	2.25680553	2.0814017
NONHSAG014898	3.69494746	3.95353234	3.78017142
NONHSAG014905	4.52900638	4.90949962	4.9637345
NONHSAG014910	6.31809665	6.20237193	6.31163252
NONHSAG014924	5.49275981	5.69810713	5.67636579
NONHSAG014926	4.72060781	5.18276663	4.74215663
NONHSAG014964	1.95411201	2.11743044	2.08398996
NONHSAG015027	6.96760284	7.03142221	7.1013897
NONHSAG015035	2.64380819	2.41903665	2.58385791
NONHSAG015052	3.50762371	3.70139117	3.93848953
NONHSAG015063	2.87779314	3.08764153	3.13269471
NONHSAG015092	2.77061453	2.65525809	2.41339533
NONHSAG015143	5.25756373	5.58343735	5.8933241
NONHSAG015144	4.64028955	4.67377288	4.82581707
NONHSAG015185	2.44476977	2.22765017	2.14723838
NONHSAG015188	2.47261476	2.62858633	2.96674268
NONHSAG015211	2.96858879	2.89907071	2.74050419
NONHSAG015231	5.78815265	6.01054388	5.7465573

NONHSAG015256	2.94378392	3.04339955	2.74582758
NONHSAG015273	3.27970399	3.26126266	3.36040029
NONHSAG015286	5.14756096	5.36580169	5.32711861
NONHSAG015320	2.46949802	2.54802104	2.54066189
NONHSAG015338	2.65498728	2.75825151	2.82224146
NONHSAG015357	3.560387	3.73952137	3.60957352
NONHSAG015371	4.62711882	4.41357177	4.57800475
NONHSAG015373	2.64987469	2.71475281	3.11835386
NONHSAG015387	5.48871615	5.75218705	6.10014752
NONHSAG015435	4.12954465	4.15431614	4.28012847
NONHSAG015438	3.14075065	2.6598189	2.95438608
NONHSAG015450	2.46099313	2.52497022	2.27798628
NONHSAG015466	4.52792063	4.55665706	4.847143
NONHSAG015491	3.4192566	3.28411134	3.15790838
NONHSAG015511	7.72482503	7.70025296	7.45094054
NONHSAG015535	2.19610366	2.26618899	2.3157862
NONHSAG015539	4.08319447	4.07433784	3.37184607
NONHSAG015555	5.45727874	5.72339121	5.99588908
NONHSAG015654	2.94862941	2.9032128	2.86280782
NONHSAG015660	3.47498439	3.36786166	3.68622
NONHSAG015664	3.21395044	3.37581246	3.16076094
NONHSAG015694	6.36866197	6.27829556	6.73007041
NONHSAG015695	4.04130152	4.15757886	4.54962281
NONHSAG015708	3.55231727	3.5532259	3.75493857
NONHSAG015712	5.27592348	5.26086785	5.21557343
NONHSAG015718	3.32229784	3.38100951	3.43766647
NONHSAG015732	6.06924909	6.09905706	6.18128425
NONHSAG015756	2.95961762	3.07456947	3.20972833
NONHSAG015757	5.07370389	4.70165547	4.98596544
NONHSAG015797	3.31572175	3.61070725	4.14957011
NONHSAG015798	3.65073703	3.79568179	3.78733521
NONHSAG015800	2.3345001	2.32766867	2.2959414
NONHSAG015828	5.47041309	5.24683984	5.01252618
NONHSAG015833	2.97060434	2.80234655	3.18315602
NONHSAG015852	3.00749437	3.34545323	2.93178783
NONHSAG015872	5.16294957	5.10358587	5.25204837
NONHSAG015874	3.29046903	3.1715414	3.24915163
NONHSAG015923	4.06315397	4.00562768	4.01834448
NONHSAG015927	2.68166905	2.56892036	2.74327405
NONHSAG015948	3.8935033	4.00050445	4.05105498
NONHSAG015952	4.13532128	4.40458398	4.10854601
NONHSAG015966	5.313804	5.44619245	5.30121271
NONHSAG015980	6.32691218	6.30166568	6.75355795
NONHSAG015981	5.99504766	6.01907153	5.82178862
NONHSAG016009	3.05719122	3.28358483	3.30869907
NONHSAG016021	5.06080768	5.00314193	5.18612125
NONHSAG016024	3.34875568	3.87111764	3.17837082
NONHSAG016025	3.87728446	4.35777139	4.27556194
NONHSAG016027	6.91152284	7.00463141	7.13426676
NONHSAG016062	3.27194701	3.37031828	3.29286808
NONHSAG016074	8.91736972	9.30622626	9.36561921
NONHSAG016107	2.52179184	2.44813063	2.48175344
NONHSAG016109	4.53946441	4.62245807	4.44621632
NONHSAG016111	5.02633167	5.35530049	5.62036557
NONHSAG016116	2.22805304	2.43736937	2.94369115
NONHSAG016136	3.33155772	2.7332764	3.34673383
NONHSAG016155	3.28646864	3.04197685	3.30027496
NONHSAG016158	3.57533486	3.23040196	3.84298894
NONHSAG016166	3.03526575	2.65229658	2.90410606
NONHSAG016202	3.41573283	3.29418474	3.4673712
NONHSAG016213	2.03989359	1.93764753	2.23631439
NONHSAG016262	3.68660396	3.82831347	3.35852222
NONHSAG016297	2.43886858	2.64267448	2.76815243
NONHSAG016313	3.04947671	3.0085126	3.10469647
NONHSAG016316	2.18350677	2.04921482	2.16182134
NONHSAG016330	2.78246741	2.80002623	2.81988116
NONHSAG016336	4.9531682	4.92812909	4.90631237
NONHSAG016353	2.16241786	2.60082881	2.43947451
NONHSAG016383	3.88217616	3.79149613	3.87861439
NONHSAG016385	3.15664209	2.9463327	3.32971849
NONHSAG016388	4.75584041	4.89868198	5.043364

NONHSAG016419	4.64420835	4.74366668	4.66035468
NONHSAG016427	5.49401665	5.51931578	5.44308136
NONHSAG016434	2.99899005	2.92634548	2.94623869
NONHSAG016460	3.62213266	3.25319355	3.57879033
NONHSAG016472	5.62197694	5.62915802	5.95011458
NONHSAG016517	3.52537061	3.74587664	3.90749311
NONHSAG016520	2.24267452	2.0018569	1.96380129
NONHSAG016540	3.0501769	3.27796514	3.29992654
NONHSAG016542	3.12477203	2.98622479	3.18317197
NONHSAG016544	2.1644993	2.40076489	2.28514881
NONHSAG016555	3.66860489	3.57989093	3.60505779
NONHSAG016582	3.88173866	4.09541643	3.94315582
NONHSAG016589	6.69931102	6.68824519	6.62380143
NONHSAG016595	3.90261589	4.17929149	4.24989628
NONHSAG016623	4.09388028	4.09789717	4.21088439
NONHSAG016650	2.78246741	3.10273352	2.86982994
NONHSAG016655	3.27833932	3.26382922	3.59396282
NONHSAG016687	3.69854782	3.84432689	3.77104622
NONHSAG016786	6.26358437	6.53543129	6.33085841
NONHSAG016826	2.45007513	2.73646529	2.63836701
NONHSAG016852	3.72646831	3.53366701	3.45770322
NONHSAG016854	2.71155378	2.39656252	2.64019987
NONHSAG016887	3.61851082	3.55722328	3.89371581
NONHSAG016890	2.34480442	2.45817035	2.48670219
NONHSAG016921	3.61869831	3.6059497	3.62156916
NONHSAG016956	5.76077248	5.34194203	5.67342835
NONHSAG016984	2.5360055	2.75033737	2.43222469
NONHSAG016991	5.88530422	5.52277005	5.75845942
NONHSAG017020	2.62986055	2.73243402	2.89198779
NONHSAG017021	3.73697558	3.7528419	3.6808719
NONHSAG017030	4.47193439	4.45525876	4.23120156
NONHSAG017048	2.76633893	3.0346654	2.45172851
NONHSAG017077	5.56453222	5.92822816	6.26088474
NONHSAG017083	3.27892989	3.50838143	3.56030548
NONHSAG017088	2.81538241	2.77478318	2.93927944
NONHSAG017091	2.79581962	2.38554806	2.62173594
NONHSAG017106	2.83832858	3.23100853	2.94764902
NONHSAG017112	4.43923303	4.41317111	4.44102858
NONHSAG017118	3.41837263	3.48460732	3.47042572
NONHSAG017164	4.76834873	4.7357798	4.70945447
NONHSAG017207	3.9018001	4.10714743	4.37915076
NONHSAG017233	2.99658438	2.79941778	2.91027069
NONHSAG017237	6.5483376	7.09312779	7.08687925
NONHSAG017254	4.75584041	4.70528574	4.7600911
NONHSAG017262	2.71317036	2.80706748	2.63461975
NONHSAG017302	3.1584389	3.4702361	3.15623627
NONHSAG017304	2.85669149	3.13594358	2.97072744
NONHSAG017314	3.65908124	3.79276928	4.17777822
NONHSAG017331	8.04972781	8.05096068	7.85170644
NONHSAG017346	4.80283817	5.16550753	5.00703689
NONHSAG017352	3.04521943	3.00756211	3.02789065
NONHSAG017356	4.45046857	4.3914212	4.31280068
NONHSAG017360	4.07606967	4.01237536	3.86690806
NONHSAG017376	5.07516632	5.06072735	5.00994294
NONHSAG017384	5.56682557	5.58717195	5.30242577
NONHSAG017387	4.53675254	4.21017779	4.37182621
NONHSAG017486	4.24266268	4.47566525	4.58173841
NONHSAG017508	2.56541765	2.36237033	2.55180884
NONHSAG017514	2.39214656	2.57338997	2.58876466
NONHSAG017529	4.93337629	5.02498788	5.23721788
NONHSAG017532	3.67570433	3.26297318	3.3565865
NONHSAG017549	3.39830676	3.33586703	3.34352599
NONHSAG017579	2.29202387	2.39503363	2.29943829
NONHSAG017593	4.69705131	4.60293638	4.51388332
NONHSAG017594	4.96412968	4.96084617	5.07746219
NONHSAG017604	3.4244461	3.18133119	3.56762272
NONHSAG017613	4.11341826	4.19817426	4.31889736
NONHSAG017640	2.37573867	2.44927654	2.40101692
NONHSAG017669	2.8821475	2.78370038	2.93628946
NONHSAG017670	3.58640515	3.42742987	3.63029513
NONHSAG017671	2.63591847	2.83006631	3.11455469

NONHSAG017675	6.46813715	6.56621072	6.23395666
NONHSAG017708	5.84455594	5.52932174	6.1818004
NONHSAG017741	4.49684179	4.53510358	4.66382531
NONHSAG017742	4.39533554	4.61984245	4.39022757
NONHSAG017775	2.91934664	2.85954948	3.16880399
NONHSAG017777	2.89336949	2.82224864	3.07577487
NONHSAG017785	2.76829041	2.88756775	2.81992073
NONHSAG017797	5.72912914	5.94683214	5.88911149
NONHSAG017837	5.29335253	5.40460222	5.33718819
NONHSAG017855	5.05002342	5.00788857	4.98906986
NONHSAG017868	4.79227652	5.04125459	4.81732078
NONHSAG017869	7.80123333	7.94712874	7.89003989
NONHSAG017885	3.57944362	3.65530351	3.76951545
NONHSAG017934	3.63862491	3.73270427	3.78383155
NONHSAG017937	4.90586104	4.50099944	4.8784314
NONHSAG017938	3.10367624	3.35608642	3.82010285
NONHSAG017946	3.69958751	4.10756533	3.7333423
NONHSAG017952	5.53066176	5.73364265	6.0734542
NONHSAG017967	2.47443494	2.79343271	2.69550709
NONHSAG017971	3.63145273	3.75229936	4.0611564
NONHSAG017972	2.73953532	2.78501174	2.80534028
NONHSAG017996	3.85203102	4.0504331	4.3752942
NONHSAG018000	3.16260644	3.34777515	3.26529543
NONHSAG018018	4.90996597	5.05555483	5.05774506
NONHSAG018031	2.90519355	3.04871099	3.02064672
NONHSAG018033	2.99173979	3.17476187	3.26633208
NONHSAG018039	3.58200245	3.46402868	3.53264393
NONHSAG018040	3.14318771	3.02386251	3.12814508
NONHSAG018059	3.46957768	3.62310856	3.71245864
NONHSAG018075	4.68547281	4.71891492	4.54778639
NONHSAG018098	4.05558022	3.71400766	4.28671045
NONHSAG018192	4.83957308	4.91218757	5.0245668
NONHSAG018224	5.50204089	5.60920557	5.79919587
NONHSAG018230	5.58146202	5.53427102	5.63654094
NONHSAG018271	3.46721089	3.60389994	3.69370715
NONHSAG018315	11.36191261	11.36255703	11.17836526
NONHSAG018332	3.84619128	3.77548932	4.30249657
NONHSAG018336	4.6135601	4.27278014	4.73527498
NONHSAG018350	3.74916681	3.73873662	3.7700364
NONHSAG018377	5.97563094	5.70555481	5.92034209
NONHSAG018379	6.44334354	6.62544421	6.64068593
NONHSAG018419	4.50052623	4.2777618	4.44493164
NONHSAG018426	3.44589266	3.37184187	3.12346661
NONHSAG018457	4.78380729	4.55716336	4.65518167
NONHSAG018506	3.80085855	3.69045664	3.7930798
NONHSAG018536	4.94065274	5.03098498	4.84740094
NONHSAG018538	8.38070593	8.3320298	8.2800223
NONHSAG018575	4.04854289	4.11642751	4.14058115
NONHSAG018592	4.9632483	4.94767531	4.75406022
NONHSAG018593	4.67117948	4.71591616	5.30394568
NONHSAG018602	3.87512647	3.80107244	4.09696016
NONHSAG018627	6.29927164	6.17527268	6.37436163
NONHSAG018649	5.17289718	5.06303099	5.02291783
NONHSAG018664	5.25735297	5.55855592	5.57540381
NONHSAG018700	3.13042897	2.74130644	2.97520089
NONHSAG018743	3.60258401	3.59747859	3.68644604
NONHSAG018775	2.5779717	2.57936113	2.29427041
NONHSAG018846	5.99413561	5.95110245	6.0909308
NONHSAG018860	6.00348875	5.97054094	5.78496914
NONHSAG018901	3.76430453	3.82854824	3.60628686
NONHSAG018925	4.99880211	4.98966539	5.11105171
NONHSAG018928	2.96195797	3.02368573	3.07158777
NONHSAG018930	4.50063636	4.48807818	4.60718352
NONHSAG018934	6.39814213	6.35016332	6.13667693
NONHSAG018936	2.78895537	2.92333698	3.11916899
NONHSAG019021	3.18920055	3.27538875	3.5823294
NONHSAG019026	3.6131506	3.98339184	3.70268733
NONHSAG019061	4.64833992	4.38965424	4.49068553
NONHSAG019100	5.57964236	5.60231855	5.60517216
NONHSAG019129	5.99041593	6.12383206	6.01599356
NONHSAG019176	6.18007186	6.27816493	6.09238212

NONHSAG019180	2.61347179	2.81536192	2.42697139
NONHSAG019199	2.2234777	2.39494338	2.39598217
NONHSAG019275	2.97588597	3.50334826	3.07144586
NONHSAG019322	2.31583028	2.53242549	2.42085087
NONHSAG019335	2.86263835	3.17979675	3.42386804
NONHSAG019355	4.20836249	4.47575054	4.50897183
NONHSAG019359	3.2782934	3.06894843	2.96944201
NONHSAG019389	3.12817415	2.77803845	2.63382474
NONHSAG019395	3.39091724	3.26189354	3.5552037
NONHSAG019396	3.23914052	3.34952761	3.73412419
NONHSAG019423	3.48550535	3.54155536	3.69232146
NONHSAG019434	3.46840156	3.19768097	3.44664666
NONHSAG019460	2.84119172	2.73313334	2.8279733
NONHSAG019464	5.32375232	5.36155906	5.7813123
NONHSAG019471	5.25376256	5.40273305	5.30111323
NONHSAG019477	4.92231653	4.99637613	4.69705131
NONHSAG019496	3.24190323	3.23259552	3.56521107
NONHSAG019516	2.91968799	2.97256329	2.8377285
NONHSAG019517	2.38741204	2.5081571	2.51948198
NONHSAG019583	4.05853248	4.2158757	4.32626384
NONHSAG019638	3.07496353	3.13961756	3.21670839
NONHSAG019649	2.23435503	2.3255605	2.30423151
NONHSAG019657	2.56669551	2.54284538	2.55721528
NONHSAG019671	3.55620238	3.67621293	3.59493463
NONHSAG019696	2.65268457	2.65325392	2.57241711
NONHSAG019733	5.05461788	4.98974426	5.26420304
NONHSAG019747	4.06731852	4.18337645	3.90618935
NONHSAG019755	3.18041052	3.72573513	3.44204993
NONHSAG019799	2.90283362	2.51910928	2.87220207
NONHSAG019803	4.75773524	4.96264926	5.01090425
NONHSAG019855	8.27674877	8.1198062	8.09729894
NONHSAG019888	4.14945446	3.97403053	4.72127727
NONHSAG019921	3.42145587	3.31330993	3.59071173
NONHSAG019946	4.67931387	5.0952491	4.28012847
NONHSAG019954	2.70729979	2.48970498	2.70720678
NONHSAG020059	3.65877486	3.5730426	3.73289197
NONHSAG020065	3.32542535	3.1355906	3.10715792
NONHSAG020081	9.96382736	9.97763269	9.8549233
NONHSAG020082	3.27632076	3.31351136	2.90652954
NONHSAG020086	5.57839458	5.26264833	5.38989576
NONHSAG020094	2.43246359	2.20901731	2.33560693
NONHSAG020096	3.90288297	4.17753931	4.2480464
NONHSAG020099	8.06487934	8.14035021	8.01551085
NONHSAG020107	3.47274608	3.21971867	3.35560252
NONHSAG020130	2.32923348	2.55501818	2.6804936
NONHSAG020182	4.7811849	4.65376259	4.91721417
NONHSAG020190	3.77191511	3.81430267	4.14293746
NONHSAG020197	4.28676432	4.09636318	4.17935778
NONHSAG020211	5.43191876	5.44127039	5.65022989
NONHSAG020225	4.11850903	4.22274469	4.31898026
NONHSAG020230	2.82491211	2.81419874	2.87362839
NONHSAG020240	2.93662329	2.94963329	3.07531637
NONHSAG020323	4.6437314	5.0735972	5.05891581
NONHSAG020329	6.6433599	6.52855659	6.6517129
NONHSAG020334	4.21474162	4.19362844	4.39225566
NONHSAG020338	3.84981267	4.01897571	3.93809324
NONHSAG020339	6.82575895	6.91695589	6.98572696
NONHSAG020347	7.32855471	7.58341024	7.45344307
NONHSAG020419	3.64520344	3.54438199	3.46627986
NONHSAG020424	3.83028952	3.68619649	3.87450611
NONHSAG020428	3.14623779	3.03728753	3.24185942
NONHSAG020432	5.83921596	6.28892507	6.26295004
NONHSAG020434	3.17634683	3.47994255	3.53366345
NONHSAG020483	3.29233382	3.23963833	3.20542029
NONHSAG020539	4.92061489	5.20799263	5.2180927
NONHSAG020550	5.87284559	6.00675842	5.90893522
NONHSAG020567	3.88672407	3.99086484	3.94131697
NONHSAG020580	4.06261232	4.20108805	4.09995319
NONHSAG020618	4.65433056	5.27810811	4.85546053
NONHSAG020638	6.93086423	6.98768571	6.99289527
NONHSAG020650	2.67928108	2.39052292	2.75064271

NONHSAG020678	6.14324689	6.08750477	6.15464498
NONHSAG020737	3.33765716	2.9810798	3.16054821
NONHSAG020798	2.21282662	2.32713003	2.47503722
NONHSAG020806	4.27471286	4.41980647	4.37215252
NONHSAG020809	3.02592873	3.29568894	3.14761945
NONHSAG020830	4.21621264	4.21624777	4.07607068
NONHSAG020837	2.7082266	2.5160807	2.67963814
NONHSAG020855	3.24580098	3.10046028	3.12415094
NONHSAG020867	2.16341674	1.99795158	2.16964675
NONHSAG020883	3.87857724	4.02641474	3.99706735
NONHSAG020899	2.59684271	2.55342297	2.57083503
NONHSAG020905	3.12906282	3.22241653	3.37343823
NONHSAG020941	5.94426321	6.29825264	6.49931449
NONHSAG020993	5.17613509	5.04735516	5.25344094
NONHSAG021003	5.20182709	5.01264078	5.29725604
NONHSAG021015	5.54605719	5.52054869	5.86882282
NONHSAG021045	3.94668143	3.82506137	3.93654274
NONHSAG021143	4.08462837	4.40711768	4.63567026
NONHSAG021154	5.03816767	4.74652702	4.8706146
NONHSAG021213	4.64974746	4.55031333	4.96885244
NONHSAG021241	2.98323462	2.62376725	3.03977147
NONHSAG021246	5.98741997	5.91432054	5.94308504
NONHSAG021274	3.85933829	3.82841758	3.9005575
NONHSAG021287	5.36824516	5.45745397	5.69582412
NONHSAG021374	2.40543328	2.41134169	2.52716535
NONHSAG021403	5.30757873	5.42182753	5.63072357
NONHSAG021436	4.17895635	3.90682872	3.87548319
NONHSAG021444	5.39410406	5.67996511	5.50952277
NONHSAG021459	2.31676951	2.29685258	2.39321869
NONHSAG021464	3.62828944	4.06265036	3.27290066
NONHSAG021479	4.66795161	4.86931145	4.97081657
NONHSAG021502	3.31175814	3.27560285	3.42606278
NONHSAG021504	2.91535644	2.80755041	3.08877196
NONHSAG021505	3.46039172	3.45273823	3.37629518
NONHSAG021537	3.38028535	3.34949678	3.50120859
NONHSAG021546	3.96734181	3.71042584	4.20386958
NONHSAG021557	6.47429344	6.34621765	5.86289719
NONHSAG021577	4.88428971	4.587239	4.6440645
NONHSAG021582	5.0992916	4.68208654	5.08994679
NONHSAG021608	2.59629263	2.63845924	2.81228677
NONHSAG021662	4.94816751	4.72309444	4.85995485
NONHSAG021671	2.91372841	2.74916011	2.92552408
NONHSAG021696	3.79846136	3.86457164	3.88135457
NONHSAG021701	4.64385325	4.82761537	4.60231103
NONHSAG021755	6.44584955	6.23050561	6.13464759
NONHSAG021758	3.64484296	3.3506061	3.68099065
NONHSAG021801	5.62652338	6.0497514	5.60683622
NONHSAG021825	4.14357387	4.10926946	3.99192723
NONHSAG021851	4.13109833	4.34030476	3.92489251
NONHSAG021881	3.91451613	3.85218269	3.7035873
NONHSAG021904	4.27926258	4.30132945	4.42980552
NONHSAG021905	5.829715	5.97299059	5.78077486
NONHSAG021933	2.17940048	2.18588158	2.18266269
NONHSAG022026	3.18241612	3.22512313	3.31834298
NONHSAG022055	3.94161833	3.93680102	4.16964393
NONHSAG022065	4.95676567	5.07795558	5.30681824
NONHSAG022068	4.56608152	4.83713402	4.94692398
NONHSAG022073	2.51183378	2.5640547	2.83135875
NONHSAG022088	3.94883732	3.65314257	4.0711566
NONHSAG022089	2.90596379	3.09191225	2.99291467
NONHSAG022092	4.50895262	4.71667974	4.90614674
NONHSAG022177	4.90456404	5.0700954	5.0405211
NONHSAG022214	4.3433012	4.19862942	4.45651881
NONHSAG022224	4.2527353	4.28775282	4.24016873
NONHSAG022252	6.7255344	6.85621811	6.61339247
NONHSAG022256	3.83105429	4.15673122	3.61323992
NONHSAG022261	3.50793649	3.46430187	3.71235411
NONHSAG022297	4.40597091	4.10508898	4.37961412
NONHSAG022308	4.93458854	4.67897371	4.90477548
NONHSAG022311	5.59720542	5.31855809	5.35540611
NONHSAG022328	4.86191375	4.79629047	4.83388053

NONHSAG022337	5.01210149	4.89769526	5.13642957
NONHSAG022342	2.5437182	2.54257453	2.48506798
NONHSAG022353	3.32624978	3.30663206	3.41860349
NONHSAG022357	7.71224055	7.59324351	7.53503344
NONHSAG022358	6.48691725	6.91354258	6.40586818
NONHSAG022437	5.19619407	5.52636745	5.74204751
NONHSAG022492	8.00232457	8.08321611	7.73564518
NONHSAG022576	3.34258853	3.40208321	3.44619445
NONHSAG022580	5.07811585	4.99040659	5.27931964
NONHSAG022588	7.76958863	8.02126049	7.81485785
NONHSAG022627	3.60738718	3.874867	3.60957352
NONHSAG022655	3.22089548	3.44843551	3.5426963
NONHSAG022656	3.69578976	3.71809894	4.16435165
NONHSAG022668	4.31200736	4.12591376	4.33594194
NONHSAG022676	3.80708868	3.87700801	4.01264881
NONHSAG022677	3.99304097	3.9840433	3.80975353
NONHSAG022735	5.78312122	5.79868924	5.78602536
NONHSAG022759	8.67174379	8.6495722	8.55750154
NONHSAG022817	4.33483084	4.42953284	4.57781955
NONHSAG022830	5.03241563	5.11184981	4.94537176
NONHSAG022841	5.70693474	5.77716443	5.58865873
NONHSAG022852	3.12378205	3.33630908	3.60949646
NONHSAG022858	3.0409454	3.10154267	3.2601537
NONHSAG022867	5.15451964	5.20551975	5.51451777
NONHSAG022906	3.47540858	3.44613754	3.53498119
NONHSAG022924	5.8828743	5.9778783	6.2264479
NONHSAG022925	5.16984764	5.11175112	5.03784471
NONHSAG022957	4.20882159	4.25171437	4.46613236
NONHSAG022968	5.49401665	5.49164742	5.69282025
NONHSAG022969	3.24079014	3.4572	3.78640462
NONHSAG022994	5.72007899	5.74360827	5.76896831
NONHSAG023009	2.75957986	2.68506802	2.96800479
NONHSAG023036	5.98517434	5.76881506	5.96414609
NONHSAG023052	4.48457845	4.49057468	5.03690448
NONHSAG023067	5.69634908	5.58197332	5.69584944
NONHSAG023085	3.21683281	2.87905579	3.08251877
NONHSAG023088	5.76761241	5.51717419	5.80799106
NONHSAG023107	3.57077815	3.46534483	3.9844874
NONHSAG023114	3.68609989	3.71578637	3.46894559
NONHSAG023118	3.93314271	3.63022893	4.02815011
NONHSAG023129	6.22996682	6.29296817	6.57883107
NONHSAG023173	4.76325145	4.75091261	4.83846805
NONHSAG023180	2.97060434	3.31443924	3.4571014
NONHSAG023185	3.01920736	3.04592431	3.36183475
NONHSAG023194	2.72863927	2.45022056	3.29353027
NONHSAG023237	4.13123284	4.13523766	4.4080267
NONHSAG023238	3.40617215	3.18181883	3.21822373
NONHSAG023245	2.47710859	2.4642742	2.39106792
NONHSAG023247	2.30236039	2.14032407	2.18270163
NONHSAG023281	2.62429932	2.35569833	2.680552
NONHSAG023286	3.28161907	3.24800863	3.26283571
NONHSAG023298	3.17186765	3.49893748	3.42595773
NONHSAG023327	4.03926405	4.15673122	4.19009682
NONHSAG023350	3.5192286	3.54895221	3.36902776
NONHSAG023373	3.78487703	3.46765091	3.92489251
NONHSAG023390	5.12108055	5.27378821	5.26178225
NONHSAG023427	3.08648531	2.75372725	2.99266698
NONHSAG023436	6.16224598	6.14024617	6.44494226
NONHSAG023437	3.41933635	3.71699677	3.74245467
NONHSAG023456	2.65390413	2.88296067	2.97826569
NONHSAG023530	2.09896668	2.44379993	2.63020471
NONHSAG023534	2.71180538	2.74585005	2.65228665
NONHSAG023539	5.49401665	5.80689906	5.91682288
NONHSAG023582	3.42440762	3.36535516	3.23809566
NONHSAG023627	3.27786782	3.34798962	3.30027496
NONHSAG023639	2.39433592	2.28954803	2.50190906
NONHSAG023666	2.6122749	2.26308683	2.80580618
NONHSAG023691	2.81032799	3.07232342	2.69706661
NONHSAG023704	4.14813139	4.23013562	4.28813532
NONHSAG023707	2.60999809	2.33130987	2.81834637
NONHSAG023711	3.16123282	2.80365623	2.90015376

NONHSAG023714	3.64140243	3.69684844	3.92030946
NONHSAG023715	2.45007513	2.46102708	2.70946368
NONHSAG023765	2.82193839	2.98062068	3.21402966
NONHSAG023770	2.87714965	2.77896976	3.00111732
NONHSAG023771	2.79893434	2.86967256	2.89338157
NONHSAG023772	2.45949069	2.50664771	2.30167738
NONHSAG023787	4.92402853	5.08684184	5.05002789
NONHSAG023790	2.47138095	2.49828184	2.91943929
NONHSAG023801	4.55043942	4.6279446	4.70525468
NONHSAG023810	3.37831051	3.18980074	3.46231506
NONHSAG023839	4.80475357	5.1236757	5.12685764
NONHSAG023915	2.88160424	3.05425764	3.2560474
NONHSAG023920	4.37894337	4.36524372	4.81412081
NONHSAG023923	3.33765716	3.92526862	3.50845836
NONHSAG023954	5.31859914	5.15111752	5.59147816
NONHSAG023966	5.03965478	5.13923203	5.39739903
NONHSAG023972	3.69876176	4.05969038	4.27715181
NONHSAG024013	3.35304511	3.5362537	3.31762805
NONHSAG024040	5.08106099	5.14870946	5.10623731
NONHSAG024043	2.97060434	3.06228904	3.03567827
NONHSAG024046	4.85378117	4.91700012	4.99580388
NONHSAG024080	2.5978397	2.77501549	2.73305112
NONHSAG024104	2.28615825	2.29875734	2.42085087
NONHSAG024105	3.1435428	3.18297518	3.45968659
NONHSAG024135	4.14471241	4.11959797	4.4080267
NONHSAG024143	2.79214269	2.44813063	2.62574606
NONHSAG024146	2.76423925	2.64018074	2.99272667
NONHSAG024157	3.27886792	3.32345136	3.7033783
NONHSAG024162	2.5026231	2.57610083	3.07501639
NONHSAG024182	4.91912075	4.94318449	5.08272562
NONHSAG024209	5.72183091	5.67716033	5.75318644
NONHSAG024232	4.43113343	4.46729956	4.34803777
NONHSAG024234	3.47416484	3.4796744	3.59559908
NONHSAG024244	4.31032418	4.18537011	4.28118547
NONHSAG024248	6.2684509	6.18240323	6.07802332
NONHSAG024264	3.91242632	3.89172587	4.22935181
NONHSAG024327	3.60738718	3.23320454	3.15964628
NONHSAG024381	5.32495672	5.36784393	5.52936667
NONHSAG024388	5.18083266	5.52351507	5.45957514
NONHSAG024397	6.12673693	6.24432881	5.82683042
NONHSAG024439	5.71037316	5.86014489	6.24824474
NONHSAG024442	3.68945001	3.65582936	3.7470424
NONHSAG024446	3.53925175	3.51077555	3.52466898
NONHSAG024447	2.87124134	2.68896316	2.8761347
NONHSAG024529	7.35403349	7.73588558	7.49079644
NONHSAG024540	3.92987457	3.44523242	3.64026218
NONHSAG024542	4.97992472	5.30932475	5.162124
NONHSAG024613	4.22223267	4.45028267	4.49864432
NONHSAG024628	2.76238982	2.68631666	2.85836103
NONHSAG024729	3.38719826	3.52231519	3.45800005
NONHSAG024733	2.33685262	2.28542614	2.34003903
NONHSAG024740	2.9402451	2.91597414	2.62036082
NONHSAG024741	3.39832882	3.34283806	3.39776549
NONHSAG024742	8.98426574	8.96147461	9.08257031
NONHSAG024745	2.88759543	2.94387156	2.90039564
NONHSAG024752	4.23303404	4.45564781	4.16944535
NONHSAG024859	2.37369395	2.47452004	2.45986628
NONHSAG024870	2.82033491	2.74858602	2.6361477
NONHSAG024871	6.47931585	6.31111689	6.57188685
NONHSAG024916	4.32292093	4.5137573	4.81610111
NONHSAG024953	3.29034331	3.59106702	3.84660213
NONHSAG024968	3.47821864	3.57687883	3.94834746
NONHSAG024985	7.00089228	6.94806601	7.02666669
NONHSAG024988	5.72339121	5.87777072	5.88403318
NONHSAG024994	4.98156845	5.07092743	4.832625
NONHSAG025009	5.33343879	5.37948333	5.59436806
NONHSAG025049	5.04679164	5.34520752	5.12420691
NONHSAG025052	7.17896104	7.20757257	7.09857407
NONHSAG025054	7.05240165	6.83986987	7.01272341
NONHSAG025151	5.74611075	5.56096944	5.479224
NONHSAG025174	3.62495137	3.88432612	3.81264348

NONHSAG025195	7.1553106	7.01705023	6.62973594
NONHSAG025224	3.73289737	3.57894389	3.60483003
NONHSAG025230	3.08389786	2.86602237	2.7539483
NONHSAG025265	2.72040866	2.61709667	2.87023731
NONHSAG025272	4.71219333	4.6721072	4.97771586
NONHSAG025274	2.58515384	2.59376531	2.79344097
NONHSAG025288	2.43668207	2.75450997	2.63533036
NONHSAG025295	4.20311322	4.14024847	4.46155177
NONHSAG025343	3.80945648	3.77097867	3.59569816
NONHSAG025349	4.28123332	4.31291261	4.05208708
NONHSAG025352	3.34870654	3.23423437	3.59482443
NONHSAG025367	4.40137576	4.27889327	4.51209639
NONHSAG025376	5.98065782	5.77470932	5.66436355
NONHSAG025378	5.43253403	5.43087296	5.35042307
NONHSAG025393	4.49697824	4.5142009	4.65383162
NONHSAG025422	2.86722968	2.97060434	2.91195568
NONHSAG025431	4.6954294	4.60153815	5.05097095
NONHSAG025438	2.49784686	2.8977894	3.01031609
NONHSAG025448	4.86491716	4.83525384	5.09941415
NONHSAG025466	4.57427658	4.72021265	4.51953935
NONHSAG025491	4.71074675	4.90085059	4.93863211
NONHSAG025494	3.70557067	4.1160498	3.94582466
NONHSAG025500	4.68548135	4.69383426	5.25151415
NONHSAG025531	4.16103285	4.14453917	4.1895136
NONHSAG025536	3.60353573	3.45575182	3.22531731
NONHSAG025542	3.93714468	3.81750522	4.1866831
NONHSAG025649	3.59138348	3.95322503	3.90900935
NONHSAG025666	4.49748901	4.50104968	4.69594464
NONHSAG025675	3.40971282	3.43089003	3.48386885
NONHSAG025695	4.21027189	4.44905396	4.50585532
NONHSAG025699	2.49413123	2.45615007	2.42595644
NONHSAG025705	4.09378206	4.36942649	4.51456372
NONHSAG025710	2.73344318	2.5369896	2.80835117
NONHSAG025719	4.28662521	4.29381947	4.46410584
NONHSAG025790	5.30754995	5.36580169	5.24898345
NONHSAG025812	2.42330989	2.41060956	2.37549195
NONHSAG025869	4.09797674	4.29017456	4.17915823
NONHSAG025872	2.97399518	2.93469422	3.42675967
NONHSAG025881	2.22868279	2.30775338	2.34239397
NONHSAG025897	5.51768424	5.33398251	5.74240814
NONHSAG025979	2.47848421	2.54445221	2.87509721
NONHSAG025986	3.41370294	3.17549623	3.55803198
NONHSAG026033	2.96364658	2.82715425	3.06085824
NONHSAG026052	2.92029113	3.15816021	3.07865162
NONHSAG026094	4.93702285	4.93997388	4.65725472
NONHSAG026202	3.89451772	3.56471021	3.72234913
NONHSAG026216	3.63817167	3.64981141	3.9475034
NONHSAG026311	4.96760132	4.88394097	4.68666665
NONHSAG026360	4.16502441	3.90144327	4.07859745
NONHSAG026376	2.22384858	2.3789018	2.49872228
NONHSAG026396	3.70931746	3.47377215	3.09948413
NONHSAG026410	4.07353073	3.96293935	3.93002004
NONHSAG026448	2.45283474	2.39984913	2.18305776
NONHSAG026475	5.11892615	5.30629122	5.04631459
NONHSAG026539	2.37592761	2.26264654	2.41708978
NONHSAG026542	2.74250017	2.80640205	2.35960069
NONHSAG026552	6.85220579	7.05273958	6.86273914
NONHSAG026569	5.56111507	5.5170175	5.56426838
NONHSAG026592	4.11199085	4.27041064	4.49821883
NONHSAG026607	3.93769153	3.80367032	4.06937186
NONHSAG026618	4.65336525	4.35499383	4.802362
NONHSAG026620	3.15826657	3.44477386	3.32924402
NONHSAG026634	3.56300806	3.65021386	4.02447308
NONHSAG026661	6.52633347	6.80605432	6.25489013
NONHSAG026676	2.41607759	2.27600078	2.35130601
NONHSAG026716	7.98671374	8.05670857	7.8539837
NONHSAG026725	6.07293576	6.39862126	6.62998375
NONHSAG026729	4.3328903	4.18844448	4.46069357
NONHSAG026751	3.11804767	3.23232677	3.45689823
NONHSAG026771	4.38100722	4.69264636	4.94289358
NONHSAG026775	5.38981239	5.67996511	5.9342264

NONHSAG026798	2.53237025	3.08041254	2.64850382
NONHSAG026834	5.91347824	5.83478925	6.07978941
NONHSAG026851	5.68084375	5.93724998	5.93547193
NONHSAG026854	4.63148129	4.853799	4.86519835
NONHSAG026890	2.86797677	2.76686213	2.88779353
NONHSAG026912	5.24153213	5.53616629	5.64555167
NONHSAG026940	6.32373963	6.20252609	6.24935318
NONHSAG026942	4.69353732	4.37100919	4.3751848
NONHSAG026944	6.39288811	6.27923323	6.22010938
NONHSAG026956	4.80105258	5.07862592	5.3417942
NONHSAG026967	4.52305582	4.6484072	4.69705131
NONHSAG026969	2.56801183	2.57905528	2.84374746
NONHSAG026973	2.61904786	2.73848666	2.68164469
NONHSAG026985	3.66256068	3.5432773	3.7135492
NONHSAG026995	5.14136619	5.02414323	5.30755481
NONHSAG027017	3.31602582	3.19367323	3.01819967
NONHSAG027018	4.38070087	4.48175512	4.52031156
NONHSAG027025	4.7896455	4.88663486	4.91192392
NONHSAG027026	3.62397862	3.40590814	3.37629245
NONHSAG027036	3.93973982	3.98593715	4.34747142
NONHSAG027063	2.61917098	2.49907315	2.71657495
NONHSAG027074	3.74780562	3.94907794	4.17640632
NONHSAG027078	3.36607309	3.38023721	3.79498218
NONHSAG027116	2.53237025	2.5482807	2.54517047
NONHSAG027134	2.77690235	2.75026798	3.07508206
NONHSAG027135	2.19483546	2.09929322	2.29037282
NONHSAG027140	2.9243406	3.21640301	3.30027496
NONHSAG027170	3.62854136	3.59832327	4.10748809
NONHSAG027173	2.41657586	2.1671195	2.11474358
NONHSAG027186	4.43583507	4.05211073	4.46087256
NONHSAG027205	4.30376221	4.74612572	4.68576272
NONHSAG027215	3.15708092	3.25069466	3.43450715
NONHSAG027327	2.49419797	2.53188918	2.80733167
NONHSAG027345	2.5355415	2.37083747	2.52100879
NONHSAG027346	2.52933027	2.56974294	2.39287687
NONHSAG027354	3.31541551	3.32096989	3.35342248
NONHSAG027362	4.71319086	4.56310186	4.47007461
NONHSAG027390	4.27722911	4.38590881	4.59496651
NONHSAG027419	3.57721964	3.63151311	3.49744323
NONHSAG027464	3.97838747	4.10475316	3.97062453
NONHSAG027468	4.31860964	4.22087457	4.53728117
NONHSAG027505	3.44412201	3.58687312	3.67817867
NONHSAG027515	3.11119995	2.76564328	2.88675749
NONHSAG027519	6.40316288	6.28959956	5.93579172
NONHSAG027535	3.85013183	3.85013183	3.9755187
NONHSAG027545	2.52901203	2.56892036	2.5829061
NONHSAG027550	2.29916015	2.3441891	2.23637102
NONHSAG027551	2.60282213	2.8303357	2.82636989
NONHSAG027552	4.14630878	3.75434705	3.7825096
NONHSAG027560	2.66422691	2.58719148	2.9542048
NONHSAG027603	3.60453862	3.45193023	3.77144405
NONHSAG027626	6.04744654	6.14614881	6.38773904
NONHSAG027634	3.89766968	3.92481106	3.97327407
NONHSAG027639	5.4232942	5.47287593	5.52426379
NONHSAG027647	4.94840115	5.08988025	4.60607631
NONHSAG027670	2.49401514	2.39374984	2.17237977
NONHSAG027674	3.41665318	3.16665852	3.33265383
NONHSAG027684	4.54504073	4.3139867	4.22450128
NONHSAG027686	3.52032902	3.95756528	3.45770322
NONHSAG027687	2.93663877	3.23141129	3.15931671
NONHSAG027711	2.46232994	2.55457413	2.884343
NONHSAG027714	4.20506477	4.24097923	4.53171747
NONHSAG027715	4.83372874	4.92724909	5.06187648
NONHSAG027736	3.15977544	3.15404879	3.41352933
NONHSAG027755	3.46865232	3.28309428	3.45770322
NONHSAG027769	3.52738695	3.6566143	3.43439556
NONHSAG027811	3.91520334	4.00280329	3.74677037
NONHSAG027819	4.87343498	4.86420915	5.18560664
NONHSAG027821	2.29271613	2.15921477	2.41847293
NONHSAG027833	2.97467253	2.8979937	2.72606926
NONHSAG027841	6.34880868	6.59295938	6.45159825

NONHSAG027868	8.69859378	8.82977412	8.93434598
NONHSAG027869	8.39717768	8.35547361	8.36554338
NONHSAG027897	3.15745816	3.385336	3.17116332
NONHSAG027917	3.15379845	2.86758132	3.1464644
NONHSAG027919	4.11670445	4.56112869	4.65257726
NONHSAG027954	4.58642719	4.90691665	4.84036499
NONHSAG027959	3.11511591	3.02984422	3.1222906
NONHSAG027967	3.14735745	3.08088127	3.07543722
NONHSAG027970	4.73541403	5.03303289	5.16827368
NONHSAG027998	3.2288218	3.29198878	3.3584162
NONHSAG028005	3.57690576	3.74612955	3.98917107
NONHSAG028023	3.82441148	3.88706368	4.00148196
NONHSAG028024	2.48670741	2.60597175	2.72881725
NONHSAG028072	2.8801165	2.6319174	2.34959303
NONHSAG028118	3.72855976	3.61679444	3.77537281
NONHSAG028155	4.64489931	4.4345922	4.70273421
NONHSAG028160	6.88437998	6.68968038	6.58591716
NONHSAG028184	3.70709076	3.70896194	3.75528963
NONHSAG028242	3.85858736	4.01636866	4.11601423
NONHSAG028243	3.81917541	3.81726668	3.78342636
NONHSAG028249	2.21397941	2.48239411	2.38037766
NONHSAG028283	3.0905651	3.1207582	3.21670839
NONHSAG028291	4.22481656	4.53348761	4.59160612
NONHSAG028305	3.15745816	2.8507591	2.64019965
NONHSAG028340	2.79369569	3.09271737	2.92386209
NONHSAG028349	3.10886565	3.22070375	3.22646947
NONHSAG028408	2.84675094	2.94137376	2.98745091
NONHSAG028420	2.53237025	2.57455905	2.2804706
NONHSAG028439	4.04668691	4.1014739	4.25506175
NONHSAG028464	2.63093181	2.61077086	2.91023669
NONHSAG028465	5.11307286	5.25024513	5.20615354
NONHSAG028467	6.02754886	6.0379153	6.4000165
NONHSAG028469	3.92766967	3.97225375	3.88885852
NONHSAG028525	5.49304442	4.96033921	5.03222475
NONHSAG028576	4.63463405	4.55514827	4.5436439
NONHSAG028585	6.31181734	6.22192683	6.18448986
NONHSAG028593	6.31238715	5.604196	6.12213583
NONHSAG028595	5.69756565	5.82846158	5.53528144
NONHSAG028611	2.5215388	2.73623967	3.05036225
NONHSAG028627	7.61771016	7.71142304	7.81376604
NONHSAG028726	3.25951172	3.0942435	3.24543446
NONHSAG028734	3.7394171	3.86541904	4.04610904
NONHSAG028743	3.40278975	3.65530351	3.57732721
NONHSAG028794	3.09320082	3.07241003	3.46170496
NONHSAG028929	4.69705131	4.8106846	4.81548759
NONHSAG028931	2.46276768	2.53161185	2.57776116
NONHSAG028939	5.9041187	5.86511757	5.32809335
NONHSAG028975	5.99583832	5.91574763	6.15030507
NONHSAG028980	5.10765205	5.19744254	5.19405008
NONHSAG029002	7.41948647	7.19338715	7.38123002
NONHSAG029008	2.99283355	3.07343932	3.05181879
NONHSAG029029	8.38284237	8.29163356	8.19831503
NONHSAG029047	3.01735234	2.87291039	3.38370861
NONHSAG029080	4.93209685	4.95136947	5.26620241
NONHSAG029181	3.25001432	3.382318	3.12697985
NONHSAG029187	5.58613317	5.38343702	5.0347369
NONHSAG029193	4.13532128	4.17824107	3.98159408
NONHSAG029202	6.94511443	6.89944195	6.87535674
NONHSAG029206	4.21585821	4.41726553	4.32671395
NONHSAG029225	3.47824721	3.42391118	3.41970675
NONHSAG029338	5.88450089	5.56254082	5.76136034
NONHSAG029343	4.44005691	4.28526564	4.30883976
NONHSAG029351	4.1316592	4.47442773	4.03732982
NONHSAG029390	2.71472249	2.88686281	2.87073619
NONHSAG029407	3.20082485	3.48099803	3.06947345
NONHSAG029451	2.71776265	2.63647363	2.69027083
NONHSAG029458	5.72339121	5.69827634	5.1115446
NONHSAG029466	2.45007513	2.54196239	2.6851808
NONHSAG029469	4.21474162	4.13192386	4.59082476
NONHSAG029483	2.52774428	2.66263688	2.58343534
NONHSAG029505	2.76558894	2.83568167	2.42733371

NONHSAG029519	3.29085282	3.20179149	3.42751946
NONHSAG029550	2.94681036	3.03543202	2.74568365
NONHSAG029563	4.01675694	4.18367575	4.04682774
NONHSAG029600	6.20752589	6.15464208	6.17696809
NONHSAG029615	3.03094183	3.25041285	3.11638824
NONHSAG029629	5.97324273	5.85077346	5.93776637
NONHSAG029651	3.63512019	3.41966455	2.79744179
NONHSAG029656	3.04911489	2.92071835	3.09653591
NONHSAG029691	4.31145781	4.76154374	4.12654044
NONHSAG029703	3.76591049	3.98719266	3.44192219
NONHSAG029707	3.10911636	3.09953267	3.12612552
NONHSAG029741	2.42111047	2.44813063	2.45870599
NONHSAG029752	2.58118646	2.53498772	2.97672841
NONHSAG029789	2.3611278	2.38231099	2.42085087
NONHSAG029815	2.3866089	2.56718027	2.35130601
NONHSAG029818	2.77366102	3.036067	2.95527928
NONHSAG029852	5.29277136	5.70837085	5.50283709
NONHSAG029854	6.12538326	6.4078468	6.39626659
NONHSAG029904	2.81859906	2.81284854	2.88778824
NONHSAG029916	2.38082239	2.51261632	2.5888434
NONHSAG029920	3.41818857	3.2468069	3.42133864
NONHSAG029991	2.52829171	2.5084397	2.58042923
NONHSAG029995	2.95288487	3.10833487	3.21574705
NONHSAG030001	2.54047226	2.76783585	2.66503477
NONHSAG030002	2.37218253	2.37014245	2.35090979
NONHSAG030016	3.35701001	3.5869608	3.55241694
NONHSAG030028	2.26184584	2.08398806	2.23040527
NONHSAG030049	2.459406	2.45038515	2.53657912
NONHSAG030056	2.72735674	2.98884153	2.76785825
NONHSAG030133	2.33048366	2.14864591	2.50091413
NONHSAG030140	2.23187696	2.43641881	2.47189447
NONHSAG030166	3.24917499	3.4840092	3.20938157
NONHSAG030168	2.64611641	2.67299535	2.68392987
NONHSAG030187	2.59736958	2.41185636	2.3378778
NONHSAG030263	2.6840751	2.8887525	2.86274475
NONHSAG030278	6.52878773	6.45004996	6.58942253
NONHSAG030325	4.03634227	4.19169738	4.49342301
NONHSAG030326	3.11998641	3.19285798	3.03718353
NONHSAG030351	2.40718079	2.51659057	2.75649242
NONHSAG030358	3.0157219	3.14602397	3.095752
NONHSAG030374	2.5034016	2.59133503	2.54862012
NONHSAG030400	5.08774367	4.95431472	4.99089079
NONHSAG030406	4.95939084	4.77401379	5.06795817
NONHSAG030459	3.16169004	3.3594789	3.35610386
NONHSAG030469	5.18869721	5.32364686	5.22810974
NONHSAG030483	3.97000334	3.95399483	4.36746863
NONHSAG030489	3.38113681	3.27324845	3.62615953
NONHSAG030493	2.56577022	2.58525406	2.56785511
NONHSAG030495	3.60738718	3.63927261	3.80710866
NONHSAG030509	3.02248268	2.90148831	2.99621914
NONHSAG030613	2.410386	2.33362544	2.62173594
NONHSAG030629	3.52053075	3.66296952	3.62710975
NONHSAG030633	8.77631216	8.65909339	8.46681792
NONHSAG030634	5.97652823	5.71396618	5.43878899
NONHSAG030637	3.37572326	3.44167999	3.55172988
NONHSAG030676	2.50430349	2.39910185	2.43576854
NONHSAG030681	4.56314976	4.41045097	4.68245559
NONHSAG030683	4.04992388	4.05895552	4.07925925
NONHSAG030688	2.43433582	2.4649526	2.45841587
NONHSAG030757	6.50284719	6.31967277	6.50585118
NONHSAG030767	6.52972781	6.43545843	6.77535695
NONHSAG030783	4.64608756	4.44311062	4.56058837
NONHSAG030819	3.50296147	3.1715414	3.26011526
NONHSAG030850	2.33836865	2.22860078	2.20319713
NONHSAG030878	3.996904	4.03912494	4.12908388
NONHSAG030888	5.29926654	5.242687	5.46574616
NONHSAG030933	4.28001544	4.39624912	4.74924043
NONHSAG030937	4.10508235	3.95999294	4.300784
NONHSAG030940	3.33383052	3.51614032	3.10114498
NONHSAG030957	3.93391369	3.89698314	3.98098296
NONHSAG030971	4.36754405	4.309144	4.41124116

NONHSAG030992	5.47827905	5.46800066	5.8257228
NONHSAG031103	4.12877569	4.32942198	4.37443605
NONHSAG031137	3.63783352	3.41029614	3.4399753
NONHSAG031167	4.21182247	4.42491194	4.13793728
NONHSAG031168	5.41362941	5.59782982	5.51054671
NONHSAG031178	3.1662929	3.15234066	3.09685159
NONHSAG031180	6.5894909	6.54075115	6.53555505
NONHSAG031184	2.96937464	3.16876683	3.19316763
NONHSAG031203	3.61559287	3.74473149	4.06212643
NONHSAG031219	2.89618814	2.67919863	2.8628664
NONHSAG031232	3.93403122	3.83153418	4.10438756
NONHSAG031237	2.81474656	2.91141361	2.94345384
NONHSAG031249	3.95571476	3.62158404	3.70442063
NONHSAG031255	2.2464837	2.1563921	2.32367632
NONHSAG031276	5.53220213	5.29543755	5.57787942
NONHSAG031296	2.51175111	2.58733236	2.8005112
NONHSAG031312	2.76487208	2.87929793	3.14714812
NONHSAG031314	3.14650185	2.71106975	3.26265662
NONHSAG031315	7.17499224	7.22781039	7.53084976
NONHSAG031322	6.00151365	6.02754922	6.50880104
NONHSAG031335	3.48493822	3.59387047	3.65102981
NONHSAG031372	2.59191466	2.59027883	2.62712954
NONHSAG031377	5.63633271	5.30873409	5.56188781
NONHSAG031385	5.25222588	5.27473419	5.02643491
NONHSAG031401	2.2989981	2.44754588	2.48223992
NONHSAG031461	3.0701292	3.09357928	3.15220889
NONHSAG031476	4.90948179	4.82123334	4.95132111
NONHSAG031485	4.69297065	4.65763758	4.92463667
NONHSAG031489	3.66324921	3.2922428	3.70414674
NONHSAG031516	6.07226148	6.06890246	6.14032624
NONHSAG031596	5.9719053	6.10502075	5.84357223
NONHSAG031603	2.92459128	3.1170606	3.14533029
NONHSAG031617	5.80955069	5.88297248	5.77166121
NONHSAG031626	1.70758428	1.89541906	2.41677854
NONHSAG031665	2.69954093	2.95972747	2.87993341
NONHSAG031701	4.08160058	3.98629926	3.91198938
NONHSAG031748	4.52159506	4.38959778	4.66539648
NONHSAG031783	7.14942594	7.11524654	7.0592883
NONHSAG031784	4.64028955	4.55945677	4.64286989
NONHSAG031801	4.1452328	4.08260711	4.316313
NONHSAG031805	2.65387135	2.60884735	2.62866663
NONHSAG031818	5.34705341	5.12415622	5.27842025
NONHSAG031824	6.83861062	6.70456134	6.8903945
NONHSAG031831	4.27076651	4.11269871	4.31814663
NONHSAG031845	5.20649768	5.41010465	5.08623788
NONHSAG031890	3.38517151	3.31529854	3.30027496
NONHSAG031913	5.03066836	5.12901944	5.1399599
NONHSAG031945	4.96714991	5.33134297	4.90216231
NONHSAG031951	4.09409045	4.09822341	4.3879195
NONHSAG031952	4.33112008	4.37535157	4.30374215
NONHSAG031964	4.21005345	4.36928571	4.38171426
NONHSAG032009	3.56469582	3.28211193	3.67257212
NONHSAG032015	5.32946558	5.13408977	5.15579631
NONHSAG032104	5.02336614	5.06825692	5.19756132
NONHSAG032106	4.13464675	4.11278357	4.31362707
NONHSAG032135	5.28060737	5.75280922	5.43318977
NONHSAG032143	2.50054046	2.49831288	2.63592884
NONHSAG032153	4.00419835	4.25118102	4.20690007
NONHSAG032186	3.34332738	3.32594897	3.55126635
NONHSAG032194	2.50241164	2.74435926	2.55155792
NONHSAG032197	3.89396331	3.94308043	3.90502483
NONHSAG032235	4.68739611	4.52527362	4.61676448
NONHSAG032245	3.78591911	3.80615672	3.84633356
NONHSAG032250	4.02939549	3.70798949	4.35658923
NONHSAG032270	4.49862691	4.54844662	4.66303664
NONHSAG032285	3.8382955	3.76700064	3.84633356
NONHSAG032299	4.51284854	4.53598255	4.5503311
NONHSAG032337	7.58190012	7.21981887	7.77152917
NONHSAG032387	2.26330253	2.39985486	2.69647523
NONHSAG032422	4.78538261	5.0210789	5.21954521
NONHSAG032427	2.71816489	2.67343012	2.72160925

NONHSAG032431	4.82595482	5.11175112	5.20533708
NONHSAG032480	3.77989231	3.51581552	3.93217082
NONHSAG032559	2.83597424	3.23320454	3.01876581
NONHSAG032592	2.45423288	2.58363048	2.53143219
NONHSAG032593	4.27209843	4.13218747	4.3732262
NONHSAG032601	3.29465834	3.36375874	3.3979758
NONHSAG032608	6.11159639	6.24453565	6.44287572
NONHSAG032613	3.89062433	3.8601912	3.81129073
NONHSAG032620	2.72746489	2.53584693	2.53935702
NONHSAG032629	2.26487452	2.30697899	2.31626042
NONHSAG032632	3.72071587	3.65714088	3.78518126
NONHSAG032643	4.06714639	4.09599722	4.5146129
NONHSAG032649	2.94019613	3.09475351	3.42648727
NONHSAG032662	2.69124935	2.83877023	2.91685977
NONHSAG032723	3.33492287	3.16499119	3.16283589
NONHSAG032740	4.33130082	4.34005525	4.21262838
NONHSAG032746	5.75670102	6.17824606	6.02984766
NONHSAG032750	3.02815808	3.1081651	3.34265716
NONHSAG032785	5.11263946	5.14293539	5.47953477
NONHSAG032791	3.78432654	3.97286512	3.78731005
NONHSAG032815	2.2673098	2.15752588	2.21587221
NONHSAG032817	3.41212799	3.26390514	3.65776323
NONHSAG032839	3.04339657	2.9130613	3.059379
NONHSAG032855	4.61580396	4.7068971	4.91555278
NONHSAG032935	7.40828853	7.53350265	6.94518915
NONHSAG032949	4.23928917	4.47030316	4.78423818
NONHSAG032951	3.17843334	3.05911712	3.21974667
NONHSAG032956	6.28636422	6.31443914	5.3018082
NONHSAG032959	3.59218287	3.43140115	3.77813352
NONHSAG032960	3.67740618	3.84932431	4.16101098
NONHSAG032961	5.17055157	4.87235618	5.15697566
NONHSAG032979	5.58881132	5.24429893	5.53839494
NONHSAG032984	3.73208942	3.59931243	3.69994568
NONHSAG032988	4.11324106	4.14469452	3.97594833
NONHSAG033011	2.8783787	3.05810037	3.10128972
NONHSAG033026	4.59476274	4.84475726	4.89108888
NONHSAG033060	3.17773172	3.61651028	3.30027496
NONHSAG033068	4.32859487	4.11787553	4.31960662
NONHSAG033071	6.4419778	6.20605038	6.47884458
NONHSAG033082	2.9524151	3.20283617	3.21670839
NONHSAG033092	3.52919506	3.50795818	3.71263604
NONHSAG033099	7.13031177	7.33888673	7.47344516
NONHSAG033167	4.16941416	3.81082336	4.17736146
NONHSAG033226	4.22113923	3.85222625	3.5204597
NONHSAG033228	4.10081662	3.97619638	4.26615601
NONHSAG033247	3.88070113	4.17663734	4.28843398
NONHSAG033254	4.82012426	4.74409577	5.05892342
NONHSAG033260	3.70926425	3.62781643	3.81524489
NONHSAG033274	2.61237282	2.51795459	2.4330373
NONHSAG033291	5.05334408	5.10713341	5.20304112
NONHSAG033405	4.33804059	4.20258243	4.17021614
NONHSAG033416	4.90836408	4.45108743	4.58268836
NONHSAG033484	5.73710579	5.50423755	5.04628313
NONHSAG033545	2.31497917	2.64103167	2.38165423
NONHSAG033550	4.25592911	4.23551484	4.44441294
NONHSAG033553	4.77042455	4.64051706	4.42368052
NONHSAG033558	2.21624205	2.17549411	2.45344237
NONHSAG033585	3.50324941	3.60808021	3.34130884
NONHSAG033587	4.84264687	5.01312027	5.15401041
NONHSAG033588	5.44736767	5.36284799	5.38796234
NONHSAG033592	3.2934056	3.48230316	3.69937918
NONHSAG033593	3.02225801	3.1715414	3.17634894
NONHSAG033604	3.70074194	3.54615825	3.78388758
NONHSAG033616	5.49838079	5.42182753	5.50583042
NONHSAG033639	4.12046992	3.76106131	3.95301514
NONHSAG033752	3.70409543	3.63060606	3.92068951
NONHSAG033788	4.36400244	4.09598057	4.29822312
NONHSAG033805	4.53100731	4.35722277	4.65980686
NONHSAG033897	5.07811585	4.89772519	5.30259541
NONHSAG033898	5.51599529	5.50898301	5.88469093
NONHSAG033967	4.74865204	4.61727049	4.90062408

NONHSAG033980	3.33835791	3.5517112	3.82307023
NONHSAG033989	5.74621957	5.3414899	5.56595553
NONHSAG034032	3.81554315	3.6221681	3.74552142
NONHSAG034050	5.29432935	5.18129275	5.10947077
NONHSAG034053	5.23069499	4.99276508	5.20655475
NONHSAG034077	5.59197802	5.52686314	5.45402747
NONHSAG034081	3.66319101	3.20466325	3.62823944
NONHSAG034105	3.27901847	3.08321237	3.20115692
NONHSAG034111	4.78540857	4.72333744	4.89359397
NONHSAG034128	3.22626121	3.24204186	3.19969454
NONHSAG034158	3.81636678	3.98980862	4.00593609
NONHSAG034163	2.94058403	2.77208188	2.80179243
NONHSAG034175	5.63464454	5.64696773	5.75996776
NONHSAG034179	6.34934374	6.24217652	5.84073587
NONHSAG034184	4.17631712	3.89813656	4.27717093
NONHSAG034187	4.71383914	5.04483119	5.13170136
NONHSAG034193	4.43243403	4.49718905	4.263883
NONHSAG034209	6.04502284	5.99387484	6.1621027
NONHSAG034242	5.2873617	5.62742979	5.30204556
NONHSAG034302	7.69844095	7.77957392	7.10737867
NONHSAG034336	2.65685812	2.82149808	2.96382997
NONHSAG034346	2.51511874	2.86449497	2.64212003
NONHSAG034364	3.14651828	3.14292361	3.09863057
NONHSAG034372	7.01319911	7.04676321	6.76795101
NONHSAG034414	4.42398751	4.46808658	4.76579002
NONHSAG034441	4.10908474	4.01075852	4.20747087
NONHSAG034453	4.23385176	4.12184998	4.21185197
NONHSAG034459	4.81103024	5.07159751	5.111647
NONHSAG034470	3.77439522	3.43722107	3.61067808
NONHSAG034501	5.26663487	5.11198783	5.68110454
NONHSAG034507	2.18014108	2.29480318	2.3953861
NONHSAG034552	4.51958521	4.44425452	3.8890294
NONHSAG034566	5.11444351	5.21159254	5.30079147
NONHSAG034569	2.84615044	2.75257364	3.11651673
NONHSAG034584	2.89377455	2.76954614	2.92643429
NONHSAG034600	3.53500512	3.27597452	3.4403299
NONHSAG034618	3.88431846	4.12214354	4.25536384
NONHSAG034646	2.26041767	2.09273409	2.30344905
NONHSAG034655	2.46305134	2.2958019	2.28243922
NONHSAG034677	2.77384281	2.87220392	3.09453858
NONHSAG034678	2.31712893	2.52924329	2.77529705
NONHSAG034689	4.66713448	4.40337995	4.37465832
NONHSAG034701	3.64035066	3.92646013	3.85674054
NONHSAG034702	4.18838426	4.55806694	4.51971676
NONHSAG034714	4.41354577	4.46387306	4.49329215
NONHSAG034778	2.84318398	2.85315647	2.96361097
NONHSAG034831	6.87464813	6.43453281	6.37069506
NONHSAG034858	2.98570219	3.152721	3.26228889
NONHSAG034900	2.85757542	2.87771832	2.93472399
NONHSAG034902	2.9104237	3.24281152	3.1963635
NONHSAG034909	4.90144698	4.78806451	4.9435007
NONHSAG034919	3.5192286	3.59325045	3.74214308
NONHSAG034946	3.50971255	3.75026617	4.24917093
NONHSAG034982	4.58995917	4.62176437	4.5883348
NONHSAG035114	3.72376371	3.81140529	4.2393573
NONHSAG035116	5.20946856	5.37547795	5.41846454
NONHSAG035135	2.94874607	2.73532859	2.9143473
NONHSAG035143	6.64829731	6.56869742	6.60147934
NONHSAG035159	5.30929281	5.41092674	5.44436589
NONHSAG035266	2.26587816	2.24986787	2.21444058
NONHSAG035270	4.23801667	4.14004099	4.33020436
NONHSAG035294	3.72235059	3.85939372	3.94957482
NONHSAG035333	3.67531004	3.74635483	4.07774941
NONHSAG035347	4.32807825	4.46445118	4.59487411
NONHSAG035395	2.55441999	2.58708052	2.57756937
NONHSAG035415	4.49955136	4.60505627	5.00267615
NONHSAG035432	4.83721124	4.87013392	4.83243445
NONHSAG035436	2.46210327	2.62245505	2.82972668
NONHSAG035491	3.18215854	3.28253439	3.65760832
NONHSAG035503	2.06615405	2.24765943	2.26393981
NONHSAG035505	3.56763509	3.06867795	3.6394607

NONHSAG035508	2.40366313	2.20877706	2.52448
NONHSAG035519	4.6971062	4.69943744	4.86208506
NONHSAG035522	6.10710993	6.14326853	5.58330171
NONHSAG035547	4.30611194	4.06223381	4.40770043
NONHSAG035579	2.47074418	2.19825337	2.48990749
NONHSAG035588	4.64201694	4.97449354	4.81109083
NONHSAG035621	3.70877555	3.5973883	3.85548866
NONHSAG035667	3.42909758	3.29334179	3.34174528
NONHSAG035675	2.89493978	3.79753922	3.06348954
NONHSAG035700	2.56515272	2.48161654	2.66528456
NONHSAG035708	2.30075889	2.37328044	2.41162411
NONHSAG035712	2.57675506	2.51336755	2.84141709
NONHSAG035715	2.2282553	2.11711692	2.3158642
NONHSAG035722	4.33585747	4.079553	4.67966891
NONHSAG035731	2.20435758	2.14783773	2.30299436
NONHSAG035768	5.90548684	6.17379252	5.64646307
NONHSAG035770	6.00151365	6.37288988	6.3834604
NONHSAG035786	3.11677026	3.19459089	3.2854391
NONHSAG035791	2.99949795	3.2048477	3.3461958
NONHSAG035807	2.48233739	2.40147171	2.55120932
NONHSAG035911	10.06499359	10.01880283	10.1585751
NONHSAG035915	2.4057953	2.37484889	2.54687456
NONHSAG035927	2.72438021	2.60653718	2.80954573
NONHSAG035942	2.5284844	2.76768751	2.68080401
NONHSAG035943	5.24959998	5.14687062	5.46496373
NONHSAG035971	4.9765425	4.73421171	3.98073746
NONHSAG035976	5.08246608	5.35606908	5.60018394
NONHSAG035982	5.04599003	5.35593122	5.2856496
NONHSAG036030	3.35217464	3.35049522	3.36339792
NONHSAG036052	6.02612015	6.05309637	5.87442452
NONHSAG036065	2.43300459	2.2732465	2.42632986
NONHSAG036067	8.25529639	8.29607854	7.98546542
NONHSAG036098	5.17378701	5.01825127	4.86772039
NONHSAG036130	3.25682294	3.58678882	3.17421063
NONHSAG036141	5.41215316	5.50110604	5.37513519
NONHSAG036146	5.24454481	5.30217255	5.67757216
NONHSAG036147	4.40241339	4.33486819	4.59274986
NONHSAG036184	3.06802729	3.37801756	3.15405526
NONHSAG036217	4.40443517	4.51647281	4.57913847
NONHSAG036227	8.14564128	7.70035818	7.44976614
NONHSAG036242	3.15745816	2.97060434	3.32262086
NONHSAG036289	3.27234737	3.2154273	3.08622424
NONHSAG036290	2.3983101	2.62993515	2.36665652
NONHSAG036298	3.57749362	3.59752261	3.37907578
NONHSAG036307	2.70199994	2.80760922	3.05293128
NONHSAG036327	3.15366882	3.13876544	3.18119217
NONHSAG036374	3.38295562	3.15504726	3.55220026
NONHSAG036383	6.37189972	6.57141794	6.12020868
NONHSAG036413	2.25369091	2.21465903	2.34243089
NONHSAG036444	2.70899718	2.73874606	2.67411901
NONHSAG036455	2.55588412	2.43770832	2.80363806
NONHSAG036462	3.48961975	3.55523271	3.56432437
NONHSAG036486	2.78814487	2.38823574	2.64561234
NONHSAG036538	3.23493086	3.14115671	3.48803689
NONHSAG036539	6.54285942	6.54646469	6.33180383
NONHSAG036566	2.96579406	2.9166454	2.90087049
NONHSAG036587	3.4610432	3.39853414	3.70703456
NONHSAG036631	5.40341636	5.50715788	5.49889074
NONHSAG036645	2.15107848	2.12740379	2.19430585
NONHSAG036667	2.91692	2.67096288	2.66950585
NONHSAG036669	3.48568156	3.38675735	3.28744118
NONHSAG036696	2.76412666	2.24967903	2.30580428
NONHSAG036703	2.21531809	2.16792111	2.24446818
NONHSAG036710	3.07402317	2.69424316	2.86116778
NONHSAG036741	2.10015509	1.963141	1.88549903
NONHSAG036745	3.79561658	3.91647206	3.98465702
NONHSAG036767	2.82427706	2.70369776	2.80397154
NONHSAG036776	3.31334428	3.33973712	3.56617632
NONHSAG036803	2.92552599	2.99063152	3.0813117
NONHSAG036808	4.29690897	4.06813058	4.19813986
NONHSAG036869	4.52759979	4.49890624	4.73663788

NONHSAG036874	2.8040915	3.20484364	3.41682487
NONHSAG036892	2.73458273	2.76714802	2.9368627
NONHSAG036926	6.46785301	6.37460114	6.76577943
NONHSAG036955	3.29917332	3.3035435	3.32424729
NONHSAG037015	3.2259639	3.3762881	3.61628802
NONHSAG037017	4.49467386	4.56714704	4.77170897
NONHSAG037020	3.42091748	3.4511483	3.37606783
NONHSAG037021	2.37541044	2.44208634	2.47459468
NONHSAG037022	6.83794797	6.91593791	7.23601098
NONHSAG037025	2.53237025	2.75056902	2.90466126
NONHSAG037030	5.7630084	6.23331	6.05130279
NONHSAG037031	5.41607779	5.518617	5.58529123
NONHSAG037032	3.28034354	3.36731125	3.13976089
NONHSAG037083	3.46582437	3.47435852	3.52592356
NONHSAG037087	7.45248508	7.4839588	7.38205667
NONHSAG037150	4.46747232	4.60365223	4.64054483
NONHSAG037173	7.56902445	7.76629064	7.27799399
NONHSAG037197	3.39002383	3.36793617	4.49210556
NONHSAG037200	5.87395277	5.8767056	5.72623244
NONHSAG037224	6.49949043	6.85133907	6.91057141
NONHSAG037227	5.76216764	5.65245471	5.77351734
NONHSAG037229	4.92493351	5.19277688	5.21897775
NONHSAG037251	2.5896158	2.64895813	2.70670517
NONHSAG037262	5.91996671	6.00797673	6.32458856
NONHSAG037272	4.58736178	4.53855472	4.42719494
NONHSAG037287	4.92688451	4.78836599	4.54623217
NONHSAG037322	2.68252545	2.56011899	2.41028631
NONHSAG037336	3.51795142	3.54370959	3.51270461
NONHSAG037338	5.05489673	5.096005	5.19742186
NONHSAG037352	5.37482447	5.21341408	5.46577274
NONHSAG037375	2.75168065	2.78363004	3.52087329
NONHSAG037376	3.68112725	3.47118464	3.55656266
NONHSAG037395	7.48830022	7.32621973	7.13357581
NONHSAG037396	5.68340447	5.89631679	5.93095165
NONHSAG037407	4.64135637	4.95825863	4.53559057
NONHSAG037426	4.72838839	4.64122568	4.90695803
NONHSAG037431	4.68057773	4.46715434	4.83942778
NONHSAG037432	6.47966468	6.60292562	6.19464634
NONHSAG037433	4.73399475	4.78177193	4.64850213
NONHSAG037464	3.88972954	4.00216013	3.99706735
NONHSAG037470	3.72021705	4.09764377	3.87811762
NONHSAG037504	3.23125972	3.45610435	3.24122648
NONHSAG037513	3.03615083	3.43059022	3.0854128
NONHSAG037525	3.89926702	3.72804808	3.96053897
NONHSAG037563	4.6373749	4.85222379	4.46410584
NONHSAG037570	3.49572793	3.75785076	3.49518357
NONHSAG037575	5.38203349	5.04765479	5.03784471
NONHSAG037577	3.46345823	3.62066018	3.62893752
NONHSAG037622	3.247747	3.19627106	3.32551883
NONHSAG037629	4.74461373	4.29207684	4.69705131
NONHSAG037701	3.07856394	3.188678	3.12978201
NONHSAG037719	2.93103137	2.83787174	2.95052613
NONHSAG037724	2.06230243	2.26247236	2.34941394
NONHSAG037726	2.26051737	2.19353377	2.20284108
NONHSAG037747	3.8977349	4.02652673	3.97137524
NONHSAG037751	4.13534472	4.15106043	4.28012847
NONHSAG037786	8.14152765	8.07592639	7.96726007
NONHSAG037791	2.41438392	2.55158122	2.42085087
NONHSAG037804	3.78573989	3.73107329	3.47348238
NONHSAG037839	5.77781647	5.78661016	5.21260945
NONHSAG037842	2.91482399	3.2215999	3.45837958
NONHSAG037849	3.06413224	3.23320454	3.42590122
NONHSAG037860	3.48165445	3.39679347	3.57281978
NONHSAG037870	2.28907306	2.3488595	2.27223022
NONHSAG037873	2.29916015	2.22845979	2.50487148
NONHSAG037878	2.27283503	2.28136139	2.37267139
NONHSAG037943	2.9406301	2.98012784	2.91446377
NONHSAG037955	2.27701828	2.21600439	2.19623336
NONHSAG037959	3.09395837	3.15156894	2.94723476
NONHSAG037962	4.08962775	3.7348137	4.21935402
NONHSAG037977	6.67602632	6.94624589	6.88597096

NONHSAG037987	2.19868334	2.33098701	2.36167055
NONHSAG037991	3.88573704	3.74359694	4.22804188
NONHSAG038027	3.46305243	3.45128192	3.65627861
NONHSAG038035	2.23078	2.16352759	2.26897923
NONHSAG038048	2.54402234	2.3672988	2.38057421
NONHSAG038071	4.11329835	3.88887709	4.10583183
NONHSAG038091	3.48201611	3.35346484	3.36902776
NONHSAG038132	4.62168437	5.11646918	5.10207346
NONHSAG038179	3.59371911	3.40604472	3.70606142
NONHSAG038210	3.96017211	3.85651275	4.48226133
NONHSAG038213	2.82368887	2.74910192	2.67352518
NONHSAG038259	3.4905646	3.61814708	3.47660931
NONHSAG038285	3.57956876	3.48521515	3.60385993
NONHSAG038292	2.44662006	2.36867492	2.37637078
NONHSAG038333	5.41845823	5.30528753	5.6633574
NONHSAG038350	3.16872387	3.05954631	3.43694885
NONHSAG038364	3.04181678	3.29479416	3.60039466
NONHSAG038417	7.06371199	7.29856019	6.96840887
NONHSAG038444	2.83808662	2.63647363	2.77972953
NONHSAG038460	10.07418568	9.89262243	9.89132321
NONHSAG038476	3.4500002	3.39675563	3.4213638
NONHSAG038511	2.3107222	2.4115998	2.43749449
NONHSAG038512	4.56608152	4.51261286	4.62681399
NONHSAG038522	5.61449152	5.50373965	5.96809585
NONHSAG038586	5.76979218	5.36662574	5.23387443
NONHSAG038591	2.65560959	2.63699217	2.69583093
NONHSAG038599	2.96804054	2.83576946	3.20765231
NONHSAG038627	6.67791278	6.43734114	6.35848388
NONHSAG038649	3.52206048	3.59430399	3.5713427
NONHSAG038724	2.48510225	2.70571212	2.69027083
NONHSAG038765	3.98125694	4.20015119	4.466243
NONHSAG038779	3.41721645	3.58746445	3.55107299
NONHSAG038780	2.14029269	2.15594391	2.28096745
NONHSAG038818	2.18481652	2.22588425	2.3554256
NONHSAG038845	5.56971458	5.42182753	5.54210452
NONHSAG038869	2.36465898	2.16879547	2.24348459
NONHSAG038874	2.4106103	2.46512774	2.35077708
NONHSAG038882	2.49069939	2.52995659	2.62120506
NONHSAG038890	6.07240303	6.3408649	6.0183246
NONHSAG038899	2.70241552	2.53845187	2.76519536
NONHSAG038901	3.70557067	3.78698462	3.95316769
NONHSAG038916	2.25235105	2.35290181	2.34854289
NONHSAG038917	2.29916015	2.51804771	2.38579653
NONHSAG038920	3.61689242	3.68526199	3.57046021
NONHSAG038949	4.80772549	5.00666637	4.71536706
NONHSAG038955	2.58959565	2.64088292	3.23049641
NONHSAG038958	2.70899718	2.550279	2.75648853
NONHSAG038962	3.30715496	3.55760107	3.51304888
NONHSAG038974	3.94396602	4.03840832	4.31442473
NONHSAG038975	3.41211454	3.39344503	3.51754957
NONHSAG038999	2.31969443	2.24329712	2.1723971
NONHSAG039007	2.13584873	2.44005583	2.21027309
NONHSAG039009	2.79368794	2.27968218	2.13339759
NONHSAG039017	3.15745816	2.91338803	3.35510895
NONHSAG039019	3.05253863	3.3162075	2.98808898
NONHSAG039034	3.37852409	3.68342172	3.36902776
NONHSAG039040	2.57409957	2.87327705	2.64270614
NONHSAG039046	3.15907689	3.2693748	3.30027496
NONHSAG039047	2.93508773	2.9045598	2.62173594
NONHSAG039060	3.11357596	3.10993661	3.02872521
NONHSAG039061	2.51837626	2.87842761	2.82094707
NONHSAG039063	3.01271667	2.96507905	2.88346862
NONHSAG039082	4.16819416	4.04565323	4.21252716
NONHSAG039102	3.3248485	3.12222045	3.45068188
NONHSAG039105	4.84275113	4.68721745	4.65991569
NONHSAG039107	5.62686323	5.70040859	5.58831428
NONHSAG039111	2.86984201	2.83576946	3.07207866
NONHSAG039112	4.3598255	4.58635215	4.92005939
NONHSAG039130	3.25999255	3.28942874	3.17017514
NONHSAG039135	4.30316622	4.58926024	4.77991248
NONHSAG039189	2.09410843	1.95943134	2.00197557

NONHSAG039190	3.4634383	3.49935276	4.21474162
NONHSAG039191	2.70899718	2.73208891	2.73497917
NONHSAG039225	3.22477119	3.4281634	3.57088306
NONHSAG039294	2.73614026	2.52608036	2.75273681
NONHSAG039315	4.35158691	4.33268305	4.50089228
NONHSAG039321	2.66913436	2.63392949	3.11969203
NONHSAG039327	3.77733073	3.95018305	3.95975237
NONHSAG039332	2.81260554	2.65583744	2.91775112
NONHSAG039337	3.22881359	2.90265306	2.83441312
NONHSAG039356	2.63922756	2.6868235	2.82216274
NONHSAG039358	4.18711901	4.12722226	4.31494006
NONHSAG039367	3.57714556	3.7276768	3.12673497
NONHSAG039377	3.54477691	3.90938969	4.10810359
NONHSAG039399	5.78302627	5.76787754	5.48617527
NONHSAG039414	2.99790105	2.7790056	2.85457347
NONHSAG039470	4.58648424	4.64683915	4.36960809
NONHSAG039489	2.65420152	2.81685802	2.77357072
NONHSAG039490	4.44197241	4.42752869	4.18065564
NONHSAG039502	4.0430506	3.91323978	4.59312362
NONHSAG039503	5.54565832	5.30512165	5.38522233
NONHSAG039511	3.76123449	3.51220446	3.76538738
NONHSAG039515	2.97280963	3.32766416	3.14785699
NONHSAG039551	4.7649135	4.75463003	5.06774181
NONHSAG039586	2.34829537	2.1283099	2.29931727
NONHSAG039670	4.96037574	4.68988226	4.97179537
NONHSAG039676	8.8720352	8.87170407	8.85958948
NONHSAG039681	2.82593998	2.87931473	2.78979906
NONHSAG039701	4.73193903	4.73863457	5.02767807
NONHSAG039715	2.65571639	2.79897713	2.8036488
NONHSAG039740	2.28184989	2.30402128	2.69027083
NONHSAG039770	2.84412826	2.55795603	2.71466166
NONHSAG039813	2.80359162	2.70795935	2.71932291
NONHSAG039828	3.06006229	2.87707643	2.73483889
NONHSAG039846	2.76086182	2.66534647	2.69699728
NONHSAG039857	3.80835846	3.83864931	3.53870241
NONHSAG039863	6.58747053	6.65668363	6.13571428
NONHSAG039875	6.32304088	6.56636844	6.42159795
NONHSAG039889	3.33518756	3.58506944	3.76104655
NONHSAG039949	2.78891813	2.95501317	2.51009204
NONHSAG039968	2.37028708	2.14864591	2.17742553
NONHSAG039971	3.42344334	3.14529559	3.06075531
NONHSAG039976	2.62891438	2.40397112	2.537219
NONHSAG039991	3.98908805	4.00341022	4.53875468
NONHSAG040004	2.49052465	2.42746153	2.48301067
NONHSAG040070	2.76306835	2.87885016	3.11561835
NONHSAG040081	2.78246741	2.82386232	2.49957752
NONHSAG040093	2.32598809	2.26627103	2.25273983
NONHSAG040100	2.56947829	2.58882175	2.63372038
NONHSAG040122	5.55289617	5.54063347	5.5221017
NONHSAG040157	4.74105633	4.52133152	4.5763955
NONHSAG040159	4.73896449	4.81931784	4.42202334
NONHSAG040185	7.64388442	7.63284087	7.61791804
NONHSAG040189	2.94094411	3.04176106	3.1399638
NONHSAG040206	5.09751707	5.09519995	4.86328266
NONHSAG040218	2.3490648	2.4378155	2.3459184
NONHSAG040274	2.5900547	2.35152196	2.19729357
NONHSAG040276	2.64156153	2.56903798	2.42085087
NONHSAG040302	5.43544176	5.63004708	5.04989581
NONHSAG040333	7.70167618	7.68107396	7.38050719
NONHSAG040375	2.92268223	3.06577179	3.23979966
NONHSAG040378	3.87125673	3.88969917	4.20861815
NONHSAG040385	3.93423447	3.5187205	3.88923272
NONHSAG040395	2.73623156	2.86646758	3.00846417
NONHSAG040459	2.4924159	2.6615613	2.79266097
NONHSAG040465	3.07171665	3.00404643	2.93111586
NONHSAG040492	3.1076794	2.6984959	3.27039118
NONHSAG040535	5.09961205	4.78011698	4.9610064
NONHSAG040538	3.42685803	3.1152811	3.09729532
NONHSAG040545	3.68118669	3.67415614	3.62810477
NONHSAG040546	3.88217616	3.95471503	4.16704038
NONHSAG040559	4.45667544	4.3469513	4.39043858

NONHSAG040605	5.95110245	5.9950337	6.10014752
NONHSAG040664	8.7778058	8.6100133	8.50140127
NONHSAG040668	4.62741842	4.56528098	4.71145404
NONHSAG040671	2.47568317	2.63167004	2.48888651
NONHSAG040680	3.34825956	3.55739761	3.60654108
NONHSAG040684	3.4408095	3.40398336	3.14521345
NONHSAG040703	5.10928212	4.81156841	4.84502947
NONHSAG040714	3.75321003	3.79866663	4.11382562
NONHSAG040766	3.43803951	3.58696927	3.59595233
NONHSAG040771	3.10787778	2.96352442	3.06947345
NONHSAG040781	6.6000968	6.53921483	6.4729208
NONHSAG040790	3.89859452	3.94012911	4.0274169
NONHSAG040792	3.66092909	3.8252624	3.8238572
NONHSAG040794	4.79362639	4.98132279	4.67265921
NONHSAG040797	3.62107362	3.51581552	3.61842634
NONHSAG040826	5.80594914	6.25188305	6.27239373
NONHSAG040838	2.96786404	3.43529554	3.31974075
NONHSAG040876	4.70065219	4.68905589	4.67011173
NONHSAG040878	4.65054788	4.74885512	4.65208897
NONHSAG040919	2.12255878	1.97512722	2.17855426
NONHSAG040944	2.83568266	2.69919191	2.92342951
NONHSAG040959	2.80640935	2.93930307	3.01885398
NONHSAG040973	4.16073788	4.31842619	4.4696693
NONHSAG040975	3.18322328	3.07214583	2.87215822
NONHSAG040987	4.10998432	3.99272834	3.91575911
NONHSAG040990	2.57578881	2.35871592	2.25943117
NONHSAG040992	3.10612286	2.96478112	2.86056291
NONHSAG041024	2.26237029	2.41549838	2.41168409
NONHSAG041029	2.4157652	2.32789812	2.45603391
NONHSAG041052	5.29623447	5.55363924	5.22941886
NONHSAG041087	3.29192842	3.33834994	3.45770322
NONHSAG041094	9.27891522	9.48313947	9.24476025
NONHSAG041153	2.39158861	2.48577253	2.81348488
NONHSAG041157	2.56136764	3.68768319	3.13359073
NONHSAG041202	2.62287684	2.82094497	2.91737466
NONHSAG041205	4.18313243	4.22325596	4.28012847
NONHSAG041207	4.39770082	4.13675164	4.36732367
NONHSAG041271	3.0371482	3.09783387	3.42283038
NONHSAG041280	6.62077167	6.50311285	6.55561959
NONHSAG041308	2.42055202	2.34568932	2.54428596
NONHSAG041310	4.54624504	4.53935145	4.44668607
NONHSAG041311	2.56188444	2.45147602	2.4461441
NONHSAG041331	8.29907539	8.23260352	7.94820375
NONHSAG041341	3.20360894	2.91725654	2.76302014
NONHSAG041345	2.68177046	2.67491761	2.64198786
NONHSAG041359	2.45126811	2.70236576	2.94433546
NONHSAG041393	2.21450948	2.14864591	2.24042719
NONHSAG041442	2.79460866	2.92882231	2.94913878
NONHSAG041473	6.6764538	6.45738989	5.82961654
NONHSAG041474	5.54156607	5.46790574	5.09867256
NONHSAG041475	3.61816437	3.75796543	3.68351656
NONHSAG041496	2.83379561	2.84584469	3.02346354
NONHSAG041512	4.82379442	4.59073131	4.93348898
NONHSAG041520	3.0794416	2.97060434	3.52123916
NONHSAG041523	5.36010632	5.42267587	5.44508494
NONHSAG041551	5.26390187	5.20668256	5.52044579
NONHSAG041552	3.84331934	3.85115494	4.21838865
NONHSAG041579	5.39640769	5.46187034	5.69458824
NONHSAG041596	10.9381425	10.85381971	10.94526314
NONHSAG041601	3.90467093	3.91240526	4.26059713
NONHSAG041618	4.08186026	4.43239554	4.25477985
NONHSAG041677	4.21474162	4.56926427	4.83769152
NONHSAG041723	4.19792799	4.12414951	4.17071282
NONHSAG041789	3.92546183	3.80097231	4.07783292
NONHSAG041804	2.36454963	2.3220657	2.55988329
NONHSAG041810	3.01752479	3.05917618	3.2924411
NONHSAG041826	2.26415447	2.04694299	2.17612167
NONHSAG041886	2.94665855	2.92921027	3.0659793
NONHSAG041903	3.79223604	3.82843162	3.9890561
NONHSAG041984	5.01531186	5.07032088	5.30394997
NONHSAG041987	3.06266418	3.02480006	3.19743915

NONHSAG041994	2.51668633	2.57271069	2.43248132
NONHSAG041998	2.64695113	2.53431759	2.69636641
NONHSAG041999	3.24580098	3.25167536	3.10617201
NONHSAG042046	2.53044387	2.59985313	2.62746484
NONHSAG042051	5.10376179	4.81929516	5.52863432
NONHSAG042059	3.18479023	3.05754571	3.24188601
NONHSAG042103	3.26435137	3.27126215	3.32157246
NONHSAG042145	3.19161216	3.16691685	3.21129736
NONHSAG042163	3.49895715	3.45524051	3.57142562
NONHSAG042168	2.70899718	2.55505891	2.36828763
NONHSAG042176	10.69962555	10.7614656	10.65350241
NONHSAG042182	4.10887527	4.04577647	4.13175591
NONHSAG042192	3.21228479	2.9096843	3.11313448
NONHSAG042210	2.7295206	3.05796309	3.15076071
NONHSAG042250	3.39601169	3.37292851	3.48966024
NONHSAG042261	3.32441706	3.04686526	2.99423558
NONHSAG042272	4.13532128	4.04215793	4.06352166
NONHSAG042283	4.04899824	3.88327909	4.02432452
NONHSAG042315	4.62218749	4.70666271	4.90138356
NONHSAG042323	4.26873902	4.24920215	4.45708134
NONHSAG042369	4.39492407	4.40128173	4.76250116
NONHSAG042381	3.20463342	2.82609438	2.98134117
NONHSAG042418	5.07931654	4.91770908	5.30804394
NONHSAG042419	5.32141724	5.28528945	5.43289407
NONHSAG042424	4.8299277	4.93778893	4.81660892
NONHSAG042444	4.21086347	4.58142645	4.41248275
NONHSAG042451	3.77822849	4.00664838	3.98018291
NONHSAG042471	3.33712322	3.22449957	2.88359152
NONHSAG042535	2.25962274	2.49652592	2.22304697
NONHSAG042546	3.09990751	3.14927616	3.2187532
NONHSAG042558	3.82395697	4.07769933	4.08530718
NONHSAG042615	2.46496187	2.64868138	2.43067398
NONHSAG042636	4.46480072	4.54725699	4.52156247
NONHSAG042638	4.48563484	4.56331415	4.61434302
NONHSAG042726	4.17832015	4.46485241	4.53199218
NONHSAG042795	3.24600897	3.08780974	3.44955502
NONHSAG042834	3.37035055	3.54814018	3.53315424
NONHSAG042877	3.24117862	3.6302526	3.2917286
NONHSAG042907	6.69170846	6.6321985	6.70650854
NONHSAG042913	2.35768186	2.42714007	2.46247436
NONHSAG042922	2.8896732	2.79863826	3.19860155
NONHSAG042934	3.59629329	3.10014169	3.27082315
NONHSAG042947	5.45727874	5.76260889	5.89002394
NONHSAG042959	3.50402881	3.46245589	3.66175172
NONHSAG042979	3.16120928	3.14870909	3.34101224
NONHSAG042982	2.66708219	2.62061878	2.80500889
LOC284379	5.95995287	5.84948744	6.05325531
EP300-AS1	3.3642677	3.52195328	3.5252719
DEPDC1-AS1	2.77075416	2.58276654	2.45486165
RUNDC3A-AS1	6.41164671	6.70560117	7.04076797
PTPRG-AS1	5.07811585	5.25105857	5.25523422
ELOVL2-AS1	5.12566011	5.82077868	5.62551848
NONHSAG016418	3.69362517	3.70964237	3.53776327
TMPO-AS1	4.36976773	4.39324698	4.54704851
NONHSAG042983	2.11070989	2.12585925	2.25105806
NONHSAG042995	3.60738718	3.34597514	3.92554149
NONHSAG043003	2.70529159	2.8800053	2.92768088
NONHSAG043014	2.87346572	2.84925257	3.25136616
NONHSAG043016	2.77700866	2.64368678	2.67977012
NONHSAG043027	4.3104293	4.41329815	4.50920342
NONHSAG043033	3.15704529	3.42359668	3.20182058
NONHSAG043043	6.67906858	6.76556251	6.62043077
NONHSAG043047	5.03347298	4.88800889	5.05748695
NONHSAG043071	5.1382567	5.13574087	5.35050024
NONHSAG043074	4.4435737	4.1424199	4.3271867
NONHSAG043077	4.01844332	4.00446362	4.32192517
NONHSAG043093	3.19534962	3.32988471	3.22650147
NONHSAG043129	3.50257299	3.52705157	3.52970377
NONHSAG043135	6.28012984	6.08807362	6.28438035
NONHSAG043144	3.55787486	3.57481718	3.61760041
NONHSAG043195	3.04444219	3.13125083	3.13951625

NONHSAG043203	5.75166172	5.66529157	5.60995901
NONHSAG043218	2.124507	2.25212939	2.14535297
NONHSAG043252	2.46792532	2.43824654	2.43759473
NONHSAG043277	4.07011076	4.4323743	4.40216027
NONHSAG043281	2.53291565	2.40865444	2.57173846
NONHSAG043292	2.92521784	3.11221819	2.74562304
NONHSAG043304	3.04881421	2.89649351	3.0595604
NONHSAG043313	3.58109326	3.46145252	3.39293526
NONHSAG043379	6.06329036	6.17468463	6.66336684
NONHSAG043614	3.81622656	3.79026349	3.71458708
NONHSAG043616	4.6748301	4.68581567	4.99285139
NONHSAG043628	9.09042173	8.98897012	8.98259655
NONHSAG043629	8.04809158	7.95053732	7.96561111
NONHSAG043634	2.31044711	2.48346496	2.26874828
NONHSAG043637	7.04809138	6.89233441	6.66053569
NONHSAG043646	5.64958026	5.82405921	5.66446755
NONHSAG043677	2.48153525	2.8924256	2.7957305
NONHSAG043699	2.70899718	2.81695384	2.77369646
NONHSAG043706	4.22166244	4.95361436	4.97030249
NONHSAG043726	2.68475405	2.47651073	2.67007493
NONHSAG043748	4.7151091	5.05226044	5.08826303
NONHSAG043765	6.87501167	6.84552067	7.07355241
NONHSAG043790	5.84407622	5.85213816	5.80030264
NONHSAG043821	5.48152919	5.60586804	5.86873249
NONHSAG043825	3.05378421	2.76717676	2.88160283
NONHSAG043835	2.95749691	3.04201536	3.30027496
NONHSAG043845	4.75584041	4.68453071	4.80579367
NONHSAG043849	4.30707392	4.03035844	4.37312139
NONHSAG043854	3.78973679	3.9566916	4.09860459
NONHSAG043856	4.82345825	4.87547668	5.05384362
NONHSAG043865	3.5628981	3.33312418	3.49267795
NONHSAG043905	8.28727033	8.09710982	8.17582615
NONHSAG043910	3.90864891	3.43843561	3.75103515
NONHSAG043913	2.91653046	3.02425677	2.84969075
NONHSAG043916	3.63107468	3.6927365	3.55788661
NONHSAG043927	3.35827731	3.59978791	3.58614656
NONHSAG043945	2.78580738	2.9519713	2.97560865
NONHSAG043980	3.85417871	4.11032974	4.16850484
NONHSAG043986	3.13410429	3.22640445	3.44273386
NONHSAG044024	2.29898806	2.41400147	2.2212069
NONHSAG044064	2.68704367	3.27169163	3.1946698
NONHSAG044066	2.78184577	2.59012826	2.83758561
NONHSAG044070	3.44149053	3.16809028	3.6246316
NONHSAG044072	2.29918785	2.51666384	2.33785355
NONHSAG044100	7.66858082	7.59632214	7.64722227
NONHSAG044111	3.00810897	2.37486666	2.72353067
NONHSAG044194	3.63482746	3.55256275	3.4923466
NONHSAG044196	4.81455565	4.78790037	4.74592505
NONHSAG044199	3.12910351	3.14616223	3.31497504
NONHSAG044203	4.5686928	4.52112704	4.42078281
NONHSAG044217	4.76743652	4.94409069	4.94035507
NONHSAG044226	3.07197497	2.89736323	3.26719002
NONHSAG044243	3.52409048	3.743166	3.74125638
NONHSAG044245	3.62340237	3.5394954	3.5912053
NONHSAG044258	3.70557067	3.35542767	3.06947345
NONHSAG044264	4.30030767	4.04486647	4.34399582
NONHSAG044265	2.57459793	2.01553036	2.14502125
NONHSAG044266	2.40850934	2.36552763	2.69819196
NONHSAG044270	3.10446649	2.80566715	3.24852682
NONHSAG044283	7.20650409	7.4216098	7.13291505
NONHSAG044290	2.2917993	2.47623558	2.46638754
NONHSAG044304	3.79813349	3.98662866	3.80850946
NONHSAG044336	3.63787516	4.01440893	3.98994243
NONHSAG044342	3.50045449	3.3459502	3.40711903
NONHSAG044349	5.5832316	5.30963864	5.59468663
NONHSAG044369	2.3696351	2.32714217	2.44306959
NONHSAG044447	2.32295611	2.54356389	2.25130867
NONHSAG044503	2.34330274	2.51719962	2.44546188
NONHSAG044507	2.44603628	1.89786814	1.95687976
NONHSAG044512	2.88042598	3.08178732	3.14461444
NONHSAG044540	3.03108136	2.85661368	2.67660968

NONHSAG044561	2.98464261	2.80282073	2.86883122
NONHSAG044585	2.38793668	2.41885651	2.56458791
NONHSAG044626	2.92112243	2.69005535	2.79608136
NONHSAG044627	2.71719187	2.77560498	2.42205412
NONHSAG044671	2.94007699	2.80260267	3.17007086
NONHSAG044686	2.5169779	2.36195156	2.57056354
NONHSAG044742	6.5530829	6.26967816	6.5488159
NONHSAG044767	3.38242267	3.42729407	3.52501516
NONHSAG044790	3.37272669	3.13648476	3.43034732
NONHSAG044806	4.95956222	5.50570323	5.10015659
NONHSAG044810	6.41690467	6.52262995	6.59255461
NONHSAG044811	2.77843757	2.63186323	2.70083281
NONHSAG044815	2.08840096	2.01954704	2.01010717
NONHSAG044846	4.60886729	4.49341189	4.57245125
NONHSAG044851	3.13216698	2.98386177	2.8952111
NONHSAG044892	4.0977541	3.85524838	4.21150028
NONHSAG044894	4.36754405	4.57285238	4.86711858
NONHSAG044918	2.58499475	2.26396749	2.48082682
NONHSAG044920	2.74871143	3.04509176	3.06733956
NONHSAG044929	5.91196996	6.04680307	5.95044949
NONHSAG044999	2.9441362	2.93821602	3.20751041
NONHSAG045020	2.39873361	2.4931202	2.51344875
NONHSAG045068	2.33994232	2.47224599	2.693263
NONHSAG045080	3.17294379	3.28118297	3.33795938
NONHSAG045081	3.86950106	4.49486367	4.06721937
NONHSAG045085	4.70063474	4.90622517	5.02545133
NONHSAG045101	4.07587147	4.11109905	4.04960863
NONHSAG045116	2.81972092	2.53685946	2.47613275
NONHSAG045138	3.352496	3.44569279	3.18276495
NONHSAG045167	3.57342153	3.48301582	3.81318422
NONHSAG045192	3.44003663	3.20319749	3.36902776
NONHSAG045208	3.70147406	3.98937757	3.41216466
NONHSAG045235	6.05821071	6.50515996	6.68984916
NONHSAG045258	7.5679228	7.81036833	7.87297741
NONHSAG045265	4.41119129	4.48799051	4.69812967
NONHSAG045281	3.11501964	2.88767986	2.80632754
NONHSAG045316	2.25757528	2.28678201	2.11327544
NONHSAG045329	2.11712851	2.28973058	2.35023385
NONHSAG045333	4.74374039	4.63606732	4.25141424
NONHSAG045338	2.76180533	2.67593572	2.65432525
NONHSAG045350	2.57793326	2.74173751	2.58629383
NONHSAG045363	5.17970156	5.30998976	5.31214778
NONHSAG045364	4.19954079	4.23634194	4.14692685
NONHSAG045389	2.69177308	2.56892036	2.65200668
NONHSAG045403	5.73134289	5.67477122	5.65830744
NONHSAG045405	3.73663368	3.68986098	3.40569716
NONHSAG045408	4.02092729	4.07040762	4.11893626
NONHSAG045419	4.17736969	4.1668902	4.10412143
NONHSAG045425	3.37472593	3.26915517	3.61094696
NONHSAG045429	3.54068197	3.51467649	3.49359466
NONHSAG045445	3.71721937	3.55874748	3.74912148
NONHSAG045449	2.44997557	2.27652226	2.54774682
NONHSAG045463	2.9690353	3.12926721	3.2929536
NONHSAG045467	4.69705131	4.60184758	5.01157716
NONHSAG045472	3.49199092	3.59471342	3.43332299
NONHSAG045505	3.61097073	3.75114245	3.90846986
NONHSAG045512	3.80334798	3.58249597	3.77352602
NONHSAG045517	3.14632446	3.10481658	2.94625006
NONHSAG046716	5.58251794	5.40503041	5.65657799
NONHSAG046750	5.64282958	5.59806533	5.59174526
NONHSAG046781	5.23105787	5.33579983	5.34776505
NONHSAG046784	5.06414122	5.1482625	5.25973687
NONHSAG046797	4.42782946	4.47185532	4.47811775
NONHSAG046806	6.46273405	6.46509986	6.4294063
NONHSAG046817	4.00836402	3.90800081	4.08861988
NONHSAG046838	4.03749849	4.01934358	4.11093298
NONHSAG046851	4.2300297	4.32895788	4.39445744
NONHSAG046903	4.49560975	4.44082782	4.47214271
NONHSAG046927	2.89097077	2.67021392	3.18042153
NONHSAG046961	2.96171708	3.04737586	3.10208974
NONHSAG047021	2.46967378	2.70896318	2.54407534

NONHSAG047026	4.35555795	4.49848735	4.90531054
NONHSAG047060	2.84818505	2.71038661	2.71842772
NONHSAG047082	3.56592043	3.38087053	3.55744255
NONHSAG047088	2.40728955	2.41901117	2.24258386
NONHSAG047097	4.58867972	4.67727673	4.38267356
NONHSAG047101	2.62301105	2.77771204	2.48722243
NONHSAG047126	2.53806408	2.42828016	2.53286349
NONHSAG047149	3.90795021	3.87050041	3.93375503
NONHSAG047152	2.95186564	2.77240944	3.12630116
NONHSAG047169	2.38354484	2.77550088	2.51790998
NONHSAG047175	3.53596191	3.14938571	3.45078807
NONHSAG047181	3.24118513	3.26886545	3.2157467
NONHSAG047192	4.68821804	4.76971929	4.62315514
NONHSAG047193	3.14716478	3.05762069	3.29312323
NONHSAG047194	3.31480699	3.38885272	3.30109313
NONHSAG047208	2.453385	2.380415	2.69616386
NONHSAG047221	3.39262124	3.55894824	3.43563722
NONHSAG047229	5.10261577	4.49112424	4.67711835
NONHSAG047232	4.0635136	4.03735772	3.79308283
NONHSAG047253	3.18249209	3.2560621	3.08625233
NONHSAG047257	3.19687246	3.52333918	3.4693196
NONHSAG047259	3.27991285	3.41051388	3.51762376
NONHSAG047273	5.89889459	5.74565719	5.86518329
NONHSAG047282	2.80831112	2.69910201	2.97849589
NONHSAG047295	2.80316702	2.82375884	2.91694104
NONHSAG047314	4.33186393	4.34594374	4.50304705
NONHSAG047336	5.37447045	5.27032104	5.36988417
NONHSAG047348	2.75444577	2.74843925	2.65545086
NONHSAG047356	2.70899718	2.71559611	2.84336781
NONHSAG047375	3.0651536	3.15596859	3.22876267
NONHSAG047399	2.41995217	2.61557265	2.4743216
NONHSAG047416	2.63306735	2.60914889	2.81412369
NONHSAG047425	2.21624039	2.26396749	2.41474011
NONHSAG047431	2.8783787	2.93479466	2.83576946
NONHSAG047443	3.15472527	2.93641411	2.74847477
NONHSAG047447	2.13697632	2.2690277	2.27739878
NONHSAG047469	3.81402396	3.77974273	3.95001519
NONHSAG047503	2.37089061	2.46781094	2.29448348
NONHSAG047517	6.41502506	6.29659295	6.36893235
NONHSAG047524	2.99393392	2.8674185	3.3363096
NONHSAG047536	2.30189895	2.14772383	2.2114064
NONHSAG047547	6.70624497	6.67694098	6.67211352
NONHSAG047553	4.49119151	4.20612099	4.52660287
NONHSAG047570	4.67656948	4.82967199	4.79962855
NONHSAG047611	3.96734181	3.89303964	4.19605958
NONHSAG047665	4.22902719	4.24204698	4.17764547
NONHSAG047674	2.69491833	2.84593493	2.54302896
NONHSAG047730	2.85355201	2.75531951	2.90183974
NONHSAG047747	3.78471091	3.6084578	3.8878548
NONHSAG047777	4.17772098	4.23040933	3.92041412
NONHSAG047807	2.82789166	2.83421095	2.98443816
NONHSAG047837	2.31953092	2.44909527	2.36537485
NONHSAG047842	6.97839544	6.76170967	6.59558458
NONHSAG047845	4.36969291	3.94345264	4.36445153
NONHSAG047847	5.20145081	5.30689494	5.50177387
NONHSAG047907	9.02837983	8.93399339	9.07036968
NONHSAG048011	4.30526316	4.27054219	4.56429081
NONHSAG048092	2.4177627	2.26396749	2.38704848
NONHSAG048098	2.22358623	2.43583518	2.47437505
NONHSAG048120	3.25654999	3.27257835	3.48171471
NONHSAG048123	2.71881683	2.60647671	2.9370804
NONHSAG048148	2.20458839	2.21978317	2.25011141
NONHSAG048155	3.83551498	3.80558725	4.32134983
NONHSAG048166	2.68198629	2.81498929	2.92653651
NONHSAG048202	3.06927386	2.94578177	3.27005596
NONHSAG048386	6.02754886	6.18294056	6.23190963
NONHSAG048397	8.6596034	8.93147855	8.58278262
NONHSAG048404	4.84280949	4.69526364	4.76147506
NONHSAG048405	2.31902482	2.14864591	2.24516342
NONHSAG048439	2.91525026	2.84327394	2.99825106
NONHSAG048458	2.36531582	2.49367124	2.69236147

NONHSAG048471	6.117183	6.16793022	6.38295741
NONHSAG048506	3.02583572	2.7178283	2.72168092
NONHSAG048520	4.22183563	4.59247743	4.27456194
NONHSAG048562	2.47642004	2.44266345	2.39201641
NONHSAG048563	5.81555331	5.87765988	5.77727881
NONHSAG048569	4.4114314	4.18751677	4.33947636
NONHSAG048593	2.67764604	2.50827863	2.58462493
NONHSAG048597	2.20058084	2.29052858	2.35994745
NONHSAG048604	3.89043196	3.86590644	3.84486627
NONHSAG048605	4.64028955	4.55059063	5.10898018
NONHSAG048651	3.41175517	3.51091438	3.27127007
NONHSAG048682	5.316775	5.3141366	5.30465999
NONHSAG048687	3.06674403	2.86066628	2.84386172
NONHSAG048746	5.86416023	5.92258866	5.99234087
NONHSAG048747	8.12691329	8.15626364	8.23870311
NONHSAG048781	5.39556485	5.25978191	5.16147535
NONHSAG048790	2.33246697	2.34723797	2.39164464
NONHSAG048794	5.0516137	5.00970981	5.38969129
NONHSAG048813	3.52952196	3.46600572	3.63819102
NONHSAG048817	3.9685536	4.15865241	4.30118488
NONHSAG048836	4.74458676	4.82821213	4.59176445
NONHSAG048840	4.45723258	4.56990567	4.48350869
NONHSAG048846	3.4474477	3.60704392	3.48384126
NONHSAG048902	2.75859726	2.62555774	3.0373263
NONHSAG048962	6.40083886	6.29407206	6.29742776
NONHSAG048976	5.31814731	5.14147879	5.39089324
NONHSAG048984	2.45061029	2.69794248	2.62349361
NONHSAG048993	5.75769268	5.83121486	5.87652883
NONHSAG049003	4.27055735	4.18012954	4.51340467
NONHSAG049010	3.86818163	4.01693912	3.89294312
NONHSAG049037	5.97892514	6.13977527	5.89752224
NONHSAG049079	7.18097259	7.06470711	7.1912868
NONHSAG049095	6.77490979	6.73023656	6.92985036
NONHSAG049099	2.88490794	2.83839398	2.65450233
NONHSAG049119	3.91879478	3.41656554	3.78011887
NONHSAG049134	3.68610145	3.82264064	3.89808642
NONHSAG049182	4.14725309	4.28976671	4.32444025
NONHSAG049240	4.50856847	4.39743016	4.4797069
NONHSAG049298	3.22220593	3.0843934	3.33922372
NONHSAG049309	6.08001407	6.47786987	6.20218517
NONHSAG049336	5.1171897	5.26560979	5.08095662
NONHSAG049339	2.70899718	2.71038661	2.62173594
NONHSAG049341	6.10137399	6.04761606	5.77606849
NONHSAG049344	4.26927583	4.33942235	4.27506439
NONHSAG049350	4.33095058	4.47255985	4.59340196
NONHSAG049357	2.96795656	3.1101115	2.92681905
NONHSAG049388	2.94575065	2.67540604	2.96328422
NONHSAG049409	2.98203902	2.96653666	2.83590664
NONHSAG049422	3.39344215	3.53576757	3.77331025
NONHSAG049424	5.25769331	5.59508368	5.46540766
NONHSAG049464	4.08708022	4.3229371	4.01274051
NONHSAG049487	4.6224907	4.66330889	4.41959351
NONHSAG049492	2.85094736	2.60821662	2.98808898
NONHSAG049507	3.59585414	3.72066172	3.70227634
NONHSAG049508	3.35789451	3.53266142	3.43881948
NONHSAG049511	3.10831405	3.17323467	3.3312777
NONHSAG049515	2.20437777	2.50935289	2.41926422
NONHSAG049523	2.33706219	2.17986691	2.07354183
NONHSAG049524	3.42313129	3.54902186	3.75655589
NONHSAG049526	3.99548554	4.20035953	3.87429189
NONHSAG049528	4.48955643	4.6562124	4.5796206
NONHSAG049529	4.05139061	3.91131379	3.94863147
NONHSAG049530	2.70148737	3.05052936	2.94232726
NONHSAG049531	4.90782521	4.89492563	5.15942098
NONHSAG049532	3.06756573	3.32286735	3.2722697
NONHSAG049534	2.34258692	2.21928501	2.67270522
NONHSAG049539	4.85993996	4.84931623	4.93167904
NONHSAG049594	2.87830454	3.25936908	3.27122804
NONHSAG049595	3.08750548	3.31198371	3.45359066
NONHSAG049661	2.36349523	2.3671541	2.15967857
NONHSAG049665	2.87921624	2.97027963	2.64525054

NONHSAG049689	2.7915451	2.83576946	2.88941629
NONHSAG049734	3.66680337	3.54195754	4.06118117
NONHSAG049792	3.89494232	3.86170978	3.96685284
NONHSAG049868	5.50506495	5.5218367	5.53119262
NONHSAG049879	3.13196648	2.99107502	2.86469744
NONHSAG049885	3.69778671	3.60116413	3.34821218
NONHSAG049895	4.12902296	4.11182033	4.14131481
NONHSAG049902	2.49507682	2.5872445	2.80711013
NONHSAG049904	6.64670082	6.72795979	6.68822517
NONHSAG049930	3.13501137	3.40282181	3.20737685
NONHSAG049931	3.11375431	3.04018045	3.25804902
NONHSAG049936	2.56859041	2.5755667	2.42383581
NONHSAG049939	5.11992741	4.62139553	4.60564218
NONHSAG049977	4.67329444	4.99668064	5.1616876
NONHSAG049980	4.55484483	4.40470684	4.37879057
NONHSAG049984	2.83152224	2.60823074	2.60157702
NONHSAG049989	4.08643967	4.41196952	4.18153501
NONHSAG050013	5.59871082	5.67181011	5.5308888
NONHSAG050025	5.30884155	5.36580169	5.27224398
NONHSAG050057	3.44917733	3.80793057	3.89287023
NONHSAG050083	5.07621638	4.952647	5.10100171
NONHSAG050088	4.08600657	4.15673122	4.2052478
NONHSAG050093	5.11019975	5.19238579	5.54643977
NONHSAG050175	4.17252984	4.27763963	4.48765006
NONHSAG050188	2.96562201	2.99792959	3.08878739
NONHSAG050210	4.90742347	4.99229253	4.93879407
NONHSAG050227	3.06072178	3.14387262	2.98808898
NONHSAG050230	3.15073229	3.43495814	3.02775279
NONHSAG050243	3.24175909	3.71825609	3.65957544
NONHSAG050259	2.35236199	2.54706392	2.23776377
NONHSAG050280	4.65933424	4.86863369	4.85546053
NONHSAG050284	3.91083018	3.96720407	4.03051769
NONHSAG050285	4.10884036	4.10740947	4.66116826
NONHSAG050296	4.83311139	5.03813887	5.09550735
NONHSAG050300	3.42186006	3.40070569	3.6851847
NONHSAG050305	5.33518355	5.62667762	6.10349371
NONHSAG050335	4.10884139	4.26488237	4.66039304
NONHSAG050348	2.50863924	2.52404775	2.36153753
NONHSAG050370	2.96510633	3.01469445	3.48884735
NONHSAG050372	4.06243376	4.15208975	4.30091166
NONHSAG050397	4.4133564	4.27787257	4.17442036
NONHSAG050419	6.13808525	6.05646147	6.12033499
NONHSAG050438	2.16341674	2.14225108	2.10321102
NONHSAG050440	2.52585527	2.53001623	2.37234733
NONHSAG050454	3.42186006	3.24527551	3.36328226
NONHSAG050461	2.58710956	2.43535018	2.19507601
NONHSAG050473	6.53237934	6.57592516	6.69218404
NONHSAG050503	4.02677749	4.08065873	4.4080267
NONHSAG050531	2.7973306	2.86113763	2.9637953
NONHSAG050576	3.20856262	3.14112895	3.48269124
RP11-48B3.4	6.75371811	6.91729303	6.54629783
NONHSAG050607	3.15380613	3.27657474	3.45800145
NONHSAG050643	1.96145634	2.12797344	2.42749272
NONHSAG050690	10.0230404	9.93955549	10.13968086
NONHSAG050699	5.83130321	5.99944793	5.75845942
NONHSAG050714	2.74660694	2.76925943	2.77597347
NONHSAG050718	4.92203618	4.52562875	4.79703449
NONHSAG050752	2.29916015	2.22880563	2.41487645
NONHSAG050804	6.44348114	6.36262005	6.25224049
NONHSAG050806	5.5403881	5.65048306	6.01362978
NONHSAG050815	2.46412538	2.50280239	2.51545041
NONHSAG050829	2.54982395	2.86278897	2.36489052
NONHSAG050841	3.6714567	3.69079921	3.82386787
NONHSAG050865	7.74293241	7.72167573	7.44272548
NONHSAG050866	5.8133671	5.83447944	5.46098341
NONHSAG050902	3.84226146	3.80240612	3.72483029
NONHSAG050903	7.49388426	7.45747466	7.1361931
NONHSAG050913	5.40889896	5.34910434	5.63269132
NONHSAG050914	2.96131944	3.6094581	3.14171124
NONHSAG050915	4.53272635	4.324533	4.46415214
NONHSAG050921	2.95178867	3.04379057	3.04115436

NONHSAG050966	5.36938008	4.86339874	5.1409209
NONHSAG050969	2.82929052	3.24950378	2.89808851
NONHSAG050970	3.17915134	3.20973999	3.43881642
NONHSAG050978	3.72586408	3.85554275	3.71009086
NONHSAG050982	6.51598527	6.3285346	6.28910598
NONHSAG051053	4.08855818	3.90594917	4.15116593
NONHSAG051068	2.08289994	2.27970686	2.1701733
NONHSAG051082	5.73540805	6.18310097	6.22117538
NONHSAG051104	4.12078225	4.66816469	4.60415279
NONHSAG051105	3.51027252	3.97446364	3.87357888
NONHSAG051129	3.14863792	3.18732871	3.04183008
NONHSAG051137	3.95481823	3.90968586	4.09709254
NONHSAG051145	3.96160499	4.0586225	4.11263711
NONHSAG051156	2.57644174	2.19299638	2.56179868
NONHSAG051181	3.19679446	3.34106836	3.461403
NONHSAG051194	2.80327319	3.32940032	3.06196792
NONHSAG051196	5.01657663	5.10224417	4.59844158
NONHSAG051205	3.84574122	4.23330063	4.41606914
NONHSAG051207	3.0501807	2.77773951	2.78331614
NONHSAG051233	2.07275468	2.38231099	2.4928212
NONHSAG051237	2.5600622	2.18079296	2.45377747
NONHSAG051255	3.2929281	3.43217214	3.34307259
NONHSAG051258	4.92374914	4.9438167	5.00854481
NONHSAG051259	3.38695965	3.63472922	3.50587204
NONHSAG051263	3.08762772	3.1236829	3.309709
NONHSAG051266	4.17370526	4.17514324	4.17668999
NONHSAG051273	2.82168634	2.39361417	2.73226453
NONHSAG051275	2.5400732	2.42726103	2.56164035
NONHSAG051297	2.53192707	2.64098598	2.62344449
NONHSAG051305	2.8783787	3.13035985	3.12224667
NONHSAG051327	5.17018891	5.30757656	5.33846185
NONHSAG051334	2.85992236	2.90860468	2.82377607
NONHSAG051339	4.94576795	5.22981082	5.27443281
NONHSAG051345	3.17530559	3.00891843	3.25905937
NONHSAG051356	4.12599422	4.3992617	4.65264845
NONHSAG051375	2.96121623	3.04018254	3.13951625
NONHSAG051392	3.32973771	3.18131893	3.39887467
NONHSAG051400	6.74543704	6.83222047	6.74760469
NONHSAG051424	5.71586025	5.95904917	6.02741081
NONHSAG051435	4.04426562	4.21230365	4.17332758
NONHSAG051445	5.60594229	5.58197332	5.93073301
NONHSAG051446	4.27071909	4.42518386	4.47428894
NONHSAG051464	4.63791806	4.69550507	4.9120642
NONHSAG051484	5.49725797	5.60529556	5.54099959
NONHSAG051497	5.2209929	5.36121223	5.20705614
NONHSAG051574	4.87633815	4.8740909	4.77427922
NONHSAG051638	7.05729063	7.20249035	7.14013838
NONHSAG051650	3.15745816	3.23320454	3.13653215
NONHSAG051658	5.15701604	5.21814627	5.55153825
NONHSAG051661	2.83101426	2.77191092	3.02319405
NONHSAG051677	5.04674344	4.76341863	5.09879461
NONHSAG051728	3.52497546	3.70117806	3.64882774
NONHSAG051730	3.02148538	3.03133431	3.16798422
NONHSAG051770	2.52771127	2.37488804	2.42783652
NONHSAG051806	3.03688443	2.60464724	2.88526575
NONHSAG051813	5.65764523	5.61889664	5.15555718
NONHSAG051826	2.66267818	2.61827798	2.58475724
NONHSAG051866	2.19898217	2.6050921	2.71093625
NONHSAG051885	2.5174436	2.41706787	2.54988408
NONHSAG051943	3.06572737	2.88993645	3.24162088
NONHSAG051945	2.43172433	2.55217942	2.67380803
NONHSAG051962	3.84591041	3.89027845	4.54487499
NONHSAG051968	2.27919247	2.44226709	2.51134198
NONHSAG051985	2.60885574	2.60253495	2.72302595
NONHSAG052010	3.15745816	2.82069791	3.19973739
NONHSAG052012	6.41561593	6.37140666	6.46674692
NONHSAG052042	2.89386614	3.19230417	2.97674401
NONHSAG052090	2.58936529	2.69354132	2.7349387
NONHSAG052112	3.78580197	3.95086028	3.87687243
NONHSAG052118	4.37999836	4.5603116	4.73623826
NONHSAG052125	8.81904091	8.84329542	8.81351251

NONHSAG052126	3.53092606	3.66743194	3.18388549
NONHSAG052157	3.68334523	3.79440347	3.99497257
NONHSAG052162	5.07169585	5.58768383	5.27210326
NONHSAG052178	6.32325877	6.36528031	6.19634712
NONHSAG052186	6.65491406	6.54925393	6.49479498
NONHSAG052188	4.29987761	4.31738494	4.28969957
NONHSAG052409	5.8133671	5.55929917	5.65204687
NONHSAG052414	4.48264445	4.65583305	4.56452989
NONHSAG052449	7.22252748	7.54407577	7.26497
NONHSAG052499	3.52697886	3.49543841	3.68836532
NONHSAG052508	4.96677669	4.99735064	4.99395733
NONHSAG052530	3.40407521	3.46912772	3.44692794
NONHSAG052547	6.02206395	5.93896014	5.78668995
NONHSAG052583	2.4953873	2.45357272	2.49318858
NONHSAG052586	2.28297128	2.02649559	2.14242301
NONHSAG052609	3.09060822	3.00714445	2.86688611
NONHSAG052620	5.22115794	5.27810811	5.43915029
NONHSAG052621	3.54293196	3.43445735	3.68200625
NONHSAG052627	6.21295456	6.10634413	6.00210714
NONHSAG052636	4.10419686	4.57986387	4.5014966
NONHSAG052644	3.44561303	3.27112551	3.83095975
NONHSAG052726	4.71455515	4.66951451	4.93702224
NONHSAG052734	3.71876378	3.53660335	3.84479406
NONHSAG052752	6.06848115	5.87591887	5.65776633
NONHSAG052756	2.71481045	2.88194911	2.88320399
NONHSAG052760	4.99194237	4.9086424	4.655213
NONHSAG052772	6.21854129	6.15542448	6.36579338
NONHSAG052776	5.44387324	5.34743473	5.30645499
NONHSAG052817	4.40229659	4.31130734	4.38146214
NONHSAG052818	3.41247096	2.74138466	3.13951625
NONHSAG052837	6.22003222	6.20697147	5.92350946
NONHSAG052879	4.281364	4.28264287	4.60965402
NONHSAG052880	5.79797099	5.33398251	5.60621294
NONHSAG052885	4.40417739	4.5137581	4.81365658
NONHSAG052886	4.44184253	4.29885907	4.64511943
NONHSAG052890	5.94398056	6.027102	5.95936713
NONHSAG052915	6.15540962	6.0131024	6.47674434
NONHSAG052978	3.28164605	3.19927692	3.51933561
NONHSAG052998	2.67321834	2.63571292	2.44252799
NONHSAG053000	2.72342192	2.70787149	2.70633604
NONHSAG053032	3.69190697	3.69610746	3.61742302
NONHSAG053081	2.4057206	2.40277308	2.42210132
NONHSAG053118	2.27288998	2.13040602	2.08782376
NONHSAG053119	2.74680156	2.55981094	2.81972469
NONHSAG053167	4.10328011	4.1820424	4.0384554
NONHSAG053168	2.46208149	2.42034686	2.36331237
NONHSAG053205	4.84933554	5.08425368	4.51142364
NONHSAG053209	2.16341674	2.27558106	2.34606078
NONHSAG053219	6.68357162	6.72988282	6.7487148
NONHSAG053240	3.42186006	3.3734278	3.27071279
NONHSAG053246	4.50207706	4.51163992	4.5971724
NONHSAG053259	3.68437931	3.6025705	3.66055974
NONHSAG053273	3.78531255	3.64194925	3.95423972
NONHSAG053274	2.97824632	2.71776246	2.94161865
NONHSAG053293	3.86807489	4.22997508	4.23518787
NONHSAG053296	4.10909118	3.82037143	3.86684116
NONHSAG053299	3.24509729	3.08394839	3.69612228
NONHSAG053306	4.8648757	5.0266092	5.46499197
NONHSAG053322	5.0242871	4.84159032	5.46433834
NONHSAG053329	2.77059327	2.60314564	2.7403214
NONHSAG053359	5.54365866	5.55204762	5.57828299
NONHSAG053361	4.19934874	4.24092878	4.40135511
NONHSAG053376	6.26301351	6.29229672	6.32919417
NONHSAG053377	6.51501635	6.47335784	6.42728493
NONHSAG053380	6.03209658	6.08109493	5.84563458
NONHSAG053392	2.68801788	2.64382657	2.79041187
NONHSAG053406	4.71508654	5.16120739	4.93182355
NONHSAG053407	4.51930888	4.52703498	4.47231737
NONHSAG053417	4.05100554	4.31818087	4.52162838
NONHSAG053440	4.24785477	3.93609266	3.98452542
NONHSAG053450	3.10844267	3.12862538	3.3653427

NONHSAG053454	3.30351679	3.01381604	2.76519536
NONHSAG053462	3.47428185	3.67911215	3.56893255
NONHSAG053469	3.60738718	3.5869608	3.34311175
NONHSAG053552	5.84887988	6.35614123	6.32423468
NONHSAG053577	7.50632392	7.40996097	7.33981714
NONHSAG053585	4.65527448	4.76960285	4.85117173
NONHSAG053586	7.52272576	7.44139026	7.55387179
NONHSAG053589	6.26844128	6.27285149	6.48137006
NONHSAG053591	5.26510365	5.42325217	5.61422735
NONHSAG053607	4.00376747	4.1729691	4.1537637
NONHSAG053614	5.41673236	5.39493118	5.6898574
NONHSAG053675	4.97317708	5.15347413	5.32826236
NONHSAG053719	2.44761003	2.42318366	2.82916347
NONHSAG053722	3.63168139	3.4856054	3.80387141
NONHSAG053746	4.55189038	4.10275167	3.80225993
NONHSAG053751	3.73492096	3.76690356	3.88580944
NONHSAG053784	5.63150425	5.68595146	5.66413948
NONHSAG053801	4.02569123	4.14754406	4.0987642
NONHSAG053821	3.04848169	2.99852547	2.77698105
NONHSAG053863	3.82864403	3.73822749	4.02901878
NONHSAG053877	4.84364347	5.01590367	4.88682444
NONHSAG053892	5.163243	4.62323187	4.77963177
NONHSAG053893	13.89315619	13.91107097	13.98330959
NONHSAG053895	14.13680683	14.08749571	14.21493961
NONHSAG053900	2.59478819	2.58258356	2.51517976
NONHSAG053985	2.82278707	2.73662012	2.98808898
NONHSAG054015	2.16289655	2.14562621	2.48244434
NONHSAG054016	2.59382793	2.4505767	2.71957506
NONHSAG054030	2.91162239	2.80780293	2.86375569
NONHSAG054071	2.60192858	2.70077696	2.9123408
NONHSAG054103	3.01290432	3.14394062	3.1283024
NONHSAG054109	2.6612788	2.78985975	2.97317967
NONHSAG054140	2.80647312	2.74081571	2.58355513
NONHSAG054179	4.19873398	4.18197583	4.22526703
NONHSAG054188	3.24580098	3.28901762	3.54719328
NONHSAG054199	5.90890596	5.85364262	6.06861587
NONHSAG054326	2.73413315	2.70865232	2.87531729
NONHSAG054344	2.08696956	2.16240837	2.10629859
NONHSAG054345	3.48465412	3.36155113	3.40819781
NONHSAG054445	3.70557067	3.95974435	3.49369898
NONHSAG054462	4.35449403	4.45442062	4.65348963
NONHSAG054468	4.73990718	4.57071124	4.66163067
NONHSAG054478	2.72121991	2.47121305	2.60736776
NONHSAG054549	5.89294704	6.00499876	5.96189595
NONHSAG054576	5.77438194	5.44603125	5.40636107
NONHSAG054653	5.79926915	5.76466451	6.03364149
NONHSAG054655	4.18415801	4.33946103	4.30965401
NONHSAG054664	3.03483027	3.16672447	3.20538309
NONHSAG054671	3.09575781	3.15193336	3.05510874
NONHSAG054679	2.49805576	2.62625509	2.54826048
NONHSAG054729	6.09645813	5.90597919	6.32478504
NONHSAG054754	4.15166648	4.25239543	4.06162282
NONHSAG054784	6.37043533	6.43919399	6.33379159
NONHSAG054798	5.95255329	6.37063655	6.37856133
NONHSAG054804	7.53874393	8.02478379	8.24829438
NONHSAG054869	3.88217616	3.79108153	3.83934551
NONHSAG054870	6.72686298	6.71487493	6.57857068
NONHSAG054897	2.22729441	2.44813063	2.69027083
NONHSAG054929	3.11125106	3.06909134	3.21229706
NONHSAG054954	3.21294977	2.87006595	3.34785786
NONHSAG054958	3.47107963	3.49795183	3.60088713
NONHSAG054963	4.64813186	4.69490963	4.7904991
NONHSAG055008	2.56526685	2.40926076	2.68343646
NONHSAG055019	2.96567446	2.74132176	2.89167255
NONHSAG055021	3.62546729	3.71239022	3.598162
NONHSAG055050	2.94477103	2.96428243	3.00313324
NONHSAG055086	2.48363852	2.97488636	2.82938773
NONHSAG055105	2.33212953	2.44623476	2.30316146
NONHSAG055108	4.56732297	4.346899	4.59866666
NONHSAG055153	2.93467604	2.92303735	3.0361864
NONHSAG055204	3.60058229	3.32170316	3.65000063

NONHSAG055266	4.94875758	4.92403672	5.09758068
NONHSAG055280	2.40469403	2.5081571	2.37843119
NONHSAG055289	5.84753185	5.50044129	5.62732939
NONHSAG055301	3.42326361	3.45409632	3.64253787
NONHSAG055339	5.24326845	4.87241451	5.02241293
NONHSAG055350	5.85830565	5.6403118	5.88215227
NONHSAG055367	3.26533559	3.32288829	3.51323331
NONHSAG055371	5.91015414	5.85664758	5.83294159
NONHSAG055384	4.35590682	4.83376281	4.78804368
NONHSAG055388	3.5192286	3.84432689	3.60734525
NONHSAG055401	2.28617389	2.47824868	2.70490693
NONHSAG055405	4.03746944	3.96281775	3.83282038
NONHSAG055409	3.49864977	3.8263118	4.14323136
NONHSAG055436	5.66338267	5.31558021	5.4554748
NONHSAG055443	2.60126632	2.63647363	2.65456836
NONHSAG055445	11.52216891	11.25726968	11.31178702
NONHSAG055446	2.55985185	2.38231099	2.59230773
NONHSAG055449	6.28993333	6.17785438	5.95484609
NONHSAG055454	2.56503078	2.50866564	2.45845475
NONHSAG055455	2.82191857	2.95477599	2.84285408
NONHSAG055463	2.97060434	2.97060434	2.80904224
NONHSAG055544	4.86429883	5.16207636	5.22173914
NONHSAG055550	2.67516401	2.48529135	2.51023769
NONHSAG055620	5.63037089	5.73282015	5.37592328
NONHSAG055690	3.37663012	3.35174984	3.53844584
NONHSAG055699	3.67565911	3.52272259	3.78722248
NONHSAG055722	2.85767182	2.97838687	2.95979154
NONHSAG055783	4.92130772	4.54236423	4.73867526
NONHSAG055861	2.66936376	2.41281048	2.58365705
NONHSAG055878	3.81345457	3.70789758	4.04303098
NONHSAG055883	4.70111961	5.35392132	5.23788254
NONHSAG056024	3.70044755	3.51488727	3.88956278
NONHSAG056026	2.38712724	2.27793397	2.3322237
NONHSAG056029	4.2876182	4.3739811	4.53622234
NONHSAG056034	5.25690059	5.31266931	5.31840443
NONHSAG056037	7.01786929	7.3193976	7.45866089

Table S2. Statistical result of 2812 lncRNA genes.

	E2+ICI VS. E2			E2 VS. ethanol			F value
	logFC	P.Value	adj.P.Val	logFC	P.Value	adj.P.Val	
LOC284379	1.513703872	7.71E-15	6.37E-13	1.688213782	1.03E-13	1.12E-11	28052.56063074108708e-15
NONHSAG000057	0.13208392	0.050668843	0.231309391	1.65023614	0.01166504	0.07593077	10.0305026355876767
NONHSAG000058	0.682581082	1.42E-10	5.80E-09	1.631051927	1.53E-10	7.69E-09	22.40814959050883e-11
NONHSAG000070	0.245331166	6.55E-05	0.000818652	1.557754826	1.25E-05	0.000189424	31.46608263829146e-05
NONHSAG000071	0.122208311	0.027279782	0.145010863	1.483133319	0.01851981	0.108721721	40.031765298007893
NONHSAG000075	0.197252896	0.000781265	0.007298728	1.431199231	0.008435938	0.058428223	50.00212851134105749
NONHSAG000111	0.064908248	0.153474348	0.464946969	1.415861319	0.986182474	0.99566177	60.251472458390719
NONHSAG000113	-0.34197815	2.07E-07	4.09E-06	1.398596434	3.68E-07	8.40E-06	78.63977732707919e-08
NONHSAG000117	0.425595969	1.37E-08	3.56E-07	1.337372257	3.17E-06	5.79E-05	83.34211217264522e-08
NONHSAG000134	0.023673593	0.6790535	0.887517619	1.262421208	0.228464582	0.551382969	90.46766100665815
NONHSAG000139	-0.07920763	0.323999216	0.664056702	1.190586388	0.82186518	0.933749876	100.581768437546692
NONHSAG000169	0.085084042	0.381946513	0.706496807	1.179289816	0.491166347	0.789462021	110.650908372592143
NONHSAG000174	0.05373022	0.459102917	0.758072461	1.100973493	0.829017664	0.933749876	120.602698290622236
NONHSAG000181	0.014891317	0.810838995	0.93441384	1.041051302	0.30408113	0.634657974	130.403415009115743
NONHSAG000185	0.040486898	0.621797661	0.857660281	1.039319309	0.847981966	0.942500114	140.882077917990269
NONHSAG000199	0.037535369	0.562136196	0.829618935	1.000373075	0.447572737	0.756417348	150.726843367656622
NONHSAG000202	0.201145118	0.032638156	0.165665152	0.965664705	0.131032172	0.410314552	160.0885344914777903
NONHSAG000215	0.065931511	0.254673746	0.590389592	0.913793467	0.161392615	0.45587637	170.327062676494942
NONHSAG000222	0.071169619	0.358110239	0.684572395	0.898564929	0.361908192	0.69136266	180.56873116774712
NONHSAG000254	0.122400737	0.142454556	0.447555229	0.815773706	0.12033588	0.388055612	190.216651036761643
NONHSAG000264	0.084856881	0.363504924	0.691126333	0.803237104	0.977744079	0.990519194	200.563608433749823
NONHSAG000267	0.062210009	0.295835763	0.632616096	0.794531373	0.365937304	0.69386861	210.522092911293647
NONHSAG000310	0.346601426	4.48E-06	6.97E-05	0.703737791	1.05E-06	2.16E-05	228.22270979369901e-07
NONHSAG000312	-0.05591931	0.494481122	0.781169053	0.661960536	0.43105091	0.746861445	230.341672924761585
NONHSAG000322	0.015263186	0.815516627	0.937160913	0.649620589	0.188631042	0.494382595	240.249365469343138
NONHSAG000329	-0.26261048	3.26E-05	0.000428811	0.608207561	0.000313786	0.003393716	255.68907553381874e-05
NONHSAG000345	-0.03862702	0.408415328	0.718779718	0.591099024	0.568625828	0.831933314	260.695774174640022
NONHSAG000364	0.149893331	0.011697492	0.0749279	0.572930329	0.46378777	0.773071255	270.0328908176658207
NONHSAG000381	0.033933238	0.695659324	0.89238585	0.56121715	0.523994052	0.806497687	280.582332152614688
NONHSAG000395	0.134786452	0.011456192	0.073718106	0.552418418	0.028222877	0.147440036	290.0237642273192418
NONHSAG000423	0.125026999	0.093798285	0.355119277	0.538851418	0.829182242	0.933749876	300.186942674015011
NONHSAG000442	0.288532368	7.96E-07	1.39E-05	0.530196909	7.88E-06	0.000129579	318.93169266678815e-07
NONHSAG000476	0.068439444	0.385777463	0.706496807	0.526393581	0.264567348	0.596084801	320.499831211230012
NONHSAG000490	0.020492558	0.720882497	0.902145786	0.507613643	0.763195234	0.91052397	330.802942816318486
NONHSAG000522	-0.12862954	0.130314078	0.429206713	0.507088851	0.796049711	0.923851337	340.162552342733074
NONHSAG000529	-0.09759968	0.206461403	0.539562699	0.503491123	0.526029661	0.807421074	350.445107123419904
NONHSAG000546	0.024899512	0.693625302	0.89238585	0.490805328	0.205362576	0.515146801	360.426364217594693
NONHSAG000628	0.064065304	0.58344282	0.841786153	0.483251217	0.007877974	0.055032275	370.0188002504661751
NONHSAG000648	-0.08524433	0.19996849	0.529483422	0.462430065	0.847289317	0.942500114	380.380399901513849
NONHSAG000655	0.207952689	0.012543125	0.078906638	0.460473564	0.01544985	0.094036749	390.0186296454403329
NONHSAG000666	-0.07049186	0.420851021	0.726032559	0.455069095	0.651908358	0.874602244	400.445548668604297
NONHSAG000716	0.031665349	0.702664386	0.895955243	0.427585582	0.408805721	0.729548907	410.466555284347764
NONHSAG000738	-0.01176519	0.880288717	0.954268263	0.407709194	0.534292862	0.810810323	420.807997308217539
NONHSAG000751	0.091673181	0.159927202	0.473883344	0.399874711	0.06692343	0.273190941	430.00794024985014428
NONHSAG000769	-0.01149189	0.881120127	0.954433666	0.395855002	0.946974882	0.977208575	440.988675414955527
NONHSAG000778	-0.13340558	0.121463669	0.411759519	0.390359106	0.431065478	0.746861445	450.296331858625433
NONHSAG000812	0.154236615	0.11108438	0.392190813	0.384550312	0.244734468	0.572540204	460.254067342443359
NONHSAG000824	0.004438129	0.928747171	0.97333618	0.380791341	0.413185262	0.733255014	470.664903349841044
NONHSAG000847	-0.01406406	0.836945428	0.944345467	0.374587431	0.957165853	0.981988142	480.977147974837632
NONHSAG000884	0.053403341	0.578618459	0.838968473	0.372629265	0.393483863	0.717559417	490.683801960981096
NONHSAG000939	-0.01813324	0.84958671	0.947934194	0.370223912	0.449223674	0.756417348	500.603565761959118
NONHSAG000946	0.169525	0.012046868	0.076990439	0.367958072	0.013302973	0.084062831	510.0167594735226111
NONHSAG000965	0.039239633	0.554790617	0.821876574	0.3643478	0.215738048	0.531221884	520.459353585209117
NONHSAG000972	-0.10115683	0.108936958	0.38678122	0.3631459	0.849910089	0.942779949	530.212582879443918
NONHSAG000976	0.612013264	1.68E-08	4.22E-07	0.361144713	1.31E-05	0.000196979	545.81191727690804e-08
NONHSAG000979	-0.0929324	0.136383831	0.440017024	0.35855784	0.592921016	0.839418789	550.314764514518315
NONHSAG001012	0.217393842	0.002938007	0.023077307	0.355374218	0.002257327	0.018835617	560.0027094039894739
NONHSAG001018	0.026198798	0.612959417	0.852440007	0.354651612	0.337011478	0.671160358	570.626351724989146
NONHSAG001041	0.206113391	0.007418582	0.051381903	0.34700431	0.011209224	0.073473983	580.0114469504953675
NONHSAG001043	0.083374032	0.204390059	0.537144716	0.342214315	0.089682009	0.32545919	590.208315697655653
NONHSAG001075	0.029128599	0.69765704	0.893356829	0.34152667	0.458242912	0.767925548	600.75637797364289
NONHSAG001146	0.372589579	9.94E-08	2.15E-06	0.331849837	5.05E-07	1.13E-05	616.52410733749822e-08
NONHSAG001183	0.072934124	0.179665404	0.504113961	0.32948437	0.51031755	0.802705923	620.401203331883023
NONHSAG001185	-0.02918656	0.640857163	0.867640994	0.32414648	0.24892442	0.577684143	630.505229244166035
NONHSAG001211	-0.02118265	0.771614522	0.918276503	0.318725869	0.741360733	0.906043339	640.936294006517061
NONHSAG001222	0.168445527	0.04236435	0.203638549	0.315711083	0.008634616	0.059076741	650.022945709591794

NONHSAG001244	-0.041523	0.633116511	0.862680714	0.312938103	0.529749584	0.808158244	660.804103484730284
NONHSAG001246	-0.09449775	0.195271753	0.526466125	0.308390534	0.048313929	0.220192491	670.131361657059017
NONHSAG001247	-0.03220088	0.617949683	0.854890583	0.305421588	0.284564857	0.615265887	680.279882670565613
NONHSAG001264	0.019831688	0.745817746	0.909586883	0.295771214	0.994350488	0.997026029	690.932852795709594
NONHSAG001304	0.010256781	0.872693853	0.952744419	0.295741436	0.028261088	0.147440036	700.0502070861121971
NONHSAG001323	0.065198539	0.397724542	0.712475957	0.290682739	0.478538245	0.781355214	710.301871462890111
NONHSAG001341	-0.09918383	0.191407524	0.523025062	0.288965867	0.166513182	0.463599078	720.296434794980987
NONHSAG001391	0.032456284	0.614377287	0.852709112	0.280300165	0.171186939	0.471015334	730.378159222277404
NONHSAG001489	0.120337371	0.07156849	0.292941183	0.278771086	0.030941509	0.155927461	740.0690984829757595
NONHSAG001510	0.006905178	0.915490555	0.967869201	0.2772733	0.404136946	0.724766002	750.658475909092374
NONHSAG001530	0.863384628	1.07E-12	6.27E-11	0.276177756	9.81E-11	5.26E-09	769.80052098395894e-13
NONHSAG001569	0.030115416	0.68649261	0.887517619	0.275986337	0.639314837	0.865923354	770.681493527881133
NONHSAG001574	0.127450481	0.080422511	0.316291051	0.274808278	0.374182209	0.700534004	780.212264227860672
NONHSAG001576	-0.10476752	0.174597206	0.498537648	0.27325389	0.061600146	0.257376775	790.152925582887343
NONHSAG001587	0.386808601	1.52E-05	0.000209967	0.267614317	0.000539193	0.005453992	803.98013336613535e-05
NONHSAG001597	0.055519216	0.370151799	0.698354362	0.259510215	0.891126319	0.958708067	810.623586776260279
NONHSAG001601	0.05251407	0.522762768	0.803843214	0.257641423	0.510000481	0.802705923	820.432021158270801
NONHSAG001622	0.57461894	9.55E-10	3.12E-08	0.256275671	6.09E-06	0.000102526	834.28866379762546e-09
NONHSAG001627	0.090800292	0.166994031	0.489663415	0.255627836	0.032302679	0.160203059	840.0930166484344517
NONHSAG001640	-0.04520196	0.57157664	0.835815659	0.254301571	0.144679458	0.430061982	850.333517481528888
NONHSAG001660	0.021641556	0.800667138	0.931013112	0.253506796	0.86385988	0.948773467	860.912023350807071
NONHSAG001683	0.083892138	0.250435246	0.586231655	0.250766021	0.844301606	0.941306101	870.347104763300935
NONHSAG001696	0.350597072	0.000177374	0.00198715	0.250443338	0.012369409	0.07923184	880.000706481384002954
NONHSAG001722	0.056245175	0.590648778	0.842243593	0.244459879	0.820581201	0.933749876	890.862467372076495
NONHSAG001723	0.190636566	0.023511899	0.129131759	0.239667216	0.029845716	0.152593006	900.0384048774194606
NONHSAG001768	-0.02439845	0.779670006	0.9209289	0.238555137	0.644040231	0.869021655	910.895958745132461
DEPDC1-AS1	-1.53289003	7.85E-19	2.21E-16	0.233896616	1.38E-17	9.68E-15	28072.16807753455556e-19
NONHSAG001788	-0.01389618	0.817834766	0.938128871	0.232811982	0.73617722	0.906043339	920.848269167785351
NONHSAG001803	0.124179343	0.073149703	0.298455899	0.231667923	0.440803501	0.752824306	930.193810427881094
NONHSAG001824	-0.0036147	0.963783713	0.986588933	0.22746982	0.260220251	0.592501494	940.440398655344022
NONHSAG001832	-0.012516	0.808319789	0.93439361	0.227345219	0.842562782	0.941172323	950.905879024447292
NONHSAG001845	-0.03362853	0.565341491	0.830383739	0.225587618	0.500852544	0.797130817	960.765081321337596
NONHSAG001849	0.321695967	4.44E-07	8.05E-06	0.225358209	1.62E-05	0.000235388	977.97137741129439e-07
NONHSAG001855	-0.07109913	0.427858992	0.73236115	0.222920972	0.144984944	0.430457013	980.339834382272654
NONHSAG001898	0.051670639	0.3567053	0.682350547	0.222390146	0.625887974	0.856580631	990.360996295046182
NONHSAG001910	0.053307565	0.547102893	0.819954522	0.22238741	0.944962345	0.976206507	1000.759063546004934
NONHSAG001920	0.046226932	0.375184815	0.699615185	0.221881067	0.739541563	0.906043339	1010.665439702416522
NONHSAG001926	0.007904071	0.925584199	0.973226917	0.220415142	0.018839909	0.109912499	1020.0296756830867234
NONHSAG001938	0.035077913	0.627968189	0.85822947	0.220411771	0.195560757	0.504065232	1030.419747003329212
NONHSAG001941	0.029403879	0.57845712	0.838968473	0.220112433	0.127540677	0.40387881	1040.298892143367265
NONHSAG001965	1.078152221	8.10E-21	4.78E-18	0.219776667	4.31E-19	4.04E-16	1053.17798546166558e-21
NONHSAG001966	0.39900918	9.75E-05	0.001142394	0.218706131	0.008563178	0.05904542	1060.000391762539251438
NONHSAG001970	-0.29697416	0.00055451	0.00547117	0.217623312	0.005230733	0.038809558	1070.00137998711195792
NONHSAG001984	-0.06895287	0.286879995	0.623902976	0.21668934	0.571549434	0.833292346	1080.562248988739487
NONHSAG001992	-0.11860563	0.064306585	0.272745276	0.210915662	0.687997403	0.890389787	1090.0596918856118391
NONHSAG002015	0.016365369	0.851700338	0.948883261	0.210177342	0.861033991	0.947290977	1100.978022057000738
NONHSAG002029	0.089425997	0.196545321	0.527031094	0.208826504	0.241677712	0.56902192	1110.35872981065274
NONHSAG002048	0.054494275	0.313388651	0.651448843	0.206516857	0.944135873	0.976126639	1120.526870989536051
NONHSAG002049	0.075149238	0.406373651	0.716440568	0.203810119	0.138467099	0.424149763	1130.3271432553899
NONHSAG002063	-0.06298199	0.367878491	0.696615702	0.203119493	0.750048761	0.906696003	1140.449225139939765
NONHSAG002096	0.287833018	0.000591889	0.00577981	0.199712568	0.000321725	0.003414264	1150.000349553516277126
NONHSAG002128	-0.04584563	0.387811383	0.706496807	0.197028241	0.778124249	0.915750268	1160.674651272050693
NONHSAG002154	-0.02434098	0.622505204	0.857660281	0.193285521	0.339291163	0.672365575	1170.340555710994745
NONHSAG002156	0.099557062	0.158978881	0.472842244	0.192661174	0.434649835	0.749377889	1180.36448663421184
NONHSAG002177	0.019038492	0.770162192	0.917667832	0.189486631	0.482358439	0.783450231	1190.776692167999425
NONHSAG002178	-0.02286694	0.610964058	0.851851115	0.188782459	0.937606711	0.975416231	1200.858861286644243
NONHSAG002204	0.032668924	0.626780049	0.85822947	0.188630508	0.006270293	0.045326643	1210.0138464857948188
NONHSAG002211	0.012125117	0.785903088	0.923896106	0.187176036	0.627734848	0.857721279	1220.74359563347209
NONHSAG002218	0.08696106	0.082685176	0.32293155	0.186289119	0.879964369	0.954988833	1230.112767609707829
NONHSAG002223	0.054994351	0.552597709	0.821852872	0.18519437	0.856585133	0.946450842	1240.828599729745502
NONHSAG002277	-0.03247976	0.739617474	0.908211501	0.184588863	0.536878407	0.812140251	1250.62679349138639
NONHSAG002281	0.000387004	0.996360293	0.997424402	0.182584339	0.59190085	0.839418789	1260.824941001563012
NONHSAG002337	0.112542755	0.187253578	0.516279483	0.181095864	0.418027776	0.735603321	1270.408337390054577
NONHSAG002360	-0.07850905	0.29996327	0.637694547	0.177128763	0.661012234	0.879681213	1280.577298052888286
NONHSAG002376	-0.06810829	0.239493969	0.574037853	0.174796579	0.167931913	0.464908894	1290.327367860271023
NONHSAG002377	0.348303927	2.13E-07	4.16E-06	0.174492678	0.000407413	0.004211927	1301.03269532501081e-06
NONHSAG002389	0.138756738	0.11940384	0.409967762	0.173531117	0.236646283	0.561560632	1310.263962285111812
NONHSAG002392	-0.017547	0.822393902	0.938461188	0.173359931	0.706995821	0.896707014	1320.930015744564325
NONHSAG002406	-0.00755682	0.907546028	0.964482022	0.171302	0.757175664	0.908740063	1330.951716917779475
NONHSAG002410	0.013177527	0.84808414	0.947482162	0.171015753	0.660057662	0.878826774	1340.80912614988917
NONHSAG002419	0.081265321	0.340676167	0.673895726	0.171004821	0.346944992	0.680102659	1350.546883473266108

NONHSAG002421	0.065306984	0.296864285	0.634333108	0.17086032	0.912999267	0.967717278	1360.51418383241377
NONHSAG002440	0.22193488	0.004267003	0.031911732	0.16904721	7.98E-05	0.001015509	1370.000281454748925748
NONHSAG002455	0.152130257	0.002976573	0.023315104	0.168636725	0.938758199	0.975531432	1380.00360413496235111
NONHSAG002458	-0.09193254	0.105370144	0.378901338	0.166124683	0.224503135	0.546109703	1390.239049374031202
NONHSAG002465	0.563432584	1.24E-07	2.58E-06	0.165979177	1.84E-08	5.40E-07	1401.16948500513484e-08
NONHSAG002516	0.063778418	0.388616299	0.706496807	0.165093231	0.298937565	0.62856697	1410.53564571898216
NONHSAG002535	0.214343644	0.003207027	0.024843418	0.162654921	0.00067573	0.006574927	1420.00129898591234831
NONHSAG002538	0.08695655	0.174807298	0.498537648	0.162609802	0.953768491	0.980474941	1430.276213798180364
NONHSAG002549	-0.03044874	0.725441408	0.903322681	0.161793859	0.35298306	0.684429948	1440.417165109954233
NONHSAG002555	0.071607003	0.232172777	0.56479361	0.161375805	0.800491595	0.925568407	1450.303142745226786
NONHSAG002615	-0.05141734	0.490890212	0.777680719	0.161008718	0.198812026	0.505936125	1460.432393672568933
NONHSAG002728	0.134268928	0.122705221	0.414222188	0.160901804	0.696532225	0.895338645	1470.270818447681734
NONHSAG002758	-0.00976392	0.908471844	0.96510118	0.160054827	0.219172403	0.53727159	1480.394263865881206
NONHSAG002759	-0.00107662	0.984627884	0.995245726	0.159590006	0.318269376	0.650416776	1490.5050014124917
NONHSAG002764	0.20380858	0.028067938	0.148539573	0.159476017	0.020418601	0.11646472	1500.0341948744703311
NONHSAG002857	0.087285793	0.286565783	0.623902976	0.158940174	0.285846545	0.615425223	1510.465350513338926
NONHSAG002865	0.094835868	0.315749961	0.653820979	0.158609501	0.107221471	0.361952913	1520.262254871806941
NONHSAG002891	-0.07973643	0.220410867	0.55388325	0.157806531	0.750828043	0.906886268	1530.266792648596722
NONHSAG002908	-0.25798013	0.000591958	0.00577981	0.1570713	5.81E-06	9.96E-05	1541.915390705669e-05
NONHSAG002936	0.394866482	7.36E-09	2.03E-07	0.156739193	2.59E-06	4.75E-05	1551.93038095798512e-08
NONHSAG002956	0.066911074	0.212026308	0.544533663	0.155937716	0.617766803	0.853798275	1560.448874341534901
NONHSAG003018	-0.04691559	0.472770621	0.764982846	0.155761943	0.264716375	0.596084801	1570.523549278199654
NONHSAG003022	0.037942722	0.572949562	0.836084156	0.154509067	0.539827099	0.812140251	1580.790596252019958
NONHSAG003057	0.048206754	0.471005962	0.764534973	0.153610532	0.827258321	0.933749876	1590.757874492841714
NONHSAG003089	-0.05698451	0.607389242	0.850163538	0.153571895	0.885814128	0.957673713	1600.867124490077197
NONHSAG003123	0.098804539	0.168501184	0.491010704	0.153357489	0.478760681	0.781355214	1610.382222053033378
NONHSAG003195	-0.02626995	0.653436613	0.872489912	0.152500934	0.604085438	0.843792169	1620.624470288004355
NONHSAG003198	-0.00230063	0.973463129	0.991085561	0.152230138	0.136632013	0.422207934	1630.21990628831213
NONHSAG003202	-0.0287852	0.647721714	0.870666685	0.151088345	0.273911343	0.603068356	1640.541788613825344
NONHSAG003261	0.233692899	0.031218825	0.161280896	0.150921904	0.009497548	0.063638173	1650.0218487542008727
NONHSAG003289	0.021986193	0.730656025	0.903917617	0.150486989	0.483299255	0.783450231	1660.77937816413588
NONHSAG003322	-0.32305176	0.002672282	0.021347893	0.147466662	0.037022194	0.178876993	1670.00884050610502909
NONHSAG003325	0.398782882	0.007072364	0.049718716	0.147397892	0.003141445	0.025167358	1680.00527050121830421
NONHSAG003332	-0.09076605	0.241074843	0.575468979	0.146429176	0.383901597	0.709976889	1690.47277830436822
NONHSAG003339	-0.05758026	0.424629522	0.729325831	0.14614543	0.481206291	0.783450231	1700.326317134216434
NONHSAG003345	-0.00028453	0.998083732	0.998438795	0.145995685	0.932600747	0.973855106	1710.995034514625781
NONHSAG003405	-0.07396095	0.227704756	0.559166483	0.145441234	0.02681254	0.141990325	1720.0829789731658876
NONHSAG003407	0.234869709	0.033039891	0.167101031	0.144591123	0.93440409	0.973855106	1730.0443202706082676
NONHSAG003412	-0.09920816	0.125321314	0.419528016	0.144036609	0.753325118	0.907097076	1740.265098063680382
NONHSAG003464	0.109710425	0.074483393	0.30118702	0.142734022	0.006614115	0.047446154	1750.0218587299639101
NONHSAG003486	-0.06125107	0.501960211	0.788995033	0.14201241	0.834366464	0.936478948	1760.787026955867231
NONHSAG003512	0.177651423	0.276274545	0.612684559	0.140438366	0.288346941	0.617112355	1770.459374903527721
NONHSAG003533	0.068633218	0.258444014	0.596670416	0.14041272	0.124831604	0.398893716	1780.27907777267233
NONHSAG003536	0.056152768	0.401938315	0.714263509	0.1401889	0.395643514	0.718406651	1790.618851515071008
NONHSAG003539	0.058182253	0.385491482	0.706496807	0.140066599	0.468271359	0.776975893	1800.645204049220645
NONHSAG003554	0.029458964	0.536340296	0.813923495	0.139227859	0.557231432	0.823835324	1810.782175016256632
NONHSAG003555	-0.09685111	0.218920948	0.551618016	0.13795388	0.083227487	0.312881942	1820.201717687599816
NONHSAG003562	0.06067592	0.434565337	0.738589088	0.137784	0.687006938	0.890259682	1830.484088148904252
NONHSAG003567	-0.07442418	0.090511349	0.347227712	0.137652716	0.213785543	0.527800657	1840.212471868842507
NONHSAG003604	-0.10285789	0.233937264	0.567585494	0.136713763	0.510304308	0.802705923	1850.486374569939242
NONHSAG003613	-0.01313799	0.848752478	0.947852251	0.13659226	0.71095539	0.896907383	1860.848123213686497
NONHSAG003615	-0.12854275	0.035583965	0.177415086	0.136521103	0.054374734	0.235959492	1870.0667889289230105
NONHSAG003627	-0.03071715	0.737718693	0.90627565	0.136446818	0.590001925	0.837921926	1880.676311114681995
NONHSAG003635	-0.01004158	0.886391506	0.956660206	0.136156613	0.241814081	0.56902192	1890.352934047279758
NONHSAG003645	-0.0282142	0.624827903	0.85822947	0.136111402	0.755872147	0.907950652	1900.883180265241406
NONHSAG003668	-0.04130653	0.636101131	0.863696948	0.136090487	0.684545591	0.889213579	1910.87555093470231
NONHSAG003758	-0.00601837	0.909901331	0.965525488	0.135986345	0.537904906	0.812140251	1920.804105818223931
NONHSAG003765	-0.03537984	0.561897192	0.829618935	0.135450771	0.769798843	0.91321261	1930.843134765295666
NONHSAG003799	-0.04461072	0.457396101	0.756971211	0.135156098	0.608989743	0.847327128	1940.746009111137531
NONHSAG003829	-0.02606256	0.666056102	0.879319135	0.132225661	0.38078362	0.707708883	1950.677306535709686
NONHSAG003840	0.104886112	0.178206975	0.502625892	0.13212346	0.111549875	0.369410385	1960.228606498130757
NONHSAG003843	-0.05377844	0.328705399	0.668343876	0.131747399	0.63958848	0.865923354	1970.616214381503499
NONHSAG003854	0.010723806	0.859076025	0.951071568	0.130985861	0.734628662	0.905644804	1980.869928194096112
NONHSAG003880	0.064287801	0.277262451	0.612912715	0.129768214	0.776948502	0.915428857	1990.525023759317815
NONHSAG003894	0.141650952	0.024755283	0.134126891	0.129037251	0.572661672	0.833292346	2000.062988058614112
NONHSAG003900	0.04261164	0.503974328	0.789138617	0.128040219	0.360600582	0.689447747	2010.635645764258101
NONHSAG003927	0.279325807	0.007173512	0.050178895	0.126585497	0.007292457	0.05152359	2020.00886650775401923
NONHSAG004034	-0.03092095	0.782584401	0.921921799	0.126317466	0.63157561	0.860043881	2030.889157245036886
NONHSAG004049	0.00446808	0.949607067	0.981209884	0.125081618	0.552382207	0.820548741	2040.805831333927254
NONHSAG004092	-0.08116297	0.350322277	0.678447826	0.124825535	0.409398355	0.729548907	2050.590253005095735
C1orf132	1.107121361	3.74E-15	3.19E-13	0.123983629	5.99E-14	7.02E-12	2061.30283118894965e-15

NONHSAG004140	0.085073072	0.338808556	0.673579125	0.121985235	0.168927054	0.466165726	2070.366111919601607
NONHSAG004156	0.038970309	0.563995036	0.830383739	0.121954725	0.1469938	0.432159398	2080.338762030463291
NONHSAG004159	0.120901312	0.226345819	0.558671887	0.121700028	0.671718859	0.88168869	2090.465355885944336
NONHSAG004205	-0.06838419	0.363329688	0.691126333	0.121316064	0.047489643	0.216787135	2100.135531398575409
NONHSAG004221	-0.04909237	0.591640764	0.843230526	0.120887489	0.187256928	0.493039777	2110.409490944575215
NONHSAG004235	0.087753993	0.330723819	0.669461779	0.120730737	0.015294989	0.093498935	2120.0483420606962451
NONHSAG004261	0.03846595	0.45447473	0.756213964	0.119636995	0.995601806	0.997026029	2130.68381346950966
NONHSAG004265	0.069209419	0.321087369	0.660012926	0.11951657	0.527569222	0.807576213	2140.599849111087628
NONHSAG004289	1.065124206	8.38E-13	5.12E-11	0.119077514	1.03E-08	3.28E-07	2153.05767652027969e-12
NONHSAG004292	1.033891891	1.51E-10	6.06E-09	0.118666355	5.66E-09	1.92E-07	2161.34555208879963e-10
NONHSAG004319	-0.01408186	0.804113939	0.932342929	0.118077727	0.437474406	0.751024438	2170.726787545358606
NONHSAG004322	-0.08596383	0.215872454	0.549316015	0.118064561	0.092246091	0.332558984	2180.215875652051003
NONHSAG004342	-0.02159931	0.759672259	0.91407719	0.11705038	0.762159598	0.910423706	2190.939332054770113
NONHSAG004374	0.076291439	0.373630617	0.698899101	0.116473864	0.440382847	0.752824306	2200.623642958048211
NONHSAG004468	-0.05599331	0.276084574	0.612684559	0.116238652	0.772707661	0.91321261	2210.52423333932751
NONHSAG004469	0.051993322	0.589574272	0.842139475	0.115746817	0.357755667	0.686695518	2220.648600010975163
NONHSAG004473	0.052368614	0.53357305	0.812788416	0.115580279	0.854372245	0.945832264	2230.812518078248322
NONHSAG004494	0.025877788	0.572874262	0.836084156	0.11547587	0.939241737	0.975673352	2240.780931574240038
NONHSAG004522	0.001917704	0.975244807	0.99239987	0.115214536	0.075913991	0.294848264	2250.126684581532366
NONHSAG004544	-0.06989043	0.373451999	0.698899101	0.114833821	0.762592062	0.910423706	2260.463254355120851
NONHSAG004554	-0.05982442	0.394946255	0.711193473	0.114698052	0.975838347	0.989919709	2270.603331079698083
NONHSAG004567	0.065561347	0.521080999	0.803843214	0.11464374	0.23495117	0.558480718	2280.488393196308417
NONHSAG004569	-0.08532665	0.334391843	0.671170494	0.11426862	0.110360712	0.367694695	2290.0412458283793918
NONHSAG004584	0.06027291	0.359889184	0.686572852	0.114022999	0.075164747	0.293560094	2300.200721753973114
NONHSAG004588	0.076547639	0.235529304	0.569603028	0.113526503	0.475953977	0.780309094	2310.485582074322845
NONHSAG004592	-0.02421955	0.610487427	0.851851115	0.113347992	0.232332627	0.556934066	2320.220812960656617
NONHSAG004614	0.131088919	0.105094941	0.37869582	0.11310428	0.486628832	0.785762791	2330.261275069696009
NONHSAG004619	-0.03910614	0.404693806	0.715272773	0.112638522	0.46909127	0.77730386	2340.660412406880025
NONHSAG004632	0.065402516	0.212123469	0.544533663	0.112362892	0.821416255	0.933749876	2350.284282676389836
NONHSAG004636	-0.0032719	0.95851357	0.983730538	0.11210668	0.383034439	0.709868831	2360.616017068680134
NONHSAG004663	0.01260847	0.86652935	0.951960393	0.111990553	0.274082987	0.603068356	2370.495362806374633
NONHSAG004683	0.071384756	0.46661655	0.764261292	0.111726401	0.761228567	0.910423706	2380.762396885495381
NONHSAG004693	0.013776599	0.850174267	0.947934194	0.109386978	0.829632029	0.933749876	2390.920555721241615
NONHSAG004694	0.018982144	0.841821238	0.945700556	0.108447123	0.597468104	0.839825518	2400.865678340967997
NONHSAG004774	0.00067441	0.993764726	0.996600003	0.108260047	0.291622063	0.620937442	2410.470724711205619
NONHSAG004778	0.054487785	0.457543051	0.756971211	0.10823357	0.503754407	0.799411621	2420.712600962255537
NONHSAG004794	0.021355814	0.74863964	0.910542676	0.107948481	0.83047953	0.933749876	2430.947405628841218
NONHSAG004884	0.094909524	0.182459925	0.509107461	0.10779323	0.363636223	0.692781206	2440.391419905562152
NONHSAG004887	0.033100923	0.752157332	0.910732246	0.107644375	0.892069925	0.958708067	2450.950360320268489
NONHSAG004895	0.079009479	0.41608337	0.724427994	0.106549101	0.077680812	0.298216479	2460.205354082459524
NONHSAG004899	0.109236246	0.23085648	0.563025517	0.106075863	0.285438717	0.615425223	2470.418197270334768
NONHSAG004902	0.446266469	3.50E-08	8.14E-07	0.105929135	1.49E-09	5.92E-08	2481.31392143507988e-09
NONHSAG004908	-0.13502594	0.055252761	0.245839814	0.105534248	0.513033925	0.804430331	2490.145332480430272
NONHSAG004919	-0.035138	0.681019897	0.887517619	0.10548991	0.417197238	0.735463454	2500.716181767923986
NONHSAG004920	-0.0968813	0.081722351	0.320060238	0.105456084	0.919675489	0.970819932	2510.146881852903698
NONHSAG004943	-0.01149991	0.887259818	0.956660206	0.105356792	0.668985628	0.880630922	2520.908266373044875
NONHSAG004961	-0.01804271	0.709781473	0.898275053	0.105193221	0.464176644	0.773261091	2530.762039515635385
NONHSAG004977	0.137060469	0.024607658	0.13358443	0.105089731	0.000701665	0.006803733	2540.00263943042420055
NONHSAG005016	0.148530859	0.022519961	0.124168882	0.105083796	0.002983388	0.024038075	2550.00830258956340811
NONHSAG005033	-0.05209213	0.43687226	0.74045563	0.10504231	0.706906209	0.896707014	2560.735870320094753
NONHSAG005060	0.024252898	0.729211411	0.903322681	0.104292838	0.350047994	0.682144809	2570.635729581071744
NONHSAG005063	0.056335155	0.384351209	0.706496807	0.10427952	0.521381657	0.80637524	2580.662469611420638
NONHSAG005080	-0.00730023	0.931908735	0.974514051	0.104159419	0.974290842	0.989451568	2590.996229979448025
NONHSAG005082	-0.09542116	0.139166072	0.443294502	0.103587392	0.739965303	0.906043339	2600.295133890806095
NONHSAG005119	0.137922576	0.025888681	0.139194975	0.103524925	0.028319755	0.147472501	2610.0394826298186934
NONHSAG005138	0.072404388	0.333270012	0.669875107	0.103511129	0.33758465	0.671160358	2620.53542614255275
NONHSAG005145	-0.10866982	0.135539035	0.439096504	0.1033089	0.446343841	0.756399448	2630.323079346272689
NONHSAG005150	-0.07547479	0.269210108	0.605799463	0.102961854	0.599525524	0.840286125	2640.538080405492302
NONHSAG005165	0.145226024	0.103757457	0.37672431	0.102538306	0.859633261	0.947213452	2650.200520383407465
NONHSAG005205	-0.03435566	0.588103417	0.842139475	0.102517736	0.413277372	0.733255014	2660.704007728552833
NONHSAG005207	-0.13616515	0.0463959	0.216360317	0.102483282	0.011973264	0.077222059	2670.0301021899670092
NONHSAG005225	0.037242991	0.564398811	0.830383739	0.102346648	0.194516147	0.503664276	2680.42419238253945
NONHSAG005226	-0.02510138	0.633801341	0.862680714	0.101257399	0.138055716	0.423350789	2690.312294534991596
NONHSAG005232	-0.14592524	0.071991137	0.294242845	0.10121175	0.18864599	0.494382595	2700.174672859755174
NONHSAG005234	-0.05313421	0.449167404	0.751820679	0.100917996	0.923721808	0.971770056	2710.708841239643988
NONHSAG005239	-0.27312648	0.000286341	0.002993279	0.100078938	0.022536568	0.124504576	2720.00119073963902801
NONHSAG005282	0.005419915	0.940805619	0.977659054	0.099850492	0.799683035	0.925568407	2730.966058439204398
NONHSAG005284	0.963687357	5.53E-11	2.42E-09	0.099592968	1.49E-12	1.23E-10	2749.81291976441641e-13
NONHSAG005342	0.524153399	1.41E-08	3.60E-07	0.099498649	3.02E-09	1.13E-07	2751.36689231395884e-09
NONHSAG005356	-0.00839203	0.894699507	0.961169104	0.099475317	0.161469724	0.45587637	2760.236466631875132
NONHSAG005375	0.060003555	0.66702502	0.880185057	0.099409888	0.658508779	0.878826774	2770.879359675335959

NONHSAG005386	0.031801413	0.682746525	0.887517619	0.099218461	0.52747879	0.807576213	2780.812056069978462
NONHSAG005407	0.083812715	0.248440305	0.584125534	0.09902559	0.445140183	0.755880336	2790.49778424515502
NONHSAG005420	-0.03176564	0.643159259	0.869920075	0.098288877	0.557930808	0.824054104	2800.57444728834801
NONHSAG005464	0.066963798	0.424133975	0.729012677	0.098086793	0.824658524	0.933749876	2810.707902346911791
NONHSAG005471	0.07585365	0.169291637	0.492293777	0.096766746	0.692858845	0.894134499	2820.361999727542914
NONHSAG005501	0.131375394	0.123181203	0.414832985	0.096725683	0.053292663	0.232952053	2830.123219132238879
NONHSAG005503	0.405310803	0.00036717	0.003754482	0.096709593	0.002868484	0.023245463	2840.000804934383677842
NONHSAG005505	-0.11429895	0.053161171	0.23880066	0.095351788	0.338435254	0.67209035	2850.150088927716722
NONHSAG005523	0.063460537	0.480179306	0.76981996	0.095348317	0.071157414	0.282879495	2860.186426028838373
NONHSAG005543	0.170085966	0.012092319	0.077105672	0.095196001	0.000904004	0.008530399	2870.00269110060646704
NONHSAG005545	-0.01425209	0.788560194	0.924700277	0.094931899	0.306707574	0.635739391	2880.565674032534437
NONHSAG005589	0.08348117	0.235580743	0.569603028	0.09490056	0.587393304	0.836463428	2890.488964668339629
NONHSAG005603	0.121870119	0.169684972	0.492927831	0.094450722	0.097011192	0.34270788	2900.205659079588534
NONHSAG005687	0.11782618	0.360382242	0.687047365	0.094443137	0.762264247	0.910423706	2910.447445972817262
NONHSAG005692	0.028056464	0.745701295	0.909586883	0.094133835	0.305141387	0.634657974	2920.371027318984927
NONHSAG005715	0.070188006	0.270753979	0.608115167	0.093540021	0.419990342	0.737671981	2930.518172687811745
NONHSAG005734	0.051592374	0.416566802	0.724427994	0.093320802	0.592106274	0.839418789	2940.70771658678162
NONHSAG005754	0.002911489	0.967969623	0.987996581	0.093232284	0.291699275	0.620937442	2950.486174541325401
NONHSAG005762	-0.15280022	0.10987811	0.389140108	0.09300826	0.103400912	0.353149595	2960.175527133669747
NONHSAG005767	0.021911801	0.694361242	0.89238585	0.092569867	0.587785112	0.836463428	2970.85303462304594
NONHSAG005812	-0.03124765	0.644288142	0.870030385	0.092564539	0.329678741	0.663397232	2980.343128392105783
NONHSAG005834	0.322966616	0.000218438	0.002380802	0.092549307	0.028161934	0.147440036	2990.000957707336760605
NONHSAG005850	0.004308371	0.947653828	0.98065537	0.092281971	0.847640539	0.942500114	3000.980838639185859
NONHSAG005870	-0.09813108	0.203464093	0.536312792	0.092193145	0.029980116	0.152724793	3010.0903196134556431
NONHSAG005912	0.126110039	0.045333953	0.212111605	0.092003894	0.080528812	0.306009485	3020.0932158141331642
NONHSAG005923	-0.44974462	0.005908956	0.042065782	0.091868803	0.201561591	0.509172255	3030.0211386451655667
NONHSAG005953	0.021604365	0.796082236	0.927815057	0.091742112	0.593439132	0.839418789	3040.865369639815107
NONHSAG005957	0.039978578	0.485135999	0.773796046	0.091143731	0.406815526	0.728176486	3050.668816373762431
NONHSAG005965	0.10742746	0.083196949	0.324479639	0.090685462	0.111715226	0.369410385	3060.156679600221992
NONHSAG005970	-0.07770163	0.119711946	0.41052438	0.090452479	0.254068576	0.58512763	3070.27005777791198
NONHSAG006053	-0.058412	0.272215652	0.60896612	0.090357356	0.126283466	0.401707133	3080.285306065355784
NONHSAG006081	0.562165566	2.53E-05	0.000342347	0.090174611	1.93E-05	0.000277852	3091.17684694199662e-05
NONHSAG006083	-0.07898044	0.386466969	0.706496807	0.089787951	0.0850706	0.316426624	3100.222285979490376
NONHSAG006087	0.194976208	0.032445705	0.165151222	0.089667624	0.003159593	0.025240838	3110.00975287049043151
NONHSAG006140	-0.02664618	0.753005858	0.910732246	0.089376025	0.888487036	0.958708067	3120.895945709339995
NONHSAG006147	0.063889729	0.401421308	0.713976418	0.089358809	0.462117302	0.771493351	3130.654851204811476
NONHSAG006149	0.098079287	0.197168105	0.527031094	0.089322701	0.276037016	0.605180312	3140.378616184669511
NONHSAG006187	-0.0045071	0.935323574	0.975411819	0.088367174	0.229632383	0.55332156	3150.354751824273471
NONHSAG006196	-0.05992973	0.344622707	0.677284107	0.088299636	0.871929306	0.950957653	3160.595727869699756
NONHSAG006210	-0.11035557	0.088513403	0.340492052	0.088298249	0.789842658	0.921569044	3170.183413739038446
NONHSAG006244	-0.03401159	0.709452848	0.898275053	0.088018616	0.084489375	0.315098308	3180.0854717493119787
NONHSAG006270	-0.01288764	0.795586876	0.927815057	0.087836884	0.335777131	0.671076968	3190.43592985137351
NONHSAG006274	-0.04261534	0.555578867	0.821876574	0.087721528	0.089423548	0.32545919	3200.220837868779448
NONHSAG006311	0.082931003	0.351222821	0.679723725	0.087548135	0.415309299	0.734035039	3210.593877180479753
NONHSAG006312	-0.0063598	0.934423128	0.975411819	0.087050075	0.585505247	0.836463428	3220.839224541167113
NONHSAG006326	0.012211082	0.809916155	0.93439361	0.086567491	0.437465823	0.751024438	3230.725725110729358
NONHSAG006406	0.005841455	0.95075486	0.981209884	0.086197241	0.242147269	0.569329532	3240.379726840603111
NONHSAG006407	0.091542407	0.267981713	0.605799463	0.085297245	0.505712845	0.80071508	3250.532575651164722
NONHSAG006417	-0.02912509	0.689572995	0.889894109	0.085011775	0.374274732	0.700534004	3260.669104762505313
NONHSAG006458	0.108168563	0.056938943	0.251067337	0.084516413	0.022651789	0.124895745	3270.0509538378014783
NONHSAG006506	0.011615147	0.84686984	0.946877928	0.0843639	0.739913382	0.906043339	3280.945175505477455
NONHSAG006557	0.157568302	0.053475738	0.239830584	0.0843235	0.989665505	0.996597046	3290.0851269709123627
NONHSAG006570	-2.52952526	1.12E-20	5.25E-18	0.084190805	8.57E-15	1.27E-12	3305.15347763818207e-20
NONHSAG006585	0.107979617	0.266391122	0.604403572	0.083269944	0.850872249	0.943475065	3310.488301065476271
NONHSAG006591	-0.27685079	0.017932427	0.105054134	0.082920001	0.352303407	0.684429948	3320.0554848822987721
NONHSAG006615	0.460783309	5.36E-08	1.19E-06	0.082918373	2.46E-06	4.61E-05	3338.50029929767582e-08
NONHSAG006619	0.254430981	6.94E-05	0.000855964	0.082539065	0.000360562	0.003769147	3340.000101704650546825
NONHSAG006623	0.14464662	0.121345385	0.411759519	0.082145544	0.183007627	0.488163241	3350.243119573537113
NONHSAG006631	0.271311379	0.048573001	0.223547101	0.08201023	0.593173277	0.839418789	3360.121136648148467
NONHSAG006633	0.024633587	0.677551135	0.887517619	0.08093041	0.995112049	0.997026029	3370.888200796959756
NONHSAG006679	-0.02826942	0.625717131	0.85822947	0.080718261	0.497479048	0.794835843	3380.780273826067217
NONHSAG006690	0.124375356	0.223705903	0.556499409	0.080561345	0.587125596	0.836463428	3390.470462083112546
NONHSAG006700	-1.37091705	1.18E-16	1.27E-14	0.080141704	1.06E-12	9.62E-11	3402.92284864459167e-16
NONHSAG006702	0.4980649	8.80E-11	3.69E-09	0.079952618	5.44E-09	1.87E-07	3419.17405944660913e-11
NONHSAG006706	0.202704363	0.021380569	0.119908916	0.07992584	0.196105037	0.504065232	3420.0677062308882069
NONHSAG006711	0.105997025	0.076092883	0.305239924	0.07979293	0.138754343	0.424269621	3430.162981632599434
NONHSAG006726	0.063461741	0.215001379	0.548282042	0.079614908	0.203845312	0.512331603	3440.347101915739493
NONHSAG006750	-0.06316883	0.274409786	0.612084166	0.079270459	0.155716273	0.445443044	3450.327917039485244
NONHSAG006793	-0.10531888	0.130494613	0.429206713	0.078957383	0.265086306	0.596338153	3460.289512244613416
NONHSAG006798	0.084536927	0.24890048	0.584718587	0.078284726	0.00257974	0.021149357	3470.00901493131582546
NONHSAG006806	0.073185123	0.331874011	0.669461779	0.077771138	0.142950292	0.430061982	3480.325384707073324

NONHSAG006870	0.030974444	0.683485528	0.887517619	0.077310625	0.115603178	0.377995506	3490.258602997492883
NONHSAG006890	0.188636449	0.001853803	0.015514567	0.077256392	0.000330483	0.003493675	3500.000620472403998267
NONHSAG006892	0.033327946	0.625075812	0.85822947	0.077032529	0.345738576	0.678791662	3510.348922309979852
NONHSAG006896	0.025599852	0.703931795	0.896492848	0.077030444	0.977457828	0.990519194	3520.913113042352213
NONHSAG006898	0.204782686	0.120814386	0.411133305	0.076971674	0.018943772	0.110289622	3530.0569405381346551
NONHSAG006900	-0.1030853	0.188005047	0.516279483	0.076636569	0.195762714	0.504065232	3540.318912718793329
NONHSAG006943	-0.03023836	0.728220827	0.903322681	0.076455419	0.792918412	0.922059223	3550.93561702818643
NONHSAG006949	0.012313755	0.82465091	0.938461188	0.076264601	0.95382996	0.980474941	3560.973566414660849
NONHSAG006969	0.024900325	0.67714175	0.887517619	0.075921872	0.235622805	0.559604162	3570.479561409509365
NONHSAG006971	0.276154231	0.00015434	0.001742989	0.075446564	2.66E-05	0.000376551	3583.61127417300671e-05
NONHSAG006980	0.066996039	0.26743908	0.605756381	0.075240756	0.754417991	0.907341414	3590.328374680770348
NONHSAG006992	0.217711028	0.002213275	0.018039796	0.075168452	2.57E-06	4.75E-05	3601.24416513306433e-05
NONHSAG007038	0.300580672	0.001439776	0.012419176	0.075126549	2.37E-06	4.46E-05	3611.08363654958833e-05
NONHSAG007047	-0.23071286	0.075215265	0.302839507	0.075123528	0.975314936	0.989745796	3620.117237024784829
NONHSAG007108	-0.01941614	0.804460632	0.932342929	0.074675409	0.74933081	0.906696003	3630.849233720267799
NONHSAG007115	-0.04902978	0.336972592	0.67346619	0.074619161	0.262062976	0.592856869	3640.476022162964287
NONHSAG007133	-0.0834021	0.327265936	0.667347218	0.0745831	0.10999089	0.367694695	3650.268733141405384
NONHSAG007156	-0.02078344	0.783094447	0.922063468	0.074240382	0.70682175	0.896707014	3660.805766738217522
NONHSAG007158	0.006169251	0.938405561	0.976844441	0.074236441	0.765195654	0.911884893	3670.952418441154343
NONHSAG007166	0.014915644	0.864047928	0.951839172	0.074175994	0.035161811	0.170902312	3680.0420573702950336
NONHSAG007181	0.38739403	1.43E-05	0.000199754	0.074143207	6.02E-06	0.000101906	3694.33330274820478e-06
NONHSAG007207	0.137054208	0.033394875	0.168593158	0.074045193	0.003120995	0.025074963	3700.00973951426956718
NONHSAG007221	0.110358065	0.245468099	0.581513307	0.073732134	0.174172809	0.475047467	3710.337503381898349
NONHSAG007222	0.10652884	0.12033006	0.411133305	0.073481633	0.233559062	0.557433838	3720.26420608540865
NONHSAG007253	-0.00906223	0.896087658	0.961169104	0.073264174	0.534953513	0.811375016	3730.722351822800972
NONHSAG007359	-0.66567112	2.44E-07	4.67E-06	0.072845391	5.09E-06	8.79E-05	3743.30528373663276e-07
NONHSAG007368	0.039827152	0.509946953	0.796208124	0.0728047	0.661461418	0.879862586	3750.54295605351522
NONHSAG007373	0.028108199	0.568167335	0.832996114	0.072769262	0.071094795	0.282879495	3760.177634867855366
NONHSAG007386	-0.12739791	0.208473282	0.542233781	0.072717496	0.482751938	0.783450231	3770.145672977060247
NONHSAG007407	0.086314931	0.275613801	0.612684559	0.072595703	0.102064743	0.350434746	3780.246640557898794
NONHSAG007504	0.000551105	0.993048073	0.996592142	0.072585166	0.530820866	0.808158244	3790.764272547214264
NONHSAG007506	-0.01200274	0.810117931	0.93439361	0.072484993	0.440121643	0.752824306	3800.728576487577277
NONHSAG007524	0.008661839	0.912319081	0.966267893	0.0723412	0.25579532	0.586701827	3810.451917468612196
NONHSAG007531	0.122523616	0.121916771	0.412055242	0.072265024	0.066939466	0.273190941	3820.142556670883361
NONHSAG007550	0.153920911	0.175639393	0.499391278	0.071877853	0.921519064	0.9716204	3830.3165525374378
NONHSAG007560	0.081977022	0.167217839	0.489780011	0.07173843	0.257882813	0.589799224	3840.335578555224707
NONHSAG007562	0.019228924	0.705744002	0.897987391	0.071697878	0.943324139	0.976126639	3850.888875491359487
NONHSAG007577	0.026665586	0.724826403	0.903322681	0.071590352	0.695082612	0.895338645	3860.909988102506699
NONHSAG007580	0.091860842	0.24006704	0.574037853	0.071569113	0.854481758	0.945832264	3870.445402151763979
NONHSAG007590	0.411573194	0.001515298	0.013030634	0.07156593	1.54E-05	0.000226053	3885.48714204659064e-05
NONHSAG007653	0.086592917	0.302041423	0.640635481	0.071334378	0.840221803	0.940566763	3890.545240183438427
NONHSAG007683	0.147819671	0.102260594	0.372966004	0.071318213	0.92730938	0.972619909	3900.183586529865845
NONHSAG007686	0.038083947	0.566003389	0.830689734	0.070566774	0.594540475	0.839418789	3910.812647518481118
NONHSAG007695	0.06257779	0.212623742	0.544533663	0.070477572	0.248875021	0.577684143	3920.37861654039516
NONHSAG007714	0.137790033	0.138615319	0.443294502	0.070368917	0.932500709	0.973855106	3930.248345464224093
NONHSAG007751	0.850546784	5.97E-17	6.72E-15	0.070367193	1.76E-16	5.50E-14	3948.30735832251985e-18
NONHSAG007766	0.09725449	0.35254807	0.680247895	0.070347976	0.614629839	0.850979373	3950.349653269240967
NONHSAG007767	0.192188555	0.02419179	0.132091871	0.070230067	0.147176138	0.432159398	3960.0721037656595156
NONHSAG007770	0.020105921	0.750020901	0.910732246	0.070171695	0.165887734	0.462866185	3970.343882411349027
NONHSAG007825	0.069696154	0.499005553	0.78522866	0.069908632	0.965924134	0.986709541	3980.748009440047659
NONHSAG007828	0.054048612	0.275547743	0.612684559	0.069593921	0.287424937	0.616551056	3990.458164017523348
NONHSAG007837	-0.08322668	0.344997297	0.677284107	0.069506656	0.604402562	0.843792169	4000.335542613704331
NONHSAG007839	0.127397279	0.093324646	0.354512009	0.069419756	0.432789599	0.748005133	4010.239067289075975
NONHSAG007862	0.194918346	0.007125143	0.049964842	0.069419247	0.002069071	0.017472155	4020.00401060553394472
NONHSAG007868	0.021151472	0.792289563	0.927698609	0.069281138	0.122082027	0.391575432	4030.250554170856391
NONHSAG007885	-0.06776803	0.153495119	0.464946969	0.068985517	0.538301616	0.812140251	4040.11715186830601
NONHSAG007890	0.05695663	0.346444215	0.677284107	0.06880725	0.282650618	0.613093982	4050.500166969050874
NONHSAG007906	0.08162517	0.117015424	0.404732317	0.068784904	0.725535228	0.902489903	4060.253852610547425
NONHSAG007910	0.001125013	0.979838184	0.993501254	0.068637381	0.422807042	0.740637724	4070.656963963762218
NONHSAG007917	-0.04573481	0.338165406	0.673579125	0.068465139	0.400245801	0.722858826	4080.575793422927323
NONHSAG007923	0.102822041	0.047925516	0.221291545	0.068402858	0.177243298	0.48167889	4090.127740196830858
NONHSAG007937	0.021525686	0.725847872	0.903322681	0.068383258	0.725650665	0.902489903	4100.920203313743778
NONHSAG007942	0.058851265	0.346169568	0.677284107	0.068161876	0.764170016	0.911300291	4110.62501279105239
NONHSAG007976	0.019957613	0.712301241	0.898275053	0.068082675	0.270695973	0.600313151	4120.528365788344977
NONHSAG007987	0.112570083	0.157489723	0.471128832	0.06791817	0.474754515	0.780269093	4130.363004106832802
NONHSAG008006	0.043437892	0.53618596	0.813923495	0.067817932	0.813668852	0.931603479	4140.820561544980265
NONHSAG008015	-0.08801485	0.316516131	0.654587752	0.067700652	0.01275507	0.081147637	4150.0410067069910414
NONHSAG008021	0.021146586	0.685922276	0.887517619	0.067505131	0.890796974	0.958708067	4160.851753781648147
NONHSAG008036	-0.02642812	0.720761222	0.902145786	0.067280526	0.42128507	0.738562105	4170.492158060824533
NONHSAG008052	-0.70198396	1.10E-08	2.96E-07	0.067183199	0.000645122	0.006342943	4186.55865227818413e-08
NONHSAG008061	0.016185515	0.860376125	0.95118497	0.067116395	0.109114419	0.36622905	4190.210709495745306

NONHSAG008068	-0.06453664	0.313130327	0.651448843	0.067039871	0.13947888	0.424928836	4200.315969263499708
NONHSAG008074	0.063982288	0.172994829	0.497084073	0.066962235	0.532721646	0.810050899	4210.388930396002005
NONHSAG008085	-0.01728673	0.711054754	0.898275053	0.066956318	0.540079043	0.812140251	4220.609444575250367
NONHSAG008088	0.069000085	0.422877916	0.727871783	0.06688508	0.476378285	0.780309094	4230.676841645983454
NONHSAG008123	0.048260848	0.542728944	0.817035827	0.066872696	0.281350731	0.612446652	4240.553483772511985
NONHSAG008125	0.014989388	0.801879207	0.931770384	0.066631562	0.587044776	0.836463428	4250.7173654233293
NONHSAG008197	-0.09347042	0.201235522	0.531836737	0.066583785	0.445141478	0.755880336	4260.432718911111709
NONHSAG008233	0.072634867	0.267549582	0.605756381	0.066307965	0.847755469	0.942500114	4270.372309232777432
NONHSAG008613	0.041202285	0.549070896	0.820817175	0.066009009	0.094426602	0.336361438	4280.23262648054829
NONHSAG008637	0.080870807	0.228617862	0.559166483	0.065847503	0.307603238	0.635739391	4290.427878578059527
NONHSAG008664	-0.49960586	2.72E-09	8.15E-08	0.065764493	0.002035046	0.017236595	4301.51005862755097e-08
NONHSAG008670	1.233406194	1.30E-12	7.48E-11	0.065696127	1.29E-11	9.08E-10	4314.86582333592006e-13
NONHSAG008671	1.621023488	2.45E-13	1.64E-11	0.065318714	1.14E-12	1.00E-10	4325.90166612558382e-14
NONHSAG008684	0.148151619	0.096778502	0.360872845	0.065256281	0.144391499	0.430061982	4330.192293350627718
NONHSAG008765	0.026299812	0.7259884	0.903322681	0.065095771	0.888907311	0.958708067	4340.938800845555664
NONHSAG008818	0.166012429	0.0732342	0.298455899	0.065080658	0.374116968	0.700534004	4350.0290139589770291
NONHSAG008819	-0.13433162	0.194387604	0.526251063	0.064875806	0.29277786	0.622291263	4360.383389686973486
NONHSAG008903	0.019611319	0.824564261	0.938461188	0.064609077	0.539546605	0.812140251	4370.685694099059171
NONHSAG008932	0.132731812	0.033537038	0.169007439	0.064543447	0.059119844	0.249242881	4380.0669747010946843
NONHSAG008943	-0.15964772	0.00844802	0.057520173	0.064462411	0.009159415	0.062213223	4390.0110807611804059
NONHSAG008986	0.022646537	0.732099325	0.904906946	0.064428953	0.989572083	0.996597046	4400.920964930769892
NONHSAG009036	0.024978053	0.762182746	0.915035797	0.064405123	0.786210453	0.920026547	4410.846548479559562
NONHSAG009072	-0.04313367	0.484990337	0.773796046	0.064343259	0.559625145	0.825637937	4420.752830077823543
NONHSAG009076	0.013782467	0.852642068	0.949198758	0.063958214	0.704062354	0.896707014	4430.929427008536561
NONHSAG009130	-0.55108146	6.94E-12	3.55E-10	0.063248398	7.67E-08	2.00E-06	4442.72076818343251e-11
NONHSAG009132	0.12906482	0.096278132	0.360018759	0.062556548	0.581640906	0.836050856	4450.232824023919712
NONHSAG009142	-0.10327643	0.096891536	0.360872845	0.062464529	0.428702389	0.746182739	4460.0495372191149767
NONHSAG009168	-0.21449613	0.000120636	0.001385962	0.062393508	1.09E-05	0.000171791	4471.75278065940168e-05
NONHSAG009184	0.030059993	0.66010566	0.875035827	0.062379625	0.146864022	0.432159398	4480.325308661910772
NONHSAG009186	0.029028613	0.710422941	0.898275053	0.062343246	0.35634494	0.686695518	4490.412182020518282
NONHSAG009191	-0.11746725	0.116262646	0.403120297	0.06229119	0.056211885	0.239714801	4500.124904078921042
NONHSAG009210	0.013844756	0.897934295	0.961169104	0.062273102	0.833586983	0.936120846	4510.977505089813578
NONHSAG009216	-0.12124734	0.099546745	0.36543792	0.062250001	0.30733759	0.635739391	4520.247101493456987
NONHSAG009222	0.037983679	0.683545706	0.887517619	0.062168448	0.022500032	0.124504576	4530.0503905047152891
NONHSAG009269	-0.06072123	0.455019213	0.756213964	0.06127223	0.541477815	0.812737703	4540.39854449018581
NONHSAG009275	0.026006205	0.696617896	0.892432585	0.060867933	0.786890487	0.920056569	4550.922269939705294
NONHSAG009283	0.544851053	8.09E-13	5.06E-11	0.060822667	1.17E-09	4.75E-08	4561.77657839762552e-12
NONHSAG009302	-0.05941354	0.239687088	0.574037853	0.060746921	0.544695814	0.815012106	4570.495982061109449
NONHSAG009308	-0.05487864	0.533940604	0.812907947	0.060720113	0.765536105	0.911884893	4580.82173604704512
NONHSAG009381	0.034481373	0.583844578	0.841933823	0.060600816	0.551759923	0.820491224	4590.801217526949628
NONHSAG009397	-0.07476427	0.205561506	0.537710656	0.060575377	0.412527705	0.733255014	4600.117166545079663
NONHSAG009424	-0.01732817	0.808055176	0.93439361	0.059949684	0.146616184	0.432159398	4610.188383688419888
NONHSAG009434	0.000813048	0.989695447	0.996279605	0.059856111	0.328343139	0.662814721	4620.529959827657075
NONHSAG009452	0.145458592	0.043081022	0.205677136	0.059481229	0.019202533	0.111565127	4630.040463401912738
NONHSAG009462	-0.04347543	0.575420332	0.836650451	0.059176423	0.261078977	0.592856869	4640.526995330761951
NONHSAG009465	-0.01571506	0.823254182	0.938461188	0.058979452	0.957892416	0.981988142	4650.972731654100252
NONHSAG009524	-0.10431037	0.095815969	0.360018759	0.058647032	0.494250985	0.792379572	4660.240890337161236
NONHSAG009530	0.009093153	0.896865606	0.961169104	0.058254519	0.803083041	0.927040029	4670.927596219557742
NONHSAG009590	0.0492646	0.353530891	0.680362681	0.057805452	0.964737678	0.986709541	4680.574693341715458
NONHSAG009627	-0.15305046	0.007789365	0.053423648	0.057614003	0.003294182	0.026020335	4690.00569942179817651
NONHSAG009630	-0.12830504	0.067602417	0.283097712	0.057409183	0.397659584	0.719574486	4700.182966225084967
NONHSAG009643	0.566549509	1.38E-08	3.56E-07	0.057361659	1.44E-08	4.36E-07	4713.32130932204702e-09
NONHSAG009663	0.004481535	0.940463037	0.977659054	0.056770143	0.517241084	0.804430331	4720.775353549127554
NONHSAG009678	0.100442772	0.216864814	0.549316015	0.05655104	0.18810259	0.494340639	4730.334454652847088
NONHSAG009688	-0.02240508	0.794419767	0.927815057	0.056511712	0.942085254	0.976126639	4740.963981253403632
NONHSAG009690	-0.01655885	0.832111468	0.942580607	0.056492675	0.619863907	0.853798275	4750.765964817474873
NONHSAG009700	-0.04204689	0.543745044	0.817395852	0.056489095	0.144098848	0.430061982	4760.110256356142893
NONHSAG009711	-0.02132293	0.736474923	0.905538909	0.056220081	0.919724146	0.970819932	4770.899236055645127
NONHSAG009736	0.031272526	0.695368098	0.89238585	0.056171675	0.94087426	0.976126639	4780.880871912764429
NONHSAG009783	0.07236844	0.347695044	0.677785637	0.056134117	0.770601452	0.91321261	4790.436853151995786
NONHSAG009834	-0.45535224	3.96E-07	7.24E-06	0.055814619	1.13E-05	0.000176412	4806.40316450663638e-07
NONHSAG009878	-0.03458168	0.402542313	0.714263509	0.055560889	0.503141376	0.799065289	4810.67227078505743
NONHSAG009880	0.045152492	0.390928124	0.707849249	0.055530166	0.093705286	0.336361438	4820.240554271477372
NONHSAG009903	0.033439071	0.608125775	0.850770985	0.055448362	0.678799204	0.88533551	4830.648331643669906
NONHSAG009919	0.051847092	0.388299236	0.706496807	0.055433669	0.306298778	0.635654734	4840.542343184865247
NONHSAG009921	0.008626761	0.901670963	0.962603929	0.055418816	0.572911169	0.833292346	4850.836842359292663
NONHSAG009942	0.151481271	0.012262784	0.077735857	0.055374581	0.040108548	0.191161417	4860.0289315450936082
NONHSAG009977	0.010522563	0.886154416	0.956660206	0.055168342	0.627580748	0.857721279	4870.881569779807554
NONHSAG009994	-0.24205093	0.002370321	0.019098407	0.054885644	0.00568439	0.041954082	4880.00394322951492447
NONHSAG010005	0.084450687	0.222205062	0.555413897	0.054862858	0.233382812	0.557433838	4890.376254120839824
NONHSAG010007	0.053045339	0.372776416	0.698899101	0.054784107	0.38981462	0.713031652	4900.596264735996092

NONHSAG010015	0.048432881	0.450156566	0.752016606	0.054727791	0.668113221	0.880630922	4910.487404358761224
NONHSAG010022	-0.04746463	0.610447879	0.851851115	0.054704143	0.745142425	0.906696003	4920.700231340589924
NONHSAG010026	0.000840483	0.990484448	0.996279605	0.054618246	0.111400206	0.369410385	4930.18169219120567
NONHSAG010034	-0.00586697	0.929030692	0.97333618	0.05458549	0.868236838	0.950047122	4940.966624730234752
NONHSAG010061	0.093993068	0.057066356	0.251127687	0.054578535	0.052572931	0.230992316	4950.0860373458400814
NONHSAG010086	0.060844613	0.344176356	0.677284107	0.054549692	0.035010205	0.170621655	4960.10383248228906
NONHSAG010089	0.058063556	0.346289747	0.677284107	0.054402925	0.189675102	0.496154779	4970.396876731284008
NONHSAG010109	0.037624371	0.60441996	0.848743629	0.054331165	0.573247302	0.833292346	4980.820010520410928
NONHSAG010134	0.877875513	9.22E-12	4.47E-10	0.05418596	4.38E-09	1.54E-07	4991.67257901455254e-11
NONHSAG010144	-0.19509703	0.034487187	0.172866259	0.054146485	0.244191974	0.571746738	5000.103200563531714
NONHSAG010152	0.065723529	0.225971155	0.558671887	0.054138784	0.031459433	0.158030541	5010.0949901729365109
NONHSAG010157	0.023771615	0.744392646	0.909586883	0.053789523	0.574905141	0.833526329	5020.851379873776354
NONHSAG010160	0.010110496	0.905275967	0.964289176	0.053598587	0.006342889	0.045733856	5030.0090398531658364
NONHSAG010163	0.076720922	0.265778802	0.603793395	0.053564903	0.349073199	0.680716946	5040.485550883519892
NONHSAG010226	0.176430296	0.005892005	0.042065782	0.053474048	1.24E-05	0.000189424	5055.88974239058485e-05
NONHSAG010232	0.026880404	0.68096481	0.887517619	0.05345169	0.23921275	0.566217384	5060.484712712450442
NONHSAG010260	-0.04356364	0.533256274	0.812788416	0.053408425	0.999117896	0.999117896	5070.769149665365294
NONHSAG010262	0.207485929	0.016270783	0.096526247	0.053152393	0.140456982	0.426067997	5080.0517342530603979
NONHSAG010264	0.185147943	0.010139502	0.06724594	0.053040014	0.000320229	0.003414264	5090.00111152632869432
NONHSAG010352	0.181054118	0.00060486	0.005885353	0.052709112	0.009109041	0.062020881	5100.00179393697613982
NONHSAG010413	0.020969611	0.726081209	0.903322681	0.052589446	0.700699098	0.895983652	5110.912297980425773
NONHSAG010420	0.312475726	5.82E-05	0.000730048	0.05243828	3.49E-07	8.04E-06	5121.0207905314928e-06
NONHSAG010447	0.030764044	0.647084035	0.870666685	0.052040025	0.685120804	0.889213579	5130.880489831610987
NONHSAG010455	0.600480724	8.46E-12	4.17E-10	0.051937872	1.30E-10	6.77E-09	5144.28151951054912e-12
NONHSAG010492	0.022403593	0.687100234	0.887517619	0.051719869	0.94397067	0.976126639	5150.876359589171857
NONHSAG010499	0.043869131	0.39959955	0.712991076	0.051560986	0.185676591	0.490255938	5160.403203404160439
NONHSAG010524	0.054834333	0.326264024	0.666754676	0.051092926	0.130807808	0.410276175	5170.304688067218498
NONHSAG010532	-0.4825249	2.27E-07	4.39E-06	0.050919174	3.00E-05	0.000419513	5185.94967491794326e-07
NONHSAG010566	-0.05238098	0.450354183	0.752016606	0.050848562	0.448141269	0.756417348	5190.32182398624838
NONHSAG010571	0.662324628	5.56E-10	1.93E-08	0.050704895	9.32E-13	8.73E-11	5201.59578086271976e-12
NONHSAG010573	-0.30402711	0.000652326	0.006281993	0.05029219	0.12822432	0.405131222	5210.00263792039873844
NONHSAG010585	-0.16466088	0.012398854	0.078173941	0.050272964	0.26767923	0.598817815	5220.0411273621353201
NONHSAG010628	0.08543007	0.438357658	0.740968838	0.049882839	0.223177375	0.543354787	5230.463172903012691
NONHSAG010678	0.044900049	0.569211888	0.833658244	0.049279455	0.309223478	0.637957756	5240.590596815762722
NONHSAG010682	0.083877077	0.151383502	0.461203041	0.049266672	0.347803168	0.680716946	5250.341733990540616
NONHSAG010721	-0.04268218	0.461141233	0.760209241	0.049243376	0.191627392	0.498018695	5260.419492351089539
NONHSAG010724	0.013706441	0.862845553	0.951781505	0.049046538	0.201521636	0.509172255	5270.285147022891203
NONHSAG010765	0.037736089	0.604402904	0.848743629	0.049014053	0.362666056	0.691401321	5280.655260096387043
NONHSAG010767	0.137597283	0.018373075	0.106772915	0.048981541	0.006989946	0.049761336	5290.0140590596893761
NONHSAG010791	0.007986626	0.87232158	0.952744419	0.048850247	0.812363302	0.931253814	5300.970669444071822
NONHSAG010808	-0.00441171	0.949889089	0.981209884	0.048742351	0.597821992	0.839825518	5310.80832782307463
NONHSAG010846	0.105991265	0.377172446	0.701925161	0.048676684	0.450531017	0.757258351	5320.631014931300395
NONHSAG010848	0.159442004	0.155045979	0.467298276	0.048563662	0.001881898	0.016133834	5330.00726717847946964
NONHSAG010888	0.00427866	0.935848484	0.975411819	0.048487366	0.599913356	0.840286125	5340.850599550318978
NONHSAG010892	0.183571673	0.077756384	0.311025534	0.04839252	0.104909355	0.357149038	5350.146226373127467
NONHSAG010908	0.036285196	0.515801474	0.799108873	0.048370505	0.163681486	0.458438583	5360.373185698874372
NONHSAG010921	0.152569584	0.040280459	0.195628066	0.048269689	0.027265464	0.143577689	5370.0489674305235531
NONHSAG010938	0.078892054	0.242229437	0.575614548	0.047929597	0.02000118	0.115017012	5380.0642693239578347
NONHSAG010946	-0.0823114	0.33924126	0.673579125	0.047902149	0.348551274	0.680716946	5390.546933776573381
NONHSAG010964	0.152158138	0.030623524	0.15888072	0.047786752	0.129323979	0.407232954	5400.0840849586651598
NONHSAG010989	-0.1066529	0.150836282	0.461203041	0.047651593	0.356049627	0.686695518	5410.342340300244202
NONHSAG010991	0.071582219	0.218350191	0.550673307	0.047546635	0.304268654	0.634657974	5420.415429988888551
NONHSAG010992	0.430860368	0.041283188	0.199291129	0.047402528	0.000662757	0.006493632	5430.00272281520356207
NONHSAG011000	0.011604142	0.868416639	0.951960393	0.047268894	0.689031031	0.89123977	5440.921251259218662
NONHSAG011016	0.057829702	0.388144776	0.706496807	0.047150934	0.48771372	0.786603727	5450.654905173623787
NONHSAG011035	0.143843227	0.184190441	0.511801898	0.04714094	0.582417088	0.836050856	5460.405023585433576
NONHSAG011091	0.007688314	0.89067068	0.958502087	0.046996635	0.264761706	0.596084801	5470.473187430548426
NONHSAG011182	0.085313461	0.257062508	0.59445705	0.046958936	0.321605637	0.65343573	5480.463277631987392
NONHSAG011270	0.051544249	0.599209401	0.847002984	0.046427993	0.353989654	0.685550212	5490.645264615485018
NONHSAG011279	-0.05150013	0.607145646	0.850163538	0.046280844	0.101591324	0.350159272	5500.085091687544062
NONHSAG011288	0.237154216	0.022262956	0.123238983	0.046278587	0.004417642	0.033215	5510.0110670342443919
NONHSAG011425	-0.10653846	0.407105168	0.717280533	0.046176449	0.072172016	0.285694163	5520.0321315335976841
NONHSAG011459	0.058404971	0.485876602	0.774497197	0.045978229	0.830473009	0.933749876	5530.772179057235677
NONHSAG011460	0.077336416	0.315245641	0.653257732	0.045886688	0.070369347	0.281878353	5540.18920268975143
NONHSAG011467	0.061383081	0.404472127	0.715272773	0.045809357	0.917730719	0.969807885	5550.658116669208672
NONHSAG011471	-0.00238403	0.967643914	0.987996581	0.045686796	0.366126586	0.69386861	5560.562485531252457
NONHSAG011496	0.124467373	0.187601654	0.516279483	0.045506062	0.569638408	0.832548442	5570.412030194806045
NONHSAG011553	0.008101222	0.86860775	0.951960393	0.045420518	0.995607784	0.997026029	5580.981042943878382
NONHSAG011555	0.005164089	0.952432622	0.981759726	0.045414779	0.794465738	0.923062508	5590.942931397464051
NONHSAG011584	-0.09506058	0.150301441	0.460902565	0.045399522	0.417442062	0.735463454	5600.348569055961886
NONHSAG011594	0.074490267	0.205060478	0.53747107	0.045341777	0.065860275	0.271064674	5610.167587240246867

NONHSAG011643	-0.01195973	0.853940315	0.949662955	0.045238547	0.401724675	0.724046292	5620.549953656349425
NONHSAG011647	-0.06745677	0.339095858	0.673579125	0.045051299	0.835946478	0.937647186	5630.598794715277044
NONHSAG011680	0.108673454	0.225416173	0.558671887	0.04500084	0.586574805	0.836463428	5640.473198103626143
NONHSAG011703	0.144124801	0.171761586	0.496021375	0.04445344	0.00909442	0.062020881	5650.0317397468202642
NONHSAG011730	0.15941581	0.052777273	0.237836044	0.044256898	0.043852572	0.203841567	5660.0744961305770866
NONHSAG011744	0.033386123	0.680321809	0.887517619	0.044012732	0.701102016	0.895983652	5670.726738180343417
NONHSAG011747	0.203244626	0.131795399	0.429940445	0.043904247	0.748060197	0.906696003	5680.151801022038207
NONHSAG011762	0.003703084	0.954873517	0.983194555	0.043889552	0.629161007	0.858449701	5690.836943879459247
NONHSAG011768	-0.01507566	0.78708646	0.924683619	0.043771287	0.612288413	0.849433959	5700.877701732634716
NONHSAG011773	0.046285825	0.400069165	0.713376342	0.043636665	0.206529985	0.516832107	5710.433089747328408
NONHSAG011781	0.704058143	2.93E-11	1.35E-09	0.04352615	6.24E-11	3.51E-09	5726.35412319019574e-12
NONHSAG011800	-0.08553856	0.297231408	0.634635322	0.043467364	0.395573501	0.718406651	5730.172819973846229
NONHSAG011802	0.830315999	2.19E-15	1.92E-13	0.043153307	4.98E-13	4.83E-11	5742.08095864025542e-15
NONHSAG011805	0.066469032	0.328602166	0.668343876	0.043002719	0.717841287	0.902031633	5750.609468240577863
NONHSAG011821	-0.03412189	0.612549516	0.852440007	0.042975111	0.855994883	0.946361193	5760.773558601049264
NONHSAG011830	-0.05258195	0.349498284	0.677785637	0.042899867	0.734167282	0.905644804	5770.418158300958187
NONHSAG011840	-0.05402291	0.37165266	0.698587753	0.042642113	0.544911424	0.815012106	5780.656251686925089
NONHSAG011877	0.967839921	2.97E-10	1.08E-08	0.042637124	9.92E-11	5.26E-09	5792.77007940073069e-11
NONHSAG011915	0.226168667	0.019333789	0.110726302	0.042241283	0.07225895	0.285694163	5800.0499413580183135
NONHSAG011924	-0.00288391	0.977029259	0.993269245	0.042182239	0.739205716	0.906043339	5810.933325666440483
NONHSAG011933	0.200489218	0.00253452	0.020363058	0.042174248	9.39E-05	0.001148295	5820.000275287684374743
NONHSAG011964	0.121495237	0.142923231	0.447555229	0.042159912	0.007091682	0.050358106	5830.0252630464709062
NONHSAG011968	0.134944544	0.058617224	0.25555292	0.042156774	0.26842363	0.599281359	5840.160979886662038
NONHSAG011977	-0.00962224	0.865117503	0.951960393	0.042100914	0.348724376	0.680716946	5850.60338244093391
NONHSAG011978	0.088927768	0.156577012	0.469898142	0.041858641	0.901330613	0.962240579	5860.288786374060962
NONHSAG012011	-0.12895005	0.07000494	0.287798088	0.041779354	0.781526726	0.917391514	5870.0791602974603169
RMST	-2.55880244	7.55E-21	4.78E-18	0.041719371	3.17E-14	4.05E-12	5884.2181223869649e-20
TMPO-AS1	-1.9980389	3.59E-20	1.28E-17	0.041257289	1.13E-15	2.26E-13	28129.71926930970724e-20
NONHSAG012031	0.0772196	0.25220892	0.587746844	0.040965354	0.535697223	0.811627473	5890.513711515740271
NONHSAG012043	-0.00412192	0.943754995	0.978915178	0.040771542	0.607148608	0.846456066	5900.813694821713328
NONHSAG012048	-0.30609324	0.001646161	0.014027286	0.040657411	0.056348323	0.239714801	5910.00635172058750489
NONHSAG012050	-0.01977549	0.777356952	0.920350018	0.040652879	0.446145922	0.756399448	5920.740104490720976
NONHSAG012055	0.397914224	2.48E-08	5.86E-07	0.040538625	1.01E-08	3.27E-07	5933.70976453007069e-09
NONHSAG012099	0.069553417	0.203692056	0.536312792	0.040473757	0.348973616	0.680716946	5940.415949458524021
NONHSAG012152	-0.08940384	0.186347216	0.515249135	0.040337856	0.493005027	0.791284324	5950.131158088887304
NONHSAG012158	-0.17650874	0.034242432	0.171945927	0.040235399	0.499890976	0.796878358	5960.092921612151269
NONHSAG012172	0.132322282	0.024586605	0.13358443	0.040155091	0.002210546	0.018500164	5970.00677548924144691
NONHSAG012182	0.119940145	0.026341949	0.141361758	0.040152241	0.202927209	0.511410961	5980.0810512422439202
NONHSAG012199	0.08572707	0.37980832	0.704499337	0.040117481	0.668806399	0.880630922	5990.676179026762781
NONHSAG012226	-0.02711834	0.634822979	0.862680714	0.040105576	0.465357527	0.774769311	6000.478865999187633
NONHSAG012236	0.06181638	0.372364491	0.698899101	0.03995531	0.237203483	0.562408258	6010.464412336314427
NONHSAG012367	0.008735621	0.918123716	0.968403559	0.039819539	0.335684872	0.671076968	6020.567930574396639
NONHSAG012392	0.11204078	0.106172788	0.380813624	0.039754897	0.210312967	0.523362888	6030.235323315263387
NONHSAG012393	0.055222264	0.553278166	0.821852872	0.039408657	0.809884207	0.930212019	6040.690150885187364
NONHSAG012430	-0.00385711	0.951513751	0.981209884	0.039384897	0.543420381	0.814551231	6050.758518653368892
NONHSAG012433	0.053483942	0.425094252	0.729325831	0.039304961	0.241125462	0.568352723	6060.48418893663039
NONHSAG012556	0.129727392	0.089781138	0.34489694	0.039239443	0.029387951	0.151076632	6070.0727710174343745
NONHSAG012565	0.032571631	0.549001364	0.820817175	0.03911531	0.914256894	0.968016258	6080.813045690757879
NONHSAG012566	0.025938709	0.751441724	0.910732246	0.039098544	0.599824991	0.840286125	6090.867996658955645
NONHSAG012599	-1.0782234	1.56E-12	8.79E-11	0.039066883	3.17E-09	1.17E-07	6103.88464412426777e-12
NONHSAG012608	0.067850905	0.330157123	0.669162012	0.038760941	0.525629001	0.807246724	6110.609570373337108
NONHSAG012629	-0.01539173	0.748564667	0.910542676	0.038714293	0.139812875	0.425031141	6120.162210422494214
NONHSAG012630	0.206745792	0.000699874	0.006671346	0.038648401	5.63E-05	0.000736624	6130.000117076961038397
NONHSAG012635	-0.055252	0.488919487	0.776248729	0.038633385	0.669503195	0.880630922	6140.781008835344948
NONHSAG012657	-1.26531742	1.69E-18	4.08E-16	0.038562804	6.19E-15	9.93E-13	6152.98423432576303e-18
NONHSAG012658	-1.26514781	3.63E-20	1.28E-17	0.038540777	1.79E-17	1.00E-14	6163.25010654730834e-20
NONHSAG012682	0.052690513	0.555614284	0.821876574	0.038209156	0.565211803	0.82952797	6170.795348722855695
NONHSAG012683	-0.13574512	0.059148868	0.257073598	0.037695939	0.206050876	0.516412712	6180.1549358095757
NONHSAG012684	0.038404456	0.603303578	0.848669165	0.037476493	0.934194601	0.973855106	6190.85372548424078
NONHSAG012695	-0.02614521	0.640458265	0.867640994	0.037455801	0.802347701	0.926952233	6200.895101062540864
NONHSAG012727	0.072840209	0.355011487	0.681428192	0.037339287	0.553003363	0.82089373	6210.640885157575377
NONHSAG012732	-0.17296027	0.06901844	0.286676296	0.037206917	0.102684917	0.351705222	6220.134876811767434
NONHSAG012739	0.038313834	0.70070628	0.895749937	0.036937645	0.447176952	0.756417348	6230.74599030381732
NONHSAG012744	-0.00976404	0.864758234	0.951960393	0.036896676	0.525169428	0.807170039	6240.694997815802369
NONHSAG012745	-0.11035732	0.303135378	0.641397052	0.036792506	0.075693159	0.294397184	6250.199842053364139
NONHSAG012747	0.092541096	0.396184192	0.711411206	0.036643358	0.298207066	0.627870613	6260.53822069201815
NONHSAG012753	0.041462182	0.503708979	0.789138617	0.036640052	0.988233761	0.996383412	6270.744332789606604
NONHSAG012762	-0.1080756	0.167730521	0.490289215	0.036512278	0.15194383	0.439574124	6280.265518147333012
NONHSAG012764	0.064624085	0.400583704	0.713841177	0.036494778	0.7016202	0.895983652	6290.698131913225321
NONHSAG012780	0.017209167	0.804692848	0.932342929	0.036443295	0.712267028	0.897756559	6300.930863242874107
NONHSAG012793	-0.323537	2.36E-06	3.82E-05	0.036415864	0.001132807	0.010376068	6311.03902925150446e-05

NONHSAG012799	0.269903951	9.61E-05	0.001130988	0.036272197	1.71E-05	0.000247805	6322.15588799633024e-05
NONHSAG012823	0.034783979	0.613984095	0.852604086	0.03610944	0.884481407	0.956890351	6330.872176068678217
NONHSAG012881	0.070201275	0.237946076	0.5711076	0.036089026	0.829789077	0.933749876	6340.323514547727561
NONHSAG012884	-0.01397348	0.86753869	0.951960393	0.035998705	0.181608897	0.485903158	6350.342647096069177
NONHSAG012909	0.177674328	0.005272472	0.038509585	0.035998012	0.528054988	0.807576213	6360.0132042713636775
NONHSAG012910	0.154415378	0.073445663	0.298884521	0.035624221	0.011915438	0.077025774	6370.0348980710572378
NONHSAG012920	-0.07086459	0.196913172	0.527031094	0.035590397	0.095549074	0.338819666	6380.214802474299101
NONHSAG012971	0.131671718	0.136449091	0.440017024	0.0354784	0.002031582	0.017236595	6390.00792786820425511
NONHSAG012980	0.068678662	0.279502711	0.614993445	0.035452094	0.899855662	0.962240579	6400.492745989890561
NONHSAG012986	0.030501381	0.614666205	0.852709112	0.035353177	0.123428765	0.395309439	6410.285722758709397
NONHSAG013018	-0.02753343	0.624282804	0.85822947	0.035338972	0.641008218	0.867427868	6420.631809297063439
NONHSAG013032	0.007763376	0.876163521	0.953958166	0.035234947	0.621490872	0.853798275	6430.792595599153582
NONHSAG013037	0.056529455	0.391705029	0.708343757	0.035205495	0.666224692	0.880630922	6440.424120585913568
NONHSAG013039	-0.01821194	0.819358087	0.938128871	0.034982401	0.261359179	0.592856869	6450.489561562956445
NONHSAG013044	-0.07936072	0.28799911	0.62446068	0.034720781	0.725507369	0.902489903	6460.551705589976958
NONHSAG013052	0.018865702	0.827792694	0.940126436	0.034624763	0.440120775	0.752824306	6470.72508861873984
NONHSAG013055	0.036512781	0.532126019	0.811903616	0.034312643	0.395006807	0.718406651	6480.6749446062418
NONHSAG013061	-0.00552173	0.935181592	0.975411819	0.03429875	0.720389419	0.902489903	6490.89527312698867
NONHSAG013087	0.047888258	0.598697929	0.847002984	0.034210934	0.012908741	0.081803217	6500.0311336599372315
NONHSAG013088	0.077404073	0.237344204	0.5711076	0.034155493	0.523248998	0.80637524	6510.491963125916097
NONHSAG013092	0.05591475	0.395957143	0.711411206	0.034073602	0.592954186	0.839418789	6520.688450212481568
NONHSAG013093	-0.15239285	0.115943653	0.402510559	0.034005482	0.737042339	0.906043339	6530.249476492935748
NONHSAG013140	-0.08440541	0.270078622	0.6070832	0.033934371	0.413447886	0.733255014	6540.515391920022568
NONHSAG013147	-0.01558712	0.824867643	0.938461188	0.033410847	0.839927753	0.940566763	6550.969988522383518
NONHSAG013148	0.018015322	0.787230648	0.924683619	0.03336962	0.596205636	0.839825518	6560.867270496743016
NONHSAG013176	-0.00992839	0.879415679	0.954268263	0.033224902	0.957597328	0.981988142	6570.977339132267637
NONHSAG013184	0.898109036	8.71E-09	2.36E-07	0.033074104	3.83E-09	1.40E-07	6581.2399629030787e-09
NONHSAG013195	0.136697865	0.111897674	0.393320325	0.033003664	0.158753504	0.451836895	6590.218338685889053
NONHSAG013196	0.03176815	0.673764483	0.885339125	0.03293832	0.09773638	0.343973343	6600.222674648551966
NONHSAG013213	0.143814177	0.012842964	0.079667507	0.032893461	7.60E-05	0.000976131	6610.000336410845246127
NONHSAG013219	-0.05287891	0.440941809	0.743808258	0.032846771	0.783010715	0.918192715	6620.554036191852817
NONHSAG013224	-0.01285709	0.830268313	0.941977934	0.032818133	0.194208135	0.503330208	6630.2625677520954
NONHSAG013270	-0.12635878	0.02720062	0.144863905	0.032499838	0.274768116	0.603336539	6640.0841927265945343
NONHSAG013289	0.115468794	0.05165841	0.233999248	0.03249659	0.125178743	0.39954895	6650.121347256030178
NONHSAG013312	-0.07204012	0.550586745	0.820922168	0.032494931	0.620132089	0.853798275	6660.549104911551426
NONHSAG013343	-0.39747215	7.75E-07	1.36E-05	0.032222128	0.00010889	0.00130854	6672.32064294896036e-06
NONHSAG013371	-0.03061378	0.774771831	0.919265143	0.032019836	0.669587849	0.880630922	6680.908555313871167
NONHSAG013387	-0.00231561	0.972792717	0.990899953	0.032017126	0.242977502	0.570085574	6690.389678955148163
NONHSAG013400	0.074701272	0.228414659	0.559166483	0.031998009	0.09960241	0.347496251	6700.231285541377553
NONHSAG013413	0.408856705	0.001181683	0.010616268	0.031988319	9.09E-06	0.00014691	6713.32940822835467e-05
NONHSAG013415	0.272228337	0.000913111	0.008363738	0.031909471	3.26E-06	5.91E-05	6721.31615579705745e-05
NONHSAG013417	0.006165589	0.921380266	0.97001921	0.031743898	0.759794739	0.909554195	6730.913859244684394
NONHSAG013418	0.073271578	0.215181142	0.548282042	0.03165374	0.174074804	0.475047467	6740.319798246568573
NONHSAG013429	0.103165576	0.067397888	0.282869943	0.03134523	0.652771558	0.874925463	6750.158643124934104
NONHSAG013433	-0.00070097	0.991295213	0.996279605	0.031196785	0.495982304	0.794249567	6760.73500716170282
NONHSAG013449	0.041177973	0.517905545	0.800192523	0.031185653	0.19556315	0.504065232	6770.427589416567617
NONHSAG013472	0.041124522	0.583135099	0.841774074	0.031096808	0.457477955	0.767100781	6780.740207459443565
NONHSAG013488	-0.13133074	0.00856008	0.058002278	0.03103253	0.052919118	0.232150639	6790.0245446767014155
NONHSAG013496	-0.08047318	0.289652623	0.626540904	0.0310152	0.482943877	0.783450231	6800.21350151646564
NONHSAG013505	0.06628849	0.328648207	0.668343876	0.030641916	0.516902033	0.804430331	6810.606281105765533
NONHSAG013509	-0.12183359	0.309478049	0.647268241	0.030384302	0.341311979	0.674686654	6820.151470610205324
NONHSAG013521	-0.10618103	0.40860321	0.718779718	0.030290075	0.803936443	0.927644349	6830.529472585495642
NONHSAG013525	0.133199642	0.031664616	0.162780438	0.030249775	0.161307212	0.45587637	6840.0909294812015937
NONHSAG013533	0.24058443	0.002203294	0.018010649	0.030218482	0.003923221	0.029961774	6850.00311825129172056
NONHSAG013546	-0.02774829	0.746510968	0.909586883	0.03012196	0.449793637	0.756923822	6860.746949018155982
NONHSAG013584	0.024943446	0.736329923	0.905538909	0.02999111	0.707402645	0.896707014	6870.917205345731826
NONHSAG013596	0.235103046	0.002326391	0.018852486	0.029871287	0.197856372	0.505333441	6880.00859690836521059
NONHSAG013598	-0.05112926	0.419315142	0.726032559	0.029591885	0.890787291	0.958708067	6890.684873707832615
NONHSAG013622	-0.05743523	0.428676739	0.73236115	0.029493775	0.311015181	0.639776657	6900.563459038335047
NONHSAG013653	-0.01869286	0.778096173	0.920490719	0.029403573	0.089623539	0.32545919	6910.104004518833855
NONHSAG013655	-0.02114656	0.736204581	0.905538909	0.029264797	0.288025227	0.617112355	6920.549779568484568
NONHSAG013658	0.04507327	0.496614176	0.783218768	0.029146788	0.394349851	0.718406651	6930.663250950333338
NONHSAG013688	-0.11594035	0.187906683	0.516279483	0.029015589	0.035656958	0.172874769	6940.102816824301654
NONHSAG013701	0.027533394	0.6129381	0.852440007	0.028979116	0.528428247	0.807576213	6950.797837277163215
NONHSAG013735	0.048475351	0.353972476	0.680362681	0.028943315	0.286578363	0.616099661	6960.507642341800029
NONHSAG013737	0.043407096	0.508822476	0.795777977	0.028747126	0.090955099	0.328768672	6970.22901616088358
NONHSAG013751	0.009890616	0.873814505	0.953128932	0.028482844	0.247200694	0.575914126	6980.450940783206643
NONHSAG013766	0.021036894	0.66196728	0.876389826	0.028355023	0.201713333	0.509172255	6990.425486299437925
NONHSAG013770	0.039685721	0.399591516	0.712991076	0.028325609	0.733686756	0.905644804	7000.476060568735423
NONHSAG013789	0.068827157	0.30672487	0.644626559	0.027926903	0.36615931	0.69386861	7010.531820944379638
NONHSAG013796	0.021862299	0.779776569	0.9209289	0.027803918	0.507224334	0.801300464	7020.798587345875275

NONHSAG013836	-0.06884758	0.426378012	0.731082299	0.02767383	0.908298281	0.965032915	7030.609022374664625
NONHSAG013843	0.033005708	0.621309475	0.857660281	0.02764069	0.085959025	0.318048391	7040.204797734578449
NONHSAG013856	0.142326565	0.045184359	0.212111605	0.027621229	0.571304618	0.833292346	7050.114938061487889
NONHSAG013867	-0.00954831	0.877592175	0.954268263	0.027610139	0.674375782	0.881776357	7060.836410954679198
NONHSAG013871	0.094035525	0.096240627	0.360018759	0.027562214	0.561841178	0.826911648	7070.0716841169248996
NONHSAG013889	-0.14620173	0.002271147	0.018457993	0.027421183	0.518777497	0.804634485	7080.00540685150951179
NONHSAG013908	-0.10086375	0.06969971	0.287383553	0.027211211	0.131402748	0.411017273	7090.150777791193282
NONHSAG013910	-0.00124939	0.982029817	0.9940489	0.027118767	0.871717722	0.950957653	7100.979776942942121
NONHSAG013921	0.021500609	0.750132533	0.910732246	0.027081382	0.366583564	0.69386861	7110.447742144801103
NONHSAG013938	-0.1978964	0.007924534	0.054218468	0.026987907	0.307522283	0.635739391	7120.0259855821457081
NONHSAG013940	0.071029303	0.335189832	0.671813121	0.026938023	0.368328576	0.695672009	7130.556191956377613
NONHSAG013950	0.062681706	0.237742696	0.5711076	0.026912311	0.532727517	0.810050899	7140.492882924567311
NONHSAG013963	0.012398652	0.862984948	0.951781505	0.026568052	0.697223376	0.895338645	7150.925794265032643
NONHSAG013971	0.050299101	0.436777422	0.74045563	0.026421528	0.070953644	0.282879495	7160.0347923818329405
NONHSAG013976	-0.06776449	0.329938017	0.669162012	0.026255077	0.066774108	0.273190941	7170.181724805522084
NONHSAG013982	0.018446587	0.769969252	0.917667832	0.026139999	0.864556926	0.948920405	7180.957178751153059
NONHSAG013992	0.05040568	0.348432692	0.677785637	0.026034671	0.710337189	0.896707014	7190.636058597232844
NONHSAG014007	0.043858052	0.590032792	0.842219396	0.025970165	0.842760445	0.941172323	7200.860141585665607
NONHSAG014012	0.079716601	0.127986223	0.42450856	0.025956012	0.18493528	0.489677973	7210.252006874973291
NONHSAG014023	-0.07220893	0.27445212	0.612084166	0.025932707	0.577705087	0.833831576	7220.248687004671843
NONHSAG014030	-0.34879297	3.81E-05	0.00049347	0.025913689	5.11E-05	0.000677771	7232.57835493580109e-05
NONHSAG014035	0.013797499	0.845783522	0.946688454	0.025855286	0.200104636	0.507425687	7240.381061445136866
NONHSAG014073	0.096247479	0.241391306	0.575614548	0.025776115	0.568317745	0.831933314	7250.498432692337906
NONHSAG014079	0.044821115	0.416483143	0.724427994	0.025718029	0.583581003	0.836050856	7260.70606567396101
NONHSAG014090	0.115981201	0.090766978	0.34773398	0.025646647	0.511610924	0.803715039	7270.228347967871791
NONHSAG014099	-0.06291232	0.428946946	0.73236115	0.025587565	0.859448485	0.947213452	7280.705054728816888
NONHSAG014114	-0.02436095	0.715511745	0.898703486	0.025454333	0.48978195	0.788812626	7290.784944370782925
NONHSAG014130	-0.09479273	0.177844169	0.502625892	0.025262288	0.281241905	0.612446652	7300.35874269057615
NONHSAG014136	0.062278748	0.33120661	0.669461779	0.025144655	0.504366607	0.799931696	7310.261542566678889
NONHSAG014165	-0.05988136	0.495698604	0.78221351	0.025109171	0.684912404	0.889213579	7320.546173512360866
NONHSAG014169	0.100786018	0.053057963	0.238718389	0.025047513	0.622131104	0.853798275	7330.128884591949309
NONHSAG014185	-0.02715582	0.650331346	0.872061952	0.025024415	0.381606707	0.708236919	7340.402956420180155
NONHSAG014197	-0.03698177	0.631072264	0.861444275	0.025010342	0.587633994	0.836463428	7350.836536984133983
NONHSAG014198	-0.03109636	0.736431679	0.905538909	0.024848755	0.800325164	0.925568407	7360.837963268857453
NONHSAG014202	-0.1169709	0.107403642	0.384248145	0.024636073	0.65665122	0.877615604	7370.101114111214912
NONHSAG014208	-0.05869606	0.515110052	0.799108873	0.024509495	0.099746678	0.347568349	7380.247628088498807
NONHSAG014212	0.049968127	0.413789026	0.724066422	0.024322515	0.537797461	0.812140251	7390.692677924574323
NONHSAG014215	-0.01878	0.678669987	0.887517619	0.02424177	0.569494268	0.832548442	7400.613125449377343
NONHSAG014232	0.195355299	0.030693931	0.158952733	0.024188999	0.003380428	0.026404896	7410.0101134332550373
NONHSAG014249	0.107502867	0.166427333	0.489021589	0.024092801	0.061363432	0.256776742	7420.150348954909463
NONHSAG014269	-0.0541552	0.336930156	0.67346619	0.024090574	0.342154208	0.675184303	7430.541068586635543
NONHSAG014294	0.049312512	0.472751809	0.764982846	0.024057069	0.767567755	0.913028987	7440.767788873121786
NONHSAG014358	0.565432855	3.53E-07	6.49E-06	0.023990946	7.04E-08	1.85E-06	7454.43266362454648e-08
NONHSAG014410	-0.1135957	0.065510897	0.276601565	0.02387826	0.466249773	0.775308891	7460.174155515125818
NONHSAG014436	0.056720501	0.211890743	0.544533663	0.023811026	0.741175844	0.906043339	7470.427961087606042
NONHSAG014438	0.037190293	0.628950046	0.858964317	0.023677931	0.575236838	0.833526329	7480.578720365326369
NONHSAG014440	-0.00963253	0.857716552	0.950687167	0.023641221	0.630157721	0.858944988	7490.789823755386346
NONHSAG014443	-0.01477235	0.833025253	0.942580607	0.023380282	0.666273211	0.880630922	7500.909967501734615
NONHSAG014445	-0.05979487	0.332141294	0.669520659	0.02335294	0.72863299	0.904080089	7510.612585591614213
NONHSAG014448	-0.20007617	0.262244286	0.6018673	0.023342832	0.07334011	0.288840881	7520.191173896901271
NONHSAG014454	0.069243359	0.251925923	0.587746844	0.02329712	0.013827218	0.086022425	7530.0458474603450576
NONHSAG014569	-0.04344039	0.597602903	0.847002984	0.023064967	0.898480694	0.962120226	7540.783398069586516
NONHSAG014574	0.028230861	0.62685235	0.85822947	0.022835826	0.484623306	0.78447362	7550.491917489896835
NONHSAG014590	0.112418996	0.142331168	0.447555229	0.022582075	0.272656057	0.602285022	7560.308985932390473
NONHSAG014611	0.001407306	0.981412647	0.9940489	0.022563311	0.383400471	0.709868831	7570.60723000724105
NONHSAG014661	0.146661324	0.061098146	0.263105646	0.022384951	0.003406688	0.026536307	7580.0120708053664295
NONHSAG014663	0.904870327	1.56E-08	3.94E-07	0.02236131	4.29E-10	1.98E-08	7594.07889378633886e-10
NONHSAG014667	0.140021621	0.1465975	0.454500739	0.022342006	0.443791566	0.755412762	7600.343235435296134
NONHSAG014746	0.069447356	0.210545761	0.544124897	0.022227405	0.163055665	0.457141107	7610.305637464051263
NONHSAG014747	-0.09254548	0.103321003	0.376345414	0.022224634	0.198053301	0.505377389	7620.226538266526455
NONHSAG014790	-0.03148846	0.709366174	0.898275053	0.022136384	0.713379187	0.897950883	7630.911616146510053
NONHSAG014806	-0.03044345	0.72870665	0.903322681	0.021670766	0.247744577	0.576651854	7640.489752592980921
NONHSAG014811	-0.0504912	0.386142894	0.706496807	0.021552128	0.98343577	0.993683573	7650.609906960761866
NONHSAG014813	0.001979435	0.977374112	0.993269245	0.021503037	0.446523145	0.756399448	7660.685952941873435
NONHSAG014829	-0.04111173	0.529490579	0.809639755	0.021368467	0.47265494	0.778445171	7670.733349816663003
NONHSAG014834	0.090758002	0.194661493	0.526251063	0.021310714	0.13962811	0.424928836	7680.27082186716415
NONHSAG014845	-0.03842452	0.626691045	0.85822947	0.021264947	0.533316495	0.810203124	7690.804728054275577
NONHSAG014849	-0.00190385	0.978676359	0.993501254	0.021094822	0.854684238	0.945832264	7700.980341915765027
NONHSAG014850	0.065154914	0.409902983	0.720098185	0.021062985	0.282213558	0.613093982	7710.527369810844408
NONHSAG014898	0.117243452	0.157354154	0.471128832	0.020900238	0.08751338	0.320009914	7720.187161500782105
NONHSAG014905	-0.02543183	0.75924616	0.91407719	0.020690247	0.636625955	0.863159203	7730.890038364148486

NONHSAG014910	0.191473605	0.02021216	0.114359345	0.020679937	0.527782187	0.807576213	7740.0536687235316164
NONHSAG014924	-1.0320384	1.10E-17	1.87E-15	0.020508552	4.49E-16	1.26E-13	7754.61194477077139e-18
NONHSAG014926	0.225221531	0.06016644	0.260289275	0.020485928	0.158729712	0.451836895	7760.146116015446056
NONHSAG014964	-0.00831923	0.869081617	0.951960393	0.020402922	0.810776131	0.930572441	7770.919727857630761
NONHSAG015027	-0.09983731	0.292457973	0.630465061	0.020130911	0.698792144	0.895443732	7780.562427652192836
NONHSAG015035	-0.02644423	0.727337649	0.903322681	0.020122053	0.508078061	0.802059678	7790.589158973836482
NONHSAG015052	-0.07363393	0.279721769	0.614994225	0.020095695	0.643127069	0.868581319	7800.550534908032325
NONHSAG015063	0.119571814	0.045219667	0.212111605	0.020010559	0.075549053	0.294397184	7810.0904319870030909
NONHSAG015092	-0.00996635	0.874488645	0.953287334	0.019553773	0.802975479	0.927040029	7820.918080498484516
NONHSAG015143	0.138945432	0.113507627	0.39613292	0.019441547	1.77E-06	3.51E-05	7835.62660822909972e-06
NONHSAG015144	-0.07225093	0.391537958	0.708343757	0.019367548	0.387956599	0.712097883	7840.60745356554065
NONHSAG015185	-0.02775119	0.57764319	0.83866595	0.019274554	0.941388297	0.976126639	7850.78634285283684
NONHSAG015188	-0.01452336	0.860035703	0.95118497	0.019247464	0.278386636	0.60825425	7860.392881971079842
NONHSAG015211	-0.08880083	0.208112704	0.541863818	0.019112062	0.026802631	0.141990325	7870.082433403318384
NONHSAG015231	0.123313441	0.147694605	0.456894641	0.019020207	0.013421598	0.084237891	7880.0442767606599404
NONHSAG015256	-0.10737356	0.195081279	0.526457347	0.018954776	0.407085985	0.728197067	7890.108130845773489
NONHSAG015273	-0.09502077	0.179333921	0.504113961	0.018950053	0.539206738	0.812140251	7900.399670871344405
NONHSAG015286	0.072923842	0.403265572	0.714566857	0.018892636	0.055448402	0.238776273	7910.153682045346367
NONHSAG015320	-0.04126927	0.473081497	0.764982846	0.0188056	0.352644098	0.684429948	7920.618695354789528
NONHSAG015338	0.053432321	0.428158173	0.73236115	0.01874414	0.279070143	0.609274256	7930.52991479701802
NONHSAG015357	0.018538171	0.767964437	0.916213829	0.018703596	0.179861663	0.484025789	7940.365419172932465
NONHSAG015371	-0.02781561	0.725665362	0.903322681	0.018543832	0.318261299	0.650416776	7950.594388966768022
NONHSAG015373	0.049801515	0.472957142	0.764982846	0.018315538	0.633458761	0.860940568	7960.763066762699608
NONHSAG015387	0.104237672	0.28818442	0.62446068	0.01830214	0.744272649	0.906696003	7970.548447559856308
NONHSAG015435	0.114257084	0.027655529	0.146730844	0.018028889	0.432478433	0.747927033	7980.0795121242488168
NONHSAG015438	0.23890209	0.002746375	0.021815836	0.017961516	0.029639926	0.151816891	7990.00850196875663342
NONHSAG015450	-0.40701052	4.06E-06	6.37E-05	0.017919438	0.000178283	0.002054633	8009.90514547909344e-06
NONHSAG015466	-0.11583787	0.151330397	0.461203041	0.017886737	0.190764957	0.497257471	8010.28103095880517
NONHSAG015491	-0.12772949	0.137035113	0.4403917	0.017875921	0.11372479	0.373155319	8020.206154836396279
NONHSAG015511	-0.0074926	0.918830417	0.968611494	0.017817784	0.786105092	0.920026547	8030.92734857329836
NONHSAG015535	0.008239464	0.856726336	0.950687167	0.017789873	0.211820176	0.525081886	8040.297691795587348
NONHSAG015539	0.274649869	0.040003391	0.195294333	0.017498606	0.398767248	0.721114792	8050.114708922717977
NONHSAG015555	-0.05921927	0.541624708	0.817035827	0.017370751	0.915651674	0.968338663	8060.739274982395087
NONHSAG015654	0.024078997	0.682699964	0.887517619	0.017366816	0.153234449	0.441943868	8070.333618864656711
NONHSAG015660	-0.15658719	0.039816581	0.194720394	0.017308659	0.027203164	0.143565953	8080.0486162633031229
NONHSAG015664	0.057324097	0.416113081	0.724427994	0.017276417	0.492014412	0.79014536	8090.327096260680183
NONHSAG015694	0.045044599	0.51526262	0.799108873	0.017259963	0.566723597	0.830446459	8100.774355799043649
NONHSAG015695	-0.08742313	0.347411567	0.677785637	0.017222841	0.271811175	0.601363513	8110.490580458627111
NONHSAG015708	-0.03096857	0.611590416	0.852226091	0.017032193	0.583465059	0.836050856	8120.571623357134038
NONHSAG015712	0.847139048	1.21E-07	2.54E-06	0.016930239	2.26E-07	5.26E-06	8134.90971926260809e-08
NONHSAG015718	-0.08146987	0.149400815	0.459389684	0.016916374	0.076391606	0.296287269	8140.168135596265448
NONHSAG015732	0.97933849	2.42E-17	2.97E-15	0.016915538	6.18E-17	2.48E-14	8153.05932582837093e-18
NONHSAG015756	0.027021601	0.67325484	0.885083034	0.0168749	0.875229783	0.952087485	8160.911968144738769
NONHSAG015757	0.115282944	0.236791918	0.571062498	0.016847364	0.721330382	0.902489903	8170.473629522432553
NONHSAG015797	0.037830872	0.647922145	0.870666685	0.016796923	0.041946268	0.19724566	8180.0996354337087503
NONHSAG015798	-0.30904395	0.000370213	0.003771882	0.016673048	0.07374825	0.289233024	8190.00160616679707175
NONHSAG015800	0.035466914	0.522484854	0.803843214	0.01656405	0.195713063	0.504065232	8200.427795228068561
NONHSAG015828	-0.05720475	0.340782263	0.673895726	0.016344252	0.390030946	0.713031652	8210.199714998993473
NONHSAG015833	0.012233369	0.855101921	0.949662955	0.016343396	0.089676767	0.32545919	8220.173919123776466
NONHSAG015852	0.237737488	0.03009168	0.156990362	0.016307621	0.093874004	0.336361438	8230.0751742071251574
NONHSAG015872	-0.00700033	0.916629746	0.968261389	0.016304563	0.007444178	0.05233257	8240.00782550140301928
NONHSAG015874	0.069360686	0.382145053	0.706496807	0.016247722	0.021526604	0.120823974	8250.0647655141885227
NONHSAG015923	0.069765059	0.131545946	0.429712658	0.016079252	0.758152538	0.908748907	8260.276409463552336
NONHSAG015927	-0.04208552	0.382454213	0.706496807	0.015960485	0.242938465	0.570085574	8270.474480783103534
NONHSAG015948	0.092761721	0.256991113	0.59445705	0.015898654	0.403284781	0.724100325	8280.497406430772354
NONHSAG015952	-0.04052744	0.555504205	0.821876574	0.015861473	0.153936848	0.442607788	8290.35271848426037
NONHSAG015966	0.086884166	0.196146251	0.527031094	0.015694271	0.083774412	0.314098197	8300.196342134725063
NONHSAG015980	-0.00398884	0.956968284	0.983730538	0.015653541	0.386545813	0.711365723	8310.583944768484763
NONHSAG015981	-0.13207471	0.093418705	0.354512009	0.015617138	0.07568793	0.294397184	8320.136131661913032
NONHSAG016009	-0.02429939	0.792436721	0.927698609	0.015540168	0.623838531	0.855234958	8330.744496073239072
NONHSAG016021	0.058398777	0.483281473	0.773030434	0.015428089	0.319834835	0.651852579	8340.589292404854992
NONHSAG016024	-0.07332883	0.393100979	0.71041128	0.015317386	0.419482529	0.737671981	8350.627398182343306
NONHSAG016025	-0.27440055	0.002836465	0.022467999	0.014997155	0.188293663	0.494380747	8360.0105348823993725
NONHSAG016027	-0.60165338	5.05E-12	2.68E-10	0.014886243	4.61E-11	2.76E-09	8371.96679751977362e-12
NONHSAG016062	0.073453399	0.253512387	0.588523431	0.014731052	0.549853355	0.819205327	8380.516029349202094
NONHSAG016074	0.207325132	0.08827947	0.340057357	0.014693233	0.142128244	0.428365084	8390.180638605270394
NONHSAG016107	-0.08558279	0.265188135	0.603793395	0.014521038	0.162660651	0.456747487	8400.333214815370971
NONHSAG016109	0.054645047	0.265823693	0.603793395	0.014509708	0.485774724	0.785056623	8410.527091302947993
NONHSAG016111	-0.01112471	0.895982368	0.961169104	0.014472347	0.768595513	0.913092768	8420.956874067207441
NONHSAG016116	-0.01035407	0.906335808	0.964289176	0.014296889	0.887456327	0.958343776	8430.966434491343608
NONHSAG016136	0.089157681	0.449735181	0.752016606	0.01422663	0.167616249	0.464829283	8440.379872388070523

NONHSAG016155	-0.03443733	0.744951389	0.909586883	0.014203438	0.64340501	0.868581319	8450.891725874997318
NONHSAG016158	-0.21816291	0.028495546	0.150336726	0.014197161	0.008201964	0.057088919	8460.018992311054247
NONHSAG016166	0.107376449	0.277409633	0.612912715	0.014161577	0.625986201	0.856580631	8470.548472655489768
NONHSAG016202	0.095175858	0.263562251	0.602748092	0.014130996	0.535575134	0.811627473	8480.529384172760296
NONHSAG016213	-0.01941035	0.686948707	0.887517619	0.014093059	0.330986212	0.665284652	8490.369292085157879
NONHSAG016262	0.015416519	0.811796945	0.93441384	0.014023603	0.312512963	0.641918518	8500.414846857260733
NONHSAG016297	0.016350068	0.793481337	0.927815057	0.013996268	0.285786931	0.615425223	8510.533832721915725
NONHSAG016313	0.001315767	0.980026131	0.993501254	0.01365476	0.239710878	0.566919251	8520.402549454455018
NONHSAG016316	-0.12839823	0.022307483	0.123238983	0.013048388	0.030203566	0.153307632	8530.0374568781814369
NONHSAG016330	-0.12985385	0.062479186	0.266603142	0.01299689	0.147918368	0.432826692	8540.146520934249086
NONHSAG016336	-0.01136256	0.843877028	0.94647636	0.012992963	0.089698034	0.32545919	8550.116448050345006
NONHSAG016353	-0.00817012	0.919060959	0.968611494	0.012768349	0.907645113	0.965032915	8560.991921461121151
NONHSAG016383	-0.03729575	0.685318852	0.887517619	0.012711714	0.217750333	0.535239454	8570.236369332832587
NONHSAG016385	0.115246834	0.140112394	0.444189463	0.01266388	0.14333985	0.430061982	8580.235651378153114
NONHSAG016388	0.09030262	0.346590267	0.677284107	0.012660817	0.039008128	0.18718576	8590.114324682308919
NONHSAG016418	-2.30285909	5.77E-20	1.80E-17	0.012617739	8.78E-16	2.19E-13	28111.34546697140877e-19
NONHSAG016419	-1.69406929	2.37E-17	2.97E-15	0.012510344	2.72E-14	3.64E-12	8603.24899516333204e-17
NONHSAG016427	0.016437265	0.824898786	0.938461188	0.012469302	0.376799101	0.704025962	8610.503210965333722
NONHSAG016434	0.045866829	0.542508904	0.817035827	0.012297407	0.747091819	0.906696003	8620.828354184405242
NONHSAG016460	0.000788898	0.990563026	0.996279605	0.01210765	0.270295976	0.600197292	8630.43796797339606
NONHSAG016472	0.015567691	0.821101439	0.938461188	0.01204036	0.305592085	0.635125604	8640.555777436330631
NONHSAG016517	0.083385918	0.344603451	0.677284107	0.012006737	0.688057257	0.890389787	8650.63344579962508
NONHSAG016520	-0.08363366	0.101589513	0.370999625	0.011760655	0.383460439	0.709868831	8660.256561653600571
NONHSAG016540	-0.11507584	0.210891198	0.544124897	0.011743416	0.730529667	0.904159077	8670.246434196040124
NONHSAG016542	-0.04011905	0.420693687	0.726032559	0.011724205	0.859044224	0.947213452	8680.695617367292976
NONHSAG016544	-0.00502985	0.939910661	0.977659054	0.011703462	0.087709393	0.320310147	8690.131596839651045
NONHSAG016555	0.333443903	2.85E-05	0.000378382	0.011544706	9.21E-06	0.000148041	8708.02672454101126e-06
NONHSAG016582	0.093147565	0.269020971	0.605799463	0.011425959	0.452184919	0.759584225	8710.524667702441467
NONHSAG016589	-0.33157685	7.70E-08	1.68E-06	0.011094383	4.03E-08	1.09E-06	8721.48018894918302e-08
NONHSAG016595	-0.03671266	0.706683736	0.898275053	0.01108852	0.929401431	0.973855106	8730.883908463546677
NONHSAG016623	-0.09007252	0.216498561	0.549316015	0.01087442	0.22548208	0.547198355	8740.36560862426703
NONHSAG016650	-0.12225137	0.169099366	0.492243704	0.010689967	0.709195412	0.896707014	8750.18683011054329
NONHSAG016655	0.018607659	0.752574939	0.910732246	0.010683178	0.683734225	0.889213579	8760.767365220252881
NONHSAG016687	0.03746076	0.605197265	0.848785392	0.010574747	0.204058107	0.512331603	8770.436889040000086
NONHSAG016786	0.230378341	0.004257079	0.031911732	0.010520982	0.000557676	0.005560938	8780.00127836016014936
NONHSAG016826	0.00050931	0.994134603	0.99661551	0.01015888	0.436393264	0.750543033	8790.667196600458833
NONHSAG016852	0.615559119	8.67E-07	1.51E-05	0.010053136	8.29E-06	0.000135609	8809.67311819166884e-07
NONHSAG016854	-0.06258651	0.277903169	0.612912715	0.010052875	0.683397094	0.889213579	8810.543530344064848
NONHSAG016887	0.071194386	0.497541812	0.783802563	0.009843009	0.083888632	0.314107636	8820.214238490825664
NONHSAG016890	-0.19098655	0.016891981	0.099581239	0.009727536	0.139154173	0.424865945	8830.0532888919188087
NONHSAG016921	0.317170344	0.000174174	0.001959108	0.009556945	0.001272394	0.011431217	8840.000336442272117296
NONHSAG016956	0.077925686	0.262733026	0.6018673	0.009500769	0.118573312	0.384412149	8850.270807107261709
NONHSAG016984	0.042323856	0.433158676	0.737760264	0.009425914	0.461130994	0.77138629	8860.676360280813037
NONHSAG016991	-0.24464237	0.000591895	0.00577981	0.009327525	0.029120756	0.150253976	8870.00234913839141117
NONHSAG017020	-0.07367092	0.182496558	0.509107461	0.009158041	0.980919464	0.992468062	8880.309856024514434
NONHSAG017021	0.042104629	0.543952812	0.817395852	0.009141112	0.516294574	0.804430331	8890.455231021254818
NONHSAG017030	0.076459953	0.403277953	0.714566857	0.008808954	0.196056445	0.504065232	8900.41899364006533
NONHSAG017048	-0.18519659	0.111297393	0.392190813	0.008773986	0.218625648	0.536921678	8910.245802057556373
NONHSAG017077	0.041605128	0.683825933	0.887517619	0.008666608	0.958350502	0.982099713	8920.879680909007792
NONHSAG017083	0.063042006	0.419787826	0.726032559	0.008653238	0.038973056	0.18718576	8930.110820138117101
NONHSAG017088	-0.1081422	0.120781438	0.411133305	0.008470159	0.619421491	0.853798275	8940.106928524377512
NONHSAG017091	-0.10752088	0.103583174	0.37672431	0.008067869	0.86002934	0.947278693	8950.139958265149939
NONHSAG017106	-0.00650565	0.943154174	0.978915178	0.008058601	0.769504788	0.91321261	8960.927234545588468
NONHSAG017112	-0.04473254	0.60606764	0.849582355	0.007824372	0.584827367	0.836463428	8970.826211582771566
NONHSAG017118	-0.05594358	0.212383417	0.544533663	0.007795495	0.387352324	0.711918127	8980.112409183703315
NONHSAG017164	0.203003924	0.012823512	0.079667507	0.007405345	0.004084561	0.030998357	8990.00831255032974382
NONHSAG017207	0.164568502	0.309593096	0.647268241	0.007368545	0.039856285	0.19028162	9000.117331961993814
NONHSAG017233	0.078251487	0.288756452	0.62508325	0.007171976	0.098064236	0.344265457	9010.241789465276691
NONHSAG017237	0.929055231	2.12E-07	4.16E-06	0.007059662	3.37E-08	9.37E-07	9022.19401249822435e-08
NONHSAG017254	-0.00291834	0.972928439	0.990899953	0.007028116	0.580687217	0.836050856	9030.803039988660395
NONHSAG017262	-0.01981742	0.767947912	0.916213829	0.007025411	0.906143428	0.964449402	9040.91242283974165
NONHSAG017302	-0.01301189	0.846427503	0.946759801	0.00690971	0.691255692	0.892769145	9050.832426090465387
NONHSAG017304	-0.06184947	0.370547411	0.698354362	0.006893743	0.730469327	0.904159077	9060.660995003803162
NONHSAG017314	0.066970691	0.414097948	0.724156361	0.006698057	0.231058595	0.555330572	9070.468817033306647
NONHSAG017331	0.353090227	5.17E-07	9.32E-06	0.006694964	4.31E-05	0.00057931	9081.26496352977041e-06
NONHSAG017346	0.423951274	7.11E-07	1.27E-05	0.006502692	1.51E-08	4.51E-07	9092.14390085589029e-08
NONHSAG017352	0.079687276	0.318109829	0.656624929	0.006484481	0.258194468	0.589799224	9100.462447691820083
NONHSAG017356	0.216170597	0.001322079	0.011654188	0.006465621	0.000321757	0.003414264	9110.000522410186123359
NONHSAG017360	0.034429021	0.633406765	0.862680714	0.006383363	0.424915189	0.741866512	9120.721208651510282
NONHSAG017376	0.188017066	0.032980557	0.167101031	0.00624378	0.053569441	0.233546153	9130.0632784817188894
NONHSAG017384	0.003886825	0.955876713	0.983730538	0.006143915	0.540879402	0.812737703	9140.793097255859793

NONHSAG017387	0.050175831	0.544155418	0.817395852	0.006127601	0.512315014	0.80404898	9150.763495120093191
NONHSAG017486	0.104190207	0.304845347	0.643123295	0.006096201	0.900366754	0.962240579	9160.448241877019299
NONHSAG017508	-0.07306495	0.154625603	0.466921698	0.006055971	0.356195278	0.686695518	9170.348352965519188
NONHSAG017514	0.02801932	0.61279865	0.852440007	0.005964974	0.708086319	0.896707014	9180.86955954948688
NONHSAG017529	0.0283584	0.709755953	0.898275053	0.005917163	0.304711492	0.634657974	9190.57812415758385
NONHSAG017532	0.022040479	0.765865251	0.915719092	0.005668087	0.981173973	0.992468062	9200.937017237683389
NONHSAG017549	0.005141618	0.926857413	0.973226917	0.0055305	0.857112613	0.946661692	9210.983690756712018
NONHSAG017579	0.062100857	0.198864498	0.528551011	0.005490607	0.08364571	0.314034362	9220.196960417601968
NONHSAG017593	0.012535861	0.834409151	0.94268326	0.005328984	0.029966877	0.152724793	9230.0564002983270788
NONHSAG017594	-0.04582151	0.430413801	0.734419665	0.005195769	0.321151222	0.65343573	9240.573906482458425
NONHSAG017604	0.074266861	0.332693853	0.669779847	0.005159893	0.299822961	0.629081811	9250.50707471228893
NONHSAG017613	0.057115901	0.210591991	0.544124897	0.005140173	0.153610622	0.442263776	9260.295535339767357
NONHSAG017640	-0.04505464	0.461773281	0.760209241	0.005131837	0.664684601	0.880630922	9270.496960023616283
NONHSAG017669	0.011490032	0.808481406	0.93439361	0.005109897	0.869348714	0.950234892	9280.918661733474403
NONHSAG017670	0.028662477	0.694170311	0.89238585	0.005023308	0.968947921	0.987561273	9290.909202646284025
NONHSAG017671	0.03911851	0.55314541	0.821852872	0.004677064	0.263580189	0.594853525	9300.530216739779588
NONHSAG017675	0.34315254	1.95E-05	0.000265784	0.004532422	0.020472866	0.116537854	9319.65201261570223e-05
NONHSAG017708	-0.08683703	0.479478087	0.76981996	0.004437634	0.576644559	0.833674033	9320.754640932773601
NONHSAG017741	0.071927064	0.293036214	0.630465061	0.004153242	0.281647502	0.612523416	9330.466810424148983
NONHSAG017742	0.082014763	0.218014011	0.55031903	0.004064775	0.924488268	0.971770056	9340.387128109522844
NONHSAG017775	0.069788304	0.383464872	0.706496807	0.00404815	0.858892303	0.947213452	9350.53022258017224
NONHSAG017777	-0.09578956	0.150857221	0.461203041	0.004040124	0.967515172	0.987174406	9360.260331055146203
NONHSAG017785	0.146843136	0.037308176	0.184652987	0.004039833	0.017691177	0.104511744	9370.0359312629397204
NONHSAG017797	0.32771664	3.43E-05	0.000445886	0.003914777	1.06E-05	0.000168965	9389.62743908723943e-06
NONHSAG017837	0.075178678	0.216146972	0.549316015	0.003908358	0.632211756	0.860493446	9390.45403587174541
NONHSAG017855	0.090132141	0.353765055	0.680362681	0.003904592	0.554669492	0.821343134	9400.639844892359463
NONHSAG017868	0.271211603	0.001534026	0.01311149	0.003713491	0.042671146	0.199985437	9410.00572585755935882
NONHSAG017869	0.065097326	0.373563462	0.698899101	0.003674967	0.955711471	0.981541511	9420.566666826400784
NONHSAG017885	0.057701741	0.476448854	0.768219138	0.003669407	0.668990482	0.880630922	9430.770697350026821
NONHSAG017934	0.072899964	0.385375883	0.706496807	0.003666176	0.653929954	0.876060519	9440.68233191430672
NONHSAG017937	-0.10153749	0.285508426	0.622846932	0.003616941	0.629939642	0.858944988	9450.559598562807938
NONHSAG017938	0.256572234	0.004696651	0.034685214	0.003512353	0.003545532	0.027465663	9460.00459050664998849
NONHSAG017946	0.029665255	0.667421857	0.88029562	0.003490537	0.706718219	0.896707014	9470.894778406049708
NONHSAG017952	0.683639981	7.49E-07	1.32E-05	0.002928276	1.25E-05	0.000189424	9481.04618302690206e-06
NONHSAG017967	-0.01590921	0.818947717	0.938128871	0.002753342	0.436222264	0.750543033	9490.722618419854643
NONHSAG017971	-0.02272229	0.695051238	0.89238585	0.002679039	0.797280511	0.92413553	9500.805994565839734
NONHSAG017972	-0.02359264	0.699979999	0.895515813	0.002675382	0.598510077	0.839825518	9510.656130191937469
NONHSAG017996	0.062065436	0.399378082	0.712991076	0.00259416	0.461710246	0.771493351	9520.65314506300952
NONHSAG018000	0.068010707	0.37079695	0.698354362	0.002589634	0.786158855	0.920026547	9530.473834311428194
NONHSAG018018	0.175231386	0.035911874	0.178733079	0.002561548	0.112189356	0.369410385	9540.0906047185173771
NONHSAG018031	-0.02025972	0.800896415	0.931013112	0.002524064	0.823214346	0.933749876	9550.89174082412311
NONHSAG018033	-0.04521964	0.587375948	0.842139475	0.002486351	0.148474601	0.434002679	9560.339014552987931
NONHSAG018039	0.047242472	0.467974699	0.764261292	0.002314622	0.028594064	0.148625706	9570.0799656692115177
NONHSAG018040	0.00373296	0.94413344	0.978946619	0.002220199	0.273322804	0.602810765	9580.467697530869766
NONHSAG018059	-0.14123209	0.022279169	0.123238983	0.002172274	0.013517708	0.084470657	9590.0236123521814292
NONHSAG018075	0.145038474	0.040195582	0.195553595	0.002133421	0.022425148	0.124377742	9600.0433282586050817
NONHSAG018098	0.085296893	0.444131744	0.747241857	0.002086541	0.968109052	0.987422072	9610.659527003251666
NONHSAG018192	0.089971565	0.170328852	0.493623942	0.00174541	0.066671871	0.273190941	9620.160498652893278
NONHSAG018224	0.074298244	0.248438162	0.584125534	0.00134189	0.703407997	0.896707014	9630.495611431348171
NONHSAG018230	0.065689853	0.217499928	0.549514643	0.001304787	0.828364584	0.933749876	9640.294531732305461
NONHSAG018271	-0.09692933	0.162163684	0.478531751	0.001249305	0.944537903	0.976126639	9650.253096525408352
NONHSAG018315	0.479606108	2.46E-11	1.15E-09	0.001223336	5.52E-10	2.43E-08	9661.56774399698783e-11
NONHSAG018332	-0.0297352	0.761840796	0.915035797	0.001203066	0.154661383	0.444236783	9670.185369546897228
NONHSAG018336	-0.05196559	0.489158161	0.776248729	0.001126666	0.753548638	0.907097076	9680.58754370702599
NONHSAG018350	-0.01325189	0.881976267	0.954625582	0.001080449	0.907809875	0.965032915	9690.965052497293553
NONHSAG018377	0.12022576	0.040107773	0.195464574	0.000863883	0.009250401	0.062679827	9700.0236607874826516
NONHSAG018379	0.470054862	2.00E-09	6.12E-08	0.000782742	2.75E-08	7.98E-07	9711.43769283335213e-09
NONHSAG018419	-0.37125232	0.000120754	0.001385962	0.000776016	0.012019381	0.077342105	9720.000502665548068815
NONHSAG018426	-0.21699068	0.030402744	0.158319475	0.000602432	0.062307203	0.259088491	9730.0643896812391767
NONHSAG018457	0.05980722	0.275921958	0.612684559	0.000588845	0.989863283	0.996597046	9740.454174870640507
NONHSAG018506	0.017403939	0.773285476	0.918276503	0.000569138	0.50089977	0.797130817	9750.613059993755507
NONHSAG018536	-0.58720439	5.35E-08	1.19E-06	0.000462876	2.12E-06	4.06E-05	9768.02037137697591e-08
NONHSAG018538	-0.16629997	0.013308609	0.082250128	0.00039964	0.01604134	0.09659154	9770.0196885254751858
NONHSAG018575	0.044901894	0.60250464	0.848669165	0.00036248	0.499119503	0.796099854	9780.775726946994442
NONHSAG018592	0.059862763	0.47110378	0.764534973	0.00028234	0.05020758	0.223890543	9790.136280487001206
NONHSAG018593	-0.10772196	0.198234699	0.528551011	0.000269528	0.996976992	0.998041759	9800.328319734789217
NONHSAG018602	0.018152504	0.849838402	0.947934194	0.000142149	0.966381111	0.986709541	9810.969647390179307
NONHSAG018627	-0.36620169	8.55E-06	0.000122099	0.000115306	0.000101086	0.001229223	9821.37473822943928e-05
NONHSAG018649	0.304290947	0.000198335	0.002187134	-7.71E-05	0.003743599	0.028762295	9830.000560988578117275
NONHSAG018664	-0.04420302	0.725307727	0.903322681	-0.00031345	0.049889798	0.223391898	9840.0480832268286027
NONHSAG018700	0.177709519	0.01382029	0.084668093	-0.00038489	0.114379539	0.374866274	9850.0436460645490218

NONHSAG018743	-0.08678536	0.162140331	0.478531751	-0.00038915	0.253436336	0.58512763	9860.327413176868359
NONHSAG018775	0.099999092	0.108260236	0.385433057	-0.00043286	0.444613991	0.755880336	9870.26990088153015
NONHSAG018846	0.064132094	0.384504678	0.706496807	-0.00058549	0.000547394	0.005517099	9880.00144156156812696
NONHSAG018860	0.058413413	0.299075412	0.637121257	-0.00059773	0.069351155	0.279792606	9890.186196580607452
NONHSAG018901	0.165982279	0.074212952	0.300948932	-0.00069145	0.148823376	0.434570439	9900.164750989825918
NONHSAG018925	-0.00535127	0.936852718	0.976076267	-0.00089641	0.873338927	0.951134416	9910.970489835688952
NONHSAG018928	-0.16117155	0.043752291	0.20817503	-0.0009115	0.872364844	0.950957653	9920.0546959562643632
NONHSAG018930	-0.01370558	0.891116314	0.958614796	-0.00102134	0.441894896	0.753473247	9930.710946593572475
NONHSAG018934	0.682143873	7.46E-12	3.75E-10	-0.00102864	1.74E-10	8.60E-09	9944.47730428881826e-12
NONHSAG018936	-0.00141864	0.983781386	0.995038946	-0.00121781	0.512395367	0.80404898	9950.741725149355226
NONHSAG019021	0.057011736	0.373147699	0.698899101	-0.00176333	0.352067531	0.684429948	9960.571603997082396
NONHSAG019026	0.047513603	0.607154894	0.850163538	-0.00184305	0.502028112	0.798023206	9970.778975073597734
NONHSAG019061	0.026713574	0.685570282	0.887517619	-0.0019015	0.619404778	0.853798275	9980.867993668552709
NONHSAG019100	0.141808084	0.099709974	0.365559903	-0.00196549	0.172741105	0.473438584	9990.210676658502921
NONHSAG019129	0.598833961	2.00E-10	7.48E-09	-0.00211346	1.07E-08	3.37E-07	10002.08588698892359e-10
NONHSAG019176	0.221225846	0.000268815	0.002863287	-0.00211884	8.42E-05	0.001052236	10010.000100270611930482
NONHSAG019180	0.132194341	0.10517832	0.37869582	-0.00231915	0.027212181	0.143565953	10020.0725373508064638
NONHSAG019199	0.00786647	0.915551947	0.967869201	-0.00233302	0.34591339	0.678791662	10030.511120906678612
NONHSAG019275	0.111196374	0.151785754	0.461928075	-0.00250463	0.018031713	0.106077773	10040.0570021578516866
NONHSAG019322	-0.05373924	0.286827309	0.623902976	-0.00262364	0.957306243	0.981988142	10050.48306660872358
NONHSAG019335	0.030392368	0.759661709	0.91407719	-0.00264367	0.256187078	0.587121487	10060.496490811817181
NONHSAG019355	-0.04531494	0.613562793	0.852440007	-0.00283452	0.93443763	0.973855106	10070.861668372443421
NONHSAG019359	0.007352111	0.927195914	0.973226917	-0.00303215	0.059496192	0.250454032	10080.10407922770487
NONHSAG019389	0.097751491	0.212481841	0.544533663	-0.00321666	0.864085651	0.948773467	10090.396873327232108
NONHSAG019395	0.171075807	0.005901949	0.042065782	-0.00338086	0.99182224	0.997026029	10100.00692214936629224
NONHSAG019396	0.197402939	0.074547001	0.30118702	-0.00347904	0.746743232	0.906696003	10110.161136530270525
NONHSAG019423	-0.20333116	0.003418542	0.026193297	-0.0035155	0.049503223	0.222724903	10120.0115989584690997
NONHSAG019434	0.044144782	0.504015987	0.789138617	-0.00365628	0.147088867	0.432159398	10130.343407358005694
NONHSAG019460	-0.0653782	0.395305525	0.711193473	-0.00368812	0.645650913	0.869586613	10140.69268125586563
NONHSAG019464	-0.02517516	0.756882429	0.913064517	-0.00376016	0.731514709	0.904979921	10150.806334666051101
NONHSAG019471	0.102324541	0.082193283	0.3214569	-0.00386632	0.054731284	0.236412243	10160.105749957072932
NONHSAG019477	0.004363961	0.958436087	0.983730538	-0.00392144	0.154819498	0.444237171	10170.245495538022801
NONHSAG019496	-0.04424687	0.619162997	0.855906179	-0.00395753	0.964478307	0.986709541	10180.832739452593487
NONHSAG019516	-0.04880231	0.45591477	0.756807752	-0.00403835	0.987500452	0.996001173	10190.681923490036479
NONHSAG019517	-0.04009456	0.384254343	0.706496807	-0.00419735	0.604637703	0.843792169	10200.375826458800517
NONHSAG019583	0.167167188	0.042942264	0.205430862	-0.00424366	0.020226379	0.115602803	10210.0418075810645278
NONHSAG019638	-0.03066567	0.60863445	0.850891147	-0.00424597	0.674413083	0.881776357	10220.859818047402556
NONHSAG019649	0.021850938	0.681128861	0.887517619	-0.00438975	0.594308121	0.839418789	10230.853952433152257
NONHSAG019657	0.028825522	0.484115465	0.773504695	-0.00443165	0.583245954	0.836050856	10240.759865349366065
NONHSAG019671	-0.02225184	0.76182017	0.915035797	-0.00444768	0.925073705	0.971770056	10250.91644131859904
NONHSAG019696	-0.03158646	0.531642164	0.811605736	-0.00447255	0.845066964	0.941492988	10260.812400184774766
NONHSAG019733	0.023828677	0.624918859	0.85822947	-0.0047031	0.587058222	0.836463428	10270.834172880412991
NONHSAG019747	0.087810018	0.2957912	0.632616096	-0.00475641	0.147409019	0.432235831	10280.323428945913983
NONHSAG019755	-0.02477668	0.764473696	0.9151554	-0.00482104	0.246493535	0.574769231	10290.479984189216386
NONHSAG019799	0.088248697	0.160497932	0.475073879	-0.00485313	0.071121137	0.282879495	10300.164538647154122
NONHSAG019803	0.234652244	0.013380461	0.082512841	-0.00488957	0.387783086	0.712097883	10310.0405863230518492
NONHSAG019855	-0.15908673	0.002888012	0.022748153	-0.00509529	0.009291928	0.062809857	10320.00567687701161002
NONHSAG019888	0.082608167	0.38995556	0.706543193	-0.0051282	0.650004425	0.873297871	10330.687179871331624
NONHSAG019921	-0.10467191	0.028102081	0.148539573	-0.0052923	0.127473104	0.40387881	10340.0784703460476707
NONHSAG019946	-1.3947445	2.94E-18	5.92E-16	-0.00537795	1.21E-12	1.03E-10	10351.35400258808034e-17
NONHSAG019954	0.021748581	0.741466536	0.909586883	-0.00559876	0.697294322	0.895338645	10360.914765609085242
NONHSAG020059	0.497846276	8.24E-05	0.000990025	-0.00583761	1.16E-05	0.000179758	10371.54658804810052e-05
NONHSAG020065	-0.03640532	0.596468507	0.846326605	-0.00609055	0.529651863	0.808158244	10380.510612903166282
NONHSAG020081	1.848672708	1.86E-23	2.61E-20	-0.00620149	8.90E-22	1.25E-18	10396.33884371272128e-24
NONHSAG020082	-0.08280782	0.333105842	0.669875107	-0.00624208	0.050328467	0.223890543	10400.14326466244228
NONHSAG020086	-0.10860639	0.148618147	0.458302307	-0.00629684	0.689823033	0.891590891	10410.323418376331396
NONHSAG020094	0.014507732	0.839781166	0.944963841	-0.00632607	0.827327551	0.933749876	10420.914418943387566
NONHSAG020096	0.213854878	0.049870367	0.229142928	-0.00638096	0.041389963	0.195282848	10430.0699592023455391
NONHSAG020099	-0.02695981	0.59478559	0.845494805	-0.00650314	0.725291212	0.902489903	10440.862118237277163
NONHSAG020107	0.026619017	0.709863942	0.898275053	-0.006576	0.372213745	0.69917505	10450.665192478679454
NONHSAG020130	-0.01299258	0.811678401	0.93441384	-0.00671966	0.498052822	0.79510374	10460.634723983614018
NONHSAG020182	0.047611371	0.499902337	0.786199872	-0.00678686	0.671837273	0.88168869	10470.789987477997855
NONHSAG020190	-0.02350355	0.765921516	0.915719092	-0.00681673	0.619145923	0.853798275	10480.722548829671791
NONHSAG020197	-0.0553171	0.502843953	0.789138617	-0.00690027	0.506159872	0.80071508	10490.740375510785146
NONHSAG020211	-0.00052886	0.994507362	0.996633893	-0.00692074	0.861379511	0.947290977	10500.978768331314614
NONHSAG020225	-0.07942629	0.37484939	0.699453541	-0.00717647	0.746031566	0.906696003	10510.455041258434339
NONHSAG020230	0.007118967	0.911242153	0.966219055	-0.00728521	0.624090375	0.855234958	10520.874720575544184
NONHSAG020240	0.005482078	0.941591053	0.9777526	-0.00730661	0.153659925	0.442263776	10530.27247992561805
NONHSAG020323	0.102045661	0.140657349	0.44491391	-0.00748834	0.011559972	0.07542144	10540.0387527177495002
NONHSAG020329	0.46915853	2.38E-10	8.82E-09	-0.00756804	3.48E-08	9.60E-07	10553.64268686027496e-10
NONHSAG020334	-0.00118011	0.988610823	0.996279605	-0.00762525	0.753538804	0.907097076	10560.932589579905783

NONHSAG020338	-0.29617234	0.000480631	0.004809735	-0.0076711	0.030618841	0.154856439	10570.00196950531676017
NONHSAG020339	-0.09052156	0.183120792	0.510285663	-0.00775664	0.12711229	0.403430881	10580.249972889434087
NONHSAG020347	0.338340263	5.40E-05	0.000687689	-0.00784826	2.17E-05	0.000308799	10591.89596205864971e-05
NONHSAG020419	-0.36089518	2.86E-06	4.55E-05	-0.00791476	0.00174531	0.015194462	10601.32507457670546e-05
NONHSAG020424	-0.1286713	0.148758707	0.458302307	-0.00792559	0.281394408	0.612446652	10610.320895082619861
NONHSAG020428	0.027220222	0.652348933	0.872061952	-0.00807836	0.122123632	0.391575432	10620.11613745201855
NONHSAG020432	-0.05214509	0.596064749	0.846326605	-0.00809544	0.965503603	0.986709541	10630.813545274023071
NONHSAG020434	0.005368905	0.935304243	0.975411819	-0.00814744	0.097927296	0.344214444	10640.173959337245793
NONHSAG020483	0.047467041	0.599315087	0.847002984	-0.00843182	0.722744373	0.902489903	10650.673930336338254
NONHSAG020539	0.125147758	0.164900325	0.485549438	-0.00851061	0.006670795	0.04773098	10660.0239476034131649
NONHSAG020550	0.413138429	0.000270489	0.002870245	-0.00853518	2.72E-05	0.000382215	10674.6971620368668e-05
NONHSAG020567	-0.02095511	0.799109011	0.929703988	-0.0085692	0.528403018	0.807576213	10680.658358197101292
NONHSAG020580	-0.01293312	0.87915882	0.954268263	-0.00863517	0.30001578	0.629081811	10690.431575823749812
NONHSAG020618	0.008913567	0.938632176	0.976844441	-0.00877288	0.886670699	0.957863237	10700.989729605253424
NONHSAG020638	-0.04609797	0.398782052	0.712889467	-0.00895855	0.749449278	0.906696003	10710.691893269621734
NONHSAG020650	0.050090483	0.547316727	0.819954522	-0.00899454	0.721005126	0.902489903	10720.830482411621112
NONHSAG020678	0.244104326	0.000752595	0.007077921	-0.00905535	0.000284324	0.003147711	10730.000366264801907948
NONHSAG020737	0.220367053	0.029684833	0.155444602	-0.00918916	0.133993904	0.416804045	10740.0828792429863895
NONHSAG020798	0.096321432	0.103826935	0.37672431	-0.00920894	0.375208366	0.701519896	10750.260749887706828
NONHSAG020806	0.07992492	0.220896764	0.55460866	-0.00942777	0.5955336	0.839418789	10760.465401028572698
NONHSAG020809	0.028123682	0.752796068	0.910732246	-0.00954976	0.724352352	0.902489903	10770.92678930433652
NONHSAG020830	-0.0364584	0.610872106	0.851851115	-0.00958961	0.84975676	0.942779949	10780.768476216282423
NONHSAG020837	0.059695926	0.265179595	0.603793395	-0.00961764	0.654948313	0.876578873	10790.528243695435958
NONHSAG020855	-0.00843998	0.887219643	0.956660206	-0.00981913	0.479994456	0.78291439	10800.659409347324091
NONHSAG020867	-0.01511064	0.722443905	0.902894338	-0.00992558	0.829459354	0.933749876	10810.937119944097629
NONHSAG020883	0.076217948	0.29438503	0.632399317	-0.01000267	0.005039938	0.03759232	10820.0167968613413362
NONHSAG020899	-0.14044856	0.014354455	0.087559061	-0.01002011	0.1521991	0.439860091	10830.047046311306066
NONHSAG020905	0.528508719	8.35E-11	3.56E-09	-0.01005069	2.20E-10	1.05E-08	10842.16006072862873e-11
NONHSAG020941	0.420590856	0.001413939	0.012271596	-0.01008626	6.01E-06	0.000101906	10852.44333572543991e-05
NONHSAG020993	0.084818575	0.208773937	0.542233781	-0.0100995	0.025428405	0.136199381	10860.078864584681439
NONHSAG021003	0.099658016	0.198663458	0.528551011	-0.01011898	0.055175958	0.237967477	10870.145851474608491
NONHSAG021015	0.013381897	0.872784361	0.952744419	-0.01023497	0.898325034	0.962120226	10880.985563261622522
NONHSAG021045	0.107251561	0.151346441	0.461203041	-0.01024565	0.336584413	0.671160358	10890.339453093421735
NONHSAG021143	0.442900421	6.93E-05	0.000855964	-0.01024931	0.000106143	0.001281006	10905.48126909770836e-05
NONHSAG021154	0.158672752	0.168061801	0.49074744	-0.01033576	0.168233615	0.465165119	10910.280009719083614
NONHSAG021213	0.072356942	0.423211368	0.727871783	-0.01040068	0.863817034	0.948773467	10920.581279981860841
NONHSAG021241	-0.07915351	0.330297608	0.669162012	-0.01047462	0.756806814	0.908685209	10930.605318636665478
NONHSAG021246	0.716102344	3.77E-10	1.33E-08	-0.01047964	4.21E-09	1.50E-07	10942.158317372239e-10
NONHSAG021274	0.074958224	0.39555711	0.711193473	-0.01059347	0.178053196	0.482403482	10950.391110961199058
NONHSAG021287	-0.03733982	0.567171629	0.831970068	-0.01060005	0.641407807	0.867551108	10960.581539781867754
NONHSAG021374	-0.08836468	0.130721533	0.429206713	-0.010678	0.267474767	0.598817815	10970.290582828471711
NONHSAG021403	0.038197351	0.683513649	0.887517619	-0.01075619	0.293829606	0.623485995	10980.324639650658912
NONHSAG021436	0.094382452	0.215257245	0.548282042	-0.0107693	0.085740376	0.317657362	10990.205037658020363
NONHSAG021444	0.14015221	0.187898583	0.516279483	-0.0107874	0.073738898	0.289233024	11000.177426115704635
NONHSAG021459	0.02173727	0.778525917	0.920611808	-0.0109184	0.265766088	0.596878743	11010.507032529127531
NONHSAG021464	0.01634633	0.887938526	0.956660206	-0.01092982	0.402383641	0.724100325	11020.665347365917052
NONHSAG021479	0.683975212	1.17E-07	2.48E-06	-0.01094113	8.70E-09	2.85E-07	11037.05160145819307e-09
NONHSAG021502	0.133384192	0.095080689	0.358400667	-0.01099098	0.827876331	0.933749876	11040.189749205101076
NONHSAG021504	-0.02207472	0.795933292	0.927815057	-0.01102427	0.517019508	0.804430331	11050.805852964548089
NONHSAG021505	-0.04905081	0.598714361	0.847002984	-0.01105028	0.511273845	0.803634462	11060.49543450389024
NONHSAG021537	0.110555883	0.05093795	0.231460286	-0.01116878	0.001343501	0.011917741	11070.00524060800284489
NONHSAG021546	-0.07673476	0.311485356	0.650740581	-0.01118565	0.292090648	0.621300229	11080.487446595903308
NONHSAG021557	-0.39239966	0.000382932	0.003887385	-0.01124823	0.000137147	0.001600234	11090.000163569357773957
NONHSAG021577	-0.12037098	0.281856372	0.617755353	-0.01129954	0.401450413	0.724046292	11100.523941152326281
NONHSAG021582	0.017377677	0.808794115	0.93439361	-0.01132364	0.562546936	0.826911648	11110.842306129981217
NONHSAG021608	-0.10919956	0.176962273	0.502137145	-0.01142901	0.669867876	0.880630922	11120.380273586039759
NONHSAG021662	-0.1910242	0.012175938	0.077463207	-0.01144841	0.034859995	0.170184558	11130.0271648395595995
NONHSAG021671	-0.05864694	0.303373217	0.641417659	-0.01161205	0.230739803	0.555038773	11140.0909939899013629
NONHSAG021696	0.137834608	0.012649294	0.079220077	-0.01179483	0.007886916	0.055032275	11150.0126598480180161
NONHSAG021701	0.440563243	1.70E-09	5.38E-08	-0.0117993	1.29E-08	3.93E-07	11169.21637907368829e-10
NONHSAG021755	0.106933411	0.186902049	0.5162756	-0.01180276	0.705989777	0.896707014	11170.391564449055408
NONHSAG021758	-0.10672389	0.199882199	0.529483422	-0.01184548	0.251728746	0.582122725	11180.368135126978186
NONHSAG021801	0.185328864	0.059638015	0.258686341	-0.0118666	0.017656319	0.104511744	11190.0436574923648609
NONHSAG021825	0.067106513	0.25270896	0.587773032	-0.01208287	0.032366775	0.160238331	11200.0979460345577779
NONHSAG021851	0.224985872	0.04474914	0.211486692	-0.01208714	0.024302017	0.132211959	11210.0479663049059595
NONHSAG021881	-0.45975765	2.83E-05	0.000377801	-0.01220741	0.012916297	0.081803217	11220.00013617099555398
NONHSAG021904	0.012752431	0.82920801	0.941353623	-0.01231184	0.225073849	0.547024774	11230.428114690321726
NONHSAG021905	0.341286892	7.65E-05	0.000927579	-0.01231814	1.14E-07	2.75E-06	11244.38186250451402e-07
RUNDC3A-AS1	1.404230939	1.40E-17	2.06E-15	-0.01245445	1.20E-16	4.22E-14	28083.05303708716224e-18
NONHSAG021933	-0.06485526	0.144720673	0.451169103	-0.0124689	0.108672864	0.365535999	11250.206063985924158
NONHSAG022026	-0.0904115	0.234238878	0.567820743	-0.01252172	0.056277142	0.239714801	11260.15294142388731

NONHSAG022055	-0.0045844	0.945253653	0.979231332	-0.01252518	0.80974602	0.930212019	11270.969276784295164
NONHSAG022065	0.091409561	0.249383254	0.584860584	-0.01253254	0.013356571	0.084212282	11280.0444431339237026
NONHSAG022068	0.182860786	0.041318182	0.199291129	-0.01254449	0.008419379	0.058428223	11290.0223234319500511
NONHSAG022073	0.072130058	0.261178568	0.6018673	-0.01259741	0.343471007	0.677208152	11300.47834610452368
NONHSAG022088	-0.03858143	0.706864294	0.898275053	-0.0126111	0.545177017	0.815012106	11310.827606861190794
NONHSAG022089	0.000921924	0.98875586	0.996279605	-0.01266496	0.341057124	0.674686654	11320.547923476096057
NONHSAG022092	-0.06711427	0.358480228	0.684814131	-0.01284064	0.722961159	0.902489903	11330.647129353804407
NONHSAG022177	-0.05383557	0.349413794	0.677785637	-0.01292079	0.51496664	0.804430331	11340.627536876315644
NONHSAG022214	-0.06753751	0.222624031	0.555966941	-0.01301725	0.897480109	0.962120226	11350.403487991657679
NONHSAG022224	0.12198913	0.112322734	0.394321507	-0.01327359	0.108025277	0.364229112	11360.181702605615443
NONHSAG022252	0.338950457	4.22E-08	9.58E-07	-0.01335587	1.82E-10	8.81E-09	11373.18238659575633e-10
NONHSAG022256	0.100439141	0.134176345	0.435183256	-0.01336945	0.632753622	0.860695556	11380.304745914207703
NONHSAG022261	0.156701734	0.050408266	0.230860006	-0.01341776	0.890859354	0.958708067	11390.0662123079084386
NONHSAG022297	0.043151485	0.479967915	0.76981996	-0.01346161	0.842379903	0.941172323	11400.63383619359796
NONHSAG022308	-0.01409864	0.839370615	0.944963841	-0.01352902	0.428310198	0.746182739	11410.573429889866484
NONHSAG022311	0.165867826	0.024530673	0.13358443	-0.01355106	0.027441633	0.144235277	11420.0376308046599188
NONHSAG022328	0.038181254	0.461466216	0.760209241	-0.01380944	0.774870498	0.913983155	11430.75601743871359
NONHSAG022337	-0.0464822	0.54046	0.816860993	-0.01383667	0.14627862	0.432159398	11440.111313557908388
NONHSAG022342	-0.13637355	0.038862993	0.190388043	-0.0139476	0.077735661	0.298216479	11450.0831532496032782
NONHSAG022353	-0.00439073	0.9482226	0.98065537	-0.01402133	0.366290466	0.69386861	11460.5536729585189
NONHSAG022357	0.323603031	0.000102881	0.001200417	-0.01406696	0.000127354	0.0015047	11477.64705136991365e-05
NONHSAG022358	0.047192292	0.65505885	0.872584313	-0.01415064	0.183629188	0.488163241	11480.394784619956279
NONHSAG022437	0.114083881	0.140288205	0.444245984	-0.01417842	0.150252825	0.436929622	11490.241399583544037
NONHSAG022492	-0.18879989	0.012393246	0.078173941	-0.01420162	0.020675036	0.117450912	11500.021543057465482
NONHSAG022576	0.069110852	0.520920881	0.803843214	-0.01421387	0.079997269	0.304454568	11510.203252752601856
NONHSAG022580	-0.27479715	0.001816221	0.015245414	-0.0142623	0.003426526	0.026617099	11520.00257568659425996
NONHSAG022588	0.597729758	3.83E-12	2.11E-10	-0.01448386	4.65E-10	2.11E-08	11534.18193026315893e-12
NONHSAG022627	0.19241697	0.015356421	0.092047713	-0.01453376	0.003321387	0.026161737	11540.00777718272037372
NONHSAG022655	0.00298732	0.969722475	0.989067682	-0.0145439	0.777772398	0.915750268	11550.939849077347607
NONHSAG022656	0.079693204	0.303119308	0.641397052	-0.01460419	0.243279761	0.570085574	11560.440093262026932
NONHSAG022668	-0.02725671	0.708538461	0.898275053	-0.0147793	0.938734989	0.975531432	11570.888930293875012
NONHSAG022676	-0.00322775	0.959893419	0.98403948	-0.01478525	0.667365979	0.880630922	11580.894089318716785
NONHSAG022677	0.082541037	0.326933943	0.667176847	-0.01485055	0.790478141	0.921569044	11590.422052829601623
NONHSAG022735	0.056645902	0.539003204	0.815757271	-0.0148796	0.708543236	0.896707014	11600.823386663484776
NONHSAG022759	0.502740595	5.59E-11	2.42E-09	-0.01489036	6.51E-10	2.81E-08	11612.83373939873155e-11
NONHSAG022817	0.001346303	0.983457064	0.995038946	-0.01495067	0.59791148	0.839825518	11620.82245442319856
NONHSAG022830	-0.15338429	0.016085417	0.096034379	-0.01496202	0.476612641	0.780309094	11630.0448502971680859
NONHSAG022841	0.040075955	0.50541979	0.790896188	-0.01518896	0.628806616	0.858449701	11640.786632985423973
NONHSAG022852	-0.0562988	0.449045408	0.751820679	-0.01532394	0.575123228	0.833526329	11650.418544210203124
NONHSAG022858	-0.05741549	0.324236635	0.6640593	-0.01539896	0.932628841	0.973855106	11660.490009863574625
NONHSAG022867	0.047409381	0.54852369	0.820817175	-0.01539989	0.118659227	0.384412149	11670.28417108387507
NONHSAG022906	-0.01847758	0.792090669	0.927698609	-0.015443	0.713820461	0.898104312	11680.816726545914249
NONHSAG022924	0.104431288	0.1078057	0.385196478	-0.01548918	0.13416596	0.416878099	11690.198092314326306
NONHSAG022925	0.336881748	0.001775001	0.014944021	-0.01570945	0.000518388	0.005300752	11700.000825833680344079
NONHSAG022957	-0.01875163	0.833305614	0.942580607	-0.01574614	0.980542506	0.992468062	11710.973475957841641
NONHSAG022968	0.086778921	0.272099771	0.60896612	-0.01577832	0.500488703	0.797130817	11720.537188085067331
NONHSAG022969	0.06613744	0.380760229	0.705334496	-0.01582553	0.936644692	0.975416231	11730.566841632628674
NONHSAG022994	0.234875746	0.000200264	0.00219977	-0.01592293	0.00734348	0.051754051	11740.000693655256230349
NONHSAG023009	0.107804819	0.172161046	0.496021375	-0.01607927	0.032596405	0.16109155	11750.0942371766694519
NONHSAG023036	-0.69741852	9.16E-10	3.03E-08	-0.01611786	7.12E-07	1.54E-05	11762.57403074304988e-09
NONHSAG023052	0.092028879	0.283669922	0.619797841	-0.01640929	0.253590987	0.58512763	11770.437708427623217
NONHSAG023067	0.063101744	0.31414105	0.651448843	-0.01650203	0.523490981	0.80637524	11780.25743327164774
NONHSAG023085	0.088021069	0.214634728	0.548282042	-0.01651171	0.294984624	0.623681777	11790.0801743921285287
NONHSAG023088	0.20736417	0.014442306	0.087573347	-0.01654839	0.001298058	0.011551073	11800.00377769878417312
NONHSAG023107	-0.03352411	0.703191046	0.895955243	-0.01658353	0.435732649	0.750543033	11810.734795019018363
NONHSAG023114	0.312061578	0.000149675	0.001697127	-0.01663003	6.57E-07	1.43E-05	11822.2183352325428e-06
NONHSAG023118	-0.05085188	0.579858864	0.839662241	-0.0166969	0.903665954	0.963636202	11830.84214131006619
NONHSAG023129	0.69939532	2.77E-09	8.20E-08	-0.01677626	1.20E-06	2.44E-05	11847.16142166344524e-09
NONHSAG023173	0.132645373	0.194232899	0.526251063	-0.01679083	0.044383264	0.205272595	11850.122899959641658
NONHSAG023180	0.024953505	0.775391988	0.919612936	-0.01685276	0.541921477	0.812737703	11860.656391025316169
NONHSAG023185	-0.06728062	0.389907241	0.706543193	-0.01685868	0.425016696	0.741866512	11870.628187139981278
NONHSAG023194	-0.22262472	0.050210304	0.230328506	-0.01703266	0.517521977	0.804430331	11880.0292937302638459
NONHSAG023237	0.421988178	7.32E-06	0.000107783	-0.01703594	8.53E-05	0.001056939	11891.14745948291031e-05
NONHSAG023238	0.314294524	5.11E-06	7.86E-05	-0.01703774	0.000200427	0.002263454	11901.23099197846423e-05
NONHSAG023245	0.057339582	0.313674607	0.651448843	-0.01716501	0.140943747	0.426624131	11910.318366349973964
NONHSAG023247	0.045454631	0.328016903	0.668343876	-0.01724599	0.173472934	0.474519348	11920.369609075121926
NONHSAG023281	-0.01031382	0.868394174	0.951960393	-0.01743157	0.240678929	0.567946237	11930.343746231031895
NONHSAG023286	-0.02617726	0.76687014	0.916074271	-0.01745175	0.838154497	0.93937443	11940.954381386868938
NONHSAG023298	-0.01060711	0.885290914	0.956660206	-0.01752239	0.518118734	0.804430331	11950.791837944859996
NONHSAG023327	0.114180242	0.146347414	0.454226189	-0.01768612	0.266508448	0.59714881	11960.312421909431478
NONHSAG023350	0.084701662	0.063217713	0.268938288	-0.01788623	0.522727059	0.80637524	11970.163781216021895

NONHSAG023373	0.018118065	0.832110803	0.942580607	-0.01794632	0.824101669	0.933749876	11980.968575796322801
NONHSAG023390	0.003358463	0.965213583	0.986974762	-0.01797565	0.677241027	0.884123383	11990.899314765442055
NONHSAG023427	0.019404194	0.750653559	0.910732246	-0.01802251	0.556197077	0.822841924	12000.653486932304439
NONHSAG023436	0.034487865	0.646689313	0.870666685	-0.01805404	0.428816254	0.746182739	12010.726133979319503
NONHSAG023437	0.096536783	0.276793644	0.612912715	-0.01810803	0.086280039	0.318398253	12020.218188186484919
NONHSAG023456	-0.01074246	0.86138652	0.951382127	-0.01816003	0.657339379	0.877865914	12030.814383174786328
NONHSAG023530	0.016567978	0.793611378	0.927815057	-0.01824732	0.516760103	0.804430331	12040.805836716752095
NONHSAG023534	-0.30124804	7.60E-06	0.000111268	-0.01831596	8.02E-05	0.00101616	12051.14668597211346e-05
NONHSAG023539	0.302334176	8.41E-06	0.000121088	-0.018336	1.71E-09	6.60E-08	12067.55853906237124e-09
NONHSAG023582	-0.01463891	0.864169775	0.951839172	-0.0184835	0.371059718	0.69793975	12070.633152489615981
NONHSAG023627	-0.01499619	0.810651308	0.93441384	-0.01849744	0.471557372	0.778445171	12080.6053978291348
NONHSAG023639	0.024027705	0.684955898	0.887517619	-0.01852917	0.490181526	0.789004266	12090.78358579571937
NONHSAG023666	-0.02269833	0.782422167	0.921921799	-0.01863449	0.263242376	0.594568322	12100.33626232175488
NONHSAG023691	-0.10386835	0.197010296	0.527031094	-0.0188273	0.5441901	0.815012106	12110.158810024062647
NONHSAG023704	-0.20035253	0.015047036	0.090994117	-0.01884288	0.212648535	0.52638	12120.0499220404852326
NONHSAG023707	-0.05216667	0.373806631	0.698899101	-0.01887469	0.528155883	0.807576213	12130.654520339953022
NONHSAG023711	-0.01077895	0.847981446	0.947482162	-0.01898154	0.662995305	0.880630922	12140.811421616493381
NONHSAG023714	-0.03414795	0.669439308	0.882022997	-0.01899703	0.929895668	0.973855106	12150.902062921655292
NONHSAG023715	0.012919458	0.823984053	0.938461188	-0.01903434	0.643208037	0.868581319	12160.781074483129584
NONHSAG023765	-0.19595182	0.014077959	0.086059173	-0.01917591	0.286045792	0.615425223	12170.0459062008373741
NONHSAG023770	-0.00362981	0.961899816	0.985377881	-0.01928307	0.742235257	0.906043339	12180.937952244602448
NONHSAG023771	0.02881249	0.65975392	0.875035827	-0.01934347	0.914401079	0.968016258	12190.843270806394668
NONHSAG023772	0.014937959	0.796143563	0.927815057	-0.01944267	0.180576855	0.484356613	12200.359870231730044
NONHSAG023787	0.031881305	0.488287389	0.776180972	-0.01953089	0.674981222	0.881992191	12210.531432902157052
NONHSAG023790	0.021712016	0.721725097	0.902397054	-0.01985943	0.06593465	0.271064674	12220.146024262099247
NONHSAG023801	-0.04586978	0.49436659	0.781169053	-0.01994127	0.755042287	0.907341414	12230.594031638432619
NONHSAG023810	0.002272016	0.979722562	0.993501254	-0.01996138	0.053281009	0.232952053	12240.0860077157162788
NONHSAG023839	0.107240645	0.170451226	0.493623942	-0.01997726	0.812281126	0.931253814	12250.337475406018397
NONHSAG023915	0.069838038	0.193684286	0.526251063	-0.02013127	0.100817176	0.349991359	12260.221243970874192
NONHSAG023920	0.040224835	0.562936034	0.830087115	-0.02023615	0.497194564	0.794835843	12270.761907110610212
NONHSAG023923	0.155616572	0.173060209	0.497084073	-0.02033572	0.021640574	0.121221704	12280.0675449286044144
NONHSAG023954	0.095146788	0.223378218	0.556499409	-0.02044729	0.355769478	0.686695518	12290.442399657081407
NONHSAG023966	-0.07198758	0.179810207	0.504113961	-0.02045382	0.729758125	0.904080089	12300.373891555257899
NONHSAG023972	0.030976511	0.763543841	0.915083941	-0.02045703	0.513389391	0.804430331	12310.804837609597714
NONHSAG024013	0.141530166	0.117951791	0.406969862	-0.02049278	0.508560599	0.802059678	12320.0795093884424344
NONHSAG024040	0.165126925	0.01841195	0.106772915	-0.02057037	0.356992677	0.686695518	12330.0567248603066087
NONHSAG024043	-0.00191908	0.977803949	0.993347076	-0.02060527	0.718225045	0.902031633	12340.90912768774952
NONHSAG024046	-0.43498422	7.92E-05	0.000955456	-0.02063571	0.00239866	0.019896847	12350.000236440138530771
NONHSAG024080	0.089193835	0.281700571	0.617755353	-0.02068623	0.974526679	0.989451568	12360.468810175799872
NONHSAG024104	-0.1161636	0.047083514	0.218120003	-0.02071289	0.950693703	0.979405751	12370.0676314896102566
NONHSAG024105	0.04470684	0.421810487	0.72679601	-0.02079341	0.041209748	0.194759347	12380.116572486626008
NONHSAG024135	-0.00786058	0.933306803	0.975272661	-0.02088609	0.091709156	0.331047686	12390.136783928416004
NONHSAG024143	0.056562833	0.577701261	0.83866595	-0.02090937	0.520660223	0.806220565	12400.781621294261957
NONHSAG024146	-0.07522256	0.174650962	0.498537648	-0.02092673	0.659949666	0.878826774	12410.377729448298059
NONHSAG024157	0.035114493	0.603904695	0.848743629	-0.02094868	0.179008511	0.483942149	12420.393596806005879
NONHSAG024162	0.006182927	0.945893433	0.979327074	-0.02101079	0.849356985	0.942779949	12430.964442248271505
NONHSAG024182	-0.16366226	0.017604174	0.103562632	-0.02152584	0.039578801	0.189278211	12440.0367880565516665
NONHSAG024209	0.019194098	0.773225577	0.918276503	-0.02154185	0.974673131	0.989451568	12450.950624346405414
NONHSAG024232	-0.03182942	0.487224987	0.775687946	-0.02162452	0.925949642	0.971770056	12460.687768505909716
NONHSAG024234	-0.18783142	0.005474021	0.039672547	-0.02168393	0.00624516	0.045261317	12470.00694784432319062
NONHSAG024244	0.054632015	0.344208804	0.677284107	-0.02185678	0.150959948	0.43807985	12480.340387916990771
NONHSAG024248	0.363986933	1.92E-07	3.86E-06	-0.0218919	9.31E-07	1.95E-05	12491.32128246091629e-07
NONHSAG024264	0.016439779	0.809230624	0.93439361	-0.02204429	0.788748303	0.921462496	12500.956758882445306
NONHSAG024327	-0.13933264	0.253659459	0.588523431	-0.0221078	0.573824787	0.833292346	12510.516369474007004
NONHSAG024381	-0.28663334	3.32E-05	0.000434732	-0.02216878	0.001416258	0.012484384	12520.000100794792827686
NONHSAG024388	0.029692354	0.700800093	0.895749937	-0.0223676	0.621228338	0.853798275	12530.67697959192497
NONHSAG024397	0.148365344	0.032157511	0.164114197	-0.02237519	0.033891147	0.166320953	12540.0492640176290442
NONHSAG024439	0.111512498	0.197655439	0.527831999	-0.02243493	0.384223319	0.709976889	12550.417410963126313
NONHSAG024442	-0.06886522	0.2879826	0.62446068	-0.02256552	0.336096771	0.671160358	12560.499212920392113
NONHSAG024446	0.011769739	0.846027057	0.946688454	-0.022572	0.573840974	0.833292346	12570.73224456413137
NONHSAG024447	-0.00302929	0.964425056	0.986886193	-0.02264102	0.128119426	0.405131222	12580.22155842206264
NONHSAG024529	0.096839706	0.3121671	0.651448843	-0.0226817	0.180044728	0.484025789	12590.373393673461376
NONHSAG024540	-0.00979571	0.912268523	0.966267893	-0.02301084	0.103192177	0.353012654	12600.15070293710264
NONHSAG024542	0.056075797	0.34777361	0.677785637	-0.02301743	0.79808768	0.924443772	12610.620043442414574
NONHSAG024613	-0.01422717	0.854130238	0.949662955	-0.02335919	0.190518451	0.497257471	12620.362056740083574
NONHSAG024628	0.076706599	0.262835364	0.6018673	-0.02348305	0.867832934	0.950047122	12630.477538850761729
NONHSAG024729	0.047567894	0.512273277	0.796980196	-0.02350217	0.527677572	0.807576213	12640.756019236537616
NONHSAG024733	0.093611299	0.048005141	0.221295832	-0.02352446	0.331456314	0.665753681	12650.137572256273436
NONHSAG024740	0.128967946	0.095655214	0.360018759	-0.02358582	0.384604011	0.709976889	12660.244533705048088
NONHSAG024741	-0.00034123	0.99341273	0.996600003	-0.02360137	0.683812981	0.889213579	12670.891918681621395
NONHSAG024742	-0.09190352	0.079009551	0.312727937	-0.02362912	0.32914235	0.663384602	12680.20826440432889

NONHSAG024745	-0.02731705	0.746559931	0.909586883	-0.02367446	0.748804574	0.906696003	12690.811385120169901
NONHSAG024752	-0.0307934	0.652423999	0.872061952	-0.02376329	0.901271192	0.962240579	12700.896011719965004
NONHSAG024859	0.036921157	0.521455627	0.803843214	-0.02377411	0.015389941	0.093875301	12710.0409131454147916
NONHSAG024870	0.052081199	0.492365471	0.779578663	-0.02379001	0.324561259	0.658014608	12720.596532677828614
NONHSAG024871	0.245355435	0.00070817	0.006727619	-0.02385854	0.111832831	0.369410385	12730.00291449067128303
NONHSAG024916	0.076943959	0.326945126	0.667176847	-0.02386475	0.538072848	0.812140251	12740.608084238971866
NONHSAG024953	-0.21024163	0.022229191	0.123238983	-0.02390892	0.311875057	0.641076506	12750.0694286063540433
NONHSAG024968	-0.12309935	0.141360422	0.446229631	-0.02391463	0.501749808	0.798023206	12760.332750421004852
NONHSAG024985	0.119385918	0.18091078	0.506694335	-0.0240128	0.357691351	0.686695518	12770.38782507992757
NONHSAG024988	0.102804545	0.142003117	0.447555229	-0.02413791	0.160562315	0.45587637	12780.250884522194773
NONHSAG024994	0.037034778	0.684289127	0.887517619	-0.02423819	0.000959924	0.009027781	12790.00158591955227443
NONHSAG025009	-0.03650665	0.619405787	0.855906179	-0.02423965	0.924853428	0.971770056	12800.815471732665386
NONHSAG025049	-0.00793974	0.929002142	0.97333618	-0.02429955	0.009334539	0.06294658	12810.0103984267512224
NONHSAG025052	0.427607884	1.14E-07	2.44E-06	-0.02432357	1.12E-07	2.73E-06	12823.25189781571458e-08
NONHSAG025054	0.44493357	4.09E-06	6.39E-05	-0.02443949	5.74E-05	0.000747788	12836.45989918297284e-06
NONHSAG025151	0.210530891	0.020528011	0.115913189	-0.02453098	0.133147822	0.414630871	12840.0619682981488941
NONHSAG025174	-0.06461068	0.475399905	0.767024669	-0.02459784	0.565855312	0.830039196	12850.436106105000951
NONHSAG025195	-0.44604492	5.22E-06	7.97E-05	-0.02462239	1.01E-06	2.10E-05	12868.64528681465851e-07
NONHSAG025224	-2.09440221	2.43E-17	2.97E-15	-0.02471784	2.08E-12	1.67E-10	12879.16167325827345e-17
NONHSAG025230	-0.03018867	0.68504307	0.887517619	-0.02480452	0.348288841	0.680716946	12880.638268750359542
NONHSAG025265	-0.12702832	0.078992619	0.312727937	-0.02491212	0.067978746	0.276319389	12890.117747234344596
NONHSAG025272	-0.05249564	0.616640457	0.854604714	-0.02493691	0.568337814	0.831933314	12900.821864307331054
NONHSAG025274	0.075000658	0.277884322	0.612912715	-0.02503156	0.629183796	0.858449701	12910.548944965429364
NONHSAG025288	-0.02809985	0.780810973	0.920990125	-0.02506562	0.742593127	0.906043339	12920.829975538837446
NONHSAG025295	0.074039314	0.21512003	0.548282042	-0.02514171	0.912346833	0.967390382	12930.324000556735571
NONHSAG025343	0.000780272	0.989021543	0.996279605	-0.02517194	0.472825161	0.778445171	12940.710922447889303
NONHSAG025349	0.042053	0.442442903	0.745892952	-0.02518505	0.07368433	0.289233024	12950.194948776936892
NONHSAG025352	-0.01773827	0.839638151	0.944963841	-0.02518548	0.337083114	0.671160358	12960.461097862471683
NONHSAG025367	-0.9236761	3.14E-14	2.33E-12	-0.0253304	4.90E-11	2.87E-09	12976.07501212442429e-14
NONHSAG025376	0.178592948	0.061448247	0.263837515	-0.02538927	0.079216507	0.303070502	12980.11128978361614
NONHSAG025378	0.108994868	0.043942208	0.208725488	-0.02546865	0.356732921	0.686695518	12990.126753408954197
NONHSAG025393	0.008042261	0.926815259	0.973226917	-0.02554728	0.396503244	0.718406651	13000.581917344674563
NONHSAG025422	-0.09070173	0.171819236	0.496021375	-0.02565004	0.759114792	0.909127255	13010.352800710202803
NONHSAG025431	-0.00474737	0.957807179	0.983730538	-0.02567703	0.82851736	0.933749876	13020.959498783246592
NONHSAG025438	-0.13130743	0.06998361	0.287798088	-0.02568116	0.741272014	0.906043339	13030.152098949143355
NONHSAG025448	0.063668382	0.420116155	0.726032559	-0.02571905	0.476501503	0.780309094	13040.674945949088936
NONHSAG025466	0.266015294	0.000274123	0.002887019	-0.02580264	0.014637302	0.089869198	13050.00106283482219119
NONHSAG025491	-0.00648282	0.928080041	0.97333618	-0.02581284	0.595146464	0.839418789	13060.79592254433221
NONHSAG025494	-0.0617612	0.373740997	0.698899101	-0.0259078	0.100176384	0.348633655	13070.253446588178504
NONHSAG025500	0.079023182	0.266522201	0.604403572	-0.025908	0.119073039	0.384865961	13080.272615007614583
NONHSAG025531	0.130715201	0.01942033	0.110995872	-0.02596901	0.049492572	0.222724903	13090.043297091155012
NONHSAG025536	0.526156173	7.59E-08	1.67E-06	-0.02597526	7.76E-07	1.65E-05	13106.70585859141806e-08
NONHSAG025542	0.032309556	0.701920049	0.895955243	-0.02607147	0.576930892	0.833674033	13110.637806370797986
NONHSAG025649	-0.08813302	0.473443414	0.765128092	-0.02607511	0.389658237	0.713031652	13120.650647596978146
NONHSAG025666	0.044381169	0.410347223	0.720284888	-0.02620814	0.42574072	0.742669296	13130.642411157004673
NONHSAG025675	0.055585446	0.261734258	0.6018673	-0.02629655	0.001761944	0.015244881	13140.00602281829026231
NONHSAG025695	0.262172171	0.000854069	0.007874233	-0.02639272	0.179460737	0.484025789	13150.00322558079499248
NONHSAG025699	0.038123254	0.468183708	0.764261292	-0.02646901	0.953815523	0.980474941	13160.719480746769663
NONHSAG025705	0.226312728	0.005793158	0.041557042	-0.02648172	0.08221402	0.310316541	13170.0199402865077907
NONHSAG025710	-0.07291438	0.268040026	0.605799463	-0.02650224	0.08499608	0.316426624	13180.214501867579255
NONHSAG025719	0.047090269	0.419520187	0.726032559	-0.02650251	0.219482793	0.53727159	13190.454710193286902
NONHSAG025790	0.011152445	0.870034926	0.951960393	-0.02651194	0.98680987	0.99566177	13200.980088801107659
NONHSAG025812	-0.02580797	0.644070487	0.870030385	-0.02651771	0.187059318	0.492981071	13210.185206046641624
NONHSAG025869	0.05311802	0.468234914	0.764261292	-0.02670491	0.899774981	0.962240579	13220.73745251279184
NONHSAG025872	-0.0053397	0.941254485	0.9777526	-0.02674966	0.045018182	0.207526438	13230.0633815842923077
NONHSAG025881	0.066279044	0.309342853	0.647268241	-0.02682823	0.110256074	0.367694695	13240.266777542303495
NONHSAG025897	0.022286232	0.776278409	0.920117545	-0.02684743	0.673438054	0.881776357	13250.775407761587476
NONHSAG025979	0.043735685	0.537212662	0.813923495	-0.02689491	0.766427375	0.912444445	13260.824324426677252
NONHSAG025986	0.060356674	0.464943451	0.763571876	-0.0269062	0.917624356	0.969807885	13270.728385493268699
NONHSAG026033	0.086585461	0.133435263	0.4337803	-0.02706626	0.77265137	0.91321261	13280.277024047194589
NONHSAG026052	0.01606471	0.818093874	0.938128871	-0.02716157	0.572885847	0.833292346	13290.849539501470023
NONHSAG026094	0.164674669	0.021273206	0.119640511	-0.02718222	0.096891467	0.34270788	13300.0588835604755497
NONHSAG026202	-0.05446612	0.447691279	0.751820679	-0.02723863	0.162437124	0.456747487	13310.371024377876998
NONHSAG026216	0.002202592	0.980428869	0.993501254	-0.0273236	0.136416728	0.422006425	13320.231881627186506
NONHSAG026311	0.395947792	2.22E-08	5.34E-07	-0.02753822	7.40E-09	2.45E-07	13332.91978022382025e-09
NONHSAG026360	-0.00229579	0.97588065	0.992468856	-0.02763436	0.273872094	0.603068356	13340.437302187485293
NONHSAG026376	-0.06994452	0.346521344	0.677284107	-0.02776936	0.371055993	0.69793975	13350.19193327933653
NONHSAG026396	-0.16313584	0.092724253	0.353786429	-0.02781055	0.009847586	0.065775323	13360.0317247839323156
NONHSAG026410	-0.09755557	0.249361035	0.584860584	-0.02783084	0.347065191	0.680102659	13370.118853279634888
NONHSAG026448	0.092545457	0.067832042	0.283097712	-0.02790659	0.56103611	0.826418828	13380.17079091381059
NONHSAG026475	-0.00522231	0.929539622	0.973506673	-0.027937	0.143591004	0.430061982	13390.257995503069607

NONHSAG026539	-0.11439707	0.03126434	0.161280896	-0.02795551	0.050399258	0.223890543	13400.0594529554064931
NONHSAG026542	0.044847099	0.628106774	0.85822947	-0.02797723	0.043103479	0.201675511	13410.104356507455693
NONHSAG026552	0.342762699	2.97E-05	0.000392617	-0.02800853	0.000254668	0.002853098	13424.87317871600875e-05
NONHSAG026569	-0.04403148	0.462019059	0.760209241	-0.02805624	0.724158248	0.902489903	13430.759918797474656
NONHSAG026592	-0.02911515	0.726930584	0.903322681	-0.02820649	0.51783932	0.804430331	13440.598781860851211
NONHSAG026607	0.081924449	0.198302129	0.528551011	-0.02830486	0.532928223	0.810050899	13450.431857212926672
NONHSAG026618	0.086501726	0.229320967	0.560252441	-0.02834478	0.313310376	0.643086698	13460.43141476294712
NONHSAG026620	-0.00910502	0.870022629	0.951960393	-0.02835581	0.440932302	0.752824306	13470.605579960134104
NONHSAG026634	-0.01207089	0.894918917	0.961169104	-0.02840994	0.905280545	0.964260944	13480.989314181154169
NONHSAG026661	0.188750505	0.012756566	0.079559806	-0.02850488	0.878341943	0.954734265	13490.0135071579287756
NONHSAG026676	-0.01122876	0.840230568	0.945079519	-0.02864077	0.932691641	0.973855106	13500.957157941161968
NONHSAG026716	0.023386289	0.595033506	0.845494805	-0.02870049	0.450414267	0.757258351	13510.737421558866087
NONHSAG026725	0.017969054	0.811629858	0.93441384	-0.02881917	0.823699387	0.933749876	13520.897959168677266
NONHSAG026729	-0.02773466	0.776471883	0.920117545	-0.02882492	0.523508969	0.80637524	13530.812807760350138
NONHSAG026751	0.07832885	0.282828838	0.619403967	-0.028845	0.718751957	0.902290403	13540.545315200017239
NONHSAG026771	-0.01101087	0.900196727	0.962481645	-0.02886937	0.78021716	0.917211812	13550.961150524517515
NONHSAG026775	0.001412874	0.986645726	0.996279605	-0.02898312	0.522057546	0.80637524	13560.763669465981081
NONHSAG026798	-0.02394923	0.703061406	0.895955243	-0.02904517	0.880613814	0.954988833	13570.859135314380091
NONHSAG026834	0.10554568	0.241879484	0.575614548	-0.02917911	0.254791317	0.586311933	13580.4081153576174
NONHSAG026851	-0.10261924	0.138923565	0.443294502	-0.02933671	0.216474534	0.532569019	13590.281609632306131
NONHSAG026854	-0.13904891	0.058982333	0.256746627	-0.0295822	0.788325445	0.921351268	13600.121857449787423
NONHSAG026890	-0.01713513	0.715575073	0.898703486	-0.02967476	0.42714906	0.744664077	13610.725735891649423
NONHSAG026912	0.053290908	0.542602622	0.817035827	-0.0296983	0.429798219	0.746421851	13620.707139845600947
NONHSAG026940	0.49633403	2.67E-07	5.07E-06	-0.02970231	6.49E-07	1.43E-05	13631.34924720991973e-07
NONHSAG026942	0.07366734	0.415518484	0.724427994	-0.0297773	0.173119869	0.474014675	13640.386134645181269
NONHSAG026944	-0.03224118	0.665474642	0.879303057	-0.02982308	0.685251005	0.889213579	13650.887897307000023
NONHSAG026956	-0.17410595	0.108283113	0.385433057	-0.02983451	0.951267113	0.979481187	13660.167531023404619
NONHSAG026967	0.055715061	0.54907253	0.820817175	-0.02983712	0.219926064	0.537299819	13670.466069264935977
NONHSAG026969	-0.03579756	0.573330534	0.836206153	-0.02989959	0.329333181	0.663384602	13680.615016820393959
NONHSAG026973	0.071130911	0.319815355	0.65884306	-0.02995078	0.103483381	0.353149595	13690.255908773184086
NONHSAG026985	-0.05371331	0.175141017	0.498983322	-0.0300951	0.86865823	0.95008438	13700.330904130159139
NONHSAG026995	-0.14856017	0.04599729	0.214857771	-0.03015917	0.024618841	0.133131118	13710.0489891538726424
NONHSAG027017	-0.06125586	0.440441312	0.743409945	-0.03019628	0.41414909	0.733427936	13720.653045742025678
NONHSAG027018	-0.05465289	0.574584764	0.836650451	-0.03021368	0.508526074	0.802059678	13730.773160315205799
NONHSAG027025	-0.04270208	0.511397513	0.796980196	-0.03025183	0.669868258	0.880630922	13740.798480967102678
NONHSAG027026	0.092962669	0.303118023	0.641397052	-0.03025679	0.28741382	0.616551056	13750.47820246834266
NONHSAG027036	-0.16212483	0.075161727	0.302839507	-0.03035344	0.368369709	0.695672009	13760.200725375148369
NONHSAG027063	-0.06944303	0.174225488	0.498394784	-0.03036665	0.388385248	0.712419647	13770.088645035356224
NONHSAG027074	-0.09030766	0.308781829	0.647015279	-0.03073072	0.648260436	0.871371102	13780.590139879788561
NONHSAG027078	-0.03853882	0.641246718	0.867750612	-0.03101434	0.754859765	0.907341414	13790.734057264807799
NONHSAG027116	0.169874935	0.013812609	0.084668093	-0.0310864	0.026202542	0.139589596	13800.0259312319260487
NONHSAG027134	0.091221017	0.181845238	0.508299015	-0.03134839	0.062936473	0.261028558	13810.157104228549185
NONHSAG027135	-0.11352051	0.060520198	0.261417505	-0.03134945	0.914550544	0.968016258	13820.0843757322066931
NONHSAG027140	-0.09723828	0.228436243	0.559166483	-0.03137848	0.689936621	0.891590891	13830.465802223839092
NONHSAG027170	0.106912872	0.255029284	0.59072681	-0.0314952	0.828780818	0.933749876	13840.476934791665884
NONHSAG027173	-0.05213479	0.386522683	0.706496807	-0.03157852	0.844564471	0.941306101	13850.658284681613202
NONHSAG027186	-0.07349708	0.432698486	0.737423118	-0.03164691	0.234114282	0.557433838	13860.4768011959493
NONHSAG027205	0.021361513	0.751218432	0.910732246	-0.03174158	0.297497959	0.62710964	13870.560620364354732
NONHSAG027215	0.058309998	0.331714182	0.669461779	-0.03175651	0.299462072	0.628892716	13880.506183999996804
NONHSAG027327	-0.10472531	0.078250347	0.312336247	-0.0317784	0.003220589	0.025582755	13890.0119381848419814
NONHSAG027345	0.010649562	0.842453731	0.945700556	-0.03177869	0.577929495	0.833831576	13900.733994235727495
NONHSAG027346	-0.01839227	0.752692366	0.910732246	-0.03209254	0.949765085	0.979141197	13910.945167309689525
NONHSAG027354	-0.23716955	0.001369936	0.012031279	-0.03209297	0.032109978	0.159797898	13920.00491834382429287
NONHSAG027362	-0.02701272	0.665505496	0.879303057	-0.03210749	0.732922672	0.905644804	13930.900045237440132
NONHSAG027390	0.081418833	0.220021657	0.553399732	-0.03214855	0.237550242	0.562755924	13940.377461286929931
NONHSAG027419	0.188163682	0.120872165	0.411133305	-0.03217535	0.266465659	0.59714881	13950.275398639639527
NONHSAG027464	0.049427939	0.338098932	0.673579125	-0.03217626	0.111996608	0.369410385	13960.273623883799181
NONHSAG027468	-0.08047061	0.262541409	0.6018673	-0.03226727	0.909758294	0.965738136	13970.463322307945156
NONHSAG027505	0.02917701	0.536926952	0.813923495	-0.0323138	0.448468957	0.756417348	13980.719499984092504
NONHSAG027515	-0.13260769	0.045067574	0.212111605	-0.03244781	0.147771095	0.432826692	13990.116293607594741
NONHSAG027519	0.178339372	0.018122051	0.105944298	-0.0325333	0.008571473	0.05904542	14000.0159729951655584
NONHSAG027535	-0.18784721	0.034665169	0.173449209	-0.03273327	0.021029539	0.118828797	14010.038739353285838
NONHSAG027545	-0.10102297	0.151021703	0.461203041	-0.03273747	0.282422122	0.613093982	14020.324381773463385
NONHSAG027550	0.007393372	0.89485042	0.961169104	-0.03279145	0.101735464	0.350159272	14030.189381244766269
NONHSAG027551	-0.06997996	0.253082087	0.588154405	-0.03279879	0.882132374	0.956266861	14040.367301347343791
NONHSAG027552	0.025676416	0.743176542	0.909586883	-0.03288074	0.632972408	0.860695556	14050.718659869534966
NONHSAG027560	-0.05273063	0.538758342	0.815757271	-0.03302229	0.556266891	0.822841924	14060.485313484016274
NONHSAG027603	-0.14110652	0.079601359	0.314291456	-0.03314915	0.359399978	0.688441919	14070.210389772918175
NONHSAG027626	0.167317709	0.076516429	0.306501706	-0.03317234	0.00268126	0.021917741	14080.0101320903310342
NONHSAG027634	0.015758166	0.755644797	0.912354301	-0.03327368	0.257555766	0.589777537	14090.499542470371268
NONHSAG027639	-0.01706122	0.745040469	0.909586883	-0.03328647	0.160760601	0.45587637	14100.335396989248657

NONHSAG027647	-0.26160773	0.011213202	0.072653279	-0.03330061	0.005276972	0.039049594	14110.00924411971434888
NONHSAG027670	-0.05013311	0.349332825	0.677785637	-0.03338207	0.051505193	0.227724217	14120.145885626599148
NONHSAG027674	0.102967123	0.166130516	0.488660054	-0.03345038	0.890978386	0.958708067	14130.242376419423887
NONHSAG027684	-0.05048544	0.527765095	0.808760462	-0.03348909	0.302028016	0.631451882	14140.577741111436193
NONHSAG027686	0.159710755	0.093968705	0.355119277	-0.03373529	0.486770548	0.785762791	14150.237447735511394
NONHSAG027687	-0.01940229	0.85636447	0.950687167	-0.03381285	0.530402009	0.808158244	14160.695512377315837
NONHSAG027711	-0.02375871	0.761097179	0.915008665	-0.03384257	0.945969945	0.976888536	14170.923612298302434
NONHSAG027714	0.009601062	0.901463522	0.962603929	-0.03393453	0.436259854	0.750543033	14180.70135064022194
NONHSAG027715	0.115643817	0.194526098	0.526251063	-0.03396953	0.315110635	0.645013248	14190.392723738160555
NONHSAG027736	0.057591426	0.428368609	0.73236115	-0.0340453	0.905778663	0.964426202	14200.690815918790382
NONHSAG027755	-0.12268854	0.205398442	0.537710656	-0.03407401	0.891895634	0.958708067	14210.301727903681842
NONHSAG027769	0.192306438	0.012600373	0.079089841	-0.03408062	0.045197181	0.208010595	14220.0309126488282971
NONHSAG027811	0.303233933	0.002567505	0.020569302	-0.03434578	0.000258861	0.002888565	14230.000597153732513255
NONHSAG027819	0.068355198	0.452717252	0.755078366	-0.03435451	0.335666617	0.671076968	14240.595840281203511
NONHSAG027821	-0.0301647	0.588285081	0.842139475	-0.03438592	0.884509232	0.956890351	14250.767547079956776
NONHSAG027833	-0.00046015	0.992768626	0.996592142	-0.03440916	0.444088885	0.755461551	14260.670583176196397
NONHSAG027841	0.1080655	0.22291885	0.556209235	-0.03440988	0.249672021	0.577841748	14270.388448362249284
NONHSAG027868	0.166165475	0.002714411	0.021623017	-0.03443884	0.000161881	0.001873294	14280.000429278060259276
NONHSAG027869	0.232145619	0.010173983	0.067315862	-0.03456588	0.048820383	0.222140643	14290.0272233338917815
NONHSAG027897	-0.02609988	0.692473992	0.89238585	-0.0346756	0.0560229	0.239714801	14300.0513412299872539
NONHSAG027917	0.094116983	0.330273911	0.669162012	-0.03486237	0.470580642	0.778395744	14310.596472814276422
NONHSAG027919	0.016838735	0.840965711	0.945162102	-0.03495913	0.14027357	0.425971144	14320.274465633365419
NONHSAG027954	0.022683773	0.712838512	0.898275053	-0.03510111	0.430648613	0.746861445	14330.497245120942323
NONHSAG027959	-0.06816354	0.205087645	0.53747107	-0.0351768	0.981726782	0.992670158	14340.333998901430163
NONHSAG027967	0.127149818	0.06186027	0.265169329	-0.03523646	0.226443082	0.548428253	14350.163487621004859
NONHSAG027970	0.05859022	0.351752478	0.679813037	-0.03525173	0.984390399	0.994290877	14360.550912256651542
NONHSAG027998	-0.02064421	0.695946717	0.89238585	-0.03530658	0.465795762	0.77504005	14370.763229203824981
NONHSAG028005	-0.01507231	0.772457941	0.918276503	-0.03540766	0.284877733	0.615265887	14380.537516077679102
NONHSAG028023	0.027548681	0.763988257	0.915083941	-0.03541218	0.052067	0.229486525	14390.11008767957172
NONHSAG028024	-0.00428411	0.95075787	0.981209884	-0.03541945	0.126031089	0.401707133	14400.221271243765
NONHSAG028072	0.088403568	0.321358302	0.660087322	-0.03543231	0.657462879	0.877865914	14410.341567186199736
NONHSAG028118	0.091283896	0.169910214	0.493072777	-0.03545666	0.968460418	0.987422297	14420.273048946669574
NONHSAG028155	0.034690463	0.556964208	0.823440249	-0.0355511	0.195610398	0.504065232	14430.426474017410137
NONHSAG028160	0.428285382	1.28E-08	3.36E-07	-0.03558857	3.47E-06	6.18E-05	14443.24847315289203e-08
NONHSAG028184	-0.02606762	0.741614463	0.909586883	-0.03564046	0.638338596	0.86506416	14450.72215877132867
NONHSAG028242	-0.52496414	1.20E-08	3.17E-07	-0.03571075	0.002278591	0.018956797	14466.80207328950412e-08
NONHSAG028243	-0.1262951	0.057731303	0.252866704	-0.03571372	0.268719256	0.599281359	14470.159049110238659
NONHSAG028249	-0.10641839	0.020735822	0.116851968	-0.03573349	0.174697693	0.475556547	14480.0652434779713686
NONHSAG028283	0.085987891	0.338885308	0.673579125	-0.03583121	0.472208754	0.778445171	14490.605421836332264
NONHSAG028291	0.042683617	0.59682278	0.846326605	-0.03584841	0.417686748	0.735463454	14500.709547063512727
NONHSAG028305	0.016552477	0.837888315	0.944345467	-0.0358662	0.773441108	0.91321261	14510.883505335013352
NONHSAG028340	-0.12711419	0.104180058	0.377032592	-0.03645071	0.051881128	0.229026265	14520.113430519859897
NONHSAG028349	-0.09686741	0.21047307	0.544124897	-0.03652433	0.146922147	0.432159398	14530.287961381303252
NONHSAG028408	0.092750353	0.186050886	0.515249135	-0.03655093	0.971152323	0.989449396	14540.318900573842124
NONHSAG028420	-0.04709818	0.405226999	0.715315958	-0.03700065	0.385830913	0.710980686	14550.241386042714059
NONHSAG028439	-0.04596597	0.618062786	0.854890583	-0.03703057	0.585524731	0.836463428	14560.831014676290008
NONHSAG028464	0.069941865	0.339572657	0.673579125	-0.0370551	0.5531983	0.82089373	14570.624328789844173
NONHSAG028465	-0.33887276	0.000540976	0.005372764	-0.03710563	0.013450502	0.084237891	14580.00184199259531908
NONHSAG028467	0.021654555	0.825136421	0.938461188	-0.03713061	0.05146516	0.227724217	14590.101430796868606
NONHSAG028469	-0.03000163	0.527352892	0.808569429	-0.03719048	0.167054497	0.463728771	14600.378683571252636
NONHSAG028525	0.267636528	0.028659481	0.150636375	-0.03747641	0.033442558	0.164694347	14610.0457998373730285
NONHSAG028576	0.131554515	0.092173648	0.35216345	-0.03750037	0.865951162	0.949709309	14620.176767485107361
NONHSAG028585	0.232275475	3.63E-06	5.73E-05	-0.03759425	4.82E-06	8.36E-05	14631.79971635188491e-06
NONHSAG028593	2.29242848	8.14E-15	6.54E-13	-0.03767138	7.55E-14	8.49E-12	14642.30339453615406e-15
NONHSAG028595	-0.50961582	8.44E-06	0.000121088	-0.03768609	0.000538266	0.005453992	14652.51602437438109e-05
NONHSAG028611	0.066448853	0.221983199	0.555352986	-0.03788469	0.551347167	0.820491224	14660.469227657853316
NONHSAG028627	0.872279129	7.69E-14	5.41E-12	-0.03789205	6.35E-15	9.93E-13	14672.00314394632898e-15
NONHSAG028726	-0.10631417	0.098055364	0.363762114	-0.03803723	0.269173876	0.599281359	14680.238409206798533
NONHSAG028734	0.045210453	0.514365141	0.799108873	-0.03806179	0.490844234	0.789462021	14690.408591724641236
NONHSAG028743	0.050470348	0.452724796	0.755078366	-0.03819551	0.55169133	0.820491224	14700.727562896232913
NONHSAG028794	-0.07666236	0.3620165	0.689228435	-0.03820435	0.087008673	0.31941043	14710.226178831614859
NONHSAG028929	-0.0549929	0.420839561	0.726032559	-0.03824194	0.18158967	0.485903158	14720.103548202155364
NONHSAG028931	0.040180787	0.400881639	0.713919676	-0.0384033	0.738133344	0.906043339	14730.695605706325129
NONHSAG028939	1.22413289	8.80E-10	2.95E-08	-0.03855296	1.66E-07	3.88E-06	14741.613080835337e-09
NONHSAG028975	0.17971554	0.018805753	0.108142692	-0.03859288	0.000136118	0.001594852	14750.000597013476040059
NONHSAG028980	-0.47148974	3.09E-09	8.96E-08	-0.03864684	1.28E-07	3.04E-06	14763.63125133713121e-09
NONHSAG029002	1.001865213	1.86E-17	2.61E-15	-0.03869425	1.04E-15	2.24E-13	14778.95684754634734e-18
NONHSAG029008	-0.02656723	0.688038656	0.888321717	-0.03880211	0.32277499	0.654887998	14780.360502488946374
NONHSAG029029	0.530340166	3.09E-09	8.96E-08	-0.03886098	6.95E-06	0.0001164	14791.21152434992216e-08
NONHSAG029047	-0.03688998	0.604865659	0.848743629	-0.038963	0.583511119	0.836050856	14800.825129086323211
NONHSAG029080	0.086209937	0.340602289	0.673895726	-0.03897467	0.518359801	0.804430331	14810.619275431298941

NONHSAG029181	0.008068329	0.905777187	0.964289176	-0.03902008	0.403301324	0.724100325	14820.660729274989467
NONHSAG029187	0.320354073	2.40E-05	0.000326681	-0.0391303	0.003931684	0.029961774	14839.98547666754975e-05
NONHSAG029193	0.273906962	0.032113246	0.164114197	-0.0391621	0.002854276	0.023197178	14840.00897142735288418
NONHSAG029202	-0.26641561	2.75E-05	0.00036963	-0.03928624	5.54E-05	0.000727361	14852.21567074474219e-05
NONHSAG029206	0.131032766	0.071390188	0.292637332	-0.03934784	0.032164157	0.159797898	14860.0708326464572761
NONHSAG029225	0.019823016	0.816466816	0.937869561	-0.03937892	0.143226536	0.430061982	14870.285906686593385
NONHSAG029338	0.094500194	0.275742002	0.612684559	-0.03941215	0.328612475	0.662882554	14880.484266837086958
NONHSAG029343	0.002429228	0.971875354	0.990544942	-0.03945039	0.804839939	0.928305951	14890.953143051195832
NONHSAG029351	-0.1254561	0.14686568	0.454830717	-0.03945574	0.171451851	0.471283094	14900.0241607668241935
NONHSAG029390	0.027816707	0.695887408	0.89238585	-0.03948799	0.232716048	0.556934066	14910.471756729180563
NONHSAG029407	0.151193279	0.045221925	0.212111605	-0.03957453	0.598450253	0.839825518	14920.112419969914392
NONHSAG029451	-0.06446862	0.237255763	0.5711076	-0.03963265	0.422995798	0.740637724	14930.478797600703025
NONHSAG029458	0.017461015	0.891474474	0.958633355	-0.03977561	0.491308157	0.789462021	14940.67411687777695
NONHSAG029466	0.022019591	0.735192655	0.905538909	-0.03994769	0.255113541	0.586573408	14950.500075494238535
NONHSAG029469	0.153565544	0.053712018	0.240506678	-0.0402113	0.084272015	0.315098308	14960.105762214408178
NONHSAG029483	0.021512642	0.714657967	0.898703486	-0.04047291	0.155873384	0.445443044	14970.332522375798183
NONHSAG029505	0.154980417	0.026979653	0.144233432	-0.04052809	0.566387071	0.83038605	14980.06916161754665
NONHSAG029519	0.059693036	0.272114606	0.60896612	-0.04069804	0.352727465	0.684429948	14990.49346515393482
NONHSAG029550	0.034148156	0.517873786	0.800192523	-0.0408081	0.050226681	0.223890543	15000.13243944084237
NONHSAG029563	-0.0281948	0.758060264	0.913419123	-0.04086045	0.670539468	0.881101394	15010.760642269126141
NONHSAG029600	0.337847208	2.80E-05	0.000375388	-0.04092301	0.024168426	0.131964298	15020.000137104567120509
NONHSAG029615	-0.0001262	0.998469659	0.998469659	-0.04095229	0.362440498	0.691401321	15030.571321482994106
NONHSAG029629	-0.14721196	0.02356265	0.129158228	-0.04095765	0.08729181	0.319939562	15040.0614906134325154
NONHSAG029651	0.217232366	0.124177401	0.416194102	-0.04116257	0.834571922	0.936478948	15050.16394267603312
NONHSAG029656	0.184203635	0.028606072	0.150636375	-0.04128249	0.057479677	0.244158387	15060.0597227140824807
NONHSAG029691	0.211164101	0.108080882	0.385433057	-0.04139492	0.112859542	0.371182494	15070.182197494465809
NONHSAG029703	0.216501351	0.062226945	0.265930349	-0.04142164	0.310211562	0.639527063	15080.171032026099221
NONHSAG029707	0.077828991	0.262476985	0.6018673	-0.04146231	0.074301749	0.290997936	15090.193111289859602
NONHSAG029741	0.091733707	0.13107365	0.429207521	-0.04148889	0.446312908	0.756399448	15100.314701029530055
NONHSAG029752	-0.75760873	5.10E-11	2.27E-09	-0.04151667	3.29E-08	9.26E-07	15111.12457057693229e-10
NONHSAG029789	0.033336531	0.678578477	0.887517619	-0.04152834	0.099269617	0.347196721	15120.225170252816794
NONHSAG029815	-0.01261012	0.853780345	0.949662955	-0.04157977	0.010909214	0.072180492	15130.0107362114548634
NONHSAG029818	0.125990611	0.018464634	0.106836525	-0.04160831	0.030834904	0.155669209	15140.0337212793003997
NONHSAG029852	-0.07928947	0.411834486	0.722444525	-0.04162831	0.068764959	0.278225993	15150.0307862749651072
NONHSAG029854	0.846576858	7.10E-10	2.40E-08	-0.04166785	2.52E-10	1.18E-08	15167.30349698857396e-11
NONHSAG029904	-0.0188135	0.819361489	0.938128871	-0.0417404	0.771648794	0.91321261	15170.872278014091432
NONHSAG029916	0.003881353	0.962307368	0.985436387	-0.04175174	0.127009987	0.403430881	15180.22013617539562
NONHSAG029920	0.012951763	0.815365243	0.937160913	-0.04177669	0.591418268	0.839418789	15190.863319863564542
NONHSAG029991	-0.03743395	0.475435277	0.767024669	-0.04195899	0.94102773	0.976126639	15200.682292875208796
NONHSAG029995	0.081974501	0.342121131	0.675447672	-0.04200022	0.02621028	0.139589596	15210.0797712642839122
NONHSAG030001	0.00458789	0.914369986	0.967765982	-0.04211377	0.249414504	0.57772124	15220.441170753821404
NONHSAG030002	0.035150337	0.516319958	0.799108873	-0.04212264	0.185514561	0.490255938	15230.410984238029278
NONHSAG030016	-0.03424511	0.586906441	0.842139475	-0.04217846	0.81732131	0.932966992	15240.727053495397395
NONHSAG030028	-0.07531156	0.203938389	0.536459073	-0.04243317	0.659466262	0.878826774	15250.429704116388224
NONHSAG030049	0.01537966	0.834099762	0.94268326	-0.04255503	0.608266804	0.847327128	15260.756887792759368
NONHSAG030056	-0.05727267	0.368579346	0.697210149	-0.04261813	0.848719686	0.942623167	15270.506090226920229
NONHSAG030133	0.007467982	0.905233538	0.964289176	-0.04286204	0.768202246	0.913083825	15280.956381356096593
NONHSAG030140	-0.10308999	0.124053541	0.416194102	-0.04291559	0.136892431	0.422548316	15290.216056829449572
NONHSAG030166	-0.05582881	0.406364219	0.716440568	-0.04292399	0.926153538	0.971770056	15300.593829744043088
NONHSAG030168	0.034495684	0.517710005	0.800192523	-0.0430196	0.941400237	0.976126639	15310.72782148024476
NONHSAG030187	-0.06747955	0.13968679	0.443840966	-0.04305624	0.904822175	0.964137915	15320.205936398034389
NONHSAG030263	-0.08786539	0.062027964	0.265483462	-0.04307171	0.801538986	0.926398532	15330.12648140961057
NONHSAG030278	0.245973046	1.22E-05	0.000172734	-0.0430832	1.03E-07	2.57E-06	15342.43058399533854e-07
NONHSAG030325	-0.02013456	0.794066047	0.927815057	-0.04316607	0.778634396	0.915750268	15350.86186420730647
NONHSAG030326	0.145994508	0.014422221	0.087573347	-0.04319842	0.049702568	0.222908488	15360.0352904201770971
NONHSAG030351	-0.12168577	0.104965495	0.37869582	-0.04325151	0.729822831	0.904080089	15370.228299882123035
NONHSAG030358	0.033042344	0.652430291	0.872061952	-0.043252	0.681304929	0.887368903	15380.688443252772465
NONHSAG030374	0.067456132	0.313117989	0.651448843	-0.04343374	0.468340732	0.776975893	15390.578260222826291
NONHSAG030400	0.266139382	0.005956161	0.04229476	-0.04373229	0.019328336	0.112064496	15400.0129027371352371
NONHSAG030406	0.018720485	0.833096607	0.942580607	-0.04381254	0.121146999	0.390223783	15410.159227553762448
NONHSAG030459	0.104021183	0.093396213	0.354512009	-0.0438775	0.094102331	0.336361438	15420.153411572957196
NONHSAG030469	0.037401043	0.59238335	0.843549299	-0.04404919	0.562485002	0.826911648	15430.810045366300251
NONHSAG030483	-0.02979325	0.686258034	0.887517619	-0.04418753	0.533700068	0.810348052	15440.816973149639596
NONHSAG030489	-0.04533207	0.456528326	0.756971211	-0.04419998	0.696954001	0.895338645	15450.754772716890456
NONHSAG030493	-0.01055472	0.868725095	0.951960393	-0.04421905	0.344708973	0.678321646	15460.596840876405391
NONHSAG030495	0.077172659	0.172927426	0.497084073	-0.04424751	0.276118898	0.605180312	15470.350564845348408
NONHSAG030509	0.046332904	0.394248736	0.710658619	-0.04470172	0.222856762	0.54304438	15480.453419316596885
NONHSAG030613	0.04480003	0.553548664	0.821852872	-0.04487723	0.899185775	0.962240579	15490.742474814170939
NONHSAG030629	-0.10277123	0.132127731	0.430026829	-0.04489135	0.89091873	0.958708067	15500.190466652040081
NONHSAG030633	1.281048	6.30E-18	1.18E-15	-0.04489328	2.22E-15	3.97E-13	15515.72159308208841e-18
NONHSAG030634	0.718996924	8.05E-13	5.06E-11	-0.04489578	1.67E-11	1.14E-09	15524.03385989056507e-13

NONHSAG030637	-0.2123476	0.003448489	0.026350951	-0.04499473	0.016282415	0.097625058	15530.00818097895092356
NONHSAG030676	-0.0264079	0.655615723	0.872912601	-0.04516674	0.994607323	0.997026029	15540.876014476757603
NONHSAG030681	0.105539277	0.226925708	0.558671887	-0.04521317	0.327588437	0.662657946	15550.43540450055048
NONHSAG030683	0.198564979	0.015384931	0.092047713	-0.04525516	1.36E-05	0.00020193	15566.81505949958205e-05
NONHSAG030688	0.079142501	0.152925017	0.464390009	-0.04534172	0.070811598	0.282879495	15570.161309187257582
NONHSAG030757	0.09317777	0.313722165	0.651448843	-0.0454544	0.884359834	0.956890351	15580.548506460064925
NONHSAG030767	0.08201692	0.22611833	0.558671887	-0.04557429	0.672738356	0.881776357	15590.46482455055238
NONHSAG030783	0.018234358	0.814647886	0.936928366	-0.04570015	0.146451573	0.432159398	15600.190087505319371
NONHSAG030819	0.084592193	0.217030616	0.549316015	-0.04570294	0.240524077	0.567946237	15610.37697611639941
NONHSAG030850	0.084696536	0.127286049	0.423583871	-0.04579952	0.0844211	0.315098308	15620.167681176379374
NONHSAG030878	0.066370685	0.457897592	0.756971211	-0.04583401	0.523483914	0.80637524	15630.721139689177843
NONHSAG030888	0.135807841	0.07907168	0.312727937	-0.04585941	0.018704256	0.10934796	15640.0502370559271937
NONHSAG030933	-1.51772564	1.13E-17	1.87E-15	-0.04591367	1.52E-13	1.58E-11	15652.88591189318401e-17
NONHSAG030937	0.091392173	0.15685079	0.470217934	-0.04594639	0.066404611	0.272598199	15660.155824174900831
NONHSAG030940	0.169617552	0.052338301	0.236616241	-0.04595336	0.710115677	0.896707014	15670.117118473951383
NONHSAG030957	0.038170382	0.552079231	0.821852872	-0.04596825	0.165920738	0.462866185	15680.375075452795958
NONHSAG030971	-0.06203468	0.26415067	0.603311472	-0.04608215	0.818132786	0.933301986	15690.354077254838846
NONHSAG030992	0.042813153	0.469650193	0.764534973	-0.04614167	0.810131307	0.930212019	15700.759464450706474
NONHSAG031103	0.193932715	0.025772251	0.139006808	-0.04637988	0.003773969	0.028916624	15710.01039106438787
NONHSAG031137	0.024863899	0.763716624	0.915083941	-0.04641399	0.105315314	0.357200785	15720.121504221944659
NONHSAG031167	-0.6402587	1.68E-07	3.43E-06	-0.04651902	1.02E-05	0.000162751	15733.30171493209998e-07
NONHSAG031168	0.01928502	0.712191595	0.898275053	-0.04655214	0.229902143	0.553497283	15740.464159459090158
NONHSAG031178	0.112224158	0.118816278	0.409096864	-0.04677326	0.872760532	0.950957653	15750.226505618517974
NONHSAG031180	0.258390828	0.008826544	0.059664045	-0.04691138	0.161172207	0.45587637	15760.0308617752362313
NONHSAG031184	-0.0446162	0.575937732	0.836969976	-0.04708782	0.91223541	0.967390382	15770.770610209743479
NONHSAG031203	-0.01532519	0.824308158	0.938461188	-0.04725087	0.883656831	0.956890351	15780.932692271111007
NONHSAG031219	0.052345041	0.396513642	0.711548411	-0.04739882	0.420611399	0.738301656	15790.630355024514184
NONHSAG031232	-0.13136513	0.096049924	0.360018759	-0.0474757	0.902266947	0.962874632	15800.177130025755761
NONHSAG031237	0.008246692	0.908890461	0.96518126	-0.04757546	0.417477424	0.735463454	15810.676899464180026
NONHSAG031249	-0.02671712	0.66302094	0.87737171	-0.04760689	0.586097928	0.836463428	15820.845116884564344
NONHSAG031255	0.004291549	0.937849493	0.97675288	-0.04775878	0.314845099	0.645013248	15830.531319714714725
NONHSAG031276	0.163226403	0.031925436	0.163821764	-0.04785523	0.190521818	0.497257471	15840.0942776659485781
NONHSAG031296	-0.01625281	0.797870514	0.928647304	-0.0479935	0.200119163	0.507425687	15850.258646087660986
NONHSAG031312	0.085156928	0.242568719	0.575614548	-0.04807311	0.371000419	0.69793975	15860.470105199803527
NONHSAG031314	0.197723796	0.009287729	0.062481088	-0.04812663	0.049218639	0.222512561	15870.0255062674678259
NONHSAG031315	0.26442297	0.010098841	0.06724594	-0.04818191	0.139322334	0.424918009	15880.0344499418411853
NONHSAG031322	0.016649892	0.867564434	0.951960393	-0.04825469	0.884749258	0.956890351	15890.95189060613753
NONHSAG031335	0.066845586	0.488243651	0.776180972	-0.04829314	0.596632653	0.839825518	15900.766552819110557
NONHSAG031372	0.05479931	0.242369055	0.575614548	-0.04832386	0.272034318	0.601384043	15910.420426868047982
NONHSAG031377	-0.05592851	0.586703876	0.842139475	-0.04833772	0.696015048	0.895338645	15920.852714960989138
NONHSAG031385	0.055015615	0.482674067	0.77249828	-0.04837269	0.068102679	0.276341607	15930.179028489376723
NONHSAG031401	0.040856109	0.448893822	0.751820679	-0.04843142	0.793193905	0.922059223	15940.569348026954927
NONHSAG031461	-0.02535075	0.578804707	0.838968473	-0.04847848	0.219575362	0.53727159	15950.464400918746377
NONHSAG031476	0.046131831	0.476916935	0.768533192	-0.04854687	0.995247832	0.997026029	15960.712854548187497
NONHSAG031485	0.031080554	0.685419348	0.887517619	-0.04870728	0.726516552	0.902772848	15970.906815680227075
NONHSAG031489	0.150600877	0.110809246	0.391944152	-0.04885679	0.035189345	0.170902312	15980.08893129572407
NONHSAG031516	0.007849356	0.932234281	0.974514051	-0.04895023	0.701539328	0.895983652	15990.921117680857649
NONHSAG031596	-0.00139091	0.981706302	0.9940489	-0.0490008	0.663922216	0.880630922	16000.874065042055507
NONHSAG031603	0.121513842	0.04121815	0.199291129	-0.04909297	0.609146889	0.847327128	16010.101576882933158
NONHSAG031617	0.000202209	0.996996397	0.997706003	-0.04910843	0.838644954	0.939549646	16020.972850035446805
NONHSAG031626	-0.01302489	0.870859893	0.952472597	-0.04918009	0.14462654	0.430061982	16030.203811870012069
NONHSAG031665	0.215257626	0.000542626	0.005372764	-0.04924595	8.12E-05	0.001024245	16040.000136246828617701
NONHSAG031701	-0.1150297	0.083795471	0.325459758	-0.04926328	0.137309294	0.423288596	16050.172424343609002
NONHSAG031748	-0.10406268	0.264709866	0.603701657	-0.04942348	0.620338481	0.853798275	16060.530597313242563
NONHSAG031783	0.252330762	4.57E-05	0.000584243	-0.04948911	3.37E-06	6.08E-05	16075.13260390220697e-06
NONHSAG031784	-0.03027722	0.67246146	0.88445352	-0.04956082	0.550021215	0.819205327	16080.590038517064693
NONHSAG031801	-0.01174672	0.879459813	0.954268263	-0.0496119	0.572821444	0.833292346	16090.841346851522602
NONHSAG031805	0.026160615	0.652545177	0.872061952	-0.04969575	0.101889626	0.35026116	16100.0943388518964268
NONHSAG031818	0.193561397	0.044361406	0.21036134	-0.04977473	0.234093775	0.557433838	16110.126589198781886
NONHSAG031824	0.147683425	0.057896149	0.253194355	-0.0498294	0.569965969	0.832594444	16120.146160317094016
NONHSAG031831	0.103890842	0.069233024	0.287014087	-0.04996394	0.010978381	0.072467623	16130.0322076822769936
NONHSAG031845	-0.44007088	5.71E-06	8.59E-05	-0.04997445	7.97E-05	0.001015509	16149.36855322865596e-06
NONHSAG031890	0.065841478	0.352849523	0.680247895	-0.04999026	0.520219694	0.805982248	16150.290690362961159
NONHSAG031913	0.073511807	0.192685682	0.525491668	-0.05019145	0.720786607	0.902489903	16160.398882517089473
NONHSAG031945	0.501474563	5.61E-06	8.49E-05	-0.05031038	3.38E-05	0.000468329	16176.33931308661718e-06
NONHSAG031951	-0.33877767	0.000104214	0.001205963	-0.05031046	0.001126787	0.010354656	16180.000221533007086511
NONHSAG031952	0.020762882	0.758150865	0.913419123	-0.05031315	0.724060359	0.902489903	16190.801826562809856
NONHSAG031964	0.08460197	0.123813808	0.416194102	-0.05033457	0.337381726	0.671160358	16200.294578666663226
NONHSAG032009	-0.12721954	0.056869723	0.251067337	-0.05036349	0.016529102	0.098893265	16210.0409207906394744
NONHSAG032015	-0.03690375	0.550590046	0.820922168	-0.0503877	0.674502469	0.881776357	16220.826521719966425
NONHSAG032104	0.050772648	0.344785476	0.677284107	-0.05046331	0.575720042	0.833526329	16230.632876776647484

NONHSAG032106	-0.08743521	0.276021189	0.612684559	-0.05074559	0.198651513	0.505936125	16240.380223735589818
NONHSAG032135	0.149827207	0.069508034	0.287014087	-0.05096476	0.002021707	0.017227391	16250.00780647383994292
NONHSAG032143	0.098603098	0.083793293	0.325459758	-0.05096898	0.105372101	0.357200785	16260.15301109815709
NONHSAG032153	0.008229275	0.91969148	0.968611494	-0.05097518	0.365458407	0.69386861	16270.607672144975106
NONHSAG032186	-0.32868201	8.34E-06	0.000120821	-0.05107944	1.55E-05	0.000227728	16285.51615473863151e-06
NONHSAG032194	0.032732931	0.589600548	0.842139475	-0.05116034	0.735704034	0.906043339	16290.860166832558696
NONHSAG032197	-0.10143572	0.128318733	0.42450856	-0.05118573	0.679652158	0.885626445	16300.285206811635668
NONHSAG032235	0.055896418	0.488695903	0.776248729	-0.05124285	0.367637274	0.695222605	16310.637521735795441
NONHSAG032245	0.00775624	0.919698681	0.968611494	-0.05128968	0.957862948	0.981988142	16320.987716580347537
NONHSAG032250	-0.03701128	0.652650806	0.872061952	-0.0513716	0.203967236	0.512331603	16330.430786618593137
NONHSAG032270	-0.08537319	0.131112824	0.429207521	-0.05141032	0.815318692	0.931603479	16340.263063826462078
NONHSAG032285	0.078738605	0.288246786	0.62446068	-0.05142573	0.972621033	0.989451568	16350.456991358548088
NONHSAG032299	-0.01141988	0.879980144	0.954268263	-0.05150952	0.442335077	0.753473247	16360.61271759222031
NONHSAG032337	0.131397255	0.272036378	0.60896612	-0.05153672	0.972834907	0.989451568	16370.454970882166105
NONHSAG032387	-0.04280474	0.541184027	0.817035827	-0.05155361	0.462261197	0.771493351	16380.730839080039954
NONHSAG032422	0.411245538	0.000384664	0.003890915	-0.05163387	0.000318337	0.003414264	16390.000274586756767902
NONHSAG032427	-0.07777244	0.383638062	0.706496807	-0.0516634	0.895951281	0.960875287	16400.550088622389339
NONHSAG032431	0.20193535	0.010774369	0.07013316	-0.05180692	0.054510524	0.236184275	16410.0294919270248582
NONHSAG032480	-0.05053161	0.477998284	0.769393918	-0.05190989	0.44699987	0.756417348	16420.693731679523247
NONHSAG032559	0.005103544	0.940186837	0.977659054	-0.05198738	0.040906901	0.193653543	16430.0682522079680911
NONHSAG032592	0.068728727	0.286133385	0.623726417	-0.0520345	0.88039425	0.954988833	16440.509235510901433
NONHSAG032593	0.019896091	0.803231116	0.93227155	-0.05207834	0.697890823	0.895443732	16450.81188549164295
NONHSAG032601	0.024097056	0.678757393	0.887517619	-0.05218716	0.137724926	0.423288596	16460.304141185862685
NONHSAG032608	0.000844149	0.99167376	0.996279605	-0.05240993	0.546906716	0.8161186	16470.785971662965949
NONHSAG032613	0.131281798	0.126548563	0.422771134	-0.05241269	0.004871988	0.036533417	16480.017935569705031
NONHSAG032620	-0.21145566	0.02222187	0.123238983	-0.05244248	0.010622199	0.070447223	16490.0202369562678326
NONHSAG032629	0.042816396	0.482055608	0.771947819	-0.05262719	0.974164042	0.989451568	16500.726924332911316
NONHSAG032632	-0.03278881	0.554136929	0.821852872	-0.05263936	0.374077016	0.700534004	16510.332380728116499
NONHSAG032643	-0.82144479	6.93E-12	3.55E-10	-0.0527088	1.47E-09	5.89E-08	16529.52312601554015e-12
NONHSAG032649	0.010216113	0.896778938	0.961169104	-0.05294112	0.253894184	0.58512763	16530.374756761801951
NONHSAG032662	-0.02284379	0.728241116	0.903322681	-0.05294608	0.146619954	0.432159398	16540.165017165349136
NONHSAG032723	0.173217746	0.003508622	0.026737792	-0.0529709	0.02145874	0.120683955	16550.00921622684869068
NONHSAG032740	-0.0335172	0.682928868	0.887517619	-0.05307637	0.431720996	0.747536601	16560.478271266003163
NONHSAG032746	-0.08935072	0.47105524	0.764534973	-0.05316032	0.377642557	0.704665475	16570.279989999828759
NONHSAG032750	0.001039783	0.987176825	0.996279605	-0.05322154	0.79820394	0.924443772	16580.959262204085432
NONHSAG032785	0.011575089	0.879844943	0.954268263	-0.05331457	0.222027727	0.541493468	16590.408052033255224
NONHSAG032791	0.065312849	0.42494422	0.729325831	-0.0533511	0.800165235	0.925568407	16600.713693850937978
NONHSAG032815	0.020964068	0.712514686	0.898275053	-0.0534296	0.717670349	0.902031633	16610.913867046791266
NONHSAG032817	-0.03910418	0.588653823	0.842139475	-0.05344073	0.645695892	0.869586613	16620.841658814814719
NONHSAG032839	-0.01724154	0.833746673	0.94268326	-0.05369462	0.161973495	0.456342619	16630.316518601291693
NONHSAG032855	0.00395167	0.959667365	0.98403948	-0.05385414	0.892907691	0.958708067	16640.981588182210758
NONHSAG032935	2.799354869	3.68E-26	1.03E-22	-0.05403162	8.31E-25	2.34E-21	16658.62416094784045e-27
NONHSAG032949	-0.00228588	0.980330588	0.993501254	-0.05414134	0.603450961	0.843391701	16660.825954465589773
NONHSAG032951	-0.06305792	0.402336904	0.714263509	-0.05415832	0.410066861	0.730277398	16670.256754222965169
NONHSAG032956	-0.72911694	5.61E-06	8.49E-05	-0.05419833	0.000360062	0.003769147	16681.60798372179397e-05
NONHSAG032959	-0.03586434	0.58882869	0.842139475	-0.05422824	0.741510816	0.906043339	16690.677946322268888
NONHSAG032960	0.089812688	0.1850129	0.513073248	-0.0544234	0.095001209	0.337302272	16700.209478420409365
NONHSAG032961	0.180135798	0.0035262	0.026799121	-0.05448001	0.544350576	0.815012106	16710.00837521852276308
NONHSAG032979	0.112910125	0.051676221	0.233999248	-0.05455578	0.155818594	0.445443044	16720.130418877673509
NONHSAG032984	0.025868952	0.645633298	0.87033597	-0.0546103	0.758793127	0.909127255	16730.740535012281532
NONHSAG032988	-0.00411092	0.965044148	0.986974762	-0.05467406	0.914658321	0.968016258	16740.99412840450246
NONHSAG033011	0.09911267	0.120783797	0.411133305	-0.05468826	0.046197367	0.211920057	16750.110951448231397
NONHSAG033026	-0.08333394	0.353187029	0.680247895	-0.05474042	0.922606416	0.971770056	16760.59025586710934
NONHSAG033060	0.019238088	0.831255606	0.942536598	-0.05503234	0.64440534	0.869097274	16770.78646628445846
NONHSAG033068	-0.02658833	0.675341439	0.886996789	-0.05503586	0.529745858	0.808158244	16780.812584817211786
NONHSAG033071	0.167786612	0.01841567	0.106772915	-0.05507198	0.092510089	0.3330837	16790.0520769126758972
NONHSAG033082	0.2433794	0.021406215	0.119908916	-0.05514769	0.005935199	0.043350075	16800.0134460959454708
NONHSAG033092	-0.05493871	0.387352163	0.706496807	-0.05532697	0.702271669	0.896028889	16810.683349023319822
NONHSAG033099	0.281190501	0.000210583	0.002304121	-0.05534377	1.22E-05	0.000188651	16822.39331720868794e-05
NONHSAG033167	0.117821956	0.081691754	0.320060238	-0.05540323	0.268998279	0.599281359	16830.208365348813826
NONHSAG033226	-0.12901025	0.156127551	0.469551521	-0.05542769	0.159174638	0.452577433	16840.262477989920872
NONHSAG033228	0.070407331	0.398652269	0.712889467	-0.05572269	0.077244208	0.297141877	16850.204930906134314
NONHSAG033247	0.029705486	0.691706426	0.892237831	-0.05577638	0.203573056	0.512331603	16860.423627832119264
NONHSAG033254	0.039819442	0.651962578	0.872061952	-0.05577724	0.727618552	0.903339235	16870.892881007550131
NONHSAG033260	0.063907137	0.36987095	0.698354362	-0.05584313	0.100907603	0.349991359	16880.254761550927043
NONHSAG033274	0.049093295	0.588871488	0.842139475	-0.05592618	0.315166502	0.645013248	16890.598993359069895
NONHSAG033291	0.041159468	0.599408229	0.847002984	-0.05616491	0.227291061	0.549563598	16900.475288581966091
NONHSAG033405	0.068096938	0.23443808	0.567820743	-0.05616978	0.188955908	0.494733719	16910.346752700931048
NONHSAG033416	0.118841941	0.245215419	0.581404518	-0.0562117	0.655563787	0.876578873	16920.255130519438605
NONHSAG033484	0.277834676	0.005375351	0.039159293	-0.05625515	0.013435597	0.084237891	16930.0102083924420708
NONHSAG033545	0.011602252	0.874637739	0.953287334	-0.05628916	0.184442228	0.488933259	16940.345594282888028

NONHSAG033550	-0.05400661	0.523043563	0.803843214	-0.05631035	0.664276222	0.880630922	16950.806057527592317
NONHSAG033553	0.182846267	0.015162903	0.091302104	-0.05647296	9.01E-05	0.00110692	16960.000400699214164033
NONHSAG033558	0.11875194	0.096794362	0.360872845	-0.05654684	0.040685427	0.192929882	16970.0937332941115131
NONHSAG033585	0.069839291	0.226001253	0.558671887	-0.05657989	0.243246167	0.570085574	16980.38662576842053
NONHSAG033587	0.124792858	0.047056056	0.218120003	-0.05661997	0.008609041	0.05904542	16990.0237357669588844
NONHSAG033588	0.092062902	0.078836968	0.312727937	-0.05662404	0.823205427	0.933749876	17000.157861963046915
NONHSAG033592	-0.01809878	0.769772648	0.917667832	-0.05680288	0.265600022	0.596878743	17010.508852506552179
NONHSAG033593	-0.06497241	0.374207535	0.699183779	-0.05691515	0.719428149	0.902489903	17020.666636856985668
NONHSAG033604	0.032993305	0.652481276	0.872061952	-0.05698328	0.002140218	0.01796505	17030.0040929424881
NONHSAG033616	0.106696935	0.13028357	0.429206713	-0.05714086	0.570780049	0.833292346	17040.305312876581352
NONHSAG033639	-0.16543947	0.024819332	0.13421531	-0.05721038	0.003375835	0.026404896	17050.00942772743691016
NONHSAG033752	0.022929431	0.708396487	0.898275053	-0.05724941	0.262617912	0.5936347	17060.516771133651824
NONHSAG033788	-0.22445734	0.000320943	0.003330224	-0.05725961	2.10E-05	0.000301438	17074.19357513240457e-05
NONHSAG033805	0.022583213	0.742792232	0.909586883	-0.05727069	0.17953849	0.484025789	17080.370572413227508
NONHSAG033897	0.079508182	0.226447169	0.558671887	-0.0574077	0.995226205	0.997026029	17090.376124954932188
NONHSAG033898	0.082261268	0.294686614	0.632460859	-0.05777069	0.072337925	0.285694163	17100.192239520322984
NONHSAG033967	0.049871116	0.511679025	0.796980196	-0.05779963	0.953187055	0.980474941	17110.765539454662982
NONHSAG033980	0.0111486	0.88183862	0.954625582	-0.05782263	0.750042324	0.906696003	17120.949753087936929
NONHSAG033989	0.066046015	0.402598742	0.714263509	-0.05792749	0.42975248	0.746421851	17130.271057468179241
EP300-AS1	-1.68685587	7.05E-16	6.84E-14	-0.05822145	2.66E-12	2.02E-10	28061.4956599549388e-15
NONHSAG034032	-0.23835829	0.001410804	0.012271596	-0.0583425	0.055862864	0.239460937	17140.00553472904054257
NONHSAG034050	-0.12476299	0.143412681	0.448583379	-0.05834996	0.043618387	0.203745687	17150.11256978292359
NONHSAG034053	0.082977629	0.191302443	0.523025062	-0.05846053	0.034109612	0.167101446	17160.0995204624977302
NONHSAG034077	0.086844614	0.173416775	0.49759997	-0.05860676	0.919229722	0.970819932	17170.262739226854428
NONHSAG034081	0.147094513	0.041005004	0.198803571	-0.05870094	0.752405997	0.906886268	17180.0871068625240894
NONHSAG034105	-0.02394277	0.708327875	0.898275053	-0.05875494	0.281016772	0.612446652	17190.54439634898219
NONHSAG034111	0.038553966	0.571008149	0.83541879	-0.05879424	0.069743763	0.280973439	17200.174157708776969
NONHSAG034128	0.011004706	0.837785494	0.944345467	-0.05886083	0.166335658	0.463563797	17210.323427072639278
NONHSAG034158	-0.01639105	0.827578502	0.940126436	-0.05893303	0.612305346	0.849433959	17220.756538480964443
NONHSAG034163	-0.25145977	0.00368677	0.027943929	-0.05915981	0.016698083	0.099692164	17230.00866921363226134
NONHSAG034175	-0.05350508	0.354525183	0.680959573	-0.05922858	0.743532971	0.906684611	17240.639316108569113
NONHSAG034179	0.168724888	0.042956382	0.205430862	-0.05947473	0.020207585	0.115602803	17250.0417883853065924
NONHSAG034184	-0.13120551	0.085607104	0.330215606	-0.05954318	0.424411062	0.741866512	17260.22265295036551
NONHSAG034187	0.095432028	0.219720003	0.553135764	-0.05960335	0.053815251	0.23425462	17270.146081443252798
NONHSAG034193	0.142660595	0.050950895	0.231460286	-0.05962566	0.10944359	0.366375446	17280.114313801541714
NONHSAG034209	0.081653507	0.24073727	0.575151404	-0.05968276	0.231477192	0.55586154	17290.38933857110753
NONHSAG034242	0.108149447	0.199138863	0.528780437	-0.05971102	0.01966014	0.113520151	17300.063060352548296
NONHSAG034302	-0.11364672	0.212598636	0.544533663	-0.05973007	0.211121659	0.524663558	17310.351250948772395
NONHSAG034336	-0.05553684	0.33794429	0.673579125	-0.05977597	0.655559779	0.876578873	17320.627186149859638
NONHSAG034346	0.021714166	0.715264503	0.898703486	-0.06007688	0.96665024	0.986709541	17330.903594327578238
NONHSAG034364	0.041134546	0.549687263	0.820881882	-0.06016232	0.588258388	0.836713499	17340.521980153165204
NONHSAG034372	0.048020153	0.539383392	0.815893545	-0.06028544	0.00728369	0.05152359	17350.01833747091942
NONHSAG034414	0.114160531	0.233804146	0.567585494	-0.06046696	0.28848684	0.617112355	17360.422773339189106
NONHSAG034441	0.032480049	0.585177391	0.842139475	-0.06062861	0.211208801	0.524663558	17370.450489664188328
NONHSAG034453	0.015471265	0.809281947	0.93439361	-0.06065316	0.767566342	0.913028987	17380.863942945879226
NONHSAG034459	-0.02082476	0.788208213	0.924700277	-0.06069366	0.604994121	0.843870767	17390.873065790904966
NONHSAG034470	0.024463722	0.735741377	0.905538909	-0.06075215	0.710477677	0.896707014	17400.776035888522542
NONHSAG034501	-0.00415861	0.958892391	0.983730538	-0.06088366	0.718155337	0.902031633	17410.925621542668386
NONHSAG034507	-0.09599472	0.186292345	0.515249135	-0.06125204	0.269171884	0.599281359	17420.363130881238254
NONHSAG034552	-0.20333074	0.011037707	0.07168137	-0.06129261	0.108392656	0.365030117	17430.0359074122062634
NONHSAG034566	0.135619133	0.12393772	0.416194102	-0.06131098	0.079662931	0.303951373	17440.160009315413073
NONHSAG034569	-0.12634415	0.119928792	0.410767067	-0.06137007	0.085183132	0.316426641	17450.16415657738612
NONHSAG034584	-0.09425262	0.207625905	0.541599299	-0.06144382	0.370634061	0.69793975	17460.427430843320282
NONHSAG034600	0.075917018	0.293887361	0.631812889	-0.06161629	0.772112435	0.91321261	17470.550907215967936
NONHSAG034618	-0.05793406	0.52851837	0.80903302	-0.06164523	0.093817356	0.336361438	17480.233453013055716
NONHSAG034646	-0.19256832	0.003001176	0.02344252	-0.0619521	0.67917651	0.885416943	17490.00159445956886875
NONHSAG034655	0.013741809	0.79664056	0.927815057	-0.06195961	0.751718173	0.906886268	17500.846086798060984
NONHSAG034677	-0.02915276	0.634522974	0.862680714	-0.06201774	0.45349306	0.761326857	17510.466758539125582
NONHSAG034678	0.006446603	0.928951789	0.97333618	-0.06207386	0.836869603	0.938308342	17520.978602171598638
NONHSAG034689	-0.00674885	0.902799616	0.963247892	-0.06212116	0.778648254	0.915750268	17530.960501080652684
NONHSAG034701	0.076548508	0.128129758	0.42450856	-0.06216551	0.180046577	0.484025789	17540.249373841167244
NONHSAG034702	-0.07699087	0.295626281	0.632616096	-0.0623405	0.761973541	0.910423706	17550.555674086086356
NONHSAG034714	-0.15018629	0.153604457	0.464946969	-0.06236687	0.391418473	0.714719964	17560.351822048197881
NONHSAG034778	0.129094595	0.057402722	0.252036973	-0.06239075	0.05422195	0.235660159	17570.0878032530737017
NONHSAG034831	0.122734019	0.273646299	0.611679961	-0.06240934	0.448623688	0.756417348	17580.529301525782934
NONHSAG034858	0.086814937	0.268522594	0.605799463	-0.06257872	0.105432664	0.357200785	17590.250960395619742
NONHSAG034900	0.120266112	0.06380557	0.271029099	-0.06269163	0.746805626	0.906696003	17600.137877812163884
NONHSAG034902	0.091451062	0.195477162	0.526515116	-0.06273379	0.483210763	0.783450231	17610.426814768273475
NONHSAG034909	-0.04108679	0.530918109	0.810940643	-0.06293157	0.098214508	0.344363088	17620.0678515701381041
NONHSAG034919	-0.03231504	0.649662973	0.871589829	-0.06305353	0.117768204	0.382848775	17630.268739595824242
NONHSAG034946	0.03445798	0.721360336	0.9023422	-0.06312202	0.815103385	0.931603479	17640.836326751697394

NONHSAG034982	0.08187349	0.159071395	0.472842244	-0.06312246	0.04201767	0.197251564	17650.112656053360005
NONHSAG035114	0.048067755	0.542034412	0.817035827	-0.06317125	0.379831347	0.706453518	17660.663513693859557
NONHSAG035116	-0.00247948	0.967533294	0.987996581	-0.06335739	0.405151815	0.726122947	17670.612286814805546
NONHSAG035135	0.086164727	0.12726193	0.423583871	-0.06366629	0.03941968	0.188838397	17680.10088701265375
NONHSAG035143	0.071932623	0.223560546	0.556499409	-0.06372669	0.094408186	0.336361438	17690.221567973303362
NONHSAG035159	-0.02270134	0.835687827	0.943377828	-0.0641497	0.735449317	0.906043339	17700.857946517756971
NONHSAG035266	-0.05262614	0.356285305	0.682203965	-0.06422661	0.086127262	0.318252116	17710.224187988514905
NONHSAG035270	-0.1073661	0.142924821	0.447555229	-0.06426662	0.029478241	0.151264256	17720.0837367111648137
NONHSAG035294	0.124473403	0.101064427	0.369561988	-0.06430855	0.781295858	0.917391514	17730.119672799144918
PTPRG-AS1	-1.9578547	8.50E-21	4.78E-18	-0.06431194	3.63E-14	4.44E-12	28094.75982478382393e-20
NONHSAG035333	0.105626132	0.094083877	0.355119277	-0.06435699	0.319732285	0.651852579	17740.238086229732312
NONHSAG035347	0.048700536	0.511056067	0.796980196	-0.06438748	0.413564172	0.733255014	17750.683897721591478
NONHSAG035395	0.158203348	0.132008176	0.430026829	-0.06445023	0.005066909	0.037693515	17760.0186088676663641
NONHSAG035415	0.069112629	0.464880394	0.763571876	-0.06451902	0.530689836	0.808158244	17770.728694829292235
NONHSAG035432	0.11194678	0.118859224	0.409096864	-0.06454123	0.049190195	0.222512561	17780.115181130677913
NONHSAG035436	0.11450501	0.136656706	0.440181737	-0.06472672	0.184480152	0.488933259	17790.261513249579229
NONHSAG035491	-0.03136739	0.745704164	0.909586883	-0.06485976	0.341422158	0.674686654	17800.622081694397905
NONHSAG035503	-0.08930696	0.097287206	0.361867225	-0.06492455	0.321542259	0.65343573	17810.244424562524155
NONHSAG035505	0.08443675	0.334063981	0.670991367	-0.06507164	0.902902105	0.96318692	17820.571022443424707
NONHSAG035508	-0.0217652	0.652805978	0.872061952	-0.06512111	0.152522701	0.440342745	17830.149735062441436
NONHSAG035519	0.029716633	0.728709644	0.903322681	-0.06513268	0.994542904	0.997026029	17840.923360144247102
NONHSAG035522	0.017012846	0.857207539	0.950687167	-0.06520971	0.195065786	0.504065232	17850.273689020176894
NONHSAG035547	-0.05450207	0.521616577	0.803843214	-0.06522759	0.098866593	0.346217759	17860.24512773709448
NONHSAG035579	-0.03327084	0.475324975	0.767024669	-0.06530598	0.862258175	0.947877244	17870.754914289766032
NONHSAG035588	-0.43037057	0.001434602	0.012412622	-0.06531975	0.087380364	0.319939562	17880.00578879256321973
NONHSAG035621	-0.00248365	0.971481068	0.99050209	-0.06552399	0.712901205	0.897950883	17890.919892369582273
NONHSAG035667	0.117969175	0.098689883	0.36482008	-0.06557373	0.021735409	0.121510872	17900.0601685256174979
NONHSAG035675	0.16192008	0.18373938	0.511053548	-0.06581105	0.029121059	0.150253976	17910.0870136734224962
NONHSAG035700	-0.08710022	0.183281835	0.510285663	-0.06582778	0.325669342	0.659310432	17920.382122272618862
NONHSAG035708	0.033761142	0.602298671	0.848669165	-0.06606655	0.846215782	0.942026436	17930.868720213758828
NONHSAG035712	-0.03749504	0.551252458	0.821474251	-0.0660744	0.575706477	0.833526329	17940.797680114649864
NONHSAG035715	-0.02128925	0.682435979	0.887517619	-0.06612686	0.354375627	0.685825371	17950.393141005552868
NONHSAG035722	0.111345761	0.262543029	0.6018673	-0.06624729	0.36066061	0.689447747	17960.487575554116491
NONHSAG035731	-0.07162522	0.247749041	0.58356857	-0.06631261	0.137693485	0.423288596	17970.293660720032376
NONHSAG035768	-0.30034752	0.000420924	0.004242436	-0.06632197	0.082595836	0.31112995	17980.00180544395338356
NONHSAG035770	-0.24568121	0.042576116	0.204307232	-0.0664213	0.747438771	0.906696003	17990.0418801237797277
NONHSAG035786	0.380103641	1.08E-07	2.32E-06	-0.06657349	9.69E-07	2.02E-05	18009.31934028366847e-08
NONHSAG035791	0.271239858	0.005688569	0.041121483	-0.06665992	0.000187732	0.002145939	18010.000612833461225238
NONHSAG035807	-0.02352906	0.784316643	0.922802677	-0.0668462	0.915279825	0.968309582	18020.962114846894078
NONHSAG035911	-0.1116997	0.016144085	0.096180437	-0.06685653	0.000627038	0.006186773	18030.00218095258391336
NONHSAG035915	0.029572129	0.585468717	0.842139475	-0.06689224	0.934720815	0.973855106	18040.839113739618142
NONHSAG035927	0.073265195	0.26432423	0.603311472	-0.0671087	0.986747748	0.99566177	18050.427500199127272
NONHSAG035942	0.094547056	0.163409165	0.481663074	-0.06718216	0.694553088	0.895088581	18060.350702126385884
NONHSAG035943	-0.30072317	0.000258252	0.002782393	-0.06719335	0.001260506	0.011360715	18070.000440341286391523
NONHSAG035971	0.114804999	0.376236478	0.701111316	-0.06721681	0.744019164	0.906696003	18080.455456063761147
NONHSAG035976	-0.00666412	0.934346718	0.975411819	-0.06726117	0.379856942	0.706453518	18090.56456832238856
NONHSAG035982	-0.0897315	0.119124197	0.409507632	-0.0674186	0.703523898	0.896707014	18100.262252539873952
NONHSAG036030	0.118276888	0.106158467	0.380813624	-0.06742714	0.173964473	0.475047467	18110.219658717360854
NONHSAG036052	0.411557823	1.39E-07	2.88E-06	-0.06746169	4.70E-09	1.63E-07	18125.02115858401732e-09
NONHSAG036065	-0.00685374	0.906037646	0.964289176	-0.06760601	0.739203207	0.906043339	18130.895175474731255
NONHSAG036067	-0.33107987	0.000331823	0.003430462	-0.06799393	0.001109196	0.010226425	18140.000490437789783291
NONHSAG036098	0.080305132	0.339903548	0.673579125	-0.06810227	0.372147813	0.69917505	18150.56201420673478
NONHSAG036130	-0.7699359	1.62E-07	3.32E-06	-0.06810482	8.24E-05	0.001034497	18165.97325278698504e-07
NONHSAG036141	0.253421403	0.000631177	0.006120236	-0.06820976	0.000101415	0.001229223	18170.000169826203968775
NONHSAG036146	-0.05462108	0.421284816	0.726335318	-0.06833109	0.594214614	0.839418789	18180.712352795830376
NONHSAG036147	0.026662705	0.66967251	0.882022997	-0.06838019	0.588805238	0.837067911	18190.848254011172647
NONHSAG036184	0.024215076	0.735802961	0.905538909	-0.06847672	0.144678685	0.430061982	18200.306710172019033
NONHSAG036217	0.040531037	0.414826213	0.724427994	-0.06857251	0.609280033	0.847327128	18210.708803928721221
NONHSAG036227	0.64507012	1.82E-06	3.04E-05	-0.06865827	0.000192853	0.002195562	18225.42898356145204e-06
NONHSAG036242	0.085459248	0.252279673	0.587746844	-0.06873206	0.021060596	0.118828797	18230.0672296793309194
NONHSAG036289	0.052826659	0.388673741	0.706496807	-0.06883575	0.226626469	0.548428253	18240.456698887927342
NONHSAG036290	0.106392771	0.059703924	0.258686341	-0.06900493	0.305942695	0.635384681	18250.16519695122865
NONHSAG036298	-0.82988734	2.36E-11	1.13E-09	-0.06907543	7.15E-06	0.000118903	18261.45927045153939e-10
NONHSAG036307	0.010014737	0.875631421	0.953958166	-0.06914966	0.469698559	0.777851795	18270.641555270478459
NONHSAG036327	-0.08756194	0.247059441	0.583317506	-0.06922849	0.713212148	0.897950883	18280.491660099504337
NONHSAG036374	0.071336944	0.404353517	0.715272773	-0.06923339	0.105178155	0.357200785	18290.264105131468547
NONHSAG036383	0.202941095	0.003368985	0.025884117	-0.06935898	0.002416707	0.01998759	18300.00304225841235826
NONHSAG036413	-0.02943811	0.647228922	0.870666685	-0.06942283	0.206585807	0.516832107	18310.208129387047223
NONHSAG036444	0.041410677	0.478919698	0.76981996	-0.06943205	0.867455922	0.950047122	18320.647208300478427
NONHSAG036455	-0.01901448	0.742612135	0.909586883	-0.06944965	0.081700455	0.309208183	18330.177114249158491
NONHSAG036462	-0.12245424	0.14459089	0.451169103	-0.06953063	0.057733723	0.244867617	18340.137718071620371

NONHSAG036486	-0.15855693	0.053975376	0.241301681	-0.06953762	0.024307818	0.132211959	18350.0522149231865049
NONHSAG036538	0.084333506	0.113543077	0.39613292	-0.06953846	0.021086618	0.118828797	18360.0610598406283319
NONHSAG036539	0.573181509	1.70E-10	6.51E-09	-0.06954767	1.28E-06	2.59E-05	18377.23177651725207e-10
NONHSAG036566	0.077200214	0.212820618	0.544541927	-0.06988915	0.191575705	0.498018695	18380.334792528375859
NONHSAG036587	0.10441271	0.278768456	0.614339262	-0.06992742	0.75225259	0.906886268	18390.532829604274609
NONHSAG036631	0.120331101	0.096132943	0.360018759	-0.06996219	0.115041316	0.376596253	18400.172569871470159
NONHSAG036645	-0.03746662	0.457213288	0.756971211	-0.0701492	0.676810353	0.883971534	18410.500881096230331
NONHSAG036667	-0.05535696	0.38071274	0.705334496	-0.070534	0.467435651	0.776390461	18420.640992151566365
NONHSAG036669	-0.18538991	0.004585825	0.034114656	-0.07060239	0.002770652	0.022582823	18430.00390113135393617
NONHSAG036696	0.019141177	0.824542493	0.938461188	-0.07069817	0.011134817	0.073156786	18440.0193675967155939
NONHSAG036703	-0.07374417	0.131572759	0.429712658	-0.07090195	0.729120557	0.904080089	18450.282400034620962
NONHSAG036710	-0.09997059	0.184439669	0.511988498	-0.07104279	0.1666967	0.46365096	18460.291547237775118
NONHSAG036741	-0.06017442	0.448425636	0.751820679	-0.0710839	0.062376568	0.259088491	18470.167911391776887
NONHSAG036745	0.05661492	0.494930003	0.781439173	-0.07124122	0.077112267	0.297141877	18480.199029184853601
NONHSAG036767	-0.06575256	0.221286893	0.554652247	-0.07131379	0.185447652	0.490255938	18490.334915723062348
NONHSAG036776	0.033296863	0.661752992	0.876389826	-0.07137661	0.458654664	0.768157781	18500.754705183189242
NONHSAG036803	-0.15128638	0.038074317	0.187406018	-0.0714341	0.74144676	0.906043339	18510.0816565713783905
NONHSAG036808	-0.01167335	0.860185336	0.95118497	-0.07143546	0.412310821	0.733255014	18520.685441458826706
NONHSAG036869	0.227654141	0.001380894	0.012059237	-0.07156277	0.925832568	0.971770056	18530.00114092958553917
NONHSAG036874	0.077311626	0.448462317	0.751820679	-0.07158749	0.023555396	0.12937065	18540.0672336724667204
NONHSAG036892	0.01174505	0.817995792	0.938128871	-0.07170549	0.337650148	0.671160358	18550.450371141698539
NONHSAG036926	0.140204605	0.133945502	0.434936202	-0.07175224	0.709684033	0.896707014	18560.144260481252762
NONHSAG036955	0.161089923	0.007308375	0.050869182	-0.07177032	0.001744857	0.015194462	18570.00360840110821768
NONHSAG037015	0.023745762	0.747339922	0.910142859	-0.07183336	0.117142998	0.381560204	18580.249904373354306
NONHSAG037017	0.014850513	0.788409887	0.924700277	-0.07188171	0.130387551	0.410122811	18590.267656668473232
NONHSAG037020	0.008859448	0.907095797	0.964368008	-0.07197491	0.876894201	0.953529194	18600.96306713374286
NONHSAG037021	-0.23027368	0.003327881	0.025638358	-0.07199581	0.070184639	0.281539523	18610.0121053744478652
NONHSAG037022	0.049458804	0.516351643	0.799108873	-0.07207397	0.067034761	0.273190941	18620.173505068785248
NONHSAG037025	-0.08062101	0.251800818	0.587746844	-0.07233059	0.357283946	0.686695518	18630.474932595029368
NONHSAG037030	-0.35307333	0.000494923	0.004935189	-0.07234504	0.858121514	0.947213452	18640.000543400998370255
NONHSAG037031	0.105891783	0.052501233	0.236971859	-0.07235859	0.35925068	0.688441919	18650.0181542497506688
NONHSAG037032	-0.03309759	0.529404766	0.809639755	-0.07239876	0.197765202	0.505333441	18660.431049351794967
NONHSAG037083	-0.0045676	0.958857876	0.983730538	-0.07241625	0.449198184	0.756417348	18670.696279303137646
NONHSAG037087	0.074868507	0.199721794	0.529483422	-0.07245979	0.000320796	0.003414264	18680.00113862919079566
NONHSAG037150	-0.02499264	0.712997837	0.898275053	-0.07259477	0.463190841	0.772534191	18690.532406704105952
NONHSAG037173	0.591574168	6.89E-09	1.92E-07	-0.0726346	3.67E-08	1.00E-06	18703.57318611730332e-09
NONHSAG037197	-2.36866601	1.04E-15	9.77E-14	-0.07275376	2.45E-11	1.60E-09	18713.40581661877511e-15
NONHSAG037200	0.403544549	3.00E-07	5.58E-06	-0.07279931	3.71E-05	0.000505536	18727.90299970901457e-07
NONHSAG037224	0.351568546	8.66E-05	0.001031556	-0.07290544	3.43E-06	6.14E-05	18736.71537615576286e-06
NONHSAG037227	0.085254022	0.217356557	0.549514643	-0.07305682	0.384803422	0.709976889	18740.443876354884861
NONHSAG037229	0.071210983	0.211155053	0.544241987	-0.07321585	0.865359034	0.9494302	18750.394253685867137
NONHSAG037251	-0.00751217	0.899424171	0.962031483	-0.07328068	0.960702776	0.984151623	18760.991801046573495
NONHSAG037262	0.051315129	0.414701485	0.724427994	-0.07329182	0.791218909	0.921713034	18770.703750917401408
NONHSAG037272	-0.08904101	0.216602168	0.549316015	-0.07367542	0.061689881	0.257376775	18780.161666424615737
NONHSAG037287	0.340539353	5.52E-05	0.000699772	-0.07374678	5.38E-05	0.00071003	18793.28797947203042e-05
NONHSAG037322	-0.01655366	0.79682552	0.927815057	-0.07382936	0.66431557	0.880630922	18800.907915353208232
NONHSAG037336	-0.20354327	0.030474137	0.158397918	-0.0742232	0.086976879	0.31941043	18810.0737991935487996
NONHSAG037338	0.111730576	0.158026126	0.471729796	-0.07430122	0.037590779	0.181312642	18820.103383820157709
NONHSAG037352	0.344093992	2.06E-06	3.39E-05	-0.07444646	0.000193944	0.002199076	18836.00835903197174e-06
NONHSAG037375	-0.07884543	0.40381925	0.715075398	-0.07448261	0.823981789	0.933749876	18840.535515405738784
NONHSAG037376	0.011265034	0.868859317	0.951960393	-0.07451643	0.214993958	0.530318429	18850.399672927080802
NONHSAG037395	0.400534128	6.40E-07	1.15E-05	-0.07472359	0.000186603	0.002141741	18862.29613132429739e-06
NONHSAG037396	0.378937245	2.58E-09	7.81E-08	-0.07482888	1.92E-09	7.28E-08	18874.45827297195569e-10
NONHSAG037407	0.278596123	0.000878375	0.008071861	-0.07500114	0.001285002	0.011507721	18880.000977726748916247
NONHSAG037426	0.174801437	0.037363993	0.184652987	-0.07503455	0.028830206	0.149576641	18890.0487289771207324
NONHSAG037431	0.18615424	0.045294529	0.212111605	-0.07508032	0.478327845	0.781355214	18900.123260290802767
NONHSAG037432	1.750679343	1.74E-18	4.08E-16	-0.07518037	3.14E-17	1.47E-14	18914.96371543677421e-19
NONHSAG037433	0.662907747	6.12E-09	1.74E-07	-0.07540768	5.35E-07	1.18E-05	18921.0081494392766e-08
NONHSAG037464	-0.06015128	0.44310541	0.746562261	-0.07541435	0.562462797	0.826911648	18930.723458579562501
NONHSAG037470	0.219412859	0.004115072	0.031023012	-0.07578789	0.0003053	0.003327536	18940.000806935668354478
NONHSAG037504	0.042789166	0.470048965	0.764534973	-0.07596909	0.046044625	0.211564519	18950.125806419538642
NONHSAG037513	0.005066224	0.951116391	0.981209884	-0.07600553	0.030056098	0.152834989	18960.0478894370064064
NONHSAG037525	-0.02333378	0.81891863	0.938128871	-0.07633313	0.880514829	0.954988833	18970.972924847053181
NONHSAG037563	0.289902176	0.000273643	0.002887019	-0.07635446	3.52E-06	6.23E-05	18981.0215514303745e-05
NONHSAG037570	0.064807098	0.27146892	0.60896612	-0.07680081	0.933796235	0.973855106	18990.468633530054959
NONHSAG037575	0.036108584	0.654808913	0.872584313	-0.07681919	0.310694987	0.639585873	19000.59226002330404
NONHSAG037577	-0.06817623	0.339299785	0.673579125	-0.07702867	0.497291092	0.794835843	19010.612983482760164
NONHSAG037622	0.020994228	0.809760958	0.93439361	-0.07706651	0.784260461	0.91889184	19020.874539487554267
NONHSAG037629	0.187769811	0.191577459	0.523025062	-0.07717517	0.077151804	0.297141877	19030.184172881172112
NONHSAG037701	-0.11469926	0.152254583	0.462853933	-0.07726322	0.814347656	0.931603479	19040.303390608056377
NONHSAG037719	0.105181183	0.137378893	0.44099252	-0.07758081	0.541850051	0.812737703	19050.322300627131623

NONHSAG037724	0.08260402	0.13624928	0.440017024	-0.07762124	0.015247067	0.093409046	19060.0487834157815067
NONHSAG037726	-0.01846607	0.683944621	0.887517619	-0.07762816	0.920320569	0.971084968	19070.912752885059191
NONHSAG037747	0.061224909	0.385847867	0.706496807	-0.07763174	0.67192705	0.88168869	19080.682857160358584
NONHSAG037751	-0.02240626	0.727413367	0.903322681	-0.0777405	0.66549838	0.880630922	19090.898898415086232
NONHSAG037786	0.695867437	1.63E-15	1.48E-13	-0.07777618	3.71E-11	2.27E-09	19105.36276118400374e-15
NONHSAG037791	0.013797353	0.807327237	0.93439361	-0.07798207	0.773237391	0.91321261	19110.866367936527151
NONHSAG037804	0.289965059	0.005106802	0.037396687	-0.07834698	0.002923655	0.023624473	19120.00426752825552634
NONHSAG037839	0.46745347	2.28E-07	4.39E-06	-0.07835029	7.87E-07	1.66E-05	19131.35249198012461e-07
NONHSAG037842	0.092069888	0.283419153	0.619797841	-0.07839667	0.832085364	0.935181473	19140.519921940362739
NONHSAG037849	-0.04871877	0.471074121	0.764534973	-0.07863077	0.485454878	0.784990867	19150.712113834743821
NONHSAG037860	0.028375362	0.711758008	0.898275053	-0.07872354	0.429513601	0.746421851	19160.728316335488443
NONHSAG037870	-0.00964689	0.842244366	0.945700556	-0.07877083	0.727257854	0.903290233	19170.939788897753397
NONHSAG037873	0.012458744	0.830427916	0.941977934	-0.07879662	0.8297368	0.933749876	19180.91097676278754
NONHSAG037878	0.0137194	0.752188237	0.910732246	-0.07900759	0.213580893	0.527758761	19190.428379741224977
NONHSAG037943	-0.01432939	0.716118898	0.898984974	-0.07902128	0.695991895	0.895338645	19200.750517801653947
NONHSAG037955	-0.01397622	0.773279207	0.918276503	-0.07937954	0.880580349	0.954988833	19210.904421406606589
NONHSAG037959	0.010087232	0.902959261	0.963247892	-0.07956734	0.177951397	0.482403482	19220.326040961008878
NONHSAG037962	-0.06806905	0.365904228	0.693811657	-0.0797284	0.971821493	0.989451568	19230.564096510580975
NONHSAG037977	0.312027184	0.01059863	0.0691493	-0.0800199	3.72E-05	0.000505536	19240.000171934102675238
NONHSAG037987	0.028200914	0.657295046	0.874320563	-0.08005677	0.705496372	0.896707014	19250.890285453347612
NONHSAG037991	0.043497944	0.59663833	0.846326605	-0.08020288	0.069886762	0.281146743	19260.171476732960462
NONHSAG038027	0.011687958	0.905309496	0.964289176	-0.08034152	0.965922123	0.986709541	19270.992660336697403
NONHSAG038035	0.049014277	0.368922253	0.697210149	-0.08053459	0.074521779	0.291453745	19280.199303185447312
NONHSAG038048	-0.01118042	0.832740141	0.942580607	-0.08062309	0.403304752	0.724100325	19290.68176316707202
NONHSAG038071	-0.01019668	0.86950079	0.951960393	-0.0806911	0.211848336	0.525081886	19300.302687295480845
NONHSAG038091	0.05329628	0.415195438	0.724427994	-0.08088928	0.067998939	0.276319389	19310.18317549907776
NONHSAG038132	-0.03410366	0.679058511	0.887517619	-0.08089684	0.609121266	0.847327128	19320.861248557527936
NONHSAG038179	0.0191616	0.871180483	0.952472597	-0.08095993	0.617084751	0.853798275	19330.786058016326011
NONHSAG038210	-0.12858986	0.191431683	0.523025062	-0.0810136	0.989747898	0.996597046	19340.321723286252915
NONHSAG038213	-0.052871	0.371203019	0.698354362	-0.08102313	0.384770401	0.709976889	19350.217274538215419
NONHSAG038259	0.215256423	0.00132089	0.011654188	-0.08110497	0.564969263	0.82952797	19360.00277564307772064
NONHSAG038285	0.175637155	0.011400252	0.07369542	-0.08115715	0.307539533	0.635739391	19370.0370374203212082
NONHSAG038292	0.018467119	0.683294021	0.887517619	-0.08129716	0.76278743	0.910423706	19380.774102149237969
NONHSAG038333	0.085125007	0.178050856	0.502625892	-0.08134772	0.667342333	0.880630922	19390.382688218688165
NONHSAG038350	0.052883761	0.549352036	0.820817175	-0.08137809	0.757829093	0.908748575	19400.833722006690679
NONHSAG038364	-0.03879363	0.620505546	0.857004713	-0.0814495	0.358429817	0.687520223	19410.651490186136587
NONHSAG038417	1.254103818	5.30E-09	1.52E-07	-0.08151149	5.67E-08	1.52E-06	19423.70745289294861e-09
NONHSAG038444	-0.14235524	0.018791503	0.108142692	-0.08152754	0.180463812	0.484356613	19430.0602193567631013
NONHSAG038460	-0.05061602	0.343649595	0.677284107	-0.08164358	0.21193739	0.525081886	19440.424402546203945
NONHSAG038476	-0.02588854	0.764088582	0.915083941	-0.08167157	0.631106348	0.859821245	19450.731762415943383
NONHSAG038511	0.053857317	0.304866057	0.643123295	-0.08182766	0.058975186	0.24900634	19460.163506311518606
NONHSAG038512	0.295612283	0.00410982	0.031023012	-0.08206752	0.007539385	0.052869705	19470.006459155359191
NONHSAG038522	0.111162919	0.136430214	0.440017024	-0.08224031	0.133126344	0.414630871	19480.223857263906836
NONHSAG038586	-0.50041507	0.000264343	0.002827383	-0.08226672	0.082650808	0.31112995	19490.0011447987635182
NONHSAG038591	0.066171052	0.149517888	0.459389684	-0.08229742	0.259121643	0.59061012	19500.313873646447854
NONHSAG038599	-0.07687095	0.374559813	0.699377288	-0.08235463	0.699286783	0.895443732	19510.426183866309353
NONHSAG038627	-0.4431494	8.35E-05	0.000999306	-0.08236626	4.69E-05	0.000624876	19523.80952394833734e-05
NONHSAG038649	-0.10166548	0.174027333	0.49833489	-0.08240285	0.565093383	0.82952797	19530.144435300668119
NONHSAG038724	0.054509573	0.465238513	0.763571876	-0.08253093	0.81472659	0.931603479	19540.754222841271294
NONHSAG038765	0.00414261	0.956998786	0.983730538	-0.08255304	0.233057742	0.557277527	19550.368397197028533
NONHSAG038779	-0.05561326	0.515920242	0.799108873	-0.08274524	0.117236137	0.381560204	19560.284283609421985
NONHSAG038780	0.008858019	0.857494622	0.950687167	-0.08292043	0.769144731	0.91321261	19570.956560589401778
NONHSAG038818	0.024447851	0.668535147	0.881350601	-0.08295728	0.262044065	0.592856869	19580.521929361474739
NONHSAG038845	-0.71647758	1.68E-09	5.37E-08	-0.08296875	1.07E-07	2.63E-06	19592.21180784739457e-09
NONHSAG038869	-0.02116534	0.728585596	0.903322681	-0.08306797	0.424914159	0.741866512	19600.722671924200896
NONHSAG038874	0.031778183	0.613285459	0.852440007	-0.08307137	0.841909954	0.941172323	19610.876804054974381
NONHSAG038882	-0.0588618	0.398714838	0.712889467	-0.08316938	0.274677831	0.603336539	19620.515394278189157
NONHSAG038890	0.844351179	3.91E-12	2.11E-10	-0.08318248	6.12E-11	3.51E-09	19631.90206407105185e-12
NONHSAG038899	-0.0198261	0.762421363	0.915035797	-0.08331687	0.621158059	0.853798275	19640.721908279658025
NONHSAG038901	-0.14855719	0.130410942	0.429206713	-0.08337297	0.024420143	0.132566492	19650.0707915058823286
NONHSAG038916	0.029775937	0.625456032	0.85822947	-0.08347565	0.583173539	0.836050856	19660.832437878845259
NONHSAG038917	-0.09365541	0.158895037	0.472842244	-0.08355757	0.794963821	0.923062508	19670.201335749375662
NONHSAG038920	-0.02084745	0.736069827	0.905538909	-0.08365233	0.369233278	0.696834885	19680.658684055108037
NONHSAG038949	0.121367582	0.068751154	0.28598853	-0.08365465	0.001083404	0.010021488	19690.00443232312161129
NONHSAG038955	0.129314783	0.0693286	0.287014087	-0.08380831	0.022090066	0.123100786	19700.0541259406775618
NONHSAG038958	0.00080444	0.988947163	0.996279605	-0.08409516	0.808669578	0.930212019	19710.963191316149007
NONHSAG038962	-0.03490049	0.534884831	0.81390484	-0.08412865	0.820756459	0.933749876	19720.818662270307484
NONHSAG038974	0.09779881	0.189655091	0.520302551	-0.08421259	0.033591006	0.165136205	19730.0981655901699533
NONHSAG038975	0.036438931	0.563804855	0.830383739	-0.08443732	0.603220426	0.843391701	19740.814792576469026
NONHSAG038999	-0.03370087	0.537177289	0.813923495	-0.08449238	0.747838872	0.906696003	19750.63284513176407
NONHSAG039007	0.009636546	0.905750353	0.964289176	-0.08455771	0.319899203	0.651852579	19760.550110544718025

NONHSAG039009	0.083435979	0.265703959	0.603793395	-0.08456884	0.480623443	0.783450231	19770.192196926272551
NONHSAG039017	0.117052251	0.055674734	0.247325989	-0.08464508	0.355766697	0.686695518	19780.155884591095757
NONHSAG039019	-0.01861175	0.82532522	0.938461188	-0.08466045	0.137890484	0.423305723	19790.181059745234379
NONHSAG039034	0.074074767	0.559052779	0.826093754	-0.08468424	0.261711819	0.592856869	19800.527772683609182
NONHSAG039040	-0.00451884	0.950057448	0.981209884	-0.08473597	0.878932639	0.954988833	19810.975520947264014
NONHSAG039046	-0.04191746	0.529853384	0.809754193	-0.08505268	0.400521607	0.722892657	19820.679124721732529
NONHSAG039047	-0.06233141	0.313803053	0.651448843	-0.0851624	0.041546295	0.195692095	19830.121597652433587
NONHSAG039060	-0.0181131	0.733655213	0.905538909	-0.08519809	0.506102231	0.80071508	19840.799108409510111
NONHSAG039061	-0.05348485	0.397482191	0.712475957	-0.08521015	0.820219284	0.933749876	19850.677724388111735
NONHSAG039063	-0.07268397	0.142661042	0.447555229	-0.08525801	0.060178005	0.252567986	19860.141041160699628
NONHSAG039082	0.039637797	0.648360729	0.870673529	-0.08527603	0.414990408	0.733932722	19870.712864730046424
NONHSAG039102	-0.05185608	0.379595543	0.704499337	-0.08530528	0.196999668	0.504520098	19880.41545405920757
NONHSAG039105	0.024986771	0.743100409	0.909586883	-0.0853179	0.07944827	0.303544207	19890.172221205259829
NONHSAG039107	0.186904751	0.003223875	0.024905323	-0.08532106	0.003681505	0.028362715	19900.00379136118426802
NONHSAG039111	-0.03444617	0.624970767	0.85822947	-0.0853522	0.524723236	0.807170039	19910.528938941676664
NONHSAG039112	0.028413091	0.743378085	0.909586883	-0.08552898	0.200929295	0.50902088	19920.408535638343933
NONHSAG039130	0.147064935	0.036672953	0.18187715	-0.08568341	0.000140381	0.001631207	19930.00064301221370755
NONHSAG039135	-0.25189239	0.000750308	0.007077921	-0.08571189	0.001239623	0.011244577	19940.00871415170056554
NONHSAG039189	-0.08182719	0.099016125	0.36482008	-0.08590367	0.827925516	0.933749876	19950.197575256514757
NONHSAG039190	0.115443848	0.250586931	0.586231655	-0.08602753	0.581106835	0.836050856	19960.511747718389512
NONHSAG039191	-0.00741385	0.895988108	0.961169104	-0.08604856	0.699747326	0.895625617	19970.864260143946983
NONHSAG039225	0.265442456	0.000340405	0.003493504	-0.08621664	0.001431004	0.012574948	19980.000563035198222948
NONHSAG039294	0.029759781	0.617562878	0.854890583	-0.086738	0.472519356	0.778445171	19990.473072748939218
NONHSAG039315	0.055516483	0.389619991	0.706543193	-0.08678926	0.147229269	0.432159398	20000.340972030538237
NONHSAG039321	0.070839449	0.332746247	0.669779847	-0.08680913	0.118507768	0.384412149	20010.284673262061945
NONHSAG039327	0.091443831	0.19704352	0.527031094	-0.08737556	0.130604995	0.410276175	20020.0249582672889824
NONHSAG039332	-0.0376578	0.46870363	0.764498032	-0.08741833	0.635268931	0.862005573	20030.48253041044961
NONHSAG039337	0.351387488	0.000640804	0.006192238	-0.08764293	0.000670192	0.006543679	20040.000564520021400964
NONHSAG039356	-0.10523663	0.05745224	0.252036973	-0.08785185	0.377079497	0.704082035	20050.159617226083416
NONHSAG039358	0.006742494	0.917644596	0.968261389	-0.08787254	0.774352932	0.913755956	20060.920591036799642
NONHSAG039367	0.120303951	0.198692575	0.528551011	-0.08835226	0.466508764	0.775308891	20070.430863313352348
NONHSAG039377	-0.02920553	0.715274269	0.898703486	-0.08859273	0.475693783	0.780309094	20080.547178099570285
NONHSAG039399	0.092148045	0.178617324	0.502774689	-0.08881481	0.920846176	0.971275111	20090.32189957037604
NONHSAG039414	-0.02349884	0.683767992	0.887517619	-0.08882708	0.659931779	0.878826774	20100.696618410577415
NONHSAG039470	-0.02215457	0.780516036	0.920990125	-0.08883472	0.293577139	0.623485995	20110.374510781766518
NONHSAG039489	-0.02007326	0.78839231	0.924700277	-0.08899328	0.897932423	0.962120226	20120.920356739977769
NONHSAG039490	-0.00513041	0.956211409	0.983730538	-0.08911115	0.781283106	0.917391514	20130.95709185604124
NONHSAG039502	0.080130667	0.434713214	0.738589088	-0.08918966	0.77523104	0.914025025	20140.728319406107102
NONHSAG039503	-0.03738643	0.590648345	0.842243593	-0.08955466	0.618265011	0.853798275	20150.833530998301643
NONHSAG039511	0.064272418	0.388013338	0.706496807	-0.08962242	0.720688834	0.902489903	20160.682670684319662
NONHSAG039515	0.060221811	0.46952471	0.764534973	-0.0899453	0.031471231	0.158030541	20170.0876453386189313
NONHSAG039551	0.034039444	0.646258562	0.870666685	-0.09002094	0.86798059	0.950047122	20180.896236439468126
NONHSAG039586	0.061829711	0.300025811	0.637694547	-0.09002182	0.86828631	0.950047122	20190.533778014878308
NONHSAG039670	0.116667401	0.15397528	0.465568266	-0.09003097	0.161120136	0.45587637	20200.26219598842385
NONHSAG039676	0.024643628	0.627087729	0.85822947	-0.09003775	0.415895358	0.734609138	20210.711977253826143
NONHSAG039681	-0.19978003	0.005393583	0.039190582	-0.09022138	0.215190194	0.530337271	20220.0192217307924133
NONHSAG039701	-0.06106436	0.449878319	0.752016606	-0.09040176	0.797249459	0.92413553	20230.572973513466139
NONHSAG039715	0.071687549	0.348009896	0.677785637	-0.09042046	0.765633084	0.911884893	20240.627139801786516
NONHSAG039740	0.082341751	0.252503897	0.587773032	-0.09043324	0.874635775	0.952087485	20250.459128755549938
NONHSAG039770	-0.00216812	0.969716296	0.989067682	-0.09049753	0.741216945	0.906043339	20260.920233558411016
NONHSAG039813	0.158323836	0.066148535	0.278875085	-0.09056854	0.105760385	0.357879907	20270.13318441802255
NONHSAG039828	-0.11596408	0.323823434	0.664056702	-0.09060054	0.88396616	0.956890351	20280.4661559279313
NONHSAG039846	-0.0423519	0.484394807	0.773504695	-0.09076212	0.81873608	0.933611459	20290.624414751605414
NONHSAG039857	0.040450849	0.640707398	0.867640994	-0.09079969	0.694086922	0.895088581	20300.880142452883665
NONHSAG039863	-0.40847972	7.20E-05	0.00088066	-0.09116828	0.018024918	0.106077773	20310.000332175467849886
NONHSAG039875	0.014741561	0.844827761	0.94647636	-0.09129104	0.019841183	0.114330752	20320.0355048901329972
NONHSAG039889	-0.01008719	0.897179508	0.961169104	-0.0915243	0.38166746	0.708236919	20330.548235414997224
NONHSAG039949	0.067208144	0.331471853	0.669461779	-0.0916001	0.014205903	0.087795603	20340.0450098879721419
NONHSAG039968	0.051191354	0.438379071	0.740968838	-0.09171134	0.582134685	0.836050856	20350.413564899615057
NONHSAG039971	0.08796745	0.201605327	0.532313785	-0.09193375	0.742684885	0.906043339	20360.409868944077192
NONHSAG039976	0.015572946	0.770715948	0.91793869	-0.09195011	0.183185163	0.488163241	20370.370766882343237
NONHSAG039991	0.038737042	0.644059939	0.870030385	-0.0919737	0.364692418	0.69386861	20380.659188468222513
NONHSAG040004	0.111791608	0.105035846	0.37869582	-0.09201806	0.144525869	0.430061982	20390.201916858580925
NONHSAG040070	-0.02875291	0.658517448	0.875035827	-0.09209768	0.338784309	0.672308734	20400.628100496868686
NONHSAG040081	0.195023667	0.067854857	0.283097712	-0.09247789	0.018560634	0.108734384	20410.04742954598468
NONHSAG040093	0.021812843	0.719771438	0.901651169	-0.09273719	0.699132558	0.895443732	20420.910079614704621
NONHSAG040100	0.028512817	0.702133023	0.895955243	-0.09293273	0.366163198	0.69386861	20430.418424580851218
NONHSAG040122	0.105893426	0.080629996	0.316664175	-0.09293842	0.19784665	0.505333441	20440.191732155757405
NONHSAG040157	-0.48808109	2.42E-08	5.77E-07	-0.09303978	2.34E-06	4.44E-05	20454.65902476069642e-08
NONHSAG040159	-0.0250365	0.734981893	0.905538909	-0.09332697	0.258861762	0.59061012	20460.308365180160937
NONHSAG040185	0.182058856	0.000232095	0.002519892	-0.09350551	0.000126013	0.001501479	20470.000119706015153905

NONHSAG040189	-0.07195972	0.342287672	0.675447672	-0.09358298	0.345770768	0.678791662	20480.173794915480258
NONHSAG040206	-0.90420486	4.29E-11	1.95E-09	-0.09360316	7.81E-10	3.28E-08	20492.59771332775106e-11
NONHSAG040218	0.005085582	0.926464869	0.973226917	-0.0939473	0.124807993	0.398893716	20500.225017041668418
NONHSAG040274	0.031978956	0.604696651	0.848743629	-0.09396475	0.69902262	0.895443732	20510.863424273003387
NONHSAG040276	0.057446648	0.320869419	0.660012926	-0.09397785	0.394530319	0.718406651	20520.558301541604395
NONHSAG040302	0.050645423	0.466892896	0.764261292	-0.09399489	0.116315513	0.379442253	20530.285445936455493
NONHSAG040333	0.566170151	6.46E-10	2.22E-08	-0.09441018	7.01E-08	1.85E-06	20549.58729673091612e-10
NONHSAG040375	-0.07124538	0.365348707	0.693811657	-0.09445825	0.054699371	0.236412243	20550.0195658620711546
NONHSAG040378	-0.01246382	0.844796052	0.94647636	-0.0945046	0.589568053	0.837728836	20560.746258945953306
NONHSAG040385	-0.03095258	0.686858062	0.887517619	-0.09467266	0.73797479	0.906043339	20570.75945039064851
NONHSAG040395	0.056751864	0.300251629	0.637694547	-0.09478374	0.003373803	0.026404896	20580.0111346969194435
NONHSAG040459	-0.00351146	0.951550268	0.981209884	-0.09490948	0.6342595	0.861196385	20590.839664013041662
NONHSAG040465	-0.03138701	0.588832052	0.842139475	-0.09496473	0.944305479	0.976126639	20600.83925389090003
NONHSAG040492	-0.09659318	0.247788362	0.58356857	-0.09519231	0.723913839	0.902489903	20610.490772682622639
NONHSAG040535	0.175627637	0.017736161	0.104121265	-0.09529423	0.000839846	0.00795167	20620.00284070430679171
NONHSAG040538	-0.01243497	0.813046147	0.935468807	-0.09530059	0.539775471	0.812140251	20630.678735760911594
NONHSAG040545	0.053263977	0.331594606	0.669461779	-0.09534728	0.284871721	0.615265887	20640.493953702791526
NONHSAG040546	0.031099342	0.746348802	0.909586883	-0.09542345	0.325155926	0.658745292	20650.600285407078791
NONHSAG040559	-0.2087813	0.000850979	0.00787156	-0.09566934	0.044109567	0.204342836	20660.00343188565690974
NONHSAG040605	0.140068169	0.019951965	0.113355636	-0.09584819	0.081308569	0.308139752	20670.053172438835487
NONHSAG040664	-0.36346286	2.03E-07	4.05E-06	-0.09608542	7.73E-06	0.000127837	20683.4193469726801e-07
NONHSAG040668	0.219939469	0.00167708	0.014204665	-0.09625655	0.055861761	0.239460937	20690.0064494910460264
NONHSAG040671	-0.02399957	0.723704313	0.903322681	-0.09630118	0.953975618	0.980474941	20700.929645176788597
NONHSAG040680	-0.02737958	0.781567628	0.921496087	-0.09648229	0.515923632	0.804430331	20710.634149932752105
NONHSAG040684	0.045376499	0.515863527	0.799108873	-0.09681528	0.145118509	0.430457013	20720.339090448952291
NONHSAG040703	0.348681931	9.03E-05	0.00107016	-0.0969192	3.07E-05	0.000427316	20733.05443267196851e-05
NONHSAG040714	0.111744198	0.145209548	0.451192539	-0.09694633	0.506569036	0.80071508	20740.339769927649446
NONHSAG040766	-0.10680875	0.061455751	0.263837515	-0.09702854	0.226308338	0.548428253	20750.16265498160896
NONHSAG040771	0.08207286	0.175499823	0.499391278	-0.09713215	0.300379873	0.629081811	20760.063770018296786
NONHSAG040781	0.103239822	0.166900506	0.489663415	-0.0971787	0.040340584	0.191942001	20770.110524008818176
NONHSAG040790	-0.02068132	0.803051494	0.93227155	-0.0974457	0.505045248	0.800556504	20780.636693932907098
NONHSAG040792	-0.06171176	0.440365096	0.743409945	-0.09751451	0.672252133	0.881703824	20790.480074069484264
NONHSAG040794	0.233881129	0.015242314	0.091584157	-0.0976164	0.652543138	0.874925463	20800.0346907485826801
NONHSAG040797	-0.06365634	0.383514305	0.706496807	-0.09777932	0.291462576	0.620937442	20810.526273300133234
NONHSAG040826	0.632361487	1.45E-05	0.00020231	-0.09805805	3.00E-08	8.56E-07	20829.60635145395587e-08
NONHSAG040838	-0.11605192	0.139438694	0.443553855	-0.09810674	0.259179548	0.59061012	20830.300296340784411
NONHSAG040876	0.496550633	1.81E-08	4.47E-07	-0.09810918	8.90E-08	2.26E-06	20849.88226638619065e-09
NONHSAG040878	0.705632605	1.18E-06	2.02E-05	-0.09815227	8.94E-08	2.26E-06	20858.99403133436818e-08
NONHSAG040919	-0.05448073	0.274480133	0.612084166	-0.09828937	0.3531678	0.684429948	20860.495950383572545
NONHSAG040944	-0.13899334	0.139869506	0.443919922	-0.09848493	0.472212153	0.778445171	20870.330962337204632
NONHSAG040959	0.223603206	0.003035457	0.023579294	-0.09875491	1.35E-05	0.000201462	20885.73295884955329e-05
NONHSAG040973	0.063904388	0.232184002	0.56479361	-0.09883506	0.750313513	0.906696003	20890.459973238783098
NONHSAG040975	-0.07798946	0.290475889	0.626875057	-0.09897633	0.296354493	0.625637264	20900.475843955667976
NONHSAG040987	-0.04721586	0.509523228	0.79598851	-0.09898115	0.809362266	0.930212019	20910.797730148916246
NONHSAG040990	0.103498325	0.08435965	0.327199082	-0.09922696	0.080687885	0.30620018	20920.133922853275419
NONHSAG040992	0.001244258	0.989150805	0.996279605	-0.09929938	0.409007851	0.729548907	20930.626868919039102
NONHSAG041024	-0.08454556	0.130577213	0.429206713	-0.0998016	0.722460397	0.902489903	20940.2817384673649
NONHSAG041029	0.003590226	0.954600521	0.983194555	-0.0998032	0.57782118	0.833831576	20950.826879124677528
NONHSAG041052	0.141748888	0.194124082	0.526251063	-0.09994971	0.020093476	0.11531195	20960.0641498702025576
NONHSAG041087	0.044803785	0.555402593	0.821876574	-0.10012898	0.584539107	0.836463428	20970.803369793321812
NONHSAG041094	0.338852038	1.52E-06	2.56E-05	-0.10026971	2.48E-06	4.62E-05	20987.60616015070889e-07
NONHSAG041153	-0.06146773	0.341747466	0.675329496	-0.10027594	0.167975618	0.464908894	20990.365463095149845
NONHSAG041157	-0.01601378	0.888300441	0.956683585	-0.10054403	0.073172658	0.288585574	21000.0998524668359381
NONHSAG041202	-0.09949063	0.041675531	0.200670538	-0.10093598	0.008603288	0.05904542	21010.0227517207255206
NONHSAG041205	0.177863289	0.007701227	0.053078064	-0.10095192	0.014133935	0.087543226	21020.0131992895921916
NONHSAG041207	0.074115283	0.31690226	0.654760584	-0.10110994	0.558835132	0.824905193	21030.599042951590151
NONHSAG041271	0.190928949	0.001533518	0.01311149	-0.10156089	0.071826839	0.284875981	21040.00610480765243432
NONHSAG041280	0.309904251	6.60E-05	0.000820776	-0.10176118	0.000293969	0.003229064	21058.93089521032528e-05
NONHSAG041308	-0.03427819	0.546396048	0.819448366	-0.10184002	0.934240224	0.973855106	21060.805066880373245
NONHSAG041310	0.07161528	0.149644719	0.459389684	-0.10194803	0.583630096	0.836050856	21070.341696222650356
NONHSAG041311	0.079504509	0.230445509	0.562511087	-0.10214788	0.166987962	0.463728771	21080.321445237787845
NONHSAG041331	-0.44145897	2.75E-07	5.15E-06	-0.10245832	0.000973025	0.009120486	21091.44765777551484e-06
NONHSAG041341	0.072137764	0.206834187	0.540035035	-0.10258004	0.824596571	0.933749876	21100.398551742679295
NONHSAG041345	-0.00322628	0.957774038	0.983730538	-0.10264942	0.439864669	0.752824306	21110.685920838234303
NONHSAG041359	0.048959888	0.503902636	0.789138617	-0.10301641	0.906955054	0.964948018	21120.772319567748948
NONHSAG041393	0.065831135	0.23209636	0.56479361	-0.10307677	0.09449699	0.336361438	21130.224022819889605
NONHSAG041442	0.162721219	0.058603497	0.25555292	-0.1030955	0.077133731	0.297141877	21140.106840903398277
NONHSAG041473	0.195285872	0.171692486	0.496021375	-0.10314129	0.872838443	0.950957653	21150.323614510990211
NONHSAG041474	0.020949411	0.715158171	0.898703486	-0.10340187	0.742683433	0.906043339	21160.921382044878418
NONHSAG041475	0.029486614	0.543499225	0.817395852	-0.10374959	0.138807984	0.424269621	21170.324934682272093
NONHSAG041496	0.047787091	0.525611319	0.806778947	-0.10378854	0.746937821	0.906696003	21180.620641643239868

NONHSAG041512	-0.10772452	0.178572611	0.502774689	-0.1038259	0.02321177	0.127732869	21190.0718919935207065
NONHSAG041520	0.184023545	0.027042422	0.144294667	-0.10460624	0.102387361	0.351113732	21200.0716053276028259
NONHSAG041523	0.41051231	0.000440404	0.004422917	-0.10463048	0.000586817	0.005810313	21210.00042063896217928
NONHSAG041551	0.125109615	0.168281808	0.490880129	-0.10466152	0.02555139	0.136597924	21220.0771665595766285
NONHSAG041552	0.044511544	0.468286702	0.764261292	-0.10479948	0.128868385	0.406253249	21230.31014582846333
NONHSAG041579	0.043618127	0.618041359	0.854890583	-0.10491166	0.708040813	0.896707014	21240.872317399900688
NONHSAG041596	0.108525987	0.284835754	0.621861911	-0.10505653	0.792266912	0.922059223	21250.532712320276221
NONHSAG041601	-0.01949413	0.824782912	0.938461188	-0.10508652	0.172518813	0.473290636	21260.33802158694972
NONHSAG041618	-0.21158216	0.012760125	0.079559806	-0.10527615	0.024994941	0.134905517	21270.0240950378879421
NONHSAG041677	0.08930884	0.228677616	0.559166483	-0.10538401	0.056280327	0.239714801	21280.152323266620228
NONHSAG041723	0.340058761	0.001373414	0.012031279	-0.10544762	0.000426625	0.004391902	21290.000641706080069349
NONHSAG041789	0.109669473	0.078489697	0.312624684	-0.10574914	0.289106465	0.617507221	21300.204409711103672
NONHSAG041804	0.000587707	0.990270677	0.996279605	-0.10581883	0.762027742	0.910423706	21310.942148587889477
NONHSAG041810	0.008907429	0.906880991	0.964368008	-0.10600593	0.54707808	0.8161186	21320.813228157867971
NONHSAG041826	-0.09288892	0.111256418	0.392190813	-0.1062153	0.343661462	0.677208152	21330.272834107876984
NONHSAG041886	-0.09950245	0.306601116	0.644626559	-0.10721719	0.026350498	0.140071077	21340.0812707427215535
NONHSAG041903	0.009359248	0.854953932	0.949662955	-0.10778346	0.334101346	0.67010912	21350.586480753265944
NONHSAG041984	0.080011919	0.417197894	0.72506828	-0.10780792	0.011899184	0.077025774	21360.0351219578956852
NONHSAG041987	0.132357908	0.0666731	0.280665802	-0.10832806	0.090169564	0.326748472	21370.124327540687096
NONHSAG041994	0.088762792	0.141390683	0.446229631	-0.10841249	0.931066707	0.973855106	21380.215970323141576
NONHSAG041998	-0.09336754	0.191545894	0.523025062	-0.1084957	0.294109504	0.623485995	21390.380646018868728
NONHSAG041999	-0.04828997	0.53615819	0.813923495	-0.10856254	0.046539588	0.213142218	21400.121569828305197
NONHSAG042046	-0.00512434	0.897834809	0.961169104	-0.10889447	0.729525588	0.904080089	21410.88536220086
NONHSAG042051	-0.0130197	0.852658628	0.949198758	-0.10918523	0.646662577	0.870055103	21420.800811024323792
NONHSAG042059	-0.01981422	0.785371033	0.923656773	-0.10934633	0.626914681	0.857433892	21430.742527903130674
NONHSAG042103	-0.00035766	0.995095127	0.996867651	-0.10943033	0.289209463	0.617507221	21440.467751287441004
NONHSAG042145	0.02385976	0.601323619	0.848669165	-0.1095292	0.327920433	0.662657946	21450.615066567334489
NONHSAG042163	-0.02333175	0.744815957	0.909586883	-0.1095356	0.403327035	0.724100325	21460.487295505450554
NONHSAG042168	0.037634274	0.536293899	0.813923495	-0.10957435	0.733368924	0.905644804	21470.621798661973819
NONHSAG042176	0.691242833	4.94E-16	4.96E-14	-0.10999994	1.91E-14	2.68E-12	21482.2963962521949e-16
NONHSAG042182	-0.15193754	0.067844058	0.283097712	-0.11028436	0.255419254	0.586701827	21490.179307153250859
NONHSAG042192	0.011826515	0.803305109	0.93227155	-0.11028691	0.321382774	0.65343573	21500.582879519345746
NONHSAG042210	-0.17861695	0.046630309	0.217093427	-0.11086738	0.401933237	0.724046292	21510.131691021236445
NONHSAG042250	-0.05396184	0.25814491	0.596469586	-0.11088859	0.55796551	0.824054104	21520.222845827207955
NONHSAG042261	-0.71070207	3.20E-10	1.14E-08	-0.11101365	0.010269649	0.068431878	21531.15864419213848e-09
NONHSAG042272	0.021712742	0.658872655	0.875035827	-0.11120766	0.932611701	0.973855106	21540.850836082302816
NONHSAG042283	-0.04528304	0.470529448	0.764534973	-0.1114547	0.44084852	0.752824306	21550.686085583390977
NONHSAG042315	0.157066931	0.06477211	0.273893496	-0.11163546	0.080011353	0.304454568	21560.115300231058403
NONHSAG042323	-0.10868659	0.20805982	0.541863818	-0.11165398	0.278297653	0.60825425	21570.391423466753891
NONHSAG042369	0.265933471	0.019954139	0.113355636	-0.11171795	0.086915673	0.31941043	21580.0543144158162849
NONHSAG042381	0.131482831	0.078306207	0.312336247	-0.11182178	0.389502066	0.713031652	21590.207488390942911
NONHSAG042418	0.178304527	0.085587729	0.330215606	-0.1121396	0.771543902	0.91321261	21600.0975859800962418
NONHSAG042419	0.067439065	0.379645161	0.704499337	-0.11222496	0.328029823	0.662657946	21610.556847836890084
NONHSAG042424	0.135220405	0.069437468	0.287014087	-0.11222884	0.164208339	0.459456565	21620.16313251305051
NONHSAG042444	-0.03461382	0.679229944	0.887517619	-0.11237137	0.434012397	0.748737951	21630.732926438156676
NONHSAG042451	-0.10181921	0.197153338	0.527031094	-0.11247829	0.843105648	0.941172323	21640.271495437786661
NONHSAG042471	0.266595055	0.004692817	0.034685214	-0.11252179	0.011745447	0.076277588	21650.00875817236410277
NONHSAG042535	0.046036978	0.4206925	0.726032559	-0.112911	0.025353343	0.13605649	21660.0737583693209768
NONHSAG042546	-0.00656563	0.926157351	0.973226917	-0.11300048	0.685227651	0.889213579	21670.912558731174898
NONHSAG042558	0.097224898	0.200366431	0.530038009	-0.11303021	0.706282404	0.896707014	21680.415304913019984
NONHSAG042615	0.063449356	0.314055733	0.651448843	-0.11364782	0.391866739	0.715074153	21690.551016777756563
NONHSAG042636	-0.01117284	0.879961095	0.954268263	-0.11373288	0.948036582	0.977945293	21700.975369260413903
NONHSAG042638	0.118475792	0.032478174	0.165151222	-0.11376191	0.090960891	0.328768672	21710.0784346106090027
NONHSAG042726	-0.07090137	0.356386069	0.682203965	-0.1137814	0.614136664	0.850979373	21720.353336297509502
NONHSAG042795	0.069057405	0.238029199	0.5711076	-0.11396736	0.335209207	0.671076968	21730.450770038589476
NONHSAG042834	0.010667857	0.863102005	0.951781505	-0.11398152	0.575939423	0.833526329	21740.846661203002531
NONHSAG042877	0.265541879	0.001228316	0.01096516	-0.11420728	0.003622414	0.027984147	21750.00205888815020327
NONHSAG042907	-0.0686749	0.100935235	0.369561988	-0.11428767	0.101114573	0.350159272	21760.165989377804142
NONHSAG042913	0.058530333	0.306697511	0.644626559	-0.11428916	0.438206358	0.751822013	21770.56245808510253
NONHSAG042922	0.052050871	0.41242595	0.722776977	-0.11455444	0.408952448	0.729548907	21780.633679893810184
NONHSAG042934	-0.20133318	0.01040518	0.068387528	-0.11455876	0.000789028	0.007546757	21790.00230471882483785
NONHSAG042947	0.15577801	0.093914174	0.355119277	-0.11462021	0.017388842	0.103377218	21800.0500435850305492
NONHSAG042959	0.133726846	0.047898382	0.221291545	-0.11470268	0.583409292	0.836050856	21810.0330833537974911
NONHSAG042979	0.038652869	0.598033425	0.847002984	-0.11478429	0.478198457	0.781355214	21820.759660328481613
NONHSAG042982	0.010978609	0.858052642	0.950687167	-0.11481275	0.782759527	0.918192715	21830.899281093134239
NONHSAG042983	0.017930342	0.71285371	0.898275053	-0.11490626	0.142001928	0.428365084	21840.306146633620786
NONHSAG042995	-0.02073933	0.872057497	0.952744419	-0.11502324	0.140749428	0.426495034	21850.26769297414708
NONHSAG043003	0.044608091	0.586619131	0.842139475	-0.11528067	0.373371235	0.700534004	21860.664892992458698
ELOVL2-AS1	-2.06343703	2.95E-18	5.92E-16	-0.1153385	2.86E-11	1.83E-09	28101.87602356063223e-17
NONHSAG043014	0.068481771	0.295088424	0.632460859	-0.11542014	0.068310503	0.276785497	21870.183728364734182
NONHSAG043016	0.070836736	0.28346555	0.619797841	-0.11560803	0.366674522	0.69386861	21880.510969343899537

NONHSAG043027	0.146740076	0.074274025	0.300948932	-0.11574123	0.09493145	0.337302272	21890.135729871304158
NONHSAG043033	-0.00470537	0.945295639	0.979231332	-0.11592955	0.84364207	0.941306101	21900.962118413525686
NONHSAG043043	-0.21513676	0.00018055	0.002014705	-0.11606318	0.012410144	0.079312104	21910.000717861786564969
NONHSAG043047	0.128191267	0.098367548	0.364439453	-0.11624106	0.000755555	0.007251266	21920.00314117791744467
NONHSAG043071	0.130180898	0.099297742	0.365000326	-0.11637094	0.150565576	0.437386776	21930.198806346407538
NONHSAG043074	0.101176973	0.246528076	0.583317506	-0.11649962	0.609896565	0.84776527	21940.50429967918807
NONHSAG043077	0.137515721	0.056406132	0.249785895	-0.11656923	0.128520773	0.405612137	21950.130043750938071
NONHSAG043093	-0.07331537	0.246969172	0.583317506	-0.11672084	0.462292424	0.771493351	21960.499037708210905
NONHSAG043129	0.140813263	0.050717277	0.231309391	-0.11688836	0.049598813	0.222798502	21970.0781787534139679
NONHSAG043135	0.327665073	0.000147706	0.001681571	-0.11700163	0.178106628	0.482403482	21980.000527104323725438
NONHSAG043144	0.15722358	0.038169593	0.187406018	-0.11702693	0.807826204	0.929984142	21990.0416055302987553
NONHSAG043195	-0.08631425	0.1930715	0.525573145	-0.11706746	0.773174171	0.91321261	22000.38781313659071
NONHSAG043203	-0.14264006	0.083319615	0.324507975	-0.11735223	0.004280485	0.032356781	22010.0154215529369421
NONHSAG043218	-0.02432899	0.627788296	0.85822947	-0.11737427	0.83247055	0.935240586	22020.773075399978413
NONHSAG043252	-0.00536976	0.930581315	0.974203743	-0.11748109	0.51039765	0.802705923	22030.714897970051074
NONHSAG043277	0.00679384	0.931242445	0.974203743	-0.11783026	0.134897247	0.418226083	22040.205660026221236
NONHSAG043281	-0.01539707	0.780684682	0.920990125	-0.1180257	0.685946424	0.889705418	22050.788236965521233
NONHSAG043292	0.190416952	0.029915747	0.156362602	-0.1180649	0.298305526	0.627870613	22060.0912800430592997
NONHSAG043304	0.027579488	0.65963661	0.875035827	-0.11852703	0.659003961	0.878826774	22070.876952874498272
NONHSAG043313	0.053132368	0.405113443	0.715315958	-0.11867046	0.094344754	0.336361438	22080.241852971190877
NONHSAG043379	0.104197245	0.113360876	0.39613292	-0.11869895	0.261592912	0.592856869	22090.262301804780573
NONHSAG043614	0.286223595	0.000264439	0.002827383	-0.11911264	0.00580658	0.042632124	22100.000810795873242952
NONHSAG043616	-0.02741131	0.702564383	0.895955243	-0.11914355	0.522914626	0.80637524	22110.58633251064468
NONHSAG043628	0.152190551	0.006319923	0.044652321	-0.11950014	0.005805452	0.042632124	22120.00723785306734654
NONHSAG043629	0.185957421	0.000769171	0.0072097	-0.11968775	0.002080673	0.017517526	22130.00115603276807475
NONHSAG043634	-0.01634715	0.773282797	0.918276503	-0.11976946	0.539824794	0.812140251	22140.652887485349822
NONHSAG043637	0.223570375	0.000721528	0.006831438	-0.12004463	0.030588583	0.154856439	22150.00281509802996135
NONHSAG043646	0.104741955	0.121682845	0.411759519	-0.12009372	0.130874014	0.410276175	22160.209133886352494
NONHSAG043677	-0.06603708	0.33002895	0.669162012	-0.120705	0.283313431	0.613773011	22170.491707032244019
NONHSAG043699	-0.11308842	0.098969658	0.36482008	-0.12092566	0.395817252	0.718406651	22180.251448738373564
NONHSAG043706	0.259984246	0.126590787	0.422771134	-0.12118072	0.000287998	0.003175882	22190.00116370896712803
NONHSAG043726	0.030529246	0.581668382	0.840519779	-0.12118137	0.552370002	0.820548741	22200.518834465554908
NONHSAG043748	-0.02210697	0.810068656	0.93439361	-0.12142253	0.950845555	0.979405751	22210.968883302782731
NONHSAG043765	0.125862188	0.176580378	0.501559619	-0.12155996	0.726520254	0.902772848	22220.368691634805432
NONHSAG043790	0.273896278	1.82E-05	0.000249894	-0.12164402	1.14E-07	2.75E-06	22232.96533839387853e-07
NONHSAG043821	0.038877169	0.632303967	0.862680714	-0.12171521	0.001761034	0.015244881	22240.00340959732371809
NONHSAG043825	0.019827893	0.735405995	0.905538909	-0.12186022	0.44208797	0.753473247	22250.739947990649837
NONHSAG043835	-0.05683721	0.305108828	0.643152942	-0.12222949	0.302571162	0.632117464	22260.128347422977182
NONHSAG043845	-0.00074504	0.991561305	0.996279605	-0.12227718	0.724512234	0.902489903	22270.921725176100039
NONHSAG043849	-0.05903605	0.352465935	0.680247895	-0.1223449	0.219723446	0.53727159	22280.436882905793729
NONHSAG043854	-0.02895322	0.581449469	0.840519779	-0.12256952	0.668990508	0.880630922	22290.616588729896493
NONHSAG043856	0.047843253	0.581607418	0.840519779	-0.1232218	0.891720533	0.958708067	22300.846092535903602
NONHSAG043865	0.05685315	0.288236196	0.62446068	-0.12342297	0.043754063	0.203841567	22310.127135364422099
NONHSAG043905	-1.36945113	2.31E-14	1.80E-12	-0.12346484	1.25E-08	3.85E-07	22321.31806915627907e-13
NONHSAG043910	0.083291199	0.298854375	0.637121257	-0.12352692	0.623959672	0.855234958	22330.298351958753932
NONHSAG043913	0.073165834	0.159336468	0.473130041	-0.12376315	0.02769454	0.145292997	22340.0815714684538987
NONHSAG043916	0.139218587	0.026597978	0.142463839	-0.12397047	0.141766921	0.428365084	22350.0771363645002973
NONHSAG043927	0.006680349	0.943508143	0.978915178	-0.1240125	0.846103137	0.942026436	22360.962571875350271
NONHSAG043945	-0.00373682	0.95471087	0.983194555	-0.12432078	0.155167855	0.444782883	22370.244949003756259
NONHSAG043980	0.031580778	0.659430893	0.875035827	-0.12453771	0.747396742	0.906696003	22380.744056393309688
NONHSAG043986	-0.03733845	0.56858415	0.833172814	-0.1245624	0.209579888	0.522000572	22390.448889400749546
NONHSAG044024	0.085391214	0.246838436	0.583317506	-0.12462213	0.403508254	0.724100325	22400.485603617215131
NONHSAG044064	-0.13268634	0.299052271	0.637121257	-0.12517331	0.751845657	0.906886268	22410.562734642131025
NONHSAG044066	-0.03126966	0.611018385	0.851851115	-0.12548168	0.546181011	0.816079173	22420.537635848407177
NONHSAG044070	0.081521482	0.261187481	0.6018673	-0.12550635	0.262012172	0.592856869	22430.428644730333003
NONHSAG044072	-0.032141	0.587637289	0.842139475	-0.12568773	0.538135411	0.812140251	22440.795296246332662
NONHSAG044100	0.977349651	5.19E-08	1.17E-06	-0.12571527	3.80E-07	8.62E-06	22453.78760773893333e-08
NONHSAG044111	0.026408473	0.702336301	0.895955243	-0.12610144	0.998667213	0.999022484	22460.905627862720961
NONHSAG044194	-0.07777953	0.292991716	0.630465061	-0.12628963	0.493934536	0.792323968	22470.222217801769722
NONHSAG044196	-0.05299629	0.360722007	0.687229189	-0.1268929	0.14933189	0.43515158	22480.340694324486902
NONHSAG044199	-0.02843061	0.654033984	0.872584313	-0.12693682	0.475042919	0.780269093	22490.767817394891073
NONHSAG044203	-0.04563046	0.574571645	0.836650451	-0.12708649	0.546942638	0.8161186	22500.794999913765208
NONHSAG044217	0.81408337	1.70E-10	6.51E-09	-0.1273054	7.45E-10	3.18E-08	22515.94165777878229e-11
NONHSAG044226	-0.01360035	0.861318505	0.951382127	-0.12746186	0.829404172	0.933749876	22520.925557308265865
NONHSAG044243	0.023017864	0.73683281	0.905582981	-0.12779328	0.621574101	0.853798275	22530.879057931351999
NONHSAG044245	-0.02383595	0.72975763	0.903559887	-0.12827459	0.777072788	0.915428857	22540.818567579082675
NONHSAG044258	0.150592763	0.17384809	0.498329082	-0.12833096	0.366273193	0.69386861	22550.379525431346075
NONHSAG044264	0.030925555	0.695530798	0.89238585	-0.12858165	0.752232932	0.906886268	22560.916332694895153
NONHSAG044265	-0.02271029	0.719845972	0.901651169	-0.12862375	0.851501911	0.943801094	22570.855877987220479
NONHSAG044266	-0.11088548	0.098867594	0.36482008	-0.12882302	0.993106543	0.997026029	22580.163700873485329
NONHSAG044270	-0.04502296	0.496903426	0.783235669	-0.12894616	0.926090641	0.971770056	22590.7595072690424

NONHSAG044283	-0.35500819	0.000241489	0.002611791	-0.12911419	0.199710191	0.507303573	22600.000845661792728096
NONHSAG044290	0.101046023	0.080154865	0.315679945	-0.12917734	0.44921458	0.756417348	22610.209318866355815
NONHSAG044304	0.436184024	5.59E-05	0.000705521	-0.12934706	0.000720958	0.006966782	22620.000117907080727573
NONHSAG044336	-0.06549332	0.457701002	0.756971211	-0.12959089	0.06503329	0.268931782	22630.173639412508093
NONHSAG044342	0.053569321	0.280335053	0.61586107	-0.12962196	0.234020978	0.557433838	22640.41817070398484
NONHSAG044349	0.207947805	0.025804251	0.139006808	-0.12987956	0.033226146	0.163915654	22650.0429276432874215
NONHSAG044369	-0.05678561	0.318271614	0.656624929	-0.13022143	0.208425633	0.520061068	22660.410564454555406
NONHSAG044447	0.161157497	0.005753716	0.041379663	-0.13084446	0.012627457	0.080517932	22670.0103600734566476
NONHSAG044503	0.010068495	0.878668775	0.954268263	-0.13086263	0.791385537	0.921713034	22680.964867351380098
NONHSAG044507	-0.01605251	0.804469485	0.932342929	-0.13087237	0.806748109	0.929744132	22690.959880340172659
NONHSAG044512	0.210758956	0.000189083	0.002101581	-0.13100745	0.009479386	0.063638173	22700.000704840162221367
NONHSAG044540	0.041432282	0.564970771	0.830383739	-0.13102754	0.16212172	0.456342619	22710.367050078007899
NONHSAG044561	0.059920923	0.368936238	0.697210149	-0.13196499	0.379086687	0.70595481	22720.211980562287329
NONHSAG044585	0.028939234	0.622321011	0.857660281	-0.13198536	0.110496063	0.367709977	22730.257768317595931
NONHSAG044626	0.012993932	0.867841909	0.951960393	-0.1322266	0.478275952	0.781355214	22740.646716184205227
NONHSAG044627	0.018858805	0.783360464	0.922063468	-0.1326414	0.722986755	0.902489903	22750.817799026796175
NONHSAG044671	0.013928973	0.837755463	0.944345467	-0.13314745	0.908407583	0.965032915	22760.948210391137477
NONHSAG044686	-0.04750158	0.349308332	0.677785637	-0.13321806	0.070006727	0.281227022	22770.0248988584179055
NONHSAG044742	0.326981732	8.65E-06	0.000122918	-0.13336294	1.89E-06	3.68E-05	22781.63759302244176e-06
NONHSAG044767	-0.05630728	0.554089848	0.821852872	-0.13340572	0.106965581	0.361523093	22790.259138633385308
NONHSAG044790	0.014170156	0.858876518	0.951071568	-0.1336596	0.052370335	0.230462255	22800.0991618088564058
NONHSAG044806	0.504927218	0.000984752	0.008990662	-0.13375814	1.12E-05	0.000176384	22813.77837425429776e-05
NONHSAG044810	0.120949557	0.054914262	0.244720927	-0.13466069	0.170127936	0.469019369	22820.139756610677492
NONHSAG044811	0.049922665	0.437111076	0.74045563	-0.13500019	0.541788909	0.812737703	22830.712604914309574
NONHSAG044815	0.026674044	0.54275353	0.817035827	-0.13507697	0.439021265	0.752760852	22840.714380562803661
NONHSAG044846	0.127476924	0.009380931	0.062807565	-0.13514341	0.043928873	0.203841567	22850.0247077015351234
NONHSAG044851	0.043398711	0.423095866	0.727871783	-0.1355645	0.37848686	0.705772579	22860.618920650161771
NONHSAG044892	-0.11598992	0.075279095	0.302839507	-0.13559776	0.976927866	0.990519194	22870.117611808088977
NONHSAG044894	0.037753055	0.671991445	0.884442916	-0.13588236	0.892756474	0.958708067	22880.842267512398928
NONHSAG044918	-0.17847418	0.022208265	0.123238983	-0.13591243	0.255652679	0.586701827	22890.0704630411723306
NONHSAG044920	-0.12334482	0.138473154	0.443294502	-0.13592942	0.600686873	0.840782223	22900.317877616409287
NONHSAG044929	0.330760116	1.29E-05	0.000181285	-0.13662543	8.49E-05	0.001056315	22911.68629539379661e-05
NONHSAG044999	0.020940699	0.692117539	0.892358789	-0.13668394	0.997852864	0.998563079	22920.89881706162494
NONHSAG045020	0.004545688	0.935868097	0.975411819	-0.13688925	0.319631405	0.651852579	22930.538890055252174
NONHSAG045068	0.014735793	0.825036854	0.938461188	-0.13730644	0.49708953	0.794835843	22940.641952029016494
NONHSAG045080	0.034695348	0.615680332	0.85369482	-0.13779211	0.973236624	0.989451568	22950.833400339708549
NONHSAG045081	0.314196888	0.000830947	0.007711628	-0.13802622	0.000110254	0.00131929	22960.000203088264741546
NONHSAG045085	-0.08747116	0.210916123	0.544124897	-0.13820213	0.942076158	0.976126639	22970.36962746100207
NONHSAG045101	-0.04723139	0.443485587	0.746755372	-0.13860679	0.966708672	0.986709541	22980.686673202098054
NONHSAG045116	-0.08387841	0.221704159	0.555148792	-0.13869618	0.219313755	0.53727159	22990.364995421581522
NONHSAG045138	0.036664341	0.552655056	0.821852872	-0.13916375	0.494865368	0.792912487	23000.443344405844362
NONHSAG045167	0.051164908	0.49345043	0.780856843	-0.13980725	0.734613303	0.905644804	23010.57914371280367
NONHSAG045192	0.063826867	0.486126441	0.774497197	-0.14033144	0.537430639	0.812140251	23020.745141917370525
NONHSAG045208	-0.37633404	0.001145538	0.010362948	-0.14043774	0.002442838	0.020144458	23030.00161816994196284
NONHSAG045235	0.068539078	0.480102763	0.76981996	-0.14055502	0.620340981	0.853798275	23040.766206770800944
NONHSAG045258	0.138545466	0.12091296	0.411133305	-0.14056756	0.000335921	0.003537864	23050.00137263073519842
NONHSAG045265	0.061902029	0.484403189	0.773504695	-0.14075797	0.357646865	0.686695518	23060.627026825483056
NONHSAG045281	0.026038203	0.716580214	0.899162678	-0.14153988	0.48785096	0.786603727	23070.560779423571258
NONHSAG045316	-0.01578609	0.755551292	0.912354301	-0.14161254	0.199653552	0.507303573	23080.403549362134222
NONHSAG045329	-0.03870756	0.487425533	0.775687946	-0.14166372	0.901243868	0.962240579	23090.757237948669391
NONHSAG045333	-0.40444658	0.001963124	0.01633226	-0.14184395	0.001823636	0.015682156	23100.00190707563865332
NONHSAG045338	0.147849406	0.035230011	0.175962327	-0.14206394	0.333962905	0.67010912	23110.104740313683905
NONHSAG045350	0.05939435	0.307518832	0.645339438	-0.14258259	0.019570815	0.113236899	23120.0618205275584899
NONHSAG045363	0.078756252	0.281499262	0.617755353	-0.14262657	0.520181451	0.805982248	23130.55155635285368
NONHSAG045364	0.113901658	0.114649204	0.39949636	-0.1426783	0.031970067	0.159679978	23140.083864464327599
NONHSAG045389	0.001123471	0.986438224	0.996279605	-0.14279462	0.310681811	0.639585873	23150.495062040810132
NONHSAG045403	0.063965794	0.334747018	0.671404147	-0.1432475	0.694403648	0.895088581	23160.620345723374415
NONHSAG045405	-2.25691709	1.30E-17	2.04E-15	-0.14327139	2.58E-12	2.02E-10	23175.56003990949711e-17
NONHSAG045408	-0.26097222	0.002151711	0.017743725	-0.14334479	0.270019525	0.600197292	23180.00730871607382473
NONHSAG045419	0.058847494	0.493790973	0.780956252	-0.14342986	0.48109863	0.783450231	23190.383465110280462
NONHSAG045425	-0.10714575	0.148801567	0.458302307	-0.14390474	0.889918827	0.958708067	23200.215684048375068
NONHSAG045429	0.052397462	0.386981809	0.706496807	-0.14403853	0.240751037	0.567946237	23210.473380141678402
NONHSAG045445	-0.07354094	0.385569246	0.706496807	-0.14452328	0.848763108	0.942623167	23220.52729248516633
NONHSAG045449	0.07298903	0.139199518	0.443294502	-0.14522987	0.737323432	0.906043339	23230.158290808318581
NONHSAG045463	-0.02137863	0.794963359	0.927815057	-0.14539961	0.061204372	0.256492839	23240.0696587443305835
NONHSAG045467	0.074909042	0.241988216	0.575614548	-0.14560993	0.691483356	0.892769145	23250.487433281719758
NONHSAG045472	0.092619715	0.154754987	0.466921698	-0.14564936	0.270430288	0.600197292	23260.325149558119692
NONHSAG045505	-0.00531481	0.948161644	0.98065537	-0.14594863	0.234627453	0.558183079	23270.367337611164402
NONHSAG045512	0.062583675	0.459630514	0.758498242	-0.14614847	0.016827546	0.100252246	23280.0477178594528619
NONHSAG045517	-0.05900367	0.513034501	0.797486466	-0.14632149	0.773348828	0.91321261	23290.804073263608296
NONHSAG046716	0.100737332	0.187950098	0.516279483	-0.14654745	0.111864292	0.369410385	23300.233777408145657

NONHSAG046750	0.0411501	0.479223555	0.76981996	-0.14680817	0.807425249	0.929984142	23310.611792741125617
NONHSAG046781	0.086900062	0.226965456	0.558671887	-0.1468486	0.949892314	0.979141197	23320.393094453899907
NONHSAG046784	0.007440048	0.909723759	0.965525488	-0.14699349	0.819678673	0.933749876	23330.97398892819756
NONHSAG046797	0.026413598	0.581436636	0.840519779	-0.14703562	0.05556646	0.238918787	23340.139324366478189
NONHSAG046806	0.038059601	0.465691951	0.763571876	-0.14708382	0.232211326	0.556934066	23350.479702533161379
NONHSAG046817	-0.07473183	0.448975643	0.751820679	-0.14768643	0.94666386	0.977208575	23360.654988660278776
NONHSAG046838	-0.10385691	0.139110733	0.443294502	-0.14778733	0.031666827	0.158729266	23370.0879035627054196
NONHSAG046851	0.119606832	0.111623224	0.39284669	-0.14790705	0.121694465	0.39109124	23380.192579738119815
NONHSAG046903	0.020870523	0.730045542	0.903559887	-0.14821346	0.39533463	0.718406651	23390.690151982325983
NONHSAG046927	-0.04131249	0.54060395	0.816860993	-0.14850824	0.151237022	0.438088981	23400.348904285062435
NONHSAG046961	0.075263667	0.277789339	0.612912715	-0.14862771	0.228631772	0.551382969	23410.411592651154856
NONHSAG047021	-0.01016698	0.863952147	0.951839172	-0.14886846	0.516936295	0.804430331	23420.795509751459693
NONHSAG047026	0.046511119	0.525958359	0.806871198	-0.14896873	0.192485389	0.499786624	23430.422428545254447
NONHSAG047060	0.065223149	0.365706428	0.693811657	-0.14901372	0.064010458	0.265091912	23440.0237898178784344
NONHSAG047082	-0.00683005	0.917585847	0.968261389	-0.14914373	0.792553362	0.922059223	23450.965015026514838
NONHSAG047088	-0.0150531	0.843665019	0.94647636	-0.14914779	0.886243731	0.957769935	23460.942571180121283
NONHSAG047097	-0.38903801	0.012274083	0.077735857	-0.1494976	0.031790792	0.159067095	23470.0262442944551075
NONHSAG047101	-0.09234037	0.177571477	0.502625892	-0.14954389	0.432302616	0.747927033	23480.104398014568324
NONHSAG047126	0.036232831	0.575404999	0.836650451	-0.14969605	0.290804962	0.620442756	23490.567523414928454
NONHSAG047149	-0.01172673	0.854763394	0.949662955	-0.14999681	0.81750735	0.932966992	23500.91661614116096
NONHSAG047152	0.046487767	0.458890052	0.758072461	-0.1501656	0.911999168	0.967390382	23510.648951276769161
NONHSAG047169	-0.00629985	0.917055919	0.968261389	-0.15088053	0.754170433	0.907341414	23520.949714661283718
NONHSAG047175	-0.14988871	0.01649525	0.097446731	-0.15158438	0.058817969	0.248715983	23530.041376499363446
NONHSAG047181	0.249852513	4.32E-05	0.000554281	-0.15187725	1.92E-06	3.72E-05	23543.35978759194718e-06
NONHSAG047192	0.228297594	0.007238076	0.05050489	-0.15200488	0.000272985	0.00303413	23550.0008889594110294
NONHSAG047193	-0.07163626	0.216818452	0.549316015	-0.15212725	0.620161394	0.853798275	23560.456518720769705
NONHSAG047194	0.123115032	0.06063121	0.26149534	-0.15226961	0.022170148	0.123206434	23570.0515175984542341
NONHSAG047208	-0.04911726	0.438468047	0.740968838	-0.15238464	0.269144983	0.599281359	23580.521494458032146
NONHSAG047221	-0.06010916	0.345460408	0.677284107	-0.15391936	0.515860684	0.804430331	23590.623736743377317
NONHSAG047229	-0.12706869	0.112769686	0.394974341	-0.15403835	0.990909644	0.997026029	23600.184126049132746
NONHSAG047232	0.061000242	0.348580524	0.677785637	-0.15405269	0.271672736	0.601363513	23610.491025050126118
NONHSAG047253	0.012264808	0.839683146	0.944963841	-0.15422628	0.113150684	0.371705285	23620.222933877658453
NONHSAG047257	-0.07970269	0.416571858	0.724427994	-0.15440851	0.285925322	0.615425223	23630.17582480807811
NONHSAG047259	0.010980682	0.887687966	0.956660206	-0.15462241	0.287446101	0.616551056	23640.508812410595651
NONHSAG047273	-0.04918729	0.601726526	0.848669165	-0.15529771	0.300904002	0.629569979	23650.288384027158027
NONHSAG047282	-0.05338577	0.318638315	0.656899517	-0.15535479	0.086698778	0.31941043	23660.223438989643834
NONHSAG047295	-0.18167999	0.010538652	0.068917884	-0.15558651	0.094406455	0.336361438	23670.0336590463509507
NONHSAG047314	0.127477638	0.194176571	0.526251063	-0.15572266	0.191469828	0.498018695	23680.320558285167041
NONHSAG047336	0.210645596	0.007673072	0.053013955	-0.15655201	0.07649522	0.296287269	23690.0248331387834973
NONHSAG047348	0.001380201	0.984069455	0.995038946	-0.15659019	0.554417344	0.821343134	23700.783426752767755
NONHSAG047356	0.016774613	0.796812876	0.927815057	-0.15723486	0.52264869	0.80637524	23710.810657319014935
NONHSAG047375	-0.0637637	0.453236999	0.755484553	-0.15726241	0.41493321	0.733932722	23720.297707856435085
NONHSAG047399	0.01804226	0.742218253	0.909586883	-0.15740299	0.294406862	0.623485995	23730.557924437604292
NONHSAG047416	0.070801587	0.308256711	0.646396622	-0.15789875	0.686912505	0.890259682	23740.58602412008084
NONHSAG047425	0.007185842	0.917249629	0.968261389	-0.15848045	0.265963394	0.596878743	23750.466038282948807
NONHSAG047431	0.082895868	0.26929208	0.605799463	-0.15860691	0.666547245	0.880630922	23760.533005908978939
NONHSAG047443	-0.00699389	0.911595545	0.966229428	-0.15873891	0.192674746	0.499816777	23770.289753293057863
NONHSAG047447	0.037106835	0.569956261	0.834313902	-0.15886788	0.118974882	0.384865961	23780.282437328703381
NONHSAG047469	0.067813676	0.467845889	0.764261292	-0.1594147	0.844002801	0.941306101	23790.75126994610624
NONHSAG047503	0.13304987	0.029521538	0.154877918	-0.16009675	0.096078128	0.340266618	23800.0747498464862217
NONHSAG047517	0.06228234	0.178135104	0.502625892	-0.16076166	0.246330331	0.574769231	23810.342338310874089
NONHSAG047524	0.041473542	0.562323503	0.829618935	-0.16098007	0.351895102	0.684429948	23820.639007264985531
NONHSAG047536	-0.02066282	0.709017984	0.898275053	-0.16102794	0.313741973	0.643502864	23830.590698045458364
NONHSAG047547	0.469936855	5.06E-06	7.81E-05	-0.16165481	0.000529602	0.005395805	23841.65916254146141e-05
NONHSAG047553	-0.01329074	0.880125766	0.954268263	-0.16170327	0.712237103	0.897756559	23850.865342317308647
NONHSAG047570	0.262740057	0.000126772	0.001449123	-0.16171749	0.000307499	0.003338559	23860.000139193884783516
NONHSAG047611	-0.02532577	0.654576227	0.872584313	-0.16242404	0.162752839	0.456747487	23870.161689018164537
NONHSAG047665	0.054801417	0.412538068	0.722776977	-0.16246747	0.489618292	0.788812626	23880.674990887914773
NONHSAG047674	-0.06889316	0.323403354	0.663803088	-0.16258376	0.993115804	0.997026029	23890.515829837537149
NONHSAG047730	0.055507009	0.324508011	0.664131387	-0.16258999	0.374431938	0.700534004	23900.551167644292064
NONHSAG047747	0.132319732	0.138707458	0.443294502	-0.16302313	0.025088912	0.135139117	23910.0733199482404418
NONHSAG047777	-0.79514392	1.71E-10	6.51E-09	-0.1635711	1.79E-08	5.31E-07	23922.27123934368851e-10
NONHSAG047807	0.194369184	0.020161773	0.114304243	-0.16434771	0.02210736	0.123100786	23930.0298696894211614
NONHSAG047837	-0.11714899	0.233145335	0.566641903	-0.16498491	0.560346336	0.825835376	23940.48618491081863
NONHSAG047842	0.12226475	0.179710804	0.504113961	-0.16504645	0.043866052	0.203841567	23950.119830915322087
NONHSAG047845	-0.06390401	0.56506952	0.830383739	-0.16529957	0.436671272	0.750562114	23960.719042207662423
NONHSAG047847	0.027728697	0.757717489	0.913419123	-0.16541078	0.930380356	0.973855106	23970.950006250922625
NONHSAG047907	0.248674267	0.0012861	0.011444662	-0.16548066	0.034184526	0.167177196	23980.00472377926750239
NONHSAG048011	-0.14581544	0.044538776	0.210846866	-0.16561947	0.201260045	0.509172255	23990.124070500863544
NONHSAG048092	-0.00879785	0.883459933	0.955834483	-0.16601911	0.208431303	0.520061068	24000.303036763313148
NONHSAG048098	-0.0308722	0.638344841	0.865907232	-0.16627798	0.612257225	0.849433959	24010.619227485004661

NONHSAG048120	-0.13314351	0.093154425	0.354512009	-0.16633964	0.673129446	0.881776357	24020.21292194909767
NONHSAG048123	0.022084695	0.762904509	0.915083941	-0.16662823	0.86070679	0.947290977	24030.954505402641757
NONHSAG048148	0.047304048	0.371280146	0.698354362	-0.16688941	0.012207317	0.078372092	24040.0376133539614945
NONHSAG048155	-0.04390685	0.658913333	0.875035827	-0.16692974	0.955598849	0.981541511	24050.889414173808478
NONHSAG048166	-0.04391271	0.533468254	0.812788416	-0.16698165	0.294936998	0.623681777	24060.569820589642374
NONHSAG048202	0.065695406	0.355549796	0.681995925	-0.16704536	0.770756112	0.91321261	24070.635927593392971
NONHSAG048386	0.064359341	0.35171604	0.679813037	-0.16710022	0.33919213	0.672365575	24080.548955479773697
NONHSAG048397	0.611705004	3.60E-08	8.29E-07	-0.1673918	7.30E-07	1.57E-05	24093.96485452836649e-08
NONHSAG048404	0.097421012	0.178128197	0.502625892	-0.16784312	0.000835543	0.007937658	24100.00316071308221511
NONHSAG048405	-0.09903579	0.085024737	0.32887704	-0.16810903	0.2844023	0.615265887	24110.216779428391883
NONHSAG048439	-0.0394524	0.523126985	0.803843214	-0.16816377	0.856167452	0.946361193	24120.687930569413973
NONHSAG048458	0.007729079	0.898600602	0.961516322	-0.16875107	0.143857571	0.430061982	24130.26661119051705
NONHSAG048471	0.812241568	3.15E-08	7.39E-07	-0.16884015	1.15E-08	3.59E-07	24144.50702640997695e-09
NONHSAG048506	-0.02459872	0.754246029	0.911448145	-0.16885628	0.790215635	0.921569044	24150.943848554736281
NONHSAG048520	0.085451562	0.376521039	0.701176928	-0.16886743	0.19685809	0.504520098	24160.414539096470311
NONHSAG048562	0.04548154	0.436536907	0.74045563	-0.1689513	0.101670796	0.350159272	24170.256555080708419
NONHSAG048563	0.449158647	2.83E-06	4.52E-05	-0.16918087	3.71E-06	6.52E-05	24181.35350616932187e-06
NONHSAG048569	0.21219637	0.067172388	0.282344926	-0.169755	0.814792573	0.931603479	24190.135281339088114
NONHSAG048593	-0.07524047	0.235781623	0.569603028	-0.1701059	0.620089645	0.853798275	24200.486921625696998
NONHSAG048597	-0.1009464	0.128225375	0.42450856	-0.17013265	0.869989162	0.95043105	24210.244875154546413
NONHSAG048604	-0.05534635	0.45684103	0.756971211	-0.17076682	0.150112184	0.436929622	24220.349760416051539
NONHSAG048605	0.043238902	0.635045903	0.862680714	-0.17096448	0.742542684	0.906043339	24230.887078269478411
NONHSAG048651	-0.0417885	0.471622387	0.764822463	-0.17211092	0.015941911	0.096198829	24240.0446684582124924
NONHSAG048682	0.16245437	0.021763509	0.121667968	-0.17224621	0.178242254	0.482403482	24250.068105639865478
NONHSAG048687	-0.04249873	0.434846585	0.738589088	-0.17229494	0.974038067	0.989451568	24260.651330118232816
NONHSAG048746	0.417825604	0.000670854	0.006438369	-0.1731776	0.000555994	0.005560938	24270.000520031196203811
NONHSAG048747	0.4281296	6.42E-06	9.54E-05	-0.17339895	3.91E-05	0.000527982	24287.40507306169244e-06
NONHSAG048781	0.134941452	0.010408912	0.068387528	-0.17369092	0.110249581	0.367694759	24290.0342939305731364
NONHSAG048790	-0.04579426	0.592464391	0.843549299	-0.17380039	0.768263275	0.913083825	24300.700036318185982
NONHSAG048794	-0.04594767	0.528340099	0.80903302	-0.17442926	0.288585614	0.617112355	24310.236652194300817
NONHSAG048813	0.000616205	0.992588826	0.996592142	-0.17449256	0.523627734	0.80637524	24320.76291176864434
NONHSAG048817	0.210850511	0.005050138	0.037078298	-0.17499615	0.004327031	0.03262094	24330.00537691592414362
NONHSAG048836	0.117598106	0.157703249	0.471266245	-0.17610959	0.937004108	0.975416231	24340.243676732222275
NONHSAG048840	-0.27341603	0.019494702	0.111194932	-0.17733589	0.158516642	0.451836895	24350.0612003957504306
NONHSAG048846	0.066876192	0.385729938	0.706496807	-0.1775138	0.00317506	0.025292542	24360.00937869529233374
NONHSAG048902	0.141226728	0.056179403	0.249174261	-0.17756855	0.254046836	0.58512763	24370.154579858466882
NONHSAG048962	0.073120991	0.11481106	0.399565225	-0.17924425	0.791584985	0.921713034	24380.140351938875833
NONHSAG048976	0.309513501	0.000103707	0.001205056	-0.17942528	2.12E-05	0.000302878	24392.57711391616743e-05
NONHSAG048984	0.048572321	0.455613645	0.75675462	-0.18039861	0.708828072	0.896707014	24400.754039136718869
NONHSAG048993	-0.02557312	0.665017512	0.879303057	-0.180632	0.476732227	0.780309094	24410.512460012263593
NONHSAG049003	0.158491109	0.06310844	0.268880204	-0.18109753	0.097584009	0.343973343	24420.124818269454493
NONHSAG049010	-0.00223374	0.967352761	0.987996581	-0.1822644	0.348073654	0.680716946	24430.53835711403281
NONHSAG049037	0.237800215	4.32E-05	0.000554281	-0.18247988	0.001779939	0.015353336	24440.00013314501330791
NONHSAG049079	-0.56338746	1.92E-09	5.93E-08	-0.1825889	1.44E-07	3.40E-06	24452.71369052302078e-09
NONHSAG049095	-0.33977152	0.002196484	0.018007328	-0.18307664	0.000427945	0.004391902	24460.000800196047466501
NONHSAG049099	0.036961968	0.420295275	0.726032559	-0.18318654	0.483387678	0.783450231	24470.678120337344229
NONHSAG049119	0.034108709	0.777354877	0.920350018	-0.18395003	0.525292024	0.807170039	24480.814254010527255
NONHSAG049134	0.032284339	0.579880538	0.839662241	-0.184138	0.109193905	0.36622905	24490.260804294893877
NONHSAG049182	0.048229371	0.36571238	0.693811657	-0.18455134	0.180686375	0.484356613	24500.389358003983518
NONHSAG049240	-0.04716126	0.59317143	0.843701599	-0.18477465	0.812306754	0.931253814	24510.729705870108271
NONHSAG049298	-0.43527241	2.30E-06	3.73E-05	-0.18514244	4.65E-05	0.000622789	24523.93900141091717e-06
NONHSAG049309	0.141533745	0.03633487	0.180518826	-0.18515219	0.937592233	0.975416231	24530.060191329112312
NONHSAG049336	0.048490463	0.471173225	0.764534973	-0.1855236	0.208176194	0.520061068	24540.445657175714164
NONHSAG049339	0.043978613	0.408722891	0.718779718	-0.18603894	0.065168451	0.269094984	24550.176819639758818
NONHSAG049341	0.342781945	6.75E-06	9.99E-05	-0.18672145	5.92E-05	0.000764215	24569.26086673242504e-06
NONHSAG049344	0.310111977	8.84E-07	1.53E-05	-0.188308	0.000128488	0.001511751	24572.70648509610961e-06
NONHSAG049350	-0.04017586	0.64067834	0.867640994	-0.18855635	0.677939517	0.884624557	24580.67588518072049
NONHSAG049357	-0.00973613	0.878696776	0.954268263	-0.1886038	0.781674989	0.917391514	24590.908362735612488
NONHSAG049388	0.066597136	0.349842921	0.67798642	-0.19057481	0.89408093	0.959235245	24600.505685555892595
NONHSAG049409	-0.01767891	0.765179804	0.915610897	-0.19123045	0.390240296	0.713031652	24610.679711107902233
NONHSAG049422	-0.15900852	0.043588593	0.207747667	-0.19130171	0.198690946	0.505936125	24620.12170558923522
NONHSAG049424	-0.21381763	0.009350343	0.062752181	-0.1917057	0.55462594	0.821343134	24630.00435091490807501
NONHSAG049464	-0.41551426	0.000338158	0.003483151	-0.19208053	0.001394611	0.012332219	24640.000553834073301412
NONHSAG049487	0.074376348	0.356085154	0.682203965	-0.19215148	0.417667324	0.735463454	24650.598945293748079
NONHSAG049492	-0.05948324	0.305849245	0.64423077	-0.19236716	0.322786189	0.654887998	24660.139419565341091
NONHSAG049507	-0.17946517	0.075768276	0.304371988	-0.19260267	0.39647322	0.718406651	24670.201676985054816
NONHSAG049508	-0.02474452	0.751467176	0.910732246	-0.19360207	0.12624297	0.401707133	24680.14557495866344
NONHSAG049511	-0.00682434	0.927034873	0.973226917	-0.19364427	0.161763128	0.456246655	24690.247028989866214
NONHSAG049515	0.034427865	0.654990912	0.872584313	-0.19416579	0.506352437	0.80071508	24700.792422245227746
NONHSAG049523	-0.15113519	0.016216567	0.096408006	-0.19439665	0.121619619	0.39109124	24710.0503151559026301
NONHSAG049524	0.071123311	0.394162812	0.710658619	-0.19545188	0.06582076	0.271064674	24720.178930337621514

NONHSAG049526	0.016707742	0.817089616	0.938128871	-0.19614977	0.614353732	0.850979373	24730.878923224002695
NONHSAG049528	-0.01231887	0.88075414	0.954404871	-0.19646839	0.209178528	0.521462785	24740.386279985503267
NONHSAG049529	0.114698502	0.085026176	0.32887704	-0.19757679	0.048993004	0.222512561	24750.100287464746068
NONHSAG049530	0.090061752	0.242298491	0.575614548	-0.19800172	0.1120711	0.369410385	24760.254862547389595
NONHSAG049531	0.020937048	0.752668071	0.910732246	-0.1985895	0.17728935	0.48167889	24770.364274931760622
NONHSAG049532	-0.08665846	0.240065815	0.574037853	-0.20034264	0.389941928	0.713031652	24780.131472307226957
NONHSAG049534	-0.05133798	0.512425389	0.796980196	-0.20117033	0.50977228	0.802705923	24790.42312753259215
NONHSAG049539	-0.10320172	0.056963357	0.251067337	-0.20169828	0.014297592	0.088168482	24800.0368904116753409
NONHSAG049594	-0.26291344	0.002124154	0.017568002	-0.2020221	0.001291738	0.011531326	24810.00162113175683912
NONHSAG049595	-0.01351334	0.849181367	0.947934194	-0.20296832	0.819167847	0.933725167	24820.970064566692969
NONHSAG049661	0.035111456	0.537151584	0.813923495	-0.20334654	0.132306424	0.412925266	24830.312767428361605
NONHSAG049665	0.086549988	0.346487349	0.677284107	-0.20336964	0.933477914	0.973855106	24840.577190102524065
NONHSAG049689	-0.02662502	0.710451858	0.898275053	-0.20437524	0.04686678	0.214291686	24850.043346486341221
NONHSAG049734	0.079548361	0.336737333	0.67346619	-0.20690979	0.274848544	0.603336539	24860.48791846629992
NONHSAG049792	0.057637263	0.503680601	0.789138617	-0.20731353	0.421232294	0.738562105	24870.687106768797968
NONHSAG049868	0.183260815	0.000695226	0.006649572	-0.20740762	0.001191812	0.010845871	24880.000814343424006884
NONHSAG049879	0.013543777	0.790950209	0.927116293	-0.20791175	0.641842305	0.867721424	24890.895456690771544
NONHSAG049885	-0.162842	0.016309921	0.096554734	-0.20824687	0.058305966	0.246922252	24900.0409109469602133
NONHSAG049895	0.11655182	0.10857105	0.385969398	-0.20899402	0.131919593	0.41217544	24910.197298663692153
NONHSAG049902	0.339120042	0.0003201	0.003330224	-0.209072	0.000395822	0.004122418	24920.000279918933896206
NONHSAG049904	-0.17713069	0.018801601	0.108142692	-0.20949447	0.149044818	0.43476559	24930.0588559030520883
NONHSAG049930	0.039033247	0.497911916	0.78394642	-0.2101074	0.247927486	0.576651854	24940.185280109153952
NONHSAG049931	0.049801999	0.51198846	0.796980196	-0.21093819	0.412357348	0.733255014	24950.683267172603938
NONHSAG049936	0.03631475	0.575418814	0.836650451	-0.21189407	0.190803987	0.497257471	24960.417082774791267
NONHSAG049939	-0.13729878	0.116520993	0.403518511	-0.21426046	0.654245583	0.87606599	24970.265419423755876
NONHSAG049977	0.030803539	0.69304152	0.89238585	-0.21501974	0.650772244	0.873494773	24980.69629499233565
NONHSAG049980	-0.06502314	0.349056015	0.677785637	-0.21602898	0.225534457	0.547198355	24990.442503698888676
NONHSAG049984	0.140771254	0.099118969	0.36482008	-0.21645091	0.025134338	0.135139117	25000.0671596536347386
NONHSAG049989	0.184166864	0.032067018	0.164114197	-0.21686883	0.174683759	0.475556547	25010.0932679698134323
NONHSAG050013	0.174363816	0.050753163	0.231309391	-0.21689822	0.378879694	0.70595481	25020.143139096076369
NONHSAG050025	0.0889482	0.27925955	0.614939588	-0.21714379	0.355477457	0.686695518	25030.501615630530037
NONHSAG050057	-0.03170809	0.684628931	0.887517619	-0.2175389	0.101454656	0.350159272	25040.100300997558442
NONHSAG050083	-0.08834593	0.143892486	0.449584078	-0.22053044	0.196733335	0.504520098	25050.276774519507228
NONHSAG050088	0.023648883	0.771681214	0.918276503	-0.2205911	0.554230782	0.821343134	25060.837421894793448
NONHSAG050093	-0.0137214	0.89782914	0.961169104	-0.2207464	0.825354359	0.933749876	25070.938795209285109
NONHSAG050175	-0.05517535	0.453634559	0.755699277	-0.2219641	0.925941564	0.971770056	25080.650090734835918
NONHSAG050188	-0.02477769	0.731314605	0.904334507	-0.22244464	0.60188929	0.842046112	25090.6825472649785
NONHSAG050210	0.222999699	0.011453179	0.073718106	-0.2227786	0.013597699	0.084782107	25100.016471720572536
NONHSAG050227	-0.05990054	0.428853373	0.73236115	-0.22287101	0.904379843	0.964031887	25110.60993051171369
NONHSAG050230	0.225056627	0.004257572	0.031911732	-0.22292142	0.001004917	0.009357037	25120.0019386832429451
NONHSAG050243	0.065063015	0.378929916	0.704263664	-0.22293203	0.246504869	0.574769231	25130.477383956749364
NONHSAG050259	-0.0149232	0.773852515	0.918561955	-0.22404833	0.481811339	0.783450231	25140.775863450459608
NONHSAG050280	-0.00084803	0.990746043	0.996279605	-0.22417859	0.858988367	0.947213452	25150.980159640718716
NONHSAG050284	-0.10429129	0.194817694	0.526251063	-0.22488198	0.514693471	0.804430331	25160.426386360061257
NONHSAG050285	-0.05675112	0.446378394	0.750278567	-0.22586391	0.220770173	0.53889386	25170.461099822095547
NONHSAG050296	-0.02341174	0.729006333	0.903322681	-0.22686822	0.700807375	0.895983652	25180.913085682915412
NONHSAG050300	-0.04242108	0.624893366	0.85822947	-0.22689599	0.932775196	0.973855106	25190.823751143330332
NONHSAG050305	1.199181143	1.81E-08	4.47E-07	-0.2274736	5.38E-10	2.40E-08	25205.05941149714532e-10
NONHSAG050335	0.070142715	0.393899609	0.710658619	-0.22832888	0.576603227	0.833674033	25210.683852834220848
NONHSAG050348	-0.02459537	0.719683461	0.901651169	-0.22852422	0.459471952	0.769068529	25220.757594931995802
NONHSAG050370	0.007816837	0.91442184	0.967765982	-0.22965567	0.666586146	0.880630922	25230.903012368356439
NONHSAG050372	0.082417877	0.181500075	0.507839016	-0.23020259	0.07122286	0.282879495	25240.171381235963676
NONHSAG050397	0.342907038	9.06E-05	0.00107016	-0.2314985	0.001017175	0.00943992	25250.00019202983227008
NONHSAG050419	-0.11803033	0.130807309	0.429206713	-0.23185069	0.249283796	0.57772124	25260.284707647068934
NONHSAG050438	0.016242207	0.735395708	0.905538909	-0.23304717	0.099436496	0.347348356	25270.215793589033289
NONHSAG050440	-0.00526102	0.925316145	0.973226917	-0.23398677	0.471849721	0.778445171	25280.670452074266723
NONHSAG050454	-0.0024073	0.975459901	0.99239987	-0.23468766	0.875155268	0.952087485	25290.986034576664758
NONHSAG050461	-0.22257129	0.008539713	0.058002278	-0.23539141	0.893345245	0.958811767	25300.00875669394035699
NONHSAG050473	0.120494599	0.05484789	0.244720927	-0.23546972	0.168673852	0.465924236	25310.139299218405914
NONHSAG050503	-0.0631241	0.413368416	0.723780813	-0.23560655	0.977838294	0.990519194	25320.646320488077126
NONHSAG050531	-0.08094665	0.22481057	0.557958801	-0.23622636	0.786555315	0.920047232	25330.439078762322688
NONHSAG050576	-0.0463765	0.378850211	0.704263664	-0.23708132	0.482469888	0.783450231	25340.645185839450919
RP11-48B3.4	1.564642924	3.78E-17	4.43E-15	-0.23730056	2.26E-15	3.97E-13	25351.91540727465504e-17
NONHSAG050607	0.173979074	0.014450225	0.087573347	-0.23785067	0.165846944	0.462866185	25360.047708006985683
NONHSAG050643	0.065464994	0.30472061	0.643123295	-0.23791934	0.104865466	0.357149038	25370.256472104776991
NONHSAG050690	1.329027951	2.38E-16	2.48E-14	-0.23811157	9.36E-16	2.19E-13	25383.97132863418166e-17
NONHSAG050699	-0.05243299	0.490581906	0.777630395	-0.23875976	0.119660337	0.386320171	25390.0770033089939001
NONHSAG050714	-0.04230986	0.39779063	0.712475957	-0.24075387	0.964425979	0.986709541	25400.632424835984897
NONHSAG050718	-1.2890928	5.04E-13	3.30E-11	-0.24104117	1.35E-10	6.90E-09	25416.47533199591244e-13
NONHSAG050752	0.059139773	0.437850327	0.740968838	-0.24119158	0.705609537	0.896707014	25420.736864020315202
NONHSAG050804	-0.52101863	3.50E-07	6.48E-06	-0.2427427	0.000803883	0.007662776	25431.7754765146375e-06

NONHSAG050806	0.305976956	6.97E-05	0.000855964	-0.24279242	1.24E-05	0.000189424	25441.49729904123382e-05
NONHSAG050815	-0.04740345	0.393423998	0.710538396	-0.24338155	0.261732173	0.592856869	25450.147496078426967
NONHSAG050829	0.091042538	0.224069503	0.556610815	-0.24374192	0.749954411	0.906696003	25460.446594861905082
NONHSAG050841	0.010940836	0.876270961	0.953958166	-0.24425852	0.23258784	0.556934066	25470.426545105214066
NONHSAG050865	0.47050773	8.48E-09	2.31E-07	-0.24490405	8.88E-08	2.26E-06	25486.12346024418139e-09
NONHSAG050866	0.243848005	0.011664741	0.074888702	-0.24535878	0.014528806	0.089398256	25490.0172625313486028
NONHSAG050902	-0.03287448	0.633952576	0.862680714	-0.24570239	0.855016222	0.945832264	25500.791519725991325
NONHSAG050903	0.173240531	0.01841524	0.106772915	-0.24704113	0.085452461	0.317008338	25510.508768297641642
NONHSAG050913	0.193726121	0.00608998	0.043136077	-0.25142021	0.000739865	0.007125004	25520.00181066177331521
NONHSAG050914	0.129351237	0.097729785	0.363033231	-0.25161176	0.142009807	0.428365084	25530.192031399654927
NONHSAG050915	0.141398693	0.073611299	0.299125683	-0.25205923	0.040592591	0.192814805	25540.0835012298786626
NONHSAG050921	-0.0805098	0.167382144	0.489780011	-0.25210735	0.359948112	0.689022526	25550.0763415734972722
NONHSAG050966	0.524591923	5.79E-06	8.67E-05	-0.2522004	8.74E-05	0.001077848	25569.83180525498183e-06
NONHSAG050969	0.127073833	0.068188902	0.284069913	-0.2524391	0.044470927	0.205340307	25570.0853324650753204
NONHSAG050970	0.071607392	0.251232925	0.587254352	-0.25263761	0.992400088	0.997026029	25580.4099194909931
NONHSAG050978	-0.06886295	0.336270604	0.67346619	-0.25283143	0.734591621	0.905644804	25590.402676239616801
NONHSAG050982	0.056651596	0.481361434	0.771275414	-0.25345216	0.49821223	0.79510374	25600.724522846472498
NONHSAG051053	-0.05017001	0.462914406	0.761237023	-0.25443545	0.308255953	0.636428591	25610.217980737510977
NONHSAG051068	0.078954423	0.136988718	0.4403917	-0.25444037	0.186108517	0.490935412	25620.262858700541248
NONHSAG051082	0.478629851	0.001146116	0.010362948	-0.25475774	3.65E-05	0.000503147	25630.000101331821504145
NONHSAG051104	0.037944178	0.634461778	0.862680714	-0.25556473	0.725424399	0.902489903	25640.884008106655028
NONHSAG051105	0.276895845	0.012862393	0.079667507	-0.25614841	0.000404066	0.004192745	25650.00143178655413886
NONHSAG051129	-0.43349731	1.49E-06	2.53E-05	-0.25677077	8.58E-06	0.000139435	25661.40367445170039e-06
NONHSAG051137	0.037081012	0.521718242	0.803843214	-0.25739066	0.668807422	0.880630922	25670.8058604268005
NONHSAG051145	0.203049569	0.023290655	0.128166968	-0.25780767	0.273099912	0.602791956	25680.0733270494670059
NONHSAG051156	-0.03126173	0.602812791	0.848669165	-0.25841837	0.3508725	0.683278027	25690.340601439746852
NONHSAG051181	0.026480481	0.699204702	0.894931098	-0.25856331	0.294448578	0.623485995	25700.56520281778311
NONHSAG051194	-0.02403161	0.696493704	0.892432585	-0.25907735	0.081947887	0.309727767	25710.0804035644722347
NONHSAG051196	-0.27309169	0.004830825	0.035560943	-0.25961824	0.814112433	0.931603479	25720.00776537387330963
NONHSAG051205	0.104653285	0.434958581	0.738589088	-0.26007192	0.503252001	0.799065289	25730.697012809962373
NONHSAG051207	0.112739911	0.204870051	0.53747107	-0.26077392	0.942892013	0.976126639	25740.359373353775472
NONHSAG051233	-0.03475895	0.672138873	0.884442916	-0.26385654	0.517702616	0.804430331	25750.558616060247088
NONHSAG051237	-0.10319456	0.228078587	0.559166483	-0.26418552	0.980351474	0.992468062	25760.384100858057246
NONHSAG051255	-0.08544096	0.115091522	0.400046181	-0.26465803	0.183790724	0.488163241	25770.235864887279474
NONHSAG051258	-0.0296463	0.583074379	0.841774074	-0.26565006	0.901101486	0.962240579	25780.845324453568074
NONHSAG051259	-0.10375886	0.192854695	0.525491668	-0.26657021	0.746441696	0.906696003	25790.229694559776919
NONHSAG051263	-0.02254962	0.72501659	0.903322681	-0.26689236	0.86138799	0.947290977	25800.864654190753586
NONHSAG051266	0.067291859	0.156455069	0.469898142	-0.26815828	0.011118298	0.073156786	25810.0378137354922982
NONHSAG051273	-0.03558361	0.575124631	0.836650451	-0.26897177	0.342038343	0.675184303	25820.6297371716788
NONHSAG051275	0.11337859	0.117371381	0.40546477	-0.26905682	0.673679173	0.881776357	25830.115904580129674
NONHSAG051297	-0.00758149	0.886148801	0.956660206	-0.27164676	0.396053163	0.718406651	25840.560465859827464
NONHSAG051305	0.012357806	0.844444561	0.94647636	-0.27178909	0.386151326	0.711105126	25850.525625763090117
NONHSAG051327	0.099668029	0.10707609	0.383564287	-0.27181307	0.405835623	0.726885205	25860.0518696727490771
NONHSAG051334	-0.00155964	0.979446192	0.993501254	-0.27269577	0.202963952	0.511410961	25870.329818523952338
NONHSAG051339	0.041406925	0.467640784	0.764261292	-0.27278007	0.049343364	0.222718361	25880.134365059201496
NONHSAG051345	-0.01020818	0.851412418	0.948883261	-0.27301713	0.457368433	0.767100781	25890.738556737049651
NONHSAG051356	0.027802403	0.645470309	0.87033597	-0.27512192	0.062342345	0.259088491	25900.147947749326428
NONHSAG051375	-0.0761741	0.209876907	0.544124897	-0.2751527	0.816439079	0.932504748	25910.279210346497286
NONHSAG051392	0.109772243	0.121677962	0.411759519	-0.27605743	0.024567151	0.133107571	25920.0699373142306412
NONHSAG051400	-0.23534385	1.97E-06	3.25E-05	-0.27697857	0.000557628	0.005560938	25937.65976036191262e-06
NONHSAG051424	-0.01017824	0.886295488	0.956660206	-0.27748346	0.812946483	0.931542588	25940.971603123414706
NONHSAG051435	-0.08809486	0.227069742	0.558671887	-0.27802358	0.547453077	0.81624499	25950.477103901470743
NONHSAG051445	0.014066139	0.82313225	0.938461188	-0.27857572	0.611768789	0.849433959	25960.753333671402687
NONHSAG051446	-0.0546009	0.565499594	0.830383739	-0.27869364	0.708622857	0.896707014	25970.632097035450726
NONHSAG051464	-0.1161676	0.221308613	0.554652247	-0.28171889	0.329811284	0.663397232	25980.430090142625105
NONHSAG051484	0.135214436	0.004544915	0.033900003	-0.2852751	0.020958618	0.118821844	25990.0110332890342147
NONHSAG051497	-0.05426452	0.420485924	0.726032559	-0.28572797	0.741791467	0.906043339	26000.504853273406779
NONHSAG051574	-0.00771821	0.915428451	0.967869201	-0.28613601	0.597935933	0.839825518	26010.792268672276828
NONHSAG051638	-0.14278858	0.008027113	0.054786997	-0.28634919	0.049170044	0.222512561	26020.0228357351133375
NONHSAG051650	-0.04637286	0.638026155	0.865892639	-0.28799424	0.872267407	0.950957653	26030.80450768423377
NONHSAG051658	-0.10226268	0.20883328	0.542233781	-0.28923733	0.433763603	0.748737951	26040.442337318135523
NONHSAG051661	0.128495027	0.091372145	0.349576152	-0.28981979	0.101380095	0.350159272	26050.157566055438276
NONHSAG051677	0.202038137	0.001314581	0.011654188	-0.29085801	0.015737631	0.095503013	26060.0039231879672766
NONHSAG051728	0.116361247	0.155530984	0.468258165	-0.29097432	0.010598183	0.070447223	26070.0362429959131991
NONHSAG051730	-0.05494777	0.461919582	0.760209241	-0.29272448	0.796696033	0.92413553	26080.586073115704215
NONHSAG051770	0.034128713	0.589201521	0.842139475	-0.29368053	0.572886312	0.833292346	26090.813864919662955
NONHSAG051806	0.21482826	0.004699526	0.034685214	-0.29589335	0.006046568	0.04404909	26100.00623720455976067
NONHSAG051813	1.106237772	2.73E-14	2.07E-12	-0.29600691	2.95E-13	2.96E-11	26118.71695789318856e-15
NONHSAG051826	-0.04437332	0.409984778	0.720098185	-0.29606531	0.337728274	0.671160358	26120.580132560176198
NONHSAG051866	-0.04367162	0.51238656	0.796980196	-0.29620988	0.86826954	0.950047122	26130.683714910150232
NONHSAG051885	-0.03792834	0.602632044	0.848669165	-0.29907322	0.943427456	0.976126639	26140.850754231896916

NONHSAG051943	0.073144737	0.307523061	0.645339438	-0.30172847	0.14466165	0.430061982	26150.322553701207113
NONHSAG051945	0.008297703	0.90052959	0.962481645	-0.30188692	0.245900859	0.574769231	26160.440236244759827
NONHSAG051962	0.069816439	0.454781141	0.756213964	-0.30402159	0.597041429	0.839825518	26170.440427067900433
NONHSAG051968	-0.04750115	0.387978769	0.706496807	-0.30486689	0.709571584	0.896707014	26180.683557670968872
NONHSAG051985	-0.06743278	0.261905987	0.6018673	-0.30524272	0.783884852	0.9188346	26190.499719031693977
NONHSAG052010	0.024301805	0.754243429	0.911448145	-0.30584843	0.307696056	0.635739391	26200.574588380948439
NONHSAG052012	-0.04914545	0.339731292	0.673579125	-0.30676953	0.705187923	0.896707014	26210.625586292444838
NONHSAG052042	0.024118649	0.779186367	0.9209289	-0.30975223	0.213059971	0.526934599	26220.268753674903202
NONHSAG052090	0.042037069	0.444305969	0.747241857	-0.31047936	0.137734376	0.423288596	26230.327134478511134
NONHSAG052112	0.012206079	0.865369161	0.951960393	-0.31244023	0.974296904	0.989451568	26240.983688743517067
NONHSAG052118	-0.01861213	0.823656758	0.938461188	-0.31454412	0.922267363	0.971770056	26250.946797342338473
NONHSAG052125	0.366286618	4.03E-08	9.22E-07	-0.31664801	3.01E-08	8.56E-07	26268.91393639746425e-09
NONHSAG052126	-0.06517669	0.454519122	0.756213964	-0.31689007	0.297329797	0.62710964	26270.204567767096283
NONHSAG052157	-0.05998181	0.433828334	0.738453557	-0.31826586	0.613140892	0.850173662	26280.72672037869732
NONHSAG052162	0.282568681	0.018871825	0.108301166	-0.31996867	0.001632889	0.014304309	26290.00492316062151121
NONHSAG052178	-0.03780614	0.564530892	0.830383739	-0.32076251	0.625382782	0.856580631	26300.822674152449654
NONHSAG052186	-0.28485366	1.06E-06	1.82E-05	-0.32208368	1.86E-06	3.65E-05	26315.28491878884262e-07
NONHSAG052188	0.116507802	0.064519851	0.273237684	-0.32382371	0.807948528	0.929984142	26320.130779681473519
NONHSAG052409	-0.01448394	0.834737311	0.94268326	-0.32411404	0.282781968	0.613093982	26330.518198589526199
NONHSAG052414	0.091743454	0.33948596	0.673579125	-0.3256043	0.136102427	0.421497825	26340.315687796871268
NONHSAG052449	0.180584977	0.023682055	0.129560191	-0.32622266	0.00950499	0.063638173	26350.0193805831686138
NONHSAG052499	0.030320786	0.665731226	0.879303057	-0.3294072	0.991089728	0.997026029	26360.87860348912033
NONHSAG052508	0.504458398	6.89E-09	1.92E-07	-0.32956541	1.71E-09	6.60E-08	26376.87876858970203e-10
NONHSAG052530	0.010814938	0.840556144	0.945079519	-0.33286748	0.484856029	0.78447362	26380.769279408598453
NONHSAG052547	0.333283628	7.84E-06	0.000114202	-0.33334275	5.84E-05	0.000757263	26391.01828037030901e-05
NONHSAG052583	-0.02496408	0.705193936	0.897693684	-0.33458906	0.29908343	0.62856697	26400.570900025446794
NONHSAG052586	0.069259849	0.196005305	0.527031094	-0.33858771	0.789271799	0.921569044	26410.389023870743164
NONHSAG052609	-0.02069007	0.860531495	0.95118497	-0.34071759	0.909597275	0.965738136	26420.957866945981611
NONHSAG052620	-0.077582	0.366856077	0.695147768	-0.341206	0.933088706	0.973855106	26430.547176056180851
NONHSAG052621	0.01361073	0.820620495	0.938461188	-0.34252031	0.993845097	0.997026029	26440.964659499487793
NONHSAG052627	0.210355895	0.001651505	0.01403031	-0.34499718	0.000250332	0.00281574	26450.000485853404817089
NONHSAG052636	0.092437742	0.247299438	0.583394312	-0.3460646	0.806002891	0.929266146	26460.325907014209356
NONHSAG052644	0.010466455	0.887209427	0.956660206	-0.34632936	0.667437489	0.880630922	26470.835991675365135
NONHSAG052726	-0.05490927	0.472236856	0.764982846	-0.34702809	0.830011164	0.933749876	26480.758611375651625
NONHSAG052734	0.029968122	0.644638369	0.870030385	-0.34826087	0.183619381	0.488163241	26490.396381355374824
NONHSAG052752	0.249777618	0.00772379	0.053103419	-0.34990599	0.006199576	0.045047048	26500.00840990237538428
NONHSAG052756	0.020746271	0.717485912	0.899897585	-0.3525615	0.829796135	0.933749876	26510.93503411963872
NONHSAG052760	0.15545439	0.148309621	0.458293026	-0.35281129	0.14343917	0.430061982	26520.242630250044291
NONHSAG052772	0.012139435	0.873807615	0.953128932	-0.35446067	0.097668273	0.343973343	26530.185884992672166
NONHSAG052776	-0.06109098	0.389639665	0.706543193	-0.35452214	0.734335927	0.905644804	26540.68315796805553
NONHSAG052817	-0.01782434	0.86992798	0.951960393	-0.35495825	0.539928696	0.812140251	26550.815218832738018
NONHSAG052818	-0.06107574	0.593058035	0.843701599	-0.35620303	0.560030899	0.825803298	26560.809057894299417
NONHSAG052837	0.197116014	0.007370636	0.051175874	-0.35649293	0.023934101	0.131194329	26570.0163865435685825
NONHSAG052879	0.207781233	0.002337213	0.018885752	-0.35849181	0.016096787	0.096718303	26580.00613116381821705
NONHSAG052880	0.265438077	0.000277961	0.002916511	-0.35937726	0.00029806	0.00326126	26590.000219755944797293
NONHSAG052885	-0.08262752	0.277830327	0.612912715	-0.36066463	0.227843769	0.550426701	26600.410870601065871
NONHSAG052886	0.080267783	0.268277287	0.605799463	-0.36646511	0.341313602	0.674686654	26610.484166528674052
NONHSAG052890	-0.02863107	0.627302288	0.85822947	-0.36881808	0.746626077	0.906696003	26620.716199007107065
NONHSAG052915	-0.79303702	3.23E-14	2.33E-12	-0.36926525	3.94E-09	1.42E-07	26631.55463560656007e-13
NONHSAG052978	0.03304396	0.585433283	0.842139475	-0.36988312	0.100939898	0.349991359	26640.242463321217672
NONHSAG052998	-0.11861556	0.104098138	0.377032592	-0.37144629	0.585640743	0.836463428	26650.249227273262832
NONHSAG053000	0.03443459	0.572629411	0.836084156	-0.37349851	0.032036786	0.159729509	26660.0817098467154513
NONHSAG053032	-0.07039813	0.395211231	0.711193473	-0.37418237	0.076984288	0.297141877	26670.0333465073296256
NONHSAG053081	0.017116432	0.760025488	0.914111066	-0.37602187	0.926081117	0.971770056	26680.915803424123101
NONHSAG053118	-0.0300596	0.608816852	0.850891147	-0.37653997	0.580229839	0.836050856	26690.825069365923832
NONHSAG053119	-0.03415184	0.589677321	0.842139475	-0.37655229	0.30498167	0.634657974	26700.58623520430453
NONHSAG053167	-0.14173158	0.010330439	0.068190599	-0.37710753	0.00677439	0.048349198	26710.0103728416986316
NONHSAG053168	0.023585406	0.723773407	0.903322681	-0.37713777	0.468774478	0.777236929	26720.545729821684929
NONHSAG053205	0.361811571	0.001994769	0.016546579	-0.37945885	0.002514299	0.020673124	26730.00230966506765425
NONHSAG053209	-0.15867924	0.010120239	0.06724594	-0.37985633	0.474931429	0.780269093	26740.0280004012121108
NONHSAG053219	-0.08603766	0.158772735	0.472842244	-0.38058799	0.646530684	0.870055103	26750.350375035779494
NONHSAG053240	-0.01028742	0.883772993	0.955834483	-0.38064805	0.362450491	0.691401321	26760.615864632174128
NONHSAG053246	-0.2845617	0.000790005	0.007355939	-0.3839114	0.134453376	0.417310037	26770.00316521262012252
NONHSAG053259	-0.05499255	0.509164499	0.795870245	-0.3842823	0.434009098	0.748737951	26780.698696996592469
NONHSAG053273	0.08321678	0.313053522	0.651448843	-0.39176466	0.408639922	0.729548907	26790.557550151765654
NONHSAG053274	-0.01716661	0.756361158	0.912827286	-0.39367965	0.926054943	0.971770056	26800.949851915937951
NONHSAG053293	-0.07640168	0.316585826	0.654587752	-0.3938509	0.702292913	0.896028889	26810.595466087994393
NONHSAG053296	0.076562299	0.223826754	0.556499409	-0.39405614	0.869471685	0.950234892	26820.414224223869823
NONHSAG053299	-0.03746555	0.632656601	0.862680714	-0.39988818	0.938315679	0.975531432	26830.875030705093509
NONHSAG053306	0.291117485	0.001161143	0.010465174	-0.40251701	0.000126763	0.001504044	26840.000258272578592043
NONHSAG053322	0.025680404	0.777649944	0.920350018	-0.40928015	0.196934878	0.504520098	26850.39353456212857

NONHSAG053329	-0.0649065	0.292937244	0.630465061	-0.40931382	0.1586786	0.451836895	26860.338419209482489
NONHSAG053359	0.443610423	1.77E-09	5.52E-08	-0.41295482	7.93E-10	3.28E-08	26872.229884254736e-10
NONHSAG053361	-0.04871939	0.553988445	0.821852872	-0.41484551	0.397033603	0.718904373	26880.682234215074943
NONHSAG053376	-0.03346556	0.550207815	0.820922168	-0.41500604	0.809675923	0.930212019	26890.68699245985526
NONHSAG053377	-0.21451301	0.000196761	0.002178312	-0.41655761	0.001251591	0.011316638	26900.000363875423609797
NONHSAG053380	0.033906102	0.572929065	0.836084156	-0.41737232	0.024124125	0.131964298	26910.0613374732505943
NONHSAG053392	-0.03290246	0.545241867	0.818153751	-0.41811053	0.650402919	0.873415954	26920.568354171748505
NONHSAG053406	0.093850427	0.325253599	0.665173178	-0.41835409	0.27829917	0.60825425	26930.125348365365158
NONHSAG053407	0.015944958	0.827032823	0.940022755	-0.42085848	0.51580982	0.804430331	26940.662925184407183
NONHSAG053417	0.101990599	0.228447299	0.559166483	-0.42299048	0.484471321	0.78447362	26950.476526737007241
NONHSAG053440	0.018645234	0.767696766	0.916213829	-0.42521961	0.773567723	0.91321261	26960.944134488155477
NONHSAG053450	0.066371913	0.294974915	0.632460859	-0.42629445	0.448035403	0.756417348	26970.199214034063047
NONHSAG053454	0.051720608	0.477877711	0.769393918	-0.42795929	0.669689563	0.880630922	26980.771964682743962
NONHSAG053462	-0.05775111	0.359782581	0.686572852	-0.42811795	0.345746875	0.678791662	26990.558787219593848
NONHSAG053469	-0.3415965	1.63E-05	0.000224117	-0.42882206	0.004089755	0.030998357	27007.13554065263898e-05
NONHSAG053552	-0.05350928	0.490230142	0.777511088	-0.4310131	0.442384186	0.753473247	27010.348066987424181
NONHSAG053577	0.979567259	1.15E-13	7.89E-12	-0.43597739	2.26E-11	1.51E-09	27021.234317560291e-13
NONHSAG053585	0.046439688	0.431738371	0.73623305	-0.43638542	0.269378249	0.599281359	27030.51998286884802
NONHSAG053586	0.796316446	1.07E-12	6.27E-11	-0.43756973	3.48E-11	2.17E-09	27046.56614827270864e-13
NONHSAG053589	0.009879347	0.910525585	0.965823442	-0.4419875	0.687901539	0.890389787	27050.862555466971614
NONHSAG053591	0.056560386	0.418383541	0.726032559	-0.44212809	0.595254761	0.839418789	27060.709915057435688
NONHSAG053607	-0.37090248	2.01E-08	4.92E-07	-0.4441217	4.07E-06	7.12E-05	27074.85310785298608e-08
NONHSAG053614	0.057742238	0.299879124	0.637694547	-0.44577355	0.892236999	0.958708067	27080.525761802475074
NONHSAG053675	-0.07446268	0.268968259	0.605799463	-0.45477851	0.017642828	0.104511744	27090.0570469074330714
NONHSAG053719	0.009112268	0.888768473	0.956821189	-0.45783208	0.757563776	0.908748575	27100.89884143777194
NONHSAG053722	0.146105226	0.046775982	0.217411671	-0.45908286	0.943110298	0.976126639	27110.0786343340237437
NONHSAG053746	0.079340272	0.320092125	0.658930494	-0.45992916	0.295416303	0.624125202	27120.495665392587787
NONHSAG053751	0.097538866	0.263648703	0.602748092	-0.46626275	0.03798223	0.182887041	27130.112429879000625
NONHSAG053784	0.077005193	0.070831777	0.290772201	-0.46741108	0.029225562	0.150516997	27140.0662179529049073
NONHSAG053801	0.061908096	0.30209198	0.640635481	-0.47005292	0.179154971	0.483942149	27150.368646894767141
NONHSAG053821	0.041350066	0.545112506	0.818153751	-0.47335614	0.34439485	0.678178093	27160.628334470909938
NONHSAG053863	-0.05290952	0.38479851	0.706496807	-0.48328466	0.982318537	0.992911476	27170.608649266292479
NONHSAG053877	0.09283404	0.123122088	0.414832985	-0.48425852	0.0158515	0.095858965	27180.0495074949929764
NONHSAG053892	-0.76271053	2.09E-08	5.07E-07	-0.48905627	1.68E-06	3.36E-05	27193.7262697513247e-08
NONHSAG053893	0.014720296	0.69329205	0.89238585	-0.48986403	0.472139376	0.778445171	27200.76877699593094
NONHSAG053895	0.039084887	0.353166291	0.680247895	-0.49220759	0.583004882	0.836050856	27210.64291495974965
NONHSAG053900	0.200786724	0.001758983	0.014853636	-0.49448983	0.036479662	0.176559051	27220.00623640458400918
NONHSAG053985	0.002509071	0.960775261	0.984584561	-0.50432536	0.414183344	0.733427936	27230.620264564586273
NONHSAG054015	-0.0657377	0.389143005	0.706543193	-0.50521624	0.96448448	0.986709541	27240.590762534782323
NONHSAG054016	0.000313153	0.99587225	0.997290871	-0.5081959	0.170524505	0.469652211	27250.28523688080895
NONHSAG054030	0.069293342	0.370600791	0.698354362	-0.50829425	0.790092246	0.921569044	27260.651222051958683
NONHSAG054071	-0.03169767	0.648046007	0.870666685	-0.51440126	0.696420371	0.895338645	27270.88398785747939
NONHSAG054103	-0.04322543	0.525184001	0.806563305	-0.51487826	0.271529293	0.601363513	27280.539234707973603
NONHSAG054109	0.03508074	0.644787811	0.870030385	-0.51878455	0.399713924	0.722362182	27290.697221749481619
NONHSAG054140	0.178339137	0.00217911	0.017917124	-0.52449127	0.003280838	0.025987932	27300.00281684666129204
NONHSAG054179	0.174445083	0.038187642	0.187406018	-0.53247944	0.005921969	0.043350075	27310.0166946319137738
NONHSAG054188	-0.08084372	0.203619998	0.536312792	-0.53535672	0.25810358	0.589799224	27320.375630784348226
NONHSAG054199	0.043513247	0.522869809	0.803843214	-0.53648968	0.647588452	0.870884135	27330.802799190208698
NONHSAG054326	0.072246097	0.210876007	0.544124897	-0.53883044	0.172161584	0.472771849	27340.315119238544924
NONHSAG054344	-0.04186438	0.465601063	0.763571876	-0.53942761	0.1157511	0.378039597	27350.068885142951237
NONHSAG054345	-0.02575423	0.600845204	0.848607088	-0.54530309	0.795041748	0.923062508	27360.870379175038843
NONHSAG054445	0.059620352	0.401397869	0.713976418	-0.54732464	0.470189686	0.778206826	27370.658419568362105
NONHSAG054462	0.033567825	0.62510934	0.85822947	-0.56182374	0.300446967	0.629081811	27380.300684657840148
NONHSAG054468	0.100333754	0.127118892	0.423583871	-0.56539202	0.645057447	0.869559703	27390.288617450369999
NONHSAG054478	-0.08148271	0.236599238	0.571062498	-0.57087962	0.634048503	0.861196385	27400.486809351056139
NONHSAG054549	0.10894164	0.171985022	0.496021375	-0.57267311	0.078009248	0.298858317	27410.179286786535072
NONHSAG054576	0.145739088	0.03403751	0.171222678	-0.57814147	0.724402607	0.902489903	27420.0740956962330257
NONHSAG054653	-0.06673354	0.427375814	0.732346611	-0.59957825	0.419972991	0.737671981	27430.649452864043128
NONHSAG054655	0.047615784	0.47840903	0.769614526	-0.60050523	0.635468546	0.862005573	27440.76774846758498
NONHSAG054664	0.032299953	0.656697148	0.873938656	-0.60263512	0.386942625	0.711630256	27450.684073142009289
NONHSAG054671	0.18804657	0.013601625	0.083693151	-0.61047214	0.001152273	0.010520106	27460.00337728934998207
NONHSAG054679	-0.07175735	0.249584887	0.584860584	-0.61385456	0.111443476	0.369410385	27470.256021463742914
NONHSAG054729	-0.37503106	2.75E-06	4.42E-05	-0.61796847	2.12E-06	4.06E-05	27489.72207162371503e-07
NONHSAG054754	0.324053996	0.001211119	0.010846073	-0.63859671	0.000577565	0.005738912	27490.000731941490614194
NONHSAG054784	0.267371402	0.003024821	0.023561766	-0.64155918	0.004964811	0.037130452	27500.00429442591533159
NONHSAG054798	1.119360474	1.09E-10	4.51E-09	-0.64564077	7.39E-11	4.07E-09	27511.41250092811537e-11
NONHSAG054804	1.001661156	3.13E-10	1.13E-08	-0.64581757	3.86E-12	2.85E-10	27523.63932080597974e-12
NONHSAG054869	-0.344326	1.87E-07	3.79E-06	-0.66897004	1.00E-07	2.52E-06	27533.98804150153189e-08
NONHSAG054870	0.169888801	0.001933586	0.016134256	-0.68054586	0.381823317	0.708236919	27540.00559663905277635
NONHSAG054897	0.018649865	0.792857084	0.927804461	-0.68171204	0.474798574	0.780269093	27550.597814218800321
NONHSAG054929	0.076889429	0.290459601	0.626875057	-0.68342805	0.18242386	0.487156595	27560.368172841348459

NONHSAG054954	0.148968952	0.037879911	0.18687423	-0.68664871	0.183842416	0.488163241	27570.107435735740998
NONHSAG054958	0.008737509	0.901245946	0.962603929	-0.69777444	0.423433113	0.740942075	27580.686467069921079
NONHSAG054963	0.10431552	0.145108781	0.451192539	-0.71835687	0.182091876	0.486732277	27590.26898582421276
NONHSAG055008	-0.00536561	0.931244545	0.974203743	-0.72594578	0.516416139	0.804430331	27600.777475999673498
NONHSAG055019	-0.0957567	0.142592354	0.447555229	-0.72766264	0.825312508	0.933749876	27610.282600120512001
NONHSAG055021	0.159735724	0.001062869	0.009672455	-0.73009617	0.000983378	0.00918691	27620.000940470459945396
NONHSAG055050	-0.1141501	0.079690544	0.314291456	-0.75922444	0.248987618	0.577684143	27630.20152750796571
NONHSAG055086	0.022811	0.748056979	0.910542676	-0.75985597	0.430015434	0.746421851	27640.726455947817165
NONHSAG055105	0.02571886	0.587667232	0.842139475	-0.7656697	0.304728122	0.634657974	27650.585856107569779
NONHSAG055108	-0.22385604	0.015136517	0.091302104	-0.77450711	0.015758676	0.095503013	27660.0210034431724624
NONHSAG055153	-0.01296639	0.845271288	0.946596121	-0.80535093	0.409345747	0.729548907	27670.554304676211711
NONHSAG055204	0.109576908	0.227084625	0.558671887	-0.80855699	0.050345325	0.223890543	27680.139454512926825
NONHSAG055266	-0.01351501	0.862828849	0.951781505	-0.82051612	0.334604946	0.670640846	27690.46986398455623
NONHSAG055280	-0.08188204	0.237919523	0.5711076	-0.82084229	0.278146603	0.60825425	27700.42040257611134
NONHSAG055289	-0.15094175	0.009972361	0.066608737	-0.85005335	0.050915351	0.225826448	27710.0272078591266315
NONHSAG055301	0.033774236	0.71349026	0.898492885	-0.85158604	0.699164064	0.895443732	27720.90833848281011
NONHSAG055339	-0.03162051	0.60322004	0.848669165	-0.87736944	0.573999742	0.833292346	27730.819929893218936
NONHSAG055350	-0.568341	1.22E-09	3.95E-08	-0.88348658	5.95E-09	1.99E-07	27745.23294963363248e-10
NONHSAG055367	0.026769509	0.711541303	0.898275053	-0.88385376	0.622039587	0.853798275	27750.875077546326058
NONHSAG055371	0.718303263	2.14E-06	3.50E-05	-0.88600593	1.61E-06	3.24E-05	27767.25135644617106e-07
NONHSAG055384	0.153155176	0.10958047	0.388575388	-0.88945037	0.069014345	0.27883382	27770.138862729382633
NONHSAG055388	0.49038472	1.96E-06	3.25E-05	-0.93188431	4.01E-07	9.01E-06	27782.99690702691982e-07
NONHSAG055401	-0.09809388	0.127689186	0.424423158	-0.94990077	0.668381731	0.880630922	27790.12624004315195
NONHSAG055405	0.205131259	0.005746974	0.041379663	-0.95132329	0.029027456	0.150253976	27800.0148263892807552
NONHSAG055409	-0.43173588	7.54E-05	0.000918305	-0.9523117	0.006496506	0.046721672	27810.000296748722090603
NONHSAG055436	0.415871493	2.73E-07	5.14E-06	-0.96060349	3.68E-05	0.000504868	27827.31397148899511e-07
NONHSAG055443	0.322831461	0.002857593	0.022571778	-1.00575782	1.37E-05	0.000202839	27835.7537687882778e-05
NONHSAG055445	0.105134885	0.078612252	0.312669947	-1.01202538	0.516468101	0.804430331	27840.200514090855241
NONHSAG055446	0.080324706	0.162176657	0.478531751	-1.01675184	0.013914227	0.086372639	27850.0460892375055923
NONHSAG055449	0.058703698	0.420112698	0.726032559	-1.01785445	0.841361051	0.941172323	27860.564918880090108
NONHSAG055454	0.024072884	0.649103936	0.871255498	-1.05233612	0.187686584	0.493708769	27870.187721751265325
NONHSAG055455	0.000806643	0.989931114	0.996279605	-1.05605984	0.053350328	0.232952053	27880.0848769582383516
NONHSAG055463	0.005279239	0.945452727	0.979231332	-1.0594024	0.60003362	0.840286125	27890.80828292788617
NONHSAG055544	0.023832267	0.766273581	0.915750663	-1.10664281	0.830422645	0.933749876	27900.953412675026924
NONHSAG055550	0.002264497	0.965587092	0.986997784	-1.12272546	0.842965161	0.941172323	27910.978223781103899
NONHSAG055620	-0.18864496	0.00697934	0.049187731	-1.13137855	0.00197372	0.016869608	27920.00384753134882629
NONHSAG055690	-0.05097259	0.503696332	0.789138617	-1.13700746	0.370916074	0.69793975	27930.296095317748854
NONHSAG055699	0.103897019	0.145100454	0.451192539	-1.1907359	0.530328151	0.808158244	27940.338097523486944
NONHSAG055722	0.094447445	0.112789614	0.394974341	-1.26974305	0.385033697	0.709976889	27950.278593729688289
NONHSAG055783	0.030426243	0.6603222	0.875035827	-1.30904487	0.595363216	0.839418789	27960.622276200719821
NONHSAG055861	-0.09129853	0.159517633	0.473168339	-1.36595467	0.853675022	0.945832264	27970.220669441708829
NONHSAG055878	0.151557195	0.010470984	0.068634979	-1.38261236	0.060016722	0.252267596	27980.0297665325830906
NONHSAG055883	0.065537252	0.622197444	0.857660281	-1.40061224	0.59409301	0.839418789	27990.836575469845401
NONHSAG056024	0.067696537	0.382224549	0.706496807	-1.53272128	0.870810572	0.950957653	28000.645462448386917
NONHSAG056026	0.154281167	0.008964909	0.060454012	-1.59514387	0.011535679	0.07542144	28010.0130306726825265
NONHSAG056029	0.050546423	0.289887594	0.62656719	-1.64381348	0.15127468	0.438088981	28020.327163156051362
NONHSAG056034	0.125030672	0.031315565	0.161280896	-2.12130125	0.10926962	0.36622905	28030.0813761061255313
NONHSAG056037	0.929649019	1.71E-10	6.51E-09	-2.55136941	7.32E-12	5.28E-10	28044.39118154665214e-12

Table S3. Probe position of re-annotated 2812 lncRNA gene

lncRNA gene	probe position	lncRNA gene	probe position	lncRNA gene	probe position	lncRNA gene	probe position	lncRNA gene	probe position
NONHSAG000051	845:153	NONHSAG030049	229:623	NONHSAG017613	869:185	NONHSAG012745	875:1139	NONHSAG012635	321:963
NONHSAG000057	842:55	NONHSAG030049	318:1115	NONHSAG017613	801:77	NONHSAG012745	971:325	NONHSAG012635	739:1107
NONHSAG000057	457:55	NONHSAG030049	560:209	NONHSAG017613	1013:1043	NONHSAG012745	252:765	NONHSAG012635	281:525
NONHSAG000057	665:391	NONHSAG030049	997:1017	NONHSAG017613	944:1093	NONHSAG012745	915:417	NONHSAG012635	1032:537
NONHSAG000057	1048:337	NONHSAG030049	588:513	NONHSAG017613	543:933	NONHSAG012745	343:369	NONHSAG012635	685:151
NONHSAG000057	395:235	NONHSAG030049	1062:509	NONHSAG017613	777:1157	NONHSAG012745	872:275	NONHSAG012635	880:365
NONHSAG000057	300:799	NONHSAG030049	303:587	NONHSAG017613	375:803	NONHSAG012745	749:165	NONHSAG012635	1155:593
NONHSAG000057	810:267	NONHSAG030049	102:1133	NONHSAG017613	377:397	NONHSAG012745	864:249	NONHSAG012635	221:503
NONHSAG000057	239:267	NONHSAG030049	473:779	NONHSAG017613	321:1045	NONHSAG012745	934:263	NONHSAG012635	463:1107
NONHSAG000057	357:931	NONHSAG030049	721:195	NONHSAG017613	276:875	NONHSAG012745	192:441	NONHSAG012635	423:477
NONHSAG000058	92:765	NONHSAG030049	390:275	NONHSAG017613	859:183	NONHSAG012745	811:435	NONHSAG012635	821:733
NONHSAG000058	245:143	NONHSAG030056	85:1105	NONHSAG017613	340:859	NONHSAG012745	543:699	NONHSAG012635	954:961
NONHSAG000058	271:883	NONHSAG030056	305:837	NONHSAG017613	1157:245	NONHSAG012745	889:921	NONHSAG012635	308:1107
NONHSAG000058	713:123	NONHSAG030056	1046:69	NONHSAG017613	796:577	NONHSAG012747	1069:867	NONHSAG012635	920:1057
NONHSAG000058	314:377	NONHSAG030056	528:227	NONHSAG017613	825:427	NONHSAG012747	1079:767	NONHSAG012635	1098:503
NONHSAG000058	482:863	NONHSAG030056	802:27	NONHSAG017613	1062:545	NONHSAG012747	195:143	NONHSAG012635	518:279
NONHSAG000058	434:527	NONHSAG030056	430:413	NONHSAG017613	20:291	NONHSAG012747	710:317	NONHSAG012635	1107:555
NONHSAG000058	907:523	NONHSAG030056	977:685	NONHSAG017613	485:431	NONHSAG012747	1051:759	NONHSAG012657	618:359
NONHSAG000058	242:747	NONHSAG030056	560:259	NONHSAG017613	960:987	NONHSAG012747	582:139	NONHSAG012657	846:673
NONHSAG000058	559:501	NONHSAG030056	729:681	NONHSAG017613	324:729	NONHSAG012747	270:219	NONHSAG012657	179:927
NONHSAG000058	539:151	NONHSAG030056	617:165	NONHSAG017613	81:425	NONHSAG012753	1104:495	NONHSAG012657	1076:1123
NONHSAG000058	128:315	NONHSAG030056	609:165	NONHSAG017613	398:789	NONHSAG012753	585:335	NONHSAG012657	775:487
NONHSAG000058	154:509	NONHSAG030133	642:1137	NONHSAG017613	845:271	NONHSAG012753	995:329	NONHSAG012657	314:67
NONHSAG000058	1050:393	NONHSAG030133	266:1043	NONHSAG017613	915:61	NONHSAG012753	1015:997	NONHSAG012657	405:1161
NONHSAG000058	524:75	NONHSAG030133	278:831	NONHSAG017613	794:921	NONHSAG012753	274:799	NONHSAG012657	426:1085
NONHSAG000058	591:1137	NONHSAG030133	328:363	NONHSAG017613	1049:845	NONHSAG012753	85:923	NONHSAG012657	567:853
NONHSAG000058	175:153	NONHSAG030133	1069:1149	NONHSAG017613	409:785	NONHSAG012753	1113:1019	NONHSAG012657	880:731
NONHSAG000058	328:161	NONHSAG030133	862:1147	NONHSAG017613	726:31	NONHSAG012753	504:207	NONHSAG012657	604:241
NONHSAG000058	972:369	NONHSAG030133	787:757	NONHSAG017613	1035:667	NONHSAG012753	4:787	NONHSAG012658	668:781
NONHSAG000058	207:657	NONHSAG030133	822:225	NONHSAG017613	395:149	NONHSAG012753	1016:525	NONHSAG012658	718:927
NONHSAG000058	485:1147	NONHSAG030133	146:187	NONHSAG017613	851:933	NONHSAG012753	761:643	NONHSAG012658	1081:497
NONHSAG000058	433:999	NONHSAG030133	648:225	NONHSAG017613	199:603	NONHSAG012762	1062:537	NONHSAG012658	689:751
NONHSAG000070	252:127	NONHSAG030133	811:235	NONHSAG017613	423:945	NONHSAG012762	818:219	NONHSAG012658	219:275
NONHSAG000070	935:921	NONHSAG030140	59:137	NONHSAG017613	1005:813	NONHSAG012762	696:805	NONHSAG012658	645:829
NONHSAG000070	255:869	NONHSAG030140	1143:967	NONHSAG017613	389:885	NONHSAG012762	913:215	NONHSAG012658	764:519
NONHSAG000070	184:417	NONHSAG030140	338:679	NONHSAG017613	141:1145	NONHSAG012762	672:829	NONHSAG012658	716:45
NONHSAG000071	293:979	NONHSAG030140	367:1115	NONHSAG017613	734:1105	NONHSAG012762	98:939	NONHSAG012658	381:1153
NONHSAG000071	938:131	NONHSAG030140	663:197	NONHSAG017613	452:275	NONHSAG012762	64:853	NONHSAG012658	806:771
NONHSAG000071	129:209	NONHSAG030140	286:197	NONHSAG017613	58:511	NONHSAG012762	946:877	NONHSAG012658	582:955
NONHSAG000071	409:381	NONHSAG030140	388:273	NONHSAG017613	1029:185	NONHSAG012762	523:53	NONHSAG012682	870:311
NONHSAG000071	1001:635	NONHSAG030140	905:1059	NONHSAG017613	172:323	NONHSAG012764	529:1157	NONHSAG012682	366:147
NONHSAG000071	956:1027	NONHSAG030140	64:173	NONHSAG017613	505:69	NONHSAG012764	983:653	NONHSAG012682	1046:83
NONHSAG000071	635:985	NONHSAG030140	395:133	NONHSAG017613	1123:973	NONHSAG012764	104:155	NONHSAG012682	921:1075
NONHSAG000071	731:181	NONHSAG030140	1040:229	NONHSAG017613	1118:227	NONHSAG012764	552:635	NONHSAG012682	498:615
NONHSAG000071	688:507	NONHSAG030166	896:519	NONHSAG017613	736:841	NONHSAG012764	242:123	NONHSAG012682	455:373
NONHSAG000071	261:699	NONHSAG030166	96:799	NONHSAG017613	630:71	NONHSAG012764	1111:185	NONHSAG012682	258:727
NONHSAG000071	493:751	NONHSAG030166	1096:513	NONHSAG017613	794:173	NONHSAG012764	1063:81	NONHSAG012682	1057:731
NONHSAG000071	794:429	NONHSAG030166	210:635	NONHSAG017613	671:851	NONHSAG012764	597:897	NONHSAG012682	309:1137
NONHSAG000071	377:311	NONHSAG030166	121:577	NONHSAG017613	91:193	NONHSAG012764	682:11	NONHSAG012682	495:1121
NONHSAG000075	688:465	NONHSAG030166	950:3	NONHSAG017640	905:955	NONHSAG012764	684:209	NONHSAG012683	373:21
NONHSAG000075	221:421	NONHSAG030166	1096:253	NONHSAG017640	167:915	NONHSAG012780	216:961	NONHSAG012683	241:613
NONHSAG000075	295:701	NONHSAG030166	429:329	NONHSAG017640	564:1035	NONHSAG012780	247:657	NONHSAG012683	1042:97
NONHSAG000075	1023:981	NONHSAG030166	353:199	NONHSAG017640	273:287	NONHSAG012780	925:521	NONHSAG012683	1099:73
NONHSAG000075	772:829	NONHSAG030166	555:37	NONHSAG017640	1020:767	NONHSAG012780	912:615	NONHSAG012683	1068:1043
NONHSAG000075	842:375	NONHSAG030166	449:783	NONHSAG017640	891:1033	NONHSAG012780	330:375	NONHSAG012683	82:1061
NONHSAG000075	356:909	NONHSAG030168	38:441	NONHSAG017640	246:43	NONHSAG012780	525:711	NONHSAG012683	374:773
NONHSAG000075	705:299	NONHSAG030168	821:1019	NONHSAG017640	680:601	NONHSAG012780	1043:413	NONHSAG012683	503:55
NONHSAG000075	1034:391	NONHSAG030168	42:123	NONHSAG017640	501:955	NONHSAG012780	346:441	NONHSAG012683	1060:1095
NONHSAG000075	575:769	NONHSAG030168	948:577	NONHSAG017640	147:1095	NONHSAG012780	454:91	NONHSAG012683	726:1101
NONHSAG000075	145:919	NONHSAG030168	85:317	NONHSAG017669	372:1077	NONHSAG012780	238:759	NONHSAG012683	131:439
NONHSAG000111	1039:435	NONHSAG030168	572:301	NONHSAG017669	610:185	NONHSAG012793	164:123	NONHSAG012684	360:649
NONHSAG000111	744:847	NONHSAG030168	764:945	NONHSAG017669	806:1113	NONHSAG012793	1116:929	NONHSAG012684	783:21
NONHSAG000111	6:687	NONHSAG030168	795:335	NONHSAG017669	133:159	NONHSAG012793	776:405	NONHSAG012684	764:47
NONHSAG000111	263:65	NONHSAG030168	981:1079	NONHSAG017669	636:1045	NONHSAG012793	920:331	NONHSAG012684	907:863
NONHSAG000111	182:681	NONHSAG030168	466:803	NONHSAG017669	1072:1093	NONHSAG012793	34:603	NONHSAG012684	132:1149
NONHSAG000111	1000:1097	NONHSAG030168	742:863	NONHSAG017669	499:1065	NONHSAG012793	917:107	NONHSAG012695	171:323
NONHSAG000111	326:577	NONHSAG030168	745:631	NONHSAG017669	968:1071	NONHSAG012793	918:325	NONHSAG012695	1064:277
NONHSAG000111	865:549	NONHSAG030168	402:237	NONHSAG017669	1097:189	NONHSAG012793	502:417	NONHSAG012695	1102:861
NONHSAG000111	469:141	NONHSAG030168	543:207	NONHSAG017669	1133:715	NONHSAG012793	620:55	NONHSAG012695	212:619
NONHSAG000111	319:917	NONHSAG030168	834:1131	NONHSAG017669	1009:751	NONHSAG012793	737:301	NONHSAG012695	1086:737
NONHSAG000111	1076:611	NONHSAG030168	451:869	NONHSAG017669	930:1045	NONHSAG012793	712:773	NONHSAG012695	338:829
NONHSAG000111	252:849	NONHSAG030168	1112:835	NONHSAG017669	620:683	NONHSAG012793	601:1019	NONHSAG012695	863:1073
NONHSAG000111	469:353	NONHSAG030168	299:1031	NONHSAG017669	283:857	NONHSAG012793	285:299	NONHSAG012695	1004:177
NONHSAG000111	105:321	NONHSAG030168	445:83	NONHSAG017670	568:609	NONHSAG012793	658:589	NONHSAG012695	1160:139
NONHSAG000111	802:175	NONHSAG030168	618:235	NONHSAG017670	760:1033	NONHSAG012793	647:435	NONHSAG012695	136:551
NONHSAG000111	61:1015	NONHSAG030168	825:767	NONHSAG017670	424:307	NONHSAG012793	150:667	NONHSAG012695	496:1077
NONHSAG000111	318:383	NONHSAG030187	1022:563	NONHSAG017670	705:1039	NONHSAG012793	511:853	NONHSAG012695	103:487
NONHSAG000111	905:267	NONHSAG030187	743:1015	NONHSAG017670	683:423	NONHSAG012793	17:329	NONHSAG012695	777:207
NONHSAG000111	931:789	NONHSAG030187	951:693	NONHSAG017670	281:809	NONHSAG012793	351:367	NONHSAG012695	713:391
NONHSAG000111	908:407	NONHSAG030187	1150:1143	NONHSAG017670	635:767	NONHSAG012793	268:939	NONHSAG012695	724:361
NONHSAG000111	154:599	NONHSAG030187	814:761	NONHSAG017670	33:711	NONHSAG012799	929:315	NONHSAG012695	568:355
NONHSAG000111	744:329	NONHSAG030187	250:617	NONHSAG017671	506:915	NONHSAG012799	174:487	NONHSAG012695	396:505
NONHSAG000111	152:645	NONHSAG030187	497:587	NONHSAG017671	895:545	NONHSAG012799	389:619	NONHSAG012695	559:409
NONHSAG000111	776:883	NONHSAG030187	1127:1127	NONHSAG017671	344:425	NONHSAG012799	255:205	NONHSAG012695	1140:29
NONHSAG000111	1129:267	NONHSAG030187	887:711	NONHSAG017671	596:297	NONHSAG012799	584:439	NONHSAG012695	676:437
NONHSAG000111	310:69	NONHSAG030187	945:639	NONHSAG017671	133:685	NONHSAG012799	307:731	NONHSAG012695	519:603
NONHSAG000111	78:423	NONHSAG030187	704:1129	NONHSAG017671	367:957	NONHSAG012799	75:845	NONHSAG012695	1112:373

NONHSAG000111	744:371	NONHSAG030187	990:229	NONHSAG017671	267:699	NONHSAG012799	760:329	NONHSAG012695	992:153
NONHSAG000111	1025:245	NONHSAG030187	141:723	NONHSAG017671	1134:763	NONHSAG012799	971:91	NONHSAG012695	480:715
NONHSAG000111	908:463	NONHSAG030187	763:751	NONHSAG017671	1007:1053	NONHSAG012799	162:419	NONHSAG012695	817:905
NONHSAG000111	105:327	NONHSAG030187	106:675	NONHSAG017675	989:549	NONHSAG012799	603:977	NONHSAG012695	555:765
NONHSAG000111	584:869	NONHSAG030187	377:1113	NONHSAG017675	213:765	NONHSAG012823	50:753	NONHSAG012695	866:43
NONHSAG000111	200:913	NONHSAG030187	1070:1135	NONHSAG017675	819:83	NONHSAG012823	7:461	NONHSAG012695	381:935
NONHSAG000111	49:535	NONHSAG030187	376:233	NONHSAG017675	1101:615	NONHSAG012823	611:587	NONHSAG012695	307:661
NONHSAG000111	996:897	NONHSAG030187	353:63	NONHSAG017675	277:9	NONHSAG012823	117:755	NONHSAG012695	347:15
NONHSAG000111	690:443	NONHSAG030187	737:673	NONHSAG017675	379:727	NONHSAG012823	613:881	NONHSAG012695	953:853
NONHSAG000111	420:435	NONHSAG030187	765:825	NONHSAG017675	276:1111	NONHSAG012823	616:55	NONHSAG012695	116:1005
NONHSAG000111	740:899	NONHSAG030187	1154:1079	NONHSAG017675	839:1127	NONHSAG012823	705:89	NONHSAG012727	407:799
NONHSAG000111	225:447	NONHSAG030263	914:1117	NONHSAG017675	602:53	NONHSAG012823	915:681	NONHSAG012727	94:227
NONHSAG000111	284:311	NONHSAG030263	231:789	NONHSAG017675	935:235	NONHSAG012823	6:913	NONHSAG012727	556:725
NONHSAG000111	1118:459	NONHSAG030263	149:869	NONHSAG017675	289:565	NONHSAG012823	58:1151	NONHSAG012727	476:843
NONHSAG000111	685:433	NONHSAG030263	856:949	NONHSAG017708	363:889	NONHSAG012823	70:157	NONHSAG012727	1008:39
NONHSAG000111	1023:189	NONHSAG030263	760:223	NONHSAG017708	857:1013	NONHSAG012881	117:1083	NONHSAG012727	976:1057
NONHSAG000113	126:969	NONHSAG030263	1098:739	NONHSAG017708	864:751	NONHSAG012881	131:855	NONHSAG012727	669:911
NONHSAG000113	835:903	NONHSAG030263	15:725	NONHSAG017708	293:87	NONHSAG012881	285:921	NONHSAG012727	387:983
NONHSAG000113	933:595	NONHSAG030263	912:1039	NONHSAG017741	1092:461	NONHSAG012881	642:995	NONHSAG012727	1155:631
NONHSAG000113	348:427	NONHSAG030263	266:363	NONHSAG017741	107:439	NONHSAG012881	76:831	NONHSAG012732	934:109
NONHSAG000113	972:1125	NONHSAG030263	576:1099	NONHSAG017741	521:499	NONHSAG012881	677:281	NONHSAG012732	921:521
NONHSAG000113	113:1039	NONHSAG030263	791:809	NONHSAG017741	146:981	NONHSAG012881	1019:917	NONHSAG012732	468:47
NONHSAG000113	765:363	NONHSAG030263	997:887	NONHSAG017741	929:919	NONHSAG012881	400:955	NONHSAG012732	949:47
NONHSAG000113	86:297	NONHSAG030263	57:519	NONHSAG017741	270:41	NONHSAG012881	724:1031	NONHSAG012732	207:343
NONHSAG000113	511:1117	NONHSAG030263	184:1107	NONHSAG017741	1119:707	NONHSAG012881	313:607	NONHSAG012732	644:1127
NONHSAG000113	485:359	NONHSAG030263	430:623	NONHSAG017741	1120:1145	NONHSAG012884	124:55	NONHSAG012732	298:463
NONHSAG000113	301:1137	NONHSAG030263	1124:1077	NONHSAG017741	85:565	NONHSAG012884	121:375	NONHSAG012732	1002:963
NONHSAG000113	252:963	NONHSAG030263	666:201	NONHSAG017741	1142:561	NONHSAG012884	712:1015	NONHSAG012732	25:947
NONHSAG000113	306:169	NONHSAG030263	410:541	NONHSAG017741	674:1117	NONHSAG012884	705:771	NONHSAG012732	584:847
NONHSAG000117	434:981	NONHSAG030263	518:1055	NONHSAG017741	317:325	NONHSAG012909	655:941	NONHSAG012732	804:949
NONHSAG000117	703:1083	NONHSAG030263	170:809	NONHSAG017741	1137:429	NONHSAG012909	980:821	NONHSAG012732	255:7
NONHSAG000117	453:547	NONHSAG030263	583:75	NONHSAG017741	341:515	NONHSAG012909	534:453	NONHSAG012732	481:229
NONHSAG000117	866:79	NONHSAG030263	1111:23	NONHSAG017741	56:119	NONHSAG012909	608:471	NONHSAG012732	521:23
NONHSAG000117	1013:699	NONHSAG030278	945:1003	NONHSAG017741	984:59	NONHSAG012909	554:149	NONHSAG012732	688:1041
NONHSAG000117	895:1157	NONHSAG030278	190:767	NONHSAG017741	605:381	NONHSAG012909	562:855	NONHSAG012732	710:963
NONHSAG000117	223:471	NONHSAG030278	394:715	NONHSAG017741	401:97	NONHSAG012909	922:1003	NONHSAG012732	428:1139
NONHSAG000117	785:507	NONHSAG030278	58:383	NONHSAG017741	213:1021	NONHSAG012909	1055:795	NONHSAG012732	1131:1151
NONHSAG000117	135:215	NONHSAG030278	872:917	NONHSAG017742	100:59	NONHSAG012909	1083:755	NONHSAG012732	741:557
NONHSAG000117	248:1	NONHSAG030278	29:309	NONHSAG017742	341:111	NONHSAG012909	849:463	NONHSAG012732	42:119
NONHSAG000117	429:465	NONHSAG030278	337:1131	NONHSAG017742	1050:889	NONHSAG012909	285:1091	NONHSAG012732	830:83
NONHSAG000134	437:433	NONHSAG030278	862:505	NONHSAG017742	56:97	NONHSAG012909	981:415	NONHSAG012732	26:111
NONHSAG000134	619:203	NONHSAG030278	314:969	NONHSAG017742	28:961	NONHSAG012909	103:415	NONHSAG012739	1129:925
NONHSAG000134	661:921	NONHSAG030278	175:981	NONHSAG017742	702:67	NONHSAG012910	886:1109	NONHSAG012739	437:1051
NONHSAG000134	603:603	NONHSAG030278	308:303	NONHSAG017742	392:701	NONHSAG012910	854:399	NONHSAG012739	250:45
NONHSAG000134	97:1137	NONHSAG030325	783:751	NONHSAG017742	476:787	NONHSAG012910	29:697	NONHSAG012739	784:169
NONHSAG000134	518:691	NONHSAG030325	391:1	NONHSAG017742	255:845	NONHSAG012910	595:1111	NONHSAG012739	525:481
NONHSAG000134	194:787	NONHSAG030325	764:349	NONHSAG017742	745:483	NONHSAG012910	764:793	NONHSAG012739	340:159
NONHSAG000134	316:1107	NONHSAG030325	699:385	NONHSAG017742	465:1023	NONHSAG012910	1012:1141	NONHSAG012739	182:453
NONHSAG000134	384:403	NONHSAG030325	712:233	NONHSAG017775	126:789	NONHSAG012910	1136:365	NONHSAG012739	120:45
NONHSAG000134	542:85	NONHSAG030325	905:609	NONHSAG017775	321:1037	NONHSAG012910	817:1013	NONHSAG012739	1001:477
NONHSAG000134	1044:1149	NONHSAG030325	388:1031	NONHSAG017775	1067:519	NONHSAG012910	1053:921	NONHSAG012739	131:637
NONHSAG000134	1045:75	NONHSAG030325	702:631	NONHSAG017775	247:279	NONHSAG012920	114:199	NONHSAG012739	366:45
NONHSAG000134	404:173	NONHSAG030325	923:401	NONHSAG017775	756:169	NONHSAG012920	437:25	NONHSAG012744	369:751
NONHSAG000134	708:945	NONHSAG030325	810:1023	NONHSAG017775	662:1159	NONHSAG012920	913:817	NONHSAG012744	826:561
NONHSAG000134	305:775	NONHSAG030325	280:989	NONHSAG017775	374:23	NONHSAG012920	473:997	NONHSAG012744	784:225
NONHSAG000134	904:919	NONHSAG030326	408:587	NONHSAG017775	77:375	NONHSAG012920	459:523	NONHSAG012744	735:299
NONHSAG000134	1062:491	NONHSAG030326	183:711	NONHSAG017775	745:697	NONHSAG012920	748:1097	NONHSAG012744	721:975
NONHSAG000134	43:401	NONHSAG030326	712:697	NONHSAG017775	666:1029	NONHSAG012920	723:937	NONHSAG012744	156:241
NONHSAG000134	20:55	NONHSAG030326	302:963	NONHSAG017775	1052:29	NONHSAG012920	46:1055	NONHSAG012744	369:1155
NONHSAG000134	541:167	NONHSAG030326	90:507	NONHSAG017777	108:461	NONHSAG012920	362:1089	NONHSAG012744	1063:41
NONHSAG000134	122:459	NONHSAG030326	290:313	NONHSAG017777	960:77	NONHSAG012920	264:707	NONHSAG012744	151:877
NONHSAG000134	427:597	NONHSAG030326	1013:1085	NONHSAG017777	396:929	NONHSAG012920	839:1149	NONHSAG012744	712:197
NONHSAG000139	1137:617	NONHSAG030326	979:145	NONHSAG017777	636:293	NONHSAG012971	59:149	NONHSAG012745	18:859
NONHSAG000139	566:883	NONHSAG030326	928:1065	NONHSAG017777	846:249	NONHSAG012971	814:845	NONHSAG012745	472:1089
NONHSAG000139	65:337	NONHSAG030326	607:653	NONHSAG017777	118:811	NONHSAG012971	493:59	NONHSAG012745	603:457
NONHSAG000139	351:531	NONHSAG030326	628:515	NONHSAG017777	369:13	NONHSAG012971	137:1	NONHSAG051945	32:213
NONHSAG000139	800:877	NONHSAG030351	402:289	NONHSAG017777	390:727	NONHSAG012971	1138:887	NONHSAG051945	438:1023
NONHSAG000139	656:719	NONHSAG030351	819:711	NONHSAG017777	901:437	NONHSAG012971	280:743	NONHSAG051945	83:1091
NONHSAG000139	767:915	NONHSAG030351	338:325	NONHSAG017777	87:447	NONHSAG012971	257:589	NONHSAG051945	1035:889
NONHSAG000139	672:1023	NONHSAG030351	486:215	NONHSAG017777	722:615	NONHSAG012971	337:1	NONHSAG051945	89:627
NONHSAG000139	810:217	NONHSAG030351	356:49	NONHSAG017785	679:199	NONHSAG012971	278:467	NONHSAG051945	745:877
NONHSAG000139	780:489	NONHSAG030351	847:829	NONHSAG017785	704:713	NONHSAG012971	851:109	NONHSAG051945	754:587
NONHSAG000139	1018:267	NONHSAG030351	660:209	NONHSAG017785	989:339	NONHSAG012971	89:671	NONHSAG051945	405:1107
NONHSAG000169	780:631	NONHSAG030351	193:725	NONHSAG017785	123:689	NONHSAG012971	782:95	NONHSAG051945	688:271
NONHSAG000169	342:1041	NONHSAG030351	805:1085	NONHSAG017785	753:345	NONHSAG012971	954:363	NONHSAG051962	1049:1025
NONHSAG000169	166:843	NONHSAG030351	155:957	NONHSAG017785	812:871	NONHSAG012971	1118:93	NONHSAG051962	144:555
NONHSAG000169	566:233	NONHSAG030351	1138:233	NONHSAG017785	268:465	NONHSAG012971	601:533	NONHSAG051962	402:119
NONHSAG000169	1103:477	NONHSAG030358	408:1131	NONHSAG017785	287:1049	NONHSAG012971	652:295	NONHSAG051962	160:677
NONHSAG000169	530:157	NONHSAG030358	547:1145	NONHSAG017785	877:201	NONHSAG012971	158:437	NONHSAG051962	513:921
NONHSAG000169	808:851	NONHSAG030358	809:677	NONHSAG017785	351:923	NONHSAG012980	703:747	NONHSAG051962	1108:117
NONHSAG000169	388:413	NONHSAG030358	951:787	NONHSAG017785	1097:399	NONHSAG012980	705:67	NONHSAG051962	182:717
NONHSAG000169	1127:375	NONHSAG030358	959:945	NONHSAG017797	724:995	NONHSAG012980	814:153	NONHSAG051962	820:243
NONHSAG000169	637:577	NONHSAG030358	127:967	NONHSAG017797	1042:173	NONHSAG012980	116:199	NONHSAG051962	205:567
NONHSAG000174	258:357	NONHSAG030358	111:531	NONHSAG017797	7:753	NONHSAG012980	305:193	NONHSAG051962	354:251
NONHSAG000174	237:1137	NONHSAG030358	374:63	NONHSAG017797	656:149	NONHSAG012980	399:135	NONHSAG051962	71:971
NONHSAG000174	493:69	NONHSAG030374	680:171	NONHSAG017797	190:121	NONHSAG012980	1080:749	NONHSAG051968	175:1099
NONHSAG000174	705:607	NONHSAG030374	403:1043	NONHSAG017797	286:1045	NONHSAG012980	881:7	NONHSAG051968	311:1041
NONHSAG000174	1093:945	NONHSAG030374	645:1067	NONHSAG017797	577:1049	NONHSAG012980	365:357	NONHSAG051968	1087:801

NONHSAG000174	24:1153	NONHSAG030374	313:1011	NONHSAG017797	26:1105	NONHSAG012980	268:717	NONHSAG051968	1075:1039
NONHSAG000174	727:59	NONHSAG030374	450:49	NONHSAG017797	106:753	NONHSAG012986	90:705	NONHSAG051968	295:5
NONHSAG000174	1080:431	NONHSAG030374	1004:945	NONHSAG017797	923:925	NONHSAG012986	953:1089	NONHSAG051968	442:423
NONHSAG000174	961:585	NONHSAG030374	407:719	NONHSAG017797	693:911	NONHSAG012986	763:387	NONHSAG051968	349:859
NONHSAG000174	98:807	NONHSAG030374	331:885	NONHSAG017837	287:177	NONHSAG012986	907:855	NONHSAG051968	186:389
NONHSAG000174	804:73	NONHSAG030374	149:263	NONHSAG017837	69:599	NONHSAG013018	55:867	NONHSAG051968	112:715
NONHSAG000174	621:213	NONHSAG030400	1157:723	NONHSAG017837	1155:243	NONHSAG013018	997:269	NONHSAG051968	976:3
NONHSAG000174	733:555	NONHSAG030400	629:819	NONHSAG017837	252:931	NONHSAG013018	63:969	NONHSAG051968	783:353
NONHSAG000181	624:453	NONHSAG030400	1031:765	NONHSAG017837	714:823	NONHSAG013018	308:557	NONHSAG051985	184:1003
NONHSAG000181	486:627	NONHSAG030400	1038:1125	NONHSAG017837	271:849	NONHSAG013018	412:583	NONHSAG051985	511:465
NONHSAG000181	1020:611	NONHSAG030400	315:687	NONHSAG017837	875:207	NONHSAG013018	1101:903	NONHSAG051985	1029:649
NONHSAG000181	1135:661	NONHSAG030400	718:399	NONHSAG017837	926:429	NONHSAG013032	412:741	NONHSAG051985	814:765
NONHSAG000181	114:73	NONHSAG030400	313:1089	NONHSAG017837	662:457	NONHSAG013032	488:537	NONHSAG051985	294:187
NONHSAG000181	1128:1067	NONHSAG030400	346:1041	NONHSAG017855	444:1113	NONHSAG013032	279:591	NONHSAG051985	642:745
NONHSAG000181	531:517	NONHSAG030400	678:35	NONHSAG017855	1104:981	NONHSAG013032	841:651	NONHSAG051985	88:687
NONHSAG000181	494:411	NONHSAG030400	644:35	NONHSAG017855	601:1065	NONHSAG013032	1018:445	NONHSAG051985	290:353
NONHSAG000181	582:851	NONHSAG030400	1146:817	NONHSAG017855	314:1099	NONHSAG013032	1156:1001	NONHSAG051985	470:225
NONHSAG000181	784:1121	NONHSAG030406	298:85	NONHSAG017855	226:407	NONHSAG013032	961:161	NONHSAG051985	133:705
NONHSAG000181	497:985	NONHSAG030406	1128:1047	NONHSAG017855	42:803	NONHSAG013032	375:101	NONHSAG052010	729:125
NONHSAG000181	257:499	NONHSAG030406	259:601	NONHSAG017855	511:1079	NONHSAG013032	240:1155	NONHSAG052010	375:323
NONHSAG000181	523:319	NONHSAG030406	355:263	NONHSAG017855	783:345	NONHSAG013032	233:1125	NONHSAG052010	301:569
NONHSAG000181	859:229	NONHSAG030406	416:581	NONHSAG017855	859:197	NONHSAG013032	1056:207	NONHSAG052010	491:123
NONHSAG000181	562:505	NONHSAG030406	643:1075	NONHSAG017855	152:97	NONHSAG013032	479:499	NONHSAG052010	51:995
NONHSAG000181	775:387	NONHSAG030406	174:265	NONHSAG017868	1016:523	NONHSAG013032	1086:855	NONHSAG052010	1074:707
NONHSAG000181	1092:963	NONHSAG030406	957:71	NONHSAG017868	45:669	NONHSAG013032	400:1147	NONHSAG052010	1017:1113
NONHSAG000181	305:659	NONHSAG030406	331:627	NONHSAG017868	790:61	NONHSAG013032	192:239	NONHSAG052012	545:673
NONHSAG000181	523:1155	NONHSAG030406	42:373	NONHSAG017868	279:103	NONHSAG013032	102:673	NONHSAG052012	435:895
NONHSAG000181	777:531	NONHSAG030459	521:205	NONHSAG017868	1008:1033	NONHSAG013032	83:633	NONHSAG052012	903:1141
NONHSAG000181	842:133	NONHSAG030459	789:973	NONHSAG017868	1101:1081	NONHSAG013032	494:57	NONHSAG052012	389:225
NONHSAG000181	692:857	NONHSAG030459	372:581	NONHSAG017868	582:379	NONHSAG013032	214:309	NONHSAG052012	598:869
NONHSAG000181	407:1063	NONHSAG030459	252:395	NONHSAG017868	1026:219	NONHSAG013032	443:723	NONHSAG052012	436:487
NONHSAG000181	615:829	NONHSAG030459	207:9	NONHSAG017868	1081:475	NONHSAG013032	1056:1055	NONHSAG052012	110:311
NONHSAG000181	203:93	NONHSAG030459	373:39	NONHSAG017869	747:603	NONHSAG013037	30:281	NONHSAG052012	1034:545
NONHSAG000181	75:547	NONHSAG030459	79:53	NONHSAG017869	227:837	NONHSAG013037	399:811	NONHSAG052012	1115:587
NONHSAG000181	873:403	NONHSAG030459	969:269	NONHSAG017869	274:673	NONHSAG013037	1059:935	NONHSAG052012	232:213
NONHSAG000181	954:189	NONHSAG030459	164:279	NONHSAG017869	843:805	NONHSAG013037	958:247	NONHSAG052012	84:441
NONHSAG000181	676:49	NONHSAG030459	779:537	NONHSAG017869	830:1063	NONHSAG013037	867:439	NONHSAG052012	892:99
NONHSAG000181	485:125	NONHSAG030459	393:757	NONHSAG017869	91:183	NONHSAG013037	711:1115	NONHSAG052012	306:849
NONHSAG000181	18:943	NONHSAG030459	472:795	NONHSAG017869	126:111	NONHSAG013037	294:949	NONHSAG052012	1162:801
NONHSAG000181	108:489	NONHSAG030459	663:909	NONHSAG017869	722:605	NONHSAG013037	204:1025	NONHSAG052012	986:269
NONHSAG000181	1137:787	NONHSAG030459	459:315	NONHSAG017869	767:387	NONHSAG013037	962:395	NONHSAG052012	237:451
NONHSAG000181	589:863	NONHSAG030459	478:769	NONHSAG017885	881:401	NONHSAG013037	438:571	NONHSAG052012	482:51
NONHSAG000181	914:241	NONHSAG030459	881:203	NONHSAG017885	548:695	NONHSAG013037	371:361	NONHSAG052012	576:1035
NONHSAG000181	540:887	NONHSAG030469	67:251	NONHSAG017885	842:947	NONHSAG013037	990:859	NONHSAG052012	692:833
NONHSAG000181	1141:165	NONHSAG030469	719:993	NONHSAG017885	863:1037	NONHSAG013037	571:813	NONHSAG052012	491:1161
NONHSAG000181	644:1045	NONHSAG030469	494:1087	NONHSAG017885	899:763	NONHSAG013037	333:299	NONHSAG052012	1112:583
NONHSAG000185	888:621	NONHSAG030469	51:1083	NONHSAG017885	208:145	NONHSAG013037	286:707	NONHSAG052042	867:197
NONHSAG000185	50:425	NONHSAG030469	61:747	NONHSAG017885	815:275	NONHSAG013037	296:79	NONHSAG052042	750:1011
NONHSAG000185	931:359	NONHSAG030469	850:545	NONHSAG017885	676:345	NONHSAG013037	53:641	NONHSAG052042	587:243
NONHSAG000185	208:305	NONHSAG030469	753:109	NONHSAG017885	727:651	NONHSAG013037	513:607	NONHSAG052042	7:449
NONHSAG000185	26:859	NONHSAG030469	657:215	NONHSAG017885	517:293	NONHSAG013037	1015:307	NONHSAG052042	778:443
NONHSAG000185	124:783	NONHSAG030469	385:651	NONHSAG017885	414:1121	NONHSAG013037	1151:587	NONHSAG052042	561:1097
NONHSAG000185	658:949	NONHSAG030469	495:925	NONHSAG017934	2:1035	NONHSAG013037	1046:447	NONHSAG056034	311:483
NONHSAG000185	604:873	NONHSAG030469	209:49	NONHSAG017934	855:459	NONHSAG013039	450:815	NONHSAG056034	111:1005
NONHSAG000185	116:905	NONHSAG030469	687:877	NONHSAG017934	616:707	NONHSAG013039	239:83	NONHSAG056034	505:477
NONHSAG000199	705:353	NONHSAG030469	715:763	NONHSAG017934	244:501	NONHSAG013039	561:675	NONHSAG056034	426:1031
NONHSAG000199	295:1011	NONHSAG030469	118:1093	NONHSAG017934	94:1161	NONHSAG013039	55:721	NONHSAG056034	80:229
NONHSAG000199	761:865	NONHSAG030469	77:105	NONHSAG017934	1074:537	NONHSAG013039	360:721	NONHSAG056034	367:697
NONHSAG000199	291:527	NONHSAG030469	491:555	NONHSAG017934	754:477	NONHSAG013039	400:411	NONHSAG056034	942:297
NONHSAG000199	472:587	NONHSAG030469	509:953	NONHSAG017934	384:145	NONHSAG013039	681:207	NONHSAG056034	327:781
NONHSAG000199	134:195	NONHSAG030469	144:289	NONHSAG017934	1076:1027	NONHSAG013039	1100:717	NONHSAG056034	437:627
NONHSAG000199	847:781	NONHSAG030469	938:297	NONHSAG017934	455:165	NONHSAG013039	382:81	NONHSAG056034	499:391
NONHSAG000199	37:93	NONHSAG030469	205:127	NONHSAG017934	707:243	NONHSAG013039	707:535	NONHSAG056034	639:545
NONHSAG000199	318:1159	NONHSAG030469	683:709	NONHSAG017938	39:513	NONHSAG013039	385:989	NONHSAG056034	90:1051
NONHSAG000199	558:281	NONHSAG030483	123:923	NONHSAG017938	290:1117	NONHSAG013044	41:1035	NONHSAG052090	1001:881
NONHSAG000199	849:525	NONHSAG030483	128:687	NONHSAG017938	1090:93	NONHSAG013044	678:915	NONHSAG052090	676:511
NONHSAG000199	334:449	NONHSAG030483	21:87	NONHSAG017938	613:191	NONHSAG013044	37:103	NONHSAG052090	620:809
NONHSAG000199	257:319	NONHSAG030483	119:61	NONHSAG017938	883:3	NONHSAG013044	1094:1059	NONHSAG052090	521:595
NONHSAG000199	73:175	NONHSAG030483	1146:543	NONHSAG017938	1037:1005	NONHSAG013044	1116:203	NONHSAG052090	772:253
NONHSAG000199	149:903	NONHSAG030483	279:257	NONHSAG017938	996:963	NONHSAG013044	97:295	NONHSAG052090	27:1017
NONHSAG000199	20:973	NONHSAG030483	910:347	NONHSAG017938	1047:901	NONHSAG013044	513:1027	NONHSAG052090	108:57
NONHSAG000199	928:579	NONHSAG030483	12:1159	NONHSAG017938	283:967	NONHSAG013044	82:241	NONHSAG052090	717:881
NONHSAG000199	666:773	NONHSAG030483	1075:903	NONHSAG017938	55:475	NONHSAG013044	960:241	NONHSAG052090	1000:191
NONHSAG000199	1094:963	NONHSAG030483	410:669	NONHSAG017938	921:473	NONHSAG013044	866:1141	NONHSAG052090	225:155
NONHSAG000199	786:53	NONHSAG030489	1050:1021	NONHSAG017937	286:783	NONHSAG013044	866:41	NONHSAG052090	814:585
NONHSAG000199	999:87	NONHSAG030489	179:113	NONHSAG017937	655:421	NONHSAG013052	167:319	NONHSAG052090	94:243
NONHSAG000199	458:405	NONHSAG030489	491:203	NONHSAG017937	559:127	NONHSAG013052	373:1015	NONHSAG052090	1050:259
NONHSAG000202	411:115	NONHSAG030489	915:183	NONHSAG017937	771:1145	NONHSAG013052	928:537	NONHSAG052090	213:15
NONHSAG000202	327:1147	NONHSAG030489	392:639	NONHSAG017937	711:263	NONHSAG013052	605:39	NONHSAG052090	718:899
NONHSAG000202	367:885	NONHSAG030489	983:57	NONHSAG017937	865:879	NONHSAG013052	511:343	NONHSAG052090	455:575
NONHSAG000202	1105:743	NONHSAG030489	859:179	NONHSAG017937	532:245	NONHSAG013052	361:213	NONHSAG052090	3:973
NONHSAG000202	402:887	NONHSAG030489	825:897	NONHSAG017937	877:931	NONHSAG013052	321:865	NONHSAG052090	759:407
NONHSAG000202	1000:957	NONHSAG030489	725:359	NONHSAG017946	307:805	NONHSAG013052	931:207	NONHSAG052090	829:165
NONHSAG000202	703:295	NONHSAG030489	246:49	NONHSAG017946	909:111	NONHSAG013052	61:683	NONHSAG052090	665:431
NONHSAG000215	786:471	NONHSAG030489	905:51	NONHSAG017946	75:1045	NONHSAG013052	850:653	NONHSAG052090	307:255
NONHSAG000215	502:249	NONHSAG030489	471:1097	NONHSAG017946	70:965	NONHSAG013055	369:477	NONHSAG052090	23:797
NONHSAG000215	596:345	NONHSAG030489	138:769	NONHSAG017946	1066:263	NONHSAG013055	572:933	NONHSAG052112	928:663
NONHSAG000215	991:981	NONHSAG030489	424:621	NONHSAG017946	950:1041	NONHSAG013055	33:575	NONHSAG052112	1157:955

NONHSAG000215	438:793	NONHSAG030489	1033:243	NONHSAG017946	126:407	NONHSAG013055	1066:209	NONHSAG052112	731:67
NONHSAG000215	504:153	NONHSAG030489	460:785	NONHSAG017946	436:543	NONHSAG013055	365:107	NONHSAG052112	4:279
NONHSAG000215	1092:903	NONHSAG030489	708:909	NONHSAG017946	908:951	NONHSAG013055	888:43	NONHSAG052112	899:557
NONHSAG000215	492:595	NONHSAG030489	958:37	NONHSAG017946	927:225	NONHSAG013055	1080:997	NONHSAG052112	174:501
NONHSAG000215	880:67	NONHSAG030489	1137:1005	NONHSAG017946	502:379	NONHSAG013055	784:845	NONHSAG052112	1033:1031
NONHSAG000215	778:267	NONHSAG030489	1148:683	NONHSAG017952	3:1031	NONHSAG013055	28:227	NONHSAG052112	181:341
NONHSAG000215	184:425	NONHSAG030489	671:663	NONHSAG017952	803:751	NONHSAG013055	883:201	NONHSAG052112	324:271
NONHSAG000222	988:565	NONHSAG030493	332:539	NONHSAG017952	198:107	NONHSAG013055	841:603	NONHSAG052112	401:173
NONHSAG000222	555:105	NONHSAG030493	955:1119	NONHSAG017952	221:235	NONHSAG013061	801:515	NONHSAG052112	593:927
NONHSAG000222	979:1121	NONHSAG030493	488:125	NONHSAG017952	572:1057	NONHSAG013061	70:493	NONHSAG052118	699:623
NONHSAG000222	169:779	NONHSAG030493	947:447	NONHSAG017952	94:573	NONHSAG013061	583:1161	NONHSAG052118	584:809
NONHSAG000222	1064:807	NONHSAG030493	90:23	NONHSAG017952	621:49	NONHSAG013061	292:857	NONHSAG052118	105:619
NONHSAG000222	829:733	NONHSAG030493	669:333	NONHSAG017952	868:317	NONHSAG013061	903:53	NONHSAG052118	516:989
NONHSAG000222	515:1135	NONHSAG030493	632:11	NONHSAG017952	24:593	NONHSAG013061	297:203	NONHSAG052118	490:393
NONHSAG000222	439:17	NONHSAG030493	519:593	NONHSAG017952	236:1139	NONHSAG013061	1013:167	NONHSAG052118	1087:1139
NONHSAG000222	193:1155	NONHSAG030493	595:133	NONHSAG017952	889:1045	NONHSAG013061	726:787	NONHSAG052118	656:125
NONHSAG000222	698:95	NONHSAG030493	588:69	NONHSAG017952	506:1035	NONHSAG013061	472:377	NONHSAG052118	590:1101
NONHSAG000222	717:411	NONHSAG030495	607:273	NONHSAG017952	710:1101	NONHSAG013061	873:83	NONHSAG052118	822:87
NONHSAG000254	733:761	NONHSAG030495	594:175	NONHSAG017952	129:901	NONHSAG013061	130:357	NONHSAG052118	101:869
NONHSAG000254	733:659	NONHSAG030495	102:723	NONHSAG017952	1113:373	NONHSAG013087	10:371	NONHSAG052125	3:267
NONHSAG000254	468:203	NONHSAG030495	1076:135	NONHSAG017952	237:901	NONHSAG013087	134:23	NONHSAG052125	1099:677
NONHSAG000254	590:1111	NONHSAG030495	1030:521	NONHSAG017952	377:513	NONHSAG013087	764:1157	NONHSAG052125	844:495
NONHSAG000254	358:409	NONHSAG030495	87:331	NONHSAG017952	965:603	NONHSAG013087	46:525	NONHSAG052125	418:1161
NONHSAG000254	339:581	NONHSAG030495	297:1091	NONHSAG017952	779:605	NONHSAG013087	944:739	NONHSAG052126	1039:337
NONHSAG000254	435:393	NONHSAG030495	453:745	NONHSAG017952	95:573	NONHSAG013087	995:189	NONHSAG052126	25:409
NONHSAG000254	773:1015	NONHSAG030495	132:961	NONHSAG017952	313:257	NONHSAG013087	328:1051	NONHSAG052126	453:303
NONHSAG000254	1129:321	NONHSAG030495	1092:129	NONHSAG017952	109:169	NONHSAG013087	867:765	NONHSAG052126	1006:607
NONHSAG000254	895:773	NONHSAG030495	135:1017	NONHSAG017952	230:239	NONHSAG013087	271:101	NONHSAG052126	976:867
NONHSAG000264	38:773	NONHSAG030495	132:381	NONHSAG017952	428:343	NONHSAG013088	851:153	NONHSAG052126	470:739
NONHSAG000264	758:619	NONHSAG030495	1028:1037	NONHSAG017952	558:1127	NONHSAG013088	941:525	NONHSAG052126	455:1029
NONHSAG000264	482:73	NONHSAG030495	882:489	NONHSAG017952	220:235	NONHSAG013088	1019:557	NONHSAG052126	913:723
NONHSAG000264	791:649	NONHSAG030495	405:627	NONHSAG017952	279:651	NONHSAG013088	320:953	NONHSAG052126	71:525
NONHSAG000264	261:255	NONHSAG030495	223:41	NONHSAG017952	500:1011	NONHSAG013088	199:625	NONHSAG052126	33:311
NONHSAG000264	566:653	NONHSAG030495	305:303	NONHSAG017952	825:831	NONHSAG013088	86:563	NONHSAG052126	428:125
NONHSAG000264	666:775	NONHSAG030495	704:1101	NONHSAG017952	788:1067	NONHSAG013088	761:793	NONHSAG052157	80:183
NONHSAG000264	443:629	NONHSAG030495	973:73	NONHSAG017952	91:265	NONHSAG013088	47:457	NONHSAG052157	599:537
NONHSAG000264	893:945	NONHSAG030495	629:559	NONHSAG017952	505:1	NONHSAG013088	14:51	NONHSAG052157	479:21
NONHSAG000264	520:943	NONHSAG030495	162:507	NONHSAG017952	1090:1085	NONHSAG013088	645:487	NONHSAG052157	644:891
NONHSAG000267	323:401	NONHSAG030509	1027:519	NONHSAG017952	795:359	NONHSAG013088	271:627	NONHSAG052157	708:1151
NONHSAG000267	624:865	NONHSAG030509	48:719	NONHSAG017952	445:291	NONHSAG013092	707:883	NONHSAG052157	47:663
NONHSAG000267	654:211	NONHSAG030509	73:853	NONHSAG017952	1045:583	NONHSAG013092	58:153	NONHSAG052157	106:1147
NONHSAG000267	925:719	NONHSAG030509	895:1155	NONHSAG017952	509:739	NONHSAG013092	1108:1079	NONHSAG052157	1097:971
NONHSAG000267	648:1137	NONHSAG030509	1039:767	NONHSAG017952	801:371	NONHSAG013092	426:933	NONHSAG052157	981:275
NONHSAG000267	666:199	NONHSAG030509	1088:117	NONHSAG017967	687:557	NONHSAG013092	308:863	NONHSAG052157	434:395
NONHSAG000267	674:1143	NONHSAG030509	420:913	NONHSAG017967	969:719	NONHSAG013092	161:131	NONHSAG052157	772:899
NONHSAG000267	978:385	NONHSAG030509	750:155	NONHSAG017967	523:149	NONHSAG013092	563:927	NONHSAG052162	139:607
NONHSAG000267	759:939	NONHSAG030509	692:243	NONHSAG017967	630:941	NONHSAG013092	1028:423	NONHSAG052162	551:853
NONHSAG000267	101:1061	NONHSAG030509	1153:515	NONHSAG017967	1026:699	NONHSAG013092	57:685	NONHSAG052162	793:43
NONHSAG000267	14:165	NONHSAG030509	790:667	NONHSAG017967	1031:269	NONHSAG013092	81:1141	NONHSAG052162	1102:501
NONHSAG000267	1055:763	NONHSAG030613	683:707	NONHSAG017967	763:729	NONHSAG013093	675:853	NONHSAG052162	972:825
NONHSAG000267	470:289	NONHSAG030613	93:825	NONHSAG017967	201:757	NONHSAG013093	57:605	NONHSAG052162	119:1155
NONHSAG000267	532:197	NONHSAG030613	365:31	NONHSAG017967	592:1085	NONHSAG013093	886:231	NONHSAG052162	644:347
NONHSAG000267	936:909	NONHSAG030613	43:177	NONHSAG017967	704:1061	NONHSAG013093	262:113	NONHSAG052162	1151:41
NONHSAG000267	876:613	NONHSAG030613	45:43	NONHSAG017967	871:475	NONHSAG013093	1012:419	NONHSAG052162	534:1047
NONHSAG000267	68:89	NONHSAG030613	605:557	NONHSAG017971	151:923	NONHSAG013093	861:433	NONHSAG052162	266:787
NONHSAG000267	647:629	NONHSAG030613	230:15	NONHSAG017971	529:445	NONHSAG013093	430:829	NONHSAG052178	1155:661
NONHSAG000267	20:825	NONHSAG030613	315:175	NONHSAG017971	1125:79	NONHSAG013093	316:1027	NONHSAG052178	73:909
NONHSAG000267	927:803	NONHSAG030613	713:115	NONHSAG017971	133:255	NONHSAG013093	499:1071	NONHSAG052178	3:1011
NONHSAG000267	1045:359	NONHSAG030613	167:687	NONHSAG017971	372:49	NONHSAG013093	896:589	NONHSAG052178	265:401
NONHSAG000310	937:833	NONHSAG030613	453:3	NONHSAG017971	714:203	NONHSAG013093	416:791	NONHSAG052178	1013:717
NONHSAG000310	707:1125	NONHSAG030629	226:339	NONHSAG017971	513:213	NONHSAG013140	846:701	NONHSAG052178	388:355
NONHSAG000310	969:813	NONHSAG030629	1091:7	NONHSAG017971	77:233	NONHSAG013140	1057:161	NONHSAG052178	807:935
NONHSAG000310	116:643	NONHSAG030629	792:17	NONHSAG017971	973:421	NONHSAG013140	191:503	NONHSAG052178	412:145
NONHSAG000310	276:109	NONHSAG030629	264:57	NONHSAG017971	1036:943	NONHSAG013140	1078:661	NONHSAG052178	609:623
NONHSAG000310	668:265	NONHSAG030629	815:543	NONHSAG017971	112:23	NONHSAG013140	285:917	NONHSAG052178	426:519
NONHSAG000310	69:947	NONHSAG030629	13:331	NONHSAG017972	6:857	NONHSAG013140	238:241	NONHSAG052186	335:205
NONHSAG000310	58:193	NONHSAG030629	897:167	NONHSAG017972	851:95	NONHSAG013140	958:161	NONHSAG052186	112:719
NONHSAG000310	340:875	NONHSAG030629	463:833	NONHSAG017972	954:27	NONHSAG013140	1020:1075	NONHSAG052186	1121:683
NONHSAG000310	413:695	NONHSAG030629	653:141	NONHSAG017972	522:643	NONHSAG013140	709:579	NONHSAG052186	283:729
NONHSAG000312	478:517	NONHSAG030629	144:119	NONHSAG017972	670:209	NONHSAG013140	1039:261	NONHSAG052186	638:477
NONHSAG000312	1033:127	NONHSAG030633	903:17	NONHSAG017972	1081:619	NONHSAG013140	390:795	NONHSAG052186	1022:837
NONHSAG000312	732:915	NONHSAG030633	208:933	NONHSAG017972	41:1075	NONHSAG013147	945:251	NONHSAG052186	810:65
NONHSAG000312	1125:15	NONHSAG030633	506:955	NONHSAG017972	38:645	NONHSAG013147	737:513	NONHSAG052186	45:741
NONHSAG000312	1013:333	NONHSAG030633	171:651	NONHSAG017972	694:273	NONHSAG013147	1050:485	NONHSAG052186	789:797
NONHSAG000312	165:795	NONHSAG030633	960:685	NONHSAG017972	87:1017	NONHSAG013147	489:791	NONHSAG052188	551:1125
NONHSAG000312	1153:267	NONHSAG030633	442:829	NONHSAG017972	1109:151	NONHSAG013147	347:717	NONHSAG052188	805:33
NONHSAG000312	650:97	NONHSAG030633	1147:993	NONHSAG018000	161:321	NONHSAG013147	330:693	NONHSAG052188	744:243
NONHSAG000312	774:239	NONHSAG030633	385:1081	NONHSAG018000	699:311	NONHSAG013147	213:787	NONHSAG052188	146:745
NONHSAG000312	209:267	NONHSAG030633	246:263	NONHSAG018000	90:1037	NONHSAG013148	451:601	NONHSAG052188	78:207
NONHSAG000312	1077:897	NONHSAG030633	862:681	NONHSAG018000	790:865	NONHSAG013148	776:945	NONHSAG052188	770:949
NONHSAG000322	227:271	NONHSAG030634	1127:635	NONHSAG018000	491:349	NONHSAG013148	570:895	NONHSAG052188	936:137
NONHSAG000322	521:539	NONHSAG030634	634:839	NONHSAG018000	141:753	NONHSAG013148	1064:529	NONHSAG052188	241:153
NONHSAG000322	554:791	NONHSAG030634	553:495	NONHSAG018000	1064:1083	NONHSAG013148	655:981	NONHSAG052188	1082:649
NONHSAG000322	706:515	NONHSAG030634	459:513	NONHSAG018000	609:371	NONHSAG013148	299:479	NONHSAG052188	450:267
NONHSAG000322	80:893	NONHSAG030634	324:495	NONHSAG017996	455:85	NONHSAG013148	807:379	NONHSAG052188	454:75
NONHSAG000322	231:247	NONHSAG030634	126:567	NONHSAG017996	619:283	NONHSAG013148	422:799	NONHSAG052188	301:699
NONHSAG000322	705:515	NONHSAG030634	123:619	NONHSAG017996	408:1079	NONHSAG013148	589:623	NONHSAG052188	847:1035
NONHSAG000322	553:791	NONHSAG030634	1095:759	NONHSAG017996	969:671	NONHSAG013148	626:761	NONHSAG052188	107:55

NONHSAG000322	522:539	NONHSAG030634	14:589	NONHSAG017996	558:485	NONHSAG013176	614:589	NONHSAG052188	225:191
NONHSAG000322	225:55	NONHSAG030634	691:1029	NONHSAG017996	863:1043	NONHSAG013176	178:905	NONHSAG052188	111:977
NONHSAG000322	226:55	NONHSAG030634	492:767	NONHSAG017996	894:777	NONHSAG013176	54:655	NONHSAG052188	648:241
NONHSAG000329	590:33	NONHSAG030637	46:1029	NONHSAG017996	1050:1123	NONHSAG013176	739:451	NONHSAG052188	246:211
NONHSAG000329	1062:17	NONHSAG030637	863:765	NONHSAG017996	581:353	NONHSAG013176	761:193	NONHSAG052188	190:893
NONHSAG000329	99:1099	NONHSAG030637	217:377	NONHSAG017996	225:225	NONHSAG013176	217:809	NONHSAG052188	692:549
NONHSAG000329	768:353	NONHSAG030637	950:331	NONHSAG018018	963:563	NONHSAG013176	387:991	NONHSAG052188	76:109
NONHSAG000329	356:661	NONHSAG030637	595:307	NONHSAG018018	759:393	NONHSAG013176	106:1107	NONHSAG052188	217:573
NONHSAG000329	105:533	NONHSAG030637	359:373	NONHSAG018018	410:1037	NONHSAG013176	451:493	NONHSAG052409	137:1121
NONHSAG000329	948:75	NONHSAG030637	870:33	NONHSAG018018	779:351	NONHSAG013184	459:259	NONHSAG052409	201:1087
NONHSAG000329	954:1011	NONHSAG030637	531:939	NONHSAG018018	1041:1083	NONHSAG013184	421:713	NONHSAG052409	792:1047
NONHSAG000329	1052:257	NONHSAG030637	684:197	NONHSAG018018	1005:371	NONHSAG013184	530:195	NONHSAG052409	420:1111
NONHSAG000329	1141:463	NONHSAG030637	694:891	NONHSAG018018	641:847	NONHSAG013184	689:793	NONHSAG052409	846:971
NONHSAG000345	1047:175	NONHSAG030637	797:21	NONHSAG018018	301:371	NONHSAG013184	657:1131	NONHSAG052414	825:69
NONHSAG000345	501:759	NONHSAG030637	732:363	NONHSAG018018	825:727	NONHSAG013184	821:697	NONHSAG052414	429:735
NONHSAG000345	801:603	NONHSAG030637	305:181	NONHSAG018018	627:63	NONHSAG013184	155:1045	NONHSAG052414	784:833
NONHSAG000345	84:763	NONHSAG030637	514:173	NONHSAG018018	966:997	NONHSAG013184	867:739	NONHSAG052414	564:799
NONHSAG000345	62:137	NONHSAG030637	790:487	NONHSAG018031	427:383	NONHSAG013184	63:735	NONHSAG052414	128:641
NONHSAG000345	114:523	NONHSAG030637	266:479	NONHSAG018031	991:53	NONHSAG013184	743:761	NONHSAG052449	116:595
NONHSAG000364	775:945	NONHSAG030637	373:713	NONHSAG018031	383:771	NONHSAG013184	333:683	NONHSAG052449	107:459
NONHSAG000364	420:909	NONHSAG030637	314:1023	NONHSAG018031	27:295	NONHSAG013195	971:515	NONHSAG052449	707:209
NONHSAG000364	800:139	NONHSAG030637	1155:817	NONHSAG018031	922:763	NONHSAG013195	449:943	NONHSAG052449	641:909
NONHSAG000364	228:785	NONHSAG030637	410:279	NONHSAG018031	851:191	NONHSAG013195	564:101	NONHSAG052499	116:463
NONHSAG000364	1150:157	NONHSAG030637	978:45	NONHSAG018031	1003:679	NONHSAG013195	1106:645	NONHSAG052499	989:783
NONHSAG000364	904:1061	NONHSAG030637	1057:245	NONHSAG018031	904:781	NONHSAG013195	372:571	NONHSAG052499	602:855
NONHSAG000364	377:411	NONHSAG030676	80:1153	NONHSAG018031	1043:25	NONHSAG013195	291:929	NONHSAG052499	486:227
NONHSAG000364	309:547	NONHSAG030676	508:611	NONHSAG018031	91:805	NONHSAG013195	350:91	NONHSAG052499	233:1069
NONHSAG000364	318:263	NONHSAG030676	1071:1027	NONHSAG018031	158:289	NONHSAG013195	948:559	NONHSAG052499	579:899
NONHSAG000364	953:841	NONHSAG030676	851:101	NONHSAG018033	18:261	NONHSAG013195	1028:1029	NONHSAG052499	292:285
NONHSAG000364	892:687	NONHSAG030676	351:167	NONHSAG018033	1091:1141	NONHSAG013195	931:571	NONHSAG052499	1027:63
NONHSAG000381	549:765	NONHSAG030676	949:591	NONHSAG018033	143:637	NONHSAG013195	1115:423	NONHSAG052499	278:1009
NONHSAG000381	897:71	NONHSAG030676	259:523	NONHSAG018033	295:1013	NONHSAG013195	340:1089	NONHSAG052499	905:117
NONHSAG000381	820:295	NONHSAG030676	486:289	NONHSAG018033	391:551	NONHSAG013195	1086:289	NONHSAG052499	48:61
NONHSAG000381	833:293	NONHSAG030676	362:745	NONHSAG018033	115:21	NONHSAG013195	1105:795	NONHSAG052508	153:705
NONHSAG000381	533:537	NONHSAG030676	762:209	NONHSAG018033	281:293	NONHSAG013196	900:189	NONHSAG052508	670:579
NONHSAG000381	1156:509	NONHSAG030676	230:771	NONHSAG018033	295:11	NONHSAG013196	236:675	NONHSAG052508	431:59
NONHSAG000381	1011:773	NONHSAG030681	1077:301	NONHSAG018033	276:919	NONHSAG013196	555:941	NONHSAG052508	612:565
NONHSAG000381	107:403	NONHSAG030681	833:19	NONHSAG018033	1152:289	NONHSAG013196	970:527	NONHSAG052508	330:1065
NONHSAG000381	1018:1093	NONHSAG030681	853:1061	NONHSAG018033	316:729	NONHSAG013196	408:949	NONHSAG052508	274:613
NONHSAG000381	1119:31	NONHSAG030681	511:705	NONHSAG018039	314:207	NONHSAG013196	569:775	NONHSAG052508	1075:293
NONHSAG000381	1054:981	NONHSAG030681	920:141	NONHSAG018039	10:861	NONHSAG013196	991:717	NONHSAG052508	840:305
NONHSAG000395	558:355	NONHSAG030681	219:501	NONHSAG018039	656:259	NONHSAG013196	30:1051	NONHSAG052508	518:1159
NONHSAG000395	246:517	NONHSAG030681	49:1019	NONHSAG018039	989:115	NONHSAG013196	316:641	NONHSAG052508	432:837
NONHSAG000395	99:81	NONHSAG030681	939:163	NONHSAG018039	830:999	NONHSAG013196	330:671	NONHSAG052508	981:343
NONHSAG000395	318:955	NONHSAG030681	504:151	NONHSAG018039	1094:729	NONHSAG013196	544:527	NONHSAG052508	840:37
NONHSAG000395	600:525	NONHSAG030681	1120:393	NONHSAG018039	173:471	NONHSAG013196	774:885	NONHSAG052508	240:753
NONHSAG000395	470:229	NONHSAG030681	80:1051	NONHSAG018039	938:201	NONHSAG013196	494:305	NONHSAG052508	801:1097
NONHSAG000395	703:691	NONHSAG030683	924:511	NONHSAG018039	623:809	NONHSAG013213	1090:259	NONHSAG052508	1000:481
NONHSAG000395	370:349	NONHSAG030683	1024:35	NONHSAG018039	264:711	NONHSAG013213	407:681	NONHSAG052530	310:253
NONHSAG000395	402:605	NONHSAG030683	247:1113	NONHSAG018039	20:61	NONHSAG013213	441:149	NONHSAG052530	253:815
NONHSAG000395	334:587	NONHSAG030683	27:523	NONHSAG018040	1035:239	NONHSAG013213	727:1141	NONHSAG052530	45:925
NONHSAG000395	972:909	NONHSAG030683	325:1131	NONHSAG018040	368:905	NONHSAG013213	1047:189	NONHSAG052530	621:1149
NONHSAG000423	1000:1135	NONHSAG030683	894:1137	NONHSAG018040	679:155	NONHSAG013213	735:495	NONHSAG052530	123:529
NONHSAG000423	314:1125	NONHSAG030683	645:985	NONHSAG018040	1029:879	NONHSAG013213	404:717	NONHSAG052530	503:623
NONHSAG000423	772:355	NONHSAG030683	750:1077	NONHSAG018040	245:809	NONHSAG013213	1091:1035	NONHSAG052530	1105:1083
NONHSAG000423	507:697	NONHSAG030683	960:463	NONHSAG018040	8:719	NONHSAG013213	6:721	NONHSAG052530	837:733
NONHSAG000423	405:537	NONHSAG030683	448:277	NONHSAG018040	465:397	NONHSAG013213	928:889	NONHSAG052530	130:597
NONHSAG000423	61:1091	NONHSAG030683	145:803	NONHSAG018040	501:69	NONHSAG013213	943:713	NONHSAG052530	1109:341
NONHSAG000423	790:1011	NONHSAG030688	934:549	NONHSAG018040	48:445	NONHSAG013213	348:709	NONHSAG052530	340:881
NONHSAG000442	317:1113	NONHSAG030688	841:627	NONHSAG018040	44:119	NONHSAG013213	31:1139	NONHSAG052530	66:993
NONHSAG000442	99:653	NONHSAG030688	1130:983	NONHSAG018040	875:81	NONHSAG013213	378:635	NONHSAG052530	993:835
NONHSAG000442	734:219	NONHSAG030688	163:433	NONHSAG018059	104:903	NONHSAG013213	118:501	NONHSAG052530	128:167
NONHSAG000442	836:201	NONHSAG030688	215:1123	NONHSAG018059	171:73	NONHSAG013219	183:41	NONHSAG052530	1122:753
NONHSAG000442	52:697	NONHSAG030688	522:739	NONHSAG018059	353:1071	NONHSAG013219	1012:455	NONHSAG052530	398:21
NONHSAG000442	13:815	NONHSAG030688	591:73	NONHSAG018059	1109:239	NONHSAG013219	226:1159	NONHSAG052530	804:501
NONHSAG000442	803:853	NONHSAG030688	375:337	NONHSAG018059	615:721	NONHSAG013219	1043:337	NONHSAG052547	367:823
NONHSAG000442	386:1125	NONHSAG030688	486:847	NONHSAG018059	521:953	NONHSAG013219	282:877	NONHSAG052547	267:67
NONHSAG000476	336:79	NONHSAG030688	846:1015	NONHSAG018059	95:1093	NONHSAG013219	990:947	NONHSAG052547	281:539
NONHSAG000476	115:969	NONHSAG030688	1065:49	NONHSAG018059	353:911	NONHSAG013219	194:889	NONHSAG052547	565:589
NONHSAG000476	862:853	NONHSAG030757	588:797	NONHSAG018059	1131:929	NONHSAG013219	1146:857	NONHSAG052547	929:509
NONHSAG000476	1054:1129	NONHSAG030757	131:161	NONHSAG018059	562:499	NONHSAG013219	316:511	NONHSAG052547	114:477
NONHSAG000476	1011:791	NONHSAG030757	327:117	NONHSAG018075	228:693	NONHSAG013219	207:305	NONHSAG052547	473:819
NONHSAG000476	259:833	NONHSAG030757	726:333	NONHSAG018075	1014:801	NONHSAG013219	610:867	NONHSAG052547	1017:725
NONHSAG000490	405:297	NONHSAG030757	175:989	NONHSAG018075	336:521	NONHSAG013224	924:445	NONHSAG052547	80:809
NONHSAG000490	1086:897	NONHSAG030757	145:825	NONHSAG018075	1146:593	NONHSAG013224	31:229	NONHSAG052547	755:1097
NONHSAG000490	701:1097	NONHSAG030757	1096:321	NONHSAG018075	624:809	NONHSAG013224	417:987	NONHSAG052547	301:257
NONHSAG000490	893:561	NONHSAG030757	87:143	NONHSAG018075	1119:853	NONHSAG013224	186:647	NONHSAG052583	469:247
NONHSAG000490	63:533	NONHSAG030757	1052:1117	NONHSAG018098	776:325	NONHSAG013224	799:749	NONHSAG052583	238:141
NONHSAG000490	62:533	NONHSAG030757	21:583	NONHSAG018098	433:121	NONHSAG013224	613:351	NONHSAG052583	730:875
NONHSAG000490	342:407	NONHSAG030757	135:909	NONHSAG018098	1137:113	NONHSAG013224	801:537	NONHSAG052583	962:609
NONHSAG000490	892:317	NONHSAG030767	970:307	NONHSAG018098	938:571	NONHSAG013224	1041:7	NONHSAG052583	538:993
NONHSAG000490	1087:897	NONHSAG030767	152:839	NONHSAG018098	615:399	NONHSAG013224	1048:83	NONHSAG052583	1141:1045
NONHSAG000490	1073:791	NONHSAG030767	1074:865	NONHSAG018098	555:287	NONHSAG013224	524:739	NONHSAG052583	768:65
NONHSAG000490	879:957	NONHSAG030767	808:385	NONHSAG018098	416:213	NONHSAG013224	687:1053	NONHSAG052583	120:1125
NONHSAG000490	343:407	NONHSAG030767	1047:943	NONHSAG018098	796:181	NONHSAG013270	686:827	NONHSAG052583	748:851
NONHSAG000490	880:957	NONHSAG030767	625:841	NONHSAG018098	808:1	NONHSAG013270	483:539	NONHSAG052583	1099:577
NONHSAG000490	700:1097	NONHSAG030767	570:1043	NONHSAG018192	447:435	NONHSAG013270	275:747	NONHSAG052586	268:233
NONHSAG000490	675:437	NONHSAG030767	459:593	NONHSAG018192	543:33	NONHSAG013270	751:277	NONHSAG052586	1132:697

NONHSAG000490	155:1091	NONHSAG030767	1088:71	NONHSAG018192	766:335	NONHSAG013270	886:795	NONHSAG052586	2:907
NONHSAG000490	1072:791	NONHSAG030767	168:845	NONHSAG018192	22:891	NONHSAG013270	1148:5	NONHSAG052586	343:583
NONHSAG000490	892:561	NONHSAG030767	823:471	NONHSAG018192	682:183	NONHSAG013270	931:787	NONHSAG052586	380:269
NONHSAG000522	984:977	NONHSAG030783	46:991	NONHSAG018192	963:751	NONHSAG013270	73:41	NONHSAG052586	1119:1117
NONHSAG000522	185:225	NONHSAG030783	92:657	NONHSAG018192	626:457	NONHSAG013270	772:287	NONHSAG052586	843:1033
NONHSAG000522	1131:543	NONHSAG030783	1143:671	NONHSAG018192	377:89	NONHSAG013270	1091:927	NONHSAG052586	42:53
NONHSAG000522	1146:899	NONHSAG030783	357:983	NONHSAG018192	970:231	NONHSAG013289	83:1153	NONHSAG052586	906:911
NONHSAG000522	967:903	NONHSAG030783	758:855	NONHSAG018192	761:607	NONHSAG013289	652:1011	NONHSAG052609	219:1121
NONHSAG000522	378:693	NONHSAG030783	102:605	NONHSAG018192	304:495	NONHSAG013289	628:697	NONHSAG052609	1069:13
NONHSAG000522	149:255	NONHSAG030783	143:451	NONHSAG018224	915:655	NONHSAG013289	879:827	NONHSAG052609	687:779
NONHSAG000522	687:129	NONHSAG030783	557:451	NONHSAG018224	991:1159	NONHSAG013289	497:399	NONHSAG052609	522:531
NONHSAG000522	673:9	NONHSAG030783	1111:883	NONHSAG018224	756:7	NONHSAG013289	66:53	NONHSAG052609	435:1133
NONHSAG000522	497:171	NONHSAG030783	151:397	NONHSAG018224	774:299	NONHSAG013289	529:43	NONHSAG052609	1066:21
NONHSAG000522	125:635	NONHSAG030819	168:805	NONHSAG018224	879:143	NONHSAG013289	1048:33	NONHSAG052609	1127:319
NONHSAG000529	885:601	NONHSAG030819	110:593	NONHSAG018224	768:399	NONHSAG013289	833:721	NONHSAG052609	154:81
NONHSAG000529	834:651	NONHSAG030819	504:1037	NONHSAG018224	896:297	NONHSAG013289	280:239	NONHSAG052609	937:591
NONHSAG000529	545:27	NONHSAG030819	310:641	NONHSAG018224	229:791	NONHSAG013289	321:105	NONHSAG052609	753:643
NONHSAG000529	957:233	NONHSAG030819	960:35	NONHSAG018224	844:451	NONHSAG013312	1014:333	NONHSAG052609	693:1131
NONHSAG000529	1128:815	NONHSAG030819	77:587	NONHSAG018224	57:693	NONHSAG013312	289:671	NONHSAG052609	666:1109
NONHSAG000529	298:471	NONHSAG030819	138:599	NONHSAG018224	1088:873	NONHSAG013312	440:851	NONHSAG052609	1132:403
NONHSAG000529	734:65	NONHSAG030819	162:1047	NONHSAG018224	223:969	NONHSAG013312	674:569	NONHSAG052609	147:429
NONHSAG000529	1125:409	NONHSAG030819	886:91	NONHSAG018224	227:273	NONHSAG013312	231:535	NONHSAG052609	730:863
NONHSAG000529	994:761	NONHSAG030819	254:263	NONHSAG018224	775:65	NONHSAG013312	1056:213	NONHSAG052609	816:633
NONHSAG000546	345:1081	NONHSAG030819	858:801	NONHSAG018224	548:1019	NONHSAG013312	926:59	NONHSAG052609	212:857
NONHSAG000546	282:747	NONHSAG030850	83:743	NONHSAG018224	1134:807	NONHSAG013343	415:1093	NONHSAG052609	801:713
NONHSAG000546	426:423	NONHSAG030850	153:521	NONHSAG018224	808:627	NONHSAG013343	281:867	NONHSAG052609	365:523
NONHSAG000546	127:515	NONHSAG030850	161:1105	NONHSAG018224	142:897	NONHSAG013343	75:315	NONHSAG052620	358:835
NONHSAG000546	1096:693	NONHSAG030850	425:1079	NONHSAG018224	264:987	NONHSAG013343	435:1051	NONHSAG052620	502:527
NONHSAG000546	1013:445	NONHSAG030850	231:715	NONHSAG018224	609:241	NONHSAG013343	827:1035	NONHSAG052620	897:739
NONHSAG000546	833:817	NONHSAG030850	121:47	NONHSAG018224	141:897	NONHSAG013343	811:1081	NONHSAG052620	937:1063
NONHSAG000546	1143:917	NONHSAG030850	914:629	NONHSAG018224	354:601	NONHSAG013343	978:235	NONHSAG052620	86:881
NONHSAG000546	803:1005	NONHSAG030850	456:369	NONHSAG018224	230:815	NONHSAG013343	248:1099	NONHSAG052620	942:379
NONHSAG000546	98:365	NONHSAG030878	1035:497	NONHSAG018224	214:907	NONHSAG013343	358:1037	NONHSAG052620	639:1109
NONHSAG000546	926:491	NONHSAG030878	1047:505	NONHSAG018224	997:821	NONHSAG013343	1075:1137	NONHSAG052620	164:899
NONHSAG000628	421:347	NONHSAG030878	928:655	NONHSAG018224	878:143	NONHSAG013343	151:111	NONHSAG052620	574:1105
NONHSAG000628	442:329	NONHSAG030878	223:523	NONHSAG018224	474:15	NONHSAG056024	690:743	NONHSAG052620	61:1081
NONHSAG000628	540:73	NONHSAG030878	1088:345	NONHSAG018224	941:219	NONHSAG056024	982:981	NONHSAG052620	161:1003
NONHSAG000628	786:769	NONHSAG030878	288:1067	NONHSAG018224	4:1093	NONHSAG056024	607:437	NONHSAG052621	159:573
NONHSAG000628	1135:33	NONHSAG030878	642:17	NONHSAG018224	1062:675	NONHSAG056024	408:231	NONHSAG052621	296:523
NONHSAG000628	914:725	NONHSAG030878	799:253	NONHSAG018224	829:933	NONHSAG056024	145:447	NONHSAG052621	933:773
NONHSAG000628	700:429	NONHSAG030878	931:861	NONHSAG018224	374:475	NONHSAG056024	23:299	NONHSAG052621	995:903
NONHSAG000628	1031:347	NONHSAG030878	26:963	NONHSAG018224	608:241	NONHSAG056024	1051:433	NONHSAG052621	44:377
NONHSAG000628	813:201	NONHSAG030878	271:901	NONHSAG018224	53:429	NONHSAG056024	395:301	NONHSAG052621	488:739
NONHSAG000628	368:91	NONHSAG030888	912:255	NONHSAG018224	451:585	NONHSAG056024	613:363	NONHSAG052621	133:859
NONHSAG000628	531:233	NONHSAG030888	1127:55	NONHSAG018224	311:559	NONHSAG056024	428:563	NONHSAG052621	1155:191
NONHSAG000648	589:109	NONHSAG030888	727:81	NONHSAG018224	109:97	NONHSAG056024	293:245	NONHSAG052621	900:1063
NONHSAG000648	447:917	NONHSAG030888	367:45	NONHSAG018224	609:441	NONHSAG056024	958:397	NONHSAG052621	399:605
NONHSAG000648	528:805	NONHSAG030888	464:187	NONHSAG018224	148:301	NONHSAG056024	1098:117	NONHSAG052627	567:465
NONHSAG000648	853:275	NONHSAG030888	778:207	NONHSAG018224	907:621	NONHSAG056024	169:375	NONHSAG052627	905:621
NONHSAG000648	190:817	NONHSAG030888	1066:945	NONHSAG018224	1152:487	NONHSAG056024	516:971	NONHSAG052627	489:55
NONHSAG000648	5:623	NONHSAG030888	825:589	NONHSAG018224	454:359	NONHSAG056024	120:183	NONHSAG052627	793:65
NONHSAG000648	406:707	NONHSAG030888	316:153	NONHSAG018224	488:557	NONHSAG056024	701:175	NONHSAG052627	764:1027
NONHSAG000648	903:1037	NONHSAG030888	807:543	NONHSAG018230	662:147	NONHSAG056024	129:959	NONHSAG052627	22:199
NONHSAG000648	101:163	NONHSAG030933	385:593	NONHSAG018230	725:157	NONHSAG056024	938:829	NONHSAG052627	46:395
NONHSAG000648	352:841	NONHSAG030933	739:317	NONHSAG018230	262:79	NONHSAG056024	446:811	NONHSAG052627	1039:577
NONHSAG000648	756:667	NONHSAG030933	781:827	NONHSAG018230	948:889	NONHSAG056024	1012:883	NONHSAG052627	753:27
NONHSAG000648	896:85	NONHSAG030933	717:1127	NONHSAG018230	282:371	NONHSAG056024	201:499	NONHSAG052627	782:555
NONHSAG000648	877:35	NONHSAG030933	508:389	NONHSAG018230	683:659	NONHSAG056024	92:323	NONHSAG052636	359:5
NONHSAG000648	514:303	NONHSAG030933	379:1063	NONHSAG018230	150:895	NONHSAG056024	985:759	NONHSAG052636	1044:759
NONHSAG000648	858:361	NONHSAG030933	141:317	NONHSAG018230	596:151	NONHSAG013371	269:797	NONHSAG052636	245:557
NONHSAG000648	137:799	NONHSAG030933	1088:1025	NONHSAG018230	526:345	NONHSAG013371	1008:811	NONHSAG052636	196:927
NONHSAG000648	549:121	NONHSAG030933	1142:231	NONHSAG018230	317:937	NONHSAG013371	411:899	NONHSAG052636	940:881
NONHSAG000655	86:901	NONHSAG030933	868:635	NONHSAG018230	186:279	NONHSAG013371	172:409	NONHSAG052636	644:695
NONHSAG000655	78:621	NONHSAG030933	366:223	NONHSAG018271	394:949	NONHSAG013371	800:67	NONHSAG052636	724:983
NONHSAG000655	979:265	NONHSAG030937	186:659	NONHSAG018271	219:415	NONHSAG013371	11:59	NONHSAG052636	1140:961
NONHSAG000655	387:259	NONHSAG030937	544:333	NONHSAG018271	971:885	NONHSAG013371	713:541	NONHSAG052636	203:31
NONHSAG000655	701:85	NONHSAG030937	483:909	NONHSAG018271	756:1007	NONHSAG013371	498:887	NONHSAG052636	868:699
NONHSAG000655	1068:653	NONHSAG030937	42:785	NONHSAG018271	336:355	NONHSAG013371	42:105	NONHSAG052644	193:111
NONHSAG000655	1060:817	NONHSAG030937	366:789	NONHSAG018271	294:79	NONHSAG013371	793:907	NONHSAG052644	794:443
NONHSAG000655	342:21	NONHSAG030937	1074:457	NONHSAG018271	534:673	NONHSAG013371	1044:555	NONHSAG052644	1110:355
NONHSAG000655	416:45	NONHSAG030937	171:1079	NONHSAG018271	752:539	NONHSAG013371	636:829	NONHSAG052644	1065:875
NONHSAG000655	1107:929	NONHSAG030937	246:387	NONHSAG018271	627:997	NONHSAG013371	176:459	NONHSAG052644	113:13
NONHSAG000666	639:653	NONHSAG030940	375:409	NONHSAG018271	975:1011	NONHSAG013371	277:45	NONHSAG052644	130:215
NONHSAG000666	100:651	NONHSAG030940	221:1077	NONHSAG018271	90:425	NONHSAG013371	548:1131	NONHSAG052644	629:905
NONHSAG000666	829:437	NONHSAG030940	250:171	NONHSAG018332	1136:1059	NONHSAG013387	602:901	NONHSAG052644	26:379
NONHSAG000666	746:777	NONHSAG030940	742:415	NONHSAG018332	513:1075	NONHSAG013387	1096:1001	NONHSAG052644	554:985
NONHSAG000666	96:595	NONHSAG030940	871:889	NONHSAG018332	855:309	NONHSAG013387	815:995	NONHSAG052644	818:351
NONHSAG000666	453:347	NONHSAG030940	1079:355	NONHSAG018332	5:39	NONHSAG013387	1136:633	NONHSAG052644	413:763
NONHSAG000666	159:177	NONHSAG030957	899:635	NONHSAG018332	1155:209	NONHSAG013387	402:149	NONHSAG052726	64:975
NONHSAG000666	681:927	NONHSAG030957	966:669	NONHSAG018332	524:189	NONHSAG013387	194:1115	NONHSAG052726	352:709
NONHSAG000666	585:299	NONHSAG030957	354:113	NONHSAG018336	444:563	NONHSAG013387	890:487	NONHSAG052726	933:1037
NONHSAG000716	694:611	NONHSAG030957	284:791	NONHSAG018336	1086:657	NONHSAG013387	591:323	NONHSAG052726	858:1131
NONHSAG000716	163:857	NONHSAG030957	261:1141	NONHSAG018336	1009:891	NONHSAG013387	34:359	NONHSAG052726	980:747
NONHSAG000716	228:203	NONHSAG030957	1075:1111	NONHSAG018336	606:945	NONHSAG013387	963:905	NONHSAG052726	17:1133
NONHSAG000716	491:1039	NONHSAG030957	878:775	NONHSAG018336	739:281	NONHSAG013387	851:303	NONHSAG052726	528:829
NONHSAG000716	585:277	NONHSAG030957	143:107	NONHSAG018336	286:653	NONHSAG013400	876:373	NONHSAG052726	845:741
NONHSAG000716	1149:883	NONHSAG030957	944:795	NONHSAG018336	929:795	NONHSAG013400	1104:205	NONHSAG052726	751:737
NONHSAG000716	1138:1133	NONHSAG030957	882:51	NONHSAG018336	139:357	NONHSAG013400	606:261	NONHSAG052726	303:923

NONHSAG000716	307:701	NONHSAG030957	378:995	NONHSAG018336	281:681	NONHSAG013400	208:131	NONHSAG052726	1037:327
NONHSAG000716	765:781	NONHSAG030957	838:597	NONHSAG018336	272:111	NONHSAG013400	808:993	NONHSAG052726	298:1025
NONHSAG000716	907:1157	NONHSAG030957	448:869	NONHSAG018336	202:371	NONHSAG013400	1123:443	NONHSAG052726	594:467
NONHSAG000716	186:711	NONHSAG030957	105:499	NONHSAG021213	1140:719	NONHSAG013400	1040:1105	NONHSAG052726	386:1065
NONHSAG000738	209:199	NONHSAG030957	800:1109	NONHSAG021213	420:551	NONHSAG013400	1095:373	NONHSAG052726	198:501
NONHSAG000738	861:325	NONHSAG030957	441:289	NONHSAG021213	804:495	NONHSAG013400	38:301	NONHSAG052726	894:173
NONHSAG000738	341:229	NONHSAG030957	416:709	NONHSAG021213	606:799	NONHSAG013400	807:1147	NONHSAG052726	397:387
NONHSAG000738	722:117	NONHSAG030957	559:215	NONHSAG021241	941:37	NONHSAG013400	209:131	NONHSAG052726	252:293
NONHSAG000738	985:937	NONHSAG030957	1144:199	NONHSAG021241	490:347	NONHSAG013400	581:23	NONHSAG052734	258:31
NONHSAG000738	773:395	NONHSAG030957	816:205	NONHSAG021241	1139:501	NONHSAG013400	184:603	NONHSAG052734	361:65
NONHSAG000738	465:83	NONHSAG030971	131:575	NONHSAG021241	565:957	NONHSAG013400	155:433	NONHSAG052734	1055:225
NONHSAG000738	122:1097	NONHSAG030971	563:915	NONHSAG021241	256:321	NONHSAG013400	1035:365	NONHSAG052734	1035:621
NONHSAG000738	1014:1157	NONHSAG030971	860:155	NONHSAG021246	1107:59	NONHSAG013400	327:747	NONHSAG052734	1160:449
NONHSAG000738	136:79	NONHSAG030971	1054:51	NONHSAG021246	635:865	NONHSAG013400	786:1123	NONHSAG052734	727:877
NONHSAG000738	956:341	NONHSAG030971	725:285	NONHSAG021246	680:249	NONHSAG013400	183:603	NONHSAG052734	267:413
NONHSAG000751	167:411	NONHSAG030971	994:845	NONHSAG021246	741:227	NONHSAG013400	787:1123	NONHSAG052734	931:621
NONHSAG000751	1007:935	NONHSAG030971	539:1063	NONHSAG021246	383:411	NONHSAG013400	852:3	NONHSAG052734	196:1139
NONHSAG000751	248:503	NONHSAG030971	657:515	NONHSAG021246	642:553	NONHSAG013400	605:261	NONHSAG052734	1162:513
NONHSAG000751	143:307	NONHSAG030971	290:89	NONHSAG021246	1032:569	NONHSAG013400	554:615	NONHSAG052752	913:649
NONHSAG000751	541:899	NONHSAG030971	722:233	NONHSAG021246	1038:155	NONHSAG013400	39:503	NONHSAG052752	398:17
NONHSAG000769	558:449	NONHSAG030971	163:903	NONHSAG021246	484:977	NONHSAG013400	405:1095	NONHSAG052752	1162:623
NONHSAG000769	345:903	NONHSAG030992	1031:943	NONHSAG021246	675:755	NONHSAG013400	475:689	NONHSAG052752	476:737
NONHSAG000769	1003:133	NONHSAG030992	1014:427	NONHSAG021246	1:797	NONHSAG013400	978:173	NONHSAG052752	278:793
NONHSAG000769	1103:413	NONHSAG030992	873:309	NONHSAG021274	598:671	NONHSAG013400	1034:1089	NONHSAG052752	1147:889
NONHSAG000769	1083:1003	NONHSAG030992	608:59	NONHSAG021274	1068:721	NONHSAG013400	2:461	NONHSAG052752	1106:145
NONHSAG000769	1102:19	NONHSAG030992	850:913	NONHSAG021274	1094:43	NONHSAG013400	559:259	NONHSAG052752	746:489
NONHSAG000769	1128:1149	NONHSAG030992	811:47	NONHSAG021274	390:175	NONHSAG013400	746:847	NONHSAG052752	445:641
NONHSAG000769	444:541	NONHSAG030992	481:653	NONHSAG021274	526:83	NONHSAG013400	661:31	NONHSAG052752	440:261
NONHSAG000769	718:331	NONHSAG030992	968:1031	NONHSAG021274	210:1129	NONHSAG013400	341:771	NONHSAG052752	304:1031
NONHSAG000769	167:781	NONHSAG030992	772:463	NONHSAG021274	421:1037	NONHSAG013400	328:319	NONHSAG052756	473:479
NONHSAG000778	666:613	NONHSAG030992	865:1135	NONHSAG021274	952:755	NONHSAG013400	52:333	NONHSAG052756	466:971
NONHSAG000778	482:137	NONHSAG031103	248:117	NONHSAG021274	110:639	NONHSAG013400	39:301	NONHSAG052756	269:549
NONHSAG000778	515:539	NONHSAG031103	1138:271	NONHSAG021274	881:947	NONHSAG013400	64:931	NONHSAG052756	338:505
NONHSAG000778	857:1059	NONHSAG031103	799:653	NONHSAG021274	910:829	NONHSAG013413	980:1147	NONHSAG052756	120:1007
NONHSAG000778	374:891	NONHSAG031103	2:533	NONHSAG021287	470:569	NONHSAG013413	244:229	NONHSAG052756	139:1149
NONHSAG000778	791:817	NONHSAG031103	642:601	NONHSAG021287	1070:313	NONHSAG013413	991:493	NONHSAG052756	26:377
NONHSAG000778	1065:601	NONHSAG031103	528:255	NONHSAG021287	629:21	NONHSAG013413	782:437	NONHSAG052756	95:633
NONHSAG000778	1132:849	NONHSAG031103	170:55	NONHSAG021287	893:363	NONHSAG013413	1067:893	NONHSAG052756	484:537
NONHSAG000778	1026:867	NONHSAG031103	261:21	NONHSAG021287	999:267	NONHSAG013413	16:627	NONHSAG052756	944:1059
NONHSAG000812	182:807	NONHSAG031103	514:461	NONHSAG021287	294:607	NONHSAG013413	258:139	NONHSAG052756	222:431
NONHSAG000812	535:723	NONHSAG031103	221:573	NONHSAG021287	731:257	NONHSAG013413	792:877	NONHSAG052756	1022:191
NONHSAG000812	1040:661	NONHSAG031103	36:213	NONHSAG021287	168:479	NONHSAG013413	371:1005	NONHSAG052756	16:819
NONHSAG000812	104:1021	NONHSAG031103	875:261	NONHSAG021287	1005:919	NONHSAG013413	1119:505	NONHSAG052756	881:1033
NONHSAG000812	702:1083	NONHSAG031103	925:999	NONHSAG021287	834:989	NONHSAG013413	1026:345	NONHSAG052756	938:595
NONHSAG000812	156:631	NONHSAG031137	933:609	NONHSAG021287	149:429	NONHSAG013413	513:809	NONHSAG052756	698:809
NONHSAG000812	295:191	NONHSAG031137	1114:1023	NONHSAG021287	206:5	NONHSAG013413	1036:491	NONHSAG052756	794:713
NONHSAG000824	598:1095	NONHSAG031137	68:553	NONHSAG021287	529:919	NONHSAG013415	975:769	NONHSAG052756	373:673
NONHSAG000824	1085:263	NONHSAG031137	267:741	NONHSAG021287	860:897	NONHSAG013415	391:807	NONHSAG052756	172:35
NONHSAG000824	689:401	NONHSAG031167	861:1143	NONHSAG021287	972:1141	NONHSAG013415	871:617	NONHSAG052760	850:957
NONHSAG000824	737:1081	NONHSAG031167	902:845	NONHSAG021287	528:921	NONHSAG013415	298:75	NONHSAG052760	14:689
NONHSAG000824	181:635	NONHSAG031167	380:171	NONHSAG021287	17:55	NONHSAG013415	265:105	NONHSAG052760	229:697
NONHSAG000824	701:1157	NONHSAG031167	96:401	NONHSAG021287	1131:1087	NONHSAG013415	588:901	NONHSAG052760	60:743
NONHSAG000824	583:585	NONHSAG031167	487:1021	NONHSAG021287	272:763	NONHSAG013415	161:685	NONHSAG052760	739:1125
NONHSAG000824	291:363	NONHSAG031167	756:607	NONHSAG021287	1047:45	NONHSAG013415	933:539	NONHSAG052760	726:1
NONHSAG000824	635:123	NONHSAG031167	858:1093	NONHSAG021374	535:647	NONHSAG013415	1113:23	NONHSAG052760	506:115
NONHSAG000824	858:1087	NONHSAG031167	637:15	NONHSAG021374	1013:511	NONHSAG013415	1005:997	NONHSAG052760	52:653
NONHSAG000824	656:77	NONHSAG031167	709:1087	NONHSAG021374	567:289	NONHSAG013415	68:1137	NONHSAG052760	973:555
NONHSAG000824	77:353	NONHSAG031167	716:699	NONHSAG021374	395:587	NONHSAG013417	65:449	NONHSAG052760	1081:303
NONHSAG000824	549:1051	NONHSAG031167	393:17	NONHSAG021374	945:1089	NONHSAG013417	1032:1087	NONHSAG052760	934:839
NONHSAG000824	743:635	NONHSAG031167	65:835	NONHSAG021374	357:317	NONHSAG013417	397:1085	NONHSAG052772	839:1155
NONHSAG000824	964:603	NONHSAG031167	289:237	NONHSAG021374	63:703	NONHSAG013417	807:319	NONHSAG052772	780:983
NONHSAG000824	571:181	NONHSAG031167	549:743	NONHSAG021374	354:967	NONHSAG013417	1048:19	NONHSAG052772	1050:1057
NONHSAG000824	745:1057	NONHSAG031167	813:993	NONHSAG021374	1093:839	NONHSAG013413	152:237	NONHSAG052772	798:527
NONHSAG000824	88:61	NONHSAG031167	465:747	NONHSAG021374	859:327	NONHSAG013413	35:377	NONHSAG052772	562:785
NONHSAG000824	361:381	NONHSAG031168	33:453	NONHSAG021374	513:405	NONHSAG013413	253:19	NONHSAG052772	1110:571
NONHSAG000824	53:951	NONHSAG031168	194:653	NONHSAG021403	1070:461	NONHSAG013413	222:671	NONHSAG052772	582:55
NONHSAG000824	869:507	NONHSAG031168	765:1051	NONHSAG021403	1033:901	NONHSAG013418	529:943	NONHSAG052772	712:771
NONHSAG000824	601:185	NONHSAG031168	977:755	NONHSAG021403	670:761	NONHSAG013418	695:179	NONHSAG052772	115:1097
NONHSAG000847	129:1015	NONHSAG031168	754:1139	NONHSAG021403	29:411	NONHSAG013418	988:291	NONHSAG052772	832:1011
NONHSAG000847	221:825	NONHSAG031168	895:779	NONHSAG021403	808:261	NONHSAG013418	325:209	NONHSAG052776	516:1091
NONHSAG000847	1128:423	NONHSAG031168	317:993	NONHSAG021436	745:551	NONHSAG013418	805:75	NONHSAG052776	71:1045
NONHSAG000847	676:265	NONHSAG031168	364:871	NONHSAG021436	105:405	NONHSAG013418	553:1021	NONHSAG052776	949:299
NONHSAG000847	6:97	NONHSAG031168	487:61	NONHSAG021436	901:99	NONHSAG013418	1127:737	NONHSAG052776	1070:341
NONHSAG000847	405:895	NONHSAG031168	685:241	NONHSAG021436	635:747	NONHSAG013418	957:561	NONHSAG052776	716:823
NONHSAG000847	914:135	NONHSAG031168	806:189	NONHSAG021436	476:197	NONHSAG013418	29:1059	NONHSAG052776	969:1113
NONHSAG000847	183:267	NONHSAG031168	1120:355	NONHSAG021436	956:1131	NONHSAG013418	452:775	NONHSAG052776	1013:887
NONHSAG000847	628:1001	NONHSAG031168	846:545	NONHSAG021436	109:271	NONHSAG013418	684:489	NONHSAG052776	1041:1117
NONHSAG000847	702:761	NONHSAG031168	937:1053	NONHSAG021436	720:577	NONHSAG013418	520:577	NONHSAG052776	475:123
NONHSAG000847	785:1101	NONHSAG031168	249:179	NONHSAG021436	837:169	NONHSAG013418	917:881	NONHSAG052776	468:1161
NONHSAG000847	60:821	NONHSAG031168	155:1153	NONHSAG021444	762:287	NONHSAG013418	990:661	NONHSAG052817	523:217
NONHSAG000847	1096:817	NONHSAG031168	634:335	NONHSAG021444	846:163	NONHSAG013418	1028:871	NONHSAG052817	300:1003
NONHSAG000847	1119:553	NONHSAG031168	946:1117	NONHSAG021444	940:747	NONHSAG013418	208:965	NONHSAG052817	855:667
NONHSAG000847	968:383	NONHSAG031168	270:587	NONHSAG021444	274:641	NONHSAG013418	61:793	NONHSAG052818	668:297
NONHSAG000847	446:85	NONHSAG031168	651:997	NONHSAG021444	576:467	NONHSAG013418	21:237	NONHSAG052818	227:1027
NONHSAG000847	552:781	NONHSAG031168	44:985	NONHSAG021459	753:543	NONHSAG013418	1162:527	NONHSAG052818	301:201
NONHSAG000847	1033:791	NONHSAG031178	415:999	NONHSAG021459	1056:127	NONHSAG013418	268:969	NONHSAG052818	399:233
NONHSAG000847	841:481	NONHSAG031178	356:461	NONHSAG021459	702:981	NONHSAG013418	1003:181	NONHSAG052817	74:995
NONHSAG000884	824:571	NONHSAG031178	954:889	NONHSAG021459	564:825	NONHSAG013418	535:643	NONHSAG052817	388:649

NONHSAG000884	614:289	NONHSAG031178	501:651	NONHSAG021459	107:379	NONHSAG013413	1069:1029	NONHSAG052817	783:439
NONHSAG000884	1007:51	NONHSAG031178	729:1069	NONHSAG021459	480:279	NONHSAG013429	820:169	NONHSAG052817	299:393
NONHSAG000884	223:297	NONHSAG031178	977:1161	NONHSAG021459	788:69	NONHSAG013429	677:369	NONHSAG052837	118:163
NONHSAG000884	333:55	NONHSAG031178	60:1055	NONHSAG021459	522:625	NONHSAG013429	690:837	NONHSAG052837	567:273
NONHSAG000884	298:17	NONHSAG031178	500:353	NONHSAG021459	763:25	NONHSAG013429	101:265	NONHSAG052837	766:853
NONHSAG000884	573:1131	NONHSAG031178	410:1053	NONHSAG021459	343:853	NONHSAG013429	665:725	NONHSAG052837	534:883
NONHSAG000884	269:795	NONHSAG031178	211:695	NONHSAG021464	173:125	NONHSAG013429	64:751	NONHSAG052837	208:43
NONHSAG000884	422:733	NONHSAG031178	37:581	NONHSAG021464	1012:49	NONHSAG013429	1127:583	NONHSAG052837	630:757
NONHSAG000884	676:43	NONHSAG031180	149:791	NONHSAG021464	601:23	NONHSAG013429	776:741	NONHSAG052837	671:71
NONHSAG000884	929:635	NONHSAG031180	96:677	NONHSAG021464	697:1031	NONHSAG013429	427:575	NONHSAG052837	577:375
NONHSAG000939	868:821	NONHSAG031180	575:127	NONHSAG021464	419:1035	NONHSAG013429	1061:1115	NONHSAG052837	557:183
NONHSAG000939	367:307	NONHSAG031180	654:761	NONHSAG021464	1014:131	NONHSAG013429	570:637	NONHSAG052837	127:199
NONHSAG000939	587:287	NONHSAG031180	592:391	NONHSAG021464	778:137	NONHSAG013433	1090:61	NONHSAG052837	248:91
NONHSAG000939	444:407	NONHSAG031180	593:369	NONHSAG021464	851:829	NONHSAG013433	966:9	NONHSAG052837	253:283
NONHSAG000939	900:37	NONHSAG031180	530:251	NONHSAG021464	392:173	NONHSAG013433	659:619	NONHSAG052837	343:813
NONHSAG000939	597:279	NONHSAG031180	267:815	NONHSAG021479	825:521	NONHSAG013433	519:939	NONHSAG052837	656:1057
NONHSAG000946	2:915	NONHSAG031184	403:553	NONHSAG021479	1056:657	NONHSAG013433	254:1147	NONHSAG052837	137:579
NONHSAG000946	742:913	NONHSAG031184	838:511	NONHSAG021479	257:189	NONHSAG013433	486:823	NONHSAG052837	302:487
NONHSAG000946	929:807	NONHSAG031184	843:173	NONHSAG021479	564:117	NONHSAG013433	773:1063	NONHSAG052837	404:819
NONHSAG000946	1106:641	NONHSAG031184	864:149	NONHSAG021479	428:389	NONHSAG013433	481:253	NONHSAG052837	410:581
NONHSAG000946	1048:809	NONHSAG031184	1099:499	NONHSAG021479	358:727	NONHSAG013433	214:471	NONHSAG052837	293:241
NONHSAG000946	254:411	NONHSAG031184	1023:889	NONHSAG021479	872:293	NONHSAG013433	744:719	NONHSAG052837	421:81
NONHSAG000946	339:491	NONHSAG031184	369:205	NONHSAG021479	373:1107	NONHSAG013433	10:1041	NONHSAG052837	757:1127
NONHSAG000946	493:887	NONHSAG031184	810:155	NONHSAG021479	410:927	NONHSAG013449	143:937	NONHSAG052837	385:405
NONHSAG000946	713:885	NONHSAG031184	536:949	NONHSAG021479	1138:261	NONHSAG013449	947:327	NONHSAG052837	142:289
NONHSAG000946	53:1089	NONHSAG031184	600:617	NONHSAG021479	688:135	NONHSAG013449	1111:57	NONHSAG052837	457:705
NONHSAG000946	688:549	NONHSAG031184	7:39	NONHSAG021502	1015:169	NONHSAG013449	148:461	NONHSAG052879	1091:1051
NONHSAG000965	551:605	NONHSAG031203	274:171	NONHSAG021502	761:461	NONHSAG013449	354:125	NONHSAG052879	224:779
NONHSAG000965	226:923	NONHSAG031203	984:121	NONHSAG021502	565:509	NONHSAG013449	190:883	NONHSAG052879	502:1101
NONHSAG000965	765:863	NONHSAG031203	711:925	NONHSAG021502	813:1097	NONHSAG013449	1061:207	NONHSAG052879	188:277
NONHSAG000965	430:835	NONHSAG031203	772:1101	NONHSAG021502	684:1027	NONHSAG013449	952:779	NONHSAG052879	1007:595
NONHSAG000965	725:1081	NONHSAG031203	1011:181	NONHSAG021502	634:105	NONHSAG013472	1035:209	NONHSAG052880	538:899
NONHSAG000965	726:1045	NONHSAG031203	335:767	NONHSAG021502	531:257	NONHSAG013472	871:255	NONHSAG052880	30:527
NONHSAG000965	353:823	NONHSAG031203	7:703	NONHSAG021502	804:5	NONHSAG013472	708:285	NONHSAG052880	348:73
NONHSAG000965	109:1063	NONHSAG031203	1052:251	NONHSAG021502	508:893	NONHSAG013472	763:491	NONHSAG052880	763:943
NONHSAG000965	1017:985	NONHSAG031203	853:81	NONHSAG021502	187:407	NONHSAG013472	303:779	NONHSAG052880	788:187
NONHSAG000965	838:75	NONHSAG031203	733:1011	NONHSAG021504	792:273	NONHSAG013472	975:745	NONHSAG052880	1090:939
NONHSAG000965	559:1075	NONHSAG031203	324:229	NONHSAG021504	706:545	NONHSAG013472	306:777	NONHSAG052880	757:631
NONHSAG000972	774:261	NONHSAG031219	699:693	NONHSAG021504	1141:39	NONHSAG013472	548:273	NONHSAG052880	1107:143
NONHSAG000972	158:601	NONHSAG031219	1002:1	NONHSAG021504	784:887	NONHSAG013472	955:115	NONHSAG052880	1145:485
NONHSAG000972	295:947	NONHSAG031219	970:1149	NONHSAG021504	425:763	NONHSAG013472	633:627	NONHSAG052880	838:619
NONHSAG000972	276:585	NONHSAG031219	581:79	NONHSAG021504	849:959	NONHSAG013488	933:1051	NONHSAG052880	49:841
NONHSAG000972	405:345	NONHSAG031219	942:1079	NONHSAG021504	414:1145	NONHSAG013488	1149:477	NONHSAG052885	908:603
NONHSAG000972	283:101	NONHSAG031219	933:671	NONHSAG021504	478:557	NONHSAG013488	308:23	NONHSAG052885	168:1129
NONHSAG000972	791:825	NONHSAG031219	921:465	NONHSAG021504	1021:917	NONHSAG013488	378:489	NONHSAG052885	998:445
NONHSAG000972	645:429	NONHSAG031219	911:809	NONHSAG021504	85:95	NONHSAG013488	111:345	NONHSAG052885	524:901
NONHSAG000972	47:681	NONHSAG031219	397:1043	NONHSAG021505	507:91	NONHSAG013488	447:931	NONHSAG052885	651:865
NONHSAG000972	978:663	NONHSAG031219	955:827	NONHSAG021505	1029:695	NONHSAG013488	339:151	NONHSAG052885	1018:501
NONHSAG000976	139:1135	NONHSAG031219	800:629	NONHSAG021505	932:407	NONHSAG013488	956:159	NONHSAG052885	81:811
NONHSAG000976	122:887	NONHSAG031232	1124:93	NONHSAG021505	11:863	NONHSAG013488	695:255	NONHSAG052885	1136:821
NONHSAG000976	335:349	NONHSAG031232	496:67	NONHSAG021505	850:961	NONHSAG013488	549:893	NONHSAG052885	259:701
NONHSAG000976	1127:1113	NONHSAG031232	1139:519	NONHSAG021505	583:621	NONHSAG013488	1125:737	NONHSAG052885	475:261
NONHSAG000976	172:819	NONHSAG031232	858:133	NONHSAG021505	315:565	NONHSAG013488	54:421	NONHSAG052886	456:857
NONHSAG000976	1009:457	NONHSAG031232	264:67	NONHSAG021505	524:503	NONHSAG013488	744:231	NONHSAG052886	224:1135
NONHSAG000976	17:747	NONHSAG031232	75:359	NONHSAG021505	695:153	NONHSAG013488	247:837	NONHSAG052886	703:961
NONHSAG000976	76:433	NONHSAG031232	933:839	NONHSAG021505	108:341	NONHSAG013488	893:31	NONHSAG052886	395:793
NONHSAG000976	767:311	NONHSAG031232	36:285	NONHSAG021537	611:71	NONHSAG013488	177:711	NONHSAG052886	1131:807
NONHSAG000976	745:347	NONHSAG031232	397:889	NONHSAG021537	433:841	NONHSAG013488	771:233	NONHSAG052886	9:1085
NONHSAG000976	627:41	NONHSAG031232	63:121	NONHSAG021537	1139:1149	NONHSAG013488	333:213	NONHSAG052886	612:969
NONHSAG000979	701:819	NONHSAG031237	542:953	NONHSAG021537	939:1143	NONHSAG013488	645:1103	NONHSAG052886	108:873
NONHSAG000979	748:477	NONHSAG031237	1006:201	NONHSAG021537	852:911	NONHSAG013488	273:415	NONHSAG052886	702:147
NONHSAG000979	1147:1099	NONHSAG031237	649:551	NONHSAG021537	86:1137	NONHSAG013488	386:361	NONHSAG052886	1132:1059
NONHSAG000979	471:1113	NONHSAG031237	440:665	NONHSAG021537	565:853	NONHSAG013488	92:33	NONHSAG052890	377:277
NONHSAG000979	912:935	NONHSAG031237	858:581	NONHSAG021537	729:157	NONHSAG013488	549:657	NONHSAG052890	222:641
NONHSAG000979	772:61	NONHSAG031237	526:253	NONHSAG021537	645:1149	NONHSAG013488	685:773	NONHSAG052890	607:1095
NONHSAG000979	302:795	NONHSAG031237	975:999	NONHSAG021537	798:135	NONHSAG013488	501:747	NONHSAG052890	1062:1075
NONHSAG000979	1132:457	NONHSAG031237	880:73	NONHSAG021537	997:749	NONHSAG013488	1035:445	NONHSAG052890	972:999
NONHSAG000979	420:81	NONHSAG031237	399:223	NONHSAG021537	594:1021	NONHSAG013488	384:493	NONHSAG052890	973:207
NONHSAG000979	24:439	NONHSAG031237	503:227	NONHSAG021537	1079:1113	NONHSAG013488	124:353	NONHSAG052890	205:273
NONHSAG000979	1099:931	NONHSAG031237	731:535	NONHSAG021537	1100:197	NONHSAG013488	469:245	NONHSAG052890	708:633
NONHSAG000979	627:709	NONHSAG031249	54:909	NONHSAG021537	233:721	NONHSAG013488	58:537	NONHSAG052890	380:529
NONHSAG001012	573:27	NONHSAG031249	1130:913	NONHSAG021537	1089:227	NONHSAG013488	215:377	NONHSAG052890	506:755
NONHSAG001012	907:843	NONHSAG031249	122:685	NONHSAG021537	834:1159	NONHSAG013488	610:995	NONHSAG052890	183:341
NONHSAG001012	846:469	NONHSAG031249	253:53	NONHSAG021537	692:205	NONHSAG013488	204:687	NONHSAG052890	636:395
NONHSAG001012	1113:715	NONHSAG031249	412:897	NONHSAG021537	1095:775	NONHSAG013488	328:87	NONHSAG052890	335:313
NONHSAG001012	872:273	NONHSAG031255	546:405	NONHSAG021537	1117:1055	NONHSAG013488	369:47	NONHSAG052890	950:75
NONHSAG001012	150:607	NONHSAG031255	1096:639	NONHSAG021537	545:1027	NONHSAG013488	273:1155	NONHSAG052890	882:505
NONHSAG001012	1113:491	NONHSAG031255	100:1069	NONHSAG021537	1072:979	NONHSAG013488	51:1009	NONHSAG052890	404:629
NONHSAG001012	449:177	NONHSAG031255	506:877	NONHSAG021537	568:757	NONHSAG013488	322:1147	NONHSAG052890	820:133
NONHSAG001012	509:687	NONHSAG031255	454:365	NONHSAG021537	460:537	NONHSAG013488	111:1149	NONHSAG052890	1125:719
NONHSAG001012	817:695	NONHSAG031255	377:283	NONHSAG021537	883:505	NONHSAG013488	562:409	NONHSAG052890	1051:1143
NONHSAG001012	474:713	NONHSAG031255	471:277	NONHSAG021537	669:59	NONHSAG013488	895:1059	NONHSAG052890	653:495
NONHSAG001012	744:1029	NONHSAG031255	341:93	NONHSAG021537	949:701	NONHSAG013488	827:361	NONHSAG052890	228:311
NONHSAG001012	434:741	NONHSAG031255	668:257	NONHSAG021537	78:893	NONHSAG013496	144:1141	NONHSAG052890	235:841
NONHSAG001012	455:431	NONHSAG031255	483:235	NONHSAG021537	1146:653	NONHSAG013496	1013:19	NONHSAG052890	885:1047
NONHSAG001012	192:379	NONHSAG031255	1068:1109	NONHSAG021537	32:85	NONHSAG013496	21:693	NONHSAG052890	1103:997
NONHSAG001012	346:629	NONHSAG031255	567:611	NONHSAG021546	593:1065	NONHSAG013496	991:11	NONHSAG052890	44:341
NONHSAG001012	244:805	NONHSAG031255	1048:35	NONHSAG021546	551:423	NONHSAG013496	570:859	NONHSAG052890	149:753

NONHSAG001012	227:699	NONHSAG031255	742:1011	NONHSAG021546	1157:547	NONHSAG013496	487:511	NONHSAG052890	189:723
NONHSAG001012	265:1121	NONHSAG031255	140:831	NONHSAG021546	592:993	NONHSAG013496	158:1105	NONHSAG052890	944:867
NONHSAG001012	477:1105	NONHSAG031276	784:17	NONHSAG021546	113:867	NONHSAG013496	511:1083	NONHSAG052890	79:883
NONHSAG001012	600:11	NONHSAG031276	532:601	NONHSAG021546	173:749	NONHSAG013496	652:137	NONHSAG052890	123:953
NONHSAG001018	158:71	NONHSAG031276	1141:303	NONHSAG021546	944:735	NONHSAG013496	302:521	NONHSAG052915	977:413
NONHSAG001018	76:851	NONHSAG031276	358:743	NONHSAG021546	290:515	NONHSAG013496	860:869	NONHSAG052915	1160:995
NONHSAG001018	834:533	NONHSAG031276	1040:1017	NONHSAG021546	495:909	NONHSAG013505	180:849	NONHSAG052915	838:1135
NONHSAG001018	172:403	NONHSAG031276	358:659	NONHSAG021557	379:47	NONHSAG013505	297:175	NONHSAG052915	601:649
NONHSAG001018	1149:473	NONHSAG031276	844:367	NONHSAG021557	816:843	NONHSAG013505	397:697	NONHSAG052915	1044:121
NONHSAG001018	1033:967	NONHSAG031276	931:1059	NONHSAG021557	821:221	NONHSAG013505	1046:1109	NONHSAG052915	1014:669
NONHSAG001018	812:1017	NONHSAG031276	679:615	NONHSAG021557	702:663	NONHSAG013505	310:763	NONHSAG052915	435:107
NONHSAG001018	603:1141	NONHSAG031276	380:615	NONHSAG021557	420:549	NONHSAG013505	999:619	NONHSAG052915	1130:163
NONHSAG001018	153:809	NONHSAG031276	961:745	NONHSAG021557	529:369	NONHSAG013505	448:417	NONHSAG052915	1099:611
NONHSAG001018	321:1015	NONHSAG031296	956:545	NONHSAG021557	70:1103	NONHSAG013505	232:385	NONHSAG052915	25:547
NONHSAG001018	478:279	NONHSAG031296	34:109	NONHSAG021557	580:957	NONHSAG013505	240:417	NONHSAG052915	891:1041
NONHSAG001018	696:283	NONHSAG031296	1007:1143	NONHSAG021557	1155:65	NONHSAG013505	292:339	NONHSAG052978	856:253
NONHSAG001018	57:839	NONHSAG031296	393:153	NONHSAG021557	324:857	NONHSAG013505	1108:161	NONHSAG052978	951:775
NONHSAG001018	193:863	NONHSAG031296	931:551	NONHSAG021577	116:481	NONHSAG013505	467:963	NONHSAG052978	744:907
NONHSAG001018	893:17	NONHSAG031296	1134:399	NONHSAG021577	362:391	NONHSAG013505	390:207	NONHSAG052978	906:397
NONHSAG001018	1064:171	NONHSAG031296	335:753	NONHSAG021577	22:623	NONHSAG013505	564:791	NONHSAG052978	545:693
NONHSAG001018	548:1099	NONHSAG031296	62:25	NONHSAG021577	777:1121	NONHSAG013505	470:527	NONHSAG052978	958:491
NONHSAG001018	287:575	NONHSAG031296	1119:833	NONHSAG021577	664:999	NONHSAG013505	19:569	NONHSAG052978	678:1067
NONHSAG001018	80:267	NONHSAG031296	498:917	NONHSAG021577	587:397	NONHSAG013505	887:85	NONHSAG052978	565:1071
NONHSAG001041	860:85	NONHSAG031296	870:521	NONHSAG021577	31:261	NONHSAG013505	94:913	NONHSAG052978	257:145
NONHSAG001041	923:217	NONHSAG031312	320:157	NONHSAG021577	835:77	NONHSAG013505	199:583	NONHSAG052978	973:451
NONHSAG001041	443:281	NONHSAG031312	495:499	NONHSAG021577	704:423	NONHSAG013505	309:763	NONHSAG052978	669:73
NONHSAG001041	395:673	NONHSAG031312	355:543	NONHSAG021582	658:525	NONHSAG013505	967:317	NONHSAG052978	249:697
NONHSAG001041	1010:283	NONHSAG031312	188:251	NONHSAG021582	543:539	NONHSAG013505	1136:91	NONHSAG052978	968:227
NONHSAG001041	864:587	NONHSAG031312	769:997	NONHSAG021582	649:1123	NONHSAG013509	428:721	NONHSAG052978	78:997
NONHSAG001041	89:1015	NONHSAG031312	425:613	NONHSAG021582	1051:1063	NONHSAG013509	960:571	NONHSAG052978	838:29
NONHSAG001041	877:721	NONHSAG031312	973:1151	NONHSAG021608	976:561	NONHSAG013509	709:607	NONHSAG052978	1161:135
NONHSAG001041	967:613	NONHSAG031312	130:155	NONHSAG021608	104:223	NONHSAG013509	9:267	NONHSAG052978	939:489
NONHSAG001041	1094:753	NONHSAG031312	712:223	NONHSAG021608	374:1065	NONHSAG013509	499:783	NONHSAG052978	995:835
NONHSAG001041	510:557	NONHSAG031312	480:481	NONHSAG021608	569:483	NONHSAG013509	999:219	NONHSAG052998	709:59
NONHSAG001041	1064:373	NONHSAG031312	360:1003	NONHSAG021608	923:881	NONHSAG013509	823:65	NONHSAG052998	148:83
NONHSAG001041	621:925	NONHSAG031314	144:375	NONHSAG021608	402:649	NONHSAG013509	656:1103	NONHSAG052998	999:1049
NONHSAG001041	997:509	NONHSAG031314	1162:451	NONHSAG021608	1135:1139	NONHSAG013509	618:207	NONHSAG052998	396:9
NONHSAG001041	844:581	NONHSAG031314	159:67	NONHSAG021608	824:141	NONHSAG013509	178:527	NONHSAG052998	356:817
NONHSAG001041	151:571	NONHSAG031314	271:493	NONHSAG021608	670:575	NONHSAG013521	608:179	NONHSAG052998	601:553
NONHSAG001041	915:273	NONHSAG031314	222:553	NONHSAG021608	931:929	NONHSAG013521	994:1153	NONHSAG052998	845:583
NONHSAG001041	115:201	NONHSAG031314	464:543	NONHSAG021608	601:113	NONHSAG013521	487:281	NONHSAG052998	683:855
NONHSAG001041	260:857	NONHSAG031314	515:131	NONHSAG021662	261:945	NONHSAG013521	803:719	NONHSAG052998	92:553
NONHSAG001043	4:253	NONHSAG031314	1131:665	NONHSAG021662	303:907	NONHSAG013521	1146:715	NONHSAG052998	335:587
NONHSAG001043	768:447	NONHSAG031314	918:493	NONHSAG021662	488:799	NONHSAG013521	401:5	NONHSAG052998	245:889
NONHSAG001043	1001:481	NONHSAG031314	452:51	NONHSAG021662	595:171	NONHSAG013525	565:227	NONHSAG053000	866:633
NONHSAG001043	1:1041	NONHSAG031315	1081:241	NONHSAG021662	351:175	NONHSAG013525	290:117	NONHSAG053000	332:707
NONHSAG001043	565:365	NONHSAG031315	630:561	NONHSAG021662	1160:593	NONHSAG013525	135:1063	NONHSAG053000	646:1039
NONHSAG001043	239:745	NONHSAG031315	135:739	NONHSAG021662	660:1135	NONHSAG013525	1115:305	NONHSAG053000	918:751
NONHSAG001043	1138:197	NONHSAG031315	996:1019	NONHSAG021662	276:311	NONHSAG013525	1024:991	NONHSAG053000	13:1081
NONHSAG001043	238:1017	NONHSAG031315	317:85	NONHSAG021662	929:419	NONHSAG013525	678:1011	NONHSAG053000	276:43
NONHSAG001043	1159:891	NONHSAG031315	306:985	NONHSAG021662	1074:897	NONHSAG013525	220:745	NONHSAG053000	569:897
NONHSAG001043	290:685	NONHSAG031315	844:151	NONHSAG021662	944:597	NONHSAG013525	729:385	NONHSAG053000	824:477
NONHSAG001075	648:761	NONHSAG031315	623:439	NONHSAG021671	1011:607	NONHSAG013525	993:645	NONHSAG053000	210:257
NONHSAG001075	203:721	NONHSAG031315	1066:125	NONHSAG021671	513:201	NONHSAG013525	718:1075	NONHSAG053032	632:29
NONHSAG001075	486:967	NONHSAG031315	385:469	NONHSAG021671	293:113	NONHSAG013525	833:83	NONHSAG053032	752:55
NONHSAG001075	577:209	NONHSAG031315	304:801	NONHSAG021671	620:233	NONHSAG013533	955:381	NONHSAG053032	481:183
NONHSAG001075	417:483	NONHSAG031322	911:935	NONHSAG021671	456:185	NONHSAG013533	883:375	NONHSAG053032	641:775
NONHSAG001075	555:881	NONHSAG031322	767:359	NONHSAG021671	669:819	NONHSAG013533	761:225	NONHSAG053032	761:125
NONHSAG001075	574:189	NONHSAG031322	171:1135	NONHSAG021671	13:1021	NONHSAG013533	434:1065	NONHSAG053032	1024:1117
NONHSAG001075	1035:843	NONHSAG031322	946:823	NONHSAG021671	405:857	NONHSAG013533	1071:43	NONHSAG053032	519:915
NONHSAG001075	126:93	NONHSAG031322	513:615	NONHSAG021671	637:59	NONHSAG013533	813:279	NONHSAG053032	1080:89
NONHSAG001075	930:667	NONHSAG031322	1132:63	NONHSAG021671	120:707	NONHSAG013533	325:1087	NONHSAG053032	502:69
NONHSAG001075	112:93	NONHSAG031322	193:321	NONHSAG021671	885:125	NONHSAG013533	1047:277	NONHSAG053032	413:197
NONHSAG001146	1064:409	NONHSAG031322	654:369	NONHSAG021671	717:621	NONHSAG013533	1061:445	NONHSAG053081	1053:721
NONHSAG001146	516:547	NONHSAG031322	651:189	NONHSAG021671	726:685	NONHSAG013533	608:287	NONHSAG053081	998:583
NONHSAG001146	301:93	NONHSAG031335	831:195	NONHSAG021671	926:213	NONHSAG013546	150:221	NONHSAG053081	1044:717
NONHSAG001146	284:151	NONHSAG031335	119:795	NONHSAG021671	982:775	NONHSAG013546	29:1021	NONHSAG053081	637:1069
NONHSAG001146	981:1037	NONHSAG031335	839:165	NONHSAG021671	856:187	NONHSAG013546	600:189	NONHSAG053081	1011:845
NONHSAG001146	1061:25	NONHSAG031335	1075:17	NONHSAG021671	1129:671	NONHSAG013546	255:629	NONHSAG053081	1047:147
NONHSAG001146	418:815	NONHSAG031335	683:735	NONHSAG021671	317:101	NONHSAG013546	382:563	NONHSAG053081	197:1111
NONHSAG001146	194:63	NONHSAG031335	78:21	NONHSAG021671	922:849	NONHSAG013546	1027:223	NONHSAG053081	437:1139
NONHSAG001146	821:547	NONHSAG031335	934:759	NONHSAG021671	807:45	NONHSAG013546	513:211	NONHSAG053081	862:3
NONHSAG001146	185:763	NONHSAG031335	836:1063	NONHSAG021671	58:119	NONHSAG013546	160:91	NONHSAG053081	681:283
NONHSAG001146	582:145	NONHSAG031335	714:711	NONHSAG021671	304:1039	NONHSAG013546	1013:257	NONHSAG053081	56:175
NONHSAG001146	970:783	NONHSAG031335	914:941	NONHSAG021696	149:805	NONHSAG013546	610:435	NONHSAG053118	244:109
NONHSAG001146	323:247	NONHSAG031372	868:667	NONHSAG021696	498:919	NONHSAG013546	1152:255	NONHSAG053118	840:315
NONHSAG001146	797:1149	NONHSAG031372	781:819	NONHSAG021696	832:965	NONHSAG013584	20:759	NONHSAG053118	446:201
NONHSAG001146	797:1033	NONHSAG031372	992:15	NONHSAG021696	86:157	NONHSAG013584	963:165	NONHSAG053118	432:621
NONHSAG001146	256:263	NONHSAG031372	536:241	NONHSAG021696	161:453	NONHSAG013584	195:1159	NONHSAG053118	461:355
NONHSAG001146	946:747	NONHSAG031372	522:363	NONHSAG021696	558:27	NONHSAG013584	69:1143	NONHSAG053118	567:657
NONHSAG001146	288:215	NONHSAG031372	610:393	NONHSAG021696	690:447	NONHSAG013584	547:927	NONHSAG053118	801:569
NONHSAG001146	857:161	NONHSAG031372	1115:577	NONHSAG021696	54:349	NONHSAG013584	557:683	NONHSAG053118	687:279
NONHSAG001146	388:575	NONHSAG031372	920:645	NONHSAG021696	150:951	NONHSAG013584	1038:27	NONHSAG053118	76:775
NONHSAG001146	60:443	NONHSAG031372	1059:1009	NONHSAG021696	387:395	NONHSAG013584	1125:685	NONHSAG053118	830:355
NONHSAG001146	488:217	NONHSAG031372	597:635	NONHSAG021696	774:751	NONHSAG013584	344:201	NONHSAG053119	1082:73
NONHSAG001183	729:147	NONHSAG031372	984:55	NONHSAG021696	815:333	NONHSAG013584	965:119	NONHSAG053119	107:233
NONHSAG001183	840:705	NONHSAG031372	145:925	NONHSAG021696	376:177	NONHSAG013584	196:237	NONHSAG053119	90:251
NONHSAG001183	937:375	NONHSAG031372	911:429	NONHSAG021696	401:625	NONHSAG013584	825:797	NONHSAG053119	1160:651

NONHSAG001183	940:443	NONHSAG031372	686:533	NONHSAG021696	718:489	NONHSAG013584	471:773	NONHSAG053119	1077:1043
NONHSAG001183	65:957	NONHSAG031372	341:43	NONHSAG021696	233:975	NONHSAG013584	22:739	NONHSAG053119	992:273
NONHSAG001183	36:735	NONHSAG031372	483:95	NONHSAG021696	893:883	NONHSAG013584	737:99	NONHSAG053119	1107:595
NONHSAG001183	669:669	NONHSAG031372	784:975	NONHSAG021696	131:559	NONHSAG013584	143:333	NONHSAG053119	306:1055
NONHSAG001183	861:303	NONHSAG031372	75:1089	NONHSAG021696	631:959	NONHSAG013584	693:211	NONHSAG053119	1113:713
NONHSAG001183	586:107	NONHSAG031372	557:197	NONHSAG021696	1063:531	NONHSAG013584	255:847	NONHSAG053119	1005:1079
NONHSAG001183	657:883	NONHSAG031372	844:273	NONHSAG021696	531:341	NONHSAG013596	86:307	NONHSAG053119	916:973
NONHSAG001183	56:831	NONHSAG031372	60:61	NONHSAG021696	631:191	NONHSAG013596	343:381	NONHSAG053167	269:209
NONHSAG001185	1015:697	NONHSAG031372	884:229	NONHSAG021696	557:1137	NONHSAG013596	906:635	NONHSAG053167	804:357
NONHSAG001185	430:1159	NONHSAG031377	118:577	NONHSAG021696	985:1143	NONHSAG013596	4:1135	NONHSAG053167	973:363
NONHSAG001185	842:943	NONHSAG031377	604:797	NONHSAG021696	55:1027	NONHSAG013596	293:213	NONHSAG053167	801:1071
NONHSAG001185	982:133	NONHSAG031377	1078:539	NONHSAG021696	943:1029	NONHSAG013596	488:1031	NONHSAG053167	1059:707
NONHSAG001185	586:621	NONHSAG031377	70:973	NONHSAG021696	270:939	NONHSAG013596	932:695	NONHSAG053167	287:999
NONHSAG001185	357:1087	NONHSAG031377	991:37	NONHSAG021696	389:615	NONHSAG013596	302:197	NONHSAG053167	815:1101
NONHSAG001185	1071:117	NONHSAG031377	842:813	NONHSAG021696	552:299	NONHSAG013596	223:449	NONHSAG053167	469:365
NONHSAG001185	410:881	NONHSAG031385	885:455	NONHSAG021696	1014:403	NONHSAG013596	409:985	NONHSAG053167	149:1107
NONHSAG001185	918:813	NONHSAG031385	399:95	NONHSAG021696	522:71	NONHSAG013596	161:451	NONHSAG053167	399:189
NONHSAG001185	1064:241	NONHSAG031385	789:71	NONHSAG021701	451:947	NONHSAG013596	366:611	NONHSAG053167	627:711
NONHSAG001211	1108:533	NONHSAG031385	112:657	NONHSAG021701	498:331	NONHSAG013596	1000:1111	NONHSAG053167	218:1059
NONHSAG001211	631:951	NONHSAG031385	883:603	NONHSAG021701	695:699	NONHSAG013596	150:689	NONHSAG053167	1020:241
NONHSAG001211	1045:659	NONHSAG031385	766:809	NONHSAG021701	967:759	NONHSAG013596	998:821	NONHSAG053167	823:1155
NONHSAG001211	1020:285	NONHSAG031385	1049:199	NONHSAG021701	959:481	NONHSAG013596	993:599	NONHSAG053167	1115:757
NONHSAG001211	648:381	NONHSAG031385	199:793	NONHSAG021701	77:1161	NONHSAG013596	9:27	NONHSAG053167	735:1117
NONHSAG001211	465:743	NONHSAG031385	556:211	NONHSAG021701	979:111	NONHSAG013596	690:271	NONHSAG053167	556:345
NONHSAG001211	118:217	NONHSAG031401	444:183	NONHSAG021701	1039:903	NONHSAG013596	935:709	NONHSAG053167	878:509
NONHSAG001211	855:789	NONHSAG031401	154:511	NONHSAG021701	209:379	NONHSAG013596	171:933	NONHSAG053167	562:707
NONHSAG001211	221:685	NONHSAG031401	88:609	NONHSAG021701	472:171	NONHSAG013596	1106:1113	NONHSAG053167	659:315
NONHSAG001211	448:347	NONHSAG031401	945:75	NONHSAG021701	559:407	NONHSAG013596	911:413	NONHSAG053167	1089:891
NONHSAG001211	260:925	NONHSAG031401	820:819	NONHSAG021701	935:223	NONHSAG013596	1054:243	NONHSAG053167	207:279
NONHSAG001222	131:835	NONHSAG031401	499:333	NONHSAG021701	960:901	NONHSAG013596	956:693	NONHSAG053168	596:1003
NONHSAG001222	355:267	NONHSAG031401	1025:1005	NONHSAG021701	200:513	NONHSAG013596	697:19	NONHSAG053168	1075:715
NONHSAG001222	844:743	NONHSAG031401	565:1009	NONHSAG021701	1124:487	NONHSAG013596	770:93	NONHSAG053168	36:303
NONHSAG001222	90:105	NONHSAG031401	1105:103	NONHSAG021701	220:575	NONHSAG013596	804:1045	NONHSAG053168	684:511
NONHSAG001222	823:311	NONHSAG031401	445:555	NONHSAG021701	1127:875	NONHSAG013596	538:477	NONHSAG053168	1101:291
NONHSAG001222	777:217	NONHSAG031461	414:1081	NONHSAG021701	238:725	NONHSAG013596	383:711	NONHSAG053168	291:555
NONHSAG001222	1003:645	NONHSAG031461	627:399	NONHSAG021701	600:337	NONHSAG013598	242:57	NONHSAG053168	504:703
NONHSAG001222	1144:43	NONHSAG031461	118:539	NONHSAG021701	369:423	NONHSAG013598	50:15	NONHSAG053168	864:809
NONHSAG001222	335:329	NONHSAG031461	97:41	NONHSAG021701	465:635	NONHSAG013598	976:203	NONHSAG053168	492:547
NONHSAG001222	307:609	NONHSAG031461	297:443	NONHSAG021701	963:365	NONHSAG013598	299:1085	NONHSAG053168	314:1147
NONHSAG001222	884:435	NONHSAG031461	397:195	NONHSAG021701	17:345	NONHSAG013598	913:151	NONHSAG053168	77:1027
NONHSAG001244	726:865	NONHSAG031461	1117:513	NONHSAG021701	637:127	NONHSAG013598	1156:901	NONHSAG053205	1008:1095
NONHSAG001244	1033:169	NONHSAG031461	366:511	NONHSAG021701	628:627	NONHSAG013598	837:1003	NONHSAG053205	901:447
NONHSAG001244	710:133	NONHSAG031461	458:71	NONHSAG021701	53:981	NONHSAG013598	1044:33	NONHSAG053205	464:735
NONHSAG001244	1012:109	NONHSAG031461	739:889	NONHSAG021701	143:695	NONHSAG013598	1135:315	NONHSAG053205	793:651
NONHSAG001244	323:473	NONHSAG031461	734:669	NONHSAG021701	829:367	NONHSAG013598	328:67	NONHSAG053205	267:1033
NONHSAG001244	1145:199	NONHSAG031476	487:757	NONHSAG021701	286:945	NONHSAG013598	970:699	NONHSAG053205	6:893
NONHSAG001244	303:335	NONHSAG031476	752:1149	NONHSAG021701	103:423	NONHSAG013622	564:795	NONHSAG053205	20:781
NONHSAG001244	1023:595	NONHSAG031476	34:989	NONHSAG021701	609:1085	NONHSAG013622	1074:1059	NONHSAG053205	1080:695
NONHSAG001244	828:893	NONHSAG031476	155:1155	NONHSAG021701	1028:929	NONHSAG013622	1142:827	NONHSAG053205	754:401
NONHSAG001244	1113:29	NONHSAG031476	324:343	NONHSAG021701	254:735	NONHSAG013622	923:565	NONHSAG053205	630:1111
NONHSAG001246	1029:959	NONHSAG031476	818:967	NONHSAG021755	992:999	NONHSAG013622	905:687	NONHSAG053205	813:1007
NONHSAG001246	161:587	NONHSAG031476	1083:415	NONHSAG021755	840:517	NONHSAG013622	999:715	NONHSAG053209	582:243
NONHSAG001246	91:649	NONHSAG031476	955:739	NONHSAG021755	44:607	NONHSAG013622	522:835	NONHSAG053209	243:637
NONHSAG001246	993:889	NONHSAG031476	1123:885	NONHSAG021755	1033:99	NONHSAG013622	572:1033	NONHSAG053209	108:405
NONHSAG001246	171:265	NONHSAG031476	608:341	NONHSAG021755	802:651	NONHSAG013622	887:369	NONHSAG053209	1116:93
NONHSAG001246	751:471	NONHSAG031476	415:1001	NONHSAG021755	413:173	NONHSAG013622	996:741	NONHSAG053209	129:669
NONHSAG001246	511:103	NONHSAG031485	1093:445	NONHSAG021755	519:1011	NONHSAG013622	1064:371	NONHSAG053209	411:1155
NONHSAG001246	504:603	NONHSAG031485	962:659	NONHSAG021755	294:1119	NONHSAG013653	1064:5	NONHSAG053209	1123:191
NONHSAG001247	953:79	NONHSAG031485	973:287	NONHSAG021755	884:337	NONHSAG013653	579:65	NONHSAG053209	41:1067
NONHSAG001247	1126:843	NONHSAG031485	244:755	NONHSAG021755	648:605	NONHSAG013653	808:669	NONHSAG053209	590:393
NONHSAG001247	196:665	NONHSAG031485	190:617	NONHSAG021755	346:415	NONHSAG013653	1060:691	NONHSAG053209	909:349
NONHSAG001247	462:855	NONHSAG031485	226:1141	NONHSAG021758	832:623	NONHSAG013653	35:761	NONHSAG053209	919:1029
NONHSAG001247	159:655	NONHSAG031485	49:427	NONHSAG021758	1012:951	NONHSAG013653	313:715	NONHSAG053219	864:109
NONHSAG001247	44:709	NONHSAG031485	286:419	NONHSAG021758	107:923	NONHSAG013653	890:1153	NONHSAG053219	982:469
NONHSAG001247	272:933	NONHSAG031485	299:1141	NONHSAG021758	1128:839	NONHSAG013653	473:933	NONHSAG053219	379:971
NONHSAG001247	474:573	NONHSAG031485	348:161	NONHSAG021758	153:243	NONHSAG013653	971:103	NONHSAG053219	314:215
NONHSAG001247	25:159	NONHSAG031485	598:151	NONHSAG021758	475:373	NONHSAG013655	112:885	NONHSAG053219	256:659
NONHSAG001247	521:727	NONHSAG031485	431:695	NONHSAG021758	710:109	NONHSAG013655	508:525	NONHSAG053219	542:681
NONHSAG001247	989:887	NONHSAG031485	226:165	NONHSAG021758	243:553	NONHSAG013655	1011:623	NONHSAG053219	745:21
NONHSAG001264	936:549	NONHSAG031485	955:1157	NONHSAG021758	441:665	NONHSAG013655	941:259	NONHSAG053219	690:993
NONHSAG001264	818:925	NONHSAG031485	105:601	NONHSAG021801	71:101	NONHSAG013655	205:1143	NONHSAG053240	958:303
NONHSAG001264	759:487	NONHSAG031485	843:1063	NONHSAG021801	470:737	NONHSAG013655	486:361	NONHSAG053240	256:725
NONHSAG001264	301:669	NONHSAG031485	1040:987	NONHSAG021801	461:415	NONHSAG013655	540:633	NONHSAG053240	116:837
NONHSAG001264	966:487	NONHSAG031485	586:341	NONHSAG021801	239:691	NONHSAG013655	962:807	NONHSAG053240	142:325
NONHSAG001264	709:967	NONHSAG031485	50:399	NONHSAG021801	893:1129	NONHSAG013655	928:789	NONHSAG053240	485:337
NONHSAG001264	73:207	NONHSAG031485	121:355	NONHSAG021801	141:679	NONHSAG013655	60:263	NONHSAG053240	804:429
NONHSAG001264	534:573	NONHSAG031485	971:461	NONHSAG021801	568:809	NONHSAG013658	685:11	NONHSAG053240	209:929
NONHSAG001264	1129:1087	NONHSAG031489	757:189	NONHSAG021801	909:1105	NONHSAG013658	1068:1013	NONHSAG053240	479:477
NONHSAG001264	463:1121	NONHSAG031489	256:869	NONHSAG021801	247:933	NONHSAG013658	424:297	NONHSAG053240	337:119
NONHSAG001264	183:509	NONHSAG031489	1150:767	NONHSAG021801	416:897	NONHSAG013658	1060:347	NONHSAG053240	1001:929
NONHSAG001304	370:719	NONHSAG031489	630:239	NONHSAG021801	339:923	NONHSAG013658	411:691	NONHSAG053240	588:623
NONHSAG001304	359:669	NONHSAG031489	202:23	NONHSAG021825	29:785	NONHSAG013658	830:269	NONHSAG053246	803:333
NONHSAG001304	806:241	NONHSAG031489	78:489	NONHSAG021825	646:687	NONHSAG013658	996:279	NONHSAG053246	801:1033
NONHSAG001304	943:1149	NONHSAG031489	672:397	NONHSAG021825	307:993	NONHSAG013658	1090:635	NONHSAG053246	5:673
NONHSAG001304	473:37	NONHSAG031489	271:395	NONHSAG021825	166:721	NONHSAG013658	986:65	NONHSAG053246	357:447
NONHSAG001304	559:525	NONHSAG031489	131:537	NONHSAG021825	52:785	NONHSAG013688	992:949	NONHSAG053246	573:605
NONHSAG001304	992:1063	NONHSAG031489	197:347	NONHSAG021825	388:681	NONHSAG013688	686:589	NONHSAG053246	313:1157
NONHSAG001304	603:1067	NONHSAG031516	825:1077	NONHSAG021825	470:147	NONHSAG013688	534:187	NONHSAG053246	833:251

NONHSAG001304	333:205	NONHSAG031516	533:967	NONHSAG021825	412:261	NONHSAG013688	612:611	NONHSAG053246	662:263
NONHSAG001304	57:1133	NONHSAG031516	804:51	NONHSAG021825	980:589	NONHSAG013688	90:801	NONHSAG053246	31:1003
NONHSAG001323	770:319	NONHSAG031516	866:143	NONHSAG021825	1009:1073	NONHSAG013701	8:67	NONHSAG053246	1035:333
NONHSAG001323	871:435	NONHSAG031516	3:773	NONHSAG021825	455:845	NONHSAG013701	15:969	NONHSAG053246	1073:631
NONHSAG001323	327:509	NONHSAG031516	375:755	NONHSAG021825	441:749	NONHSAG013701	719:877	NONHSAG053259	279:1151
NONHSAG001323	65:427	NONHSAG031516	12:865	NONHSAG021825	361:741	NONHSAG013701	756:691	NONHSAG053259	664:439
NONHSAG001323	328:421	NONHSAG031516	978:345	NONHSAG021825	569:255	NONHSAG013701	1039:705	NONHSAG053259	810:225
NONHSAG001323	259:105	NONHSAG031516	79:1019	NONHSAG021825	853:1153	NONHSAG013701	1149:543	NONHSAG053259	722:905
NONHSAG001323	313:777	NONHSAG031516	119:53	NONHSAG021825	435:619	NONHSAG013701	736:51	NONHSAG053259	560:17
NONHSAG001323	599:807	NONHSAG031516	490:145	NONHSAG021825	466:1129	NONHSAG013701	748:1119	NONHSAG053259	55:1059
NONHSAG001323	1084:11	NONHSAG031596	209:1007	NONHSAG021825	581:857	NONHSAG013701	576:173	NONHSAG053259	1044:491
NONHSAG001323	385:1099	NONHSAG031596	590:549	NONHSAG021825	787:27	NONHSAG013735	172:509	NONHSAG053259	418:73
NONHSAG001323	358:153	NONHSAG031596	427:777	NONHSAG021825	1141:1111	NONHSAG013735	951:11	NONHSAG053259	14:191
NONHSAG001341	256:115	NONHSAG031596	758:799	NONHSAG021825	834:1039	NONHSAG013735	1100:957	NONHSAG053259	618:687
NONHSAG001341	882:131	NONHSAG031596	322:329	NONHSAG021851	281:677	NONHSAG013735	819:245	NONHSAG053273	986:241
NONHSAG001341	309:321	NONHSAG031596	528:963	NONHSAG021851	968:83	NONHSAG013735	294:871	NONHSAG053273	1037:323
NONHSAG001341	62:939	NONHSAG031596	334:1103	NONHSAG021851	202:1151	NONHSAG013735	591:975	NONHSAG053273	505:315
NONHSAG001341	980:1009	NONHSAG031596	511:167	NONHSAG021851	707:1031	NONHSAG013735	1114:989	NONHSAG053273	939:933
NONHSAG001341	613:415	NONHSAG031596	420:1	NONHSAG021851	985:57	NONHSAG013735	188:1085	NONHSAG053273	617:809
NONHSAG001341	355:39	NONHSAG031596	426:735	NONHSAG021851	868:961	NONHSAG013735	347:961	NONHSAG053273	228:1075
NONHSAG001341	101:547	NONHSAG031596	597:439	NONHSAG021851	176:649	NONHSAG013735	317:785	NONHSAG053273	517:269
NONHSAG001341	374:147	NONHSAG031603	2:715	NONHSAG021851	737:47	NONHSAG013735	699:89	NONHSAG053273	1068:997
NONHSAG001341	791:601	NONHSAG031603	879:963	NONHSAG021851	959:9	NONHSAG013735	598:399	NONHSAG053273	890:297
NONHSAG001391	336:1149	NONHSAG031603	343:733	NONHSAG021881	570:255	NONHSAG013735	154:455	NONHSAG053273	1037:581
NONHSAG001391	269:873	NONHSAG031603	105:967	NONHSAG021881	702:1003	NONHSAG013735	513:337	NONHSAG053273	610:961
NONHSAG001391	811:191	NONHSAG031603	202:747	NONHSAG021881	687:699	NONHSAG013735	1146:727	NONHSAG053274	63:239
NONHSAG001391	507:67	NONHSAG031603	1038:1105	NONHSAG021881	624:1133	NONHSAG013735	470:197	NONHSAG053274	747:845
NONHSAG001391	430:193	NONHSAG031603	1153:887	NONHSAG021881	742:1073	NONHSAG013735	1074:1071	NONHSAG053274	800:473
NONHSAG001391	603:555	NONHSAG031603	209:265	NONHSAG021881	1026:395	NONHSAG013735	822:575	NONHSAG053274	770:193
NONHSAG001391	332:865	NONHSAG031603	511:487	NONHSAG021881	438:837	NONHSAG013735	389:535	NONHSAG053274	708:1045
NONHSAG001391	829:973	NONHSAG031603	903:547	NONHSAG021881	467:733	NONHSAG013735	1069:683	NONHSAG053274	758:743
NONHSAG001391	968:883	NONHSAG031603	67:875	NONHSAG021881	610:1153	NONHSAG013735	360:871	NONHSAG053274	666:435
NONHSAG001391	356:945	NONHSAG031603	440:297	NONHSAG021881	439:479	NONHSAG013735	1099:145	NONHSAG053274	727:219
NONHSAG001391	164:23	NONHSAG031603	385:761	NONHSAG021904	568:753	NONHSAG013737	940:715	NONHSAG053274	1131:405
NONHSAG001489	767:655	NONHSAG031603	1058:375	NONHSAG021904	182:113	NONHSAG013737	1106:65	NONHSAG053274	1151:9
NONHSAG001489	605:463	NONHSAG031603	349:981	NONHSAG021904	264:727	NONHSAG013737	589:755	NONHSAG053293	518:971
NONHSAG001489	236:79	NONHSAG031603	829:477	NONHSAG021904	1020:267	NONHSAG013737	977:1005	NONHSAG053293	494:709
NONHSAG001489	672:39	NONHSAG031603	631:281	NONHSAG021904	997:41	NONHSAG013737	533:935	NONHSAG053293	1131:285
NONHSAG001489	827:219	NONHSAG031603	814:151	NONHSAG021904	487:525	NONHSAG013737	20:1067	NONHSAG053293	309:625
NONHSAG001489	751:417	NONHSAG031603	482:281	NONHSAG021904	654:769	NONHSAG013737	740:507	NONHSAG053293	524:835
NONHSAG001489	639:849	NONHSAG031603	1008:113	NONHSAG021904	269:831	NONHSAG013737	146:33	NONHSAG053293	1120:543
NONHSAG001489	61:1055	NONHSAG031603	972:911	NONHSAG021904	673:251	NONHSAG013737	903:35	NONHSAG053293	547:373
NONHSAG001510	907:245	NONHSAG031603	835:627	NONHSAG021904	186:165	NONHSAG013737	677:367	NONHSAG053293	893:95
NONHSAG001510	673:581	NONHSAG031617	1142:771	NONHSAG021904	634:459	NONHSAG013737	109:317	NONHSAG053293	980:771
NONHSAG001510	1137:999	NONHSAG031617	570:645	NONHSAG021904	216:111	NONHSAG013751	1023:645	NONHSAG053296	1161:9
NONHSAG001510	472:35	NONHSAG031617	87:149	NONHSAG021904	137:391	NONHSAG013751	1054:1155	NONHSAG053296	964:61
NONHSAG001510	621:875	NONHSAG031617	401:471	NONHSAG021904	414:921	NONHSAG013751	659:839	NONHSAG053296	709:643
NONHSAG001510	698:945	NONHSAG031617	731:725	NONHSAG021904	1020:171	NONHSAG013751	501:107	NONHSAG053296	68:575
NONHSAG001510	83:243	NONHSAG031617	905:295	NONHSAG021904	1000:259	NONHSAG013751	457:371	NONHSAG053296	324:603
NONHSAG001510	767:429	NONHSAG031617	994:585	NONHSAG021904	493:371	NONHSAG013751	212:825	NONHSAG053296	878:459
NONHSAG001530	224:1019	NONHSAG031617	299:753	NONHSAG021904	612:1135	NONHSAG013751	1038:145	NONHSAG053296	1042:975
NONHSAG001530	25:677	NONHSAG031617	553:269	NONHSAG021905	184:1017	NONHSAG013751	611:569	NONHSAG053296	504:1063
NONHSAG001530	948:325	NONHSAG031617	653:97	NONHSAG021905	775:1043	NONHSAG013751	395:219	NONHSAG053296	877:271
NONHSAG001530	645:1121	NONHSAG031617	27:385	NONHSAG021905	590:451	NONHSAG013751	312:465	NONHSAG053296	392:1067
NONHSAG001530	342:297	NONHSAG031617	443:713	NONHSAG021905	973:701	NONHSAG013766	679:145	NONHSAG053296	454:803
NONHSAG001530	1098:455	NONHSAG031617	616:845	NONHSAG021905	331:1019	NONHSAG013766	163:187	NONHSAG053296	116:1107
NONHSAG001530	1071:517	NONHSAG031617	960:1081	NONHSAG021905	512:65	NONHSAG013766	926:577	NONHSAG053296	2:585
NONHSAG001530	152:99	NONHSAG031617	485:157	NONHSAG021905	584:371	NONHSAG013766	447:1159	NONHSAG053296	115:441
NONHSAG001530	972:855	NONHSAG031617	1137:89	NONHSAG021905	311:261	NONHSAG013766	391:737	NONHSAG053296	795:381
NONHSAG001530	322:1113	NONHSAG031617	1028:169	NONHSAG021905	1143:595	NONHSAG013766	844:1093	NONHSAG053296	861:907
NONHSAG001569	141:1069	NONHSAG031617	483:773	NONHSAG021905	757:1015	NONHSAG013766	1062:301	NONHSAG053296	714:291
NONHSAG001569	154:1071	NONHSAG031617	455:817	NONHSAG021905	247:573	NONHSAG013766	830:217	NONHSAG053296	62:547
NONHSAG001569	706:175	NONHSAG031617	838:365	NONHSAG021921	106:357	NONHSAG013766	856:461	NONHSAG053296	673:21
NONHSAG001569	208:859	NONHSAG031617	168:319	NONHSAG021921	913:7	NONHSAG013766	152:31	NONHSAG053296	556:267
NONHSAG001569	720:579	NONHSAG031617	733:381	NONHSAG021921	453:433	NONHSAG013766	312:171	NONHSAG053296	769:1075
NONHSAG001569	237:607	NONHSAG031626	682:471	NONHSAG021921	805:1101	NONHSAG013766	586:239	NONHSAG053296	740:1145
NONHSAG001569	425:73	NONHSAG031626	391:299	NONHSAG021921	776:1073	NONHSAG013766	55:303	NONHSAG053299	713:733
NONHSAG001569	609:515	NONHSAG031626	687:33	NONHSAG021921	601:761	NONHSAG013766	650:713	NONHSAG053299	40:457
NONHSAG001569	1079:331	NONHSAG031626	479:607	NONHSAG021921	214:17	NONHSAG013766	671:349	NONHSAG053299	340:11
NONHSAG001569	738:933	NONHSAG031665	408:235	NONHSAG021921	410:837	NONHSAG013766	161:899	NONHSAG053299	591:209
NONHSAG001569	417:337	NONHSAG031665	21:273	NONHSAG021921	974:1079	NONHSAG013766	65:125	NONHSAG053299	919:173
NONHSAG001574	181:87	NONHSAG031665	769:609	NONHSAG021921	861:975	NONHSAG013766	169:171	NONHSAG053299	313:519
NONHSAG001574	290:101	NONHSAG031665	1101:557	NONHSAG021933	274:1105	NONHSAG013766	968:23	NONHSAG053299	460:1023
NONHSAG001574	477:1123	NONHSAG031665	123:939	NONHSAG021933	10:281	NONHSAG013766	750:143	NONHSAG053299	414:615
NONHSAG001574	231:1089	NONHSAG031665	120:837	NONHSAG021933	622:679	NONHSAG013766	871:707	NONHSAG053299	334:351
NONHSAG001574	584:1129	NONHSAG031665	1069:25	NONHSAG021933	344:235	NONHSAG013766	884:609	NONHSAG053299	1068:213
NONHSAG001574	367:641	NONHSAG031665	138:45	NONHSAG021933	728:613	NONHSAG013770	944:1	NONHSAG053306	281:1093
NONHSAG001574	509:675	NONHSAG031665	532:221	NONHSAG021933	685:347	NONHSAG013770	1130:589	NONHSAG053306	341:625
NONHSAG001574	869:395	NONHSAG031665	381:1123	NONHSAG021933	391:193	NONHSAG013770	1146:23	NONHSAG053306	790:847
NONHSAG001574	331:425	NONHSAG031665	547:5	NONHSAG021933	839:13	NONHSAG013770	588:373	NONHSAG053306	861:509
NONHSAG001574	961:1065	NONHSAG031665	249:1097	NONHSAG021933	762:739	NONHSAG013770	867:899	NONHSAG053306	438:507
NONHSAG001576	322:595	NONHSAG031665	859:635	NONHSAG021933	171:749	NONHSAG013770	47:243	NONHSAG053306	4:745
NONHSAG001576	966:761	NONHSAG031665	190:1151	NONHSAG022026	111:309	NONHSAG013770	1161:679	NONHSAG053306	811:901
NONHSAG001576	1108:763	NONHSAG031665	328:627	NONHSAG022026	108:297	NONHSAG013770	188:377	NONHSAG053306	280:503
NONHSAG001576	448:133	NONHSAG031665	602:413	NONHSAG022026	883:87	NONHSAG013770	986:751	NONHSAG053306	969:479
NONHSAG001576	1042:389	NONHSAG031665	544:311	NONHSAG022026	339:963	NONHSAG013770	3:287	NONHSAG053306	900:153
NONHSAG001576	151:561	NONHSAG031665	17:1015	NONHSAG022026	359:1057	NONHSAG013770	983:1015	NONHSAG053306	395:591
NONHSAG001576	1150:425	NONHSAG031665	680:999	NONHSAG022026	858:7	NONHSAG013770	80:121	NONHSAG053322	951:323

NONHSAG001576	212:431	NONHSAG031701	510:815	NONHSAG022026	339:767	NONHSAG013770	199:443	NONHSAG053322	485:311
NONHSAG001576	899:689	NONHSAG031701	589:403	NONHSAG022026	728:141	NONHSAG013770	384:1061	NONHSAG053322	853:267
NONHSAG001576	872:1009	NONHSAG031701	1042:335	NONHSAG022026	486:487	NONHSAG013770	1045:897	NONHSAG053322	685:383
NONHSAG001576	640:511	NONHSAG031701	824:547	NONHSAG022026	698:535	NONHSAG013770	760:683	NONHSAG053322	358:423
NONHSAG001587	232:707	NONHSAG031701	284:1159	NONHSAG022026	653:605	NONHSAG013770	152:837	NONHSAG053322	104:313
NONHSAG001587	641:997	NONHSAG031701	313:647	NONHSAG022055	541:105	NONHSAG013770	29:1115	NONHSAG053322	158:481
NONHSAG001587	146:677	NONHSAG031701	715:573	NONHSAG022055	1104:139	NONHSAG013770	826:345	NONHSAG053322	218:1031
NONHSAG001587	440:1133	NONHSAG031701	116:177	NONHSAG022055	602:559	NONHSAG013770	1085:311	NONHSAG053329	957:1143
NONHSAG001587	811:563	NONHSAG031701	679:883	NONHSAG022055	394:101	NONHSAG013770	185:699	NONHSAG053329	982:591
NONHSAG001587	883:649	NONHSAG031701	147:853	NONHSAG022055	549:841	NONHSAG013770	903:151	NONHSAG053329	923:125
NONHSAG001587	178:529	NONHSAG031701	653:191	NONHSAG022055	871:529	NONHSAG013789	749:1041	NONHSAG053329	337:287
NONHSAG001587	995:353	NONHSAG031701	381:145	NONHSAG022055	1120:135	NONHSAG013789	1001:1083	NONHSAG053329	771:375
NONHSAG001587	968:735	NONHSAG031701	1153:227	NONHSAG022055	377:285	NONHSAG013789	441:977	NONHSAG053329	27:859
NONHSAG001587	78:935	NONHSAG031701	495:71	NONHSAG022055	1048:1019	NONHSAG013789	862:263	NONHSAG053329	288:795
NONHSAG001587	37:1043	NONHSAG031701	623:779	NONHSAG022055	1087:7	NONHSAG013789	724:103	NONHSAG053329	265:137
NONHSAG001597	779:257	NONHSAG031701	388:667	NONHSAG022055	224:919	NONHSAG013789	620:425	NONHSAG053329	277:255
NONHSAG001597	521:967	NONHSAG031701	1128:805	NONHSAG022055	672:969	NONHSAG013789	1043:491	NONHSAG053329	274:605
NONHSAG001597	333:787	NONHSAG031701	475:549	NONHSAG022055	917:381	NONHSAG013789	832:113	NONHSAG053329	1108:767
NONHSAG001597	1034:749	NONHSAG031748	545:485	NONHSAG022055	497:625	NONHSAG013789	360:771	NONHSAG053359	462:575
NONHSAG001597	794:269	NONHSAG031748	573:407	NONHSAG022055	750:527	NONHSAG013789	570:721	NONHSAG053359	439:265
NONHSAG001597	508:329	NONHSAG031748	707:265	NONHSAG022055	166:633	NONHSAG013789	638:497	NONHSAG053359	326:275
NONHSAG001597	650:257	NONHSAG031748	353:135	NONHSAG022055	343:577	NONHSAG013789	1085:545	NONHSAG053359	948:707
NONHSAG001597	26:225	NONHSAG031748	151:867	NONHSAG022055	905:239	NONHSAG013789	301:1065	NONHSAG053359	365:675
NONHSAG001597	1111:711	NONHSAG031748	521:285	NONHSAG022065	374:805	NONHSAG013789	282:143	NONHSAG053359	123:429
NONHSAG001597	964:181	NONHSAG031748	137:367	NONHSAG022065	696:1137	NONHSAG013789	335:267	NONHSAG053359	1120:365
NONHSAG001601	810:337	NONHSAG031748	421:923	NONHSAG022065	858:421	NONHSAG013789	1148:1119	NONHSAG053359	796:745
NONHSAG001601	323:715	NONHSAG031748	1087:1147	NONHSAG022065	1121:3	NONHSAG013789	624:369	NONHSAG053359	802:1079
NONHSAG001601	714:931	NONHSAG031748	73:81	NONHSAG022065	997:207	NONHSAG013789	946:243	NONHSAG053359	915:427
NONHSAG001601	494:379	NONHSAG031748	74:167	NONHSAG022065	358:133	NONHSAG013789	629:1115	NONHSAG053359	1073:107
NONHSAG001601	816:1105	NONHSAG031783	901:689	NONHSAG022065	1129:793	NONHSAG013789	427:577	NONHSAG053361	968:785
NONHSAG001601	697:545	NONHSAG031783	553:647	NONHSAG022065	1040:669	NONHSAG013789	1070:191	NONHSAG053361	386:429
NONHSAG001601	835:1003	NONHSAG031783	989:219	NONHSAG022065	485:697	NONHSAG013789	533:315	NONHSAG053361	1157:331
NONHSAG001601	341:293	NONHSAG031783	527:1113	NONHSAG022065	779:49	NONHSAG013796	32:917	NONHSAG053361	877:859
NONHSAG001622	336:883	NONHSAG031783	719:477	NONHSAG022065	229:225	NONHSAG013796	557:1151	NONHSAG053361	503:539
NONHSAG001622	310:679	NONHSAG031783	434:215	NONHSAG022068	630:1085	NONHSAG013796	380:613	NONHSAG053361	360:993
NONHSAG001622	261:1069	NONHSAG031783	38:321	NONHSAG022068	1132:245	NONHSAG013796	696:867	NONHSAG053361	4:693
NONHSAG001622	1144:895	NONHSAG031783	1081:787	NONHSAG022068	469:591	NONHSAG013796	664:535	NONHSAG053361	184:597
NONHSAG001622	264:1141	NONHSAG031783	91:385	NONHSAG022068	24:495	NONHSAG013796	383:1053	NONHSAG053361	715:733
NONHSAG001622	489:31	NONHSAG031783	261:903	NONHSAG022068	813:863	NONHSAG013796	511:647	NONHSAG053361	272:331
NONHSAG001622	993:1	NONHSAG031783	560:893	NONHSAG022068	158:141	NONHSAG013796	857:265	NONHSAG053376	1029:679
NONHSAG001622	1073:421	NONHSAG031784	169:971	NONHSAG022068	648:545	NONHSAG013836	748:1033	NONHSAG053376	277:429
NONHSAG001622	891:23	NONHSAG031784	565:737	NONHSAG022068	409:861	NONHSAG013836	299:1113	NONHSAG053376	519:1157
NONHSAG001622	237:861	NONHSAG031784	282:349	NONHSAG022068	1006:127	NONHSAG013836	1086:35	NONHSAG053376	297:769
NONHSAG001622	552:307	NONHSAG031784	1105:801	NONHSAG022073	392:825	NONHSAG013836	942:245	NONHSAG053376	849:491
NONHSAG001627	1082:463	NONHSAG031784	913:81	NONHSAG022073	136:955	NONHSAG013836	1097:1125	NONHSAG053376	1006:1131
NONHSAG001627	1084:243	NONHSAG031784	302:279	NONHSAG022073	910:157	NONHSAG013836	569:1141	NONHSAG053376	85:139
NONHSAG001627	351:727	NONHSAG031784	867:11	NONHSAG022073	28:185	NONHSAG013836	611:247	NONHSAG053376	167:341
NONHSAG001627	877:441	NONHSAG031784	56:899	NONHSAG022073	83:973	NONHSAG013836	963:567	NONHSAG053376	339:1115
NONHSAG001627	1133:1041	NONHSAG031784	76:113	NONHSAG022073	76:855	NONHSAG013836	115:1001	NONHSAG053376	593:401
NONHSAG001627	562:851	NONHSAG031784	373:1117	NONHSAG022073	476:1145	NONHSAG013836	736:875	NONHSAG053376	183:693
NONHSAG001627	71:743	NONHSAG031784	145:103	NONHSAG022073	1015:585	NONHSAG013836	420:1115	NONHSAG053377	710:1019
NONHSAG001627	459:395	NONHSAG031801	515:865	NONHSAG022073	382:839	NONHSAG013843	951:347	NONHSAG053377	916:979
NONHSAG001627	962:1091	NONHSAG031801	589:1117	NONHSAG022073	154:761	NONHSAG013843	1028:585	NONHSAG053377	190:257
NONHSAG001627	550:1071	NONHSAG031801	297:133	NONHSAG022073	795:479	NONHSAG013843	331:1047	NONHSAG053377	216:645
NONHSAG001627	325:975	NONHSAG031801	344:17	NONHSAG022088	690:483	NONHSAG013843	286:931	NONHSAG053377	824:893
NONHSAG001640	70:975	NONHSAG031801	1080:125	NONHSAG022088	809:165	NONHSAG013843	802:613	NONHSAG053377	768:291
NONHSAG001640	602:1071	NONHSAG031801	1140:613	NONHSAG022088	1019:633	NONHSAG013843	798:557	NONHSAG053377	378:269
NONHSAG001640	302:955	NONHSAG031801	217:577	NONHSAG022088	38:409	NONHSAG013843	155:725	NONHSAG053377	1025:269
NONHSAG001640	572:197	NONHSAG031801	475:63	NONHSAG022088	249:359	NONHSAG013843	388:7	NONHSAG053377	1064:1009
NONHSAG001640	544:83	NONHSAG031801	205:343	NONHSAG022088	624:973	NONHSAG013843	852:129	NONHSAG053377	675:237
NONHSAG001640	746:773	NONHSAG031801	51:1137	NONHSAG022089	830:41	NONHSAG013843	972:1107	NONHSAG053377	466:301
NONHSAG001640	119:391	NONHSAG031801	949:1013	NONHSAG022089	936:531	NONHSAG013843	560:151	NONHSAG053377	979:1071
NONHSAG001640	908:61	NONHSAG031805	984:129	NONHSAG022089	967:115	NONHSAG013843	1015:135	NONHSAG053377	674:655
NONHSAG001640	126:863	NONHSAG031805	654:717	NONHSAG022089	1100:557	NONHSAG013843	206:1047	NONHSAG053377	115:827
NONHSAG001640	324:639	NONHSAG031805	628:775	NONHSAG022089	763:445	NONHSAG013843	1036:673	NONHSAG053377	1001:767
NONHSAG001640	85:17	NONHSAG031805	288:819	NONHSAG022089	140:887	NONHSAG013843	546:441	NONHSAG053377	982:99
NONHSAG001660	389:439	NONHSAG031805	797:313	NONHSAG022089	40:207	NONHSAG013843	246:199	NONHSAG053377	1085:101
NONHSAG001660	723:715	NONHSAG031805	61:333	NONHSAG022089	806:997	NONHSAG013843	1069:173	NONHSAG053377	62:585
NONHSAG001660	46:709	NONHSAG031805	537:189	NONHSAG022089	582:99	NONHSAG013843	703:1059	NONHSAG053377	664:361
NONHSAG001660	955:841	NONHSAG031805	829:289	NONHSAG022092	737:1113	NONHSAG013843	787:531	NONHSAG053377	819:869
NONHSAG001660	1065:843	NONHSAG031805	1117:795	NONHSAG022092	782:1067	NONHSAG013843	822:1135	NONHSAG053377	565:15
NONHSAG001660	622:257	NONHSAG031805	451:959	NONHSAG022092	511:687	NONHSAG013843	920:1003	NONHSAG053377	688:353
NONHSAG001660	306:921	NONHSAG031805	946:175	NONHSAG022092	441:37	NONHSAG013856	192:739	NONHSAG053380	529:677
NONHSAG001660	202:533	NONHSAG031818	116:451	NONHSAG022092	694:213	NONHSAG013856	566:293	NONHSAG053380	288:37
NONHSAG001683	213:593	NONHSAG031818	1059:179	NONHSAG022092	462:681	NONHSAG013856	479:1095	NONHSAG053380	954:807
NONHSAG001683	1160:645	NONHSAG031818	613:795	NONHSAG022092	555:907	NONHSAG013856	951:353	NONHSAG053380	615:131
NONHSAG001683	428:1063	NONHSAG031818	623:679	NONHSAG022092	431:973	NONHSAG013856	13:159	NONHSAG053380	1057:655
NONHSAG001683	475:185	NONHSAG031818	785:973	NONHSAG022092	803:29	NONHSAG013856	1018:557	NONHSAG053380	726:177
NONHSAG001683	933:167	NONHSAG031824	809:275	NONHSAG022092	193:483	NONHSAG013856	719:739	NONHSAG053380	1061:15
NONHSAG001683	407:349	NONHSAG031824	361:217	NONHSAG022092	497:781	NONHSAG013856	1154:1025	NONHSAG053380	535:169
NONHSAG001683	512:909	NONHSAG031824	208:159	NONHSAG022177	983:529	NONHSAG013856	408:225	NONHSAG053380	588:221
NONHSAG001683	1120:765	NONHSAG031824	803:625	NONHSAG022177	995:725	NONHSAG013856	513:899	NONHSAG053380	293:661
NONHSAG001683	988:597	NONHSAG031824	805:559	NONHSAG022177	160:697	NONHSAG013867	35:613	NONHSAG053380	730:1109
NONHSAG001683	329:931	NONHSAG031824	466:741	NONHSAG022177	1090:307	NONHSAG013867	312:219	NONHSAG053392	261:261
NONHSAG001683	94:669	NONHSAG031824	686:679	NONHSAG022177	406:227	NONHSAG013867	152:225	NONHSAG053392	406:127
NONHSAG001696	1078:973	NONHSAG031824	309:277	NONHSAG022177	968:219	NONHSAG013867	246:919	NONHSAG053392	818:353
NONHSAG001696	456:415	NONHSAG031824	944:483	NONHSAG022177	368:803	NONHSAG013867	311:15	NONHSAG053392	1109:617
NONHSAG001696	1017:781	NONHSAG031824	610:87	NONHSAG022177	387:113	NONHSAG013867	38:291	NONHSAG053392	416:557

NONHSAG001696	619:767	NONHSAG031831	542:1119	NONHSAG022177	363:743	NONHSAG013867	1147:559	NONHSAG053392	886:491
NONHSAG001696	95:627	NONHSAG031831	693:563	NONHSAG022177	150:929	NONHSAG013867	266:475	NONHSAG053392	122:935
NONHSAG001696	948:419	NONHSAG031831	468:931	NONHSAG022177	81:613	NONHSAG013867	274:725	NONHSAG053392	142:503
NONHSAG001722	519:1021	NONHSAG031831	439:733	NONHSAG022177	281:817	NONHSAG013867	795:99	NONHSAG053406	508:579
NONHSAG001722	78:163	NONHSAG031831	740:1071	NONHSAG022177	1110:547	NONHSAG013871	1153:261	NONHSAG053406	1078:817
NONHSAG001722	11:1029	NONHSAG031831	300:553	NONHSAG022177	502:773	NONHSAG013871	916:959	NONHSAG053406	1068:63
NONHSAG001722	37:457	NONHSAG031831	1059:1053	NONHSAG022177	818:479	NONHSAG013871	422:607	NONHSAG053406	888:263
NONHSAG001722	621:1139	NONHSAG031831	47:159	NONHSAG022177	723:655	NONHSAG013871	73:863	NONHSAG053407	420:887
NONHSAG001722	422:1159	NONHSAG031831	299:227	NONHSAG022177	275:423	NONHSAG013871	946:93	NONHSAG053407	905:819
NONHSAG001722	758:259	NONHSAG031831	893:785	NONHSAG022214	778:383	NONHSAG013871	836:121	NONHSAG053407	316:121
NONHSAG001722	668:1	NONHSAG031831	280:177	NONHSAG022214	906:277	NONHSAG013871	335:787	NONHSAG053407	789:295
NONHSAG001722	522:475	NONHSAG031831	943:1009	NONHSAG022214	903:729	NONHSAG013871	675:351	NONHSAG053407	72:991
NONHSAG001722	213:53	NONHSAG031831	388:897	NONHSAG022214	730:1137	NONHSAG013871	363:419	NONHSAG053407	1084:435
NONHSAG001723	1100:95	NONHSAG031831	104:1145	NONHSAG022214	135:571	NONHSAG013871	630:1161	NONHSAG053407	573:219
NONHSAG001723	702:503	NONHSAG031831	839:421	NONHSAG022214	826:815	NONHSAG013871	875:597	NONHSAG053407	818:549
NONHSAG001723	754:519	NONHSAG031831	766:1119	NONHSAG022214	1020:575	NONHSAG013889	396:119	NONHSAG053407	747:251
NONHSAG001723	114:327	NONHSAG031831	232:1015	NONHSAG022214	887:105	NONHSAG013889	459:271	NONHSAG053417	72:1099
NONHSAG001723	93:243	NONHSAG031831	231:55	NONHSAG022214	591:115	NONHSAG013889	831:1003	NONHSAG053417	1001:187
NONHSAG001723	1002:75	NONHSAG031831	124:25	NONHSAG022214	775:135	NONHSAG013889	386:979	NONHSAG053417	1118:783
NONHSAG001723	1010:975	NONHSAG031831	999:1	NONHSAG022214	437:625	NONHSAG013889	510:243	NONHSAG053417	460:91
NONHSAG001723	1044:677	NONHSAG031831	1092:403	NONHSAG022214	226:801	NONHSAG013889	605:953	NONHSAG053417	634:483
NONHSAG001723	1160:1071	NONHSAG031831	939:1049	NONHSAG022214	892:155	NONHSAG013889	51:921	NONHSAG053417	874:27
NONHSAG001768	1087:1127	NONHSAG031831	541:1055	NONHSAG022214	151:917	NONHSAG013889	274:929	NONHSAG053417	537:61
NONHSAG001768	578:647	NONHSAG031831	996:1075	NONHSAG022214	657:973	NONHSAG013889	676:1095	NONHSAG053417	708:453
NONHSAG001768	843:509	NONHSAG031831	147:985	NONHSAG022214	578:815	NONHSAG013889	362:589	NONHSAG053417	855:525
NONHSAG001768	544:181	NONHSAG031831	333:443	NONHSAG022214	382:189	NONHSAG013889	400:299	NONHSAG053417	569:143
NONHSAG001768	1059:169	NONHSAG031831	665:1105	NONHSAG022214	141:51	NONHSAG013908	446:733	NONHSAG053417	984:1125
NONHSAG001768	656:451	NONHSAG031831	342:475	NONHSAG022214	1155:283	NONHSAG013908	1117:333	NONHSAG053440	989:701
DEPDC1-AS1	4:167	NONHSAG031831	349:1145	NONHSAG022214	88:299	NONHSAG013908	635:249	NONHSAG053440	864:379
DEPDC1-AS1	418:1029	NONHSAG031831	848:723	NONHSAG022214	537:983	NONHSAG013908	674:1145	NONHSAG053440	215:197
DEPDC1-AS1	651:513	NONHSAG031831	498:217	NONHSAG022214	415:45	NONHSAG013908	98:529	NONHSAG053440	229:329
DEPDC1-AS1	477:537	NONHSAG031845	61:593	NONHSAG022224	787:335	NONHSAG013908	323:363	NONHSAG053440	205:1025
DEPDC1-AS1	876:641	NONHSAG031845	868:905	NONHSAG022224	164:187	NONHSAG013908	668:399	NONHSAG053440	951:1105
DEPDC1-AS1	1033:1059	NONHSAG031845	835:619	NONHSAG022224	1034:957	NONHSAG013908	745:717	NONHSAG053440	277:117
DEPDC1-AS1	1032:961	NONHSAG031845	549:1073	NONHSAG022224	1120:737	NONHSAG013908	894:397	NONHSAG053440	1084:1147
DEPDC1-AS1	1142:237	NONHSAG031845	875:793	NONHSAG022224	935:661	NONHSAG013908	982:695	NONHSAG053440	532:1133
DEPDC1-AS1	540:1033	NONHSAG031845	911:377	NONHSAG022224	863:203	NONHSAG013908	910:687	NONHSAG053440	912:819
DEPDC1-AS1	768:1033	NONHSAG031845	123:615	NONHSAG022224	954:1031	NONHSAG013910	1085:699	NONHSAG053440	980:899
DEPDC1-AS1	610:725	NONHSAG031845	605:141	NONHSAG022224	154:941	NONHSAG013910	268:3	NONHSAG053450	1007:829
NONHSAG001788	809:217	NONHSAG031845	637:757	NONHSAG022224	645:119	NONHSAG013910	285:1013	NONHSAG053450	258:945
NONHSAG001788	1063:1149	NONHSAG031845	172:333	NONHSAG022224	63:641	NONHSAG013910	172:291	NONHSAG053450	536:1153
NONHSAG001788	170:713	NONHSAG031845	906:547	NONHSAG022224	616:655	NONHSAG013910	262:1007	NONHSAG053450	875:357
NONHSAG001788	524:879	NONHSAG031890	749:769	NONHSAG022252	1035:229	NONHSAG013910	403:1145	NONHSAG053450	236:107
NONHSAG001788	597:677	NONHSAG031890	974:109	NONHSAG022252	414:1045	NONHSAG013910	1161:23	NONHSAG053450	607:333
NONHSAG001788	704:817	NONHSAG031890	873:669	NONHSAG022252	179:111	NONHSAG013910	315:689	NONHSAG053450	211:733
NONHSAG001788	531:475	NONHSAG031890	1041:633	NONHSAG022252	697:13	NONHSAG013910	1058:1155	NONHSAG053450	1023:749
NONHSAG001788	1005:1057	NONHSAG031890	716:615	NONHSAG022252	27:215	NONHSAG013910	840:731	NONHSAG053450	209:735
NONHSAG001788	1108:1149	NONHSAG031890	276:637	NONHSAG022252	798:1071	NONHSAG013910	1023:929	NONHSAG053450	621:69
NONHSAG001788	814:621	NONHSAG031890	414:207	NONHSAG022252	106:743	NONHSAG013921	1077:981	NONHSAG053454	207:1083
NONHSAG001788	233:529	NONHSAG031890	582:253	NONHSAG022252	211:273	NONHSAG013921	1002:859	NONHSAG053454	1115:807
NONHSAG001803	1039:701	NONHSAG031890	244:239	NONHSAG022252	951:603	NONHSAG013921	229:1127	NONHSAG053454	443:129
NONHSAG001803	144:401	NONHSAG031890	648:115	NONHSAG022252	298:121	NONHSAG013921	270:549	NONHSAG041789	657:537
NONHSAG001803	456:1017	NONHSAG031890	18:159	NONHSAG022252	1113:297	NONHSAG013921	811:593	NONHSAG041789	174:1021
NONHSAG001803	516:279	NONHSAG031913	752:395	NONHSAG022252	747:357	NONHSAG013921	239:761	NONHSAG041789	663:219
NONHSAG001803	782:751	NONHSAG031913	930:507	NONHSAG022252	552:719	NONHSAG013921	162:17	NONHSAG041804	883:165
NONHSAG001803	493:797	NONHSAG031913	833:1155	NONHSAG022252	855:743	NONHSAG013921	370:653	NONHSAG041804	618:619
NONHSAG001803	362:571	NONHSAG031913	585:431	NONHSAG022252	300:723	NONHSAG013921	369:285	NONHSAG041804	194:717
NONHSAG001803	337:1145	NONHSAG031913	1015:707	NONHSAG022252	219:265	NONHSAG013938	207:101	NONHSAG041804	1020:1091
NONHSAG001803	320:263	NONHSAG031913	56:481	NONHSAG022252	876:159	NONHSAG013938	1093:519	NONHSAG041804	402:1133
NONHSAG001803	489:261	NONHSAG031913	196:1103	NONHSAG022252	520:363	NONHSAG013938	1112:445	NONHSAG041804	457:85
NONHSAG001803	416:287	NONHSAG031913	1063:605	NONHSAG022252	774:723	NONHSAG013938	899:283	NONHSAG041804	758:747
NONHSAG001824	279:945	NONHSAG031913	1157:489	NONHSAG022252	1139:723	NONHSAG013938	135:895	NONHSAG041804	209:333
NONHSAG001824	21:1101	NONHSAG031913	265:207	NONHSAG022256	85:277	NONHSAG013938	34:1037	NONHSAG041804	423:1119
NONHSAG001824	164:677	NONHSAG031913	497:1035	NONHSAG022256	303:129	NONHSAG013938	894:635	NONHSAG041804	322:1117
NONHSAG001824	155:141	NONHSAG031945	829:19	NONHSAG022256	100:279	NONHSAG013938	719:467	NONHSAG041804	821:235
NONHSAG001824	710:243	NONHSAG031945	868:67	NONHSAG022256	795:689	NONHSAG013938	35:195	NONHSAG041804	1077:21
NONHSAG001824	43:1091	NONHSAG031945	67:533	NONHSAG022256	402:889	NONHSAG013938	701:63	NONHSAG041804	1018:1115
NONHSAG001824	535:15	NONHSAG031945	996:531	NONHSAG022256	1111:255	NONHSAG013938	567:835	NONHSAG041804	1013:1079
NONHSAG001824	956:35	NONHSAG031945	5:547	NONHSAG022256	75:275	NONHSAG013940	11:895	NONHSAG041804	62:1073
NONHSAG001824	963:37	NONHSAG031945	911:807	NONHSAG022256	428:773	NONHSAG013940	258:935	NONHSAG041804	60:45
NONHSAG001824	646:1095	NONHSAG031945	1040:605	NONHSAG022256	1011:835	NONHSAG013940	937:661	NONHSAG041804	217:547
NONHSAG001824	351:517	NONHSAG031945	494:865	NONHSAG022261	388:1091	NONHSAG013940	1025:11	NONHSAG041804	470:761
NONHSAG001832	852:931	NONHSAG031945	1153:343	NONHSAG022261	130:175	NONHSAG013940	981:1107	NONHSAG041804	858:837
NONHSAG001832	368:943	NONHSAG031945	836:37	NONHSAG022261	213:1131	NONHSAG013940	100:227	NONHSAG041804	814:9
NONHSAG001832	793:447	NONHSAG031945	48:639	NONHSAG022261	792:381	NONHSAG013940	1055:1001	NONHSAG041804	467:587
NONHSAG001832	188:145	NONHSAG031951	1048:323	NONHSAG022261	57:411	NONHSAG013940	478:55	NONHSAG041810	842:539
NONHSAG001832	218:235	NONHSAG031951	343:423	NONHSAG022261	1057:141	NONHSAG013940	24:907	NONHSAG041810	736:675
NONHSAG001832	452:393	NONHSAG031951	135:389	NONHSAG022261	740:631	NONHSAG013940	796:479	NONHSAG041810	511:433
NONHSAG001832	625:35	NONHSAG031951	345:325	NONHSAG022261	291:729	NONHSAG013940	884:131	NONHSAG041810	233:641
NONHSAG001832	1003:877	NONHSAG031951	781:543	NONHSAG022261	105:1057	NONHSAG013950	774:1029	NONHSAG041810	120:281
NONHSAG001832	167:533	NONHSAG031951	504:323	NONHSAG022261	723:415	NONHSAG013950	728:1011	NONHSAG041810	290:239
NONHSAG001832	731:643	NONHSAG031951	942:85	NONHSAG022261	5:509	NONHSAG013950	160:577	NONHSAG041810	303:163
NONHSAG001832	820:631	NONHSAG031951	726:155	NONHSAG022297	1123:487	NONHSAG013950	656:521	NONHSAG041810	618:403
NONHSAG001832	440:623	NONHSAG031951	697:621	NONHSAG022297	750:255	NONHSAG013950	498:843	NONHSAG041810	479:723
NONHSAG001832	399:117	NONHSAG031952	497:685	NONHSAG022297	934:825	NONHSAG013950	845:105	NONHSAG041810	519:227
NONHSAG001832	1024:489	NONHSAG031952	338:25	NONHSAG022297	171:987	NONHSAG013950	362:611	NONHSAG041810	879:519
NONHSAG001832	996:715	NONHSAG031952	928:543	NONHSAG022297	437:513	NONHSAG013950	885:37	NONHSAG041826	654:613
NONHSAG001832	500:563	NONHSAG031952	531:493	NONHSAG022297	952:451	NONHSAG013950	411:959	NONHSAG041826	356:185

NONHSAG001832	529:213	NONHSAG031952	141:829	NONHSAG022297	833:619	NONHSAG013950	516:863	NONHSAG041826	992:825
NONHSAG001832	265:929	NONHSAG031952	390:791	NONHSAG022297	732:897	NONHSAG013950	243:1067	NONHSAG041826	253:261
NONHSAG001832	1046:739	NONHSAG031952	960:491	NONHSAG022297	1014:1139	NONHSAG013950	171:209	NONHSAG041826	990:65
NONHSAG001832	550:857	NONHSAG031952	133:903	NONHSAG022297	319:767	NONHSAG013950	483:209	NONHSAG041826	1088:1075
NONHSAG001832	360:875	NONHSAG031952	1045:437	NONHSAG022308	925:803	NONHSAG013950	1096:269	NONHSAG041826	191:217
NONHSAG001832	885:291	NONHSAG031952	805:359	NONHSAG022308	440:193	NONHSAG013950	1148:417	NONHSAG041826	424:235
NONHSAG001832	1124:1093	NONHSAG031964	1090:771	NONHSAG022308	787:905	NONHSAG013950	652:289	NONHSAG041886	805:969
NONHSAG001832	242:205	NONHSAG031964	979:689	NONHSAG022308	1015:123	NONHSAG013950	911:213	NONHSAG041886	137:91
NONHSAG001832	99:21	NONHSAG031964	1061:491	NONHSAG022308	327:407	NONHSAG013950	39:685	NONHSAG041886	651:397
NONHSAG001832	119:249	NONHSAG031964	78:125	NONHSAG022308	682:611	NONHSAG013950	217:857	NONHSAG041886	823:395
NONHSAG001832	949:155	NONHSAG031964	48:913	NONHSAG022308	562:949	NONHSAG013950	977:213	NONHSAG041886	959:273
NONHSAG001832	647:1097	NONHSAG031964	681:679	NONHSAG022308	808:575	NONHSAG013950	87:557	NONHSAG041886	698:699
NONHSAG001832	730:669	NONHSAG031964	156:849	NONHSAG022308	745:985	NONHSAG013963	922:133	NONHSAG041903	811:1045
NONHSAG001832	907:21	NONHSAG031964	53:609	NONHSAG022308	1157:579	NONHSAG013963	97:965	NONHSAG041903	48:293
NONHSAG001832	211:575	NONHSAG031964	646:685	NONHSAG022311	691:263	NONHSAG013963	641:107	NONHSAG041903	218:823
NONHSAG001832	80:379	NONHSAG031964	557:447	NONHSAG022311	625:749	NONHSAG013963	440:461	NONHSAG041903	1023:811
NONHSAG001845	424:373	NONHSAG031964	67:485	NONHSAG022311	1033:287	NONHSAG013963	469:1019	NONHSAG041903	374:1093
NONHSAG001845	320:243	NONHSAG032009	884:815	NONHSAG022311	874:627	NONHSAG013963	860:821	NONHSAG041903	359:563
NONHSAG001845	205:859	NONHSAG032009	361:519	NONHSAG022311	102:229	NONHSAG013963	369:433	NONHSAG041903	469:1093
NONHSAG001845	259:647	NONHSAG032009	904:691	NONHSAG022311	155:207	NONHSAG013963	977:821	NONHSAG041903	901:165
NONHSAG001845	430:249	NONHSAG032009	451:821	NONHSAG022311	489:805	NONHSAG013963	669:647	NONHSAG041903	1085:761
NONHSAG001845	118:1147	NONHSAG032009	589:343	NONHSAG022311	1081:321	NONHSAG013963	974:1025	NONHSAG041903	339:113
NONHSAG001845	836:5	NONHSAG032009	87:147	NONHSAG022311	591:1049	NONHSAG013963	328:645	NONHSAG041903	448:213
NONHSAG001845	91:365	NONHSAG032009	243:223	NONHSAG022311	721:807	NONHSAG013971	812:177	NONHSAG041903	456:977
NONHSAG001845	103:755	NONHSAG032009	1031:155	NONHSAG022311	747:475	NONHSAG013971	43:811	NONHSAG041903	1047:1009
NONHSAG001845	113:679	NONHSAG032009	918:999	NONHSAG022328	415:385	NONHSAG013971	965:1157	NONHSAG041903	792:499
NONHSAG001845	1029:33	NONHSAG032009	783:901	NONHSAG022328	660:333	NONHSAG013971	299:187	NONHSAG041903	761:437
NONHSAG001849	1128:247	NONHSAG032009	903:831	NONHSAG022328	898:809	NONHSAG013971	372:15	NONHSAG041903	192:121
NONHSAG001849	138:469	NONHSAG032015	670:527	NONHSAG022328	811:95	NONHSAG013971	361:317	NONHSAG041903	743:483
NONHSAG001849	1080:469	NONHSAG032015	51:941	NONHSAG022328	102:761	NONHSAG013971	894:997	NONHSAG041903	1117:869
NONHSAG001849	215:193	NONHSAG032015	1017:7	NONHSAG022328	152:1143	NONHSAG013971	978:339	NONHSAG041903	370:619
NONHSAG001849	228:511	NONHSAG032015	801:263	NONHSAG022328	974:329	NONHSAG013971	271:277	NONHSAG041903	359:43
NONHSAG001849	1062:465	NONHSAG032015	557:81	NONHSAG022328	32:655	NONHSAG013971	25:495	NONHSAG041903	976:445
NONHSAG001849	934:1147	NONHSAG032015	254:951	NONHSAG022328	363:799	NONHSAG013971	688:415	NONHSAG041903	841:1149
NONHSAG001849	153:465	NONHSAG032015	333:785	NONHSAG022328	583:87	NONHSAG013976	732:1011	NONHSAG041903	558:707
NONHSAG001849	244:179	NONHSAG032015	73:465	NONHSAG022328	149:1105	NONHSAG013976	1000:975	NONHSAG041903	139:569
NONHSAG001855	637:413	NONHSAG032104	752:939	NONHSAG022328	687:977	NONHSAG013976	515:523	NONHSAG041903	572:733
NONHSAG001855	103:113	NONHSAG032104	795:609	NONHSAG022328	278:365	NONHSAG013976	191:1077	NONHSAG041903	185:467
NONHSAG001855	981:445	NONHSAG032104	1130:665	NONHSAG022328	717:491	NONHSAG013976	608:1035	NONHSAG041903	588:533
NONHSAG001855	423:169	NONHSAG032104	983:773	NONHSAG022328	287:971	NONHSAG013976	626:607	NONHSAG041903	398:191
NONHSAG001855	629:1045	NONHSAG032104	486:815	NONHSAG022328	978:815	NONHSAG013976	225:539	NONHSAG041903	26:829
NONHSAG001855	1066:191	NONHSAG032104	68:379	NONHSAG022328	577:535	NONHSAG013976	732:41	NONHSAG041903	996:301
NONHSAG001855	335:437	NONHSAG032104	1085:779	NONHSAG022328	1072:1111	NONHSAG013976	445:569	NONHSAG041903	461:205
NONHSAG001855	1149:369	NONHSAG032104	837:635	NONHSAG022328	331:411	NONHSAG013976	292:5	NONHSAG041903	928:865
NONHSAG001855	735:697	NONHSAG032104	223:929	NONHSAG022328	305:411	NONHSAG013976	744:519	NONHSAG041903	949:447
NONHSAG001898	743:551	NONHSAG032104	24:97	NONHSAG022328	551:441	NONHSAG013982	3:1067	NONHSAG041984	1132:1077
NONHSAG001898	562:1041	NONHSAG032106	1022:293	NONHSAG022328	771:1009	NONHSAG013982	928:871	NONHSAG041984	325:909
NONHSAG001898	24:319	NONHSAG032106	1048:79	NONHSAG022328	835:333	NONHSAG013982	481:639	NONHSAG041984	511:145
NONHSAG001898	1148:287	NONHSAG032106	685:1137	NONHSAG022328	356:95	NONHSAG013982	263:677	NONHSAG041984	293:207
NONHSAG001898	586:601	NONHSAG032106	650:691	NONHSAG022328	702:747	NONHSAG013982	420:1125	NONHSAG041984	4:177
NONHSAG001898	545:449	NONHSAG032106	409:477	NONHSAG022328	1129:495	NONHSAG013982	634:457	NONHSAG041984	844:605
NONHSAG001898	289:353	NONHSAG032106	181:979	NONHSAG022328	175:905	NONHSAG013982	218:269	NONHSAG041984	461:683
NONHSAG001898	772:1113	NONHSAG032106	118:439	NONHSAG022328	280:881	NONHSAG013982	273:417	NONHSAG041984	17:929
NONHSAG001898	585:75	NONHSAG032106	754:127	NONHSAG022328	1083:803	NONHSAG013982	410:361	NONHSAG041987	133:753
NONHSAG001898	999:625	NONHSAG032106	6:987	NONHSAG022337	1092:1017	NONHSAG013982	1124:727	NONHSAG041987	628:151
NONHSAG001898	83:751	NONHSAG032106	129:987	NONHSAG022337	113:995	NONHSAG013992	546:293	NONHSAG041987	513:659
NONHSAG001898	826:217	NONHSAG032106	154:321	NONHSAG022337	662:121	NONHSAG013992	601:945	NONHSAG041987	186:143
NONHSAG001898	415:1065	NONHSAG032135	258:957	NONHSAG022337	722:1079	NONHSAG013992	964:617	NONHSAG041987	274:475
NONHSAG001898	749:283	NONHSAG032135	998:789	NONHSAG022337	476:963	NONHSAG013992	97:191	NONHSAG041987	471:623
NONHSAG001898	1009:173	NONHSAG032135	938:401	NONHSAG022337	1048:49	NONHSAG013992	973:61	NONHSAG041987	751:697
NONHSAG001898	493:559	NONHSAG032135	803:1011	NONHSAG022337	583:137	NONHSAG013992	168:361	NONHSAG041987	704:351
NONHSAG001898	630:1031	NONHSAG032135	942:613	NONHSAG022337	567:839	NONHSAG013992	682:987	NONHSAG041987	973:635
NONHSAG001898	396:267	NONHSAG032135	931:937	NONHSAG022337	824:895	NONHSAG013992	829:603	NONHSAG041987	137:453
NONHSAG001898	1069:901	NONHSAG032135	487:801	NONHSAG022337	1069:267	NONHSAG013992	508:1133	NONHSAG041987	698:11
NONHSAG001898	1157:1025	NONHSAG032135	69:1097	NONHSAG022337	27:301	NONHSAG013992	538:207	NONHSAG041987	200:805
NONHSAG001898	213:827	NONHSAG032135	464:1161	NONHSAG022342	1084:1123	NONHSAG013992	176:117	NONHSAG041987	276:1159
NONHSAG001910	556:971	NONHSAG032143	660:113	NONHSAG022342	105:45	NONHSAG013992	1053:33	NONHSAG041987	674:925
NONHSAG001910	174:641	NONHSAG032143	1013:1045	NONHSAG022342	1083:1161	NONHSAG013992	338:101	NONHSAG041987	681:331
NONHSAG001910	109:301	NONHSAG032143	467:11	NONHSAG022342	289:1113	NONHSAG013992	992:731	NONHSAG041987	1148:461
NONHSAG001910	547:647	NONHSAG032143	71:35	NONHSAG022342	864:661	NONHSAG013992	564:1137	NONHSAG041987	191:729
NONHSAG001910	849:219	NONHSAG032143	358:247	NONHSAG022342	109:615	NONHSAG013992	87:365	NONHSAG041987	612:1149
NONHSAG001910	232:789	NONHSAG032143	984:805	NONHSAG022342	651:625	NONHSAG013992	244:751	NONHSAG041987	447:175
NONHSAG001920	1075:963	NONHSAG032143	787:367	NONHSAG022342	289:69	NONHSAG013992	224:1087	NONHSAG041987	948:557
NONHSAG001920	685:31	NONHSAG032143	1109:665	NONHSAG022342	69:105	NONHSAG013992	382:771	NONHSAG041987	397:375
NONHSAG001920	1049:197	NONHSAG032143	169:1089	NONHSAG022342	684:153	NONHSAG013992	779:549	NONHSAG041987	1086:1003
NONHSAG001920	144:163	NONHSAG032143	369:1133	NONHSAG022342	664:103	NONHSAG013992	1056:723	NONHSAG041994	964:491
NONHSAG001920	193:603	NONHSAG032143	446:39	NONHSAG022353	312:1139	NONHSAG013992	566:1049	NONHSAG041994	155:635
NONHSAG001920	606:321	NONHSAG032143	124:1143	NONHSAG022353	573:333	NONHSAG014007	743:799	NONHSAG041994	805:825
NONHSAG001920	798:37	NONHSAG032143	1126:973	NONHSAG022353	89:203	NONHSAG014007	30:427	NONHSAG041994	203:745
NONHSAG001920	326:317	NONHSAG032143	1077:539	NONHSAG022353	1029:263	NONHSAG014007	460:569	NONHSAG041994	1018:29
NONHSAG001920	829:55	NONHSAG032143	961:199	NONHSAG022353	469:839	NONHSAG014007	290:359	NONHSAG041994	730:1023
NONHSAG001920	1061:871	NONHSAG032143	458:129	NONHSAG022353	1097:295	NONHSAG014007	829:807	NONHSAG041994	364:539
NONHSAG001920	930:249	NONHSAG032143	895:89	NONHSAG022353	96:805	NONHSAG014007	347:473	NONHSAG041994	111:783
NONHSAG001920	703:915	NONHSAG032143	1082:549	NONHSAG022353	146:891	NONHSAG014007	124:1031	NONHSAG041994	695:565
NONHSAG001926	964:905	NONHSAG032143	734:433	NONHSAG022353	49:795	NONHSAG014007	704:807	NONHSAG041994	993:977
NONHSAG001926	830:549	NONHSAG032143	780:203	NONHSAG022353	835:51	NONHSAG014007	51:117	NONHSAG041998	83:945
NONHSAG001926	899:473	NONHSAG032143	915:1145	NONHSAG022353	725:1011	NONHSAG014007	456:373	NONHSAG041998	364:263
NONHSAG001926	994:1149	NONHSAG032143	429:213	NONHSAG022357	632:749	NONHSAG014012	1141:203	NONHSAG041998	442:85

NONHSAG001926	218:403	NONHSAG032143	494:473	NONHSAG022357	473:603	NONHSAG014012	583:983	NONHSAG041998	520:113
NONHSAG001926	429:253	NONHSAG032143	1056:349	NONHSAG022357	719:827	NONHSAG014012	571:987	NONHSAG041998	629:1049
NONHSAG001926	1008:369	NONHSAG032143	350:185	NONHSAG022357	127:527	NONHSAG014012	258:521	NONHSAG041998	546:949
NONHSAG001938	324:215	NONHSAG032143	1065:429	NONHSAG022357	595:1063	NONHSAG014012	630:579	NONHSAG041998	995:277
NONHSAG001938	27:935	NONHSAG032143	717:1023	NONHSAG022357	907:715	NONHSAG014012	687:615	NONHSAG041998	972:971
NONHSAG001938	247:653	NONHSAG032143	1033:805	NONHSAG022357	1029:1109	NONHSAG014012	1045:109	NONHSAG041998	430:683
NONHSAG001938	30:875	NONHSAG032143	79:967	NONHSAG022357	199:997	NONHSAG014012	57:249	NONHSAG041998	726:579
NONHSAG001938	381:1045	NONHSAG032143	492:1053	NONHSAG022357	163:731	NONHSAG014012	174:193	NONHSAG041998	629:847
NONHSAG001938	866:299	NONHSAG032143	782:263	NONHSAG022357	347:853	NONHSAG014012	135:715	NONHSAG041999	866:507
NONHSAG001938	616:669	NONHSAG032143	4:251	NONHSAG022358	970:833	NONHSAG014012	1141:183	NONHSAG041999	721:481
NONHSAG001938	280:425	NONHSAG032143	315:413	NONHSAG022358	1128:215	NONHSAG014012	483:1051	NONHSAG041999	667:753
NONHSAG001938	265:227	NONHSAG032153	544:391	NONHSAG022358	1150:329	NONHSAG014012	1083:401	NONHSAG041999	31:221
NONHSAG001941	1125:421	NONHSAG032153	828:339	NONHSAG022358	144:665	NONHSAG014012	1025:387	NONHSAG041999	533:265
NONHSAG001941	1090:931	NONHSAG032153	759:1	NONHSAG022358	265:531	NONHSAG014012	460:1057	NONHSAG041999	285:309
NONHSAG001941	151:361	NONHSAG032153	469:57	NONHSAG022358	684:955	NONHSAG014012	404:553	NONHSAG041999	388:1051
NONHSAG001941	517:779	NONHSAG032153	1114:419	NONHSAG022358	1053:253	NONHSAG014012	265:957	NONHSAG041999	992:199
NONHSAG001941	1053:771	NONHSAG032153	152:681	NONHSAG022358	837:577	NONHSAG014012	980:73	NONHSAG041999	56:1097
NONHSAG001941	340:649	NONHSAG032153	1078:115	NONHSAG022358	426:689	NONHSAG014012	33:1005	NONHSAG041999	170:137
NONHSAG001941	348:951	NONHSAG032153	458:825	NONHSAG022358	544:349	NONHSAG014012	734:77	NONHSAG041999	124:1109
NONHSAG001941	391:1093	NONHSAG032153	1023:789	NONHSAG022437	507:639	NONHSAG014012	1091:545	NONHSAG042046	812:679
NONHSAG001941	1108:927	NONHSAG032186	771:845	NONHSAG022437	953:597	NONHSAG014023	772:283	NONHSAG042046	703:103
NONHSAG001941	927:415	NONHSAG032186	87:133	NONHSAG022437	827:413	NONHSAG014023	759:657	NONHSAG042046	707:277
NONHSAG001941	628:1109	NONHSAG032186	518:1033	NONHSAG022437	957:231	NONHSAG014023	82:891	NONHSAG042046	428:337
NONHSAG001941	512:163	NONHSAG032186	1143:349	NONHSAG022437	784:317	NONHSAG014023	980:25	NONHSAG042046	1016:689
NONHSAG001941	517:743	NONHSAG032186	72:305	NONHSAG022437	963:649	NONHSAG014023	1153:931	NONHSAG042046	1152:11
NONHSAG001941	854:789	NONHSAG032186	49:61	NONHSAG022437	615:883	NONHSAG014023	728:589	NONHSAG042046	622:1147
NONHSAG001941	910:543	NONHSAG032186	601:1101	NONHSAG022437	961:333	NONHSAG014023	948:407	NONHSAG042046	775:1083
NONHSAG001941	263:933	NONHSAG032186	437:741	NONHSAG022437	384:1135	NONHSAG014023	1125:1033	NONHSAG053454	782:1111
NONHSAG001941	894:1085	NONHSAG032186	980:119	NONHSAG022437	396:281	NONHSAG014023	653:315	NONHSAG053454	682:1125
NONHSAG001941	91:1069	NONHSAG032186	375:197	NONHSAG022492	497:123	NONHSAG014023	525:855	NONHSAG053454	212:285
NONHSAG001941	604:179	NONHSAG032186	691:617	NONHSAG022492	973:353	NONHSAG014023	1132:805	NONHSAG053454	473:149
NONHSAG001941	456:831	NONHSAG032186	581:609	NONHSAG022492	471:215	NONHSAG014030	326:641	NONHSAG053454	51:639
NONHSAG001941	660:961	NONHSAG032186	817:197	NONHSAG022492	58:777	NONHSAG014030	584:491	NONHSAG053454	233:41
NONHSAG001941	190:23	NONHSAG032186	615:427	NONHSAG022492	845:681	NONHSAG014030	1156:259	NONHSAG053454	374:559
NONHSAG001965	1086:1011	NONHSAG032186	460:45	NONHSAG022492	589:549	NONHSAG014030	476:689	NONHSAG053454	811:807
NONHSAG001965	445:169	NONHSAG032186	889:979	NONHSAG022492	1064:803	NONHSAG014030	227:313	NONHSAG053462	614:613
NONHSAG001965	992:1015	NONHSAG032186	137:585	NONHSAG022492	592:713	NONHSAG014030	454:371	NONHSAG053462	344:641
NONHSAG001965	183:157	NONHSAG032186	719:715	NONHSAG022492	691:1045	NONHSAG014030	992:931	NONHSAG053462	259:103
NONHSAG001965	858:1075	NONHSAG032186	255:281	NONHSAG022492	1018:885	NONHSAG014030	835:953	NONHSAG053462	732:759
NONHSAG001965	565:1157	NONHSAG032186	583:257	NONHSAG022492	381:1111	NONHSAG014030	185:399	NONHSAG053462	258:265
NONHSAG001965	1105:799	NONHSAG032186	549:35	NONHSAG022576	568:1067	NONHSAG014030	1063:843	NONHSAG053462	273:117
NONHSAG001965	1089:219	NONHSAG032186	629:787	NONHSAG022576	825:349	NONHSAG014030	524:847	NONHSAG053462	237:1003
NONHSAG001965	548:683	NONHSAG032186	290:877	NONHSAG022576	548:1105	NONHSAG014030	47:367	NONHSAG053462	125:627
NONHSAG001965	781:1079	NONHSAG032186	1056:421	NONHSAG022576	27:293	NONHSAG014030	708:1095	NONHSAG053462	562:943
NONHSAG001965	27:457	NONHSAG032186	1088:633	NONHSAG022576	1067:61	NONHSAG014030	685:303	NONHSAG053462	510:87
NONHSAG001965	1159:515	NONHSAG032186	708:229	NONHSAG022576	944:395	NONHSAG014030	296:845	NONHSAG053469	986:347
NONHSAG001965	218:611	NONHSAG032186	894:287	NONHSAG022576	128:547	NONHSAG014030	335:821	NONHSAG053469	967:339
NONHSAG001965	552:355	NONHSAG032186	738:47	NONHSAG022576	1104:625	NONHSAG014030	92:1147	NONHSAG053469	458:99
NONHSAG001965	547:1077	NONHSAG032186	330:835	NONHSAG022576	114:753	NONHSAG014030	32:1103	NONHSAG053469	56:701
NONHSAG001965	1075:1145	NONHSAG032186	1046:823	NONHSAG022576	122:47	NONHSAG014030	633:153	NONHSAG053469	457:515
NONHSAG001965	786:3	NONHSAG032186	240:39	NONHSAG022576	1146:69	NONHSAG014030	884:193	NONHSAG053469	260:953
NONHSAG001965	308:723	NONHSAG032186	142:203	NONHSAG022580	642:545	NONHSAG014030	946:529	NONHSAG053469	944:1015
NONHSAG001965	878:219	NONHSAG032186	1136:619	NONHSAG022580	131:497	NONHSAG014035	602:87	NONHSAG053469	129:923
NONHSAG001965	475:705	NONHSAG032186	1115:163	NONHSAG022580	1121:1141	NONHSAG014035	1078:779	NONHSAG053469	150:139
NONHSAG001965	761:283	NONHSAG032186	824:791	NONHSAG022580	755:353	NONHSAG014035	788:215	NONHSAG053552	1088:1047
NONHSAG001966	367:301	NONHSAG032186	493:193	NONHSAG022580	951:245	NONHSAG014035	532:493	NONHSAG053552	528:325
NONHSAG001966	363:1075	NONHSAG032194	759:1093	NONHSAG022580	208:1061	NONHSAG014035	344:1137	NONHSAG053552	96:63
NONHSAG001966	1133:603	NONHSAG032194	189:719	NONHSAG022580	74:873	NONHSAG014035	35:877	NONHSAG053552	1074:427
NONHSAG001966	293:205	NONHSAG032194	727:107	NONHSAG022580	290:633	NONHSAG014035	330:1149	NONHSAG053552	391:645
NONHSAG001966	517:351	NONHSAG032194	309:641	NONHSAG022580	126:465	NONHSAG014035	436:557	NONHSAG053552	803:401
NONHSAG001966	25:201	NONHSAG032194	415:159	NONHSAG022588	1139:1135	NONHSAG014035	360:331	NONHSAG053577	241:481
NONHSAG001966	640:775	NONHSAG032194	740:291	NONHSAG022588	1052:929	NONHSAG014035	709:1011	NONHSAG053577	478:47
NONHSAG001966	1121:1115	NONHSAG032194	60:15	NONHSAG022588	510:147	NONHSAG014035	1144:929	NONHSAG053577	367:1057
NONHSAG001966	1088:1129	NONHSAG032194	514:329	NONHSAG022588	703:431	NONHSAG014073	488:889	NONHSAG053577	171:775
NONHSAG001966	751:557	NONHSAG032194	828:73	NONHSAG022588	333:743	NONHSAG014073	884:115	NONHSAG053577	825:713
NONHSAG001966	433:731	NONHSAG032194	644:771	NONHSAG022627	1158:1139	NONHSAG014073	467:429	NONHSAG053577	1155:1103
NONHSAG001970	954:79	NONHSAG032194	250:911	NONHSAG022627	594:493	NONHSAG014073	489:985	NONHSAG053577	224:1011
NONHSAG001970	1036:201	NONHSAG032197	1156:977	NONHSAG022627	353:743	NONHSAG014073	482:203	NONHSAG053577	378:855
NONHSAG001970	857:949	NONHSAG032197	435:685	NONHSAG022627	84:243	NONHSAG014073	1012:345	NONHSAG053577	867:993
NONHSAG001970	739:463	NONHSAG032197	928:793	NONHSAG022627	765:989	NONHSAG014073	134:439	NONHSAG053585	760:923
NONHSAG001970	145:153	NONHSAG032197	712:1039	NONHSAG022627	341:501	NONHSAG014073	945:93	NONHSAG053585	195:1027
NONHSAG001970	1045:395	NONHSAG032197	966:679	NONHSAG022627	476:71	NONHSAG014073	326:355	NONHSAG053585	774:823
NONHSAG001970	858:533	NONHSAG032197	570:615	NONHSAG022627	190:687	NONHSAG014073	978:845	NONHSAG053585	797:159
NONHSAG001970	884:805	NONHSAG032197	883:5	NONHSAG022627	970:1127	NONHSAG014079	661:1135	NONHSAG053585	419:271
NONHSAG001970	647:457	NONHSAG032197	500:95	NONHSAG022627	948:461	NONHSAG014079	746:661	NONHSAG053585	74:727
NONHSAG001970	312:5	NONHSAG032197	727:185	NONHSAG022627	602:1049	NONHSAG014079	803:959	NONHSAG053585	636:509
NONHSAG001970	862:607	NONHSAG032197	1081:39	NONHSAG022656	338:587	NONHSAG014079	241:947	NONHSAG053585	388:489
NONHSAG001984	204:225	NONHSAG032197	670:1111	NONHSAG022656	335:537	NONHSAG014079	890:915	NONHSAG053585	1017:929
NONHSAG001984	103:1159	NONHSAG032235	973:83	NONHSAG022656	273:687	NONHSAG014079	639:761	NONHSAG053585	50:587
NONHSAG001984	241:423	NONHSAG032235	503:707	NONHSAG022656	923:795	NONHSAG014079	683:525	NONHSAG053585	823:683
NONHSAG001984	391:293	NONHSAG032235	14:993	NONHSAG022656	413:239	NONHSAG014079	785:877	NONHSAG053586	650:985
NONHSAG001984	503:299	NONHSAG032235	24:945	NONHSAG022656	1026:931	NONHSAG014079	498:1131	NONHSAG053586	552:913
NONHSAG001984	346:949	NONHSAG032235	959:85	NONHSAG022656	1113:223	NONHSAG014079	292:541	NONHSAG053586	1055:339
NONHSAG001984	65:1129	NONHSAG032235	552:855	NONHSAG022656	466:279	NONHSAG014079	677:999	NONHSAG053586	223:143
NONHSAG001984	998:781	NONHSAG032235	312:941	NONHSAG022656	583:453	NONHSAG014079	761:57	NONHSAG053586	391:329
NONHSAG001984	152:397	NONHSAG032235	386:1129	NONHSAG022656	653:299	NONHSAG014079	837:145	NONHSAG053586	485:55
NONHSAG001984	655:897	NONHSAG032235	989:891	NONHSAG022655	667:763	NONHSAG014079	742:583	NONHSAG053586	302:171
NONHSAG001992	520:921	NONHSAG032235	278:75	NONHSAG022655	1002:923	NONHSAG014079	166:839	NONHSAG053586	166:1075

NONHSAG001992	652:401	NONHSAG032235	394:697	NONHSAG022655	896:905	NONHSAG014079	505:979	NONHSAG053586	876:1107
NONHSAG001992	1029:569	NONHSAG032245	383:885	NONHSAG022655	438:491	NONHSAG014079	828:801	NONHSAG053586	1154:17
NONHSAG001992	653:257	NONHSAG032245	748:587	NONHSAG022655	342:405	NONHSAG014079	212:631	NONHSAG053586	269:239
NONHSAG001992	121:457	NONHSAG032245	59:189	NONHSAG022655	926:407	NONHSAG014079	123:51	NONHSAG053586	84:171
NONHSAG001992	1051:75	NONHSAG032245	212:1025	NONHSAG022655	618:437	NONHSAG014079	1130:253	NONHSAG053586	218:731
NONHSAG001992	424:173	NONHSAG032245	615:495	NONHSAG022655	713:911	NONHSAG014079	788:585	NONHSAG053586	1125:437
NONHSAG001992	958:943	NONHSAG032245	260:583	NONHSAG022655	665:575	NONHSAG014090	790:1089	NONHSAG053586	318:471
NONHSAG001992	730:65	NONHSAG032245	290:185	NONHSAG022655	887:1127	NONHSAG014090	618:447	NONHSAG053586	788:285
NONHSAG001992	544:229	NONHSAG032245	1075:759	NONHSAG022655	480:105	NONHSAG014090	852:855	NONHSAG053586	1110:483
NONHSAG001992	88:83	NONHSAG032245	109:877	NONHSAG022655	1031:927	NONHSAG014090	803:903	NONHSAG053586	913:979
NONHSAG002015	822:567	NONHSAG032245	351:477	NONHSAG022655	727:297	NONHSAG014090	769:201	NONHSAG053586	1031:139
NONHSAG002015	366:841	NONHSAG032245	212:155	NONHSAG022655	569:537	NONHSAG014090	870:147	NONHSAG053589	488:809
NONHSAG002015	106:897	NONHSAG032250	556:291	NONHSAG022655	953:1161	NONHSAG014090	781:1117	NONHSAG053589	470:615
NONHSAG002015	534:645	NONHSAG032250	553:901	NONHSAG022655	169:1073	NONHSAG014090	1090:1021	NONHSAG053589	216:635
NONHSAG002015	542:99	NONHSAG032250	521:53	NONHSAG022655	346:43	NONHSAG014090	901:545	NONHSAG053589	775:361
NONHSAG002015	931:343	NONHSAG032250	908:771	NONHSAG022655	166:1047	NONHSAG014090	826:347	NONHSAG053589	377:49
NONHSAG002015	877:1149	NONHSAG032250	570:227	NONHSAG022655	1135:629	NONHSAG014090	388:781	NONHSAG053589	689:717
NONHSAG002029	232:1065	NONHSAG032250	602:805	NONHSAG022655	69:235	NONHSAG014099	1092:13	NONHSAG053589	837:1039
NONHSAG002029	1131:51	NONHSAG032250	558:531	NONHSAG022655	222:871	NONHSAG014099	224:955	NONHSAG053589	659:83
NONHSAG002029	1154:7	NONHSAG032250	436:569	NONHSAG022655	605:753	NONHSAG014099	423:229	NONHSAG053589	245:487
NONHSAG002029	352:91	NONHSAG032250	516:819	NONHSAG022655	747:965	NONHSAG014099	183:1011	NONHSAG053589	475:795
NONHSAG002029	1072:129	NONHSAG032270	368:1117	NONHSAG022655	431:1081	NONHSAG014099	159:797	NONHSAG053591	1108:791
NONHSAG002029	183:681	NONHSAG032270	288:191	NONHSAG022655	854:665	NONHSAG014099	95:499	NONHSAG053591	606:705
NONHSAG002029	552:381	NONHSAG032270	43:653	NONHSAG022655	803:705	NONHSAG014099	1042:45	NONHSAG053591	690:1143
NONHSAG002029	756:617	NONHSAG032270	1016:183	NONHSAG022655	464:51	NONHSAG014099	833:331	NONHSAG053591	484:841
NONHSAG002029	666:339	NONHSAG032270	403:107	NONHSAG022655	54:383	NONHSAG014099	1024:817	NONHSAG053591	934:1085
NONHSAG002029	744:21	NONHSAG032270	819:883	NONHSAG022655	1102:351	NONHSAG014099	1063:155	NONHSAG053591	353:355
NONHSAG002029	833:455	NONHSAG032270	1092:1089	NONHSAG022655	660:325	NONHSAG014099	293:477	NONHSAG053591	333:853
NONHSAG002048	578:881	NONHSAG032270	1147:175	NONHSAG022668	328:879	NONHSAG014114	559:293	NONHSAG053591	283:123
NONHSAG002048	289:1151	NONHSAG032270	740:797	NONHSAG022668	16:453	NONHSAG014114	1144:289	NONHSAG053591	14:145
NONHSAG002048	1092:515	NONHSAG032270	414:505	NONHSAG022668	638:969	NONHSAG014114	767:1087	NONHSAG053591	248:459
NONHSAG002048	441:547	NONHSAG032270	752:53	NONHSAG022668	522:591	NONHSAG014114	695:231	NONHSAG053591	343:495
NONHSAG002048	235:343	NONHSAG032285	81:475	NONHSAG022668	878:805	NONHSAG014114	472:711	NONHSAG053607	1105:369
NONHSAG002048	164:139	NONHSAG032285	242:619	NONHSAG022668	412:157	NONHSAG014114	105:235	NONHSAG053607	654:577
NONHSAG002048	249:973	NONHSAG032285	51:727	NONHSAG022668	847:433	NONHSAG014114	739:569	NONHSAG053607	293:459
NONHSAG002048	1014:629	NONHSAG032285	849:1019	NONHSAG022668	1124:963	NONHSAG014114	451:963	NONHSAG053607	1048:1145
NONHSAG002048	203:961	NONHSAG032285	396:955	NONHSAG022668	793:1003	NONHSAG014114	179:989	NONHSAG053607	699:51
NONHSAG002048	598:595	NONHSAG032285	660:17	NONHSAG022676	538:27	NONHSAG014114	1091:849	NONHSAG053607	318:721
NONHSAG002048	1063:1083	NONHSAG032285	950:857	NONHSAG022676	405:1087	NONHSAG014114	622:1043	NONHSAG053607	65:317
NONHSAG002049	57:1145	NONHSAG032285	209:957	NONHSAG022676	40:617	NONHSAG014130	672:313	NONHSAG053607	232:311
NONHSAG002049	266:167	NONHSAG032285	1130:1111	NONHSAG022676	582:737	NONHSAG014130	58:83	NONHSAG053607	630:1017
NONHSAG002049	506:791	NONHSAG032285	568:127	NONHSAG022676	50:1083	NONHSAG014130	33:857	NONHSAG053607	34:689
NONHSAG002049	293:823	NONHSAG032285	1127:1085	NONHSAG022676	141:867	NONHSAG014130	550:351	NONHSAG053607	1072:263
NONHSAG002049	598:677	NONHSAG032299	711:99	NONHSAG022676	1032:805	NONHSAG014130	886:1117	NONHSAG053607	682:947
NONHSAG002049	252:1127	NONHSAG032299	945:763	NONHSAG022676	1133:555	NONHSAG014130	689:731	NONHSAG053607	355:583
NONHSAG002049	1147:761	NONHSAG032299	1035:205	NONHSAG022676	328:1121	NONHSAG014130	291:197	NONHSAG053607	687:273
NONHSAG002049	105:883	NONHSAG032299	1110:829	NONHSAG022676	651:477	NONHSAG014130	558:583	NONHSAG053607	1102:347
NONHSAG002049	428:419	NONHSAG032299	1145:361	NONHSAG022676	757:875	NONHSAG014130	538:979	NONHSAG053607	623:541
NONHSAG002063	434:165	NONHSAG032299	469:143	NONHSAG022677	105:897	NONHSAG014130	902:139	NONHSAG053607	29:201
NONHSAG002063	571:827	NONHSAG032337	1084:745	NONHSAG022677	955:113	NONHSAG014130	800:1149	NONHSAG053607	254:1113
NONHSAG002063	955:803	NONHSAG032337	520:409	NONHSAG022677	657:615	NONHSAG014130	328:267	NONHSAG053607	561:1005
NONHSAG002063	991:471	NONHSAG032337	562:209	NONHSAG022677	53:733	NONHSAG014130	931:281	NONHSAG053607	387:9
NONHSAG002063	556:1069	NONHSAG032337	844:763	NONHSAG022677	1139:995	NONHSAG014130	372:349	NONHSAG053607	914:909
NONHSAG002063	122:69	NONHSAG032337	564:1041	NONHSAG022677	282:337	NONHSAG014130	11:15	NONHSAG053607	1048:559
NONHSAG002063	1070:1111	NONHSAG032387	867:207	NONHSAG022677	285:747	NONHSAG014130	23:1125	NONHSAG053614	855:387
NONHSAG002063	127:863	NONHSAG032387	659:261	NONHSAG022677	111:65	NONHSAG014130	555:121	NONHSAG053614	87:189
NONHSAG002063	246:563	NONHSAG032387	325:51	NONHSAG022677	467:579	NONHSAG014130	167:125	NONHSAG053614	530:435
NONHSAG002063	741:1149	NONHSAG032387	474:649	NONHSAG022677	226:773	NONHSAG014130	932:239	NONHSAG053614	732:329
NONHSAG002063	540:655	NONHSAG032387	917:243	NONHSAG022677	836:983	NONHSAG014130	279:433	NONHSAG053614	161:499
NONHSAG002096	561:845	NONHSAG032387	596:713	NONHSAG022735	248:891	NONHSAG014130	267:209	NONHSAG053614	1080:117
NONHSAG002096	643:687	NONHSAG032387	1050:53	NONHSAG022735	515:345	NONHSAG014136	753:561	NONHSAG053614	404:377
NONHSAG002096	880:807	NONHSAG032387	367:933	NONHSAG022735	861:697	NONHSAG014136	211:531	NONHSAG053614	522:659
NONHSAG002096	615:715	NONHSAG032387	347:771	NONHSAG022735	417:759	NONHSAG014136	390:67	NONHSAG053614	1123:1021
NONHSAG002096	1010:27	NONHSAG032387	374:169	NONHSAG022735	60:831	NONHSAG014136	35:785	NONHSAG053614	454:605
NONHSAG002096	628:63	NONHSAG032387	859:861	NONHSAG022735	341:933	NONHSAG014136	686:955	NONHSAG053614	376:255
NONHSAG002096	791:765	NONHSAG032387	460:593	NONHSAG022735	852:101	NONHSAG014136	43:1119	NONHSAG053614	899:163
NONHSAG002096	1007:669	NONHSAG032387	860:861	NONHSAG022735	264:237	NONHSAG014136	552:593	NONHSAG053614	1004:407
NONHSAG002096	178:171	NONHSAG032387	14:547	NONHSAG022735	770:581	NONHSAG014136	20:705	NONHSAG053614	726:995
NONHSAG002096	1014:133	NONHSAG032387	751:915	NONHSAG022735	428:957	NONHSAG014136	299:475	NONHSAG053614	900:529
NONHSAG002128	672:1133	NONHSAG032387	918:243	NONHSAG022735	1150:437	NONHSAG014136	1007:451	NONHSAG053614	190:491
NONHSAG002128	646:605	NONHSAG032387	454:259	NONHSAG022759	784:131	NONHSAG014136	991:897	NONHSAG053614	1093:91
NONHSAG002128	1139:25	NONHSAG032387	279:873	NONHSAG022759	241:883	NONHSAG014165	503:865	NONHSAG053614	1149:395
NONHSAG002128	372:801	NONHSAG032387	295:71	NONHSAG022759	967:729	NONHSAG014165	856:793	NONHSAG053614	1029:385
NONHSAG002128	493:361	NONHSAG032387	265:547	NONHSAG022759	959:909	NONHSAG014165	631:349	NONHSAG053614	654:185
NONHSAG002128	111:815	NONHSAG032387	784:499	NONHSAG022759	518:45	NONHSAG014165	1057:569	NONHSAG053675	669:385
NONHSAG002128	594:371	NONHSAG032387	637:43	NONHSAG022759	820:67	NONHSAG014165	1117:1153	NONHSAG053675	774:97
NONHSAG002128	650:231	NONHSAG032387	286:575	NONHSAG022759	679:867	NONHSAG014165	757:203	NONHSAG053675	299:125
NONHSAG002128	960:555	NONHSAG032387	512:9	NONHSAG022759	181:969	NONHSAG014165	451:825	NONHSAG053675	230:839
NONHSAG002128	248:1151	NONHSAG032387	324:51	NONHSAG022759	726:375	NONHSAG014165	731:677	NONHSAG053675	347:397
NONHSAG002128	686:39	NONHSAG032387	883:905	NONHSAG022759	1138:667	NONHSAG014169	492:365	NONHSAG053675	235:793
NONHSAG002128	71:941	NONHSAG032387	840:1065	NONHSAG022759	646:537	NONHSAG014169	1002:993	NONHSAG053675	298:91
NONHSAG002128	195:1089	NONHSAG032387	238:609	NONHSAG022817	1049:503	NONHSAG014169	97:163	NONHSAG053675	651:163
NONHSAG002128	255:311	NONHSAG032387	1070:387	NONHSAG022817	89:651	NONHSAG014169	238:81	NONHSAG053675	474:275
NONHSAG002128	59:1019	NONHSAG032387	832:447	NONHSAG022817	727:271	NONHSAG014169	1071:977	NONHSAG053675	773:509
NONHSAG002128	738:1127	NONHSAG032387	166:259	NONHSAG022817	1003:913	NONHSAG014169	647:211	NONHSAG053719	550:373
NONHSAG002128	603:573	NONHSAG032387	93:283	NONHSAG022817	1152:925	NONHSAG014169	278:507	NONHSAG053719	446:217
NONHSAG002128	667:995	NONHSAG032387	94:481	NONHSAG022817	678:497	NONHSAG014169	126:187	NONHSAG053719	398:323
NONHSAG002128	671:571	NONHSAG032387	508:149	NONHSAG022817	1029:497	NONHSAG014169	1132:793	NONHSAG053719	48:1031

NONHSAG002128	813:731	NONHSAG032387	484:225	NONHSAG022817	1031:335	NONHSAG014169	158:397	NONHSAG053719	52:1061
NONHSAG002128	33:353	NONHSAG032387	476:995	NONHSAG022817	170:325	NONHSAG014169	441:541	NONHSAG053719	1037:827
NONHSAG002128	1010:161	NONHSAG032387	370:155	NONHSAG022817	643:75	NONHSAG014169	839:157	NONHSAG053719	164:671
NONHSAG002128	232:139	NONHSAG032387	142:1043	NONHSAG022817	1161:797	NONHSAG014169	935:569	NONHSAG053719	462:789
NONHSAG002128	546:193	NONHSAG032387	42:1127	NONHSAG022830	285:953	NONHSAG014169	142:509	NONHSAG053719	185:873
NONHSAG002154	273:205	NONHSAG032387	617:1027	NONHSAG022830	386:639	NONHSAG014169	707:633	NONHSAG053719	106:397
NONHSAG002154	326:243	NONHSAG032387	1130:705	NONHSAG022830	1112:455	NONHSAG014169	153:941	NONHSAG053722	578:953
NONHSAG002154	906:723	NONHSAG032422	1001:935	NONHSAG022830	927:821	NONHSAG014169	972:627	NONHSAG053722	641:413
NONHSAG002154	661:1087	NONHSAG032422	781:647	NONHSAG022830	84:39	NONHSAG014169	310:275	NONHSAG053722	402:1055
NONHSAG002154	7:271	NONHSAG032422	975:243	NONHSAG022830	554:445	NONHSAG014169	882:581	NONHSAG053722	891:999
NONHSAG002154	468:357	NONHSAG032422	127:587	NONHSAG022830	251:357	NONHSAG014185	1159:739	NONHSAG053722	66:1055
NONHSAG002154	513:923	NONHSAG032422	725:313	NONHSAG022830	664:609	NONHSAG014185	587:1119	NONHSAG053722	836:863
NONHSAG002154	118:221	NONHSAG032422	948:261	NONHSAG022830	955:1037	NONHSAG014185	1045:997	NONHSAG053722	259:923
NONHSAG002154	920:1077	NONHSAG032422	985:957	NONHSAG022830	615:1019	NONHSAG014185	451:803	NONHSAG053722	1105:671
NONHSAG002154	770:291	NONHSAG032422	76:201	NONHSAG022830	1039:473	NONHSAG014185	79:177	NONHSAG053722	300:417
NONHSAG002154	651:827	NONHSAG032427	165:231	NONHSAG022841	239:1145	NONHSAG014185	97:157	NONHSAG053746	355:305
NONHSAG002156	437:137	NONHSAG032427	965:1067	NONHSAG022841	761:1023	NONHSAG014185	1045:541	NONHSAG053746	1030:1131
NONHSAG002156	529:753	NONHSAG032427	482:149	NONHSAG022841	39:1161	NONHSAG014185	978:113	NONHSAG053746	331:689
NONHSAG002156	450:25	NONHSAG032427	585:821	NONHSAG022841	410:441	NONHSAG014185	933:483	NONHSAG053746	171:571
NONHSAG002156	1037:1029	NONHSAG032427	177:897	NONHSAG022841	1112:625	NONHSAG014185	686:335	NONHSAG053746	109:691
NONHSAG002156	960:927	NONHSAG032427	715:971	NONHSAG022841	292:937	NONHSAG014185	22:617	NONHSAG053746	1058:341
NONHSAG002156	46:425	NONHSAG032427	585:505	NONHSAG022841	326:89	NONHSAG014197	89:99	NONHSAG053746	239:409
NONHSAG002156	362:781	NONHSAG032427	767:1009	NONHSAG022841	1131:261	NONHSAG014197	378:17	NONHSAG053746	180:961
NONHSAG002156	806:637	NONHSAG032431	430:1027	NONHSAG022852	736:413	NONHSAG014197	179:533	NONHSAG053746	321:277
NONHSAG002156	919:555	NONHSAG032431	435:513	NONHSAG022852	251:657	NONHSAG014197	289:399	NONHSAG053751	473:759
NONHSAG002156	337:951	NONHSAG032431	1091:127	NONHSAG022852	288:1045	NONHSAG014197	526:903	NONHSAG053751	1105:87
NONHSAG002177	763:1145	NONHSAG032431	888:323	NONHSAG022852	181:777	NONHSAG014197	647:555	NONHSAG053751	183:207
NONHSAG002177	420:1053	NONHSAG032431	755:913	NONHSAG022852	267:595	NONHSAG014197	1080:257	NONHSAG053751	922:795
NONHSAG002177	1010:1041	NONHSAG032431	837:795	NONHSAG022852	1074:415	NONHSAG014197	968:143	NONHSAG053751	406:113
NONHSAG002177	783:1115	NONHSAG032431	425:865	NONHSAG022852	638:491	NONHSAG014197	387:147	NONHSAG053751	218:937
NONHSAG002177	91:629	NONHSAG032431	249:1067	NONHSAG022852	1044:777	NONHSAG014197	407:645	NONHSAG053751	308:617
NONHSAG002177	891:195	NONHSAG032431	157:1147	NONHSAG022852	994:257	NONHSAG014197	1045:1061	NONHSAG053751	294:989
NONHSAG002177	439:169	NONHSAG032431	1066:413	NONHSAG022852	892:377	NONHSAG014198	389:57	NONHSAG053751	494:901
NONHSAG002177	1020:143	NONHSAG032431	227:99	NONHSAG022858	326:437	NONHSAG014198	616:565	NONHSAG053751	649:139
NONHSAG002177	838:515	NONHSAG032480	199:647	NONHSAG022858	214:755	NONHSAG014198	802:1149	NONHSAG053751	957:11
NONHSAG002177	16:745	NONHSAG032480	1066:853	NONHSAG022858	488:517	NONHSAG014198	146:1121	NONHSAG053784	743:293
NONHSAG002178	1080:557	NONHSAG032480	647:889	NONHSAG022858	77:207	NONHSAG014198	708:215	NONHSAG053784	567:1079
NONHSAG002178	705:739	NONHSAG032480	975:145	NONHSAG022858	476:581	NONHSAG014198	197:37	NONHSAG053784	350:923
NONHSAG002178	802:665	NONHSAG032480	563:1147	NONHSAG022858	75:361	NONHSAG014198	599:787	NONHSAG053784	500:359
NONHSAG002178	798:559	NONHSAG032480	777:641	NONHSAG022858	686:151	NONHSAG014198	531:921	NONHSAG053784	307:787
NONHSAG002178	958:1107	NONHSAG032480	545:645	NONHSAG022858	1146:169	NONHSAG014198	870:341	NONHSAG053784	613:403
NONHSAG002178	1139:727	NONHSAG032480	1126:37	NONHSAG022858	976:145	NONHSAG014202	236:389	NONHSAG053784	1059:691
NONHSAG002178	229:465	NONHSAG032480	210:169	NONHSAG022858	636:725	NONHSAG014202	958:381	NONHSAG053784	423:887
NONHSAG002178	835:283	NONHSAG032559	271:1149	NONHSAG022858	409:313	NONHSAG014202	973:1073	NONHSAG053784	1106:663
NONHSAG002178	861:87	NONHSAG032559	477:495	NONHSAG022867	159:713	NONHSAG014202	482:375	NONHSAG053784	1061:749
NONHSAG002178	230:465	NONHSAG032559	305:647	NONHSAG022867	1148:453	NONHSAG014202	658:1123	NONHSAG053784	448:995
NONHSAG002178	1039:3	NONHSAG032559	53:1073	NONHSAG022867	419:255	NONHSAG014202	26:499	NONHSAG053784	746:171
NONHSAG002178	360:1069	NONHSAG032559	593:857	NONHSAG022867	926:915	NONHSAG014202	869:649	NONHSAG053784	908:197
NONHSAG002178	321:513	NONHSAG032559	731:375	NONHSAG022867	502:595	NONHSAG014208	263:111	NONHSAG053784	937:625
NONHSAG002178	345:611	NONHSAG032559	914:327	NONHSAG022867	736:121	NONHSAG014208	170:77	NONHSAG053784	183:237
NONHSAG002178	322:513	NONHSAG032559	491:1011	NONHSAG022867	783:829	NONHSAG014208	895:611	NONHSAG053784	830:967
NONHSAG002178	1038:3	NONHSAG032559	1002:479	NONHSAG022867	294:653	NONHSAG014208	887:127	NONHSAG053784	63:123
NONHSAG002178	706:739	NONHSAG032559	540:27	NONHSAG022867	1148:787	NONHSAG014208	1133:857	NONHSAG053784	869:1143
NONHSAG002178	1058:173	NONHSAG032559	729:323	NONHSAG022867	488:417	NONHSAG014208	865:603	NONHSAG053784	257:649
NONHSAG002178	359:1069	NONHSAG032592	110:747	NONHSAG022906	583:835	NONHSAG014208	947:975	NONHSAG053784	937:1089
NONHSAG002178	872:805	NONHSAG032592	1012:925	NONHSAG022906	205:385	NONHSAG014208	210:901	NONHSAG053784	696:577
NONHSAG002178	1115:673	NONHSAG032592	464:253	NONHSAG022906	134:1071	NONHSAG014208	898:181	NONHSAG053784	870:963
NONHSAG002178	1079:557	NONHSAG032592	642:425	NONHSAG022906	1003:883	NONHSAG014208	905:823	NONHSAG053784	789:163
NONHSAG002204	371:277	NONHSAG032592	497:765	NONHSAG022906	509:237	NONHSAG014212	262:133	NONHSAG053784	441:139
NONHSAG002204	1114:321	NONHSAG032592	1046:1105	NONHSAG022906	456:947	NONHSAG014212	1162:469	NONHSAG053784	1102:79
NONHSAG002204	1113:145	NONHSAG032592	995:705	NONHSAG022906	977:1081	NONHSAG014212	570:589	NONHSAG053784	795:327
NONHSAG002204	51:599	NONHSAG032592	708:113	NONHSAG022906	389:931	NONHSAG014212	576:987	NONHSAG053784	362:1139
NONHSAG002204	749:833	NONHSAG032592	489:571	NONHSAG022906	1032:159	NONHSAG014212	863:837	NONHSAG053784	397:341
NONHSAG002204	1148:725	NONHSAG032592	452:549	NONHSAG022906	1084:687	NONHSAG014212	861:279	NONHSAG053784	916:237
NONHSAG002204	678:139	NONHSAG032593	1032:879	NONHSAG022906	767:483	NONHSAG014212	930:725	NONHSAG053784	737:413
NONHSAG002204	1024:381	NONHSAG032593	492:841	NONHSAG022906	391:161	NONHSAG014212	47:141	NONHSAG053784	324:993
NONHSAG002204	309:1127	NONHSAG032593	1107:689	NONHSAG022906	632:915	NONHSAG014212	875:533	NONHSAG053801	580:951
NONHSAG002204	938:939	NONHSAG032593	730:1003	NONHSAG022906	407:419	NONHSAG014212	765:793	NONHSAG053801	670:389
NONHSAG002204	110:555	NONHSAG032593	907:69	NONHSAG022906	717:295	NONHSAG014212	1044:631	NONHSAG053801	608:1027
NONHSAG002211	640:777	NONHSAG032593	530:911	NONHSAG022906	706:655	NONHSAG014212	522:723	NONHSAG053801	1013:1027
NONHSAG002211	665:3	NONHSAG032593	470:567	NONHSAG022906	563:179	NONHSAG014212	829:631	NONHSAG053801	143:579
NONHSAG002211	995:287	NONHSAG032593	1087:35	NONHSAG022906	167:667	NONHSAG014212	942:219	NONHSAG053801	1073:393
NONHSAG002211	154:551	NONHSAG032593	825:309	NONHSAG022924	471:605	NONHSAG014212	943:135	NONHSAG053801	85:65
NONHSAG002211	880:699	NONHSAG032593	108:51	NONHSAG022924	1150:381	NONHSAG014212	1055:537	NONHSAG053801	594:117
NONHSAG002211	797:329	NONHSAG032601	788:597	NONHSAG022924	89:275	NONHSAG014212	1147:229	NONHSAG053801	342:941
NONHSAG002211	694:1031	NONHSAG032601	352:643	NONHSAG022924	1152:625	NONHSAG014212	111:917	NONHSAG053801	558:655
NONHSAG002211	64:223	NONHSAG032601	454:285	NONHSAG022924	605:591	NONHSAG014212	868:883	NONHSAG053801	704:663
NONHSAG002211	800:711	NONHSAG032601	668:1075	NONHSAG022924	363:505	NONHSAG014215	1059:171	NONHSAG053821	65:255
NONHSAG002211	639:43	NONHSAG032601	962:593	NONHSAG022924	985:497	NONHSAG014215	1052:543	NONHSAG053821	498:1117
NONHSAG002211	187:1007	NONHSAG032601	366:1073	NONHSAG022924	484:1087	NONHSAG014215	138:589	NONHSAG053821	55:527
NONHSAG002211	382:269	NONHSAG032601	908:583	NONHSAG022924	691:447	NONHSAG014215	546:365	NONHSAG053821	575:1063
NONHSAG002211	543:697	NONHSAG032601	632:855	NONHSAG022924	314:247	NONHSAG014215	99:743	NONHSAG053821	1134:573
NONHSAG002211	465:1053	NONHSAG032601	63:717	NONHSAG022924	427:117	NONHSAG014215	39:27	NONHSAG053821	920:823
NONHSAG002211	791:873	NONHSAG032601	389:99	NONHSAG022925	842:1115	NONHSAG014215	1091:291	NONHSAG053821	4:763
NONHSAG002211	446:349	NONHSAG032601	533:191	NONHSAG022925	1137:413	NONHSAG014215	675:645	NONHSAG053821	98:83
NONHSAG002211	147:949	NONHSAG032608	968:1005	NONHSAG022925	687:889	NONHSAG014215	314:107	NONHSAG053821	890:793
NONHSAG002211	305:673	NONHSAG032608	311:681	NONHSAG022925	878:115	NONHSAG014215	622:1067	NONHSAG053821	252:1041
NONHSAG002211	1067:721	NONHSAG032608	1139:999	NONHSAG022925	511:777	NONHSAG014215	264:1149	NONHSAG053863	614:647

NONHSAG002211	680:227	NONHSAG032608	275:625	NONHSAG022925	311:1071	NONHSAG014215	948:1097	NONHSAG053863	944:189
NONHSAG002211	1004:285	NONHSAG032608	132:747	NONHSAG022925	503:41	NONHSAG014215	242:181	NONHSAG053863	201:839
NONHSAG002211	1006:233	NONHSAG032608	663:1113	NONHSAG022925	748:47	NONHSAG014215	489:101	NONHSAG053863	357:467
NONHSAG002218	257:123	NONHSAG032608	593:511	NONHSAG022925	326:309	NONHSAG014215	77:19	NONHSAG053863	1033:1063
NONHSAG002218	566:593	NONHSAG032608	1090:975	NONHSAG022925	899:305	NONHSAG014215	668:913	NONHSAG053863	1074:171
NONHSAG002218	689:579	NONHSAG032608	1064:1143	NONHSAG022925	611:927	NONHSAG014215	797:667	NONHSAG053863	56:1123
NONHSAG002218	121:13	NONHSAG032608	1154:483	NONHSAG022957	818:359	NONHSAG014215	935:215	NONHSAG053863	401:883
NONHSAG002218	696:499	NONHSAG032613	877:25	NONHSAG022957	432:185	NONHSAG014215	644:1113	NONHSAG053863	536:7
NONHSAG002218	780:147	NONHSAG032613	280:319	NONHSAG022957	140:739	NONHSAG014215	448:743	NONHSAG053863	324:763
NONHSAG002218	970:1023	NONHSAG032613	1030:357	NONHSAG022957	746:167	NONHSAG014215	422:237	NONHSAG053863	757:787
NONHSAG002218	860:719	NONHSAG032613	211:65	NONHSAG022957	1142:1095	NONHSAG014215	1098:267	NONHSAG053863	192:989
NONHSAG002218	668:931	NONHSAG032613	113:63	NONHSAG022957	548:829	NONHSAG014232	659:883	NONHSAG053863	778:61
NONHSAG002218	953:35	NONHSAG032613	895:1127	NONHSAG022957	152:919	NONHSAG014232	713:255	NONHSAG053863	104:163
NONHSAG002218	1109:309	NONHSAG032613	746:609	NONHSAG022957	894:883	NONHSAG014232	409:953	NONHSAG053863	491:245
NONHSAG002218	1100:303	NONHSAG032613	449:965	NONHSAG022968	943:451	NONHSAG014232	346:431	NONHSAG053863	505:763
NONHSAG002218	1006:1103	NONHSAG032613	379:579	NONHSAG022968	752:1025	NONHSAG014232	863:481	NONHSAG053863	79:957
NONHSAG002218	182:731	NONHSAG032613	805:1091	NONHSAG022968	252:485	NONHSAG014232	642:515	NONHSAG053863	112:1099
NONHSAG002218	766:161	NONHSAG032613	1029:1145	NONHSAG022968	260:921	NONHSAG014232	986:149	NONHSAG053863	267:35
NONHSAG002218	122:879	NONHSAG032620	639:717	NONHSAG022968	883:753	NONHSAG014232	368:989	NONHSAG053863	18:127
NONHSAG002223	180:351	NONHSAG032620	905:581	NONHSAG022968	594:323	NONHSAG014232	932:1143	NONHSAG053863	367:429
NONHSAG002223	544:1147	NONHSAG032620	822:1083	NONHSAG022968	370:699	NONHSAG014249	719:431	NONHSAG053863	57:621
NONHSAG002223	811:1009	NONHSAG032620	728:833	NONHSAG022968	475:967	NONHSAG014249	197:399	NONHSAG053877	701:701
NONHSAG002223	888:667	NONHSAG032620	145:397	NONHSAG022968	325:207	NONHSAG014249	591:455	NONHSAG053877	725:833
NONHSAG002223	959:781	NONHSAG032620	887:1021	NONHSAG022968	828:965	NONHSAG014249	921:607	NONHSAG053877	956:743
NONHSAG002223	704:165	NONHSAG032620	451:275	NONHSAG022968	223:553	NONHSAG014249	461:899	NONHSAG053877	418:371
NONHSAG002223	553:643	NONHSAG032620	577:1005	NONHSAG022969	394:1127	NONHSAG014249	331:463	NONHSAG053877	880:355
NONHSAG002223	784:987	NONHSAG032620	296:185	NONHSAG022969	16:1037	NONHSAG014249	654:829	NONHSAG053877	141:143
NONHSAG002223	258:711	NONHSAG032620	1150:839	NONHSAG022969	575:299	NONHSAG014249	982:871	NONHSAG053877	829:261
NONHSAG002223	824:411	NONHSAG032620	63:1011	NONHSAG022969	620:359	NONHSAG014249	712:911	NONHSAG053877	223:681
NONHSAG002223	894:181	NONHSAG032620	940:739	NONHSAG022969	1124:83	NONHSAG014249	503:393	NONHSAG053877	679:653
NONHSAG002277	297:1049	NONHSAG032620	528:245	NONHSAG022969	23:335	NONHSAG014249	595:223	NONHSAG053877	993:1107
NONHSAG002277	765:17	NONHSAG032620	970:793	NONHSAG022969	1085:1147	NONHSAG014269	905:381	NONHSAG053877	159:1
NONHSAG002277	713:189	NONHSAG032620	122:627	NONHSAG022969	1058:365	NONHSAG014269	333:339	NONHSAG053892	179:663
NONHSAG002277	62:51	NONHSAG032620	469:377	NONHSAG022969	155:567	NONHSAG014269	515:759	NONHSAG053892	739:695
NONHSAG002277	521:455	NONHSAG032629	1161:491	NONHSAG022969	174:11	NONHSAG014269	362:339	NONHSAG053892	145:135
NONHSAG002277	932:233	NONHSAG032629	751:679	NONHSAG022994	327:787	NONHSAG014269	44:983	NONHSAG053892	618:409
NONHSAG002277	24:1111	NONHSAG032629	579:121	NONHSAG022994	953:667	NONHSAG014269	287:609	NONHSAG053892	1017:1055
NONHSAG002277	1127:841	NONHSAG032629	34:55	NONHSAG022994	945:111	NONHSAG014269	722:313	NONHSAG053892	778:1033
NONHSAG002281	35:1005	NONHSAG032629	773:103	NONHSAG022994	27:459	NONHSAG014269	135:721	NONHSAG053892	966:837
NONHSAG002281	969:861	NONHSAG032629	989:603	NONHSAG022994	249:377	NONHSAG014269	187:801	NONHSAG053892	232:563
NONHSAG002281	170:525	NONHSAG032629	44:703	NONHSAG022994	571:723	NONHSAG014269	205:601	NONHSAG053893	1129:147
NONHSAG002281	519:833	NONHSAG032629	383:695	NONHSAG022994	1130:957	NONHSAG014269	190:581	NONHSAG053893	1145:279
NONHSAG002281	27:95	NONHSAG032629	968:627	NONHSAG022994	221:65	NONHSAG014269	941:1107	NONHSAG053893	929:1
NONHSAG002337	1146:975	NONHSAG032629	230:1107	NONHSAG022994	661:545	NONHSAG014269	112:757	NONHSAG053893	11:445
NONHSAG002337	248:515	NONHSAG032629	979:129	NONHSAG022994	469:427	NONHSAG014269	924:483	NONHSAG053893	1130:147
NONHSAG002337	859:377	NONHSAG032632	468:99	NONHSAG022994	167:831	NONHSAG014269	870:219	NONHSAG053893	1144:279
NONHSAG002337	860:639	NONHSAG032632	889:1149	NONHSAG022994	75:533	NONHSAG014269	719:785	NONHSAG053893	12:445
NONHSAG002337	550:15	NONHSAG032632	89:159	NONHSAG022994	307:977	NONHSAG014269	171:331	NONHSAG053893	893:891
NONHSAG002337	12:925	NONHSAG032632	207:95	NONHSAG022994	506:1043	NONHSAG014269	517:637	NONHSAG053893	892:891
NONHSAG002337	1027:779	NONHSAG032632	688:1123	NONHSAG022994	358:853	NONHSAG014269	968:343	NONHSAG053895	1139:349
NONHSAG002337	320:863	NONHSAG032632	829:865	NONHSAG022994	550:711	NONHSAG014269	795:263	NONHSAG053895	330:539
NONHSAG002337	623:677	NONHSAG032632	349:115	NONHSAG022994	1031:967	NONHSAG014269	1137:105	NONHSAG053895	243:801
NONHSAG002337	340:209	NONHSAG032632	759:731	NONHSAG022994	411:355	NONHSAG014269	56:785	NONHSAG053895	168:947
NONHSAG002360	303:977	NONHSAG032632	596:57	NONHSAG022994	873:383	NONHSAG014294	705:145	NONHSAG053895	967:1123
NONHSAG002360	372:575	NONHSAG032632	85:799	NONHSAG022994	983:1001	NONHSAG014294	326:809	NONHSAG053895	894:171
NONHSAG002360	770:971	NONHSAG032632	155:1095	NONHSAG022994	134:1143	NONHSAG014294	163:203	NONHSAG053900	940:1015
NONHSAG002360	1097:253	NONHSAG032643	581:595	NONHSAG022994	614:847	NONHSAG014294	999:415	NONHSAG053900	97:263
NONHSAG002360	959:877	NONHSAG032643	509:1009	NONHSAG022994	416:29	NONHSAG014294	531:807	NONHSAG053900	1154:553
NONHSAG002360	617:511	NONHSAG032643	385:1001	NONHSAG022994	1100:877	NONHSAG014294	862:273	NONHSAG053900	1005:49
NONHSAG002360	296:255	NONHSAG032643	830:803	NONHSAG022994	720:327	NONHSAG014294	53:317	NONHSAG053900	584:631
NONHSAG002360	55:689	NONHSAG032643	243:971	NONHSAG022994	566:885	NONHSAG014294	559:441	NONHSAG053900	873:285
NONHSAG002360	779:335	NONHSAG032643	1086:193	NONHSAG022994	457:597	NONHSAG014294	399:1025	NONHSAG053900	77:189
NONHSAG002360	805:947	NONHSAG032643	95:1123	NONHSAG022994	152:521	NONHSAG014294	802:383	NONHSAG053900	305:513
NONHSAG002360	490:1005	NONHSAG032643	161:825	NONHSAG022994	786:445	NONHSAG014294	494:425	NONHSAG053900	619:311
NONHSAG002376	861:887	NONHSAG032643	144:27	NONHSAG022994	681:931	NONHSAG014358	285:475	NONHSAG053900	166:147
NONHSAG002376	526:1137	NONHSAG032643	155:271	NONHSAG022994	737:811	NONHSAG014358	536:1103	NONHSAG053900	1118:1117
NONHSAG002376	415:83	NONHSAG032643	880:853	NONHSAG022994	1104:115	NONHSAG014358	809:757	NONHSAG053900	105:715
NONHSAG002376	26:435	NONHSAG032649	204:741	NONHSAG023009	990:43	NONHSAG014358	86:289	NONHSAG053985	937:463
NONHSAG002376	555:1013	NONHSAG032649	744:681	NONHSAG023009	687:845	NONHSAG014358	553:257	NONHSAG053985	1048:875
NONHSAG002376	767:233	NONHSAG032649	1040:1099	NONHSAG023009	1067:757	NONHSAG014358	84:415	NONHSAG053985	658:1057
NONHSAG002376	917:531	NONHSAG032649	655:949	NONHSAG023009	40:759	NONHSAG014358	383:1111	NONHSAG053985	107:1125
NONHSAG002376	595:353	NONHSAG032649	967:1081	NONHSAG023009	1089:773	NONHSAG014358	672:351	NONHSAG053985	174:183
NONHSAG002376	875:1131	NONHSAG032649	865:299	NONHSAG023009	887:67	NONHSAG014358	305:429	NONHSAG053985	292:1025
NONHSAG002376	468:669	NONHSAG032649	1127:967	NONHSAG023036	1120:1117	NONHSAG014358	732:447	NONHSAG053985	1129:373
NONHSAG002377	1011:911	NONHSAG032649	1037:693	NONHSAG023036	912:265	NONHSAG014358	721:703	NONHSAG053985	719:973
NONHSAG002377	971:165	NONHSAG032649	862:453	NONHSAG023036	1097:871	NONHSAG014358	26:549	NONHSAG053985	352:29
NONHSAG002377	912:65	NONHSAG032649	478:37	NONHSAG023036	1120:1101	NONHSAG014358	360:1059	NONHSAG053985	553:651
NONHSAG002377	725:395	NONHSAG032662	879:643	NONHSAG023036	550:1073	NONHSAG014358	523:299	NONHSAG053985	398:747
NONHSAG002377	1016:895	NONHSAG032662	1128:527	NONHSAG023036	182:1139	NONHSAG014358	48:679	NONHSAG054015	424:1029
NONHSAG002377	1106:737	NONHSAG032662	889:977	NONHSAG023036	1158:281	NONHSAG014358	148:397	NONHSAG054015	367:365
NONHSAG002377	451:859	NONHSAG032662	295:29	NONHSAG023036	196:1157	NONHSAG014358	467:45	NONHSAG054015	764:1009
NONHSAG002377	56:987	NONHSAG032662	573:403	NONHSAG023036	470:477	NONHSAG014358	1114:1047	NONHSAG054015	18:405
NONHSAG002377	477:515	NONHSAG032662	697:737	NONHSAG023036	987:43	NONHSAG014358	1137:775	NONHSAG054015	101:597
NONHSAG002377	554:153	NONHSAG032662	485:917	NONHSAG023036	286:867	NONHSAG014358	678:13	NONHSAG054015	844:289
NONHSAG002377	702:31	NONHSAG032662	1116:297	NONHSAG023036	488:713	NONHSAG014358	731:1003	NONHSAG054015	557:777
NONHSAG002377	76:407	NONHSAG032662	429:843	NONHSAG023036	995:397	NONHSAG014358	300:981	NONHSAG054015	955:165
NONHSAG002377	118:327	NONHSAG032662	200:785	NONHSAG023036	919:795	NONHSAG014410	63:977	NONHSAG054015	100:181
NONHSAG002377	834:689	NONHSAG032662	490:525	NONHSAG023036	977:1151	NONHSAG014410	435:903	NONHSAG054015	475:41

NONHSAG002377	810:735	NONHSAG032723	624:1023	NONHSAG023036	824:179	NONHSAG014410	916:423	NONHSAG054016	626:529
NONHSAG002377	1004:1051	NONHSAG032723	334:1157	NONHSAG023036	779:505	NONHSAG014410	64:851	NONHSAG054016	673:679
NONHSAG002377	105:155	NONHSAG032723	209:907	NONHSAG023036	729:745	NONHSAG014410	328:947	NONHSAG054016	523:1049
NONHSAG002377	889:707	NONHSAG032723	345:665	NONHSAG023036	989:367	NONHSAG014410	543:1127	NONHSAG054016	178:115
NONHSAG002377	181:1029	NONHSAG032723	1154:503	NONHSAG023036	1101:947	NONHSAG014410	313:697	NONHSAG054016	970:631
NONHSAG002377	826:93	NONHSAG032723	505:433	NONHSAG023052	55:665	NONHSAG014410	905:961	NONHSAG054016	516:291
NONHSAG002377	615:939	NONHSAG032723	488:951	NONHSAG023052	113:27	NONHSAG014410	155:201	NONHSAG054016	927:871
NONHSAG002377	897:977	NONHSAG032723	561:1031	NONHSAG023052	823:949	NONHSAG014436	304:369	NONHSAG054016	261:253
NONHSAG002389	838:719	NONHSAG032723	611:59	NONHSAG023052	728:131	NONHSAG014436	730:431	NONHSAG054016	14:347
NONHSAG002389	369:1069	NONHSAG032723	1030:701	NONHSAG023052	394:17	NONHSAG014436	438:1019	NONHSAG054016	532:137
NONHSAG002389	294:15	NONHSAG032723	247:665	NONHSAG023052	164:347	NONHSAG014436	618:175	NONHSAG054016	703:311
NONHSAG002389	574:961	NONHSAG032723	404:977	NONHSAG023052	267:1031	NONHSAG014436	346:583	NONHSAG054016	94:899
NONHSAG002389	886:379	NONHSAG032723	974:713	NONHSAG023052	3:831	NONHSAG014436	981:173	NONHSAG054016	1031:375
NONHSAG002389	116:205	NONHSAG032723	487:1013	NONHSAG023052	389:957	NONHSAG014436	931:399	NONHSAG054016	992:389
NONHSAG002389	814:389	NONHSAG032723	924:591	NONHSAG023052	302:701	NONHSAG014436	966:449	NONHSAG054016	808:239
NONHSAG002389	771:1117	NONHSAG032740	868:907	NONHSAG023052	1155:997	NONHSAG014438	390:459	NONHSAG054016	448:1159
NONHSAG002389	633:53	NONHSAG032740	437:995	NONHSAG023067	299:549	NONHSAG014438	305:643	NONHSAG054016	644:127
NONHSAG002389	352:1107	NONHSAG032740	555:847	NONHSAG023067	51:607	NONHSAG014438	112:585	NONHSAG054016	1007:53
NONHSAG002392	471:21	NONHSAG032740	576:1101	NONHSAG023067	621:333	NONHSAG014438	1080:629	NONHSAG054016	524:919
NONHSAG002392	446:25	NONHSAG032740	227:945	NONHSAG023067	143:489	NONHSAG014438	291:1	NONHSAG054016	696:321
NONHSAG002392	929:1085	NONHSAG032740	855:571	NONHSAG023067	794:31	NONHSAG014438	790:947	NONHSAG054016	986:397
NONHSAG002392	47:769	NONHSAG032740	677:51	NONHSAG023085	234:235	NONHSAG014438	863:321	NONHSAG054030	184:741
NONHSAG002392	1032:265	NONHSAG032740	813:1069	NONHSAG023085	417:479	NONHSAG014436	665:251	NONHSAG054030	1080:1095
NONHSAG002392	946:1143	NONHSAG032740	500:1089	NONHSAG023085	169:741	NONHSAG014436	474:861	NONHSAG054030	916:607
NONHSAG002392	10:1113	NONHSAG032740	496:1153	NONHSAG023085	836:55	NONHSAG014436	441:297	NONHSAG054030	856:31
NONHSAG002392	337:49	NONHSAG032740	835:531	NONHSAG023085	630:327	NONHSAG014436	519:637	NONHSAG054030	345:427
NONHSAG002392	723:21	NONHSAG032746	533:811	NONHSAG023085	820:385	NONHSAG014436	918:1023	NONHSAG054030	507:861
NONHSAG002406	969:305	NONHSAG032746	801:1143	NONHSAG023085	513:1111	NONHSAG014436	488:311	NONHSAG054030	956:1145
NONHSAG002406	461:763	NONHSAG032746	940:1047	NONHSAG023085	220:717	NONHSAG014436	140:303	NONHSAG054030	714:217
NONHSAG002406	668:403	NONHSAG032746	940:577	NONHSAG023085	678:959	NONHSAG014436	419:1067	NONHSAG054030	328:369
NONHSAG002406	246:807	NONHSAG032746	494:45	NONHSAG023085	536:1	NONHSAG014436	842:707	NONHSAG054030	1134:687
NONHSAG002406	943:107	NONHSAG032746	841:795	NONHSAG023085	835:819	NONHSAG014436	1064:611	NONHSAG054071	419:29
NONHSAG002406	791:547	NONHSAG032746	576:621	NONHSAG023088	454:749	NONHSAG014436	1098:533	NONHSAG054071	687:461
NONHSAG002406	1143:497	NONHSAG032746	1025:747	NONHSAG023088	1064:863	NONHSAG014436	754:861	NONHSAG054071	264:755
NONHSAG002406	927:147	NONHSAG032746	1147:685	NONHSAG023088	342:1015	NONHSAG014436	284:419	NONHSAG054071	220:873
NONHSAG002406	440:643	NONHSAG032746	368:1007	NONHSAG023088	681:985	NONHSAG014436	948:381	NONHSAG054071	565:537
NONHSAG002406	1029:859	NONHSAG032746	636:111	NONHSAG023088	972:975	NONHSAG014436	372:765	NONHSAG054071	167:611
NONHSAG002406	577:753	NONHSAG032750	388:1043	NONHSAG023088	1054:491	NONHSAG014436	715:759	NONHSAG054071	186:83
NONHSAG002410	960:289	NONHSAG032750	355:1009	NONHSAG023107	1111:579	NONHSAG014436	1098:281	NONHSAG054071	822:481
NONHSAG002410	315:831	NONHSAG032750	350:475	NONHSAG023107	9:501	NONHSAG014436	563:545	NONHSAG054071	1058:671
NONHSAG002410	527:283	NONHSAG032750	95:575	NONHSAG023107	474:617	NONHSAG014436	932:301	NONHSAG054071	755:705
NONHSAG002410	633:323	NONHSAG032750	725:279	NONHSAG023107	981:143	NONHSAG014440	390:575	NONHSAG054103	785:217
NONHSAG002410	171:661	NONHSAG032750	14:267	NONHSAG023114	736:611	NONHSAG014440	130:831	NONHSAG054103	155:1067
NONHSAG002410	568:27	NONHSAG032750	974:1149	NONHSAG023114	569:257	NONHSAG014440	900:881	NONHSAG054103	663:267
NONHSAG002410	772:197	NONHSAG032750	317:1051	NONHSAG023114	310:647	NONHSAG014440	267:771	NONHSAG054103	71:907
NONHSAG002410	5:693	NONHSAG032750	278:881	NONHSAG023114	426:843	NONHSAG014440	283:1047	NONHSAG054103	489:177
NONHSAG002410	891:741	NONHSAG032750	569:1075	NONHSAG023114	306:491	NONHSAG014440	418:111	NONHSAG054103	548:1111
NONHSAG002410	463:189	NONHSAG032750	505:789	NONHSAG023114	305:541	NONHSAG014440	330:1089	NONHSAG054103	1056:51
NONHSAG002410	659:685	NONHSAG032785	462:47	NONHSAG023114	57:263	NONHSAG014440	634:73	NONHSAG054103	110:925
NONHSAG002419	149:7	NONHSAG032785	776:439	NONHSAG023114	867:259	NONHSAG014443	273:677	NONHSAG054103	241:421
NONHSAG002419	479:87	NONHSAG032785	717:425	NONHSAG023118	743:993	NONHSAG014443	304:231	NONHSAG054103	572:661
NONHSAG002419	830:963	NONHSAG032785	220:867	NONHSAG023118	253:127	NONHSAG014443	925:237	NONHSAG054103	485:619
NONHSAG002419	2:157	NONHSAG032785	816:179	NONHSAG023118	670:93	NONHSAG014443	502:515	NONHSAG054109	311:1043
NONHSAG002419	621:1041	NONHSAG032785	762:73	NONHSAG023118	773:269	NONHSAG014436	174:563	NONHSAG054109	656:327
NONHSAG002419	100:375	NONHSAG032785	1131:77	NONHSAG023118	632:565	NONHSAG014436	223:503	NONHSAG054109	382:243
NONHSAG002419	444:1069	NONHSAG032785	782:183	NONHSAG023118	1098:315	NONHSAG014436	85:633	NONHSAG054109	649:867
NONHSAG002419	1065:113	NONHSAG032785	953:867	NONHSAG023118	420:985	NONHSAG014436	1120:751	NONHSAG054109	143:1027
NONHSAG002419	99:983	NONHSAG032785	170:299	NONHSAG023118	215:285	NONHSAG014436	969:815	NONHSAG054109	1082:533
NONHSAG002419	289:97	NONHSAG032791	557:645	NONHSAG023118	1118:275	NONHSAG014436	479:741	NONHSAG054109	997:473
NONHSAG002419	494:821	NONHSAG032791	1038:267	NONHSAG023129	1071:1039	NONHSAG014436	1134:499	NONHSAG054109	143:197
NONHSAG002421	321:1051	NONHSAG032791	806:43	NONHSAG023129	704:917	NONHSAG014436	254:879	NONHSAG054109	726:103
NONHSAG002421	1057:333	NONHSAG032791	290:683	NONHSAG023129	1103:355	NONHSAG014436	572:341	NONHSAG054109	593:1121
NONHSAG002421	81:135	NONHSAG032791	1015:589	NONHSAG023129	780:1147	NONHSAG014436	621:919	NONHSAG054109	695:53
NONHSAG002421	660:67	NONHSAG032791	268:711	NONHSAG023129	1123:937	NONHSAG014436	715:787	NONHSAG054140	270:761
NONHSAG002421	432:899	NONHSAG032791	1098:693	NONHSAG023129	112:1131	NONHSAG014436	795:133	NONHSAG054140	84:599
NONHSAG002421	972:1089	NONHSAG032791	96:429	NONHSAG023129	769:603	NONHSAG014436	390:1069	NONHSAG054140	563:1155
NONHSAG002421	317:845	NONHSAG032791	900:143	NONHSAG023129	607:195	NONHSAG014436	857:649	NONHSAG054140	640:573
NONHSAG002421	532:791	NONHSAG032791	187:687	NONHSAG023173	841:67	NONHSAG014436	620:437	NONHSAG054140	561:605
NONHSAG002421	938:463	NONHSAG032791	636:755	NONHSAG023173	443:785	NONHSAG014436	182:277	NONHSAG054140	594:147
NONHSAG002421	250:989	NONHSAG032815	629:275	NONHSAG023173	98:373	NONHSAG014436	407:373	NONHSAG054140	875:1081
NONHSAG002421	810:179	NONHSAG032815	249:863	NONHSAG023173	641:495	NONHSAG014445	1001:911	NONHSAG054140	281:835
NONHSAG002440	1021:815	NONHSAG032815	901:697	NONHSAG023173	637:711	NONHSAG014445	899:729	NONHSAG054140	424:303
NONHSAG002440	489:193	NONHSAG032815	888:577	NONHSAG023173	1026:1099	NONHSAG014445	743:567	NONHSAG054140	732:183
NONHSAG002440	925:603	NONHSAG032815	88:259	NONHSAG023173	129:231	NONHSAG014445	1022:1129	NONHSAG054179	294:747
NONHSAG002440	1084:547	NONHSAG032815	664:591	NONHSAG023173	502:217	NONHSAG014445	430:113	NONHSAG054179	1125:731
NONHSAG002440	28:1017	NONHSAG032815	128:129	NONHSAG023173	809:345	NONHSAG014445	945:77	NONHSAG054179	862:803
NONHSAG002440	988:361	NONHSAG032815	172:385	NONHSAG023173	456:643	NONHSAG014445	461:529	NONHSAG054179	118:963
NONHSAG002440	985:975	NONHSAG032815	12:1049	NONHSAG023173	191:881	NONHSAG014445	86:447	NONHSAG054179	280:615
NONHSAG002440	783:653	NONHSAG032815	669:301	NONHSAG023180	534:941	NONHSAG014445	184:697	NONHSAG054179	127:439
NONHSAG002440	341:523	NONHSAG032815	824:399	NONHSAG023180	103:117	NONHSAG014436	1072:753	NONHSAG054179	149:489
NONHSAG002455	801:769	NONHSAG032817	273:1109	NONHSAG023180	704:701	NONHSAG014436	349:441	NONHSAG054188	945:1125
NONHSAG002455	1092:725	NONHSAG032817	435:469	NONHSAG023180	1125:749	NONHSAG014436	479:393	NONHSAG054188	651:975
NONHSAG002455	684:823	NONHSAG032817	701:651	NONHSAG023180	165:133	NONHSAG014436	711:409	NONHSAG054188	289:5
NONHSAG002455	122:919	NONHSAG032817	275:857	NONHSAG023180	132:369	NONHSAG014436	841:977	NONHSAG054188	119:683
NONHSAG002455	524:903	NONHSAG032817	194:963	NONHSAG023180	575:1103	NONHSAG014436	227:1053	NONHSAG054188	890:133
NONHSAG002455	10:717	NONHSAG032817	897:149	NONHSAG023180	354:253	NONHSAG014436	585:7	NONHSAG054188	350:905
NONHSAG002455	920:227	NONHSAG032817	635:445	NONHSAG023180	1067:509	NONHSAG014436	1067:857	NONHSAG054188	844:405
NONHSAG002455	289:627	NONHSAG032817	27:195	NONHSAG023185	398:1101	NONHSAG014436	543:613	NONHSAG054188	1158:733

NONHSAG002455	1124:617	NONHSAG032817	175:957	NONHSAG023185	449:465	NONHSAG014436	1073:527	NONHSAG054188	77:531
NONHSAG002455	205:219	NONHSAG032817	780:509	NONHSAG023185	843:381	NONHSAG014436	43:691	NONHSAG054188	328:931
NONHSAG002455	175:1087	NONHSAG032817	507:1131	NONHSAG023185	56:871	NONHSAG014448	534:477	NONHSAG054188	1126:1003
NONHSAG002458	211:489	NONHSAG032839	60:783	NONHSAG023185	98:749	NONHSAG014448	111:949	NONHSAG054199	368:883
NONHSAG002458	142:961	NONHSAG032839	959:445	NONHSAG023185	729:289	NONHSAG014448	114:949	NONHSAG054199	1104:813
NONHSAG002458	1011:361	NONHSAG032839	32:199	NONHSAG023185	382:49	NONHSAG014448	113:949	NONHSAG054199	524:1129
NONHSAG002458	1132:317	NONHSAG032839	180:749	NONHSAG023185	517:771	NONHSAG014448	112:949	NONHSAG054199	775:817
NONHSAG002458	210:743	NONHSAG032839	651:213	NONHSAG023185	605:975	NONHSAG014454	21:769	NONHSAG054199	729:757
NONHSAG002458	213:177	NONHSAG032839	440:159	NONHSAG023185	112:117	NONHSAG014454	239:863	NONHSAG054199	706:319
NONHSAG002458	965:199	NONHSAG032839	558:1121	NONHSAG023185	153:883	NONHSAG014454	980:29	NONHSAG054199	526:1061
NONHSAG002458	795:569	NONHSAG032839	860:727	NONHSAG023185	560:617	NONHSAG014454	971:563	NONHSAG054199	118:59
NONHSAG002458	756:221	NONHSAG032855	198:157	NONHSAG023185	23:1025	NONHSAG014454	1092:401	NONHSAG054199	372:451
NONHSAG002458	965:135	NONHSAG032855	301:1013	NONHSAG023185	236:325	NONHSAG014454	940:551	NONHSAG054199	223:133
NONHSAG002458	211:189	NONHSAG032855	299:725	NONHSAG023185	756:257	NONHSAG014454	69:231	NONHSAG054199	189:795
NONHSAG002458	891:503	NONHSAG032855	592:1091	NONHSAG023185	387:1079	NONHSAG014569	760:1119	NONHSAG054199	277:127
NONHSAG002458	316:25	NONHSAG032855	436:1055	NONHSAG023185	961:879	NONHSAG014569	747:1061	NONHSAG054199	132:107
NONHSAG002458	1149:1063	NONHSAG032855	1158:355	NONHSAG023185	192:481	NONHSAG014569	767:1121	NONHSAG054199	363:1069
NONHSAG002458	397:1	NONHSAG032855	221:59	NONHSAG023185	719:197	NONHSAG014569	227:447	NONHSAG054199	590:93
NONHSAG002458	494:835	NONHSAG032855	1059:11	NONHSAG023185	353:461	NONHSAG014569	94:751	NONHSAG054326	440:75
NONHSAG002458	502:273	NONHSAG032855	462:985	NONHSAG023185	264:1135	NONHSAG014569	1084:567	NONHSAG054326	461:281
NONHSAG002458	255:461	NONHSAG032855	877:989	NONHSAG023185	209:259	NONHSAG014569	217:1011	NONHSAG054326	669:41
NONHSAG002458	772:271	NONHSAG032855	1105:1123	NONHSAG023185	191:535	NONHSAG014569	650:501	NONHSAG054326	1043:947
NONHSAG002458	460:147	NONHSAG032855	529:967	NONHSAG023185	330:213	NONHSAG014569	86:125	NONHSAG054326	1133:743
NONHSAG002458	404:237	NONHSAG032855	28:257	NONHSAG023185	485:923	NONHSAG014569	1030:957	NONHSAG054326	722:1127
NONHSAG002458	750:303	NONHSAG032855	34:207	NONHSAG023185	332:267	NONHSAG014569	1071:289	NONHSAG054326	281:135
NONHSAG002465	94:173	NONHSAG032855	429:919	NONHSAG023185	1088:279	NONHSAG014574	845:231	NONHSAG054326	314:299
NONHSAG002465	473:679	NONHSAG032855	969:567	NONHSAG023185	749:87	NONHSAG014574	995:417	NONHSAG054326	1069:743
NONHSAG002465	1156:837	NONHSAG032855	799:185	NONHSAG023185	151:589	NONHSAG014574	769:269	NONHSAG054326	283:403
NONHSAG002465	30:1107	NONHSAG032855	533:1021	NONHSAG023185	412:807	NONHSAG014574	588:675	NONHSAG054326	370:243
NONHSAG002465	563:631	NONHSAG032855	830:911	NONHSAG023185	573:721	NONHSAG014574	22:235	NONHSAG054326	753:239
NONHSAG002465	1043:1057	NONHSAG032855	568:957	NONHSAG023185	572:223	NONHSAG014574	1073:781	NONHSAG054326	598:19
NONHSAG002516	550:203	NONHSAG032855	712:379	NONHSAG023185	844:277	NONHSAG014574	1034:143	NONHSAG054326	536:787
NONHSAG002516	355:243	NONHSAG032855	747:717	NONHSAG023185	1033:267	NONHSAG014574	58:1079	NONHSAG054326	711:1035
NONHSAG002516	1122:713	NONHSAG032935	831:329	NONHSAG023185	673:561	NONHSAG014574	461:551	NONHSAG054326	713:797
NONHSAG002516	690:757	NONHSAG032935	651:1155	NONHSAG023185	1008:821	NONHSAG014574	825:1055	NONHSAG054326	1061:351
NONHSAG002516	1017:1129	NONHSAG032935	121:521	NONHSAG023185	950:739	NONHSAG014574	918:595	NONHSAG054326	1091:339
NONHSAG002516	863:1035	NONHSAG032935	824:1	NONHSAG023185	206:983	NONHSAG014611	426:31	NONHSAG054326	1159:1093
NONHSAG002516	997:223	NONHSAG032935	182:197	NONHSAG023185	1125:1057	NONHSAG014611	811:103	NONHSAG054326	556:567
NONHSAG002516	707:1033	NONHSAG032935	990:841	NONHSAG023185	696:691	NONHSAG014611	788:343	NONHSAG054326	564:331
NONHSAG002516	402:1127	NONHSAG032935	454:45	NONHSAG023194	457:233	NONHSAG014611	529:379	NONHSAG054344	1035:279
NONHSAG002516	842:309	NONHSAG032935	380:19	NONHSAG023194	37:371	NONHSAG014611	790:361	NONHSAG054344	20:683
NONHSAG002516	102:63	NONHSAG032949	209:157	NONHSAG023194	418:109	NONHSAG014611	729:739	NONHSAG054344	590:931
NONHSAG002535	632:483	NONHSAG032949	1086:305	NONHSAG023194	1116:639	NONHSAG014611	1098:961	NONHSAG054344	766:719
NONHSAG002535	1050:241	NONHSAG032949	1131:837	NONHSAG023237	939:197	NONHSAG014611	736:637	NONHSAG054344	414:253
NONHSAG002535	1150:433	NONHSAG032949	435:1027	NONHSAG023237	24:395	NONHSAG014611	1053:1005	NONHSAG054344	225:81
NONHSAG002535	679:409	NONHSAG032949	279:929	NONHSAG023237	320:869	NONHSAG014661	846:963	NONHSAG054344	1087:19
NONHSAG002535	433:409	NONHSAG032949	451:809	NONHSAG023237	940:1133	NONHSAG014661	1134:233	NONHSAG054344	854:1037
NONHSAG002535	108:251	NONHSAG032949	15:21	NONHSAG023237	43:231	NONHSAG014661	402:29	NONHSAG054344	102:1017
NONHSAG002535	569:497	NONHSAG032949	733:753	NONHSAG023237	1016:941	NONHSAG014661	68:159	NONHSAG054344	451:77
NONHSAG002535	1080:847	NONHSAG032949	1133:897	NONHSAG023237	889:1005	NONHSAG014661	960:167	NONHSAG054344	20:839
NONHSAG002535	165:975	NONHSAG032949	1158:801	NONHSAG023237	155:305	NONHSAG014661	203:209	NONHSAG054345	396:829
NONHSAG002535	47:153	NONHSAG032951	793:965	NONHSAG023237	342:485	NONHSAG014661	218:197	NONHSAG054345	877:471
NONHSAG002535	800:719	NONHSAG032951	414:483	NONHSAG023237	933:305	NONHSAG014661	741:1123	NONHSAG054345	299:273
NONHSAG002538	333:789	NONHSAG032951	1140:513	NONHSAG023237	267:1159	NONHSAG014663	828:181	NONHSAG054345	517:649
NONHSAG002538	925:1025	NONHSAG032951	116:299	NONHSAG023237	330:495	NONHSAG014663	65:83	NONHSAG054345	390:271
NONHSAG002538	794:507	NONHSAG032951	822:945	NONHSAG023237	6:89	NONHSAG014663	857:171	NONHSAG054345	627:91
NONHSAG002538	771:535	NONHSAG032951	1068:643	NONHSAG023237	1028:1143	NONHSAG014663	728:541	NONHSAG054345	847:1011
NONHSAG002538	484:465	NONHSAG032951	133:635	NONHSAG023237	849:331	NONHSAG014663	484:1063	NONHSAG054345	909:331
NONHSAG002538	1075:787	NONHSAG032951	310:245	NONHSAG023237	666:189	NONHSAG014663	1100:75	NONHSAG054345	775:185
NONHSAG002538	524:595	NONHSAG032951	91:801	NONHSAG023237	651:395	NONHSAG014663	925:1131	NONHSAG054345	539:667
NONHSAG002538	699:323	NONHSAG032951	401:267	NONHSAG023237	34:1005	NONHSAG014663	274:807	NONHSAG054345	3:257
NONHSAG002538	407:909	NONHSAG032951	183:445	NONHSAG023237	757:965	NONHSAG014663	606:311	NONHSAG054445	460:347
NONHSAG002538	781:265	NONHSAG032956	24:513	NONHSAG023237	697:663	NONHSAG014663	895:261	NONHSAG054445	137:1067
NONHSAG002549	1041:841	NONHSAG032956	245:339	NONHSAG023237	1009:1161	NONHSAG014663	766:1077	NONHSAG054445	161:969
NONHSAG002549	286:427	NONHSAG032956	443:219	NONHSAG023237	678:559	NONHSAG014667	1006:313	NONHSAG054445	779:105
NONHSAG002549	347:835	NONHSAG032956	672:95	NONHSAG023238	1065:995	NONHSAG014667	1040:157	NONHSAG054445	805:467
NONHSAG002549	1126:229	NONHSAG032956	743:923	NONHSAG023238	782:999	NONHSAG014667	1152:201	NONHSAG054445	160:471
NONHSAG002549	597:531	NONHSAG032956	459:393	NONHSAG023238	974:619	NONHSAG014667	124:461	NONHSAG054445	1079:593
NONHSAG002549	864:1161	NONHSAG032956	623:1041	NONHSAG023238	352:931	NONHSAG014667	546:1005	NONHSAG054445	716:489
NONHSAG002549	792:447	NONHSAG032956	1158:143	NONHSAG023238	977:403	NONHSAG014667	782:79	NONHSAG054445	445:171
NONHSAG002549	1072:519	NONHSAG032956	980:361	NONHSAG023238	981:593	NONHSAG014667	555:19	NONHSAG054445	53:33
NONHSAG002549	381:1069	NONHSAG032956	798:707	NONHSAG023238	381:1043	NONHSAG014667	776:809	NONHSAG054445	605:531
NONHSAG002549	354:1057	NONHSAG032956	287:1145	NONHSAG023238	295:253	NONHSAG014746	843:797	NONHSAG054462	301:581
NONHSAG002549	597:443	NONHSAG032959	291:681	NONHSAG023238	926:277	NONHSAG014746	62:217	NONHSAG054462	1095:979
NONHSAG002555	119:105	NONHSAG032959	846:707	NONHSAG023238	507:749	NONHSAG014746	817:737	NONHSAG054462	1012:1031
NONHSAG002555	937:1003	NONHSAG032959	212:29	NONHSAG023238	1009:481	NONHSAG014746	622:941	NONHSAG054462	833:267
NONHSAG002555	118:225	NONHSAG032959	1120:755	NONHSAG023238	79:369	NONHSAG014746	563:35	NONHSAG054462	62:1141
NONHSAG002555	655:67	NONHSAG032959	1116:985	NONHSAG023238	835:193	NONHSAG014746	824:1117	NONHSAG054462	139:329
NONHSAG002555	362:535	NONHSAG032959	52:511	NONHSAG023238	857:583	NONHSAG014746	399:343	NONHSAG054462	297:265
NONHSAG002555	920:443	NONHSAG032959	688:867	NONHSAG023238	162:919	NONHSAG014746	710:251	NONHSAG054462	77:305
NONHSAG002555	44:677	NONHSAG032959	783:1025	NONHSAG023238	908:947	NONHSAG014746	740:215	NONHSAG054462	771:1137
NONHSAG002555	799:965	NONHSAG032959	452:1117	NONHSAG023238	715:755	NONHSAG014746	833:99	NONHSAG054468	936:1141
NONHSAG002615	567:911	NONHSAG032959	578:265	NONHSAG023238	299:1133	NONHSAG014746	715:235	NONHSAG054468	1062:325
NONHSAG002615	952:211	NONHSAG032960	150:275	NONHSAG023238	977:107	NONHSAG014747	826:1035	NONHSAG054468	966:693
NONHSAG002615	138:417	NONHSAG032960	739:301	NONHSAG023238	513:1049	NONHSAG014747	188:133	NONHSAG054468	691:1153
NONHSAG002615	166:743	NONHSAG032960	814:1081	NONHSAG023238	22:57	NONHSAG014747	53:197	NONHSAG054468	21:813
NONHSAG002728	947:57	NONHSAG032960	323:847	NONHSAG023238	683:593	NONHSAG014747	97:593	NONHSAG054468	574:1087
NONHSAG002728	203:1151	NONHSAG032960	987:865	NONHSAG023245	378:335	NONHSAG014747	392:611	NONHSAG054468	497:699

NONHSAG002728	87:513	NONHSAG032960	1068:925	NONHSAG023245	88:767	NONHSAG014747	83:35	NONHSAG054468	861:1083
NONHSAG002728	1158:575	NONHSAG032960	57:165	NONHSAG023245	636:817	NONHSAG014747	1074:869	NONHSAG054468	793:1019
NONHSAG002728	1064:75	NONHSAG032960	497:397	NONHSAG023245	258:1057	NONHSAG014747	10:1067	NONHSAG054468	111:409
NONHSAG002728	37:883	NONHSAG032960	936:113	NONHSAG023245	993:1037	NONHSAG014747	174:129	NONHSAG054468	535:877
NONHSAG002758	756:371	NONHSAG032960	745:197	NONHSAG023245	787:181	NONHSAG014747	1156:179	NONHSAG054478	198:79
NONHSAG002758	703:643	NONHSAG032960	835:259	NONHSAG023245	904:51	NONHSAG014747	984:119	NONHSAG054478	1083:617
NONHSAG002758	647:473	NONHSAG032960	1007:563	NONHSAG023245	728:729	NONHSAG014790	356:1023	NONHSAG054478	775:631
NONHSAG002758	418:253	NONHSAG032960	160:1151	NONHSAG023245	367:195	NONHSAG014790	973:359	NONHSAG054478	1014:899
NONHSAG002758	31:999	NONHSAG032960	810:561	NONHSAG023245	819:509	NONHSAG014790	1025:1027	NONHSAG054478	737:205
NONHSAG002758	232:571	NONHSAG032960	326:393	NONHSAG023245	825:837	NONHSAG014790	1031:669	NONHSAG054478	673:7
NONHSAG002758	733:1005	NONHSAG032960	699:527	NONHSAG023245	329:921	NONHSAG014790	1026:471	NONHSAG054478	95:213
NONHSAG002758	292:1009	NONHSAG032960	909:749	NONHSAG023245	593:949	NONHSAG014790	1152:519	NONHSAG054478	284:231
NONHSAG002759	41:103	NONHSAG032960	96:863	NONHSAG023245	567:41	NONHSAG014790	1018:483	NONHSAG054478	907:833
NONHSAG002759	903:513	NONHSAG032961	1011:385	NONHSAG023245	706:683	NONHSAG014790	853:565	NONHSAG054478	814:649
NONHSAG002759	159:895	NONHSAG032961	439:357	NONHSAG023245	840:339	NONHSAG014790	385:23	NONHSAG054478	308:667
NONHSAG002759	829:149	NONHSAG032961	189:389	NONHSAG023245	1030:765	NONHSAG014790	106:171	NONHSAG054549	725:837
NONHSAG002759	607:705	NONHSAG032961	531:147	NONHSAG023245	616:951	NONHSAG014790	831:469	NONHSAG054549	723:141
NONHSAG002759	999:979	NONHSAG032961	872:301	NONHSAG023245	565:645	NONHSAG014806	409:153	NONHSAG054549	1139:67
NONHSAG002759	565:605	NONHSAG032961	854:251	NONHSAG023247	173:1111	NONHSAG014806	983:631	NONHSAG054549	1140:691
NONHSAG002759	217:1059	NONHSAG032961	898:595	NONHSAG023247	115:331	NONHSAG014806	954:461	NONHSAG054549	574:513
NONHSAG002759	1062:735	NONHSAG032961	878:625	NONHSAG023247	107:735	NONHSAG014806	534:835	NONHSAG054549	1061:125
NONHSAG002759	785:681	NONHSAG032961	860:207	NONHSAG023247	686:99	NONHSAG014806	295:461	NONHSAG054549	339:449
NONHSAG002759	294:1115	NONHSAG032979	230:911	NONHSAG023247	913:101	NONHSAG014806	1028:573	NONHSAG054549	448:1107
NONHSAG002759	48:959	NONHSAG032979	242:643	NONHSAG023247	148:365	NONHSAG014806	261:1109	NONHSAG054549	594:181
NONHSAG002759	1132:551	NONHSAG032979	1053:907	NONHSAG023247	521:555	NONHSAG014806	12:645	NONHSAG054549	1034:67
NONHSAG002759	628:591	NONHSAG032979	185:1017	NONHSAG023247	883:549	NONHSAG014806	627:903	NONHSAG054549	230:333
NONHSAG002759	269:753	NONHSAG032979	421:201	NONHSAG023247	201:349	NONHSAG014806	1077:1045	NONHSAG054549	726:597
NONHSAG002759	933:973	NONHSAG032979	1008:1115	NONHSAG023247	1035:615	NONHSAG014811	731:173	NONHSAG054549	582:493
NONHSAG002759	616:727	NONHSAG032979	761:637	NONHSAG023281	527:9	NONHSAG014811	698:305	NONHSAG054549	620:783
NONHSAG002759	172:975	NONHSAG032979	62:39	NONHSAG023281	687:209	NONHSAG014811	1009:233	NONHSAG054549	321:911
NONHSAG002759	616:665	NONHSAG032979	1020:7	NONHSAG023281	823:143	NONHSAG014811	138:477	NONHSAG054549	892:395
NONHSAG002759	76:45	NONHSAG032979	908:1005	NONHSAG023281	655:857	NONHSAG014811	144:1129	NONHSAG054549	603:687
NONHSAG002759	145:997	NONHSAG032979	912:351	NONHSAG023281	341:279	NONHSAG014811	315:3	NONHSAG054549	415:557
NONHSAG002759	1057:287	NONHSAG032984	888:753	NONHSAG023281	334:1095	NONHSAG014811	661:59	NONHSAG054549	1047:113
NONHSAG002759	1149:723	NONHSAG032984	203:1007	NONHSAG023281	880:141	NONHSAG014811	149:515	NONHSAG054549	143:1097
NONHSAG002764	444:939	NONHSAG032984	979:245	NONHSAG023281	22:377	NONHSAG014813	636:277	NONHSAG054549	692:15
NONHSAG002764	221:697	NONHSAG032984	1114:993	NONHSAG023281	676:709	NONHSAG014813	509:287	NONHSAG054549	301:113
NONHSAG002764	892:883	NONHSAG032984	535:1147	NONHSAG023281	1031:185	NONHSAG014813	562:11	NONHSAG054576	962:147
NONHSAG002764	313:895	NONHSAG032984	726:431	NONHSAG023281	383:283	NONHSAG014813	468:205	NONHSAG054576	4:443
NONHSAG002764	1098:589	NONHSAG032984	634:395	NONHSAG023286	1117:79	NONHSAG014813	954:19	NONHSAG054576	124:905
NONHSAG002764	123:643	NONHSAG032984	445:485	NONHSAG023286	1128:343	NONHSAG014813	508:337	NONHSAG054576	246:669
NONHSAG002764	1161:541	NONHSAG032984	542:887	NONHSAG023286	70:553	NONHSAG014813	273:1135	NONHSAG054576	773:985
NONHSAG002857	763:857	NONHSAG032984	197:1015	NONHSAG023286	790:993	NONHSAG014813	451:1035	NONHSAG054576	707:891
NONHSAG002857	302:585	NONHSAG032984	216:1001	NONHSAG023286	265:733	NONHSAG014829	548:1041	NONHSAG054576	538:1041
NONHSAG002857	341:1097	NONHSAG032984	855:839	NONHSAG023286	703:857	NONHSAG014829	76:141	NONHSAG054576	306:771
NONHSAG002857	347:155	NONHSAG032984	349:341	NONHSAG023286	586:1127	NONHSAG014829	1156:1071	NONHSAG054576	81:385
NONHSAG002857	922:59	NONHSAG032984	449:103	NONHSAG023298	227:1009	NONHSAG014829	76:533	NONHSAG054576	355:1073
NONHSAG002857	2:21	NONHSAG032984	898:679	NONHSAG023298	40:907	NONHSAG014829	521:847	NONHSAG054576	225:635
NONHSAG002857	800:1061	NONHSAG032984	30:915	NONHSAG023298	61:167	NONHSAG014829	82:897	NONHSAG054653	66:229
NONHSAG002857	447:53	NONHSAG032984	1073:789	NONHSAG023298	90:135	NONHSAG014829	43:393	NONHSAG054653	511:179
NONHSAG002857	499:325	NONHSAG032984	25:71	NONHSAG023298	37:927	NONHSAG014829	1077:193	NONHSAG054653	112:767
NONHSAG002857	835:719	NONHSAG032984	365:141	NONHSAG023298	532:183	NONHSAG014829	863:771	NONHSAG054653	374:555
NONHSAG002857	958:375	NONHSAG032984	1042:1001	NONHSAG023298	339:1033	NONHSAG014829	808:511	NONHSAG054653	884:491
NONHSAG002865	281:1067	NONHSAG032984	804:1115	NONHSAG023298	1082:267	NONHSAG014829	132:131	NONHSAG054653	328:811
NONHSAG002865	667:389	NONHSAG032988	615:1133	NONHSAG023298	153:315	NONHSAG014834	1092:113	NONHSAG054653	219:281
NONHSAG002865	1137:277	NONHSAG032988	717:341	NONHSAG023327	321:245	NONHSAG014834	1017:623	NONHSAG054655	344:391
NONHSAG002865	1:989	NONHSAG032988	256:979	NONHSAG023327	358:625	NONHSAG014834	531:801	NONHSAG054655	877:349
NONHSAG002865	906:361	NONHSAG032988	262:899	NONHSAG023327	530:243	NONHSAG014834	104:841	NONHSAG054655	718:481
NONHSAG002865	19:919	NONHSAG032988	793:889	NONHSAG023327	725:531	NONHSAG014834	477:837	NONHSAG054655	285:243
NONHSAG002865	495:1	NONHSAG032988	791:887	NONHSAG023327	799:567	NONHSAG014834	1051:541	NONHSAG054655	545:801
NONHSAG002865	350:929	NONHSAG032988	836:1129	NONHSAG023327	912:545	NONHSAG014834	788:679	NONHSAG054655	533:733
NONHSAG002865	947:1133	NONHSAG032988	968:461	NONHSAG023327	804:977	NONHSAG014834	1161:291	NONHSAG054655	16:301
NONHSAG002891	642:753	NONHSAG032988	199:861	NONHSAG023327	560:1145	NONHSAG014834	887:295	NONHSAG054655	640:371
NONHSAG002891	48:441	NONHSAG032988	1129:1063	NONHSAG023327	185:141	NONHSAG014834	315:291	NONHSAG054655	525:833
NONHSAG002891	865:295	NONHSAG033011	14:779	NONHSAG023327	230:909	NONHSAG014845	299:927	NONHSAG054655	181:659
NONHSAG002891	444:929	NONHSAG033011	366:463	NONHSAG023327	461:155	NONHSAG014845	114:1035	NONHSAG054664	971:1139
NONHSAG002891	547:197	NONHSAG033011	509:1035	NONHSAG023350	680:211	NONHSAG014845	454:95	NONHSAG054664	1098:519
NONHSAG002891	769:895	NONHSAG033011	455:55	NONHSAG023350	970:825	NONHSAG014845	376:671	NONHSAG054664	726:853
NONHSAG002891	488:371	NONHSAG033011	854:27	NONHSAG023350	681:617	NONHSAG014845	889:711	NONHSAG054664	381:1007
NONHSAG002891	263:705	NONHSAG033011	354:127	NONHSAG023350	1045:621	NONHSAG014845	708:27	NONHSAG054664	462:29
NONHSAG002891	336:921	NONHSAG033011	729:177	NONHSAG023350	1079:57	NONHSAG014845	21:911	NONHSAG054664	70:373
NONHSAG002891	735:781	NONHSAG033011	557:851	NONHSAG023350	955:57	NONHSAG014845	193:471	NONHSAG054664	92:513
NONHSAG002908	1122:101	NONHSAG033011	1054:49	NONHSAG023350	909:207	NONHSAG014845	36:653	NONHSAG054664	465:1075
NONHSAG002908	1139:903	NONHSAG033011	626:447	NONHSAG023350	438:5	NONHSAG014845	923:197	NONHSAG054664	877:1135
NONHSAG002908	688:423	NONHSAG033011	973:1115	NONHSAG023350	75:347	NONHSAG014845	513:551	NONHSAG054664	433:1013
NONHSAG002908	125:907	NONHSAG033026	380:445	NONHSAG023350	510:829	NONHSAG014849	449:859	NONHSAG054671	984:753
NONHSAG002908	381:217	NONHSAG033026	520:151	NONHSAG023350	590:771	NONHSAG014849	238:769	NONHSAG054671	1084:699
NONHSAG002908	566:437	NONHSAG033026	493:671	NONHSAG023373	385:965	NONHSAG014849	814:229	NONHSAG054671	563:787
NONHSAG002908	1083:757	NONHSAG033026	114:805	NONHSAG023373	1106:225	NONHSAG014849	226:789	NONHSAG054671	26:617
NONHSAG002908	1140:707	NONHSAG033026	887:1135	NONHSAG023373	838:913	NONHSAG014849	926:681	NONHSAG054671	249:741
NONHSAG002908	621:991	NONHSAG033026	267:9	NONHSAG023373	468:717	NONHSAG014849	155:251	NONHSAG054671	87:917
NONHSAG002936	201:149	NONHSAG033026	220:617	NONHSAG023373	103:405	NONHSAG014849	676:241	NONHSAG054671	1156:239
NONHSAG002936	578:125	NONHSAG033026	770:191	NONHSAG023373	736:135	NONHSAG014849	1119:857	NONHSAG054671	615:719
NONHSAG002936	758:531	NONHSAG033026	413:1127	NONHSAG023373	755:779	NONHSAG014849	1147:253	NONHSAG054671	962:1153
NONHSAG002936	390:95	NONHSAG033026	1007:75	NONHSAG023373	146:793	NONHSAG014849	246:1063	NONHSAG054671	997:777
NONHSAG002936	1145:97	NONHSAG033026	73:851	NONHSAG023373	543:579	NONHSAG014850	417:669	NONHSAG054671	685:771
NONHSAG002936	393:1005	NONHSAG033060	788:457	NONHSAG023373	234:1041	NONHSAG014850	449:1151	NONHSAG054679	959:467
NONHSAG002936	986:379	NONHSAG033060	682:881	NONHSAG023373	33:1151	NONHSAG014850	379:721	NONHSAG054679	540:453

NONHSAG002936	172:1033	NONHSAG033060	293:965	NONHSAG023390	832:501	NONHSAG014850	471:289	NONHSAG054679	819:577
NONHSAG002936	783:611	NONHSAG033060	832:587	NONHSAG023390	1144:23	NONHSAG014850	218:229	NONHSAG054679	888:171
NONHSAG002936	800:551	NONHSAG033060	470:983	NONHSAG023390	568:855	NONHSAG014850	548:201	NONHSAG054679	55:65
NONHSAG002936	1101:351	NONHSAG033060	1116:493	NONHSAG023390	1116:229	NONHSAG014898	202:1129	NONHSAG054679	288:1077
NONHSAG002956	406:885	NONHSAG033060	581:711	NONHSAG023390	232:1145	NONHSAG014898	1054:611	NONHSAG054679	296:153
NONHSAG002956	1090:705	NONHSAG033060	121:679	NONHSAG023427	258:681	NONHSAG014898	771:533	NONHSAG054679	751:1101
NONHSAG002956	278:653	NONHSAG033060	770:377	NONHSAG023427	830:141	NONHSAG014898	878:733	NONHSAG054679	522:717
NONHSAG002956	364:503	NONHSAG033060	549:173	NONHSAG023427	739:849	NONHSAG014898	585:493	NONHSAG054679	1013:707
NONHSAG002956	262:519	NONHSAG033060	979:961	NONHSAG023427	1083:871	NONHSAG014898	851:811	NONHSAG054679	703:297
NONHSAG002956	130:915	NONHSAG033068	816:569	NONHSAG023427	928:605	NONHSAG014898	542:695	NONHSAG054729	215:303
NONHSAG002956	139:269	NONHSAG033068	121:1091	NONHSAG023427	1049:591	NONHSAG014898	240:411	NONHSAG054729	1081:867
NONHSAG002956	167:379	NONHSAG033068	757:1003	NONHSAG023427	1010:927	NONHSAG014898	963:717	NONHSAG054729	998:893
NONHSAG002956	680:807	NONHSAG033068	989:943	NONHSAG023427	365:987	NONHSAG014898	806:405	NONHSAG054729	978:445
NONHSAG002956	633:693	NONHSAG033068	326:1099	NONHSAG023427	904:719	NONHSAG014905	579:247	NONHSAG054729	9:331
NONHSAG002956	837:131	NONHSAG033068	1080:639	NONHSAG023427	120:479	NONHSAG014905	1128:115	NONHSAG054729	386:623
NONHSAG002956	588:789	NONHSAG033068	57:613	NONHSAG023436	82:383	NONHSAG014905	754:223	NONHSAG054754	1000:485
NONHSAG002956	174:763	NONHSAG033068	650:983	NONHSAG023436	251:499	NONHSAG014905	233:121	NONHSAG054754	1011:511
NONHSAG002956	1056:861	NONHSAG033068	137:63	NONHSAG023436	707:259	NONHSAG014905	891:145	NONHSAG054754	981:15
NONHSAG002956	317:697	NONHSAG033068	43:411	NONHSAG023436	264:205	NONHSAG014905	87:271	NONHSAG054754	991:591
NONHSAG002956	149:601	NONHSAG033068	650:437	NONHSAG023436	241:749	NONHSAG014905	276:91	NONHSAG054754	855:949
NONHSAG002956	404:547	NONHSAG033068	67:947	NONHSAG023436	513:759	NONHSAG014905	195:969	NONHSAG054754	530:7
NONHSAG002956	88:77	NONHSAG033068	282:261	NONHSAG023436	938:1093	NONHSAG014905	169:277	NONHSAG054754	113:629
NONHSAG003018	165:647	NONHSAG033068	1131:859	NONHSAG023436	1071:557	NONHSAG014905	844:65	NONHSAG054754	1052:687
NONHSAG003018	862:1091	NONHSAG033068	778:1097	NONHSAG023436	71:93	NONHSAG014905	847:95	NONHSAG054754	141:591
NONHSAG003018	778:479	NONHSAG033068	936:533	NONHSAG023436	453:953	NONHSAG014910	647:549	NONHSAG054754	215:681
NONHSAG003018	195:641	NONHSAG033068	1066:539	NONHSAG023436	161:137	NONHSAG014910	1055:273	NONHSAG054784	449:1125
NONHSAG003018	795:903	NONHSAG033068	757:329	NONHSAG023436	768:489	NONHSAG014910	483:221	NONHSAG054784	624:217
NONHSAG003018	353:1007	NONHSAG033068	541:97	NONHSAG023436	142:1149	NONHSAG014910	739:321	NONHSAG054784	108:1041
NONHSAG003018	769:167	NONHSAG033071	841:51	NONHSAG023436	2:505	NONHSAG014910	550:1145	NONHSAG054784	33:177
NONHSAG003018	540:1111	NONHSAG033071	1090:285	NONHSAG023436	459:449	NONHSAG014910	29:419	NONHSAG054784	1086:203
NONHSAG003018	549:385	NONHSAG033071	815:1031	NONHSAG023436	139:459	NONHSAG014910	374:239	NONHSAG054784	84:235
NONHSAG003018	338:837	NONHSAG033071	370:627	NONHSAG023436	458:1021	NONHSAG014910	125:235	NONHSAG054784	875:253
NONHSAG003018	779:545	NONHSAG033071	1078:691	NONHSAG023436	955:753	NONHSAG014910	539:221	NONHSAG054784	918:209
NONHSAG003022	50:103	NONHSAG033071	518:479	NONHSAG023436	707:983	NONHSAG014910	1101:15	NONHSAG054784	572:849
NONHSAG003022	883:397	NONHSAG033071	699:743	NONHSAG023436	789:273	NONHSAG014910	295:1041	NONHSAG054784	978:797
NONHSAG003022	706:681	NONHSAG033071	643:201	NONHSAG023437	844:675	NONHSAG014910	513:739	NONHSAG054784	967:1047
NONHSAG003022	543:465	NONHSAG033071	468:773	NONHSAG023437	817:187	NONHSAG014910	442:1043	NONHSAG054784	312:663
NONHSAG003022	775:911	NONHSAG033071	910:1089	NONHSAG023437	797:205	NONHSAG014910	302:693	NONHSAG054784	626:171
NONHSAG003022	76:89	NONHSAG033071	523:717	NONHSAG023437	214:1083	NONHSAG014910	319:795	NONHSAG054784	1039:681
NONHSAG003022	247:627	NONHSAG033071	449:861	NONHSAG023437	778:347	NONHSAG014924	819:237	NONHSAG054784	220:1097
NONHSAG003022	298:161	NONHSAG033071	71:603	NONHSAG023437	854:407	NONHSAG014924	349:775	NONHSAG054784	728:345
NONHSAG003022	190:225	NONHSAG033071	382:999	NONHSAG023437	552:227	NONHSAG014924	223:465	NONHSAG054784	1127:219
NONHSAG003022	621:101	NONHSAG033071	106:695	NONHSAG023437	62:803	NONHSAG014924	487:981	NONHSAG054784	1001:133
NONHSAG003022	252:533	NONHSAG033071	918:97	NONHSAG023437	762:1083	NONHSAG014924	746:63	NONHSAG054784	968:835
NONHSAG003057	143:991	NONHSAG033071	132:341	NONHSAG023437	455:1071	NONHSAG014926	128:707	NONHSAG054784	701:597
NONHSAG003057	397:1121	NONHSAG033071	990:269	NONHSAG023456	915:169	NONHSAG014926	949:259	NONHSAG056037	790:1039
NONHSAG003057	895:151	NONHSAG033071	950:97	NONHSAG023456	349:615	NONHSAG014926	332:739	NONHSAG056037	941:653
NONHSAG003057	332:335	NONHSAG033071	936:675	NONHSAG023456	38:255	NONHSAG014926	962:207	NONHSAG056037	356:457
NONHSAG003089	531:901	NONHSAG033071	331:1151	NONHSAG023456	388:593	NONHSAG014926	956:293	NONHSAG056037	826:739
NONHSAG003089	262:877	NONHSAG033071	835:777	NONHSAG023456	749:909	NONHSAG014926	454:249	NONHSAG056037	509:449
NONHSAG003089	794:347	NONHSAG033071	232:657	NONHSAG023456	582:433	NONHSAG014926	1071:821	NONHSAG056037	1114:815
NONHSAG003089	196:371	NONHSAG033071	1104:1071	NONHSAG023456	531:1029	NONHSAG014926	900:757	NONHSAG056037	45:859
NONHSAG003089	118:301	NONHSAG033071	482:161	NONHSAG023456	506:693	NONHSAG014926	491:689	NONHSAG056037	10:307
NONHSAG003089	710:511	NONHSAG033071	537:85	NONHSAG023456	548:595	NONHSAG014926	753:759	NONHSAG056037	279:85
NONHSAG003089	1095:1047	NONHSAG033071	1065:771	NONHSAG023456	1051:121	NONHSAG014964	174:649	NONHSAG056037	124:833
NONHSAG003089	326:833	NONHSAG033071	188:437	NONHSAG023456	657:389	NONHSAG014964	692:273	NONHSAG056037	379:297
NONHSAG003089	1122:239	NONHSAG033071	568:967	NONHSAG023530	48:267	NONHSAG014964	968:825	NONHSAG056037	530:181
NONHSAG003123	619:593	NONHSAG033071	392:651	NONHSAG023530	947:285	NONHSAG014964	684:839	NONHSAG056037	934:1087
NONHSAG003123	720:705	NONHSAG033071	2:925	NONHSAG023530	450:227	NONHSAG014964	391:1151	NONHSAG056037	907:689
NONHSAG003123	795:473	NONHSAG033071	299:289	NONHSAG023530	31:1069	NONHSAG014964	560:123	NONHSAG056037	539:889
NONHSAG003123	222:489	NONHSAG033071	160:551	NONHSAG023530	430:935	NONHSAG014964	192:1131	NONHSAG056037	834:151
NONHSAG003123	1145:881	NONHSAG033071	519:815	NONHSAG023530	1064:815	NONHSAG014964	736:591	NONHSAG056037	690:93
NONHSAG003123	1053:777	NONHSAG033071	210:471	NONHSAG023530	47:113	NONHSAG014964	124:1159	NONHSAG056037	597:179
NONHSAG003123	285:845	NONHSAG033071	233:321	NONHSAG023530	985:925	NONHSAG014964	1001:559	NONHSAG056037	229:7
NONHSAG003123	941:7	NONHSAG033071	993:37	NONHSAG023530	930:865	NONHSAG015027	300:1071	NONHSAG056037	385:351
NONHSAG003123	581:283	NONHSAG033071	814:3	NONHSAG023534	668:533	NONHSAG015027	353:19	NONHSAG056037	897:1095
NONHSAG003123	988:599	NONHSAG033071	435:339	NONHSAG023534	59:27	NONHSAG015027	423:83	NONHSAG056037	606:221
NONHSAG003123	170:1017	NONHSAG033071	1150:991	NONHSAG023534	411:5	NONHSAG015027	831:687	NONHSAG056037	162:349
NONHSAG003195	827:753	NONHSAG033071	434:991	NONHSAG023534	983:203	NONHSAG015027	609:109	NONHSAG056037	305:1081
NONHSAG003195	545:793	NONHSAG033071	1034:691	NONHSAG023534	586:705	NONHSAG015027	361:1	NONHSAG056037	486:149
NONHSAG003195	184:795	NONHSAG033071	769:913	NONHSAG023534	1048:713	NONHSAG015027	200:705	NONHSAG056037	175:157
NONHSAG003195	59:81	NONHSAG033071	429:25	NONHSAG023534	643:959	NONHSAG015027	990:731	NONHSAG056037	70:945
NONHSAG003195	368:1031	NONHSAG033071	484:367	NONHSAG023534	71:27	NONHSAG015027	869:1083	NONHSAG056037	71:1049
NONHSAG003195	76:337	NONHSAG033071	754:985	NONHSAG023534	711:103	NONHSAG015035	615:227	NONHSAG056037	785:861
NONHSAG003195	868:1009	NONHSAG033071	852:643	NONHSAG023534	906:289	NONHSAG015035	1104:831	NONHSAG056037	1097:369
NONHSAG003195	1012:235	NONHSAG033071	793:591	NONHSAG023534	296:207	NONHSAG015035	439:963	NONHSAG056037	629:805
NONHSAG003195	796:837	NONHSAG033071	128:951	NONHSAG023539	652:117	NONHSAG015035	859:357	NONHSAG056037	210:713
NONHSAG003195	370:273	NONHSAG033071	225:283	NONHSAG023539	972:299	NONHSAG015035	888:1119	NONHSAG056037	429:1033
NONHSAG003195	1028:293	NONHSAG033071	485:287	NONHSAG023539	829:537	NONHSAG015035	682:975	NONHSAG056037	59:303
NONHSAG003198	1087:1031	NONHSAG033071	550:1151	NONHSAG023539	580:455	NONHSAG015035	494:81	NONHSAG054798	691:17
NONHSAG003198	1001:447	NONHSAG033071	842:151	NONHSAG023539	1098:731	NONHSAG015052	1128:857	NONHSAG054798	149:683
NONHSAG003198	849:193	NONHSAG033071	120:689	NONHSAG023539	209:1127	NONHSAG015052	733:385	NONHSAG054798	420:291
NONHSAG003198	551:1141	NONHSAG033071	984:799	NONHSAG023539	74:267	NONHSAG015052	101:923	NONHSAG054798	1120:283
NONHSAG003198	445:285	NONHSAG033071	151:629	NONHSAG023539	110:553	NONHSAG015052	1158:1077	NONHSAG054798	148:1049
NONHSAG003198	677:575	NONHSAG033071	162:791	NONHSAG023539	188:225	NONHSAG015052	997:339	NONHSAG054798	935:999
NONHSAG003198	596:673	NONHSAG033071	763:1015	NONHSAG023539	311:967	NONHSAG015052	670:903	NONHSAG054798	966:1095
NONHSAG003198	176:267	NONHSAG033071	898:1091	NONHSAG023539	307:759	NONHSAG015052	538:579	NONHSAG054798	270:979
NONHSAG003198	572:727	NONHSAG033071	361:951	NONHSAG023582	213:869	NONHSAG015052	624:57	NONHSAG054798	698:1085

NONHSAG003198	1039:249	NONHSAG033071	9:437	NONHSAG023582	960:505	NONHSAG015052	703:1041	NONHSAG054798	894:823
NONHSAG003198	330:619	NONHSAG033071	210:411	NONHSAG023582	1:1101	NONHSAG015052	180:717	NONHSAG056037	224:373
NONHSAG003198	1111:225	NONHSAG033071	854:315	NONHSAG023582	421:1157	NONHSAG015052	462:1017	NONHSAG056037	386:273
NONHSAG003198	739:295	NONHSAG033071	618:565	NONHSAG023582	664:289	NONHSAG015063	47:689	NONHSAG056037	840:95
NONHSAG003198	295:569	NONHSAG033071	151:1011	NONHSAG023582	304:153	NONHSAG015063	991:385	NONHSAG056037	898:1131
NONHSAG003198	154:269	NONHSAG033071	494:851	NONHSAG023582	583:665	NONHSAG015063	926:1005	NONHSAG056037	118:237
NONHSAG003198	854:667	NONHSAG033071	584:333	NONHSAG023582	498:325	NONHSAG015063	742:963	NONHSAG056037	41:949
NONHSAG003198	1101:95	NONHSAG033071	526:65	NONHSAG023582	199:783	NONHSAG015063	1023:1103	NONHSAG056037	1020:363
NONHSAG003198	27:829	NONHSAG033071	915:807	NONHSAG023627	801:239	NONHSAG015063	825:89	NONHSAG056037	723:519
NONHSAG003198	357:671	NONHSAG033071	413:135	NONHSAG023627	586:37	NONHSAG015063	831:1107	NONHSAG056037	437:851
NONHSAG003198	802:1071	NONHSAG033071	1138:661	NONHSAG023627	189:545	NONHSAG015063	194:745	NONHSAG056037	145:553
NONHSAG003198	976:613	NONHSAG033071	619:793	NONHSAG023627	444:627	NONHSAG015063	923:219	NONHSAG056037	914:1069
NONHSAG003202	1107:577	NONHSAG033071	368:105	NONHSAG023627	435:65	NONHSAG015063	1083:1157	NONHSAG056037	105:859
NONHSAG003202	697:365	NONHSAG033071	1157:805	NONHSAG023627	388:503	NONHSAG015063	807:731	NONHSAG056037	694:869
NONHSAG003202	11:29	NONHSAG033071	229:73	NONHSAG023627	532:1153	NONHSAG015092	690:601	NONHSAG056037	611:195
NONHSAG003202	276:507	NONHSAG033071	1015:105	NONHSAG023627	1125:213	NONHSAG015092	266:1127	NONHSAG056037	324:201
NONHSAG003202	1002:203	NONHSAG033082	1082:777	NONHSAG023627	664:1077	NONHSAG015092	557:361	NONHSAG056037	643:251
NONHSAG003202	1073:195	NONHSAG033082	794:311	NONHSAG023639	747:225	NONHSAG015092	1020:1149	NONHSAG056037	234:669
NONHSAG003202	288:203	NONHSAG033082	563:795	NONHSAG023639	31:207	NONHSAG015092	868:557	NONHSAG056037	551:1085
NONHSAG003202	384:707	NONHSAG033082	429:1051	NONHSAG023639	874:727	NONHSAG015092	474:643	NONHSAG056037	821:665
NONHSAG003261	165:403	NONHSAG033082	875:65	NONHSAG023639	30:207	NONHSAG015092	416:983	NONHSAG056037	204:1049
NONHSAG003261	31:433	NONHSAG033082	1128:967	NONHSAG023639	873:727	NONHSAG015092	1027:79	NONHSAG056037	410:1081
NONHSAG003261	512:1079	NONHSAG033082	473:183	NONHSAG023639	1100:323	NONHSAG015092	450:735	NONHSAG056037	828:171
NONHSAG003261	70:899	NONHSAG033082	369:1019	NONHSAG023639	157:869	NONHSAG015092	421:905	NONHSAG056037	615:89
NONHSAG003261	590:105	NONHSAG033082	726:191	NONHSAG023639	159:535	NONHSAG015092	233:957	NONHSAG056037	577:1097
NONHSAG003289	141:919	NONHSAG033092	418:43	NONHSAG023639	686:607	NONHSAG015143	324:535	NONHSAG056037	866:183
NONHSAG003289	1086:1047	NONHSAG033092	1119:523	NONHSAG023639	757:1093	NONHSAG015143	36:763	NONHSAG056037	37:215
NONHSAG003289	658:1019	NONHSAG033092	158:1107	NONHSAG023639	801:285	NONHSAG015143	1127:1025	NONHSAG056037	969:939
NONHSAG003289	734:809	NONHSAG033092	1137:151	NONHSAG023639	685:355	NONHSAG015143	1103:675	NONHSAG056037	671:373
NONHSAG003289	17:511	NONHSAG033092	165:1089	NONHSAG023639	756:1093	NONHSAG015143	184:1161	NONHSAG056037	223:851
NONHSAG003289	945:3	NONHSAG033092	222:1083	NONHSAG023639	685:607	NONHSAG015143	955:19	NONHSAG056037	606:461
NONHSAG003289	506:711	NONHSAG033092	696:629	NONHSAG023639	634:219	NONHSAG015143	762:419	NONHSAG056037	551:833
NONHSAG003289	73:1063	NONHSAG033092	206:191	NONHSAG023639	200:995	NONHSAG015143	711:1051	NONHSAG056037	260:247
NONHSAG003289	225:367	NONHSAG033092	314:479	NONHSAG023639	748:225	NONHSAG015143	16:243	NONHSAG056037	839:365
NONHSAG003289	986:131	NONHSAG033092	416:777	NONHSAG023639	686:355	NONHSAG015144	598:571	NONHSAG056037	688:1133
NONHSAG003322	996:491	NONHSAG033092	129:177	NONHSAG023639	664:673	NONHSAG015144	655:541	NONHSAG056037	707:169
NONHSAG003322	177:679	NONHSAG033092	421:333	NONHSAG023639	762:139	NONHSAG015144	78:527	NONHSAG056037	169:385
NONHSAG003322	1013:229	NONHSAG033092	913:915	NONHSAG023639	1099:323	NONHSAG015144	1104:897	NONHSAG056037	1050:733
NONHSAG003322	660:715	NONHSAG033092	686:661	NONHSAG023639	158:869	NONHSAG015144	876:1151	NONHSAG056037	142:263
NONHSAG003322	799:9	NONHSAG033092	21:547	NONHSAG023666	899:979	NONHSAG015144	1047:731	NONHSAG056037	24:1003
NONHSAG003322	27:1155	NONHSAG033092	248:1005	NONHSAG023666	532:269	NONHSAG015144	892:767	NONHSAG056037	435:657
NONHSAG003322	1021:361	NONHSAG033092	114:91	NONHSAG023666	882:289	NONHSAG015144	661:1115	NONHSAG056037	465:549
NONHSAG003322	559:533	NONHSAG033092	977:215	NONHSAG023666	409:161	NONHSAG015144	439:193	NONHSAG056037	901:1017
NONHSAG003322	1048:1055	NONHSAG033092	512:77	NONHSAG023666	1068:261	NONHSAG015144	99:1085	NONHSAG056037	451:1011
NONHSAG003322	980:1109	NONHSAG033092	204:1143	NONHSAG023666	984:693	NONHSAG015144	1042:263	NONHSAG056037	525:1053
NONHSAG003322	322:613	NONHSAG033092	887:969	NONHSAG023666	231:701	NONHSAG015185	114:303	NONHSAG056037	39:455
NONHSAG003325	197:205	NONHSAG033092	537:1021	NONHSAG023666	668:607	NONHSAG015185	83:155	NONHSAG056037	331:905
NONHSAG003325	276:1107	NONHSAG033099	600:47	NONHSAG023666	8:9	NONHSAG015185	1098:637	NONHSAG056037	986:739
NONHSAG003325	188:499	NONHSAG033099	966:19	NONHSAG023666	398:889	NONHSAG015185	1122:31	NONHSAG056037	827:1085
NONHSAG003325	556:125	NONHSAG033099	423:499	NONHSAG023666	665:287	NONHSAG015185	722:621	NONHSAG056037	231:591
NONHSAG003325	360:139	NONHSAG033099	777:1055	NONHSAG023691	464:587	NONHSAG015185	489:379	NONHSAG056037	949:129
NONHSAG003325	311:721	NONHSAG033099	1117:891	NONHSAG023691	691:303	NONHSAG015185	976:551	NONHSAG056037	885:419
NONHSAG003325	1013:551	NONHSAG033099	1052:275	NONHSAG023691	947:11	NONHSAG015185	414:1017	NONHSAG056037	179:677
NONHSAG003325	266:503	NONHSAG033099	1033:227	NONHSAG023691	1039:553	NONHSAG015185	75:63	NONHSAG054804	898:651
NONHSAG003325	943:871	NONHSAG033099	268:583	NONHSAG023691	790:877	NONHSAG015185	1133:1121	NONHSAG054804	47:803
NONHSAG003325	266:209	NONHSAG033099	969:37	NONHSAG023691	1134:159	NONHSAG015185	633:709	NONHSAG054804	118:957
NONHSAG003325	698:585	NONHSAG033167	1037:91	NONHSAG023691	445:87	NONHSAG015185	740:511	NONHSAG054804	1042:517
NONHSAG003332	588:975	NONHSAG033167	754:799	NONHSAG023691	950:285	NONHSAG015185	983:377	NONHSAG054804	1089:443
NONHSAG003332	640:421	NONHSAG033167	547:741	NONHSAG023691	1048:291	NONHSAG015185	296:167	NONHSAG054804	238:1065
NONHSAG003332	419:49	NONHSAG033167	902:939	NONHSAG023704	16:455	NONHSAG015185	247:273	NONHSAG054804	844:549
NONHSAG003332	564:1051	NONHSAG033167	880:53	NONHSAG023704	1047:1129	NONHSAG015185	364:795	NONHSAG054804	286:239
NONHSAG003332	351:761	NONHSAG033167	1114:1125	NONHSAG023704	681:853	NONHSAG015185	37:759	NONHSAG054804	402:1161
NONHSAG003332	1149:107	NONHSAG033167	226:693	NONHSAG023704	554:9	NONHSAG015185	1034:831	NONHSAG054804	492:895
NONHSAG003332	631:227	NONHSAG033167	244:699	NONHSAG023704	942:505	NONHSAG015185	566:787	NONHSAG054804	1142:603
NONHSAG003332	258:241	NONHSAG033167	170:291	NONHSAG023704	1133:105	NONHSAG015185	798:725	NONHSAG054869	581:659
NONHSAG003332	670:495	NONHSAG033167	243:929	NONHSAG023704	130:193	NONHSAG015185	1043:777	NONHSAG054869	1151:469
NONHSAG003332	689:531	NONHSAG033167	271:973	NONHSAG023704	717:1037	NONHSAG015185	870:1105	NONHSAG054869	837:1153
NONHSAG003332	80:609	NONHSAG033167	1007:1155	NONHSAG023704	659:595	NONHSAG015188	398:875	NONHSAG054869	387:1097
NONHSAG003339	145:1159	NONHSAG033226	74:181	NONHSAG023704	165:105	NONHSAG015188	668:923	NONHSAG054869	39:227
NONHSAG003339	269:147	NONHSAG033226	259:361	NONHSAG023704	553:809	NONHSAG015188	27:1083	NONHSAG054869	155:675
NONHSAG003339	406:515	NONHSAG033226	477:203	NONHSAG023707	404:1097	NONHSAG015188	921:751	NONHSAG054869	72:867
NONHSAG003339	819:1147	NONHSAG033226	180:257	NONHSAG023707	374:549	NONHSAG015188	304:1045	NONHSAG054869	76:1121
NONHSAG003339	778:121	NONHSAG033226	875:741	NONHSAG023707	200:677	NONHSAG015188	632:1077	NONHSAG054869	843:591
NONHSAG003339	837:25	NONHSAG033226	137:233	NONHSAG023707	809:867	NONHSAG015188	333:571	NONHSAG054870	739:631
NONHSAG003339	736:97	NONHSAG033226	1051:531	NONHSAG023707	569:61	NONHSAG015188	624:393	NONHSAG054870	465:285
NONHSAG003345	515:635	NONHSAG033226	741:1151	NONHSAG023707	963:329	NONHSAG015188	86:521	NONHSAG054870	1094:849
NONHSAG003345	335:525	NONHSAG033226	274:199	NONHSAG023707	967:1061	NONHSAG015211	397:763	NONHSAG054870	1120:1075
NONHSAG003345	529:851	NONHSAG033226	890:985	NONHSAG023707	430:807	NONHSAG015211	964:277	NONHSAG054870	636:71
NONHSAG003345	805:203	NONHSAG033226	498:695	NONHSAG023707	711:251	NONHSAG015211	394:339	NONHSAG054870	364:247
NONHSAG003345	280:485	NONHSAG033228	11:585	NONHSAG023707	401:369	NONHSAG015211	798:991	NONHSAG054870	346:5
NONHSAG003405	959:679	NONHSAG033228	56:471	NONHSAG023707	557:579	NONHSAG015211	510:713	NONHSAG054870	558:739
NONHSAG003405	153:567	NONHSAG033228	763:821	NONHSAG023711	6:23	NONHSAG015211	1132:747	NONHSAG054870	552:597
NONHSAG003405	643:259	NONHSAG033228	113:871	NONHSAG023711	127:929	NONHSAG015211	1032:1129	NONHSAG054897	785:465
NONHSAG003405	489:29	NONHSAG033228	551:379	NONHSAG023711	705:603	NONHSAG015211	405:47	NONHSAG054897	625:733
NONHSAG003405	981:267	NONHSAG033228	272:67	NONHSAG023711	967:1023	NONHSAG015211	928:669	NONHSAG054897	1055:1029
NONHSAG003405	254:243	NONHSAG033247	992:239	NONHSAG023711	843:543	NONHSAG015211	310:1089	NONHSAG054897	705:633
NONHSAG003405	506:275	NONHSAG033247	234:91	NONHSAG023711	903:123	NONHSAG015211	1057:977	NONHSAG054897	362:985
NONHSAG003405	395:29	NONHSAG033247	391:511	NONHSAG023711	853:309	NONHSAG015231	829:647	NONHSAG054897	603:313

NONHSAG003405	33:549	NONHSAG033247	993:239	NONHSAG023711	275:1111	NONHSAG015231	677:771	NONHSAG054897	716:189
NONHSAG003407	917:429	NONHSAG033247	769:421	NONHSAG023711	608:583	NONHSAG015231	400:1067	NONHSAG054897	271:903
NONHSAG003407	797:293	NONHSAG033247	498:809	NONHSAG023711	1011:631	NONHSAG015231	1004:1129	NONHSAG054897	907:229
NONHSAG003407	831:511	NONHSAG033247	916:139	NONHSAG023711	213:759	NONHSAG015231	640:829	NONHSAG054897	340:105
NONHSAG003407	979:463	NONHSAG033247	10:1019	NONHSAG023714	340:1137	NONHSAG015231	98:977	NONHSAG054897	726:217
NONHSAG003407	541:779	NONHSAG033247	235:91	NONHSAG023714	185:451	NONHSAG015231	480:837	NONHSAG054929	639:25
NONHSAG003407	497:581	NONHSAG033247	209:183	NONHSAG023714	833:925	NONHSAG015231	593:765	NONHSAG054929	1047:761
NONHSAG003407	49:941	NONHSAG033247	885:343	NONHSAG023714	357:67	NONHSAG015231	554:167	NONHSAG054929	898:753
NONHSAG003407	596:81	NONHSAG033247	768:421	NONHSAG023714	994:893	NONHSAG015231	895:697	NONHSAG054929	386:863
NONHSAG003407	494:467	NONHSAG033247	709:891	NONHSAG023714	411:9	NONHSAG015231	509:173	NONHSAG054929	186:253
NONHSAG003407	57:145	NONHSAG033247	9:1019	NONHSAG023714	1101:589	NONHSAG015256	385:1089	NONHSAG054929	997:561
NONHSAG003412	294:593	NONHSAG033247	499:809	NONHSAG023714	996:139	NONHSAG015256	600:1151	NONHSAG054929	97:253
NONHSAG003412	357:871	NONHSAG033247	710:891	NONHSAG023714	72:827	NONHSAG015256	380:933	NONHSAG054929	771:615
NONHSAG003412	818:199	NONHSAG033247	917:139	NONHSAG023714	496:799	NONHSAG015256	458:643	NONHSAG054929	1056:865
NONHSAG003412	556:1045	NONHSAG033247	1034:319	NONHSAG023714	857:605	NONHSAG015256	599:687	NONHSAG054929	1104:803
NONHSAG003412	261:293	NONHSAG033247	746:479	NONHSAG023715	996:17	NONHSAG015256	676:603	NONHSAG054954	151:769
NONHSAG003412	994:501	NONHSAG033247	856:293	NONHSAG023715	1075:75	NONHSAG015256	788:925	NONHSAG054954	1110:29
NONHSAG003412	430:753	NONHSAG033247	816:47	NONHSAG023715	189:777	NONHSAG015256	751:657	NONHSAG054954	71:723
NONHSAG003412	707:1089	NONHSAG033254	126:871	NONHSAG023715	454:1083	NONHSAG015256	607:537	NONHSAG054954	709:1063
NONHSAG003412	591:733	NONHSAG033254	880:43	NONHSAG023715	1012:889	NONHSAG015256	303:249	NONHSAG054954	498:1127
NONHSAG003412	439:817	NONHSAG033254	126:459	NONHSAG023715	671:827	NONHSAG015256	300:67	NONHSAG054954	538:75
NONHSAG003412	116:743	NONHSAG033254	294:63	NONHSAG023715	961:233	NONHSAG015273	367:127	NONHSAG054954	1126:191
NONHSAG003464	19:295	NONHSAG033260	943:835	NONHSAG023715	983:929	NONHSAG015273	1113:1071	NONHSAG054954	177:597
NONHSAG003464	1113:791	NONHSAG033260	885:259	NONHSAG023715	657:1045	NONHSAG015273	730:575	NONHSAG054954	391:115
NONHSAG003464	700:653	NONHSAG033260	960:263	NONHSAG023715	929:1069	NONHSAG015273	104:281	NONHSAG054954	978:135
NONHSAG003464	267:769	NONHSAG033260	661:957	NONHSAG023715	113:605	NONHSAG015273	645:11	NONHSAG054958	1107:289
NONHSAG003464	532:407	NONHSAG033260	309:565	NONHSAG023765	1073:307	NONHSAG015273	369:369	NONHSAG054958	913:1161
NONHSAG003464	1:323	NONHSAG033260	644:1083	NONHSAG023765	412:369	NONHSAG015273	758:1115	NONHSAG054958	830:545
NONHSAG003464	670:373	NONHSAG033260	105:511	NONHSAG023765	607:311	NONHSAG015273	224:1107	NONHSAG054958	253:381
NONHSAG003464	559:295	NONHSAG033260	884:185	NONHSAG023765	238:221	NONHSAG015273	448:153	NONHSAG054958	398:917
NONHSAG003464	506:957	NONHSAG033260	661:1101	NONHSAG023765	50:177	NONHSAG015273	318:573	NONHSAG054958	285:181
NONHSAG003464	1090:677	NONHSAG033260	570:153	NONHSAG023765	1110:371	NONHSAG015273	706:285	NONHSAG054958	1115:751
NONHSAG003464	193:659	NONHSAG033260	749:227	NONHSAG023765	979:87	NONHSAG015286	508:175	NONHSAG054958	550:85
NONHSAG003486	438:867	NONHSAG033260	78:1015	NONHSAG023765	257:261	NONHSAG015286	24:195	NONHSAG054958	427:975
NONHSAG003486	505:1033	NONHSAG033260	412:965	NONHSAG023765	367:741	NONHSAG015286	974:561	NONHSAG054958	648:1143
NONHSAG003486	1081:543	NONHSAG033274	713:869	NONHSAG023765	782:651	NONHSAG015286	145:85	NONHSAG054958	1010:1013
NONHSAG003486	498:27	NONHSAG033274	505:269	NONHSAG023770	972:969	NONHSAG015286	905:847	NONHSAG054963	925:773
NONHSAG003486	775:469	NONHSAG033274	518:727	NONHSAG023770	1147:1069	NONHSAG015286	327:201	NONHSAG054963	736:1109
NONHSAG003486	126:1037	NONHSAG033274	406:553	NONHSAG023770	673:177	NONHSAG015286	228:731	NONHSAG054963	648:481
NONHSAG003486	261:119	NONHSAG033274	715:509	NONHSAG023770	15:835	NONHSAG015286	762:825	NONHSAG054963	399:645
NONHSAG003486	971:427	NONHSAG033274	131:841	NONHSAG023770	472:907	NONHSAG015286	237:1065	NONHSAG054963	234:847
NONHSAG003486	1161:827	NONHSAG033274	20:241	NONHSAG023770	6:1113	NONHSAG015320	598:299	NONHSAG054963	737:1109
NONHSAG003486	1149:507	NONHSAG033274	402:437	NONHSAG023770	569:785	NONHSAG015320	1004:5	NONHSAG054963	497:277
NONHSAG003512	1094:779	NONHSAG033274	1022:931	NONHSAG023770	555:1103	NONHSAG015320	382:1101	NONHSAG054963	715:321
NONHSAG003512	533:1049	NONHSAG033291	854:1161	NONHSAG023770	617:53	NONHSAG015320	189:503	NONHSAG054963	716:321
NONHSAG003512	350:195	NONHSAG033291	17:915	NONHSAG023770	1130:663	NONHSAG015320	605:771	NONHSAG054963	649:481
NONHSAG003512	811:743	NONHSAG033291	634:791	NONHSAG023770	653:289	NONHSAG015320	901:525	NONHSAG054963	282:1
NONHSAG003533	843:67	NONHSAG033291	440:807	NONHSAG023771	1041:69	NONHSAG015320	869:53	NONHSAG054963	400:645
NONHSAG003533	410:193	NONHSAG033291	173:967	NONHSAG023771	367:657	NONHSAG015320	1068:751	NONHSAG054963	1005:737
NONHSAG003533	1038:587	NONHSAG033291	85:135	NONHSAG023771	1161:171	NONHSAG015320	47:591	NONHSAG054963	832:1137
NONHSAG003533	1061:747	NONHSAG033291	73:3	NONHSAG023771	12:743	NONHSAG015320	800:243	NONHSAG054963	496:277
NONHSAG003533	1070:675	NONHSAG033405	584:659	NONHSAG023771	434:321	NONHSAG015320	621:699	NONHSAG054963	1006:737
NONHSAG003533	504:735	NONHSAG033405	722:393	NONHSAG023771	572:251	NONHSAG015338	846:883	NONHSAG054963	833:1137
NONHSAG003533	921:595	NONHSAG033405	539:87	NONHSAG023771	1110:737	NONHSAG015338	309:617	NONHSAG054963	233:847
NONHSAG003533	232:1105	NONHSAG033405	932:933	NONHSAG023771	1085:817	NONHSAG015338	372:353	NONHSAG054963	18:1041
NONHSAG003533	406:551	NONHSAG033405	764:1053	NONHSAG023771	647:535	NONHSAG015338	24:851	NONHSAG055008	1079:827
NONHSAG003533	35:555	NONHSAG033405	1085:125	NONHSAG023772	500:1135	NONHSAG015338	751:605	NONHSAG055008	403:61
NONHSAG003533	833:1119	NONHSAG033405	449:967	NONHSAG023772	114:807	NONHSAG015338	620:397	NONHSAG055008	424:1103
NONHSAG003536	280:973	NONHSAG033405	635:913	NONHSAG023772	124:1115	NONHSAG015338	1131:835	NONHSAG055008	160:17
NONHSAG003536	221:353	NONHSAG033405	234:643	NONHSAG023772	278:85	NONHSAG015338	1116:657	NONHSAG055008	481:289
NONHSAG003536	836:207	NONHSAG033405	376:957	NONHSAG023772	892:259	NONHSAG015338	1099:449	NONHSAG055008	991:685
NONHSAG003536	760:617	NONHSAG033405	790:527	NONHSAG023772	453:267	NONHSAG015357	222:173	NONHSAG055008	179:1055
NONHSAG003536	161:959	NONHSAG033405	392:509	NONHSAG023772	818:15	NONHSAG015357	633:607	NONHSAG055008	1143:1107
NONHSAG003536	878:245	NONHSAG033405	238:865	NONHSAG023772	545:729	NONHSAG015357	973:713	NONHSAG055008	1087:879
NONHSAG003536	740:85	NONHSAG033405	885:347	NONHSAG023772	86:353	NONHSAG015357	554:1011	NONHSAG055008	1021:717
NONHSAG003536	304:259	NONHSAG033405	110:1065	NONHSAG023772	991:361	NONHSAG015357	1120:1127	NONHSAG055008	448:453
NONHSAG003536	278:869	NONHSAG033405	218:755	NONHSAG023787	957:563	NONHSAG015357	243:585	NONHSAG055019	958:689
NONHSAG003536	1053:559	NONHSAG033405	316:1155	NONHSAG023787	909:141	NONHSAG015357	129:563	NONHSAG055019	899:587
NONHSAG003539	1021:285	NONHSAG033405	361:731	NONHSAG023787	1082:959	NONHSAG015357	23:249	NONHSAG055019	1039:347
NONHSAG003539	990:277	NONHSAG033416	891:369	NONHSAG023787	1108:585	NONHSAG015357	281:981	NONHSAG055019	734:1037
NONHSAG003539	867:1119	NONHSAG033416	573:1001	NONHSAG023787	561:45	NONHSAG015357	957:751	NONHSAG055019	557:267
NONHSAG003539	761:355	NONHSAG033416	303:813	NONHSAG023787	708:93	NONHSAG015357	343:567	NONHSAG055019	357:187
NONHSAG003539	571:675	NONHSAG033416	459:865	NONHSAG023787	222:1105	NONHSAG015371	904:263	NONHSAG055019	716:593
NONHSAG003539	180:307	NONHSAG033416	304:813	NONHSAG023787	569:845	NONHSAG015371	499:867	NONHSAG055019	18:767
NONHSAG003539	558:905	NONHSAG033416	340:431	NONHSAG023787	764:459	NONHSAG015371	769:473	NONHSAG055019	66:1005
NONHSAG003539	871:397	NONHSAG033416	626:1141	NONHSAG023787	892:501	NONHSAG015371	1067:579	NONHSAG055019	1019:587
NONHSAG003539	174:289	NONHSAG033416	340:495	NONHSAG023787	1135:537	NONHSAG015371	794:179	NONHSAG055019	387:769
NONHSAG003539	792:95	NONHSAG033416	336:861	NONHSAG023787	563:91	NONHSAG015371	500:407	NONHSAG055008	22:443
NONHSAG003539	1142:473	NONHSAG033416	698:279	NONHSAG023787	921:305	NONHSAG015371	910:17	NONHSAG055008	321:1009
NONHSAG003554	160:973	NONHSAG033416	1042:483	NONHSAG023787	711:291	NONHSAG015371	555:93	NONHSAG055008	168:145
NONHSAG003554	609:433	NONHSAG033416	641:923	NONHSAG023787	174:843	NONHSAG015371	406:591	NONHSAG055008	907:541
NONHSAG003554	404:177	NONHSAG033416	572:947	NONHSAG023787	218:797	NONHSAG015371	885:1151	NONHSAG055008	519:681
NONHSAG003554	1156:289	NONHSAG033416	843:497	NONHSAG023787	1026:603	NONHSAG015371	364:425	NONHSAG055008	149:1117
NONHSAG003554	703:717	NONHSAG033484	144:45	NONHSAG023787	192:791	NONHSAG015373	943:629	NONHSAG055008	691:703
NONHSAG003554	829:1069	NONHSAG033484	1077:599	NONHSAG023787	221:711	NONHSAG015373	800:617	NONHSAG055008	701:911
NONHSAG003554	698:739	NONHSAG033484	1131:755	NONHSAG023787	627:809	NONHSAG015373	679:841	NONHSAG055008	853:1119
NONHSAG003554	905:149	NONHSAG033484	195:645	NONHSAG023787	963:901	NONHSAG015373	1056:331	NONHSAG055008	60:607
NONHSAG003554	54:785	NONHSAG033484	685:1133	NONHSAG023787	1124:991	NONHSAG015373	269:725	NONHSAG055021	426:65

NONHSAG003554	348:747	NONHSAG033484	301:125	NONHSAG023790	105:513	NONHSAG015373	179:1009	NONHSAG055021	898:1055
NONHSAG003554	437:1043	NONHSAG033484	1018:775	NONHSAG023790	185:829	NONHSAG015373	392:475	NONHSAG055021	566:393
NONHSAG003554	475:155	NONHSAG033484	531:781	NONHSAG023790	68:825	NONHSAG015387	1065:997	NONHSAG055021	467:561
NONHSAG003554	562:1147	NONHSAG033484	441:807	NONHSAG023790	594:1115	NONHSAG015387	84:453	NONHSAG055021	108:947
NONHSAG003554	804:1095	NONHSAG033484	1051:817	NONHSAG023790	360:1133	NONHSAG015387	590:347	NONHSAG055021	723:1053
NONHSAG003554	601:91	NONHSAG033484	83:343	NONHSAG023790	193:609	NONHSAG015387	252:277	NONHSAG055021	627:581
NONHSAG003554	1071:267	NONHSAG033545	41:139	NONHSAG023790	1106:147	NONHSAG015387	228:709	NONHSAG055021	155:15
NONHSAG003554	881:621	NONHSAG033545	136:1145	NONHSAG023790	339:833	NONHSAG015387	384:159	NONHSAG055021	193:1105
NONHSAG003554	1059:871	NONHSAG033545	709:293	NONHSAG023790	1029:467	NONHSAG015387	806:313	NONHSAG055021	741:513
NONHSAG003554	628:475	NONHSAG033545	1043:1029	NONHSAG023790	156:243	NONHSAG015387	493:535	NONHSAG055021	1100:849
NONHSAG003554	267:29	NONHSAG033545	663:173	NONHSAG023790	671:281	NONHSAG015387	756:1037	NONHSAG055021	750:179
NONHSAG003554	1159:829	NONHSAG033550	933:519	NONHSAG023801	1117:499	NONHSAG015387	13:107	NONHSAG055021	153:583
NONHSAG003554	413:1063	NONHSAG033550	58:611	NONHSAG023801	1089:81	NONHSAG015387	383:507	NONHSAG055021	1132:425
NONHSAG003555	445:1093	NONHSAG033550	360:1089	NONHSAG023801	695:143	NONHSAG015435	542:427	NONHSAG055021	172:423
NONHSAG003555	199:227	NONHSAG033550	1001:631	NONHSAG023801	822:291	NONHSAG015435	99:933	NONHSAG055021	184:351
NONHSAG003555	163:1033	NONHSAG033550	92:575	NONHSAG023801	153:125	NONHSAG015435	1154:1141	NONHSAG055021	1129:323
NONHSAG003555	1083:237	NONHSAG033550	901:203	NONHSAG023801	724:93	NONHSAG015435	331:1071	NONHSAG055021	935:1119
NONHSAG003555	946:1055	NONHSAG033550	713:91	NONHSAG023801	56:1075	NONHSAG015435	591:473	NONHSAG055021	149:965
NONHSAG003555	159:577	NONHSAG033550	1076:527	NONHSAG023801	363:865	NONHSAG015435	1146:955	NONHSAG055021	613:199
NONHSAG003562	247:293	NONHSAG033550	634:629	NONHSAG023801	1145:409	NONHSAG015435	180:337	NONHSAG055021	1002:383
NONHSAG003562	356:867	NONHSAG033550	1136:983	NONHSAG023801	741:473	NONHSAG015435	210:801	NONHSAG055021	718:819
NONHSAG003562	1135:1097	NONHSAG033550	796:461	NONHSAG023801	636:971	NONHSAG015435	168:475	NONHSAG055021	859:291
NONHSAG003562	814:121	NONHSAG033553	532:1005	NONHSAG023810	98:631	NONHSAG015435	90:949	NONHSAG055021	173:929
NONHSAG003562	690:929	NONHSAG033553	175:1105	NONHSAG023810	704:251	NONHSAG015435	770:651	NONHSAG055021	542:733
NONHSAG003562	516:913	NONHSAG033553	765:525	NONHSAG023810	409:311	NONHSAG015438	920:273	NONHSAG055021	209:961
NONHSAG003562	494:809	NONHSAG033553	427:1051	NONHSAG023810	536:499	NONHSAG015438	1104:101	NONHSAG055021	1124:863
NONHSAG003562	653:451	NONHSAG033553	101:555	NONHSAG023810	1119:575	NONHSAG015438	364:303	NONHSAG055021	732:107
NONHSAG003562	396:1	NONHSAG033553	245:661	NONHSAG023810	752:1007	NONHSAG015438	71:903	NONHSAG055021	852:611
NONHSAG003567	738:607	NONHSAG033553	1144:743	NONHSAG023810	920:675	NONHSAG015438	1109:463	NONHSAG055021	15:225
NONHSAG003567	585:717	NONHSAG033553	11:549	NONHSAG023839	666:635	NONHSAG015438	1006:683	NONHSAG055021	517:81
NONHSAG003567	906:243	NONHSAG033553	112:939	NONHSAG023839	1048:985	NONHSAG015438	1146:701	NONHSAG055021	784:359
NONHSAG003567	1121:593	NONHSAG033553	14:1105	NONHSAG023839	931:1123	NONHSAG015438	1161:301	NONHSAG055021	20:475
NONHSAG003567	769:585	NONHSAG033553	143:667	NONHSAG023839	1057:739	NONHSAG015438	794:403	NONHSAG055021	1071:451
NONHSAG003567	221:275	NONHSAG033553	157:47	NONHSAG023839	917:971	NONHSAG015438	1078:371	NONHSAG055021	410:1129
NONHSAG003567	123:921	NONHSAG033553	621:165	NONHSAG023839	489:251	NONHSAG015438	803:27	NONHSAG055021	122:809
NONHSAG003567	411:1093	NONHSAG033553	750:571	NONHSAG023839	718:201	NONHSAG015450	404:843	NONHSAG055021	72:1115
NONHSAG003567	851:407	NONHSAG033553	572:699	NONHSAG023839	979:143	NONHSAG015450	713:687	NONHSAG055021	271:877
NONHSAG003567	1101:1077	NONHSAG033558	758:553	NONHSAG023839	957:17	NONHSAG015450	135:615	NONHSAG055021	510:315
NONHSAG003567	462:489	NONHSAG033558	250:693	NONHSAG023839	704:1067	NONHSAG015450	638:1097	NONHSAG055021	944:621
NONHSAG003567	607:807	NONHSAG033558	340:251	NONHSAG023839	647:1157	NONHSAG015450	831:633	NONHSAG055050	144:273
NONHSAG003567	588:25	NONHSAG033558	158:253	NONHSAG023915	509:53	NONHSAG015450	1039:817	NONHSAG055050	183:17
NONHSAG003567	332:61	NONHSAG033585	1084:915	NONHSAG023915	927:925	NONHSAG015450	141:221	NONHSAG055050	602:1097
NONHSAG003567	987:173	NONHSAG033585	1119:893	NONHSAG023915	59:279	NONHSAG015450	508:209	NONHSAG055050	607:29
NONHSAG003567	333:13	NONHSAG033585	242:485	NONHSAG023915	21:63	NONHSAG015450	573:867	NONHSAG055050	516:693
NONHSAG003567	977:201	NONHSAG033585	276:5	NONHSAG023915	229:841	NONHSAG015450	492:305	NONHSAG055050	783:481
NONHSAG003567	169:735	NONHSAG033585	627:609	NONHSAG023915	401:375	NONHSAG015450	156:735	NONHSAG055050	819:751
NONHSAG003567	224:949	NONHSAG033585	847:1061	NONHSAG023915	28:711	NONHSAG015466	848:1145	NONHSAG055050	863:1053
NONHSAG003567	518:195	NONHSAG033585	132:797	NONHSAG023915	179:843	NONHSAG015466	1095:1071	NONHSAG055050	1002:1127
NONHSAG003567	800:313	NONHSAG033585	523:811	NONHSAG023915	694:245	NONHSAG015466	584:787	NONHSAG055050	752:461
NONHSAG003567	314:127	NONHSAG033585	745:745	NONHSAG023915	1105:279	NONHSAG015466	1059:317	NONHSAG055086	618:35
NONHSAG003604	1078:443	NONHSAG033585	821:979	NONHSAG023915	393:1139	NONHSAG015466	722:135	NONHSAG055086	2:1121
NONHSAG003604	124:415	NONHSAG033585	644:1035	NONHSAG023915	197:615	NONHSAG015466	459:53	NONHSAG055086	319:1019
NONHSAG003604	29:813	NONHSAG033585	982:577	NONHSAG023915	760:731	NONHSAG015466	897:981	NONHSAG055086	565:217
NONHSAG003604	516:293	NONHSAG033585	489:709	NONHSAG023915	819:25	NONHSAG015466	917:271	NONHSAG055086	1072:801
NONHSAG003604	96:1137	NONHSAG033585	815:171	NONHSAG023915	432:707	NONHSAG015466	500:613	NONHSAG055086	525:25
NONHSAG003604	201:209	NONHSAG033585	961:1095	NONHSAG023915	1152:655	NONHSAG015466	617:781	NONHSAG055086	446:1159
NONHSAG003604	489:745	NONHSAG033585	877:781	NONHSAG023915	305:527	NONHSAG015466	1154:111	NONHSAG055086	744:737
NONHSAG003604	408:401	NONHSAG033585	326:811	NONHSAG023915	552:925	NONHSAG015491	975:343	NONHSAG055086	703:589
NONHSAG003604	432:795	NONHSAG033585	897:561	NONHSAG023915	283:595	NONHSAG015491	102:787	NONHSAG055086	152:23
NONHSAG003604	6:411	NONHSAG033585	511:929	NONHSAG023915	294:469	NONHSAG015491	663:347	NONHSAG055086	434:217
NONHSAG003613	922:331	NONHSAG033585	213:69	NONHSAG023920	1111:53	NONHSAG015491	1123:483	NONHSAG055105	840:751
NONHSAG003613	84:829	NONHSAG033585	378:555	NONHSAG023920	792:1089	NONHSAG015491	657:157	NONHSAG055105	465:711
NONHSAG003613	627:733	NONHSAG033585	553:1075	NONHSAG023920	404:267	NONHSAG015491	892:169	NONHSAG055105	558:463
NONHSAG003613	539:665	NONHSAG033587	762:15	NONHSAG023920	677:233	NONHSAG015491	602:503	NONHSAG055105	1147:839
NONHSAG003613	840:289	NONHSAG033587	144:447	NONHSAG023920	810:263	NONHSAG015511	451:1115	NONHSAG055105	586:273
NONHSAG003613	525:745	NONHSAG033587	201:701	NONHSAG023920	654:1011	NONHSAG015511	865:711	NONHSAG055105	929:137
NONHSAG003613	166:371	NONHSAG033587	112:629	NONHSAG023920	1131:485	NONHSAG015511	511:369	NONHSAG055105	876:835
NONHSAG003613	153:35	NONHSAG033587	659:113	NONHSAG023920	1070:911	NONHSAG015511	316:513	NONHSAG055105	254:1037
NONHSAG003613	626:451	NONHSAG033587	1077:25	NONHSAG023920	575:107	NONHSAG015511	832:1157	NONHSAG055105	1072:1117
NONHSAG003613	1116:151	NONHSAG033587	730:485	NONHSAG023920	515:379	NONHSAG015511	680:133	NONHSAG055105	505:275
NONHSAG003615	70:245	NONHSAG033587	1156:1097	NONHSAG023920	39:1051	NONHSAG015511	436:691	NONHSAG055105	957:1033
NONHSAG003615	332:777	NONHSAG033587	596:537	NONHSAG023920	556:1133	NONHSAG015535	917:581	NONHSAG055105	315:787
NONHSAG003615	817:711	NONHSAG033587	687:703	NONHSAG023920	219:1139	NONHSAG015535	386:591	NONHSAG055105	472:1107
NONHSAG003615	156:1019	NONHSAG033587	400:1101	NONHSAG023920	499:359	NONHSAG015535	274:463	NONHSAG055105	58:909
NONHSAG003615	90:459	NONHSAG033587	11:71	NONHSAG023920	54:553	NONHSAG015535	145:1057	NONHSAG055105	758:1077
NONHSAG003615	903:1117	NONHSAG033587	40:945	NONHSAG023920	1072:1083	NONHSAG015535	167:277	NONHSAG055105	1011:193
NONHSAG003615	329:673	NONHSAG033587	394:479	NONHSAG023920	261:869	NONHSAG015535	985:621	NONHSAG055105	993:285
NONHSAG003615	468:953	NONHSAG033587	25:1025	NONHSAG023920	211:487	NONHSAG015535	980:371	NONHSAG055105	192:1075
NONHSAG003615	152:201	NONHSAG033587	904:911	NONHSAG023923	456:163	NONHSAG015535	261:247	NONHSAG055105	679:925
NONHSAG003615	573:1145	NONHSAG033587	58:643	NONHSAG023923	534:251	NONHSAG015535	925:1049	NONHSAG055105	1041:563
NONHSAG003615	976:277	NONHSAG033587	376:533	NONHSAG023923	1132:919	NONHSAG015535	805:595	NONHSAG055105	1015:823
NONHSAG003627	117:1049	NONHSAG033587	1011:229	NONHSAG023923	954:237	NONHSAG015539	803:1109	NONHSAG055108	62:537
NONHSAG003627	22:657	NONHSAG033587	142:1123	NONHSAG023923	543:395	NONHSAG015539	1046:959	NONHSAG055108	635:955
NONHSAG003627	898:1063	NONHSAG033587	72:1043	NONHSAG023923	650:191	NONHSAG015539	84:693	NONHSAG055108	502:201
NONHSAG003627	272:255	NONHSAG033587	1127:675	NONHSAG023923	1126:209	NONHSAG015539	258:715	NONHSAG055108	591:837
NONHSAG003627	10:161	NONHSAG033587	926:677	NONHSAG023954	911:371	NONHSAG015555	1056:401	NONHSAG055108	179:901
NONHSAG003627	336:851	NONHSAG033587	728:649	NONHSAG023954	359:1021	NONHSAG015555	1060:321	NONHSAG055108	731:111
NONHSAG003627	1023:1151	NONHSAG033587	178:545	NONHSAG023954	64:409	NONHSAG015555	864:115	NONHSAG055108	224:595

NONHSAG003627	973:149	NONHSAG033587	136:663	NONHSAG023954	499:509	NONHSAG015555	1120:843	NONHSAG055108	338:927
NONHSAG003627	341:223	NONHSAG033587	19:59	NONHSAG023954	422:591	NONHSAG015555	996:943	NONHSAG055108	632:115
NONHSAG003627	1063:717	NONHSAG033587	41:709	NONHSAG023954	1107:945	NONHSAG015654	1062:1009	NONHSAG055108	135:467
NONHSAG003627	549:505	NONHSAG033587	920:555	NONHSAG023954	1155:195	NONHSAG015654	1040:395	NONHSAG055108	124:421
NONHSAG003635	585:87	NONHSAG033587	80:651	NONHSAG023954	1070:491	NONHSAG015654	43:175	NONHSAG055153	411:425
NONHSAG003635	157:1017	NONHSAG033587	514:653	NONHSAG023954	1038:219	NONHSAG015654	159:379	NONHSAG055153	366:183
NONHSAG003635	622:871	NONHSAG033587	913:477	NONHSAG023954	393:443	NONHSAG015654	317:235	NONHSAG055153	900:759
NONHSAG003635	481:555	NONHSAG033587	397:303	NONHSAG023954	278:1073	NONHSAG015654	882:745	NONHSAG055153	146:521
NONHSAG003635	967:331	NONHSAG033587	83:959	NONHSAG023966	945:1127	NONHSAG015654	879:507	NONHSAG055153	1040:921
NONHSAG003635	965:453	NONHSAG033587	138:91	NONHSAG023966	80:657	NONHSAG015654	589:711	NONHSAG055153	748:1109
NONHSAG003635	298:7	NONHSAG033587	385:333	NONHSAG023966	297:963	NONHSAG015654	1125:201	NONHSAG055153	993:643
NONHSAG003635	937:263	NONHSAG033587	234:449	NONHSAG023966	1096:969	NONHSAG015654	953:527	NONHSAG055153	11:773
NONHSAG003635	544:1075	NONHSAG033587	578:1143	NONHSAG023966	1039:189	NONHSAG015654	365:1025	NONHSAG018457	490:667
NONHSAG003635	428:201	NONHSAG033587	704:1049	NONHSAG023966	894:699	NONHSAG015654	398:581	NONHSAG051638	845:717
NONHSAG003635	405:891	NONHSAG033587	306:619	NONHSAG023966	113:341	NONHSAG015654	414:731	NONHSAG051638	720:871
NONHSAG003635	951:717	NONHSAG033587	96:793	NONHSAG023966	727:75	NONHSAG015654	991:1103	NONHSAG051638	1104:719
NONHSAG003635	672:469	NONHSAG033587	656:205	NONHSAG023966	789:945	NONHSAG015654	426:107	NONHSAG051638	279:247
NONHSAG003635	850:95	NONHSAG033587	27:1137	NONHSAG023966	567:427	NONHSAG015654	234:487	NONHSAG051638	168:1127
NONHSAG003645	625:121	NONHSAG033587	859:853	NONHSAG023966	287:119	NONHSAG015654	556:665	NONHSAG051650	666:1155
NONHSAG003645	481:573	NONHSAG033587	283:957	NONHSAG023972	780:1155	NONHSAG015654	419:1055	NONHSAG051650	1020:677
NONHSAG003645	986:917	NONHSAG033587	208:51	NONHSAG023972	687:401	NONHSAG015654	445:941	NONHSAG051650	9:511
NONHSAG003645	1061:777	NONHSAG033587	103:509	NONHSAG023972	565:881	NONHSAG015654	271:275	NONHSAG051650	1125:1123
NONHSAG003645	638:587	NONHSAG033587	33:815	NONHSAG023972	491:297	NONHSAG015660	550:261	NONHSAG051650	1121:21
NONHSAG003645	1017:793	NONHSAG033587	973:397	NONHSAG023972	585:59	NONHSAG015660	1121:761	NONHSAG051650	674:579
NONHSAG003645	431:501	NONHSAG033587	752:507	NONHSAG023972	159:987	NONHSAG015660	220:821	NONHSAG051650	667:585
NONHSAG003645	1031:213	NONHSAG033587	357:1103	NONHSAG023972	465:275	NONHSAG015660	153:669	NONHSAG051650	1148:241
NONHSAG003645	571:525	NONHSAG033587	443:771	NONHSAG023972	5:183	NONHSAG015660	815:853	NONHSAG051650	797:1051
NONHSAG003645	731:737	NONHSAG033587	305:765	NONHSAG023972	189:567	NONHSAG015660	348:349	NONHSAG051650	1042:319
NONHSAG003645	843:203	NONHSAG033587	1070:147	NONHSAG024013	237:71	NONHSAG015660	535:119	NONHSAG051650	766:221
NONHSAG003645	856:861	NONHSAG033587	978:737	NONHSAG024013	754:161	NONHSAG015660	1134:1137	NONHSAG051658	861:527
NONHSAG003645	461:957	NONHSAG033587	169:809	NONHSAG024013	769:459	NONHSAG015660	253:835	NONHSAG051658	420:825
NONHSAG003645	168:225	NONHSAG033587	459:23	NONHSAG024013	870:375	NONHSAG015660	1091:921	NONHSAG051658	77:397
NONHSAG003645	268:275	NONHSAG033588	52:999	NONHSAG024013	591:991	NONHSAG015664	636:273	NONHSAG051658	1041:133
NONHSAG003645	395:183	NONHSAG033588	493:1	NONHSAG024013	158:845	NONHSAG015664	674:1103	NONHSAG051658	178:203
NONHSAG003645	820:53	NONHSAG033588	247:683	NONHSAG024013	857:767	NONHSAG015664	629:585	NONHSAG051658	865:1091
NONHSAG003645	607:229	NONHSAG033588	734:253	NONHSAG024013	705:111	NONHSAG015664	303:251	NONHSAG051658	44:209
NONHSAG003645	795:537	NONHSAG033588	864:603	NONHSAG024013	579:1125	NONHSAG015664	1150:83	NONHSAG051658	715:163
NONHSAG003645	1113:583	NONHSAG033588	321:65	NONHSAG024040	524:783	NONHSAG015664	774:553	NONHSAG051658	366:765
NONHSAG003668	231:131	NONHSAG033588	1155:401	NONHSAG024040	318:941	NONHSAG015664	131:399	NONHSAG051658	877:945
NONHSAG003668	238:419	NONHSAG033588	56:813	NONHSAG024040	1111:1129	NONHSAG015664	936:847	NONHSAG051658	1013:143
NONHSAG003668	156:1103	NONHSAG033588	409:481	NONHSAG024040	639:1089	NONHSAG015694	850:241	NONHSAG051661	933:281
NONHSAG003668	549:463	NONHSAG033588	90:501	NONHSAG024040	463:1047	NONHSAG015694	902:1143	NONHSAG051661	357:747
NONHSAG003668	971:645	NONHSAG033588	1006:329	NONHSAG024040	388:533	NONHSAG015694	676:567	NONHSAG051661	102:1043
NONHSAG003668	830:263	NONHSAG033588	142:429	NONHSAG024040	998:1133	NONHSAG015694	298:49	NONHSAG051661	922:1073
NONHSAG003668	495:869	NONHSAG033588	790:681	NONHSAG024040	43:955	NONHSAG015694	866:875	NONHSAG051661	882:251
NONHSAG003668	966:1117	NONHSAG033587	985:543	NONHSAG024040	156:499	NONHSAG015694	141:57	NONHSAG051661	328:881
NONHSAG003668	914:1025	NONHSAG033587	154:427	NONHSAG024040	845:565	NONHSAG015694	51:851	NONHSAG051661	120:1041
NONHSAG003758	417:199	NONHSAG033587	231:681	NONHSAG024040	538:1063	NONHSAG015694	913:773	NONHSAG051661	222:35
NONHSAG003758	468:1009	NONHSAG033587	823:839	NONHSAG024043	717:765	NONHSAG015694	899:675	NONHSAG051677	526:675
NONHSAG003758	97:873	NONHSAG033588	15:1001	NONHSAG024043	712:867	NONHSAG015694	366:555	NONHSAG051677	299:265
NONHSAG003758	244:7	NONHSAG033588	169:525	NONHSAG024043	85:163	NONHSAG015694	1053:887	NONHSAG051677	254:871
NONHSAG003758	450:13	NONHSAG033588	306:905	NONHSAG024043	623:229	NONHSAG015694	243:841	NONHSAG051677	73:757
NONHSAG003758	612:635	NONHSAG033588	398:487	NONHSAG024043	973:67	NONHSAG015694	468:1137	NONHSAG051677	227:501
NONHSAG003758	322:1011	NONHSAG033588	110:601	NONHSAG024043	81:1155	NONHSAG015694	264:1125	NONHSAG051677	342:577
NONHSAG003758	455:1077	NONHSAG033588	419:203	NONHSAG024043	290:861	NONHSAG015694	842:991	NONHSAG051677	584:533
NONHSAG003758	1141:739	NONHSAG033588	882:197	NONHSAG024043	506:875	NONHSAG015694	420:457	NONHSAG051677	78:659
NONHSAG003758	219:27	NONHSAG033592	917:627	NONHSAG024043	758:1045	NONHSAG015694	654:591	NONHSAG051677	552:421
NONHSAG003758	697:1043	NONHSAG033592	983:915	NONHSAG024043	512:567	NONHSAG015694	25:1045	NONHSAG051677	989:715
NONHSAG003765	516:549	NONHSAG033592	61:345	NONHSAG024043	20:121	NONHSAG015694	655:121	NONHSAG051728	302:231
NONHSAG003765	542:311	NONHSAG033592	988:623	NONHSAG024046	594:1149	NONHSAG015694	79:321	NONHSAG051728	406:1117
NONHSAG003765	1120:1159	NONHSAG033592	477:7	NONHSAG024046	379:969	NONHSAG015694	1077:1149	NONHSAG051728	948:719
NONHSAG003765	462:291	NONHSAG033592	531:1115	NONHSAG024046	1144:469	NONHSAG015694	807:757	NONHSAG051728	178:691
NONHSAG003765	562:745	NONHSAG033592	320:779	NONHSAG024046	624:785	NONHSAG015694	861:435	NONHSAG051728	482:719
NONHSAG003765	743:451	NONHSAG033592	929:15	NONHSAG024046	10:219	NONHSAG015694	1032:75	NONHSAG051728	1100:1125
NONHSAG003765	605:415	NONHSAG033592	95:415	NONHSAG024046	139:1095	NONHSAG015694	175:551	NONHSAG051728	979:173
NONHSAG003765	1108:121	NONHSAG033592	233:675	NONHSAG024046	860:203	NONHSAG015694	638:795	NONHSAG051728	1151:455
NONHSAG003765	48:907	NONHSAG033592	1084:1135	NONHSAG024080	362:699	NONHSAG015694	866:113	NONHSAG051728	540:477
NONHSAG003765	734:513	NONHSAG033593	282:9	NONHSAG024080	738:681	NONHSAG015694	76:973	NONHSAG051728	505:21
NONHSAG003765	666:597	NONHSAG033593	724:1153	NONHSAG024080	1149:927	NONHSAG015694	774:37	NONHSAG051728	120:939
NONHSAG003799	385:219	NONHSAG033593	884:181	NONHSAG024080	335:597	NONHSAG015694	184:845	NONHSAG051730	5:71
NONHSAG003799	277:1065	NONHSAG033593	1090:673	NONHSAG024080	147:955	NONHSAG015695	750:1147	NONHSAG051730	852:815
NONHSAG003799	1116:1087	NONHSAG033593	1060:473	NONHSAG024080	362:849	NONHSAG015695	384:1007	NONHSAG051730	898:335
NONHSAG003799	110:719	NONHSAG033593	777:627	NONHSAG024080	153:663	NONHSAG015695	405:485	NONHSAG051730	208:99
NONHSAG003799	701:987	NONHSAG033593	622:771	NONHSAG024080	268:665	NONHSAG015695	1008:89	NONHSAG051730	974:255
NONHSAG003799	788:143	NONHSAG033593	960:1123	NONHSAG024080	556:1101	NONHSAG015695	289:1051	NONHSAG051730	1104:1151
NONHSAG003799	301:1131	NONHSAG033593	1029:471	NONHSAG024104	1085:1135	NONHSAG015695	1038:321	NONHSAG051730	299:551
NONHSAG003799	362:641	NONHSAG033593	323:955	NONHSAG024104	1161:835	NONHSAG015695	975:1153	NONHSAG051730	769:375
NONHSAG003829	52:945	NONHSAG033593	557:1019	NONHSAG024104	277:609	NONHSAG015695	508:953	NONHSAG051730	1008:23
NONHSAG003829	450:601	NONHSAG033604	739:209	NONHSAG024104	198:91	NONHSAG015695	929:993	NONHSAG051730	364:1059
NONHSAG003829	456:73	NONHSAG033604	777:545	NONHSAG024104	1040:1033	NONHSAG015695	715:1143	NONHSAG051770	398:509
NONHSAG003829	467:431	NONHSAG033604	607:309	NONHSAG024104	974:1065	NONHSAG015708	823:1109	NONHSAG051770	31:1025
NONHSAG003829	51:269	NONHSAG033604	166:69	NONHSAG024104	783:689	NONHSAG015708	866:1039	NONHSAG051770	1150:39
NONHSAG003829	411:707	NONHSAG033604	37:847	NONHSAG024104	1049:201	NONHSAG015708	1153:799	NONHSAG051770	412:721
NONHSAG003829	2:1031	NONHSAG033604	539:171	NONHSAG024104	268:239	NONHSAG015708	386:69	NONHSAG051770	111:741
NONHSAG003829	674:383	NONHSAG033604	230:559	NONHSAG024105	191:459	NONHSAG015708	668:717	NONHSAG051770	972:335
NONHSAG003829	578:661	NONHSAG033604	764:781	NONHSAG024105	512:503	NONHSAG015708	927:997	NONHSAG051770	832:573
NONHSAG003829	532:75	NONHSAG033604	962:731	NONHSAG024105	1134:673	NONHSAG015708	966:919	NONHSAG051770	751:805
NONHSAG003829	1145:467	NONHSAG033604	217:343	NONHSAG024105	335:461	NONHSAG015708	744:507	NONHSAG051770	67:873

NONHSAG003840	150:991	NONHSAG033616	245:769	NONHSAG024105	83:1097	NONHSAG015708	940:57	NONHSAG051806	253:633
NONHSAG003840	485:369	NONHSAG033616	760:965	NONHSAG024105	1020:407	NONHSAG015708	564:77	NONHSAG051806	370:611
NONHSAG003840	245:905	NONHSAG033616	123:851	NONHSAG024105	317:337	NONHSAG015712	386:1147	NONHSAG051806	1138:25
NONHSAG003840	567:441	NONHSAG033616	228:703	NONHSAG024105	738:883	NONHSAG015712	36:273	NONHSAG051806	454:685
NONHSAG003840	491:281	NONHSAG033616	108:19	NONHSAG024105	1017:325	NONHSAG015712	1058:343	NONHSAG051806	101:1077
NONHSAG003840	92:703	NONHSAG033616	491:653	NONHSAG024105	816:497	NONHSAG015712	585:549	NONHSAG051806	281:933
NONHSAG003840	404:767	NONHSAG033616	158:1021	NONHSAG024105	1026:623	NONHSAG015712	1065:1013	NONHSAG051806	353:741
NONHSAG003840	1031:747	NONHSAG033616	866:509	NONHSAG024135	174:569	NONHSAG015712	866:19	NONHSAG051806	681:1053
NONHSAG003840	869:9	NONHSAG033616	991:787	NONHSAG024135	366:357	NONHSAG015712	1026:681	NONHSAG051806	740:425
NONHSAG003840	966:1039	NONHSAG033616	49:53	NONHSAG024135	70:233	NONHSAG015712	926:711	NONHSAG051806	628:325
NONHSAG003840	1155:699	NONHSAG033616	187:493	NONHSAG024135	183:799	NONHSAG015712	995:361	NONHSAG051813	1063:419
NONHSAG003843	20:1129	NONHSAG033616	270:989	NONHSAG024135	591:529	NONHSAG015712	132:15	NONHSAG051813	257:301
NONHSAG003843	199:69	NONHSAG033616	603:471	NONHSAG024135	1157:1111	NONHSAG015718	1069:347	NONHSAG051813	290:777
NONHSAG003843	8:933	NONHSAG033616	716:279	NONHSAG024135	923:277	NONHSAG015718	630:559	NONHSAG051813	1143:237
NONHSAG003843	54:287	NONHSAG033616	591:955	NONHSAG024135	656:251	NONHSAG015718	567:555	NONHSAG051813	46:217
NONHSAG003843	952:1061	NONHSAG033616	973:395	NONHSAG024135	1057:181	NONHSAG015718	254:615	NONHSAG051813	920:81
NONHSAG003843	147:1085	NONHSAG033616	855:1015	NONHSAG024135	631:1051	NONHSAG015718	1053:781	NONHSAG051813	75:1033
NONHSAG003843	469:43	NONHSAG033616	1064:535	NONHSAG024135	736:129	NONHSAG015718	907:195	NONHSAG051813	1128:963
NONHSAG003843	32:1105	NONHSAG033616	453:975	NONHSAG024143	1116:177	NONHSAG015718	1069:975	NONHSAG051813	915:933
NONHSAG003843	404:279	NONHSAG033616	1134:265	NONHSAG024143	811:615	NONHSAG015718	596:453	NONHSAG051813	210:1107
NONHSAG003843	710:645	NONHSAG033616	1041:495	NONHSAG024143	182:361	NONHSAG015718	497:179	NONHSAG051813	499:777
NONHSAG003843	849:1103	NONHSAG033639	254:97	NONHSAG024143	1100:785	NONHSAG015718	327:807	NONHSAG051813	122:379
NONHSAG003854	435:851	NONHSAG033639	108:915	NONHSAG024143	467:347	NONHSAG015718	1069:653	NONHSAG051813	484:51
NONHSAG003854	458:433	NONHSAG033639	219:1155	NONHSAG024146	230:313	NONHSAG015718	547:39	NONHSAG051813	914:753
NONHSAG003854	920:301	NONHSAG033639	939:629	NONHSAG024146	135:279	NONHSAG015718	423:959	NONHSAG051826	93:1071
NONHSAG003854	156:23	NONHSAG033639	89:1127	NONHSAG024146	13:63	NONHSAG015718	364:553	NONHSAG051826	653:873
NONHSAG003854	651:1073	NONHSAG033639	43:457	NONHSAG024146	653:969	NONHSAG015718	879:253	NONHSAG051826	1098:719
NONHSAG003854	813:1071	NONHSAG033639	405:97	NONHSAG024146	1144:163	NONHSAG015718	1160:251	NONHSAG051826	210:977
NONHSAG003854	962:687	NONHSAG033639	423:905	NONHSAG024146	617:1041	NONHSAG015718	280:33	NONHSAG051826	465:733
NONHSAG003854	261:57	NONHSAG033639	761:713	NONHSAG024146	310:23	NONHSAG015718	783:731	NONHSAG051826	479:253
NONHSAG003854	146:547	NONHSAG033639	95:841	NONHSAG024146	1102:893	NONHSAG015732	521:603	NONHSAG051826	544:1041
NONHSAG003854	171:387	NONHSAG033752	67:793	NONHSAG024146	904:1065	NONHSAG015732	657:1043	NONHSAG051826	388:875
NONHSAG003854	569:195	NONHSAG033752	488:251	NONHSAG024146	457:329	NONHSAG015732	712:557	NONHSAG051826	1012:169
NONHSAG003880	752:697	NONHSAG033752	665:751	NONHSAG024146	519:219	NONHSAG015732	596:835	NONHSAG051826	951:1131
NONHSAG003880	129:1113	NONHSAG033752	713:643	NONHSAG024146	1032:851	NONHSAG015732	875:931	NONHSAG051826	288:201
NONHSAG003880	140:369	NONHSAG033752	320:681	NONHSAG024157	282:1135	NONHSAG015732	377:933	NONHSAG051826	626:53
NONHSAG003880	621:809	NONHSAG033752	325:327	NONHSAG024157	736:145	NONHSAG015732	901:281	NONHSAG051826	336:591
NONHSAG003880	358:569	NONHSAG033752	26:793	NONHSAG024157	751:1115	NONHSAG015732	416:675	NONHSAG051826	534:725
NONHSAG003880	130:283	NONHSAG033752	389:1003	NONHSAG024157	399:73	NONHSAG015732	1120:989	NONHSAG051826	874:925
NONHSAG003880	246:45	NONHSAG033752	848:89	NONHSAG024157	1133:1115	NONHSAG015732	832:561	NONHSAG051826	246:1081
NONHSAG003880	504:633	NONHSAG033752	704:305	NONHSAG024157	226:709	NONHSAG015732	57:453	NONHSAG051826	251:503
NONHSAG003880	804:1109	NONHSAG033752	651:1001	NONHSAG024157	558:389	NONHSAG015732	434:779	NONHSAG051826	108:717
NONHSAG003880	865:1047	NONHSAG033752	556:629	NONHSAG024157	278:57	NONHSAG015732	608:201	NONHSAG051826	883:979
NONHSAG003880	1160:117	NONHSAG033752	593:895	NONHSAG024157	722:131	NONHSAG015732	269:1045	NONHSAG051826	1061:273
NONHSAG003894	579:397	NONHSAG033752	344:1005	NONHSAG024157	802:113	NONHSAG015732	217:1097	NONHSAG051826	765:333
NONHSAG003894	377:227	NONHSAG033752	1160:731	NONHSAG024157	282:659	NONHSAG015732	1035:871	NONHSAG051826	937:1105
NONHSAG003894	556:521	NONHSAG033752	909:687	NONHSAG024162	1000:379	NONHSAG015732	562:797	NONHSAG051866	692:457
NONHSAG003894	253:577	NONHSAG033752	475:885	NONHSAG024162	88:1143	NONHSAG015732	729:529	NONHSAG051866	1013:169
NONHSAG003894	197:989	NONHSAG033752	707:1003	NONHSAG024162	240:983	NONHSAG015732	676:1159	NONHSAG051866	423:565
NONHSAG003894	1087:609	NONHSAG033752	1155:943	NONHSAG024162	215:55	NONHSAG015732	183:1131	NONHSAG051866	675:823
NONHSAG003894	195:359	NONHSAG033752	162:835	NONHSAG024162	526:951	NONHSAG015732	93:1009	NONHSAG051866	656:39
NONHSAG003894	295:1077	NONHSAG033752	936:293	NONHSAG024162	836:665	NONHSAG015732	1090:31	NONHSAG051866	165:679
NONHSAG003894	77:229	NONHSAG033752	404:1027	NONHSAG024162	955:857	NONHSAG015756	188:217	NONHSAG051866	230:165
NONHSAG003894	412:487	NONHSAG033752	1090:85	NONHSAG024162	705:927	NONHSAG015756	468:961	NONHSAG051885	300:729
NONHSAG003894	210:273	NONHSAG033752	822:983	NONHSAG024162	649:391	NONHSAG015756	1105:1127	NONHSAG051885	1035:859
NONHSAG003894	49:483	NONHSAG033752	1041:635	NONHSAG024182	104:541	NONHSAG015756	107:151	NONHSAG051885	307:973
NONHSAG003894	981:1007	NONHSAG033752	903:405	NONHSAG024182	1087:789	NONHSAG015756	1029:743	NONHSAG051885	1048:679
NONHSAG003894	572:161	NONHSAG033752	357:809	NONHSAG024182	13:133	NONHSAG015756	157:199	NONHSAG051885	271:961
NONHSAG003894	937:171	NONHSAG033752	256:147	NONHSAG024182	19:265	NONHSAG015756	1030:969	NONHSAG051885	847:477
NONHSAG003894	345:873	NONHSAG033788	292:717	NONHSAG024182	202:149	NONHSAG015756	1093:211	NONHSAG051885	755:689
NONHSAG003894	617:791	NONHSAG033788	1102:219	NONHSAG024182	78:933	NONHSAG015756	1018:219	NONHSAG051885	461:357
NONHSAG003894	304:657	NONHSAG033788	82:827	NONHSAG024182	1044:741	NONHSAG015756	758:745	NONHSAG051885	230:1079
NONHSAG003894	679:701	NONHSAG033788	410:371	NONHSAG024182	602:189	NONHSAG015756	393:123	NONHSAG051885	161:205
NONHSAG003894	262:1095	NONHSAG033788	568:499	NONHSAG024182	536:159	NONHSAG015756	605:101	NONHSAG051885	598:527
NONHSAG003900	712:45	NONHSAG033788	294:625	NONHSAG024182	154:799	NONHSAG015756	842:361	NONHSAG051943	776:435
NONHSAG003900	1126:589	NONHSAG033788	393:471	NONHSAG024209	361:9	NONHSAG015756	999:225	NONHSAG051943	538:667
NONHSAG003900	879:499	NONHSAG033805	569:1029	NONHSAG024209	533:533	NONHSAG015756	278:579	NONHSAG051943	64:1135
NONHSAG003900	336:1083	NONHSAG033805	290:1059	NONHSAG024209	121:189	NONHSAG015756	1154:1087	NONHSAG051943	26:805
NONHSAG003900	632:701	NONHSAG033805	687:479	NONHSAG024209	1070:347	NONHSAG015756	870:1063	NONHSAG051943	405:807
NONHSAG003900	820:43	NONHSAG033805	965:871	NONHSAG024209	759:155	NONHSAG015756	915:191	NONHSAG051943	708:47
NONHSAG003900	512:1027	NONHSAG033805	35:357	NONHSAG024209	293:557	NONHSAG015756	450:577	NONHSAG051943	537:717
NONHSAG003900	558:815	NONHSAG033805	122:143	NONHSAG024209	926:881	NONHSAG015756	1066:1067	NONHSAG051943	527:741
NONHSAG003900	192:1129	NONHSAG033805	1153:777	NONHSAG024209	759:945	NONHSAG015756	116:57	NONHSAG051943	624:753
NONHSAG003900	1160:667	NONHSAG033805	329:485	NONHSAG024209	640:291	NONHSAG015756	300:1061	NONHSAG051943	597:187
NONHSAG003900	53:1033	NONHSAG033805	81:985	NONHSAG024209	179:787	NONHSAG015757	1098:779	NONHSAG051943	356:469
NONHSAG003900	862:361	NONHSAG033805	324:895	NONHSAG024209	935:51	NONHSAG015757	711:315	NONHSAG051945	1153:331
NONHSAG003900	837:711	NONHSAG033897	66:657	NONHSAG024232	351:317	NONHSAG015757	573:619	NONHSAG051945	549:579
NONHSAG003900	190:1161	NONHSAG033897	945:1	NONHSAG024232	629:885	NONHSAG015757	36:255	NONHSAG055153	220:495
NONHSAG003900	478:171	NONHSAG033897	446:357	NONHSAG024232	414:495	NONHSAG015757	1140:371	NONHSAG055153	545:401
NONHSAG003900	438:1129	NONHSAG033897	589:445	NONHSAG024232	911:75	NONHSAG015757	375:271	NONHSAG055204	326:1085
NONHSAG003900	579:123	NONHSAG033897	1152:725	NONHSAG024232	1042:485	NONHSAG015757	1137:763	NONHSAG055204	1076:769
NONHSAG003900	365:861	NONHSAG033897	1021:427	NONHSAG024232	368:303	NONHSAG015757	404:1077	NONHSAG055204	1002:1111
NONHSAG003927	468:811	NONHSAG033897	301:77	NONHSAG024232	251:315	NONHSAG015757	407:757	NONHSAG055204	771:229
NONHSAG003927	1084:657	NONHSAG033897	921:783	NONHSAG024232	478:543	NONHSAG015757	63:711	NONHSAG055204	66:699
NONHSAG003927	327:239	NONHSAG033897	1108:649	NONHSAG024232	366:763	NONHSAG015757	175:807	NONHSAG055204	1089:619
NONHSAG003927	547:465	NONHSAG033897	770:393	NONHSAG024232	668:73	NONHSAG015797	738:343	NONHSAG055204	114:1157
NONHSAG003927	1012:977	NONHSAG033897	570:399	NONHSAG024232	538:503	NONHSAG015797	856:991	NONHSAG055204	659:515
NONHSAG003927	806:699	NONHSAG033898	332:773	NONHSAG024232	889:677	NONHSAG015797	734:53	NONHSAG055204	176:849

NONHSAG003927	406:485	NONHSAG033898	347:139	NONHSAG024232	868:331	NONHSAG015797	60:253	NONHSAG055266	263:513
NONHSAG003927	790:399	NONHSAG033898	902:915	NONHSAG024232	1078:611	NONHSAG015797	531:107	NONHSAG055266	894:857
NONHSAG003927	1040:563	NONHSAG033898	894:993	NONHSAG024232	980:575	NONHSAG015797	315:601	NONHSAG055266	218:583
NONHSAG003927	1064:447	NONHSAG033898	52:75	NONHSAG024232	406:1065	NONHSAG015797	1079:37	NONHSAG055266	192:73
NONHSAG003927	1088:597	NONHSAG033898	252:455	NONHSAG024232	209:421	NONHSAG015798	368:701	NONHSAG055266	369:353
NONHSAG004034	467:421	NONHSAG033898	1115:505	NONHSAG024232	539:1125	NONHSAG015798	1062:201	NONHSAG055266	480:627
NONHSAG004034	367:789	NONHSAG033898	837:105	NONHSAG024232	9:491	NONHSAG015798	470:707	NONHSAG055266	577:293
NONHSAG004034	827:1013	NONHSAG033898	432:919	NONHSAG024232	164:437	NONHSAG015798	883:1087	NONHSAG055266	577:125
NONHSAG004034	115:449	NONHSAG033898	1154:977	NONHSAG024232	192:455	NONHSAG015798	267:215	NONHSAG055266	46:757
NONHSAG004034	643:987	NONHSAG033898	910:773	NONHSAG024232	681:605	NONHSAG015798	720:981	NONHSAG055266	1119:157
NONHSAG004034	394:927	NONHSAG033967	464:55	NONHSAG024234	791:689	NONHSAG015798	242:305	NONHSAG055280	326:1011
NONHSAG004034	999:359	NONHSAG033967	521:123	NONHSAG024234	1013:1143	NONHSAG015798	841:907	NONHSAG055280	256:777
NONHSAG004034	311:363	NONHSAG033967	158:115	NONHSAG024234	907:823	NONHSAG015798	43:705	NONHSAG055280	729:163
NONHSAG004034	151:961	NONHSAG033967	319:847	NONHSAG024234	643:337	NONHSAG015798	316:1067	NONHSAG055280	105:1129
NONHSAG004049	977:1073	NONHSAG033967	474:997	NONHSAG024234	821:1089	NONHSAG015798	696:433	NONHSAG055280	106:889
NONHSAG004049	1007:575	NONHSAG033967	259:1109	NONHSAG024234	765:659	NONHSAG015798	404:541	NONHSAG055280	396:699
NONHSAG004049	226:117	NONHSAG033967	458:869	NONHSAG024234	704:205	NONHSAG015798	743:625	NONHSAG055280	875:979
NONHSAG004049	792:385	NONHSAG033967	1072:793	NONHSAG024234	1108:929	NONHSAG015798	923:429	NONHSAG055280	614:21
NONHSAG004049	894:105	NONHSAG033967	1044:133	NONHSAG024234	404:359	NONHSAG015798	321:183	NONHSAG055280	243:819
NONHSAG004049	633:391	NONHSAG033967	310:511	NONHSAG024234	59:723	NONHSAG015798	253:91	NONHSAG055280	192:499
NONHSAG004049	497:589	NONHSAG033967	611:663	NONHSAG024234	881:723	NONHSAG015798	688:215	NONHSAG055280	914:539
NONHSAG004092	167:385	NONHSAG033967	644:249	NONHSAG024244	1060:427	NONHSAG015798	528:409	NONHSAG055289	915:1091
NONHSAG004092	681:911	NONHSAG033967	812:263	NONHSAG024244	1107:207	NONHSAG015798	845:885	NONHSAG055289	224:485
NONHSAG004092	1041:911	NONHSAG033967	361:419	NONHSAG024244	1109:361	NONHSAG015798	328:517	NONHSAG055289	1019:223
NONHSAG004092	310:1047	NONHSAG033967	145:529	NONHSAG024244	905:989	NONHSAG015798	973:1059	NONHSAG055289	1116:613
NONHSAG004092	369:867	NONHSAG033967	346:167	NONHSAG024244	537:329	NONHSAG015798	589:1103	NONHSAG055289	553:801
NONHSAG004092	927:343	NONHSAG033967	4:509	NONHSAG024244	754:663	NONHSAG015798	1055:923	NONHSAG055289	180:261
NONHSAG004092	1044:219	NONHSAG033967	673:137	NONHSAG024244	906:87	NONHSAG015798	832:591	NONHSAG055289	199:671
NONHSAG004092	99:1061	NONHSAG033967	39:1087	NONHSAG024244	772:1131	NONHSAG015798	330:1035	NONHSAG055289	708:783
NONHSAG004092	84:945	NONHSAG033967	987:967	NONHSAG024244	655:249	NONHSAG015798	970:999	NONHSAG055289	617:875
NONHSAG004092	641:953	NONHSAG033967	988:793	NONHSAG024244	768:1067	NONHSAG015798	808:869	NONHSAG055289	613:913
NONHSAG004092	29:243	NONHSAG033967	733:277	NONHSAG024244	450:365	NONHSAG015798	875:751	NONHSAG055301	1004:267
C1orf132	700:1087	NONHSAG033980	479:79	NONHSAG024248	873:1061	NONHSAG015798	60:671	NONHSAG055301	913:305
C1orf132	601:381	NONHSAG033980	211:7	NONHSAG024248	379:145	NONHSAG015798	1139:227	NONHSAG055301	1077:207
C1orf132	1071:865	NONHSAG033980	1056:241	NONHSAG024248	709:533	NONHSAG015798	1025:589	NONHSAG055301	1009:107
C1orf132	724:535	NONHSAG033980	386:401	NONHSAG024248	249:859	NONHSAG015800	875:825	NONHSAG055301	50:207
C1orf132	872:1029	NONHSAG033980	248:1155	NONHSAG024248	984:915	NONHSAG015800	175:281	NONHSAG055301	568:881
C1orf132	619:55	NONHSAG033980	570:49	NONHSAG024248	502:1041	NONHSAG015800	809:499	NONHSAG055301	1039:961
C1orf132	289:751	NONHSAG033980	492:1093	NONHSAG024248	89:345	NONHSAG015800	205:807	NONHSAG055301	1017:199
C1orf132	832:727	NONHSAG033980	669:1063	NONHSAG024248	248:859	NONHSAG015800	546:283	NONHSAG055301	359:945
C1orf132	463:455	NONHSAG033980	465:79	NONHSAG024248	1034:381	NONHSAG015800	1142:681	NONHSAG055339	159:475
C1orf132	127:921	NONHSAG033980	123:705	NONHSAG024248	135:155	NONHSAG015800	103:287	NONHSAG055339	636:477
C1orf132	1073:827	NONHSAG033989	1051:573	NONHSAG024264	382:161	NONHSAG015800	704:139	NONHSAG055339	963:863
C1orf132	636:113	NONHSAG033989	1059:1031	NONHSAG024264	1091:1135	NONHSAG015800	139:1097	NONHSAG055339	509:963
C1orf132	436:647	NONHSAG033989	893:667	NONHSAG024264	207:419	NONHSAG015800	190:1019	NONHSAG055339	90:1039
C1orf132	461:97	NONHSAG033989	730:867	NONHSAG024264	1043:51	NONHSAG015800	1020:755	NONHSAG055339	581:725
C1orf132	289:423	NONHSAG033989	515:135	NONHSAG024264	441:735	NONHSAG015828	640:773	NONHSAG055339	1077:603
C1orf132	1104:1139	NONHSAG033989	796:897	NONHSAG024264	149:335	NONHSAG015828	540:1059	NONHSAG055339	905:761
C1orf132	685:1143	NONHSAG033989	1082:619	NONHSAG024264	985:823	NONHSAG015828	95:553	NONHSAG055339	956:657
C1orf132	841:431	NONHSAG033989	556:719	NONHSAG024264	729:455	NONHSAG015828	865:661	NONHSAG055339	627:947
C1orf132	23:671	NONHSAG033989	897:429	NONHSAG024264	39:171	NONHSAG015828	298:119	NONHSAG055339	35:233
C1orf132	1125:155	EP300-AS1	667:451	NONHSAG024327	715:761	NONHSAG015828	251:1117	NONHSAG055350	113:985
NONHSAG004140	405:99	EP300-AS1	207:1119	NONHSAG024327	359:941	NONHSAG015828	1106:617	NONHSAG055350	993:131
NONHSAG004140	803:495	EP300-AS1	1107:837	NONHSAG024327	321:417	NONHSAG015828	670:1141	NONHSAG055350	1128:997
NONHSAG004140	369:1039	EP300-AS1	650:697	NONHSAG024327	594:85	NONHSAG015828	609:723	NONHSAG055350	301:269
NONHSAG004140	481:421	EP300-AS1	440:923	NONHSAG024327	405:613	NONHSAG015828	137:221	NONHSAG055350	570:291
NONHSAG004140	615:849	EP300-AS1	152:3	NONHSAG024381	990:475	NONHSAG015833	991:965	NONHSAG055350	1143:443
NONHSAG004140	204:5	EP300-AS1	125:371	NONHSAG024381	524:1133	NONHSAG015833	683:1093	NONHSAG055350	854:851
NONHSAG004140	285:689	EP300-AS1	444:689	NONHSAG024381	380:25	NONHSAG015833	1027:87	NONHSAG055350	362:915
NONHSAG004140	752:1057	EP300-AS1	63:1099	NONHSAG024381	351:411	NONHSAG015833	859:711	NONHSAG055350	342:65
NONHSAG004156	1114:425	EP300-AS1	452:1003	NONHSAG024381	208:1063	NONHSAG015833	953:55	NONHSAG055350	1084:159
NONHSAG004156	986:1007	EP300-AS1	1018:667	NONHSAG024381	126:437	NONHSAG015833	1143:1157	NONHSAG055350	57:747
NONHSAG004156	1056:935	NONHSAG034032	845:863	NONHSAG024381	767:417	NONHSAG015833	313:461	NONHSAG055350	85:487
NONHSAG004156	42:685	NONHSAG034032	460:697	NONHSAG024381	464:1061	NONHSAG015833	21:747	NONHSAG055350	53:355
NONHSAG004156	964:319	NONHSAG034032	501:181	NONHSAG024388	542:957	NONHSAG015833	830:833	NONHSAG055350	1009:347
NONHSAG004156	1159:705	NONHSAG034032	1131:387	NONHSAG024388	42:901	NONHSAG015833	591:1115	NONHSAG055350	157:809
NONHSAG004156	311:761	NONHSAG034032	320:677	NONHSAG024388	687:1009	NONHSAG015833	686:565	NONHSAG055350	362:825
NONHSAG004156	1050:15	NONHSAG034032	507:977	NONHSAG024388	17:419	NONHSAG015852	280:213	NONHSAG055350	1033:293
NONHSAG004156	762:513	NONHSAG034032	872:597	NONHSAG024388	702:661	NONHSAG015852	1010:991	NONHSAG055350	316:345
NONHSAG004156	172:841	NONHSAG034032	462:11	NONHSAG024388	185:639	NONHSAG015852	269:1093	NONHSAG055350	752:1151
NONHSAG004156	818:187	NONHSAG034032	24:87	NONHSAG024388	796:995	NONHSAG015852	208:207	NONHSAG055350	617:485
NONHSAG004159	1131:183	NONHSAG034032	1129:853	NONHSAG024388	1090:239	NONHSAG015872	531:977	NONHSAG055350	170:1107
NONHSAG004159	127:297	NONHSAG034032	1091:423	NONHSAG024388	23:429	NONHSAG015872	1045:881	NONHSAG055350	565:773
NONHSAG004159	1010:527	NONHSAG034032	751:1085	NONHSAG024388	125:973	NONHSAG015872	904:95	NONHSAG055350	426:949
NONHSAG004159	1152:465	NONHSAG034032	697:899	NONHSAG024397	399:491	NONHSAG015872	374:955	NONHSAG055350	1133:493
NONHSAG004159	747:465	NONHSAG034032	209:569	NONHSAG024397	236:881	NONHSAG015872	1067:983	NONHSAG055350	1153:993
NONHSAG004159	213:137	NONHSAG034032	359:231	NONHSAG024397	682:291	NONHSAG015872	752:433	NONHSAG055350	225:813
NONHSAG004159	742:157	NONHSAG034050	160:499	NONHSAG024397	794:833	NONHSAG015872	239:405	NONHSAG055350	875:315
NONHSAG004159	287:595	NONHSAG034050	324:9	NONHSAG024397	1147:615	NONHSAG015872	963:1	NONHSAG055350	224:395
NONHSAG004159	157:475	NONHSAG034050	896:955	NONHSAG024397	958:865	NONHSAG015872	798:797	NONHSAG055350	613:921
NONHSAG004159	1098:139	NONHSAG034050	557:49	NONHSAG024397	191:731	NONHSAG015872	1096:937	NONHSAG055367	411:669
NONHSAG004205	605:155	NONHSAG034050	695:115	NONHSAG024397	45:703	NONHSAG015872	1113:215	NONHSAG055367	534:379
NONHSAG004205	1141:205	NONHSAG034050	397:903	NONHSAG024397	595:1127	NONHSAG015874	1102:275	NONHSAG055367	635:795
NONHSAG004205	578:91	NONHSAG034050	1000:719	NONHSAG024397	1087:707	NONHSAG015874	900:561	NONHSAG055367	1145:503
NONHSAG004205	345:1071	NONHSAG034050	916:1027	NONHSAG024397	1004:741	NONHSAG015874	452:71	NONHSAG055367	791:979
NONHSAG004205	74:205	NONHSAG034050	924:79	NONHSAG024397	1052:383	NONHSAG015874	374:765	NONHSAG055367	754:739
NONHSAG004205	134:299	NONHSAG034050	869:123	NONHSAG024397	798:609	NONHSAG015874	698:35	NONHSAG055367	240:863
NONHSAG004205	725:251	NONHSAG034050	147:469	NONHSAG024397	1128:461	NONHSAG015874	814:519	NONHSAG055367	491:899

NONHSAG004205	570:551	NONHSAG034050	24:133	NONHSAG024397	397:583	NONHSAG015874	714:399	NONHSAG055367	533:547
NONHSAG004205	930:779	NONHSAG034050	209:29	NONHSAG024397	163:709	NONHSAG015874	236:15	NONHSAG055367	339:931
NONHSAG004205	154:1143	NONHSAG034050	1148:9	NONHSAG024397	1080:795	NONHSAG015874	27:1065	NONHSAG055371	245:437
NONHSAG004205	680:817	NONHSAG034050	746:351	NONHSAG024439	942:1139	NONHSAG015874	181:573	NONHSAG055371	638:823
NONHSAG004221	832:21	NONHSAG034050	389:989	NONHSAG024439	896:117	NONHSAG015874	1101:943	NONHSAG055371	163:719
NONHSAG004221	1077:267	NONHSAG034050	862:37	NONHSAG024439	873:987	NONHSAG015923	599:49	NONHSAG055371	804:347
NONHSAG004221	1103:139	NONHSAG034050	110:469	NONHSAG024439	470:893	NONHSAG015923	412:503	NONHSAG055371	555:861
NONHSAG004221	948:149	NONHSAG034050	642:483	NONHSAG024439	1088:957	NONHSAG015923	195:655	NONHSAG055371	379:239
NONHSAG004221	537:481	NONHSAG034050	907:1005	NONHSAG024439	355:529	NONHSAG015923	1124:811	NONHSAG055371	250:215
NONHSAG004221	710:235	NONHSAG034050	326:969	NONHSAG024439	986:213	NONHSAG015923	777:909	NONHSAG055371	1054:75
NONHSAG004221	726:167	NONHSAG034053	1134:129	NONHSAG024439	390:491	NONHSAG015923	949:857	NONHSAG055371	703:869
NONHSAG004235	951:513	NONHSAG034053	842:985	NONHSAG024442	261:213	NONHSAG015923	527:587	NONHSAG055371	457:9
NONHSAG004235	101:865	NONHSAG034053	943:151	NONHSAG024442	880:261	NONHSAG015923	752:463	NONHSAG055371	878:285
NONHSAG004235	1030:71	NONHSAG034053	550:987	NONHSAG024442	851:1027	NONHSAG015923	579:943	NONHSAG055384	748:945
NONHSAG004235	1092:179	NONHSAG034053	260:343	NONHSAG024442	475:361	NONHSAG015923	50:343	NONHSAG055384	303:59
NONHSAG004235	623:601	NONHSAG034053	806:667	NONHSAG024442	853:635	NONHSAG015923	206:485	NONHSAG055384	305:177
NONHSAG004235	66:839	NONHSAG034053	965:473	NONHSAG024442	796:67	NONHSAG015923	821:605	NONHSAG055384	798:475
NONHSAG004235	804:139	NONHSAG034053	1105:713	NONHSAG024442	825:1101	NONHSAG015923	145:937	NONHSAG055384	1052:23
NONHSAG004235	190:917	NONHSAG034053	968:105	NONHSAG024442	272:843	NONHSAG015923	248:1021	NONHSAG055384	121:471
NONHSAG004235	728:453	NONHSAG034053	597:499	NONHSAG024442	932:267	NONHSAG015923	132:165	NONHSAG055384	1114:753
NONHSAG004235	1044:439	NONHSAG034053	71:989	NONHSAG024446	632:389	NONHSAG015923	420:393	NONHSAG055384	454:199
NONHSAG004235	303:157	NONHSAG034077	878:259	NONHSAG024446	425:1105	NONHSAG015923	5:265	NONHSAG055384	189:335
NONHSAG004261	234:741	NONHSAG034077	1112:651	NONHSAG024446	603:217	NONHSAG015923	91:933	NONHSAG055384	305:1071
NONHSAG004261	106:535	NONHSAG034077	886:627	NONHSAG024446	965:543	NONHSAG015923	1087:499	NONHSAG055384	122:623
NONHSAG004261	635:681	NONHSAG034077	678:605	NONHSAG024446	183:1099	NONHSAG015923	205:773	NONHSAG055388	707:497
NONHSAG004261	298:599	NONHSAG034077	934:111	NONHSAG024446	950:197	NONHSAG015923	791:253	NONHSAG055388	963:1117
NONHSAG004261	203:501	NONHSAG034077	853:1	NONHSAG024446	663:833	NONHSAG015923	951:235	NONHSAG055388	655:1111
NONHSAG004261	661:973	NONHSAG034077	356:941	NONHSAG024446	595:19	NONHSAG015923	605:521	NONHSAG055388	675:25
NONHSAG004261	919:1037	NONHSAG034077	628:229	NONHSAG024446	938:981	NONHSAG015923	192:381	NONHSAG055388	354:319
NONHSAG004261	693:267	NONHSAG034077	576:411	NONHSAG024446	1159:391	NONHSAG015923	1036:681	NONHSAG055388	936:915
NONHSAG004261	606:71	NONHSAG034077	625:239	NONHSAG024446	328:1039	NONHSAG015923	870:151	NONHSAG055388	622:693
NONHSAG004261	130:821	NONHSAG034077	763:41	NONHSAG024446	1143:1057	NONHSAG015923	806:695	NONHSAG055388	1128:15
NONHSAG004261	155:107	NONHSAG034081	902:1117	NONHSAG024446	1035:765	NONHSAG015923	31:349	NONHSAG055388	709:205
NONHSAG004261	1101:105	NONHSAG034081	833:27	NONHSAG024446	815:823	NONHSAG015923	963:189	NONHSAG055388	179:675
NONHSAG004261	1128:679	NONHSAG034081	1008:957	NONHSAG024446	212:279	NONHSAG015923	767:351	NONHSAG055388	916:137
NONHSAG004261	936:469	NONHSAG034081	133:1071	NONHSAG024446	475:47	NONHSAG015923	612:893	NONHSAG055401	847:719
NONHSAG004261	663:845	NONHSAG034081	416:737	NONHSAG024446	126:485	NONHSAG015923	583:849	NONHSAG055401	884:387
NONHSAG004261	565:35	NONHSAG034081	752:99	NONHSAG024446	2:923	NONHSAG015923	492:255	NONHSAG055401	447:517
NONHSAG004261	23:577	NONHSAG034081	296:277	NONHSAG024446	1136:967	NONHSAG015923	1104:271	NONHSAG055401	242:821
NONHSAG004261	808:1037	NONHSAG034081	989:1089	NONHSAG024446	655:127	NONHSAG015923	303:9	NONHSAG055401	568:191
NONHSAG004261	59:867	NONHSAG034081	69:505	NONHSAG024446	980:489	NONHSAG015923	788:1041	NONHSAG055401	54:313
NONHSAG004261	1122:771	NONHSAG034081	761:311	NONHSAG024446	941:183	NONHSAG015923	719:591	NONHSAG055401	709:131
NONHSAG004261	1000:427	NONHSAG034081	432:1049	NONHSAG024447	529:875	NONHSAG015923	43:577	NONHSAG055401	434:643
NONHSAG004261	1106:453	NONHSAG034105	1077:345	NONHSAG024447	717:1005	NONHSAG015923	406:41	NONHSAG055401	848:1155
NONHSAG004261	654:859	NONHSAG034105	736:427	NONHSAG024447	22:831	NONHSAG015923	825:569	NONHSAG055401	742:1139
NONHSAG004261	677:649	NONHSAG034105	18:1023	NONHSAG024447	969:543	NONHSAG015923	421:471	NONHSAG055401	895:921
NONHSAG004261	547:273	NONHSAG034105	471:1117	NONHSAG024447	444:517	NONHSAG015923	832:379	NONHSAG055405	625:947
NONHSAG004261	606:31	NONHSAG034105	941:539	NONHSAG024447	753:149	NONHSAG015923	743:871	NONHSAG055405	1071:485
NONHSAG004261	248:747	NONHSAG034105	479:877	NONHSAG024447	1082:221	NONHSAG015923	778:133	NONHSAG055405	795:149
NONHSAG004261	151:1055	NONHSAG034105	484:565	NONHSAG024447	8:1043	NONHSAG015923	808:809	NONHSAG055405	570:253
NONHSAG004261	162:871	NONHSAG034105	910:1009	NONHSAG024447	1122:547	NONHSAG015923	83:83	NONHSAG055405	733:701
NONHSAG004261	1106:203	NONHSAG034105	246:71	NONHSAG024447	378:973	NONHSAG015923	628:555	NONHSAG055405	764:43
NONHSAG004265	924:959	NONHSAG034105	42:471	NONHSAG024447	309:339	NONHSAG015923	461:127	NONHSAG055405	174:151
NONHSAG004265	844:9	NONHSAG034105	934:371	NONHSAG024529	873:121	NONHSAG015923	836:803	NONHSAG055405	848:121
NONHSAG004265	214:559	NONHSAG034111	772:135	NONHSAG024529	882:613	NONHSAG015923	151:965	NONHSAG055405	760:281
NONHSAG004265	955:1125	NONHSAG034111	1000:627	NONHSAG024529	438:841	NONHSAG015923	435:451	NONHSAG055405	476:79
NONHSAG004265	558:187	NONHSAG034111	1064:749	NONHSAG024529	38:843	NONHSAG015923	363:557	NONHSAG055405	69:307
NONHSAG004265	348:95	NONHSAG034111	994:153	NONHSAG024529	247:329	NONHSAG015923	201:269	NONHSAG055409	431:463
NONHSAG004265	507:115	NONHSAG034111	95:1087	NONHSAG024540	652:639	NONHSAG015923	1154:387	NONHSAG055409	128:447
NONHSAG004265	245:55	NONHSAG034111	174:1133	NONHSAG024540	462:615	NONHSAG015923	379:95	NONHSAG055409	301:243
NONHSAG004265	891:55	NONHSAG034111	456:495	NONHSAG024540	875:337	NONHSAG015923	316:813	NONHSAG055409	1105:1029
NONHSAG004265	249:259	NONHSAG034111	271:689	NONHSAG024540	916:779	NONHSAG015923	729:389	NONHSAG055409	661:769
NONHSAG004265	1072:649	NONHSAG034111	1043:559	NONHSAG024540	381:319	NONHSAG015923	661:355	NONHSAG055409	186:781
NONHSAG004289	80:879	NONHSAG034111	819:339	NONHSAG024540	864:937	NONHSAG015923	224:365	NONHSAG055409	177:981
NONHSAG004289	127:451	NONHSAG034111	91:921	NONHSAG024540	548:87	NONHSAG015923	764:1115	NONHSAG055409	376:1085
NONHSAG004289	772:925	NONHSAG034128	838:1119	NONHSAG024540	1006:719	NONHSAG015923	557:555	NONHSAG055409	1146:5
NONHSAG004289	192:59	NONHSAG034128	870:991	NONHSAG024540	931:707	NONHSAG015923	863:1047	NONHSAG055436	74:71
NONHSAG004289	1037:739	NONHSAG034128	646:593	NONHSAG024540	926:695	NONHSAG015923	1024:745	NONHSAG055436	880:185
NONHSAG004289	731:961	NONHSAG034128	426:381	NONHSAG024540	36:919	NONHSAG015923	1013:73	NONHSAG055436	1012:971
NONHSAG004289	246:267	NONHSAG034128	834:1117	NONHSAG024540	337:455	NONHSAG015923	753:13	NONHSAG055436	1121:195
NONHSAG004289	1041:235	NONHSAG034128	1014:943	NONHSAG024540	1120:487	NONHSAG015923	958:29	NONHSAG055436	1039:491
NONHSAG004289	382:381	NONHSAG034128	341:1011	NONHSAG024540	1032:193	NONHSAG015923	1036:57	NONHSAG055436	4:1065
NONHSAG004289	670:1053	NONHSAG034128	368:461	NONHSAG024540	481:267	NONHSAG015923	598:85	NONHSAG055443	1068:933
NONHSAG004289	1162:333	NONHSAG034128	439:585	NONHSAG024540	402:893	NONHSAG015923	493:809	NONHSAG055443	287:831
NONHSAG004289	585:29	NONHSAG034128	150:967	NONHSAG024542	737:1001	NONHSAG015923	260:641	NONHSAG055443	562:1107
NONHSAG004289	200:1093	NONHSAG034128	1094:451	NONHSAG024542	825:813	NONHSAG015923	166:1063	NONHSAG055443	71:371
NONHSAG004289	214:585	NONHSAG034128	1017:501	NONHSAG024542	811:73	NONHSAG015923	203:197	NONHSAG055443	912:829
NONHSAG004289	562:53	NONHSAG034128	984:1087	NONHSAG024542	943:413	NONHSAG015923	534:751	NONHSAG055443	67:353
NONHSAG004289	785:279	NONHSAG034128	154:623	NONHSAG024542	773:307	NONHSAG015923	637:27	NONHSAG055443	1145:1113
NONHSAG004289	447:1151	NONHSAG034128	930:661	NONHSAG024542	392:463	NONHSAG015923	768:783	NONHSAG055443	112:447
NONHSAG004289	1144:817	NONHSAG034128	812:677	NONHSAG024542	57:529	NONHSAG015923	387:31	NONHSAG055443	881:1133
NONHSAG004289	269:763	NONHSAG034128	114:771	NONHSAG024542	870:597	NONHSAG015923	936:371	NONHSAG055443	487:547
NONHSAG004289	889:699	NONHSAG034128	980:657	NONHSAG024542	347:537	NONHSAG015923	503:703	NONHSAG055443	262:1031
NONHSAG004292	208:19	NONHSAG034128	909:833	NONHSAG024542	274:435	NONHSAG015923	190:339	NONHSAG055445	1094:697
NONHSAG004292	562:867	NONHSAG034128	544:737	NONHSAG024542	222:457	NONHSAG015923	711:491	NONHSAG055445	1069:967
NONHSAG004292	493:19	NONHSAG034128	441:599	NONHSAG024613	976:265	NONHSAG015923	873:517	NONHSAG055445	1030:419
NONHSAG004292	521:937	NONHSAG034128	890:1055	NONHSAG024613	524:929	NONHSAG015923	224:289	NONHSAG055445	1089:905
NONHSAG004292	669:171	NONHSAG034158	140:773	NONHSAG024613	757:739	NONHSAG015923	966:183	NONHSAG055445	217:251

NONHSAG004292	1092:1095	NONHSAG034158	462:59	NONHSAG024613	1114:75	NONHSAG015923	263:1051	NONHSAG055445	68:109
NONHSAG004292	1036:139	NONHSAG034158	570:189	NONHSAG024613	267:679	NONHSAG015923	971:535	NONHSAG055446	876:551
NONHSAG004292	450:125	NONHSAG034158	111:49	NONHSAG024613	1014:1119	NONHSAG015923	336:797	NONHSAG055446	955:261
NONHSAG004292	712:13	NONHSAG034158	814:269	NONHSAG024628	742:829	NONHSAG015923	558:799	NONHSAG055446	502:283
NONHSAG004292	671:255	NONHSAG034158	261:49	NONHSAG024628	446:681	NONHSAG015923	1012:1035	NONHSAG055446	653:205
NONHSAG004292	932:347	NONHSAG034158	489:493	NONHSAG024628	286:75	NONHSAG015923	6:1125	NONHSAG055446	647:321
NONHSAG004292	703:1135	NONHSAG034158	273:127	NONHSAG024628	742:593	NONHSAG015923	651:205	NONHSAG055446	982:725
NONHSAG004292	139:829	NONHSAG034158	164:1047	NONHSAG024628	1102:211	NONHSAG015923	625:1011	NONHSAG055446	488:141
NONHSAG004292	1027:497	NONHSAG034158	904:1063	NONHSAG024628	266:313	NONHSAG015923	131:805	NONHSAG055446	598:889
NONHSAG004292	132:1007	NONHSAG034163	720:31	NONHSAG024628	235:777	NONHSAG015923	75:1073	NONHSAG055446	295:379
NONHSAG004292	394:151	NONHSAG034163	794:305	NONHSAG024628	322:783	NONHSAG015923	889:1035	NONHSAG055446	237:955
NONHSAG004319	784:859	NONHSAG034163	822:487	NONHSAG024628	100:513	NONHSAG015923	631:661	NONHSAG055446	572:579
NONHSAG004319	887:1109	NONHSAG034163	933:607	NONHSAG024729	795:899	NONHSAG015923	503:255	NONHSAG055449	657:881
NONHSAG004319	256:571	NONHSAG034163	1052:567	NONHSAG024729	1160:459	NONHSAG015923	716:175	NONHSAG055449	1081:827
NONHSAG004319	655:791	NONHSAG034163	117:285	NONHSAG024729	352:1069	NONHSAG015923	266:361	NONHSAG055449	315:761
NONHSAG004319	553:161	NONHSAG034163	170:753	NONHSAG024729	846:1105	NONHSAG015923	692:859	NONHSAG055449	457:303
NONHSAG004319	844:875	NONHSAG034163	829:43	NONHSAG024729	992:1075	NONHSAG015923	572:123	NONHSAG055449	411:233
NONHSAG004319	219:563	NONHSAG034163	974:281	NONHSAG024729	366:1011	NONHSAG015923	371:975	NONHSAG055449	500:589
NONHSAG004319	831:149	NONHSAG034163	337:979	NONHSAG024729	1151:1085	NONHSAG015923	820:483	NONHSAG055449	404:965
NONHSAG004319	369:803	NONHSAG034163	1020:295	NONHSAG024729	232:623	NONHSAG015923	884:267	NONHSAG055449	633:141
NONHSAG004319	424:1137	NONHSAG034175	711:801	NONHSAG024729	215:557	NONHSAG015923	610:461	NONHSAG055449	850:1145
NONHSAG004319	1137:1017	NONHSAG034175	622:513	NONHSAG024729	425:343	NONHSAG015923	323:813	NONHSAG055449	173:659
NONHSAG004322	780:709	NONHSAG034175	549:959	NONHSAG024729	407:77	NONHSAG015923	1139:711	NONHSAG055449	195:191
NONHSAG004322	193:575	NONHSAG034175	1091:527	NONHSAG024733	57:265	NONHSAG015923	398:563	NONHSAG055454	1138:1041
NONHSAG004322	116:543	NONHSAG034175	688:833	NONHSAG024733	1052:209	NONHSAG015923	897:407	NONHSAG055454	376:143
NONHSAG004322	1127:947	NONHSAG034175	417:703	NONHSAG024733	648:853	NONHSAG015923	225:1059	NONHSAG055454	301:995
NONHSAG004322	830:123	NONHSAG034175	519:479	NONHSAG024733	134:29	NONHSAG015923	535:185	NONHSAG055454	616:1053
NONHSAG004322	813:1037	NONHSAG034175	95:961	NONHSAG024733	783:163	NONHSAG015923	1141:579	NONHSAG055454	1108:1059
NONHSAG004322	317:425	NONHSAG034175	338:579	NONHSAG024733	1026:1119	NONHSAG015923	380:507	NONHSAG055454	66:689
NONHSAG004322	808:349	NONHSAG034175	463:761	NONHSAG024733	110:87	NONHSAG015923	1027:651	NONHSAG055454	507:1105
NONHSAG004322	187:121	NONHSAG034179	963:803	NONHSAG024733	342:153	NONHSAG015923	931:145	NONHSAG055454	681:401
[truncated: 779,686 more chars]
